# TET1 is a Tumor Suppressor That Inhibits Papillary Thyroid Carcinoma Cell Migration and Invasion

**DOI:** 10.1155/2020/3909610

**Published:** 2020-02-08

**Authors:** Shuang Yu, Yali Yin, Shubin Hong, Siting Cao, Yanrui Huang, Shuwei Chen, Yujie Liu, Hongyu Guan, Quan Zhang, Yanbing Li, Haipeng Xiao

**Affiliations:** ^1^Department of Endocrinology, The First Affiliated Hospital of Sun Yat-Sen University, Guangzhou 510080, China; ^2^Department of Endocrinology, Peking University Shenzhen Hospital, Shenzhen 518036, China; ^3^Department of Head and Neck Surgery, Sun Yat-Sen University Cancer Center, Guangzhou 510060, China; ^4^State Key Laboratory of Oncology in South China, Collaborative Innovation Center of Cancer Medicine, Guangzhou 510060, China; ^5^Breast Tumor Center, Sun Yat-Sen Memorial Hospital, Sun Yat-Sen University, Guangzhou 510120, China

## Abstract

**Background:**

Ten-eleven translocation (TET) enzymes catalyze the oxidation of 5-methylcytosine (5mC) to 5-hydroxymethylcytosine (5hmC) promoting demethylation in cells. However, the expression pattern and biologic significance of TET in papillary thyroid carcinoma (PTC) remain unclear. This study aimed to elucidate the biological functions of TET1 and the miRNA and mRNA expression levels in PTC cells with downregulated TET1.

**Methods:**

The expression of the TET family in 49 PTC tissues and corresponding tumor-adjacent tissues, as well as PTC cell lines (BCPAP, K1, and TPC-1) and the normal thyroid epithelial cell line (Nthy-ori 3-1), were detected using quantitative real-time polymerase chain reaction. The 5hmC level was detected in PTC tissues and cell lines using immunohistochemistry and dot blot assay, respectively. After silencing the *TET1* gene with siRNAs in BCPAP and TPC-1 cells, cell proliferation was detected using EdU assay. Transwell assay was used to investigate cell migration and invasion. miRNA and mRNA expression arrays were conducted in TET1-depleted BCPAP cells.

**Results:**

The expression level of TET1 decreased in PTC tissues and cell lines and was consistent with the reduction in the 5hmC level. The knockdown of the *TET1* gene with siRNAs in BCPAP and TPC-1 cells, cell proliferation was detected using EdU assay. Transwell assay was used to investigate cell migration and invasion. miRNA and mRNA expression arrays were conducted in TET1-depleted BCPAP cells. *WNT4*, *FZD4*, *CDK6*, *MCF2L*, and *EDN1* was upregulated as potential target genes of dysregulated miRNAs.

**Conclusion:**

The study showed that TET1 dysfunction inhibited the migration and invasion of BCPAP cells and might have a potential role in the pathogenesis of PTC.

## 1. Introduction

The incidence of thyroid carcinoma (TC), the most common endocrine malignancy, has increased rapidly worldwide in recent years. Furthermore, the prevalence of papillary thyroid carcinoma (PTC), accounting for more than 90% of TC, has also increased more than threefold worldwide in the last 30 years [1, 2]. Although early PTC has a good prognosis, its 5-year survival rate is only 59% in the advanced stage, and the recurrence rate of total PTC is as high as 35% [3]. Therefore, searching for specific molecular markers and exploring the underlying mechanisms of PTC pathogenesis are of great importance for providing new therapeutic targets and improving the prognosis of patients with PTC.

DNA methylation has an essential role in the remodeling of the chromatin structure during development and tissue differentiation [4, 5]. Aberrant DNA methylation, characterized by genome-wide hypomethylation and regional hypermethylation, is common in various forms of cancers. It is closely associated with tumor initiation and progression [6, 7]. In normal cells, DNA methylation is mediated through the coordinated actions of several DNA methyltransferases (DNMTs), which transfer a methyl group from *S*-adenosyl methionine to carbon-5 position of the cytosine residues of CpG dinucleotides [4, 5, 8].

5-Hydroxymethylcytosine (5hmC), an intermediate of DNA demethylation process, is a recently discovered epigenetic modification catalyzed by the ten-eleven translocation (TET) proteins, leading to eventual DNA demethylation [9, 10]. TET family members, including TET1, TET2, and TET3, progressively oxidize 5-methylcytosine (5mC) to 5hmC, 5-formylcytosine, and 5-carboxylcytosine. 5hmC was found to be downregulated in hematological malignancies and a variety of solid tumors such as prostate, breast, liver, and stomach tumors [11–14]. The degradation of TET proteins was found in many malignancies, and the functional experiments confirmed that TET family members acted as tumor-suppressor genes for the conversion of 5mC into 5hmC in cancers. TET2 mutations with decreased 5hmC are associated with myeloid cancers [15]. TET1 is downregulated in prostate and breast cancer tissues. It maintains the expression of tissue inhibitors of metalloproteinase family proteins 2 and 3 by inhibiting their DNA methylation [14]. However, to date, the expression patterns of TET family members and 5hmC in PTC remain unknown. This study aimed to elucidate the biological functions of TET1 and explore the possible microRNA (miRNA) and mRNA expression levels with downregulated TET1 in BCPAP cells.

## 2. Methods

### 2.1. Clinical Specimens

A total of 49 patients with pathologically confirmed PTC were enrolled at the First Affiliated Hospital of Sun Yat-sen University and Sun Yat-sen University Cancer Center (Guangzhou, China) between December 2014 and December 2016. PTC and adjacent normal thyroid tissue samples were obtained and stored at –80°C. FFPE surgical samples previously diagnosed as PTC, and benign thyroid nodules were obtained from the First Affiliated Hospital of Sun Yat-sen University between December 2014 and July 2015. The surgical procedure was performed on all patients, and the final diagnoses were based on the pathological examination. All participants provided informed consent, and the study was approved by the ethics committee of Sun Yat-sen University.

### 2.2. Cell Culture and Transfection

Human thyroid normal cell line Nthy-ori 3-1 and PTC cell lines BCPAP and TPC-1 were kindly given by Dr. Haixia Guan (The First Affiliated Hospital of China Medical University, Shenyang, China). K1, another PTC cell line, was purchased from the European Collection of Cell Cultures (Salisbury, UK). The cells were maintained at 37°C in Dulbecco's modified Eagle's medium (Invitrogen, USA) with 10% fetal bovine serum (FBS). RNA oligoribonucleotides were transfected using Lipofectamine 2000 (Invitrogen, USA) as per manufacturer's instructions. All siRNA oligoribonucleotides and negative control (NC) were obtained from GenePharma (Shanghai, China). The sequences of siRNAs were as follows: TET1 si-RNA1 sense: 5′-GAAGCGAAGAAACCCUUUATT-3′; antisense: 5′-UAAAGGGUUUCUUCGCUUCTT-3′; TET1 si-RNA2 sense: 5′-GCGAAAGGUACAAAUUAAUTT-3′; antisense: 5′-AUUAAUUUGUACCUUUCGCTT-3′, and NC sense: 5′-UUCUCCGAACGUGUCACGUTT-3′; antisense: 5′-ACGUGACACGUUCGGAGAATT-3′.

### 2.3. RNA Isolation and Quantitative Real-Time Polymerase Chain Reaction Analysis

Total RNA was extracted with TRIzol reagent (Life Technologies, USA) according to the manufacturer's instructions. The human TET1, TET2, and TET3 cDNAs were reverse-transcribed using a PrimeScript real-time polymerase chain reaction (RT-PCR) kit (TaKaRa, China) and quantified by SYBR Premix Ex Taq (TaKaRa, China) using a Light Cycler 480 system (Roche Diagnostics, Switzerland). GAPDH was used as a reference for the normalization of the expression of mRNAs. The oligonucleotide sequences of primers were as follows: TET1 forward primer: 5′-CCCTTGGAAATGCCATAGGAA-3′; reverse primer: 5′-GAGAGCCTGCTGGAACTGTTTG-3′; TET2 forward primer: 5′-TAAGGCAGGAGTTTGGCAAGTG-3′; reverse primer: 5′-ACCTGTAGGTGTTTGCCTGTTTAAG-3′; TET3 forward primer: 5′-GCCAACTTCAACATACCCTGGAC-3′; reverse primer: 5′-CACCTGGATGTGGGACTGTGTAA-3′, and GAPDH forward primer: 5′-GCACCGTCAAGGCTGAGAAC-3′; reverse primer: 5′-TGGTGAAGACGCCAGTGGA-3′. The relative expression was quantified using the comparative cycle threshold method (2^−ΔΔCT^).

### 2.4. Western Blot Assay

The proteins were resolved using 10% SDS-PAGE (BioRad, USA), transferred to PVDF membranes (Millipore, USA), and blotted with the following antibodies overnight at 4°C: anti-TET1 (GTX124207, GeneTex, USA), anti-TET2 (ab94580, Abcam, UK), anti-TET3 (GTX121453, GeneTex, USA), and anti-GAPDH (#sc-25778, Santa Cruz, USA). The membranes were incubated with peroxidase-conjugated anti-rabbit secondary antibodies (CST, USA). The reactions were detected by enhanced chemiluminescence (Thermo, USA).

### 2.5. Immunohistochemistry

The paraffin-embedded tissue sections were deparaffinized and hydrated using xylene and graded alcohol to water. Antigen retrieval was performed by incubating the tissue sections with boiled sodium citrate buffer (pH 6.0) for 3 min. The endogenous peroxidase activity was quenched with 3% H_2_O_2_. The slides were blocked with 5% BSA to reduce nonspecific binding and then incubated with 5hmC (#39791, Avtive Motif, USA) primary antibody overnight at 4°C. After incubation with the secondary antibody (Gene Tech, USA) for 30 min at room temperature, the slides were detected with the DAB Enhancer solution (GeneTech, USA) and counterstained with hematoxylin. The images were taken by light microscopy.

### 2.6. Dot Blot Assay

The genomic DNA was extracted with a DNeasy Blood and Tissue kit (Omega, USA) according to the manufacturer's instructions. Then, the DNA was broken into fragments of about 500 bp by sonication. A proper amount of broken DNA samples were boiled at 95°C for 10 min and quickly placed into the ice water for DNA denaturation. The denatured DNA (1-2 *µ*L) was dripped on a nitrocellulose membrane. After washing with 2x SSC buffer and ultraviolet crosslinking, the membrane was dried at 70°C for 1-2 h, blocked, incubated, and subjected to Western blot analysis. To ensure the equal spotting of DNA as a control, the same blot was stained in 0.02% methylene blue liquid.

### 2.7. EdU Assay

EdU assay was performed to assess the cell proliferative ability using the EdU kit (Ribobio, China) following the manufacturer's instructions.

### 2.8. Cell Migration and Invasion Assays

The cells resuspended in 100 *μ*L of serum-free medium were plated in the top chamber of each insert (Corning, USA) with a non-Matrigel-coated membrane for the Transwell migration assay and a Matrigel-coated membrane (BD Bioscience, USA) for the invasion assay. The lower chambers of the inserts were filled with 600 *μ*L of the medium with 10% FBS. After several hours of incubation, the cells that invaded to the lower surface of the insert were fixed, stained, and imaged using a DMI4000B inverted microscope (Leica, Germany).

### 2.9. miRNA Array and mRNA Expression Array

BCPAP cells following siRNA-mediated knockdown of TET1 and control cells were used for gene expression profiling analysis. Total RNA was harvested from the aforementioned cell lines using TRIzol reagent (Invitrogen, USA) and an miRNeasy mini kit (Qiagen, Germany) according to the manufacturer's instructions. For the miRNA array, total RNA was labeled using a miRCURY Array Power Labeling kit (Exiqon, Denmark) and then hybridized on a miRCURY LNA Array (v.18.0, Exiqon, Denmark) using a hybridization system (Nimblegen Systems, USA). Following several washing steps using a wash buffer kit (Exiqon, Denmark), the slides were scanned using an Axon GenePix 4000B microarray scanner (Axon Instruments, USA). For mRNA expression array, total RNA was transcribed into fluorescent cRNA using a Quick Amp Labeling Kit (Agilent Technologies, USA). The labeled cRNA was then hybridized onto the Human Genome Oligo Microarray (4x44K, Agilent Technologies, USA). After the washing steps, the arrays were scanned using the Agilent Scanner G2505C (Agilent Technologies, USA).

The Agilent Feature Extraction software (version 11.0.1.1, Agilent Technologies, USA) was used to analyze the acquired array images. Quantize normalization and subsequent data processing were performed using the GeneSpring GX v12.1 software package (Agilent Technologies, USA). The differentially expressed mRNAs and miRNAs with statistical significance were identified using volcano plot filtering. The threshold used to screen upregulated or downregulated miRNAs and mRNAs was a fold change of ≥2 and ≥1.5, respectively, and *P* values of less than 0.05 were considered statistically significant.

### 2.10. Statistical Analysis

SPSS software (version 21.0) was used for all statistical analyses. The significance of different groups of data was calculated with the two-tailed Students *t*-test or one-way analysis of variance. All data were presented as the mean ± standard deviation (SD) from at least three replicates. *P* < 0.05 was considered statistically significant.

## 3. Results

### 3.1. TET Expression in PTC Tissues and Cell Lines

**Figure 1:**
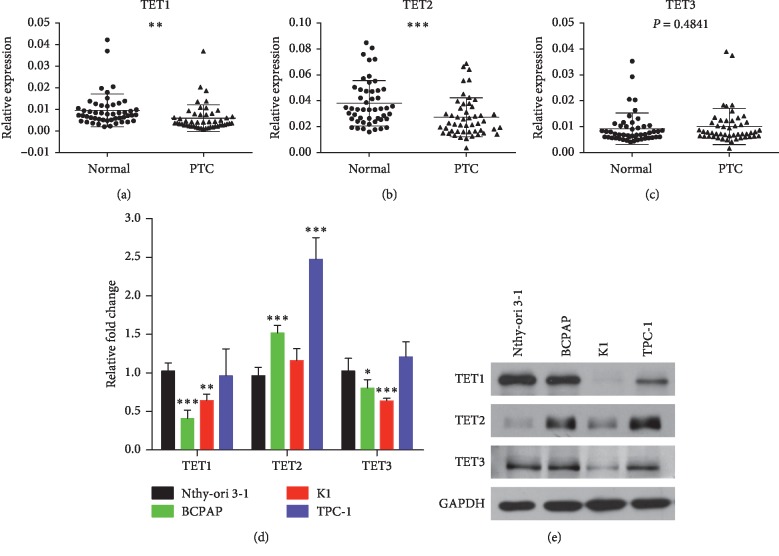
TET expression in PTC tissues and cell lines. (a) Relative expression level of *TET1* was downregulated in PTC tissues compared with matched normal thyroid tissues (*n* = 49). (b) *TET2* gene was significantly lowly expressed in PTC tissues compared with matched normal thyroid tissues (*n* = 49). (c) *TET3* expression was not different in PTC and matched normal thyroid tissues (*n* = 49). (d) Relative expression levels of the TET family in PTC cell lines (BCPAP, K1, and TPC-1) compared with Nthy-ori 3-1 cells detected using qRT-PCR. (e) Expression levels of TET1 and TET3 proteins significantly decreased in PTC cells compared with Nthy-ori 3-1 cells as detected using Western blot assays. The data represented the mean ± SD from three independent experiments. ^*∗*^*P* < 0.05, ^*∗∗*^*P* < 0.01, and ^*∗∗∗*^*P* < 0.001.

The gene expression of *TET1*, *TET2*, and *TET3* was examined using qRT-PCR in clinical tissues. The downregulation of *TET1* in PTC tissues compared with matched normal thyroid tissues was confirmed (Figure 1(a), *P* < 0.01). Meanwhile, the *TET2* gene was significantly lowly expressed in PTC (Figure 1(b), *P* < 0.001). However, the *TET3* expression level was not significantly different in PTC and matched normal thyroid tissues (Figure 1(c), *P*=0.4841). The mRNA expression levels of *TET1* and *TET3* genes significantly decreased in BCPAP and K1 cells (Figure 1(d)). On the contrary, TET2 was highly expressed in PTC cells compared with Nthy-ori 3-1 cells. The expression levels of TET1 and TET3 proteins in PTC cell lines were found to be decreased using Western blot assay (Figure 1(e)).

The expression level of TET1 significantly reduced in both PTC cell lines and clinical tissues. Also, TET3 expression was downregulated in PTC cell lines but had no difference in PTC tissues. However, TET2 expression was not aligned in cells and tissues: strikingly upregulated in PTC cell lines but decreased in PTC tissues. These observations provided a clue that TET1 was the major factor in PTC.

To explore the correlation between the expression of *TET1* and clinicopathological characteristics of patients with PTC, 36 patients were divided into 2 groups (*n* = 16 > mean; *n* = 33 ≤ mean) according to the mean expression level of *TET1*. As shown in Supplementary [Supplementary-material supplementary-material-1], the *TET1* expression level positively correlated with tumor size (*P*=0.027). The data showed that more invasive and advanced-stage cancers had a lower expression level of *TET1*. However, no significant *P* value was found. This was probably due to the small number of clinical samples.

### 3.2. 5hmC Expression in PTC Tissues and Cell Lines

**Figure 2:**
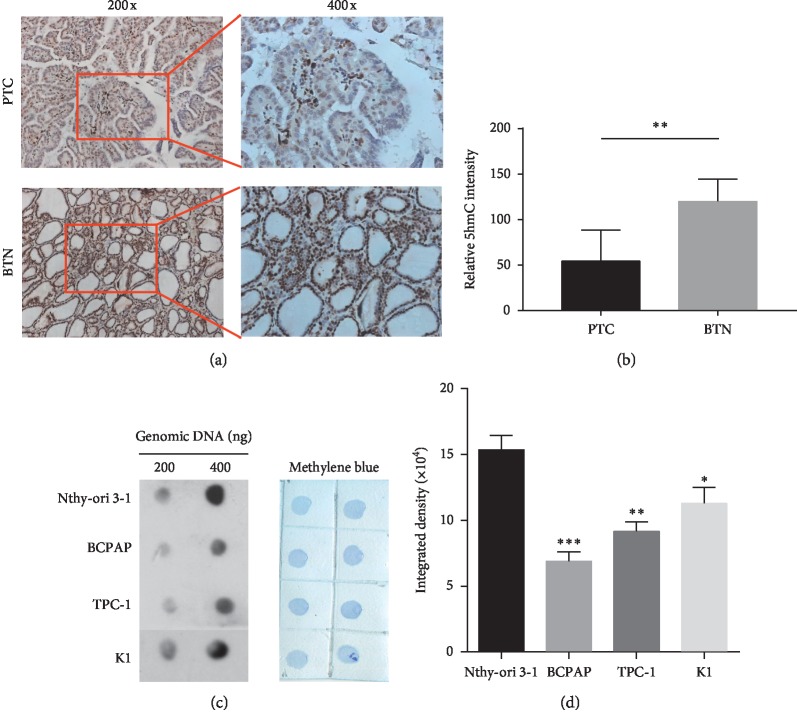
5hmC expression in PTC tissues and cell lines. (a, b) 5hmC level significantly decreased in PTC tissues compared with thyroid benign nodule tissues as detected by immunohistochemical analysis. Brownish-yellow is the color of 5hmC, and blue is the color of hematoxylin-stained nuclei. The staining range and degree of 5hmC were significantly weaker in PTC tissues than in thyroid benign nodule tissues. (c) Dot blot assay showed the expression level of 5hmC in PTC cell lines when the DNA sample size was 200 ng and 400 ng (left). Methylene blue staining of the identical membrane was used as the sample control (right). (d) ImageJ software was used to analyze exposure grayscale; it showed that the expression level of 5hmC in PTC cell lines decreased compared with the normal Nthy-ori 3-1 cells. The data represented the mean ± SD from three independent experiments. ^*∗*^*P* < 0.05, ^*∗∗*^*P* < 0.01, and ^*∗∗∗*^*P* < 0.001.

PTC tissues and benign thyroid nodule tissues were chosen to assess 5hmC levels using immunohistochemical (IHC) analysis. As shown in Figures 2(a) and 2(b), a significant decrease in 5hmC levels was seen in PTC tissues compared with benign thyroid nodules under the same IHC condition (*P*=0.001), suggesting that the loss of 5hmC was a hallmark of PTC. Furthermore, considering that 5hmC was a modification of DNA, global 5hmC expression was detected in the normal thyroid cell line and PTC cell lines using dot blot assay. A previous study revealed that 5hmC was highly expressed in neuronal tissues but lowly expressed in endocrine tissues [16]. Compared with other studies on gastric cancer and laryngeal squamous cell carcinoma [17, 18], the present study showed that detecting 5hmC expression with higher amounts of DNA, such as 200 ng and 400 ng, was more sensitive. The data demonstrated that the 5hmC level decreased in PTC cell lines (BCPAP, TPC-1, and K1) compared with the normal thyroid cell line Nthy-ori 3-1 (Figures 2(c) and 2(d)).

### 3.3. TET1 Inhibited PTC Cell Invasion and Migration

**Figure 3:**
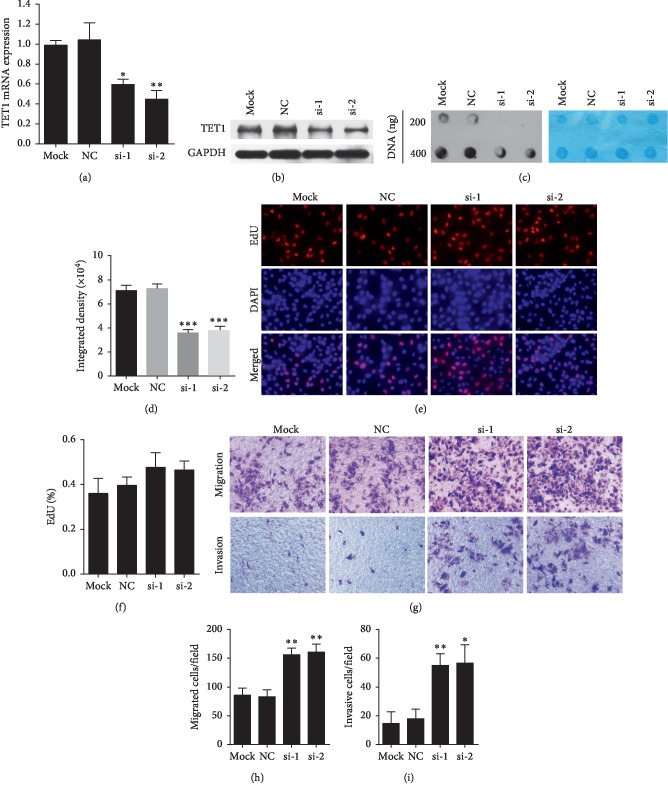
TET1 inhibited the invasion and migration in BCPAP cells. (a, b) TET1 level significantly declined after transfection with si-TET1 RNAs as detected using qRT-PCR and Western blot assays. (c) Dot blot assay showed the expression of 5hmC after 48 h of transfection of si-TET1 RNAs (left). Methylene blue staining of the identical membrane was used as the sample control (right). (d) ImageJ software was used to analyze exposure grayscale and showed that the downregulated TET1 expression level was consistent with the reduction in the 5hmC level. (e) EdU incorporation assay was conducted to examine the potential role of TET1 in cell proliferation after si-TET1 and si-TET2 transfection. Fluorescent images of proliferative cells (red) stained with EdU and nuclei (blue) counterstained with DAPI in BCPAP cells. Magnification, 400x. (f) Quantification of EdU incorporation assay in BCPAP cells. (g) Transwell migration and invasion assays of BCPAP cells after si-TET1 and si-TET2 transfection were performed without and with the Matrigel-coated chamber. Magnification, 200x. (h) Quantification of migrated cells. (i) Diagrams of invasive cells. ^*∗*^*P* < 0.05, ^*∗∗*^*P* < 0.01, and ^*∗∗∗*^*P* < 0.001.

Furthermore, the *TET1* gene was silenced in PTC cells with si-TET1 RNAs, and the biological function of TET1 was explored. BCPAP cells were chosen for the higher background expression of the *TET1* gene. The results of qRT-PCR and Western blot assays demonstrated a significant decline in the TET1 level after transfection (Figures 3(a) and 3(b)). Dot blot assay showed a concomitant decrease in the 5hmC level after 48 h of transfection (Figures 3(c) and 3(d)) in BCPAP cells. Thus, the study confirmed the high transfection efficiency of si-RNA1 and si-RNA2 and suggested that TET1 was the main regulator of 5hmC generation in BCPAP cells.

Then, the study examined the potential role of TET1 in cell proliferation in BCPAP and TPC-1 cells. EdU incorporation assay was conducted to examine the potential role of TET1 in cell proliferation after si-TET1 and si-TET2 transfection. A growth-promoting tendency was observed in the si-TET1 group, but the result had no statistical significance in BCPAP (Figures 3(e) and 3(f)) and TPC-1 cells (Supplementary Figures [Supplementary-material supplementary-material-1] and [Supplementary-material supplementary-material-1]).

To determine whether TET1 could influence the migration and invasion ability of BCPAP and TPC-1 cells, transwell assays without and with the Matrigel-coated chamber were performed. Strikingly, si-TET1 and si-TET2 were found to promote cell migration and invasion (Figures 3(g)–3(i)) in BCPAP cells, but discrepancy results in TPC-1 cells (Supplementary Figures [Supplementary-material supplementary-material-1]–[Supplementary-material supplementary-material-1]). Collectively, the results indicated different cells may have different biology function, and *TET1* downregulation facilitated the migration and invasion in BCPAP cells.

### 3.4. Chip Results after si-TET1

The data suggested that TET1 likely inhibited the migration and invasion of BCPAP cells. The microarray analysis was conducted to understand the specific mechanism involved in this process. The miRNA and gene expression levels between control (NC group) and TET1-depleted (si-TET1 RNA group) BCPAP cells were analyzed ([Supplementary-material supplementary-material-1] and [Supplementary-material supplementary-material-1]).

**Figure 4:**
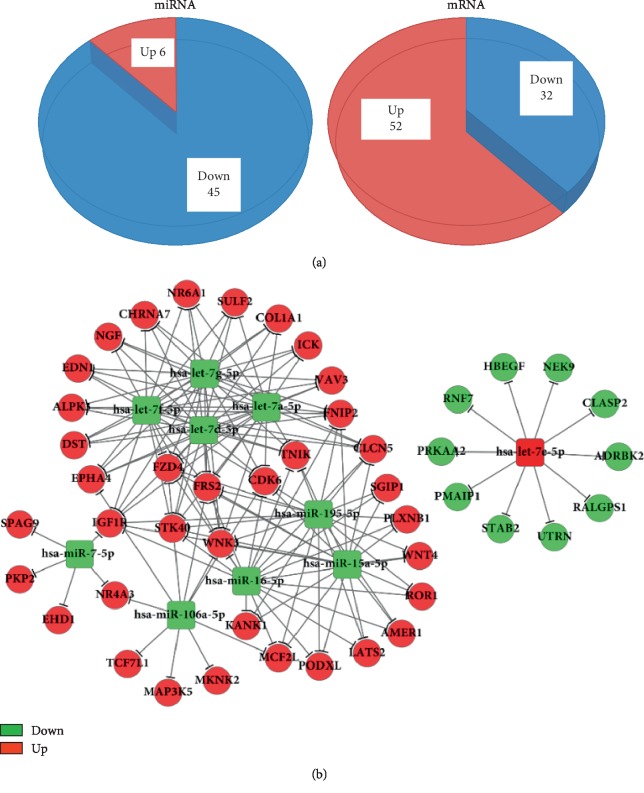
Results of miRNA chip and mRNA expression profiles after si-TET1 in BCPAP cells. (a) The expression of miRNAs and mRNAs was analyzed in NC and si-TET1 RNA groups in BCPAP cells. The fold change of miRNAs and mRNAs were set to be two times and 1.5 times, respectively, indicating obvious differences. The red ones are upregulated, and the green ones are downregulated. (b) Regulatory network map of classical oncogenes miR-7 and miR-15/16 and let-7. The statistically differentially expressed miRNAs are labeled in squares, and the statistically differentially expressed mRNAs are labeled in circles. The upregulated expression is shown in red, and the downregulated expression is shown in green.

The fold change in the differential expression of genes and mRNAs was set to be 1.5 and 2, respectively. Then, the genes and miRNAs with a negative expression trend were picked out. Furthermore, 6 miRNAs were found to be upregulated and 45 downregulated, while 52 mRNAs were upregulated and 32 downregulated (Figure 4(a)). A gene coexpression network was constructed to predict the miRNA targets. Strings between genes and miRNAs showed the potential regulatory relationship (Supplementary [Supplementary-material supplementary-material-1]).

Surprisingly, the expression levels of classic tumor-suppressor genes miR-7 and miR-15/16 significantly decreased after silencing *TET1*, while let-7e expression was upregulated. Simultaneously, the expression levels of potential target genes, including *WNT4* and *FZD4* (parts of the canonical Wnt pathway), significantly increased. The expression of proto-oncogenes *CDK6*, *MCF2L*, and *EDN1* was also upregulated along with *WNT4* and *FZD4* (Figure 4(b)).

## 4. Discussion

The newly discovered TET family members catalyze the conversion of 5mC into 5hmC, greatly promoting DNA epigenetic modification. The expression of 5hmC is generally decreased in human tumors [11–14]. Meanwhile, 5hmC has become an important factor for DNA epigenetic modification with the downregulation of the expression of single or multiple members of the TET family.

This study reported that the expression level of TET1 decreased, and the global 5hmC content reduced in PTC tissues and cell lines. The cancer cells with TET1 knockdown showed lower levels of 5hmC, contributing to aberrant DNA methylation patterns in PTC. TET1 was first identified as one of the MLL fusion partners in leukemias [19]; it showed the highest expression levels in embryonic stem cells and in early embryogenesis [20]. Multiple cell lines of evidence supported the tumor-suppressive function of TET1 proteins in endocrine carcinomas, such as breast cancer and ovarian cancer [21, 22]. The present study also showed a significant reduction of TET1 in PTC as other endocrine carcinomas. Good et al. uncovered an intricate network connecting TET1 to the hypomethylation and activation of cancer-specific oncogenic pathways, including PI3K, EGFR, and PDGF, using bioinformatic analyses in both breast and ovarian cancer cell lines [21]. Duan et al. reported that TET1 expression was undetected in six types of ovarian cancer cell lines, and the ectopic expression of TET1 inhibited colony formation, cell migration, and invasion in SKOV3 and OVCAR3 cells [22]. The present study provided evidence that ectopic TET1 exerted potent antitumor effects by inhibiting the migration and invasion in BCPAP cells, but discrepancy results in TPC-1 cells. PTC cell lines originate from dedifferentiated in vivo human thyroid tumors and have different mutational status. As reported, BCPAP cells showed *BRAF* and *TP53* mutations, and TPC-1 cells harbored the *RET/PTC1* gene rearrangement [23]. The discrepancies of biological function may due to different mutational status of cancer cells, but further study needs to investigate the role of TET1 in different genetic alterations.

miRNAs are an abundant class of 17- to 25-nucleotide, small, noncoding RNAs, identified as important regulators of many diverse biological processes. miRNAs could regulate the expression of protein-coding genes at the posttranscriptional level through binding to the 3′-untranslated region of target mRNAs [24]. Recent studies have focused on the regulating effect of miRNAs in tumorigenesis and progression by acting as oncogenes or tumor suppressors [25, 26]. In the present study, the miRNA array and mRNA expression array indicated that the expression of miR-7, miR-15/16 cluster (including miR-195, miR-15a, and miR-16), and let-7 family (let-7a, let-7d, let-7f, and let-7g) was significantly downregulated, while the expression of let-7e was upregulated after siRNA-TET1 treatment in BCPAP cells. A previous study reported that miR-7 functioned as a tumor suppressor and played an important role in inhibiting tumorigenesis and reversing the metastasis of hepatocellular carcinoma (HCC) through the PI3K/Akt/mTOR-signaling pathway *in vitro* and *in vivo* [26]. The miR-15 family members are known as tumor suppressors in breast cancer, prostate cancer, and HCC [27–29]. The let-7 family is considered a tumor suppressor because it inhibits the expression of multiple oncogenes, including *RAS*, *MYC*, and *HMGA2* [30, 31]. The study indicated that the expression of let-7e, with the discrepant expression as other let-7 family members, was upregulated in BCPAP cells after siRNA-TET1. Similarly, a previous study reported that the expression level of let-7e increased in tissue and sera specimens of PTC [32].

In the coexpression network, the expression levels of *WNT4* and *FZD4* were found to be significantly increased as the target genes of dysregulated miRNAs after silencing TET1 in BCPAP cells. WNT4 is an extracellular ligand, and FZD4 is a cell membrane receptor of the Wnt pathway, which plays a key role in the activation of the Wnt signaling pathway [33]. The present study also found that the expression of proto-oncogenes *CDK6*, *MCF2L*, and *EDN1* was upregulated along with *WNT4* and *FZD4*. The expression of *CDK6*, as a proto-oncogene, was upregulated in numerous tumors [34, 35]. The overexpression of the *MCF2L* gene, encoding guanosine conversion factor, could activate the key regulatory factors of the Rho GTP family (Cdc42 and Rac1) and promote tumor metastasis in breast cancer cells [36]. The overexpression of EDN1 in zebrafish could promote cell proliferation, invasion, and metastasis and induce liver cancer [37]. The findings indicated that specific tumor-related miRNAs and the expression of their potential target genes were altered in TET1-depleted BCPAP cells. However, the relationship of TET1 with tumorigenesis deserves further exploration.

Taken together, the results suggested that the downregulated expression of TET1 might be correlated with the low expression of 5hmC and exerted a tumor-suppressive function in the BCPAP cell line. Specific miRNAs and mRNAs were dysregulated in BCPAP cells with the downregulated expression of TET1. These findings provided us new insights into the mechanism of thyroid cancer.

## Data Availability

All data used to support the findings of this study are available from the corresponding author upon request.

## Supplementary Materials

Supplementary Materials Supplementary Figure 1: the biological function of TET1 in TPC-1 cells. Supplementary Figure 2: coexpression network of miRNA chips and mRNA expression profiles after si-TET1 in BCPAP cells. Supplementary Table 1: association between TET1 relative expression and clinicopathologic features in PTC. Supplementary Table 2: miRNA expression profiling data between control and TET1-depleted BCPAP cells. Supplementary Table 3: gene expression profiling data between control and TET1-depleted BCPAP cells.
