# Supplementary material for: TET1 is a Tumor Suppressor That Inhibits Papillary Thyroid Carcinoma Cell Migration and Invasion
Source: Int J Endocrinol. 2020 Feb 8;2020:3909610. doi: 10.1155/2020/3909610 (PMC7031722; doi:10.1155/2020/3909610)
Supplement: Supplementary Materials — Supplementary Figure 1: the biological function of TET1 in TPC-1 cells. Supplementary Figure 2: coexpression network of miRNA chips and mRNA expression profiles after si-TET1 in BCPAP cells. Supplementary Table 1: association between TET1 relative expression and clinicopathologic features in PTC. Supplementary Table 2: miRNA expression profiling data between control and TET1-depleted BCPAP cells. Supplementary Table 3: gene expression profiling data between control and TET1-depleted BCPAP cells. [file 3909610.f1.pdf]

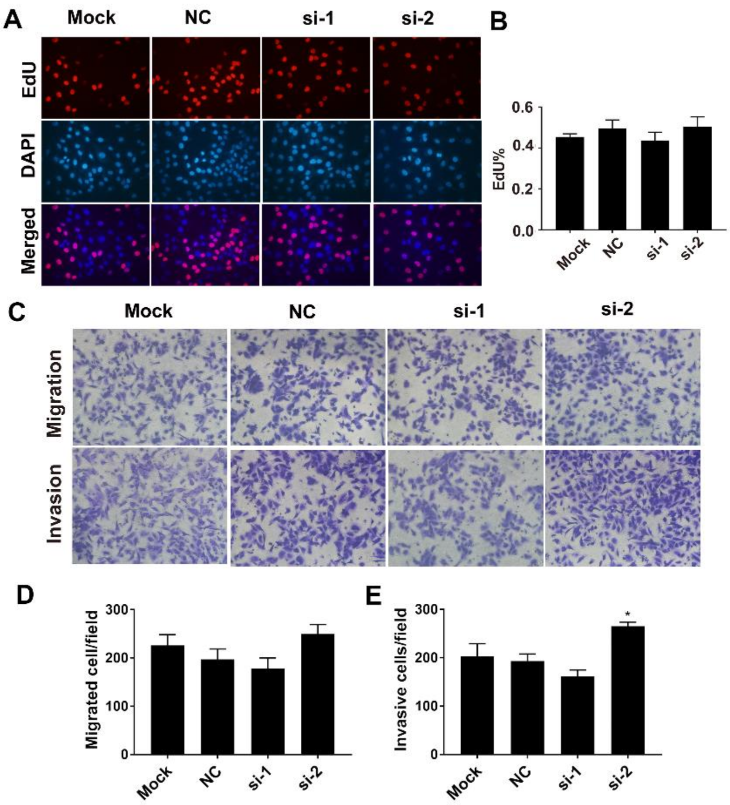

Supplementary Figure 1. The biological function of TET1 in TPC-1 cells. (A) EdU incorporation assay was conducted to examine the potential role of TET1 in cell proliferation after si-TET1 and si-TET2 transfection. Fluorescent images of proliferative cells (red) stained with EdU and nuclei (blue) counterstained with DAPI in BCPAP cells. Magnification, 400×. **(B)** Quantification of EdU incorporation assay in BCPAP cells. **(C)** Transwell migration and invasion assays of BCPAP cells after si-TET1 and si-TET2 transfection were performed without and with the Matrigel-coated chamber. Magnification, 200×. (**D)** Quantification of migrated cells. **(E)** Diagrams of invasive cells. $^{*}P < 0.05$.

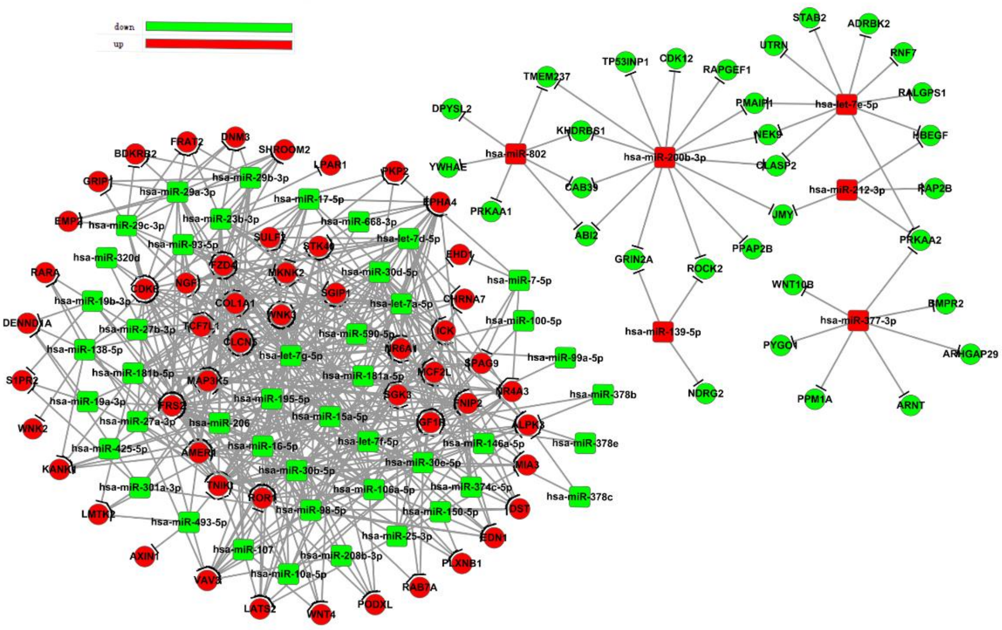

Supplementary Figure 2. Co-expression network of miRNAs chip and mRNAs expression profile after si-TET1. The expression of miRNA and mRNA was analyzed between NC group and si-TET1 RNA group in BCPAP cells. The statistically differently expressed miRNAs were labeled in squares, and the statistically differently expressed mRNAs was labeled in circles. The expression was up-regulated in red, and down-regulated in green.

Supplementary Table 1 Association between TET1 relative expression and clinicopathologic features in PTC

Characteristics	TET1		*P*-value
	Low (%)	High (%)	
**Age (years)**			0.226
＜45	26(78.8)	10(62.5)	
≥45	7(21.2)	6(37.5)	
**Gender**			0.165
Male	8(24.2)	7(43.8)	
Female	25(75.8)	9(56.2)	
**Tumor size**			0.027*
≤2cm	25(78.1)	8(47.1)	
＞2cm	7(21.8)	9(52.9)	
**Extrathyroidal invasion**			0.884
Negative	11(33.3)	5(31.4)	
Positive	22(66.7)	11(68.6)	
**Lymphatic metastasis**			0.46
Negative	7(30.4)	2(7.7)	
Positive	16(69.6)	24(92.3)	
**TNM stage**			0.593
I and II	32(97)	15(93.8)	
III and IV	1(3)	1(6.2)	

The TNM stage base on Eighth Edition of American Joint Committee on Cancer (AJCC) TNM Staging System of differentiated thyroid cancer. *$P<0.05$ was considered significant.

Supplementary Table 2: miRNA Expression Profiling Data between control and TET1-depleted

	mRNAs	Fold change	Normalized		P-value
ID	Name	T-si vs T-mo	T-mo	T-si	Test vs Control
42638	hsa-miR-23a-5p	1.49104898	0.244057052	0.363901019	0.070032103
17519	ebv-miR-BART1-3p	N/A	N/A	N/A	N/A
46507	hsa-miR-921	0.17597165	0.678288431	0.119359534	0.002453128
17928	hsa-miR-181a-2-3p	0.472750235	0.215530903	0.101892285	0.045911202
11134	hsa-miR-502-5p	1.566829352	0.107765452	0.168850073	0.045757314
42812	hsa-miR-508-5p	1.131380318	1.435816165	1.624454148	0.179585193
42918	hsa-miR-19b-2-5p	N/A	N/A	N/A	N/A
42516	kshv-miR-K12-12-5p	1.492540029	0.025356577	0.037845706	0.144101925
46752	hsa-miR-1270	0.475079054	0.183835182	0.087336245	0.071115731
46427	hsa-miR-1248	1.705760033	0.066561014	0.113537118	0.854252363
11052	hsa-miR-31-5p	2.364123555	1.264659271	2.989810771	0.878550049
42696	hsa-miR-943	1.174004881	0.42155309	0.494905386	0.354996583
42754	hsa-miR-586	N/A	N/A	0.011644833	N/A
42818	hsa-miR-597-5p	0.793786589	2.171156894	1.723435226	0.457147529
42810	hsa-miR-149-5p	0.734788937	0.142630745	0.104803493	0.586702973
42591	hsa-miR-634	1.035119336	0.399366086	0.413391557	0.18406124
17585	ebv-miR-BART20-3p	0.114810771	0.025356577	0.002911208	N/A
42848	hsa-miR-1180-3p	0.638946902	0.072900158	0.04657933	0.960239966
17506	hsa-miR-24-3p	1.907296508	8.858954041	16.89665211	0.728608282
42795	kshv-miR-K12-3-5p	0.847833389	0.412044374	0.349344978	0.943920129
42485	ebv-miR-BART10-5p	1.033296943	0.025356577	0.026200873	0.439927373
46633	hsa-miR-1267	0.423122843	0.282091918	0.119359534	0.225085575
42896	hsa-miR-450b-5p	0.122464823	0.047543582	0.005822416	0.284475621
28480	hsa-miR-504-5p	N/A	0.006339144	N/A	N/A
42681	hsa-miR-1307-3p	0.472364317	0.110935024	0.052401747	N/A
42610	hcmv-miR-UL36-3p	N/A	N/A	0.011644833	N/A
10919	hsa-miR-103a-3p	1.566321246	3.033280507	4.751091703	0.788093663
46541	hsa-miR-1225-5p	N/A	N/A	N/A	N/A
5250	hsa-miR-105-5p	0.393636931	0.022187005	0.008733624	0.48962413
42734	hsa-miR-2110	N/A	0.003169572	N/A	N/A
46411	hsa-miR-1203	N/A	N/A	N/A	N/A
46747	hsa-miR-1263	N/A	0.025356577	N/A	N/A
146190	hsa-miR-3927-3p	0.667989943	0.034865293	0.023289665	N/A
146061	hsa-miR-1914-3p	N/A	N/A	N/A	N/A
146138	hsa-miR-1909-5p	N/A	N/A	0.014556041	N/A
146117	hsv1-miR-H6-3p	0.523489528	0.611727417	0.320232897	0.596213602
145831	hsa-miR-625-5p	2.181404658	0.025356577	0.055312955	0.84701133
145963	hsa-miR-646	N/A	N/A	N/A	N/A
145977	hsa-miR-1247-5p	1.44838204	0.16481775	0.238719068	0.52799145
145675	hsa-miR-501-5p	1.211253639	0.507131537	0.61426492	0.513846967
145838	hsa-miR-125b-1-3p	0.74297926	0.497622821	0.369723435	0.265832166
145690	hsa-miR-512-5p	0.114810771	0.076069731	0.008733624	0.363536305
145678	hsa-miR-150-5p	0.174441172	0.817749604	0.142649199	0.155876801
145715	hsa-miR-648	9.184861718	0.003169572	0.029112082	N/A
147199	hsa-miR-27b-3p	1.521378754	1.337559429	2.034934498	0.898409831
147535	hsa-miR-4309	N/A	0.025356577	N/A	N/A
147892	hsa-miR-3152-3p	N/A	N/A	N/A	N/A
147611	hsa-miR-4305	1.251909234	0.462757528	0.579330422	0.057746225
147627	hsa-miR-4294	N/A	N/A	N/A	N/A

147770	hsa-miR-4276	N/A	N/A	N/A	N/A
147165	hsa-let-7b-5p	2.602377487	0.722662441	1.880640466	0.606061957
147705	hsa-miR-4320	0.476906281	0.329635499	0.15720524	0.010172972
147848	hsa-miR-466	N/A	N/A	N/A	N/A
147900	hsv2-miR-H6-3p	0.35795741	3.57844691	1.280931587	0.152827557
147891	hsa-miR-3175	0.089326563	31.74326466	2.835516739	0.050831136
147186	hsa-miR-200b-3p	1.420380343	1.467511886	2.084425036	0.752181658
148493	hsa-miR-3613-3p	1.050348898	67.77812995	71.19068413	0.866831901
148231	hsa-miR-3918	N/A	N/A	0.002911208	N/A
148635	hsa-miR-933	1.067740175	0.253565769	0.270742358	0.255646805
147660	hsa-miR-4296	N/A	N/A	N/A	N/A
147709	hsa-miR-3185	N/A	N/A	N/A	N/A
148251	hsa-miR-3689a-5p/hsa-miR-3689b-5p/hsa-	0.612324115	0.028526149	0.017467249	0.962208639
148599	hsa-miR-3680-5p	2.143134401	0.152139461	0.326055313	0.026860398
148665	hsa-miR-510-5p	N/A	0.006339144	N/A	N/A
148363	hsa-miR-3652	N/A	0.034865293	N/A	N/A
147703	hsa-miR-3181	N/A	N/A	N/A	N/A
148062	hsa-miR-3667-3p	2.099396964	0.022187005	0.04657933	N/A
148206	hsa-miR-3664-5p	1.47613849	0.088748019	0.131004367	0.839011952
148306	hsa-miR-381-3p	N/A	N/A	N/A	N/A
146024	hsa-miR-383-5p	0.442234083	0.085578447	0.037845706	0.001694175
30442	hsa-miR-802	0.714378134	0.085578447	0.061135371	0.537743022
168574	hsa-miR-4428	N/A	0.009508716	N/A	N/A
168970	hsa-miR-5590-5p	N/A	N/A	N/A	N/A
168938	hsa-miR-5692a	N/A	N/A	N/A	N/A
168825	hsa-miR-4729	N/A	0.006339144	N/A	N/A
168640	hsa-miR-4475	0.838554622	2.003169572	1.679767103	0.4772033
168874	hsa-miR-4535	N/A	N/A	N/A	N/A
168656	hsa-miR-5002-3p	0.696092426	5.236133122	3.644832606	0.873166725
168861	hsa-miR-4754	0.5357836	0.076069731	0.040756914	0.706461844
168667	hsa-miR-4999-3p	N/A	N/A	N/A	N/A
168910	hsa-miR-4735-5p	1.593292339	0.310618067	0.494905386	0.013109072
168664	hsa-miR-5683	N/A	N/A	N/A	N/A
168766	hsa-miR-4772-5p	N/A	N/A	N/A	N/A
168569	hsa-miR-5088-5p	0.211958347	0.041204437	0.008733624	N/A
168883	hsa-miR-3689d	N/A	N/A	N/A	N/A
168669	hsa-miR-5693	N/A	0.006339144	N/A	N/A
168791	hsa-miR-4785	N/A	N/A	N/A	N/A
168804	hsa-miR-4761-3p	N/A	N/A	N/A	N/A
169368	hsa-miR-3529-3p	N/A	N/A	N/A	N/A
169178	hsa-miR-4665-5p	N/A	N/A	N/A	N/A
169221	hsa-miR-4748	N/A	0.015847861	N/A	N/A
169076	hsa-miR-345-3p	N/A	N/A	N/A	N/A
169346	hsa-miR-5195-3p	N/A	N/A	N/A	N/A
169031	hsa-miR-4726-5p	1.339512978	2.247226624	3.010189229	0.168237719
169276	hsa-miR-4474-3p	N/A	N/A	N/A	N/A
169170	hsa-miR-4472	1.274913641	0.212361331	0.270742358	0.163091566
169168	hsa-miR-5580-3p	1.887999353	0.057052298	0.107714702	0.170449824
169335	hsa-miR-4699-5p	0.59120949	0.275752773	0.163027656	0.047536113
169043	hsa-miR-4462	0.344432314	0.025356577	0.008733624	0.094033435
169136	hsa-miR-5006-3p	1.224648229	0.323296355	0.395924309	0.554731391

169243	hsa-miR-3682-5p	2.725829929	0.098256735	0.26783115	0.120561205
169205	hsa-miR-4640-3p	N/A	0.006339144	N/A	N/A
169078	hsa-miR-548ai/hsa-miR-570-5p	N/A	N/A	N/A	N/A
169130	hsa-miR-4764-3p	0.776364755	5.489698891	4.262008734	0.462637022
169358	hsa-miR-4417	0.823751095	3.933438986	3.240174672	0.235021324
169278	hsa-miR-4525	N/A	N/A	N/A	N/A
169167	hsa-miR-4451	0.665012469	2.04437401	1.359534207	0.035546967
169086	hsa-miR-4786-5p	N/A	0.04437401	N/A	N/A
169398	hsa-miR-5096	0.485237978	0.167987322	0.081513828	0.021057106
169386	hsa-miR-4427	N/A	N/A	N/A	N/A
28019	hsa-miR-10a-3p	0.721667706	0.088748019	0.064046579	0.309948487
28191	hsa-miR-30e-5p	0.715403802	3.153724247	2.256186317	0.119294697
42959	hsa-miR-514a-3p	3.17937521	0.041204437	0.131004367	0.333579948
46415	hsa-miR-548l	0.635875042	0.041204437	0.026200873	0.079731948
46558	hsa-miR-1268a/hsa-miR-1268b	N/A	N/A	N/A	N/A
42756	hsa-miR-548d-5p	3.36778263	0.009508716	0.03202329	0.904474789
17419	kshv-miR-K12-9-3p	N/A	N/A	N/A	N/A
11260	hsa-miR-151a-5p	2.838957258	0.313787639	0.890829694	0.431351155
42598	hsa-miR-452-3p	N/A	0.003169572	N/A	N/A
31517	hsa-miR-561-3p	N/A	N/A	N/A	N/A
11113	hsa-miR-448	N/A	0.009508716	N/A	N/A
14313	hsa-miR-499a-5p	1.734918324	0.057052298	0.098981077	0.507052562
11181	hsa-miR-95-3p	1.12259421	0.028526149	0.03202329	0.210917881
17470	kshv-miR-K12-2-5p	0.631459243	0.050713154	0.03202329	0.96094343
42811	hsa-miR-542-5p	0.174949747	0.133122029	0.023289665	0.019716398
46796	hsa-miR-1302	N/A	N/A	N/A	N/A
17289	hsa-miR-616-5p	0.918486172	0.057052298	0.052401747	0.718294917
10954	hsa-miR-147a	1.951783115	0.025356577	0.049490539	0.967010949
46875	hsa-miR-2276-3p	N/A	N/A	N/A	N/A
11018	hsa-miR-218-5p	2.602377487	0.019017433	0.049490539	0.109023588
11077	hsa-miR-363-3p	N/A	N/A	0.008733624	N/A
46924	hsa-miR-1252-5p	1.102183406	0.095087163	0.104803493	0.930631294
46454	hsa-miR-520g-3p	N/A	0.025356577	N/A	N/A
42686	hsa-miR-136-3p	N/A	N/A	N/A	N/A
17302	hsa-miR-578	1.102183406	0.015847861	0.017467249	0.327392238
42932	hsa-miR-920	1.850681092	0.212361331	0.3930131	0.154293925
13485	hsa-miR-10a-5p	0.64294032	0.063391442	0.040756914	0.609250132
46791	hsa-miR-1204	N/A	N/A	0.180494905	N/A
46750	hsa-miR-1254	N/A	N/A	0.023289665	N/A
46624	hsa-miR-1236-3p	2.331541821	0.041204437	0.096069869	0.038239439
46917	hsa-miR-205-5p	1.999058139	0.053882726	0.107714702	0.027542661
42769	hsa-let-7b-3p	0.533314551	0.196513471	0.104803493	0.055798094
11240	hsa-miR-409-3p	N/A	N/A	N/A	N/A
42964	hsa-miR-7-2-3p	0.512821446	0.380348653	0.195050946	0.419437572
46803	hsa-miR-503-5p	N/A	N/A	N/A	N/A
146105	hsa-miR-1539	1.292684242	0.085578447	0.11062591	0.334089575
146077	hsv2-miR-H3	3.673944687	0.009508716	0.034934498	0.363881374
42838	hsa-miRPlus-C1076	1.836972344	0.009508716	0.017467249	0.801047311
146152	hsa-miR-3179	0.612324115	0.009508716	0.005822416	N/A
145745	hsa-miR-335-3p	1.457663194	3.860538827	5.627365357	0.063991541

145697	hsa-miR-367-5p	N/A	N/A	N/A	N/A
145701	hsa-miR-668-3p	0.911329137	2.440570523	2.224163028	0.232229676
145843	hsa-miR-330-5p	1.224648229	0.199683043	0.244541485	0.701682812
145754	hsa-miR-523-3p	0.524849241	0.04437401	0.023289665	0.175116945
145696	hsa-miR-655-3p	1.695666779	0.041204437	0.069868996	0.015435059
145638	hsa-miR-29a-5p	1.779165522	0.453248811	0.806404658	0.825973621
145741	hsa-miR-545-3p	N/A	N/A	0.07860262	N/A
147827	hsa-miR-3130-3p	N/A	N/A	N/A	N/A
147820	hsa-miR-3133	1.262554008	0.998415214	1.26055313	0.974764477
147845	hsa-miR-3173-3p	0.262424621	0.04437401	0.011644833	0.206435767
145897	hsa-miR-92b-3p	0.688864629	0.152139461	0.104803493	0.147953634
147195	hsa-miR-18a-5p	0.086108079	0.101426307	0.008733624	N/A
147203	hsa-miR-302a-3p	4.652769366	0.434231379	2.020378457	0.047750212
147865	hsa-miR-4330	1.224648229	0.019017433	0.023289665	0.789407905
147870	hsa-miR-548s	N/A	N/A	N/A	N/A
147919	kshv-miR-K12-12-3p	N/A	N/A	0.011644833	N/A
147837	hsa-miR-3119	2.755458515	0.028526149	0.07860262	0.886848162
147981	hsa-miR-4325	0.237028689	0.098256735	0.023289665	0.098215536
147562	hsa-miR-4253	0.612324115	0.009508716	0.005822416	N/A
147846	hsa-miR-4313	1.224648229	0.019017433	0.023289665	0.110859586
147748	hsv1-miR-H12	N/A	N/A	N/A	N/A
148430	hsa-miR-374c-5p	0.958173846	0.513470681	0.491994178	0.335558523
148495	hsa-miR-3915	0.789140324	2.228209192	1.758369723	0.095368145
148335	hsa-miR-3606-5p	121.2401747	0.003169572	0.384279476	0.05180655
148208	hsa-miR-3610	N/A	N/A	N/A	N/A
148562	hsa-miR-128-1-5p	N/A	N/A	N/A	N/A
147715	hsa-miR-4251	0.222663314	0.209191759	0.04657933	0.170507358
148042	hsa-miR-3662	N/A	N/A	0.017467249	N/A
148332	hsa-miR-3692-5p	N/A	N/A	N/A	N/A
147334	hsa-miR-3613-5p	N/A	N/A	0.066957787	N/A
147722	hsa-miR-4306	0.525051417	4.114104596	2.160116448	0.018836493
147718	hsv1-miR-H3-5p	N/A	N/A	0.040756914	N/A
148040	hsa-miR-1247-3p	N/A	N/A	N/A	N/A
147595	hsa-miR-3178	N/A	N/A	N/A	N/A
147712	hsv1-miR-H1-5p	N/A	N/A	0.034934498	N/A
148580	hsa-miR-134-3p	N/A	N/A	N/A	N/A
148453	hsa-miR-3074-5p	N/A	N/A	N/A	N/A
146094	hsa-miR-1911-3p	N/A	N/A	N/A	N/A
46592	hsa-miR-548j-5p	N/A	N/A	N/A	N/A
27565	hsa-miR-423-5p	0.540062262	1.530903328	0.826783115	0.084587496
168805	hsa-miR-4478	1.914810155	0.561014263	1.074235808	0.800547979
168648	hsa-miR-4687-5p	1.625013996	0.041204437	0.066957787	0.992384608
46474	hsa-miR-766-5p	N/A	N/A	0.049490539	N/A
168799	hsa-miR-4710	N/A	N/A	0.017467249	N/A
168591	hsa-miR-4789-3p	N/A	N/A	0.069868996	N/A
168675	hsa-miR-4433a-3p	N/A	N/A	N/A	N/A
168885	hsa-miR-5194	N/A	N/A	N/A	N/A
168673	hsa-miR-4656	N/A	N/A	N/A	N/A
168814	hsa-miR-4463	N/A	N/A	0.002911208	N/A
168744	hsa-miR-4746-5p	N/A	N/A	N/A	N/A
168628	hsa-miR-5695	N/A	N/A	N/A	N/A
168742	hsa-miR-4722-3p	N/A	N/A	0.034934498	N/A

168933	hsa-miR-5581-3p	0.832223167	8.808240887	7.330422125	0.443390625
168619	hsa-miR-1260b	0.810018509	145.6038035	117.9417758	0.112329016
168568	hsa-miR-1290	0.298209796	1.464342314	0.436681223	0.067939126
168620	hsa-miR-548au-3p	N/A	0.006339144	N/A	N/A
168954	hsa-miR-5580-5p	0.992958024	0.117274168	0.116448326	0.30315019
168811	hsa-miR-1255b-2-3p	2.046247674	1.001584786	2.049490539	0.151363504
168801	hsa-miR-4477b	0.2922456	0.069730586	0.020378457	0.054603484
168697	hsa-miR-3189-5p	N/A	N/A	0.017467249	N/A
169333	hsa-miR-3194-3p	N/A	N/A	N/A	N/A
169279	hsa-miR-499b-3p	N/A	N/A	N/A	N/A
169117	hsa-miR-4759	N/A	N/A	N/A	N/A
169195	hsa-miR-4798-3p	N/A	N/A	N/A	N/A
168988	hsa-miR-4799-5p	N/A	N/A	N/A	N/A
169151	hsa-miR-4526	N/A	N/A	N/A	N/A
169147	hsa-miR-548am-5p/hsa-miR-548au-5p/hsa-miR-548c-	N/A	N/A	0.005822416	N/A
169174	hsa-miR-4709-5p	N/A	N/A	N/A	N/A
169241	hsa-miR-5571-3p	N/A	N/A	N/A	N/A
169211	hsa-miR-5704	0.473351936	8.253565769	3.906841339	0.432925118
169188	hsa-miR-4443	2.675207779	5.296354992	14.16885007	0.046324238
169336	hsa-miR-17-5p	1.558643201	1.359746434	2.119359534	0.641291282
169354	hsa-miR-4493	N/A	N/A	N/A	N/A
169090	hsa-miR-5186	N/A	N/A	N/A	N/A
169222	hsa-miR-4760-5p	1.662022597	0.066561014	0.11062591	0.624760865
169176	hsa-miR-5587-5p	N/A	N/A	N/A	N/A
169353	hsa-miR-5582-3p	N/A	0.019017433	N/A	N/A
169193	hsa-miR-4725-5p	0.813516324	0.332805071	0.270742358	0.567790268
169317	hsa-miR-3614-5p	N/A	N/A	0.005822416	N/A
169032	hsa-miR-4737	N/A	N/A	N/A	N/A
169412	hsa-miR-1260a	0.621999225	14.94453249	9.295487627	0.020310757
17608	hsa-miR-425-5p	1.184714048	0.437400951	0.518195051	0.469577052
30787	hsa-miR-125b-5p	1.207477543	3.334389857	4.026200873	0.71273492
11159	hsa-miR-518e-3p	N/A	N/A	N/A	N/A
42613	ebv-miR-BART19-5p	1.607350801	0.012678288	0.020378457	0.997522958
17541	ebv-miR-BART1-5p	0.60275655	0.101426307	0.061135371	0.569738298
11084	hsa-miR-372-3p	N/A	0.041204437	N/A	N/A
17358	ebv-miR-BART16	1.764934212	0.161648177	0.285298399	0.02075781
17312	hsa-miR-592	N/A	N/A	N/A	N/A
11171	hsa-miR-521	N/A	0.028526149	N/A	N/A
46301	hsa-miR-621	N/A	N/A	N/A	N/A
42524	hsa-miR-21-3p	1.249514183	0.624405705	0.780203785	0.553216287
14301	hsa-miR-361-5p	1.598970452	0.868462758	1.388646288	0.9635871
46689	hsa-miR-1229-3p	0.688864629	0.038034865	0.026200873	0.062802106
46647	hsa-miR-1324	N/A	N/A	N/A	N/A
42970	hsa-miR-744-3p	1.246516947	0.04437401	0.055312955	0.74551103
27740	hsa-miR-574-5p	1.62188381	0.500792393	0.812227074	0.468950238
11141	hsa-miR-509-3p	1.669974858	0.034865293	0.058224163	0.417203352
42766	hsa-miR-554	0.459243086	0.126782884	0.058224163	0.669954323
46501	hsa-miR-1305	N/A	N/A	N/A	N/A
17272	hsa-miR-551a	0.406924253	0.250396197	0.101892285	0.177257069
42965	hsa-miR-424-5p	1.233395716	0.110935024	0.136826783	0.989605987

42683	hsa-miR-922	0.172216157	0.050713154	0.008733624	0.109321345
42865	hsa-miR-181a-5p	1.349258662	2.351822504	3.173216885	0.76666281
42667	hsa-miR-302b-5p	0.384903819	3.857369255	1.484716157	0.165106942
10138	hsa-miR-130a-3p	2.258772981	3.809825674	8.605531295	0.646264874
46381	hsa-miR-1298-5p	N/A	0.019017433	N/A	N/A
42523	hsa-miR-26b-3p	2.170967315	0.034865293	0.075691412	0.348262157
42892	hsa-miR-450b-3p	0.083498743	0.034865293	0.002911208	0.06971137
42824	ebv-miR-BART9-5p	0.483413775	0.12044374	0.058224163	0.017134743
17606	kshv-miR-K12-4-5p	N/A	N/A	0.005822416	N/A
46925	hsa-miR-1277-3p	0.834987429	0.034865293	0.029112082	0.945255025
46832	hsa-miR-1202	N/A	0.022187005	N/A	N/A
146064	hsa-miR-718	0.947188865	0.202852615	0.192139738	0.51723498
146091	hsa-miR-1914-5p	1.224648229	0.019017433	0.023289665	0.381362824
42780	hsa-miR-1307-5p	1.016895404	0.354992076	0.360989811	0.311338232
145934	hsa-miRPlus-B1114	0.814994772	0.22503962	0.183406114	0.52078176
146020	hsa-miR-449c-3p	N/A	N/A	0.049490539	N/A
145990	ebv-miR-BART21-3p	3.444323144	0.012678288	0.043668122	0.181927579
145717	hsa-miR-516a-3p/hsa-miR-516b-3p	N/A	N/A	0.008733624	N/A
145984	ebv-miR-BART2-3p	0.425339234	0.472266244	0.200873362	0.628137738
146009	hsa-miR-376a-3p	1.056259098	0.063391442	0.066957787	0.469949411
145785	hsa-miR-526b-3p	N/A	N/A	N/A	N/A
145641	hsa-miR-369-5p	2.143134401	0.019017433	0.040756914	0.519548409
145842	hsa-miR-658	0.612324115	0.028526149	0.017467249	0.550747944
146086	hsa-miR-30a-5p	1.343672078	6.881141046	9.245997089	0.973192408
145905	hsa-miR-518a-5p/hsa-miR-527	0.087474874	0.066561014	0.005822416	0.059348078
147854	hsa-miR-548u	N/A	N/A	N/A	N/A
147835	hsa-miR-3143	N/A	0.003169572	N/A	N/A
147796	hsa-miR-4327	N/A	N/A	N/A	N/A
147776	hsa-miR-4317	0.830630973	0.364500792	0.302765648	0.108397383
147840	hsv2-miR-H9-3p	0.837443274	0.107765452	0.090247453	0.967631539
147901	kshv-miR-K12-7-5p	0.183697234	0.031695721	0.005822416	N/A
147941	hsa-miR-3160-3p	N/A	N/A	N/A	N/A
147767	hsa-miR-4279	0.943503305	0.814580032	0.768558952	0.390872692
147832	hsa-miR-4326	1.920471086	0.034865293	0.066957787	0.010346842
147851	hsa-miR-3201	0.472490483	1.318541997	0.622998544	0.081102181
147706	hsa-miR-4255	0.846120595	0.522979398	0.442503639	0.66587998
147821	hsa-miR-3169	N/A	N/A	N/A	N/A
147654	hsa-miR-3138	0.94035489	0.133122029	0.125181951	0.486484582
147623	hsa-miR-4304	N/A	0.183835182	N/A	N/A
147836	hsv2-miR-H7-5p	1.322488168	1.765451664	2.334788937	0.651941687
148683	hsa-miR-888-3p	N/A	N/A	N/A	N/A
148467	hsa-miR-3939	N/A	N/A	N/A	N/A
147986	hsa-miRPlus-K1303*	0.46313027	2.621236133	1.213973799	0.049080277
148387	hsa-miR-3942-5p	N/A	N/A	N/A	N/A
148285	hsa-miR-3941	1.592460666	0.928684628	1.478893741	0.246531042
148466	hsa-miR-3937	N/A	N/A	0.026200873	N/A
148682	hsa-miR-483-3p	0.886682634	2.288431062	2.029112082	0.572719182
148264	hsa-miR-3665	N/A	0.028526149	N/A	N/A
148640	hsa-miR-34b-3p	2.449296458	0.019017433	0.04657933	0.309918025
148327	hsa-miR-3651	1.972273423	2.237717908	4.413391557	0.553822329

147555	hsv2-miR-H19	N/A	N/A	N/A	N/A
148012	hsa-miR-203b-3p	N/A	N/A	N/A	N/A
148172	hsa-miR-216a-3p	1.246516947	0.088748019	0.11062591	0.319424228
148661	hsa-miR-486-3p	4.592430859	0.003169572	0.014556041	0.188610927
145759	hsa-miR-181c-3p	N/A	0.028526149	N/A	N/A
17835	hsa-miR-450a-5p	0.293915575	0.079239303	0.023289665	0.053653448
42732	hsa-miR-532-3p	1.049698482	0.022187005	0.023289665	0.314990503
145734	hsa-miR-33b-3p	2.296215429	0.006339144	0.014556041	N/A
42493	hsa-miR-892b	N/A	N/A	N/A	N/A
168878	hsa-miR-5100	1.029953853	263.3122029	271.1994178	0.666384163
168605	hsa-miR-4653-3p	2.302179625	0.244057052	0.561863173	0.023522021
168856	hsa-miR-4494	N/A	N/A	N/A	N/A
168558	hsa-miR-5585-3p	0.167561666	0.469096672	0.07860262	0.121276203
168700	hsa-miR-5696	0.950158109	0.091917591	0.087336245	0.275821827
168829	hsa-miR-4794	N/A	N/A	N/A	N/A
168602	hsa-miR-4666a-3p	N/A	N/A	N/A	N/A
168894	hsa-miR-3978	N/A	N/A	N/A	N/A
168918	hsa-miR-4491	N/A	0.079239303	N/A	N/A
168704	hsa-miR-506-5p	2.296215429	0.145800317	0.334788937	0.043243537
168708	hsa-miR-296-5p	1.301188743	0.038034865	0.049490539	0.469862353
168621	hsa-miR-4796-5p	4.710453952	6.117274168	28.81513828	0.116765661
168670	hsa-miR-4694-5p	1.937170835	0.348652932	0.675400291	0.03786363
168767	hsa-miR-4681	N/A	N/A	N/A	N/A
168594	hsa-miR-3150b-5p	0.114810771	0.025356577	0.002911208	N/A
168844	hsa-miR-4532	0.421233171	22.36450079	9.420669578	0.414661464
168975	hsa-miR-4528	N/A	N/A	N/A	N/A
168570	hsa-miR-2682-3p	2.908539544	0.019017433	0.055312955	0.115636434
168649	hsa-miR-3129-3p	N/A	N/A	N/A	N/A
168786	hsa-miR-3618	0.153081029	0.019017433	0.002911208	0.791698559
169074	hsa-miR-3972	N/A	0.006339144	N/A	N/A
169106	hsa-miR-4783-3p	0.656061551	0.066561014	0.043668122	0.750152507
169071	hsa-miR-4639-5p	0.229621543	0.038034865	0.008733624	0.108473469
168984	hsa-miR-4637	N/A	N/A	N/A	N/A
169265	hsa-miR-4733-3p	N/A	N/A	0.029112082	N/A
169099	hsa-miR-4790-3p	N/A	N/A	N/A	N/A
169201	hsa-miR-5047	N/A	N/A	N/A	N/A
169132	hsa-miR-382-3p	0.967603079	0.592709984	0.573508006	0.463612208
169122	hsa-miR-5687	N/A	N/A	N/A	N/A
169227	hsa-miR-3064-5p	0.229621543	0.025356577	0.005822416	0.567638531
169289	hsa-miR-4692	N/A	N/A	N/A	N/A
169308	hsa-miR-4503	0.635067582	0.554675119	0.352256186	0.062962377
169282	hsa-miR-4290	0.956335877	1.153724247	1.103347889	0.551041803
169172	hsa-miR-4445-3p	0.952504178	0.171156894	0.163027656	0.768632813
169199	hsa-miR-4518	0.956167656	1.236133122	1.181950509	0.427550787
169034	hsa-miR-642b-5p	1.043450277	0.9318542	0.972343523	0.283216653
169173	hsa-miR-5590-3p	N/A	0.025356577	N/A	N/A
169231	hsa-miR-4766-3p	0.306162057	0.038034865	0.011644833	N/A
169315	hsa-miR-4676-3p	N/A	N/A	N/A	N/A
169005	hsa-miR-520b/hsa-miR-520c-3p	N/A	0.012678288	N/A	N/A
169416	hsa-miRPlus-A1086	N/A	N/A	N/A	N/A
13170	hsa-miR-380-5p	N/A	N/A	N/A	N/A

46324	hsa-miR-320b	1.423838001	0.789223455	1.123726346	0.496102543
17676	hsa-miR-152-3p	2.102312793	0.28526149	0.599708879	0.620002797
17593	hsa-miR-650	1.632864305	0.028526149	0.04657933	N/A
46483	hsa-miR-27a-3p	0.184526567	2.808240887	0.518195051	0.205541767
46438	hsa-let-7g-5p	0.6405463	4.985736926	3.193595342	0.168742098
42703	hsa-miR-490-3p	0.838617809	0.291600634	0.244541485	0.311185009
17625	hsa-miR-627-5p	0.671201433	0.082408875	0.055312955	0.518593601
27568	hsa-miR-744-5p	1.581837296	0.228209192	0.360989811	0.87851094
42478	hsa-miR-593-5p	N/A	N/A	N/A	N/A
42457	hsa-miR-323a-5p	N/A	N/A	N/A	N/A
17589	ebv-miR-BART3-3p	1.080571967	0.107765452	0.116448326	0.487220962
42832	hsa-miR-638	0.713441858	1.036450079	0.73944687	0.724987869
46462	hsa-miR-1224-5p	0.702371778	0.053882726	0.037845706	0.19985045
46822	hsa-miR-1178-3p	N/A	N/A	N/A	N/A
28759	hsa-miR-758-3p	N/A	N/A	0.002911208	N/A
42496	hsa-miR-181c-5p	N/A	N/A	N/A	N/A
10306	hsa-miR-146b-5p	2.927674672	0.050713154	0.148471616	0.881176315
17882	hsa-miR-20b-3p	1.971922336	2.114104596	4.168850073	0.27852144
27551	hsa-miR-612	0.357884258	1.163232964	0.416302766	0.141491465
42504	hsa-miR-593-3p	5.281295488	0.012678288	0.066957787	0.56753489
42897	ebv-miR-BART15	1.469577875	0.031695721	0.04657933	0.604116172
42874	hsa-miR-16-2-3p	1.17921773	0.491283677	0.579330422	0.984857311
17490	hsa-miR-571	0.32417159	0.053882726	0.017467249	0.60948667
46565	hsa-miR-1207-5p	N/A	0.006339144	N/A	N/A
15619	hsa-miR-649	N/A	N/A	N/A	N/A
10943	hsa-miR-136-5p	0.333994972	0.034865293	0.011644833	0.072654841
10977	hsa-miR-183-5p	1.205926089	0.881141046	1.062590975	0.80819536
42530	hsa-let-7a-2-3p	1.368724491	0.808240887	1.106259098	0.21766485
10990	hsa-miR-196a-5p	0.734788937	0.015847861	0.011644833	0.853152491
14285	hsa-miR-487b-3p	0.483661328	29.09033281	14.069869	0.102070169
27546	hsa-miR-380-3p	N/A	N/A	N/A	N/A
31026	hsa-miR-101-3p	1.904748677	1.245641838	2.372634643	0.659401164
11040	hsa-miR-29b-3p	0.594157344	19.60380349	11.64774381	0.07441137
13130	hsa-miR-517-5p	0.102054019	0.085578447	0.008733624	0.13039413
11138	hsa-miR-506-3p	0.459243086	0.006339144	0.002911208	N/A
146045	hsv2-miR-H4-5p	N/A	N/A	0.014556041	N/A
145987	hsa-miR-2054	N/A	0.022187005	N/A	N/A
146111	hsa-miR-767-5p	0.708299681	6.066561014	4.296943231	0.390523585
145933	hsa-miR-652-5p	0.568011185	0.24088748	0.136826783	0.518765931
146058	hsv1-miR-H3-3p	1.316496846	0.095087163	0.125181951	0.254247833
146098	hsv1-miR-H5-3p	0.906433741	12.80190174	11.60407569	0.611754628
146161	hsa-miR-2115-3p	2.440791956	0.684627575	1.671033479	0.043338943
146168	hsa-miR-1912	N/A	0.028526149	N/A	N/A
146090	hsv1-miR-H7-3p	1.237959623	0.145800317	0.180494905	0.696072957
145736	hsa-miR-605-5p	1.502977372	0.069730586	0.104803493	0.536344819
145721	hsa-miR-875-5p	N/A	N/A	N/A	N/A
145693	hsa-miR-92a-3p	1.53578045	0.976228209	1.499272198	0.915210745
145962	hsa-miR-639	0.447467622	0.247226624	0.11062591	0.087595881
145705	hsa-miR-431-5p	0.706527824	0.041204437	0.029112082	0.260693323
145727	hsa-miR-501-3p	N/A	N/A	0.029112082	N/A
145637	hsa-miR-187-3p	0.265586363	0.263074485	0.069868996	0.029269539
145748	hsa-miR-518f-3p	N/A	N/A	N/A	N/A

147193	hsa-miR-572	N/A	N/A	N/A	N/A
147608	hsa-miR-4307	1.294230515	0.278922345	0.360989811	0.228313812
147872	hsv2-miR-H13	N/A	0.04437401	N/A	N/A
147938	hsa-miR-4287	0.516648472	0.152139461	0.07860262	0.922594796
147814	hsa-miR-3159	N/A	N/A	N/A	N/A
147760	hsa-miR-4316	N/A	N/A	0.002911208	N/A
147544	hsa-miR-4314	N/A	0.003169572	N/A	N/A
147830	hsv1-miR-H18	0.765405143	0.038034865	0.029112082	0.510766603
147671	hsa-miR-4323	3.673944687	0.006339144	0.023289665	0.410927575
147897	hsa-miR-3136-5p	N/A	0.003169572	N/A	N/A
147698	hsv2-miR-H22	N/A	N/A	N/A	N/A
148627	hsa-miR-615-5p	N/A	N/A	0.005822416	N/A
148669	hsa-miR-185-3p	N/A	N/A	N/A	N/A
148687	hsa-miR-1908-5p	0.56522226	0.741679873	0.419213974	0.438401319
148234	hsa-miR-3667-5p	0.428807241	2.152139461	0.922852984	0.202602569
148677	hsa-miR-637	N/A	N/A	N/A	N/A
148654	hsa-miR-184	N/A	N/A	N/A	N/A
148474	hsa-miR-3622a-5p	N/A	N/A	N/A	N/A
148625	hsa-miR-941	0.673556526	0.047543582	0.03202329	0.690425063
147640	kshv-miR-K12-10a-5p	0.586418094	0.412044374	0.241630277	0.323419577
147318	hsa-miR-3660	0.273779532	0.329635499	0.090247453	0.093756493
147666	hsa-miR-4277	0.275545852	0.031695721	0.008733624	0.947505384
148181	hsa-miR-203b-5p	0.083498743	0.069730586	0.005822416	0.133009358
148386	hsa-miR-219b-5p	1.723110421	0.383518225	0.66084425	0.418447043
148284	hsa-miR-208b-3p	N/A	0.079239303	N/A	N/A
11164	hsa-miR-519e-3p	2.097623825	0.234548336	0.491994178	0.086551241
17886	hsa-miR-301b-3p	1.818417067	0.313787639	0.570596798	0.732416418
168684	hsa-miR-5010-5p	0.56522226	0.041204437	0.023289665	N/A
168679	hsa-miR-4763-3p	N/A	0.009508716	N/A	N/A
168718	hsa-miR-4684-3p	N/A	N/A	N/A	N/A
168945	hsa-miR-326	12.8588064	0.003169572	0.040756914	N/A
168964	hsa-miR-4450	1.591390559	5.65451664	8.998544396	0.003939014
168932	hsa-miR-5094	0.574053857	0.025356577	0.014556041	N/A
168934	hsa-miR-4999-5p	N/A	0.006339144	N/A	N/A
168795	hsa-miR-4706	0.057405386	0.050713154	0.002911208	N/A
168755	hsa-miR-4479	N/A	N/A	N/A	N/A
168827	hsa-miR-548ac	N/A	N/A	N/A	N/A
168676	hsa-miR-499b-5p	N/A	N/A	N/A	N/A
168852	hsa-miR-4764-5p	1.943360703	0.820919176	1.595342067	0.017937284
168611	hsa-miR-4753-3p	1.250161734	0.114104596	0.142649199	0.236340177
168572	hsa-miR-4507	0.300883401	0.183835182	0.055312955	0.103499047
168743	hsa-miR-548ad-3p	N/A	0.006339144	N/A	N/A
168812	hsa-miR-875-3p	N/A	N/A	0.014556041	N/A
169186	hsa-miR-4520-3p	0.870144794	0.12044374	0.104803493	0.419264512
169118	hsa-miR-5009-3p	0.563616514	0.557844691	0.31441048	0.296669959
169320	hsa-miR-4468	0.271958667	3.489698891	0.949053857	0.049773471
169321	hsa-miR-4691-3p	N/A	0.076069731	N/A	N/A
169070	hsa-miR-4695-3p	1.886243905	16.30744849	30.75982533	0.010986391
169113	hsa-miR-4755-5p	0.918486172	0.003169572	0.002911208	N/A
169182	hsa-miR-4728-3p	0.892613322	0.22503962	0.200873362	0.179296101
169036	hsa-miR-4745-3p	N/A	N/A	N/A	N/A
169230	hsa-miR-4747-3p	N/A	N/A	N/A	N/A

169157	hsa-miR-4755-3p	N/A	0.04437401	N/A	N/A
169000	hsa-miR-5707	N/A	N/A	N/A	N/A
169319	hsa-miR-3136-3p	0.864167097	1.179080824	1.018922853	0.6404409
168996	hsa-miR-4524a-3p	N/A	N/A	N/A	N/A
169064	hsa-miR-4778-3p	N/A	0.025356577	N/A	N/A
169112	hsa-miR-4727-3p	N/A	N/A	N/A	N/A
169169	hsa-miR-5684	0.989550506	25.03011094	24.76855895	0.983711859
168987	hsa-miR-4523	N/A	N/A	N/A	N/A
169365	hsa-miR-3974	N/A	0.031695721	N/A	N/A
169160	hsa-miR-4633-3p	N/A	N/A	N/A	N/A
168992	hsa-miR-5092	N/A	N/A	N/A	N/A
46340	hsa-miR-1289	N/A	N/A	N/A	N/A
46627	hsa-miR-1271-5p	N/A	N/A	N/A	N/A
42750	hsa-miR-636	1.29668636	0.269413629	0.349344978	0.610978004
42717	hsa-miR-92b-5p	N/A	N/A	0.008733624	N/A
11045	hsa-miR-302c-5p	0.743869029	0.833597464	0.620087336	2.21858E-05
42640	hsa-miR-20b-5p	3.259829534	0.548335975	1.787481805	0.519435323
17562	ebv-miR-BHRF1-2-5p	N/A	N/A	N/A	N/A
10937	hsa-miR-132-3p	N/A	0.019017433	N/A	N/A
32731	hsa-miR-190b	0.204108038	0.028526149	0.005822416	0.003717058
17359	ebv-miR-BART4-5p	0.114810771	0.050713154	0.005822416	N/A
19016	hsa-miR-217	N/A	N/A	0.03202329	N/A
46684	hsa-miR-1225-3p	N/A	N/A	N/A	N/A
46531	hsa-miR-1231	N/A	N/A	N/A	N/A
17571	kshv-miR-K12-11-3p	N/A	0.003169572	N/A	N/A
46556	hsa-miR-623	N/A	0.038034865	N/A	N/A
27838	hsa-miR-302d-3p	0.244929646	0.047543582	0.011644833	N/A
42700	hsa-miR-631	N/A	0.012678288	N/A	N/A
17508	hcmv-miR-UL112-3p	0.503685965	0.098256735	0.049490539	0.47563342
42778	hsa-let-7g-3p	N/A	N/A	0.020378457	N/A
17463	hsa-miR-151a-3p	1.434759445	0.969889065	1.391557496	0.92260401
46944	hsa-miR-1297	1.426070635	0.12044374	0.171761281	0.5425645
46860	hsa-miR-1205	0.229621543	0.025356577	0.005822416	0.986498528
46661	hsa-miR-1294	N/A	0.050713154	N/A	N/A
42541	hsa-miR-302a-5p	N/A	N/A	N/A	N/A
11058	hsa-miR-325	N/A	0.047543582	N/A	N/A
42744	hsa-miR-23a-3p	1.238957173	11.80031696	14.62008734	0.837840087
14271	hsa-miR-539-5p	3.061620573	0.009508716	0.029112082	0.796842819
42971	hsa-miR-96-3p	N/A	0.006339144	N/A	N/A
42502	hsa-miR-204-3p	0.946780769	1.337559429	1.266375546	0.176562444
29872	hsa-miR-340-5p	1.002702669	1.106180666	1.109170306	0.340174581
46744	hsa-miR-526b-5p	1.607350801	0.012678288	0.020378457	0.405317442
11074	hsa-miR-34c-5p	0.8036754	0.076069731	0.061135371	0.666974367
46416	hsa-miR-1293	0.605365886	0.139461173	0.084425036	0.467879635
46789	hsa-miR-513b-5p	0.360418624	1.001584786	0.360989811	0.108234456
42522	ebv-miR-BART19-3p	0.396709265	8.798732171	3.490538574	0.316353171
46622	hsa-miR-518f-5p	N/A	0.025356577	N/A	N/A
17612	hsa-miR-555	0.918486172	0.019017433	0.017467249	0.233187052
146005	hsa-miR-3129-5p	1.413055649	0.123613312	0.174672489	0.253047187
17858	hsa-miRPlus-A1073	1.959437166	0.142630745	0.279475983	0.381323108
146085	hsa-miR-3170	N/A	N/A	0.011644833	N/A
146116	hsa-miR-2116-3p	0.102054019	0.028526149	0.002911208	N/A

145980	hsa-miR-939-5p	0.510270095	0.028526149	0.014556041	N/A
145973	hsa-miR-664a-3p	2.022733592	0.282091918	0.570596798	0.013219761
145965	hsa-miR-518d-3p	N/A	N/A	N/A	N/A
145687	hsa-miR-384	N/A	N/A	N/A	N/A
145651	hsa-miR-99a-3p	1.377729258	0.006339144	0.008733624	N/A
145677	hsa-miR-139-5p	8.419456574	0.019017433	0.160116448	0.048883571
145633	hsa-let-7d-3p	1.05410158	0.944532488	0.995633188	0.304338086
146008	hsa-miR-26b-5p	2.23129625	0.605388273	1.350800582	0.91085795
145643	hsa-miR-382-5p	5.204754973	0.047543582	0.247452693	0.178628507
147864	hsv2-miR-H24	2.181404658	0.025356577	0.055312955	0.585122589
147895	kshv-miR-K12-2-3p	N/A	N/A	N/A	N/A
147953	hsa-miR-491-5p	1.257926713	0.145800317	0.183406114	0.209770617
147817	hsa-miR-3196	N/A	0.206022187	N/A	N/A
147831	kshv-miR-K12-1-3p	4.467182744	0.069730586	0.311499272	0.022199174
147898	hsa-miR-2861	N/A	0.009508716	N/A	N/A
147743	hsa-miR-4275	0.787953327	11.01743265	8.681222707	0.849657069
147799	hsa-miR-3156-5p	N/A	0.003169572	N/A	N/A
147763	hsa-miR-4272	N/A	N/A	N/A	N/A
147921	hsa-miR-3131	N/A	0.019017433	N/A	N/A
148593	hsa-miR-3605-3p	1.489437035	0.117274168	0.174672489	0.204850998
148058	hsa-miR-3672	N/A	N/A	N/A	N/A
148420	hsa-miR-3607-3p	2.541701733	2.614896989	6.64628821	0.18114966
148434	hsa-miR-3692-3p	0.880215915	0.076069731	0.066957787	0.498654526
148063	hsa-miR-3713	N/A	N/A	0.011644833	N/A
148382	hsa-miR-3609	N/A	N/A	N/A	N/A
148465	hsa-miR-3611	0.680199665	1.343898574	0.91411936	0.058507972
147589	hsa-miR-1193	N/A	N/A	0.005822416	N/A
147621	hsa-miR-4278	N/A	0.025356577	N/A	N/A
147633	hsa-miR-3163	0.714378134	0.028526149	0.020378457	N/A
147957	hsa-miR-412-5p	N/A	N/A	N/A	N/A
148380	hsa-miR-3913-5p	N/A	N/A	N/A	N/A
148080	hsa-miR-3921	N/A	0.015847861	N/A	N/A
148595	hsa-miR-34a-3p	2.119583473	0.041204437	0.087336245	0.666110681
148480	hsa-miR-494-5p	2.020669578	0.047543582	0.096069869	0.13897214
148089	hsa-miR-208a-3p	0.520475497	0.095087163	0.049490539	0.018042639
148215	hsa-miR-3591-3p	2.174375427	0.155309033	0.337700146	0.073487774
13132	hsa-miR-519e-5p	0.927795153	1.876386688	1.740902475	0.768434583
32711	hsa-miR-633	N/A	N/A	N/A	N/A
42629	hsa-miR-376c-3p	0.612324115	0.057052298	0.034934498	0.840463615
168734	hsa-miR-4465	N/A	N/A	N/A	N/A
46863	hsa-miR-3157-3p	0.4018377	0.050713154	0.020378457	0.871660856
168730	hsa-miR-4464	N/A	N/A	0.011644833	N/A
168644	hsa-miR-4775	1.35720838	6.171156894	8.375545852	0.488050449
168770	hsa-miR-4699-3p	0.459243086	0.101426307	0.04657933	0.24620115
168890	hsa-miR-1306-5p	0.706527824	0.041204437	0.029112082	0.231808989
168877	hsa-miR-5583-5p	55.10917031	0.003169572	0.174672489	0.078254109
168618	hsa-miR-4783-5p	N/A	N/A	N/A	N/A
168855	hsa-miR-4675	N/A	N/A	N/A	N/A
168919	hsa-miR-4456	5.284577671	11.08716323	58.59097525	0.000553108
168560	hsa-miR-4676-5p	0.667989943	0.069730586	0.04657933	0.213474101
168963	hsa-miR-664b-5p	9.903676982	0.145800317	1.443959243	0.03875293
168671	hsa-miR-3140-5p	N/A	N/A	N/A	N/A

168745	hsa-miR-4667-3p	1.377729258	0.025356577	0.034934498	0.552341323
168668	hsa-miR-4732-3p	1.679925485	0.611727417	1.027656477	0.024576344
168839	hsa-miR-4774-3p	N/A	N/A	N/A	N/A
168917	hsa-miR-4511	0.191293737	4.215530903	0.806404658	0.192974122
168661	hsa-miR-4531	2.755458515	0.510301109	1.406113537	0.006720285
168776	hsa-miR-4795-3p	0.725900362	6.484944532	4.707423581	0.760316346
168576	hsa-miR-4693-3p	N/A	N/A	N/A	N/A
169098	hsa-miR-3691-3p	N/A	N/A	N/A	N/A
169107	hsa-miR-4782-5p	0.223415555	0.117274168	0.026200873	0.008490062
169272	hsa-miR-4419b	1.469170338	17.14421553	25.18777293	0.457338239
169159	hsa-miR-4521	2.038315371	1.286846276	2.622998544	0.276103852
169023	hsa-miR-4712-3p	0.332143023	3.286846276	1.091703057	0.242564455
169194	hsa-miR-4513	0.812506998	0.16481775	0.133915575	0.163949719
169311	hsa-miR-4714-5p	0.979246007	8.624405705	8.445414847	0.386429339
169156	hsa-miR-4515	N/A	N/A	N/A	N/A
169309	hsa-miR-4802-5p	N/A	N/A	N/A	N/A
169126	hsa-miR-4486	N/A	0.107765452	N/A	N/A
169083	hsa-miR-371b-3p	1.597367255	0.072900158	0.116448326	0.034469704
169247	hsa-miR-4477a	1.020540191	0.114104596	0.116448326	0.330306267
169297	hsa-miR-4777-3p	N/A	N/A	N/A	N/A
169092	hsa-miR-548am-3p	N/A	N/A	N/A	N/A
169120	hsa-miR-4787-3p	N/A	0.019017433	N/A	N/A
169256	hsa-miR-4666a-5p	N/A	N/A	N/A	N/A
168998	hsa-miR-4508	0.384482584	0.136291601	0.052401747	0.247759584
168982	hsa-miR-4757-3p	N/A	N/A	N/A	N/A
169419	hsa-miR-300	N/A	N/A	0.005822416	N/A
169415	hsa-miR-187-5p	N/A	0.025356577	N/A	N/A
169407	hsa-miR-4301	0.875254796	28.2155309	24.69577875	0.812212345
42843	hsa-miR-654-5p	N/A	0.038034865	N/A	N/A
46617	hsa-miR-1197	N/A	0.009508716	N/A	N/A
17626	hsa-miR-575	0.432228787	0.053882726	0.023289665	0.868121683
42926	hsa-miR-92a-1-5p	N/A	N/A	0.017467249	N/A
10928	hsa-miR-125a-5p	1.081590363	3.194928685	3.455604076	0.639952033
11154	hsa-miR-517c-3p	N/A	N/A	N/A	N/A
42746	hsa-miR-647	1.407138913	0.643423138	0.905385735	0.035028943
29529	hsa-miR-369-3p	0.247284739	0.082408875	0.020378457	N/A
42782	hcmv-miR-UL148D	0.699798988	0.066561014	0.04657933	0.872307703
28251	hsa-miR-770-5p	N/A	N/A	N/A	N/A
33902	hsa-miR-128-3p	1.419478629	0.139461173	0.197962154	0.827867394
42940	hsa-miR-876-5p	N/A	N/A	N/A	N/A
46336	hsa-miR-1284	1.704179162	0.263074485	0.448326055	0.151296312
42791	hsa-miR-548b-5p	0.699798988	0.066561014	0.04657933	0.568849013
17445	hsa-miR-610	1.377729258	0.012678288	0.017467249	N/A
11083	hsa-miR-371a-3p	N/A	N/A	0.029112082	N/A
32608	hsa-miR-761	1.189857086	0.139461173	0.165938865	0.910670503
42615	hsa-miR-135b-3p	1.074455522	0.167987322	0.180494905	0.883249269
46818	ebv-miR-BART17-5p	N/A	N/A	N/A	N/A
46703	hsa-miR-548e-3p	0.455351195	0.748019017	0.340611354	0.13226057
17415	ebv-miR-BART10-3p	0.612324115	0.190174326	0.116448326	0.230055643
17315	kshv-miR-K12-3-3p	13.77729258	0.003169572	0.043668122	0.05195679
42850	hsa-miR-150-3p	N/A	0.015847861	N/A	N/A
46300	hiv1-miR-TAR-3p	N/A	N/A	N/A	N/A

31038	hiv1-miR-N367	N/A	0.028526149	N/A	N/A
46450	hsa-miR-548o-3p	N/A	N/A	N/A	N/A
46695	hsa-miR-1228-3p	2.066593886	0.038034865	0.07860262	0.042796653
17624	hsa-miR-532-5p	1.190630223	0.085578447	0.101892285	0.830396457
42666	hsa-miR-26a-2-3p	0.229621543	0.063391442	0.014556041	0.808233181
42581	hsa-miR-513a-5p	1.175280095	2.437400951	2.864628821	0.981601647
46223	hsa-miR-1306-3p	N/A	N/A	N/A	N/A
17299	hcmv-miR-UL22A-3p	N/A	N/A	0.040756914	N/A
19585	hsa-miR-148b-3p	1.444654129	0.630744849	0.911208151	0.89351931
14272	hsa-miR-542-3p	0.209966124	3.147385103	0.66084425	0.00411771
42772	hsa-miR-566	N/A	N/A	N/A	N/A
46326	hsa-miR-1233-3p	N/A	N/A	N/A	N/A
42773	ebv-miR-BART17-3p	0.765405143	0.057052298	0.043668122	0.711264657
27541	hcmv-miR-UL70-3p	0.183697234	0.015847861	0.002911208	N/A
10964	hsa-miR-155-5p	0.825693904	1.851030111	1.528384279	0.288647382
17332	ebv-miR-BART9-3p	1.632864305	0.028526149	0.04657933	0.395630758
42507	hsa-miR-202-5p	0.459243086	0.012678288	0.005822416	N/A
17848	hsa-miRPlus-A1087	0.079868363	0.072900158	0.005822416	0.960316453
42492	hsa-miRPlus-A1031	0.918486172	0.038034865	0.034934498	0.800680428
146006	hsa-miR-670-5p	1.530810286	0.019017433	0.029112082	0.512335949
146166	5p	1.836972344	0.022187005	0.040756914	N/A
146089	hsv1-miR-H8-5p	1.040950995	0.095087163	0.098981077	0.862622762
145844	hsa-miR-374a-5p	2.202492351	0.621236133	1.368267831	0.698052596
145859	hsa-miR-33a-5p	2.160859573	1.204437401	2.602620087	0.617840167
105441	hsa-miR-518c-3p	N/A	N/A	N/A	N/A
145827	hsa-miR-200a-5p	1.085483658	0.034865293	0.037845706	N/A
145950	hsa-miR-33b-5p	6.176819505	0.126782884	0.783114993	0.409587768
145732	hsa-miR-346	N/A	N/A	0.014556041	N/A
145821	hsa-miR-518c-5p	0.13121231	0.066561014	0.008733624	0.005614652
145889	hsa-miR-196b-5p	N/A	0.028526149	N/A	N/A
147794	kshv-miR-K12-8-3p	0.600984779	0.256735341	0.154294032	0.473854431
147863	hsa-miR-3155a	N/A	N/A	N/A	N/A
147793	hsa-miR-4252	1.864805258	0.10459588	0.195050946	0.655559476
147801	hsv1-miR-H11/hsv2-miR-H11-3p	N/A	N/A	N/A	N/A
147554	hsa-miR-4273	0.791798424	0.091917591	0.072780204	0.359872321
147826	hsa-miR-3153	0.734788937	0.015847861	0.011644833	0.581506976
147803	hsv2-miR-H10	0.173491832	0.570522979	0.098981077	0.193671001
147537	hsa-miR-4264	0.070652782	0.041204437	0.002911208	0.291070933
147795	hsa-miR-3186-3p	0.918486172	0.082408875	0.075691412	0.352244881
147673	hsa-miR-4262	N/A	N/A	N/A	N/A
147884	hsv2-miR-H25	0.435692158	0.494453249	0.215429403	0.093608023
147929	hsa-miR-1273c	0.423916695	0.123613312	0.052401747	0.537903549
148239	hsa-miR-3916	0.64834318	0.053882726	0.034934498	0.695405196
148691	hsa-miR-602	N/A	N/A	N/A	N/A
147376	hsa-miR-3679-5p	0.918486172	0.003169572	0.002911208	N/A
147572	hsa-miR-3120-3p	N/A	N/A	0.002911208	N/A
148398	hsa-miR-3908	N/A	N/A	N/A	N/A
148241	hsa-miR-3649	0.498606779	0.110935024	0.055312955	0.93902257
148147	hsa-miR-181b-3p	0.948114758	0.098256735	0.093158661	0.872827487
148289	hsa-miR-3675-5p	N/A	N/A	N/A	N/A
147964	hsa-miRPlus-J1005	N/A	N/A	N/A	N/A

148489	hsa-miR-3674	N/A	0.003169572	N/A	N/A
148038	hsa-miR-3679-3p	1.093661369	0.614896989	0.672489083	0.416317236
148637	hsa-miR-198	N/A	N/A	N/A	N/A
148388	hsa-miR-3677-3p	N/A	N/A	N/A	N/A
147602	hsa-miR-2682-5p	N/A	0.006339144	N/A	N/A
146158	hsa-miR-3202	0.600317759	0.484944532	0.291120815	0.0572046
11155	hsa-miR-518a-3p	N/A	N/A	N/A	N/A
42755	hcmv-miR-US33-3p	N/A	0.053882726	N/A	N/A
168579	hsa-miR-5706	N/A	0.095087163	N/A	N/A
168690	hsa-miR-4466	N/A	N/A	N/A	N/A
168698	hsa-miR-3127-3p	N/A	N/A	0.064046579	N/A
46808	hsa-miR-4485-3p	0.293915575	0.079239303	0.023289665	0.05106265
168843	hsa-miR-5694	0.796021349	0.095087163	0.075691412	0.010820739
168732	hsa-miR-5011-5p	N/A	N/A	0.002911208	N/A
168674	hsa-miR-4716-5p	1.29668636	0.107765452	0.139737991	0.521028985
168575	hsa-miR-4763-5p	N/A	N/A	N/A	N/A
168756	hsa-miR-548al	N/A	0.04437401	N/A	N/A
168665	hsa-miR-3619-3p	0.5844912	0.069730586	0.040756914	0.390133846
168862	hsa-miR-1343-3p	N/A	N/A	N/A	N/A
168943	hsa-miR-4769-3p	0.688864629	0.126782884	0.087336245	0.054302695
168598	hsa-miR-5007-3p	0.800298613	0.862123613	0.689956332	0.265461046
168940	hsa-miR-5579-5p	N/A	N/A	N/A	N/A
168735	hsa-miR-5586-3p	N/A	N/A	N/A	N/A
46762	hsa-miR-3928-3p	N/A	N/A	N/A	N/A
168642	hsa-miR-642b-3p	0.210255871	0.263074485	0.055312955	0.124739909
168909	hsa-miR-3152-5p	2.686572052	0.126782884	0.340611354	0.008802766
168872	hsa-miR-24-1-5p	2.276248339	0.072900158	0.165938865	0.36808437
169192	hsa-miR-4437	N/A	N/A	N/A	N/A
169019	hsa-miR-4524a-5p	0.291227323	0.129952456	0.037845706	0.253051475
169312	hsa-miR-548an	1.2420438	0.836767036	1.03930131	0.549959143
169299	hsa-miR-4538	N/A	N/A	N/A	N/A
169066	hsa-miR-4802-3p	N/A	N/A	N/A	N/A
169109	hsa-miR-5003-5p	1.198025441	0.072900158	0.087336245	0.158594261
169006	hsa-miR-4527	N/A	0.006339144	N/A	N/A
169018	hsa-miR-4640-5p	0.8767368	0.069730586	0.061135371	0.598110521
169009	hsa-miR-548ap-5p/hsa-miR-548j-5p	0.29481071	2.99207607	0.88209607	0.018060039
169142	hsa-miR-550a-5p	0.085440574	0.681458003	0.058224163	0.07668785
169295	hsa-miR-4725-3p	N/A	0.152139461	N/A	N/A
169370	hsa-miR-4693-5p	N/A	N/A	0.005822416	N/A
168995	hsa-miR-4791	1.285216874	3.508716323	4.509461426	0.912386893
168976	hsa-miR-3135b	0.459243086	0.158478605	0.072780204	0.780387266
169016	hsa-miR-4743-5p	0.656061551	0.04437401	0.029112082	0.126953344
169154	hsa-miR-4803	N/A	N/A	0.017467249	N/A
169050	hsa-miR-4787-5p	1.264684806	1.648177496	2.084425036	0.932758442
169372	hsa-miR-5011-3p	N/A	N/A	N/A	N/A
169133	hsa-miR-4537	N/A	0.006339144	N/A	N/A
169339	hsa-miR-5579-3p	N/A	N/A	N/A	N/A
169397	hsa-miR-3184-5p	N/A	0.015847861	N/A	N/A
46210	hsa-miR-1249-3p	1.148107715	0.050713154	0.058224163	0.501546591
42543	hsa-miR-608	N/A	N/A	0.049490539	N/A
42923	hsa-miR-30c-5p	2.304935235	5.00792393	11.54294032	0.530166876

42633	hsa-miR-885-3p	N/A	N/A	0.005822416	N/A
42808	hsa-miR-874-3p	0.392363025	0.652931854	0.256186317	0.103228187
45775	hsa-miR-1279	1.836972344	0.003169572	0.005822416	0.374209703
17810	hsa-miR-29b-1-5p	1.94502954	0.161648177	0.31441048	0.076541067
17353	hsa-miR-609	N/A	N/A	N/A	N/A
42584	hsa-miR-432-3p	1.005961045	0.066561014	0.066957787	0.287307789
42446	hsa-miR-576-5p	2.723117453	0.450079239	1.225618632	0.009929784
10975	hsa-miR-182-5p	1.397696348	0.218700475	0.305676856	0.732206021
17437	kshv-miR-K12-7-3p	N/A	0.009508716	N/A	N/A
46356	hsa-miR-30b-3p	1.757103981	0.072900158	0.128093159	0.975719307
33596	hsa-miR-126-5p	1.078222897	0.145800317	0.15720524	N/A
29190	hsa-miR-708-5p	3.124530584	0.694136292	2.168850073	0.329833312
14962	hsa-miR-581	N/A	N/A	0.005822416	N/A
17946	hsa-miR-192-3p	5.143522562	0.015847861	0.081513828	0.093777396
17563	hsa-miR-644a	2.361821585	0.022187005	0.052401747	N/A
17623	ebv-miR-BART14-3p	0.153081029	0.038034865	0.005822416	0.042695245
11121	hsa-miR-489-3p	0.082252493	0.212361331	0.017467249	0.192821685
46929	hsa-miR-548n	0.756400377	0.053882726	0.040756914	0.512148421
42663	hsa-miR-20a-3p	2.334485687	0.152139461	0.355167394	0.032717113
10988	hsa-miR-194-5p	0.724583536	1.426307448	1.033478894	0.908931401
14854	hsa-miR-569	1.148107715	0.050713154	0.058224163	N/A
32946	hsa-miR-486-5p	1.902578499	0.04437401	0.084425036	0.086246117
46735	hsa-miR-1179	N/A	N/A	0.002911208	N/A
46479	hsa-miR-1304-5p	0.494901961	1.347068146	0.666666667	0.155687864
31076	hsa-miR-559	0.862820343	0.10459588	0.090247453	0.424087914
42557	hsa-miR-624-5p	N/A	N/A	N/A	N/A
27537	ebv-miR-BART13-3p	0.817955347	0.637083994	0.521106259	0.883282799
42553	hsa-miR-216a-5p	1.331804949	0.063391442	0.084425036	0.147888166
32891	hsa-miR-769-5p	3.149095446	0.04437401	0.139737991	0.20800536
11245	hsa-miR-433-5p	0.485610738	56.14263074	27.26346434	0.062542729
31867	hsa-miR-145-3p	2.187242494	0.374009509	0.818049491	0.028574352
146165	hsa-miR-1973	0.289813598	3.264659271	0.946142649	0.186376893
146096	hsa-miR-764	0.524849241	0.022187005	0.011644833	N/A
146151	hsa-miR-449c-5p	N/A	N/A	N/A	N/A
146173	hsa-miR-2114-3p	N/A	N/A	0.008733624	N/A
17932	hsa-miR-381-5p	N/A	N/A	0.008733624	N/A
146136	hsa-miR-518d-5p/hsa-miR-520c-5p/hsa-miR-	0.833441156	0.171156894	0.142649199	0.536313977
145852	hsa-miR-210-3p	3.63720524	0.475435816	1.729257642	0.532404019
145742	hsa-miR-935	2.844344274	0.098256735	0.279475983	0.033069634
145750	hsa-miR-614	3.673944687	0.003169572	0.011644833	0.500767772
145981	hsa-miR-1285-3p	0.299282011	0.846275753	0.253275109	0.449617818
145634	hsa-miR-132-5p	1.683891315	0.019017433	0.03202329	0.255294982
145847	hsa-miR-766-3p	N/A	0.053882726	N/A	N/A
147954	hsa-miR-4318	N/A	N/A	N/A	N/A
147902	hsa-miR-3193	N/A	0.019017433	N/A	N/A
147751	hsa-miR-4274	2.69805313	0.050713154	0.136826783	0.460270512
147979	hsa-miR-3150a-3p	N/A	N/A	0.005822416	N/A
147540	hsa-miR-4310	N/A	N/A	N/A	N/A
147828	hsa-miR-3121-3p	0.393636931	0.022187005	0.008733624	0.69765073
147564	hsa-miR-4266	N/A	N/A	0.005822416	N/A
147614	hsa-miR-4299	0.449313506	1.17274168	0.526928675	0.398141981

147822	hsv2-miR-H23-5p	N/A	N/A	N/A	N/A
147780	hsa-miR-4267	N/A	N/A	N/A	N/A
148351	hsa-miR-3945	N/A	0.022187005	N/A	N/A
148454	hsa-miR-3682-3p	N/A	N/A	0.017467249	N/A
148282	hsa-miR-3714	2.066593886	0.025356577	0.052401747	0.108466658
148039	hsa-miR-3925-5p	N/A	N/A	N/A	N/A
148601	hsa-miR-3617-5p	N/A	N/A	N/A	N/A
148471	hsa-miR-152-5p	N/A	N/A	0.011644833	N/A
147638	hsv2-miR-H12	N/A	N/A	0.014556041	N/A
147600	hsa-miR-4292	37.65793304	0.003169572	0.119359534	0.103929995
147707	hsa-miR-3154	N/A	N/A	N/A	N/A
148371	hsa-miR-3620-3p	1.347113052	0.047543582	0.064046579	0.47558347
148227	hsa-miR-1251-3p	0.896746854	0.535657686	0.480349345	0.291294711
148192	hsa-miR-421	0.688864629	0.012678288	0.008733624	0.660617764
46264	hsa-miR-298	0.614679207	0.412044374	0.253275109	0.712978465
42738	hsa-miR-340-3p	2.377258327	0.053882726	0.128093159	0.476661207
168631	hsa-miR-4723-5p	1.607350801	0.038034865	0.061135371	0.356908497
168952	hsa-miR-4781-3p	N/A	N/A	N/A	N/A
168564	hsa-miR-548at-5p	2.93915575	0.015847861	0.04657933	0.281195316
168595	hsa-miR-1185-2-3p	1.705760033	0.022187005	0.037845706	0.555895412
168622	hsa-miR-4652-5p	N/A	N/A	N/A	N/A
168571	hsa-miR-550b-2-5p	0.039730954	2.491283677	0.098981077	0.008455631
168727	hsa-miR-4426	0.574053857	0.40570523	0.232896652	0.845326456
168951	hsa-miR-548as-3p	0.934829342	0.890649762	0.832605531	0.473113601
168800	hsa-miR-4724-5p	0.367394469	0.015847861	0.005822416	N/A
168567	hsa-miR-3177-5p	N/A	0.050713154	N/A	N/A
168748	hsa-miR-320e	0.086649639	0.167987322	0.014556041	0.022954357
168655	hsa-miR-5588-5p	0.612324115	0.028526149	0.017467249	0.322872284
168725	hsa-miR-4700-5p	N/A	0.063391442	N/A	N/A
168691	hsa-miR-3677-5p	N/A	N/A	N/A	N/A
168626	hsa-miR-4662a-5p	N/A	N/A	N/A	N/A
168900	hsa-miR-4721	N/A	N/A	N/A	N/A
168736	hsa-miR-4796-3p	0.414438882	0.259904913	0.107714702	0.231221727
168971	hsa-miR-4449	0.097589156	0.507131537	0.049490539	0.16349105
168875	hsa-miR-5091	0.570094865	0.091917591	0.052401747	0.063319929
168999	hsa-miR-4749-3p	N/A	N/A	0.037845706	N/A
169139	hsa-miR-5192	N/A	N/A	N/A	N/A
169218	hsa-miR-5189-5p	0.070652782	0.206022187	0.014556041	0.042062363
169101	hsa-miR-4469	N/A	N/A	0.011644833	N/A
169119	hsa-miR-4786-3p	N/A	N/A	N/A	N/A
169367	hsa-miR-4444	0.19336551	0.06022187	0.011644833	0.404698316
169087	hsa-miR-149-3p	2.657486657	0.237717908	0.631732169	0.107867584
169081	hsa-miR-4707-3p	1.530810286	0.009508716	0.014556041	0.92958538
169033	hsa-miR-5680	N/A	N/A	N/A	N/A
169027	hsa-miR-3973	N/A	N/A	N/A	N/A
169004	hsa-miR-4798-5p	N/A	N/A	N/A	N/A
169055	hsa-miR-3529-5p	N/A	N/A	0.026200873	N/A
169255	hsa-miR-4708-5p	N/A	N/A	N/A	N/A
169359	hsa-miR-3160-5p	1.577400165	0.145800317	0.229985444	0.110439197
169141	hsa-miR-4423-3p	0.511776428	0.858954041	0.439592431	0.2734359
169048	hsa-miR-4736	N/A	N/A	N/A	N/A
168997	hsa-miR-5584-5p	N/A	0.038034865	N/A	N/A

169273	hsa-miR-548ar-3p	N/A	N/A	N/A	N/A
169303	hsa-miR-378g	N/A	0.053882726	N/A	N/A
169138	hsa-miR-4504	0.04638819	0.313787639	0.014556041	0.146357739
169420	hsa-miR-193b-5p	3.673944687	0.012678288	0.04657933	0.046045774
169408	hsa-miR-181d-5p	1.933292991	0.846275753	1.636098981	0.094273803
42576	hsa-miR-342-5p	1.34492618	0.088748019	0.119359534	0.034664884
46869	hsa-miR-1258	0.710962099	0.757527734	0.538573508	0.782019119
11004	hsa-miR-203a-3p	1.732598915	0.278922345	0.483260553	0.115185835
19588	hsa-miR-17-3p	0.769640587	0.801901743	0.617176128	0.125951417
46732	hsa-miR-1264	0.531966035	21.79714739	11.59534207	0.014878188
29328	hsa-miR-582-3p	N/A	0.003169572	N/A	N/A
42622	hsa-miR-579-3p	N/A	N/A	N/A	N/A
46404	hsa-miR-1244	2.337964801	0.034865293	0.081513828	0.021134459
17875	hsa-miR-500a-5p	0.560759979	0.30110935	0.168850073	0.430690346
42572	hsa-miR-154-3p	0.156142649	0.316957211	0.049490539	0.150458709
29577	hsa-miR-374a-3p	N/A	N/A	0.066957787	N/A
46625	hsa-miR-1303	5.970160116	0.006339144	0.037845706	N/A
10995	hsa-miR-199a-3p/hsa-miR-199b-3p	N/A	0.009508716	N/A	N/A
27549	hsa-miR-548d-3p	N/A	N/A	0.049490539	N/A
11182	hsa-miR-98-5p	0.694204665	1.908082409	1.324599709	0.232761446
27536	hsa-miR-190a-5p	2.236783971	0.269413629	0.602620087	0.665804506
42837	hsa-miR-577	1.342402866	0.041204437	0.055312955	0.275689511
46788	hsa-miR-1299	1.388663617	0.532488114	0.73944687	0.125119047
11061	hsa-miR-329-3p	N/A	N/A	N/A	N/A
17349	hsa-miR-595	1.010334789	0.095087163	0.096069869	0.291564722
46766	hsa-miR-1208	N/A	N/A	0.03202329	N/A
46320	hsa-miR-31-3p	2.949961469	0.269413629	0.794759825	0.90840357
11065	hsa-miR-335-5p	0.874748735	0.066561014	0.058224163	0.400699494
10925	hsa-miR-10b-5p	N/A	0.006339144	N/A	N/A
46872	hsa-miR-1262	N/A	0.129952456	N/A	N/A
21702	hsa-miR-219a-1-3p	0.408216076	0.028526149	0.011644833	0.295140633
46246	hsa-miR-1226-5p	N/A	N/A	N/A	N/A
46640	hsa-miR-1257	0.561297105	0.057052298	0.03202329	0.025658484
11022	hsa-miR-221-3p	1.035756141	7.546751189	7.816593886	0.464998865
42627	hsa-miR-212-3p	3.187687302	0.107765452	0.343522562	0.026797316
45745	hsa-miR-302f	0.918486172	0.015847861	0.014556041	0.826588289
10936	hsa-miR-130b-3p	1.596335745	0.858954041	1.371179039	0.792235135
17851	hsa-miR-200c-5p	1.326702248	0.114104596	0.151382824	0.091715954
42906	ebv-miR-BHRF1-1	1.060265916	0.472266244	0.500727802	0.358866703
42730	hsa-miR-423-3p	0.872887567	0.893819334	0.780203785	0.452023644
17825	hsa-miR-338-5p	0.5844912	0.034865293	0.020378457	0.50123587
146109	hsa-miR-1910-5p	1.836972344	0.006339144	0.011644833	N/A
146046	jcv-miR-J1-5p	0.137772926	0.063391442	0.008733624	0.265219406
146196	hsa-miR-711	0.256752619	1.099841521	0.282387191	0.015368207
42839	hsa-miR-135a-5p	0.852543575	0.618066561	0.526928675	0.715859551
146103	hsa-miR-1913	1.211620056	0.148969889	0.180494905	0.863061777
146149	hsv2-miR-H2	N/A	N/A	N/A	N/A
145686	hsa-miR-548c-3p	N/A	N/A	N/A	N/A
145819	hsa-miR-520c-3p	N/A	N/A	N/A	N/A
145724	hsa-miR-887-3p	1.312123103	0.022187005	0.029112082	0.103312604
145753	hsa-miR-484	0.918486172	0.101426307	0.093158661	0.856084098

145974	hsa-miR-200b-5p	1.88070978	0.066561014	0.125181951	0.174629571
145839	hsa-miR-662	N/A	N/A	N/A	N/A
145826	hsa-miR-18b-3p	2.165003119	0.04437401	0.096069869	0.007937586
145976	hsa-miR-663b	0.946319086	0.10459588	0.098981077	0.40701043
147816	hsa-miR-3162-5p	18.36972344	0.003169572	0.058224163	0.572334526
147819	hsa-miR-3117-3p	N/A	N/A	N/A	N/A
147871	hsa-miR-3180-5p	1.0715672	0.057052298	0.061135371	0.835381227
147683	hsa-miR-3188	N/A	N/A	N/A	N/A
147809	hsa-miR-514b-3p	1.085483658	0.069730586	0.075691412	0.393666617
147805	hsa-miR-3183	0.943999677	0.114104596	0.107714702	0.737404277
147852	hsa-miR-4295	N/A	0.139461173	N/A	N/A
147882	hsa-miR-2909	N/A	N/A	N/A	N/A
147866	hsa-miR-3134	N/A	N/A	N/A	N/A
147786	hsa-miR-3198	N/A	0.003169572	N/A	N/A
148384	hsa-miR-3648	N/A	0.025356577	N/A	N/A
148085	hsa-miR-3687	0.807102587	1.69889065	1.371179039	0.97431486
148188	hsa-miR-2277-5p	N/A	N/A	N/A	N/A
147280	hsa-miR-1185-1-3p	N/A	0.012678288	N/A	N/A
147469	hsa-miR-3909	N/A	N/A	N/A	N/A
147779	hsa-miR-3180-3p	N/A	N/A	N/A	N/A
148590	hsa-miR-3919	N/A	N/A	N/A	N/A
148065	hsa-miR-3689b-3p/hsa-miR-3689c	0.628437907	0.06022187	0.037845706	0.202096257
147386	hsa-miR-212-5p	N/A	0.003169572	N/A	N/A
148402	hsa-miR-3920	2.440548971	0.110935024	0.270742358	0.19496702
148628	hsa-miR-3199	N/A	N/A	0.017467249	N/A
148064	hsa-miR-3926	0.71963865	0.614896989	0.442503639	0.441284604
148317	hsa-miR-3621	0.206659389	0.126782884	0.026200873	0.268353681
148187	hsa-miR-410-5p	1.301188743	0.038034865	0.049490539	0.301248738
11144	hsa-miR-512-3p	N/A	N/A	N/A	N/A
11037	hsa-miR-299-3p	0.953015727	0.42155309	0.401746725	0.33979348
42774	kshv-miR-K12-9-5p	N/A	N/A	N/A	N/A
168646	hsa-miR-4731-3p	N/A	0.031695721	N/A	N/A
168958	hsa-miR-2681-5p	0.222865515	8.725832013	1.944687045	0.265370127
168808	hsa-miR-4779	N/A	N/A	N/A	N/A
168796	hsa-miR-3664-3p	0.224021018	0.259904913	0.058224163	0.008093906
168625	hsa-miR-4539	N/A	0.012678288	N/A	N/A
168593	hsa-miR-4726-3p	N/A	N/A	N/A	N/A
168632	hsa-miR-5188	0.401177868	0.551505547	0.22125182	0.340404281
168898	hsa-miR-5008-3p	N/A	N/A	N/A	N/A
168559	hsa-miR-15a-3p	3.181898524	0.088748019	0.282387191	0.205800677
168765	hsa-miR-4448	0.126687748	0.183835182	0.023289665	0.007248708
168849	hsa-miR-4635	1.978277908	0.041204437	0.081513828	0.278264262
168614	hsa-miR-5197-5p	0.508643737	0.795562599	0.404657933	0.120958325
168683	hsa-miR-4792	N/A	N/A	N/A	N/A
168790	hsa-miR-3120-5p	2.792197962	0.079239303	0.22125182	0.130617311
168806	hsa-miR-4659a-3p	0.288667083	0.110935024	0.03202329	0.217649528
168924	hsa-miR-4720-5p	N/A	N/A	N/A	N/A
168891	hsa-miR-1271-3p	N/A	N/A	N/A	N/A
168928	hsa-miR-4431	0.765405143	0.608557845	0.465793304	0.857379147
168702	hsa-miR-4540	2.992038287	0.209191759	0.625909753	0.079092137
169080	hsa-miR-4684-5p	0.180913943	0.209191759	0.037845706	0.099597321

169056	hsa-miR-4669	0.471655061	0.117274168	0.055312955	0.21949349
169253	hsa-miR-4690-5p	0.202066958	0.158478605	0.03202329	0.288778529
169022	hsa-miR-4797-5p	0.450361352	32.94136292	14.83551674	0.193531692
169266	hsa-miR-378d	0.482437181	0.313787639	0.151382824	0.307208447
169334	hsa-miR-548ao-5p	N/A	N/A	N/A	N/A
169262	hsa-miR-5009-5p	1.0715672	0.019017433	0.020378457	0.455304149
169079	hsa-miR-4667-5p	0.630077494	2.896988906	1.825327511	0.196801012
169213	hsa-miR-4768-3p	0.275545852	0.095087163	0.026200873	0.090822543
169355	hsa-miR-2355-5p	1.093435919	0.066561014	0.072780204	0.40516844
169238	hsa-miR-4654	1.323057462	0.266244057	0.352256186	0.119772369
169363	hsa-miR-320c	1.343711251	0.513470681	0.689956332	0.545313137
169061	hsa-miR-548x-3p	N/A	N/A	N/A	N/A
169082	hsa-miR-1275	0.351495066	4.431061807	1.557496361	0.279925046
169096	hsa-miR-4804-3p	3.107219177	0.297939778	0.925764192	0.076140285
169304	hsa-miR-4793-5p	N/A	N/A	N/A	N/A
169171	hsa-miR-4436b-5p	1.516055488	0.263074485	0.398835517	0.08850935
169356	hsa-miR-548aa/hsa-miR-548ap-3p/hsa-	0.958420353	0.072900158	0.069868996	0.884612602
169084	hsa-miR-5708	N/A	N/A	N/A	N/A
169020	hsa-miR-4506	0.293405305	0.228209192	0.066957787	0.350593906
169414	hsa-miR-525-5p	N/A	N/A	N/A	N/A
169399	hsa-miR-4750-5p	0.395138216	1.385103011	0.547307132	0.207500958
17418	kshv-miR-K12-4-3p	0.135355857	0.6022187	0.081513828	0.165806078
42757	hsa-miR-619-3p	N/A	N/A	N/A	N/A
11108	hsa-miR-425-3p	0.367394469	0.095087163	0.034934498	0.494139601
42829	hsa-miR-127-3p	0.69736913	0.171156894	0.119359534	0.790586399
46259	hsa-miR-885-5p	0.942037099	0.123613312	0.116448326	0.371354309
46737	hsa-miR-1265	2.415618632	1.267828843	3.062590975	0.019182246
42535	hsa-miR-661	N/A	N/A	N/A	N/A
42542	hsa-miR-589-5p	1.394336101	4.557844691	6.355167394	0.244363754
42607	hsa-miR-653-5p	N/A	N/A	0.008733624	N/A
17646	ebv-miR-BHRF1-3	2.161143934	0.053882726	0.116448326	0.279933537
17893	hsa-miR-362-3p	1.607350801	0.050713154	0.081513828	0.841489369
6880	hsa-miR-297	0.709739315	0.069730586	0.049490539	0.339878024
32095	hsa-miR-624-3p	0.656061551	0.022187005	0.014556041	0.750176903
42567	hsa-miR-590-3p	1.836972344	0.028526149	0.052401747	0.727466744
17325	hsa-miR-643	2.959566553	0.028526149	0.084425036	0.154492756
17630	hsa-miR-588	0.531755152	0.06022187	0.03202329	0.764169262
42941	hsa-miR-218-1-3p	0.494569477	0.041204437	0.020378457	0.16886247
42944	hsa-miR-564	N/A	N/A	0.023289665	N/A
42562	hsa-miR-105-3p	N/A	0.006339144	N/A	N/A
42571	hsa-miR-129-1-3p	0.594660919	0.494453249	0.294032023	0.340024406
17439	hcmv-miR-UL36-5p	0.480117772	0.139461173	0.066957787	0.001483658
46443	hsa-miR-193a-5p	0.475644625	0.177496038	0.084425036	0.012956869
10923	hsa-miR-107	0.674513282	1.419968304	0.957787482	0.247093926
46850	hsa-miR-1237-3p	3.444323144	0.012678288	0.043668122	0.812503134
27672	hsa-miR-615-3p	2.364613336	0.148969889	0.352256186	0.034402584
17503	hsa-miR-590-5p	2.074001033	0.196513471	0.407569141	0.854500639
13140	hsa-miR-138-5p	1.118248987	2.754358162	3.080058224	0.400611667
11013	hsa-miR-181a-3p	2.684805733	0.206022187	0.553129549	0.486869157
29802	hsa-miR-144-3p	1.396098981	0.079239303	0.11062591	0.490454491
42551	hsa-miR-122-3p	3.706747765	0.088748019	0.328966521	0.186016813

42899	hsa-miR-377-5p	0.898943913	0.148969889	0.133915575	0.420097177
42885	hsa-miR-376a-5p	3.980106744	0.028526149	0.113537118	0.021963568
42956	hsa-miR-545-5p	N/A	N/A	0.049490539	N/A
17732	hsa-miR-192-5p	3.193241644	0.339144216	1.082969432	0.309187998
46746	hsa-miR-1269a	N/A	N/A	0.005822416	N/A
42669	hsa-miR-505-3p	2.536771332	0.066561014	0.168850073	0.521180653
4040	hsa-miR-9-5p	1.530810286	0.038034865	0.058224163	0.89036346
42793	hsa-miRPlus-A1072	1.169425365	1.194928685	1.397379913	0.128845163
17892	hsa-miR-372-5p	0.332784845	0.218700475	0.072780204	0.205724858
146010	hsa-miR-2116-5p	2.5348614	0.725832013	1.839883552	0.007035361
146140	hsa-miR-1976	1.320323872	0.050713154	0.066957787	0.544781939
146142	hsa-miR-1972	0.777180607	0.123613312	0.096069869	0.041231024
146163	hsa-miR-224-3p	0.439275995	0.072900158	0.03202329	0.525104011
145749	hsa-miR-137	N/A	N/A	N/A	N/A
145970	hsa-miR-129-2-3p	N/A	N/A	N/A	N/A
145822	hsa-miR-214-5p	7.347889374	0.012678288	0.093158661	0.153193484
145789	hsa-miR-550a-3-5p/hsa-miR-550a-5p	0.187612628	5.35340729	1.004366812	0.050380456
145768	hsa-miR-665	0.315912441	0.995245642	0.31441048	0.168909379
145972	hsa-miR-141-5p	N/A	N/A	0.008733624	N/A
145746	hsa-let-7i-3p	4.139655985	0.22503962	0.931586608	0.235393122
145676	hsa-miR-30e-3p	1.978837534	1.600633914	3.167394469	0.476570755
147604	hsa-miR-4285	0.306162057	0.228209192	0.069868996	0.274008503
147915	hsa-miR-3174	0.484476222	0.288431062	0.139737991	0.073108319
147804	hsv1-miR-H17	0.262424621	0.110935024	0.029112082	0.215854273
147613	hsa-miR-3145-3p	N/A	N/A	0.061135371	N/A
147570	hsa-miR-4271	N/A	0.006339144	N/A	N/A
147523	hsa-miR-4263	N/A	N/A	N/A	N/A
147947	hsa-miR-4308	2.036998221	0.713153724	1.452692868	0.06252813
147694	hsa-miR-4281	N/A	N/A	N/A	N/A
147772	hsa-miR-4303	0.447467622	0.123613312	0.055312955	0.462196352
148374	hsa-miR-3936	N/A	0.012678288	N/A	N/A
148263	hsa-miR-1273e	0.6600528	0.833597464	0.550218341	0.617170169
148585	hsa-miR-3912-3p	0.019135129	0.152139461	0.002911208	0.099822853
148331	hsa-miR-3655	N/A	N/A	N/A	N/A
147262	hsa-miR-548h-3p/hsa-miR-548z	2.087468572	0.034865293	0.072780204	0.954107535
148214	hsa-miR-3675-3p	0.840648361	0.187004754	0.15720524	0.154060419
147684	hsa-miR-3200-5p	0.471655061	0.117274168	0.055312955	0.326182792
148228	hsa-miR-3656	0.010934359	0.266244057	0.002911208	0.243205628
148641	hsa-miR-518b	0.250496229	0.034865293	0.008733624	0.609625841
148353	hsa-miR-3681-5p	0.796021349	0.047543582	0.037845706	0.11229413
148132	hsa-miR-3622a-3p	1.049698482	0.133122029	0.139737991	0.722977928
148272	hsa-miR-3615	N/A	N/A	N/A	N/A
148216	hsa-miR-3907	0.752029131	0.979397781	0.736535662	0.61215145
148621	hsa-miR-892a	0.765405143	0.228209192	0.174672489	0.495214508
147389	hsa-miR-3691-5p	N/A	N/A	N/A	N/A
148250	hsa-let-7f-2-3p	1.28588064	0.015847861	0.020378457	0.45919055
46367	hsa-miR-1283	N/A	N/A	N/A	N/A
145999	hsa-miR-517a-3p/hsa-miR-517b-3p	N/A	N/A	0.029112082	N/A
145723	hsa-miR-519c-3p	N/A	N/A	N/A	N/A

168563	hsa-miR-4761-5p	N/A	N/A	N/A	N/A
168635	hsa-miR-378e	N/A	0.155309033	N/A	N/A
168803	hsa-miR-4696	N/A	N/A	N/A	N/A
168692	hsa-miR-3688-5p	0.37419807	0.085578447	0.03202329	0.283260664
168739	hsa-miR-1269b	N/A	N/A	N/A	N/A
168711	hsa-miR-548ak	N/A	N/A	N/A	N/A
168768	hsa-miR-4423-5p	1.312763163	1.299524564	1.705967977	0.107836661
168589	hsa-miR-4671-3p	0.326304298	0.24088748	0.07860262	0.348287227
168923	hsa-miR-4688	0.206401387	0.282091918	0.058224163	0.03891258
168772	hsa-miR-224-5p	15.61426492	0.003169572	0.049490539	0.986073701
168973	hsa-miR-1268b	0.730078752	0.123613312	0.090247453	0.883030737
168793	hsa-miR-4483	0.207959133	0.671949287	0.139737991	0.160569074
168731	hsa-miR-548ah-3p	N/A	N/A	N/A	N/A
168950	hsa-miR-4646-3p	1.646940722	0.091917591	0.151382824	0.785479202
168578	hsa-miR-5190	0.204108038	0.028526149	0.005822416	0.477100392
168722	hsa-miR-4742-3p	1.384107634	0.912836767	1.263464338	0.025037359
168893	hsa-miR-4505	0.32417159	0.161648177	0.052401747	0.305144039
168789	hsa-miR-4686	3.90356623	0.012678288	0.049490539	N/A
168561	hsa-miR-1245b-5p	N/A	N/A	N/A	N/A
168627	hsa-miR-4678	N/A	N/A	N/A	N/A
169166	hsa-miR-4683	0.834987429	0.10459588	0.087336245	0.85581485
169307	hsa-miR-4685-3p	1.836972344	0.091917591	0.168850073	0.075038966
169257	hsa-miR-4740-5p	N/A	0.003169572	N/A	N/A
169269	hsa-miR-4499	N/A	0.057052298	N/A	N/A
169165	hsa-miR-5585-5p	N/A	0.025356577	N/A	N/A
169298	hsa-miR-4433a-5p	N/A	0.003169572	N/A	N/A
169236	hsa-miR-659-5p	0.532526557	0.595879556	0.317321689	0.27143774
169162	hsa-miR-5691	N/A	0.031695721	N/A	N/A
169376	hsa-miR-5701	1.631783997	23.05546751	37.62154294	0.433605771
169329	hsa-miR-370-3p	N/A	N/A	N/A	N/A
169179	hsa-miR-4650-5p	0.402844812	0.722662441	0.291120815	0.015398758
169073	hsa-miR-584-3p	N/A	N/A	N/A	N/A
169274	hsa-miR-5196-3p	1.098695484	0.500792393	0.550218341	0.252060336
169010	hsa-miR-2681-3p	0.629607456	0.393026941	0.247452693	0.615613126
169343	hsa-miR-5682	N/A	N/A	N/A	N/A
169135	hsa-miR-3173-5p	0.816432153	0.028526149	0.023289665	0.8200634
169042	hsa-miR-4704-3p	N/A	N/A	N/A	N/A
169088	hsa-miR-4492	N/A	N/A	N/A	N/A
169054	hsa-miR-4422	N/A	N/A	N/A	N/A
169411	hsa-miR-205-3p	5.548792749	0.307448494	1.705967977	0.005113441
169417	hsa-miR-551b-5p	0.091848617	0.063391442	0.005822416	N/A
169393	hsa-miR-4747-5p	1.976385423	0.709984152	1.403202329	0.010505717
42460	hsa-miR-223-5p	N/A	N/A	N/A	N/A
42859	hsa-miR-675-3p	1.968184654	0.04437401	0.087336245	0.091521659
42905	hcmv-miR-US25-1-5p	N/A	N/A	N/A	N/A
11277	hsa-miR-7-1-3p	0.367394469	0.031695721	0.011644833	0.206604999
17818	hsa-miR-27a-5p	1.024465345	0.082408875	0.084425036	0.548341438
17652	hsa-miR-558	N/A	N/A	N/A	N/A
42483	hsa-miR-522-3p	N/A	N/A	N/A	N/A
28520	hsa-miR-454-5p	N/A	N/A	N/A	N/A
32884	hsa-miR-342-3p	1.669974858	0.10459588	0.174672489	0.40906187
17529	hcmv-miR-US25-2-3p	N/A	0.031695721	N/A	N/A

19596	hsa-miR-30d-5p	0.568940827	1.565768621	0.890829694	0.360892318
11102	hsa-miR-410-3p	N/A	0.006339144	N/A	N/A
42736	hsa-miR-148b-5p	3.36778263	0.019017433	0.064046579	0.012516969
11005	hsa-miR-204-5p	0.119802544	0.072900158	0.008733624	0.375741464
46266	hsa-miR-1825	0.632734918	0.142630745	0.090247453	0.095590875
31349	hsa-miR-524-3p	N/A	N/A	0.002911208	N/A
10986	hsa-miR-193a-3p	2.03388155	4.583201268	9.321688501	0.516551862
17391	hsa-miR-635	0.8585849	0.145800317	0.125181951	0.676714378
46623	hsa-miR-1273a	0.284293339	0.266244057	0.075691412	0.229762112
18739	hsa-miR-186-5p	0.974152	0.627575277	0.611353712	0.465539334
42451	hsa-miR-139-3p	1.836972344	0.003169572	0.005822416	N/A
17863	hsa-miR-934	0.688029641	0.87163233	0.599708879	0.9950995
42509	hsa-miR-219a-5p	0.542741829	0.069730586	0.037845706	0.324767435
42929	hsa-miR-25-5p	0.648990229	0.529318542	0.343522562	0.522304606
42682	hsa-miR-25-3p	1.04517392	0.275752773	0.288209607	0.401672276
11168	hsa-miR-520d-3p	N/A	N/A	0.029112082	N/A
17854	hsa-miR-106b-3p	0.908048829	0.278922345	0.253275109	0.17716641
14279	hsa-miR-362-5p	1.443335413	0.022187005	0.03202329	0.042412829
14300	hsa-miR-29c-5p	1.0715672	0.038034865	0.040756914	0.558092954
17460	hsa-miR-657	1.48707285	0.066561014	0.098981077	0.385468506
17853	hsa-miR-30d-3p	0.492046163	0.088748019	0.043668122	0.626666057
10998	hsa-miR-19b-3p	0.695045243	9.302694136	6.465793304	0.068355235
17530	hsa-miR-641	1.078222897	0.072900158	0.07860262	0.227348078
42869	hsa-miR-936	0.459243086	0.209191759	0.096069869	0.21251102
42447	hsa-miR-556-3p	0.153081029	0.019017433	0.002911208	0.768460965
46258	hsa-miR-1184	0.941448326	0.253565769	0.238719068	0.276607862
146122	hsa-miR-2053	2.099396964	0.022187005	0.04657933	0.666236972
146000	hsa-miR-1911-5p	N/A	N/A	N/A	N/A
146042	hsv1-miR-H8-3p	1.622007495	0.148969889	0.241630277	0.191108112
146035	hsv1-miR-H2-5p	5.510917031	0.003169572	0.017467249	0.415217233
145968	hsa-let-7d-5p	0.470048806	2.155309033	1.013100437	0.125673568
145689	hsa-miR-543	0.918486172	0.003169572	0.002911208	0.974105733
146115	hsa-miR-940	0.918486172	0.047543582	0.043668122	0.371945879
145714	hsa-miR-28-3p	1.342402866	0.247226624	0.331877729	0.979788104
146178	hsa-miR-502-3p	2.684805733	0.041204437	0.11062591	0.579796905
147867	hsa-miR-3118	N/A	N/A	N/A	N/A
147841	kshv-miR-K12-11-5p	N/A	N/A	N/A	N/A
147925	hsa-miR-3126-5p	0.669020792	0.256735341	0.171761281	0.939296855
147588	hsa-miR-4288	1.023117165	11.0459588	11.30131004	0.379610489
147649	hsa-miR-500a-5p/hsa-miR-500b-5p	0.524849241	0.088748019	0.04657933	0.777347774
147977	hsa-miR-3190-3p	N/A	N/A	N/A	N/A
147857	hsa-miR-3130-5p	N/A	N/A	0.005822416	N/A
147569	hsa-miR-4256	N/A	N/A	N/A	N/A
147616	hsa-miR-4291	0.134220042	4.424722662	0.593886463	0.204512809
147815	hsv1-miR-H14-5p	N/A	N/A	0.037845706	N/A
148247	hsa-miR-2355-3p	2.043955143	0.450079239	0.919941776	0.088677855
148072	hsa-miR-3668	N/A	N/A	N/A	N/A
147997	hsa-miR-3934-5p	0.031620016	1.933438986	0.061135371	0.112988347
147682	hsv1-miR-H6-5p	1.049698482	0.066561014	0.069868996	0.543933235
148189	hsa-miR-3681-3p	0.344432314	0.025356577	0.008733624	0.982647425
148393	hsa-miR-676-3p	1.1756623	0.079239303	0.093158661	0.662207528

148379	hsa-miR-3654	0.381516023	14.92551506	5.694323144	0.095084665
147415	hsa-miR-495-5p	1.59978086	0.576862124	0.922852984	0.122685196
148165	hsa-miR-3605-5p	N/A	0.003169572	N/A	N/A
148032	hsa-miR-3685	0.227353856	9.949286846	2.262008734	0.217660759
148559	hsa-miR-411-3p	1.435134643	0.050713154	0.072780204	0.254504693
148278	hsa-miR-138-2-3p	0.486650201	10.46275753	5.091703057	0.208754949
17327	hsa-miR-630	1.303657792	0.589540412	0.768558952	0.139257599
17488	kshv-miR-K12-6-3p	0.685803008	0.237717908	0.163027656	0.907101141
17936	hsa-miR-16-1-3p	1.093435919	0.332805071	0.363901019	0.723214894
168897	hsa-miR-5187-5p	0.027014299	0.215530903	0.005822416	0.005700685
168882	hsa-miR-664b-3p	1.684885348	2.928684628	4.934497817	0.019182538
168710	hsa-miR-4536-5p	0.459243086	0.006339144	0.002911208	N/A
168590	hsa-miR-4520-5p	0.367394469	0.063391442	0.023289665	0.892928669
168728	hsa-miR-4641	N/A	N/A	N/A	N/A
168719	hsa-miR-4495	N/A	N/A	N/A	N/A
168785	hsa-miR-520d-5p	N/A	N/A	N/A	N/A
168615	hsa-miR-4655-3p	N/A	N/A	N/A	N/A
168781	hsa-miR-4776-3p	0.577923883	0.282091918	0.163027656	0.905071157
168782	hsa-miR-5093	N/A	0.022187005	N/A	N/A
168820	hsa-miR-4711-5p	N/A	N/A	N/A	N/A
168750	hsa-miR-4645-5p	0.825423696	0.719492868	0.593886463	0.993564004
168921	hsa-miR-4718	N/A	0.079239303	N/A	N/A
168759	hsa-miR-3689f	N/A	N/A	N/A	N/A
168962	hsa-miR-548ab	N/A	N/A	0.014556041	N/A
168666	hsa-miR-4659a-5p	N/A	0.028526149	N/A	N/A
168955	hsa-miR-4756-3p	1.206278506	0.475435816	0.573508006	0.189509301
168703	hsa-miR-4716-3p	0.268825221	0.129952456	0.034934498	0.011901826
168663	hsa-miR-4442	N/A	0.006339144	N/A	N/A
168779	hsa-miR-4797-3p	N/A	N/A	N/A	N/A
169102	hsa-miR-4639-3p	2.592974475	2.580031696	6.689956332	0.017701712
169285	hsa-miR-4467	0.717293963	1.664025357	1.193595342	0.787676584
169249	hsa-miR-4482-5p	N/A	0.291600634	N/A	N/A
168993	hsa-miR-4799-3p	0.027832914	0.10459588	0.002911208	0.364599493
169011	hsa-miR-4801	0.918486172	0.022187005	0.020378457	N/A
169049	hsa-miR-3162-3p	1.130444519	0.041204437	0.04657933	0.226470599
169001	hsa-miR-4670-5p	N/A	N/A	N/A	N/A
169039	hsa-miR-4782-3p	N/A	N/A	N/A	N/A
169181	hsa-miR-5191	0.232920352	59.56893819	13.87481805	0.105682599
169379	hsa-miR-4694-3p	0.196971393	1.359746434	0.26783115	0.007769662
169232	hsa-miR-3156-3p	0.543122337	8.694136292	4.721979622	0.708591869
169046	hsa-miR-4715-5p	N/A	0.006339144	N/A	N/A
169275	hsa-miR-548aq-3p	N/A	N/A	N/A	N/A
169245	hsa-miR-2467-3p	0.229621543	0.012678288	0.002911208	N/A
169038	hsa-miR-488-3p	N/A	0.019017433	N/A	N/A
169240	hsa-miR-3944-5p	1.443335413	0.04437401	0.064046579	0.141281133
169115	hsa-miR-450a-2-3p	N/A	0.015847861	N/A	N/A
169287	hsa-miR-548as-5p	N/A	N/A	N/A	N/A
169361	hsa-miR-4481	N/A	0.003169572	N/A	N/A
169225	hsa-miR-640	N/A	N/A	N/A	N/A
169395	hsa-miR-4484	0.543104867	0.729001585	0.395924309	0.24895266
42777	hsa-miR-548am-5p/hsa-miR-548c-	N/A	0.006339144	N/A	N/A

11184	hsa-miR-99b-5p	0.915096924	1.717908082	1.572052402	0.5995847
17953	hsa-miR-183-3p	1.305217191	1.023771791	1.336244541	0.04570175
42501	hcmv-miR-US33-5p	1.705760033	0.022187005	0.037845706	0.172755885
46584	hsa-miR-1468-5p	N/A	N/A	N/A	N/A
42676	hsa-miR-495-3p	N/A	N/A	N/A	N/A
17958	hsa-miR-576-3p	N/A	0.050713154	N/A	N/A
17631	hcmv-miR-UL22A-5p	0.574053857	0.025356577	0.014556041	0.884787421
11093	hsa-miR-379-5p	N/A	N/A	N/A	N/A
42480	hsa-miR-485-5p	N/A	N/A	0.026200873	N/A
42538	hsa-miR-196a-3p	0.659503186	1.933438986	1.27510917	0.081336454
45936	hsa-miR-1292-5p	N/A	0.012678288	N/A	N/A
42847	hsa-miR-497-5p	N/A	N/A	0.005822416	N/A
46275	hsa-miR-1251-5p	N/A	N/A	N/A	N/A
42672	hsa-miR-323b-5p	0.704884736	0.136291601	0.096069869	0.824050525
42635	hsa-miR-541-3p	N/A	N/A	N/A	N/A
10976	hsa-miR-182-3p	1.010334789	0.031695721	0.03202329	0.786284607
17866	hsa-miR-331-5p	N/A	N/A	0.075691412	N/A
46515	hsa-miR-1537-3p	4.592430859	0.003169572	0.014556041	N/A
42954	ebv-miR-BART7-5p	0.673556526	0.28526149	0.192139738	0.987673384
46215	hsa-miR-1301-3p	0.918486172	0.253565769	0.232896652	0.957454344
42723	hsa-miR-195-3p	1.805837219	0.187004754	0.337700146	0.042386234
10997	hsa-miR-19a-3p	0.859170575	12.85895404	11.04803493	0.185636384
46738	hsa-miR-1182	N/A	N/A	0.064046579	N/A
42950	hsa-miR-24-2-5p	0.85557616	0.231378764	0.197962154	0.333500649
11149	hsa-miR-515-5p	1.607350801	0.012678288	0.020378457	0.620301606
11053	hsa-miR-32-5p	2.122389775	1.356576862	2.879184862	0.567722318
42442	hsa-miR-498	15.16736708	1.179080824	17.88355167	0.013540625
42533	hiv1-miR-H1	N/A	0.015847861	N/A	N/A
46729	hsa-miR-302d-5p	N/A	0.028526149	N/A	N/A
42654	hsa-miR-483-5p	0.720536566	0.367670365	0.264919942	0.566058049
28884	hsa-miR-876-3p	1.530810286	0.019017433	0.029112082	0.164803164
42599	hsa-miR-153-3p	N/A	N/A	N/A	N/A
17814	hsa-miR-570-3p	0.765405143	0.019017433	0.014556041	0.644246255
46321	hsa-miR-1245a	N/A	N/A	N/A	N/A
17283	ebv-miR-BART11-5p	0.306162057	0.019017433	0.005822416	N/A
42912	hsa-miR-339-3p	N/A	N/A	N/A	N/A
146066	hsa-miR-3116	0.384482584	0.136291601	0.052401747	0.123026422
146072	hsa-miR-1469	0.373135007	0.202852615	0.075691412	0.465416956
146052	hsa-miR-1471	0.240555902	0.133122029	0.03202329	0.605237777
145762	hsa-miR-549a	N/A	N/A	0.005822416	N/A
145716	hsa-miR-671-5p	0.712084785	0.282091918	0.200873362	0.209425721
145722	hsa-miR-520e	0.771528384	0.079239303	0.061135371	0.560842192
145751	hsa-miR-23b-5p	N/A	N/A	0.014556041	N/A
145692	hsa-miR-499a-3p	1.377729258	0.012678288	0.017467249	0.534884899
147936	kshv-miR-K12-8-5p	0.816432153	0.085578447	0.069868996	0.305537046
46567	hsa-miR-3176	1.176810408	0.304278922	0.358078603	0.189923468
147735	hsa-miR-4289	0.456241497	0.484944532	0.22125182	0.131345685
147868	hsa-miR-4269	N/A	N/A	N/A	N/A
147887	hsa-miR-3147	N/A	N/A	0.034934498	N/A
147942	hsa-miR-4268	1.725865145	0.786053883	1.356622999	0.135288215
147967	hsa-miR-3180/hsa-miR-3180-3p	N/A	N/A	N/A	N/A

56873	hsa-miR-3124-5p	N/A	N/A	N/A	N/A
46710	hsa-miR-3157-5p	N/A	N/A	N/A	N/A
46274	hsa-miR-3187-3p	N/A	0.003169572	N/A	N/A
148650	hsa-miR-516a-5p	N/A	0.009508716	N/A	N/A
148524	hsa-miR-3914	0.248935504	0.339144216	0.084425036	0.30407588
148499	hsa-miR-3910	0.489064065	0.244057052	0.119359534	0.21813537
147530	hsa-miR-378b	0.183697234	0.380348653	0.069868996	0.075412642
147276	hsa-miR-3616-3p	N/A	N/A	0.026200873	N/A
148196	hsa-miR-548y	N/A	N/A	N/A	N/A
148514	hsa-miR-365a-5p	N/A	N/A	0.017467249	N/A
148622	hsa-miR-877-3p	1.134600565	0.215530903	0.244541485	0.499550306
147679	hsa-miR-3197	N/A	0.041204437	N/A	N/A
147752	hsa-miR-4302	N/A	N/A	N/A	N/A
148639	hsa-miR-4298	0.344432314	0.152139461	0.052401747	0.128197714
148645	hsa-miR-129-5p	2.985080058	0.012678288	0.037845706	N/A
148261	hsa-miR-208a-5p	0.620598765	0.469096672	0.291120815	0.47458994
17566	hsa-miR-629-3p	0.287503982	2.288431062	0.657933042	0.021295399
11023	hsa-miR-222-3p	1.528705765	10.60538827	16.2125182	0.892156567
10916	hsa-miR-1-3p	1.836972344	0.009508716	0.017467249	N/A
168783	hsa-miR-4770	N/A	N/A	N/A	N/A
168583	hsa-miR-539-3p	0.254049367	0.148969889	0.037845706	0.093195909
168956	hsa-miR-4524b-3p	N/A	N/A	N/A	N/A
168613	hsa-miR-548ar-5p	N/A	N/A	N/A	N/A
168823	hsa-miR-4522	0.714378134	0.085578447	0.061135371	0.885206716
168693	hsa-miR-651-5p	N/A	N/A	N/A	N/A
168612	hsa-miR-4733-5p	0.42862688	0.047543582	0.020378457	N/A
168887	hsa-miR-5089-5p	0.096053735	19.73058637	1.895196507	0.072691581
168609	hsa-miR-5197-3p	N/A	N/A	N/A	N/A
168680	hsa-miR-378f	0.141305565	0.082408875	0.011644833	0.030645483
168747	hsa-miR-4520-2-3p	0.134412611	0.129952456	0.017467249	0.515433575
168942	hsa-miR-4636	0.134526627	16.81458003	2.262008734	0.32486786
168847	hsa-miR-4740-3p	N/A	N/A	N/A	N/A
168637	hsa-miR-3940-5p	0.772246753	1.134706815	0.876273654	0.918832146
168682	hsa-miR-4502	0.086108079	0.101426307	0.008733624	0.312782293
168773	hsa-miR-5702	N/A	0.015847861	N/A	N/A
168858	hsa-miR-4638-3p	N/A	N/A	0.005822416	N/A
168643	hsa-miR-4703-5p	N/A	N/A	N/A	N/A
168905	hsa-miR-4509	N/A	N/A	N/A	N/A
168581	hsa-miR-3942-3p	N/A	N/A	N/A	N/A
169254	hsa-miR-4762-3p	0.367394469	0.015847861	0.005822416	N/A
169226	hsa-miR-4419a	0.29950636	0.145800317	0.043668122	0.085021694
169200	hsa-miR-4677-5p	0.711826783	0.126782884	0.090247453	0.719321078
169382	hsa-miR-4680-5p	N/A	N/A	N/A	N/A
169197	hsa-miR-4662a-3p	N/A	N/A	N/A	N/A
169103	hsa-miR-4652-3p	0.070652782	0.041204437	0.002911208	0.228050834
169125	hsa-miR-4418	N/A	0.006339144	N/A	N/A
169357	hsa-miR-4689	N/A	N/A	0.017467249	N/A
169095	hsa-miR-548ah-3p/hsa-miR-548av-3p/hsa-	N/A	N/A	N/A	N/A
169271	hsa-miR-4784	0.385903886	1.667194929	0.643377001	0.198970437
168994	hsa-miR-3591-5p	0.878613904	2.04437401	1.796215429	0.634741748
169220	hsa-miR-4697-3p	2.099396964	0.022187005	0.04657933	0.10539598

169252	hsa-miR-4712-5p	N/A	N/A	N/A	N/A
169306	hsa-miR-4758-5p	N/A	N/A	0.026200873	N/A
169325	hsa-miR-4446-5p	0.834987429	0.034865293	0.029112082	N/A
169239	hsa-miR-4732-5p	0.215751785	0.472266244	0.101892285	0.111866854
169288	hsa-miR-4730	N/A	N/A	N/A	N/A
169316	hsa-miR-3976	0.856372718	4.40570523	3.772925764	0.015771489
169235	hsa-miR-4420	N/A	0.009508716	N/A	N/A
169131	hsa-miR-4724-3p	N/A	N/A	N/A	N/A
169387	hsa-miR-5703	N/A	0.003169572	N/A	N/A
169389	hsa-miR-4717-3p	N/A	N/A	N/A	N/A
42834	hsa-miR-219a-2-3p	0.083498743	0.034865293	0.002911208	0.06049206
42743	hsa-let-7e-3p	1.212401747	0.079239303	0.096069869	0.806935557
17535	hsa-miR-548a-3p	1.312123103	0.022187005	0.029112082	0.347344728
42761	hsa-miR-675-5p	0.384482584	0.136291601	0.052401747	0.301933004
46642	hsa-miR-1276	1.836972344	0.015847861	0.029112082	0.958747383
42855	ebv-miR-BART6-5p	N/A	N/A	N/A	N/A
19595	hsa-miR-30a-3p	0.420680689	0.415213946	0.174672489	0.418564882
42514	hsa-miR-937-3p	0.339012063	6.64659271	2.253275109	0.218221662
28950	hsa-miR-455-3p	0.984564314	0.440570523	0.433770015	0.303392956
17338	hsa-miR-660-5p	N/A	0.025356577	N/A	N/A
46402	hsa-miR-1255b-5p	0.317937521	0.082408875	0.026200873	0.01935488
17520	hcmv-miR-US5-2-3p	0.765405143	0.038034865	0.029112082	0.544166036
42556	hsa-miR-889-3p	1.001984915	0.034865293	0.034934498	N/A
4700	hsa-miR-140-5p	1.258666235	0.256735341	0.323144105	0.630107524
42525	hsa-miR-671-3p	0.562943138	0.098256735	0.055312955	0.948284399
10947	hsa-miR-142-3p	1.920471086	0.034865293	0.066957787	0.725603252
42942	hsa-miR-134-5p	1.722161572	0.025356577	0.043668122	0.961487058
45736	hsa-miR-548f-3p	N/A	N/A	0.005822416	N/A
11091	hsa-miR-377-3p	1.419478629	0.069730586	0.098981077	0.425852554
46810	hsa-miR-1827	0.866249991	0.668779715	0.579330422	0.515774128
17306	ebv-miR-BART12	1.586476115	0.034865293	0.055312955	0.548521199
28047	hsa-miR-890	0.653837614	0.187004754	0.122270742	0.844669147
10955	hsa-miR-148a-3p	0.378200188	0.053882726	0.020378457	0.223144102
14328	hsa-miR-124-3p	1.942951517	0.082408875	0.160116448	0.266486483
42624	kshv-miR-K12-10b	2.265599224	0.047543582	0.107714702	0.280889549
42823	hsa-miR-27b-5p	1.590549224	0.129952456	0.206695779	0.741848387
42617	hsa-miR-541-5p	N/A	0.015847861	N/A	N/A
46517	hsa-miR-1266-5p	N/A	0.006339144	N/A	N/A
17427	hsa-miR-200c-3p	0.1948304	0.10459588	0.020378457	0.356741722
46675	hsa-miR-1181	N/A	N/A	0.005822416	N/A
17444	hsa-miR-632	0.349899494	0.066561014	0.023289665	0.269862715
33043	hsa-miR-544a	N/A	0.006339144	N/A	N/A
17619	hcmv-miR-US25-2-5p	0.631459243	0.050713154	0.03202329	0.89289883
11011	hsa-miR-211-5p	0.8036754	0.025356577	0.020378457	N/A
46880	hsa-miR-1183	0.355543034	0.098256735	0.034934498	0.64831099
11165	hsa-miR-520a-3p	N/A	N/A	N/A	N/A
46345	hsa-miR-1207-3p	1.078222897	0.072900158	0.07860262	0.880059729
17569	hsa-miR-563	N/A	N/A	N/A	N/A
146084	ebv-miR-BART4-3p	0.820076939	0.088748019	0.072780204	0.402569822
146131	hsa-miR-2117	N/A	N/A	0.011644833	N/A
145798	hsa-miR-142-5p	N/A	0.028526149	N/A	N/A
146137	hsa-miR-133a-3p	N/A	N/A	0.052401747	N/A

145971	hsa-miR-611	N/A	N/A	0.029112082	N/A
145824	hsa-miR-509-3-5p	N/A	N/A	0.026200873	N/A
147669	hsa-miR-548w	N/A	N/A	N/A	N/A
147903	hsa-miR-3065-3p	N/A	N/A	N/A	N/A
147879	hsa-miR-3132	N/A	N/A	N/A	N/A
147911	hsa-miR-4280	N/A	N/A	N/A	N/A
147885	kshv-miR-K12-5-5p	2.840899089	1.226624406	3.484716157	0.014111026
147651	hsa-miR-3123	0.524849241	0.04437401	0.023289665	0.622604234
147543	hsa-miR-3167	N/A	N/A	N/A	N/A
147198	hsa-miR-26a-5p	0.556658286	0.10459588	0.058224163	0.092329073
148006	hsa-miR-4315	2.985080058	0.012678288	0.037845706	0.318039052
147862	hsa-miR-3158-3p	1.683891315	0.019017433	0.03202329	0.123078236
147886	hsa-miR-3122	N/A	0.022187005	N/A	N/A
147883	hsa-miR-3191-3p	N/A	N/A	N/A	N/A
148662	hsa-miR-583	N/A	0.022187005	N/A	N/A
148406	hsa-miR-3684	N/A	N/A	N/A	N/A
147556	hsa-miR-4254	2.388064047	0.031695721	0.075691412	0.313955288
147975	hsa-miR-487a-5p	2.071933922	0.136291601	0.282387191	0.018052946
147610	hsa-miR-3186-5p	N/A	N/A	N/A	N/A
148106	hsa-miR-3678-3p	N/A	N/A	N/A	N/A
147670	hsa-miR-3177-3p	N/A	N/A	N/A	N/A
148554	hsa-miR-3917	0.841945657	0.038034865	0.03202329	0.099881816
148504	hsa-miR-874-5p	1.000129387	0.142630745	0.142649199	0.444852276
148685	hsa-miR-202-3p	N/A	N/A	N/A	N/A
148684	hsa-miR-628-3p	0.348391307	0.091917591	0.03202329	0.394307684
148566	hsa-miR-3666	N/A	N/A	N/A	N/A
148481	hsa-miR-3646	0.282521696	5.00792393	1.414847162	0.158045166
148349	hsa-miR-3938	0.125248114	0.069730586	0.008733624	0.134542198
148052	hsa-miR-374c-3p	1.965133205	0.136291601	0.26783115	0.165563901
145769	hsa-miR-371a-5p	0.918486172	0.022187005	0.020378457	N/A
42528	hsa-miR-296-3p	2.219674915	0.038034865	0.084425036	0.101552784
145744	hsa-miR-568	N/A	N/A	N/A	N/A
168707	hsa-miR-4767	N/A	N/A	0.005822416	N/A
168896	hsa-miR-548ao-3p	0.787273862	0.04437401	0.034934498	0.607360282
168775	hsa-miR-4647	N/A	N/A	N/A	N/A
168851	hsa-miR-4650-3p	0.236182158	0.221870048	0.052401747	0.216948956
168616	hsa-miR-4536-3p	1.231981452	0.529318542	0.652110626	0.259853492
168724	hsa-miR-4529-3p	N/A	0.072900158	N/A	N/A
168737	hsa-miR-5685	N/A	N/A	N/A	N/A
168587	hsa-miR-4461	0.612324115	0.066561014	0.040756914	0.150051698
168854	hsa-miR-4498	N/A	N/A	N/A	N/A
168716	hsa-miR-4487	0.951289249	0.088748019	0.084425036	0.820484092
168912	hsa-miR-4446-3p	N/A	N/A	N/A	N/A
168949	hsa-miR-4512	N/A	N/A	N/A	N/A
168892	hsa-miR-4424	N/A	N/A	0.002911208	N/A
168733	hsa-miR-5583-3p	N/A	N/A	0.002911208	N/A
168601	hsa-miR-146b-3p	N/A	N/A	N/A	N/A
168633	hsa-miR-3190-5p	N/A	N/A	0.002911208	N/A
168754	hsa-miR-5692b/hsa-miR-5692c	1.498582701	0.06022187	0.090247453	0.167280105
168714	hsa-miR-4452	N/A	N/A	N/A	N/A
168926	hsa-miR-4776-5p	N/A	N/A	N/A	N/A

169196	hsa-miR-5705	N/A	N/A	N/A	N/A
169007	hsa-miR-4746-3p	N/A	N/A	N/A	N/A
169270	hsa-miR-4458	0.918486172	0.110935024	0.101892285	0.691570908
169030	hsa-miR-5586-5p	N/A	N/A	N/A	N/A
169337	hsa-miR-3155a/hsa-miR-3155b	N/A	N/A	N/A	N/A
169375	hsa-miR-660-3p	1.945945279	0.374009509	0.727802038	0.094355235
169380	hsa-miR-3124-3p	1.156407289	2.10459588	2.433770015	0.227951505
169377	hsa-miR-4715-3p	0.275545852	0.380348653	0.104803493	0.194150618
169224	hsa-miR-4440	N/A	N/A	N/A	N/A
169040	hsa-miR-4753-5p	N/A	0.076069731	N/A	N/A
169085	hsa-miR-5007-5p	N/A	N/A	N/A	N/A
169349	hsa-miR-4517	1.049698482	0.022187005	0.023289665	0.552306944
169189	hsa-miR-4795-5p	1.93445995	3.045958796	5.892285298	0.012855003
169183	hsa-miR-4644	0.457045751	1.324881141	0.605531295	0.999839461
169360	hsa-miR-4445-5p	2.350534504	0.589540412	1.38573508	0.026079145
169207	hsa-miR-4804-5p	N/A	N/A	0.03202329	N/A
169013	hsa-miR-5195-5p	N/A	N/A	N/A	N/A
169163	hsa-miR-4685-5p	N/A	N/A	N/A	N/A
169203	hsa-miR-5095	0.284434427	0.491283677	0.139737991	0.215073037
169409	hsa-miR-4286	0.599848348	14.38985737	8.631732169	0.040113596
169396	hsa-miR-5700	N/A	N/A	N/A	N/A
17660	hsa-miR-550a-3p	0.826637555	0.063391442	0.052401747	0.50089565
42801	hsa-miR-92a-2-5p	N/A	N/A	N/A	N/A
27544	hsa-miR-363-5p	0.121150017	1.033280507	0.125181951	0.07540369
46380	hsa-miR-1255a	0.437882942	2.998415214	1.312954876	0.181298434
11073	hsa-miR-34b-5p	N/A	0.028526149	N/A	N/A
17942	hsa-miR-125a-3p	1.802954337	0.171156894	0.308588064	0.653928146
42708	hsa-miR-99a-5p	1.020540191	0.028526149	0.029112082	0.521500782
11139	hsa-miR-507	N/A	0.006339144	N/A	N/A
46295	hsa-miR-1286	0.888857586	0.098256735	0.087336245	0.29781249
46626	hsa-miR-30c-2-3p	1.241657232	0.171156894	0.212518195	0.961631339
17347	hsa-miR-708-3p	1.357318454	0.28526149	0.387190684	0.313066622
17935	hsa-miR-101-5p	N/A	N/A	N/A	N/A
11041	hsa-miR-29c-3p	0.447182156	1.568938193	0.701601164	0.13397164
11043	hsa-miR-302b-3p	N/A	N/A	N/A	N/A
46249	hsa-miR-449b-5p	0.918486172	0.009508716	0.008733624	N/A
30493	hsa-miR-548a-5p	N/A	0.04437401	N/A	N/A
42630	hsa-miR-140-3p	1.191369745	0.437400951	0.521106259	0.484686399
42873	hsa-miR-944	0.223415555	0.351822504	0.07860262	0.070494411
46368	hsa-miR-1282	0.979718583	0.047543582	0.04657933	0.391103373
42898	hsa-miR-124-5p	0.190359828	0.611727417	0.116448326	0.123807201
27533	hsa-miR-320a	2.616293944	0.522979398	1.368267831	0.437834038
9938	hsa-let-7i-5p	2.019146173	4.58637084	9.26055313	0.669693159
42609	hsa-miR-135a-3p	0.503685965	0.098256735	0.049490539	0.154571725
11020	hsa-miR-22-3p	1.106655436	8.462757528	9.365356623	0.854525135
27720	hsa-miR-15a-5p	1.607350801	3.740095087	6.011644833	0.904949967
42490	hsa-miR-505-5p	0.82150316	1.020602219	0.838427948	0.888710088
4610	hsa-miR-126-3p	1.856308895	0.30110935	0.558951965	0.711869342
46222	hsa-miR-1228-5p	0.918486172	0.019017433	0.017467249	0.645327568
42870	hsa-miR-616-3p	N/A	N/A	0.017467249	N/A
21498	hsa-miR-654-3p	0.64834318	0.107765452	0.069868996	0.900916079

46800	hsa-miR-1224-3p	N/A	N/A	0.020378457	N/A
17402	ebv-miR-BART2-5p	2.449296458	0.009508716	0.023289665	0.70581187
11007	hsa-miR-206	0.360977098	1.451664025	0.524017467	0.025349581
42476	hsa-miR-374b-3p	2.264760424	0.231378764	0.524017467	0.311114854
17561	ebv-miR-BART6-3p	0.1674452	1.182250396	0.197962154	0.108449956
17592	hsa-miR-604	0.250496229	0.10459588	0.026200873	0.605307814
46221	hsa-miR-519d-3p	0.765405143	0.019017433	0.014556041	0.695868601
13148	hsa-miR-195-5p	0.220581325	0.805071315	0.177583697	0.040604508
29575	hsa-miR-32-3p	1.595265456	1.445324881	2.305676856	0.022048937
17898	hsa-miR-99b-3p	0.422548118	1.309033281	0.553129549	0.402974464
17509	ebv-miR-BART8-5p	N/A	N/A	N/A	N/A
17961	hsa-miR-629-5p	1.295721564	0.177496038	0.229985444	0.75405371
46361	hsa-miR-1278	N/A	N/A	0.008733624	N/A
146113	3p	1.822276565	0.792393027	1.443959243	0.058558013
146114	hsa-miR-1538	3.520863658	0.019017433	0.066957787	0.014505415
146068	hsa-miR-1915-3p	4.408733624	0.031695721	0.139737991	0.193536339
146110	hsa-miR-3164	N/A	N/A	N/A	N/A
146132	ebv-miR-BART21-5p	N/A	0.066561014	N/A	N/A
145833	hsa-miR-596	N/A	0.015847861	N/A	N/A
145820	hsa-let-7c-5p	0.563071957	0.729001585	0.410480349	0.497530932
145846	hsa-let-7e-5p	2.281589853	1.492868463	3.406113537	0.037340866
147923	hsa-miR-3142	0.125423532	3.017432647	0.37845706	0.006591233
147978	hsa-miR-3140-3p	N/A	N/A	N/A	N/A
147881	hsa-miR-3115	N/A	N/A	N/A	N/A
147855	hsv2-miR-H23-3p	N/A	N/A	N/A	N/A
147771	hsa-miR-4328	N/A	N/A	N/A	N/A
147631	hsa-miR-4258	3.326961022	0.142630745	0.474526929	0.181374679
147930	hsa-miR-3144-3p	0.900935481	0.497622821	0.448326055	0.50688693
147818	hsa-miR-4270	0.612324115	0.009508716	0.005822416	0.827451153
147889	hsv1-miR-H14-3p	0.524849241	0.04437401	0.023289665	0.484834294
147904	hsa-miR-3148	1.176306852	0.18066561	0.212518195	0.050431783
148361	hsa-miR-3911	0.775962455	0.183835182	0.142649199	0.154694548
148140	hsa-miR-181d-3p	N/A	N/A	N/A	N/A
147606	hsa-miR-4259	N/A	N/A	N/A	N/A
148307	hsa-miR-3612	N/A	N/A	N/A	N/A
148221	hsa-miR-3650	0.775808708	0.326465927	0.253275109	0.749174399
147688	hsa-miR-548v	N/A	0.015847861	N/A	N/A
148688	hsa-miR-765	0.483413775	0.06022187	0.029112082	0.058410786
147311	hsa-miR-3688-3p	0.459243086	0.006339144	0.002911208	0.566392666
148659	hsv2-miR-H21	N/A	N/A	N/A	N/A
148156	hsa-miR-3686	0.738369569	5.366085578	3.962154294	0.75488967
148273	hsa-miR-3150b-3p	0.918486172	0.019017433	0.017467249	0.38575467
148118	hsa-miR-676-5p	2.105663821	1.061806656	2.23580786	0.365972508
148663	hsa-miR-557	N/A	N/A	0.008733624	N/A
148049	hsa-miR-3924	0.87459946	0.928684628	0.812227074	0.593625913
148413	hsa-miR-3614-3p	1.734918324	0.057052298	0.098981077	7.67031E-05
148482	hsa-miR-874-5p	7.347889374	0.006339144	0.04657933	0.567752323
148316	hsa-miR-134-3p	0.889303501	1.89540412	1.68558952	0.537701381
17578	kshv-miR-K12-5-3p	0.459243086	0.215530903	0.098981077	0.12170889
42764	hsa-miR-412-3p	N/A	0.015847861	N/A	N/A
17328	ebv-miR-BART8-3p	5.510917031	0.053882726	0.296943231	0.053919997
168850	hsa-miR-3191-5p	2.933230032	0.098256735	0.288209607	0.024757831

168636	hsa-miR-122-5p	N/A	N/A	N/A	N/A
168903	hsa-miR-4439	N/A	0.050713154	N/A	N/A
168687	hsa-miR-29a-3p	0.86193562	11.0681458	9.540029112	0.169580132
168914	hsa-miR-5689	4.016846191	0.237717908	0.954876274	0.008500495
168831	hsa-miR-433-3p	N/A	0.019017433	N/A	N/A
168941	hsa-miR-4501	0.164015388	0.266244057	0.043668122	0.161828608
168930	hsa-miR-5582-5p	N/A	N/A	0.002911208	N/A
168623	hsa-miR-4672	N/A	N/A	N/A	N/A
168599	hsa-miR-4453	N/A	N/A	N/A	N/A
168758	hsa-miR-4425	0.23571769	0.358161648	0.084425036	0.028315689
168818	hsa-miR-4774-5p	1.063510304	0.06022187	0.064046579	0.196824152
168967	hsa-miR-4476	0.059901272	0.145800317	0.008733624	0.027782931
168723	hsa-miR-4789-5p	N/A	N/A	N/A	N/A
168709	hsa-miR-4429	0.215225948	3.083993661	0.663755459	0.128944116
168658	hsa-miR-5196-5p	N/A	N/A	N/A	N/A
168721	hsa-miR-4701-5p	0.766010206	0.801901743	0.61426492	0.944256883
168653	hsa-miR-3158-5p	0.918486172	0.396196513	0.363901019	0.104229159
168846	hsa-miR-1273f	0.300883401	0.183835182	0.055312955	0.043146547
168915	hsa-miR-4780	5.846364154	1.45800317	8.524017467	0.031852576
169310	hsa-miR-3913-3p	N/A	0.063391442	N/A	N/A
169149	hsa-miR-4659b-5p	0.13121231	0.687797147	0.090247453	0.21853918
169065	hsa-miR-378h	0.317937521	0.082408875	0.026200873	N/A
169047	hsa-miR-4665-3p	1.607350801	0.012678288	0.020378457	N/A
169362	hsa-miR-5591-3p	N/A	0.012678288	N/A	N/A
169324	hsa-miR-4642	N/A	N/A	0.026200873	N/A
169216	hsa-miR-4701-3p	N/A	N/A	N/A	N/A
169123	hsa-miR-3184-3p	1.585597181	0.30110935	0.477438137	0.24637111
169052	hsa-miR-4719	N/A	N/A	N/A	N/A
169062	hsa-miR-3922-5p	N/A	N/A	N/A	N/A
169116	hsa-miR-4788	0.223183556	0.678288431	0.151382824	0.203766397
169100	hsa-miR-4758-3p	0.226586897	0.719492868	0.163027656	0.161941075
169369	hsa-miR-4490	N/A	0.152139461	N/A	N/A
169371	hsa-miR-4474-5p	1.033296943	0.025356577	0.026200873	N/A
169035	hsa-miR-4658	1.475769916	0.282091918	0.416302766	0.033498971
169233	hsa-miR-4643	N/A	N/A	N/A	N/A
169012	hsa-miR-4711-3p	0.737800367	0.193343899	0.142649199	0.493817666
169152	hsa-miR-5690	0.5357836	0.038034865	0.020378457	0.612980533
169404	hsa-miR-3663-5p	0.516648472	0.050713154	0.026200873	0.765898273
17536	hsa-miR-562	N/A	N/A	N/A	N/A
42934	hsa-miR-345-5p	1.065443959	0.079239303	0.084425036	0.235632895
11148	hsa-miR-515-3p	0.229621543	0.025356577	0.005822416	N/A
17653	hsa-miR-133a-5p	N/A	N/A	0.03202329	N/A
42692	hsa-miR-127-5p	N/A	0.015847861	N/A	N/A
46704	hsa-miR-548i	N/A	N/A	N/A	N/A
11044	hsa-miR-302c-3p	N/A	N/A	N/A	N/A
46511	hiv1-miR-TAR-5p	N/A	N/A	0.005822416	N/A
19582	hsa-miR-106b-5p	2.376164631	0.782884311	1.860262009	0.586164236
13147	hsa-miR-96-5p	1.920471086	0.174326466	0.334788937	0.796773885
46690	hsa-miR-1238-3p	2.755458515	0.012678288	0.034934498	0.104920329
46696	hsa-miR-1234-3p	N/A	N/A	0.029112082	N/A
46918	hsa-miR-375	0.517252739	0.6022187	0.311499272	0.627709097
46806	hsa-miR-1227-3p	1.180910792	0.133122029	0.15720524	0.400096334

27229	hsa-miR-511-5p	4.592430859	0.003169572	0.014556041	N/A
29379	hsa-miR-452-5p	1.145125617	0.244057052	0.279475983	0.808801717
17752	hsa-let-7f-5p	0.331809141	0.605388273	0.200873362	0.153918748
29490	hsa-miR-7-5p	1.762808864	0.510301109	0.899563319	0.579572953
42482	hsa-miR-591	N/A	N/A	0.008733624	N/A
17650	kshv-miR-K12-6-5p	1.198025441	0.072900158	0.087336245	0.679229176
17280	hsa-miR-15b-5p	2.487795878	3.175911252	7.901018923	0.725911149
32190	hsa-miR-607	N/A	N/A	0.017467249	N/A
10967	hsa-miR-16-5p	2.385475504	6.522979398	15.56040757	0.773049353
17613	hsa-miR-645	2.296215429	0.019017433	0.043668122	0.076479526
46440	hsa-miR-1287-5p	0.528562797	0.335974643	0.177583697	0.597427275
13178	hsa-miR-18a-3p	1.198025441	0.072900158	0.087336245	0.64116533
11092	hsa-miR-378a-5p	5.510917031	0.006339144	0.034934498	0.684251764
46406	hsa-miR-500a-3p	N/A	N/A	0.069868996	N/A
28889	hsa-miR-888-5p	N/A	N/A	0.029112082	N/A
42887	hsa-miR-331-3p	2.344423267	0.573692552	1.344978166	0.542221768
42508	hsa-miR-106a-3p	N/A	0.041204437	N/A	N/A
146044	hsa-miR-2115-5p	0.626240572	0.069730586	0.043668122	0.386816398
146074	hsa-miR-2114-5p	0.612324115	0.123613312	0.075691412	0.230393188
145986	hsa-miR-2052	N/A	N/A	0.011644833	N/A
17820	hsa-miRPlus-C1066	0.489859292	0.237717908	0.116448326	0.052493067
146069	hsa-miR-1915-5p	4.898592916	0.019017433	0.093158661	0.352124623
145946	hsa-miR-449a	N/A	N/A	0.020378457	N/A
145698	hsa-miR-496	N/A	N/A	N/A	N/A
145757	hsa-miR-33a-3p	1.180910792	0.022187005	0.026200873	0.511990698
145708	hsa-miR-324-3p	1.28588064	0.031695721	0.040756914	0.590352379
145694	hsa-miR-100-3p	N/A	N/A	N/A	N/A
147850	hsv2-miR-H11-3p	N/A	N/A	N/A	N/A
147909	hsa-miR-1273d	N/A	N/A	0.020378457	N/A
147798	hsa-miR-3141	N/A	N/A	0.005822416	N/A
148000	hsa-miR-3195	1.683891315	0.019017433	0.03202329	0.155020882
147937	hsv1-miR-H13	N/A	N/A	N/A	N/A
147584	hsa-miR-548t-5p	3.265728611	0.028526149	0.093158661	0.069779169
147914	hsa-miR-514b-5p	N/A	N/A	0.026200873	N/A
147187	hsa-miR-215-5p	0.036739447	0.079239303	0.002911208	N/A
145670	hsa-miR-18b-5p	0.419174897	0.472266244	0.197962154	0.083001328
148018	hsa-miR-3671	N/A	N/A	N/A	N/A
148168	hsa-miR-3658	0.918486172	0.012678288	0.011644833	0.366015599
148033	hsa-miR-3065-5p	0.918486172	0.04437401	0.040756914	0.319251799
148265	hsa-miR-3935	1.254961304	1.920760697	2.410480349	0.299646575
148666	hsa-miR-938	0.236182158	0.110935024	0.026200873	0.119323142
147501	hsa-miR-98-3p	2.891530541	0.085578447	0.247452693	0.448230051
148262	hsa-miR-3680-3p	N/A	N/A	0.03202329	N/A
148667	hsa-miR-617	N/A	N/A	N/A	N/A
147731	hsa-miR-3189-3p	N/A	N/A	0.034934498	N/A
148154	hsa-miR-3922-3p	N/A	N/A	0.011644833	N/A
147576	hsv1-miR-H1-3p	1.836972344	0.047543582	0.087336245	0.11874317
147268	hsa-miR-3940-3p	N/A	0.003169572	N/A	N/A
147742	hsa-miR-4265	N/A	N/A	0.017467249	N/A
148338	hsa-miR-3064-3p	0.367394469	0.063391442	0.023289665	0.743312766
148057	hsa-miR-186-3p	0.540285983	0.107765452	0.058224163	0.186298381
42702	hsa-miR-30c-1-3p	0.247284739	0.247226624	0.061135371	0.255514897

146157	hsv1-miR-H4-5p	0.196818465	0.04437401	0.008733624	0.278798199
46281	hsa-miR-1291	3.36778263	0.009508716	0.03202329	N/A
146123	hsv1-miR-H2-3p	1.055281985	0.148969889	0.15720524	0.28011466
146014	bkv-miR-B1-3p/jcv-miR-J1-3p	N/A	N/A	0.011644833	N/A
168868	hsa-miR-5681b	1.13444751	4.475435816	5.077147016	0.205435216
168871	hsa-miR-151a-5p/hsa-miR-151b	1.439727074	1.267828843	1.825327511	0.503054991
168802	hsa-miR-4516	0.273779532	0.329635499	0.090247453	0.136377683
168760	hsa-miR-4749-5p	N/A	N/A	N/A	N/A
168603	hsa-miR-4664-5p	1.102183406	0.031695721	0.034934498	0.710177489
168863	hsa-miR-4441	0.918486172	0.196513471	0.180494905	0.506554998
168948	hsa-miR-4514	1.419478629	0.069730586	0.098981077	0.096111561
168959	hsa-miR-4778-5p	0.272144051	0.342313788	0.093158661	0.03571717
168638	hsa-miR-4530	0.354228455	0.928684628	0.328966521	0.242428455
168610	hsa-miR-211-3p	N/A	N/A	0.005822416	N/A
168881	hsa-miR-4720-3p	N/A	N/A	0.002911208	N/A
168939	hsa-miR-4738-5p	N/A	N/A	N/A	N/A
168717	hsa-miR-5193	0.867459162	0.513470681	0.445414847	0.775801221
168629	hsa-miR-5087	N/A	N/A	N/A	N/A
168815	hsa-miR-4488	N/A	0.003169572	N/A	N/A
168929	hsa-miR-4648	0.841945657	0.038034865	0.03202329	N/A
168686	hsa-miR-4768-5p	2.219674915	0.076069731	0.168850073	0.338660386
168606	hsa-miR-4633-5p	1.481058952	0.253565769	0.375545852	0.03146643
168557	hsa-miR-4777-5p	0.775890145	0.694136292	0.538573508	0.245569331
168582	hsa-miR-4731-5p	N/A	N/A	N/A	N/A
169068	hsa-miR-513c-3p	0.826637555	0.095087163	0.07860262	0.114123567
169204	hsa-miR-4709-3p	1.430718844	0.659270998	0.943231441	0.052194005
169129	hsa-miR-4284	0.727610433	91.57210777	66.62882096	0.015333384
169251	hsa-miR-4653-5p	N/A	N/A	N/A	N/A
169024	hsa-miR-3960	0.633131245	0.979397781	0.620087336	0.642592832
169206	hsa-miR-4645-3p	3.320680775	0.041204437	0.136826783	0.025534909
169026	hsa-miR-4679	0.114810771	0.278922345	0.03202329	0.143261317
169028	hsa-miR-4708-3p	1.299832329	1.251980983	1.627365357	0.290949646
169229	hsa-miR-4666b	N/A	0.012678288	N/A	N/A
168991	hsa-miR-873-3p	N/A	N/A	N/A	N/A
169137	hsa-miR-4524b-5p	0.165910439	3.89540412	0.64628821	0.040793836
169259	hsa-miR-4773	0.315729622	0.101426307	0.03202329	0.020676153
168979	hsa-miR-4734	1.469577875	0.015847861	0.023289665	N/A
169140	hsa-miR-3150a-5p	N/A	N/A	N/A	N/A
169217	hsa-miR-2467-5p	N/A	N/A	N/A	N/A
169342	hsa-miR-5090	N/A	N/A	N/A	N/A
169053	hsa-miR-130b-5p	1.612951326	0.129952456	0.209606987	0.522704114
168980	hsa-miR-4324	0.653538238	0.329635499	0.215429403	0.260144615
168986	hsa-miR-4677-3p	0.436048991	0.627575277	0.273653566	0.043760932
169124	hsa-miR-548aj-3p	N/A	N/A	N/A	N/A
169391	hsa-miR-513a-3p	N/A	N/A	N/A	N/A
17539	ebv-miR-BART3-5p	N/A	N/A	N/A	N/A
42603	hsa-miR-424-3p	N/A	N/A	N/A	N/A
42786	hsa-miR-188-3p	0.05102701	0.057052298	0.002911208	0.590087181
42866	hsa-miR-451a	1.804169266	0.088748019	0.160116448	0.530218939
46335	hsa-miR-548m	N/A	N/A	N/A	N/A

17902	hsa-miR-15b-3p	0.035326391	0.082408875	0.002911208	0.031952959
46634	hsa-miR-1281	6.276322174	0.019017433	0.119359534	0.499992277
17459	hsa-miR-580-3p	N/A	N/A	0.026200873	N/A
17883	hsa-miR-19b-1-5p	0.56522226	0.082408875	0.04657933	0.224774269
17657	ebv-miR-BART18-5p	N/A	N/A	N/A	N/A
13137	hsa-miR-518e-5p/hsa-miR-519a-5p/hsa-miR-519b-5p/hsa-miR-519c-5p/hsa-miR-522-	0.699798988	0.266244057	0.186317322	0.999880879
17499	hcmv-miR-US5-1	2.296215429	0.012678288	0.029112082	N/A
29562	hsa-miR-199a-5p	N/A	N/A	0.020378457	N/A
46940	hsa-miR-548g-3p	N/A	N/A	N/A	N/A
17614	sv40-miR-S1-3p	0.42862688	0.047543582	0.020378457	N/A
17393	hsa-miR-603	3.214701601	0.006339144	0.020378457	0.326919632
10946	hsa-miR-141-3p	0.040521449	0.431061807	0.017467249	0.381279833
42710	hsa-miR-1296-5p	N/A	N/A	N/A	N/A
42674	hsa-miR-431-3p	0.441803475	1.251980983	0.553129549	0.268020451
42949	hsa-miR-155-3p	2.89676408	0.041204437	0.119359534	0.132931472
30033	hsa-miR-877-5p	0.931993321	0.64659271	0.602620087	0.843974721
13171	hsa-miR-429	1.230115409	0.532488114	0.655021834	N/A
46235	hsa-miR-524-5p	N/A	N/A	0.002911208	N/A
11024	hsa-miR-223-3p	0.85725376	0.095087163	0.081513828	0.647842325
42458	hcmv-miR-US25-1-3p	0.399341814	0.072900158	0.029112082	0.596047034
42549	hsa-miR-19a-5p	2.927674672	0.050713154	0.148471616	0.235416341
13183	hsa-miR-487a-3p	0.861080786	0.152139461	0.131004367	0.542582849
17298	hsa-miR-548b-3p	0.590455396	0.04437401	0.026200873	0.948105724
42616	ebv-miR-BART7-3p	N/A	N/A	N/A	N/A
46779	hsa-miR-1206	3.673944687	0.003169572	0.011644833	0.809742621
46829	hsa-miR-664a-5p	N/A	N/A	0.037845706	N/A
46414	hsa-miR-548h-5p	N/A	N/A	N/A	N/A
17634	hsa-miR-567	N/A	0.034865293	N/A	N/A
42532	hsa-miR-22-5p	1.884644636	0.916006339	1.726346434	0.17658467
42455	hsa-miR-520f-3p	N/A	N/A	0.005822416	N/A
42845	hsa-miR-125b-2-3p	0.773462039	0.06022187	0.04657933	0.474710892
146169	mcv-miR-M1-3p	0.339440542	0.583201268	0.197962154	0.048478231
146179	hsa-miR-2113	0.416292294	0.440570523	0.183406114	0.552361209
146202	hsv1-miR-H7-5p	N/A	N/A	N/A	N/A
146127	hsa-miR-449b-3p	0.148142931	0.098256735	0.014556041	0.29495248
30209	hsa-miR-651-3p	1.0715672	0.057052298	0.061135371	0.820248277
146180	hsa-miR-1909-3p	1.530810286	0.009508716	0.014556041	0.978501474
146134	hsv2-miR-H4-3p	N/A	N/A	N/A	N/A
146160	hsa-miR-133b	2.877923338	0.047543582	0.136826783	0.079427289
145707	hsa-miR-216b-5p	N/A	N/A	N/A	N/A
145755	hsa-miR-146a-3p	N/A	N/A	N/A	N/A
145835	hsa-miR-509-5p	N/A	N/A	0.011644833	N/A
145836	hsa-miR-218-2-3p	1.377729258	0.177496038	0.244541485	0.082358389
147843	hsa-miR-3144-5p	N/A	0.015847861	N/A	N/A
147939	hsa-miR-3192-5p	N/A	N/A	N/A	N/A
147162	hsa-let-7a-5p	0.5415352	1.096671949	0.593886463	0.075054974
147906	hsa-miR-4322	N/A	0.012678288	N/A	N/A
147931	hsa-miR-3166	N/A	N/A	N/A	N/A
147824	hsv2-miR-H9-5p	0.918486172	0.006339144	0.005822416	N/A

147894	hsa-miR-3137	N/A	N/A	0.026200873	N/A
147506	hsa-miR-21-5p	0.876517528	24.13946117	21.15866084	0.463689656
147823	hsa-miR-3146	0.806475663	0.389857369	0.31441048	0.474401183
147880	hsa-miR-323b-3p	N/A	N/A	0.037845706	N/A
148102	hsa-miR-3619-5p	N/A	N/A	0.014556041	N/A
148643	hsa-miR-642a-5p	N/A	N/A	0.037845706	N/A
148674	hsa-miR-4321	1.131871848	0.313787639	0.355167394	0.494289988
147493	hsa-miR-3944-3p	11.94032023	0.003169572	0.037845706	0.623699552
148577	hsa-miR-3943	7.80713246	0.006339144	0.049490539	0.006394246
148620	hsa-miR-454-3p	5.652222595	0.041204437	0.232896652	0.372976008
148678	hsa-miR-301a-5p	1.458772155	0.053882726	0.07860262	0.09572862
148167	hsa-miR-3670	2.372755944	0.038034865	0.090247453	0.052605712
147612	hsv1-miR-H5-5p	N/A	N/A	N/A	N/A
148217	hsa-miR-23c	0.426258776	0.573692552	0.244541485	0.336146452
148657	hsa-miR-381-5p	0.797367116	0.288431062	0.229985444	0.73758572
148418	hsa-miR-3607-5p	0.846105468	1.166402536	0.986899563	0.783739247
147603	hsa-miR-4300	0.554007532	0.199683043	0.11062591	0.517118788
147738	hsv2-miR-H20	0.523537118	0.316957211	0.165938865	0.552031132
148300	hsa-miR-370-5p	N/A	N/A	N/A	N/A
28534	hsa-miRPlus-D1058	N/A	N/A	0.002911208	N/A
17493	hsa-miR-622	0.987158035	0.339144216	0.334788937	0.32153347
17577	hsa-miR-613	N/A	N/A	N/A	N/A
168886	hsa-miR-4745-5p	N/A	N/A	N/A	N/A
168935	hsa-miR-4687-3p	0.740714655	0.196513471	0.145560408	0.091869226
168562	hsa-miR-4738-3p	N/A	0.028526149	N/A	N/A
168634	hsa-miR-548av-5p/hsa-miR-548k	N/A	N/A	N/A	N/A
168953	hsa-miR-4704-5p	0.995026686	0.114104596	0.113537118	0.626524763
168837	hsa-miR-4793-3p	N/A	N/A	N/A	N/A
168904	hsa-miR-4473	2.246198856	0.320126783	0.719068413	0.185672359
168588	hsa-miR-548at-3p	N/A	N/A	N/A	N/A
168857	hsa-miR-2392	0.247284739	0.082408875	0.020378457	0.803895034
168565	hsa-miR-4649-5p	N/A	N/A	N/A	N/A
168864	hsa-miR-320d	0.069319711	0.335974643	0.023289665	0.012812121
168652	hsa-miR-4760-3p	N/A	N/A	N/A	N/A
168869	hsa-miR-5010-3p	0.742605841	0.148969889	0.11062591	0.561252131
168920	hsa-miR-3117-5p	N/A	N/A	N/A	N/A
168860	hsa-miR-4766-5p	0.793238057	0.069730586	0.055312955	0.108518623
168901	hsa-miR-4742-5p	N/A	N/A	N/A	N/A
168705	hsa-miR-5589-3p	N/A	N/A	N/A	N/A
168586	hsa-miR-34a-5p	2.430280181	1.020602219	2.480349345	0.603256888
168672	hsa-miR-1587	0.635875042	0.288431062	0.183406114	0.340451098
168792	hsa-miR-4434	0.555925841	0.722662441	0.401746725	0.237634883
169328	hsa-miR-4769-5p	N/A	0.047543582	N/A	N/A
169383	hsa-miR-548ah-5p	0.976700084	0.900158479	0.879184862	0.236792652
169094	hsa-miR-4482-3p	1.301188743	0.076069731	0.098981077	0.440725019
169089	hsa-miR-4470	N/A	0.450079239	N/A	N/A
169209	hsa-miR-4662b	N/A	N/A	N/A	N/A
169366	hsa-miR-548ae-3p	N/A	N/A	N/A	N/A
169384	hsa-miR-1295b-5p	N/A	N/A	N/A	N/A
168983	hsa-miR-219b-3p	N/A	N/A	N/A	N/A
169184	hsa-miR-1304-3p	0.631459243	0.050713154	0.03202329	0.405381594

169292	hsa-miR-4717-5p	N/A	0.003169572	N/A	N/A
169187	hsa-miRPlus-C1110	0.635875042	0.041204437	0.026200873	0.798793596
169177	hsa-miR-4664-3p	1.305217191	0.06022187	0.07860262	0.203030833
169057	hsa-miR-4659b-3p	0.734788937	0.047543582	0.034934498	0.838386385
169185	hsa-miR-5187-3p	0.62177805	1.334389857	0.829694323	0.98367677
169261	hsa-miR-4772-3p	N/A	N/A	N/A	N/A
169161	hsa-miR-4489	10.10334789	0.022187005	0.224163028	0.047261576
169210	hsa-miR-3187-5p	N/A	N/A	N/A	N/A
169281	hsa-miR-4752	N/A	N/A	N/A	N/A
169264	hsa-miR-4762-5p	1.022362584	0.532488114	0.544395924	0.427034285
169390	hsa-miR-4800-5p	0.659281485	0.516640254	0.340611354	0.29378939
169406	hsa-miR-376b-3p	0.494569477	0.041204437	0.020378457	0.054892602
42545	ebv-miR-BART11-3p	N/A	N/A	N/A	N/A
17498	hsa-miR-601	0.506104625	0.155309033	0.07860262	0.220828916
17336	hsa-miR-618	N/A	0.123613312	N/A	N/A
13179	hsa-miR-455-5p	1.486161653	0.456418384	0.678311499	0.73539234
46401	hsa-miR-519a-3p	N/A	N/A	N/A	N/A
42882	ebv-miR-BART13-5p	N/A	N/A	N/A	N/A
10972	hsa-miR-181b-5p	0.223227019	1.251980983	0.279475983	0.027729304
17492	sv40-miR-S1-5p	0.820947817	0.716323296	0.588064047	0.661401468
11063	hsa-miR-330-3p	0.238806405	0.158478605	0.037845706	0.396873876
42852	hsa-miR-760	N/A	N/A	N/A	N/A
42673	hsa-miR-337-3p	0.614435577	0.459587956	0.282387191	0.055614793
17532	ebv-miR-BART5-5p	0.299042009	0.136291601	0.040756914	0.29925236
46272	hsa-miR-1200	1.049698482	0.022187005	0.023289665	0.267493471
17918	hsa-miR-222-5p	1.775739932	0.095087163	0.168850073	0.7700293
46419	hsa-miR-1185-5p	N/A	N/A	0.014556041	N/A
29883	hsa-miR-606	N/A	N/A	N/A	N/A
27545	hsa-miR-373-5p	N/A	N/A	0.020378457	N/A
42641	hsa-miR-145-5p	N/A	N/A	0.040756914	N/A
42951	ebv-miR-BHRF1-2-3p	0.259060202	0.123613312	0.03202329	0.144375584
17668	hsa-miR-552-3p	0.245709677	0.497622821	0.122270742	0.241364268
30687	hsa-miR-93-5p	2.983451537	0.446909667	1.333333333	0.696508497
17917	hsa-miR-873-5p	N/A	N/A	N/A	N/A
46495	hsa-miR-525-3p	N/A	N/A	0.002911208	N/A
42788	hsa-miR-924	N/A	N/A	0.029112082	N/A
46363	hsa-miR-1272	0.242618989	0.335974643	0.081513828	0.15950045
17567	kshv-miR-K12-1-5p	0.851279867	0.259904913	0.22125182	0.948306177
14270	hsa-miR-493-3p	3.214701601	0.012678288	0.040756914	0.378626962
11104	hsa-miR-422a	0.592571724	0.294770206	0.174672489	0.099276624
42694	hsa-miR-485-3p	1.066887868	0.725832013	0.774381368	0.490073935
46231	hsa-miR-519b-3p	N/A	N/A	N/A	N/A
42718	hsa-miR-130a-5p	7.80713246	0.006339144	0.049490539	0.003932809
23767	hsa-miR-759	0.05102701	0.057052298	0.002911208	0.068803489
46355	hsa-miR-548p	N/A	N/A	N/A	N/A
30592	hsa-miR-599	N/A	0.012678288	N/A	N/A
11151	hsa-miR-516b-5p	0.477020238	0.982567353	0.468704512	0.020721554
17944	hsa-miR-337-5p	N/A	N/A	0.005822416	N/A
146025	hsa-miR-2277-3p	N/A	N/A	N/A	N/A
146033	hsa-miR-103b	N/A	N/A	N/A	N/A
17841	hsa-miRPlus-C1100	0.739560294	0.244057052	0.180494905	0.335328489
42721	hsa-miR-503-3p	N/A	N/A	N/A	N/A

146129	mcv-miR-M1-5p	N/A	N/A	N/A	N/A
146185	bkv-miR-B1-5p	N/A	0.110935024	N/A	N/A
145956	hsa-miRPlus-A1083	0.030616206	0.190174326	0.005822416	0.059569266
145943	hsa-miR-100-5p	0.951653728	1.141045959	1.08588064	0.4755351
145647	hsa-miR-584-5p	1.757792501	0.551505547	0.969432314	0.167924404
145845	hsa-miR-20a-5p	2.383313256	2.206022187	5.257641921	0.502455461
147790	hsv2-miR-H7-3p	1.691948211	0.12044374	0.203784571	0.487938435
147514	hsa-miR-494-3p	N/A	N/A	N/A	N/A
147516	hsa-miR-4319	3.980106744	0.009508716	0.037845706	0.15299808
147618	hsa-miR-4261	N/A	N/A	N/A	N/A
147797	hsa-miR-4260	N/A	N/A	N/A	N/A
147893	hsv2-miR-H5	1.836972344	0.003169572	0.005822416	0.495367069
147701	hsa-miR-491-3p	1.358213562	22.15213946	30.08733624	0.644544482
147842	hsv2-miR-H11-5p	0.665902475	0.126782884	0.084425036	0.821056175
147838	hsa-miR-3194-5p	N/A	N/A	N/A	N/A
147907	hsa-miR-4312	1.353558569	0.12044374	0.163027656	0.230324851
148547	hsa-miR-3622b-3p	0.966827549	0.06022187	0.058224163	0.949003127
148358	hsa-miR-3657	N/A	N/A	0.017467249	N/A
148024	hsa-miR-3923	N/A	N/A	N/A	N/A
147545	hsa-miR-3128	N/A	N/A	N/A	N/A
147664	hsa-miR-4311	0.658913993	0.145800317	0.096069869	0.161402798
148086	hsa-miR-548aa/hsa-miR-548t-3p	2.431286925	0.053882726	0.131004367	0.502296434
147596	hsa-miR-3168	N/A	N/A	N/A	N/A
148162	hsa-miR-3661	N/A	N/A	N/A	N/A
147360	hsa-miR-95-5p	N/A	N/A	N/A	N/A
148452	hsa-miR-3678-5p	N/A	N/A	N/A	N/A
147768	hsa-miR-4257	N/A	N/A	N/A	N/A
148082	hsa-miR-3663-3p	N/A	N/A	N/A	N/A
148610	hsa-miR-26a-1-3p	8.266375546	0.003169572	0.026200873	0.928864758
17872	hsa-miR-148a-5p	0.787273862	0.04437401	0.034934498	0.85895179
11085	hsa-miR-373-3p	N/A	N/A	N/A	N/A
146032	ebv-miR-BART5-3p	N/A	N/A	N/A	N/A
168749	hsa-miR-138-1-3p	0.983511741	0.358161648	0.352256186	0.431229906
168746	hsa-miR-4756-5p	N/A	N/A	0.020378457	N/A
168798	hsa-miR-4668-5p	0.743392131	93.19175911	69.27802038	0.504328114
168911	hsa-miR-4682	3.181560348	0.307448494	0.978165939	0.018212596
168908	hsa-miR-4655-5p	0.656061551	0.022187005	0.014556041	0.225649406
168769	hsa-miR-5002-5p	0.887796896	1.328050713	1.179039301	0.721412806
168645	hsa-miR-378i	N/A	N/A	N/A	N/A
168916	hsa-miR-4670-3p	N/A	N/A	N/A	N/A
168906	hsa-miR-4703-3p	N/A	0.034865293	N/A	N/A
168889	hsa-miR-4697-5p	N/A	N/A	N/A	N/A
168834	hsa-miR-5571-5p	3.960030544	0.193343899	0.765647744	0.029701452
168853	hsa-miR-4671-5p	N/A	N/A	N/A	N/A
168946	hsa-miR-4673	N/A	N/A	N/A	N/A
168947	hsa-miR-3975	N/A	N/A	N/A	N/A
168757	hsa-miR-4713-3p	N/A	N/A	N/A	N/A
168597	hsa-miR-5699-3p	1.018081058	0.263074485	0.26783115	0.38811922
168764	hsa-miR-4435	N/A	N/A	N/A	N/A
168833	hsa-miR-4660	N/A	N/A	N/A	N/A
168788	hsa-miR-4790-5p	N/A	N/A	N/A	N/A

169014	hsa-miR-4714-3p	N/A	N/A	N/A	N/A
168978	hsa-miR-371b-5p	1.791800892	1.160063391	2.07860262	0.268923984
169228	hsa-miR-4698	0.288741741	2.228209192	0.643377001	0.155677368
169258	hsa-miR-5692c	1.078222897	0.291600634	0.31441048	0.415828889
169097	hsa-miR-4735-3p	N/A	N/A	N/A	N/A
169293	hsa-miR-4661-3p	0.796021349	0.047543582	0.037845706	0.183175375
169318	hsa-miR-4649-3p	0.765405143	0.057052298	0.043668122	0.541735329
169110	hsa-miR-4497	0.337625352	1.603803487	0.541484716	0.116059534
169234	hsa-miR-4663	N/A	N/A	N/A	N/A
169128	hsa-miR-4510	0.382702572	0.114104596	0.043668122	0.083147899
169164	hsa-miR-4705	N/A	N/A	N/A	N/A
169267	hsa-miR-548aj-5p/hsa-miR-548g-5p/hsa-miR-	N/A	N/A	N/A	N/A
169063	hsa-miR-4690-3p	N/A	N/A	N/A	N/A
169091	hsa-miR-5006-5p	0.340994866	0.529318542	0.180494905	0.012487346
169305	hsa-miR-4455	1.101813049	1.572107765	1.73216885	0.601652284
168990	hsa-miR-5688	N/A	N/A	N/A	N/A
169108	hsa-miR-4713-5p	N/A	N/A	0.005822416	N/A
169029	hsa-miR-4471	0.510270095	0.085578447	0.043668122	0.572007798
169041	hsa-miR-1245b-3p	N/A	N/A	N/A	N/A
169402	hsa-miR-625-3p	N/A	N/A	0.011644833	N/A
169410	hsa-miR-556-5p	N/A	N/A	N/A	N/A
13143	hsa-miR-301a-3p	2.68825221	0.779714739	2.096069869	0.798710723
17888	hsa-let-7a-3p	0.300099442	0.320126783	0.096069869	0.12021186
17546	hsa-miR-585-3p	N/A	N/A	0.008733624	N/A
17377	hsa-miR-600	0.549147817	1.198098257	0.657933042	0.244213953
46741	hsa-miR-1295a	N/A	N/A	0.03202329	N/A
42660	hsa-miR-144-5p	N/A	N/A	N/A	N/A
17641	hsa-miR-573	1.224648229	0.114104596	0.139737991	0.908171133
17482	hsa-miR-411-5p	N/A	N/A	N/A	N/A
42739	hsa-miR-339-5p	3.120497891	0.247226624	0.77147016	0.42365766
46214	hsa-miR-1250-5p	N/A	N/A	N/A	N/A
46398	hsa-miR-513c-5p	N/A	N/A	N/A	N/A
42957	hsa-miR-323a-3p	1.190630223	0.085578447	0.101892285	0.322019191
46467	hsa-miR-143-5p	N/A	N/A	N/A	N/A
42475	hsa-miR-221-5p	2.116511613	0.072900158	0.154294032	0.619796018
11038	hsa-miR-299-5p	0.870144794	0.06022187	0.052401747	0.550242646
46614	hsa-miR-1323	0.628437907	0.06022187	0.037845706	0.28875967
46309	hsa-miR-1226-3p	2.449296458	0.009508716	0.023289665	0.774433838
33407	hsa-miR-626	0.459243086	0.012678288	0.005822416	0.53209152
42592	hsa-miR-338-3p	6.429403202	0.012678288	0.081513828	0.031556778
11111	hsa-miR-432-5p	1.102183406	0.047543582	0.052401747	0.244165901
46717	hsa-miR-1256	N/A	0.003169572	N/A	N/A
42969	hsa-miR-10b-3p	1.508941568	0.04437401	0.066957787	0.467792708
17570	hsa-miR-589-3p	N/A	N/A	N/A	N/A
17636	ebv-miR-BART20-5p	N/A	N/A	N/A	N/A
42466	ebv-miR-BART18-3p	0.81849907	1.251980983	1.024745269	0.583151512
42497	hcmv-miR-UL70-5p	N/A	N/A	0.005822416	N/A
42902	hsa-miR-185-5p	1.066813131	0.510301109	0.544395924	0.877918474
42783	hsa-miR-197-3p	1.108015064	0.399366086	0.442503639	0.78473033
17870	hsa-miR-628-5p	N/A	N/A	N/A	N/A
10952	hsa-miR-146a-5p	N/A	N/A	0.128093159	N/A

42747	hsa-miR-93-3p	0.607613929	0.206022187	0.125181951	0.75167721
42800	hsa-miR-582-5p	0.734788937	0.015847861	0.011644833	N/A
13177	hsa-miR-143-3p	0.459243086	0.057052298	0.026200873	0.141532511
42827	hsa-miR-652-3p	1.25104151	0.183835182	0.229985444	0.668494017
46707	hsa-miR-1253	0.32206658	0.244057052	0.07860262	0.216989624
42749	hsa-miR-659-3p	0.862820343	0.418383518	0.360989811	0.797699967
42661	hsa-miR-492	0.283362755	0.297939778	0.084425036	0.074132394
11140	hsa-miR-508-3p	3.673944687	0.012678288	0.04657933	N/A
46866	hsa-miR-1321	0.869105195	1.47385103	1.280931587	0.866910426
146107	hsa-miR-762	1.836972344	0.003169572	0.005822416	N/A
146121	hsa-miR-1470	N/A	N/A	0.072780204	N/A
146153	ebv-miR-BART22	N/A	N/A	N/A	N/A
145995	hsa-miR-196b-3p	2.137246669	0.494453249	1.056768559	0.760168848
146029	hsa-miR-365b-5p	1.176810408	0.101426307	0.119359534	0.150430324
28302	hsa-miRPlus-A1015	0.48459347	35.19809826	17.05676856	0.426083929
145840	hsa-let-7f-1-3p	2.296215429	0.323296355	0.742358079	0.296360071
146112	hsa-miR-30b-5p	1.46105713	2.323296355	3.394468705	0.891693442
145640	hsa-miR-328-3p	1.734918324	0.028526149	0.049490539	0.02194543
145648	hsa-miR-598-3p	0.494569477	0.041204437	0.020378457	0.229251029
145756	hsa-miR-488-5p	N/A	N/A	N/A	N/A
147792	hsa-miR-3165	0.196818465	0.266244057	0.052401747	0.286343091
147652	hsa-miR-4293	N/A	N/A	N/A	N/A
147988	hsa-miR-3135a	0.3049935	0.830427892	0.253275109	0.121397152
147632	hsa-miR-4297	2.969771955	0.570522979	1.694323144	0.027945126
147996	hsa-miR-3127-5p	N/A	N/A	0.011644833	N/A
147917	hsa-miR-3151-5p	N/A	N/A	0.026200873	N/A
147905	hsa-miR-3139	N/A	0.022187005	N/A	N/A
147860	hsv1-miR-H16	N/A	N/A	N/A	N/A
147935	hsa-miR-3125	0.261415295	0.412044374	0.107714702	0.31308064
148243	hsa-miR-3689a-3p	11.94032023	0.003169572	0.037845706	0.066176219
148673	hsv1-miR-H15	0.13121231	0.04437401	0.005822416	0.865672165
148288	hsa-miR-550b-3p	1.808269651	0.304278922	0.550218341	0.115116134
148290	hsa-miR-3616-5p	N/A	N/A	0.002911208	N/A
148098	hsa-miR-374b-5p	1.580053135	0.453248811	0.716157205	0.921950095
147739	hsa-miR-3161	0.234025244	1.156893819	0.270742358	0.29892766
148624	hsa-miR-942-5p	1.939026363	0.057052298	0.11062591	0.548250715
148626	hsa-miR-497-3p	N/A	N/A	N/A	N/A
147314	hsa-miR-181b-2-3p	N/A	N/A	N/A	N/A
148668	hsa-miR-378a-3p	1.44151302	0.912836767	1.315866084	0.248005912
148377	hsa-miR-3653-3p	0.706067546	0.973058637	0.687045124	0.549326822
148346	hsa-miR-3929	N/A	N/A	N/A	N/A
148449	hsa-miR-3659	N/A	N/A	N/A	N/A
148536	hsa-miR-1-5p	11.57292576	0.015847861	0.183406114	0.108068585
148059	hsa-miR-493-5p	0.332975534	19.47068146	6.483260553	0.105186353
148383	hsa-miR-103a-2-5p	0.676779284	0.12044374	0.081513828	0.490332946
42570	hsa-miR-194-3p	0.290048265	0.06022187	0.017467249	0.23582849
42620	hsa-miR-188-5p	0.995026686	0.038034865	0.037845706	0.826037093
42453	hsa-miR-376b-5p/hsa-miR-376c-5p	4.592430859	0.003169572	0.014556041	N/A
168819	hsa-miR-200a-3p	1.574547723	1.109350238	1.746724891	N/A
168899	hsa-miR-1285-5p	0.444103204	0.288431062	0.128093159	0.44416723
168922	hsa-miR-5584-3p	0.677717952	0.326465927	0.22125182	0.502488077

168836	hsa-miR-4496	0.004156046	1.400950872	0.005822416	0.263501793
168840	hsa-miR-4457	N/A	N/A	N/A	N/A
168848	hsa-miR-4700-3p	0.777180607	0.082408875	0.064046579	0.616814009
168841	hsa-miR-5588-3p	0.257176128	0.158478605	0.040756914	0.059848254
168972	hsa-miR-5004-3p	0.659757673	0.22503962	0.148471616	0.922002115
168866	hsa-miR-4771	N/A	N/A	0.04657933	N/A
168729	hsa-miR-4661-5p	N/A	N/A	N/A	N/A
168659	hsa-miR-4438	N/A	N/A	N/A	N/A
168968	hsa-miR-147b	1.4569091	0.091917591	0.133915575	0.163043388
168695	hsa-miR-5589-5p	N/A	N/A	N/A	N/A
168681	hsa-miR-4751	N/A	N/A	N/A	N/A
168753	hsa-miR-4741	N/A	N/A	N/A	N/A
168960	hsa-miR-4436a	0.262424621	0.155309033	0.040756914	0.007766495
168879	hsa-miR-4460	N/A	N/A	N/A	N/A
168701	hsa-miR-4728-5p	0.344432314	0.025356577	0.008733624	N/A
46731	hsa-miR-4657	0.189632178	40.71315372	7.720524017	0.059988369
168867	hsa-miR-5003-3p	N/A	N/A	0.023289665	N/A
169121	hsa-miR-1295b-3p	N/A	N/A	N/A	N/A
169134	hsa-miR-4519	N/A	N/A	N/A	N/A
169296	hsa-miR-5004-5p	0.340180064	0.085578447	0.029112082	0.250008088
169260	hsa-miR-4436b-3p	0.3409532	0.836767036	0.285298399	0.03353311
169378	hsa-miR-4430	N/A	N/A	N/A	N/A
169322	hsa-miR-4534	0.344432314	0.076069731	0.026200873	0.017343482
169326	hsa-miR-451b	0.381678618	3.790808241	1.446870451	0.126657205
169223	hsa-miR-4680-3p	N/A	0.041204437	N/A	N/A
169069	hsa-miR-3977	N/A	N/A	N/A	N/A
169338	hsa-miR-4727-5p	N/A	N/A	N/A	N/A
169212	hsa-miR-514a-5p	0.119403202	0.633914422	0.075691412	0.290437624
169202	hsa-miR-1277-5p	N/A	N/A	N/A	N/A
169017	hsa-miR-197-5p	1.377729258	0.025356577	0.034934498	N/A
169313	hsa-miR-4800-3p	1.365719164	2.545166403	3.475982533	0.248347742
169144	hsa-miR-1273g-5p	N/A	N/A	N/A	N/A
169323	hsa-miR-4723-3p	2.185997089	0.158478605	0.34643377	0.006916701
169345	hsa-miR-5008-5p	N/A	N/A	N/A	N/A
169155	hsa-miR-4480	1.15809126	0.072900158	0.084425036	0.994023421
169381	hsa-miR-4421	0.931993321	2.58637084	2.410480349	0.946961476
168985	hsa-miR-4722-5p	0.510270095	0.085578447	0.043668122	0.829332835
169385	hsa-miR-4500	1.266053761	2.437400951	3.08588064	0.947019538
169388	hsa-miR-663a	0.141305565	0.082408875	0.011644833	0.248654347
29736	hsa-miR-656-3p	1.520772186	0.193343899	0.294032023	0.037730824
17822	hsa-miR-490-5p	0.688864629	0.076069731	0.052401747	0.271129436
14280	hsa-miR-367-3p	N/A	N/A	N/A	N/A
28396	hsa-miR-767-3p	N/A	N/A	N/A	N/A
29460	hsa-miR-553	1.428756267	0.028526149	0.040756914	N/A
42656	kshv-miR-K12-10a-3p	1.804169266	0.088748019	0.160116448	0.174608576
42668	hsa-let-7c-3p	1.417092951	0.110935024	0.15720524	0.8209079
11014	hsa-miR-214-3p	0.392270136	0.304278922	0.119359534	0.193820441
17655	ebv-miR-BART14-5p	0.357189067	0.057052298	0.020378457	0.833115223
46500	hsa-miR-520g-3p/hsa-miR-520h	0.845007278	0.079239303	0.066957787	0.615347412
19591	hsa-miR-199b-5p	N/A	0.006339144	N/A	N/A
42925	hsa-miR-409-5p	N/A	N/A	N/A	N/A

46439	hsa-miR-1243	1.102183406	0.031695721	0.034934498	0.369634467
27961	hsa-miR-891a-5p	0.562822369	2.332805071	1.312954876	0.299277134
28966	hsa-miR-574-3p	0.728618229	1.226624406	0.893740902	0.166437631
10985	hsa-miR-191-5p	2.529685378	0.567353407	1.435225619	0.383725799
46705	hsa-miR-548k	0.357189067	0.228209192	0.081513828	0.437948684
46751	hsa-miR-2278	N/A	N/A	N/A	N/A
46408	hsa-miR-1322	N/A	0.126782884	N/A	N/A
42729	hsa-miR-34c-3p	N/A	0.066561014	N/A	N/A
42889	hsa-miR-379-3p	N/A	0.025356577	N/A	N/A
13133	hsa-miR-520a-5p	0.262424621	0.155309033	0.040756914	0.055839238
11078	hsa-miR-365a-3p/hsa-miR-365b-3p	0.918486172	1.524564184	1.400291121	0.392721708
46801	hsa-miR-106a-5p	1.197366462	0.960380349	1.14992722	0.459542127
42705	hsa-miR-191-3p	N/A	N/A	N/A	N/A
46606	hsa-miR-1288-3p	N/A	N/A	0.014556041	N/A
45764	hsa-miR-302e	2.257945172	0.152139461	0.343522562	0.157204388
29852	hsa-miR-9-3p	0.680663859	0.354992076	0.241630277	0.578268942
42477	hsa-miR-324-5p	0.635391119	0.925515055	0.588064047	0.465415787
42792	hsa-miR-29b-2-5p	N/A	N/A	N/A	N/A
42828	hsa-miR-769-3p	N/A	0.025356577	N/A	N/A
21501	hsa-miR-891b	N/A	N/A	N/A	N/A
10987	hsa-miR-193b-3p	2.0136043	0.329635499	0.663755459	0.632049192
30317	hsa-miR-561-5p	N/A	N/A	N/A	N/A
17824	hsa-miRPlus-A1025	N/A	N/A	N/A	N/A
146148	hsa-miR-548q	N/A	N/A	N/A	N/A
145914	hsa-miR-135b-5p	1.611853184	0.64659271	1.042212518	0.800857606
146049	hsa-miR-28-5p	0.738069245	0.177496038	0.131004367	0.174408043
145857	hsa-miR-154-5p	0.061232411	0.047543582	0.002911208	0.799574515
147943	hsa-miR-3074-3p	0.599417986	4.06022187	2.433770015	0.031465647
147667	hsa-miR-3182	3.878279009	4.288431062	16.63173217	0.129336479
147926	hsa-miR-4329	0.359666106	0.979397781	0.352256186	0.145163631
147591	hsa-miR-4283	N/A	N/A	N/A	N/A
147933	hsa-miR-4282	N/A	N/A	0.008733624	N/A
147856	hsa-miR-3200-3p	N/A	0.012678288	N/A	N/A
147806	hsa-miR-3149	1.008533836	0.64659271	0.652110626	0.282108158
147874	hsa-miR-544b	N/A	0.031695721	N/A	N/A
147952	hsa-miR-3171	N/A	0.088748019	N/A	N/A
147755	hsa-miR-378c	0.599567362	0.228209192	0.136826783	0.104188697
147916	hsa-miR-3126-3p	0.684362246	0.161648177	0.11062591	0.992787467
148077	hsa-miR-3690	0.246423119	0.129952456	0.03202329	0.242771299
147662	hsv2-miR-H6-5p	N/A	0.019017433	N/A	N/A
148256	hsa-miR-3683	N/A	N/A	N/A	N/A
148644	hsa-miR-551b-3p	0.826637555	0.031695721	0.026200873	N/A
148648	hsa-miR-1261	0.087474874	0.066561014	0.005822416	0.000397206
148652	hsa-miR-620	1.258112454	1.362916006	1.714701601	0.253786834
148445	hsa-miR-3622b-5p	N/A	N/A	0.005822416	N/A
148550	hsa-miR-328-5p	N/A	N/A	N/A	N/A
148509	hsa-miR-328-5p	N/A	N/A	N/A	N/A
148068	hsa-miR-758-5p	1.090702329	0.304278922	0.331877729	0.240473481
17594	hsa-miR-587	N/A	N/A	N/A	N/A
168965	hsa-miR-4765	0.598497441	0.491283677	0.294032023	0.701537319
168895	hsa-miR-548ag	0.183697234	0.015847861	0.002911208	N/A

168624	hsa-miR-5581-5p	N/A	N/A	N/A	N/A
168685	hsa-miR-4432	N/A	N/A	N/A	N/A
168944	hsa-miR-4707-5p	0.174396109	0.500792393	0.087336245	0.054164478
168870	hsa-miR-1246	1.282134354	18.02852615	23.11499272	0.934870542
168660	hsa-miR-5000-5p	0.29983941	3.990491284	1.19650655	0.295594801
168832	hsa-miR-4674	5.510917031	0.003169572	0.017467249	0.137862971
168689	hsa-miR-361-3p	2.143134401	0.114104596	0.244541485	0.772542075
168639	hsa-miR-4533	0.836057926	0.494453249	0.413391557	0.538733304
168650	hsa-miR-3121-5p	1.266877478	0.091917591	0.116448326	0.215944645
168584	hsa-miR-5587-3p	N/A	N/A	N/A	N/A
168821	hsa-miR-548ax	N/A	0.003169572	N/A	N/A
168657	hsa-miR-5001-3p	1.428756267	0.114104596	0.163027656	0.505466725
168884	hsa-miR-548aq-5p	N/A	N/A	N/A	N/A
168809	hsa-miR-5698	0.176631956	0.082408875	0.014556041	0.109717349
168974	hsa-miR-4757-5p	N/A	N/A	N/A	N/A
168696	hsa-miR-4739	0.354940419	0.935023772	0.331877729	0.181769148
168741	hsa-miR-5681a	N/A	N/A	N/A	N/A
168925	hsa-miR-1273g-3p	1.479334751	16.94770206	25.0713246	0.716665954
169314	hsa-miR-4668-3p	N/A	N/A	N/A	N/A
169045	hsa-miR-4651	N/A	0.012678288	N/A	N/A
169067	hsa-miR-4691-5p	N/A	N/A	N/A	N/A
169059	hsa-miR-642a-3p	0.918486172	0.028526149	0.026200873	0.404181685
169330	hsa-miR-23b-3p	1.022186223	6.091917591	6.227074236	0.37073626
169198	hsa-miR-3145-5p	1.159507366	0.446909667	0.518195051	0.476300574
169237	hsa-miR-5697	N/A	N/A	N/A	N/A
169219	hsa-miR-5591-5p	N/A	N/A	N/A	N/A
169143	hsa-miR-4459	0.627632217	0.380348653	0.238719068	0.030697183
169002	hsa-miR-4529-5p	N/A	N/A	N/A	N/A
169015	hsa-miR-4454	0.72856753	382.9572108	279.0101892	0.236901459
169072	hsa-miR-3925-3p	20.20669578	0.003169572	0.064046579	0.173890212
169302	hsa-miR-4695-5p	N/A	0.019017433	N/A	N/A
169341	hsa-miR-4632-3p	1.616535662	0.079239303	0.128093159	0.253228195
169263	hsa-miR-4634	N/A	N/A	N/A	N/A
169244	hsa-miR-5572	0.671201433	0.082408875	0.055312955	0.088961957
169350	hsa-miR-4781-5p	N/A	N/A	N/A	N/A
169037	hsa-miR-548aw	N/A	N/A	N/A	N/A
169003	hsa-miR-4744	N/A	N/A	N/A	N/A
169021	hsa-miR-4646-5p	N/A	0.082408875	N/A	N/A

Supplementary Table 3: Gene Expression Profiling Data between control and TET1-depleted BCPAP cells.

Gene	Normalized Intensity		Annotations		
**ProbeName**	**[T-mo](normalized)**	**[T-si](normalized)**	**GenbankAccession**	**GenomicCoordinates**	**GeneSymbol**
A_23_P42935	6.10148	6.419123	NM_004333	chr7:140434478-140434419	BRAF
A_23_P117082	11.291861	11.041611	NM_015987	chr12:13127906-13127847	HEBP1
A_23_P2683	9.117691	9.155592	NM_024604	chr12:48060809-48057361	RPAP3
A_24_P358131	8.3936615	7.819448		chr2:032048352-032048291	
A_32_P14850	11.255683	11.315578	XM_005276535	chr16:74425436-74425495	
A_23_P158596	5.231988	5.0086346	NM_001040196	chr1:11808472-11808531	AGTRAP
A_23_P350107	5.5971766	5.591467	NM_030961	chr7:100733350-100733409	TRIM56
A_23_P388190	8.872561	8.735666	NM_033081	chr20:61509639-61509580	DIDO1
A_23_P106544	10.530521	10.517489	NM_020188	chr16:81009944-81009885	CMC2
A_32_P85539	5.4872293	3.8670735	NM_013320	chr12:104500200-104500259	HCFC2
A_23_P94998	8.197138	7.9646444	NM_012318	chr4:1814976-1814917	LETM1
A_23_P103905	8.909562	9.22694	NM_016406	chr1:161127456-161127942	UFC1
A_24_P497186	7.1811476	7.189038	NM_182972	chr1:234740031-234740001	IRF2BP2
A_23_P118536	6.3354917	6.7153206	NM_018042	chr17:33738460-33738401	SLFN12
A_23_P434289	3.925648	4.1427608	NM_080865	chr3:51991401-51991460	GPR62
A_33_P3326898	5.521083	5.3538465		chr9:045352252-045352193	
A_24_P67898	9.445157	9.535366	AF307332	chr10:103556891-103556832	MGEA5
A_24_P28657	8.718547	8.400198	NM_015446	chr1:247003155-247003096	AHCTF1
A_23_P2873	8.227465	8.394832	NM_182923	chr14:104142084-104143801	KLC1
A_23_P171095	4.9895034	4.9846764	NM_001145073	chrX:49645828-49645887	USP27X
A_33_P3223631	7.258556	7.776242	XM_005272716	chr7:56887238-56887179	
A_23_P213014	2.3221061	2.3900566	NM_001001290	chr4:9828161-9828102	SLC2A9
A_23_P256455	10.436537	10.460293	NM_002947	chr7:7676682-7676623	RPA3
A_33_P3217009	5.1020656	5.095834	NR_036484	chr7:121946604-121946663	FEZF1-AS1
A_23_P112846	4.6182566	3.7087674	NM_001144978	chr4:75168172-75168231	MTHFD2L
A_23_P31135	11.503753	11.362622	NM_005891	chr6:160199814-160199873	ACAT2
A_33_P3346083	4.4932013	3.916531	NM_181807	chr11:31312257-31312198	DCDC1
A_23_P332509	7.360915	7.507805	NM_015909	chr2:15319139-15307416	NBAS
A_23_P105138	9.279639	9.582962	NM_001752	chr11:34493034-34493093	CAT
A_23_P144264	6.633747	7.192122	NM_014820	chr3:100083234-100083175	TOMM70A
A_23_P100344	8.710055	8.409205	NM_014321	chr16:46731519-46731578	ORC6
A_23_P162486	2.7473912	2.9909055	NM_002831	chr12:7069332-7069391	PTPN6
A_23_P103104	5.616111	5.6489744	NM_002405	chr22:37865384-37865325	MFNG
A_23_P1170	4.294766	4.7135525	NM_174890	chr10:46112017-46111958	ZFAND4
A_33_P3785051	4.2934117	3.798913	BC062748	chr7:105206847-105206788	EFCAB10
A_23_P102517	8.977334	9.049128	NM_002601	chr2:232597716-232597657	PDE6D
A_32_P196263	7.4088945	8.118879	NM_182920	chr3:64501648-64501589	ADAMTS9
A_33_P3225690	5.8416796	6.0298996	NM_014643	chr18:74072289-74072230	ZNF516
A_24_P277934	2.3469286	2.9851525	NM_000089	chr7:94058643-94058702	COL1A2
A_23_P13914	9.233305	8.997145	NM_032656	chr12:125431661-125431602	DHX37
A_33_P3775741	11.696018	11.618288	NR_003111	chr3:129115974-129115915	RPL32P3
A_23_P163113	7.405538	7.3700233	NM_017922	chr14:45584252-45584311	PRPF39
A_23_P120557	6.4931545	6.652171	NM_017714	chr20:13370757-13370698	TASP1
A_24_P413669	4.5606623	4.685399	NM_001018053	chr1:207253693-207253752	PFKFB2
A_33_P3379886	9.762145	8.477814	NM_002006	chr4:123819317-123819376	FGF2
A_24_P915007	11.978752	12.042385	NM_052876	chr19:13251892-13251951	NACC1
A_23_P53057	6.647155	6.2605047	NM_013250	chr11:6978926-6978985	ZNF215
A_23_P49338	11.308997	10.791008	NM_016639	chr16:3072180-3072239	TNFRSF12A
A_23_P371266	3.801145	3.6944067	NM_015569	chr1:172381687-172381746	DNM3
A_23_P200874	5.4338264	5.227505	NM_022778	chr1:26604377-26604436	CEP85
A_23_P11262	8.691206	8.863168	NM_012151	chrX:154116220-154116279	F8A1
A_23_P103511	8.581188	7.5406065	NM_001085375	chr1:162356483-162356542	C1orf226
A_24_P89080	8.350286	8.33107	NM_000788	chr4:71895933-71895992	DCK
A_23_P157607	9.579358	9.719589	NM_018142	chr8:19709238-19709297	INTS10
A_23_P145777	13.548189	13.682939	NM_002489	chr7:10973244-10973185	NDUFA4

A_23_P107693	4.4624724	4.968377	NM_017652	chr19:58291275-58291334	ZNF586
A_23_P160828	5.9016857	5.7280655	NM_017891	chr1:1017648-1017589	C1orf159
A_23_P120237	10.075642	9.845373	NM_020151	chr2:96851060-96851001	STARD7
A_23_P99360	6.7248673	6.991843	NM_213590	chr13:50592487-50592546	TRIM13
A_33_P3329339	3.9376726	2.3900566	NR_046244	chr3:24541439-24541498	THRB-AS1
A_32_P34876	5.196819	5.182065	NM_020212	chr15:90281345-90281404	WDR93
A_32_P210642	8.814714	8.921987	NM_201446	chr9:139566984-139567043	EGFL7
A_32_P50123	5.61018	5.4086924	NM_001271870	chr1:143916261-144013912	SRGAP2B
A_23_P10995	5.7007732	5.447278	NM_014483	chr3:29925698-29938927	RBMS3
A_33_P3315314	11.575678	11.773636	NM_001276687	chr1:237167614-237167555	MT1HL1
A_23_P120414	10.999892	10.882481	NM_003404	chr20:43536954-43537013	YWHAB
A_23_P9280	6.6087503	6.7148824	NR_029410	chr9:88454999-88455058	LOC389765
A_23_P104146	8.77069	8.876785	NM_005095	chr1:35887010-35887069	ZMYM4
A_23_P122228	10.832217	10.799177	NM_004553	chr5:1802479-1814502	NDUFS6
A_23_P213518	11.471643	11.369351	NM_001042440	chr5:96110256-96110315	CAST
A_33_P3406939	7.6054716	7.8756385	NM_194313	chr9:34252568-34252509	KIF24
A_23_P77160	9.120805	9.1233015	NM_017610	chr15:59388241-59388300	RNF111
A_33_P3388745	10.054527	10.814599	AK123339	chr3:129327672-129327731	LOC100132207
A_23_P331598	7.303387	6.6328263	NM_006391	chr11:9467009-9467068	IPO7
A_33_P3372297	4.6034927	4.2484884	NM_001159746	chr17:914102-914043	ABR
A_23_P118493	9.284735	9.82879	NM_005486	chr17:53038843-53038902	TOM1L1
A_23_P100203	9.035399	9.322905	NM_001537	chr16:83846244-83846303	HSBP1
A_33_P3343106	6.2234874	6.0606813	NM_014209	chr19:36135710-36135769	ETV2
A_23_P355455	5.660327	5.3956013	NM_014744	chr3:17200038-17199979	TBC1D5
A_23_P109345	12.4784975	12.449509	NM_004339	chr21:46269935-46269876	PTTG1IP
A_24_P381136	4.574436	4.382963	NM_016223	chr11:47200985-47200795	PACSIN3
A_23_P370097	9.377767	8.978645	NM_001044385	chr2:202488995-202488936	TMEM237
A_23_P70998	8.437103	8.228438	NR_034084	chr7:7918719-7918778	RPA3-AS1
A_23_P109055	4.8447123	4.8151436	NM_033550	chr20:45314596-45314537	TP53RK
A_23_P214211	8.985145	8.869719	NM_020320	chr6:88224675-88224159	RARS2
A_23_P106505	7.3316326	7.1672993	NM_014793	chr15:43620314-43620255	LCMT2
A_24_P158089	2.4718862	2.9971216	NM_000602	chr7:100781555-100781614	SERPINE1
A_23_P120048	5.208634	5.337511	NM_013450	chr2:160175985-160175926	BAZ2B
A_33_P3301445	8.604591	9.002659		chr10:088818952-088818893	
A_23_P215214	6.3087463	6.8068495	NM_022458	chr7:156473705-156473646	LMBR1
A_23_P45365	7.8164945	7.961656	NM_033380	chrX:107940580-107940639	COL4A5
A_23_P250800	4.590456	4.9691033	NM_006100	chr3:98510738-98510797	ST3GAL6
A_23_P321160	4.338269	4.192491	NM_032530	chr17:5082968-5082909	ZNF594
A_23_P134454	11.711741	11.50742	NM_001753	chr7:116200742-116200801	CAV1
A_23_P217475	6.833783	6.950801	NM_006123	chrX:148568759-148568700	IDS
A_23_P89509	10.740824	10.778682	NM_006461	chr17:26905097-26905038	SPAG5
A_23_P253524	8.983018	9.048733	NM_001813	chr4:104027398-104027339	CENPE
A_23_P114405	11.760796	11.966198	NM_012286	chrX:102930926-102930867	MORF4L2
A_33_P3409508	4.776958	5.261626	NM_002751	chr22:50703284-50703225	MAPK11
A_23_P254353	5.2736177	5.607813	NM_006647	chr9:140328686-140328745	NOXA1
A_23_P102391	2.8466818	4.089618	NM_014585	chr2:190425809-190425750	SLC40A1
A_23_P56810	8.975378	8.864941	NM_018158	chr2:27911628-27916604	SLC4A1AP
A_23_P124164	7.6659484	7.3923244	NM_020701	chr3:128853750-128853691	ISY1
A_33_P3225552	3.9054732	3.362534	AF132198	chr4:76877229-76877288	SDAD1
A_23_P98431	9.611312	9.365772	NM_000190	chr11:118964129-118964188	HMBS
A_23_P51397	11.956767	11.441853	NM_001008493	chr1:225683355-225683296	ENAH
A_23_P108554	11.830832	11.696658	NM_004939	chr2:15770975-15771034	DDX1
A_33_P3372788	7.0087047	6.9545684	NM_001037501	chr1:148023663-148023604	NBPF8
A_24_P122732	9.913929	9.669206	NM_173854	chr1:205758434-205758375	SLC41A1
A_33_P3269864	3.9032073	3.039041	NR_028335	chr17:2310351-2310292	LOC284009
A_23_P211504	9.47694	10.03593	NM_016657	chr22:38877404-38877463	KDELR3
A_23_P114095	4.4541187	4.760958	NM_015884	chrX:21900749-21900808	MBTPS2
A_33_P3424577	5.422681	5.5493298	A23747		
A_33_P3362567	6.733415	6.952334		chr2:203065870-203065811	

A_33_P3323607	6.552709	6.728293		chr7:31553759-31553818	CCDC129
A_33_P3291877	6.7812734	6.7709293	NM_020732	chr6:157469939-157469998	ARID1B
A_23_P24997	11.91446	11.769716	NM_000075	chr12:58142322-58142263	CDK4
A_23_P48088	5.3811665	3.5196304	NM_001242	chr12:6560640-6560699	CD27
A_23_P103149	11.093978	11.325022	NM_001098	chr22:41924818-41924877	ACO2
A_23_P110492	8.482451	8.350286	NM_005885	chr5:10435312-10435371	MARCH6
A_23_P20876	6.494845	6.4859343	NM_177995	chr9:96871739-96871798	PTPDC1
A_33_P3234222	5.8665214	5.907586	NM_001010873	chr6:41011969-41012028	TSPO2
A_33_P3354569	8.658588	7.2897186	NM_001083112	chr2:157442855-157442914	GPD2
A_24_P132470	9.024059	8.864455	NM_020728	chr7:158523966-158523907	ESYT2
A_24_P281988	5.290047	5.6380105	NM_015184	chr3:17109488-17109547	PLCL2
A_24_P410086	10.891301	11.038669	NM_032627	chr19:18545192-18545251	SSBP4
A_33_P3235454	11.1771145	11.279803		chr15:060682740-060682799	
A_23_P37778	7.907386	7.746727	NM_013241	chr16:67263789-67263730	FHOD1
A_33_P3409513	7.362008	7.594404	NM_002751	chr22:50702337-50702278	MAPK11
A_24_P200162	11.302273	11.208988	NM_014056	chr3:42826804-42826745	HIGD1A
A_23_P5983	5.7398787	5.7996216	NM_006227	chr20:44527701-44527642	PLTP
A_23_P386254	3.780123	3.549402	NM_001189	chr4:13542835-13542776	NKX3-2
A_23_P409516	8.163386	8.205421	NM_138333	chr9:71396108-71396167	FAM122A
A_33_P3270404	5.533292	5.6708155	NM_001077198	chr2:220084835-220084776	ATG9A
A_23_P128613	9.086022	9.388833	NM_024089	chr13:103436747-103436688	KDELC1
A_33_P3349384	5.0847545	4.547609		chr12:55828616-55828557	
A_23_P114656	11.512401	11.811838	NM_004964	chr1:32799037-32799096	HDAC1
A_33_P3253365	5.529438	5.810566	AB051501	chr9:43908953-43909012	
A_23_P102202	11.297329	11.222994	NM_000179	chr2:48033919-48033978	MSH6
A_33_P3388618	5.455374	5.981595	NM_001251902	chr17:7292997-7293056	TNK1
A_23_P36397	3.9873857	2.3900566	NM_000785	chr12:58156635-58156576	CYP27B1
A_23_P8083	3.850131	4.3208127	NM_025261	chr6:31686608-31686549	LY6G6C
A_32_P212471	3.4118347	4.404833	NM_001144956	chr1:151701998-151702057	RIIAD1
A_33_P3215768	7.5619473	6.984602	NM_007210	chr12:51745941-51745882	GALNT6
A_23_P206441	8.35522	8.058582	NM_000135	chr16:89804072-89804013	FANCA
A_23_P153037	4.8419466	5.003033	NM_020787	chr17:16524397-16524338	ZNF624
A_33_P3310552	4.8645525	5.2693844	XM_005246263	chr2:153501972-153502031	FMNL2
A_24_P151498	8.202114	8.699926	NM_206962	chr21:48064366-48068394	PRMT2
A_33_P3237977	10.803867	10.739543	NM_197958	chr15:71143871-71143812	LARP6
A_23_P102412	7.8052588	7.129819	NM_019091	chr2:179369325-179369384	PLEKHA3
A_23_P134109	8.348719	8.663328	NM_001431	chr6:131161674-131161615	EPB41L2
A_33_P3392580	7.9405785	7.345918	NM_013411	chr1:33473692-33473633	AK2
A_23_P145761	8.191435	8.431735	NM_005738	chr7:12728281-12728340	ARL4A
A_23_P104372	11.417451	11.359117	NM_015190	chr10:75002733-75002674	DNAJC9
A_23_P321320	8.789278	8.72299	NM_052902	chr2:220481096-220481155	STK11IP
A_23_P209519	2.3626873	2.3900566	NM_001039550	chr2:220148183-220149384	DNAJB2
A_23_P108948	5.1649756	4.914299	NM_018000	chr2:216808159-216808100	MREG
A_23_P160406	10.625783	10.651138	NM_016121	chr1:215794818-215794877	KCTD3
A_23_P111635	6.3994718	6.4115086	NM_016086	chr7:75651210-75643188	STYXL1
A_24_P170753	6.718912	6.493614	AK090610	chr12:132589812-132593138	EP400NL
A_23_P212196	7.0576954	7.0922	NM_016828	chr3:9808037-9808096	OGG1
A_24_P406754	8.507337	8.605041	NM_032211	chr10:100007805-100007746	LOXL4
A_24_P400172	5.7769995	5.271161	NR_026966	chr2:178148443-178148384	LOC100130691
A_24_P7790	3.4452565	2.3900566	NM_172006	chr20:44313580-44313521	WFDC10B
A_23_P304682	8.021553	8.322578	NM_001424	chr16:10622683-10622624	EMP2
A_33_P3358295	6.5046453	6.7245264	NM_032548	chr3:127399373-127399432	ABTB1
A_23_P204423	11.072306	11.249504	NM_002710	chr12:111158224-111158165	PPP1CC
A_23_P104025	9.550377	9.166455	NM_052965	chr1:184042890-184042949	TSEN15
A_23_P110846	7.9280467	8.368755	NM_004779	chr5:154255813-154255872	CNOT8
A_23_P252335	10.0028	9.92042	NM_018944	chr21:33641386-33641327	MIS18A
A_23_P425752	4.845662	4.290412	NM_033219	chr9:100849857-100849798	TRIM14
A_23_P117506	8.204364	8.413242	NM_016029	chr14:60616799-60616244	DHRS7
A_23_P316741	11.368554	11.451174	NM_001025237	chr11:866883-866942	TSPAN4

A_23_P3592	4.8933277	5.047087	NM_001040667	chr16:67203615-67203674	HSF4
A_33_P3364060	7.6497602	7.9551888	NM_005144	chr8:21972885-21972826	HR
A_23_P434919	8.901342	9.02135	NM_152304	chr1:28920713-28920772	RAB42
A_33_P3231878	9.97661	9.70004	NM_002645	chr11:17108358-17108299	PIK3C2A
A_24_P19810	7.844189	7.8842278	NM_024664	chr1:42925441-42925500	PPCS
A_33_P3256565	4.067924	3.5714874		chr7:057928889-057928948	
A_23_P122001	7.862738	7.761674	NM_002439	chr5:80169024-80169083	MSH3
A_33_P3419696	10.151905	8.8896885	NM_002006	chr4:123819331-123819390	FGF2
A_24_P58620	6.144579	5.9264	NM_001286563	chr11:123489842-123489901	GRAMD1B
A_33_P3405459	5.300635	4.9095044	NM_024059	chr20:62187956-62188015	C20orf195
A_23_P8416	5.1639233	5.3874063	NM_022087	chr7:151817927-151817986	GALNT11
A_23_P355623	5.674665	5.6966233	NM_019004	chr7:92029770-92029829	ANKIB1
A_23_P102770	10.25984	9.711737	NM_016100	chr20:20013859-20013918	NAA20
A_23_P205575	7.813557	6.7960086	NM_022571	chr14:59930349-59930290	GPR135
A_23_P112026	3.5531652	3.1795416	NM_002164	chr8:39785529-39785588	IDO1
A_33_P3315410	10.260192	9.128119	NM_004645	chr17:55015642-55015583	COIL
A_23_P250118	7.946102	7.6304746	NM_024610	chr3:122459319-122459260	HSPBAP1
A_23_P120442	5.15201	5.653133	NM_181659	chr20:46283630-46283689	NCOA3
A_23_P10206	2.3221061	2.3900566	NM_005328	chr8:122626146-122626087	HAS2
A_24_P48723	10.556958	9.837924	NM_000961	chr20:48120507-48120448	PTGIS
A_23_P412321	3.642667	3.111399	NM_000579	chr3:46417558-46417617	CCR5
A_33_P3222019	6.921363	6.94669	NM_014653	chr12:108589508-108589567	WSCD2
A_33_P3619819	14.263504	14.263504		chr11:77854072-77854131	KCTD21-AS1
A_33_P3219682	7.486825	7.614846		chr6:147486881-147486940	
A_33_P3252612	15.945585	16.026499	NM_017781	chr7:1029119-1029178	CYP2W1
A_23_P115645	2.3221061	2.8813477	NM_001025076	chr10:11367869-11367928	CELF2
A_24_P191664	7.166096	6.987988	NM_014498	chr3:167727993-167727934	GOLIM4
A_33_P3318653	6.7826138	6.21848	NM_001005479	chr3:97983847-97983906	OR5H6
A_23_P218807	7.5763283	7.213853	NM_017590	chr22:41755957-41756016	ZC3H7B
A_23_P14543	5.9825196	5.9673023	NM_006020	chr14:78139939-78139880	ALKBH1
A_33_P3210647	5.8061104	6.0408564	NM_001856	chr1:32148595-32148536	COL16A1
A_33_P3341105	5.136415	5.0608788	NM_002602	chr17:79617549-79617490	PDE6G
A_23_P50096	14.009318	13.589389	NM_001071	chr18:673276-673335	TYMS
A_33_P3264121	12.108402	11.900848	NM_004607	chr5:76987202-76987143	TBCA
A_32_P142128	5.466263	5.300311	NR_026566	chr10:46737617-46737612	BMS1P1
A_24_P384569	5.348492	5.3011765	NM_001163287	chr22:29686421-29686480	EWSR1
A_23_P123905	10.683936	10.520412	NM_016042	chr9:37780699-37780640	EXOSC3
A_23_P501010	7.8214617	6.6300144	NM_000494	chr10:105791142-105791083	COL17A1
A_23_P107432	4.0481596	3.4727798	NR_104343	chr17:18317599-18317658	FLJ35934
A_33_P3281816	6.9013505	6.9268894	NM_006367	chr1:40529942-40530001	CAP1
A_23_P115202	5.346184	5.3413167	NM_016190	chr1:152381792-152381733	CRNN
A_33_P3257528	5.7896137	5.6786747	NM_015555	chr6:56963879-56963938	ZNF451
A_23_P115167	4.2073326	4.682435	NM_015350	chr1:90058368-90058427	LRRC8B
A_32_P131143	10.150496	10.38434	NR_024482	chr22:17646234-17646293	CECR5-AS1
A_23_P88404	4.8881063	5.0028057	NM_003239	chr14:76424963-76424904	TGFB3
A_23_P106741	10.895468	11.054829	NM_002811	chr16:74340102-74340161	PSMD7
A_23_P3221	10.718498	10.673907	NM_021199	chr15:45983364-45983423	SQRDL
A_23_P74526	6.253726	6.10148	NM_203456	chr1:40211127-40214590	PPIE
A_23_P117274	5.580777	5.8766994	NM_005932	chr13:24411757-24411698	MIPEP
A_33_P3211804	7.8113656	7.8900347	NM_001001890	chr21:36164142-36164083	RUNX1
A_33_P3335682	8.435082	8.831393	NM_033256	chr19:38741996-38741937	PPP1R14A
A_23_P105625	8.557239	8.394559	NM_032314	chr12:120941375-120941316	COQ5
A_23_P388812	6.7942705	6.8961635	NM_152515	chr2:113495817-113495758	CKAP2L
A_23_P133284	8.984365	8.948069	NM_018343	chr5:96498802-96498743	RIOK2
A_23_P30736	5.295876	5.356612	NM_002120	chr6:32780722-32780663	HLA-DOB
A_23_P53137	2.3221061	2.3900566	NM_000559	chr11:5269593-5269534	HBG1
A_24_P91094	3.9488416	3.8870652	NM_006695	chr17:42393791-42393850	RUNDC3A
A_33_P3215239	8.109566	8.27455	NM_001284259	chr10:91522529-91522588	KIF20B
A_23_P115492	5.852904	5.9482746	NM_024749	chr1:213163328-213163387	VASH2

A_32_P169574	5.2628107	5.298292	NM_017602	chrX:48783309-48783250	OTUD5
A_33_P3242418	9.120598	9.509018	AK128128	chr16:33347299-33347240	
A_23_P308021	6.9596243	6.834096	NM_145200	chr11:67225969-67226140	CABP4
A_23_P139722	10.122078	10.14983	NM_001065	chr12:6438123-6438064	TNFRSF1A
A_23_P120428	4.811366	4.849025	NM_014477	chr20:44005865-44004147	TP53TG5
A_24_P393470	8.104538	7.9208126	NM_001145783	chr19:19292747-19292688	MEF2BNB
A_23_P54636	9.625463	9.802909	NM_004691	chr16:67472152-67472093	ATP6V0D1
A_24_P22050	5.468662	5.660445	NM_017817	chr13:111176078-111176019	RAB20
A_23_P24444	9.63634	9.558603	NM_001360	chr11:71145573-71145514	DHCR7
A_23_P107724	6.271994	6.190667	NM_013380	chr19:44830983-44830924	ZNF112
A_23_P23669	4.9889913	4.6992245	NM_017734	chr1:100155262-100155321	PALMD
A_33_P3386181	4.951394	4.99088	NM_018197	chr20:50768744-50768685	ZFP64
A_23_P395524	10.801881	10.689648	NM_007062	chr12:108106141-108106200	PWP1
A_24_P398092	9.362856	9.106262	NM_002874	chr9:110094244-110094303	RAD23B
A_23_P316531	5.249217	5.303042	NM_005298	chr1:200843145-200843204	GPR25
A_23_P37441	11.180854	11.488429	NM_004048	chr15:45007773-45007832	B2M
A_23_P137035	11.477748	11.275029	NM_003662	chrX:15403117-15403058	PIR
A_24_P405002	6.299017	6.3209696	NM_152835	chr1:26451687-26451746	PDIK1L
A_23_P139509	8.935379	9.190092	NM_016570	chr12:29498382-29496163	ERGIC2
A_23_P87664	7.166331	7.3521037	NM_014706	chr12:108917104-108917045	SART3
A_23_P79794	4.4754853	4.126813	NM_021809	chr20:35221586-35221645	TGIF2
A_33_P3305655	4.2184744	3.8910928	NR_027992	chr15:20874916-20874857	NBEAP1
A_23_P150583	5.934448	5.778198	NM_003357	chr11:62189823-62190532	SCGB1A1
A_33_P3296372	8.604095	7.9445453	NM_020365	chr1:45316573-45316514	EIF2B3
A_23_P110879	5.07927	5.013651	NM_147686	chr6:111894122-111888861	TRAF3IP2
A_23_P310350	11.797928	12.235644	NM_013276	chr17:3512057-3511998	SHPK
A_23_P416314	10.393925	10.082521	NM_054108	chr11:63229094-63229035	HRASLS5
A_23_P122624	9.630812	9.346394	NM_032860	chr6:144184627-144184686	LTV1
A_23_P83976	6.6220837	6.8404818	NM_145036	chr17:63685309-63685250	CEP112
A_33_P3253427	7.486617	7.4304924	NR_015341	chr17:28935520-28935579	LRRC37BP1
A_33_P3396646	5.0289464	4.9027243	NM_001080450	chr6:107389936-107389877	BEND3
A_23_P58321	8.849172	9.268862	NM_001237	chr4:122740054-122739995	CCNA2
A_23_P118002	11.494883	11.270748	NM_003366	chr16:21994578-21994637	UQCRC2
A_23_P214139	9.345391	9.439686	NM_002912	chr6:111620573-111620514	REV3L
A_24_P218001	5.9021015	6.1763186	NM_021148	chr7:64378605-64378664	ZNF273
A_32_P94087	13.909961	13.917352	NR_027153	chr4:54853065-54853006	RPL21P44
A_23_P102420	12.702684	12.37811	NM_006430	chr2:62095734-62095675	CCT4
A_23_P101208	11.618288	11.73415	NM_001914	chr18:71920777-71920718	CYB5A
A_24_P86868	9.667	9.883261	NM_212554	chr10:126448941-126448882	METTL10
A_33_P3387463	8.940174	8.3718815	NM_015994	chr14:67804649-67804590	ATP6V1D
A_23_P103256	7.2398353	7.893263	NM_021023	chr1:196748420-196748479	CFHR3
A_23_P201845	7.193121	7.3143144	NM_007167	chr1:35470800-35470741	ZMYM6
A_23_P125078	9.241953	9.612076	NM_173626	chr17:78227156-78227215	SLC26A11
A_23_P103414	9.21717	9.06761	NM_016258	chr1:29095983-29096042	YTHDF2
A_23_P257538	6.5717278	5.760868	NM_006606	chr20:18467660-18467601	RBBP9
A_23_P110196	7.910946	8.007641	NM_016323	chr4:89426889-89426948	HERC5
A_23_P109026	8.882342	9.052754	NM_022358	chr20:43379593-43379652	KCNK15
A_23_P381449	6.1564054	6.1012363	NM_003110	chr17:46005939-46005998	SP2
A_23_P19938	11.905529	12.262457	NM_006854	chr7:6502520-6502461	KDELR2
A_24_P913339	7.014399	6.820813	NM_017877	chr2:27003342-27003401	SLC35F6
A_23_P102864	2.3309216	2.3900566	NM_002772	chr21:19647550-19642419	TMPRSS15
A_23_P102320	9.1714	9.036449	NM_138285	chr2:184026315-184026374	NUP35
A_23_P120270	9.75067	9.582312	NM_139279	chr2:47132606-47132547	MCFD2
A_23_P110569	4.8611126	4.7419868	NM_018700	chr5:114460760-114460701	TRIM36
A_23_P46844	8.393177	8.081436	NM_030912	chr10:104417554-104417613	TRIM8
A_33_P3310371	4.7907248	4.667725	NR_027436	chrUn_gl000219:78868-58957	LOC283788
A_23_P151820	5.140075	4.4519806	NM_024832	chr14:93154782-93154841	RIN3
A_23_P33196	5.7303677	5.807313	NM_000393	chr2:189897488-189897429	COL5A2
A_23_P162589	8.958401	8.622114	NM_001017535	chr12:48235561-48235502	VDR

A_33_P3264346	7.4653263	7.7923717		chr16:3050184-3050243	
A_24_P369898	4.523347	3.5473418	BC128044	chr17:73616680-73616739	MYO15B
A_33_P3238461	13.992674	14.054972		chr2:016010261-016010320	
A_23_P123539	8.538822	8.750403	NM_002717	chr8:26228096-26228155	PPP2R2A
A_23_P119143	2.974934	3.5008297	NM_003259	chr19:10407322-10407381	ICAM5
A_23_P206369	11.310212	11.112316	NM_014187	chr16:67263111-67263170	TMEM208
A_23_P251767	4.855671	4.0712385	NM_017869	chr16:88066814-88068950	BANP
A_33_P3260307	9.693462	9.434108	NM_001042476	chr16:8946862-8946803	CARHSP1
A_23_P10182	7.210951	6.2436275	NM_003500	chr3:58490967-58490908	ACOX2
A_23_P11025	6.678634	6.3494077	NM_001178106	chrX:152141632-152141691	ZNF185
A_24_P940921	9.201248	8.6097145	NM_015046	chr9:135137022-135136963	SETX
A_23_P114689	6.5488605	6.7114954	NM_017707	chr1:23755346-23755287	ASAP3
A_23_P252748	9.811802	9.586176	NM_007152	chr11:3380673-3380614	ZNF195
A_23_P120467	5.971603	5.757236	NM_199427	chr20:50700673-50700614	ZFP64
A_23_P422044	4.853849	5.0588627	NM_032009	chr5:140720840-140720899	PCDHGA2
A_33_P3335902	4.056716	3.7203805	NM_001184768	chrX:100870867-100870808	ARMCX6
A_33_P3349637	8.419403	8.5352335	NM_002587	chr5:141242277-141242218	PCDH1
A_23_P169470	9.769509	9.482103	NM_001261	chr9:130552256-130552315	CDK9
A_23_P112159	9.765581	10.075642	NM_012154	chr8:141542006-141541947	AGO2
A_23_P109442	8.289227	8.043175	NM_022081	chr22:26848159-26848100	HPS4
A_23_P131024	2.3221061	2.3900566	NM_014383	chr19:36207866-36207925	ZBTB32
A_33_P3343705	3.4658313	2.3900566	NR_037597	chr2:95596591-95596532	LOC442028
A_24_P9671	11.691533	11.674963	NM_001539	chr9:33036682-33037066	DNAJA1
A_33_P3346826	4.307011	4.2527356	NM_001012633	chr16:3119014-3119073	IL32
A_23_P168080	4.0015106	4.009615	NM_006929	chr6:31936701-31936760	SKIV2L
A_33_P3284584	11.647006	11.79233		chr7:138089164-138089105	XLOC_014512
A_24_P729905	7.663861	7.525032	NM_001077685	chr10:51483236-51483177	AGAP7
A_33_P3377005	6.827089	6.7141123	NM_173573	chr11:554917-554858	C11orf35
A_24_P270769	11.42845	11.554306	NM_018206	chr16:46694239-46694180	VPS35
A_23_P123563	14.867456	14.878323	NM_001010	chr9:19376365-19376306	RPS6
A_23_P105562	3.6945791	3.2275832	NM_000552	chr12:6058242-6058183	VWF
A_23_P115356	10.173088	10.18672	NM_003689	chr1:19630652-19630593	AKR7A2
A_23_P112634	6.1745605	6.770384	NM_174921	chr4:39553321-39553262	SMIM14
A_33_P3354851	5.6723332	5.5536327	X58768	chr14:22636811-22636870	
A_32_P180265	6.147812	6.4382086		chr9:67786621-67786680	
A_33_P3251252	5.5942254	5.694901		chr3:045718927-045718868	
A_24_P93741	6.290715	5.7658234	NM_052859	chr3:53122589-53122530	RFT1
A_23_P102037	5.645531	5.896036	NM_025147	chr2:198338889-198338948	COQ10B
A_33_P3284763	4.8914747	4.624734	NM_004021	chrX:31152279-31152220	DMD
A_23_P122863	7.966835	7.5763283	NM_001001555	chr7:50657786-50657752	GRB10
A_23_P120710	8.228674	8.509924	NM_003316	chr21:38574855-38574914	TTC3
A_24_P167970	4.2289557	3.8149638	NM_004608	chr16:30100056-30099920	TBX6
A_23_P82299	9.886154	9.7054825	NM_145030	chr7:100032975-100032916	PPP1R35
A_33_P3878964	8.963385	8.858656	NM_024953	chr12:112465702-112465643	NAA25
A_24_P934800	7.28886	7.3256874	NM_001142725	chr16:20808395-20808336	ERI2
A_23_P74290	2.3221061	2.8440819	NM_052942	chr1:89726446-89726387	GBP5
A_23_P113825	9.291588	9.388187	NM_144653	chr9:138903283-138903224	NACC2
A_23_P102364	9.27188	8.84148	NM_019850	chr2:233743527-233743468	NGEF
A_24_P115007	7.306528	7.0761576	NM_170740	chr6:24536869-24536928	ALDH5A1
A_23_P63178	8.685317	8.686109	NM_005644	chr1:28929789-28929730	TAF12
A_23_P106727	7.6983104	7.5072203	NM_014700	chr16:572086-572145	RAB11FIP3
A_23_P148150	8.070583	8.070492	NM_015456	chr9:140167572-140167631	NELFB
A_24_P149713	5.6851397	5.485355	NM_014007	chr9:129598599-129598658	ZBTB43
A_23_P141208	7.051969	6.9843864	NM_015510	chr17:21094329-21094388	DHRS7B
A_33_P3404899	2.3221061	2.3900566	NM_012302	chr1:82450973-82451032	LPHN2
A_33_P3222598	9.422632	9.279421		chr21:039874471-039874530	
A_23_P80778	8.384398	8.215807	NM_032839	chr3:122598486-122598545	DIRC2
A_24_P49597	11.478488	11.151855		chr18:057429001-057429060	
A_23_P101185	11.611178	11.361875	NM_004539	chr18:55268249-55268190	NARS

A_23_P121064	8.446472	7.9166727	NM_002852	chr3:157160969-157161028	PTX3
A_33_P3291160	4.572304	4.369715		chr9:139948324-139948383	
A_23_P403955	8.525805	8.192653	NM_007375	chr1:11085296-11085355	TARDBP
A_24_P135921	7.4369993	7.157545		chrX:103892628-103892569	
A_24_P271014	8.12757	8.095913	NM_001042388	chr18:9547630-9547571	PPP4R1
A_23_P111621	9.192605	9.347628	NM_005685	chr7:74016831-74016890	GTF2IRD1
A_23_P108501	8.909851	9.682512	NM_004438	chr2:222290776-222290717	EPHA4
A_32_P66222	4.5405846	4.0936646	NM_001012421	chr9:42372006-42372227	ANKRD20A2
A_33_P3235640	4.153529	3.8393123	NM_001193273	chr1:205064098-205064039	RBBP5
A_33_P3784283	6.971564	7.821193	NM_002227	chr1:65299832-65299773	JAK1
A_23_P163278	6.0894394	6.1675234	NM_017851	chr15:65550972-65550913	PARP16
A_33_P3338036	4.7405963	5.1807833	NM_001001668	chr19:57089867-57089926	ZNF470
A_23_P106661	5.7504535	5.734664	NM_052999	chr16:66612972-66613031	CMTM1
A_23_P120316	11.54124	10.537747	NM_006636	chr2:74441985-74442044	MTHFD2
A_24_P383076	5.8251686	6.2244177	NM_181806	chr4:57215519-57215460	AASDH
A_23_P112311	7.4416747	7.518872	NM_012210	chr9:119462472-119462531	TRIM32
A_23_P156708	4.8516836	4.7616863	NM_032470	chr6:32009022-32008964	TNXB
A_23_P142950	9.170874	9.086022	NM_015575	chr2:233723189-233723248	GIGYF2
A_33_P3315263	3.337761	2.9194846	NM_175834	chr12:53215380-53215321	KRT79
A_23_P344421	6.823765	4.5863733	NM_019055	chr11:124754315-124754256	ROBO4
A_23_P306987	4.1633596	3.16999	NM_031439	chr8:10582106-10582047	SOX7
A_33_P3854217	6.1838484	6.611165	NM_024874	chr1:35900305-35900246	KIAA0319L
A_23_P163992	5.0267715	5.436636	NM_005310	chr17:37902381-37902440	GRB7
A_23_P12363	9.93817	10.308033	NM_005012	chr1:64644540-64644599	ROR1
A_33_P3272165	9.545284	9.386511	NM_152416	chr8:96070070-96070129	NDUFAF6
A_23_P330561	9.536938	10.654071	NM_174918	chr19:7744452-7744511	C19orf59
A_24_P108738	6.0185823	5.976105	NM_153334	chr22:20780247-20780188	SCARF2
A_23_P102731	10.733592	10.623363	NM_175839	chr20:4168300-4168359	SMOX
A_24_P268893	7.354264	7.3281727	NM_144721	chr4:76455000-76455059	THAP6
A_33_P3381827	6.0388064	6.0051546	NM_001278649	chr20:60854299-60854358	OSBPL2
A_33_P3434927	4.6594286	4.110121	AF521131	chr7:078944864-078944808	MAGI2-IT1
A_23_P100074	8.927507	8.927876	NM_020371	chr15:34158739-34158680	AVEN
A_23_P339098	9.511988	9.177886	NM_017515	chr11:107661964-107661905	SLC35F2
A_23_P113634	10.08733	10.089412	NM_001755	chr16:67134630-67134689	CBFB
A_23_P35645	8.243035	8.0630865	NM_032905	chr10:6158615-6158674	RBM17
A_24_P282237	5.6676865	5.4042773	NM_000947	chr6:57190782-57190841	PRIM2
A_33_P3250394	7.3362503	7.3236094		chr11:003635894-003635953	
A_24_P364236	6.3174806	5.4399986	NM_004549	chr11:77779494-77779435	NDUFC2
A_24_P712562	4.3619223	4.5646634	NM_001085430	chr17:54869564-54869505	C17orf67
A_32_P74955	6.8751817	6.881298	NM_152641	chr12:46301413-46301472	ARID2
A_23_P49220	7.521411	7.4870644	NM_014972	chr16:89972605-89972664	TCF25
A_24_P586390	6.2811785	6.35105	NR_028327	chr1:665140-665081	LOC100133331
A_23_P165840	10.591017	10.309349	NM_002539	chr2:10580847-10580788	ODC1
A_33_P3441639	4.760958	4.374796	AK056793	chr15:69689302-69689243	LOC145694
A_23_P34915	4.210499	2.3900566	NM_001040619	chr1:212793856-212793915	ATF3
A_23_P324538	3.768331	3.6725578	NM_178433	chr1:152586425-152586484	LCE3B
A_23_P122924	6.1984425	5.0856247	NM_002192	chr7:41729305-41729246	INHBA
A_24_P366122	4.498152	4.429224	NM_024722	chr17:43213872-43213931	ACBD4
A_23_P121396	6.388009	6.508811	NM_145261	chr3:180702067-180702008	DNAJC19
A_23_P110851	6.056216	5.6305547	NM_198253	chr5:1253364-1253305	TERT
A_24_P883629	5.170369	5.466263	NM_024093	chr2:105964250-105964309	C2orf49
A_23_P11995	12.7016535	12.735723	NM_002574	chr1:45980589-45980295	PRDX1
A_24_P409661	10.715145	10.680315		chr1:078557627-078557688	
A_33_P3238148	4.5306187	4.071977	XM_005250341	chr7:91808677-91808618	LRRD1
A_23_P109677	12.565779	11.977153		chr3:060676328-060676269	
A_33_P3420695	5.6081324	5.736352		chrY:008492853-008492794	
A_23_P218317	8.66456	9.327567	NM_001038618	chr17:80443420-80443479	NARF
A_33_P3242919	12.651925	12.573992	NM_005968	chr19:8553826-8553885	HNRNPM
A_23_P122144	4.462675	4.8153305	NM_152404	chr5:35954141-35954082	UGT3A1

A_23_P321855	7.299123	7.4384117	NM_003899	chr13:111957595-111957654	ARHGEF7
A_33_P3662000	4.745923	3.837684	AK056246	chr2:237968136-237968077	
A_23_P103433	5.9279723	6.316764	NM_145047	chr1:36883674-36883615	OSCP1
A_23_P51996	9.644272	9.6847515	NM_007269	chr1:109351984-109352043	STXBP3
A_23_P117225	7.6568384	7.9814034	NM_000123	chr13:103527950-103528009	ERCC5
A_33_P3379962	13.605386	13.437158	NM_002116	chr6:29913492-29913551	HLA-A
A_24_P289404	11.816893	11.576559	NM_001029	chr12:56437160-56437219	RPS26
A_33_P3298062	7.0059104	6.5360694	NM_001023587	chr3:183701626-183701567	ABCC5
A_33_P3409277	7.1750975	7.0647035	XR_243508	chr16:88588489-88588430	
A_23_P34018	14.704337	14.72209	NM_001000	chrX:118920630-118920571	RPL39
A_23_P70328	8.530284	8.250126	NM_018132	chr6:49460399-49460458	CENPQ
A_24_P358164	13.583082	13.705366	NM_002295	chr3:39452431-39452489	RPSA
A_23_P134614	7.162099	6.803239	NM_006348	chr7:106843982-106843923	COG5
A_32_P396186	6.539602	6.030257	NM_014818	chr11:8633658-8633599	TRIM66
A_32_P54137	13.253873	13.298884	NM_006004	chr1:46775954-46782248	UQCRH
A_33_P3416347	5.1087103	5.124682	M27390	chr7:142353832-142353891	
A_23_P58489	8.613163	8.603679	NM_024091	chr5:7861310-7859642	FASTKD3
A_33_P3215128	6.019103	5.3335114	NM_001010867	chr1:228369854-228369913	IBA57
A_23_P252928	3.4172914	3.3544486	NM_005367	chrX:151899971-151899912	MAGEA12
A_23_P142022	7.409822	7.248019	NM_015629	chr19:54629959-54631473	PRPF31
A_23_P87879	2.3221061	2.8387508	NM_001781	chr12:9905320-9905261	CD69
A_23_P167595	6.0534463	6.065847	NM_003337	chr5:133726638-133726697	UBE2B
A_23_P57137	8.182419	7.937991	NM_018347	chr20:3805889-3805948	AP5S1
A_33_P3326733	9.322466	9.274622	XR_171057	chr10:30653375-30653316	MTPAP
A_23_P52311	6.518583	7.0205083	NM_006951	chr10:105147370-105147429	TAF5
A_33_P3318459	9.061487	9.02963	NM_003352	chr2:203070969-203070910	SUMO1
A_33_P3332215	4.3470526	4.7532444	NM_001044392	chr1:155162034-155161975	MUC1
A_33_P3354646	5.1066284	5.0636454	NM_006229	chr10:118351377-118351436	PNLIPRP1
A_23_P212968	7.194082	7.2278385	NM_001073	chr4:70074162-70074103	UGT2B11
A_23_P360626	7.659268	7.0895123	NM_178836	chr17:17104468-17104409	PLD6
A_33_P3240931	7.9995184	8.122895	NM_001048249	chr5:60453607-60453548	SMIM15
A_23_P101342	5.436636	5.0933695	NM_032885	chr19:10663725-10663784	ATG4D
A_33_P3564409	8.641719	8.412813	AK094541	chrX:51933603-51933662	
A_24_P256243	4.0861855	4.3413615	NM_175698	chrX:52787003-52787062	SSX2
A_32_P32391	6.133232	5.744159		chr13:64316583-64316642	OR7E156P
A_33_P3347055	9.880028	10.038288		chr18:044663478-044663537	
A_33_P3407266	4.881018	4.9333577	NM_012204	chr9:135553366-135553425	GTF3C4
A_23_P115366	9.325415	9.566481	NM_016308	chr1:47843918-47843977	CMPK1
A_23_P58132	12.128654	12.539214	NM_004310	chr4:40245659-40245718	RHOH
A_23_P121082	9.043909	8.946409	NM_000158	chr3:81538971-81538912	GBE1
A_23_P141447	2.3221061	2.3900566	NM_001034836	chr17:34249654-34249595	RDM1
A_33_P3782469	6.4985676	5.6449814	AL833463	chr15:55831224-55831165	PYGO1
A_24_P296808	2.3221061	2.3900566	NM_018215	chr19:46969838-46969779	PNMAL1
A_23_P34835	13.8081455	13.630709	NM_005572	chr1:156107471-156107530	LMNA
A_33_P3227375	3.2837646	3.475859	NM_003247	chr6:169615960-169615901	THBS2
A_32_P222695	4.03692	2.3900566	NM_001001669	chr5:149014156-149014215	ARHGEF37
A_23_P102842	9.070186	9.13384	NM_021100	chr20:34257106-34257047	NFS1
A_23_P138849	7.132637	6.6320534	NM_006388	chr11:65486362-65486421	KAT5
A_33_P3297642	8.89352	8.683501	NM_022063	chr10:120070356-120070297	FAM204A
A_23_P122531	11.196339	11.160891	NM_001040437	chr6:31807322-31807381	C6orf48
A_24_P925040	6.766923	7.0059104	NM_001233	chr7:116148261-116148320	CAV2
A_24_P197284	5.917979	5.4799333	NM_173821	chr2:242815327-242815386	CXXC11
A_33_P3416376	4.687558	4.2482047	BU633092	chr19:51162656-51162715	
A_33_P3619171	10.504837	9.524509	NM_021127	chr18:57570691-57570750	PMAIP1
A_33_P3391429	6.2027583	6.1911983	NM_024836	chr1:249143236-249143295	ZNF672
A_23_P341527	6.884735	6.435534	NM_152494	chr1:155015973-155018415	DCST1
A_23_P111929	5.525871	5.225788	NM_016023	chr8:92090690-92090749	OTUD6B
A_23_P72853	7.6911316	7.959303	NM_032889	chr12:53647679-53647738	MFSD5
A_33_P3312030	8.343532	8.256505		chr4:158558989-158559048	

A_33_P3408762	11.627833	11.332406	NM_001282625	chr1:156106099-156106158	LMNA
A_33_P3364289	5.764965	5.8236294	NM_032855	chr19:16268351-16268410	HSH2D
A_33_P3221980	2.3221061	2.3900566		chr7:136623056-136622997	
A_23_P417148	7.9252763	7.8504543	NM_030665	chr17:17714187-17714246	RAI1
A_23_P306845	6.0009828	5.800666	NM_152762	chr11:65715525-65715584	TSGA10IP
A_23_P105276	7.953441	8.161015	NM_003428	chr12:133635754-133635813	ZNF84
A_23_P101093	7.7236543	7.674554	NM_016429	chr17:46105838-46103783	COPZ2
A_33_P3253733	4.5917215	4.459644	XR_247077	chr1:322787-322845	
A_32_P12994	5.400723	5.82648	NR_034128	chr2:114764618-114764677	LOC440900
A_23_P115922	10.715368	11.393358	NM_004096	chr10:72183525-72183584	EIF4EBP2
A_33_P3371333	4.451642	4.395827	NM_177987	chr10:93730-93671	TUBB8
A_23_P110606	5.5632925	5.530565	NM_014757	chr5:179203516-179203575	MAML1
A_24_P645765	8.542141	7.3187623	NM_020782	chr12:27955429-27955488	KLHL42
A_23_P114929	11.639368	11.950979	NM_015415	chr1:167889288-167889229	MPC2
A_23_P113613	8.447488	7.9602785	NM_022842	chr3:45123898-45123839	CDCP1
A_24_P64362	6.446855	6.5069947	NM_017787	chr10:104575444-104575503	WBP1L
A_23_P382188	5.9347463	5.9015374	NM_001013841	chr19:4325469-4325410	STAP2
A_23_P10291	4.672311	4.771925	NM_001910	chr1:206332029-206332088	CTSE
A_33_P3382560	14.73961	14.704337	NM_000984	chr17:27050625-27050910	RPL23A
A_23_P218597	6.714448	6.737535	NM_002518	chr2:101612199-101612258	NPAS2
A_23_P204016	6.7733545	6.861025	NM_000725	chr12:49222321-49222380	CACNB3
A_23_P115636	5.2392454	4.8475747	NM_004412	chr10:17196716-17195617	TRDMT1
A_33_P3275826	10.577028	10.9200325		chr11:000627479-000627420	
A_23_P126197	11.205332	11.214775	NM_005626	chr1:29474558-29474499	SRSF4
A_23_P109322	4.625274	4.5629344	NM_006198	chr21:41301047-41301106	PCP4
A_23_P109593	6.128632	6.0774746	NM_014346	chr22:47507418-47507477	TBC1D22A
A_32_P129950	7.061103	7.5317597	NM_001012754	chr13:39623818-39623877	NHLRC3
A_33_P3233784	6.130227	6.0744286	NM_001001663	chr22:25331267-25331208	TMEM211
A_23_P30024	8.489635	8.727526	NM_003998	chr4:103537856-103537915	NFKB1
A_33_P3220475	6.2584343	6.471947	NR_026900	chr1:180169795-180169854	FLJ23867
A_23_P101351	3.8658178	3.7767878	NM_024106	chr19:9639038-9638979	ZNF426
A_23_P212339	7.0356708	7.1108794	NM_024513	chr3:45959650-45959591	FYCO1
A_33_P3239884	4.1330185	4.502633	NM_004540	chr21:22910417-22910476	NCAM2
A_23_P62709	4.7434745	5.0457115	NM_005416	chr1:152976230-152976289	SPRR3
A_23_P55731	8.287058	8.219979	NM_015125	chr19:42799560-42799619	CIC
A_33_P3276207	6.776064	5.7039576	NM_001134456	chr3:101542152-101542211	NXPE3
A_24_P177353	8.065882	8.180781	NM_001081550	chrX:122734545-122734486	THOC2
A_23_P12343	2.3412583	2.3900566	NM_000849	chr1:110279673-110279614	GSTM3
A_23_P115885	7.6337376	7.825731	NM_004897	chr10:89311995-89312054	MINPP1
A_24_P345846	3.8374093	2.3900566	NM_058172	chr4:80954642-80952804	ANTXR2
A_23_P12272	9.788723	9.509762	NM_138558	chr1:28177929-28177988	PPP1R8
A_23_P302134	6.161351	5.757711	NR_024279	chr1:16161218-16161159	FLJ37453
A_24_P225635	5.3110394	4.466801	XM_005264641	chr2:38789997-38789938	HNRNPLL
A_24_P375683	13.376355	13.277287		chr3:121213021-121213081	
A_23_P115785	4.4672856	4.786661	NM_145235	chr10:127697791-127697956	FANK1
A_32_P10396	8.474821	8.840009	NM_014991	chr4:85591282-85591223	WDFY3
A_23_P114947	8.065376	8.824706	NM_002923	chr1:192781051-192781110	RGS2
A_33_P3279847	3.965826	3.6352131	NM_139165	chr6:150210634-150210575	RAET1E
A_24_P206344	9.589754	9.336777	NM_152557	chr7:149170045-149169986	ZNF746
A_23_P218096	8.750832	8.863794	NM_022118	chr13:79896558-79896499	RBM26
A_33_P3228751	4.010154	4.197734	NR_049748	chr15:85748229-85748170	LOC642423
A_23_P136986	6.4710026	6.091945	NM_198450	chrX:84342680-84342739	APOOL
A_23_P110184	6.866215	7.246371	NM_006745	chr4:166263190-166263249	MSMO1
A_33_P3388016	11.553421	11.696018	NM_015933	chr22:040360768-040360826	TMA7
A_32_P108826	7.2390666	6.8854923	NM_194314	chr1:197123395-197123336	ZBTB41
A_24_P280868	8.717062	8.773839	NM_001137610	chr8:12283380-12283321	FAM86B2
A_23_P123330	16.000206	15.878998	NM_000989	chr8:99054999-99054940	RPL30
A_23_P368101	9.306962	9.359292	NM_024632	chr5:153832984-153833043	SAP30L
A_23_P133058	7.051199	7.2884007	NM_203462	chr4:6709854-6709795	MRFAP1L1

A_23_P114221	11.60569	11.621399	NM_002893	chrX:16863069-16863010	RBBP7
A_24_P125881	11.397626	11.441093	NM_005137	chr22:19023859-19023801	DGCR2
A_24_P286935	5.780335	5.1972733	NM_004311	chr10:104433706-104433647	ARL3
A_33_P3336242	4.319883	3.762084	NM_001256869	chr8:11990099-11990040	USP17L7
A_23_P83045	12.994773	12.856339	NM_007126	chr9:35057026-35056967	VCP
A_23_P119857	6.9923267	6.828021	NM_001008237	chr2:20097656-20096855	TTC32
A_23_P314145	5.2533555	5.1987886	NM_005715	chr6:149397569-149397628	UST
A_33_P3354881	4.9482856	4.98754	XR_108874	chr8:103876780-103876839	LOC100996457
A_33_P3285391	5.5076356	5.54313	AK025047	chr3:113308673-113308732	
A_23_P200443	11.030645	11.4105015	NM_003029	chr1:154935140-154935081	SHC1
A_33_P3215948	5.2018805	5.136725	NM_005797	chr11:118124256-118124197	MPZL2
A_23_P148969	9.191185	9.037247	NM_017768	chr1:70610619-70610560	LRRC40
A_32_P194264	7.268324	6.906359	NM_001008708	chr2:54001454-54001513	CHAC2
A_33_P3370424	6.800048	7.078028	NM_017617	chr9:139390582-139390523	NOTCH1
A_23_P100883	9.910976	10.00566	NM_015355	chr17:30327536-30327595	SUZ12
A_32_P120895	8.642183	8.636275	NM_153374	chr15:52015651-52015592	LYSMD2
A_33_P3267543	3.7525246	3.6734934	BC048256	chr9:130698694-130698635	DPM2
A_23_P120254	6.442684	6.6938696	NM_020185	chr6:351033-351092	DUSP22
A_33_P3285945	4.3303995	3.5296898	NM_002989	chr9:34709062-34709003	CCL21
A_23_P105313	9.359698	9.2895565	NM_001414	chr12:124105818-124105759	EIF2B1
A_24_P510377	2.9175076	2.3900566	BC031250	chr1:44569746-44569805	
A_23_P252062	7.2200007	7.712204	NM_138711	chr3:12458499-12458558	PPARG
A_33_P3369336	5.549855	5.4140253	NM_002054	chr2:163008728-163008669	GCG
A_24_P547010	12.850865	12.778569		chr2:139036482-139036541	
A_23_P121898	6.1104774	6.439843	NM_024615	chr5:50129844-50130785	PARP8
A_33_P3246733	5.152401	5.0764017	NM_020310	chr17:2289691-2289632	MNT
A_23_P112113	10.650059	10.703346	NM_014846	chr8:126036867-126036808	KIAA0196
A_23_P98183	8.408279	8.218501	NM_005343	chr11:533812-533600	HRAS
A_33_P3246418	9.600181	9.249154	NM_005586	chr6:41621911-41621970	MDFI
A_33_P3378707	9.384888	8.607582		chr15:21932782-21932723	LOC646214
A_33_P3419098	4.3413615	4.042767	NM_201380	chr8:145001685-145001626	PLEC
A_24_P69095	7.312767	7.0854564	NM_003633	chr5:73924314-73924255	ENC1
A_23_P166716	9.396374	9.024059	NM_017819	chr3:101284983-101285042	TRMT10C
A_33_P3242014	9.099537	9.04056	NM_004426	chr12:9093957-9094016	PHC1
A_23_P113393	4.5773926	4.6662717	NM_017413	chrX:128779470-128779411	APLN
A_33_P3340025	10.406868	10.181066	NM_021067	chr20:25429111-25429170	GINS1
A_23_P112412	11.129656	10.952884	NM_017746	chr9:103064570-103064511	TEX10
A_24_P154948	10.492203	10.455351	NM_002047	chr7:30665891-30668190	GARS
A_23_P11237	4.318839	4.5760636	NM_004606	chrX:70680545-70680604	TAF1
A_23_P15654	6.6719956	6.4937983	NM_004295	chr17:27076538-27076597	TRAF4
A_23_P100730	3.582073	3.0941544	NM_003726	chr17:46210889-46210830	SKAP1
A_23_P201086	7.74363	7.7949553	NM_001024227	chr1:228285947-228286006	ARF1
A_23_P10785	6.786807	6.70068	NM_145206	chr10:114298077-114428023	VTI1A
A_24_P211709	4.193317	3.5278368	NM_003399	chrX:128902573-128902632	XPNPEP2
A_23_P101707	9.704746	9.810196	NM_005817	chr19:4839174-4839115	PLIN3
A_33_P3411741	5.823571	5.9930053	NM_001040455	chr11:117063914-117063973	SIDT2
A_23_P100441	7.3321133	7.109371	NM_024946	chr16:57186520-57186461	FAM192A
A_32_P6832	8.032389	8.324331	NM_175921	chr5:41921429-41921488	C5orf51
A_33_P3323041	5.741361	5.663086	AL832454	chr9:733619-733678	KANK1
A_23_P101319	4.489909	4.984305	NM_152354	chr19:44890444-44890385	ZNF285
A_23_P306890	8.485591	8.684528	NM_007195	chr18:51820933-51820992	POLI
A_23_P43049	8.815816	9.006567	NM_006571	chr8:30038065-30038124	DCTN6
A_23_P23765	7.288043	7.194082	NM_014288	chr1:63920569-63920094	ITGB3BP
A_23_P23850	5.3740897	5.8727236	NM_021080	chr1:57480698-57480639	DAB1
A_23_P254801	6.0344667	6.032581	NM_002660	chr20:39803549-39803609	PLCG1
A_33_P3280066	16.214172	16.13095	NM_012232	chr17:40555884-40555825	PTRF
A_33_P3746549	7.4134245	6.5907516	NM_052909	chr5:182635-182694	PLEKHG4B
A_23_P252653	8.436866	8.455063	NM_006374	chr2:242435021-242434962	STK25
A_23_P113462	6.926665	7.350527	NM_017641	chr12:39687030-39687018	KIF21A

A_24_P220485	7.143962	7.5347767	NM_182487	chr9:127577020-127577079	OLFML2A
A_23_P110288	5.126916	5.504531	NM_024751	chr4:106744140-106744199	GSTCD
A_23_P142421	6.7349663	6.5886345	NM_014727	chr19:36229074-36229223	KMT2B
A_23_P103775	6.8415112	7.0946627	NM_032270	chr1:90180324-90180383	LRRC8C
A_33_P3278303	8.354865	8.3460865		chr21:039618944-039618885	
A_24_P266285	4.2598367	3.9879653	NM_152558	chr7:2649695-2649754	IQCE
A_23_P54041	6.923934	7.0051365	NM_024328	chr14:24028493-24028552	THTPA
A_23_P69537	9.583407	9.300844	NM_006681	chr4:56461505-56461446	NMU
A_23_P250122	10.781036	10.634556	NM_020223	chr7:300381-300440	FAM20C
A_24_P316489	6.0293546	6.3279767	NM_004544	chr2:240961604-240960786	NDUFA10
A_23_P393034	9.487399	9.843386	NM_005329	chr16:69151369-69151428	HAS3
A_23_P11862	9.097275	8.908999	NM_018186	chr1:169822110-169822169	C1orf112
A_24_P246963	5.4933805	5.2519298		chr12:053126329-053126388	
A_23_P104054	9.8006935	9.430839	NM_016227	chr1:172580756-172580815	SUCO
A_33_P3346573	6.114746	5.7388554	NM_001134364	chr3:47910775-47910716	MAP4
A_33_P3387646	3.2502813	3.5005348		chr5:175602897-175602838	LOC643201
A_33_P3334384	5.431155	5.4335537	NR_033827	chr1:44168387-44168328	KDM4A-AS1
A_33_P3400823	6.3376627	5.665078	NM_001013615	chr1:46686803-46686862	LURAP1
A_23_P346405	7.1520195	7.41978	NM_138357	chr10:74646758-74646817	MCU
A_23_P106145	9.538067	9.7539425	NM_014584	chr14:53113136-53112990	ERO1L
A_23_P105619	6.356994	6.024378	NM_138341	chr12:112369549-112369490	TMEM116
A_33_P3221708	11.223735	11.3123255		chr6:4703963-4704022	XLOC_014512
A_23_P117558	11.007809	10.637595	NM_002013	chr14:45587328-45587269	FKBP3
A_23_P123848	6.713484	6.457532	NM_032552	chr9:124547348-124547407	DAB2IP
A_33_P3345812	5.879078	6.013812	NM_001039966	chr7:1132661-1132720	GPER1
A_23_P100292	11.041611	10.620188	NM_178167	chr16:2047756-2047697	ZNF598
A_23_P52826	8.629563	8.583399	NM_032725	chr11:116618997-116618938	BUD13
A_23_P150064	4.071977	4.3160615	NM_024756	chr10:88696220-88696161	MMRN2
A_23_P212458	9.601952	9.498814	NM_013336	chr3:127789960-127790019	SEC61A1
A_33_P3231156	8.526061	8.712591		chr16:056682743-056682802	
A_33_P3418942	8.584695	9.013121	NM_007006	chr16:56481803-56481744	NUDT21
A_23_P201918	9.70311	9.649359	NM_012089	chr1:229652770-229652711	ABCB10
A_24_P15821	13.117857	12.518466		chr11:057485866-057485805	
A_33_P3391905	2.3221061	2.8707676	NR_002784	chrX:027478385-027478326	SMEK3P
A_23_P212002	9.504021	9.615696	NM_005385	chr3:42689920-42689979	NKTR
A_33_P3293760	6.5875645	6.778498	NM_198695	chr21:46032417-46032476	KRTAP10-8
A_33_P3324765	8.464094	8.72152	NM_001893	chr17:80206811-80206752	CSNK1D
A_23_P258418	5.425033	5.362341	NM_024309	chr4:2746193-2746134	TNIP2
A_23_P103503	6.9302087	6.9413056	NM_002393	chr1:204518623-204518682	MDM4
A_32_P106732	8.767604	8.64519	NM_020937	chr14:45669210-45669269	FANCM
A_24_P406830	8.301263	8.003413	NM_201278	chr11:95566186-95566127	MTMR2
A_24_P351466	4.5707297	4.706768	NM_020890	chr3:108269504-108269445	KIAA1524
A_33_P3266928	5.916657	5.930913	NM_001080434	chr19:48988597-48988538	LMTK3
A_33_P3274084	9.539421	9.624451	NM_138328	chr17:30651534-30651593	RHBDL3
A_23_P81880	10.355734	10.7189455	NM_005730	chr12:58213870-58213811	CTDSP2
A_23_P136817	7.045635	7.3169575	NM_014708	chr12:123106487-123107037	KNTC1
A_23_P10385	9.265461	8.377263	NM_016448	chr1:212277852-212277911	DTL
A_24_P98555	6.088536	6.3418107	NM_207009	chr10:120877058-120877117	FAM45A
A_23_P64689	5.7546277	5.589144	NM_014871	chr12:56711143-56711084	PAN2
A_23_P106835	8.983759	9.195328	NM_031885	chr16:56518692-56518633	BBS2
A_32_P93036	6.16293	6.159211	CU678501	chr19:045411956-045411897	
A_24_P195794	14.224985	14.257444	AK130930	chr17:45236838-45236779	
A_33_P3466016	7.819448	7.8994527	AK054953	chr3:160983747-160983806	LOC200830
A_23_P25019	8.438028	8.166409	NM_000946	chr12:57133111-57132284	PRIM1
A_24_P280113	9.65705	9.564299	NM_001560	chrX:117928406-117928465	IL13RA1
A_24_P50818	5.2163076	4.5524106	NM_001206774	chr2:197645020-197644961	GTF3C3
A_24_P288779	3.2033176	3.0007355	NM_130775	chrX:52842182-52842241	XAGE5
A_33_P3361584	6.2473807	5.8295336	AK126579	chr19:4449885-4449944	LOC100130930
A_24_P29277	7.8205647	7.8285284	NM_001130105	chr5:74667084-74667025	COL4A3BP

A_23_P373568	5.471758	5.355454	NM_025129	chr19:50310363-50310304	FUZ
A_23_P213255	9.071903	9.18846	NM_020159	chr4:95211718-95211777	SMARCAD1
A_24_P73290	10.097739	9.795473	NM_001681	chr12:110788669-110788728	ATP2A2
A_23_P29939	7.4610853	7.3297386	NM_007308	chr4:90647744-90647685	SNCA
A_23_P391164	5.43948	5.723182	NM_032434	chr2:27845367-27845426	ZNF512
A_23_P121141	4.4775715	4.91091	NM_014065	chr3:130742901-130737554	ASTE1
A_24_P120346	11.198201	11.135401	NM_020202	chr3:100074086-100074145	NIT2
A_23_P77669	6.122469	6.1175823	NM_017530	chr16:71893808-71893749	ZNF821
A_23_P33613	9.88269	9.595903	NM_024096	chr16:30435368-30435309	DCTPP1
A_33_P3378659	7.0476274	7.2930284	NM_001003799	chr7:38299809-38299750	TARP
A_24_P263910	4.5832376	3.2553728	AK090420	chr14:50466711-50466652	C14orf182
A_23_P121527	7.19527	7.908369	NM_015990	chr4:39123524-39123583	KLHL5
A_33_P3474319	4.374522	3.2821105	NM_198277	chr11:124959065-124959124	SLC37A2
A_33_P3381429	16.355698	16.30209		chr1:198918760-198918819	
A_24_P84508	14.393929	14.506384		chr9:006639362-006639423	
A_23_P36700	5.358402	5.23232	NM_018009	chr12:6566840-6566899	TAPBPL
A_24_P264166	4.694538	4.958637	NM_001282468	chr15:28946770-28946711	GOLGA8M
A_23_P100499	4.722272	5.0746336	NM_024589	chr16:4850504-4849729	ROGDI
A_24_P245246	11.320145	11.286869	NM_003559	chr17:36922033-36921974	PIP4K2B
A_23_P56736	6.6407485	6.514957	NM_080386	chr2:132240430-132240489	TUBA3D
A_24_P159648	6.324201	6.2657986	NM_006340	chr17:79078484-79079905	BAIAP2
A_24_P945283	5.8676815	5.4567165	NM_021120	chrX:69725254-69725313	DLG3
A_23_P204550	9.642089	9.446329	NM_017988	chr12:100733740-100733799	SCYL2
A_33_P3802146	12.983658	12.820715	AK131313	chr10:42755760-42755701	
A_33_P3246010	8.41609	8.624057	AK092494	chr16:88775474-88775415	
A_33_P3241511	3.9732277	3.8927033	NM_000185	chr22:21141453-21141512	SERPIND1
A_24_P291426	7.75659	7.887694	NM_016316	chr2:100019124-100018768	REV1
A_23_P210131	7.376853	7.2229443		chr2:96657375-96657316	LOC100996862
A_23_P104109	7.677295	8.183846	NM_012424	chr1:213446224-213446283	RPS6KC1
A_33_P3242099	9.24477	8.844548	NM_203459	chr1:200822485-200822544	CAMSAP2
A_23_P113572	4.176116	4.647652	NM_001770	chr16:28950600-28950659	CD19
A_23_P117662	4.765423	4.7162085	NM_002112	chr15:50534229-50534170	HDC
A_23_P12113	7.774537	7.7115064	NM_014053	chr1:213068636-213068695	FLVCR1
A_33_P3228593	5.6242156	5.9193153	AK023344	chrX:48934649-48934590	WDR45
A_23_P55190	11.716484	11.575249	NM_004247	chr17:42928387-42928328	EFTUD2
A_23_P354798	7.190092	7.1793327	NM_144576	chr12:56664424-56664483	COQ10A
A_23_P117782	9.339848	8.969382	NM_018357	chr15:71123971-71123912	LARP6
A_23_P90233	5.537571	5.8130875	NM_024303	chr19:56732987-56732928	ZSCAN5A
A_23_P50907	10.922192	10.975435	NM_002210	chr2:187545295-187545354	ITGAV
A_33_P3374589	5.217824	5.3560715		chr22:046189559-046189618	
A_23_P99632	7.640828	7.796222	NM_017999	chr14:24629168-24629505	RNF31
A_33_P3282000	3.4493027	3.3856578	XR_108434	chr2:160473700-160473759	LOC643072
A_23_P112531	10.651667	10.606349	NM_001035254	chr9:130703275-130703216	FAM102A
A_23_P106481	10.016502	10.106634	NM_016454	chr15:34520664-34520723	EMC4
A_24_P53282	5.8716455	6.3844805	NM_001304	chr17:28795047-28795106	CPD
A_23_P115726	4.0237575	3.97397	NM_194298	chr10:61411130-61411071	SLC16A9
A_23_P153480	4.2730947	4.238456	NM_012427	chr19:51446925-51446866	KLK5
A_23_P101960	11.404576	11.517992	NM_006887	chr2:43451215-43451156	ZFP36L2
A_23_P209694	7.402326	7.596458	NM_022894	chr2:61025238-61025297	PAPOLG
A_23_P110062	7.4828787	7.420541	NM_003907	chr3:183861961-183862392	EIF2B5
A_23_P97283	6.9017806	6.886508	NM_024897	chr1:156213417-156213358	PAQR6
A_23_P112596	9.682512	9.540537	NM_001123	chr10:76429970-76468080	ADK
A_33_P3687604	4.075752	3.8925238	AK093315	chr2:207118334-207118393	LOC285173
A_24_P481783	6.036212	5.203382	NM_001163692	chr15:65385408-65385349	UBAP1L
A_33_P3294881	8.775371	8.78706	NM_016474	chr3:14712642-14712701	CCDC174
A_24_P343233	6.0041647	6.1134305	NM_002124	chr6:32548583-32548524	HLA-DRB1
A_33_P3252196	9.26936	9.365152	NM_004456	chr7:148504564-148504505	EZH2
A_23_P107744	5.243499	5.3763742	NM_030760	chr19:10623943-10623884	S1PR5
A_23_P115919	8.483959	8.354865	NM_001037537	chr10:13320342-13320283	PHYH

A_23_P145024	6.426716	5.4681196	NM_000024	chr5:148208080-148208139	ADRB2
A_23_P122304	11.6586	11.3728485	NM_001527	chr6:114262873-114262195	HDAC2
A_33_P3406245	7.8886166	7.496139	NM_005681	chr1:222742915-222742856	TAF1A
A_23_P93258	7.6876945	7.520683	NM_003537	chr6:26031981-26031922	HIST1H3B
A_23_P11685	6.760496	6.450508	NM_024420	chr1:186957652-186957711	PLA2G4A
A_33_P3325009	15.537646	15.546938	BC000737	chr12:101818474-101818533	RGS4
A_23_P122615	7.5088835	7.817448	NM_032870	chr6:99848620-99848561	PNISR
A_23_P49539	7.038975	7.000249	NM_001080519	chr17:79432934-79432993	BAHCC1
A_33_P3315504	7.9646444	7.826234	NM_153332	chr8:8890645-8890704	ERI1
A_23_P322845	7.518872	7.412111	NM_001102559	chr8:38120995-38120936	PPAPDC1B
A_24_P103264	6.518406	7.066556	NM_003360	chr4:115597201-115597260	UGT8
A_23_P213863	6.2681413	6.593206	NM_001190946	chr5:176949061-176949002	FAM193B
A_23_P94275	4.215438	3.757731	NM_014420	chr8:42232341-42232282	DKK4
A_23_P121676	2.9770162	3.1641748	NM_025212	chr4:105393495-105393436	CXXC4
A_33_P3303982	4.329755	4.413885	NM_001143975	chr11:89819626-89819685	UBTFL1
A_23_P121196	8.98567	9.15336	NM_024334	chr3:14184283-14184342	TMEM43
A_23_P414713	2.3221061	2.3900566	NM_032423	chr19:52921177-52921236	ZNF528
A_23_P115482	10.974328	10.819522	NM_014176	chr1:202300965-202300906	UBE2T
A_23_P8497	10.111533	10.822683	NM_000823	chr7:31015475-31016094	GHRHR
A_23_P138881	6.0226874	5.68952	NM_001104	chr11:66329598-66329745	ACTN3
A_33_P3233070	3.744021	4.2905917	XR_158888	chr16:11035738-11035797	LOC100505564
A_33_P3319580	4.0787253	4.2722654	AK097459	chr7:539803-539862	
A_33_P3362453	4.2905917	3.9619133		chrX:051665138-051665079	
A_23_P216630	9.51847	9.5077715	NM_080546	chr9:108153508-108153567	SLC44A1
A_23_P11372	10.516293	10.695933	NM_000194	chrX:133634514-133634573	HPRT1
A_23_P388433	9.513282	10.234175	NM_001170330	chr4:120218473-120218414	C4orf3
A_23_P133923	9.115098	8.923636	NM_033177	chr6:31629940-31629881	GPANK1
A_23_P23966	4.9576054	5.204214	NM_153034	chr10:48373505-48373564	ZNF488
A_23_P122563	8.58181	8.317878	NM_014260	chr6:33258190-33258514	PFDN6
A_23_P206901	10.097204	10.045073	NM_017668	chr16:15818834-15818893	NDE1
A_23_P102965	7.9694653	8.083874	NM_015367	chr22:18211363-18211422	BCL2L13
A_23_P122775	7.79753	7.850127	NM_032730	chr6:107031286-107031227	RTN4IP1
A_23_P12173	10.130947	10.066513	NM_181715	chr1:153920247-153920188	CRTC2
A_33_P3348924	3.0759823	2.877716	AK128128	chr16:33346963-33346904	
A_24_P177604	5.002389	4.713369	NM_033215	chrX:49143802-49143861	PPP1R3F
A_24_P171268	7.7710648	7.907981	NM_182663	chr1:206762349-206762408	RASSF5
A_23_P105747	6.035947	6.0953755	NM_018171	chr12:105568002-105567943	APPL2
A_33_P3393801	4.5157804	4.4875374	NM_005764	chr1:47650734-47650675	PDZK1IP1
A_23_P124003	4.821108	4.8234577	NM_170683	chr12:133198203-133198262	P2RX2
A_23_P100455	5.833468	5.647027	NM_022764	chr16:86564700-86564641	MTHFSD
A_24_P42501	8.162231	8.1227	NM_001037171	chrX:23740101-23740042	ACOT9
A_32_P162192	2.3221061	2.3900566	NR_027457	chr14:106950219-106950278	LINC00221
A_23_P205046	8.433685	8.759153	NM_017664	chr13:111531510-111531451	ANKRD10
A_23_P420417	6.7954903	6.8179593	NM_138463	chr17:27051715-27051656	TLCD1
A_23_P104318	10.369637	10.882939	NM_019058	chr10:74035363-74035422	DDIT4
A_24_P40529	4.772234	4.435755	NM_018196	chrX:154720085-154720026	TMLHE
A_23_P206532	9.600967	9.880028	NM_001031835	chr16:47733400-47733459	PHKB
A_23_P115215	8.679623	8.644885	NM_005997	chr1:151149155-151149096	VPS72
A_23_P372096	5.7295656	5.325279	AK097804	chr20:31052830-31052771	C20orf112
A_33_P3217238	10.498325	10.214254	NM_014109	chr8:124332837-124332778	ATAD2
A_23_P106056	12.009898	12.189296	NM_001344	chr14:23034008-23033949	DAD1
A_23_P120062	7.704926	7.684237	NM_003203	chr2:75891606-75891547	GCFC2
A_23_P323094	5.682889	5.9839773	NM_004426	chr12:9092293-9092352	PHC1
A_23_P104651	10.810476	10.644176	NM_080668	chr11:64845055-64844996	CDCA5
A_32_P91250	9.470165	9.530449	NM_003347	chr22:21947172-21947231	UBE2L3
A_33_P3404824	6.115056	6.1564646	NM_001172831	chr5:150275045-150274986	ZNF300
A_33_P3419180	7.9636755	7.9044094	NR_039982	chr14:39307190-39307131	LINC00639
A_23_P17330	6.4891043	6.4102483	NM_017859	chr20:62576002-62575943	UCKL1
A_33_P3329644	7.7989483	7.979163	NM_001136152	chr3:129817087-129817146	ALG1L2

A_33_P3274439	4.0070157	2.8725853		chr11:104439552-104439493	
A_23_P136635	7.900839	8.386878	NM_003664	chr5:77298511-77298452	AP3B1
A_32_P177955	4.5703783	4.640927	NR_038853	chr9:102648745-102648686	LOC441461
A_23_P56798	10.007291	9.952119	NM_004300	chr2:277899-277958	ACP1
A_23_P97584	10.648223	10.495584	NM_014597	chr1:94337636-94336298	DNTTIP2
A_23_P111672	5.775161	5.5471926	NM_152829	chr7:115898128-115898187	TES
A_23_P117157	8.864941	8.910944	NM_003850	chr13:48517447-48517388	SUCLA2
A_23_P135769	13.459215	13.565643	NM_001101	chr7:5567240-5567181	ACTB
A_23_P101992	3.7684977	4.265373	NM_006770	chr2:119751977-119752036	MARCO
A_33_P3412125	12.857992	12.866091		chr19:021416316-021416375	
A_23_P152272	9.349108	9.128883	NM_006711	chr16:2303753-2303694	RNPS1
A_23_P15414	6.6894445	6.540509	NM_003693	chr17:1537812-1537753	SCARF1
A_24_P175783	4.7264695	5.154352	NM_015313	chr11:120359849-120359908	ARHGEF12
A_23_P340922	3.9476178	4.5168962	NM_032370	chr19:8577309-8576824	ZNF414
A_24_P230176	10.719231	10.414299	NM_199287	chr17:79640236-79640295	CCDC137
A_23_P113283	5.958472	6.615861	NM_022470	chr3:178742220-178742161	ZMAT3
A_23_P354027	5.4586587	5.8964834	NM_001002914	chr17:7257934-7257993	KCTD11
A_23_P158277	6.5031614	6.248504	NM_181719	chr1:20009254-20009195	TMCO4
A_33_P3831566	5.033678	4.7847314	AF072164	chr9:91769268-91769209	
A_23_P35168	6.8502045	7.2777843	NM_013339	chr1:63902525-63902584	ALG6
A_23_P75083	5.814952	6.0886364	NM_212479	chr10:294888-294947	ZMYND11
A_23_P161004	4.5891266	5.125531	NM_205860	chr1:200017524-200017583	NR5A2
A_23_P411296	10.7528	10.728892	NM_005194	chr20:48808989-48809048	CEBPB
A_23_P429581	6.3122206	7.0266795	NM_153704	chr8:94822080-94827556	TMEM67
A_23_P354791	2.3221061	2.3900566	NM_182964	chr11:20124939-20125241	NAV2
A_24_P87490	7.344114	7.6876945	NM_145301	chr17:15449189-15449130	TVP23C
A_23_P45087	7.803683	7.938961	NM_016220	chr7:64171177-64171236	ZNF107
A_23_P142294	9.326906	9.395388	NM_014297	chr19:44010989-44010930	ETHE1
A_33_P3237948	3.9118087	3.6008744	NM_001008536	chr1:152058198-152058139	TCHHL1
A_23_P130429	6.441343	6.560277	NM_005406	chr18:18535191-18535132	ROCK1
A_33_P3345931	4.3702726	3.4467793	XM_001127575	chr12:52241701-52241760	LOC728503
A_23_P120973	4.786576	4.7580843	NM_017911	chr22:45728525-45728584	FAM118A
A_33_P3254121	6.327351	6.8273478	AK001769	chr6:167347487-167347428	RNASET2
A_32_P117313	7.684828	7.0005636	NM_001171796	chr8:93895961-93895902	TRIQK
A_24_P920188	6.1253614	6.505995	NM_006965	chr18:32912748-32912689	ZNF24
A_23_P372638	7.746727	7.717627	NM_025134	chr16:53191437-53243436	CHD9
A_23_P111481	11.67376	11.583111	NM_015908	chr7:100486219-100486278	SRRT
A_23_P10442	7.0382633	5.983557	NM_080597	chr18:21742185-21742126	OSBPL1A
A_23_P108376	6.391721	6.203273	NM_022065	chr2:43459873-43458445	THADA
A_23_P1691	5.30378	5.0570655	NM_002421	chr11:102660756-102660697	MMP1
A_33_P3416814	4.0426054	4.0202365	XM_005265917	chr5:180237808-180237749	MGAT1
A_23_P384761	3.9641287	3.310227	NM_002052	chr8:11617080-11617139	GATA4
A_23_P329198	8.936651	8.934701	NM_001031716	chr2:192551555-192551614	NABP1
A_33_P3260322	7.3893185	7.499661	NM_033334	chr9:127279618-127279559	NR6A1
A_23_P23584	10.312432	10.292791	NM_020248	chr1:9908447-9908388	CTNNBIP1
A_23_P30634	2.3221061	2.8559165	NM_021813	chr6:90636664-90636605	BACH2
A_23_P11915	5.0605516	5.053573	NM_017686	chr1:118412260-118412201	GDAP2
A_23_P117494	10.800127	10.649348	NM_005956	chr14:64926496-64926555	MTHFD1
A_33_P3405491	4.0699472	3.8242173	AY465895	chr6:42327093-42327152	HCRP1
A_23_P151544	5.3632784	5.5878344	NM_020414	chr14:94526639-94526580	DDX24
A_24_P350649	7.693034	7.930737	NM_017990	chr16:70194784-70194843	PDPR
A_23_P54929	7.157105	7.4085526	NM_020424	chr16:20935567-20935626	LYRM1
A_33_P3244369	4.948641	4.9587545	BX646214	chr9:42494551-42494610	
A_23_P80902	8.5473795	8.703567	NM_020242	chr3:44894386-44894445	KIF15
A_33_P3251984	4.5629344	4.5648403	NM_001098515	chr11:68773595-68773536	MRGPRF
A_33_P3236340	8.286297	8.771827	AF289593	chr4:6693935-6693876	
A_23_P119254	8.432004	8.48196	NM_018154	chr19:14230697-14230638	ASF1B
A_23_P108564	2.6717112	3.1030393	NM_020981	chr2:168726358-168726417	B3GALT1
A_23_P148475	7.211272	7.5499377	NM_012310	chrX:69640147-69640206	KIF4A

A_32_P135902	10.241947	10.212454	NM_001416	chr17:7480389-7480448	EIF4A1
A_33_P3298661	6.2221794	6.8088727	NM_021964	chr3:124952272-124952213	ZNF148
A_23_P96331	4.706068	5.3836856	NM_000377	chrX:48549526-48549585	WAS
A_23_P112825	8.788225	8.825663	NM_016309	chr16:25182080-25186270	LCMT1
A_23_P383132	7.788469	8.070118	NM_015094	chr22:21805554-21805613	HIC2
A_23_P149259	5.870351	5.7149043	NM_032323	chr1:156261873-156261932	TMEM79
A_23_P117797	6.4567103	6.309174	NM_017882	chr15:68500008-68499949	CLN6
A_23_P114282	10.824588	10.472368	NM_014060	chrX:119746090-119746149	MCTS1
A_33_P3361817	7.39334	7.506898		chr1:024228141-024228082	
A_23_P80382	7.6381545	7.845008	NM_015366	chr22:45133267-45133326	PRR5
A_33_P3328609	6.0978947	6.6492853	NM_003628	chr2:159537064-159537124	PKP4
A_23_P394836	6.282742	6.2955265	NM_022489	chr14:105185512-105185589	INF2
A_32_P122940	6.1701875	6.432891		chr21:46716334-46716393	LOC642852
A_23_P119923	7.001111	7.0144687	NM_020184	chr2:97477481-97477540	CNNM4
A_33_P3234764	4.888313	4.453865	AK130920	chr1:210799982-210800041	
A_23_P156531	11.920317	12.048147	NM_002793	chr6:170852761-170852702	PSMB1
A_23_P106241	8.552269	8.6177025	NM_004239	chr14:92435951-92435892	TRIP11
A_23_P102582	11.818749	11.571212	NM_018840	chr20:35240817-35240876	C20orf24
A_24_P227585	6.8806157	6.835864	NM_018559	chr13:45589665-45589724	GPALPP1
A_33_P3247534	6.9986625	6.977308	NR_027420	chr21:9917195-9917136	LOC389834
A_24_P180680	12.766243	13.229299	NM_018407	chr8:98864581-98864640	LAPTM4B
A_33_P3280927	9.963184	9.238232	NR_024542	chr9:139619106-139619047	SNHG7
A_23_P76034	5.356965	5.202171	NM_203286	chr11:119547906-119547847	PVRL1
A_23_P316472	5.08128	4.7785625	NM_144666	chr11:6593108-6593167	DNHD1
A_24_P940135	6.565402	6.3246264	NM_004388	chr1:85019462-85019403	CTBS
A_33_P3374623	5.1701274	4.256274	NM_019112	chr19:1065272-1065331	ABCA7
A_23_P212545	9.251625	9.549081	NM_004162	chr3:20026192-20026251	RAB5A
A_33_P3337019	4.76834	5.2343316	XR_247237	chr11:64947580-64947521	LOC728975
A_23_P41424	10.558995	10.538397	NM_022154	chr4:103183010-103182951	SLC39A8
A_24_P940776	7.877159	7.485192	NM_018429	chr5:70862873-70862932	BDP1
A_24_P626981	2.3221061	2.8609424	NR_002949	chr3:131083311-131083370	NUDT16P1
A_24_P204675	11.668272	11.750135	NM_001135865	chr16:22545395-22545455	NPIPB5
A_33_P3382309	4.459644	4.511349	NM_199454	chr1:3350316-3350375	PRDM16
A_33_P3253234	9.2499075	9.142192	NM_001243197	chrX:53295566-53295507	IQSEC2
A_33_P3252588	5.5773745	5.3052173	NR_024485	chr1:227921673-227921732	LOC100130093
A_23_P136238	9.517648	9.740978	NM_053045	chr9:140098995-140098936	TMEM203
A_33_P3278684	5.6067386	5.2450695		chr1:201869056-201869115	
A_23_P111804	5.9809566	5.6227865	NM_022750	chr7:139724005-139723946	PARP12
A_23_P112201	5.9613824	6.096854	NM_015061	chr9:7175334-7175393	KDM4C
A_23_P118306	10.001595	9.609197	NM_005147	chr16:4506392-4506451	DNAJA3
A_23_P56213	4.4940767	4.1248775	NM_020895	chr19:35512470-35512614	GRAMD1A
A_24_P221092	6.1151395	5.8686275	AF289615	chr13:99189066-99189007	
A_33_P3273125	3.4652135	4.203796	NR_026810	chr17:16692558-16692616	FAM106CP
A_23_P116414	8.498264	8.358639	NM_007069	chr11:63342381-63342322	PLA2G16
A_24_P941148	8.79413	8.29318	NM_017645	chr9:19053295-19053236	HAUS6
A_24_P379693	5.6535378	5.6150594	NM_012100	chr2:220238798-220238739	DNPEP
A_33_P3280549	9.000854	9.334223		chr1:155815540-155815599	
A_23_P60101	6.4220247	5.7761087	NM_030895	chr8:144379467-144379526	ZNF696
A_33_P3303430	5.8562565	5.7905636		chr2:203705169-203705110	
A_23_P14174	4.3865385	4.9205503	NM_006573	chr13:108922693-108922752	TNFSF13B
A_23_P218841	9.67777	9.681814	NM_018297	chr3:25760904-25760845	NGLY1
A_33_P3330283	9.688896	9.582101	NM_138473	chr12:53810152-53810211	SP1
A_23_P12053	7.8743854	7.9048724	NM_015001	chr1:16266686-16266745	SPEN
A_33_P3313555	8.406145	8.374377	NM_001042414	chr13:20315765-20315706	PSPC1
A_23_P100196	10.373914	10.366682	NM_005153	chr16:84812831-84812890	USP10
A_33_P3646051	3.785473	3.6532593	AK094324	chr19:37294755-37294814	ZNF790-AS1
A_23_P6546	5.310084	5.428817	NM_020461	chr22:50656772-50656713	TUBGCP6
A_23_P115137	10.929781	10.962393	NM_006402	chr1:110944166-110944107	LAMTOR5
A_33_P3349774	5.009262	4.8908396	NM_144703	chr20:60699744-60699803	LSM14B

A_23_P37068	6.8177214	6.923565	NM_145725	chr14:103372362-103372421	TRAF3
A_23_P118061	9.310001	9.331588	NM_181641	chr16:66592129-66592188	CKLF
A_33_P3213204	7.75548	7.237857	NM_022149	chr3:184428985-184428926	MAGEF1
A_23_P129433	6.703924	6.4590287	NM_004594	chr16:67306015-67306074	SLC9A5
A_23_P157495	7.485192	7.19527	NM_005605	chr8:22398566-22398625	PPP3CC
A_23_P119337	4.7785625	4.847023	NM_012068	chr19:50436754-50436813	ATF5
A_33_P3400374	9.45542	10.01072	NM_001037335	chr20:62189535-62189476	HELZ2
A_24_P256307	9.907475	10.021875	NM_001659	chr12:49330310-49330251	ARF3
A_23_P423864	11.860668	11.823778	NM_198040	chr1:33789459-33789400	PHC2
A_32_P139229	4.31654	4.4858804	NM_213598	chr19:57841743-57841802	ZNF543
A_23_P76705	5.5441704	5.613639	NM_006322	chr13:113158954-113158433	TUBGCP3
A_23_P132121	6.5772815	6.628251	NM_173354	chr21:44834819-44834760	SIK1
A_23_P120921	6.66422	6.5740767	NM_138797	chr22:38227345-38227286	ANKRD54
A_23_P424734	4.9190016	4.518847	NM_178516	chr16:67218599-67218459	EXOC3L1
A_24_P135406	7.154489	7.1421585	NM_017634	chr8:25290129-25290070	KCTD9
A_23_P253068	4.5109634	4.2184744	NM_013366	chr9:140074667-140070265	ANAPC2
A_33_P3224426	5.018037	5.0892935		chr11:134605634-134605693	LOC729305
A_23_P26810	5.23658	5.024614	NM_000546	chr17:7572691-7572632	TP53
A_24_P204214	8.1438465	8.380993	NM_006311	chr17:16068436-16068377	NCOR1
A_23_P122197	12.472273	12.659221	NM_031966	chr5:68471363-68473123	CCNB1
A_23_P324327	7.176006	7.9433155	NM_016235	chr16:19870688-19870629	GPRC5B
A_23_P168868	12.182161	12.075822	NM_014754	chr8:97346598-97346657	PTDSS1
A_23_P170978	8.846014	8.698742	NM_017865	chr1:249144393-249144334	ZNF692
A_23_P152838	2.3221061	2.3900566	NM_002985	chr17:34199391-34199332	CCL5
A_24_P681011	6.584602	6.8111806	NM_022740	chr7:139246970-139246911	HIPK2
A_33_P3383955	7.7599173	7.9636755	NM_000107	chr11:47238539-47238598	DDB2
A_24_P128312	6.441928	6.8513355	NM_007135	chr9:130207248-130207307	ZNF79
A_33_P3244872	8.979932	9.167845	NM_014810	chr1:180053181-180053240	CEP350
A_23_P96542	8.882624	8.882624	NM_001017980	chrX:150573488-150573547	VMA21
A_24_P343377	10.490299	10.572796	NM_001003714	chr7:99057746-99055957	ATP5J2
A_33_P3396522	11.240308	11.199257	NM_002696	chr11:62534122-62534181	POLR2G
A_23_P121956	7.3509283	7.4760213	NM_017872	chr5:157166422-157166481	THG1L
A_32_P182156	10.257715	10.062071	NM_145806	chr10:135125327-135126332	ZNF511
A_23_P110234	3.4740367	3.3835855	NM_001890	chr4:70810602-70810661	CSN1S1
A_23_P329361	6.56385	6.6556435	NM_153603	chr16:23400358-23400299	COG7
A_24_P32887	8.978973	8.917227	NM_007371	chr9:136901234-136901175	BRD3
A_33_P3269408	13.179645	13.017186	NM_207426	chr10:129539033-129539092	FOXI2
A_23_P101737	4.454238	4.84148	NM_032482	chr19:2232170-2232229	DOT1L
A_23_P99515	6.3332767	6.1253614	NM_032849	chr13:31499299-31499358	MEDAG
A_23_P167040	9.3222065	9.410728	NM_006810	chr3:122880268-122880751	PDIA5
A_24_P306720	5.8453474	5.971603	NR_036500	chr14:89885621-89885680	FOXN3-AS1
A_23_P2041	5.3979297	4.274914	NM_032867	chr11:12380556-12380615	MICALCL
A_23_P58102	7.1000266	7.091585	NM_015004	chr3:45038715-45043059	EXOSC7
A_33_P3288294	5.164337	5.3602123	AJ535838	chr8:33246513-33246454	FUT10
A_33_P3310430	8.802715	8.899624	NM_001137610	chr8:12283457-12283398	FAM86B2
A_23_P325155	4.0281677	4.0448995	NM_138939	chr3:112648223-112648164	CD200R1
A_23_P761	13.7511425	13.792873	NM_002796	chr1:151374330-151374389	PSMB4
A_23_P110212	6.464305	6.6331954	NM_001995	chr4:185676745-185676732	ACSL1
A_23_P117599	8.763117	8.779316	NM_012111	chr14:77928985-77929044	AHSA1
A_33_P3212823	15.2344475	15.340809	NM_001135865	chr16:22547334-22547393	NPIPB5
A_23_P161399	7.6015034	8.208789	NM_130439	chr10:112045945-112046001	MXI1
A_23_P104362	11.603579	11.828948	NM_004092	chr10:135176314-135176255	ECHS1
A_23_P374288	9.030766	9.071903	NM_007359	chr17:38328012-38328071	CASC3
A_23_P11214	7.619623	7.5121803	NM_017544	chrX:118722626-118722567	NKRF
A_33_P3286499	4.16282	3.566854		chrX:152160822-152160763	
A_23_P113382	4.6838646	4.8104987	NM_021150	chr12:66742192-66742133	GRIP1
A_23_P87560	6.7287927	7.2605147	NM_001731	chr12:92537607-92537548	BTG1
A_24_P135193	5.193813	5.1054034	NM_018314	chr11:18586542-18586483	UEVLD
A_23_P25994	6.042195	6.6001396	NM_001008530	chr14:93178016-93176171	LGMN

A_23_P104734	9.911865	10.079368	NM_152713	chr11:125489999-125490703	STT3A
A_23_P206759	5.5418854	6.0021954	NM_014761	chr16:71961764-71961823	IST1
A_33_P3684897	3.950736	3.8951437	DA730659	chr7:97937254-97937195	FLJ30064
A_23_P68486	7.5170097	8.143698	NM_080821	chr20:54943040-54943099	FAM210B
A_33_P3336282	3.8973174	3.5442605	NM_005640	chr18:23873435-23873494	TAF4B
A_33_P3358686	3.5975356	3.4404993		chr17:041999971-041999912	
A_23_P117873	3.9644418	3.4387443	NM_012125	chr15:34356428-34356487	CHRM5
A_23_P123265	5.386597	5.6281633	NM_015411	chr7:56147679-56147738	SUMF2
A_33_P3282202	8.154556	7.64608	AK124041	chr5:170014065-170014124	
A_23_P40194	9.574403	9.348744	NM_017895	chr20:47860532-47860591	DDX27
A_23_P106708	16.487724	16.538507	NM_002952	chr16:2012137-2012081	RPS2
A_33_P3233546	4.2434	4.2259293		chr7:005534179-005534120	
A_33_P3331267	4.460213	5.3723493		chr6:109315720-109315661	
A_33_P3345608	4.6772003	4.451642	NM_001202438	chr10:127429706-127429765	C10orf137
A_24_P58727	12.269806	11.652713		chr15:076039248-076039308	
A_23_P361419	8.595882	8.405464	NM_018369	chr5:59893300-59893241	DEPDC1B
A_23_P364613	7.4090033	8.026605	NM_138413	chr10:99372173-99372232	HOGA1
A_32_P738377	4.4195404	4.7907248	NR_026961	chr21:45226433-45226374	LOC284837
A_23_P154256	7.2052517	7.05053	NM_032213	chr2:85618691-85618750	ELMOD3
A_24_P23522	5.233269	5.083472	NM_182529	chr7:108204772-108204713	THAP5
A_33_P3246997	4.815822	4.5356646	BC056409	chr17:48814662-48814721	LUC7L3
A_33_P3385909	4.240522	3.9287295	NM_001243467	chr21:43829657-43829716	UBASH3A
A_33_P3277259	7.0147424	7.1472325	NM_001282203	chr17:79860851-79860792	PCYT2
A_23_P52266	9.495037	9.440264	NM_001548	chr10:91163438-91163497	IFIT1
A_33_P3285047	7.826402	7.6497602	NM_153028	chr16:3368494-3368553	ZNF75A
A_24_P195037	7.905998	8.155939	NM_198077	chr1:85724296-85724237	C1orf52
A_23_P77440	4.562775	5.004822	NM_173165	chr16:68225293-68225352	NFATC3
A_24_P140827	10.405651	10.318715	NM_005389	chr6:150131752-150131811	PCMT1
A_23_P323783	5.0690627	5.3122234	NM_144703	chr20:60709375-60709434	LSM14B
A_23_P101392	4.862002	5.0896406	NM_024074	chr19:16799430-16799489	TMEM38A
A_33_P3286218	7.84158	7.6063466	NR_002771	chr1:64014962-64015021	DLEU2L
A_33_P3221999	5.6759176	5.5751123	NM_001165958	chr17:38060909-38060850	GSDMB
A_33_P3313929	10.536964	10.611544	NM_031409	chr6:167552098-167552157	CCR6
A_33_P3390456	5.4976926	5.4947095	NM_001190979	chr12:42604410-42604351	YAF2
A_23_P75707	3.5417771	4.1737785	NM_001005199	chr11:56057842-56057783	OR8H1
A_33_P3355407	8.175696	8.089315	NR_004404	chr1:28835114-28835173	RNU105A
A_23_P90079	6.360353	6.7042174	NM_001080821	chr19:12501716-12501657	ZNF799
A_33_P3267745	3.8333662	4.278188	NM_001256526	chr9:139009978-139009919	C9orf69
A_23_P115597	6.394235	6.5285797	NM_014915	chr10:27302014-27301955	ANKRD26
A_33_P3374365	6.2403355	6.206378		chrX:102788564-102788505	
A_23_P109774	8.407054	8.074771	NM_014415	chr3:101368633-101368574	ZBTB11
A_32_P128391	3.8967292	3.5901437		chr1:37920866-37920807	LOC728431
A_24_P399980	5.209718	4.961007	NM_014799	chrX:65486328-65486387	HEPH
A_23_P12405	3.976498	3.6494646	NM_031475	chr1:6520696-6520755	ESPN
A_23_P118246	10.091444	10.081409	NM_016095	chr16:85711713-85711654	GINS2
A_23_P101461	9.286822	9.332237	NM_030818	chr19:13874029-13874088	CCDC130
A_23_P103897	4.7994328	4.842098	XM_005271374	chr1:85009909-85009968	
A_23_P11160	6.0081553	6.0098352	NM_024597	chrX:135299676-135299617	MAP7D3
A_24_P926760	7.710331	7.8226504	NM_005385	chr3:42672047-42672701	NKTR
A_23_P86283	9.150382	9.1358595	NM_006762	chr1:31205902-31205843	LAPTM5
A_33_P3356752	4.235714	4.2791834	NM_001039966	chr7:1132386-1132445	GPER1
A_33_P3242713	6.1184897	6.3955464	AK022058	chr18:8015115-8015174	
A_33_P3210069	3.9730635	3.5336924		chr16:033573750-033573809	
A_23_P31143	8.3961315	7.497409	NM_001003395	chr6:125584445-125584504	TPD52L1
A_33_P3376026	3.904319	4.384908	NR_047572	chr16:3086692-3086751	LOC100128770
A_23_P1145	8.7806	8.806864	NM_018109	chr10:30602535-30602476	MTPAP
A_23_P103476	8.662113	8.248489	NM_013319	chr1:11346237-11346296	UBIAD1
A_33_P3385431	3.7548242	3.589046		chr14:039839168-039839109	
A_23_P402287	7.0353875	7.104932	NM_153371	chr13:28120442-28120383	LNX2

A_33_P3354256	4.4871597	3.8789263	NM_001282971	chr8:125565128-125565069	MTSS1
A_23_P112061	8.724403	9.014381	NM_152419	chr8:43056897-43056956	HGSNAT
A_33_P3389634	12.0726595	12.222317	NM_001747	chr2:85622075-85622016	CAPG
A_23_P162874	13.717503	13.182049	NM_005348	chr14:102549512-102549453	HSP90AA1
A_23_P205531	5.0768304	5.637397	NM_001282192	chr14:21167969-21168028	RNASE4
A_32_P141612	4.9837446	5.0522685	NM_002552	chr2:148691827-148691768	ORC4
A_24_P11506	5.73197	5.741361	NM_001032998	chr2:143715219-143715278	KYNU
A_24_P541919	7.46159	8.074628	NM_144973	chr12:31535725-31535666	DENND5B
A_23_P148541	2.3221061	2.3900566	NM_139250	chrX:153815008-153815067	CTAG1A
A_32_P97169	4.9884644	5.008374	NM_005708	chr13:95059334-95059393	GPC6
A_23_P107513	6.498874	6.9283323	NM_024899	chr18:12674543-12673452	CEP76
A_33_P3355296	5.677155	5.7007732	XM_005247043	chr2:242688306-242688365	D2HGDH
A_23_P216845	4.4215746	5.2632036	NM_004188	chr9:135866653-135866712	GFI1B
A_23_P79518	2.372614	2.9296362	NM_000576	chr2:113587488-113587429	IL1B
A_23_P118749	8.906865	9.031422	NM_016041	chr17:5377972-5377913	DERL2
A_23_P53267	9.499698	9.416941	NM_023012	chr12:122989804-122989745	RSRC2
A_23_P123596	4.839961	4.777589	NM_000170	chr9:6532808-6532749	GLDC
A_33_P3405531	6.340761	6.2327185	NM_007114	chr3:69069159-69069100	TMF1
A_33_P3406072	2.6070008	3.1039	NM_001244959	chr9:85863178-85863119	FRMD3
A_23_P17471	4.354182	5.1547446	NM_022760	chr20:2819526-2819370	PCED1A
A_23_P82941	9.113756	9.235094	NM_006421	chr8:68110253-68110194	ARFGEF1
A_33_P3340847	4.8570404	4.803994	NM_032587	chr5:40843751-40843810	CARD6
A_33_P3358347	4.339543	4.9442687		chr11:047187868-047187809	
A_24_P413126	8.7206135	7.433044	NM_020182	chr20:56223548-56223489	PMEPA1
A_33_P3344264	7.566716	7.208109	NM_020175	chr19:5785214-5785155	DUS3L
A_24_P169343	8.425108	8.457846	NM_153698	chr9:99404109-99404050	AAED1
A_32_P109922	8.455063	8.344564	NM_020863	chr8:135490097-135490038	ZFAT
A_33_P3308706	4.502633	4.4684305	NM_001282659	chr11:11901729-11901788	USP47
A_32_P167122	7.2517495	7.338168	NM_018254	chr1:211489128-211489187	RCOR3
A_23_P17393	12.117973	11.847378	NM_001316	chr20:47712950-47713009	CSE1L
A_24_P109554	4.766823	4.8355975	NM_007018	chr9:123858822-123860703	CNTRL
A_33_P3261937	5.6040454	5.1195984	NM_001012264	chr14:21501049-21500990	RNASE13
A_24_P236753	4.0477104	3.2400548	NM_003585	chr17:11929-11331	DOC2B
A_33_P3250845	10.073508	8.906865	NM_001184889	chr15:23005425-23005366	NIPA2
A_23_P110699	6.1584425	6.1338305	NM_052860	chr5:150274493-150274434	ZNF300
A_23_P112950	7.357461	7.4758234	NM_018179	chr12:14650947-14651006	ATF7IP
A_23_P104942	10.367952	10.339214	NM_015959	chr11:57508264-57508323	TMX2
A_23_P162734	10.484283	9.898451	NM_005977	chr13:26787364-26787305	RNF6
A_33_P3318627	4.078477	3.566197	AK308862	chr16:10273875-10273816	GRIN2A
A_33_P3245784	4.689187	4.4528284	NM_175888	chr3:40558702-40558761	ZNF620
A_23_P170186	7.854101	8.199578	NM_017570	chr8:145106261-145106202	OPLAH
A_33_P3303146	6.517535	6.304325	NM_032852	chr1:63329990-63330049	ATG4C
A_24_P418044	10.716174	10.843962	NR_024240	chr6:29977388-29977447	HLA-J
A_23_P82523	4.3923836	4.750773	NM_000927	chr7:87138603-87135313	ABCB1
A_23_P121806	9.779233	9.599868	NM_021204	chr4:83381838-83381897	ENOPH1
A_23_P325501	8.797267	8.668288	NM_015358	chr21:37748528-37748587	MORC3
A_33_P3297611	5.5327134	5.9112034	AI924184	chr2:119254784-119254843	
A_23_P76961	14.673007	14.656517	NM_001032	chr14:50052691-50050358	RPS29
A_23_P98085	9.699641	9.623254	NM_000314	chr10:89726025-89726084	PTEN
A_23_P117068	12.544102	12.341772	NM_003095	chr12:96255070-96259159	SNRPF
A_33_P3457052	7.119712	7.1000266		chr6:111599771-111599830	
A_23_P156327	10.114497	10.158998	NM_000358	chr5:135399343-135399402	TGFBI
A_33_P3369646	5.848875	5.954315		chr9:140446712-140446653	
A_33_P3215452	5.2398076	5.310084	NM_001271592	chr12:11325867-11325926	LOC100129361
A_33_P3334590	4.0870833	4.7313056	NM_017762	chr15:31260183-31260124	MTMR10
A_24_P333019	5.985116	6.3718143	NM_007219	chr20:3914467-3914408	RNF24
A_23_P92895	8.77371	8.487568	NM_018047	chr5:150070867-150070808	RBM22
A_23_P256021	10.253034	10.034353	NM_031206	chrX:64732577-64732518	LAS1L
A_23_P75299	6.097716	6.182459	NM_022126	chr10:126302618-126302677	LHPP

A_23_P502930	11.283609	11.4189825	NM_006712	chr7:150773779-150773720	FASTK
A_23_P123172	4.9992476	5.0414834	NR_002157	chr7:143997420-143997479	OR2A9P
A_23_P106389	4.137886	3.38095	NM_003612	chr15:74702424-74702365	SEMA7A
A_23_P109171	6.862732	6.570348	NM_001195	chr20:17474629-17474570	BFSP1
A_23_P501007	9.456326	9.088555	NM_001039348	chr2:56093847-56093788	EFEMP1
A_33_P3323722	11.939297	11.863987	NM_001282431	chr2:235401768-235401709	ARL4C
A_33_P3349334	9.538986	9.252088	NM_199421	chr14:55516046-55516105	SOCS4
A_23_P500364	10.032911	9.92691	NM_001707	chr7:72950825-72950766	BCL7B
A_24_P316305	8.083874	7.5357122	AK001173	chr15:35147853-35147794	AQR
A_33_P3365408	5.3588276	5.3090734	NR_026947	chr12:7271213-7271272	C1RL-AS1
A_23_P360209	11.525513	11.778658	HV963894	chrM:10200-10259	ND3
A_23_P404685	10.496923	10.887427	NM_178348	chr1:152800026-152800085	LCE1A
A_23_P100420	9.331588	9.595449	NM_015144	chr16:87440191-87440132	ZCCHC14
A_24_P260443	3.2537484	3.2502813	NM_003248	chr5:79378905-79378964	THBS4
A_23_P54477	11.996162	11.893164	NM_018648	chr15:34634079-34634020	NOP10
A_23_P108244	11.39393	11.336784	NM_001863	chr19:36149509-36149568	COX6B1
A_23_P51565	6.1647863	5.552494	NM_003281	chr1:201386936-201384353	TNNI1
A_24_P341535	5.6156273	5.7863445	NR_027001	chr15:84873671-84873612	LOC388152
A_23_P111961	8.590418	8.423832	NM_032509	chr8:33358375-33358434	MAK16
A_33_P3384322	5.472887	5.73197	BX537921	chr16:8743278-8743337	LOC100132006
A_23_P23728	4.6826572	4.9159102	NM_014849	chr1:149876051-149875992	SV2A
A_23_P32029	6.0965266	6.8648624	NM_007001	chr9:99084331-99083616	SLC35D2
A_33_P3268368	8.572012	8.584695	NM_016263	chr19:3536696-3536755	FZR1
A_33_P3231162	4.170315	4.598899	NM_173632	chr19:58268715-58268774	ZNF776
A_33_P3330608	3.8894062	3.3992033	NM_032152	chr19:8555000-8554941	PRAM1
A_23_P160618	6.3620133	6.500387	NM_003975	chr1:156776243-156776184	SH2D2A
A_23_P114155	9.004953	8.876369	NM_015698	chrX:48970514-48970455	GPKOW
A_23_P167401	5.2826176	5.1205773	NM_018931	chr5:140581576-140581635	PCDHB11
A_33_P3350227	6.168247	6.0096025	NM_181491	chr9:136210661-136210602	MED22
A_33_P3341568	7.177975	7.4388566		chr13:019241753-019241812	
A_23_P31721	8.033253	7.878978	NM_001951	chr8:86126199-86126258	E2F5
A_24_P74329	6.3418107	6.5228567	NM_001076678	chr19:21580050-21587970	ZNF493
A_33_P3384932	7.55612	7.6420555	NM_001243750	chr11:67395469-67395410	NUDT8
A_32_P203066	2.3221061	2.3900566	DB090170	chrX:115827146-115827087	
A_23_P106822	8.889372	8.811218	NM_014062	chr16:69776266-69776207	NOB1
A_33_P3398897	7.609253	6.446024	NM_000320	chr4:17488089-17488030	QDPR
A_33_P3262022	5.46148	5.42905		chr12:050273146-050273205	
A_23_P156852	11.773367	11.802288	NM_206836	chr6:4116182-4116123	ECI2
A_23_P390722	4.1159716	3.755066	NM_022347	chr1:179834222-179834163	TOR1AIP2
A_33_P3282836	15.455059	15.462484	NM_001031	chr19:8387220-8387279	RPS28
A_33_P3538104	5.7859735	6.0055604	XR_243451	chr16:52314138-52314197	
A_33_P3422240	3.6725578	4.387325	NM_020894	chr4:1379688-1379747	UVSSA
A_23_P105066	8.980867	9.052461	NM_001014795	chr11:6631739-6631798	ILK
A_32_P506600	14.203674	13.962357	NM_006325	chr12:131360738-131360797	RAN
A_33_P3227944	3.8804078	3.9525404	NR_026906	chr17:43723534-43723593	CRHR1-IT1
A_33_P3839897	4.989785	5.391179	DW419002	chr2:122288790-122288849	RNU4ATAC
A_23_P117298	4.5920625	4.921529	NM_000131	chr13:113773878-113773937	F7
A_23_P10497	9.49355	9.290078	NM_198531	chr18:77137941-77138000	ATP9B
A_23_P362759	4.640093	4.9926677	NM_018699	chr4:121616308-121616249	PRDM5
A_23_P301896	7.733854	7.88196	NM_015866	chr1:14113806-14113865	PRDM2
A_32_P233938	6.06243	6.1948824	NM_001040057	chr7:92191489-92191430	FAM133B
A_24_P248606	8.941015	7.9335666	NM_004457	chr2:223807495-223807554	ACSL3
A_23_P70213	4.39859	4.8034315	NM_000038	chr5:112180822-112180881	APC
A_23_P121825	7.8682895	7.7384562	NM_024941	chr5:64961513-64961572	TRAPPC13
A_23_P252403	8.296107	8.707744	NM_016144	chr5:115628532-115628591	COMMD10
A_23_P122532	6.024997	6.283783	NM_021184	chr6:31626656-31626597	C6orf47
A_23_P31477	6.0686703	6.288436	NM_015332	chr7:44423748-44423689	NUDCD3
A_33_P3403778	10.32852	10.25834	NM_152600	chr19:56088952-56088893	ZNF579
A_23_P202837	9.874785	9.790122	NM_053056	chr11:69468233-69468292	CCND1

A_33_P3299982	11.010656	10.89695	NM_003367	chr19:35770605-35770664	USF2
A_33_P3399380	6.814863	6.617707		chr12:4208461-4208520	XLOC_014512
A_32_P168886	13.7675	13.723041	NM_022170	chr7:73611194-73611253	EIF4H
A_24_P262738	6.2567806	6.224799	NM_024050	chr19:17425175-17426778	DDA1
A_33_P3255499	11.144116	11.210442	NR_024872	chr9:37877631-37877572	SLC25A51
A_23_P120931	7.6736054	7.7793922	NM_014508	chr22:39414287-39414346	APOBEC3C
A_23_P23303	9.416334	9.443571	NM_003686	chr1:242048717-242048776	EXO1
A_23_P117387	7.1701736	7.8531237	NM_054024	chr14:39716981-39717040	MIA2
A_23_P121345	5.768587	6.05955	NM_018138	chr3:186264732-186264673	TBCCD1
A_33_P3318002	2.3376355	2.3900566	NM_031500	chr5:140189110-140189169	PCDHA4
A_24_P43092	7.4429994	8.002132	NM_001008491	chr2:242282456-242283173	SEPT2
A_33_P3292844	7.4999795	7.597224	NM_020999	chr10:71331901-71331842	NEUROG3
A_23_P142849	8.450116	8.001765	NM_005168	chr2:151325166-151325107	RND3
A_23_P102242	8.705704	8.942351	NM_022826	chr2:160623821-160623880	MARCH7
A_33_P3408643	7.0495615	6.4809527		chr2:038179970-038180029	
A_33_P3356811	7.1603737	7.4903736	NM_178353	chr1:152760119-152760178	LCE1E
A_33_P3762733	6.954383	7.178857		chr21:24733607-24733548	D21S2088E
A_33_P3209960	3.669887	3.825212	NM_153819	chr11:64508476-64508420	RASGRP2
A_33_P3364508	7.3002768	7.4683924	NM_018275	chr7:99752106-99752047	C7orf43
A_24_P12401	9.580386	9.776228	NM_001025366	chr6:43753511-43753570	VEGFA
A_33_P3355608	6.8019996	6.9165077	NM_031852	chr5:140216279-140216338	PCDHA7
A_23_P48717	10.875428	11.137152	NM_006432	chr14:74946813-74946754	NPC2
A_33_P3349414	10.314959	10.289147	NM_014007	chr9:129597442-129597501	ZBTB43
A_24_P265856	7.583913	7.5896087	NM_020654	chr3:101043356-101043297	SENP7
A_33_P3329784	6.0266027	5.9416256	NM_015114	chr12:133311069-133311010	ANKLE2
A_33_P3329793	4.632411	4.322644	NR_003228	chr16:90066550-90066609	AFG3L1P
A_23_P320897	8.120427	8.06064	NM_001195215	chr1:197479387-197479328	DENND1B
A_33_P3349546	7.3569975	6.0920196	NM_025000	chr2:172341501-172341560	DCAF17
A_33_P3399935	4.088124	3.8285866	NM_021135	chr6:166827367-166827308	RPS6KA2
A_23_P122375	6.2784443	5.9381795	NM_021943	chr6:38121738-38121797	ZFAND3
A_23_P102542	6.719896	6.613103	NM_203437	chr2:64819947-64820006	AFTPH
A_23_P7313	12.932745	13.483522	NM_001040058	chr4:88903920-88903979	SPP1
A_24_P831309	7.473709	7.6962156	NM_207401	chr1:247275358-247275299	C1orf229
A_24_P411186	2.3221061	2.3900566	NM_022893	chr2:60684496-60684437	BCL11A
A_32_P397824	4.668627	5.1163263	NM_018086	chr2:164464661-164464602	FIGN
A_23_P500861	7.739944	7.9289293	NM_182961	chr6:152443111-152443052	SYNE1
A_24_P45620	6.812739	6.497229	NM_021995	chr1:7909705-7907889	UTS2
A_23_P76435	6.716808	6.859538	NM_176818	chr12:120894917-120894976	GATC
A_33_P3241646	6.7875876	7.397587	NM_024594	chr5:167986045-167984576	PANK3
A_23_P205867	4.521297	4.7528315	NM_014249	chr15:72110325-72110384	NR2E3
A_33_P3245489	9.552821	9.807876	NM_213604	chr19:1506798-1506739	ADAMTSL5
A_33_P3234031	5.3668947	5.9450016	AK125981	chr17:28904990-28905049	
A_23_P416434	6.3540883	6.3959603	NM_015288	chr5:133918544-133918603	JADE2
A_23_P100711	8.760151	8.877874	NM_000304	chr17:15133267-15133208	PMP22
A_33_P3402611	6.2701325	6.069779	XR_247172	chr9:43066785-43066726	
A_23_P66766	7.678155	7.8651524	NM_001160246	chr17:5324688-5324747	RPAIN
A_24_P315862	5.227505	5.687112	S73508	chr14:107083393-107083334	
A_23_P35796	5.405517	5.564062	NM_006244	chr11:64701663-64701722	PPP2R5B
A_23_P381102	9.307959	9.599095	NM_207310	chr2:130897033-130896974	CCDC74B
A_33_P3574055	9.85832	9.972441	NR_036592	chr9:37087746-37087805	LOC100506710
A_33_P3357626	11.899172	12.025831		chr1:012280908-012280849	
A_23_P435657	5.1353602	4.815618	NR_003683	chr2:73899939-73899998	ALMS1P
A_23_P95923	9.148609	9.294467	NM_017656	chr19:9759781-9759722	ZNF562
A_23_P98844	4.6414585	4.905406	NM_182947	chr12:58010715-58010774	ARHGEF25
A_23_P111240	8.594679	8.690812	NM_014721	chr6:144146229-144146288	PHACTR2
A_32_P204019	13.293835	13.345445	NM_000992	chr3:52028003-52027944	RPL29
A_23_P208210	5.31962	5.7011313	NM_014650	chr19:52536848-52536789	ZNF432
A_23_P126393	9.683207	9.829681	NM_012432	chr1:150937122-150937181	SETDB1
A_33_P3412233	7.104003	6.996408	NM_152992	chr7:76254917-76254858	POMZP3

A_33_P3251342	6.0492525	5.9901137	NM_017460	chr7:99354663-99354604	CYP3A4
A_33_P3409506	7.338572	7.1181464	NM_182505	chr9:74526696-74526752	C9orf85
A_24_P74932	8.698742	8.96821	NM_002668	chrX:49029760-49029819	PLP2
A_33_P3423551	7.809699	8.061101	NM_003897	chr6:30711635-30711576	IER3
A_24_P108291	7.2282095	7.3664618	NM_018439	chr18:22033169-22033228	IMPACT
A_33_P3375368	5.949162	5.8462257	NM_001195381	chr2:241570164-241570223	GPR35
A_23_P380857	5.1357226	5.252243	NM_030643	chr22:36585942-36585883	APOL4
A_33_P3211604	11.3728485	11.281217	NM_015646	chr12:69054183-69054242	RAP1B
A_24_P133253	8.457016	7.8743854	NM_000899	chr12:88886712-88886653	KITLG
A_33_P3405168	6.727592	6.329244	NM_172250	chr4:146579784-146579843	MMAA
A_32_P88120	4.7933245	4.837842	NM_013313	chr22:22052454-22052395	YPEL1
A_33_P3598363	4.865967	4.92681	AK055411	chr18:75900753-75900812	
A_23_P80377	7.1738963	6.4614286	NM_007229	chr22:43266674-43266615	PACSIN2
A_23_P62764	6.9717846	7.2632966	NM_024296	chr1:32669575-32669634	CCDC28B
A_24_P398432	5.9907885	5.431155	NM_001040118	chr11:72397168-72397109	ARAP1
A_33_P3284552	4.2741795	4.728648	AK023303	chr19:19019818-19019759	COPE
A_23_P110243	7.5060096	7.745161	NM_020961	chr4:119631543-119631602	METTL14
A_33_P3233105	4.825173	4.744808	NM_001207008	chr6:168291553-168291612	MLLT4
A_33_P3247589	3.367062	2.3900566		chr17:35218995-35218936	
A_33_P3349496	4.3737993	3.3543055	XR_246020	chr12:94671645-94671586	LOC101928753
A_23_P125772	4.549592	4.7233834	NM_014370	chrX:153050866-153050925	SRPK3
A_23_P115703	8.804932	8.745977	NM_001011663	chr10:105063057-105062998	PCGF6
A_33_P3211054	5.1844425	5.4078474	NM_152373	chr1:41007295-41007354	ZNF684
A_33_P3371493	5.8692575	5.9655223	NM_003286	chr20:39742734-39742793	TOP1
A_23_P3065	8.193537	8.339475	NM_018168	chr14:57937011-57936952	C14orf105
A_24_P389994	4.9726243	5.1632285	NM_023018	chr1:1685079-1685020	NADK
A_23_P113184	8.29533	8.502892	NM_001080432	chr16:54148113-54148172	FTO
A_24_P100351	12.0060215	11.968481	NM_019067	chrX:54588995-54589054	GNL3L
A_23_P12874	10.804133	10.51021	NM_012341	chr10:1063404-1063463	GTPBP4
A_33_P3241582	15.031535	14.993443	NM_001019	chr16:18796069-18794375	RPS15A
A_23_P114061	3.99865	3.86843	NM_001007471	chr9:73150331-73150272	TRPM3
A_23_P100127	8.078167	8.140608	NM_170589	chr15:40917525-40917584	CASC5
A_33_P3229869	4.508518	4.860474	NR_040082	chr15:26260736-26260795	LOC100128714
A_23_P108437	6.561235	7.119169	NM_003468	chr2:208627844-208627785	FZD5
A_33_P3336632	7.302538	8.104538	NR_003287	chrUn_gl000220:117900-117959	RNA28S5
A_33_P3300152	4.789667	4.8387847	AK000041	chr14:95930166-95930107	
A_23_P217958	8.929662	8.667175	NM_033500	chr10:71161158-71161217	HK1
A_33_P3230798	5.023862	5.640936		chr9:113550154-113550213	MUSK
A_24_P320665	4.6257157	4.81012	NM_032581	chr7:22983499-22983440	FAM126A
A_33_P3259615	6.326584	6.5796337	NM_001080441	chr11:118398249-118398308	TTC36
A_24_P57170	6.4309087	6.5878897	NM_001039887	chr19:36253157-36253216	C19orf55
A_23_P109877	8.9671335	9.286822	NM_025222	chr3:52289613-52289554	WDR82
A_23_P87238	2.4874923	3.0270064	NM_006512	chr11:18254043-18253984	SAA4
A_33_P3256848	4.0365324	4.7089562	NM_021641	chr10:127731711-127731652	ADAM12
A_24_P88031	6.0297766	5.995077	NM_023080	chr8:146280237-146280296	C8orf33
A_23_P79441	6.6844144	6.85402	NM_017880	chr2:70377259-70377200	C2orf42
A_33_P3407945	9.2767515	9.173637	NM_018279	chr12:72097781-72097840	TMEM19
A_23_P58443	7.8300934	7.84141	NM_020690	chr5:139928822-139928881	ANKHD1-EIF4EBP3
A_24_P6911	7.041284	7.069086	NR_023383	chr7:76635087-76641250	DTX2P1-UPK3BP1-PMS2P11
A_23_P6802	8.285862	7.7781396	NM_004704	chr3:51967609-51967550	RRP9
A_23_P203305	6.931096	6.759354	NM_144981	chr11:31477853-31455105	IMMP1L
A_24_P84880	9.863575	10.059792		chr1:202843170-202843229	LOC148709
A_32_P167791	11.433928	11.539543	NM_003932	chr22:41220550-41220523	ST13
A_23_P102508	6.006038	6.1000805	NM_021095	chr2:27423111-27423052	SLC5A6
A_33_P3254695	6.5022697	6.352267	NR_004404	chr1:28835168-28835227	RNU105A

A_33_P3261438	3.6521225	3.2867758		chr15:072120048-072120107	
A_33_P3421530	4.2972684	3.509339	XM_003960940	chr9:133393884-133393943	LOC101060175
A_23_P111381	10.108512	10.138596	NM_004849	chr6:106633925-106633866	ATG5
A_33_P3378545	4.4706063	4.7457805	DB092099	chr14:80078042-80077983	
A_23_P18413	9.00023	9.002588	NM_016589	chr3:119242693-119242752	TIMMDC1
A_32_P142818	4.6846404	4.1108255	NM_178120	chr2:172953991-172954050	DLX1
A_33_P3294986	7.406079	7.3968296	NM_005357	chr19:42905725-42905666	LIPE
A_32_P26330	7.8565383	7.6788087	NM_005153	chr16:84778252-84778310	USP10
A_33_P3364582	8.927876	9.171151	NM_019105	chr6:32010593-32010534	TNXB
A_33_P3247988	5.2935114	5.7076764	NM_017573	chr19:1481487-1481428	PCSK4
A_33_P3254996	5.4821444	5.807773	NR_026719	chr21:38592834-38592893	DSCR9
A_23_P119943	4.975405	5.174006	NM_000597	chr2:217529086-217529145	IGFBP2
A_23_P149813	5.2554526	5.6692467	NM_012425	chr10:16633585-16633526	RSU1
A_23_P101871	2.5433073	2.8314295	NM_024907	chr19:39432700-39432641	FBXO17
A_33_P3397865	11.236134	11.276537	NM_003283	chr19:55644255-55644196	TNNT1
A_23_P27709	5.015725	5.327399	NM_033378	chr19:49535289-49535348	CGB2
A_24_P158065	8.921387	8.761313	NM_001040057	chr7:92190661-92190602	FAM133B
A_33_P3214943	8.106397	8.498657	NM_001134434	chr10:73846187-73846128	SPOCK2
A_23_P10121	4.1661677	3.8417912	NM_003012	chr8:41120220-41120161	SFRP1
A_33_P3410981	3.5252228	4.125794	NM_032101	chr5:140799732-140799791	PCDHGB7
A_23_P500873	5.500589	5.3241606	NM_133629	chr17:33427686-33427627	RAD51D
A_23_P169003	7.137463	7.434521	NM_022071	chr8:19252242-19252301	SH2D4A
A_23_P142634	10.979118	10.4301605	NM_014168	chr2:170672009-170668981	METTL5
A_33_P3364989	4.685571	5.2633934	NM_001024675	chr20:32256272-32256331	ACTL10
A_24_P415601	8.198608	8.115564	NM_002939	chr11:498093-498034	RNH1
A_23_P250564	10.762769	10.53638	NM_005400	chr2:46414736-46414795	PRKCE
A_33_P3386508	4.717788	4.92521	NR_034118	chr22:42520497-42520556	NDUFA6-AS1
A_23_P327426	11.139745	11.061897	NM_152902	chr1:168169147-168169206	TIPRL
A_33_P3254666	5.483293	5.9391475	AK097997	chr10:102764939-102764998	LZTS2
A_33_P3324298	4.8559103	5.5345783		chr6:167871558-167871499	
A_23_P501634	8.842437	8.698269	NM_078476	chr6:26469772-26469831	BTN2A1
A_24_P315444	5.965232	6.004886		chr17:016520831-016520769	
A_23_P209962	7.8187895	7.783066	NM_024624	chr2:17851726-17847762	SMC6
A_23_P259632	8.009302	8.342077	NM_031925	chr7:75616562-75616503	TMEM120A
A_23_P10815	8.905253	9.256479	NM_001020658	chr1:31404653-31404594	PUM1
A_24_P299318	11.061897	11.099016	NM_182705	chr17:289939-289880	FAM101B
A_23_P123672	9.085695	8.87285	NM_014290	chr9:100258236-100258295	TDRD7
A_33_P3407195	9.330807	9.184313	NM_148894	chr4:13612618-13612559	BOD1L1
A_24_P400997	7.3896704	7.1557355	NM_015295	chr18:2673319-2674029	SMCHD1
A_33_P3354176	6.234325	5.924569		chr10:95185557-95185498	MYOF
A_23_P102890	11.709219	11.609968	NM_032476	chr21:35515227-35515286	MRPS6
A_24_P175187	4.1456714	3.893358	NM_017654	chr7:92729841-92729782	SAMD9
A_23_P48414	5.004606	5.62109	NM_003914	chr13:37014154-37014213	CCNA1
A_33_P3419790	3.0234725	4.1966934	AK123258	chr6:11717597-11717538	ADTRP
A_33_P3309924	5.9002466	4.9356937	NM_001527	chr6:114257403-114257344	HDAC2
A_33_P3328292	4.82407	4.9868636	NM_002657	chr20:30784257-30784198	PLAGL2
A_23_P123308	4.126315	3.7552881	NM_031271	chr8:30689830-30689771	TEX15
A_33_P3223467	8.457683	7.7702584	NM_025054	chr8:67542631-67542572	VCPIP1
A_23_P40059	7.8341594	7.978067	NM_000534	chr2:190732610-190738235	PMS1
A_33_P3296852	16.640553	16.640553	AK000470	chrX:3810701-3810642	
A_33_P3357748	11.396209	11.292916		chr8:129113404-129113463	PVT1
A_33_P3277238	3.8581793	4.273923	XM_005269387	chr10:74292845-74292786	MICU1
A_23_P209195	6.920917	7.2321596	NM_031448	chr19:30192602-30192543	C19orf12
A_23_P216501	13.176956	13.322228	NM_213674	chr9:35682147-35682088	TPM2
A_33_P3267305	11.506347	11.850737	NM_173595	chr12:56631650-56631591	ANKRD52
A_24_P111996	4.237815	4.8076544	NM_000410	chr6:26094484-26094543	HFE
A_23_P253301	9.652121	9.720337	NM_053024	chr3:149683394-149683335	PFN2
A_23_P116168	7.854397	7.965057	NM_032424	chr11:105879100-105879041	MSANTD4
A_23_P404005	11.176093	10.804133	NM_152274	chrX:152853470-152853411	FAM58A

A_23_P143274	5.912217	5.805595	NM_024958	chr20:335267-335326	NRSN2
A_23_P124024	8.133852	7.6657114	NM_032286	chr5:6374450-6372669	MED10
A_33_P3279940	7.285628	6.613397	NM_032027	chr1:62166666-62166607	TM2D1
A_33_P3311498	5.734664	4.9196157	NR_026837	chr12:72647369-72647310	TRHDE-AS1
A_23_P367628	9.18846	9.308734	NM_014802	chr12:22601833-22601774	C2CD5
A_33_P3299892	14.082455	14.037533	NM_006004	chr1:46782387-46782446	UQCRH
A_33_P3223660	7.1355968	6.7778907	NR_023389	chr9:98637536-98637477	LINC00476
A_32_P331052	4.4142246	4.6340876	NM_080833	chr20:60987695-60986009	RBBP8NL
A_23_P161615	6.446615	6.290221	NM_002689	chr11:65062111-65063057	POLA2
A_24_P395317	4.516325	4.457915	NM_014953	chr13:73330377-73330318	DIS3
A_33_P3388815	7.0686193	7.1145096	NM_003838	chr1:74671138-74671197	FPGT
A_23_P92334	3.2200987	3.327208	NM_024943	chr4:38969231-38969172	TMEM156
A_33_P3231602	4.9688425	5.018555	NM_152484	chr19:37902622-37902563	ZNF569
A_33_P3316348	6.781677	7.114929		chr15:043885724-043885783	
A_24_P216087	8.601424	8.960193	NM_019088	chr19:39876843-39876784	PAF1
A_23_P301336	5.0687747	5.6480737	NM_014472	chr10:100004055-100004114	R3HCC1L
A_23_P28105	8.500758	8.233505	NM_004622	chr2:122522998-122523057	TSN
A_33_P3355717	8.574082	8.729408	AK097098	chr14:69262570-69262511	ZFP36L1
A_23_P212945	2.3221061	2.3900566	NM_000200	chr4:70898958-70902030	HTN3
A_23_P36345	5.6691146	5.5218563	NM_006645	chr11:72468822-72466750	STARD10
A_23_P117852	11.885905	11.933537	NM_014736	chr15:64657906-64657847	KIAA0101
A_23_P52531	6.3623505	5.937695	NM_152644	chr10:124609990-124608947	FAM24B
A_23_P204751	7.4708767	7.6843433	NM_020039	chr12:50477278-50477337	ASIC1
A_23_P429092	4.8121057	5.2240334	NM_001145207	chr6:135357521-135357462	HBS1L
A_23_P28886	12.644803	12.546871	NM_002592	chr20:5096102-5095957	PCNA
A_23_P54230	7.0495615	7.158974	NM_207660	chr14:89041174-89042219	ZC3H14
A_32_P85999	4.668851	4.200089	NM_001257	chr16:83830027-83830086	CDH13
A_23_P40174	5.1216097	5.031865	NM_004994	chr20:44645121-44645180	MMP9
A_23_P392384	6.6291075	6.962084	NM_001185095	chr9:133998333-133998392	AIF1L
A_33_P3353672	9.19384	8.828288	NM_004249	chr4:13369441-13369382	RAB28
A_33_P3286492	5.4243875	5.835919	BC054009	chr8:76402375-76402434	HNF4G
A_23_P311201	6.477784	5.972143	XM_003846425	chr1:24291533-24291474	
A_33_P3267198	4.12871	4.312472	AK125684	chr10:79627888-79627947	
A_23_P56553	7.8831525	7.720968	NM_024770	chr2:172180571-172180512	METTL8
A_33_P3344204	6.617707	5.606936	NM_024786	chr5:795933-795874	ZDHHC11
A_23_P210900	7.250166	7.2073135	NM_018677	chr20:33515343-33515401	ACSS2
A_23_P101013	4.4815574	4.252093	NM_007267	chr17:76113361-76109658	TMC6
A_23_P107211	7.1277933	7.0785236	NM_201434	chr17:40278796-40277881	RAB5C
A_23_P112482	8.234884	8.446196	NM_004925	chr9:33441824-33441765	AQP3
A_23_P121665	6.8641896	6.9596243	NM_020777	chr4:7744241-7744300	SORCS2
A_33_P3382086	7.463331	7.4828787	NM_138418	chr16:698235-698294	FAM195A
A_23_P11800	9.511553	9.542791	NM_018584	chr1:20809460-20809401	CAMK2N1
A_23_P30307	8.621838	8.77371	NM_004270	chr5:156565858-156565799	MED7
A_23_P2967	6.561014	6.634517	NM_003917	chr14:24029590-24029531	AP1G2
A_33_P3259557	7.6897593	7.628486	NR_036476	chr12:56229970-56230029	TMEM198B
A_32_P130577	4.8374195	4.5206065	BC066989	chr2:190649903-190649962	
A_23_P89410	9.151805	9.370842	NM_003766	chr17:40962879-40962820	BECN1
A_23_P145694	11.28883	10.2648	NM_001673	chr7:97481520-97481461	ASNS
A_23_P101380	4.351853	4.780489	NM_198540	chr19:41931342-41931283	B3GNT8
A_24_P248185	2.3221061	2.3900566	NM_153685	chr12:6803073-6803014	PIANP
A_33_P3332438	8.257322	8.868467	NM_138785	chr6:149901799-149901858	GINM1
A_33_P3406408	7.8246317	7.9655123	NM_022077	chr20:35944983-35945042	MANBAL
A_33_P3306659	9.645008	9.759247	AB002446	chr5:114504690-114504749	LOC728254
A_24_P269895	8.310453	8.256001	NM_194247	chr2:178080329-178080388	HNRNPA3
A_33_P3613516	3.506776	3.7889378	AK024653	chr3:128219146-128219205	LOC254057
A_33_P3479999	15.801052	15.777722	BC014228	chr12:51517952-51517893	LOC494150
A_33_P3259960	4.881247	4.8182755	AK125579	chr7:55505936-55505877	LOC100128019
A_24_P64100	10.84107	10.24888	XM_005273526	chr8:23432812-23432871	SLC25A37
A_23_P153855	6.5521784	6.5927296	NM_020855	chr19:22849435-22849494	ZNF492

A_24_P535219	6.6352577	6.928837	KC839989	chr6:170107576-170107517	PHF10
A_33_P3240295	8.109865	7.62881		chr2:074534419-074534360	
A_33_P3379506	9.036449	9.0948925	NM_138798	chr2:99787060-99787001	MITD1
A_33_P3272539	7.8807936	7.9789567	NM_001265594	chr1:6528219-6528160	PLEKHG5
A_23_P116387	8.529621	8.549762	NM_001040694	chr11:61919742-61919801	INCENP
A_33_P3288484	5.183432	5.01116	NM_020466	chr6:90342022-90341963	LYRM2
A_23_P256375	9.09079	9.125559	NM_004604	chr16:31051321-31051380	STX4
A_24_P56388	10.814958	10.638592	NM_181054	chr14:62214845-62214904	HIF1A
A_33_P3645465	6.3560457	6.3376627	NR_026932	chr10:112629567-112629508	PDCD4-AS1
A_23_P114164	9.044441	8.957512	NM_013444	chrX:56593053-56593112	UBQLN2
A_23_P1331	6.169977	5.4277916	NM_080801	chr10:71718811-71718870	COL13A1
A_32_P90080	6.9971433	6.997992	NM_031905	chr7:102739132-102739191	ARMC10
A_33_P3302280	3.319395	3.6679544	NM_001243533	chr1:26450313-26450372	PDIK1L
A_23_P312174	7.4870644	7.2177043	NM_015120	chr2:73836752-73836811	ALMS1
A_32_P57854	12.546871	12.840096	NM_001134693	chr2:27293465-27293406	OST4
A_33_P3414058	4.970782	4.0996695	NM_130803	chr11:64571867-64571808	MEN1
A_32_P76035	6.4393454	5.9153385	NM_033109	chr2:55862315-55862256	PNPT1
A_33_P3414922	2.3221061	2.3900566	BC070144	chr4_gl000194_random:159296-159237	
A_33_P3296482	5.6163673	5.5390196	NM_002844	chr6:128561243-128561184	PTPRK
A_33_P3238573	10.965921	10.846319	NM_014856	chr1:153902036-153901977	DENND4B
A_33_P3613000	5.130788	5.6813455	NM_001105539	chr8:81431950-81432009	ZBTB10
A_33_P3371910	7.3331876	7.555506	NM_138394	chr2:38809115-38809056	HNRNPLL
A_23_P122815	9.903686	10.040499	NM_001219	chr7:128410369-128410428	CALU
A_23_P200801	5.064486	5.0549912	NM_001002811	chr1:144890739-144890680	PDE4DIP
A_23_P111701	11.893164	11.98762	NM_004126	chr7:93555764-93555823	GNG11
A_33_P3403048	4.2645354	4.845662	AK294208	chr2:85770672-85770731	
A_23_P94762	5.9431615	6.250575	NM_058230	chr5:178311237-178311296	ZNF354B
A_23_P64888	4.86602	4.826601	NM_023921	chr12:10978070-10978011	TAS2R10
A_33_P3244141	9.628123	9.821574	NM_001025579	chr17:8371379-8371438	NDEL1
A_33_P3239084	5.65913	5.6688375	NM_201400	chr16:5134996-5134937	FAM86A
A_33_P3259253	4.4732285	5.140075	NM_006139	chr2:204599903-204599962	CD28
A_23_P90589	9.160014	9.116583	NM_022915	chr2:224831754-224831813	MRPL44
A_23_P56140	7.969014	8.100261	NM_001319	chr19:1980005-1980195	CSNK1G2
A_33_P3360326	5.1270876	5.131476	BQ932264	chr11:61593923-61593982	
A_33_P3423425	10.285808	10.1778555	NM_014106	chr15:35270627-35270568	ZNF770
A_33_P3215611	6.556939	6.6875815		chr4:165878012-165877953	
A_23_P819	10.352814	10.27903	NM_005101	chr1:949567-949626	ISG15
A_23_P4611	5.6100736	5.5231314	NM_012254	chr19:59009780-59009721	SLC27A5
A_23_P129466	7.0923405	7.1885424	NM_024997	chr16:10577266-10577325	ATF7IP2
A_23_P164638	3.950175	4.4319587	NM_024691	chr19:58005503-58005562	ZNF419
A_23_P207981	6.6148667	6.7388844	NM_004232	chr18:67993891-67993950	SOCS6
A_23_P119040	3.875134	3.3292782	NM_001142966	chr18:19085780-19085839	GREB1L
A_32_P215179	6.3399367	6.228157	NM_006985	chr16:15039523-15039745	NPIPA1
A_24_P319354	9.554891	9.999754	NM_001005781	chr2:203084816-203084757	SUMO1
A_32_P30710	14.971375	14.953241	NM_000978	chr17:37006703-37006644	RPL23
A_23_P216468	6.3316355	7.0497046	NM_004170	chr9:4587002-4587061	SLC1A1
A_23_P107036	9.923998	9.822338	NM_003876	chr17:21101502-21101443	TMEM11
A_33_P3591810	7.332845	7.3754406	AF119858	chr7:46822781-46822840	
A_32_P208120	6.2195454	6.291869	NM_153498	chr10:12871082-12871141	CAMK1D
A_23_P122174	8.675222	8.340543	NM_022550	chr5:82500709-82554376	XRCC4
A_33_P3395403	5.704751	6.0290055		chr1:144921882-144921823	
A_33_P3405743	10.41574	10.679169	NM_001130042	chr1:75171765-75171706	CRYZ
A_23_P210210	10.827929	11.255164	NM_001430	chr2:46613666-46613725	EPAS1
A_23_P47517	8.800651	8.520388	NM_017966	chr11:60897849-60897790	VPS37C
A_23_P146198	4.8287263	4.6267014	NM_000780	chr8:59403473-59403414	CYP7A1
A_23_P8432	10.667409	10.732501	NM_006833	chr7:99689299-99689358	COPS6
A_23_P300056	4.5168962	4.512016	NM_044472	chr1:22416563-22416622	CDC42
A_23_P117602	4.39669	4.184182	NM_004131	chr14:25100295-25100236	GZMB

A_33_P3271430	8.90419	9.266534	NM_203351	chr17:61773608-61773667	MAP3K3
A_23_P341503	3.979405	3.9149709	NM_178339	chr3:37441068-37441127	C3orf35
A_23_P205686	6.9721518	7.59341	NM_000021	chr14:73686629-73686688	PSEN1
A_23_P110504	9.905243	9.936146	NM_030782	chr5:1318471-1318412	CLPTM1L
A_23_P89249	8.681072	8.763874	NM_001005862	chr17:37884829-37884888	ERBB2
A_24_P289170	5.256281	5.077692	NM_001080414	chr14:91875104-91875045	CCDC88C
A_23_P108922	8.728976	7.872838	XM_005264399	chr2:74382244-74382185	MOB1A
A_23_P355471	7.710079	7.887507	NM_201636	chr19:3599878-3595904	TBXA2R
A_24_P942730	7.1509075	7.1800604	NM_152455	chr15:43651055-43650996	ZSCAN29
A_33_P3436316	8.793272	8.882125	NM_001164603	chr20:30956857-30956916	ASXL1
A_23_P146417	11.01481	11.185318	NM_032012	chr9:111777792-111777733	TMEM245
A_23_P14915	8.164878	7.8218737	NM_001896	chr16:58192239-58192180	CSNK2A2
A_32_P25253	8.241516	8.362931	NM_030940	chr9:88889135-88887000	ISCA1
A_33_P3264505	9.436399	8.609337	NM_015962	chr14:75203324-75203383	FCF1
A_23_P114670	6.7232094	6.403056	NM_014448	chr1:3397479-3397538	ARHGEF16
A_33_P3369956	8.952178	9.299647	NM_148416	chr16:28847384-28847443	ATXN2L
A_33_P3514487	9.512687	9.026928	NM_198481	chr19:54544173-54544114	VSTM1
A_24_P247536	9.866249	9.763328	NM_001040057	chr7:92208653-92207671	FAM133B
A_24_P932646	4.4028473	4.481802	AY358123	chr2:10143804-10143745	
A_32_P79115	6.3030963	6.395406		chr1:149287832-149287773	
A_23_P101615	6.35743	6.268946	NM_001042474	chr19:36673518-36673459	ZNF565
A_23_P87580	5.7333574	5.599586	NM_012404	chr12:48866669-48866728	ANP32D
A_33_P3752697	12.521011	12.54159	AK098235	chrX:154589233-154589292	
A_23_P128828	11.848478	11.86194	NM_004643	chr14:23792691-23793231	PABPN1
A_23_P140563	6.268288	6.1151395	NM_001040655	chr15:99676551-99676528	TTC23
A_32_P153725	9.425696	9.583407	NM_015275	chr12:105562796-105562855	KIAA1033
A_23_P303810	7.9529543	7.958541	NM_153605	chr3:97663710-97663769	CRYBG3
A_32_P157945	2.3221061	3.1851518	NM_004415	chr6:7586582-7586641	DSP
A_23_P96990	9.478113	9.320981	NM_002533	chr1:224415182-224415123	NVL
A_33_P3287997	4.7244725	5.0993657	NM_001012715	chr9:132084813-132084872	C9orf106
A_23_P124438	5.147001	5.287931	NM_001039127	chr4:156150-156209	ZNF718
A_32_P8251	10.116556	10.073508	NM_020901	chr11:611931-611990	PHRF1
A_23_P115407	2.3221061	2.98612	NM_146421	chr1:110231672-110231731	GSTM1
A_23_P145197	9.85144	9.3222065	NM_004053	chr6:41900519-41900578	BYSL
A_23_P39375	3.9483678	3.8348007	NM_018316	chr19:18780745-18780804	KLHL26
A_33_P3337727	3.7412047	4.585135		chr17:042101761-042101702	
A_23_P213699	7.2040467	6.3837595	NM_013982	chr5:139227327-139227268	NRG2
A_32_P231568	2.3221061	3.807558	NM_152573	chr9:85597575-85597516	RASEF
A_33_P3263523	9.229008	9.260631	NM_024319	chr1:228289841-228289782	C1orf35
A_33_P3280729	7.2605147	6.5766582	NM_001145204	chr16:13334091-13334150	SHISA9
A_23_P53736	8.944113	9.035948	NM_033624	chr12:117581868-117581809	FBXO21
A_33_P3357580	10.277651	9.757161	NM_016183	chr1:19586563-19586622	MRTO4
A_33_P3266410	5.178631	5.1759424	NM_002383	chr16:29819090-29819149	MAZ
A_23_P118815	11.999136	11.852174	NM_001012271	chr17:76220720-76220779	BIRC5
A_33_P3738458	5.8269725	5.193813	NM_022648	chr2:218667628-218667569	TNS1
A_23_P204503	6.7145166	6.780275	NM_006253	chr12:120118705-120118764	PRKAB1
A_24_P66001	6.294408	6.397453	NM_001003684	chr22:30165937-30165996	UQCR10
A_33_P3375762	4.7606754	4.8245153	NR_040117	chr9:90473890-90473831	LOC392364
A_32_P220307	14.783885	14.867456	NM_001000	chrX:118920597-118920538	RPL39
A_23_P120594	7.6167493	7.609253	NM_032501	chr20:24987088-24987029	ACSS1
A_23_P205216	6.182459	6.073065	NM_006649	chrX:129059066-129059125	UTP14A
A_23_P106859	10.606829	10.476372	NM_006067	chr16:85813076-85813017	EMC8
A_24_P411899	8.38307	8.820303	NM_183419	chr8:101269850-101269791	RNF19A
A_23_P12189	9.040247	8.90419	NM_213566	chr1:10521342-10521283	DFFA
A_33_P3287310	6.861025	7.041284		chr10:050463136-050463195	
A_24_P208909	7.244713	7.0087047	NM_015271	chr4:154259817-154259876	TRIM2
A_33_P3236563	5.9015374	5.5396805	NM_001161473	chr11:67786661-67786720	ALDH3B1
A_24_P49383	8.066417	8.142876	NM_024684	chr11:77580798-77580857	AAMDC
A_33_P3314301	4.152301	4.717788	NM_014979	chr5:75621242-75621301	SV2C

A_33_P3218694	6.1371427	5.9049497	NM_002687	chr14:39652268-39652327	PNN
A_23_P302094	10.270771	10.108512	NM_033416	chr2:131103666-131103813	IMP4
A_33_P3220663	3.7356572	3.50148	NM_145064	chr12:57637301-57637242	STAC3
A_23_P14948	10.284864	10.604258	NM_003791	chr16:84087586-84087527	MBTPS1
A_23_P164826	11.114081	11.149814	NM_006397	chr19:12924276-12924335	RNASEH2A
A_33_P3245454	2.521959	2.3900566	NR_027420	chr21:9921497-9921438	LOC389834
A_23_P329152	8.325163	7.668565	NM_012218	chr19:10802660-10802719	ILF3
A_23_P94009	11.360216	11.186832	NM_016200	chr7:117832101-117832160	NAA38
A_23_P36226	8.201383	7.064947	NM_032315	chr1:9642451-9642510	SLC25A33
A_23_P13438	7.7939425	7.5073686	NM_032320	chr11:13410160-13410101	BTBD10
A_23_P166051	6.7865624	6.5589294	NM_031229	chr20:411040-411099	RBCK1
A_24_P124672	13.646116	13.57192	NM_001037494	chr12:120935925-120935984	DYNLL1
A_33_P3228375	4.939454	4.6561155	NM_020750	chr6:43491457-43491398	XPO5
A_23_P100795	5.808791	5.8400164	NM_213662	chr17:40465982-40465923	STAT3
A_33_P3411848	7.9268246	8.241175	NM_032488	chr19:42891318-42891259	CNFN
A_33_P3803639	11.222994	10.860369	NM_021138	chr9:139820997-139821056	TRAF2
A_24_P187614	4.7155333	4.800382	AK097637	chr22:41768715-41768656	LOC100129648
A_23_P154037	8.776876	7.9342175	NM_001159	chr2:201535624-201535683	AOX1
A_32_P176550	5.3882203	5.622055	NM_152405	chr5:78617809-78617868	JMY
A_23_P108823	8.676395	8.266755	NM_032523	chr2:179260253-179260312	OSBPL6
A_23_P1956	10.041927	9.963184	NM_001080501	chr11:62558150-62558091	TMEM223
A_33_P3377274	4.435755	4.8291903		chr16:029144039-029143980	
A_33_P3277407	4.529186	4.9063034	NM_182931	chr7:104751253-104751312	KMT2E
A_33_P3312754	6.5228567	6.831626		chr16:79755283-79755224	
A_32_P74409	5.0669622	5.1442804	NM_001145033	chr11:43965338-43965397	C11orf96
A_23_P107612	4.6287947	4.721886	NM_004163	chr18:52556632-52556691	RAB27B
A_33_P3233160	8.466138	8.58671	NM_001282761	chr2:55761036-55761095	CCDC104
A_23_P32233	4.79294	5.1242785	NM_004235	chr9:110248108-110248049	KLF4
A_24_P96474	7.729416	8.036665	NM_032287	chr22:44888791-44888732	LDOC1L
A_24_P156576	5.6364613	5.8655252	NM_017856	chrX:14038346-14038287	GEMIN8
A_24_P56194	7.004422	6.6231937	NM_001310	chr12:12797484-12797543	CREBL2
A_24_P233960	6.980636	6.628088	NM_030918	chr1:151670954-151671013	SNX27
A_23_P120243	2.5023205	3.0715272	NM_024501	chr2:177055372-177055431	HOXD1
A_23_P204791	2.5928478	3.806982	NM_000620	chr12:117651049-117650990	NOS1
A_23_P123393	5.4678526	5.52225	NM_004519	chr8:133141528-133141469	KCNQ3
A_33_P3372124	9.08893	9.418352	NM_001143906	chr12:112591305-112591364	TRAFD1
A_23_P61580	6.1791	6.077261	NM_182543	chr10:18834783-18834724	NSUN6
A_23_P1014	7.9136724	7.606887	NR_026761	chr1:211605740-211605799	LINC00467
A_33_P3577671	10.458079	10.270273	NM_130434	chr15:65739034-65738975	DPP8
A_23_P11664	10.997825	10.995432	NM_004768	chr1:70716494-70716553	SRSF11
A_23_P106463	8.790794	8.958401	NM_002537	chr15:64980123-64980064	OAZ2
A_33_P3258392	4.0135803	4.266578	NM_001955	chr6:12296218-12296277	EDN1
A_32_P42925	12.638172	12.741961	NM_033546	chr18:3278058-3278117	MYL12B
A_23_P123866	8.268076	8.42935	NM_016525	chr9:34251982-34252041	UBAP1
A_23_P121716	10.844669	10.167114	NM_005139	chr4:79525495-79531210	ANXA3
A_23_P105691	3.875278	3.0642855	NM_018654	chr12:13102731-13102672	GPRC5D
A_33_P3274701	4.255438	4.6074076	AK024162	chr1:2144562-2144503	
A_23_P106633	8.360956	8.237112	NM_018380	chr16:68055311-68055252	DDX28
A_33_P3223825	4.646744	5.1293306	NM_001110199	chr7:75915038-75915097	SRRM3
A_23_P19987	11.539543	11.634156	NM_006547	chr7:23350828-23350769	IGF2BP3
A_23_P11859	6.021137	5.9515257	NM_016371	chr1:162773299-162774089	HSD17B7
A_23_P27606	7.725787	7.8698473	NM_004843	chr19:14163217-14163276	IL27RA
A_24_P414952	7.7285795	8.022236	NM_022484	chr7:112406317-112406258	TMEM168
A_23_P87257	9.102738	9.062921	NM_022061	chr11:6704008-6703605	MRPL17
A_33_P3214803	4.537468	4.609988	NM_021620	chr6:100062311-100062370	PRDM13
A_33_P3258801	5.212534	5.0669622	NM_020427	chr8:143822421-143822362	SLURP1
A_23_P137578	9.095693	8.05346	NM_015176	chr1:224346716-224346775	FBXO28
A_23_P113701	6.742962	6.544529	NM_002607	chr7:536988-536929	PDGFA
A_23_P108785	11.861443	12.12283	NM_005721	chr2:114715503-114715562	ACTR3

A_33_P3394710	5.0657177	5.320107	NM_001168398	chr6:88210965-88211024	SLC35A1
A_33_P3365010	6.52512	6.434149	NM_021795	chr1:205592863-205592804	ELK4
A_23_P386764	9.378145	9.283915	NM_020438	chr9:131852483-131852542	DOLPP1
A_23_P129486	8.972734	8.951726	NM_016332	chr16:1988536-1988477	MSRB1
A_33_P3237760	5.399063	4.9383335		chr8:145538275-145538216	
A_24_P80204	2.3221061	2.832371	NM_005434	chr2:110841931-110841872	MALL
A_33_P3416009	4.5606294	2.3900566	AK001274	chr21:34947842-34947783	DONSON
A_24_P375322	3.922895	3.3586793		chr2:91969703-91969762	GGT8P
A_23_P111487	6.6375914	6.4880457	NM_001128853	chr7:100485375-100485434	SRRT
A_23_P89310	7.8674846	7.671803	NM_014964	chr17:19239914-19239973	EPN2
A_23_P388900	3.5751357	3.6189904	NM_018420	chr1:116611811-116611870	SLC22A15
A_24_P206121	6.718018	6.880954	NM_004137	chr5:169812341-169810819	KCNMB1
A_23_P396353	8.325855	7.8883724	NM_020921	chr14:51187234-51187175	NIN
A_33_P3229246	5.2600203	4.980129	NM_003528	chr1:149858160-149858101	HIST2H2BE
A_24_P943613	8.5044	7.212795	NM_015173	chr4:38140705-38140764	TBC1D1
A_23_P89431	7.803832	8.242332	NM_002982	chr17:32584050-32584109	CCL2
A_23_P121602	9.914526	9.810885	NM_003864	chr4:174298478-174298537	SAP30
A_23_P45294	9.885595	10.009428	NM_207318	chrX:103436010-103436069	FAM199X
A_23_P47034	7.1106377	7.079374	NM_002729	chr10:94454858-94454917	HHEX
A_24_P709377	2.3221061	2.3900566	NR_015377	chr2:114024248-114024307	PAX8-AS1
A_33_P3618429	7.736155	7.617489		chr15:101088249-101088190	PRKXP1
A_24_P58177	5.0607805	5.160762	NM_001286473	chr14:91633926-91633985	C14orf159
A_24_P16124	9.274622	9.674784		chr6:29718692-29718633	IFITM4P
A_33_P3255814	8.531144	7.575131	NM_001286264	chr6:24425650-24425709	MRS2
A_23_P82065	2.3221061	2.3900566	NM_022726	chr6:80625140-80625081	ELOVL4
A_23_P92672	4.344023	4.7093124	NM_002538	chr5:68849515-68849574	OCLN
A_33_P3269803	8.873998	8.842437	NM_014718	chr12:7311467-7311526	CLSTN3
A_24_P341187	4.654019	5.268415	NM_020944	chr9:35738622-35738563	GBA2
A_33_P3288384	5.3520803	5.798298	NM_001143937	chr11:14535571-14535512	PSMA1
A_24_P407930	10.333681	10.093741	NM_031216	chr18:12987277-12987336	SEH1L
A_32_P48559	3.8737683	4.2590213	NM_001109660	chr16:30035091-30035150	C16orf92
A_23_P203743	3.7394218	3.899777	NM_012296	chr11:77926747-77926688	GAB2
A_33_P3274768	6.224799	6.7131405	AK128756	chr9:70348528-70348587	LOC100132790
A_23_P94494	6.6069927	6.9949	NM_007005	chr9:82340031-82340090	TLE4
A_24_P79054	6.4236917	6.2627244	NM_000660	chr19:41847802-41838142	TGFB1
A_33_P3389178	8.555081	8.880371	U78169	chr12:48138315-48138256	RAPGEF3
A_33_P3244728	10.546116	10.712155	NM_004525	chr2:169983828-169983769	LRP2
A_23_P10873	4.982411	4.8636184	NM_003263	chr4:38798072-38798013	TLR1
A_24_P108863	6.4947047	7.3222127	NM_001037540	chrX:17772278-17772337	SCML1
A_33_P3246068	11.649645	11.645525	NM_014017	chr1:156028235-156028294	LAMTOR2
A_23_P5551	13.917352	13.650108	NM_005381	chr2:232319780-232319721	NCL
A_33_P3385101	9.136642	9.38109	NM_019009	chr11:1297951-1297892	TOLLIP
A_33_P3254650	4.1793323	4.261988		chr14:021853025-021853084	
A_23_P13137	3.5176904	3.6359134	AY358815	chr11:113118623-113118683	
A_24_P780052	13.739934	13.874355	NM_002295	chr3:39452399-39452458	RPSA
A_23_P76690	10.618973	9.853637	NM_006002	chr13:76178950-76179910	UCHL3
A_24_P721699	2.528599	2.3900566	NR_015380	chr19:58866199-58866258	A1BG-AS1
A_23_P211627	7.8586316	8.303961	NM_007172	chr22:45580381-45580440	NUP50
A_23_P144165	8.386878	8.191202	NM_014648	chr3:108412711-108412770	DZIP3
A_33_P3351092	5.428461	5.586828	NM_181616	chr21:32007721-32007780	KRTAP20-2
A_23_P20852	7.679099	8.181625	NM_001698	chr9:93976528-93976469	AUH
A_33_P3413168	6.976555	6.658374	BC007696	chr9:116980054-116980113	COL27A1
A_23_P10463	12.348225	12.340577	NM_004552	chr1:39500182-39500241	NDUFS5
A_23_P121120	6.1175823	5.2736177	NM_023915	chr3:151012341-151012282	GPR87
A_23_P250571	8.000462	7.815775	NM_005509	chr5:118584473-118584532	DMXL1
A_23_P10081	7.1490088	7.214443	NM_182896	chr3:93773223-93773282	ARL13B
A_23_P201264	5.1561027	4.55311	NM_022787	chr1:10045342-10045401	NMNAT1
A_23_P31725	2.3221061	2.3900566	NM_001715	chr8:11421858-11421917	BLK
A_33_P3327470	6.538065	6.389991		chr19:049542303-049542244	

A_23_P204472	14.458849	14.626991	NM_053275	chr12:120636676-120636503	RPLP0
A_24_P551028	7.641576	6.683916	NM_001001664	chr2:139330359-139330418	SPOPL
A_23_P420209	6.8653545	7.2420197	NM_004751	chr15:59911364-59911423	GCNT3
A_23_P125643	6.7291546	6.5546293	NM_001031739	chrX:15262247-15262188	ASB9
A_33_P3215134	7.8119936	7.6386356	NM_015694	chr7:149128625-149128566	ZNF777
A_24_P244162	6.329493	6.0261564	NM_018425	chr10:99435228-99435287	PI4K2A
A_33_P3388855	6.2258935	6.5483055	NM_133259	chr2:44190787-44190728	LRPPRC
A_24_P415327	5.511244	5.2229905	NM_000195	chr10:100179822-100177976	HPS1
A_23_P156826	4.9942474	5.05908	NM_032744	chr6:11714061-11714002	ADTRP
A_33_P3305536	4.7128105	4.6311617	AK091261	chr11:28147072-28147131	METTL15
A_33_P3284029	11.741284	11.656988	NM_001316	chr20:47711430-47711489	CSE1L
A_23_P209477	8.951726	8.950091	NM_177983	chr2:27605078-27605019	PPM1G
A_32_P56143	4.3174176	4.1692634		chrX:122648510-122648451	
A_33_P3315554	11.635044	12.284242	NM_001282771	chr2:241418904-241418845	ANKMY1
A_23_P102404	13.025798	12.738014	NM_006429	chr2:73479769-73479828	CCT7
A_32_P164246	4.1474714	4.527407	NM_033260	chr6:1314629-1314688	FOXQ1
A_23_P27346	8.254156	8.543917	NM_005359	chr18:48605060-48605119	SMAD4
A_23_P132669	8.625935	8.979401	NM_001010983	chr3:52728844-52728785	GLT8D1
A_33_P3400688	3.778824	4.0376596	NM_205850	chr15:48434530-48434589	SLC24A5
A_32_P32653	10.006894	9.673813	NM_152699	chr3:196661451-196661510	SENP5
A_23_P432545	8.762079	8.8604355	NM_173584	chr11:831224-831283	EFCAB4A
A_23_P101655	9.566097	9.1197815	NM_004924	chr19:39220305-39220364	ACTN4
A_23_P67748	5.2967186	5.243911	NM_004218	chr19:8467435-8468429	RAB11B
A_23_P202245	8.333729	8.211886	NM_020975	chr10:43625254-43625313	RET
A_23_P426021	9.380321	8.93746	NM_015187	chr4:25749158-25749099	SEL1L3
A_23_P118392	7.416483	7.046368	NM_016084	chr17:17397846-17397787	RASD1
A_23_P410725	2.622517	2.3900566	NM_152356	chr19:11918405-11918464	ZNF491
A_32_P233304	8.656458	8.303549	NM_173083	chr1:226419007-226418948	LIN9
A_23_P83149	5.767167	5.6249933	NM_016014	chr9:74481744-74477494	ABHD17B
A_24_P93896	5.861161	5.8176785	NM_199077	chr10:104687119-104687178	CNNM2
A_23_P29495	10.985409	11.045767	NM_001904	chr3:41281749-41281808	CTNNB1
A_33_P3252394	4.745452	4.724252	NM_006705	chr9:92221400-92221459	GADD45G
A_23_P250212	9.731794	9.913929	NM_001080826	chr8:8175388-8175329	SGK223
A_23_P155830	7.8463745	7.924945	NM_020773	chr4:7034494-7034553	TBC1D14
A_33_P3230528	7.068528	6.6091037		chr17:17000529-17000588	MPRIP
A_33_P3308432	10.462874	10.60462		chr1:002310557-002310498	
A_23_P334608	10.586732	10.602222	NM_000181	chr7:65425765-65425706	GUSB
A_23_P415558	7.1561666	7.3393126	NM_012256	chr7:148952401-148952460	ZNF212
A_32_P226009	4.270191	4.5453186	NM_023073	chr5:37206345-37205544	C5orf42
A_33_P3383524	5.941796	6.274874	NR_024172	chr15:98285957-98285898	LINC00923
A_23_P212800	5.1584907	4.281467	NM_004464	chr4:81207806-81207865	FGF5
A_32_P184518	15.63471	15.442646	NM_000982	chr13:27830365-27830424	RPL21
A_23_P49041	4.443568	4.5846825	NM_024956	chr15:43476683-43476742	TMEM62
A_23_P253921	2.3221061	2.3900566	NM_018651	chr3:44613534-44613593	ZKSCAN7
A_33_P3230290	11.400044	11.632503	NM_030649	chr1:1227895-1227836	ACAP3
A_23_P115105	11.049141	11.095562	NM_001114600	chr1:16722798-16722857	SZRD1
A_23_P74653	9.528321	9.343572	NM_006600	chr1:27269453-27269512	NUDC
A_33_P3345309	8.883937	8.924818		chr2:233390356-233390415	PRSS56
A_23_P16722	8.5616	8.483505	NM_014689	chr2:225630183-225630124	DOCK10
A_23_P93464	6.953741	7.7217007	NM_000056	chr6:80912859-80912918	BCKDHB
A_23_P128408	8.803267	9.104091	NM_016399	chr12:120882083-120882024	TRIAP1
A_33_P3304252	11.224058	10.989109	NM_031903	chr7:42977163-42977222	MRPL32
A_33_P3269069	5.2548304	5.737452	NM_001134492	chr1:87564043-87564102	HS2ST1
A_23_P107963	5.6194897	5.3245378	NM_000148	chr19:49251325-49251268	FUT1
A_23_P3849	6.757166	6.602585	NM_016292	chr16:3721731-3716069	TRAP1
A_23_P112652	8.878671	8.673521	NM_015442	chr3:32815146-32815205	CNOT10
A_23_P415401	4.8107843	5.032923	NM_001206	chr9:72999896-72999837	KLF9
A_23_P316239	8.591153	8.784786	NM_001013845	chrX:149100773-149100714	CXorf40B
A_24_P391586	6.5480156	5.9959416	NM_178507	chr11:120100173-120100232	OAF

A_33_P3222783	7.212795	7.481653	NM_001280790	chr9:136231776-136231717	SURF4
A_23_P101950	12.327538	12.330388	NM_005917	chr2:63834237-63834296	MDH1
A_23_P113789	4.068201	4.0035124	NM_006677	chr3:49148212-49148153	USP19
A_33_P3412538	9.730862	9.819443	NM_032217	chr4:73940618-73940559	ANKRD17
A_23_P215956	11.623884	11.294033	NM_002467	chr8:128753268-128753327	MYC
A_23_P10518	5.6208234	5.8913307	NM_016521	chrX:132351715-132351656	TFDP3
A_23_P64204	7.8528337	7.9455476	NM_004724	chr11:113604384-113604325	ZW10
A_33_P3793702	6.078019	6.357938	NR_027321	chr8:125963234-125963293	LINC00964
A_33_P3223121	5.0791097	5.3591027	NM_001113239	chr7:139416115-139416056	HIPK2
A_24_P339071	5.837058	5.780335	NM_001802	chr16:22358232-22358173	CDR2
A_23_P82738	10.213593	10.270771	NM_012415	chr8:95384415-95384356	RAD54B
A_23_P70409	7.893263	8.026331	NM_203290	chr6:43487873-43488021	POLR1C
A_24_P4816	5.4140253	6.5744877	NM_031412	chr12:10374676-10374735	GABARAPL1
A_23_P119789	7.834527	7.860906	NM_024121	chr2:120979636-120979577	TMEM185B
A_23_P26037	4.1805053	4.539452	NM_032892	chr15:44165889-44165830	FRMD5
A_23_P142289	8.932078	8.804932	NM_002067	chr19:3121011-3121070	GNA11
A_23_P97195	7.0817204	7.2785225	NM_019557	chr1:26158765-26158824	MTFR1L
A_23_P56228	7.1499596	7.203074	NM_016573	chr19:19740535-19740476	GMIP
A_24_P273043	5.3729157	5.1897516		chr6:003978834-003978894	
A_24_P822704	3.603043	3.7884169	NM_001005209	chr2:220415029-220415088	TMEM198
A_23_P202658	13.954257	14.063696	NM_000852	chr11:67354050-67354109	GSTP1
A_24_P108451	13.005178	13.293835	NM_000175	chr19:34891174-34891233	GPI
A_23_P49412	7.9383764	7.966835	NM_024031	chr16:30666890-30667223	PRR14
A_23_P89931	6.987988	7.0239844	NM_022752	chr19:42585435-42585494	ZNF574
A_23_P359430	8.036665	7.995593	NM_015383	chr1:145369062-145369121	NBPF14
A_23_P212617	11.453451	11.745298	NM_003234	chr3:195776652-195776593	TFRC
A_24_P842006	4.3161564	5.017564	NM_001014979	chr16:30770688-30770535	C16orf93
A_33_P3240912	5.2062054	4.9756703	NM_032121	chrX:77126365-77126306	MAGT1
A_33_P3759737	4.4239383	4.712425		chr2:239419392-239419333	LINC01107
A_33_P3358601	4.305903	4.9557834	NM_001170820	chr11:1753699-1753640	IFITM10
A_33_P3401084	9.287131	9.576632		chr17:037308291-037308350	
A_23_P93032	10.271232	10.065493	NM_032367	chr5:76372621-76372562	ZBED3
A_23_P118353	6.905698	7.1052265	NM_014230	chr17:74041401-74040009	SRP68
A_23_P108028	8.115882	8.441713	NM_007145	chr19:36728855-36728914	ZNF146
A_33_P3343467	4.8927026	5.3666434	NR_015401	chr7:44081962-44082021	LINC00957
A_33_P3219105	11.169504	11.040688		chr1:040598754-040598813	
A_33_P3214884	9.8630295	8.658588	NM_002267	chr13:50273552-50273493	KPNA3
A_24_P363896	7.1581097	7.16957	NM_032888	chr9:117072895-117072954	COL27A1
A_23_P209778	8.394869	8.708113	NM_004805	chr2:128604595-128604536	POLR2D
A_33_P3418400	4.0810685	3.7388065	NM_001131028	chr5:81550614-81550673	ATG10
A_33_P3383611	8.1797695	8.25147	NM_199349	chr7:128530153-128530094	KCP
A_23_P83159	8.149439	8.160403	NM_018847	chr9:21331160-21331101	KLHL9
A_23_P105227	3.928296	3.8111715	NM_001014811	chr11:86198388-86176193	ME3
A_23_P128940	6.578019	6.6560206	NM_024558	chr14:50576138-50576079	VCPKMT
A_23_P219197	10.283176	10.337336	NM_134427	chr9:116359708-116359767	RGS3
A_24_P56052	8.999285	9.270045	NM_053023	chr11:58384672-58384731	ZFP91
A_23_P34827	4.7372727	4.983132	NM_020897	chr1:155259312-155259371	HCN3
A_33_P3378284	7.5268216	7.970763	NM_144613	chr19:55861416-55861357	COX6B2
A_24_P250922	2.6775665	2.8801413	NM_000963	chr1:186641528-186641469	PTGS2
A_23_P106103	4.038941	4.6177974	NM_004857	chr14:64936246-64936305	AKAP5
A_23_P119141	9.887251	9.850304	NM_203500	chr19:10597321-10597262	KEAP1
A_23_P210425	7.777026	8.172044	NM_181526	chr20:35177541-35177600	MYL9
A_23_P71981	7.404214	7.4999027	NM_005702	chr17:27187807-27187866	ERAL1
A_23_P34527	6.960344	7.11206	NM_025207	chr1:154961130-154961189	FLAD1
A_32_P136402	9.763328	9.764388	NM_025075	chr3:63821001-63820842	THOC7
A_33_P3593774	4.8853335	4.971138	NM_003629	chr1:46511650-46511591	PIK3R3
A_24_P282210	10.601184	10.548663	NM_005632	chr16:604502-604561	CAPN15
A_23_P56380	11.414224	11.215775	NM_018471	chr2:187373716-187373775	ZC3H15
A_24_P915806	3.5591903	3.797933	NM_001024074	chr2:138722169-138724696	HNMT

A_33_P3383233	5.305557	5.5773745	NM_001282213	chr14:21485301-21485242	NDRG2
A_23_P114353	3.8951437	3.4509306	NM_032946	chrX:101087159-101087100	NXF5
A_23_P104876	7.6556625	7.76081	NM_017425	chr11:124561634-124564213	SPA17
A_33_P3326025	8.114321	8.4632015	AY203940	chr19:8398739-8398680	KANK3
A_23_P104563	3.8860931	4.7169747	NM_001031847	chr11:68527122-68527063	CPT1A
A_33_P3314810	5.1128626	5.382273	XR_246852	chr7:63230062-63230003	LOC101930210
A_32_P52018	4.356121	3.5769901	NM_030948	chr6:13278552-13283703	PHACTR1
A_24_P28811	7.1994047	7.3869653	NR_103761	chr2:131198475-131198416	CYP4F62P
A_23_P363406	4.755164	4.993062	NM_015305	chr14:77253935-77253876	ANGEL1
A_23_P360167	9.50061	9.651842	NM_006400	chr12:57924362-57924303	DCTN2
A_23_P396666	7.6820354	7.766199	NM_015079	chr15:78287623-78287564	TBC1D2B
A_33_P3329098	4.618443	4.1550503	NM_004906	chr6:160176584-160176643	WTAP
A_23_P43726	9.4249115	9.649215	NM_015231	chr11:47800345-47800286	NUP160
A_24_P915196	6.7648067	6.323497	NM_153045	chr9:117408425-117408484	C9orf91
A_33_P3395971	7.6710744	7.1808662	NM_145232	chr19:51600922-51600863	CTU1
A_23_P109333	9.241002	8.817034	NM_004649	chr21:45565392-45565451	C21orf33
A_23_P210886	8.482736	8.300675	NM_138578	chr20:30253383-30253324	BCL2L1
A_23_P96087	6.1891155	6.327021	NM_006026	chr3:129034525-129034466	H1FX
A_23_P502142	7.392914	7.3404465	NM_002037	chr6:111982770-111982711	FYN
A_33_P3209581	4.846062	5.0195546	NM_001170738	chr12:176547-208312	IQSEC3
A_33_P3396200	10.232068	10.058578	NM_002319	chr7:100172312-100172253	LRCH4
A_23_P106675	8.499775	8.410017	NM_002661	chr16:81991575-81991634	PLCG2
A_33_P3309643	4.816511	4.88765		chr7:143526270-143526211	
A_24_P35891	7.365104	7.5380583	NM_016423	chr14:21558392-21558333	ZNF219
A_23_P388146	10.892431	11.028464	XR_243967	chr19:58379773-58379832	
A_24_P294124	7.700069	7.501716	NM_014755	chr2:64859160-64859101	SERTAD2
A_33_P3311473	4.581826	4.8467917	NM_020453	chr4:47595261-47595320	ATP10D
A_23_P66260	7.6971006	8.376837	NM_003414	chr16:31927638-31927697	ZNF267
A_23_P26777	9.733401	9.8203745	NM_024844	chr17:73230872-73231242	NUP85
A_33_P3211965	6.5392265	6.7445807	BM674043	chr16:30832743-30832684	
A_24_P185036	8.879179	8.369848	NM_018348	chr16:71316895-71316836	CMTR2
A_23_P34433	8.970504	9.046381	NM_001009881	chr1:52889223-52889164	ZCCHC11
A_32_P228501	10.428835	10.384741	NM_139286	chr9:116029538-116029479	CDC26
A_23_P123732	6.1234136	6.235303	NM_001001551	chr9:86258959-86259018	IDNK
A_23_P21425	8.111082	8.350471	NM_178471	chrX:129518731-129518672	GPR119
A_23_P321452	7.9259453	8.210513	NM_173791	chr10:119042996-119042937	PDZD8
A_23_P378690	6.5017376	6.740136	NM_001008495	chr8:91634707-91634648	TMEM64
A_23_P63896	9.110252	9.517648	NM_000043	chr10:90774545-90774604	FAS
A_24_P82155	2.3221061	2.3900566	NM_182703	chr15:65250029-65250088	ANKDD1A
A_33_P3317431	14.503544	14.526909		chr7:144737677-144737618	
A_23_P205255	5.716335	6.0916953	NM_001242417	chr14:102675959-102676018	WDR20
A_23_P266	5.392952	5.31962	NR_023916	chr1:70385301-70385360	PIN1P1
A_23_P216307	4.279873	4.2639728	NM_004349	chr8:92972310-92972251	RUNX1T1
A_33_P3266614	5.1802063	5.3580627		chr21:047053112-047053053	
A_23_P11841	3.8231695	3.7618148	NM_001684	chr1:203690464-203690523	ATP2B4
A_32_P22401	10.750368	10.655569	NM_018067	chr1:36646159-36646218	MAP7D1
A_23_P24104	13.191831	12.102104	NM_002658	chr10:75677050-75677109	PLAU
A_23_P99253	5.870637	6.3532476	NM_004664	chr12:81191501-81191442	LIN7A
A_24_P135444	8.690077	8.866781	NM_001144	chr16:56396402-56396343	AMFR
A_23_P57413	5.9223757	5.9696608	NM_014634	chr22:22285539-22279996	PPM1F
A_23_P377291	6.201584	6.134747	NM_003236	chr2:70675378-70675319	TGFA
A_23_P159476	9.715432	9.67514	NM_003731	chr9:140083706-140084306	SSNA1
A_23_P148255	2.3221061	2.3900566	NM_153488	chrX:151886664-151886723	MAGEA2B
A_23_P214533	5.9888964	5.65385	NM_030899	chr6:28293096-28293037	ZSCAN31
A_23_P141520	8.752386	8.991302	NM_174893	chr17:6920231-6920290	C17orf49
A_23_P10858	7.2267094	7.6915407	NM_015114	chr12:133302989-133302930	ANKLE2
A_23_P115792	9.026928	8.638604	NM_001001974	chr10:124189516-124189575	PLEKHA1
A_23_P11697	3.9360533	3.6168718	NM_145205	chr1:34329965-34330024	HMGB4
A_23_P24215	7.848129	8.048493	NM_015188	chr10:96295315-96295374	TBC1D12

A_23_P162322	5.7273183	5.6514206	NM_003394	chr12:49359496-49359437	WNT10B
A_23_P47377	11.268042	11.302273	NM_016142	chr11:43877854-43877913	HSD17B12
A_23_P14105	7.0015225	7.359319	NM_001268	chr13:49063410-49063351	RCBTB2
A_24_P256404	4.866878	4.799622	AK093202	chr1:16864566-16864625	LOC100133616
A_23_P202501	4.208373	4.0044274	NM_001031709	chr10:90074354-90074295	RNLS
A_33_P3236020	5.864071	5.6941648	NM_138379	chr5:156346459-156346400	TIMD4
A_33_P3334535	8.251077	8.031742	NM_017676	chr5:102421763-102421704	GIN1
A_23_P204609	9.5499115	9.817699	NM_017599	chr12:95694737-95694796	VEZT
A_23_P123727	6.4560432	6.6084547	NM_024617	chr9:88903200-88903141	ZCCHC6
A_33_P3420486	4.308138	4.227016	AK025118	chr5:179032932-179032873	
A_23_P56703	5.264602	4.751463	NM_001277053	chr2:85049013-85048954	TRABD2A
A_33_P3344603	6.336094	5.978769	NM_001002844	chr15:56991785-56991726	ZNF280D
A_33_P3351197	3.9028976	3.738409	NR_015363	chr9:41954897-41954838	MGC21881
A_32_P122793	6.8578224	6.311244	NR_026920	chr22:31322460-31322519	MORC2-AS1
A_33_P3259203	5.049308	5.009774	XM_005272414	chr13:114515136-114515195	TMEM255B
A_23_P15466	9.1382885	9.073066	NM_004589	chr17:10584322-10584263	SCO1
A_23_P9883	7.575131	7.042818	NM_001079821	chr1:247612029-247612089	NLRP3
A_24_P140608	2.3827417	3.6671486	NM_001945	chr5:139713111-139713052	HBEGF
A_32_P88719	4.1741886	4.961618	NM_001031834	chrX:102192709-102192768	RAB40AL
A_23_P100517	6.7052073	6.8389306	NM_145254	chr16:75481508-75481449	TMEM170A
A_24_P374516	11.997747	12.092395	NM_021109	chrX:12994394-12994453	TMSB4X
A_33_P3235420	8.112337	7.9568305	NM_177532	chr4:74439028-74438969	RASSF6
A_32_P197524	7.8835206	7.5368094	NM_032025	chr3:150302684-150302743	EIF2A
A_23_P108404	6.94669	7.4363413	NM_001037131	chr2:237033264-237033323	AGAP1
A_33_P3299872	7.7738504	7.613041	NM_138571	chr6:126301280-126301339	HINT3
A_23_P154235	9.412224	9.512272	NM_004688	chr2:152127069-152127010	NMI
A_33_P3401301	11.403793	11.49802	NM_001000	chrX:118923925-118920691	RPL39
A_23_P16022	6.7106256	6.599594	NM_005773	chr19:58452297-58452238	ZNF256
A_23_P377141	5.697415	6.16088	NM_015186	chr9:79985462-79985832	VPS13A
A_33_P3316691	5.8484354	6.112818		chr18:014449514-014449573	
A_23_P45799	5.423615	5.400723	NM_004153	chr1:52840560-52840501	ORC1
A_23_P410613	10.489464	10.628776	NM_152261	chr12:107367533-107367592	C12orf23
A_33_P3313456	6.708885	6.971564	NM_001098843	chrX:36403070-36403129	CXorf30
A_33_P3412311	4.696085	4.5766897	AK093358	chr9:67793478-67793537	
A_24_P309645	5.5775127	5.9924	NM_017901	chr12:113730870-113731124	TPCN1
A_23_P115743	6.096609	5.847641	NM_022063	chr10:120069008-120068949	FAM204A
A_33_P3256920	4.323733	4.0838785	NM_058238	chr22:46319002-46318943	WNT7B
A_23_P331253	7.5733104	7.7325077	NM_020383	chr10:111624955-111624896	XPNPEP1
A_23_P109928	9.323842	9.333247	NM_014814	chr3:64004350-64004291	PSMD6
A_24_P751074	6.237657	6.8462687	NM_005238	chr11:128328679-128328656	ETS1
A_33_P3264577	6.6094685	6.6747675	NM_004082	chr2:74588737-74588678	DCTN1
A_33_P3360525	12.784931	12.595854	BU535024	chr10:97949085-97949026	
A_23_P404965	6.450806	6.0913506	NM_005275	chr6:30509291-30509232	GNL1
A_33_P3273664	6.585714	6.1775913	NM_198150	chr5:94903693-94903752	ARSK
A_23_P156861	6.8479967	6.757449	NM_012419	chr6:153332781-153332722	RGS17
A_23_P79628	7.19516	7.350246	NM_014614	chr2:54092618-54092559	PSME4
A_23_P396981	6.0285993	6.300085	NM_001012506	chr3:56653364-56653423	CCDC66
A_23_P11543	7.0396204	7.70289	NM_000147	chr1:24171909-24171850	FUCA1
A_23_P26854	2.3221061	2.3900566	NM_014859	chr17:12894799-12894858	ARHGAP44
A_33_P3258046	5.4549413	5.660081	NM_170685	chr17:47915730-47915671	TAC4
A_24_P214598	6.323139	6.2166853	NM_152542	chr4:89189978-89189919	PPM1K
A_23_P16157	13.420244	13.364828	NM_003685	chr19:6413914-6413855	KHSRP
A_23_P120744	8.187274	8.214201	NM_003906	chr21:47656825-47656766	MCM3AP
A_33_P3708413	5.2892013	5.8973475	NM_003480	chr12:8800746-8800687	MFAP5
A_33_P3411025	6.437312	6.719293	NM_001204300	chr10:99019229-99019170	ARHGAP19
A_33_P3299435	7.151745	6.9637947	NM_005146	chr11:65747203-65747262	SART1
A_24_P21056	8.634343	8.544758	NM_014660	chr7:11091294-11091353	PHF14
A_23_P385206	7.886476	6.9808445	NM_177424	chr1:28150636-28150695	STX12
A_23_P105571	8.238194	8.57851	NM_020244	chr12:102110521-102110580	CHPT1

A_33_P3275500	8.1280775	7.4908714		chr1:17524033-17524092	LOC400743
A_23_P67725	11.886883	11.7751875	NM_032737	chr19:2428242-2428183	LMNB2
A_33_P3328511	6.443676	6.608905	NM_001039083	chr17:44416935-44416876	ARL17B
A_23_P254079	4.768109	5.515504	NM_003943	chr4:77231410-77231469	STBD1
A_33_P3392823	8.10319	8.051502	NM_018467	chr19:17330512-17330571	USE1
A_33_P3422030	5.7258916	5.6182275		chr19:35650773-35650832	FXYD5
A_33_P3222565	4.908549	5.0720525	NM_030927	chr10:82276016-82276075	TSPAN14
A_24_P116805	6.9400377	7.3069706	NM_213662	chr17:40474463-40474404	STAT3
A_33_P3250398	7.0263815	6.6467724	BC025419	chr20:57571712-57571653	CTSZ
A_33_P3275199	7.251292	7.4743643	NM_003724	chr8:143739085-143739026	JRK
A_23_P123071	8.78143	9.114072	NM_001233	chr7:116140389-116140448	CAV2
A_33_P3316555	9.505653	9.229008	NM_004528	chr1:165623528-165623587	MGST3
A_23_P153256	6.2875304	6.464305	NM_198542	chr19:58019003-58019062	ZNF773
A_33_P3406066	6.6162305	7.1504865	NM_173632	chr19:58269249-58269308	ZNF776
A_33_P3351982	8.215336	8.325855	NM_001008215	chr2:99220631-99220572	COA5
A_33_P3411315	8.588668	9.150382	NM_033185	chr17:39150112-39150053	KRTAP3-3
A_32_P94	4.4385085	4.553525	NM_198560	chr3:9540200-9540141	LHFPL4
A_23_P125829	12.830639	13.410927	NM_000291	chrX:77381462-77381521	PGK1
A_23_P152038	5.641778	5.887508	NM_052903	chr15:22868945-22869952	TUBGCP5
A_33_P3298251	4.2150097	4.4608183		chr6:029044462-029044521	
A_23_P110345	7.4169016	7.2818403	NM_012110	chr4:54876291-54876232	CHIC2
A_23_P380965	10.20569	10.30835	NM_016475	chr14:59971064-59971123	JKAMP
A_24_P234116	11.869513	11.860668	NM_017860	chr1:151023792-151023851	C1orf56
A_33_P3319953	4.1942787	4.9363		chrX:76949251-76949192	ATRX
A_32_P85813	8.560089	7.993722	NM_001100426	chr4:99364725-99364784	RAP1GDS1
A_23_P206310	7.5959263	7.798022	NM_014732	chr16:85127639-85127698	KIAA0513
A_23_P208013	5.6817484	5.6752157	NM_017757	chr18:72777025-72777084	ZNF407
A_23_P1199	10.309881	10.462874	NM_014142	chr10:12214844-12214785	NUDT5
A_24_P129632	8.812031	8.982283	NM_004747	chr10:79550783-79550724	DLG5
A_33_P3349843	3.9515328	4.543262	BU171032	chr5:180222497-180222438	
A_33_P3324890	9.114072	9.250853		chr4:013339392-013339451	
A_33_P3215277	7.8584876	7.8246317	NM_173500	chr15:43036609-43036550	TTBK2
A_23_P17287	9.845925	9.912645	NM_001039613	chr2:9628439-9628498	IAH1
A_23_P122233	10.786055	10.736013	NM_001014990	chr5:154346304-154346363	MRPL22
A_23_P357546	4.4107084	4.866154	NM_015488	chr2:219210607-219210666	PNKD
A_33_P3313221	4.5076046	4.4335694	NM_001242739	chr1:43316875-43316934	ZNF691
A_23_P71415	7.425277	7.6221232	NM_018024	chr8:124453600-124453659	WDYHV1
A_24_P100742	6.5300794	6.427474	NM_014189	chr4:2909528-2910257	ADD1
A_33_P3295550	7.649143	7.4090033	NM_030625	chr10:70332072-70332131	TET1
A_23_P251421	10.639288	9.847651	NM_031942	chr2:174233490-174233549	CDCA7
A_33_P3294961	14.025549	14.032981	XM_005250562	chr7:73640637-73640696	LAT2
A_23_P121657	4.5559044	4.629378	NM_005114	chr4:11400729-11400670	HS3ST1
A_33_P3267160	5.8481092	5.5083537	AK124658	chr6:84675872-84675931	
A_33_P3283485	5.058455	5.6595626	XR_109251	chr16:6427992-6428051	LOC440337
A_23_P256084	6.41668	6.7989554	NM_000047	chrX:2854805-2853195	ARSE
A_23_P73097	9.821574	9.548103	NM_170587	chr8:54871444-54871503	RGS20
A_33_P3421219	13.650108	13.660247	AK001442	chr1:182806677-182806618	LOC647070
A_23_P98898	5.3280163	5.272232	NM_001798	chr12:56365789-56365848	CDK2
A_33_P3321417	4.8036456	4.897825	NM_019100	chr1:44684321-44684380	DMAP1
A_33_P3223097	6.006634	5.8824434	NR_026761	chr1:211565258-211565317	LINC00467
A_33_P3296074	9.803411	9.820163	NM_003432	chr5:43175762-43175821	ZNF131
A_24_P332081	6.1061163	5.6645303	NM_001105521	chr10:133998130-133998189	JAKMIP3
A_23_P70249	8.085194	8.398382	NM_001790	chr5:137621299-137621240	CDC25C
A_23_P356152	4.520443	4.8597	NM_001278547	chr10:49634025-49634084	MAPK8
A_23_P90444	9.082555	8.921083	NM_024321	chr19:36128152-36128211	RBM42
A_33_P3299791	4.8766575	4.95572		chr2:67402720-67402661	LOC644838
A_33_P3312222	3.8246334	3.1010718		chr5:017522186-017522245	
A_33_P3397433	8.630993	8.637686		chr3:131962370-131962311	
A_23_P256735	6.9186373	7.3951645	NM_016134	chr8:98155350-98155409	CPQ

A_23_P106773	7.0051365	6.920917	NM_177528	chr16:28603326-28603267	SULT1A2
A_23_P143446	9.666323	9.497883	NM_017446	chr21:26965250-26965191	MRPL39
A_23_P45488	5.71145	5.6766424	NM_021806	chrX:153735560-153735205	FAM3A
A_24_P59667	2.3221061	2.3900566	NM_000215	chr19:17935656-17935597	JAK3
A_24_P296698	10.894218	10.52139	NM_145109	chr17:21218464-21218523	MAP2K3
A_33_P3344229	9.752222	9.645744	NM_003538	chr6:26022181-26022240	HIST1H4A
A_33_P3305023	5.7894397	5.942989	NM_130844	chr16:78134036-78134095	WWOX
A_23_P44724	9.4304	9.818493	NM_001321	chr12:77252613-77252554	CSRP2
A_33_P3309039	10.868946	10.768726	NM_024811	chr11:61170262-61170203	CPSF7
A_33_P3357651	7.4844866	7.6112456	NM_198699	chr21:46117543-46117602	KRTAP10-12
A_23_P12816	8.753195	8.848029	NM_018063	chr10:96354481-96354540	HELLS
A_23_P153320	6.636947	6.8053393	NM_000201	chr19:10396298-10396358	ICAM1
A_33_P3211739	10.438267	10.439577	NM_194071	chr7:137559941-137559882	CREB3L2
A_24_P93452	13.219345	13.122759		chr11:118432021-118432080	
A_23_P344281	2.3221061	2.3900566	NM_001010879	chr19:58102876-58102935	ZIK1
A_23_P250102	3.733239	4.0818353	NM_012298	chr3:12876193-12876252	CAND2
A_23_P133424	12.9688225	12.972569	NM_006930	chr5:133493214-133493155	SKP1
A_33_P3358342	10.329317	10.068425	NM_005067	chr3:150458997-150458938	SIAH2
A_33_P3298930	2.3221061	2.3900566		chr5:71870050-71869991	
A_23_P120227	2.3221061	2.8678474	NM_030915	chr2:30482471-30482530	LBH
A_23_P417331	6.910077	7.4145446	NM_004586	chrX:20173538-20173480	RPS6KA3
A_23_P126939	7.3053923	7.5969253	NM_003929	chr1:205739514-205739455	RAB7L1
A_24_P329795	4.2120757	4.6436534	NM_007021	chr10:45471769-45471710	C10orf10
A_23_P103310	4.141824	4.0787253	NM_002963	chr1:153431390-153430429	S100A7
A_33_P3262028	5.3516397	5.6666207		chr3:197366275-197366334	XLOC_014512
A_32_P187599	12.062395	12.068261	NM_001018067	chr1:67885815-67885756	SERBP1
A_23_P254179	9.457054	9.499588	NM_015339	chr20:49507472-49507413	ADNP
A_23_P155514	4.629378	4.9448967	NM_001622	chr3:186338832-186338891	AHSG
A_24_P129277	3.863964	3.1770675	NM_006092	chr7:30465046-30464987	NOD1
A_23_P413923	6.354451	6.710007	NM_022160	chr9:22451878-22451937	DMRTA1
A_33_P3358069	8.951029	9.150564	NM_174855	chr20:2640801-2640742	IDH3B
A_23_P84070	8.966146	8.779782	NM_016648	chr4:113574268-113574327	LARP7
A_23_P99138	12.758359	12.708443	NM_016497	chr12:6601595-6601536	MRPL51
A_23_P427622	5.712416	6.0845895	NM_139015	chr12:121205342-121205283	SPPL3
A_24_P72479	9.534749	9.714747	NM_006409	chr7:98961217-98963533	ARPC1A
A_33_P3209229	7.690025	7.9531574	NM_014353	chr16:2204081-2204140	RAB26
A_33_P3262124	4.3363676	4.84366	NM_022468	chr16:3100486-3100545	MMP25
A_23_P105118	3.8812041	3.1531444	NM_001005237	chr11:4945276-4945217	OR51G1
A_23_P117582	11.116295	10.558288	NM_130469	chr14:75937082-75937141	JDP2
A_23_P114232	12.345019	12.437426	NM_006406	chrX:23700566-23700625	PRDX4
A_33_P3277953	5.9499264	5.6208234	NR_027333	chr10:25464780-25464721	GPR158-AS1
A_33_P3235217	9.9478	9.539421	NM_001905	chr1:41475866-41475925	CTPS1
A_23_P100868	9.8550205	9.671054	NM_001033580	chr17:34869010-34868951	MYO19
A_23_P203013	5.7845826	6.0160437	NM_002519	chr11:108028826-108028767	NPAT
A_23_P130444	2.3221061	2.3900566	NM_018260	chr19:53087417-53087476	ZNF701
A_23_P384056	10.439577	10.276246	NM_022757	chr3:123632725-123632666	CCDC14
A_23_P100539	4.0188026	3.723091	NM_001171	chr16:16243914-16243855	ABCC6
A_33_P3277437	6.5338	6.623427	NM_001142725	chr16:20809108-20809049	ERI2
A_23_P301138	9.977053	9.979052	NM_001005910	chr3:48731295-48731236	IP6K2
A_23_P253221	8.866781	8.46291	NM_032995	chr2:131804762-131804821	ARHGEF4
A_33_P3260563	4.0910563	4.5894675	NM_007017	chr5:157079204-157079145	SOX30
A_23_P15603	6.3059993	6.029647	NM_024864	chr17:34965169-34965228	MRM1
A_24_P636974	3.723091	4.3266883		chr1:137158-137219	LOC101930216
A_23_P121637	5.479686	5.544874	NM_003619	chr4:119203016-119202957	PRSS12
A_24_P332230	9.713991	9.477348	NM_016648	chr4:113567826-113568004	LARP7
A_33_P3409086	4.546877	4.9455924	NM_006271	chr1:153603076-153603135	S100A1
A_23_P122976	5.9522047	6.2502546	NM_002069	chr7:79848271-79848330	GNAI1
A_33_P3298771	5.0504913	5.2158766		chr11:006078605-006078546	
A_33_P3229156	3.2703228	4.3072686	NM_022082	chr20:61599882-61599941	SLC17A9

A_24_P649624	9.4174595	9.059372	NM_183416	chr1:10368324-10368383	KIF1B
A_33_P3233273	6.947016	5.969927	NM_001142928	chr7:150035004-150035063	LRRC61
A_23_P354074	7.570449	7.861485	NM_000081	chr1:235824436-235824377	LYST
A_32_P56525	9.13957	9.168491	NM_014719	chr7:143550522-143550463	FAM115A
A_23_P105028	9.368131	9.634018	NM_015459	chr11:63396800-63396741	ATL3
A_23_P114952	9.475201	9.346716	NM_016456	chr1:201104631-201104572	TMEM9
A_23_P66110	5.9677114	6.027178	NM_000548	chr16:2136775-2136834	TSC2
A_33_P3237784	8.694674	8.587215	NM_001282167	chrX:48379104-48379163	PORCN
A_23_P215913	10.0259695	10.529507	NM_001831	chr8:27457462-27457403	CLU
A_33_P3252191	4.158456	4.195213		chr2:046238044-046238103	
A_23_P105002	7.263638	7.292393	NM_000327	chr11:62382504-62382563	ROM1
A_23_P203888	6.986639	7.1877213	NM_002429	chr12:56230495-56230436	MMP19
A_23_P411833	8.855042	9.042355	NM_145294	chr16:717721-717780	WDR90
A_33_P3214314	7.2230926	7.6515155	NM_001284241	chr10:86133480-86133539	CCSER2
A_33_P3363485	4.639143	4.967735	NM_018013	chr6:107827569-107827628	SOBP
A_23_P103919	9.144638	9.194231	NM_003779	chr1:161141406-161141347	B4GALT3
A_23_P148807	9.368971	8.665404	NM_003503	chr1:91991047-91991106	CDC7
A_33_P3380751	4.937869	4.787607	NM_003034	chr12:22487308-22487249	ST8SIA1
A_23_P35684	6.8967237	7.0991707	NM_014937	chr10:121588076-121588135	INPP5F
A_23_P98252	8.324828	8.516182	NM_001667	chr11:64789269-64789328	ARL2
A_23_P30200	3.346899	3.1717076	NM_020957	chr5:140565335-140565394	PCDHB16
A_23_P165691	12.198191	12.105285	NM_005805	chr2:162267829-162267888	PSMD14
A_33_P3308903	6.2294354	6.4052444	NM_080875	chr1:1565024-1565083	MIB2
A_33_P3255509	5.187837	5.441803	NM_001161357	chr19:17899295-17899354	FCHO1
A_33_P3391455	5.414499	5.1528683	NM_001160161	chr3:38651416-38651357	SCN5A
A_24_P301547	4.101936	4.683078	NM_017941	chr17:71232691-71232750	C17orf80
A_24_P92174	3.7880683	4.26165	DQ100868	chr14:106494270-106494211	
A_33_P3297921	8.872078	8.943328	NM_016491	chr1:54678253-54678312	MRPL37
A_23_P58993	3.3723485	3.3866239	NM_005943	chr6:39873884-39873825	MOCS1
A_33_P3346866	9.901852	9.906871	NM_017822	chr12:49047334-49047275	KANSL2
A_23_P84910	5.6853642	6.0646095	NM_003446	chrX:47272965-47273024	ZNF157
A_23_P257795	10.925762	11.137785	NM_002488	chr5:140025208-140025149	NDUFA2
A_24_P661641	5.9324846	6.1184897	NR_028408	chr12:46120164-46120105	LINC00938
A_24_P45446	5.182065	5.1561027	NM_052941	chr1:89647795-89647736	GBP4
A_33_P3391578	7.8446856	8.008957	NR_003040	chr11:118873886-118873827	RPL23AP64
A_23_P110167	8.467059	8.193537	NM_002413	chr4:140625256-140625315	MGST2
A_23_P53467	9.479867	9.690325	NM_201612	chr12:99007538-99007479	IKBIP
A_23_P121095	10.502427	10.570412	NM_001068	chr3:25639773-25639714	TOP2B
A_23_P146187	10.956079	10.090082	NM_015169	chr8:67342692-67342751	RRS1
A_24_P743672	6.932765	7.2273817			
A_33_P3246995	12.536509	12.603408		chr7:124940812-124940753	
A_33_P3270966	13.579134	13.743806		chr6:154898103-154898162	
A_23_P115824	10.162213	10.189776	NM_012207	chr10:70102702-70102761	HNRNPH3
A_23_P100654	8.196144	8.515499	NM_020899	chr17:7363005-7362946	ZBTB4
A_33_P3216150	4.8920774	5.1484375	NM_002723	chr12:11461387-11461328	PRB4
A_23_P84334	6.3492064	6.270206	NM_018266	chr3:119149952-119149893	TMEM39A
A_23_P109254	11.248156	11.270389	NM_184234	chr20:34292430-34292371	RBM39
A_23_P113803	8.997559	9.030188	NM_007044	chr6:149916283-149916224	KATNA1
A_23_P104972	3.851746	3.2124805	NM_023945	chr11:60215137-60215196	MS4A5
A_23_P35916	7.572239	7.9941063	NM_000051	chr11:108236085-108236144	ATM
A_23_P157117	5.1417246	5.05534	NM_182898	chr7:28859103-28859162	CREB5
A_24_P330518	6.4750795	7.2532177	NM_001218	chr15:63616558-63616499	CA12
A_33_P3414880	5.257186	5.495815		chr17:043296741-043296682	
A_33_P3444555	4.0309744	4.8328743		chrX:153655315-153655256	
A_23_P119778	7.3821583	6.2879086	NM_020342	chr2:196601344-196601403	SLC39A10
A_33_P3220919	6.3471212	4.967139	NM_005160	chr22:26125179-26125238	ADRBK2
A_32_P182439	9.69522	9.160014	NM_006591	chr11:74353632-74353691	POLD3
A_33_P3311956	8.078494	8.206033	NM_001042548	chr2:36782886-36782827	FEZ2
A_23_P113034	6.495709	6.55177	NM_032024	chr10:77807021-77818442	C10orf11

A_33_P3367830	9.809709	10.10964	NM_016938	chr11:65633975-65633916	EFEMP2
A_33_P3390625	2.354017	2.3900566	NR_002793	chr13:53126804-53145367	TPTE2P3
A_23_P115955	10.889472	9.701489	NM_181515	chr11:68660384-68658831	MRPL21
A_23_P122674	10.274292	10.029007	NM_017906	chr6:10709515-10709574	PAK1IP1
A_24_P419120	8.761313	8.983262	NM_015250	chr9:95474086-95474027	BICD2
A_24_P212481	7.4594517	7.997917	NM_024717	chr5:94208929-94208870	MCTP1
A_32_P783	14.744372	14.761112		chr2:217364080-217364688	RPL37A
A_24_P153043	14.172079	14.277659	NR_000029	chr2:114368843-114368816	RPL23AP7
A_23_P120860	9.181051	9.49333	NM_003634	chr22:29951282-29951223	NIPSNAP1
A_33_P3265075	8.545082	8.530695	NM_001267803	chr11:73638720-73638779	PAAF1
A_33_P3298980	7.1163282	7.032381		chr7:138734075-138734016	
A_24_P273413	11.019741	10.842691	NM_019063	chr2:42483688-42483747	EML4
A_23_P114826	10.936483	10.828398	NM_031280	chr1:36921953-36921894	MRPS15
A_23_P217737	5.038973	4.3617325	NM_000052	chrX:77305601-77305657	ATP7A
A_23_P153853	7.708973	8.109624	NM_001398	chr19:39321753-39308192	ECH1
A_23_P150857	5.5567527	5.849881	NM_000456	chr12:56398680-56398739	SUOX
A_33_P3416221	6.6341853	6.7021413	AK091719	chr2:240951285-240951226	NDUFA10
A_23_P103110	4.9651227	4.8049603	NM_012323	chr22:38611984-38612043	MAFF
A_33_P3389394	7.072112	7.1277933	NR_002312	chr14:20811297-20811238	RPPH1
A_23_P407206	6.2882233	6.2096224	NM_018941	chr8:1734642-1734701	CLN8
A_33_P3321836	6.6821275	6.7973614	NM_012401	chr22:50716342-50716283	PLXNB2
A_33_P3385266	6.1594706	6.6722155	NM_001079528	chr16:16315206-16315147	ABCC6
A_23_P110882	8.144496	7.8185167	NM_021648	chr6:116571248-116571189	TSPYL4
A_23_P138507	11.116045	11.028203	NM_001786	chr10:62552004-62553650	CDK1
A_23_P7761	7.4849095	6.772123	NM_018356	chr5:31554157-31554216	C5orf22
A_23_P306655	6.5450873	5.917074	NM_198722	chr3:49754603-49754544	AMIGO3
A_23_P115861	6.0719595	5.883601	NM_145312	chr10:44112506-44112565	ZNF485
A_33_P3279470	4.3653517	4.5775013	NM_001138	chr16:67516612-67516553	AGRP
A_23_P49988	4.976909	5.0557914	NM_033062	chr17:39333880-39333821	KRTAP4-2
A_33_P3214720	5.6994963	5.735465	NM_025079	chr1:37949708-37949767	ZC3H12A
A_24_P278367	8.874915	8.55826	NM_005736	chr10:104239489-104239430	ACTR1A
A_33_P3268567	10.534589	10.419801	NM_003581	chr2:106510646-106510705	NCK2
A_23_P104798	2.3875215	2.913951	NM_001562	chr11:112020870-112020811	IL18
A_23_P66664	4.924267	5.61018	NM_133439	chr17:35822207-35825575	TADA2A
A_32_P53558	10.037647	9.908209		chr3:098379592-098379651	
A_33_P3420247	4.545495	5.193159	NR_034157	chr9:93224953-93224894	LOC340515
A_33_P3235029	5.457038	5.2437596	DR731407	chr14:20908874-20908933	
A_23_P117037	8.241981	8.278156	NM_015416	chr12:51453755-51453814	LETMD1
A_33_P3254012	3.240689	4.6110897	NR_003594	chr8:86775512-86775453	REXO1L2P
A_23_P11331	10.157155	10.139798	NM_153333	chrX:102508057-102507998	TCEAL8
A_24_P252973	6.545926	6.9198503	NM_006831	chr11:57428642-57428701	CLP1
A_23_P141146	5.795319	6.2256885	NM_032875	chr17:37417070-37417011	FBXL20
A_24_P48069	4.9193535	5.3020725	NM_018110	chr16:57506193-57506134	DOK4
A_33_P3424794	6.378858	6.565402	AF354444	chrUn_gl000212:35529-35470	
A_23_P105900	11.863987	11.813187	NR_026983	chr13:77502690-77502749	BTF3P11
A_23_P103486	6.691474	6.475452	NM_000775	chr1:60359279-60359220	CYP2J2
A_24_P34545	5.215121	5.369791	NM_032329	chr2:242650818-242650877	ING5
A_33_P3386581	10.197037	9.803209	NM_052855	chr17:48770651-48770592	ANKRD40
A_33_P3370015	6.1004705	6.440217		chr10:071283234-071283175	
A_23_P106887	9.658953	9.986378	NM_004960	chr16:31201099-31201403	FUS
A_23_P208880	11.561938	11.548651	NM_013282	chr19:4962065-4962124	UHRF1
A_33_P3250680	4.110121	4.597598	NM_000074	chrX:135742456-135742515	CD40LG
A_23_P64954	4.9933352	4.8145785	NM_003481	chr12:6973900-6973959	USP5
A_23_P204702	8.601205	9.243197	NM_003217	chr12:50152262-50152523	TMBIM6
A_23_P4679	6.4269557	5.7548275	NM_006494	chr19:42752305-42752246	ERF
A_23_P353035	12.285617	12.606205	NM_001553	chr4:57897482-57897423	IGFBP7
A_32_P13795	6.332418	6.743329	NM_016544	chr2:25167079-25167020	DNAJC27
A_23_P149892	6.307554	6.5130186	NM_018590	chr10:43679826-43679885	CSGALNACT2
A_33_P3236591	7.4308624	7.672755	NM_001013838	chr16:67691397-67691456	RLTPR

A_23_P397969	8.185893	8.541131	NM_004514	chr17:80559860-80559919	FOXK2
A_23_P71530	7.622586	8.0497055	NM_002546	chr8:119936779-119936720	TNFRSF11B
A_32_P84728	3.2950256	4.2011547		chr4:186393318-186393377	
A_32_P230547	8.856534	9.113114	NM_033407	chr1:62939716-62939657	DOCK7
A_24_P56130	12.196877	12.433278	NM_079423	chr12:56553503-56553806	MYL6
A_23_P80940	8.132425	7.804492	NM_002703	chr4:57261568-57261509	PPAT
A_33_P3403867	7.9409647	7.2670283	NM_020182	chr20:56227153-56227094	PMEPA1
A_24_P251688	9.467321	9.672081	NM_018358	chr3:183911585-183911644	ABCF3
A_33_P3256760	4.152668	3.8655114	NR_033201	chr17:46683697-46683756	HOXB-AS3
A_23_P24234	4.6130066	5.165751	NM_001030015	chr10:88426117-88426176	OPN4
A_33_P3410194	7.4962716	5.910614	NM_005324	chr17:73772926-73772867	H3F3B
A_23_P96633	3.525743	4.6931086	NM_001031705	chrX:134290762-134290704	CXorf48
A_23_P83683	3.8485622	4.1552763	NM_014429	chr3:108677414-108677355	MORC1
A_23_P5325	7.4313664	7.5061064	NM_000122	chr2:128018872-128018813	ERCC3
A_33_P3343972	4.683078	5.266422	NM_080860	chr21:43892970-43892911	RSPH1
A_33_P3280801	6.264101	6.643715	NM_005358	chr13:76430684-76430743	LMO7
A_23_P45871	3.7092896	4.278018	NM_006820	chr1:79107590-79107649	IFI44L
A_23_P132526	7.766199	7.900264	NM_014602	chr3:130399467-130398281	PIK3R4
A_33_P3272698	10.710367	10.393127	NM_017823	chr1:159752201-159752260	DUSP23
A_23_P127684	4.641648	5.1592855	NM_003697	chr11:55761573-55761514	OR5F1
A_23_P208706	10.90935	10.928947	NM_138764	chr19:49464904-49464963	BAX
A_23_P380951	3.7480354	4.4541187	NM_144689	chr19:37619928-37619987	ZNF420
A_23_P129801	5.742934	6.8168826	NM_006822	chr17:80615575-80615516	RAB40B
A_23_P121222	7.295928	6.7564597	NM_020165	chr3:8921693-8921634	RAD18
A_23_P337270	4.6887693	5.2963486	XM_005265644	chr3:27152608-27152549	NEK10
A_23_P102060	9.987243	10.046393	NM_006751	chr2:182795035-182795094	SSFA2
A_23_P375549	8.199578	8.052155	NM_173794	chrX:44397762-44386602	FUNDC1
A_33_P3239839	9.533902	9.137922	NM_024963	chr7:5515499-5515440	FBXL18
A_23_P83438	12.29028	12.352022	NM_023079	chr17:47006292-47006351	UBE2Z
A_33_P3252491	9.253901	9.429392		chrX:040767312-040767371	
A_32_P126698	4.9608455	5.0607805	NM_001284269	chr14:90262091-90262032	EFCAB11
A_23_P258310	8.68032	8.269371	NM_144651	chr8:52232460-52232401	PXDNL
A_23_P110430	5.4352264	4.8068776	NM_002448	chr4:4865044-4865103	MSX1
A_33_P3212704	6.4339104	6.787264		chr19:058152352-058152293	
A_33_P3221489	7.979669	7.9373684	NM_018240	chr1:158065293-158065352	KIRREL
A_23_P148047	6.007289	6.298681	NM_000958	chr5:40693563-40693622	PTGER4
A_23_P90099	10.575333	10.990342	NM_198536	chr19:11453529-11453470	TMEM205
A_33_P3317593	11.514585	11.431446	NM_001127	chr22:29723913-29723854	AP1B1
A_23_P49279	6.4614286	6.372274	NM_001001436	chr16:46836784-46836725	C16orf87
A_33_P3346806	4.069604	4.1104813	AK092544	chr8:12436191-12436250	LOC100131581
A_23_P160809	8.755114	8.905253	NM_007357	chr1:230829207-230829266	COG2
A_23_P256956	9.584475	10.041447	NM_005733	chr5:137522081-137522804	KIF20A
A_24_P246467	6.29665	6.766816	NM_001880	chr2:175939522-175939463	ATF2
A_24_P187948	11.054829	10.872307	NM_197966	chr22:18218241-18218182	BID
A_23_P56127	5.434559	5.200999	NM_019108	chr19:44237539-44237480	SMG9
A_33_P3394828	9.260273	8.951388	NM_024078	chr12:132636912-132636971	NOC4L
A_23_P12329	7.138208	6.817392	NM_016022	chr1:150238325-150238266	APH1A
A_23_P105833	8.314498	8.412592	NM_017693	chr13:103492701-103492760	BIVM
A_33_P3370944	4.50456	4.9814086	NM_013435	chr18:56934326-56934267	RAX
A_23_P117734	6.8397365	6.913615	NM_152449	chr15:100267828-100267769	LYSMD4
A_32_P170003	8.703567	8.321959	NM_015650	chr2:239309319-239309378	TRAF3IP1
A_23_P120883	12.287416	11.954972	NM_002133	chr22:35790122-35790181	HMOX1
A_23_P336565	4.609335	5.1355166	NM_001145268	chr7:102427831-102427890	FAM185A
A_33_P3257367	7.7815228	7.3526626	NM_015906	chr1:114935511-114935452	TRIM33
A_23_P340251	10.343465	10.651667	NM_002865	chr8:61504465-61504524	RAB2A
A_23_P18579	9.211948	9.45035	NM_006607	chr4:37962342-37962401	PTTG2
A_33_P3330353	5.5083537	5.4199696	NM_019556	chrX:134023212-134023153	MOSPD1
A_33_P3263614	4.2642035	4.415555	NM_001143778	chr1:23779252-23779193	ASAP3
A_33_P3221343	6.7344604	6.813891	NM_173497	chr10:93222889-93222948	HECTD2

A_23_P208325	5.59987	5.7964826	NM_004234	chr19:44791103-44791044	ZNF235
A_24_P143574	5.318757	5.4103928	NM_006065	chr20:1552568-1552509	SIRPB1
A_23_P122052	10.545507	10.610851	NM_001008397	chr5:54460254-54460313	GPX8
A_32_P50066	7.156895	5.8990884	NM_001039580	chr4:156263949-156263890	MAP9
A_33_P3365097	7.2493377	7.3186474	NM_001184765	chr1:86814480-86814421	ODF2L
A_23_P215744	6.5824223	6.531816	NM_033427	chr7:117351142-117351083	CTTNBP2
A_24_P203056	7.7967725	8.474438	NM_020993	chr12:122499758-122499817	BCL7A
A_23_P218608	9.490384	9.391392	NM_015904	chr2:100015969-100016028	EIF5B
A_23_P116624	3.9160337	3.621534	NM_004211	chr11:20673953-20676268	SLC6A5
A_23_P120845	9.515909	9.283689	NM_005080	chr22:29190792-29190733	XBP1
A_32_P224522	7.5402184	7.677927	NM_024103	chr19:6440285-6440226	SLC25A23
A_23_P214977	9.743678	9.905243	NM_007214	chr6:108192374-108192315	SEC63
A_33_P3347193	6.147024	6.4279485	NM_182546	chr7:54617657-54617716	VSTM2A
A_33_P3327592	9.781462	9.637704	NM_007282	chr3:149679866-149679925	RNF13
A_24_P163537	7.8722267	8.204115	NM_182547	chr7:44620742-44619219	TMED4
A_32_P182511	4.8104987	5.056625	NM_020947	chr16:84510344-84510285	TLDC1
A_33_P3257628	5.3704867	5.323536	NM_001195139	chr16:70543214-70543155	COG4
A_23_P18739	7.059093	7.247345	NM_018241	chr4:148555766-148555825	TMEM184C
A_24_P386622	4.7670393	4.7809567	NM_004041	chr11:74978736-74977300	ARRB1
A_33_P3362631	7.5879416	7.672366	NM_021927	chr4:44702403-44702462	GUF1
A_24_P382630	7.236638	7.187898	NM_015666	chr20:60777313-60777372	MTG2
A_33_P3270315	4.429224	5.1027184	BC009492	chr12:129298767-129298728	
A_33_P3307955	8.659407	8.859724	NM_001122674	chr1:94944201-94944260	ABCD3
A_33_P3876591	5.218555	5.6588993	CD050382		SNORA76
A_23_P101237	8.337454	8.461521	NM_031446	chr18:33557460-33557519	C18orf21
A_24_P389608	4.9370356	5.221525	NM_153256	chr10:11913563-11913622	PROSER2
A_33_P3388958	5.883762	6.220872	NM_152760	chr11:65621110-65621169	SNX32
A_23_P78750	6.795055	6.561648	NM_020309	chr19:49933575-49933516	SLC17A7
A_33_P3422265	5.824388	6.4185367		chr11:134606350-134606409	LOC729305
A_23_P29036	8.375738	8.830338	NM_005534	chr21:34809222-34809281	IFNGR2
A_24_P58187	8.358639	8.953343		chr11:110659013-110658954	
A_23_P503127	5.697221	5.511244	NM_145733	chr22:42393420-42393479	SEPT3
A_33_P3413038	7.001685	6.9355702	NM_001163257	chrX:153044726-153044785	PLXNB3
A_24_P928604	11.879787	11.899172		chr1:033445625-033445684	
A_23_P150325	6.751632	6.5139465	NM_032021	chr11:100864382-100864441	TMEM133
A_33_P3216483	7.2497854	7.425636	XR_110541	chr11:111327269-111327328	LOC644277
A_24_P218074	4.930252	5.1103573	NM_207336	chr7:149462406-149462347	ZNF467
A_32_P149492	7.211545	6.993589	NM_183372	chr1:146032638-146032579	NBPF11
A_33_P3357879	4.030805	4.07944		chr10:133274691-133274632	
A_33_P3249489	7.285084	7.084009	NM_001258272	chr14:64065387-64065328	WDR89
A_33_P3299599	7.962306	7.59466	NM_001166175	chr5:172659197-172659138	NKX2-5
A_33_P3357753	7.694184	7.4508257	NR_024397	chr10:88998510-88998451	NUTM2A-AS1
A_24_P386323	10.744322	10.18659	NM_005833	chr9:127996052-127996111	RABEPK
A_23_P30956	8.158207	8.054075	NM_015323	chr6:97002960-97003019	UFL1
A_33_P3659876	8.875906	9.165677	NM_001281932	chr7:158437043-158436984	NCAPG2
A_33_P3384442	15.3013	15.357016	BC015386	chr20:60920615-60920556	LAMA5
A_23_P140527	6.129348	6.385237	NM_012182	chr15:60297908-60297967	FOXB1
A_23_P2294	5.76822	6.127284	NM_033647	chr12:66725393-66731807	HELB
A_24_P182764	8.539679	8.247118	NM_178326	chr2:242613194-242613253	ATG4B
A_33_P3702055	6.268946	6.5489855	NM_000578	chr2:219259681-219259740	SLC11A1
A_24_P210513	5.586828	6.1811056	NM_018167	chr14:93760281-93760222	BTBD7
A_23_P341443	9.566481	9.879256	NM_020310	chr17:2287635-2287576	MNT
A_33_P3371785	9.271452	9.391754	NM_001128175	chr18:32407580-32407639	DTNA
A_23_P42829	12.2197	12.265369	NM_014390	chr7:127732513-127732572	SND1
A_33_P3267627	3.9953353	4.365801	NM_001037729	chr6:49936452-49936393	DEFB113
A_24_P117029	10.267225	10.266063	NM_000527	chr19:11244293-11244352	LDLR
A_23_P345808	2.3221061	2.3900566	NM_173698	chrX:92966744-92966803	FAM133A
A_23_P122464	6.371586	6.405402	NM_006299	chr6:28200866-28200925	ZSCAN9
A_33_P3362611	6.4134684	6.1503167	NM_022159	chr1:79355513-79355454	ELTD1

A_23_P209538	5.043878	5.5962615	NM_001115016	chr2:97260032-97259973	KANSL3
A_32_P22078	11.228376	11.248156	NM_001013	chr19:54705133-54705397	RPS9
A_33_P3340385	3.0661922	3.646058	NM_001256648	chr19:21987813-21987754	ZNF43
A_23_P82950	9.671054	9.808617	NM_006197	chr8:17872304-17882883	PCM1
A_33_P3279181	5.0178003	5.3898025		chr1:148340545-148340486	
A_24_P127159	6.2580824	5.706334	M69012	chr7:65970933-65970874	
A_23_P45496	9.840884	9.766874	NM_001493	chrX:153671602-153671661	GDI1
A_24_P374973	5.602715	5.572234		chr2:203905122-203905181	
A_33_P3256033	9.290078	9.165012	NM_005188	chr11:119178466-119178525	CBL
A_33_P3241433	2.398368	3.0762486	NM_001172674	chr19:53643632-53643573	ZNF347
A_33_P3366102	7.1477785	7.455567	NM_001172428	chr7:23164703-23164762	KLHL7
A_23_P31109	9.934178	10.333681	NM_138785	chr6:149911979-149912038	GINM1
A_23_P102694	6.7336273	6.631346	NM_080831	chr20:210130-210189	DEFB129
A_23_P131240	8.0791445	8.280425	NM_181713	chr2:24222677-24222736	UBXN2A
A_23_P48056	10.730089	10.394686	NM_006825	chr12:106632531-106632472	CKAP4
A_23_P110433	9.104214	9.085695	NM_015342	chr5:64881994-64883124	PPWD1
A_23_P1280	7.286814	7.489407	NM_001198799	chr10:73857061-73857002	ASCC1
A_23_P14804	11.5070505	11.462726	NM_005724	chr15:77338573-77338514	TSPAN3
A_33_P3294133	12.228779	12.258757	NM_001417	chr12:53434050-53434110	EIF4B
A_23_P122041	10.523773	10.770266	NM_002715	chr5:133533156-133533097	PPP2CA
A_33_P3310751	4.5822544	4.4253607		chr9:045352157-045352098	
A_23_P122796	6.21848	6.1525927	NM_016063	chr6:125596785-125596726	HDDC2
A_23_P327022	10.1431	9.993919	NM_199072	chr7:114658631-114658690	MDFIC
A_23_P92320	10.104904	9.99128	NM_017426	chr4:77036115-77036056	NUP54
A_24_P110558	5.6637607	5.1784678	NM_001007189	chr5:139508041-139508100	IGIP
A_23_P103617	3.6161575	3.3062353	NM_003568	chr1:150967943-150968002	ANXA9
A_24_P67806	6.3444753	5.9707294	NM_000254	chr1:237066633-237066692	MTR
A_24_P248863	8.618015	8.444057	NM_024936	chr4:25371551-25371610	ZCCHC4
A_23_P318300	9.868448	9.036919	NM_133646	chr2:174091756-174091815	ZAK
A_23_P368645	8.130319	8.301751	NM_003659	chr2:178402806-178402865	AGPS
A_24_P83738	10.164182	10.4509535	NM_198188	chr9:119380715-119380656	ASTN2
A_33_P3209356	8.403543	8.442746	NM_022457	chr1:175956140-175956081	RFWD2
A_33_P3277323	15.442646	15.393423		chr4:165841855-165841796	
A_32_P16315	5.054326	4.856745	NM_177987	chr10:92952-92893	TUBB8
A_23_P416395	6.983492	6.0482483	NM_003714	chr5:172742046-172741987	STC2
A_23_P55174	5.047087	5.472524	NM_138387	chr17:42151599-42152071	G6PC3
A_23_P44505	6.424517	7.2493377	NM_003597	chr2:10194646-10194705	KLF11
A_33_P3254606	10.126242	10.1153	NM_001083961	chr19:36595952-36596011	WDR62
A_23_P256903	7.3320637	7.306185	AF086546	chrX:135930362-135930421	
A_24_P122337	5.706742	5.997055	NM_080737	chrX:99929857-99929798	SYTL4
A_23_P95165	5.4492664	5.6394234	NM_020210	chr15:90771532-90771591	SEMA4B
A_33_P3306272	5.8123407	6.193036	NM_001145678	chr5:93489771-93489712	KIAA0825
A_23_P147431	9.720337	9.938884	NM_002350	chr8:56922829-56922888	LYN
A_23_P108932	5.9682803	6.056545	NR_002229	chr2:54756368-54756427	RPL23AP32
A_23_P215406	10.228036	10.560978	NM_018890	chr7:6443013-6443072	RAC1
A_33_P3389478	4.3538404	5.1282563		chr9:15453352-15453411	SNAPC3
A_23_P102471	9.965945	9.966324	NM_000251	chr2:47710161-47710220	MSH2
A_24_P414256	11.915518	12.065742	AK126594	chr3:48485552-48485611	TMA7
A_23_P35576	4.0107465	4.198723	NM_014803	chr10:97920015-97920074	ZNF518A
A_23_P71316	9.327567	9.788723	NM_001008712	chr8:30335321-30336857	RBPMS
A_33_P3413483	6.1048865	5.640476	AK090738	chr15:45369321-45369380	SORD
A_24_P364838	5.9959416	6.2446804	NM_004785	chr16:2088290-2088349	SLC9A3R2
A_33_P3245922	8.627237	8.449537	NM_006724	chr6:161455421-161455480	MAP3K4
A_33_P3263277	14.72209	14.828998	M29548	chr6:74227383-74227324	EEF1A1
A_23_P104201	10.984541	10.880321	NM_139312	chr10:27400808-27400749	YME1L1
A_33_P3397801	10.43513	10.669281		chr18:53707878-53707819	FLJ45743
A_33_P3306342	4.280023	4.9489975	NM_001001922	chr11:5798991-5798932	OR52N5
A_23_P114445	5.9112034	5.6078753	NM_020932	chrX:75651239-75651298	MAGEE1
A_23_P11397	4.1831565	4.6547594		chrY:23749523-23749464	TTTY13

A_33_P3293237	3.7995932	4.5325503		chr8:70762583-70762642	
A_33_P3386775	5.4281464	5.549203	NM_002390	chr17:42857869-42857928	ADAM11
A_23_P40782	6.7580476	7.126709	NM_016094	chr3:149469205-149468595	COMMD2
A_33_P3410296	9.6598015	10.000362	NM_006426	chr10:134018522-134018581	DPYSL4
A_33_P3671378	5.275075	5.5754776	NM_016174	chr9:131196441-131196500	CERCAM
A_33_P3245699	6.202997	6.342806	XR_112969	chr7:1878356-1878415	LOC100128374
A_33_P3345504	5.6541123	5.595624	NM_018835	chr9:125639810-125639751	RC3H2
A_23_P157147	9.790903	9.790602	NM_018106	chr7:6628473-6628532	ZDHHC4
A_24_P135483	4.310754	4.795478	NM_030927	chr10:82280514-82280573	TSPAN14
A_23_P312610	5.5429554	5.3773136	BC010145	chr11:63999402-64001390	DNAJC4
A_23_P59677	8.05346	7.907851	NM_021930	chr7:105207999-105208058	RINT1
A_24_P216681	8.220393	8.037224	NM_032881	chr1:36859567-36859508	LSM10
A_23_P106798	6.2046285	6.8991084	NM_003031	chr16:48395600-48395541	SIAH1
A_33_P3413741	4.6724215	4.9482856	NM_000916	chr3:8792187-8792128	OXTR
A_33_P3398513	5.072648	5.2418547	NM_001101330	chr2:43902476-43902417	LOC728819
A_24_P314534	4.4652786	4.870277	NM_181621	chr21:31744070-31744011	KRTAP13-2
A_23_P354175	8.6177025	8.508512	NM_138385	chr4:1717772-1717713	TMEM129
A_23_P40108	5.1171055	5.6157646	NM_001853	chr20:61472271-61472330	COL9A3
A_24_P274270	8.377487	9.090264	NM_139266	chr2:191841733-191841674	STAT1
A_33_P3242416	4.841236	5.2913194		chr1:223738794-223738735	
A_23_P427502	5.8176785	5.8219852	NM_001206998	chr22:29452861-29452920	ZNRF3
A_23_P256473	8.616576	7.860559	NM_006379	chr7:80372471-80372412	SEMA3C
A_23_P40956	3.670609	2.9567783	NM_016362	chr3:10327552-10327493	GHRL
A_23_P53788	8.132593	8.0739155	NM_152912	chr13:28010017-28009958	MTIF3
A_33_P3377399	4.1787453	4.8181047		chr8:106810426-106810367	
A_33_P3373745	6.4874387	6.526367	NM_014299	chr19:15365064-15365005	BRD4
A_23_P110403	2.3221061	2.3900566	NM_014476	chr4:186423262-186423203	PDLIM3
A_23_P47148	2.3221061	3.2619832	NM_016931	chr11:89059885-89059826	NOX4
A_24_P580698	4.7900653	4.914469	NM_001271560	chrX:139174919-139174978	LOC389895
A_23_P126757	7.859126	7.6485343	NM_023015	chr1:153746191-153746250	INTS3
A_23_P55376	8.494968	8.450336	NM_015443	chr17:44107406-44107347	KANSL1
A_33_P3238295	4.4018345	4.709425	NR_026788	chr12:8353386-8353445	FAM66C
A_33_P3291559	6.9859056	7.477378	AK022341	chr7:151826776-151826835	
A_24_P128205	10.901456	10.995993	NM_002807	chr2:232011055-232018321	PSMD1
A_33_P3406255	6.766621	7.09581		chr12:042876894-042876953	
A_33_P3414312	7.732954	8.099182	NM_004505	chr17:5074130-5074189	USP6
A_32_P211248	5.766877	6.6569138	NR_036513	chr12:111375193-111375252	LOC100131138
A_23_P164047	10.4621525	10.459755	NM_012329	chr17:53470223-53470164	MMD
A_33_P3366053	4.3892045	4.1843734	NM_001125	chr3:119308728-119308787	ADPRH
A_33_P3372090	4.626846	4.508873		chr5:180412991-180413050	
A_33_P3300273	5.8824434	5.951279	NM_198077	chr1:85715907-85715848	C1orf52
A_33_P3269718	9.848006	9.864635	NM_001159508	chr15:40710974-40711033	IVD
A_23_P126716	12.324432	12.456548	NM_178191	chr1:28564499-28564558	ATPIF1
A_33_P3360753	6.35105	6.3638177	AK293727	chr20:62055455-62055396	KCNQ2
A_23_P329573	6.419443	6.0808206	NM_000211	chr21:46305985-46305926	ITGB2
A_24_P196499	4.803259	5.0657177	AB030181	chr1:235344269-235344210	ARID4B
A_23_P103276	7.862271	7.6339936	NM_138417	chr1:52498019-52497960	KTI12
A_23_P111303	9.624956	9.63386	NM_014892	chr6:155154867-155154926	SCAF8
A_33_P3327799	11.058315	10.703902		chr18:000856842-000856901	
A_33_P3545065	8.426781	8.506512	NR_033412	chr17:30369620-30369679	SH3GL1P1
A_24_P194420	4.68926	4.7392197	NM_024821	chr22:42209346-42209405	CCDC134
A_23_P118888	8.982283	9.23577	NM_000430	chr17:2583481-2583540	PAFAH1B1
A_33_P3378435	3.9604492	3.9113586		chr12:123850487-123850546	LOC100293704
A_32_P75299	12.549358	12.427046	NM_001134484	chr9:37588890-37588831	TOMM5
A_23_P138680	5.4211216	5.360458	NM_172200	chr10:5994720-5994661	IL15RA
A_33_P3407540	4.920019	5.335009	AK098253	chrX:13733706-13733647	TRAPPC2
A_33_P3287105	5.092864	5.8343306		chr1:161209136-161209077	
A_33_P3228300	8.51833	8.481804	NM_005772	chr9:4860233-4860292	RCL1
A_23_P146050	9.399793	9.56476	NM_024699	chr8:82614467-82614408	ZFAND1

A_23_P72059	7.418249	7.3883557	NM_019086	chr12:118501609-118501550	VSIG10
A_23_P112452	3.821013	3.5703301	NR_003191	chr9:124217497-124217438	GGTA1P
A_33_P3342156	4.0243917	4.344548	AB305786	chr14:22695063-22695122	
A_23_P122947	6.025404	6.718322	NM_015060	chr7:32619842-32620430	AVL9
A_33_P3305810	4.079998	4.3381405	NM_020702	chr9:34372125-34372066	KIAA1161
A_33_P3348574	4.7169747	5.1067867	NM_001013358		OR9G9
A_23_P80832	6.855128	6.8080263	NM_080652	chr3:185209359-185209300	TMEM41A
A_33_P3378334	8.990627	8.947336	NM_001042550	chr9:106896796-106896855	SMC2
A_23_P163467	11.058243	10.473253	NM_207380	chr15:40623743-40623684	C15orf52
A_23_P59179	6.4713526	6.57219	NM_021976	chr6:33162245-33162186	RXRB
A_33_P3363082	3.909959	4.516325	NR_003008	chr2:234184514-234184573	SCARNA5
A_23_P120194	9.76585	9.888712	NM_016467	chr2:190636267-190636208	ORMDL1
A_32_P203515	9.488208	9.291588	NM_015330	chr22:24812928-24812987	SPECC1L
A_23_P103720	7.058788	6.538715	NM_024758	chr1:15900155-15900096	AGMAT
A_23_P14157	8.903873	9.217501	NM_198968	chr13:96234481-96234422	DZIP1
A_23_P130020	10.170838	10.018127	NM_016001	chr17:49362719-49365438	UTP18
A_33_P3225696	15.130064	15.14681	NM_133444	chr19:42731919-42731978	ZNF526
A_33_P3300308	6.42418	6.375584	NM_032514	chr20:33147644-33147703	MAP1LC3A
A_33_P3308050	10.989109	11.39463	NM_004846	chr2:233431864-233431923	EIF4E2
A_23_P255714	6.561648	6.7914705	NM_025103	chr9:27056396-27056455	IFT74
A_33_P3280044	11.452857	11.30285	NM_001256182	chr16:89351907-89351848	ANKRD11
A_23_P213661	9.649359	9.747165	NM_015216	chr5:102538799-102538858	PPIP5K2
A_24_P522631	9.952806	9.577155	NM_001130924	chr1:9674658-9674716	TMEM201
A_23_P398491	6.7634325	6.6854134	NR_002208	chr15:40824156-40824097	MRPL42P5
A_24_P213643	6.155717	6.5388017	NM_031945	chr17:79612546-79612605	TSPAN10
A_33_P3404744	5.3335114	5.571997	NM_133465	chr9:115422644-115422703	KIAA1958
A_24_P324405	11.580425	11.346262	NM_013275	chr16:89349843-89349784	ANKRD11
A_23_P93009	4.9281816	4.5853877	NM_001204199	chr5:112227745-112227804	SRP19
A_33_P3320301	10.42384	10.329317	NR_015432	chr20:18550098-18550157	LINC00493
A_33_P3289005	5.1485567	5.483524	NM_001039569	chr2:224629990-224629931	AP1S3
A_23_P111865	2.9192019	3.4716008	NM_145914	chr7:99661851-99661910	ZSCAN21
A_23_P17914	10.198396	9.613512	NM_025225	chr22:44342759-44342818	PNPLA3
A_23_P5757	10.249646	9.975906	NM_016058	chr2:73957755-73957696	TPRKB
A_23_P130585	4.2477813	4.725143	NM_054113	chr19:16275614-16275555	CIB3
A_23_P100189	4.788579	4.819787	NM_002761	chr16:11374862-11374803	PRM1
A_33_P3239195	9.919792	10.034136		chr10:126855412-126855471	
A_33_P3357087	10.463749	10.595984	NM_002437	chr2:27532421-27532362	MPV17
A_33_P3322654	11.046217	11.223735	NM_018226	chr2:241518082-241518141	RNPEPL1
A_23_P125624	9.2895565	9.158234	NM_001037171	chrX:23721956-23721897	ACOT9
A_33_P3393106	4.918062	4.663101	NM_024948	chr10:15858894-15858835	FAM188A
A_23_P108492	3.6940675	3.367062	NM_138395	chr2:198571975-198572034	MARS2
A_33_P3224281	3.9537294	4.6612267		chr4:069375330-069375271	
A_32_P201496	6.586898	6.52901	NM_001018052	chr22:41936782-41936723	POLR3H
A_23_P253484	7.9812117	7.716818	NM_016228	chr4:170981876-170981817	AADAT
A_33_P3386995	4.8586864	4.901466	NM_015914	chr16:11824561-11824502	TXNDC11
A_33_P3354539	5.1004696	4.6027937	NM_145165	chr14:65401707-65401766	CHURC1
A_32_P107493	4.4044957	4.543372	AK056168	chr16:89737094-89737153	SPATA33
A_23_P118834	10.843325	11.078617	NM_001067	chr17:38545768-38545709	TOP2A
A_23_P164258	5.9755073	6.211321	NM_016518	chr17:27383435-27383494	PIPOX
A_33_P3277328	3.821889	4.358451	NR_037167	chr1:177991769-177991710	LOC730102
A_23_P109821	10.9461355	10.891301	NM_006354	chr3:9821731-9821672	TADA3
A_23_P308603	6.734207	6.2240133	NM_005417	chr20:36033496-36033555	SRC
A_32_P51084	10.627693	10.620754	NM_015135	chr7:135333302-135333361	NUP205
A_32_P129288	8.431232	8.706441	NM_030981	chr11:66044665-66044724	RAB1B
A_23_P121702	11.035284	10.686781	NM_001014446	chr4:48887525-48887466	OCIAD2
A_33_P3360456	2.8097553	3.7548242	NM_001085384	chr19:58213458-58213399	ZNF154
A_23_P105307	5.4886947	5.5159307	NM_201444	chr12:56346854-56346913	DGKA
A_24_P103686	5.6553197	5.7479324	NM_178819	chr8:41476425-41476484	AGPAT6
A_23_P43273	10.106634	10.353097	NM_000127	chr8:118819498-118817116	EXT1

A_33_P3264731	3.990366	4.792497		chr1:228699703-228699644	
A_24_P942354	9.873704	9.766432	NM_006224	chr17:1421436-1421377	PITPNA
A_23_P106158	5.31244	5.280031	NM_024884	chr14:50734549-50734490	L2HGDH
A_23_P408095	12.113966	12.250114	NM_001011546	chr20:17588567-17588626	DSTN
A_33_P3238323	5.189941	5.1173153	NM_031229	chr20:408036-408095	RBCK1
A_33_P3358750	3.9781132	4.906703	NM_001010985	chr1:109835046-109834987	MYBPHL
A_23_P214821	4.840287	4.7548704	NM_001955	chr6:12296672-12296731	EDN1
A_23_P138168	11.82115	11.796828	NM_001839	chr1:95363319-95363260	CNN3
A_33_P3259890	4.5447617	5.1014047	NR_033932	chr5:98106843-98106784	RGMB-AS1
A_23_P209337	8.462435	8.68032	NM_145280	chr2:208477667-208477608	METTL21A
A_24_P235305	5.616593	5.092003	NM_001042510	chr8:102209672-102209613	ZNF706
A_23_P385105	5.2747936	3.8650916	NM_032726	chr2:219501765-219501824	PLCD4
A_33_P3392517	5.9711065	5.5918474	NM_001009812	chr2:74724708-74724649	LBX2
A_23_P208835	7.13004	6.8149586	NM_030662	chr19:4097325-4095437	MAP2K2
A_33_P3229452	5.6843305	6.0258646	AK126441	chr4:143768284-143768343	FLJ44477
A_23_P133691	5.692933	6.16293	NM_021244	chr6:90087437-90082293	RRAGD
A_24_P194931	7.99166	8.125347	NM_020463	chr2:55776250-55776191	SMEK2
A_23_P315836	6.424416	6.220217	NM_017451	chr17:79091053-79091112	BAIAP2
A_33_P3377199	14.563592	14.478864	NM_002574	chr1:45976799-45976740	PRDX1
A_23_P398637	6.1534653	5.8484354	NM_152292	chr4:100468125-100468066	TRMT10A
A_24_P153576	7.7702584	7.531129	NM_173082	chr6:146266660-146266601	SHPRH
A_33_P3238521	10.197508	10.398786		chr7:63227848-63227789	XLOC_014512
A_23_P59630	7.0416303	6.912079	BC101598	chr7:75145092-75144095	PMS2P3
A_23_P380998	10.02828	9.896716	NM_015361	chr2:136482502-136482561	R3HDM1
A_33_P3388651	6.647763	6.8527513	NM_001003408	chr10:116196035-116195976	ABLIM1
A_33_P3303602	4.595816	4.67688	AK123378	chr11:46389751-46389810	DGKZ
A_33_P3246885	7.5662603	7.827514	NM_001190348	chr19:35994272-35994213	DMKN
A_24_P15765	13.335219	13.342301		chr1:240176168-240176229	RPS7P5
A_23_P156907	4.9448967	4.666358	NM_018013	chr6:107980433-107980492	SOBP
A_23_P53276	10.9747305	11.006214	NM_003920	chr12:56811004-56810945	TIMELESS
A_24_P910060	7.059537	7.068386		chr9:019200632-019200573	
A_24_P251962	6.8961635	6.666526	NM_018936	chr5:140476664-140476723	PCDHB2
A_33_P3291510	5.978769	6.0705853	NM_004679	chrY:16168702-16168761	VCY
A_33_P3318343	3.3860488	3.997396	NM_020994	chrX:153880477-153880418	CTAG2
A_24_P139620	5.814802	5.767167	NM_001014443	chr1:161134334-161134393	USP21
A_23_P339240	6.196733	6.627893	NM_014996	chr3:155197964-155197905	PLCH1
A_33_P3365200	3.7886894	4.5124564		chr10:111702479-111702420	
A_33_P3357573	4.6752253	4.4440975	NM_018717	chr4:140810806-140810747	MAML3
A_33_P3404286	9.564299	9.830259	NM_001080398	chr9:114132775-114132716	KIAA0368
A_33_P3335498	3.6593246	4.4385085	NM_001159296	chrX:56291992-56292051	KLF8
A_33_P3211078	6.254555	6.446855	NM_003484	chr12:66219101-66219160	HMGA2
A_24_P161293	11.727966	11.69906	XM_005272558	chr1:144201845-144201904	
A_23_P400235	8.243563	8.489331	NM_000255	chr6:49399349-49399290	MUT
A_24_P330822	5.337838	5.5652566	NM_000458	chr17:36059175-36059116	HNF1B
A_33_P3304107	5.576347	5.237066	NM_017658	chr14:45398109-45398050	KLHL28
A_23_P164284	3.3482437	3.9143891	NM_001307	chr17:7163986-7163822	CLDN7
A_23_P11598	5.1831064	5.001174	NM_152373	chr1:41012893-41012952	ZNF684
A_32_P227317	5.4107275	5.5731697	NR_024004	chr2:114357599-114357540	DDX11L2
A_24_P88801	5.14113	5.447977	NM_000272	chr2:110881447-110881388	NPHP1
A_33_P3228435	7.3153296	7.477811	NM_001278717	chr19:35630984-35631043	FXYD1
A_23_P105535	6.515929	6.207474	NM_172240	chr12:89814106-89814047	POC1B
A_33_P3238325	4.4477634	4.4245515		chr9:140074118-140074059	
A_33_P3279851	4.451868	4.7344704	NR_037931	chr2:87120433-87120492	ANAPC1P1
A_23_P102262	11.348846	11.242524	NM_014763	chr2:75882659-75882718	MRPL19
A_23_P118741	4.358273	4.4807568	NM_201566	chr17:6942119-6942178	SLC16A13
A_33_P3362371	8.074771	8.499909	NM_001265591	chr11:63449006-63449065	RTN3
A_24_P349274	4.412528	4.3631735	NM_001004727	chr11:48267082-48267141	OR4X2
A_23_P146367	11.413149	11.257251	NM_032310	chr9:95875446-95875505	C9orf89
A_24_P141019	4.425459	4.8987794	NM_018412	chr7:116829387-116829446	ST7

A_23_P92161	3.7154899	3.6174834	NM_025047	chr3:160395648-160395707	ARL14
A_23_P112135	12.082267	12.430465	NM_014294	chr8:71485966-71485907	TRAM1
A_23_P114466	7.126709	7.450178	NM_033284	chrY:6955393-6955452	TBL1Y
A_23_P428219	5.347296	5.6599035	NM_001991	chr17:40852733-40852674	EZH1
A_23_P116512	7.3594065	6.8484516	NM_024841	chr11:36486438-36486497	PRR5L
A_23_P502750	6.261287	5.9204025	NM_002392	chr12:69233822-69233881	MDM2
A_23_P406385	2.3221061	2.3900566	NM_153350	chr16:742597-742538	FBXL16
A_23_P67529	9.106262	8.764688	NM_002250	chr19:44270995-44270936	KCNN4
A_33_P3381613	4.731693	4.7364006		chr7:064328916-064328857	
A_33_P3266923	4.8326592	5.1673365	NM_014916	chr7:97835444-97835503	LMTK2
A_23_P70148	8.279307	8.715376	NM_182977	chr5:43704546-43704605	NNT
A_33_P3317211	4.0490775	3.6705103	NM_001110792	chrX:153297718-153297659	MECP2
A_23_P65262	6.8121147	7.011495	NM_001278432	chr13:33016630-33016571	N4BP2L2
A_23_P364544	5.337511	4.9726243	NM_175874	chr12:14976513-14976572	C12orf60
A_23_P75288	9.543051	9.39014	NM_032810	chr10:89513058-89512999	ATAD1
A_23_P384857	6.0106654	6.322032		chr6:027932965-027933024	
A_23_P110571	6.2850127	6.0415688	NM_001164664	chr5:66462808-66462867	MAST4
A_33_P3253596	5.9193153	6.6378703	NM_012310	chrX:69626140-69626199	KIF4A
A_23_P401106	5.831026	4.9878078	NM_002599	chr11:72287309-72287250	PDE2A
A_23_P67702	10.423117	10.699971	NM_003429	chr19:21132382-21132441	ZNF85
A_24_P274615	7.71321	8.797267	NM_020801	chr5:90664749-90664690	ARRDC3
A_23_P118289	8.382776	8.467059	NM_004765	chr16:30904169-30904018	BCL7C
A_23_P35444	5.714733	5.821801	NM_032727	chr10:105049985-105050044	INA
A_24_P106357	7.2002172	6.912958	NM_139281	chr5:110465700-110465759	WDR36
A_33_P3397150	5.4567804	5.759205	NM_001190467	chr19:7933666-7933607	FLJ22184
A_23_P3312	5.976105	6.166003	NM_005545	chr15:74469121-74469180	ISLR
A_23_P29257	3.275464	3.7654839	NM_005318	chr22:38203147-38203206	H1F0
A_23_P38677	6.503394	6.0401382	NM_006553	chr18:12432141-12432200	SLMO1
A_33_P3734384	6.5538526	6.8428845	AK097526	chr7:44998779-44998720	LOC285957
A_33_P3224423	5.5118265	5.92906	NM_013284	chr7:44112870-44112811	POLM
A_23_P103601	3.496038	4.8092394	NM_020379	chr1:26110835-26110894	MAN1C1
A_33_P3299130	3.63555	4.260125		chr9:136877785-136877844	
A_23_P6943	4.006128	4.0667005	NM_005290	chr3:98251643-98251702	GPR15
A_23_P67771	8.059565	8.070779	NM_000465	chr2:215593719-215593660	BARD1
A_33_P3362696	6.8197575	6.7680387	NM_018157	chr12:107283012-107283071	RIC8B
A_23_P5441	10.327527	10.8009615	NM_005689	chr2:220074557-220074498	ABCB6
A_24_P41149	13.328317	13.371204		chr12:013004509-013004568	
A_32_P133670	5.9901137	5.795319	NM_006305	chr15:69079782-69076906	ANP32A
A_23_P27947	8.058582	7.8934636	NM_032346	chr19:34912548-34916929	PDCD2L
A_23_P103442	9.674642	9.716117	NM_022756	chr1:37959110-37959051	MEAF6
A_24_P898945	7.991934	7.2988186	NM_001098801	chr18:13663574-13663515	FAM210A
A_33_P3380897	10.542131	9.5572815	NM_001695	chr8:104085226-104085285	ATP6V1C1
A_33_P3388822	4.598899	4.9688425	NM_181756	chr19:44778614-44778673	ZNF233
A_33_P3255209	9.706449	9.324396	NM_002410	chr2:135212103-135212162	MGAT5
A_33_P3376836	10.509649	10.238424		chr6:001069414-001069473	
A_33_P3404336	4.000232	4.733893	AK130766	chr17:80214991-80215050	
A_23_P6836	6.283783	6.4967732	NM_016291	chr3:48728903-48728844	IP6K2
A_23_P421423	11.756321	11.506347	NM_006291	chr14:103603617-103603676	TNFAIP2
A_23_P153050	7.7206388	7.8224087	NM_017758	chr17:18112546-18112605	ALKBH5
A_23_P395585	5.4546065	5.0799975	NM_152533	chr3:126268613-126268554	C3orf22
A_23_P65174	7.75624	7.903441	NM_001040443	chr13:50100525-50100584	PHF11
A_23_P117163	8.289926	8.213383	NM_018191	chr13:50106428-50106369	RCBTB1
A_23_P363174	6.4208064	6.335672	NM_003511	chr6:27833502-27833561	HIST1H2AL
A_23_P105873	3.9963562	3.6889877	NM_016179	chr13:38211126-38211067	TRPC4
A_24_P81841	9.829986	10.532987	NM_004064	chr12:12874940-12874999	CDKN1B
A_33_P3307068	4.7935724	4.7812824		chr9:131020849-131020824	GOLGA2
A_23_P350555	5.2992864	5.2749243	NM_144659	chr21:33949064-33949005	TCP10L
A_23_P31873	7.297796	7.5602274	NM_001002814	chr8:37718295-37718236	RAB11FIP1
A_23_P310956	4.436105	4.9826236	NM_058175	chr21:47549291-47549350	COL6A2

A_24_P808100	5.327399	5.1669846	NM_014060	chrX:119754663-119754722	MCTS1
A_23_P408353	9.550068	9.312238	NM_002116	chr6:29912381-29913024	HLA-A
A_33_P3287636	5.0706725	5.2992864	X58736	chr14:22521237-22521296	
A_32_P334325	4.91029	5.0188284	NM_015347	chr12:130921667-130921608	RIMBP2
A_33_P3251054	7.89793	8.272902	NM_194328	chr9:36336667-36336608	RNF38
A_23_P2705	4.993062	5.6170397	NM_005767	chr13:48985730-48985671	LPAR6
A_33_P3358856	11.564716	11.469954		chr19:001307493-001307434	
A_33_P3243502	4.8749356	5.231343	NR_047662	chr8:12522907-12522848	LOC729732
A_33_P3260066	4.094975	4.506756	NM_001178020	chr16:66516040-66516099	BEAN1
A_23_P35848	6.849818	6.7864704	NM_018161	chr11:71208610-71209409	NADSYN1
A_23_P118633	8.779316	8.926376	NM_022827	chr17:48632985-48633044	SPATA20
A_32_P806841	7.398059	7.7093277	NM_005738	chr7:12728454-12728514	ARL4A
A_23_P121564	6.793053	7.470336	NM_000857	chr4:156727773-156727832	GUCY1B3
A_23_P110531	2.420579	2.8980076	NM_013409	chr5:52780837-52780896	FST
A_24_P918032	4.831756	5.105775		chr15:23188417-23188358	WHAMMP3
A_33_P3326992	5.9736333	6.2968583	NM_014644	chr1:144851846-144851787	PDE4DIP
A_33_P3336484	7.4216957	7.3786974	NM_001277325	chr16:15460570-15460511	NPIPA5
A_24_P19544	7.60707	7.693696	NM_013233	chr2:168810921-168810862	STK39
A_23_P165891	7.5072203	7.211545	NM_005680	chr2:10059793-10059852	TAF1B
A_23_P58293	9.732883	9.857082	NM_181886	chr4:103722623-103720611	UBE2D3
A_33_P3345011	4.016538	4.3417296	NM_178525	chr19:8807860-8807801	ACTL9
A_33_P3361771	5.630767	5.5895195	NM_003292	chr1:186280923-186280864	TPR
A_33_P3328327	7.996203	8.061798		chr8:86090317-86090258	
A_33_P3312790	5.9213023	6.280441	NM_032445	chr15:66206222-66206163	MEGF11
A_33_P3336642	3.6126099	3.9327245	NM_032361	chr5:175395005-175394946	THOC3
A_23_P10374	7.2191863	6.599884	NM_014925	chr12:57647721-57647662	R3HDM2
A_33_P3437283	5.7250533	5.257186	AK128004	chr13:101329094-101329153	LOC643551
A_23_P124044	9.1461525	9.104904	NM_021008	chr11:644441-644382	DEAF1
A_23_P55127	5.4246173	5.870637	NM_020233	chr17:10608748-10608807	ADPRM
A_33_P3265965	6.1411896	6.3768716	NM_020827	chr4:186097000-186096941	KIAA1430
A_23_P157593	4.710769	4.8692822	NM_004198	chr8:42608298-42608239	CHRNA6
A_33_P3399181	5.412567	5.231192	XM_005256816	chr17:4643132-4643191	ZMYND15
A_33_P3789382	6.557312	6.6741633	NR_027182	chr10:65226248-65226307	JMJD1C-AS1
A_23_P204364	7.4494224	7.246896	NM_001033714	chr12:6666630-6666571	NOP2
A_23_P202496	10.33584	9.662827	NM_022451	chr10:96093097-96093038	NOC3L
A_23_P361495	4.569685	4.5000434	NM_006856	chr12:53909992-53909933	ATF7
A_23_P120335	10.780368	9.888103	NM_006554	chr2:177202234-177202293	MTX2
A_23_P205768	9.598134	9.448041	NM_006628	chr15:52843577-52843518	ARPP19
A_33_P3212274	5.1192975	5.2110662	BC016059	chr5:76012549-76012608	F2R
A_23_P91019	9.012939	9.149056	NM_003690	chr2:179296179-179296141	PRKRA
A_23_P75149	8.264774	8.398528	NM_213649	chr10:120900750-120900691	SFXN4
A_33_P3339256	3.922708	4.2150097	AK126938	chr16:12297459-12297518	
A_24_P101391	11.147322	11.433283	NM_004559	chr1:43162388-43162450	YBX1
A_33_P3382849	4.1630845	4.002593		chr22:51183222-51183281	ACR
A_23_P127584	13.974512	14.317977	NM_006169	chr11:114183072-114183131	NNMT
A_23_P26954	10.871487	11.20318	NM_006373	chr17:41166784-41166725	VAT1
A_23_P27075	9.70089	10.130947	NM_007278	chr17:7144673-7144192	GABARAP
A_23_P314120	7.3923244	7.922201	NM_005198	chr22:51018626-51018460	CHKB
A_33_P3748714	5.0221214	5.491689	NM_032664	chr8:33228739-33228680	FUT10
A_23_P100632	13.132952	13.389293	NM_001002033	chr17:73132162-73132103	HN1
A_33_P3370787	6.5663595	6.734207	NM_004442	chr1:23240041-23240100	EPHB2
A_23_P104046	8.200932	8.088572	NM_006085	chr1:220233109-220233050	BPNT1
A_23_P320739	4.2259293	4.3951063	NM_002397	chr5:88016876-88016817	MEF2C
A_24_P481824	8.113338	7.660679	NM_181787	chr8:95805979-95806038	DPY19L4
A_33_P3210079	9.127678	9.112377	NM_007165	chr19:2248589-2248648	SF3A2
A_24_P391604	10.0222435	10.43356	NM_181605	chr21:31964993-31965052	KRTAP6-3
A_33_P3364389	6.1586285	6.41238	AK293395	chr11:1857162-1857221	SYT8
A_23_P20882	11.873241	12.175588	NM_004888	chr9:117360145-117360204	ATP6V1G1
A_23_P83073	9.898451	10.192258	NM_032558	chr9:97222444-97222503	HIATL1

A_23_P79161	10.327096	10.360087	NM_013237	chr5:176732922-176732981	PRELID1
A_23_P391926	6.530013	6.821227	NM_001008701	chr19:14258817-14258758	LPHN1
A_23_P138693	6.6324987	6.754805	NM_004808	chr10:15151758-15151213	NMT2
A_24_P181101	7.884597	7.0526867	NM_022918	chr11:87033570-87033629	TMEM135
A_23_P102058	4.5484424	5.0703864	NM_002381	chr2:20192429-20192370	MATN3
A_23_P102937	10.882481	10.912896	NM_006936	chr21:46225999-46225940	SUMO3
A_23_P250982	8.898422	8.823733	NM_016048	chr5:128449367-128449426	ISOC1
A_33_P3260455	9.213438	8.683346		chr1:247497826-247497885	
A_23_P211738	9.614126	9.630013	NM_014517	chr3:33430099-33430040	UBP1
A_33_P3543133	3.922064	4.530376	NR_038970	chr14:21668348-21668289	LINC00641
A_23_P105794	4.60956	5.13411	NM_033255	chr13:43528115-43500538	EPSTI1
A_23_P364517	9.921932	10.003486	NM_021237	chr3:53919421-53919362	SELK
A_23_P100386	3.9431856	3.8566804	NM_152456	chr16:70688562-70690551	IL34
A_24_P185709	5.160762	5.47651	NM_012156	chr20:34820431-34820490	EPB41L1
A_23_P256223	10.471599	10.562885	NM_003372	chrX:154464539-154464598	VBP1
A_23_P250054	4.419346	4.72666	NM_022064	chr3:49758085-49758290	RNF123
A_23_P218282	4.791488	4.905675	NM_017810	chr16:3432833-3432774	ZSCAN32
A_24_P414999	9.308734	10.16886	NM_018407	chr8:98831373-98831432	LAPTM4B
A_23_P158925	10.286337	10.294849	NM_145290	chr4:22389118-22389059	GPR125
A_23_P217015	9.314166	9.195974	NM_003011	chr9:131455241-131455956	SET
A_23_P15876	9.260934	9.518099	NM_052947	chr18:56148827-56148768	ALPK2
A_24_P383523	6.3494077	6.3537807	NM_015589	chr14:55255724-55255783	SAMD4A
A_23_P157620	7.6813955	7.6763735	NM_022749	chr8:21961565-21961624	FAM160B2
A_23_P142424	8.891129	9.273784	NM_024660	chr19:36230233-36230174	IGFLR1
A_23_P169437	2.3221061	2.3900566	NM_005564	chr9:130914244-130914303	LCN2
A_24_P77947	2.826053	3.4873948	NM_024553	chr7:92887515-92887574	CCDC132
A_33_P3358208	6.3185444	4.98483	NM_013358	chr1:17572442-17572501	PADI1
A_23_P110961	8.436712	8.436161	NM_016098	chr6:166778507-166778448	MPC1
A_33_P3214948	5.910614	5.1289444	NM_014767	chr10:73818885-73818826	SPOCK2
A_24_P517901	9.285362	10.474212	NM_002136	chr12:54675934-54675993	HNRNPA1
A_23_P122906	9.244483	9.014129	NM_015570	chr7:70257414-70257473	AUTS2
A_33_P3379941	9.55136	9.193922	NM_014983	chr5:149432590-149432649	HMGXB3
A_23_P110362	8.225527	8.682662	NM_021970	chr4:100803178-100803119	LAMTOR3
A_33_P3382118	8.108358	8.3312845		chr17:080533168-080533109	
A_23_P43086	7.990534	7.7392445	NM_018688	chr8:22478533-22478474	BIN3
A_23_P313031	4.2211494	4.5310106	XM_005275941	chr2:242946331-242946432	LOC100509490
A_23_P306956	5.5231314	5.6163673	NM_032838	chr19:36939236-36939177	ZNF566
A_23_P169838	9.675963	9.717148	NM_025154	chr7:914243-914302	SUN1
A_23_P201863	4.4848256	4.908549	NM_212503	chr1:205500947-205501006	CDK18
A_33_P3389298	4.7847314	5.2380686	NM_001099438	chr19:35435506-35435565	ZNF30
A_23_P359245	12.086992	12.115099	NM_000245	chr7:116438290-116438349	MET
A_33_P3301297	6.9272757	7.102534		chr9:109879516-109879575	
A_32_P163147	4.5760636	4.543979	NM_182607	chrX:107322177-107322236	VSIG1
A_33_P3248231	4.1980734	4.453151	XR_171099	chr10:43248938-43248879	LOC283028
A_33_P3257330	5.8773694	5.600644	NM_173674	chr6:117853430-117853489	DCBLD1
A_33_P3282649	3.9104667	3.9163437			
A_24_P317907	7.7474575	7.91111	NM_001034954	chr10:97072614-97072555	SORBS1
A_33_P3411090	2.4306183	2.3900566	NM_001012969	chr15:43641446-43641505	ADAL
A_24_P40721	3.998157	5.0074415	NM_018327	chr20:13055000-13055059	SPTLC3
A_33_P3293474	10.253834	11.206932	NM_014322	chr1:241756511-241756452	OPN3
A_23_P94932	11.480977	11.492129	NM_015702	chr2:150426616-150426557	MMADHC
A_33_P3240333	7.850127	7.619299	NM_002653	chr5:134364825-134364766	PITX1
A_24_P21985	5.2175293	5.3571672	NM_018416	chr12:8207493-8207552	FOXJ2
A_32_P478512	6.0105886	5.9116287	NR_026771	chr15:30506598-30506657	DKFZP434L187
A_23_P132444	11.342649	11.6149025	NM_006756	chr8:54879408-54879349	TCEA1
A_23_P323166	11.842713	11.808479	NM_016333	chr16:2821188-2821247	SRRM2
A_33_P3272568	3.7276106	4.1612153		chrX:23693494-23693553	PRDX4
A_23_P350689	6.358934	6.6458836	NM_173570	chr3:113681444-113681503	ZDHHC23
A_23_P106682	9.208762	9.362601	NM_001424	chr16:10626759-10626700	EMP2

A_24_P183150	2.7714765	3.1890645	NM_002090	chr4:74902761-74902702	CXCL3
A_33_P3367855	10.416797	10.825691	NM_005027	chr19:18281283-18281342	PIK3R2
A_33_P3243832	3.9814696	4.6706514	NM_014795	chr2:145182422-145182363	ZEB2
A_23_P112341	8.754484	8.207339	NM_024945	chr9:86618846-86618905	RMI1
A_24_P119259	5.4777975	5.502879	NM_003666	chr1:169346119-169346178	BLZF1
A_33_P3391803	10.673907	10.859557		chr2:038459340-038459399	
A_24_P130792	4.4389954	4.7029557	NM_199044	chr1:46810756-46810815	NSUN4
A_24_P291133	5.883601	5.979553	NM_175866	chr1:162470837-162473545	UHMK1
A_33_P3212360	6.8665857	7.292017		chr9:136443225-136443166	
A_32_P104746	3.0069628	3.3371162	NM_020972	chr4:2271519-2271460	ZFYVE28
A_24_P370970	8.368755	8.468277	NM_015471	chr1:212957817-212957758	NSL1
A_33_P3280905	4.7278957	4.8357663		chrX:103159435-103159376	
A_33_P3322909	7.919725	8.452227	NM_001278513	chr13:113917835-113917894	CUL4A
A_24_P135628	8.428337	8.650884	XM_003846495	chr10:48277761-48277820	ANXA8
A_33_P3310475	11.72962	11.857566	NM_018243	chr4:77959708-77959767	SEPT11
A_23_P338505	4.610253	4.4431353	NM_152266	chr19:33464453-33465009	C19orf40
A_33_P3884179	8.520672	8.646752	NR_040097	chr2:98088895-98088836	LOC100506123
A_23_P107994	8.084062	8.18557	NM_017854	chr19:47549376-47549317	TMEM160
A_23_P83266	8.534409	8.521772	NM_004435	chr9:131583081-131584619	ENDOG
A_23_P101476	6.149144	6.585714	NM_030824	chr19:12460558-12460499	ZNF442
A_23_P157449	9.656863	9.452606	NM_005034	chr8:101165845-101165907	POLR2K
A_24_P201404	7.9440513	7.6898403	NM_014039	chr11:93496063-93496122	C11orf54
A_23_P2129	8.506681	7.5950146	NM_018480	chr11:85345243-85345302	TMEM126B
A_33_P3327500	4.1650724	4.4799194	NM_001015072	chr7:100486564-100486505	UFSP1
A_23_P202156	4.10153	3.9480991	NM_001261403	chr10:104161053-104161229	NFKB2
A_23_P355824	9.80728	9.683207	NM_015246	chr16:4740912-4740971	MGRN1
A_23_P25121	10.644176	10.460846	NM_016594	chr12:49317611-49315972	FKBP11
A_23_P29747	15.8631735	15.809681	NM_000984	chr17:27050638-27050923	RPL23A
A_33_P3472460	2.3221061	4.2741795	NR_104185	chr17:20808214-20808273	LOC440416
A_23_P131417	9.897303	10.125074	NM_005336	chr2:242169588-242169338	HDLBP
A_23_P127565	9.818493	9.953522	NM_178834	chr11:111431460-111431519	LAYN
A_33_P3321442	3.5254931	3.56352		chr19:56720404-56720463	
A_24_P93948	2.773994	2.3900566	XM_005268858	chr12:57980041-57980100	KIF5A
A_32_P230868	9.814969	9.852966	NM_024519	chr16:67580460-67580519	FAM65A
A_33_P3618561	4.6564326	4.7870264	BC034282		LOC730961
A_33_P3332081	6.5231276	5.9409237	NM_006558	chr8:136659779-136659838	KHDRBS3
A_23_P120146	8.702801	8.84838	NM_004257	chr2:105883957-105883898	TGFBRAP1
A_23_P40821	8.6497135	9.315982	NM_032383	chr3:148890246-148890304	HPS3
A_33_P3287785	4.0552297	4.1829467	NM_006158	chr8:24808537-24808478	NEFL
A_23_P135857	6.7364736	7.0054936	NM_004836	chr2:88856641-88856582	EIF2AK3
A_33_P3307363	7.166954	7.1635284	NM_012302	chr1:82458036-82458095	LPHN2
A_32_P159400	8.397781	8.669704	NM_015902	chr8:103271354-103271295	UBR5
A_33_P3371752	9.275441	9.300365	AK129853	chr1:51923267-51923208	EPS15
A_23_P218434	6.184138	6.8862453	NM_001032374	chr19:44679287-44679346	ZNF226
A_23_P106720	3.7695165	3.8051293	NM_006453	chr16:2026105-2026260	TBL3
A_23_P27677	6.6980715	6.7296367	NM_001571	chr19:50165258-50164080	IRF3
A_33_P3219197	4.4491844	4.2181735	NM_015527	chr16:30369553-30369494	TBC1D10B
A_24_P306726	14.765073	14.973163	NM_003295	chr13:45913711-45913652	TPT1
A_24_P93371	8.350471	8.473176	NM_017828	chr15:75631361-75631420	COMMD4
A_23_P49429	8.36564	8.452592	NM_032349	chr16:4745528-4745587	NUDT16L1
A_23_P435002	7.9655123	8.051934	NM_152546	chr5:121363253-121363312	SRFBP1
A_23_P368779	8.545707	7.6710744	NM_153608	chr19:48790154-48790213	ZNF114
A_23_P201079	2.9734101	3.3673034	NM_012231	chr1:14151185-14151244	PRDM2
A_33_P3241482	9.668042	9.781686	NM_012279	chr5:176492916-176492975	ZNF346
A_23_P117146	7.3664618	7.582761	NM_012406	chr12:108127258-108127199	PRDM4
A_33_P3346348	4.900118	4.836022	NM_001145088	chr8:124113168-124113227	TBC1D31
A_23_P19313	7.9194107	8.004091	NM_003194	chr6:170881641-170881700	TBP
A_33_P3297651	3.7862577	4.2801642	AK125068	chr3:128702545-128702486	LOC100130540
A_32_P173058	7.76081	7.6001005	NM_015012	chr11:9302564-9302505	TMEM41B

A_33_P3300495	5.8082113	5.4035807	BC022294	chr2:87229764-87229705	
A_33_P3338968	7.9519696	8.31131		chr9:014069158-014069217	
A_24_P24685	5.0944314	5.434559		chr5:179121278-179121337	
A_23_P121253	5.365175	6.1847053	NM_003810	chr3:172223925-172223866	TNFSF10
A_24_P941322	9.00584	8.881049	NM_006775	chr6:163994506-163994565	QKI
A_24_P235049	8.791383	8.661538	NM_015440	chr6:151413640-151413699	MTHFD1L
A_24_P142118	10.570412	10.57857	NM_003246	chr15:39888831-39888890	THBS1
A_33_P3401317	2.670023	2.3900566	NM_001145442	chr14:19984047-19983988	POTEM
A_33_P3243069	5.4604654	5.2904744	NM_001162893	chr1:175130034-175129975	KIAA0040
A_23_P142174	4.8885717	4.9729795	NM_004497	chr19:46376181-46376240	FOXA3
A_32_P9963	8.453252	8.252111	NM_004506	chr6:122753899-122753958	HSF2
A_33_P3378101	6.4489226	6.5441623	NM_032785	chr1:48998586-48998527	AGBL4
A_23_P28434	9.312238	9.384396	NM_003761	chr2:85809032-85809091	VAMP8
A_33_P3354067	5.1227202	5.6759176		chr12:009659100-009659041	
A_23_P406616	7.5700827	8.157678	NM_175884	chr7:106300047-106299988	CCDC71L
A_24_P475814	9.361589	9.265098	NM_025136	chr19:46052480-46052421	OPA3
A_24_P1255	6.163642	5.337838	NM_078469	chr10:127530380-127530439	BCCIP
A_23_P430558	4.405595	4.133281	NM_000751	chr2:233400127-233400186	CHRND
A_33_P3214501	6.2414656	5.8301144	NM_022767	chr15:89174348-89174407	AEN
A_23_P207614	8.930827	9.063983	NM_014680	chr17:26941993-26941934	KIAA0100
A_23_P408913	6.2078514	6.2762055	NM_152517	chr2:178415631-178415572	TTC30B
A_23_P100141	9.020357	8.602188	NM_001193388	chr16:1415345-1415286	UNKL
A_23_P29773	2.3221061	2.3900566	NM_014398	chr3:182840122-182840063	LAMP3
A_32_P118847	5.8686275	6.242008	NR_021491	chr5:92746914-92746855	NR2F1-AS1
A_24_P286527	6.0506587	6.154252	NM_022777	chr7:100957126-100957067	RABL5
A_23_P254797	3.5083678	3.8887336	NM_025227	chr20:31608368-31609120	BPIFB2
A_33_P3245927	7.18169	7.16426	NM_024623	chr12:123463936-123463995	OGFOD2
A_33_P3214105	5.472524	5.7687664	NM_001674	chr1:212788502-212788561	ATF3
A_23_P129101	7.064947	7.39334	NM_000520	chr15:72638915-72638655	HEXA
A_33_P3289466	4.2804785	4.130497		chr14:050360985-050360926	
A_23_P349127	7.5423965	7.6625757	NM_138477	chr15:43016300-43016241	CDAN1
A_33_P3297245	12.639039	12.455055	NM_012250	chr11:14299525-14299466	RRAS2
A_23_P93543	7.6515155	7.704346	NM_000288	chr6:137234615-137234674	PEX7
A_24_P114438	4.777166	4.7586875	NM_013341	chr2:174937535-174937476	OLA1
A_24_P179504	5.165289	4.7004395	NM_144668	chr12:122405914-122405973	WDR66
A_24_P419309	7.2504597	7.308468	NM_004814	chr1:31732941-31732882	SNRNP40
A_23_P201002	10.966759	10.82207	NM_018150	chr1:45117315-45117374	RNF220
A_33_P3327140	9.180775	8.884526		chr1:076531900-076531959	
A_33_P3456341	5.88803	5.9206495	NM_001170804	chr12:50855064-50855123	LARP4
A_23_P148798	9.441832	9.152109	NM_015984	chr1:192985403-192985344	UCHL5
A_33_P3277826	4.553525	4.485135	AY280370	chr19:58549738-58549797	ZSCAN1
A_33_P3265872	5.2280054	5.0431004	XR_242313	chr7:92546426-92546485	
A_33_P3216994	5.4074173	5.50714	NM_001278187	chr10:69832700-69832641	HERC4
A_23_P161488	9.382366	9.688301	NM_001033925	chr10:121336982-121336676	TIAL1
A_33_P3394366	5.435527	5.5418854		chr16:19320354-19320413	CLEC19A
A_33_P3393941	10.748498	11.096948	NM_003791	chr16:84087745-84087686	MBTPS1
A_23_P63908	8.876785	8.585315	NM_139169	chr10:116737308-116737367	TRUB1
A_33_P3224996	5.684789	6.0376387	XM_005255732	chr16:3102880-3102821	LOC101929668
A_23_P302787	9.444025	9.200206		chr2:177494727-177494668	LINC01116
A_33_P3781394	7.3864155	7.2040467	XR_108586	chr5:77654697-77654638	LOC728769
A_33_P3256282	5.190321	5.01703	NM_198693	chr21:45970780-45970721	KRTAP10-2
A_23_P258393	5.3488865	5.318757	NM_144641	chr3:52284096-52284155	PPM1M
A_33_P3299766	5.9565387	6.0281386		chr10:116593165-116593224	FAM160B1
A_33_P3231267	4.4726524	4.2569323		chr5:52409064-52409123	LOC257396
A_24_P20630	8.746318	7.3331876	NM_016269	chr4:108968805-108968746	LEF1
A_33_P3358253	8.242332	8.193386	NM_001567	chr11:71949338-71949397	INPPL1
A_23_P55880	4.4013906	4.7698674	NM_144976	chr19:12636990-12636931	ZNF564
A_23_P163682	6.577023	6.6851177	NM_022450	chr16:108531-108472	RHBDF1
A_23_P58009	6.801244	7.311735	NM_024616	chr3:111836665-111836724	C3orf52

A_33_P3388865	5.1349597	5.560713	NM_201550	chr12:70003844-70003785	LRRC10
A_23_P68401	7.433044	7.4270253	NM_030877	chr20:36431407-36468519	CTNNBL1
A_24_P704446	6.5956993	6.589198	NM_001123376	chr10:45423425-45427494	TMEM72
A_24_P200427	11.63315	11.42845	NM_001079525	chr4:57327235-57327294	PAICS
A_33_P3263569	4.7520556	4.8496637	NM_152763	chr1:109358710-109358651	AKNAD1
A_33_P3367293	4.477987	5.297611	NR_003669	chr16:56711498-56711557	MT1IP
A_23_P39910	6.38098	6.383277	NM_001008215	chr2:99216377-99216318	COA5
A_23_P155989	9.797458	9.912215	NM_022145	chr5:64813872-64813813	CENPK
A_33_P3356525	8.329837	8.612374	AK127393	chr7_gl000195_random:165765-165824	FLJ45482
A_33_P3346048	4.468006	4.923317	NR_073404	chr5:68928092-68928151	LOC441081
A_24_P37253	6.254915	6.561014	NM_194317	chr2:150329943-150330002	LYPD6
A_23_P41588	7.9480743	8.251291	NM_012208	chr5:140078491-140078550	HARS2
A_23_P206454	6.6633406	6.7885075	NM_145039	chr16:90036439-90036380	CENPBD1
A_33_P3216898	5.5159307	5.397432	NR_024011	chr9:107539985-107540044	LOC286367
A_23_P139396	10.133285	10.080393	NM_016401	chr11:86048425-86048484	C11orf73
A_23_P119964	9.958747	9.439233	NM_005760	chr2:37430001-37429942	CEBPZ
A_33_P3666797	4.8145785	4.4374385	NM_180989	chr13:95285260-95285319	GPR180
A_32_P416583	4.7765884	4.8563643	NM_032206	chr16:57077428-57077487	NLRC5
A_23_P355776	4.8338327	4.859888	NR_040415	chr20:62926397-62934547	LINC00266-1
A_33_P3211513	6.8597803	7.5201054	NM_001162407	chr2:201726021-201725962	CLK1
A_24_P246943	13.125466	12.600441	NM_021130	chr9:007598010-007598069	PPIA
A_23_P41267	7.0820775	7.295369	NR_026854	chr4:39483057-39483116	LOC401127
A_33_P3249349	5.933475	6.264101		chr22:22893243-22893184	PRAME
A_24_P172768	9.836361	9.464689	NM_004124	chr14:54941669-54941610	GMFB
A_24_P30194	8.826335	8.30494	NM_012420	chr10:91179559-91179618	IFIT5
A_23_P367405	11.625049	11.7877	NM_000281	chr10:72643511-72643452	PCBD1
A_23_P784	9.096776	8.945919	NM_032368	chr1:9992022-9991963	LZIC
A_23_P148484	7.38492	7.132637	NM_016120	chrX:73811184-73811125	RLIM
A_33_P3719214	4.5466447	4.4185343		chr13:64577541-64577482	
A_23_P144843	2.3221061	2.3900566	NM_007036	chr5:54274617-54274558	ESM1
A_23_P397019	6.497038	6.3856173	NM_001015882	chr9:114416315-114416374	DNAJC25
A_24_P931964	3.7873478	4.193317	AY358109	chr12:3720609-3720550	LOC100128816
A_23_P117694	4.985014	4.7084236	NM_006091	chr15:69019818-69019877	CORO2B
A_33_P3281196	9.64168	9.581428	NM_033281	chr5:68524132-68524191	MRPS36
A_33_P3421733	6.39133	6.2070913	NM_001199142	chr16:28726632-28726691	EIF3C
A_24_P206047	6.9939566	7.02382	NM_001151	chr4:186066313-186066372	SLC25A4
A_24_P117323	4.789376	4.6620493	NM_032775	chr22:20800911-20800852	KLHL22
A_23_P108265	4.404833	4.7221203	NM_012377	chr19:15052882-15052941	OR7C2
A_23_P60537	9.350312	9.393226	NM_004697	chr9:116054126-116054185	PRPF4
A_24_P100190	7.605653	7.6125917		chr21:46723274-46723215	LINC00315
A_23_P40847	9.467686	9.142797	NM_004267	chr3:142841583-142841642	CHST2
A_23_P429998	5.311756	3.5406988	NM_006732	chr19:45978205-45978264	FOSB
A_33_P3357964	6.1419735	6.1493683	AF111845	chr16:2652389-2652330	PDPK1
A_23_P111811	8.7585335	8.659407	NM_019071	chr7:120615261-120615320	ING3
A_33_P3256880	4.749814	4.3591633	AK131413	chrX:149095851-149095792	LOC642980
A_23_P100315	9.410921	9.579358	NM_020664	chr16:462221-462280	DECR2
A_23_P359854	8.56926	7.9120274	NM_001080450	chr6:107386517-107386458	BEND3
A_23_P140069	8.840009	9.0256405	NM_012158	chr13:77579939-77579880	FBXL3
A_33_P3406449	5.4132633	5.537571	NM_145648	chr12:129283948-129283889	SLC15A4
A_33_P3214293	4.9965653	4.91029	NM_000945	chr2:68408138-68408079	PPP3R1
A_33_P3352562	5.340315	5.209199	NM_001042680	chr19:3546158-3546099	MFSD12
A_24_P298174	9.661062	9.4846115	NM_006807	chr17:46153462-46153403	CBX1
A_33_P3314794	4.9276757	4.9250298	NM_024726	chr2:237240062-237240003	IQCA1
A_23_P21086	5.4207287	5.9148912	NM_139278	chr8:22004762-22004703	LGI3
A_23_P431569	3.4734845	3.3081698	XR_109114	chr12:754498-754557	LOC100049716
A_23_P383278	7.427164	7.4751854	NM_023078	chr8:144686701-144686642	PYCRL
A_33_P3372742	6.7568	6.9887676		chr11:071290819-071290760	
A_23_P104892	12.322415	12.544102	NM_001418	chr11:10818627-10818593	EIF4G2

A_23_P61202	8.562235	8.369637	NM_058190	chr21:46393155-46396628	FAM207A
A_33_P3376131	4.901839	5.405022	XM_001716359	chr2:113500330-113500389	NT5DC4
A_33_P3308105	11.555136	11.6586	NM_003878	chr8:63927801-63927742	GGH
A_33_P3266530	11.2311325	11.227634	NM_022156	chr17:80015933-80015874	DUS1L
A_33_P3393694	12.506763	12.495016	NM_015710	chr19:48259801-48259860	GLTSCR2
A_33_P3244347	8.130052	8.635457	NM_001040716	chr11:66616157-66616098	PC
A_23_P370830	7.229971	7.702538	NM_020805	chr18:30253267-30253208	KLHL14
A_23_P115346	9.075166	8.75259	NM_006893	chr1:206770531-206770472	EIF2D
A_23_P87575	6.472507	6.0200443	NM_001240	chr12:49087490-49087431	CCNT1
A_33_P3418496	6.6279907	5.715922		chr1:242539084-242539025	
A_33_P3408152	4.167954	3.6567633	NM_021076	chr22:29886587-29886646	NEFH
A_23_P119478	5.0416117	5.0734997	NM_005755	chr19:4237260-4237319	EBI3
A_23_P146004	6.522181	6.485446	NM_024814	chr7:107399807-107399866	CBLL1
A_23_P58538	9.328628	9.091334	NR_015370	chr5:111497889-111497948	EPB41L4A-AS1
A_32_P181638	4.7353306	5.3454976	NM_147147	chr6:105545173-105545114	BVES
A_33_P3327986	13.685942	13.674838		chr7:55816857-55816916	XLOC_014512
A_24_P544882	8.142876	7.791456	NM_003144	chr6:7281648-7281589	SSR1
A_23_P45166	8.79506	8.757027	NM_007198	chr8:37636119-37636178	PROSC
A_33_P3286621	7.149177	7.058125	NR_003013	chr17:75085516-75085575	SCARNA16
A_23_P116840	7.399332	7.280778	NM_021934	chr12:52470600-52470659	C12orf44
A_32_P190303	5.7964826	5.0708447	NM_198461	chr2:100890153-100890094	LONRF2
A_23_P117515	4.4374385	5.221336	NM_002892	chr14:58838770-58838829	ARID4A
A_33_P3368706	10.280151	10.227705	NM_001142590	chr1:41236715-41236774	NFYC
A_33_P3287745	7.581188	7.3164606	NM_182958	chr16:31142601-31142660	KAT8
A_23_P211909	7.740572	7.5894156	NM_002670	chr3:142432120-142432179	PLS1
A_23_P120153	9.798756	10.120924	NM_173647	chr2:101893115-101893056	RNF149
A_33_P3219286	5.0793653	4.915118		chr1:249141984-249141925	
A_24_P397515	8.057641	8.168045	NM_006353	chr6:26546223-26546282	HMGN4
A_23_P348298	10.252058	10.221976	NM_013299	chr11:64812090-64812149	SAC3D1
A_33_P3415086	6.403131	6.224964		chr11:094771189-094771248	
A_23_P116797	8.016396	7.9676075	NM_021640	chr12:53700512-53700571	C12orf10
A_23_P400078	6.199734	6.1715994	NM_005957	chr1:11846464-11846405	MTHFR
A_24_P399362	8.667175	8.711836	NM_020232	chr18:12720519-12720578	PSMG2
A_24_P117147	4.3271875	4.405984	NM_013289	chr19:55333311-55340821	KIR3DL1
A_33_P3303572	4.8941097	4.9621105	NM_001005610	chrX:68836480-68836539	EDA
A_23_P154379	6.6732945	7.5563526	NM_003960	chr2:73868104-73868045	NAT8
A_33_P3391275	6.3776855	6.922508		chr20:46999255-46999314	LINC00494
A_23_P214487	16.026499	15.801052	NM_003542	chr6:26104468-26104527	HIST1H4C
A_23_P36183	10.060403	10.029409	NM_005316	chr11:18387590-18387649	GTF2H1
A_33_P3269233	4.389865	5.0200915		chr2:092194575-092194516	
A_24_P196704	8.417896	8.734887	NM_182931	chr7:104715141-104715200	KMT2E
A_23_P106898	4.8049603	4.9895034	NM_152288	chr16:30965489-30965548	ORAI3
A_23_P51202	6.7606516	7.079731	NM_001077195	chr1:23686589-23686530	ZNF436
A_33_P3732466	7.220895	7.1863832	XR_110899	chr11:82902029-82901970	LOC100506282
A_32_P170749	9.002588	8.952944	NM_001282718	chr7:99780378-99780428	STAG3
A_23_P8961	7.0723777	6.1327705	NM_000880	chr8:79645431-79645372	IL7
A_33_P3665777	15.415177	14.765073	NM_001017963	chr14:102547454-102547395	HSP90AA1
A_23_P134125	7.823497	7.8777714	NM_005923	chr6:136878408-136878349	MAP3K5
A_23_P161152	7.5898666	7.641865	NM_014317	chr10:27024507-27031483	PDSS1
A_33_P3255716	6.2855325	6.524664	XM_005275940	chr2:242836276-242836217	LOC285095
A_33_P3229370	7.7239413	7.2540617	NM_001546	chr6:19840841-19840900	ID4
A_23_P256784	6.087932	6.2725973	NM_002457	chr11:1103558-1103831	MUC2
A_33_P3316522	6.352879	6.7934976	NM_001037500	chr20:30053368-30053309	DEFB124
A_33_P3277753	6.570348	6.64179	NM_144994	chr2:97503711-97503652	ANKRD23
A_33_P3257538	5.003033	4.9193535	NM_001083612	chr15:78807389-78807448	HYKK
A_23_P416894	5.334353	5.3559065	NM_017439	chr7:76940446-76940387	GSAP
A_23_P166807	10.34423	10.43281	NM_033010	chr3:51991551-51991492	PCBP4
A_33_P3416772	6.2118797	6.0176544	NM_017891	chr1:1017674-1017615	C1orf159
A_23_P206018	10.655113	10.627693	NM_001018004	chr15:63363361-63363420	TPM1

A_23_P94174	11.585232	11.4669075	NM_014175	chr8:55060159-55060218	MRPL15
A_23_P118	6.435804	6.4368033	NM_001439	chr1:101338624-101338565	EXTL2
A_23_P5731	7.1877213	6.795055	NM_016044	chr2:96076673-96076732	FAHD2A
A_23_P406928	13.150339	13.25647	HV444971	chrM:13887-13946	ND5
A_33_P3365985	5.2113256	5.2129364	XM_005250748	chr7:56887317-56887258	
A_23_P130182	7.2321596	7.3370876	NM_004217	chr17:8110917-8110655	AURKB
A_33_P3273298	4.384	3.7379482	NM_001004301	chr19:53987010-53987069	ZNF813
A_23_P414328	3.9524324	3.678636	NM_145811	chr17:64876716-64876775	CACNG5
A_33_P3399061	4.6662717	4.4239383	NR_027673	chr21:46554660-46554719	ADARB1
A_33_P3665553	2.5907788	2.3900566	AK126241	chrUn_gl000218:60884-60825	LOC100233156
A_33_P3352088	4.0979366	3.1915479	NM_001105521	chr10:133978179-133978238	JAKMIP3
A_33_P3229161	7.4135857	7.3644357	NM_021645	chr13:52605988-52606047	UTP14C
A_23_P61569	6.0747595	6.1038485	NM_003762	chr1:171673669-171673610	VAMP4
A_32_P231617	10.791008	11.082241	NM_014220	chr3:149093493-149093343	TM4SF1
A_23_P401	12.863685	13.047541	NM_016343	chr1:214837308-214837367	CENPF
A_23_P377245	5.3291264	4.641648	NR_037869	chr1:35441388-35441329	LOC653160
A_33_P3334313	6.739435	6.537446	NM_020445	chr7:152551946-152552005	ACTR3B
A_23_P29422	6.421357	6.931096	NM_004130	chr3:148714611-148714670	GYG1
A_33_P3329467	8.637686	8.679256	NM_016086	chr7:75625720-75625661	STYXL1
A_24_P876522	9.48987	8.872078	NM_001008397	chr5:54463052-54463111	GPX8
A_33_P3333587	7.3038206	7.236234	NM_032875	chr17:37409110-37409051	FBXL20
A_33_P3722568	5.6813455	5.3892198	NR_027232	chrY:231742-231801	LINC00685
A_23_P140035	8.760824	8.367172	NM_007187	chr13:41657065-41657124	WBP4
A_33_P3210168	10.611544	10.759848	NM_006221	chr19:9960239-9960298	PIN1
A_33_P3330149	7.8756385	7.9457545	NM_000280	chr11:31810639-31810580	PAX6
A_23_P126908	7.026047	7.1701736	NM_003820	chr1:2495144-2495203	TNFRSF14
A_23_P106016	6.8581505	7.0730677	NM_002742	chr14:30046087-30046028	PRKD1
A_33_P3369039	10.695141	10.738668	NM_015629	chr19:54634808-54634867	PRPF31
A_23_P364478	7.9985027	8.003629	NM_032182	chr10:126524866-126524925	FAM175B
A_23_P356965	4.2070856	4.692587	AK125941	chr17:80111566-80111507	CCDC57
A_33_P3252954	4.4803886	4.557972	AK129677	chr5:1036945-1037004	NKD2
A_23_P101281	8.328941	8.146224	NM_032828	chr19:58376178-58376237	ZNF587
A_24_P147461	3.5690072	3.7443976	NM_001031848	chr18:61647137-61648978	SERPINB8
A_33_P3398526	6.75872	6.8739595	NM_207002	chr2:111878705-111878764	BCL2L11
A_33_P3308626	8.033814	7.990534	NM_014940	chr16:77229498-77229557	MON1B
A_23_P104199	10.790112	9.956935	NM_133376	chr10:33200566-33200507	ITGB1
A_23_P206280	11.687202	11.024889	NM_201525	chr16:57698776-57698835	GPR56
A_23_P312851	3.7379482	3.9331138	NM_006928	chr12:56349072-56348089	PMEL
A_23_P158053	6.641115	6.5215397	NM_024112	chr9:130922757-130925772	C9orf16
A_23_P104762	7.297626	7.572239	NM_006106	chr11:102100584-102100643	YAP1
A_23_P209805	7.636244	7.610586	NM_005966	chr2:191555149-191555208	NAB1
A_33_P3376071	14.57595	14.611225		chr21:040499847-040499906	
A_23_P254978	9.593639	9.415003	NM_032026	chr8:125520914-125520855	TATDN1
A_33_P3225278	5.6666207	5.973983	BC039705	chr4:730227-730168	
A_23_P114057	10.079368	10.45255	NM_017789	chr2:97525582-97525523	SEMA4C
A_23_P213369	5.907586	5.991623	NM_014773	chr5:141319101-141319160	KIAA0141
A_33_P3421365	5.875481	5.70728	NM_194320	chr9:97063672-97063731	ZNF169
A_24_P281636	5.237066	5.3594112	AY010113	chr11:89790865-89790806	
A_24_P277673	4.506756	4.5582066	NM_003547	chr6:26247146-26247087	HIST1H4G
A_24_P416257	6.868987	7.146774	NM_015044	chr16:23475820-23475761	GGA2
A_33_P3364373	9.032133	9.158515	NM_001004325	chr11:1619006-1618947	KRTAP5-2
A_32_P12639	10.086377	9.901192	NM_002816	chr17:65338318-65337126	PSMD12
A_23_P338233	7.141268	7.1452456	NM_181708	chr12:50231772-50231713	BCDIN3D
A_33_P3217427	3.74966	4.103785	AK091848	chr1:15893031-15893090	DNAJC16
A_33_P3379939	9.947005	9.81586	NM_018950	chr6:29693280-29693338	HLA-F
A_23_P69362	8.184296	7.971656	NM_024661	chr3:48473899-48473840	CCDC51
A_23_P68730	7.777684	7.5398407	NM_003681	chr21:45181808-45181867	PDXK
A_33_P3317580	4.272771	4.815822	NM_001495	chr8:21549591-21549532	GFRA2
A_24_P119577	9.161728	9.594309	NM_001048183	chr1:28826368-28826427	PHACTR4

A_33_P3227703	5.6559134	4.7738457		chr22:024820118-024820059	
A_23_P48481	4.1766124	4.4964685	NM_133510	chr14:68878192-68934895	RAD51B
A_24_P372932	2.5642447	3.21226	NM_000319	chr12:7362698-7362757	PEX5
A_23_P111088	3.9774194	4.5466447	NM_033057	chr6:27879926-27879867	OR2B2
A_33_P3249354	8.544116	9.004681	NM_017785	chr5:169031146-169031205	SPDL1
A_24_P178175	7.109371	7.274812	NM_005265	chr22:25023871-25023930	GGT1
A_32_P80089	8.367632	8.220129	NR_027436	chrUn_gl000219:88312-83276	LOC283788
A_23_P20980	11.369351	11.209715	NM_001916	chr8:145151308-145151367	CYC1
A_33_P3372859	6.9527483	6.9168816	NM_001111322	chr12:113595070-113595011	DDX54
A_24_P350622	3.892023	4.334922	NM_002255	chr19:55317688-55320335	KIR2DL4
A_33_P3378654	4.3837314	4.331378	AK056520	chr10:52419349-52419290	FLJ31958
A_23_P99027	9.968062	10.117357	NM_002834	chr12:112946884-112946943	PTPN11
A_23_P59418	4.340288	4.293103	NM_005011	chr7:129395171-129395230	NRF1
A_33_P3229122	9.519344	9.290873	NM_003522	chr6:26200153-26200212	HIST1H2BF
A_33_P3298634	5.136725	5.18042	NM_001198989	chr20:34284359-34284300	NFS1
A_33_P3394352	4.79404	4.916588	BC029560	chr17:426225-426166	VPS53
A_23_P253464	8.264963	8.559543	NM_139076	chr4:84382373-84382314	FAM175A
A_24_P281395	7.6916647	7.4343715		chr10:081784573-081784514	
A_33_P3304824	6.7125983	6.452161	NR_104113	chr14:97411668-97411727	LINC00618
A_23_P13740	7.165923	6.492554	NM_014903	chr12:78606196-78606255	NAV3
A_33_P3320403	4.905675	5.3744297	AK130324	chr19:50004555-50004614	
A_32_P207243	6.4229097	6.3765936	AF132973	chr19:19626985-19627044	
A_23_P57784	9.173637	9.306452	NM_021101	chr3:190024060-190024001	CLDN1
A_23_P431789	11.43738	11.472702	NM_001660	chr3:57557101-57557072	ARF4
A_33_P3334220	7.997722	7.355002	NM_001093	chr12:109705969-109706028	ACACB
A_23_P130064	10.163528	10.116556	NM_007241	chr17:47007918-47007859	SNF8
A_23_P402733	5.295994	5.533925	NM_032195	chr21:34932355-34932414	SON
A_24_P286898	8.974962	9.349894	NM_001278547	chr10:49647005-49647064	MAPK8
A_33_P3235816	3.4600832	2.3900566	BX647611	chr7:35129516-35129457	
A_32_P52816	8.236721	6.835293	NM_016551	chr12:27124794-27124735	TM7SF3
A_23_P313512	8.756769	8.860194	NM_152640	chr12:2055353-2055294	DCP1B
A_23_P65983	7.8959913	8.136269	NM_033212	chr16:57546437-57546378	CCDC102A
A_23_P71790	5.93297	5.8653693	NM_206920	chr9:139754501-139755057	MAMDC4
A_24_P284893	12.2763605	12.406378	NM_002794	chr1:36068938-36068879	PSMB2
A_24_P302998	11.662766	11.748842	NM_007100	chr4:667722-667153	ATP5I
A_24_P16815	5.0548425	5.659393	NM_005096	chrX:70469481-70469422	ZMYM3
A_23_P35309	6.3790894	6.147603	NM_014409	chr1:229729423-229729364	TAF5L
A_24_P152404	6.1460705	6.0381446	BC032118	chr10:103789345-103789286	C10orf76
A_24_P945228	4.361496	3.8609686	NM_207352	chr4:187133518-187133577	CYP4V2
A_33_P3323699	6.0086436	6.1656246		chr1:11908277-11908218	NPPA
A_23_P112478	7.9145374	7.875041	NM_001161	chr9:34343183-34343242	NUDT2
A_23_P209904	7.4393616	6.983492	NM_002081	chr2:241407118-241407177	GPC1
A_23_P131375	7.348153	7.7420993	NM_152391	chr2:11318674-11318733	PQLC3
A_33_P3301559	7.493564	7.7479515	NM_015041	chr16:3586522-3586581	CLUAP1
A_24_P319923	9.465654	9.552821	NM_053025	chr3:123331220-123331161	MYLK
A_23_P7732	9.312776	8.987828	NM_004365	chr5:89695237-89695178	CETN3
A_23_P107116	4.113488	3.6956859	NM_007148	chr17:19320489-19320548	RNF112
A_33_P3407414	5.677516	5.742934		chrX:62646711-62646652	LOC92249
A_24_P246777	8.297493	8.088122		chrX:100165888-100165947	
A_33_P3239152	9.458644	9.040716	NM_024816	chr16:28915801-28915742	RABEP2
A_33_P3245011	8.722453	8.744506	NM_015533	chr11:61114329-61114388	DAK
A_24_P101101	10.134979	10.245322		chr17:058804791-058804732	
A_32_P201979	7.4300046	7.5780125	NM_182765	chr10:93273969-93274028	HECTD2
A_23_P102681	7.1058836	5.3325677	BC002831	chr20:57210641-57210700	
A_33_P3209950	4.9686093	5.1752524	AK124023	chr11:64510864-64510805	RASGRP2
A_33_P3415820	5.7220335	5.311389	NM_003246	chr15:39874546-39874605	THBS1
A_33_P3273290	4.4441385	4.870777	BC070146	chr19:53311236-53311177	ZNF28
A_33_P3673310	6.0109215	6.0587373		chr15:035234990-035234931	
A_23_P161125	6.444951	6.745218	NM_020963	chr1:113241083-113241378	MOV10

A_23_P72912	3.762084	4.2127166	AB018282	chr12:51890993-51891052	SLC4A8
A_33_P3334248	3.7732852	4.392532	AK096322	chr16:30673909-30673968	FBRS
A_23_P344408	4.342222	4.931839	NM_022579	chr17:61987253-61987194	CSHL1
A_23_P216596	8.287242	9.241002	NM_153366	chr9:113137663-113132253	SVEP1
A_33_P3420747	8.622412	8.337454	NM_022828	chr5:112930732-112930791	YTHDC2
A_23_P73142	7.9120274	7.6114407	NM_016360	chr17:61684812-61685208	TACO1
A_23_P406438	7.539339	7.844189	NM_182691	chr7:104757205-104757146	SRPK2
A_23_P91930	9.817699	9.654675	NM_024638	chr3:113806986-113807045	QTRTD1
A_23_P112103	6.069144	6.2910852	NM_024736	chr8:144644181-144644240	GSDMD
A_24_P208567	5.2287993	4.0158944	NM_003855	chr2:103014718-103014777	IL18R1
A_23_P215778	5.8462257	5.7894397	NM_181581	chr7:107217921-107217980	DUS4L
A_33_P3421626	8.259128	7.460016	NM_001080392	chr7:141356707-141356648	KIAA1147
A_23_P395172	6.2829266	6.424416	NM_007011	chr15:89744716-89744775	ABHD2
A_33_P3270445	5.0738387	4.732232	NM_001703	chr1:32193176-32193117	BAI2
A_23_P59877	13.344613	12.394901	NM_001444	chr8:82196174-82196772	FABP5
A_23_P34375	3.797933	3.9640045	NM_003196	chr1:23724296-23724047	TCEA3
A_33_P3284711	3.9811668	2.3900566	NR_024348	chr16:30934023-30933964	FBXL19-AS1
A_24_P260639	8.22673	8.007996	NM_005320	chr6:26234758-26234699	HIST1H1D
A_23_P252052	4.9944134	6.094391	NM_182909	chr3:99567410-99567351	FILIP1L
A_33_P3343210	7.309901	7.1581097	NM_016612	chr8:23429876-23429935	SLC25A37
A_23_P151297	5.4230795	5.286751	NM_015319	chr12:53457567-53457626	TENC1
A_33_P3321342	6.663813	7.1406965	NM_016133	chr2:118864705-118864764	INSIG2
A_33_P3251148	12.993248	12.934422	NM_001256530	chr22:43559157-43559216	TSPO
A_33_P3263603	6.2665257	6.417935		chr3:197951242-197951183	
A_32_P75425	7.4743643	7.57953	NR_037596	chr4:120326792-120326733	LINC01061
A_33_P3335401	9.059977	9.803411		chr20:049626841-049626900	
A_33_P3230017	8.934896	9.051731	NR_001587	chr1:220439717-220439658	AURKAPS1
A_33_P3254831	4.636106	4.2972684		chr15:102304845-102304786	
A_23_P30474	9.1197815	8.9612	NM_018034	chr5:37752589-37752648	WDR70
A_23_P375147	8.183525	7.530717	XM_005252056	chr9:125607126-125607067	RC3H2
A_33_P3265270	5.275695	5.6301746	NM_012434	chr6:74345187-74345128	SLC17A5
A_33_P3405946	11.327766	11.354592	NM_184234	chr20:34292455-34292396	RBM39
A_23_P74042	8.448131	8.46183	NM_012302	chr1:82456855-82456914	LPHN2
A_24_P929369	8.652025	8.368539	NM_007347	chr15:51297921-51297980	AP4E1
A_23_P39517	7.8895426	7.690781	NM_018079	chr2:45616162-45616103	SRBD1
A_32_P2738	9.298541	9.361589	NM_015631	chr10:97423752-97423693	TCTN3
A_33_P3273552	9.621923	9.118975	NM_002282	chr12:52709761-52709702	KRT83
A_23_P414273	9.1349325	9.236638	NM_032947	chr5:150176190-150176249	SMIM3
A_23_P67162	7.70178	7.5996957	NM_006221	chr19:9959972-9960031	PIN1
A_33_P3345108	4.674733	4.839042		chr18:046051006-046051065	
A_23_P202104	11.620441	11.513375	NM_005729	chr10:81114318-81114377	PPIF
A_33_P3305467	5.3263283	5.4766765		chr15:078285247-078285306	
A_33_P3365750	3.8665364	3.231843	NM_183387	chr14:89081388-89081329	EML5
A_23_P86975	2.3221061	2.3900566	NM_021571	chr11:105009673-105009614	CARD18
A_32_P219279	8.119932	7.7870274	NM_052906	chr22:37764246-37764187	ELFN2
A_33_P3729436	4.5944386	4.4064527	BF570972		SNAR-B1
A_23_P33856	6.531816	6.6502395	NM_001042749	chrX:123227937-123229223	STAG2
A_24_P374652	10.095954	10.126242	NM_022731	chr1:205687212-205687153	NUCKS1
A_33_P3409886	7.460921	7.750209	NM_014232	chr17:8063853-8063794	VAMP2
A_24_P390070	10.24084	10.375347	NM_003769	chr12:120903559-120903500	SRSF9
A_23_P983	12.978469	13.019882	NM_004905	chr1:173457084-173457143	PRDX6
A_24_P641130	5.251264	4.794443	NM_080686	chr6:31604067-31604126	PRRC2A
A_33_P3327587	5.8205466	5.9141903	BC065739	chr20:45380091-45380032	LOC100127904
A_33_P3235034	3.882818	3.9591334	XR_243769	chr17:78978467-78978526	
A_23_P47614	12.449509	11.766264	NM_003311	chr11:2949755-2949696	PHLDA2
A_23_P71037	7.616535	7.4917502	NM_000600	chr7:22771207-22771266	IL6
A_23_P376088	5.451688	5.476982	NM_017806	chr20:62370066-62370125	LIME1
A_33_P3362498	6.0281386	6.430361	NM_001184	chr3:142185226-142185167	ATR
A_33_P3355040	4.0896845	3.465114	AK093722	chr9:44239592-44239545	LOC728903

A_33_P3263027	7.240153	7.258403	BE266556	chrX:135057131-135057190	LOC100289120
A_33_P3393582	4.231759	4.215438	AK124193	chr15:43485023-43485082	CCNDBP1
A_32_P119197	7.034176	6.89246	NM_001043352	chr1:154128004-154127945	TPM3
A_23_P146233	2.3221061	2.3900566	NM_000237	chr8:19824187-19824246	LPL
A_24_P78556	3.7564027	3.109996	NM_007211	chr12:26218258-26218317	RASSF8
A_33_P3247190	6.686458	6.8950095	NM_032449	chr1:52818653-52818594	CC2D1B
A_33_P3288942	8.259384	8.296419	NM_001282695	chr10:14572391-14572332	FAM107B
A_24_P392060	7.716818	7.377846	NM_198567	chr5:175772878-175772937	SIMC1
A_24_P132039	7.68357	6.979429	NM_004290	chr5:141368097-141368156	RNF14
A_33_P3325306	13.354326	13.0325985		chr2:198367823-198367882	HSPE1
A_23_P62659	11.05386	11.271833	NM_000310	chr1:40538584-40538525	PPT1
A_23_P35609	7.594155	7.782379	NM_000375	chr10:127496016-127486690	UROS
A_32_P67623	7.9314036	7.581188	NM_017848	chrX:54094874-54094815	FAM120C
A_23_P122662	7.2734313	6.0729184	NM_018988	chr6:13363878-13363819	GFOD1
A_23_P165676	5.928748	5.5757203	XM_005263792	chr2:132247616-132247557	
A_23_P43326	9.949963	10.250599	NM_006415	chr9:94793776-94793717	SPTLC1
A_33_P3332112	7.6871543	8.273557	NM_000043	chr10:90774148-90774207	FAS
A_33_P3292337	8.459158	8.011172	NM_004521	chr10:32306139-32306080	KIF5B
A_33_P3274134	4.9905977	5.103758	NM_001137560	chr6:44244205-44244264	TMEM151B
A_23_P422083	8.0497055	8.135998	NM_018710	chr8:92006816-92006757	TMEM55A
A_33_P3797820	5.7170568	6.163642	XM_005270112	chr10:89117016-89117075	
A_23_P216355	6.3019156	6.3749547	NM_013432	chr8:145657715-145656523	TONSL
A_24_P244410	8.1279125	7.9802647	NM_014042	chr11:71821178-71820960	ANAPC15
A_23_P218646	5.491036	5.459496	NM_003823	chr20:62328874-62329690	TNFRSF6B
A_23_P418199	6.2984896	6.235501		chr9:34665625-34665327	
A_23_P429491	7.9719305	7.6920033	NM_145018	chr11:82645351-82645410	C11orf82
A_23_P218086	9.629495	9.798552	NM_001143819	chr12:113736326-113736385	TPCN1
A_33_P3554053	7.158597	7.456907	BQ009527	chrX:1517725-1517666	LINC00106
A_23_P152353	6.532503	6.857108	NM_001083614	chr16:23533750-23533691	EARS2
A_33_P3232552	5.781561	6.584879	NM_000857	chr4:156726320-156726379	GUCY1B3
A_33_P3215392	3.9453075	4.044582	NM_138568	chr19:45716229-45716170	EXOC3L2
A_23_P216402	8.420075	8.257015	NM_031432	chr9:134400144-134400085	UCK1
A_33_P3313552	6.670829	6.8502045		chrY:004866492-004866551	
A_33_P3374504	10.56963	11.341372	NM_001282512	chr22:19882715-19882656	TXNRD2
A_23_P107322	6.918786	7.3759837	NM_032854	chr17:27942109-27942050	CORO6
A_24_P22746	5.1807833	5.2018805	NM_175907	chr18:72909521-72909462	ZADH2
A_23_P111888	2.333263	2.3900566	NM_138455	chr8:104390268-104390327	CTHRC1
A_33_P3270852	13.382492	13.407759	NM_001032	chr14:50053013-50052719	RPS29
A_24_P134816	4.8268127	5.9997034	NM_182557	chr11:118767694-118767635	BCL9L
A_24_P941930	6.9061484	6.295235	NM_033083	chr3:15483644-15483703	EAF1
A_23_P109143	12.393663	12.314587	NM_000311	chr20:4681923-4681982	PRNP
A_33_P3364864	4.9454775	5.2717924	NM_005746	chr7:105913036-105912977	NAMPT
A_24_P355626	3.946475	3.2174268	NM_022169	chr11:119033297-119033356	ABCG4
A_33_P3313635	6.9758325	7.178406	BE561442	chr16:30195440-30195383	
A_23_P103631	12.394055	12.108402	NM_006824	chr1:43630074-43630015	EBNA1BP2
A_33_P3275235	9.041778	9.212635	NM_005163	chr14:105236034-105235975	AKT1
A_33_P3392123	10.652837	10.658477	NR_026544	chr12:47602603-47602544	PCED1B-AS1
A_33_P3374215	5.522471	5.672081		chr1:144917504-144917445	
A_23_P116614	7.235528	7.0263815	NM_001014811	chr11:86152353-86152294	ME3
A_23_P53193	8.69232	8.815816	NM_206927	chr11:85406136-85406077	SYTL2
A_23_P47077	7.5169077	7.6721125	NM_004281	chr10:121436388-121436447	BAG3
A_24_P148094	8.976902	9.342943	NM_017526	chr1:65897532-65897591	LEPROT
A_33_P3228271	7.012703	6.783636	XM_005260672	chr20:23608763-23608704	CST3
A_33_P3299314	8.636275	8.497133	NM_001136134	chr19:55903389-55903448	RPL28
A_33_P3352004	5.775445	5.876253		chr7:117244906-117244847	
A_23_P332260	4.1838646	4.4204073	NM_021635	chr6:138537141-138537123	PBOV1
A_23_P200216	8.572351	8.457016	NM_002370	chr1:53692805-53692746	MAGOH
A_23_P31840	12.672419	12.8388405	NM_032378	chr8:144662715-144662357	EEF1D
A_23_P207445	7.3396583	7.260766	NM_002758	chr17:67537962-67538021	MAP2K6

A_23_P336929	7.3236094	6.830694	NM_017592	chr19:39890666-39890725	MED29
A_33_P3308672	5.1257524	4.4846864	XR_243216	chr15:98417705-98417646	
A_33_P3407742	7.8350854	8.336011	NM_001033556	chr2:44436360-44436419	PPM1B
A_33_P3331345	6.391018	6.780546	NM_003584	chr2:74002134-74002075	DUSP11
A_23_P209320	6.6546235	6.5017376	NM_022817	chr2:239152876-239152817	PER2
A_24_P366535	9.81586	9.594027		chr5:176692668-176692609	
A_33_P3293114	11.009319	11.042234	NM_001190987	chr1:70715651-70715710	SRSF11
A_33_P3251289	6.339784	6.5985756	NM_018445	chr15:101814685-101814626	VIMP
A_23_P29723	5.863	5.3046308	NM_001012410	chr3:20202365-20202306	SGOL1
A_23_P104741	4.423244	4.646744	NM_032531	chr11:126293470-126293411	KIRREL3
A_32_P186027	8.881175	8.762079	NM_006305	chr15:69071634-69071575	ANP32A
A_33_P3242234	5.540865	6.0266027	NM_001007523	chrX:154612797-154612856	F8A2
A_23_P16652	6.7476616	7.0275054	NM_152791	chr19:2853940-2853999	ZNF555
A_24_P704878	4.976081	4.6460447			
A_23_P87603	7.676975	7.831796	NM_134323	chr12:53899897-53899956	TARBP2
A_33_P3301124	6.4850745	6.348262		chr5:042423563-042423504	
A_33_P3305458	6.0452843	5.9317355	NM_004381	chr6:32083106-32083047	ATF6B
A_33_P3265783	2.5819547	3.2060108	NM_003154	chr4:70867943-70868002	STATH
A_23_P31671	11.965001	11.889375	NM_006294	chr8:97244123-97244064	UQCRB
A_23_P86182	11.92667	12.061581	NM_031901	chr1:150280584-150280643	MRPS21
A_23_P121875	7.1875763	6.341478	NM_022483	chr5:43445659-43445600	C5orf28
A_23_P24345	10.873702	10.857246	NM_152264	chr11:47437938-47437997	SLC39A13
A_23_P116829	6.1338305	6.234325	NM_003348	chr12:93802692-93802633	UBE2N
A_23_P99891	9.868714	9.862705	NM_022566	chr15:81296168-81296227	MESDC1
A_24_P126417	7.1573577	7.5170097	NM_014709	chr2:61415492-61415433	USP34
A_23_P406330	5.9655223	5.7759533	NM_022733	chr1:40888681-40888740	SMAP2
A_23_P4190	6.652808	6.7981067	NM_025149	chr17:48551355-48551608	ACSF2
A_23_P170518	9.648082	9.546438	NM_017653	chr18:46570276-46570217	DYM
A_24_P261383	10.481866	10.343465	NM_024116	chr11:93469411-93469352	TAF1D
A_23_P48676	11.264576	11.178787	NM_002863	chr14:51372220-51372161	PYGL
A_33_P3370634	6.9781938	7.7695527	XR_245653	chr14:54903142-54897120	CNIH1
A_24_P341731	7.8140144	7.717897		chr22:031552562-031552503	
A_33_P3232508	12.0555	12.013686	NM_003073	chr22:24176642-24176701	SMARCB1
A_24_P664995	11.811838	9.935502	NM_001127322	chr12:54624854-54624795	CBX5
A_23_P400515	4.298929	4.5260878	NM_020816	chr1:20990700-20990641	KIF17
A_24_P92952	6.2047257	5.821155	NM_006015	chr1:27107178-27107237	ARID1A
A_23_P368195	4.5219107	4.7516623	NM_173491	chr5:157183386-157183445	LSM11
A_33_P3422260	6.824478	6.91469		chr11:134605845-134605904	LOC729305
A_33_P3247644	3.6737442	3.709077	NM_152793	chr7:30197507-30197566	MTURN
A_23_P107412	9.494283	9.602171	NM_000918	chr17:79801646-79801587	P4HB
A_33_P3269899	5.5463624	6.0105886	NM_021111	chr9:36083497-36083556	RECK
A_23_P215060	6.5589294	6.254127	NM_001018111	chr7:131185194-131185135	PODXL
A_33_P3411397	14.103653	14.097578	NM_001636	chrX:1505209-1505150	SLC25A6
A_23_P205830	10.4976425	9.6535	NM_025238	chr15:83685175-83685163	BTBD1
A_33_P3352970	7.193224	7.1513953	NM_001570	chr3:10285302-10285361	IRAK2
A_23_P101246	6.070945	6.027674	NM_001163922	chr19:51835018-51834959	VSIG10L
A_23_P132294	9.521087	9.325415	NM_001001560	chr22:38029480-38029539	GGA1
A_33_P3350643	6.410093	6.5727625	NM_001174146	chr9:129463145-129463204	LMX1B
A_23_P94517	6.423235	6.6957035	NM_014618	chr9:121929219-121929160	BRINP1
A_23_P214587	5.395346	5.4074173	NM_003449	chr6:30152921-30152862	TRIM26
A_24_P185205	5.5126076	5.290155	NM_138349	chr17:27899345-27899404	TP53I13
A_23_P376599	10.215844	10.412879	NM_006788	chr18:9537396-9537455	RALBP1
A_33_P3259861	8.95073	9.302041		chr15:22546691-22546750	REREP3
A_33_P3253804	8.379686	8.722453	NM_005195	chr8:48649932-48649873	CEBPD
A_23_P216149	11.366173	11.01481	NM_017489	chr8:73958353-73958412	TERF1
A_33_P3243929	4.921088	4.63536	AK124016	chr15:74937721-74937780	FLJ42022
A_23_P160546	8.297184	8.152092	NM_001040217	chr1:150969378-150969319	FAM63A
A_23_P106998	8.554378	8.258059	NM_016070	chr17:55926606-55918568	MRPS23
A_33_P3215729	6.085961	5.6576195		chr3:125514712-125514771	

A_33_P3346791	12.083563	12.210431	NM_005698	chr1:155225832-155225773	SCAMP3
A_33_P3327097	4.052471	4.1295877		chrX:149919702-149919761	MTMR1
A_23_P52806	9.230713	8.837922	NM_012104	chr11:117156561-117156502	BACE1
A_23_P56833	7.8086257	8.017231	NM_016115	chr2:53897810-53897751	ASB3
A_23_P45970	7.625904	7.5352573	NM_017850	chr1:38148026-38147967	C1orf109
A_33_P3243878	10.4172325	10.454265	NM_001267536	chr15:84911302-84911361	GOLGA6L4
A_33_P3211679	7.129819	7.326207	NM_001080509	chr12:31145238-31145297	TSPAN11
A_33_P3424339	9.16902	9.19851		chr1:120005154-120005095	
A_24_P317719	7.2527976	7.476321	NM_017778	chr8:38174772-38174713	WHSC1L1
A_33_P3312212	4.6939883	4.7462993	NM_206907	chr5:40769578-40769519	PRKAA1
A_33_P3270228	6.134973	6.185747		chr1:11880499-11880558	CLCN6
A_32_P155091	12.230672	12.196877	NM_145714	chr16:28848437-28848496	ATXN2L
A_33_P3236133	5.3536854	5.467161	NM_018130	chr3:72799684-72799625	SHQ1
A_24_P100517	9.186398	8.999285	NM_178448	chr9:139957282-139957223	SAPCD2
A_23_P142708	7.871766	7.6659484	NM_030805	chr2:97372119-97372060	LMAN2L
A_33_P3326210	7.719289	8.102465	NM_001017420	chr8:27660830-27660889	ESCO2
A_33_P3406899	7.0434155	7.4849095	NM_001265608	chr3:42253931-42253990	TRAK1
A_23_P420348	2.622221	2.3900566	NM_174981	chr21:15003321-15003380	POTED
A_23_P427299	5.697958	5.791787	XR_241774	chr5:34194565-34194624	LOC646652
A_23_P142688	5.198405	5.7349076	NM_024293	chr2:220047524-220047583	FAM134A
A_23_P163380	10.748753	10.779886	NM_006441	chr15:80137648-80137589	MTHFS
A_23_P403424	8.861438	8.932896	NM_005090	chr15:42140278-42140337	JMJD7-PLA2G4B
A_33_P3367984	7.917122	8.370047	NM_173076	chr2:215796336-215796277	ABCA12
A_23_P41716	11.921628	12.009898	NM_006098	chr5:180668610-180668551	GNB2L1
A_33_P3325087	7.1862273	7.2455864	NM_032141	chr17:28512706-28512765	NSRP1
A_33_P3313519	4.62694	4.460213	NM_139320	chr15:30653544-30653485	CHRFAM7A
A_24_P49687	13.589389	13.692521		chr4:062641433-062641374	
A_24_P687594	8.05484	8.162937	NM_153713	chr1:145500965-145501024	LIX1L
A_23_P88909	3.2154315	3.8528576	NM_004209	chr16:2043586-2043645	SYNGR3
A_32_P89310	6.148428	6.477091	NM_001080475	chr2:208686126-208686067	PLEKHM3
A_33_P3544887	4.0201077	4.56765	NM_001145418	chr22:28375312-28375253	TTC28
A_23_P78152	7.2937226	7.192215	NM_024039	chr17:5393710-5393769	MIS12
A_24_P288915	4.4098597	4.0638804	AK093811	chr17:18506954-18506895	
A_23_P403745	5.55077	5.244362	NM_138383	chr16:70695274-70695215	MTSS1L
A_23_P152047	4.3463597	3.864708	NM_138967	chr15:75313582-75313641	SCAMP5
A_23_P102832	7.938961	7.9972854	NM_007186	chr20:34099581-34099640	CEP250
A_33_P3421363	5.467285	5.64311	NM_194320	chr9:97054625-97054684	ZNF169
A_33_P3300733	4.684873	5.016343		chr13:37368939-37368998	
A_23_P145984	5.8444524	5.8085327	NM_012338	chr7:120427465-120427406	TSPAN12
A_33_P3257150	9.24774	9.136426	NM_001039802	chr1:22418031-22418090	CDC42
A_32_P46238	3.8014333	4.14012	NR_001443	chr17:20422735-20422676	LOC339240
A_23_P148463	8.531353	8.508735	NM_003588	chrX:119659059-119659000	CUL4B
A_23_P323751	11.05041	10.804405	NM_030919	chr20:37581326-37581385	FAM83D
A_32_P207428	5.836693	5.689207	NM_138374	chr19:53857741-53857800	ZNF845
A_23_P216568	8.478773	8.512772	NM_017832	chr9:111702937-111702996	FAM206A
A_23_P337033	5.6945734	5.446971	NM_012384	chr20:62219546-62219487	GMEB2
A_33_P3289045	6.228157	6.3645816	NM_001080843	chr22:24300034-24299975	GSTT2B
A_33_P3250750	8.695426	8.737499	CU677518	chr12:120535066-120535125	
A_24_P930418	4.9927425	5.162415	AK057533	chr8:30402947-30403006	RBPMS
A_23_P159741	6.2633395	6.633477	NM_017745	chrX:39911360-39911301	BCOR
A_23_P2114	9.5784	9.618504	NM_017907	chr11:71808801-71808742	LAMTOR1
A_33_P3296067	8.304273	8.475586	NR_003595	chr7:48886139-48886081	CDC14C
A_33_P3306153	5.6172504	5.6173534	NM_032506	chr2:61345191-61345250	KIAA1841
A_23_P92025	5.696399	5.713983	NM_022094	chr3:9911691-9911632	CIDEC
A_23_P161698	2.3221061	2.3900566	NM_002422	chr11:102706753-102706694	MMP3
A_33_P3218564	9.56476	9.731586		chr14:019625495-019625436	
A_32_P4364	10.792545	10.814958	NM_005745	chrX:152966108-152966049	BCAP31
A_23_P171336	5.475253	5.672494	NM_022052	chrX:102330853-102330794	NXF3
A_24_P141688	7.4315934	6.9898434	NM_005016	chr12:53874218-53874277	PCBP2

A_23_P122579	7.248019	7.2310615	NM_001350	chr6:33287319-33287260	DAXX
A_33_P3334225	10.28187	10.119309	NM_198839	chr17:35441992-35441933	ACACA
A_23_P137381	8.824068	8.874915	NM_002167	chr1:23885725-23885666	ID3
A_23_P96853	8.711836	8.641489	NM_007051	chr1:50941241-50941182	FAF1
A_33_P3370226	15.26942	15.2344475	NM_000971	chr8:74204068-74204009	RPL7
A_32_P351037	4.489956	4.755164	AK094515	chr2:196863916-196863857	DNAH7
A_23_P112801	11.407869	11.560923	NM_007236	chr15:41573979-41574038	CHP1
A_33_P3350094	2.6693282	2.3900566	NM_001145112	chr15:44958077-44958018	PATL2
A_33_P3328026	7.970331	8.47213	NM_015076	chr6:110931295-110931236	CDK19
A_24_P678104	4.3834295	4.508518	NM_015894	chr20:62272003-62271944	STMN3
A_23_P217120	7.7629046	7.9090395	NM_024757	chr9:140730311-140730370	EHMT1
A_33_P3302662	5.5731697	5.8352833		chr9:82191102-82191161	TLE4
A_23_P108751	11.045132	10.602913	NM_001039492	chr2:105977361-105977302	FHL2
A_24_P258955	8.0394535	8.234884	NM_020933	chr19:9273878-9273937	ZNF317
A_23_P128554	11.069391	11.077798	NM_032565	chr13:50234979-50234920	EBPL
A_33_P3356776	2.3221061	2.3900566	NM_001010917	chr10:99627719-99627778	GOLGA7B
A_33_P3238976	9.305081	9.647271	NM_020810	chr14:61439147-61439088	TRMT5
A_24_P261724	9.338753	9.5075655	NM_014868	chr12:121013680-121013739	RNF10
A_23_P59388	4.6345425	3.7626448	NM_001723	chr6:56480038-56479979	DST
A_33_P3250463	5.9153385	5.564905	NM_001128144	chr2:190670420-190670479	PMS1
A_32_P183904	4.6717644	4.9651227	NM_138356	chr15:45460065-45460006	SHF
A_33_P3395237	8.871297	8.90369		chr5:102368601-102368660	
A_23_P171237	6.470692	5.931234	NM_052957	chrX:70833291-70833350	ACRC
A_23_P254025	4.961263	5.5143976	NM_003408	chr9:115804911-115804852	ZFP37
A_24_P97770	4.9413576	5.1982045	NM_001031711	chr5:172336704-172336763	ERGIC1
A_23_P200404	11.19707	11.026471	NM_001625	chr1:33478807-33478748	AK2
A_23_P12680	8.268459	8.806389	NM_001042465	chr10:73577030-73576971	PSAP
A_33_P3278191	4.112914	4.2486897		chrX:136059137-136059078	
A_23_P77818	12.245443	12.393663	NM_006356	chr17:73035037-73034978	ATP5H
A_33_P3235521	4.3661175	4.338861		chr17:044171979-044171920	
A_23_P360964	5.7090793	5.593428	NM_145056	chr19:47150965-47150906	DACT3
A_23_P40354	8.662591	9.168171	NM_012325	chr20:31436638-31436697	MAPRE1
A_33_P3368375	4.673874	6.8415112	NM_007322	chr19:5923265-5923206	RANBP3
A_23_P115316	8.162937	8.224453	NM_022371	chr1:179064718-179064777	TOR3A
A_23_P104996	3.6414113	3.3316953	NM_004183	chr11:61730281-61730340	BEST1
A_23_P216689	8.362812	8.545082	NM_007371	chr9:136898324-136898265	BRD3
A_33_P3231653	7.175806	7.936412	NM_016548	chr9:88642401-88642342	GOLM1
A_33_P3404097	13.9824295	13.884267		chr9:69080401-69080342	PGM5P2
A_23_P253571	7.843839	7.4874587	NM_032758	chr22:41856199-41856140	PHF5A
A_23_P41365	5.4788713	5.7769995	NM_012390	chr4:71232551-71232610	SMR3A
A_23_P26094	9.796334	9.7178135	NM_024755	chr15:59171417-59171358	SLTM
A_33_P3385477	10.904322	10.598296	NM_031921	chr1:1431522-1431581	ATAD3B
A_23_P31315	12.464917	12.435078	NM_016587	chr7:26251960-26252019	CBX3
A_23_P103877	2.8342128	3.020331	NM_001010847	chr1:13801828-13801769	LRRC38
A_24_P285501	7.7626257	7.6897593	NM_172070	chr2:170940151-170940210	UBR3
A_24_P419132	4.257105	4.626305	NM_006733	chrX:100387440-100395708	CENPI
A_32_P184367	15.809681	15.829811	NM_006013	chrX:153629189-153629249	RPL10
A_33_P3358804	4.458634	4.8261223		chr17:015515414-015515355	
A_33_P3328726	4.6083646	4.8085327	NM_025055	chr15:74628335-74628394	CCDC33
A_23_P205997	7.0544443	7.6434145	NM_031301	chr15:63597940-63597999	APH1B
A_24_P186608	5.183666	5.1556153	NR_003561	chr7:102854532-102854473	DPY19L2P2
A_33_P3280950	5.29465	5.3939238	NR_026971	chr12:9220592-9220651	A2M-AS1
A_23_P206371	9.239421	9.077531	NM_003946	chr16:67209502-67209561	NOL3
A_23_P398172	6.741004	7.1603737	NM_020819	chr6:71269898-71269957	FAM135A
A_23_P259054	9.823785	9.893587	NM_153816	chr6:86217734-86216997	SNX14
A_33_P3274618	5.2998543	5.2925677	NR_029401	chr1:243245408-243245349	LOC731275
A_33_P3372332	10.179343	10.34423	NM_000101	chr16:88713223-88713164	CYBA
A_23_P127233	10.385995	10.033768	NM_005871	chr10:112052932-112052873	SMNDC1
A_23_P123256	8.023833	8.0136385	NM_014891	chr7:98995635-98995576	PDAP1

A_32_P725218	6.8828745	6.669077	NM_181725	chr17:60503886-60503945	METTL2A
A_33_P3386262	10.9184675	10.603556	NM_030928	chr16:88875606-88875665	CDT1
A_23_P106433	9.286153	9.374195	NM_033544	chr15:91505419-91505478	RCCD1
A_33_P3266744	9.739009	9.566302	NM_032872	chr1:27680362-27680421	SYTL1
A_24_P759674	5.6883945	5.0624433	NM_024928	chr10:105637425-105637366	OBFC1
A_23_P310274	4.077421	4.654019	NM_002770	chr7:142482228-142482287	PRSS2
A_23_P12062	8.677442	7.4135857	NM_017646	chr1:40306920-40306861	TRIT1
A_32_P45009	10.069782	10.358288	NM_005896	chr2:209101385-209101326	IDH1
A_23_P119344	6.0744286	5.9445333	NM_003598	chr19:49845754-49845695	TEAD2
A_23_P119627	10.759848	10.899256	NM_015965	chr19:19638092-19638151	NDUFA13
A_23_P256682	8.987042	8.922142	NM_014481	chrX:55034072-55034131	APEX2
A_33_P3213089	5.4057217	5.1684155		chr9:38566457-38566398	
A_32_P207124	7.4189415	7.704633	NM_173571	chrX:120070943-120070884	CT47A11
A_24_P658427	10.021287	9.664812	NM_005596	chr9:14081998-14081939	NFIB
A_23_P350895	6.0753603	6.289069	AK022408	chr9:125752685-125752744	RABGAP1
A_23_P163942	10.381052	10.467494	NM_003168	chr17:56423349-56423290	SUPT4H1
A_33_P3233659	7.710408	7.4648724	NM_001104546	chr10:92668240-92668299	RPP30
A_33_P3313421	8.224926	8.248929	NM_017754	chr6:34845215-34845274	UHRF1BP1
A_24_P406301	12.966296	13.007282	NM_004546	chr7:140404686-140404745	NDUFB2
A_24_P346807	9.379831	9.451525	NM_022079	chr10:69682610-69682551	HERC4
A_23_P250404	7.3719296	7.176229	NM_005732	chr5:131976479-131977910	RAD50
A_33_P3683076	5.834753	5.9586964	AK091003	chrX:54841064-54841123	MAGED2
A_23_P422212	6.5530725	6.753975	NM_173508	chr1:234460130-234460189	SLC35F3
A_23_P130466	7.223214	7.4950504	NM_021089	chr19:58807042-58807101	ZNF8
A_33_P3210278	8.73177	8.62549	NM_182914	chr14:64693075-64693134	SYNE2
A_23_P211039	6.987436	6.297576	NM_006988	chr21:28209768-28209709	ADAMTS1
A_23_P102109	10.813198	10.889472	NM_006000	chr2:220115206-220115147	TUBA4A
A_23_P104617	5.792715	5.9604897	NM_152312	chr11:45950427-45950486	GYLTL1B
A_23_P212522	7.289969	6.2047257	NM_014616	chr3:182638622-182638681	ATP11B
A_24_P407717	6.6205277	6.271994	NM_002086	chr17:73314921-73314862	GRB2
A_23_P36825	5.011899	4.6474757	NM_003979	chr12:13065707-13065766	GPRC5A
A_23_P119130	12.474697	12.486422	NM_001022	chr19:42364865-42365189	RPS19
A_23_P52082	8.150714	8.192009	NM_015434	chr1:212115412-212115353	INTS7
A_23_P108761	8.879908	9.084095	NM_017546	chr2:101885805-101885864	CNOT11
A_33_P3348234	3.5926955	3.7501924	AK024925	chr1:144990583-144990642	
A_33_P3381305	8.192653	8.325163		chr14:104670847-104670906	
A_32_P228414	6.6644273	6.9473996	NM_001282679	chr9:128121700-128121759	GAPVD1
A_33_P3397443	10.096541	10.108088	NM_182687	chr16:3022879-3022820	PKMYT1
A_33_P3303772	5.224393	5.2355075	NM_001044	chr5:1393704-1393645	SLC6A3
A_24_P418637	9.319168	9.29677	NM_012090	chr1:39749808-39750014	MACF1
A_24_P191790	5.0799975	5.3550777	NM_018126	chr4:41957209-41957268	TMEM33
A_33_P3380932	6.149564	6.443676	NM_176814	chr7:127011714-127011655	ZNF800
A_23_P404091	7.8185167	7.7512665	NM_152407	chr5:148733595-148733654	GRPEL2
A_33_P3272553	6.312803	6.0619726	NM_014551	chr22:50958122-50958181	NCAPH2
A_23_P73457	10.413191	10.488071	NM_025158	chr5:179036864-179036923	RUFY1
A_24_P178963	5.125531	5.191		chr11:71314553-71316426	
A_23_P131626	11.191802	11.401465	NM_014014	chr2:96940734-96940675	SNRNP200
A_33_P3401058	3.9121625	3.9809566	AK022140	chr5:93198713-93198654	
A_24_P586523	7.8994527	8.074123	NM_019109	chr16:5133744-5134795	ALG1
A_23_P74162	5.0393543	5.0621443	NM_018134	chr1:32673568-32673627	IQCC
A_23_P397347	5.260845	5.33002	NM_153255	chr6:119232007-119231948	MCM9
A_23_P325661	5.7469444	5.76822	NM_003435	chr19:58133133-58133192	ZNF134
A_33_P3284596	4.85774	5.070076	NM_001143968	chr17:37316904-37316845	ARL5C
A_33_P3371055	11.878414	11.686246	NM_004161	chr2:65315237-65315178	RAB1A
A_32_P193646	10.624737	10.902374	NM_002139	chrX:135955608-135955590	RBMX
A_33_P3289780	9.421664	9.629012	NM_001127393	chr14:104378686-104378627	C14orf2
A_33_P3408918	3.782917	4.7337556	NM_030754	chr11:18266850-18266791	SAA2
A_33_P3214874	10.611015	10.054527	NM_012316	chr1:32642036-32642095	KPNA6
A_23_P358662	6.7042174	6.852448	NM_001040157	chr4:175241026-175241085	CEP44

A_23_P47208	9.818236	9.679346	NM_003860	chr11:65771313-65771372	BANF1
A_32_P42197	13.538105	13.666697	NM_031157	chr12:54678778-54678837	HNRNPA1
A_33_P3244361	6.1775913	6.473879		chrX:073436828-073436887	
A_23_P433791	7.1748886	7.1463184	NM_001010984	chr1:231489251-231489310	SPRTN
A_23_P144311	10.372511	10.710367	NM_000938	chr4:57891553-57891612	POLR2B
A_33_P3374723	9.921019	9.654087	NM_001128128	chr10:31818063-31818122	ZEB1
A_33_P3368144	7.66892	8.030988			
A_33_P3333360	7.9342175	8.173709	NM_001199054	chr16:4562678-4562619	CDIP1
A_33_P3389599	2.5359733	2.3900566		chr21:15284044-15282817	ANKRD20A11P
A_24_P7157	5.4608164	5.5364103	NM_020734	chr12:8925927-8925986	RIMKLB
A_23_P48936	11.6149025	11.648045	NM_005902	chr15:67487391-67487450	SMAD3
A_23_P25403	6.3038588	6.349578	NM_013320	chr12:104497095-104497154	HCFC2
A_33_P3400263	8.634847	8.501423	NM_017444	chr8:141527098-141527157	CHRAC1
A_23_P118300	7.714539	7.755068	NM_031208	chr16:1878332-1878391	FAHD1
A_23_P88865	11.66214	11.403793	NM_144601	chr16:66647556-66647615	CMTM3
A_23_P71908	7.060481	7.351213	NM_001039697	chr9:15460250-15460309	SNAPC3
A_33_P3262118	3.8993988	2.3900566	BC051733	chr1:23416979-23416920	LUZP1
A_33_P3369844	12.997727	13.900791	NM_013230	chrY:21152726-21152667	CD24
A_23_P120472	5.05908	4.7917786	NM_003222	chr20:55213646-55213705	TFAP2C
A_32_P92281	5.7311244	5.406355		chr7:76682142-76682201	LOC100132832
A_33_P3250944	5.931554	5.857339	NM_001010979	chr1:154171921-154171862	C1orf189
A_33_P3220386	4.9010086	4.4287057	AY203962	chr8:9225342-9225401	LOC157273
A_33_P3281478	5.3416185	5.296517		chr15:030873472-030873413	
A_24_P782308	5.8585744	5.3312078	NM_001144967	chr18:56068043-56068102	NEDD4L
A_33_P3816266	6.9552274	7.0914097	XR_247053	chr1:211860454-211860513	
A_24_P184388	6.6429663	6.901546	NM_145309	chr11:71807735-71807794	LRTOMT
A_33_P3263170	12.917785	12.653652	NM_003096	chr2:70508734-70508675	SNRPG
A_23_P257895	7.48403	7.733671	NM_138957	chr22:22123603-22123544	MAPK1
A_23_P153616	10.547763	10.7008915	NM_130760	chr19:505192-505251	MADCAM1
A_33_P3354203	3.9329104	4.1224174	AB085898	chr11:21305600-21305659	NELL1
A_33_P3353259	14.453099	14.481888		chr10:121398160-121398219	
A_33_P3221373	3.8572478	4.167478		chr8:97384252-97384193	
A_33_P3386932	9.294804	9.342443	NM_201400	chr16:5135517-5135458	FAM86A
A_33_P3240078	3.1384754	3.0907245			
A_33_P3307307	7.178406	7.3679523	NM_001197104	chr11:118395871-118395930	KMT2A
A_33_P3405434	4.6638393	5.083715	AK057346	chr1:20675681-20675740	VWA5B1
A_24_P354689	7.84141	7.9252763	NM_004598	chr5:136311246-136311187	SPOCK1
A_24_P186124	8.081035	7.9845934	NM_182501	chr2:242035047-242034988	MTERFD2
A_24_P373126	4.5574427	4.8749356	NM_152307	chr14:104000891-104000950	TRMT61A
A_23_P501372	7.1309085	7.251292	NM_139162	chr17:18168888-18168947	MIEF2
A_23_P393777	4.2140627	4.6918535	NM_000953	chr14:52743026-52743085	PTGDR
A_23_P502047	7.260766	7.002904	NM_003741	chr3:184107493-184107552	CHRD
A_33_P3407424	11.401465	10.8890705	NM_152243	chr22:37965352-37965411	CDC42EP1
A_23_P29769	9.498962	9.656092	NM_015472	chr3:149238652-149238593	WWTR1
A_23_P57306	9.562331	9.315119	NM_005441	chr21:37788958-37789017	CHAF1B
A_33_P3628481	8.699083	8.124513	NR_046216	chr7:127944338-127944279	MGC27345
A_23_P210379	9.10108	9.045373	NM_175609	chr20:61920897-61920956	ARFGAP1
A_33_P3217028	6.0018473	6.2672987	NM_001014842	chr14:24659646-24659587	TM9SF1
A_23_P413641	10.63839	10.368965	NM_020820	chr20:47241677-47241619	PREX1
A_23_P51761	4.151589	4.476744	NM_001005279	chr1:158669945-158669886	OR6K2
A_33_P3447304	8.095573	8.351849	NM_001198533	chr8:107763236-107763295	OXR1
A_33_P3356325	5.4830704	5.7723846	NM_001987	chr12:11905396-11905455	ETV6
A_23_P108641	10.163082	10.127256	NM_032822	chr2:70523227-70523168	FAM136A
A_23_P168882	6.404135	5.8362103	NM_033285	chr8:95938753-95938694	TP53INP1
A_33_P3416124	4.376402	4.5813537	NR_024569	chr4:1190500-1190441	LOC100130872
A_33_P3363560	10.39362	10.428835	NM_001136216	chr1:15546776-15546835	TMEM51
A_23_P257578	8.646133	8.411724	NM_001035235	chr5:139930412-139930353	SRA1
A_24_P65098	5.25691	5.1134	NM_001110503	chr15:42551014-42550955	TMEM87A
A_33_P3406706	6.9734244	6.751632	NM_005107	chr3:38567063-38567122	EXOG

A_33_P3341494	7.002904	7.0905914	NM_033160	chr9:40772281-40772222	ZNF658
A_33_P3335920	6.3660126	6.718912	NM_182961	chr6:152443587-152443528	SYNE1
A_23_P157361	6.8422556	6.9459877	NM_018051	chr7:158738494-158738553	WDR60
A_33_P3423285	4.71216	3.8103294	BC132680	chr3:9438206-9438147	SETD5-AS1
A_23_P113793	7.7558928	6.922124	NM_024508	chr3:111311904-111311845	ZBED2
A_23_P216023	2.453722	3.1026096	NM_001146	chr8:108262371-108262312	ANGPT1
A_23_P211207	7.3628874	7.4653263	NM_001112	chr21:46646134-46646193	ADARB1
A_23_P10870	8.402374	8.470701	NM_014908	chr9:131708168-131708109	DOLK
A_24_P941708	5.9912634	6.144804	NM_001042417	chr10:70137124-70137065	RUFY2
A_33_P3272117	4.0993257	2.3900566	NM_024083	chr17:79967005-79967064	ASPSCR1
A_23_P393080	6.393606	6.712003	NM_005185	chr10:5567079-5567138	CALML3
A_33_P3392320	2.3221061	2.3900566	NM_018057	chr12:85277583-85277524	SLC6A15
A_23_P13713	5.0989556	5.03341	NM_006262	chr12:49692312-49692371	PRPH
A_23_P122852	8.131224	7.76504	NM_003078	chr7:150936124-150936065	SMARCD3
A_33_P3291294	11.208088	11.173113	NM_001440	chr8:28611117-28611176	EXTL3
A_24_P287189	4.8311214	6.0548344	NM_019009	chr11:1296280-1296221	TOLLIP
A_23_P34788	10.300921	10.367952	NM_006845	chr1:45233066-45233125	KIF2C
A_33_P3257944	12.738014	12.837569		chrX:091931850-091931791	
A_23_P215296	8.330036	8.522147	NM_003718	chr7:40134550-40134609	CDK13
A_33_P3320619	4.0623436	4.143748	NM_006044	chrX:48664784-48664843	HDAC6
A_33_P3259042	3.1649218	3.8966012	NM_001146312	chr17:12659814-12659873	MYOCD
A_23_P110661	10.538397	10.281297	NM_015360	chr5:54720638-54720697	SKIV2L2
A_33_P3317277	6.787031	6.868987	NM_001135155	chr19:38702761-38702702	DPF1
A_23_P158775	6.7015643	6.900079	NM_003361	chr16:20344432-20344373	UMOD
A_23_P107454	3.6268485	3.3114145	NM_031958	chr17:39164902-39164843	KRTAP3-1
A_24_P238143	8.705949	8.637051	NM_001006607	chr17:44626593-44626652	LRRC37A2
A_24_P374382	9.46335	9.550068	NR_001283	chr22:25161307-25161366	TOP1P2
A_33_P3221055	3.8085108	4.150577	XR_159064	chr1:158505-158446	LOC100996442
A_33_P3310706	5.232462	5.27728	DB043695	chr10:123286385-123286444	
A_24_P212819	8.241175	8.415231	AK057884	chr13:46039332-46039273	
A_23_P115149	7.8651524	7.6404552	NM_024102	chr1:111983462-111983403	WDR77
A_23_P54758	8.719505	8.404302	NM_016641	chr16:19514113-19514054	GDE1
A_33_P3315874	11.417999	11.133727	NM_014966	chr3:47891623-47891682	DHX30
A_33_P3727762	13.416193	13.209328	NM_003011	chr9:131457367-131457426	SET
A_23_P59022	6.9514723	7.020099	NM_033502	chr6:42196111-42196052	TRERF1
A_23_P310560	5.9774375	6.0963283	NM_152395	chr3:131104865-131104924	NUDT16
A_32_P175739	10.552189	10.760913	NM_000189	chr2:75120331-75120390	HK2
A_33_P3261914	4.649285	4.633793	BC036215	chr7:62809243-62809184	
A_33_P3423859	6.2657986	5.9559116	NM_006242	chr20:58511973-58511914	PPP1R3D
A_23_P19182	6.360988	6.5824223	NM_016606	chr5:137782577-137782636	REEP2
A_23_P426472	6.7923355	6.801244	NM_003425	chr19:44417365-44417306	ZNF45
A_23_P127948	7.5926766	7.229013	NM_001124	chr11:10328780-10328839	ADM
A_33_P3330413	9.124618	9.397516		chr8:048504446-048504387	
A_33_P3235568	8.863527	9.115098	NM_001206541	chr1:19665623-19665564	CAPZB
A_33_P3369317	4.4875374	5.018037	NM_001135005	chr9:34997178-34997237	DNAJB5
A_23_P60458	7.070635	6.8761635	NM_178001	chr9:131910772-131910831	PPP2R4
A_33_P3861385	2.3221061	2.3900566		chr9:69082606-69082547	
A_32_P351936	7.4071918	7.367791	NR_027776	chr7:74310325-74312274	PMS2P5
A_32_P74712	7.4564056	7.9965186	NM_021627	chr3:185348389-185348448	SENP2
A_33_P3293432	7.3488903	7.351409	NM_199340	chr17:62855653-62855594	LRRC37A3
A_32_P62997	11.362622	11.167954	NM_018492	chr8:27667843-27667784	PBK
A_33_P3375234	6.810077	7.077297	NM_018145	chr15:41029887-41029828	RMDN3
A_33_P3371514	5.7388554	5.352405	NM_022755	chr9:95377887-95377828	IPPK
A_23_P373724	9.149056	8.601868	NM_003622	chr12:27848353-27848412	PPFIBP1
A_24_P181585	12.039624	11.873241	NM_018509	chr17:48458788-48458729	LRRC59
A_33_P3258383	4.39216	4.6464267	XR_246487	chr11:118390390-118390331	
A_33_P3267532	3.640403	4.0852013	NM_170736	chr21:39671301-39671360	KCNJ15
A_33_P3259428	8.026939	7.6612253	XM_005268471	chr5:140051863-140051922	WDR55
A_33_P3272209	5.5392866	5.864071	NM_017694	chr2:191366970-191367029	MFSD6

A_33_P3298617	4.55311	4.985014		chr19:7982723-7982782	XLOC_014512
A_24_P277955	7.5550523	7.7583933	NM_016068	chr7:100884127-100883486	FIS1
A_33_P3222917	12.250114	12.299675	NM_001024736	chr15:74006791-74006850	CD276
A_23_P111132	2.335195	2.9561467	NM_005345	chr6:31785491-31785550	HSPA1A
A_33_P3336810	6.855777	6.8121147	NM_001012758	chr1:145586552-145586493	NUDT17
A_33_P3357431	3.7432256	3.6143503	AK097866	chr20:60293941-60293882	
A_23_P301372	6.807865	6.884194	NM_153365	chr4:16163192-16163133	TAPT1
A_24_P737939	4.059485	4.641821	NM_001012974	chr6:43475030-43474971	LRRC73
A_23_P55123	4.6033783	4.8268127	NM_001303	chr17:14111229-14111288	COX10
A_24_P64329	5.410092	5.361347	NM_173575	chr10:134022595-134021654	STK32C
A_32_P119616	8.959106	9.184591	NM_000126	chr15:76584972-76584808	ETFA
A_24_P49106	4.0305614	4.064535	NM_152278	chrX:102585937-102586332	TCEAL7
A_24_P309594	9.395388	9.824819	NM_017842	chr12:48176468-48176527	SLC48A1
A_23_P53891	5.0466356	4.9746885	NM_001730	chr13:73650979-73651038	KLF5
A_24_P408424	11.711084	11.627833	NM_002473	chr22:36677646-36677587	MYH9
A_23_P208937	6.3749547	6.717104	NM_024760	chr19:2993527-2994022	TLE6
A_23_P70231	9.727572	10.368453	NM_001182	chr5:125882063-125880700	ALDH7A1
A_23_P110712	9.777238	9.406553	NM_004417	chr5:172195355-172195296	DUSP1
A_23_P66241	5.887508	6.81907	NM_176870	chr16:56667810-56667869	MT1M
A_23_P119593	9.165245	9.427806	NM_024794	chr19:15337870-15337811	EPHX3
A_24_P348203	7.3252916	7.1803274	NM_025061	chr19:7966011-7966070	LRRC8E
A_33_P3325914	6.1397424	6.388009	NM_172208	chr6:33271970-33271911	TAPBP
A_33_P3221748	4.203611	4.2424064	NM_001031680	chr1:25227597-25227538	RUNX3
A_24_P194881	7.5464706	7.107771	NM_033517	chr22:51171138-51171197	SHANK3
A_23_P93431	8.949412	9.214494	NM_016108	chr6:143661259-143661318	AIG1
A_23_P211797	8.822646	8.735383	NM_130837	chr3:193414874-193414933	OPA1
A_32_P42574	15.252923	15.384792	NM_032800	chr1:230973004-230972945	C1orf198
A_23_P146284	11.006214	11.042962	NM_003129	chr8:126034443-126034502	SQLE
A_33_P3362548	5.2913194	5.3168516		chr9:136890483-136890542	
A_24_P920125	6.8780727	4.980983	NM_021130	chr7:44842121-44842180	PPIA
A_23_P6151	9.00904	9.295179	NM_002657	chr20:30780542-30780483	PLAGL2
A_33_P3344086	10.049836	10.070845	NM_021066	chr6:27782191-27782132	HIST1H2AJ
A_23_P23947	4.388271	4.846635	NM_005204	chr10:30749980-30750039	MAP3K8
A_23_P57547	8.005846	8.487146	NM_006358	chr22:41166404-41166345	SLC25A17
A_33_P3364498	11.109577	11.081654		chr9:068409003-068408944	
A_33_P3317523	12.810267	13.150339	NM_203401	chr1:26227291-26227232	STMN1
A_24_P242132	5.0429254	5.730037	NM_178564	chr8:144916834-144916775	NRBP2
A_33_P3230269	6.190317	6.253726	NM_198182	chr2:10142232-10142291	GRHL1
A_24_P50554	14.213579	14.103653		chr4:057576536-057576595	
A_23_P12336	8.682662	8.886275	NM_018137	chr1:107601422-107601481	PRMT6
A_23_P339954	11.560923	11.63739	NM_001282524	chr16:28354364-28354305	NPIPB6
A_33_P3318581	11.425524	11.741284	NM_182943	chr3:145787511-145787452	PLOD2
A_33_P3304888	7.3759837	7.9032598	NM_004505	chr17:5074145-5074204	USP6
A_23_P105664	10.952884	10.751918	NM_014167	chr12:82746915-82746856	CCDC59
A_33_P3685572	5.7123065	5.2533555	BX649145	chr8:102179109-102179050	
A_24_P167377	4.735596	5.1839323	NM_206899	chr12:56031265-56031324	OR10P1
A_24_P178093	7.2897186	6.8503885	NM_006114	chr19:45397039-45397098	TOMM40
A_23_P211355	7.8942914	7.719603	NM_022720	chr22:20098916-20098975	DGCR8
A_33_P3306894	4.2999516	4.366888	NM_012194	chr11:33604987-33605046	KIAA1549L
A_24_P60845	6.056545	6.083721	NM_000665	chr7:100490066-100490007	ACHE
A_23_P76799	9.375848	9.307386	NM_013448	chr14:35222268-35222209	BAZ1A
A_23_P52727	5.713983	5.3137693	NM_182964	chr11:20143014-20143073	NAV2
A_33_P3322428	5.4861016	5.7504535	NM_001199867	chr19:45783668-45783727	MARK4
A_33_P3279059	10.137608	10.0970125	NM_003804	chr6:3115338-3115397	RIPK1
A_33_P3349536	7.162941	7.194732	NM_001114121	chr11:125525158-125525217	CHEK1
A_23_P213319	6.321783	5.4383855	NM_197941	chr5:64444890-64444831	ADAMTS6
A_23_P130642	7.5365005	8.063571	NM_198460	chr1:89851275-89851334	GBP6
A_33_P3257187	6.649771	6.1737294	NM_206907	chr5:40759657-40759598	PRKAA1
A_23_P80839	7.246371	7.236638	NM_024871	chr3:183533854-183533795	MAP6D1

A_33_P3218951	4.628356	3.8142939	NM_002945	chr17:1802714-1802773	RPA1
A_24_P165595	7.9213758	7.9649215	NM_012097	chr2:152658509-152658450	ARL5A
A_24_P122403	8.286754	8.431232	NM_003198	chr1:24088279-24088338	TCEB3
A_33_P3233981	11.94223	11.829987	NM_002482	chr1:46083827-46083886	NASP
A_24_P98613	8.686721	8.7350025	NM_030927	chr10:82279096-82279184	TSPAN14
A_32_P26969	7.9941063	7.843543	NM_207332	chr8:614562-614503	ERICH1
A_23_P116602	8.351849	8.387245	NM_020798	chr11:77925675-77925734	USP35
A_23_P205646	8.135998	7.890714	NM_198794	chr14:50885844-50885785	MAP4K5
A_23_P115223	10.687778	10.41574	NM_006118	chr1:154248146-154248205	HAX1
A_32_P42018	3.6715672	4.3361483		chr11:22879505-22869046	CCDC179
A_33_P3283906	9.999372	10.007814	NM_016101	chr16:69376816-69376875	NIP7
A_23_P90014	6.0639906	6.1713095	NM_001080452	chr19:6731507-6731258	GPR108
A_23_P131449	3.5142505	3.7567353	NM_001145054	chr2:74641561-74641502	C2orf81
A_23_P127394	7.7739286	8.328941	NM_021117	chr11:45904680-45904739	CRY2
A_24_P115621	10.293638	10.392106	NM_004096	chr10:72188314-72188373	EIF4EBP2
A_23_P30666	6.733033	5.662486	NM_014452	chr6:47199542-47199483	TNFRSF21
A_33_P3224858	5.9148912	5.928748	NM_001135652	chr2:37368837-37368778	EIF2AK2
A_23_P110811	14.438628	14.44517	NM_001867	chr5:85916510-85916569	COX7C
A_23_P383819	7.334392	7.0382633	NM_016569	chr12:115108899-115108840	TBX3
A_23_P345710	5.996362	6.429083	NM_152531	chr3:194790184-194790125	XXYLT1
A_33_P3390032	8.29041	8.595882	NM_001145297	chr17:74079718-74079659	EXOC7
A_33_P3461633	5.2450695	5.631528	NR_036515	chr19:13945856-13945797	LOC284454
A_23_P202458	2.3221061	2.875301	NM_006963	chr10:45500488-45500547	ZNF22
A_23_P43248	9.292591	9.216216	NM_003184	chr8:120743869-120743810	TAF2
A_23_P62731	10.204222	10.269918	NM_022100	chr1:174983720-174983661	MRPS14
A_24_P258235	11.094612	11.572023	NM_001004739	chr11:55595193-55595252	OR5L2
A_23_P217938	8.111427	8.013854	NM_006542	chr1:229441171-229441230	SPHAR
A_33_P3376781	6.152378	6.4887977		chr4:113378970-113379029	
A_23_P32115	4.2361	4.1787453	NM_178536	chr9:139848647-139848706	LCN12
A_23_P53162	8.025816	8.108358	NM_003156	chr11:4114005-4114064	STIM1
A_24_P415280	4.96362	5.145789	NM_018144	chr10:12203038-12203097	SEC61A2
A_23_P69030	8.537611	8.238194	NM_001850	chr3:99514765-99514824	COL8A1
A_23_P420692	5.076062	6.5001802	NM_015053	chr1:203047602-203047661	PPFIA4
A_32_P182388	6.895335	6.751809	NM_021217	chr19:2933493-2933434	ZNF77
A_33_P3275330	3.7931855	4.3011785	NM_023018	chr1:1688682-1688623	NADK
A_23_P18123	7.2943497	6.934741	NM_014932	chr3:174000648-174000707	NLGN1
A_23_P114414	2.761383	4.7415442	NM_001031855	chrX:118151680-118151739	LONRF3
A_33_P3391970	7.170024	6.7657504		chr5:070355550-070355491	
A_23_P10911	5.3608217	5.5760117	NM_173542	chr12:113827005-113827064	PLBD2
A_23_P79221	7.802809	8.00912	NM_001105	chr2:158593493-158593434	ACVR1
A_33_P3816042	4.031328	4.457274	NR_027714	chr2:92130429-92130488	ACTR3BP2
A_33_P3325708	4.423746	4.920019	XM_005275928	chr2:122552104-122552163	
A_33_P3237005	4.3695927	5.3738594	NR_002438	chr8:135612860-135612919	ZFAT-AS1
A_23_P125705	2.3221061	2.3900566	NM_021963	chrX:72432731-72432672	NAP1L2
A_33_P3213134	8.068399	8.205227		chr14:105059131-105059190	
A_23_P71440	8.995356	8.636701	NM_001002296	chr8:41368181-41368240	GOLGA7
A_23_P68922	3.9497352	4.0661306	NM_033386	chr22:38336920-38336979	MICALL1
A_23_P137705	7.703806	7.6531897	NM_005149	chr1:168283513-168283572	TBX19
A_33_P3342295	10.512367	10.887814	NM_199425	chr20:25062369-25062310	VSX1
A_33_P3607359	5.4442797	5.2986736	NR_027282	chr10:124648065-124648124	LOC399815
A_33_P3359354	4.77281	4.708026	NM_001282671	chr1:2116762-2116703	C1orf86
A_24_P101201	11.10861	11.496664	NM_005313	chr15:44062500-44062755	PDIA3
A_23_P118150	10.404868	11.317156	NM_015161	chr16:18803812-18803753	ARL6IP1
A_33_P3383531	4.3417296	4.676006		chr2:111964026-111964085	
A_23_P303210	8.190836	7.9317927	NM_153687	chr12:99018948-99018889	IKBIP
A_33_P3317850	9.557933	9.70089	NM_003831	chr18:21063036-21063095	RIOK3
A_32_P191262	4.748889	5.0860157	NM_001097	chr22:51183605-51183664	ACR
A_23_P317056	7.998061	8.284512		chrM:14508-14449	ND6
A_33_P3357949	8.717649	9.0647335	NM_004956	chr7:13934144-13934085	ETV1

A_23_P301925	14.731188	14.946226	HV963900	chr1:567567-567626	COX1
A_33_P3336567	5.0206747	4.740123		chr7:157812575-157812634	
A_23_P171117	10.201681	10.338143	NM_024657	chrX:106184156-106184097	MORC4
A_23_P256244	4.445431	4.6756554	AK096148	chr8:107719530-107719589	OXR1
A_24_P185854	6.713712	6.713712	NM_004010	chrX:31137408-31137349	DMD
A_23_P148959	8.934475	8.171221	NM_032027	chr1:62146823-62146764	TM2D1
A_33_P3296687	4.8328743	5.000847	NM_004138	chr17:39502430-39502371	KRT33A
A_23_P145247	7.164803	7.3120055	NM_001023560	chr6:28245079-28245138	ZSCAN26
A_33_P3273255	8.914458	9.313232	BC071639	chr19:53953157-53953216	
A_23_P40588	8.225279	8.3358	NM_172002	chr22:29141953-29147244	HSCB
A_33_P3335845	7.6563864	7.961073	NM_001006622	chr2:128522175-128522116	WDR33
A_24_P763243	15.16313	15.339501	NM_001402	chr6:74227853-74227794	EEF1A1
A_33_P3316928	9.439686	9.887742	NM_020651	chr2:64320119-64320060	PELI1
A_23_P257091	6.0269537	6.1424913	NM_005085	chr9:134098303-134103591	NUP214
A_23_P24135	6.2044716	6.5135016	NM_001057	chr10:71164785-71164726	TACR2
A_23_P85004	5.857339	5.7022214	NM_007309	chrX:96369895-96369954	DIAPH2
A_24_P310756	8.217353	6.9085865	NM_015176	chr1:224349468-224349527	FBXO28
A_33_P3251144	5.0354476	4.6616297	NM_001127370	chr7:21947808-21947749	CDCA7L
A_33_P3242798	7.5053444	7.8535595	NM_001875	chr2:211543741-211543800	CPS1
A_33_P3366667	5.586421	5.8123407	NM_000503	chr8:72110532-72110473	EYA1
A_23_P27215	15.205528	15.218346	NM_018955	chr17:16285505-16285564	UBB
A_23_P139704	9.5629635	9.663436	NM_001946	chr12:89742203-89742144	DUSP6
A_23_P501080	8.153679	8.347727	NM_007139	chr7:64865861-64865920	ZNF92
A_23_P215331	3.9699986	4.0752845	NM_001883	chr7:30695225-30694667	CRHR2
A_24_P379104	5.6348267	5.9565387	NM_006875	chrX:48770830-48770771	PIM2
A_23_P156788	4.183386	4.379977	NM_003764	chr6:144508698-144508757	STX11
A_23_P40952	7.993722	8.246457	NM_002880	chr3:12625670-12625611	RAF1
A_24_P196117	10.026819	9.94143	NM_207118	chr6:158613211-158613270	GTF2H5
A_23_P99452	6.0182095	5.8942757	NM_000059	chr13:32972996-32973055	BRCA2
A_33_P3403132	13.231004	13.271389	NM_005234	chr19:17342753-17342694	NR2F6
A_33_P3646133	8.191725	8.13185	NM_032815	chr16:28976611-28976670	NFATC2IP
A_33_P3357669	10.962393	10.970034		chr3:043527287-043527228	
A_23_P152087	9.344394	9.356319	NM_018145	chr15:41028290-41028231	RMDN3
A_33_P3250253	5.4194136	5.652053	AK096194	chr11:67008076-67008135	LOC100131150
A_33_P3358099	7.730506	8.040771	NM_181449	chr17:72606150-72606091	CD300E
A_24_P929754	7.029006	7.244122	NM_199054	chr19:2038249-2038190	MKNK2
A_24_P410017	12.551935	12.654364	NM_001277406	chr2:131220693-131220634	POTEI
A_32_P119248	5.4163404	4.9734597	NM_207305	chr9:116549-116490	FOXD4
A_23_P389102	2.3221061	2.3900566	NM_015194	chr17:30819891-30819832	MYO1D
A_24_P71468	2.3221061	2.3900566	NM_012413	chr2:37600060-37600119	QPCT
A_23_P144458	5.7759533	5.7090793	NM_001221	chr4:114386707-114381361	CAMK2D
A_33_P3383326	7.8531237	7.9282217	NM_057159	chr9:113636129-113636070	LPAR1
A_33_P3417880	5.6635776	5.7720876	NM_001007531	chr6:28228606-28228665	NKAPL
A_24_P135902	15.509981	15.480215	NM_002952	chr16:2013159-2012861	RPS2
A_23_P21162	9.770326	10.163082	NM_152773	chr3:196018207-196018148	TCTEX1D2
A_23_P376686	5.563221	5.341913	NM_019602	chr6:32362725-32362666	BTNL2
A_23_P395075	7.4797087	8.453941	NM_018433	chr2:86718371-86719192	KDM3A
A_24_P916656	5.7144113	4.921088	NM_014478	chr7:65618600-65618659	CRCP
A_23_P39402	5.5684795	5.6040454	NM_198867	chr19:36503981-36502366	ALKBH6
A_33_P3331366	10.455351	9.25594	NM_005082	chr17:54965333-54965274	TRIM25
A_23_P99604	7.7469187	7.9529543	NM_017769	chr14:31084686-31084745	G2E3
A_33_P3327762	4.7441754	4.7933245		chr9:000405038-000405097	
A_23_P151368	6.53583	5.919621	NM_174928	chr13:21303314-21303255	N6AMT2
A_33_P3531857	11.2506695	11.327057	NM_001349	chr2:136669012-136668953	DARS
A_33_P3237719	5.2406187	5.03683		chr6:159187821-159187880	
A_23_P118690	11.0795965	11.047953	NM_001032293	chr17:30696956-30697015	ZNF207
A_23_P110791	4.762603	4.4162335	NM_005211	chr5:149432920-149432861	CSF1R
A_33_P3220939	8.800205	8.326876	NM_001258213	chr13:113832042-113831983	PCID2
A_33_P3245439	5.323369	5.098238	NM_001250	chr20:44755280-44755339	CD40

A_23_P59637	8.817034	8.386516	NM_014705	chr7:111366325-111366266	DOCK4
A_23_P430181	6.661681	6.6889725	NM_024784	chr11:62518522-62518463	ZBTB3
A_33_P3256952	5.242975	5.0310154	NM_022073	chr14:34393481-34393422	EGLN3
A_24_P186216	6.467751	6.8369226	NM_001100400	chr4:39900034-39899975	PDS5A
A_33_P3424364	2.3221061	3.409425	NM_001134848	chr5:42801648-42801707	CCDC152
A_24_P404033	7.301172	7.3313723	NM_182616	chr15:90444899-90444840	C15orf38
A_33_P3699445	5.474748	4.8726635	BC025792	chr11:64546653-64546712	
A_23_P401568	11.133727	11.239555	NM_005911	chr2:85771929-85771988	MAT2A
A_23_P28969	7.668565	7.4222326	NM_176812	chr20:32438804-32438863	CHMP4B
A_23_P80032	7.959534	7.4862456	NM_005225	chr20:32264048-32263989	E2F1
A_24_P406006	7.5275235	7.74832	NM_024830	chr5:1462269-1462210	LPCAT1
A_33_P3401647	2.5863547	2.3900566	NM_033256	chr19:38743594-38743535	PPP1R14A
A_23_P57268	6.304325	7.068927	NM_001338	chr21:18938329-18938388	CXADR
A_24_P187056	2.490728	2.3900566		chr1:142711308-142700309	ANKRD20A12P
A_24_P16340	12.941564	13.201168		chr1:220490434-220490375	
A_24_P400473	9.437453	9.593639	NM_014655	chr1:156182353-156182412	SLC25A44
A_24_P244356	8.372268	8.648808	NM_001282144	chr11:119054656-119054715	NLRX1
A_24_P264909	9.561395	9.524292	NM_019080	chr13:80127860-80127919	NDFIP2
A_23_P14184	7.4759235	7.382924	NM_018676	chr13:52951493-52951434	THSD1
A_33_P3310409	4.1108255	3.8002393		chrX:052573223-052573282	
A_23_P36464	9.527785	9.337106	NM_018164	chr12:27058389-27058330	ASUN
A_23_P253421	7.350246	7.130242	NM_181575	chr2:74755130-74755071	AUP1
A_24_P366415	12.866091	12.388254		chr2:174350887-174350828	
A_33_P3318946	4.8651037	4.4018345	NM_021817	chr1:156595457-156595516	HAPLN2
A_23_P217054	6.6517606	6.800461	NM_024345	chr9:37861377-37861436	DCAF10
A_23_P110643	4.557972	4.924016	NM_016508	chr5:133643945-133643886	CDKL3
A_24_P185986	7.0927105	6.6726036	NM_145052	chrX:74524196-74524255	UPRT
A_23_P120170	6.3395624	6.2514095	NM_145702	chr2:233413008-233412949	TIGD1
A_24_P166311	5.885665	5.888351	NM_022090	chr5:159820990-159820931	C5orf54
A_33_P3230264	5.673222	6.115056	NM_001164617	chrX:132669886-132669827	GPC3
A_23_P32454	4.6670704	4.4968333	NM_003235	chr8:134147066-134147125	TG
A_24_P110983	7.796222	8.218985	NM_005465	chr1:243665260-243665201	AKT3
A_24_P401637	6.395406	6.4104643	AK095727	chr19:6378537-6378478	LOC100130856
A_23_P254733	10.198949	10.103994	NM_024629	chr4:185616396-185616337	CENPU
A_23_P103661	10.350051	10.464956	NM_139118	chr1:155629483-155629424	YY1AP1
A_32_P144596	5.3395076	5.1510715	NM_003747	chr8:9639084-9639143	TNKS
A_23_P42884	13.103125	13.0954075	NM_032014	chr7:43906220-43906161	MRPS24
A_23_P101374	2.8392873	3.109451	NM_030622	chr19:41713082-41713141	CYP2S1
A_23_P420269	5.7379885	5.473322	NM_020748	chr17:59943270-59943211	INTS2
A_33_P3358307	6.210803	6.23512	NM_020205	chr1:149912323-149912264	OTUD7B
A_33_P3321275	5.997055	6.41612	NM_170606	chr7:151921158-151921100	KMT2C
A_23_P117727	6.8943014	6.601973	NM_139242	chr15:65298471-65297236	MTFMT
A_33_P3303136	9.764388	9.930538	NM_001195291	chr6:2948591-2948532	SERPINB6
A_23_P140511	8.175134	8.411174	NM_006660	chr15:65447239-65447180	CLPX
A_23_P121686	9.579995	9.421128	NM_004757	chr4:107258100-107258159	AIMP1
A_24_P373844	7.6205926	7.671503	NM_024076	chr19:34306314-34306373	KCTD15
A_33_P3221019	4.83436	5.034026	NM_173059	chr7:100352964-100353023	ZAN
A_23_P329962	2.3221061	2.3900566	NM_001030019	chr7:48026942-48026883	SUN3
A_23_P83094	7.66433	8.006728	NM_007005	chr9:82341057-82341116	TLE4
A_32_P182662	8.364084	9.043018	NM_022831	chr1:222843215-222843156	AIDA
A_32_P113114	6.521018	6.8042493	NM_152289	chr19:9721227-9721168	ZNF561
A_33_P3249214	8.038796	8.057641	NM_001286790	chr4:78634645-78634586	CNOT6L
A_33_P3394769	11.157707	11.296013	NM_001080483	chr9:136379767-136379708	TMEM8C
A_23_P77562	8.253607	8.540957	NM_194280	chr16:29979401-29979460	TMEM219
A_33_P3301689	7.0773544	7.170601	NM_182975	chr1:90470748-90470807	ZNF326
A_24_P921366	10.994911	10.837336	NM_033138	chr7:134655197-134655256	CALD1
A_23_P37127	5.508082	5.203005	NM_004496	chr14:38059605-38059546	FOXA1
A_33_P3243717	5.684507	5.789916	NM_021045	chr10:38120602-38120543	ZNF248
A_32_P49350	12.3095	12.670279		chr20:004610767-004610708	

A_23_P103099	8.097064	8.213221	NM_001031695	chr22:36174111-36164377	RBFOX2
A_23_P122007	7.6114407	7.364299	NM_033211	chr5:102613812-102613871	C5orf30
A_24_P96762	6.0855923	6.1650233	NM_017846	chr1:28904907-28904966	TRNAU1AP
A_32_P23308	6.357938	6.257674		chr5:169761760-169761819	LOC257358
A_23_P107644	12.427046	12.179079	NM_006938	chr18:19209071-19209130	SNRPD1
A_33_P3224780	4.3372445	4.745452	XM_005263597	chr2:120187501-120187558	TMEM37
A_33_P3364884	9.6065	9.817288	NM_000925	chr3:58413486-58413427	PDHB
A_33_P3270863	5.148839	4.1630845	NM_000379	chr2:31557247-31557188	XDH
A_23_P91640	4.1103725	3.5622792	NM_020437	chr22:26838475-26838534	ASPHD2
A_23_P252306	12.771793	12.8290205	NM_002165	chr20:30194158-30194217	ID1
A_24_P778906	7.055529	7.298196	NR_046228	chr1:142697696-142697637	ANKRD20A12P
A_33_P3248384	10.529507	10.827929		chr6:29870010-29870069	XLOC_014422
A_33_P3401394	3.910712	4.3819904		chr5:072175115-072175056	
A_24_P66125	5.5635433	5.660327	NM_001042749	chrX:123235840-123235899	STAG2
A_24_P325176	7.7545404	7.9785275	NM_015312	chr4:123283212-123283271	KIAA1109
A_23_P159125	8.737355	8.982774	NM_004695	chr17:73102149-73102208	SLC16A5
A_23_P214727	6.8297772	7.018	NM_030784	chr6:97246699-97246640	GPR63
A_24_P175176	8.20571	8.164579	NM_020432	chr7:77586179-77586238	PHTF2
A_33_P3831730	8.30791	8.437103		chr7:10489507-10489448	MGC4859
A_33_P3304528	7.5543447	7.472636	NM_001267595	chr12:48437493-48437434	SENP1
A_23_P37910	5.657242	5.606451	NM_002746	chr16:30128296-30128237	MAPK3
A_24_P388570	5.084532	4.8956685	NM_032778	chr3:97677915-97673301	MINA
A_24_P381199	6.5072823	6.612828	NM_001003818	chr11:5633867-5633926	TRIM6
A_23_P79426	7.958541	7.5926766	NM_016289	chr2:231684957-231685016	CAB39
A_33_P3416301	4.23112	4.1152515	NM_212540	chr2:11605958-11605899	E2F6
A_23_P218331	7.849162	7.8292365	NM_001017916	chr17:61510687-61510628	CYB561
A_23_P82868	6.3410287	6.0106654	NM_000930	chr8:42032871-42032812	PLAT
A_33_P3591761	6.3307905	6.880237	NM_144963	chr8:124824840-124824899	FAM91A1
A_23_P31798	5.5587397	5.5169296	NM_000015	chr8:18258142-18258201	NAT2
A_33_P3422330	8.960728	9.113756	NM_198317	chr1:901036-901095	KLHL17
A_23_P96688	4.268749	4.682235	NM_016028	chr11:67941288-67939135	SUV420H1
A_23_P203891	11.876175	11.5050535	NM_006312	chr12:124809053-124808994	NCOR2
A_23_P29029	6.3645816	6.114746	NR_026845	chr21:33765742-33765801	C21orf119
A_24_P109069	4.7168307	4.806468	NM_181519	chr10:46963983-46963924	SYT15
A_33_P3368646	5.6599035	6.086866	NM_173515	chr6:154743768-154743709	CNKSR3
A_23_P84448	8.180781	8.082573	NM_006000	chr2:220115722-220115663	TUBA4A
A_23_P167856	5.3250384	5.136415	NM_018426	chr6:44122880-44122939	TMEM63B
A_23_P108657	7.0785236	7.1750975	NM_152528	chr2:160112861-160112802	WDSUB1
A_23_P132004	9.594495	9.76585	NM_178466	chr20:31815418-31815477	BPIFA3
A_33_P3350547	4.516033	4.4672856	NM_001099404	chr3:38592239-38592180	SCN5A
A_32_P182473	6.637231	6.867626	NM_145233	chr19:12258592-12258533	ZNF625
A_33_P3221528	10.015991	9.193239	NM_004136	chr15:78793507-78793566	IREB2
A_33_P3378920	10.896184	10.986963	NM_001198845	chr11:66413847-66413906	RBM14-RBM4
A_33_P3423445	3.208131	3.0628664		chr19:23332658-23332717	ZNF730
A_23_P103628	9.902796	9.969467	NM_018072	chr1:236716891-236715359	HEATR1
A_33_P3241369	5.9690537	6.130227	NM_053049	chr10:5415878-5415937	UCN3
A_23_P252322	14.611225	14.637443	NM_006886	chr20:57603871-57603812	ATP5E
A_33_P3395581	3.693755	3.9374378	NR_028346	chr11:89727098-89727044	TRIM53AP
A_33_P3212679	12.235644	12.083563	NM_003096	chr2:70508737-70508678	SNRPG
A_33_P3307133	4.568883	4.8419466	NM_021956	chr6:102134127-102134186	GRIK2
A_33_P3391476	8.19973	8.551639	NM_001130042	chr1:75172848-75172789	CRYZ
A_23_P138025	10.040699	10.060047	NM_018066	chr1:27206022-27205963	GPN2
A_24_P26897	8.768257	9.053835	NM_005539	chr10:134596451-134596510	INPP5A
A_23_P256933	14.242917	14.17963	NM_000986	chr3:101401280-101400008	RPL24
A_24_P469641	8.143114	8.217001	NM_207111	chr7:5659694-5659655	RNF216
A_33_P3306452	4.865713	4.3449144	XM_005272753	chr9:70434183-70434124	
A_33_P3232493	6.0171595	5.9893136	AK130794	chr8:066645279-066645220	
A_33_P3402783	6.216202	6.9334254		chr22:024271626-024271685	
A_32_P186474	11.045767	11.171302	NM_013277	chr12:50383073-50383014	RACGAP1

A_33_P3370570	2.3221061	2.3900566	NM_173800	chr5:115338954-115339013	AQPEP
A_23_P12463	9.672606	9.632752	NM_002826	chr1:180167049-180167108	QSOX1
A_24_P178065	3.6870549	3.6050181	BC038806	chr3:111604399-111604458	PHLDB2
A_32_P358887	8.84148	8.8967285	NM_003759	chr4:72437442-72437501	SLC4A4
A_23_P83818	4.4335694	4.6680737	NM_000093	chr9:137734063-137734122	COL5A1
A_23_P152995	3.7316346	3.1293483	NM_001045	chr17:28525002-28524943	SLC6A4
A_32_P140898	5.1242785	4.7264695	NM_002158	chr2:48602485-48602544	FOXN2
A_23_P5903	11.248809	10.7724695	NM_016354	chr20:61303447-61303506	SLCO4A1
A_33_P3274622	5.4098186	5.383994		chr1:243264899-243264840	LOC731275
A_33_P3339915	4.2236996	4.123085	NR_003594	chr8:86775854-86775795	REXO1L2P
A_23_P69573	3.9142025	3.660012	NM_000856	chr4:156643328-156651224	GUCY1A3
A_33_P3415625	4.0806565	4.250492	NM_001079910	chr12:85518235-85518294	LRRIQ1
A_23_P421306	5.33002	4.905281	NM_177963	chr11:66818219-66818278	SYT12
A_23_P124427	6.94683	7.0637445	NM_012224	chr4:170315411-170315352	NEK1
A_33_P3326817	5.3241606	5.0714164		chr2:038830256-038830315	
A_23_P11224	9.118174	8.881489	NM_173470	chrX:135044415-135044356	MMGT1
A_33_P3353552	8.683	9.506384	NM_017842	chr12:48175294-48175353	SLC48A1
A_33_P3233081	5.9600024	6.278035	NM_002441	chr6:31730225-31730284	MSH5
A_33_P3247919	4.500938	4.7932596		chr9:092035519-092035460	
A_24_P382287	5.0720525	5.27269	NM_004361	chr18:63529955-63530014	CDH7
A_33_P3293753	6.637438	7.0155	NM_198690	chr21:46047633-46047692	KRTAP10-9
A_23_P259328	7.9347377	7.8974786	NM_003630	chr6:143810712-143810771	PEX3
A_33_P3219295	3.8306093	4.3186884		chr16:71657672-71657613	
A_23_P70318	6.7881184	6.8685455	NM_014936	chr6:46114082-46114141	ENPP4
A_33_P3263284	6.0543213	6.4879026	NR_024563	chr12:132857426-132857485	LOC100130238
A_23_P44581	7.3655663	7.42642	NM_014287	chr16:14978301-14980638	NOMO1
A_23_P319874	4.754073	4.6564326	NM_001029840	chr3:44400419-44400478	TCAIM
A_33_P3289352	3.7884169	4.251568	NM_138300	chr1:154931357-154931298	PYGO2
A_23_P140170	10.525234	11.229525	NM_006364	chr14:39501728-39501669	SEC23A
A_24_P409126	4.7586875	4.6371546	AK056071	chr13:49720040-49720099	FNDC3A
A_23_P10305	4.46479	4.5825725	NM_175859	chrX:16688736-16685822	CTPS2
A_23_P213883	7.8297863	7.8040013	NM_133433	chr5:37064923-37064982	NIPBL
A_33_P3788618	5.23232	5.5834236	NM_001010887	chr9:19451559-19451618	ACER2
A_23_P5912	10.6830435	10.539213	NM_017798	chr20:61826871-61826812	YTHDF1
A_33_P3276604	3.3688972	3.391696		chr8:94242001-94242060	
A_23_P7582	6.6985664	6.6553636	NM_003202	chr5:133483237-133483296	TCF7
A_33_P3364741	11.845602	11.907459	NM_006039	chr17:60770827-60770886	MRC2
A_33_P3613358	4.9352293	5.1445713	AK097143	chr6:141903110-141903051	
A_24_P323941	5.7213173	5.171273	NM_001012971	chr20:55101088-55101147	FAM209A
A_24_P185314	8.814234	8.665142	NM_003826	chr18:10552603-10552662	NAPG
A_33_P3556532	2.3221061	2.3900566		chr17:76498343-76498284	DNAH17
A_32_P1533	5.252243	4.9208446	NR_026921	chr5:177045687-177045628	LOC202181
A_33_P3398448	10.034353	10.336729	NM_032789	chr8:145051380-145051321	PARP10
A_32_P69930	6.9973497	7.3278074	NM_153376	chr4:7043019-7042960	CCDC96
A_32_P11450	5.645251	5.4404473	NM_014753	chr10:43318687-43319089	BMS1
A_33_P3422085	3.6575816	3.5696318	NM_001009615	chrX:142795579-142795520	SPANXN2
A_33_P3499692	8.392352	8.633617	AF258587	chr5:86344779-86344720	LOC645261
A_23_P424316	7.5607004	7.498988	NM_005650	chr22:42556763-42556704	TCF20
A_32_P71788	7.127641	6.561235	NM_002014	chr12:2909056-2909213	FKBP4
A_23_P125383	4.7695084	4.9460373	NM_016192	chr2:192815005-192814946	TMEFF2
A_24_P413437	9.076081	9.282178	NM_007363	chrX:70519825-70519884	NONO
A_23_P256504	5.4414306	5.759685	NM_001633	chr9:116823757-116823373	AMBP
A_23_P35591	10.451376	9.42388	NM_016046	chr10:99196229-99196170	EXOSC1
A_33_P3359748	5.1389337	5.228359	AK093718	chr1:109779432-109779491	SARS
A_33_P3277075	7.526361	7.023241	NM_000814	chr15:26788916-26788857	GABRB3
A_33_P3288659	4.8291903	5.1077957	NM_030812	chr1:18152913-18152972	ACTL8
A_23_P83498	3.726499	3.7399855	NM_006546	chr17:47132379-47132438	IGF2BP1
A_23_P57521	11.276537	11.389118	NM_016091	chr22:38274137-38282789	EIF3L
A_33_P3421490	5.411585	5.0989556	NM_015206	chr15:79764573-79764632	KIAA1024

A_33_P3376551	2.3221061	2.3900566	NM_001039585	chr1:79005189-79005248	PTGFR
A_23_P36513	5.949614	5.9625874	NM_001206710	chr12:49397028-49396969	PRKAG1
A_33_P3240767	8.579056	8.44455	NM_022094	chr3:9908458-9908399	CIDEC
A_33_P3363720	5.355454	5.150092	NR_033691	chr1:23695872-23695931	C1orf213
A_33_P3253960	4.25949	4.097224	AB209247	chr17:34953383-34953442	DHRS11
A_33_P3398697	8.3512945	8.087764	NM_012407	chr22:38471648-38471707	PICK1
A_33_P3361746	6.257674	6.6180677	NM_014112	chr8:116424922-116424863	TRPS1
A_33_P3405429	4.4994073	4.387789	AK125833	chr1:20665630-20665689	VWA5B1
A_23_P110473	5.459496	5.1389337	NM_004536	chr5:70264650-70264591	NAIP
A_24_P167614	9.383031	9.637447	NM_012141	chr13:51939974-51939915	INTS6
A_23_P106973	12.272379	12.093865	NM_006640	chr17:75496557-75496616	SEPT9
A_23_P402787	6.5740767	6.819344	NM_145253	chr16:4659051-4658992	UBALD1
A_24_P56689	5.042549	4.9398	NM_003456	chr16:3169835-3169894	ZNF205
A_23_P398836	5.199451	4.4833727	NM_020784	chr14:52897879-52897820	TXNDC16
A_23_P351342	5.2742515	5.1051993	NM_015164	chr1:16060316-16060375	PLEKHM2
A_23_P61524	5.276688	5.3239174	NM_022903	chr3:49200268-49200209	CCDC71
A_33_P3400699	4.255922	4.6156583	NM_206883	chr7:102993259-102993200	SLC26A5
A_33_P3236921	10.418505	10.295642	NM_001618	chr1:226549220-226549161	PARP1
A_23_P259071	4.3741517	4.3303995	NM_001657	chr4:75320518-75320577	AREG
A_33_P3362770	5.159744	5.200232	NM_018427	chr16:15186426-15186368	RRN3
A_24_P356453	9.711385	9.878318	NM_001040431	chr17:40950487-40950128	COA3
A_24_P169843	7.95766	7.4988337		chr1:182929155-182929094	
A_23_P156667	9.242172	9.295967	NM_002714	chr6:30568347-30568288	PPP1R10
A_24_P77676	12.124558	12.157899	NM_004134	chr5:137891563-137891504	HSPA9
A_32_P150086	5.5270243	5.2145844		chr18:14225703-14225762	ANKRD20A5P
A_23_P369746	4.5709414	4.814845	AF161372	chr17:6917043-6916080	
A_23_P132175	5.9172935	5.861161	NM_023004	chr22:20229029-20228970	RTN4R
A_23_P123974	12.335812	12.204439	NM_012145	chr2:242615249-242615190	DTYMK
A_33_P3312529	2.3221061	2.3900566	NR_028067	chr15:22414326-22414385	OR4N3P
A_24_P134789	6.9891253	7.065818	NM_181688	chr21:46057781-46057840	KRTAP10-10
A_23_P83939	11.385564	11.174517	NM_032796	chrX:16778461-16778520	SYAP1
A_33_P3216869	6.625035	6.8937654	NM_004378	chr15:78640361-78640420	CRABP1
A_23_P101811	6.503675	6.759975	NM_021030	chr19:19821542-19821483	ZNF14
A_23_P163148	7.5110993	7.8164945	NM_022067	chr14:77893216-77893157	VIPAS39
A_24_P196534	9.097973	9.014074	NM_004571	chr21:44453529-44453588	PKNOX1
A_23_P26336	9.160913	9.373037	NM_014153	chr16:11844952-11844893	ZC3H7A
A_23_P53614	5.6254625	5.6817484	NM_006768	chr12:112096551-112093412	BRAP
A_23_P130653	8.116887	8.434114	NM_031429	chr19:12936357-12936298	RTBDN
A_23_P61398	10.368965	10.215844	NM_001001852	chr22:50357570-50357629	PIM3
A_23_P63681	8.204115	8.397781	NM_004969	chr10:94216145-94215371	IDE
A_32_P393316	8.002132	7.719289	NM_001098531	chr12:48128514-48128455	RAPGEF3
A_32_P40375	6.340506	6.2799573		chrX:3740445-3736716	
A_23_P114084	3.885419	3.1666842	NM_000444	chrX:22266184-22266243	PHEX
A_23_P315206	5.27269	5.084015	NM_004059	chr9:131595801-131595742	CCBL1
A_23_P360874	8.714224	8.615656	NM_152892	chr7:102113497-102113556	LRWD1
A_24_P121956	8.577265	9.095693	NM_173804	chr19:55738395-55738336	TMEM86B
A_33_P3235611	6.390515	5.567811	NM_080669	chr17:26721769-26721710	SLC46A1
A_33_P3286988	3.8579106	3.6824799	BF106382	chr15:30337988-30337929	
A_33_P3270451	7.5195417	7.962306	NM_030810	chr6:7883436-7883377	TXNDC5
A_23_P42241	6.1928554	6.3431454	NM_030876	chr6:29323467-29323408	OR5V1
A_24_P85619	6.8768167	6.8742104	AF363068	chr20:46141044-46140985	
A_23_P502464	4.396132	3.8783827	NM_000625	chr17:26083921-26083862	NOS2
A_24_P832426	8.078644	7.8247776	NM_194318	chr13:31905614-31905673	B3GALTL
A_23_P65068	5.7684984	5.4990044	NM_001008394	chr12:104698595-104698654	EID3
A_33_P3413355	6.7357006	5.767686	NM_003654	chr11:45670488-45670429	CHST1
A_32_P722809	5.154352	5.3138766	BC034142	chr2:89999481-89999540	
A_33_P3375451	3.738409	3.8582757	NM_025081	chr14:24885710-24885769	NYNRIN
A_33_P3423949	9.158937	9.399312	NM_005189	chr17:77761302-77761361	CBX2
A_33_P3411805	6.7808757	7.0811005		chr13:19184622-19184563	

A_33_P3305105	4.3555527	4.83436	NM_198315	chr11:123989295-123989354	VWA5A
A_23_P102117	4.332517	4.964322	NM_025216	chr2:219758393-219758452	WNT10A
A_33_P3311740	3.780861	3.6883402	NM_001004309	chr15:90902129-90902188	ZNF774
A_23_P156809	9.168491	9.27916	NM_001127395	chr2:208476430-208476371	METTL21A
A_24_P337657	4.3770437	4.308875	NM_003131	chr6:43148285-43148344	SRF
A_23_P203255	6.569267	6.4619374	NM_006595	chr11:43352112-43356884	API5
A_33_P3222630	6.0705853	6.119978	NM_001029860	chr8:101146035-101145976	FBXO43
A_24_P823684	8.877096	8.939971	NM_007355	chr6:44219211-44219270	HSP90AB1
A_33_P3305472	6.9152927	6.354451	AK123993	chr17:74556378-74556437	
A_32_P749354	7.2490845	7.346287	NM_032325	chr11:65764297-65764238	EIF1AD
A_23_P207967	4.695652	4.2319474	NM_014772	chr18:46389183-46389242	CTIF
A_33_P3358957	6.5668926	6.94683	NM_001004318	chr19:39601419-39601478	PAPL
A_33_P3357773	4.277241	3.5919254		chr22:019160303-019160362	
A_24_P397150	7.2537518	7.1255684	NM_005255	chr4:843550-843491	GAK
A_33_P3237699	5.362596	5.328274	NM_022780	chr2:87002357-87002416	RMND5A
A_24_P60680	5.189084	5.415827	NM_013941	chr6:29408300-29408359	OR10C1
A_33_P3280461	6.6032066	6.7750454	NM_005119	chr1:36769990-36770049	THRAP3
A_24_P295379	8.00912	8.28427	NM_020960	chr9:132902156-132902215	GPR107
A_33_P3238690	7.1345625	7.072112	NM_001127208	chr4:106199618-106199677	TET2
A_23_P122805	8.658955	8.470314	NM_032842	chr7:129805035-129804976	TMEM209
A_33_P3363420	5.287554	4.3176613	NM_174938	chr9:85862416-85862357	FRMD3
A_23_P121506	5.9472656	6.0346365	NM_003704	chr4:2733511-2733570	FAM193A
A_23_P116207	6.219015	5.2496514	NM_020153	chr11:118416530-118416173	IFT46
A_33_P3227212	5.000847	4.8787575		chr1:119976895-119976836	
A_33_P3276455	5.3673244	6.3019156	NM_006618	chr1:202710744-202710685	KDM5B
A_23_P384698	7.185726	6.6611896	NR_103821	chr3:10046661-10046720	EMC3-AS1
A_33_P3390708	5.4664574	5.5481033	NM_013390	chr9:74315618-74315559	TMEM2
A_23_P52639	13.472548	13.633335	NM_004074	chr11:63743901-63743960	COX8A
A_33_P3250068	10.5778055	10.4976425	NM_014748	chr2:27599807-27599866	SNX17
A_23_P48977	9.837172	9.927243	NM_022839	chr15:89021314-89021373	MRPS11
A_24_P376294	7.893499	7.755634	NM_007040	chr19:41812745-41812804	HNRNPUL1
A_33_P3218960	4.4226356	4.7421236	NM_021098	chr16:1271711-1271770	CACNA1H
A_23_P120863	7.342049	7.7246323	NM_004861	chr22:30950697-30950638	GAL3ST1
A_24_P366777	7.6633196	7.8305397	AK075065	chr1:145291283-145291342	NOTCH2NL
A_33_P3288594	2.3221061	2.3900566		chr10:38503136-38503195	LOC100129055
A_33_P3215288	4.9386644	4.9862895	AK128288	chr20:58894494-58894553	LOC284757
A_23_P410040	9.084606	9.23749	NM_173162	chr2:163291767-163291708	KCNH7
A_23_P115842	10.646789	10.507333	NM_018237	chr10:70550998-70551057	CCAR1
A_24_P252846	8.115136	8.123161	NM_138787	chr11:36680594-36680653	C11orf74
A_24_P254346	8.84838	9.068194	NM_006052	chr21:38596089-38596030	DSCR3
A_23_P358470	8.593726	8.507699	NM_152683	chr4:185612697-185612840	PRIMPOL
A_24_P35478	7.4648724	7.439836	NM_019619	chr10:34420439-34408658	PARD3
A_33_P3272080	7.9721475	8.012765	CV575364	chr9:133771324-133771265	QRFP
A_33_P3217819	8.177182	8.360045	NM_057749	chr8:95894420-95894361	CCNE2
A_23_P204269	5.4690814	5.115642	NM_006313	chr12:62799655-62799714	USP15
A_33_P3381328	7.427405	6.993982	NR_027294	chr9:38623172-38623231	FAM201A
A_23_P118406	7.843543	7.9100685	NM_001033046	chr17:80400963-80400904	C17orf62
A_23_P155907	11.047953	11.1594	NM_015143	chr4:99983579-99983638	METAP1
A_33_P3304282	15.393423	15.3013	NM_001019	chr16:18794337-18794278	RPS15A
A_23_P215454	4.0818353	4.398746	NM_001278939	chr7:73483599-73483658	ELN
A_23_P83278	10.53638	10.435975	NM_016410	chr9:33280983-33281042	CHMP5
A_23_P11774	9.985661	9.56349	NM_016037	chr1:38489282-38489341	UTP11L
A_33_P3265606	13.480921	13.618645	NM_002061	chr1:94352869-94352810	GCLM
A_23_P6771	6.5794363	6.163325	NM_014583	chr3:8609688-8609747	LMCD1
A_33_P3284197	9.792053	8.95796	NM_001243766	chr1:46654450-46654391	POMGNT1
A_33_P3231888	7.0937514	5.8205466	NM_020199	chr5:133291966-133291907	C5orf15
A_33_P3401422	4.6612267	4.653941	NM_001128215	chr10:90580151-90580210	LIPM
A_32_P121085	6.641053	5.3632784	NM_001144875	chr5:176928994-176928935	DOK3
A_23_P321703	8.677659	8.419894	NM_004049	chr15:80253252-80253220	BCL2A1

A_24_P251841	7.3200006	7.4543805	NM_030648	chr4:140427751-140427692	SETD7
A_23_P57036	6.8831882	6.6407485	NM_001250	chr20:44757581-44757640	CD40
A_23_P122724	4.2197094	4.533566	NM_004665	chr6:133065225-133065166	VNN2
A_23_P126291	11.9539795	11.773367	NM_003094	chr1:203832818-203834192	SNRPE
A_24_P497226	7.8103285	7.8869476	NM_003161	chr17:58027510-58027569	RPS6KB1
A_23_P39237	8.413404	8.739321	NM_003407	chr19:39899758-39899817	ZFP36
A_23_P204850	10.322798	10.3165455	NM_000321	chr13:49055845-49055904	RB1
A_33_P3227920	7.8504543	8.227465	NM_004696	chr1:110905674-110905615	SLC16A4
A_23_P372660	8.324563	8.223541	NM_001282679	chr9:128126942-128127001	GAPVD1
A_23_P343808	3.9100661	3.8492146	NM_005633	chr2:39213330-39213271	SOS1
A_23_P165722	10.386993	10.776199	NM_004846	chr2:233433793-233433852	EIF4E2
A_33_P3393091	6.0399323	6.277448	DA197111	chr2:132121559-132121618	
A_33_P3399291	9.751883	9.86075	NM_001242840	chr1:228334579-228334638	GUK1
A_24_P918266	7.698199	7.5791	NM_181783	chr12:88592056-88592115	TMTC3
A_33_P3234939	6.589198	6.085234	NM_006631	chr19:9524282-9524223	ZNF266
A_23_P365189	3.8207862	3.280357	NM_015099	chr17:4872611-4872552	CAMTA2
A_33_P3252322	8.121542	8.038796	AK000470	chrX:3811034-3810975	
A_32_P209094	6.418096	6.486295	NM_018291	chr1:60228206-60228265	FGGY
A_23_P258340	14.037533	13.530484	NM_021130	chr7:44839400-44839459	PPIA
A_33_P3222648	7.8433633	8.162231	NM_006282	chr20:43708442-43708501	STK4
A_33_P3365037	9.074101	8.868793	NM_202001	chr19:45917281-45917222	ERCC1
A_23_P310331	10.620754	10.278298	NM_003624	chr19:5916228-5916169	RANBP3
A_33_P3225685	7.9891315	7.9276247	NM_012200	chr11:62382931-62382872	B3GAT3
A_24_P377225	6.631346	5.43677	NM_022832	chr4:53462225-53462166	USP46
A_33_P3342792	6.524379	6.503675		chr3:176136071-176136012	
A_24_P179351	15.191169	15.346972	NM_003295	chr13:45913718-45913659	TPT1
A_24_P317450	9.2285185	9.433584	NR_028496	chr6:159262350-159262291	OSTCP1
A_23_P60166	8.423495	8.523548	NM_022783	chr8:121062582-121062641	DEPTOR
A_33_P3402868	4.239761	3.6562836	NM_000836	chr19:48948128-48948187	GRIN2D
A_24_P116351	10.835084	10.831766	NM_006427	chr14:105223086-105225812	SIVA1
A_23_P203920	3.859378	3.9615278	NM_005086	chr12:26385695-26385754	SSPN
A_23_P252541	5.4067554	5.358402	NM_177403		RAB7B
A_33_P3346552	15.284194	15.031535	NR_003287	chrUn_gl000220:117028-117087	RNA28S5
A_33_P3250953	7.078028	5.16174	NM_032826	chr7:133974151-133974092	SLC35B4
A_24_P391431	7.8657947	8.083696	NM_015975	chrX:77385726-77385667	TAF9B
A_23_P62868	10.341118	10.386993	NM_001001998	chr1:11128107-11126789	EXOSC10
A_24_P376422	8.933968	8.332668	BC035371	chr7:102220041-102219982	
A_23_P503233	12.513302	12.627634	NM_080738	chr1:236647551-236647610	EDARADD
A_32_P215113	5.3796153	5.276688	NM_001017928	chr3:122087034-122081827	CCDC58
A_33_P3295148	7.596458	7.803683	NR_048575	chr10:75491317-75491376	GLUD1P3
A_33_P3247022	8.823733	8.829409	NM_057749	chr8:95892677-95892618	CCNE2
A_23_P209394	7.610586	8.184699	NM_001127184	chr2:202000731-202000790	CFLAR
A_23_P101480	4.221576	3.8299763	NM_014975	chr19:12985699-12985758	MAST1
A_33_P3213857	9.443571	9.141499	NM_001142279	chr13:51522141-51522200	RNASEH2B
A_33_P3241190	9.236638	9.17665	NM_005431	chr7:152343990-152343931	XRCC2
A_24_P83183	7.665114	7.6290717	NM_005663	chr4:1984828-1984769	NELFA
A_32_P114284	4.3218455	4.342977	NM_001079526	chr2:213864595-213864536	IKZF2
A_33_P3385681	3.8322704	4.001942	AK128224	chr2:129136116-129136057	LOC100130768
A_33_P3256894	6.6736746	6.6894445	NM_001169107	chr10:46252526-46252585	FAM21C
A_33_P3372389	4.675468	4.7520556	NM_015102	chr1:5964844-5964785	NPHP4
A_33_P3290830	5.1267066	5.4586587	NR_026916	chr21:14424272-14424331	ANKRD30BP2
A_23_P95029	7.1825285	6.4743757	NM_021021	chr8:121550176-121550117	SNTB1
A_33_P3314151	6.396752	6.4107804	NM_001144958	chr12:3726180-3726121	EFCAB4B
A_23_P7535	4.7364006	4.8735466	NM_022304	chr5:175110791-175110850	HRH2
A_23_P421011	5.1213017	4.245704	NM_030929	chr10:102825270-102825329	KAZALD1
A_33_P3413053	10.229553	10.035442	NM_002484	chr16:10863148-10863207	NUBP1
A_23_P8558	11.368754	11.948728	NM_148912	chr7:73151670-73151611	ABHD11
A_23_P13183	9.861961	9.980201	NM_000401	chr11:44266287-44266346	EXT2

A_24_P250227	3.9664583	4.422182	NM_021724	chr17:38251258-38251199	NR1D1
A_33_P3373765	3.7932866	3.514813	NM_000797	chr11:640644-640703	DRD4
A_24_P542375	14.818495	14.73961	NM_002823	chr2:232578073-232578132	PTMA
A_24_P192434	10.571036	10.032239	NM_017489	chr8:73959665-73959724	TERF1
A_33_P3642648	5.1866903	4.651767	NM_006635	chr19:57805041-57805100	ZNF460
A_33_P3282075	6.658374	5.8186913	NM_001258248	chr17:45922339-45922280	SP6
A_33_P3380618	4.4782057	5.1267066	NM_005529	chr1:22173072-22173013	HSPG2
A_23_P17706	7.2371225	7.11322	NM_014339	chr22:17590940-17590999	IL17RA
A_23_P201368	8.374377	7.9127927	NM_004388	chr1:85020831-85020772	CTBS
A_23_P6344	11.399037	11.133199	NM_022044	chr22:21998511-21998570	SDF2L1
A_33_P3326588	10.117357	10.885145	NM_003840	chr8:22993186-22993127	TNFRSF10D
A_24_P282309	7.386058	7.595692	NM_133337	chr10:95162101-95162042	MYOF
A_23_P71727	12.875509	12.997727	NM_001827	chr9:91931523-91931582	CKS2
A_33_P3419557	4.787607	5.123822	NM_001005516	chr3:98110416-98110475	OR5K3
A_33_P3375528	3.7350247	4.2984324	NM_152622	chr5:56233467-56233408	MIER3
A_23_P55468	7.8218737	8.00027	NM_013337	chr17:904651-904710	TIMM22
A_33_P3339441	3.704312	4.4333725		chr1:4009335-4009394	LOC728716
A_33_P3227774	7.3139334	7.4791174	NR_102747	chr15:82975465-82975524	LOC727751
A_23_P121356	10.138596	10.400059	NM_020235	chr3:107524534-107524593	BBX
A_33_P3355266	6.3690853	6.0626493	NM_022164	chr1:32052808-32052867	TINAGL1
A_23_P411612	7.553856	7.9633856	NM_207344	chr12:56864656-56864715	SPRYD4
A_24_P400507	6.7673273	7.028169	NM_173654	chr3:69025317-69025258	EOGT
A_23_P257593	6.3323393	6.1312428	NM_058169	chr12:12618917-12618976	LOH12CR1
A_23_P61371	8.183846	8.843342	NM_198282	chr5:138855656-138855597	TMEM173
A_23_P130735	4.6336136	4.321289	NM_014037	chr19:49793142-49793083	SLC6A16
A_33_P3291650	3.8826132	3.9356117	AY271360	chr2:90025322-90025381	
A_23_P66767	3.6191828	3.5740998	NM_153338	chr17:4461325-4461266	GGT6
A_33_P3225086	7.975233	8.027766	NM_057174	chr11:45935433-45935374	PEX16
A_33_P3271594	5.951279	5.887148	NM_032546	chr2:27530243-27530302	TRIM54
A_23_P1492	8.351512	8.393813	NM_021732	chr10:99437351-99437292	AVPI1
A_33_P3590279	13.513447	13.520017		chr17:062385763-062385704	
A_23_P217564	7.337954	7.2334247	NM_004458	chrX:108885329-108885270	ACSL4
A_33_P3300877	10.438438	10.7528		chr17:081012921-081012862	
A_33_P3299754	7.6445456	8.045162	NM_001256412	chr10:27815759-27815818	RAB18
A_33_P3335248	7.3186474	7.4045157	NM_001039707	chr9:139299621-139299562	SDCCAG3
A_23_P161481	4.47001	4.7876883	NM_014431	chr10:72327843-72327902	PALD1
A_23_P89799	8.862941	9.172905	NM_006111	chr18:47309991-47309932	ACAA2
A_32_P155631	10.38434	10.069782	NM_003002	chr11:111965866-111965925	SDHD
A_33_P3351207	7.6330523	7.38148	NR_015363	chr9:41953643-41953584	MGC21881
A_23_P354208	8.029421	7.6229615	NM_152348	chr17:1641714-1641773	WDR81
A_24_P315184	12.896294	12.983658	AB209471	chr2:204055610-204055672	NBEAL1
A_23_P85269	2.722268	2.3900566	NM_133378	chr2:179391858-179391799	TTN
A_23_P251705	4.556199	4.903976	NM_015185	chrX:62875523-62875464	ARHGEF9
A_33_P3394599	7.2618084	7.4288006	NM_006339	chr19:3578051-3578110	HMG20B
A_33_P3268466	4.0126705	4.304839	NM_004870	chr17:7487223-7487282	MPDU1
A_23_P301572	3.6130643	3.8221283	NM_015688	chr4:17634145-17634086	FAM184B
A_23_P96369	2.3221061	2.3900566	NM_018015	chrX:105922517-105922576	CXorf57
A_33_P3286349	4.2574086	4.116638	NM_001256716	chr19:55670224-55670165	DNAAF3
A_33_P3366987	4.744542	5.443965	NM_145290	chr4:22422569-22422510	GPR125
A_33_P3398091	6.5766582	6.55236	NM_001130031	chr19:9763360-9763301	ZNF562
A_33_P3234010	2.5749693	3.0371757		chr19:45041376-45041317	CEACAM22P
A_33_P3341189	10.081409	9.962486	NM_003336	chrX:118717243-118717302	UBE2A
A_23_P55682	2.8298392	2.3900566	NM_023926	chr19:58595438-58595379	ZSCAN18
A_23_P105368	4.1801043	4.6086936	AK001057	chr12:129298442-129298383	
A_33_P3294504	4.3129134	4.778125	NM_001159397	chr1:157768011-157767952	FCRL1
A_33_P3427239	5.577944	5.8436556	BC035129	chr4:6987771-6987712	LOC100134937
A_24_P694760	9.320981	9.203926	NM_178040	chr12:1604999-1605058	ERC1
A_32_P159612	5.1499505	5.3979297	NM_183377	chr17:31340342-31340283	ASIC2
A_33_P3259722	3.9529974	3.6015873	NM_032125	chr1:27657216-27657275	TMEM222

A_24_P161463	12.431994	12.412671	NR_003605	chr20:47905716-47905775	ZFAS1
A_24_P151834	7.3973966	7.3823924	BC052308	chr17:4097851-4097792	ANKFY1
A_23_P150741	7.8691874	7.537962	NM_001286577	chr11:73724032-73723973	C2CD3
A_33_P3394605	8.732461	8.905959	NM_006339	chr19:3578660-3578719	HMG20B
A_24_P338145	8.215183	7.7783127	NM_013442	chr9:35101776-35101717	STOML2
A_33_P3291394	8.218246	8.081989	NM_007266	chr2:27873264-27873323	GPN1
A_23_P55706	10.3993	10.717293	NM_006509	chr19:45541385-45541444	RELB
A_23_P39131	5.831581	5.317926	NM_015710	chr19:48254779-48254838	GLTSCR2
A_23_P150281	5.119916	5.003954	NM_001258320	chr11:44954519-44954460	TP53I11
A_24_P250650	8.639809	8.750832	NM_013412	chr2:114400839-114400898	RABL2A
A_23_P138253	10.445537	10.541378	NM_015607	chr1:153617779-153617838	CHTOP
A_33_P3221971	5.821155	5.5884976	NM_001013657	chr15:90785253-90785312	GDPGP1
A_33_P3851513	8.028761	8.023833	NM_194282	chr4:83847040-83846981	LIN54
A_23_P98310	9.203494	9.19384	NM_001326	chr11:33106284-33106225	CSTF3
A_23_P27167	8.199427	8.329837	NM_002936	chr2:3596301-3596242	RNASEH1
A_23_P2601	14.016186	13.998194	NM_003299	chr12:104341536-104341595	HSP90B1
A_24_P177553	4.0959606	3.2859964	BU963192	chr11:122928691-122928632	
A_23_P68327	9.75628	9.929283	NM_080667	chr2:55772050-55772109	CCDC104
A_33_P3407746	6.4673595	6.8597803	NM_177968	chr2:44461288-44461347	PPM1B
A_23_P207058	8.265329	8.541426	NM_003955	chr17:76353186-76353127	SOCS3
A_33_P3284557	9.227913	9.015497	NM_016653	chr2:174131452-174131511	ZAK
A_33_P3248024	4.4393673	4.763158	NR_046322	chr2:96874597-96874656	STARD7-AS1
A_24_P121271	8.994227	9.198666	NM_007007	chr12:69656195-69656254	CPSF6
A_33_P3326275	4.7298727	4.5866156	AK026773	chr2:47799886-47799945	
A_33_P3404651	6.5464697	6.70734	NM_020458	chr2:47301124-47301183	TTC7A
A_24_P12413	8.91461	9.16416	NM_012288	chr6:52362614-52362555	TRAM2
A_32_P15421	4.532748	4.60956	NM_152267	chr22:31602122-31602181	RNF185
A_24_P92163	5.448083	5.334353		chr22:036933622-036933563	
A_24_P55295	9.526562	9.720745	NM_000165	chr6:121770327-121770386	GJA1
A_24_P109082	6.452161	5.9021015	NM_020935	chr2:219315159-219315100	USP37
A_24_P330773	7.1808662	6.5383635	NM_005831	chr17:46941924-46941983	CALCOCO2
A_23_P77000	5.559658	5.9016857	NM_014909	chr14:77249212-77249271	VASH1
A_24_P631848	3.9258654	4.4082756	BC036435	chr1:17200364-17200423	LOC440570
A_33_P3258467	7.8364506	6.7212496	NM_178313	chr2:54889385-54889444	SPTBN1
A_24_P28977	8.737499	8.513751	NM_003304	chr3:142526520-142526579	TRPC1
A_33_P3215193	6.007695	5.46975	NM_022787	chr1:10041167-10041226	NMNAT1
A_32_P34444	11.326168	11.121452	NM_025135	chr18:34359879-34359938	FHOD3
A_23_P34126	4.4287577	5.0768304	NM_001711	chrX:152773933-152773992	BGN
A_23_P102122	12.720637	12.652946	NM_152862	chr2:219118658-219118717	ARPC2
A_23_P103398	8.483089	8.24066	NM_000447	chr1:227083660-227083719	PSEN2
A_23_P251927	9.533114	9.470915	NM_017812	chr7:132470202-132470143	CHCHD3
A_23_P69840	7.896909	7.902265	NM_080670	chr5:139948223-139948282	SLC35A4
A_23_P152782	6.7339025	6.903557	NM_005533	chr17:41165122-41165181	IFI35
A_23_P215634	11.600517	12.233913	NM_001013398	chr7:45952667-45952608	IGFBP3
A_23_P206324	6.355789	6.254411	NM_031463	chr16:84156601-84156542	HSDL1
A_23_P4592	4.8111076	4.6148696	NM_001245	chr19:52023094-52023035	SIGLEC6
A_32_P94001	8.209272	8.078494	NR_003612	chr4:183959310-183959369	FAM92A1P2
A_23_P394166	10.879364	10.652139	NM_013354	chr8:17086944-17086885	CNOT7
A_24_P922631	4.1770935	4.2007523	NM_001102609	chr5:169673051-169673110	C5orf58
A_23_P26557	8.732645	8.577486	NM_025108	chr16:2514795-2514854	C16orf59
A_33_P3214239	5.5103707	5.158676		chr20:57394059-57394000	GNAS-AS1
A_23_P106024	4.113659	4.7290406	NM_002226	chr14:105608733-105608674	JAG2
A_23_P104073	6.3850484	5.1953154	NM_002960	chr1:153520057-153519998	S100A3
A_33_P3232993	4.704277	4.3240037	NM_001039469	chr11:63676425-63676484	MARK2
A_23_P12147	4.8298655	5.1781707	NM_152485	chr1:209956256-209956197	C1orf74
A_23_P113161	3.8713975	3.119477	NM_030806	chr1:184597996-184598055	C1orf21
A_23_P28375	6.5859013	6.8967237	NM_178439	chr2:70106612-70106671	GMCL1
A_23_P124837	5.5352955	5.364086	NM_002332	chr12:57605946-57606005	LRP1
A_23_P62605	14.921533	14.780634	NM_000975	chr1:24021173-24021232	RPL11

A_33_P3655815	4.2007523	4.251352	BQ322426	chr12:49061730-49061789	SNORA2B
A_24_P297551	2.455706	2.3900566	NM_178539	chr12:62102619-62102560	FAM19A2
A_24_P175989	9.927243	10.059064	NM_016226	chr12:110933835-110930996	VPS29
A_23_P338519	7.255847	7.7858586	NM_020345	chr3:23934716-23934657	NKIRAS1
A_33_P3407364	3.7680402	4.113488	NM_032946	chrX:101096050-101095991	NXF5
A_33_P3284108	3.7613318	4.282284	NM_006178	chr17:44720564-44720623	NSF
A_23_P252740	9.221853	8.964469	NM_024094	chr8:120847137-120847079	DSCC1
A_23_P113972	7.2329884	7.444126	NM_001024924	chr4:56768536-56768595	EXOC1
A_32_P135348	8.7887	8.713093	NM_033394	chr2:160088949-160089008	TANC1
A_23_P105392	12.752876	12.48328	NM_006431	chr12:69995181-69995240	CCT2
A_23_P113317	6.244502	6.069144	NM_177938	chr3:49043252-49043507	P4HTM
A_33_P3231833	4.92681	4.978095		chr20:044482603-044482544	
A_23_P54376	8.498657	8.014406	NM_004809	chr15:74275622-74275563	STOML1
A_24_P323104	12.01219	11.341736	NM_005219	chr5:140894648-140894589	DIAPH1
A_33_P3305482	8.366688	7.8341594	NM_020365	chr1:45316544-45316485	EIF2B3
A_33_P3358243	9.032493	9.057674	NM_020702	chr9:34369047-34368988	KIAA1161
A_33_P3408034	4.4671946	5.3119907		chr2:228734889-228734830	
A_33_P3322430	13.743806	13.853955		chr17:003553740-003553799	
A_24_P131222	5.4708385	5.048872	NM_022089	chr1:17313405-17313346	ATP13A2
A_24_P518369	6.137902	6.289295		chr4:146545763-146545704	
A_32_P751535	11.83872	11.834877		chr1:168907735-168907794	XLOC_014512
A_24_P185394	11.642081	11.402478	NM_019884	chr19:42734408-42734349	GSK3A
A_33_P3502311	6.2338266	6.16254	XR_248076	chr15:028517921-028517861	LOC100996379
A_33_P3291349	7.169269	7.0531864	XM_005258293	chr18:33605629-33605570	RPRD1A
A_23_P90369	11.587711	11.4088955	NM_001033026	chr19:1009717-1009658	TMEM259
A_23_P64611	9.104904	9.055459	NM_176798	chr11:73008625-73008684	P2RY6
A_33_P3264940	4.0251155	3.8810165		chrX:047603225-047603284	
A_33_P3416762	11.315578	11.15636	NM_006330	chr8:54959263-54959204	LYPLA1
A_23_P206107	4.0206385	3.8717923	NM_001099436	chr15:75128999-75128940	ULK3
A_24_P288298	3.7390807	3.5438209	NM_002255	chr19:55325337-55325396	KIR2DL4
A_23_P210619	10.402258	10.3993	NM_016407	chr20:55093726-55093785	RTFDC1
A_33_P3417820	9.858577	9.672739	NM_024313	chr22:38089401-38089460	NOL12
A_23_P436158	4.1058817	2.9422896	NM_012465	chr10:98125121-98125062	TLL2
A_33_P3327245	6.02244	5.9296026	NM_031915	chr13:50059800-50059859	SETDB2
A_33_P3369266	3.9090233	4.234363		chr1:000025909-000025850	
A_24_P359117	7.779931	7.710331	NM_001002909	chr17:42472986-42472927	GPATCH8
A_23_P502343	6.143322	6.4752893	NM_025220	chr20:3649151-3649092	ADAM33
A_23_P117546	7.2407427	7.469075	NM_006939	chr14:50584382-50584323	SOS2
A_24_P69379	6.534383	6.6703277	NM_022900	chr7:94185895-94185954	CASD1
A_33_P3214690	6.680815	6.5508084	NM_014932	chr3:173998992-173999051	NLGN1
A_24_P353619	3.9617648	3.775355	NM_000478	chr1:21903084-21903884	ALPL
A_33_P3358740	8.740137	8.712727	NM_145798	chr17:45884793-45884734	OSBPL7
A_24_P303454	5.486904	4.378404	NM_012454	chr6:155578174-155578233	TIAM2
A_23_P24709	7.4026613	7.514761	NM_002556	chr11:59342359-59342300	OSBP
A_33_P3254510	6.0023336	6.0477314		chr18:012056847-012056788	
A_23_P35316	4.5227675	4.9281816	NM_020394	chr1:247150771-247150712	ZNF695
A_23_P11279	7.4288006	7.757888	NM_018466	chrX:110928330-110931172	ALG13
A_23_P312150	6.759199	6.0811105	NM_001956	chr1:41944515-41944456	EDN2
A_23_P119362	10.9396515	10.78867	NM_001425	chr19:48832675-48832734	EMP3
A_23_P26468	7.2263336	7.5475903	NM_001278720	chr16:726855-726988	RHBDL1
A_33_P3339825	4.382963	4.312263	NM_206538	chr19:50984174-50984233	EMC10
A_23_P71649	4.3523912	4.704277	NM_005592	chr9:113563075-113563134	MUSK
A_32_P114574	12.649714	12.396936	NM_014412	chr1:174979115-174979174	CACYBP
A_24_P134195	6.1811056	6.331771	NM_001020818	chr19:54377897-54377956	MYADM
A_23_P46455	7.278142	7.0458136	NM_014280	chr1:28526929-28526870	DNAJC8
A_24_P849801	8.935998	8.902313	NM_000983	chr1:6246098-6246039	RPL22
A_24_P289383	7.675664	8.017714	NM_017780	chr8:61779398-61779457	CHD7
A_23_P109907	14.398101	14.646114	NM_175924	chr3:121707033-121706974	ILDR1
A_33_P3281333	9.053422	9.33115	NM_006750	chr16:69342827-69342886	SNTB2

A_33_P3348091	7.1557355	7.286308	NM_001042544	chr19:41125304-41125363	LTBP4
A_23_P215669	3.789216	4.7148485	NM_032959	chr7:102277685-102277626	POLR2J2
A_23_P9465	12.908557	13.612024	NM_004957	chr9:130575924-130575983	FPGS
A_23_P65768	10.701667	10.363949	NM_016304	chr15:55474228-55474169	RSL24D1
A_33_P3229328	4.911075	5.1430893	NM_001143988	chr1:108993343-108993401	NBPF6
A_24_P399942	8.944494	7.463331	NM_173694	chrX:138808858-138808799	ATP11C
A_23_P2661	11.560005	11.467915	NM_015646	chr12:69054229-69054288	RAP1B
A_23_P129786	11.003538	10.847843	NM_001005291	chr17:17715478-17715419	SREBF1
A_24_P665504	5.0350046	5.26474	NR_028327	chr1:664729-664670	LOC100133331
A_23_P63798	11.3043995	11.360216	NM_001300	chr10:3821446-3821387	KLF6
A_23_P427703	12.175588	12.368598	NR_001447	chr16:56652580-56652639	MT1L
A_33_P3375673	13.671231	13.7675		chr17:004608351-004608292	
A_32_P8813	5.743139	4.831443	NR_024433	chr15:57599800-57599859	LINC00926
A_33_P3419938	13.512057	13.640844	NM_000551	chr3:10193621-10193680	VHL
A_23_P80694	6.912651	6.5488605	NM_022899	chr3:53901581-53901522	ACTR8
A_24_P71439	6.9966755	7.280497	NM_015317	chr2:20453583-20451419	PUM2
A_33_P3412556	4.3735623	4.6490674		chr13:20753271-20753212	XLOC_014512
A_33_P3296169	7.634221	7.45415	NM_031942	chr2:174232387-174232446	CDCA7
A_33_P3357082	6.5820127	6.5396442	NM_024770	chr2:172187129-172187070	METTL8
A_23_P94857	6.508426	6.567596	NM_016219	chr9:140002868-140002927	MAN1B1
A_33_P3245631	4.9720135	5.3250384	NM_001080494	chr1:51768238-51768179	TTC39A
A_33_P3398877	5.578723	5.724071		chr5:000851156-000851097	
A_23_P86731	8.326187	8.027285	NM_005674	chr10:44051978-44051919	ZNF239
A_23_P90484	6.2725973	5.8049917	NM_017827	chr19:39406083-39406024	SARS2
A_33_P3396434	11.3815155	11.559139		chr1:036788514-036788455	
A_33_P3240532	7.236234	7.645057	NM_015149	chr1:183897605-183897664	RGL1
A_33_P3262515	9.843386	9.732883	NM_007368	chr13:114747318-114747259	RASA3
A_24_P184305	5.232784	5.1663756	NM_024649	chr11:66300734-66300793	BBS1
A_33_P3396591	4.062729	3.4154456	NM_001271168	chr20:2819902-2819843	PCED1A
A_23_P81717	5.2921166	5.920101	NM_024919	chr6:168457299-168457240	FRMD1
A_23_P58862	5.6057014	5.7535534	NM_020819	chr6:71212478-71232258	FAM135A
A_33_P3238685	7.508208	7.7934647	NM_001127208	chr4:106193920-106193979	TET2
A_23_P394567	4.9331098	5.172639	NM_020853	chr12:13236125-13236184	KIAA1467
A_23_P120822	6.336301	6.344873	NM_016327	chr22:24921819-24921878	UPB1
A_23_P111947	4.5481334	4.8881063	NM_004063	chr8:95140517-95140458	CDH17
A_24_P90900	8.554045	9.10108	NM_001907	chr16:67964417-67964358	CTRL
A_33_P3396951	7.119169	8.241981	NM_004331	chr8:26252758-26252817	BNIP3L
A_23_P123424	5.2520814	5.43723	NM_000749	chr8:42591664-42591723	CHRNB3
A_23_P347528	8.413242	8.161865	NM_005653	chr12:51488723-51488664	TFCP2
A_23_P145397	9.895347	10.261396	NM_005190	chr6:99991411-99991352	CCNC
A_23_P408108	6.6082516	6.468895	NM_006980	chr7:91502411-91502352	MTERF
A_33_P3286372	5.0414834	4.6622176	XM_005246164	chr2:10351473-10351532	C2orf48
A_32_P150391	2.3221061	4.9629045	NM_001105520	chr17:6556004-6556063	C17orf100
A_23_P215790	8.3460865	8.670724	NM_005228	chr7:55274841-55274900	EGFR
A_23_P107684	6.6672363	6.5521784	NM_014347	chr19:58984466-58984525	ZNF324
A_23_P385771	7.1186676	7.156895	NM_152911	chr10:135205077-135205136	PAOX
A_24_P161581	5.3466315	5.658328	NM_001145176	chr19:55949046-55948987	SHISA7
A_23_P371254	3.5807562	3.7914321	NM_176884	chr12:11244304-11244245	TAS2R43
A_33_P3274001	6.696031	6.6821275		chr6:108939648-108939589	
A_33_P3380529	3.3300316	2.3900566	NM_001282786	chr10:25145902-25145843	PRTFDC1
A_23_P200780	3.6007204	4.495825	NM_003243	chr1:92148947-92148888	TGFBR3
A_23_P155979	4.811835	4.9025173	NM_001963	chr4:110932689-110932748	EGF
A_33_P3298206	2.3221061	2.3900566	NM_020958	chr14:94693632-94693691	PPP4R4
A_23_P33364	10.431498	10.232708	NM_001009555	chr4:152041658-152041599	SH3D19
A_33_P3724750	5.991813	5.928192	BC014063	chr3:65707579-65707520	
A_23_P107073	11.1904335	11.337995	NM_002945	chr17:1801166-1801225	RPA1
A_33_P3244956	8.370982	8.2853	NM_020645	chr11:9005482-9005423	NRIP3
A_23_P219144	12.370544	12.128654	NM_007096	chr9:36211781-36211840	CLTA
A_23_P89921	7.583286	7.2618084	NM_013256	chr19:44980178-44980119	ZNF180

A_33_P3333708	8.532419	8.8117485		chrX:006144427-006144368	
A_32_P213349	5.7687664	5.8043594		chr2:162279245-162279186	
A_23_P92499	2.3221061	2.8412242	NM_003264	chr4:154626406-154626465	TLR2
A_33_P3319581	9.184313	9.545284	NM_001013690	chr12:52213847-52213788	FIGNL2
A_24_P414269	10.52139	10.519317	NM_005787	chr3:183960286-183960227	ALG3
A_24_P294703	7.290197	7.4547415	NM_130809	chr5:126889970-126890029	PRRC1
A_23_P202269	2.3221061	2.3900566	NM_020987	chr10:61788365-61788306	ANK3
A_32_P9575	6.801798	6.847789	NM_032351	chr17:36454586-36455369	MRPL45
A_33_P3397579	7.4312563	7.3206153	BC040611	chr8:58896451-58896510	
A_23_P70566	4.3576393	4.294564	NM_022110	chr6:32097350-32097291	FKBPL
A_33_P3316983	9.034696	8.920211	NM_001195304	chr10:112660189-112660130	BBIP1
A_23_P416608	8.045444	8.613993	NM_013995	chrX:119571253-119571194	LAMP2
A_24_P15391	4.8179293	4.15767	NM_001135649	chr2:88747798-88747739	FOXI3
A_23_P107653	11.017508	11.165808	NM_001014763	chr19:51848634-51848575	ETFB
A_23_P47790	7.799367	7.649498	NM_005371	chr12:58162716-58162657	METTL1
A_23_P218751	7.530717	7.3362503	NM_053004	chr22:19776111-19776052	GNB1L
A_24_P392958	5.216123	5.260546	NM_144628	chr20:416773-416714	TBC1D20
A_23_P201386	9.423573	9.652121	NM_012137	chr1:85784533-85784474	DDAH1
A_23_P87545	11.218395	11.795233	NM_021034	chr11:319914-319855	IFITM3
A_23_P69310	2.3221061	2.3900566	NM_003965	chr3:46450722-46450781	CCRL2
A_24_P16249	6.378084	6.6291075	NM_033273	chr7:57188441-57188382	ZNF479
A_33_P3388397	5.8137035	5.9890947	NM_153212	chr1:35229266-35229325	GJB4
A_23_P1043	8.764399	8.802715	NM_018265	chr1:200884343-200884402	C1orf106
A_33_P3258013	6.153989	6.5453615	NM_001270449	chr12:109928340-109928399	UBE3B
A_23_P118607	7.717366	7.5004487	NM_025267	chr17:41107916-41107240	AARSD1
A_23_P64499	4.8864484	5.4563956	NM_053005	chr11:1491219-1491160	MOB2
A_23_P29836	9.225712	9.219402	NM_144638	chr3:44907045-44907104	TMEM42
A_33_P3375185	5.3052173	5.1844425	NM_001013632	chr1:45271755-45271696	TCTEX1D4
A_23_P112406	8.45166	8.281007	NM_012087	chr9:135933512-135933571	GTF3C5
A_33_P3334575	9.888103	10.171531		chr3:172143366-172143307	XLOC_014512
A_23_P101623	4.136405	4.360976	NM_022103	chr19:56952634-56952575	ZNF667
A_24_P203964	7.0773544	7.134044		chr5:69493105-69492762	SMA4
A_23_P41948	8.917393	9.265607	NM_017785	chr5:169031233-169031292	SPDL1
A_33_P3395636	7.630849	7.3411303		chr7:064800206-064800147	
A_23_P137248	5.7180953	5.642849	NR_028062	chrY:7248885-7248944	PRKY
A_23_P45536	2.3221061	2.3900566	NM_005369	chrX:138664211-138664152	MCF2
A_32_P19806	4.826378	3.6305912	NM_001277115	chr7:21940916-21940975	DNAH11
A_33_P3391796	4.2849064	2.9719877	NM_005450	chr17:54672456-54672515	NOG
A_33_P3527721	8.040771	7.6820354	AK094436	chr18:8767695-8767754	LOC284219
A_24_P935682	4.8543344	4.6130066	AY358248	chr11:95988484-95988425	LOC100131541
A_23_P77593	4.0984683	4.532382	NM_018052	chr16:70729456-70726825	VAC14
A_33_P3401826	4.633498	4.9720135	NM_207315	chr2:6988526-6988467	CMPK2
A_23_P106117	3.893187	3.72952	NM_007374	chr14:60978278-60978337	SIX6
A_33_P3352906	4.954936	5.0504913	NR_002813	chr4:21845023-21844964	KCNIP4-IT1
A_24_P337334	7.860906	7.763225	NM_001012732	chr4:183812095-183812036	DCTD
A_33_P3370094	3.0536704	3.4198833	NM_007289	chr3:154898216-154898275	MME
A_32_P69849	2.3221061	2.3900566	NM_001012978	chrX:101408900-101408841	BEX5
A_33_P3345936	13.182049	12.957378	NM_015603	chr19:47774918-47774977	CCDC9
A_24_P181295	4.410121	3.9732277	NM_001001872	chr14:58598313-58598254	C14orf37
A_33_P3666346	4.5849743	4.035756	NM_015446	chr1:247058043-247057984	AHCTF1
A_33_P3353502	9.249154	8.990462	NM_182797	chr20:9319625-9319684	PLCB4
A_23_P70719	3.9918125	4.0598497	NM_000426	chr6:129833622-129835560	LAMA2
A_33_P3369505	6.1656246	6.40654	NM_001031740	chr1:38261451-38261510	MANEAL
A_23_P338952	8.150939	8.555744	NM_022753	chr1:33324215-33324274	S100PBP
A_23_P78170	7.3914967	6.9736342	NM_014520	chr17:4442814-4442755	MYBBP1A
A_24_P127192	5.3020725	5.133809		chr2:049142724-049142665	
A_33_P3268124	5.9269743	6.2051687	NM_018293	chr3:88193550-88193609	ZNF654
A_23_P88643	2.3221061	2.3900566	NM_001005241	chr15:22383159-22383218	OR4N4
A_23_P36928	10.124041	9.967492	NM_015972	chr13:28197204-28197263	POLR1D

A_23_P151805	2.6353612	2.3900566	NM_006329	chr14:92336140-92336081	FBLN5
A_24_P331704	9.349894	8.890718	NM_182507	chr12:52563544-52563485	KRT80
A_32_P73452	6.828534	6.885841	NM_020959	chr19:17434100-17434041	ANO8
A_33_P3386955	10.95074	10.794189	NM_031902	chr2:95753031-95752972	MRPS5
A_33_P3312564	12.223671	12.375852		chr1:175266308-175266367	
A_23_P146637	10.43281	10.716174	NM_005866	chr9:34635009-34634950	SIGMAR1
A_23_P103070	10.504837	11.014345	NM_003405	chr22:32353138-32353197	YWHAH
A_24_P32520	7.558831	7.464118	NM_001077239	chr11:117156011-117156070	RNF214
A_33_P3278560	4.738442	5.207967	NM_001010879	chr19:58102575-58102634	ZIK1
A_23_P74843	11.135401	11.248809	NM_018060	chr1:220320905-220320964	IARS2
A_24_P385280	11.319585	11.703935	NM_000696	chr1:165632035-165631976	ALDH9A1
A_23_P139260	7.7212796	7.5686164	NM_183233	chr11:2946335-2946394	SLC22A18
A_33_P3332547	7.6102395	8.00259	NM_001197113	chr3:159606672-159606731	IQCJ-SCHIP1
A_24_P16214	6.686841	6.7351837		chr19:36806544-36806485	LINC00665
A_33_P3279831	8.3543005	9.675963	NM_006813	chr6:89793857-89793916	PNRC1
A_33_P3230738	15.1751585	15.284194		chr17:015948236-015948295	
A_24_P85200	7.959303	8.392894	NM_194278	chr14:74182252-74182193	ELMSAN1
A_33_P3407105	5.3119907	5.3775578	NM_017798	chr20:61834205-61834146	YTHDF1
A_33_P3235098	5.0224442	4.722272	NR_075100	chr10:89127618-89127677	NUTM2D
A_23_P103131	10.080197	9.721608	NM_019008	chr22:39912040-39912099	MIEF1
A_33_P3520693	4.5672026	4.539672	NR_036538	chr3:149690360-149690419	LOC646903
A_23_P69468	13.254755	13.117857	NM_004547	chr3:120321089-120321148	NDUFB4
A_24_P827	5.177889	5.392615	NM_003599	chr6:44982622-44982563	SUPT3H
A_23_P93046	5.490097	5.2823396	NM_007277	chr5:464408-464467	EXOC3
A_32_P78491	4.9045753	4.368519	NM_004956	chr7:13931096-13931037	ETV1
A_33_P3290622	6.960627	7.302538	NM_016484	chrX:69507134-69506916	PDZD11
A_33_P3303355	7.9507046	8.301263		chrX:103358074-103358133	ZCCHC18
A_23_P123402	9.943419	10.111533	NM_184086	chr8:67064697-67064756	TRIM55
A_33_P3256680	5.1211843	5.376808	NM_004225	chr8:8642058-8641999	MFHAS1
A_33_P3333642	9.459723	9.484048	AK131315	chr11:59036408-59036349	
A_33_P3283237	7.8581486	7.751709	NM_206923	chrX:21875319-21875378	YY2
A_23_P204847	2.9240675	2.3900566	NM_002298	chr13:46700396-46700337	LCP1
A_32_P131050	4.995269	4.4527254	NM_021964	chr3:124944735-124944676	ZNF148
A_24_P362572	10.593769	11.027037	NM_014670	chr2:201687619-201687678	BZW1
A_33_P3226192	3.4984162	3.4915745	NM_016346	chr15:72106902-72106961	NR2E3
A_33_P3461039	4.042398	3.3739667	AK055712	chr12:107347872-107347813	
A_33_P3232544	5.283816	5.2858086			
A_24_P781757	3.675928	2.3900566	NR_015434	chr1:1336593-1336652	LOC148413
A_23_P88893	10.204974	10.323662	NM_017702	chr16:90025597-90025656	DEF8
A_33_P3229527	5.422309	5.8602753		chr17:058073036-058072977	
A_24_P409402	10.1807995	10.307714		chr5:060686671-060686612	
A_24_P289636	8.167431	8.225279	AK098314	chr15:90820324-90820383	
A_23_P72096	2.3848786	2.3900566	NM_000575	chr2:113532421-113532362	IL1A
A_24_P168925	10.515417	9.88221	NM_001143981	chrX:109917279-109917220	CHRDL1
A_32_P150142	9.469839	10.534589		chr7:56564630-56564689	DKFZp434L192
A_24_P333326	6.5727625	6.5450873	NM_203355	chr14:39762553-39763230	CTAGE5
A_24_P332623	8.5856495	8.572351	NR_027466	chr13:75812141-75812082	CTAGE11P
A_23_P158041	2.3221061	2.3900566	NM_001170	chr9:33395133-33387089	AQP7
A_23_P417113	6.55177	6.5118184	BC011923	chr7:155493680-155493739	RBM33
A_33_P3251332	5.886187	5.9318953	NM_015473	chr14:31762417-31762358	HEATR5A
A_23_P378526	6.5895023	6.518583	NM_016434	chr20:62327408-62327467	RTEL1
A_32_P210202	9.3168335	9.137022	NM_203394	chr12:77415579-77415520	E2F7
A_23_P42257	9.912215	10.209457	NM_003897	chr6:30711371-30711312	IER3
A_23_P64828	2.9059443	3.6881678	NM_002534	chr12:113354461-113354520	OAS1
A_24_P29733	6.095657	6.0909038	NM_012395	chr7:90839264-90839323	CDK14
A_23_P206733	5.000176	5.0805483	NM_001266	chr16:55846905-55846846	CES1
A_33_P3309034	7.7527757	7.7428713	NR_027269	chr10:38681035-38680976	SEPT7P9
A_24_P225468	11.347689	11.310212	NM_030920	chr1:150191136-150191077	ANP32E
A_23_P50241	5.888665	5.814802	NM_001294	chr19:45495582-45495641	CLPTM1

A_33_P3232011	2.3221061	2.3900566	NM_022449	chr2:238483032-238482973	RAB17
A_33_P3231407	4.669301	4.846374	XM_005244930	chr1:158909269-158909328	PYHIN1
A_33_P3229380	6.3144464	6.435328	NR_029401	chr1:243255878-243255819	LOC731275
A_23_P149529	2.3221061	2.3900566	NM_002353	chr1:59041468-59041409	TACSTD2
A_32_P85591	5.864142	5.879078		chrX:103231321-103231580	H2BFXP
A_23_P119196	9.193239	9.11738	NM_016270	chr19:16438246-16438305	KLF2
A_23_P167308	11.206932	11.213191	NM_002938	chr4:2517413-2517472	RNF4
A_33_P3854953	4.2625504	3.8313398		chr12:127354127-127354068	LOC440117
A_24_P295245	8.057941	8.007069	NM_032467	chr8:62596658-62596599	ASPH
A_23_P136909	8.480531	7.5526357	NM_030763	chrX:80370173-80370114	HMGN5
A_32_P108474	9.129967	8.974329	NM_002940	chr4:146048917-146048976	ABCE1
A_33_P3560878	3.7710056	3.749434		chr14:88553392-88553451	LOC283587
A_24_P118052	3.7209752	2.9895751	NM_199254	chr13:20012241-20010397	TPTE2
A_33_P3372526	9.703857	9.748213	NR_104261	chr2:207606083-207606024	MDH1B
A_33_P3236316	5.600644	5.8061104	NR_103825	chr4:119554066-119554125	LOC729218
A_33_P3398922	9.330259	9.496032	NM_001204142	chr18:47795483-47795424	MBD1
A_33_P3404749	4.192491	4.275831	NM_001277313	chr15:33066404-33066345	FMN1
A_33_P3258612	11.580002	11.508441	NM_002592	chr20:5098223-5098164	PCNA
A_23_P319719	4.9283047	4.757128	NM_138575	chr12:133294613-133294672	PGAM5
A_23_P115762	10.514431	10.582123	NM_007265	chr10:74894380-74894321	ECD
A_24_P9090	9.353835	9.046831	NM_031372	chr4:83344462-83344403	HNRNPDL
A_23_P339773	6.9860287	7.546673	NM_182752	chr1:3545945-3546004	TPRG1L
A_23_P39602	6.888089	7.0937514	NM_147233	chr2:24980905-24980964	NCOA1
A_23_P29555	12.091214	11.886883	NM_183352	chr3:10342717-10342658	SEC13
A_33_P3210561	8.033119	8.232511		chr2:026363183-026363124	
A_23_P254181	4.474042	4.61145	NR_034104	chr20:43285478-43285419	LOC79015
A_23_P325080	6.087327	6.0427823	NM_017432	chr19:50358243-50358302	PTOV1
A_32_P415151	4.0919447	4.0400977	NM_182552	chr6:170034569-170033151	WDR27
A_32_P64096	5.769771	5.2600203	NM_001012981	chr16:25247615-25247556	ZKSCAN2
A_33_P3409740	5.0127487	4.906801	XR_252379	chr8:11873031-11873090	LOC649294
A_32_P95729	9.82827	9.637137	NM_018193	chr15:89858528-89858587	FANCI
A_33_P3409625	11.795233	11.799826	NM_005775	chr8:22432948-22433007	SORBS3
A_33_P3317168	5.405978	5.9637947	NM_139053	chr1:110292762-110292703	EPS8L3
A_23_P134347	2.3221061	2.3900566	NM_019029	chr7:29105730-29105671	CPVL
A_32_P194779	6.8736377	7.1335535	NM_001099270	chr9:129647686-129647745	ZBTB34
A_23_P373079	6.8455834	6.6207337	NM_152716	chr11:59404705-59404646	PATL1
A_23_P368259	5.896036	5.857787	NM_152361	chr19:40022341-40022282	EID2B
A_23_P11507	9.202512	9.098446	NM_015534	chr1:78030621-78030562	ZZZ3
A_23_P65089	10.986963	10.766441	NM_016355	chr12:12982717-12982776	DDX47
A_32_P151800	10.189776	10.241552	NM_207418	chr1:143897200-143897141	FAM72D
A_24_P264664	9.108621	9.180775	NM_001271	chr15:93570825-93570884	CHD2
A_33_P3366039	10.823296	10.9396515	NM_000992	chr3:52029100-52028126	RPL29
A_23_P104151	6.8335705	7.432471	NM_016486	chr1:46159443-46159502	TMEM69
A_32_P61857	6.9660816	6.479394	NM_020854	chr18:59973874-59973933	KIAA1468
A_23_P99642	5.9658065	5.525871	NM_001126106	chr14:23242663-23242604	SLC7A7
A_23_P103837	8.569053	8.659764	NM_001127320	chr1:154234145-154235609	UBAP2L
A_33_P3410831	11.778658	11.723917		chrX:20005093-20005034	LOC729609
A_33_P3381127	8.8967285	9.434757	NM_000043	chr10:90774517-90774576	FAS
A_33_P3396239	11.557556	11.670421	NM_004175	chr22:24967914-24967973	SNRPD3
A_23_P416212	4.241172	4.4107084	NM_033194	chr17:40275148-40275207	HSPB9
A_33_P3395196	4.7762656	4.950611	DA380124	chr13:103929303-103929244	
A_24_P652700	6.297844	6.454549	NM_014985	chr15:49030758-49030699	CEP152
A_33_P3403459	7.1145096	7.4267755	NR_023921	chr14:24409944-24409885	DHRS4-AS1
A_23_P124108	11.11721	11.564716	NM_000632	chr16:31343816-31343875	ITGAM
A_33_P3380913	5.1929255	5.7123065		chr17:030761821-030761762	
A_33_P3317073	4.5479126	4.207639	NM_001244766	chr4:71847715-71847774	MOB1B
A_33_P3399318	10.147709	10.053511	NM_018841	chr1:68167216-68167157	GNG12
A_33_P3309799	6.485446	6.888318	NM_006778	chr6:30121832-30121773	TRIM10
A_33_P3421203	5.8067565	5.347445		chr8:8096389-8096448	FAM86B3P

A_23_P205336	8.050212	7.8302975	NM_016472	chr14:96853368-96853427	GSKIP
A_23_P37347	10.795741	10.851455	NM_012245	chr14:78184191-78184132	SNW1
A_33_P3414242	6.3004065	6.7532325	NM_001008229	chr6:29633982-29634041	MOG
A_24_P272917	4.8245153	5.027153	NM_001242326	chr4:9328261-9328320	USP17L25
A_23_P94819	5.9255543	6.061118	NM_006987	chr17:62615-62556	RPH3AL
A_32_P221452	5.861489	5.831026	NM_173825	chr3:120406018-120405959	RABL3
A_33_P3351474	7.4760213	7.882756	NM_001128429	chr4:95210929-95210988	SMARCAD1
A_33_P3317317	8.860194	9.183765	NM_001012709	chr11:1642696-1642637	KRTAP5-4
A_33_P3369311	9.680312	10.043764	NM_001142568	chr3:107524368-107524427	BBX
A_23_P344037	5.905467	5.9630685	NM_018223	chr12:133417771-133417712	CHFR
A_23_P29803	11.517313	11.503753	NM_006232	chr3:184086244-184086303	POLR2H
A_23_P309779	6.903425	6.5167065	NM_018177	chr4:40156199-40156258	N4BP2
A_23_P21990	4.227883	4.6234207	NM_152685	chr5:138707745-138707686	SLC23A1
A_33_P3283744	4.711354	4.4389954	XR_158980	chr2:133111716-133111775	FAM201B
A_33_P3325280	5.490257	5.300635		chr3:195712530-195712471	
A_33_P3234794	4.9826236	4.8737874		chr20:37272398-37272457	ARHGAP40
A_24_P225010	14.92621	14.875682	NM_001018	chr19:1440214-1440368	RPS15
A_33_P3223869	10.155862	10.267225		chr2:085329585-085329526	
A_32_P35800	7.7152395	7.767809	NM_015325	chr5:5489893-5489952	KIAA0947
A_24_P51061	8.177811	8.305476	NM_080927	chr3:98568429-98568370	DCBLD2
A_33_P3243332	2.8694663	4.2219644	NM_152432	chr11:100859541-100859600	ARHGAP42
A_24_P43588	8.687333	8.747749	NM_001003690	chr6:43608159-43608218	MAD2L1BP
A_23_P251945	9.572214	9.665884	NM_016221	chr5:150088924-150088865	DCTN4
A_24_P252043	5.3918705	6.1701875	NM_003463	chr6:64288845-64288904	PTP4A1
A_24_P190168	8.476421	8.605446	NM_014573	chr17:26654924-26654983	TMEM97
A_23_P46903	7.4295206	7.2184057	NM_172171	chr10:75576809-75574975	CAMK2G
A_33_P3361157	4.3945856	4.5650954		chr11:005922849-005922908	
A_33_P3238058	6.0482483	6.3142214	NR_027181	chr2:121223062-121223003	LINC01101
A_23_P22096	8.9924555	9.108971	NM_153831	chr8:141669169-141669110	PTK2
A_33_P3349693	4.129409	4.1898804	NM_182551	chr2:30790982-30791041	LCLAT1
A_32_P37592	5.0217576	3.9840906	NR_003003	chr18:47340748-47340807	SCARNA17
A_32_P234935	12.053645	11.932268	NM_007375	chr1:11084030-11084089	TARDBP
A_33_P3416513	4.4921365	4.5570655	NM_001040032	chr8:10558043-10558102	C8orf74
A_33_P3322769	3.8617039	4.3470526	NM_001005238	chr11:4936039-4935980	OR51G2
A_33_P3397073	4.79203	4.704943	NR_037169	chr6:32120829-32120770	LOC100507547
A_23_P333228	7.16798	6.056863	NM_020814	chr2:217122655-217122596	MARCH4
A_33_P3296991	6.6710477	6.9400377	NR_024413	chr3:187897865-187897924	FLJ42393
A_24_P182122	11.682007	11.716484	BC061915	chrM:3369-3428	ND1
A_23_P355311	4.8525186	5.2595897	NM_003738	chr1:45288983-45288924	PTCH2
A_33_P3229177	10.120717	10.122078	NM_019896	chr2:75186472-75186531	POLE4
A_33_P3216913	6.428755	6.7829804	NM_198147	chr17:27888454-27888395	ABHD15
A_23_P502312	10.166935	10.128957	NM_078481	chr19:14519267-14519326	CD97
A_24_P51588	8.337846	8.432004	NM_030937	chr1:1322722-1322663	CCNL2
A_33_P3350828	8.965092	8.970504	NM_021235	chr19:16472467-16472408	EPS15L1
A_33_P3265376	5.0401444	4.5219107	NM_001145724	chr19:19049274-19049215	HOMER3
A_24_P419300	8.563757	7.6813955	NR_024158	chr5:470888-470829	PP7080
A_33_P3666884	5.680666	6.0123596	NM_001098537	chr9:140354470-140354411	PNPLA7
A_23_P218817	6.462702	6.3492064	NM_152246	chr22:51008055-51007842	CPT1B
A_23_P161634	9.040716	8.947707	NM_025128	chr11:65633599-65633658	MUS81
A_33_P3211423	9.428935	9.00904	BX648891	chr5:167975662-167975603	PANK3
A_24_P287233	12.109067	12.058304	NM_001002252	chr12:123467393-123467452	ARL6IP4
A_23_P68031	3.5434268	4.3178806	NM_003151	chr2:191895762-191895703	STAT4
A_33_P3215999	3.824346	4.19468	NM_001145526	chr17:76203143-76203202	AFMID
A_33_P3339066	6.928837	6.832191	NM_017619	chr1:104068819-104068878	RNPC3
A_23_P141126	5.862241	5.858223	NM_000154	chr17:73754580-73754445	GALK1
A_33_P3222210	5.148025	4.521109	NM_001130111	chr19:1881265-1880087	ABHD17A
A_24_P131589	7.577094	7.681879	NM_006889	chr3:121839246-121839305	CD86
A_33_P3340869	4.4468956	3.9476178		chr19:35068247-35068188	SCGB1B2P
A_23_P111288	12.442451	12.35481	NM_016462	chr6:10731156-10731215	TMEM14C

A_23_P94365	6.805592	6.7696767	NM_174922	chr8:145618056-145618191	ADCK5
A_32_P80255	8.704763	9.020357	NM_001257191	chr11:118618713-118618654	DDX6
A_24_P314515	4.7426796	5.3395076	NR_024345	chr12:121408137-121408078	HNF1A-AS1
A_23_P137361	7.3168006	7.3200006	NM_017818	chr1:3548874-3548815	WRAP73
A_24_P184799	3.318596	3.0077906	NM_004086	chr14:31358977-31359036	COCH
A_24_P56270	6.5792546	6.398038	NM_006482	chr12:68056135-68056194	DYRK2
A_24_P106794	7.2963967	7.4912276	NM_032869	chr8:110253441-110253382	NUDCD1
A_33_P3304247	3.850337	3.829535	AK130267	chr1:19679296-19679237	
A_23_P61426	8.11023	8.1922865	NM_012331	chr8:10286295-10286354	MSRA
A_23_P142590	7.7672606	7.9560714	NM_030768	chr2:239092737-239092678	ILKAP
A_23_P4353	9.993337	10.563667	NM_015626	chr17:25639668-25639727	WSB1
A_33_P3231086	4.5638022	5.1755705		chr20:062689140-062689199	
A_33_P3253214	7.957975	8.58634	L19779	chr1:149823132-149823191	
A_33_P3314471	3.7832932	3.190127		chr2:86385558-86385499	IMMT
A_33_P3294608	11.833224	12.064273	NM_017458	chr16:29859281-29859340	MVP
A_23_P303181	10.288681	10.438438	NM_020126	chr19:49133578-49133637	SPHK2
A_33_P3399101	9.738386	9.813002		chr9:90343780-90343839	CTSL
A_23_P421221	7.838186	7.665114	NM_001136108	chr8:23152365-23153526	R3HCC1
A_23_P132159	5.1650925	5.875481	NM_017414	chr22:18659618-18659677	USP18
A_23_P326204	4.537943	4.595602	NM_152621	chr4:108831701-108831760	SGMS2
A_23_P65618	3.7018118	3.5252228	NM_000359	chr14:24718510-24718451	TGM1
A_24_P4877	7.8777714	7.663861	NM_033114	chr12:42706792-42706733	ZCRB1
A_23_P11005	8.049463	8.218655	NM_014272	chr15:79051849-79051790	ADAMTS7
A_23_P350005	3.1837196	3.120202	NM_173553	chr4:189012558-189012499	TRIML2
A_23_P41075	7.7444544	7.587784	NM_032355	chr3:49946475-49946416	MON1A
A_33_P3240392	8.981721	9.155949	NM_001455	chr6:109005852-109005911	FOXO3
A_23_P218579	5.7076764	5.882281	NM_024506	chr2:220101864-220101805	GLB1L
A_33_P3372667	5.289629	5.1858664		chr6:153310292-153310351	
A_23_P67424	5.0946903	4.9786463	NM_153257	chr19:37129709-37129650	ZNF461
A_23_P56894	5.6766424	6.1102905	NM_177538	chr2:204150409-204154500	CYP20A1
A_23_P134650	8.298243	8.265329	NM_015545	chr7:99016854-99016795	PTCD1
A_24_P145911	9.805195	9.651554	XM_005247704	chr3:185649620-185649561	TRA2B
A_23_P251505	9.122016	8.80377	NM_148956	chr7:72717662-72717603	NSUN5
A_23_P140698	7.3222127	7.188125	NM_018163	chr15:41067300-41067241	DNAJC17
A_23_P155257	10.133879	10.125859	NM_032682	chr3:71008467-71008408	FOXP1
A_24_P296457	5.5468464	5.5074406	NM_000294	chr16:30764789-30764848	PHKG2
A_23_P50052	7.6517835	7.9743814	XR_109479	chr18:45778947-45779006	
A_24_P228228	4.939168	2.3900566	NM_004775	chr18:29203275-29203216	B4GALT6
A_23_P168993	4.640927	4.4290605	NM_000025	chr8:37821117-37821058	ADRB3
A_33_P3316587	7.153358	6.1171474	NM_007105	chr11:2909425-2909366	SLC22A18AS
A_24_P319942	6.633477	6.4513083	NM_007107	chr3:156259127-156259068	SSR3
A_23_P361085	13.189826	12.945196	NR_003038	chr6:86387159-86386868	SNHG5
A_24_P12281	5.658038	5.514187	NM_007077	chr14:31535524-31539091	AP4S1
A_33_P3287477	3.5943034	3.608598	NM_001010912	chr10:124457839-124457780	C10orf120
A_33_P3359683	4.295247	4.593394	NM_001172128	chr15:81578106-81578165	IL16
A_23_P1361	9.759965	9.855939	NM_002860	chr10:97366196-97366137	ALDH18A1
A_33_P3391865	6.217305	6.0041647	NM_001031714	chr14:105181133-105181192	INF2
A_23_P145408	9.269537	9.077146	NM_032020	chr6:143816971-143816912	FUCA2
A_33_P3251547	3.9143891	4.4599295	XM_005249927	chr7:39987179-39987121	
A_23_P360769	7.894802	8.408651	NM_002372	chr5:109200837-109202595	MAN2A1
A_23_P147805	9.548355	9.456008	XM_005249841	chr7:48134390-48146494	UPP1
A_33_P3380837	13.77849	13.887369	NM_133463	chr7:2752675-2752734	AMZ1
A_23_P258071	9.081067	8.974962	NM_006978	chrX:119004579-119004520	RNF113A
A_23_P156408	6.1994843	6.51905		chr6:72124457-72124398	LINC00472
A_33_P3376828	4.6081553	4.456169	NM_138410	chr3:32490977-32491036	CMTM7
A_23_P146512	7.9335666	8.916543	NM_016548	chr9:88641621-88641562	GOLM1
A_33_P3249072	6.84852	7.4312563	XM_005269472	chr10:99361936-99361995	HOGA1
A_33_P3403107	6.8333063	6.5619364	NM_199285	chr19:42814913-42814972	PRR19
A_33_P3324786	4.0802355	4.381217	XM_005260184	chr20:61477326-61477267	

A_32_P148710	10.2312975	10.168236	NM_005507	chr11:65623161-65622908	CFL1
A_23_P203737	8.957512	9.020862	NM_033547	chr11:77590054-77589995	INTS4
A_33_P3208995	4.261179	4.626846	NR_045983	chr20:45085434-45085375	ZNF663P
A_32_P22501	5.408266	5.346734	NR_034170	chr15:44828355-44828296	EIF3J-AS1
A_32_P86763	7.3475184	7.4078307	NM_004613	chr20:36757912-36757853	TGM2
A_23_P15516	9.315982	9.490384	NM_032376	chr17:42088952-42088893	TMEM101
A_33_P3258324	6.578757	6.730048		chr19:15962862-15962803	
A_23_P200325	8.270436	8.363361	NM_014857	chr1:174927036-174927095	RABGAP1L
A_33_P3386970	5.5547895	5.30378	BC039426	chr1:159994900-159994841	
A_23_P3038	4.564444	4.55682	NM_002083	chr14:65406455-65406396	GPX2
A_23_P150394	4.7093124	4.727216	NM_022003	chr11:117708225-117708166	FXYD6
A_24_P655268	8.396639	8.448746		chr15:41591663-41591722	OIP5-AS1
A_33_P3364211	4.5039487	4.258608	AK126423	chr3:73674377-73674436	
A_23_P201342	11.991159	11.561938	NM_004421	chr1:1270778-1270719	DVL1
A_23_P128372	9.374195	9.209742	NM_002014	chr12:2912469-2912528	FKBP4
A_33_P3270509	6.8848925	7.5883737	NM_003031	chr16:48395444-48395385	SIAH1
A_23_P395595	9.771654	9.82827	NM_015308	chr11:47738209-47738150	FNBP4
A_33_P3347108	8.470701	8.177483	NM_138376	chr14:20757363-20757304	TTC5
A_23_P37569	5.8281417	5.5978923	NM_006122	chr15:91465544-91465603	MAN2A2
A_24_P18802	9.238232	9.306962	NM_020857	chr15:41195886-41195945	VPS18
A_23_P104583	4.8104353	4.4886646	NM_020441	chr11:67207834-67207704	CORO1B
A_33_P3377994	6.742379	6.923934	NM_032387	chr17:40946814-40946873	WNK4
A_23_P310582	3.159115	3.35	NM_138729	chr1:113084697-113084638	ST7L
A_33_P3331346	9.999754	9.804845	NM_173547	chr17:73885133-73885074	TRIM65
A_23_P416191	4.386862	4.5732055	NM_176885	chr12:11183401-11183342	TAS2R31
A_23_P141779	6.1022253	6.1006308	NM_014593	chr18:47810087-47809888	CXXC1
A_24_P220771	5.1453376	5.152401	NM_032217	chr4:74005746-74005687	ANKRD17
A_23_P166	6.813891	6.67606	NM_145279	chr1:47073825-47073766	MOB3C
A_33_P3217153	7.0862703	7.063233	NM_001077243	chr11:105852760-105852819	GRIA4
A_33_P3418209	3.6888678	3.6588063	NM_004791	chr13:102367945-102368004	ITGBL1
A_23_P134684	9.212635	9.218625	NM_024567	chr8:28909311-28909370	HMBOX1
A_23_P250813	7.09562	7.009017	NM_000553	chr8:31030776-31030835	WRN
A_23_P144054	7.649498	7.5140605	NM_006254	chr3:53226222-53226281	PRKCD
A_23_P145218	5.3965774	5.8096724	BC166633	chr6:26431721-26431780	BTN2A3P
A_24_P399622	6.3389993	5.995548	NM_199444	chr19:19030033-19023798	COPE
A_33_P3238866	8.081989	7.9934306	NM_016038	chr7:66459320-66459261	SBDS
A_33_P3387856	5.794906	5.6361036	NM_001100625	chr16:81066653-81066712	CENPN
A_24_P392925	4.435634	4.2175126	NM_001014985	chr17:4693360-4693419	GLTPD2
A_33_P3610123	9.120174	8.821939	NM_015425	chr2:86253606-86253547	POLR1A
A_33_P3261084	6.502059	6.571455	NM_174855	chr20:2644152-2644093	IDH3B
A_23_P12128	5.382273	5.6070976	NM_000549	chr1:115576682-115576741	TSHB
A_32_P360193	3.9686298	3.9611514	NM_144666	chr11:6569440-6569499	DNHD1
A_23_P133470	7.930476	8.282475	NM_014819	chr5:108671278-108671219	PJA2
A_32_P7823	8.59982	8.678358	NM_144578	chr14:55531342-55531401	MAPK1IP1L
A_23_P78608	4.770191	4.869569	NM_024898	chr19:6467293-6467234	DENND1C
A_24_P818010	7.5058002	7.527775	NR_039983	chr1:134920-134861	LOC729737
A_23_P70746	5.0332203	5.4761496	NM_017651	chr6:135749806-135748426	AHI1
A_23_P11843	6.662739	6.986639	NM_201630	chr1:204586412-204586353	LRRN2
A_23_P153251	7.1235614	7.404814	NM_133502	chr19:58724097-58724156	ZNF274
A_23_P251342	7.203844	7.4128137	NM_002093	chr3:119582401-119582342	GSK3B
A_24_P360763	7.376501	7.573101	NM_001881	chr10:35467876-35468082	CREM
A_23_P200560	12.177249	12.153799	NM_001039802	chr1:22419269-22419328	CDC42
A_23_P133974	10.103994	10.333071	NM_024294	chr6:34574417-34574358	C6orf106
A_23_P122216	4.0874853	4.5832376	NM_002317	chr5:121405845-121405786	LOX
A_23_P117928	9.387709	9.572214	NM_014952	chr15:40760131-40760190	BAHD1
A_33_P3215059	6.917272	7.122594	NM_145080	chr16:27244420-27244361	NSMCE1
A_33_P3224307	6.9311476	6.5294867	NR_003010	chr12:7076683-7076624	SCARNA12
A_32_P96036	8.375235	7.724992	NM_001093725	chr1:156042043-156041984	MEX3A
A_33_P3410935	12.025831	11.780402	NM_001086521	chr17:79215007-79215067	C17orf89

A_33_P3281532	4.350077	4.6299295	NM_022780	chr2:86968111-86968170	RMND5A
A_23_P38328	5.791787	5.8269725	AK023149	chr17:42809611-42809670	DBF4B
A_23_P89824	5.4567165	4.945758	NM_032933	chr18:20876971-20876912	TMEM241
A_33_P3259148	8.105752	8.045856	NM_001145045	chr17:18561832-18561773	ZNF286B
A_23_P6762	9.853773	10.006038	NM_032492	chr3:9935405-9935464	JAGN1
A_33_P3276505	6.104472	5.8479204	NM_001010986	chrX:138820135-138820076	ATP11C
A_23_P211212	11.55192	11.205332	NM_030582	chr21:46933558-46933617	COL18A1
A_23_P407684	6.767451	6.203182	NM_178167	chr16:2048465-2048406	ZNF598
A_33_P3321602	5.277042	5.5487933	BC068508	chr1:28816307-28816366	PHACTR4
A_33_P3382399	5.0275593	5.3818765	NM_031958	chr17:39165089-39165030	KRTAP3-1
A_23_P111228	6.9653244	6.1581416	NM_017421	chr6:99823868-99819457	COQ3
A_33_P3297030	7.445252	7.838186	AK128440	chr17:441183-441124	VPS53
A_33_P3312182	3.8666034	3.4116647	NM_153256	chr10:11912346-11912405	PROSER2
A_24_P125690	10.347855	10.310577	NM_023937	chr19:17417290-17417349	MRPL34
A_33_P3392677	4.4895163	3.9267602		chr7:066313630-066313689	
A_33_P3352843	5.606936	5.726768		chr4:9925687-9925746	
A_32_P481377	4.7267547	5.333091	NM_175858	chr21:32253283-32253224	KRTAP11-1
A_23_P502224	12.899967	13.101461	NM_007326	chr22:43014929-43014870	CYB5R3
A_24_P183864	9.443718	9.373334	NM_005536	chr8:82570477-82570418	IMPA1
A_23_P380614	7.9934306	7.799457	NM_006045	chr20:50213418-50213359	ATP9A
A_24_P151727	11.80975	11.785743	NM_007363	chrX:70520608-70520667	NONO
A_33_P3268900	13.153513	13.213173	AF370457	chr8:26254988-26255047	BNIP3L
A_23_P300076	5.1103573	5.069311	NM_032263	chr3:197640793-197639603	IQCG
A_23_P106737	8.469263	8.353823	NM_201412	chr16:256079-256020	LUC7L
A_23_P368225	7.063613	6.856087	NM_152463	chr17:48458627-48458686	EME1
A_23_P1505	6.369954	6.2996783	NM_002335	chr11:68216359-68216418	LRP5
A_23_P62351	8.765022	8.912523	NM_019007	chrX:100870531-100870472	ARMCX6
A_23_P8763	10.753524	10.744663	NM_002835	chr7:77269186-77269245	PTPN12
A_23_P143906	9.02583	9.344716	NM_022443	chr3:158322916-158322975	MLF1
A_33_P3264746	8.573952	8.79845		chr22:017011355-017011414	
A_33_P3353622	4.727577	4.9177094	NM_021163	chr7:5104801-5104860	RBAK
A_23_P66421	8.380993	8.068399	NM_015654	chr17:72766942-72766883	NAT9
A_32_P23010	8.792773	8.525387	NM_001042631	chr19:36487032-36487091	SDHAF1
A_33_P3398143	4.2324862	4.8020024		chr21:046327961-046327902	
A_23_P144622	6.094391	6.586898	NM_005471	chr5:141381281-141381222	GNPDA1
A_24_P576174	8.212871	7.9189568	NM_018403	chr3:53317681-53317622	DCP1A
A_33_P3422289	5.9450016	5.6945734	XR_248830	chr1:93730287-93730228	LOC100131564
A_23_P133629	8.473993	8.774906	NM_004661	chr5:137523767-137523708	CDC23
A_23_P118174	8.077831	8.033253	NM_005030	chr16:23700970-23701212	PLK1
A_33_P3290149	3.8248277	3.9689412		chr19:054945882-054945823	
A_32_P4228	7.1032615	7.276844	XR_109647	chr20:11791036-11790977	LINC00687
A_24_P366526	11.269348	11.354872	BC105992	chr15:030338228-030338287	SYNGR2
A_33_P3555009	10.316018	10.284694	BX537909	chr19:52892163-52892104	
A_33_P3210218	7.1392946	7.27096	NM_020474	chr18:33289953-33290012	GALNT1
A_32_P34387	5.3939238	5.489819	NM_018418	chr14:88904417-88904476	SPATA7
A_23_P255805	8.705313	8.886627	NM_003416	chr8:146068266-146068325	ZNF7
A_24_P935782	5.472048	5.2580423	NM_001008727	chr19:9677344-9677285	ZNF121
A_24_P295806	6.2102346	6.392474	NM_023947	chr11:900087-900028	CHID1
A_33_P3417990	7.2714305	7.0320816	NM_006296	chr2:58373548-58373607	VRK2
A_23_P96777	8.703916	8.765022	NM_020710	chr1:3697186-3697127	LRRC47
A_33_P3280421	5.2661715	5.1287575	NR_003595	chr7:48886065-48886006	CDC14C
A_23_P254472	9.433584	9.487399	NM_024573	chr6:151790809-151790868	C6orf211
A_32_P44775	6.8465133	6.6622534	NM_182505	chr9:74586446-74586505	C9orf85
A_32_P18824	9.917894	9.971139	NM_013263	chr16:50353177-50353118	BRD7
A_23_P161644	3.8774645	3.4170022	NM_006328	chr11:66396612-66396671	RBM14
A_33_P3263061	8.673521	8.944113	NM_016282	chr9:4711217-4711158	AK3
A_24_P129341	6.7160845	5.714733	NM_020299	chr7:134225799-134225858	AKR1B10
A_23_P58529	10.160134	9.960455	NM_014473	chr5:61684607-61684548	DIMT1
A_23_P27404	3.973278	3.8246334	NM_001136499	chr19:52567757-52567719	ZNF841

A_33_P3337733	3.8591135	4.2178783	AK127731	chr17:14671053-14670994	FLJ45831
A_23_P79962	7.5563526	7.0488687	NM_170784	chr20:10386142-10386083	MKKS
A_33_P3255131	5.673872	5.8955994	NM_001100915	chr16:67323452-67323393	KCTD19
A_23_P75255	10.1778555	10.217921	NM_004896	chr10:70931959-70932018	VPS26A
A_23_P353005	5.111308	5.5646954	NM_152553	chr6:125404466-125404525	RNF217
A_33_P3315801	4.3207	3.7467809	NM_016507	chr17:37687510-37687569	CDK12
A_23_P217384	8.541131	8.814966	NM_003916	chrX:15844638-15844579	AP1S2
A_33_P3292387	5.4213066	5.1948266	NM_001105659	chr1:74648282-74648223	LRRIQ3
A_23_P24485	4.5153236	4.8220983	NM_006165	chr11:129734922-129734863	NFRKB
A_23_P379649	2.3221061	2.3900566	NM_001003940	chr15:40380509-40380450	BMF
A_33_P3333752	4.3186884	4.390802	XM_005270875	chr1:43618590-43618649	FAM183A
A_24_P117942	7.204693	7.063613	NM_207377	chr14:58874116-58874175	TOMM20L
A_23_P349928	13.517996	13.506524	NM_003113	chr2:231380490-231380696	SP100
A_23_P4489	3.413969	2.3900566	NM_138966	chr18:70417312-70416280	NETO1
A_33_P3409392	10.760913	10.860082	NM_003506	chr8:104344731-104344789	FZD6
A_33_P3324086	10.526705	10.3044815	NM_001031713	chr6:13791080-13791021	MCUR1
A_33_P3327108	7.8842278	7.4308624	NM_016231	chr17:26523332-26523391	NLK
A_24_P362646	9.540537	9.541327	NM_005783	chr2:99944009-99938625	TXNDC9
A_23_P211417	3.9255233	3.5956872	NM_021026	chr22:29838127-29838186	RFPL1
A_23_P404494	2.3221061	2.3900566	NM_002185	chr5:35876781-35876840	IL7R
A_23_P64121	5.491689	4.9190016	NM_012194	chr11:33695366-33695425	KIAA1549L
A_23_P217098	7.741705	7.8871584	NM_033305	chr9:80032127-80032186	VPS13A
A_23_P208288	6.3714175	6.900463	NM_020657	chr19:57870937-57870996	ZNF304
A_33_P3416366	3.8149638	3.7495089	NM_005483	chr19:4431965-4432024	CHAF1A
A_33_P3297562	8.373526	8.259859	NM_033267	chr5:2746939-2746880	IRX2
A_23_P398275	8.121316	8.177182	NR_027001	chr15:84867734-84867675	LOC388152
A_24_P933418	7.7579827	7.7909927	NM_005759	chr2:204295853-204295912	ABI2
A_33_P3288159	10.751483	10.864839	NM_018136	chr1:197057458-197057399	ASPM
A_23_P94552	11.50742	11.840261	NM_013390	chr9:74298770-74298711	TMEM2
A_33_P3227320	8.993664	8.99647	NM_152622	chr5:56215591-56215532	MIER3
A_33_P3651994	8.35991	8.120427	AK022657	chr5:60187783-60187724	ERCC8
A_33_P3394213	3.3602316	3.5360801	NM_138690	chr19:1009648-1009707	GRIN3B
A_23_P121614	3.730667	3.1153684	NM_152291	chr4:71347754-71347813	MUC7
A_23_P255317	8.149119	7.4239516	NM_004290	chr5:141364394-141364453	RNF14
A_33_P3215913	4.724004	4.7887297		chr22:038642708-038642649	
A_32_P96692	7.732791	7.58062	NM_006502	chr6:43588198-43588257	POLH
A_32_P1445	7.699514	7.693483	NM_080423	chr18:12785507-12785459	PTPN2
A_33_P3336552	3.3673034	2.3900566	HY006159	chr3:120086872-120086931	
A_23_P1594	11.249504	11.5070505	NM_003377	chr11:64006111-64006170	VEGFB
A_33_P3691758	4.2776318	3.8248277	X64985	chr11:55277854-55277913	OR4C1P
A_32_P3998	6.761148	6.893416	NM_001004301	chr19:53996095-53996154	ZNF813
A_23_P154708	5.092003	4.934907	NM_017545	chr20:7863975-7863916	HAO1
A_23_P133807	5.174217	4.9476676	NM_138572	chr6:42044918-42044977	TAF8
A_33_P3294031	9.5167	9.586984		chr11:2661862-2661803	KCNQ1OT1
A_23_P360179	7.3391767	7.4718556	NM_032442	chr17:7219137-7219078	NEURL4
A_32_P137966	4.539452	4.258389	NM_152317	chr12:100649936-100649877	DEPDC4
A_23_P116091	7.831416	7.453476	NM_130443	chr11:66272183-66272242	DPP3
A_23_P71864	5.3385973	5.4529543	NM_032809	chr9:131833547-131833606	FAM73B
A_33_P3385662	4.045641	4.1303205		chr15:100332814-100332873	
A_24_P910733	9.753205	9.874785	NM_178335	chr3:191100585-191107314	CCDC50
A_23_P151459	9.063983	8.9641	NM_015057	chr13:77619287-77619228	MYCBP2
A_32_P103633	8.7391205	8.844009	NM_004526	chr3:127340805-127340864	MCM2
A_33_P3308219	4.228411	4.0493164		chr8:007085546-007085487	
A_23_P6914	9.046831	8.1279125	NM_022776	chr3:125248023-125247964	OSBPL11
A_33_P3247753	6.4513083	6.2171454		chrX:73027005-73027064	TSIX
A_33_P3335137	4.470966	4.5358963	NM_013325	chr2:242608040-242608099	ATG4B
A_33_P3303577	7.765369	7.7848506	NM_198563	chr3:52873986-52873927	TMEM110
A_33_P3387365	3.629222	4.3677444	NM_007238	chr20:32290650-32290591	PXMP4
A_23_P202594	9.508649	9.481033	NM_024834	chr10:121589539-121589480	MCMBP

A_33_P3318960	9.57275	9.335954	NM_021974	chr22:38352789-38352848	POLR2F
A_23_P69249	11.367223	11.24106	NM_178042	chr3:179305796-179305855	ACTL6A
A_33_P3409210	3.8830042	4.0240335	XR_110076	chr5:177378897-177378956	
A_23_P317691	9.409898	9.47298	NM_016290	chr5:176332000-176331981	UIMC1
A_24_P48177	9.849198	9.94519	NM_006927	chr16:70413942-70413883	ST3GAL2
A_23_P47839	6.409606	6.219015	NM_020936	chr12:124104623-124104682	DDX55
A_23_P136172	7.375324	7.786662	NM_152417	chr8:56652369-56652310	TMEM68
A_23_P89621	5.461274	5.2120976	NM_003655	chr17:77807836-77807777	CBX4
A_23_P201628	12.394901	12.663231	NM_002293	chr1:183114406-183114465	LAMC1
A_32_P231086	8.462111	8.495375	NM_198181	chr15:82730411-82730470	GOLGA6L9
A_23_P110957	9.692306	9.628123	NM_001452	chr6:1395386-1395445	FOXF2
A_33_P3290707	5.273427	5.54274	NM_007289	chr3:154802056-154802115	MME
A_23_P65041	5.9384727	6.1769648	NR_026583	chr12:45456428-45456401	RACGAP1P
A_23_P115124	6.749103	6.329611	NM_145214	chr1:228581716-228581657	TRIM11
A_23_P25293	10.95436	10.987789	NM_014865	chr12:6640598-6640657	NCAPD2
A_33_P3287562	7.191671	6.4290123	NM_018844	chr7:107260832-107260891	BCAP29
A_23_P427217	7.502629	7.6811924	NM_032776	chr10:64928279-64928220	JMJD1C
A_23_P125837	6.9736342	6.809534	NM_016500	chrX:75395344-75395403	PBDC1
A_23_P215549	3.2771387	3.7316346	NM_000940	chr7:94989364-94989305	PON3
A_23_P379026	8.594799	8.800651	NM_019096	chr6:43588488-43588429	GTPBP2
A_33_P3320077	4.4270096	4.411089	NM_001282787	chr9:14180718-14180659	NFIB
A_23_P31489	6.3859663	6.4309087	NM_001077664	chr7:43916099-43916040	URGCP
A_24_P196384	5.3137693	4.932816	NM_001282380	chr15:90440348-90440289	C15orf38
A_23_P110802	8.514037	8.268076	NM_022909	chr5:68505649-68505708	CENPH
A_33_P3417150	4.622403	4.3625903	NM_002563	chr3:152555149-152555208	P2RY1
A_23_P420256	5.1974297	5.3210583	NM_021925	chr2:20885301-20885242	C2orf43
A_33_P3232038	6.8430905	6.9717846	NM_001204513	chr7:5112794-5112853	RBAK-RBAKDN
A_23_P112004	6.478795	6.691071	NM_012472	chr8:133584643-133584584	LRRC6
A_24_P323997	4.242616	4.0128975	NM_198479	chr19:48305115-48305056	TPRX1
A_23_P3355	9.599095	9.768507	NM_002693	chr15:89859772-89859713	POLG
A_23_P391228	9.12239	8.992191	NM_001031740	chr1:38266643-38266702	MANEAL
A_23_P101829	3.9724252	3.6036024	NM_004720	chr19:19735330-19735271	LPAR2
A_23_P17844	4.3580875	3.9604492	NM_002854	chr22:37209739-37196953	PVALB
A_23_P380766	9.625977	9.679792	NM_006035	chr14:103398858-103398799	CDC42BPB
A_23_P133279	6.9864464	6.671459	NM_199133	chr5:10226717-10226658	FAM173B
A_23_P259580	5.771495	5.983322	NM_172208	chr6:33281029-33273141	TAPBP
A_23_P141479	8.405464	8.466138	NM_003170	chr17:27028961-27029020	SUPT6H
A_23_P50081	8.147058	8.933968	NM_014214	chr18:12030648-12030707	IMPA2
A_23_P145357	8.419093	8.010693	NM_001188	chr6:33540644-33540585	BAK1
A_33_P3272330	9.413418	9.505235	NM_175629	chr2:25457154-25457095	DNMT3A
A_23_P16242	3.0907245	3.5053272	NM_021143	chr19:12242156-12242149	ZNF20
A_24_P323967	6.289069	6.594199	NM_001145434	chr19:52888043-52888102	ZNF880
A_32_P231446	9.674395	9.55136	NM_198268	chr1:114520268-114520327	HIPK1
A_23_P29365	6.5001802	6.812612	NM_012234	chr3:72424889-72424830	RYBP
A_24_P282751	4.4245515	4.55637	NM_014860	chr2:27874473-27874414	SUPT7L
A_33_P3387561	7.821193	8.194221	NM_206818	chr19:54599098-54599039	OSCAR
A_23_P154306	8.72789	8.753195	NM_004180	chr2:162091954-162092013	TANK
A_24_P169896	4.5863733	4.0395274	NM_001243541	chr17:76887029-76886970	LOC100653515
A_23_P54891	7.5210104	7.614069	NM_022744	chr16:31500989-31500930	C16orf58
A_33_P3437907	3.9897435	4.405595	NM_198475	chr17:42432015-42431956	FAM171A2
A_23_P149042	6.815504	6.91243	NM_013328	chr1:226108312-226108253	PYCR2
A_23_P390097	4.7650323	4.8853335	NM_152574	chr9:15172034-15171975	TTC39B
A_33_P3319406	3.6129677	4.838311	BX537570	chr10:24885677-24885618	ARHGAP21
A_33_P3399268	5.3075223	5.2398076		chr10:5995250-5995191	IL15RA
A_33_P3389540	14.322374	14.369245		chr9:034224352-034224293	
A_33_P3828101	6.0466585	5.3256803	NM_152549	chr5:114603559-114603500	CCDC112
A_23_P320530	4.6485853	4.3914757	NM_001010880	chr19:40579524-40579465	ZNF780A
A_23_P255672	3.7009113	3.629222	NM_032432	chr4:7967135-7967076	ABLIM2
A_24_P185158	4.5915146	4.846635	NM_178126	chr17:40732173-40732114	FAM134C

A_33_P3420167	11.670421	11.82115		chr7:071407341-071407400	
A_33_P3318534	11.464255	12.273699	NM_152282	chr3:141011914-141011973	ACPL2
A_33_P3818787	6.0897393	5.9347463	NR_027277	chr4:40045054-40044995	LOC344967
A_33_P3399373	10.398786	10.4621525	NM_001136053	chr3:127291992-127291933	TPRA1
A_23_P146922	4.1752224	3.3335478	NM_000820	chr13:114531609-114531550	GAS6
A_33_P3379763	6.5985756	6.451538		chr18:012102230-012102171	
A_23_P150238	7.0702577	6.6716356	NM_031450	chr11:65684564-65684505	C11orf68
A_23_P141802	2.839057	2.3900566	NM_001040147	chr18:61471776-61471835	SERPINB7
A_33_P3312544	4.777589	5.1880226	AK296023	chr4:47034971-47035030	GABRB1
A_33_P3609431	13.535304	13.568987	AK127846	chr19:53516237-53516293	
A_33_P3272563	6.386614	6.0825458	NM_004808	chr10:15147956-15147897	NMT2
A_32_P28223	5.480764	5.6067386	XR_247266	chr11:76493751-76493692	
A_33_P3401093	3.9301133	4.026166	NM_174899	chr2:230876799-230876858	FBXO36
A_24_P357709	6.273546	6.4339104	NM_001009921	chr3:184717629-184742162	VPS8
A_33_P3275651	4.375395	4.067924		chr11:055052971-055052912	
A_23_P84565	6.4022636	6.1747637	NM_001722	chr8:22108244-22108303	POLR3D
A_23_P201636	10.032239	9.498183	NM_005562	chr1:183213610-183213669	LAMC2
A_33_P3259022	5.0526075	5.1331124	NM_001172504	chr16:55734158-55734217	SLC6A2
A_24_P339664	10.838594	10.536037	NM_020170	chr19:3209364-3209423	NCLN
A_23_P429689	7.204541	7.462387	NM_148894	chr4:13570795-13570736	BOD1L1
A_24_P338648	9.693044	9.68684	NM_001177	chr12:101787185-101787126	ARL1
A_23_P135257	4.795188	4.636106	NM_002771	chr9:33797923-33797982	PRSS3
A_23_P62133	4.684317	3.8330789	NM_000252	chrX:149840555-149840614	MTM1
A_24_P935345	5.550372	5.4651427	AY358191	chr19:40780795-40780736	LOC100507646
A_32_P62571	10.153238	10.075157	NM_005105	chr1:145511112-145511171	RBM8A
A_33_P3249936	3.8554707	3.446045	NM_198463	chr3:58727842-58727783	C3orf67
A_23_P137143	10.842691	10.629435	NM_001363	chrX:154005587-154005646	DKC1
A_33_P3407636	4.278188	3.9064698	AK296555	chr17:1302259-1302200	YWHAE
A_33_P3343957	5.8052697	6.2462683	NM_015252	chr2:63272576-63272635	EHBP1
A_23_P128166	9.230438	9.154627	NM_014999	chr12:72180219-72180278	RAB21
A_33_P3230594	5.3826523	5.2452483	NR_037169	chr6:32120745-32120686	LOC100507547
A_23_P304287	9.967065	10.0259695	NM_002803	chr7:103007997-103008056	PSMC2
A_33_P3404418	4.2268157	4.3526073	NM_004672	chr1:27684040-27683981	MAP3K6
A_33_P3258478	4.6404276	4.676511	XM_003403416	chr19:55301610-55301669	1060P11.3
A_23_P75402	10.61262	10.150496	NM_004551	chr11:47603979-47605883	NDUFS3
A_33_P3271810	3.7443976	3.4579675	NM_015020	chr16:71686757-71686698	PHLPP2
A_24_P606663	13.758507	13.7511425		chr7:054724304-054724363	
A_33_P3362526	4.417255	4.322843	NM_016060	chr17:6546705-6546646	MED31
A_32_P41375	8.5568285	8.274496		chr7:128295430-128295489	LINC01000
A_33_P3378702	5.4632163	5.673872	AK127786	chr3:9862279-9862220	
A_33_P3352407	15.980293	16.062252		chr2:181737784-181737725	
A_23_P314835	8.350611	8.360432	NM_178456	chr20:56736045-56736104	C20orf85
A_33_P3324495	7.4478745	7.204693	NM_173832	chr8:144344816-144344875	ZFP41
A_23_P115838	4.269282	4.605746	NM_015652	chr10:98745056-98745115	C10orf12
A_32_P87013	6.6395173	4.0281677	NM_000584	chr4:74609265-74609324	IL8
A_33_P3337034	7.2785225	7.3964815	NM_003913	chr6:4056609-4056668	PRPF4B
A_23_P301247	8.213596	8.816771	NM_003517	chr1:149858863-149858922	HIST2H2AC
A_33_P3327158	6.0759387	6.3901005	AK127761	chr11:45073130-45073071	LOC100131432
A_23_P17593	8.562775	8.222954	NM_001794	chr20:60511966-60512025	CDH4
A_33_P3235990	7.1479993	6.7094836	NM_007039	chr14:88932219-88932160	PTPN21
A_33_P3312246	9.232864	9.434869	NM_017934	chr6:79650912-79650853	PHIP
A_23_P388670	10.309349	10.489464	NM_000895	chr12:96394826-96394767	LTA4H
A_33_P3423631	5.9586964	6.208288	NM_007331	chr4:1944069-1944128	WHSC1
A_33_P3378126	8.782967	8.940729	NM_058229	chr8:124515447-124515388	FBXO32
A_33_P3331726	6.9088264	6.9660816	NR_027451	chr20:34636801-34636742	LINC00657
A_24_P74571	6.621154	6.9135523	NM_001002880	chr22:39067174-39069203	CBY1
A_24_P383478	5.6886067	5.679678	NM_000125	chr6:152129048-152129107	ESR1
A_33_P3219434	4.3208127	4.4480877	NM_017898	chr1:220936332-220936391	MARC2
A_33_P3349501	6.397167	6.6686845		chr2:069159486-069159545	

A_23_P72025	7.4937696	7.5361857	NM_000387	chr3:48895022-48894963	SLC25A20
A_23_P128215	11.815793	11.946984	NM_003877	chr12:93969799-93969858	SOCS2
A_33_P3339860	10.068425	9.796847	NM_021623	chr8:38831317-38831376	PLEKHA2
A_23_P38732	7.166653	7.2946033	NM_001792	chr18:25532140-25532081	CDH2
A_33_P3218955	5.0457115	4.8104353	NM_021140	chrX:44971784-44971843	KDM6A
A_23_P255653	8.99647	8.480158	NM_003844	chr8:23049312-23049253	TNFRSF10A
A_23_P336612	3.6717439	3.5484123	NM_173528	chr15:81441233-81441292	C15orf26
A_23_P73982	8.797011	8.928362	NM_018087	chr1:54233538-54233479	NDC1
A_33_P3347132	8.626977	8.647105	NM_014285	chr9:133579095-133579154	EXOSC2
A_33_P3363690	5.7667723	5.740258		chr4:013979769-013979828	
A_33_P3332970	5.397279	5.2998543	NM_005127	chr12:10005037-10004978	CLEC2B
A_33_P3257703	4.4075108	4.9088283	NM_203299	chr9:35045807-35045866	C9orf131
A_24_P406245	7.444126	7.24331	BC010535	chr7:72492954-72493013	
A_33_P3280965	9.157548	9.185856	NR_003142	chr16:2015445-2015504	SNHG9
A_33_P3278362	6.8010178	6.123601	NM_020349	chr10:99343557-99343616	ANKRD2
A_24_P280497	5.8738112	5.729842	NM_001142641	chr12:133159754-133159813	FBRSL1
A_23_P15045	7.0796432	7.0667562	NM_004424	chr16:2285440-2285499	E4F1
A_33_P3214056	7.2246337	7.191671	NR_024584	chr1:144480832-144480774	LOC728875
A_24_P339944	2.5660706	2.3900566	NM_002608	chr22:39620158-39620099	PDGFB
A_33_P3284939	9.678596	9.5784	NM_199129	chr20:48740366-48740307	TMEM189
A_23_P164022	15.183809	15.239232	NM_033375	chr17:1368434-1368375	MYO1C
A_23_P212383	8.398528	8.704763	NM_014016	chr3:45786011-45786070	SACM1L
A_33_P3391626	13.342301	13.526618		chr19:034513738-034513679	
A_33_P3318671	11.122928	10.868946	NM_007260	chr1:24121957-24122016	LYPLA2
A_24_P233944	4.0945826	4.0135803	NM_001007794	chr1:111726084-111726143	CEPT1
A_23_P215088	8.471514	8.854102	NM_016478	chr7:129658450-129658391	ZC3HC1
A_33_P3341901	9.629012	9.122016	NM_058163	chrX:54471669-54471728	TSR2
A_33_P3293913	4.282597	4.5330544	NM_001080512	chr10:60588786-60588845	BICC1
A_23_P119907	4.7148485	5.07876	NM_018089	chr2:220100233-220100292	ANKZF1
A_33_P3307402	4.495825	4.432361		chr6:154840150-154840091	
A_23_P389987	3.7642934	3.292569	NM_016170	chr2:74743804-74743863	TLX2
A_33_P3404954	10.353236	10.417723	NM_001172702	chr14:61550371-61550430	SLC38A6
A_23_P48455	6.4730377	6.740841	NM_030943	chr14:103397053-103397112	AMN
A_23_P400945	5.4735756	5.76289	NM_005240	chr1:157103323-157103264	ETV3
A_33_P3339202	6.1333632	6.422513		chr1:031974776-031974835	
A_23_P141974	10.606349	10.819114	NM_003290	chr19:16212086-16212145	TPM4
A_33_P3264875	5.904082	6.0902963	NM_001004105	chr5:176867938-176867997	GRK6
A_23_P500271	5.508731	5.161072	NM_001098627	chr7:128589323-128589382	IRF5
A_33_P3332744	3.8744178	3.8930635	NM_004762	chr17:76671834-76671775	CYTH1
A_24_P280762	4.0073276	4.1872644	NM_030568	chr6:73951921-73951862	KHDC1
A_33_P3245133	5.647027	5.7833557		chr7:056437086-056437027	
A_33_P3764802	6.9011364	6.5621614	NM_012241	chr6:13612209-13612268	SIRT5
A_23_P213431	9.094298	8.911225	NM_032380	chr5:74017216-74017157	GFM2
A_33_P3414122	8.564591	8.438826	NM_001012756	chr19:37001663-37001604	ZNF260
A_33_P3250612	5.7412233	5.7303677	NM_005943	chr6:39873491-39873432	MOCS1
A_23_P417282	8.302593	8.259384	NM_000875	chr15:99507366-99507425	IGF1R
A_33_P3318027	9.955585	10.161468	NM_001320	chr6:31637220-31637279	CSNK2B
A_23_P143143	11.354872	11.001852	NM_002166	chr2:8822642-8823030	ID2
A_23_P170587	11.850737	11.720884	NM_020197	chr1:214510246-214510305	SMYD2
A_23_P111797	6.491426	6.6346817	XR_242367	chr7:65306579-65306638	DKFZP434F142
A_33_P3376463	5.755817	5.782801	BC051670	chr3:15079609-15079668	NR2C2
A_24_P296508	4.878249	4.977684	NM_152346	chr17:1477761-1477702	SLC43A2
A_23_P24633	8.65471	8.621362	NM_199297	chr11:134119120-134119061	THYN1
A_33_P3258617	4.0209684	4.650141		chr9:19123462-19123403	
A_23_P160869	6.507464	6.639808	NM_014813	chr1:113667055-113667114	LRIG2
A_33_P3270203	5.3643913	5.514924	NM_001257281	chr2:233001259-233001318	DIS3L2
A_24_P267293	3.397655	2.3900566	NM_015077	chr17:26727741-26727800	SARM1
A_23_P24755	7.534216	7.5088835	NM_003164	chr11:62574597-62574538	STX5
A_33_P3403392	3.9183936	3.8999164	NM_023928	chr12:125613943-125614002	AACS

A_23_P12989	10.5309305	10.786566	NM_012094	chr11:64088515-64089091	PRDX5
A_33_P3214209	7.9587917	7.7939425	NR_027027	chr5:21474481-21474540	GUSBP1
A_33_P3305958	4.152505	3.8697941	NM_001172631	chr14:102900827-102900886	TECPR2
A_33_P3219245	4.734852	5.304996	NR_023390	chr9:98568545-98568486	LINC00476
A_23_P136870	3.8801718	3.8083	NM_175868	chrX:151870169-151870228	MAGEA6
A_24_P93855	5.2536445	4.966527	NM_001012426	chr6:41568054-41568113	FOXP4
A_24_P615822	6.366758	6.145152	NM_014753	chr10:43279997-43280967	BMS1
A_24_P678418	3.9214635	4.131047	NR_015415	chr14:95646100-95646159	DICER1-AS1
A_23_P131208	3.06639	3.2512817	NM_006186	chr2:157181786-157181727	NR4A2
A_33_P3216621	5.4247904	5.6820393		chr16:054371169-054371228	
A_33_P3307500	6.7615633	6.5802917	NM_001142620	chr15:74486784-74486725	STRA6
A_33_P3365352	6.3973713	6.700514	NR_049793	chr6:138187838-138187779	LOC100130476
A_23_P22915	4.8874636	5.0178003	NM_133496	chr1:101431369-101431428	SLC30A7
A_23_P141738	8.122292	8.532848	NM_001007559	chr18:23596972-23596913	SS18
A_33_P3841819	6.430058	6.5340815	NR_027007	chr3:9431671-9431612	SETD5-AS1
A_24_P186944	12.048147	11.921628		chr15:66671636-66671608	TIPIN
A_33_P3373358	10.582123	10.61262	NM_005497	chr17:42875875-42875816	GJC1
A_33_P3323914	12.300437	12.31344		chr11:012983354-012983295	
A_23_P83234	3.8320518	3.7656605	NM_006626	chr9:125671190-125671131	ZBTB6
A_33_P3228366	7.1255684	7.0576954	NM_020750	chr6:43494483-43494424	XPO5
A_23_P63980	8.283178	8.24211	NM_024036	chr11:66627660-66627719	LRFN4
A_32_P28939	9.556538	9.391273	NM_001001655	chr12:109526142-109526083	ALKBH2
A_33_P3246950	3.955484	3.853053		chr7:13894064-13894005	
A_23_P46369	9.393226	9.75809	NM_002870	chr1:153955751-153955282	RAB13
A_33_P3422301	3.8452365	3.7010279	NM_032406	chr5:140866830-140866889	PCDHGC4
A_24_P322635	9.525609	9.392318	NM_182764	chr20:44994828-44994769	ELMO2
A_33_P3315763	16.538507	16.507711		chr17:026889952-026889893	
A_33_P3503937	8.784712	8.413981	AK094426	chr1:205861553-205861612	LOC284581
A_33_P3356990	4.399301	4.0347276	NM_130464	chr16:21413514-21413455	NPIPB3
A_23_P98057	6.5907516	6.8242016	NM_001005368	chr10:44140152-44140093	ZNF32
A_23_P88781	8.3071785	8.251077	NM_020313	chr16:57462700-57462641	CIAPIN1
A_33_P3226761	4.836022	4.6150694	NM_018419	chr20:62679140-62679081	SOX18
A_23_P58396	10.917094	10.488684	NM_016205	chr4:157682958-157682899	PDGFC
A_23_P92948	7.837289	8.071546	NM_130809	chr5:126874767-126874826	PRRC1
A_33_P3338937	4.4833727	4.789376		chr17:048874755-048874696	
A_33_P3417865	13.711731	13.671231	NM_004926	chr14:69255413-69255354	ZFP36L1
A_33_P3280721	6.8739595	6.889674	NM_001002292	chr1:68564267-68564208	WLS
A_24_P414371	8.071546	8.158992	NM_000944	chr4:101945353-101945294	PPP3CA
A_23_P203900	7.6697435	7.649143	NM_005505	chr12:125262284-125262225	SCARB1
A_33_P3265593	7.4884577	7.7206388	AK128756	chr9:70348758-70348817	LOC100132790
A_24_P262407	4.1205544	4.222075	NM_199334	chr17:38245528-38245587	THRA
A_33_P3290343	4.7548704	5.242975	NM_000104	chr2:38297765-38297706	CYP1B1
A_24_P83678	6.667677	6.9011364	NM_198468	chr6:97597747-97594723	MMS22L
A_23_P160240	4.866154	4.8832746	NM_016361	chr1:147127356-147127297	ACP6
A_24_P342096	7.0032144	7.390796	NR_024060	chr9:45728209-45728268	FAM27A
A_33_P3235766	9.7539425	8.081035	NM_001076786	chr11:33001700-33001759	QSER1
A_33_P3329991	12.102104	12.394055			
A_23_P217899	5.893821	5.8889523	NM_030937	chr1:1321399-1321340	CCNL2
A_23_P116743	7.7819	7.7626257	NR_002809	chr12:122233266-122233207	LOC338799
A_33_P3342260	4.378404	4.2605658	NM_000162	chr7:44183938-44183879	GCK
A_24_P124349	4.1152515	3.9408236	NM_025208	chr11:103778445-103778386	PDGFD
A_32_P125338	8.401861	8.121649	NM_207334	chr1:20881440-20881499	FAM43B
A_23_P150092	10.723414	10.726904	NM_012247	chr10:13361160-13361101	SEPHS1
A_23_P87310	2.7373495	2.3900566	NM_002315	chr11:8245980-8245921	LMO1
A_33_P3376745	3.79009	3.6439977		chr15:090838212-090838271	
A_23_P27353	5.3413167	5.237788	NM_007163	chr18:43262570-43262629	SLC14A2
A_23_P19852	8.771827	8.699083	NM_152558	chr7:2654270-2654329	IQCE
A_33_P3378531	16.151003	16.204042	NM_020682	chr10:104660633-104660692	AS3MT
A_33_P3379456	7.5355325	7.636244	NM_001282687	chr2:218223-218164	SH3YL1

A_24_P303589	9.336257	9.541637	NM_003592	chr7:148495708-148495767	CUL1
A_33_P3645805	13.682939	13.638138	AK021797	chr1:206091729-206091670	
A_23_P28466	4.0962114	3.260577	NM_178821	chr2:228786256-228788687	DAW1
A_23_P136405	4.252093	4.9822083	NM_005018	chr2:242792108-242792049	PDCD1
A_24_P276888	6.880954	6.963524	NM_001199803	chr2:25044791-25044850	CENPO
A_23_P73023	8.205227	8.588215	NM_015173	chr4:38134749-38134808	TBC1D1
A_24_P349002	10.098356	9.777238	NM_172020	chr7:72417943-72418002	POM121
A_24_P712350	8.32017	8.071796	NM_001821	chr1:241792606-241792547	CHML
A_24_P701776	4.921529	4.645653	NM_001003702	chr7:143884665-143884606	ARHGEF35
A_33_P3281616	8.830886	8.600946	NM_207327	chr22:46640007-46639948	CDPF1
A_33_P3399560	6.468508	6.2310796	NM_001177701	chr22:37163889-37163830	IFT27
A_23_P99930	9.1318245	8.688465	NM_017858	chr15:66629272-66629213	TIPIN
A_33_P3355055	6.454549	6.1886425	NM_001278378	chr12:123465758-123465817	ARL6IP4
A_23_P29975	2.3221061	2.3900566	NM_018302	chr4:37593257-37593316	C4orf19
A_32_P150300	5.5372386	5.874843	NR_104626	chr1:101701366-101701307	LOC101928370
A_23_P13772	4.5813537	4.4658537	NM_016569	chr12:115109713-115109654	TBX3
A_24_P192197	8.1137705	7.9519696	NM_130395	chr6:2785385-2785444	WRNIP1
A_23_P421401	7.9865203	7.916069	NM_002609	chr5:149493523-149493464	PDGFRB
A_32_P183609	5.4164343	5.0861244	NM_001040445	chr2:239360417-239360476	ASB1
A_23_P147900	4.162922	4.199911	NM_007252	chr7:39504255-39504314	POU6F2
A_32_P76811	13.565643	13.579134	NR_026972	chr6:29694594-29694535	HLA-F-AS1
A_33_P3281010	4.0951605	4.89968		chr9:42468064-42468123	FAM95B1
A_33_P3827416	15.587135	15.63471	AK024156	chr10:49264602-49264543	
A_24_P324011	6.055647	6.319057	NM_015353	chr17:73061300-73061359	KCTD2
A_24_P69439	11.127678	10.861808	NM_030780	chr8:104410937-104410878	SLC25A32
A_33_P3249574	5.375851	5.0226364	NM_004866	chr5:77776394-77776453	SCAMP1
A_23_P152655	2.3760443	2.3900566	NM_000873	chr17:62080019-62079960	ICAM2
A_23_P171249	9.896127	9.915903	NM_001551	chrX:69385969-69386028	IGBP1
A_23_P372144	9.370357	9.285682	NM_021231	chr19:3610779-3610720	CACTIN
A_23_P418413	9.100391	9.435334	NM_005109	chr3:38296728-38296787	OXSR1
A_33_P3676515	2.8154364	3.1346974		chr21:46490932-46490873	SSR4P1
A_23_P208674	8.814966	9.023564	NM_175063	chr19:50986430-50986489	EMC10
A_23_P62890	5.884002	6.2939854	NM_002053	chr1:89518848-89518789	GBP1
A_23_P258124	3.8103294	3.3993106	NM_012279	chr5:176493232-176493291	ZNF346
A_33_P3285444	6.9992833	6.433487	NM_017489	chr8:73959745-73959805	TERF1
A_23_P154411	10.066283	9.834597	NM_004792	chr2:170493828-170493887	PPIG
A_33_P3252286	7.468062	7.3437166	NM_004750	chr19:18704106-18704047	CRLF1
A_33_P3424222	5.800823	5.8223753	NM_002123	chr6:32629184-32629125	HLA-DQB1
A_24_P372562	10.902374	10.753524	NM_175732	chr11:47593122-47593181	PTPMT1
A_23_P92362	12.265369	12.386805	NM_002494	chr4:140213694-140211198	NDUFC1
A_23_P334021	8.089315	8.223315	NM_000876	chr6:160527114-160527173	IGF2R
A_23_P209032	7.044625	7.2825003	NM_018443	chr19:35176592-35176651	ZNF302
A_23_P252082	4.1768503	4.093318	NM_018487	chr7:150502028-150502087	TMEM176A
A_23_P16166	5.0938907	4.811835	NM_006702	chr19:7625582-7625641	PNPLA6
A_23_P30069	6.800461	7.0837283	NM_001012967	chr4:169278384-169278328	DDX60L
A_23_P215566	7.4755106	7.8297863	NM_001621	chr7:17384999-17385058	AHR
A_24_P190472	4.9159102	5.449656	NM_003064	chr20:43882279-43882220	SLPI
A_24_P301186	6.0314264	5.842947	NM_001008726	chr14:64064455-64064396	WDR89
A_23_P200551	9.278196	9.338753	NM_032236	chr1:22005812-22005753	USP48
A_23_P119095	8.925818	8.89252	NM_006663	chr19:45883072-45883013	PPP1R13L
A_23_P152858	10.745641	10.73518	NM_018405	chr17:30179032-30178973	COPRS
A_33_P3368188	5.307687	4.9899635	NM_006640	chr17:75478357-75478416	SEPT9
A_23_P301877	6.5167065	6.239206	NM_004269	chr9:134889828-134889769	MED27
A_23_P11744	9.86075	9.779233	NM_182905	chr9:14773-14714	WASH1
A_33_P3244882	6.5619364	6.9152927	NM_024852	chr1:36439052-36439111	AGO3
A_33_P3399028	6.759975	6.5268483	NM_006852	chr17:60629675-60629734	TLK2
A_32_P89679	7.006458	6.164159	NM_001013620	chr12:38722948-38723007	ALG10B
A_23_P97795	8.303098	8.591741	NM_145698	chr10:27493451-27493392	ACBD5
A_33_P3419165	4.2234073	3.5105782	NM_013342	chr19:54618723-54618664	TFPT

A_24_P346886	12.534498	12.594133	NM_005004	chr10:102283579-102283520	NDUFB8
A_23_P46606	9.061892	8.186397	NM_014873	chr1:211923228-211923169	LPGAT1
A_23_P152055	8.719109	8.627237	NM_024580	chr15:82422717-82422658	EFTUD1
A_24_P244952	9.154627	9.2285185	NM_015092	chr16:18816853-18816794	SMG1
A_23_P80321	12.213637	12.086992	NM_021974	chr22:38363169-38363682	POLR2F
A_33_P3310296	4.9521637	5.118637		chr20:043252771-043252830	
A_23_P141248	5.71321	5.728876	NM_021947	chr17:2227643-2227702	SRR
A_33_P3221438	8.021079	8.457683	NM_152531	chr3:194789120-194789061	XXYLT1
A_33_P3281151	9.478785	9.656423	NM_003902	chr1:78414935-78414876	FUBP1
A_23_P79999	7.7969275	7.9985027	NM_001247	chr20:25207043-25207102	ENTPD6
A_33_P3275873	4.282284	3.7663755	NM_000542	chr2:85885472-85885413	SFTPB
A_23_P69863	2.3221061	2.3900566	NM_015669	chr5:140517455-140517514	PCDHB5
A_23_P328237	5.751359	5.9806275	NM_194285	chr11:18628959-18628900	SPTY2D1
A_33_P3460043	5.991167	6.1071796	NR_027071	chr8:104145252-104145193	C8orf56
A_33_P3292130	4.511349	4.5801187		chr7:45851074-45851133	XLOC_014512
A_33_P3368109	4.9065194	4.925545		chr9:098982070-098982129	
A_23_P376449	7.1803274	7.4444027		chr10:89148611-89148552	XLOC_014512
A_33_P3342822	4.1509485	4.260907	BX646214	chr9:42494610-42494669	
A_33_P3216694	4.6458216	3.8546896	NM_024503	chr1:41975744-41975685	HIVEP3
A_24_P734953	10.014084	9.531587	NM_001013642	chr1:27326501-27326560	TRNP1
A_33_P3266769	3.9251919	3.2426972		chr2:48935968-48935909	LHCGR
A_23_P352358	8.715376	8.999543	NM_138346	chr1:11980356-11980297	KIAA2013
A_33_P3363799	6.9982204	6.7545004	NM_001242607	chr11:113149074-113149133	NCAM1
A_23_P91512	2.5246549	2.3900566	NM_144492	chr21:37833014-37832955	CLDN14
A_23_P311144	6.987171	6.7165465	NM_144978	chr2:109473349-109473408	CCDC138
A_23_P66694	5.9870872	4.9602575	NM_006495	chr17:29631839-29631780	EVI2B
A_23_P72503	8.818959	8.281347	NM_007246	chr4:166243858-166243917	KLHL2
A_24_P93206	4.4559917	4.918062	NM_001286390	chr14:105061569-105061510	TMEM179
A_33_P3219697	13.498016	13.560461	NM_198493	chr1:173577774-173577715	ANKRD45
A_23_P414964	6.108982	6.656313	NM_173548	chr19:58929400-58929459	ZNF584
A_24_P332341	7.776242	7.8388395	NR_003655	chr7:44056055-44054346	POLR2J4
A_33_P3241657	6.196144	6.316386	NM_024766	chr2:44599967-44600026	CAMKMT
A_33_P3213767	6.1054554	6.296438	BC018040	chr22:30762715-30762774	CCDC157
A_24_P221968	8.054075	7.7829137		chr16:53404723-53404664	LOC643802
A_24_P25326	6.1713095	5.950123	NM_007167	chr1:35452948-35452889	ZMYM6
A_24_P242688	8.247517	8.67049	NM_000182	chr2:26415256-26415197	HADHA
A_33_P3243439	7.0322657	7.58308	NM_019858	chr12:6936523-6936582	GPR162
A_23_P30275	6.4368033	6.547765	NM_024028	chr5:148749022-148749081	PCYOX1L
A_23_P129209	8.148889	8.649179	NM_002168	chr15:90630706-90630472	IDH2
A_33_P3351944	4.770904	4.6155605	NM_201283	chr7:55224558-55224617	EGFR
A_23_P132784	9.889473	9.952806	NM_001013439	chr3:180694169-180694228	FXR1
A_33_P3235390	8.035044	7.5202866	NM_024818	chr3:132396464-132396523	UBA5
A_23_P92860	8.096398	7.819146	NM_021147	chr5:54527110-54527051	CCNO
A_23_P15073	6.049929	6.0357122	NM_001005920	chr16:732615-732556	JMJD8
A_23_P92410	9.846745	10.015118	NM_004346	chr4:185549080-185549021	CASP3
A_24_P23445	5.290155	5.0057607	NR_029422	chr22:43011300-43011359	RNU12
A_33_P3293346	6.341478	6.568267	NM_016121	chr1:215785203-215785262	KCTD3
A_23_P69226	8.534889	8.708657	NM_018447	chr3:10018659-10016132	EMC3
A_33_P3215640	3.8832693	4.3865385	NM_153370	chr6:36932552-36932611	PI16
A_33_P3222056	4.294273	4.1798987		chr8:11416180-11416121	
A_23_P383009	3.9883847	2.9153037	NM_000599	chr2:217537226-217537167	IGFBP5
A_23_P23346	12.406378	12.245443	NM_006818	chr1:151040758-151040817	MLLT11
A_33_P3258846	6.880237	7.1719007		chr19:019002848-019002907	
A_24_P355267	9.37877	8.934475	NM_001006641	chr9:130871435-130871494	SLC25A25
A_23_P8640	4.89968	4.403175	NM_001039966	chr7:1133336-1133395	GPER1
A_23_P202071	2.3221061	2.3900566	NM_001025077	chr10:11376225-11376284	CELF2
A_33_P3420053	2.3221061	2.3900566	NM_001136038	chr19:53611413-53611354	ZNF415
A_23_P92042	5.329921	5.5017366	NM_002222	chr3:4888682-4888741	ITPR1
A_23_P91919	10.996225	11.065117	NM_014445	chr3:150260406-150260347	SERP1

A_23_P63050	10.763852	10.832217	NM_000374	chr1:45480655-45481044	UROD
A_23_P113748	6.57219	6.85849	NM_024697	chr3:21478580-21478521	ZNF385D
A_24_P207150	7.3109226	7.3396583	NM_130839	chr15:25582924-25582865	UBE3A
A_32_P221822	7.0715423	6.3023005	NM_016052	chr1:218511102-218511161	RRP15
A_23_P213718	13.128433	13.160629	NM_014402	chr5:132203295-132203354	UQCRQ
A_23_P20035	4.7421236	5.1216097	NM_138445	chr7:1098557-1098616	GPR146
A_24_P178300	4.338008	4.679688	NM_001080835	chr2:240981376-240981317	PRR21
A_33_P3246990	9.450083	9.092338	NM_001167	chrX:123047703-123047762	XIAP
A_23_P405148	10.6778555	10.466264	NM_020194	chr2:228221775-228221834	MFF
A_33_P3210139	8.823239	8.378871	NM_015885	chr11:82896736-82896795	PCF11
A_23_P18292	14.828998	14.805878	NM_001034996	chr3:40503688-40503748	RPL14
A_24_P301837	5.022897	5.6799	NM_007232	chr20:60791786-60791727	HRH3
A_23_P98022	8.57851	8.619221	NM_012238	chr10:69677560-69677619	SIRT1
A_23_P131935	8.641283	8.309002	NM_017671	chr20:6055889-6055830	FERMT1
A_24_P62800	2.3221061	2.3900566	NM_018203	chr1:205305747-205305688	KLHDC8A
A_23_P354805	6.3151207	5.5369024	NM_007249	chr13:74260392-74260333	KLF12
A_24_P373768	5.7330456	5.839156	NM_000164	chr19:46181460-46184896	GIPR
A_33_P3360675	6.4813247	6.4395947	NM_001164761	chr7:618953-618894	PRKAR1B
A_33_P3269862	5.776423	5.781561		chr6:031334405-031334346	
A_23_P200710	8.720944	8.916017	NM_002646	chr1:204391950-204391891	PIK3C2B
A_23_P18325	10.7008915	10.841731	NM_007217	chr3:167402154-167402095	PDCD10
A_33_P3372189	13.630709	13.512057	BC029410	chr7:56804002-56803943	
A_32_P72394	5.743804	5.7055063	NM_002735	chr7:720361-720302	PRKAR1B
A_23_P334751	7.4683924	7.522696	NM_152490	chr1:235613554-235613495	B3GALNT2
A_33_P3346538	4.616888	4.609747		chr16:69776228-69776287	
A_33_P3321055	5.8115587	5.6100736	BC035796	chr13:24168423-24168482	TNFRSF19
A_23_P164237	8.443366	8.586117	NM_018428	chr17:30200435-30195111	UTP6
A_24_P942694	6.1071796	6.242222	NM_018017	chr10:115882172-115882113	C10orf118
A_33_P3371320	6.515606	6.6756105	NM_001261840	chr2:28521247-28521306	BRE
A_23_P78209	8.605041	8.270276	NM_002359	chr17:79876286-79876227	MAFG
A_23_P22994	7.312498	7.427405	NM_014223	chr1:41232354-41235065	NFYC
A_33_P3242080	10.60462	10.639288	NM_018332	chr16:70407128-70407187	DDX19A
A_24_P194688	5.6466627	5.832809	NM_181723	chr8:16979882-16979941	MICU3
A_23_P81241	10.8946705	10.793166	NM_030571	chr5:141532114-141532173	NDFIP1
A_33_P3272921	5.6953545	6.492727	NM_005224	chr19:972667-972726	ARID3A
A_23_P369479	5.5030675	4.780237	NM_170721	chr17:55710117-55710176	MSI2
A_24_P234856	4.628119	4.040212	NM_001745	chr5:134087772-134087831	CAMLG
A_33_P3216714	5.7422047	4.223155	NM_001256864	chr1:65880801-65880860	DNAJC6
A_33_P3302632	4.149013	4.566723	NM_003523	chr6:26184399-26184458	HIST1H2BE
A_33_P3246543	6.189319	6.560549	NM_182796	chr5:162939135-162939194	MAT2B
A_24_P159094	8.991302	8.629412	NM_175748	chr14:93695362-93695421	UBR7
A_24_P693461	2.3221061	2.3900566	NM_001101341	chr14:36942820-36942761	SFTA3
A_23_P35148	5.040458	4.928867	NM_005645	chr1:109607271-109607212	TAF13
A_24_P914513	5.3165846	5.6442685	NM_183050	chr6:81055230-81055289	BCKDHB
A_24_P520767	7.58018	7.7056274		chr1:91297731-91297672	LOC149351
A_24_P187921	5.0574703	5.552109	NM_024641	chr6:96034591-96034650	MANEA
A_33_P3210521	6.140853	5.185571	NM_021925	chr2:20883887-20883828	C2orf43
A_23_P419239	7.3281727	7.7710648	NM_018638	chr12:22826525-22826584	ETNK1
A_23_P303718	9.045843	8.917393	NM_015548	chr6:56323594-56323535	DST
A_23_P357284	3.111399	2.9974437	NM_005282	chr19:46093270-46093211	GPR4
A_33_P3384795	3.0252945	2.3900566	AK093443	chr9:43029503-43029444	FAM95B1
A_23_P2645	4.5017076	4.4369593	NM_006843	chr12:113830767-113830708	SDS
A_23_P363313	5.044646	5.1677456	NM_153357	chr17:6945224-6945165	SLC16A11
A_33_P3404411	6.3701143	6.7711124	NM_005932	chr13:24334295-24334236	MIPEP
A_24_P258051	7.1728954	7.162508	NM_032844	chr10:27459944-27460003	MASTL
A_24_P370887	9.182217	9.298541	NM_004781	chr1:7840956-7841015	VAMP3
A_23_P357248	3.4660888	3.4737518	NM_024325	chr20:2474457-2474198	ZNF343
A_33_P3352304	2.934075	2.3900566	XR_113307	chrX:55682871-55682812	XAGE-4
A_33_P3362193	12.341772	12.200829	NM_005053	chr19:13064389-13064448	RAD23A

A_32_P857658	15.546938	15.526994	NM_001003	chr15:69747814-69747873	RPLP1
A_23_P111297	8.610489	8.53804	NM_006638	chr6:4996277-4996218	RPP40
A_33_P3327617	3.9204366	4.0273347	NM_201563	chr1:161561128-161561187	FCGR2C
A_33_P3279379	7.70101	8.0436325	NR_033796	chr12:6994312-6994371	DSTNP2
A_33_P3414574	8.128782	7.854101	NM_005880	chr16:46989352-46989293	DNAJA2
A_33_P3319765	5.1124616	4.897033	NM_001243744	chr9:97872930-97872871	FANCC
A_23_P416965	7.9814034	8.329617	NM_015398	chr4:187088233-187088379	FAM149A
A_23_P119418	7.717627	8.055726	NM_003796	chr19:30503228-30503287	URI1
A_33_P3326099	5.010709	4.979638	NM_003934	chr9:133491803-133491862	FUBP3
A_24_P135501	13.285886	13.197346		chr15:048022876-048022815	
A_23_P395464	2.8572116	2.3900566	NM_144690	chr19:56894761-56894702	ZNF582
A_33_P3254380	5.758069	4.1887226	NM_001257291	chrX:46466224-46466165	SLC9A7
A_23_P52885	6.6730084	6.871932	NM_024631	chr11:124637239-124637180	MSANTD2
A_32_P81676	4.0103164	5.100659	NR_024281	chr8:9758237-9758102	LINC00599
A_23_P206684	4.763158	4.9153156	NM_199424	chr16:69974995-69975054	WWP2
A_23_P423926	7.4267755	7.730288	NM_198935	chr20:60757079-60757138	SS18L1
A_23_P330070	4.6616297	5.6044765	NM_001032281	chr2:188343465-188343406	TFPI
A_33_P3299811	5.362341	4.7180285	NR_027286	chr1:213031260-213031201	FLVCR1-AS1
A_23_P22129	5.513728	5.8381395	NM_177977	chr17:39879840-39879781	HAP1
A_23_P391506	7.861485	7.904107	NM_006469	chr1:185266169-185266110	IVNS1ABP
A_33_P3779229	4.4660354	4.4307094	NM_001039547	chr3:141883589-141883530	GK5
A_24_P237878	9.650277	9.838757	NM_207368	chr17:79780401-79780342	FAM195B
A_33_P3293734	8.1704	7.983845		chr5:092604130-092604189	
A_24_P192262	8.31072	8.479695	NM_005402	chr7:39747399-39747458	RALA
A_23_P317347	6.593206	6.738345	NM_052911	chr18:19109806-19109747	ESCO1
A_33_P3248654	11.451174	11.067415	NM_006513	chr1:109780709-109780768	SARS
A_23_P79818	9.962486	10.056347	NM_016470	chr20:42825757-42825698	OSER1
A_24_P160413	5.7015686	5.5000196	NR_026850	chr1:32697338-32697279	MTMR9LP
A_24_P309415	11.20102	11.211504	NM_052932	chr11:102267545-102267486	TMEM123
A_23_P88119	12.004002	11.480977	NM_006644	chr13:31710855-31710796	HSPH1
A_23_P138760	7.3737464	7.4708767	NM_013246	chr11:67132122-67132063	CLCF1
A_23_P110624	2.3221061	2.8310142	NM_001332	chr5:10973143-10973084	CTNND2
A_33_P3252635	6.944681	6.775626	NM_001278665	chr19:24289385-24289444	ZNF254
A_23_P212595	4.393775	3.9635687	NM_014703	chr3:51433957-51433898	VPRBP
A_33_P3224891	6.770384	7.036346	AK129559	chrX:48432961-48432902	
A_24_P122636	8.0194645	7.8556585	NM_006085	chr1:220231157-220231098	BPNT1
A_23_P356004	8.23133	8.207529	NM_013434	chr2:96051766-96051825	KCNIP3
A_33_P3718352	6.7076406	6.8363543		chr10:86054356-86054415	LINC00858
A_32_P138042	4.700032	4.800843	BX445743	chrX:077381418-077381359	
A_33_P3380462	7.2782273	7.448288	NM_022049	chr1:101005696-101005755	GPR88
A_24_P243396	8.221958	7.881619	NM_001008657	chr5:149763654-149763713	TCOF1
A_24_P401090	8.436161	8.545707		chr1:039175954-039176015	
A_23_P160934	7.704346	7.907386	NM_030920	chr1:150204124-150202979	ANP32E
A_23_P132417	8.872437	8.974696	NM_018385	chr3:194361992-194361933	LSG1
A_33_P3235360	5.2577834	5.233269		chr16:005312247-005312188	
A_23_P315571	9.178626	8.674129	NM_015150	chr3:16357514-16357455	RFTN1
A_23_P37654	9.604943	9.640451	NM_001164273	chr15:42061855-42061914	MGA
A_24_P159181	10.01922	9.793676	NM_005707	chr15:65421454-65421395	PDCD7
A_33_P3844650	7.8869476	8.094422	NM_012098	chr9:129850994-129850935	ANGPTL2
A_23_P63243	10.278298	10.409571	NM_138740	chr1:154179830-154179771	C1orf43
A_24_P8109	3.7312005	3.6065817	NM_001012302	chr11:418916-418783	ANO9
A_33_P3362143	4.6464267	4.5164533	NR_026962	chr22:28320884-28320943	TTC28-AS1
A_33_P3328426	11.858713	11.944817	NM_001204831	chr3:43407920-43407861	ANO10
A_33_P3760937	5.3879523	5.4922705	XR_250496	chr9:90459517-90459458	LOC497256
A_23_P356139	7.3094645	7.416742	NM_018121	chr10:102724369-102724428	FAM178A
A_23_P204947	7.8425303	7.2283554	NM_004004	chr13:20761974-20761915	GJB2
A_23_P217228	3.2178135	3.4647973	NM_016157	chrX:54957644-54957703	TRO
A_24_P259276	8.303669	8.341151	NM_207340	chr11:66306938-66306879	ZDHHC24
A_33_P3240353	9.485801	9.679291	NM_017767	chr8:145637858-145637799	SLC39A4

A_33_P3238177	9.575886	9.273457	NM_001007094	chr10:38412110-38412169	ZNF37A
A_33_P3234641	13.0984	13.153513	BU566292	chr14:50053013-50052719	
A_23_P170088	3.7343905	3.8826132	NR_104599	chr9:140287094-140287035	EXD3
A_23_P361584	3.6562836	2.3900566	NM_152680	chr4:153547361-153547302	TMEM154
A_23_P259863	3.9834235	4.208373	NM_020406	chr19:43866549-43866608	CD177
A_33_P3379956	5.3594112	4.4434347	AK126066	chr11:30964811-30964752	
A_23_P259586	9.866744	10.020062	NM_003318	chr6:80749430-80749489	TTK
A_24_P231829	3.2314794	3.6969368	NM_017614	chr5:78384375-78384434	BHMT2
A_24_P917819	3.9470692	4.1997113		chr21:14439275-14439334	ANKRD30BP2
A_24_P101047	4.8951983	5.201668	NM_020931	chr6:56919360-56919419	KIAA1586
A_23_P394323	3.8146625	3.1159995	AK095745	chr15:38775061-38775120	FAM98B
A_33_P3295423	6.2462683	6.3593807	NM_001040653	chr3:126177730-126177671	ZXDC
A_23_P118462	11.246752	11.296958	NM_080822	chr17:1946601-1946660	OVCA2
A_24_P417935	5.0369325	4.9058075	NR_027032	chr12:58121827-58121886	AGAP2-AS1
A_33_P3343432	7.872352	7.587314	NM_053055	chr1:151867544-151867485	THEM4
A_33_P3385351	4.174982	4.44366		chr1:002629340-002629281	
A_24_P111054	5.122322	5.5948524	BC035878	chr1:9100715-9100656	SLC2A5
A_33_P3312039	7.3446684	7.2230926	NM_002874	chr9:110081100-110081159	RAD23B
A_33_P3246883	6.213171	6.358934	NM_033317	chr19:35990920-35990861	DMKN
A_33_P3429576	8.776428	9.301542	NM_001284	chr5:115249151-115249210	AP3S1
A_23_P65830	7.170601	7.477108	NM_198527	chr15:91474362-91474303	HDDC3
A_24_P941505	11.204557	11.403077	NM_014612	chr9:96327562-96327621	FAM120A
A_32_P202703	8.128647	8.538477	NM_001242480	chr7_gl000195_random:44089-44030	LOC389831
A_23_P11353	9.5075655	10.030073	NM_005765	chrX:40464988-40465047	ATP6AP2
A_23_P354827	5.27728	5.397279	NM_001277090	chr19:58053312-58053253	ZNF550
A_24_P943922	3.455466	3.6818862	NM_020925	chr1:65157944-65158003	CACHD1
A_24_P405054	7.295369	7.2413588	NM_001114600	chr1:16721536-16721595	SZRD1
A_24_P339416	4.5886135	4.622114	NM_014960	chr17:66416529-66416588	ARSG
A_33_P3288364	9.234101	9.439858	NM_152339	chr16:89762825-89762766	SPATA2L
A_24_P338992	6.9411535	6.951689	NM_025083	chr15:74923334-74923275	EDC3
A_23_P259692	12.846558	12.057644	NM_058179	chr9:80944778-80944837	PSAT1
A_33_P3394378	6.127775	6.198984		chr9:067671021-067670962	
A_33_P3365002	7.7648993	7.843839	NM_001069	chr6:3154964-3154905	TUBB2A
A_23_P27035	2.3221061	2.3900566	NM_015544	chr17:31268428-31268487	TMEM98
A_24_P384755	5.8293576	5.765334	NM_024894	chr2:10803099-10799332	NOL10
A_24_P167825	5.9381795	5.9619026	NM_015289	chr15:42451768-42451709	VPS39
A_23_P101551	8.564386	9.0874815	NM_001190	chr19:49298622-49298563	BCAT2
A_23_P371885	10.158998	10.245807	NM_016632	chr17:44594232-44594173	ARL17A
A_32_P166693	9.873188	9.693044	NM_020733	chr3:124684882-124684823	HEG1
A_23_P54116	5.8182206	5.794906	NM_014992	chr14:59835594-59835653	DAAM1
A_24_P332471	12.937544	13.132952		chr2:085101956-085102015	
A_23_P141100	6.414828	6.1405997	NM_012091	chr16:75633770-75633711	ADAT1
A_33_P3218252	8.240465	8.078644	NM_015092	chr16:18887575-18887516	SMG1
A_23_P210253	9.766432	9.713281	NM_152879	chr2:234380636-234380695	DGKD
A_33_P3333982	4.749543	4.8938246	NM_022359	chr1:144951824-144951765	PDE4DIP
A_33_P3359308	5.451829	5.625507	NM_052920	chr2:23929475-23929534	KLHL29
A_24_P912372	5.972143	6.2973003	NM_015038	chr1:39881763-39881822	KIAA0754
A_33_P3502640	5.767686	5.553525	NM_020892	chr7:76134780-76134839	DTX2
A_24_P280328	4.039819	3.7484016	NR_001524	chrY:27877000-27877059	TTTY3
A_23_P16953	5.9242783	5.996362	NM_000867	chr2:231973300-231973241	HTR2B
A_33_P3263307	12.025342	11.747846	NM_001278651	chr22:41641680-41641621	RANGAP1
A_23_P64083	10.080616	10.091444	NM_006842	chr11:65831195-65835470	SF3B2
A_32_P2103	8.067934	8.515959		chr2:148782621-148782680	
A_23_P73239	10.934387	10.99204	NM_205842	chr2:183790076-183790017	NCKAP1
A_23_P3982	10.717293	10.769851	NM_032339	chr17:37885527-37885468	MIEN1
A_23_P16143	9.298204	9.420826	NM_002096	chr19:6380461-6380402	GTF2F1
A_33_P3256272	4.423313	4.380759	NM_198694	chr21:45999947-45999888	KRTAP10-5
A_33_P3288684	5.9266715	5.7332015		chr9:068175482-068175423	

A_23_P211106	6.4346986	6.0789557	NM_017438	chr21:37407353-37407294	SETD4
A_23_P55281	2.3221061	2.3900566	NM_004502	chr17:46685048-46684989	HOXB7
A_23_P20255	10.065823	9.968062	NM_014066	chr8:146076032-146075973	COMMD5
A_33_P3232280	7.827084	7.679099	NM_018164	chr12:27066562-27066503	ASUN
A_23_P103864	7.1378727	7.141268	NM_024525	chr1:231042303-231042244	TTC13
A_24_P113686	8.4423485	8.322927	NM_017953	chr1:86118985-86118926	ZNHIT6
A_24_P296070	7.2832785	7.5145974	NM_031431	chr13:46110179-46110238	COG3
A_23_P65208	6.4129434	6.6125617	NM_001039650	chr13:20411813-20411754	ZMYM5
A_23_P68240	6.878755	7.1016593	NM_207328	chr2:96687802-96687743	GPAT2
A_32_P132477	5.739237	5.835789		chr1:077165734-077165675	
A_33_P3331085	6.9671087	6.964199	NM_021982	chr5:134060716-134060775	SEC24A
A_23_P110076	7.464118	7.0927105	NM_182627	chr3:196281406-196281347	WDR53
A_24_P168574	6.3425756	6.175616	AJ224867	chr20:57475008-57475067	GNAS
A_24_P339514	4.37985	4.2935834	NM_000767	chr19:41524193-41524252	CYP2B6
A_23_P128574	5.8130875	5.9600024	NM_017993	chr13:43787918-43787859	ENOX1
A_23_P337800	3.8703885	4.03168	NM_172140	chr19:39789091-39789150	IFNL1
A_33_P3278103	3.5654721	3.0581608	NR_103451	chr9:137711325-137711266	LOC101448202
A_23_P55107	6.328259	5.960212	NM_014683	chr17:19677641-19677582	ULK2
A_33_P3333712	4.3827024	4.472479	NM_017988	chr12:100732441-100732950	SCYL2
A_23_P383915	6.9314284	6.730869	NM_144587	chr10:124097550-124097609	BTBD16
A_33_P3777584	4.5310106	4.847998	NM_032861	chr6:158530984-158530925	SERAC1
A_23_P15123	9.134692	9.223946	NM_019116	chr16:23582031-23582090	UBFD1
A_33_P3420083	5.405022	5.1270876	AK094642	chr1:1874574-1874515	LOC728690
A_32_P49848	8.116404	8.514936	NM_012249	chr2:46808256-46808315	RHOQ
A_23_P145006	7.6339936	7.7239413	NM_054023	chr5:147261662-147261721	SCGB3A2
A_33_P3373119	6.9283323	6.800048		chr19:033953336-033953277	
A_33_P3843285	3.8002393	3.1198504	XR_110023	chr3:65858567-65858508	
A_33_P3217517	4.4486923	3.7381186	NM_138464	chr5:442804-442745	C5orf55
A_23_P98483	9.313752	9.441832	NM_021211	chr11:10874603-10874544	ZBED5
A_24_P346587	8.809992	8.784468	NM_014637	chr8:66621870-66621929	MTFR1
A_24_P827491	9.637704	9.64168	NM_006191	chr6:112938170-112938111	PA2G4
A_23_P384499	9.622824	9.28156	NM_020761	chr17:78939983-78940042	RPTOR
A_24_P935881	7.5094757	7.696659	NM_022978	chr5:69321216-69321275	SERF1B
A_33_P3292218	10.036879	10.560606		chr5:096672618-096672561	
A_33_P3418000	3.4247403	2.955738	NM_001085399	chr4:37592482-37592423	RELL1
A_33_P3350634	8.582073	8.59982	NM_001256270	chr16:29815326-29815385	KIF22
A_33_P3410849	9.485204	9.930028	NM_001013842	chr8:22460863-22460922	C8orf58
A_23_P406025	3.5769901	2.3900566	NM_015225	chr9:79226442-79226383	PRUNE2
A_23_P121945	5.7847614	5.7469444	NM_001001502	chr5:176047719-176047660	SNCB
A_23_P59602	7.982261	8.110642	NM_019005	chr7:7635996-7636055	MIOS
A_24_P944040	8.335191	8.587709	NM_015056	chr21:45115366-45115425	RRP1B
A_23_P18372	8.503965	7.9300733	NM_032047	chr3:182990933-182990992	B3GNT5
A_23_P128084	5.9197063	6.1669655	NM_002206	chr12:56078479-56078420	ITGA7
A_33_P3262789	6.331165	6.5231276	NM_138393	chr19:1497862-1497921	REEP6
A_32_P205241	4.420999	4.375395	NM_021954	chr13:20712694-20712635	GJA3
A_33_P3309289	3.1535103	3.2415175	AK021929	chr11:126310189-126310248	ST3GAL4
A_23_P209625	6.896516	7.5210104	NM_000104	chr2:38295503-38295444	CYP1B1
A_23_P55136	7.0691614	6.917654	NM_016492	chr17:8192623-8192684	RANGRF
A_24_P367211	4.3308163	4.0305614	NM_014598	chr17:36552120-36552179	SOCS7
A_33_P3274304	8.155133	7.979789	NM_133264	chr17:38438199-38438258	WIPF2
A_33_P3344399	5.724732	5.15664	NM_001199018	chr15:71341786-71341845	LRRC49
A_33_P3268649	4.9153156	5.6368937	NM_001282864	chr22:24217368-24217427	SLC2A11
A_23_P15202	7.9817204	7.710749	NM_001361	chr16:72058288-72058347	DHODH
A_33_P3313704	5.776289	5.714176	XR_110629	chr7:1887050-1887109	LOC100128374
A_23_P153236	9.334599	9.584815	NM_014453	chr19:59063333-59063274	CHMP2A
A_33_P3382867	3.058825	2.3900566	NR_027420	chr21:9915274-9915250	LOC389834
A_23_P207336	5.507352	5.408266	NM_002722	chr17:42018512-42018263	PPY
A_24_P417596	8.216555	8.414668	NM_138439	chr16:2949287-2949346	FLYWCH2
A_33_P3376762	6.8991084	6.8481255	NR_027001	chr15:84873659-84873600	LOC388152

A_23_P83110	9.66034	9.660485	NM_018249	chr9:123156834-123152051	CDK5RAP2
A_33_P3286754	6.203182	6.0318623	NM_033382	chr22:30812904-30812963	SEC14L2
A_23_P91619	14.032981	14.074741	NM_002415	chr22:24237346-24237405	MIF
A_23_P212792	6.9506598	6.700929	NM_025009	chr4:56899111-56899170	CEP135
A_24_P79712	8.715676	9.062575	NM_198076	chr1:245007601-245007660	COX20
A_23_P27584	10.498568	10.690393	NM_001020818	chr19:54379561-54379620	MYADM
A_23_P420942	3.3788934	3.6651568	XM_005255956	chr16:56660614-56660673	MT1E
A_23_P47777	8.378871	8.630661	NM_138396	chr12:58153045-58153104	MARCH9
A_33_P3363425	4.6706514	3.3107214	NM_174938	chr9:85859651-85859592	FRMD3
A_33_P3229012	6.048882	6.3255253		chrUn_gl000225:000078890-000078949	
A_23_P44257	10.728892	10.851663	NM_017845	chr4:47453025-47452966	COMMD8
A_23_P349406	2.3221061	2.3900566	NM_173642	chr1:42880642-42880701	RIMKLA
A_33_P3338961	13.305304	13.254755		chr3:136454659-136454600	
A_23_P75380	8.711037	8.616576	NM_003977	chr11:67257891-67258280	AIP
A_23_P129425	4.611724	4.6902776	NM_018430	chr16:67861409-67861468	TSNAXIP1
A_33_P3382105	12.928442	12.899967		chr3:032305943-032306002	
A_23_P69586	10.111377	10.392603	NM_005245	chr4:187509580-187509521	FAT1
A_32_P29615	4.8721523	5.2281847	NM_001277120	chr19:53344799-53344740	ZNF468
A_24_P96593	6.5000267	5.9687257	NM_005665	chr1:92974495-92974436	EVI5
A_24_P205589	9.109415	9.110252	NM_007274	chr1:6354983-6354924	ACOT7
A_24_P876408	7.3814206	7.802153	NM_001144936	chr11:63527824-63527765	C11orf95
A_23_P76015	8.6009035	8.895346	NM_014786	chr11:73079944-73080003	ARHGEF17
A_33_P3313825	7.3522797	7.256115	AJ786388	chr3:30703057-30703116	TGFBR2
A_33_P3449397	10.431042	10.202089	NM_019852	chr14:21966376-21966317	METTL3
A_23_P18142	14.990484	14.58107	NM_001007074	chr3:12877621-12877562	RPL32
A_23_P131596	7.5482244	7.225475	NM_032319	chr2:73455396-73455337	PRADC1
A_23_P211136	5.720338	5.2892013	NM_018963	chr21:40558504-40558445	BRWD1
A_33_P3326423	8.268956	8.083288	NM_001174084	chr10:103338781-103338722	POLL
A_24_P204204	7.933044	7.7690716		chr14:100431463-100431524	
A_33_P3218148	4.9805007	4.8249464	NM_023073	chr5:37231117-37231058	C5orf42
A_33_P3405349	5.942989	6.143322	AK001787	chr7:99753492-99753433	C7orf43
A_33_P3250133	4.971819	4.8586864	NM_019086	chr12:118503705-118503646	VSIG10
A_33_P3236102	6.8449306	6.959997	NM_203434	chr9:131937927-131937868	IER5L
A_33_P3364869	6.6231937	6.7476616	AK023341	chr7:105900620-105900561	NAMPT
A_33_P3324894	5.9944687	6.071889	AK130532	chr12:123334593-123334652	
A_33_P3405474	5.6896124	5.7327466		chr9:16727463-16727522	
A_33_P3352349	8.434397	8.686721	NM_001190460	chr17:39346767-39346826	KRTAP9-1
A_23_P164341	5.446414	5.4338264	NM_014232	chr17:8062851-8062792	VAMP2
A_33_P3355743	6.4594173	6.696252	NM_018040	chr1:217784291-217784232	GPATCH2
A_33_P3376454	6.5430255	6.356994	AY358688	chr5:14342798-14342857	LOC100133299
A_23_P314712	3.7284498	2.3900566	NM_012189	chr18:21739982-21741487	CABYR
A_32_P9842	9.839824	9.895959	NM_001077526	chr3:9743997-9744056	MTMR14
A_24_P106591	8.228208	8.412128	NM_201280	chr6:8014278-8014219	BLOC1S5
A_32_P151875	7.874225	7.714252	NM_020781	chr7:148879766-148879825	ZNF398
A_23_P200685	7.3434234	7.4232044	NM_017898	chr1:220957294-220957353	MARC2
A_33_P3272160	11.119812	10.781036	NM_020385	chr9:136271247-136271188	REXO4
A_33_P3306085	3.6720004	4.260461	NM_001099218	chr2:17695356-17695297	RAD51AP2
A_24_P235266	8.73814	8.232075	NM_001001555	chr7:50657872-50657813	GRB10
A_23_P309224	6.0408564	5.999323	NM_018238	chr7:141353184-141353243	AGK
A_33_P3471712	16.37596	16.37596		chr1:850266-850325	LOC284600
A_23_P80643	7.48358	7.3771267	NM_006515	chr3:4358640-4358699	SETMAR
A_33_P3241269	4.789015	3.3979383	NM_001025195	chr16:55836823-55836764	CES1
A_23_P393531	4.3852086	4.269282	NM_004027	chr2:99185080-99189285	INPP4A
A_33_P3418170	7.119381	6.9734244	NM_014314	chr9:32455787-32455728	DDX58
A_33_P3314531	7.2174025	7.8782134		chr15:077570938-077570997	
A_33_P3294669	8.088572	8.00945		chr6:003694273-003694214	
A_23_P111995	11.416096	11.70069	NM_002318	chr8:23154845-23154786	LOXL2
A_23_P8906	9.595903	9.453505	NM_013437	chr8:105502522-105502463	LRP12

A_32_P524014	10.340813	10.43513	NM_007124	chr6:145173938-145173997	UTRN
A_33_P3242355	7.008299	7.143154	BC027847	chr10:135137970-135137911	
A_33_P3318911	7.6176443	7.4536934	NM_001135642	chr17:1397962-1397903	INPP5K
A_23_P26704	7.187898	6.844386	NM_024311	chr17:74774485-74774544	MFSD11
A_32_P113508	8.191027	8.240465	NM_030803	chr2:234203900-234203959	ATG16L1
A_23_P390116	7.2925625	7.1789603	NM_153023	chr13:24877575-24877634	SPATA13
A_33_P3421978	4.2494764	4.294701	NM_001080516	chr5:145246194-145246135	GRXCR2
A_24_P212860	4.7640047	5.315427	NM_016481	chr9:100675688-100672845	C9orf156
A_23_P11652	10.893549	10.647438	NM_003368	chr1:62917270-62917329	USP1
A_24_P319364	9.865618	9.889473	NM_016946	chr1:160966092-160966033	F11R
A_23_P40049	8.632567	8.433338	NM_004341	chr2:27465770-27465829	CAD
A_23_P146084	8.1264925	7.706561	NM_000637	chr8:30537028-30536969	GSR
A_33_P3242388	8.64519	7.95766	NM_001166304	chr3:196462796-196462855	PIGX
A_23_P154058	6.6570716	5.824388	NM_001034116	chr2:27589964-27589768	EIF2B4
A_23_P215832	10.936023	10.574993	NM_004889	chr7:99057727-99056846	ATP5J2
A_33_P3330503	6.5861707	6.045348	NM_001182	chr5:125879077-125879018	ALDH7A1
A_33_P3211924	9.056101	8.942505	NM_015156	chr14:103196738-103196797	RCOR1
A_23_P204436	2.683886	3.5166879	NM_139201	chr12:110386508-110386449	GIT2
A_24_P914940	6.400327	6.410998	NM_001145784	chr19:19288116-19288057	MEF2BNB
A_24_P320328	10.106107	10.208358	NM_006713	chr5:32591694-32591753	SUB1
A_23_P318581	9.168171	8.945455	NM_020827	chr4:186081462-186081403	KIAA1430
A_33_P3410892	5.1413255	5.277042		chr19:198082-198141	
A_24_P130962	4.934128	5.095264	NM_022371	chr1:179063261-179063320	TOR3A
A_23_P381461	9.496876	9.311007	NM_144999	chr17:79988608-79988667	LRRC45
A_23_P143047	7.06697	6.153282	NM_080653	chr2:46739193-46739134	ATP6V1E2
A_24_P170395	7.246896	7.1136684		chrX:149284149-149284208	
A_33_P3261565	5.5849223	5.5372386	NM_017580	chr10:126675946-126676005	ZRANB1
A_33_P3373298	8.16066	7.942092	NR_040585	chr7:66785323-66785382	STAG3L4
A_32_P107746	6.979984	6.9939566	NM_207042	chr1:150594599-150594586	ENSA
A_23_P71368	2.3221061	2.3900566	NM_006269	chr8:55542995-55543054	RP1
A_23_P129133	2.3221061	2.3900566	NM_000275	chr15:28000126-28000067	OCA2
A_23_P151337	9.485647	9.353698	NR_002605	chr13:50679272-50679331	DLEU1
A_24_P153763	4.0288296	3.828104	XR_250407	chr8:125205353-125205294	
A_33_P3233947	11.828948	12.129554	NM_198076	chr1:245006469-245006528	COX20
A_23_P214144	5.5563173	5.4558	NM_000493	chr6:116441120-116441061	COL10A1
A_33_P3338047	5.5379806	5.0875816	NM_001098537	chr9:140437218-140437159	PNPLA7
A_33_P3229067	6.754261	7.1096416	NM_003520	chr6:27806454-27806513	HIST1H2BN
A_23_P121447	2.3221061	2.9430876	NM_032487	chr3:169485353-169485294	ACTRT3
A_23_P113005	7.6299815	7.5559635	NM_004428	chr1:155106767-155106826	EFNA1
A_24_P291973	9.100807	9.206402	NM_001011553	chr7:35913298-35913357	SEPT7
A_33_P3277097	6.073313	6.1869287	NR_003673	chr6:31680333-31680274	LY6G6E
A_32_P73821	12.433278	12.577812	NM_001007553	chr1:115259551-115259517	CSDE1
A_24_P58242	11.375101	11.5372305		chr8:069438016-069437957	
A_33_P3265030	14.790661	15.191169	NM_000407	chr22:19712238-19712297	GP1BB
A_33_P3235335	6.207474	6.50097	NM_021090	chr22:30421915-30421974	MTMR3
A_23_P208516	9.486972	9.581781	NM_024298	chr19:54677445-54677386	MBOAT7
A_23_P128543	10.056347	10.232068	NM_014166	chr13:48650836-48650777	MED4
A_32_P190151	4.5257616	4.2934117	NR_024241	chr3:75471879-75471820	FAM86DP
A_33_P3813684	9.796847	9.682794		chr1:95944853-95944912	FLJ31662
A_33_P3397506	4.1939964	3.8482833		chr17:058178846-058178787	
A_23_P104116	12.603408	12.615626	NM_005105	chr1:145509245-145509304	RBM8A
A_33_P3250383	6.327021	6.0861936	NM_033133	chr17:40129673-40129732	CNP
A_23_P132956	13.781273	13.758507	NM_004181	chr4:41270340-41270399	UCHL1
A_23_P151746	9.466926	9.619779	NM_194279	chr14:74961983-74962042	ISCA2
A_33_P3212570	4.070278	4.0049853	NR_102400	chr16:031214716-031214775	C16orf98
A_33_P3337009	4.524761	4.2530036		chr1:42923068-42923127	PPCS
A_23_P130158	7.846579	7.012703	NM_030753	chr17:44841763-44841704	WNT3
A_32_P34552	6.221509	5.8288107	NM_002690	chr8:42210075-42213081	POLB
A_33_P3250268	5.2343316	5.2182875		chr15:29087527-29087468	XLOC_014512

A_23_P360245	9.961436	10.102524	NM_145266	chr5:162880784-162880725	NUDCD2
A_23_P165624	3.9578185	4.502428	NM_007115	chr2:152226595-152226654	TNFAIP6
A_23_P256148	7.1980104	7.4187365	NM_024595	chr1:39469807-39469866	AKIRIN1
A_24_P87114	4.6148696	5.0793176	NM_032663	chr12:109520778-109520837	USP30
A_33_P3503279	6.2380342	6.5199304			
A_33_P3387861	10.852718	11.270128	NM_001100624	chr16:81065030-81065089	CENPN
A_33_P3343428	6.4014664	6.4600964	NM_147686	chr6:111880566-111880507	TRAF3IP2
A_23_P37870	10.816252	10.646789	NM_005861	chr16:732419-732478	STUB1
A_33_P3388312	8.831744	8.500758	NM_003930	chr7:26706783-26706724	SKAP2
A_24_P929322	7.229307	7.3509283	NR_024413	chr3:187898304-187898363	FLJ42393
A_23_P135730	6.7861457	7.4071918	NM_145295	chr19:11729303-11729362	ZNF627
A_23_P16992	6.699718	6.9218397	NM_003628	chr2:159537233-159537292	PKP4
A_33_P3325634	6.802895	7.000963	NM_001130514	chr19:41950070-41950129	C19orf69
A_33_P3224680	6.5915704	6.3660126	AK289404	chr1:100606037-100606096	TRMT13
A_33_P3369079	5.9291806	5.684507		chr22:51022297-51022356	CHKB-AS1
A_33_P3263423	5.4773035	5.7434874	NM_003268	chr1:223305886-223305827	TLR5
A_23_P38457	4.882966	5.4093876	NM_020791	chr17:27871269-27871328	TAOK1
A_23_P61633	8.071796	8.161596	NM_005781	chr3:195590540-195590481	TNK2
A_33_P3234697	9.540901	9.797848	NM_020169	chr3:158384375-158384316	LXN
A_24_P297537	4.473855	4.29975	NM_182574	chr19:49218116-49217812	MAMSTR
A_23_P321501	2.3221061	2.3900566	NM_182908	chr14:24114500-24114560	DHRS2
A_23_P73780	9.155772	8.974194	NM_001569	chrX:153276900-153276841	IRAK1
A_23_P110005	6.4246907	6.607434	NM_032316	chr3:49460649-49460590	NICN1
A_23_P2431	6.033798	5.962516	NM_004054	chr12:8211407-8211348	C3AR1
A_24_P557479	2.3221061	2.3900566	NM_017523	chr17:6678819-6678878	XAF1
A_23_P116037	2.8550105	3.03792	NM_003273	chr11:64880991-64881050	TM7SF2
A_23_P135616	8.087241	7.982395	NM_016930	chr4:4421658-4421599	STX18
A_23_P382199	10.518948	10.641827	NM_003574	chr18:9954334-9954393	VAPA
A_23_P82296	5.953005	6.002944	NM_005273	chr7:100275726-100275785	GNB2
A_23_P106002	9.433028	9.46238	NM_020529	chr14:35870847-35870788	NFKBIA
A_23_P408955	8.298032	8.185893	NM_004091	chr1:23832992-23832933	E2F2
A_33_P3316505	5.581339	5.5453606	NR_002907	chr1:28834024-28834083	SNORA73A
A_23_P215024	7.3146825	7.224336	NM_018214	chr6:53788424-53788483	LRRC1
A_33_P3324687	9.841906	10.823772		chr15:036911228-036911287	
A_23_P255968	6.329952	6.6826286	NM_003967	chr6:132910215-132910156	TAAR5
A_33_P3369834	4.934907	5.1257524	NM_138393	chr19:1497438-1497497	REEP6
A_33_P3211929	5.052946	6.3702974	NM_173587	chr11:63678786-63678727	RCOR2
A_23_P66719	7.433317	7.8488703	NM_144683	chr17:27225108-27225049	DHRS13
A_33_P3474328	4.943586	5.103961	NM_021959	chr6:30037099-30037158	PPP1R11
A_33_P3308115	12.514994	12.500863	BC029410	chr7:56804200-56804141	
A_23_P98763	4.2467976	4.714142	NM_145309	chr11:71804624-71804683	LRTOMT
A_24_P192988	3.7746947	3.647499	NM_152723	chr11:85396304-85396245	CCDC89
A_33_P3317942	4.302544	3.6287117		chr4:099877351-099877410	
A_24_P143076	5.350876	5.3092794	NM_144671	chr12:111799200-111799141	FAM109A
A_24_P109432	6.5975466	6.410093	NM_001114132	chr2:203914691-203921201	NBEAL1
A_23_P141656	7.3346786	6.9194193	NM_001388	chr17:18011046-18011105	DRG2
A_33_P3311076	9.740978	9.852472	NM_001190807	chr18:71930657-71930598	CYB5A
A_23_P346048	9.273784	9.631545	NR_002824	chr15:23282829-23282770	HERC2P2
A_23_P163161	7.63547	7.623539	NM_020195	chr14:24909270-24909211	SDR39U1
A_23_P72117	5.478556	5.315325	NM_006714	chr6:123130605-123130664	SMPDL3A
A_33_P3317198	10.221976	9.8550205	NM_004128	chr13:45858028-45858087	GTF2F2
A_33_P3306973	5.54313	5.2195034		chr12:007590340-007590281	
A_33_P3257513	9.107715	9.617315	NM_001008781	chr11:92629072-92629131	FAT3
A_33_P3271316	5.593428	5.5505266	NM_017793	chr15:75247515-75247456	RPP25
A_24_P402836	7.351213	7.790676	NM_003441	chr4:367324-367383	ZNF141
A_33_P3243494	4.0400977	3.0460608		chr16:51069088-51069029	
A_33_P3259620	10.599971	10.458079	NM_014639	chr5:94800285-94800226	TTC37
A_33_P3310780	12.820715	12.627634	NM_005231	chr11:70282582-70282641	CTTN
A_23_P409541	6.928114	6.889866	NM_152705	chr13:28240914-28240973	POLR1D

A_33_P3226565	4.60716	4.5707297		chr10:093669067-093669126	
A_24_P1054	5.5481033	5.724732	NM_013432	chr8:145663855-145662425	TONSL
A_24_P921321	10.086915	10.656244	NM_002843	chr11:48192048-48192107	PTPRJ
A_23_P165061	11.665095	11.60569	NM_198969	chr19:3053028-3052969	AES
A_33_P3254670	3.8423502	4.5017076		chr1:38569273-38569332	
A_23_P15146	5.5878344	5.394764	NM_001012631	chr16:3119308-3119367	IL32
A_23_P140760	4.539672	4.753558	NM_170776	chr16:57719826-57722310	GPR97
A_24_P3005	3.7877889	4.529186	NM_002977	chr2:167055617-167055558	SCN9A
A_33_P3233700	4.5320187	4.44946	NR_024561	chr19:35566138-35566079	HPN-AS1
A_33_P3389827	5.874655	5.8416796	NM_001165978	chr2:95956002-95956061	PROM2
A_23_P38041	8.572544	8.894363	NM_016256	chr16:5075069-5075010	NAGPA
A_23_P87742	5.021414	4.7274194	NM_001039670	chr12:6648906-6648847	IFFO1
A_33_P3421325	3.5624413	4.167107	NM_001001662	chr9:99607232-99607173	ZNF782
A_33_P3243907	8.291226	8.252829	NM_001909	chr11:1774484-1774425	CTSD
A_23_P69908	10.757578	10.540964	NM_002064	chr5:95149862-95149803	GLRX
A_33_P3312549	5.5531225	5.317341		chrY:026335550-026335491	
A_33_P3301782	4.6750994	4.9569573	NR_024284	chr10:31605790-31605731	ZEB1-AS1
A_23_P43226	10.291437	10.158388	NM_017634	chr8:25285893-25285834	KCTD9
A_33_P3212092	7.160764	7.1399984	NM_145341	chr10:112659602-112659661	PDCD4
A_23_P83931	10.247489	9.910976	NM_001047160	chr10:5500236-5500295	NET1
A_23_P108673	9.007405	8.912196	NM_032181	chr2:75719826-75719767	EVA1A
A_23_P301360	5.174518	4.5076046	NM_152412	chr8:125991516-125991575	ZNF572
A_33_P3256510	3.6574252	3.9017446	NM_022055	chr2:47747995-47747936	KCNK12
A_23_P126605	3.9720635	3.7977507	NM_013353	chr1:151143021-151142547	TMOD4
A_33_P3379199	8.849824	8.901769	NM_013387	chr22:30163478-30163537	UQCR10
A_33_P3404126	7.0284967	7.045635	XM_005272255	chr4:728154-728213	PCGF3
A_33_P3224090	3.9773042	3.2230756	XR_243240	chr15:94304343-94304284	
A_33_P3234859	4.756803	5.0127487	NM_007124	chr6:144795835-144795894	UTRN
A_24_P218970	8.93746	9.259395	NM_001417	chr12:53435296-53435355	EIF4B
A_23_P12849	7.3352823	7.6299815	NM_178150	chr10:5966356-5966415	FBXO18
A_33_P3383606	7.543785	7.677295	NM_001135914	chr7:128516990-128516931	KCP
A_23_P133338	4.654356	4.2289557	NM_017675	chr5:176017621-176017680	CDHR2
A_24_P383850	5.2588563	5.162595	NM_003760	chr1:21268056-21267997	EIF4G3
A_23_P48198	5.8970094	6.0417585	NM_031302	chr12:104383204-104383145	GLT8D2
A_33_P3310548	4.5049996	4.770904	AY358263	chr2:102737796-102737855	LOC100131131
A_23_P44932	9.512272	9.624956	NM_032025	chr3:150293519-150299433	EIF2A
A_23_P68628	5.6449814	5.399818	NM_031232	chr20:32245523-32245464	NECAB3
A_23_P405942	7.894574	7.7807646	NM_015155	chr10:855899-855840	LARP4B
A_33_P3362891	9.057674	8.592282	NM_002137	chr7:26240281-26240222	HNRNPA2B1
A_33_P3227934	5.824589	5.899844	AK126997	chr16:1358740-1358681	LOC100130430
A_23_P111273	7.5091405	7.359719	NM_016495	chr6:13307878-13306747	TBC1D7
A_32_P43812	8.404892	7.9868984	NM_001040402	chr4:52782383-52782442	DCUN1D4
A_23_P50674	3.8457837	3.9676802	NM_001031735	chr19:2097434-2097493	IZUMO4
A_33_P3403117	8.637417	9.264941	NM_005654	chr5:92929717-92929776	NR2F1
A_23_P424878	5.975012	5.892473	NM_025251	chr8:145755108-145755049	ARHGAP39
A_23_P46539	9.769011	10.014084	NM_032636	chr1:109822518-109822459	PSRC1
A_24_P252130	7.28298	7.0884132	NM_006238	chr6:35395809-35395868	PPARD
A_23_P11295	10.053511	10.086093	NM_001018024	chrX:154290221-154290162	CMC4
A_33_P3404588	4.1133003	4.0861855	NM_139241	chr12:32764149-32764208	FGD4
A_33_P3399768	5.1293306	5.6081324	NR_034126	chr1:112141688-112141629	LOC100129269
A_24_P633902	7.318993	7.373562	NM_014455	chr1:145663334-145682047	RNF115
A_33_P3383189	4.308523	4.307011	NM_001145250	chr2:175202002-175202061	SP9
A_23_P126031	3.9941556	4.053121	NM_001004469	chr1:159505557-159505498	OR10J5
A_33_P3382303	3.9015274	3.823917	NM_005892	chr17:43322187-43322246	FMNL1
A_23_P43504	5.260546	5.0944314	NM_001606	chr9:139902359-139902300	ABCA2
A_33_P3289391	7.373562	7.471079		chr3:033155587-033155528	
A_32_P88310	6.219974	6.003479	NM_001256932	chr16:30709143-30709084	LOC730183
A_23_P56938	6.4844847	6.4650373	NM_002908	chr2:61149596-61149655	REL
A_33_P3399363	4.4527254	3.9893937	NM_001033045	chr2:175299107-175299048	GPR155

A_33_P3257222	8.086	7.5543447	NM_144589	chr10:76993805-76993746	COMTD1
A_23_P256172	11.7751875	11.985635	NM_199069	chr3:49060618-49060677	NDUFAF3
A_33_P3255766	7.456062	7.273315	NM_001142356	chr2:201758042-201758101	NIF3L1
A_33_P3273272	4.2031813	3.2786803	NM_001172655	chr19:53085632-53085691	ZNF701
A_24_P95822	7.1736164	7.7494016	NM_012428	chr15:73866013-73862716	NPTN
A_33_P3271455	10.719801	11.28883	NM_012293	chr2:1635749-1635690	PXDN
A_24_P255303	10.697929	10.803867		chr1:211615949-211616010	
A_33_P3278931	4.621416	4.4726524	XM_003403711	chr19:56758795-56758854	ZSCAN5D
A_33_P3251538	7.9676075	8.011551	NM_001006618	chr9:128305398-128305339	MAPKAP1
A_23_P147665	3.9956195	4.217281	NM_198474	chr11:7531816-7531875	OLFML1
A_24_P6030	5.4016685	4.386292	BC045820	chr21:35774897-35774956	SMIM11
A_24_P53353	6.098605	5.9085836	NM_001025300	chr18:8635551-8635610	RAB12
A_24_P164894	5.1163263	5.200063	NM_006052	chr21:38610861-38610802	DSCR3
A_23_P72138	11.440688	11.375101	NM_020191	chr3:139075773-139075832	MRPS22
A_23_P34741	10.158388	9.903686	NM_015871	chr1:26497166-26497225	ZNF593
A_33_P3286208	4.0498366	3.9451604	NM_203467	chr14:50065700-50065759	LRR1
A_23_P89550	5.3312078	5.129622	NM_033004	chr17:5418168-5418109	NLRP1
A_24_P923102	5.7909365	5.2516313	NM_001199290	chr2:170608203-170608262	PHOSPHO2-KLHL23
A_24_P5550	4.334748	4.6988163	NM_024297	chr17:7139861-7139802	PHF23
A_23_P205746	7.7893987	7.911289	NM_001008707	chr14:100407492-100407551	EML1
A_33_P3296682	11.244692	11.368754	NM_002492	chr3:179342191-179342250	NDUFB5
A_33_P3567512	6.728293	6.5038238	BC125059	chr13:32526757-32526816	
A_33_P3264524	3.2169561	3.23983		chrY:025012909-025012850	
A_23_P213375	7.16426	6.754261	NM_018936	chr5:140476755-140476814	PCDHB2
A_33_P3318661	4.0139084	3.5022333	NM_203394	chr12:77417635-77417576	E2F7
A_23_P14083	10.30715	10.000765	NM_181847	chr12:47470990-47470931	AMIGO2
A_24_P88800	8.996651	9.074101	NM_004718	chr2:42580432-42580373	COX7A2L
A_23_P39386	5.268415	5.400199	NM_014266	chr19:36395060-36395119	HCST
A_33_P3239267	13.296448	13.376355	NM_203370	chr3:49842402-49842461	FAM212A
A_33_P3272291	5.8766994	6.131588	NM_001818	chr10:5246396-5246455	AKR1C4
A_33_P3289848	6.344099	6.5817933	NM_001804	chr5:149563184-149563243	CDX1
A_23_P146077	9.182727	9.24774	NM_018660	chr8:28203450-28203391	ZNF395
A_23_P406105	11.1594	11.11167	NM_006836	chr12:120565144-120565085	GCN1L1
A_24_P280833	6.9383564	6.9540567		chr1:104616336-104616395	LOC100129138
A_23_P91283	4.2788625	3.9988756	NM_020356	chr20:55028074-55028133	CASS4
A_33_P3244224	4.245359	3.764617	AK128074	chr1:7447714-7447655	LOC100129048
A_23_P135778	8.595508	8.764399	NM_018461	chr10:133769421-133769480	PPP2R2D
A_23_P48628	6.0467944	6.3015623	NM_006977	chr14:64953889-64953830	ZBTB25
A_33_P3296352	6.3959603	7.229307	NM_002706	chr2:44445135-44445194	PPM1B
A_23_P39840	4.098418	3.3727272	NM_006634	chr2:85818953-85820097	VAMP5
A_32_P93328	6.042391	6.263526	NR_024454	chr7:140396338-140396279	NDUFB2-AS1
A_32_P134290	6.8685455	6.9870863	NM_017742	chr18:60245525-60245584	ZCCHC2
A_23_P75786	2.3221061	2.3900566	NM_016582	chr11:60705358-60704803	SLC15A3
A_32_P216872	5.7324815	5.704751	BX647358	chr16:70010702-70010643	PDXDC2P
A_23_P9061	13.633335	13.513447	NM_003756	chr8:117657150-117657091	EIF3H
A_24_P171983	9.582101	9.028243	NM_014165	chr6:97339244-97339185	NDUFAF4
A_33_P3277110	8.8864975	8.264049	NM_006933	chr21:35478440-35478499	SLC5A3
A_23_P86801	4.586767	4.833164	NM_032645	chr11:47464364-47464305	RAPSN
A_23_P118722	5.09053	4.6641464	NM_001671	chr17:7076840-7076781	ASGR1
A_23_P114774	9.928231	10.076309	NM_006556	chr1:154897457-154897398	PMVK
A_23_P55948	9.022004	8.837372	NM_020719	chr19:50129602-50129661	PRR12
A_33_P3403356	7.292017	7.529522		chr10:051620362-051620303	
A_24_P314554	6.06447	6.103551	NR_001555	chrY:27603553-27604968	GOLGA2P2Y
A_24_P406334	6.2141004	6.087932	NM_012449	chr7:89790531-89790590	STEAP1
A_23_P88381	8.520388	9.016097	NM_001005743	chr14:73742568-73742509	NUMB
A_33_P3279730	5.0146437	4.3342905	XM_005261384	chr22:24310354-24310413	DDT
A_23_P50799	6.6156826	6.7766104	NM_013939	chr19:15839700-15839759	OR10H2
A_32_P16854	8.528401	8.602742	NM_015391	chr3:134201667-134197518	ANAPC13

A_23_P29663	4.475304	4.268749	NM_015896	chr3:50378905-50378846	ZMYND10
A_23_P152949	5.4593124	5.362596	NM_033413	chr17:45914424-45914483	LRRC46
A_24_P280029	4.977394	4.8423223	NM_020315	chr22:38062262-38062321	PDXP
A_23_P430902	4.9055386	4.9051003	NM_178832	chr10:99374762-99374703	MORN4
A_33_P3373165	4.714538	4.5892935	NM_001177376	chr4:190992732-190992791	DUX4L4
A_33_P3232504	4.96278	4.688545	NM_199001	chr9:140120698-140120757	C9orf169
A_33_P3313796	7.9445453	7.7665563	NM_030771	chr11:27360176-27360117	CCDC34
A_23_P64404	4.645653	4.45704	NM_021727	chr11:61645033-61644427	FADS3
A_33_P3381458	3.5733461	3.497325	AK094155	chr1:1194777-1194718	
A_33_P3338724	6.8371134	6.7897096	NM_198988	chr19:54973035-54972976	LENG9
A_33_P3402635	5.105775	5.7808294	NM_001214	chr16:90095456-90095397	C16orf3
A_24_P282762	5.284636	5.328771	NM_012290	chr2:171863068-171863009	TLK1
A_33_P3281728	3.944707	4.1127496		chr1:149287210-149287151	
A_23_P90845	8.660514	8.954823	NM_022173	chr2:70439815-70439756	TIA1
A_33_P3304819	5.443965	5.6116652		chr7:105258244-105258303	
A_23_P150903	3.5991716	3.296414	NM_018099	chr12:29485623-29486586	FAR2
A_23_P77310	8.402874	8.596495	NM_022473	chr15:42705253-42705194	ZNF106
A_23_P305205	5.626084	6.0214543	NM_015314	chr7:36364662-36364603	KIAA0895
A_23_P62901	7.457054	8.275514	NM_006763	chr1:203278454-203278513	BTG2
A_24_P418809	12.746756	13.002138	NM_001077489	chr20:57485083-57485394	GNAS
A_33_P3832852	6.85849	6.680068	NM_018097	chr15:42860987-42861046	HAUS2
A_23_P89710	8.76256	8.398947	NM_032142	chr18:13124695-13124754	CEP192
A_33_P3398156	7.502299	7.2666388	NM_001037160	chr2:10197031-10196972	CYS1
A_23_P144202	3.6036024	3.513947	NM_021937	chr3:128060330-128060389	EEFSEC
A_33_P3258747	4.347986	4.523347	NM_001286556	chr21:33785261-33785320	EVA1C
A_23_P93629	9.412672	9.59147	NM_015905	chr7:138270224-138270283	TRIM24
A_33_P3405763	5.6301746	5.332139	NM_001039770	chr3:33131976-33131917	TMPPE
A_23_P21230	7.2374043	7.2323685	NM_015294	chr17:57076176-57076117	TRIM37
A_33_P3263193	9.587885	9.37877	NM_001257097	chr12:2998466-2998525	RHNO1
A_24_P363745	9.519755	9.2263975	NM_014665	chr8:145750486-145750545	LRRC14
A_24_P49447	6.923565	6.9586844	NM_020829	chr9:5775976-5776035	KIAA1432
A_32_P64475	6.9630666	6.84852	NM_152608	chr1:226172967-226172908	SDE2
A_23_P341065	6.000111	5.7252607	NM_031454	chr22:50655467-50655526	SELO
A_33_P3239148	5.161545	5.196819	NM_001172773	chr19:57912622-57912681	ZNF548
A_33_P3259560	5.376808	5.6343284		chr7:090121653-090121712	
A_24_P462853	4.0400167	3.0340014	NR_026795	chr10:27224382-27223415	LINC00202-1
A_33_P3411325	8.522992	8.490051		chr16:032252763-032252704	
A_33_P3260572	7.162698	7.466359	NM_024692	chr2:29386760-29386819	CLIP4
A_23_P83436	10.995993	11.072306	NM_000285	chr19:33877995-33877936	PEPD
A_33_P3798739	5.8697453	6.065276	AK094356	chr9:73674019-73673960	LOC286382
A_23_P156061	5.948556	6.4989552	NM_005575	chr5:96362390-96362449	LNPEP
A_24_P7629	3.7197669	3.644261		chr3:129102815-129102756	RPL32P3
A_33_P3234571	5.611101	5.4913054	NM_145109	chr17:21205459-21205518	MAP2K3
A_23_P256470	2.3221061	2.3900566	NM_000905	chr7:24331284-24331343	NPY
A_33_P3276142	3.7758489	4.1579337	NR_003260	chr15:100340038-100339979	DNM1P46
A_33_P3374117	4.146751	3.6768656	XR_241210	chr1:16543682-16543623	
A_23_P147383	6.103551	6.118986	NM_003801	chr8:145139458-145139629	GPAA1
A_33_P3234487	10.918064	10.8495	NM_001039182	chr16:30204739-30204680	BOLA2B
A_23_P144656	3.831904	4.186547	NM_006727	chr5:24487499-24487440	CDH10
A_33_P3212555	4.2089643	5.1639233	NM_000313	chr3:090251481-090251422	PROS1
A_33_P3304382	6.5735207	6.2409782	NM_152416	chr8:96070824-96070883	NDUFAF6
A_33_P3364696	7.717897	7.9383764	NM_213622	chr2:74089372-74089431	STAMBP
A_23_P418597	5.9379463	6.0543213	NM_033396	chr11:57068385-57068043	TNKS1BP1
A_33_P3391387	4.8225546	4.7981105	NR_026682	chr5:172382521-172382462	LOC100268168
A_23_P41599	2.6757104	2.3900566	NM_019120	chr5:140559850-140559909	PCDHB8
A_24_P15292	7.196001	6.8911753	NM_032018	chr1:231487123-231487182	SPRTN
A_33_P3243897	7.9633856	7.874225	NM_024813	chr1:92853630-92853689	RPAP2
A_23_P147388	8.782466	8.694257	NM_015254	chr8:28924878-28924819	KIF13B
A_33_P3492750	6.5469017	6.4552407		chr1:78758896-78758955	MGC27382

A_33_P3454968	9.768507	9.57275	XR_244001	chr19:46692429-46692370	LOC645553
A_23_P82959	4.5894675	4.399301	NM_003923	chr8:145699649-145699590	FOXH1
A_23_P99693	7.4282966	7.1502852	NM_014950	chr14:65000010-65000069	ZBTB1
A_23_P215491	11.870502	12.416422	NM_002991	chr7:75442741-75442682	CCL24
A_33_P3330079	6.171033	5.3729157	XR_110532	chr11:123306328-123306387	LOC100128242
A_33_P3666817	5.5993214	5.399063	NM_001001791	chr10:75670098-75670039	C10orf55
A_23_P19084	8.533153	8.7485285	NM_004499	chr5:177634122-177634181	HNRNPAB
A_23_P337934	8.977723	8.849824	NM_017556	chr1:16112685-16112744	FBLIM1
A_33_P3288039	5.1945496	4.870519	NM_198531	chr18:77119376-77119435	ATP9B
A_33_P3389230	5.6941648	5.888665	NM_001142797	chrX:70836916-70836857	CXCR3
A_32_P16258	4.9068217	5.311756	XM_005264223	chr2:72403823-72403764	EXOC6B
A_32_P412313	4.619222	4.187986	NM_017873	chr9:132397750-132397691	ASB6
A_33_P3335596	4.338861	4.0468583	NM_001193653	chr17:80401579-80401520	C17orf62
A_33_P3315899	8.220129	7.952551		chr20:019193130-019193071	
A_23_P16063	4.805685	5.0970435	NM_002088	chr19:42507588-42507529	GRIK5
A_23_P8452	10.591174	10.340965	NM_001040167	chr7:2567971-2568030	LFNG
A_33_P3295838	5.741748	5.7717953	NM_001171251	chr17:42225966-42226025	C17orf53
A_33_P3331080	3.6143503	4.2248807	NM_001252231	chr5:134028724-134028783	SEC24A
A_23_P119916	4.291148	4.3515806	NM_006522	chr2:219738852-219738911	WNT6
A_32_P178966	5.1331124	5.2483006	NM_001100829	chr6:11583573-11583632	TMEM170B
A_33_P3272140	8.421174	8.343532	NM_138285	chr2:184026087-184026146	NUP35
A_33_P3223958	4.8169045	6.0639906	NM_016347	chr2:73927999-73927940	NAT8B
A_23_P368718	6.3814273	6.050591	NM_020456	chr13:50486991-50486932	SPRYD7
A_24_P328969	8.839419	8.233047	NM_030799	chr5:143538082-143538023	YIPF5
A_23_P141651	5.090745	5.1802063	NM_006311	chr17:15942883-15942824	NCOR1
A_23_P214168	9.069736	8.815406	NM_004370	chr6:75794348-75794289	COL12A1
A_33_P3233135	3.5448117	3.252514	NM_001199866	chr20:16347850-16347791	KIF16B
A_33_P3342345	4.2482047	4.517539		chr15:100348110-100348169	XLOC_014512
A_23_P99186	5.581539	5.717761	NM_183415	chr12:109974008-109974067	UBE3B
A_33_P3277965	4.9739523	4.8090596	NM_205843	chr19:3463424-3463483	NFIC
A_23_P352870	5.2409906	4.7469163	NM_002856	chr19:45377626-45377685	PVRL2
A_23_P156319	12.096716	11.647006	NM_015315	chr5:154196833-154196892	LARP1
A_23_P124733	6.8294153	7.1846337	NM_015697	chr4:84193253-84191101	COQ2
A_33_P3332627	7.143154	7.3670654		chr7:057245525-057245466	
A_32_P34589	8.246457	8.186765	NM_016625	chr3:158261998-158262057	RSRC1
A_32_P116840	8.293711	8.041875	NM_203356	chr14:39819450-39819509	CTAGE5
A_23_P321201	9.050679	8.62449	NM_015213	chr11:9160649-9160590	DENND5A
A_32_P64200	4.27617	3.7258053	NM_002098	chr6:42151260-42151201	GUCA1B
A_23_P69141	7.716153	7.9480743	NM_016141	chr3:32567461-32567450	DYNC1LI1
A_33_P3380850	4.0854425	4.5301065	NM_003990	chr10:102568921-102568980	PAX2
A_23_P50897	8.404302	8.191435	NM_032390	chr2:122484915-122484856	NIFK
A_33_P3409904	5.364086	5.3309894	NM_003762	chr1:171672343-171672284	VAMP4
A_33_P3253440	5.3687115	5.991813	NM_152391	chr2:11318091-11318150	PQLC3
A_33_P3702281	11.889375	11.878414	AK095567	chr17:4690387-4690328	LOC284014
A_23_P69791	7.0897985	6.8335705	NM_018569	chr4:113190479-113190538	AP1AR
A_23_P112173	3.6801577	3.136942	NM_020344	chr9:19550140-19528083	SLC24A2
A_33_P3217356	3.9758537	3.9384403		chr1:48260318-48260259	TRABD2B
A_23_P213093	10.392106	10.756537	NM_001079839	chr4:48862818-48862877	OCIAD1
A_23_P202988	8.6277275	7.697406	NM_015423	chr11:105968976-105969035	AASDHPPT
A_32_P389118	7.4530363	7.6381545	NM_019024	chr2:37208378-37208319	HEATR5B
A_24_P146892	8.490051	8.345644	NM_032790	chr12:122079698-122079757	ORAI1
A_32_P205624	6.6956124	6.766621	NM_012435	chr19:416892-416833	SHC2
A_33_P3334895	5.0970435	4.754453	NM_001134408	chr16:9857277-9857218	GRIN2A
A_33_P3291567	6.3116655	6.384362	NM_145003	chr8:143310898-143310839	TSNARE1
A_33_P3347049	4.1812854	4.231759		chr5:099728171-099728112	
A_23_P43779	10.421682	10.411342	NM_016129	chr4:83996680-83996739	COPS4
A_23_P209426	7.2884007	7.0159397	NM_015049	chr2:202242412-202242353	TRAK2
A_23_P47857	10.1094	10.0250435	NM_016551	chr12:27126799-27126740	TM7SF3
A_33_P3388870	8.469552	8.560639	NM_001487	chr12:56113416-56113475	BLOC1S1

A_24_P693321	7.415049	7.2714305	NR_024456	chr16:21876568-21876509	LOC100190986
A_33_P3224345	3.76602	3.4556336		chr15:031130005-031130064	
A_23_P215419	4.8787575	4.881018	NM_004968	chr7:8153625-8153566	ICA1
A_23_P211056	6.312649	6.7155766	NM_013329	chr21:34117925-34117206	PAXBP1
A_33_P3349947	10.179716	9.9033785	NM_030752	chr6:160199617-160199558	TCP1
A_33_P3382538	2.6491604	3.6247807	NM_003772	chr11:96125502-96125561	JRKL
A_33_P3407324	8.778071	9.399793	NM_001252668	chr2:189458677-189458736	GULP1
A_33_P3360867	5.7426653	6.06447		chr7:066367818-066367877	
A_33_P3419339	8.261865	7.9642587	NM_006496	chr1:110138366-110138425	GNAI3
A_33_P3216008	8.995847	8.599561	NM_145061	chr13:21727794-21727735	SKA3
A_24_P311771	7.6349745	7.7605305	NM_016107	chr5:32385686-32385627	ZFR
A_32_P46191	11.841438	12.088271	NM_001159522	chr7:63538596-63538655	ZNF727
A_33_P3314974	4.318446	4.691977	NR_028339	chr18:77920426-77920485	PARD6G-AS1
A_32_P174083	6.154981	5.5933204	NM_018947	chr7:25158379-25158320	CYCS
A_32_P203786	6.444034	6.2127433	NR_026858	chr15:30892382-30892441	ULK4P1
A_33_P3315824	4.815618	4.3976183		chr10:002264349-002264408	
A_23_P54179	8.29318	8.229275	NM_021188	chr14:74398545-74398604	ZNF410
A_33_P3242543	4.107775	4.432846	NM_001270458	chrX:43606008-43606067	MAOA
A_33_P3294654	4.6680737	4.5339804	NM_030907	chr1:16558314-16558255	RSG1
A_23_P20275	10.390371	10.39362	NM_024613	chr8:96168760-96168819	PLEKHF2
A_23_P431853	10.3673525	10.401059	HW291277	chrM:4914-4973	ND2
A_33_P3240053	4.578471	4.4869914	NM_001015072	chr7:100486523-100486464	UFSP1
A_23_P130553	4.682435	4.0379205	NM_138392	chr19:41092832-41094570	SHKBP1
A_33_P3278152	7.1711655	6.877204		chr15:085810402-085810343	
A_23_P173	5.52225	5.6230164	NM_001231	chr1:160171421-160171480	CASQ1
A_33_P3269678	10.450223	9.928231	NR_015395	chr2:112124646-112124587	MIR4435-1HG
A_23_P205098	7.137723	6.6227107	NM_015032	chr13:33351525-33351584	PDS5B
A_23_P316085	4.831443	4.7346573	NR_045180	chr13:36939665-36939724	SPG20OS
A_33_P3271341	5.200232	5.3868804	NM_001278081	chr16:57850762-57850821	LOC388282
A_33_P3288110	7.996925	8.111427	NM_001005476	chr2:159514766-159514825	PKP4
A_23_P164179	6.644638	6.9259367	NM_005749	chr17:48940557-48940498	TOB1
A_33_P3298750	4.354736	3.5208948	BC012881	chr20:47319537-47319596	
A_23_P78458	5.8889523	5.904082	NM_021632	chr19:52467706-52467647	ZNF350
A_24_P250335	9.218336	9.071014	NM_004596	chr19:41269546-41270937	SNRPA
A_33_P3360887	12.563514	12.555401	BC104974	chr20:62201648-62201589	HELZ2
A_33_P3280355	13.4944725	13.267508		chrX:016492298-016492239	
A_33_P3384958	5.5748367	5.887812	NM_014839	chr1:99771632-99771691	LPPR4
A_33_P3342628	8.867746	8.877096	NM_021170	chr1:934404-934345	HES4
A_33_P3336437	3.4666417	3.202313	NM_015072	chr14:76173990-76174049	TTLL5
A_23_P135357	8.6097145	8.635883	NM_018146	chr17:695542-695601	RNMTL1
A_23_P152807	8.657386	8.83657	NM_018346	chr17:48563055-48563114	RSAD1
A_24_P304051	12.041058	11.809391	NM_004832	chr10:106025842-106025901	GSTO1
A_33_P3216664	5.5176673	5.898294	NR_026925	chr2:239133900-239133841	LOC151174
A_24_P54131	6.3209696	6.1900525	NM_022836	chr1:114455973-114456032	DCLRE1B
A_23_P141894	3.930831	3.9672356	NM_006505	chr19:45162098-45162157	PVR
A_23_P386561	7.469075	6.322444	NM_001002926	chr7:19735257-19735198	TWISTNB
A_23_P60146	6.7715163	6.995493	NM_006207	chr8:17500476-17500535	PDGFRL
A_23_P30805	7.7420993	7.779931	NM_021968	chr6:27792176-27792235	HIST1H4J
A_32_P471485	2.6769981	3.3310664	NM_001282941	chr10:63998464-63998405	RTKN2
A_23_P363826	4.321289	4.2113853	NM_033406	chr11:33768335-33768289	FBXO3
A_24_P118884	3.4043765	2.8946695		chr1_gl000191_random:000078041-000077982	
A_23_P22499	8.621458	8.381455	NM_019067	chrX:54585075-54586998	GNL3L
A_32_P115050	3.647499	2.3900566	NR_037595	chr4:145564837-145564778	HHIP-AS1
A_33_P3252989	9.722679	10.006217	NM_015037	chr10:75561488-75561547	ZSWIM8
A_33_P3599591	5.2024612	4.669301	NM_002581	chr9:119164007-119164066	PAPPA
A_23_P25873	8.444539	8.241516	NM_007086	chr14:55408024-55407965	WDHD1
A_23_P50775	9.7251005	9.778433	NM_024509	chr19:36436025-36436084	LRFN3
A_23_P393015	9.3309555	9.330259	NM_138403	chr7:101256776-101256717	MYL10

A_24_P367242	3.553961	4.351853	NM_181607	chr21:31852472-31852413	KRTAP19-1
A_33_P3327454	10.887814	10.730089	NM_133368	chr16:57272881-57272940	RSPRY1
A_23_P35995	9.451525	8.72127	NM_024769	chr11:122943288-122943229	CLMP
A_24_P80338	8.828889	8.562235	NM_003157	chr3:52744873-52744814	NEK4
A_23_P155969	8.083696	8.287402	NM_014264	chr4:128816182-128816241	PLK4
A_23_P129659	7.1322618	6.5911217	NM_138447	chr16:30615108-30615049	ZNF689
A_33_P3368895	5.8381395	5.942605		chr2:106471591-106471532	
A_23_P168551	7.6372237	7.4980125	NM_001040661	chr7:5343106-5343165	SLC29A4
A_23_P100220	6.1327705	5.967511	NM_024939	chr16:68263113-68263054	ESRP2
A_32_P54018	9.547588	9.82332	NM_020696	chr3:44795802-44795743	KIAA1143
A_23_P156471	9.815199	9.803656	NM_001253	chr6:44413487-44413546	CDC5L
A_33_P3392740	4.358451	4.5405846	AL831857	chr2:85999972-86000031	ATOH8
A_24_P101402	12.375852	12.1346035	NM_006392	chr20:2638880-2638939	NOP56
A_33_P3245006	6.913238	6.913894	NM_015533	chr11:61116171-61116230	DAK
A_24_P152094	12.519176	11.939888	XM_003960530	chr13:53394782-53394841	LOC101060458
A_33_P3351920	4.053121	4.5239897			
A_33_P3379775	6.41404	6.5031614		chr9:84248423-84248384	TLE1
A_33_P3367361	9.210712	9.223632	NM_001282695	chr10:14560784-14560725	FAM107B
A_33_P3226542	7.19625	7.599153	NR_003271	chr17:18965382-18965441	SNORD3B-1
A_23_P152420	7.1259456	7.307734	NM_014615	chr16:85706645-85706704	GSE1
A_32_P352697	5.6445413	5.4477177	NM_001031732	chr4:69176348-69176289	YTHDC1
A_33_P3251901	6.0346365	6.3174806	NM_004307	chr4:40812316-40812257	APBB2
A_33_P3389942	13.165405	13.058784	NM_017838	chr5:177576527-177576468	NHP2
A_23_P94422	10.360766	10.479876	NM_014791	chr9:36677192-36677251	MELK
A_23_P87769	9.694223	9.730131	NM_017915	chr12:102591152-102591211	PARPBP
A_24_P920979	6.790106	6.968	NM_198243	chr15:101191472-101191531	ASB7
A_33_P3772937	6.0123596	6.01385	AL133645	chr3:160287003-160287062	KRT8P12
A_24_P408457	5.7934327	5.964403	NM_000262	chr22:42455043-42454984	NAGA
A_23_P145863	11.354592	11.373728	NM_005620	chr1:152005219-152005160	S100A11
A_33_P3325723	6.5541735	6.738007	NM_001822	chr2:175664776-175664717	CHN1
A_24_P212531	4.9489975	4.82407	NM_152490	chr1:235647708-235647649	B3GALNT2
A_24_P181422	4.9235854	4.960019	NM_172097	chr15:43931908-43931849	CATSPER2
A_23_P62099	2.3221061	2.3900566	NM_016249	chrX:141290382-141290323	MAGEC2
A_24_P99046	3.660012	3.880737	NM_015000	chr12:27478395-27478454	STK38L
A_33_P3217230	3.1424992	3.351147	NM_003430	chr19:23540740-23540681	ZNF91
A_24_P373174	6.3565416	5.4698677	NM_004580	chr15:55496584-55496525	RAB27A
A_33_P3403494	5.429881	5.7733383	NR_033930	chr2:131295999-131295940	LOC646743
A_23_P121182	9.293677	9.570557	NM_012260	chr3:15602366-15602307	HACL1
A_33_P3275741	6.154252	6.304735	AK309540	chr1:27697023-27696964	FCN3
A_24_P174613	8.229275	8.605788	NM_033632	chr4:153242517-153242458	FBXW7
A_33_P3261640	5.8108625	5.7311244		chr2:098201033-098200974	
A_33_P3350858	3.7199419	3.2311819		chr15:33534864-33534805	TMCO5B
A_23_P206856	8.023308	8.341095	NM_030581	chr16:74907915-74907856	WDR59
A_23_P140928	3.9113586	2.9833174	NM_024847	chr16:19074010-19074069	TMC7
A_32_P69465	9.439858	9.681005	NM_001145450	chr2:39109569-39109628	MORN2
A_33_P3219720	11.584587	11.555136	BC021819	chr10:38091839-38091780	ZNF248
A_23_P413761	13.315342	13.025798	NM_003017	chr6:36570424-36570483	SRSF3
A_24_P68991	4.021136	3.5132487	NM_006168	chr4:85418721-85416948	NKX6-1
A_33_P3299590	6.2348223	6.266289	NM_001128854	chr7:100479801-100479860	SRRT
A_33_P3884230	4.8518696	4.3801565	NM_002501	chr19:13186372-13186431	NFIX
A_33_P3266010	11.088631	11.030645	NM_004292	chr11:66099619-66099560	RIN1
A_33_P3821660	5.2166824	5.058665	NM_001173989	chr9:139726728-139726787	RABL6
A_33_P3300346	3.7711203	3.0639935	NM_001014283	chr13:114128499-114128440	DCUN1D2
A_33_P3253304	5.6576195	4.8755803		chr10:099477277-099477218	
A_23_P163567	6.126498	6.0894394	NM_018667	chr16:68392309-68392250	SMPD3
A_33_P3354796	7.2994494	6.6429663	NR_024492	chr16:2604120-2604061	FLJ42627
A_23_P215175	8.179366	8.263453	NM_005692	chr7:150912012-150911266	ABCF2
A_23_P422178	7.721563	7.6020308	NM_003588	chrX:119666403-119666344	CUL4B
A_33_P3324552	4.921929	4.9145694		chr17:020690483-020690542	

A_33_P3319134	5.660081	5.3381567	AF289610	chr2:91833775-91833716	LOC100506191
A_33_P3219010	4.4997497	4.7330284	AK299501	chr4:106394684-106394625	PPA2
A_33_P3271196	4.8257947	4.878249	NM_130847	chr11:94609844-94609903	AMOTL1
A_33_P3224100	4.206569	5.122165	NM_152632	chrX:35990017-35990076	CXorf22
A_32_P104063	10.321963	10.144569	NR_034105	chr16:54952978-54952919	CRNDE
A_33_P3211956	12.044691	12.030514	NM_005873	chr20:62704593-62704534	RGS19
A_33_P3380056	8.034847	7.4968834	NM_001198807	chr9:103207453-103207512	MSANTD3
A_33_P3273233	5.024614	5.4065456	XR_109334	chr16:87728930-87728871	
A_23_P10077	3.0922573	2.3900566	NM_020376	chr11:824896-824955	PNPLA2
A_33_P3329750	4.9925957	5.094888		chr10:38147400-38147459	
A_32_P142881	7.5583973	7.5733104	NM_015092	chr16:18870910-18870477	SMG1
A_32_P145153	15.617697	15.455059	NM_000993	chr2:101622437-101622496	RPL31
A_23_P46936	2.3221061	2.3900566	NM_000399	chr10:64571909-64571850	EGR2
A_33_P3287314	4.0158944	4.164521		chr7:014522742-014522801	
A_23_P11286	9.588752	8.808231	NM_019597	chrX:100668686-100668745	HNRNPH2
A_23_P426663	6.60544	6.5057845	NM_198159	chr3:70017269-70017328	MITF
A_33_P3423979	7.0176992	6.958085	NM_001166109	chr4:169837061-169837120	PALLD
A_33_P3225882	5.1653714	5.3668947	NM_001098787	chr11:206013-205954	BET1L
A_33_P3395310	4.696906	5.1020656		chr4:054244117-054244058	
A_33_P3309929	11.095562	10.966759	NM_003883	chr5:141000518-141000459	HDAC3
A_33_P3407863	10.258841	10.708387	XR_133565	chrX:151073089-151073030	
A_33_P3240244	3.7289357	3.6422482	NM_001136003	chr1:151810645-151810586	C2CD4D
A_23_P393749	3.837684	2.3900566	NM_178019	chr5:134346067-134346126	CATSPER3
A_33_P3256677	6.559472	6.5691133	NM_138414	chr16:28602171-28602230	CCDC101
A_23_P207940	9.457543	9.303282	AK091525	chr18:12785560-12785501	
A_33_P3399208	5.984374	6.2457557	XM_005275377		HLA-B
A_24_P185604	7.856241	7.6030097	NM_032013	chr20:35280899-35280840	NDRG3
A_33_P3238250	8.305476	8.353155	NM_016946	chr1:160968009-160967950	F11R
A_33_P3298430	5.85499	5.966026	NM_001010924	chr10:15254973-15254914	FAM171A1
A_24_P112160	5.3143587	5.5954523	NM_030570	chr7:76143315-76143374	UPK3B
A_33_P3221384	7.5998216	7.526454		chr2:133033186-133033245	
A_23_P415411	9.98218	9.868448	NM_003545	chr6:26205172-26205231	HIST1H4E
A_23_P70384	7.7512665	6.7842183	NM_003958	chr6:37348954-37349013	RNF8
A_23_P50535	8.5984125	8.889839	NM_004409	chr19:46273080-46273021	DMPK
A_33_P3248272	7.055211	6.9337826	NM_005671	chr8:30623758-30623817	UBXN8
A_33_P3399064	5.286504	5.6122456		chrUn_gl000220:156050-156109	RNA5-8S5
A_24_P271830	4.574752	4.545495	NR_034145	chr17:12540240-12540299	LINC00670
A_33_P3418055	4.0980496	4.10099	NM_004234	chr19:44791624-44791565	ZNF235
A_33_P3380472	2.974103	2.3900566	NM_032554	chr12:123212374-123212315	HCAR1
A_23_P103282	10.814599	11.298914	NM_004872	chr1:54497886-54497827	TMEM59
A_32_P31182	11.783738	11.874897	NM_000971	chr8:74202885-74202845	RPL7
A_33_P3229002	5.2136645	5.4951124	NM_145272	chr17:34092038-34092097	C17orf50
A_33_P3293446	5.1764216	5.8010283	NM_020848	chr10:30303489-30303430	KIAA1462
A_33_P3399638	2.3221061	2.3900566	NM_033468	chr19:22273712-22273771	ZNF257
A_33_P3287567	6.759354	5.688092	NM_018844	chr7:107263682-107263741	BCAP29
A_23_P64173	3.9341667	3.6367135	NM_001017534	chr11:104915286-104915227	CARD16
A_23_P96209	7.6293726	7.699514	NM_025232	chr8:21995837-21995778	REEP4
A_33_P3363537	8.320787	8.29524	AW571659	chr7_gl000195_random:59862-59803	
A_23_P390704	4.739774	5.0651355	NM_007271	chr6:36462749-36462690	STK38
A_33_P3268304	7.193801	7.5029564	NM_001161404	chr2:128396064-128396005	LIMS2
A_33_P3317253	5.143901	4.8365364	NM_001001484	chr10:16555655-16555714	PTER
A_33_P3292417	11.237803	10.922672	NM_017588	chr9:137024979-137025038	WDR5
A_33_P3287770	10.419078	10.18601	NM_016111	chr16:1560392-1560451	TELO2
A_24_P96961	9.420826	8.539679	NM_025106	chr1:9429495-9429554	SPSB1
A_24_P335358	7.3804927	6.8059673	NM_025215	chr12:132426120-132426179	PUS1
A_23_P151075	6.2327185	6.0604877	NM_001175	chr12:15095117-15095058	ARHGDIB
A_33_P3219840	4.0317836	3.2540548	NR_026751	chr6:29968848-29968789	ZNRD1-AS1

A_24_P182494	7.2124596	7.2033157	NM_007207	chr1:221875832-221875773	DUSP10
A_23_P501795	7.9208126	7.5280356	NM_133640	chr9:136208145-136208086	MED22
A_23_P150950	7.516669	7.4650598	NM_144982	chr12:72003855-72003796	ZFC3H1
A_23_P70060	7.128351	7.165923	NM_176895	chr5:54721742-54721155	PPAP2A
A_33_P3620488	5.9126263	6.3074646	NM_021185	chr19:38861499-38861558	CATSPERG
A_33_P3232204	4.2367606	4.520443	AF289611	chr14:105891919-105891978	LOC100128343
A_33_P3245188	4.0208216	3.8936157	BC003386	chr12:78883587-78883646	
A_33_P3364268	3.997396	3.8980637	NM_030915	chr2:30480566-30480625	LBH
A_33_P3347035	4.578062	4.7021	BC069659	chr3:99653121-99653180	
A_33_P3374833	10.30001	10.134979	NM_012388	chr15:45901829-45901888	BLOC1S6
A_32_P228775	6.1715994	5.999906	NM_174907	chr3:73047288-73096374	PPP4R2
A_23_P324994	7.3044124	7.468062	NM_018846	chr7:23214406-23214465	KLHL7
A_23_P111188	5.5169296	5.3826523	NM_005453	chr6:33282716-33282657	ZBTB22
A_23_P252642	12.934422	12.806373	NM_152384	chr2:170362885-170362944	BBS5
A_24_P673063	11.014345	10.113346	NM_001444	chr8:82195741-82196134	FABP5
A_33_P3408844	4.469822	4.20646	NR_029411	chr7:76178891-76178950	LOC100133091
A_33_P3230414	4.225382	3.7957575	NM_001004309	chr15:90904655-90904714	ZNF774
A_33_P3424207	9.0531645	8.686992	NM_024816	chr16:28915816-28915757	RABEP2
A_23_P84836	7.951111	8.121242	NM_006310	chr17:45700259-45700318	NPEPPS
A_23_P230	5.0872536	4.8775144	NM_004623	chr1:55207100-55207159	TTC4
A_23_P150080	8.368539	8.132593	NM_183005	chr10:15145897-15145956	RPP38
A_33_P3275600	3.6008744	3.5029473	NM_001206670	chr15:42276061-42276002	PLA2G4E
A_33_P3339375	7.557264	7.6390924	NM_001039841	chr15:30927761-30927820	ARHGAP11B
A_24_P169634	2.3221061	2.3900566		chr10:81682676-81682735	MBL1P
A_33_P3260445	4.997044	5.1317472	AK026669	chr6:159058312-159058253	DYNLT1
A_24_P4170	5.853998	5.9325604	NM_020361	chr8:68396957-68396078	CPA6
A_23_P385427	8.887455	9.126371		chr19:019946094-019946153	
A_23_P16573	7.5566254	7.4729104	NM_019070	chr19:19038775-19038834	DDX49
A_23_P352389	4.296053	4.696906	NM_152343	chr17:43331858-43331799	SPATA32
A_23_P17955	6.17679	6.520181	NM_012157	chr3:33427777-33427836	FBXL2
A_33_P3351851	6.0963283	5.764965	NM_003546	chr6:27841113-27841054	HIST1H4L
A_23_P4919	9.506932	9.725924	NM_006801	chr19:48887620-48887561	KDELR1
A_23_P131050	4.0022273	3.83845	NM_030924	chr19:6192982-6193041	ACSBG2
A_23_P41942	5.8750486	5.7859735	NM_006467	chr5:89781367-89781426	POLR3G
A_24_P204238	9.905848	9.884188		chr7:148334540-148334599	
A_23_P162945	9.662827	9.911865	NM_003136	chr14:35492223-35492282	SRP54
A_33_P3235891	7.954262	8.245813	AK128523	chr4:173908683-173908624	
A_23_P62741	6.8462687	6.6066446	NM_022159	chr1:79383345-79358842	ELTD1
A_23_P14886	8.543917	8.880909	NM_032520	chr16:1412854-1413003	GNPTG
A_23_P19657	6.099721	6.506564	NM_032832	chr6:150141039-150140980	LRP11
A_24_P108301	7.2099605	7.051199	NM_173630	chr18:67671163-67671104	RTTN
A_33_P3329187	11.748842	11.429198	NM_001130823	chr19:10244082-10244023	DNMT1
A_23_P28084	9.819443	9.84982	NM_001271610	chr19:13254992-13254933	STX10
A_33_P3414561	3.962335	3.5665817		chr15:057138683-057138742	
A_23_P121106	4.005909	3.5909886	NM_003865	chr3:57232831-57232512	HESX1
A_24_P941376	3.2358127	3.5510755	NM_015428	chr19:50551363-50551422	ZNF473
A_23_P97394	10.583321	9.684581	NM_003567	chr1:94027798-94027739	BCAR3
A_33_P3316639	7.369612	7.7637963	NM_001193517	chr2:86756500-86756441	CHMP3
A_23_P310	7.345435	7.645521	NM_023009	chr1:32800015-32799956	MARCKSL1
A_23_P61447	8.367172	8.36564	NM_004453	chr4:159629645-159629704	ETFDH
A_33_P3302796	12.280785	12.156997	NM_007279	chr19:56186011-56186070	U2AF2
A_23_P211047	8.182029	8.228208	NM_206866	chr21:30717980-30718039	BACH1
A_33_P3310047	11.069655	10.890676	NM_020717	chrX:50335055-50334996	SHROOM4
A_33_P3382236	4.2713385	4.2631316	NM_001258299	chr10:118674946-118674887	KIAA1598
A_24_P41408	4.763719	4.540166	XR_108730	chr7:1888534-1888593	LOC100128374
A_24_P166663	10.203238	10.571036	NM_001259	chr7:92234502-92234443	CDK6
A_33_P3368900	5.860708	5.9531884		chr4:057976492-057976551	
A_32_P192474	4.757128	5.158902	NM_030651	chr6:32116446-32116387	PRRT1
A_23_P71752	11.124192	11.489645	NM_006007	chr9:74969825-74969766	ZFAND5

A_33_P3352053	4.674185	5.0377903	AK125828	chr1:1108495-1108436	TTLL10-AS1
A_32_P26376	6.4995375	6.25922	NM_152305	chr3:119213403-119213462	POGLUT1
A_24_P345679	4.966527	5.6218414	NM_001195432	chr3:158323779-158323838	MLF1
A_24_P648176	5.714176	6.360988	NM_001001660	chr12:25357149-25357207	LYRM5
A_33_P3277140	12.043724	12.223671	NM_001032363	chr1:19950017-19950076	MINOS1
A_33_P3251093	9.100168	8.765392	NM_078483	chr5:150871862-150871921	SLC36A1
A_23_P79836	8.956975	9.246224	NM_006811	chr20:43128430-43128371	SERINC3
A_33_P3306207	5.9551477	6.137329	NM_198508	chr7:139138917-139138858	KLRG2
A_23_P68211	9.763165	9.648082	NM_003124	chr2:73118969-73119028	SPR
A_23_P59798	9.883702	10.201681	NM_013446	chr7:140153026-140152967	MKRN1
A_33_P3308158	8.583399	8.80174		chr7:102246615-102246556	
A_24_P933011	4.7932596	4.6364408	NM_001080209	chr18:5891958-5891899	TMEM200C
A_33_P3512350	5.0320835	5.1043897		chr2:64843555-64843614	LOC339807
A_23_P102258	10.995432	10.836863	NM_053050	chr2:74699266-74699207	MRPL53
A_23_P169154	5.8209257	6.0316124	NM_024896	chr9:5785753-5785694	ERMP1
A_24_P336931	5.7707105	5.3106036	NM_001164315	chr2:97914922-97915318	ANKRD36
A_24_P832156	9.616314	9.842416	NM_198841	chr9:96208883-96208824	FAM120AOS
A_33_P3237899	3.5268881	3.406035	NM_004067	chr7:29440292-29440351	CHN2
A_24_P374427	5.602486	5.174518	NM_178566	chr9:14617312-14617253	ZDHHC21
A_33_P3258699	6.40427	6.4307175	NM_001127386	chr10:135485016-135485075	DUX4L2
A_33_P3217119	5.931234	6.4528074	NM_206837	chr1:36893980-36893921	OSCP1
A_33_P3229288	7.032381	7.282345	NM_000789	chr17:61574915-61574974	ACE
A_33_P3375348	3.8122566	3.7844532		chrX:048912525-048912466	
A_33_P3244998	14.957516	14.957516	NM_001007022	chr1:86816981-86816922	ODF2L
A_23_P121885	5.529925	6.0450172	NM_031916	chr5:10461437-10464984	ROPN1L
A_23_P132644	11.73924	10.755817	NM_020792	chr3:172348635-172348576	NCEH1
A_23_P207927	8.500267	8.367632	NM_017941	chr17:71244655-71244714	C17orf80
A_23_P434301	12.774128	12.791592	NM_002823	chr2:232577717-232577776	PTMA
A_32_P7204	5.9707294	5.933475	NM_004249	chr4:13378218-13371580	RAB28
A_24_P110799	8.388909	8.375935	NM_002340	chr21:47609358-47609299	LSS
A_32_P28402	7.510259	6.8977795	NR_015341	chr17:28964201-28964260	LRRC37BP1
A_23_P251377	7.719603	7.8463745	NM_001006109	chr3:129020861-129020920	HMCES
A_32_P34920	2.3221061	2.3900566	NM_004472	chr5:72742495-72742436	FOXD1
A_33_P3270369	3.738911	3.9820237	AK130406	chr3:15504225-15504166	
A_23_P149221	7.790676	8.055392	NM_024602	chr1:45468884-45468825	HECTD3
A_24_P84008	7.5439587	7.4488	AI198876	chr15:035384686-035384745	
A_32_P215938	9.944758	9.996573	NM_001145638	chr9:139253964-139254023	GPSM1
A_33_P3236632	6.0214543	6.3372717	NM_198551	chr1:222835614-222835673	MIA3
A_23_P210581	4.474537	4.745169	NM_002237	chr20:49620322-49620263	KCNG1
A_23_P384635	8.230398	8.197138	NM_173543	chr3:137780951-137780892	DZIP1L
A_23_P13852	12.608693	12.227722	NM_007178	chr12:16056307-16056366	STRAP
A_33_P3228837	5.1871824	5.5545835	NM_001145873	chr2:87016506-87016447	CD8A
A_33_P3254912	2.3221061	2.3900566	NM_001005194	chr11:124440780-124440839	OR8A1
A_23_P218637	9.591877	9.7221	NM_005054	chr2:110614795-110614854	RGPD5
A_33_P3492042	4.438177	4.3619223		chr21:31581529-31581470	LINC00307
A_24_P73943	6.3760476	6.4147234	NM_004375	chr17:53038762-53038703	COX11
A_33_P3344406	5.0624433	4.4393673	NM_001145785	chr19:19256517-19256458	MEF2B
A_23_P163639	9.031422	8.807974	NM_013275	chr16:89334490-89334431	ANKRD11
A_32_P173298	3.7814946	3.4360757	NM_012426	chr16:70562923-70562982	SF3B3
A_23_P68899	9.214494	9.212167	NM_012473	chr22:36863931-36863872	TXN2
A_33_P3211229	3.2671356	3.4318094	NM_017515	chr11:107677584-107677525	SLC35F2
A_33_P3230990	6.4200506	6.6985664	NM_173050	chr22:43599347-43599288	SCUBE1
A_23_P37167	3.4312232	3.7873478	NM_002471	chr14:23854238-23854179	MYH6
A_23_P137103	14.116425	13.966563	NM_001416	chr17:7481719-7481778	EIF4A1
A_33_P3266080	8.281887	8.364084	NM_006473	chr11:62554755-62554814	TAF6L
A_23_P12477	4.9063034	5.2356853	NM_012236	chr1:41493592-41493533	SCMH1
A_24_P66932	8.936964	8.7806	NR_033244	chr5:141276021-141275962	LOC729080
A_32_P226149	12.268318	12.2763605	NM_145690	chr8:101931167-101931108	YWHAZ
A_23_P108342	4.543262	4.639143	NM_016536	chr19:38055552-38055493	ZNF571

A_23_P347029	3.8887336	4.315841	NM_176890	chr12:11138946-11138887	TAS2R50
A_23_P409951	15.149481	14.93526	NM_004152	chr19:2273198-2273257	OAZ1
A_32_P116857	5.8709784	4.9663167	NM_016953	chr2:178489104-178489045	PDE11A
A_23_P7144	10.1319065	9.656863	NM_001511	chr4:74736850-74736909	CXCL1
A_24_P152356	10.616875	10.817555		chr1:171794101-171794042	
A_23_P333129	6.900267	6.602396	NM_001177376	chr4:190993379-190993446	DUX4L4
A_23_P252362	8.493911	8.298345	NM_016640	chr5:44812022-44812081	MRPS30
A_23_P34733	9.801134	9.625463	NM_000143	chr1:241667485-241667426	FH
A_24_P305223	4.018555	4.6083646	NM_172241	chr18:19995982-19995923	CTAGE1
A_23_P115872	11.502492	11.635044	NM_018131	chr10:95288754-95288813	CEP55
A_33_P3404959	7.3891296	7.7388678	NM_001013649	chr2:85833846-85833787	C2orf68
A_23_P397543	4.4714527	4.204613	NR_026873	chr7:65841248-65841189	LINC00174
A_33_P3352887	5.1921296	5.054326	NR_027002	chr1:149291566-149291625	LOC388692
A_23_P152002	2.716063	2.3900566	NM_004049	chr15:80263195-80263136	BCL2A1
A_23_P45345	9.909222	9.718708	NM_014500	chrX:135594216-135594275	HTATSF1
A_33_P3258339	4.4845004	4.8409386	NM_001113567	chr17:16346634-16346575	FAM211A
A_33_P3306217	5.7603273	5.838744	NM_172016	chr6:30309778-30309837	TRIM39
A_24_P118472	8.073063	8.228928		chr7:105170888-105170949	
A_23_P358195	5.298943	5.3796153	NM_001164465	chr15:82637381-82637322	GOLGA6L10
A_23_P161190	16.084991	15.910685	NM_003380	chr10:17278347-17279256	VIM
A_24_P401645	6.4535213	6.404907	NM_001254728	chr14:31562244-31562303	AP4S1
A_24_P196592	6.4562674	6.6861315	NM_001032278	chr17:34106300-34106073	MMP28
A_33_P3287379	5.573608	5.831581	NM_032382	chr16:69364937-69364878	COG8
A_33_P3229722	4.9571886	5.0485096	NM_006814	chr20:1106195-1106254	PSMF1
A_33_P3404331	5.822172	5.8750486	NM_015093	chr6:149700594-149700653	TAB2
A_23_P369987	7.5590744	8.013339	NM_001039479	chr14:75128314-75128255	AREL1
A_33_P3368471	6.0548344	6.6205277	NM_152729	chr6:116544229-116544288	NT5DC1
A_24_P83075	6.46312	6.5287275	AF426264	chr21:46714317-46714258	LINC00205
A_24_P67585	6.9575615	7.05664	NM_016206	chr3:87017874-87017815	VGLL3
A_23_P161156	6.886508	6.7796063	NM_182755	chr10:31133829-31133770	ZNF438
A_23_P47058	5.564177	5.3993134	NM_022034	chr10:124591875-124591816	CUZD1
A_32_P199767	4.335478	3.9954464	NM_001037735	chrX:47918269-47918210	ZNF630
A_33_P3331711	4.4246993	4.7690544	AK124300	chr13:115058194-115058253	LOC100130463
A_33_P3812815	5.352765	5.2431164	NM_000296	chr16:2165439-2165380	PKD1
A_33_P3225512	4.177886	4.757071	NM_002535	chr12:113448429-113448488	OAS2
A_33_P3227252	9.94143	10.18218		chr6:000145863-000145922	
A_23_P1998	5.3494167	5.758069	NM_001164	chr11:6422656-6422597	APBB1
A_23_P155755	2.3221061	2.3900566	NM_002993	chr4:74703368-74703427	CXCL6
A_33_P3344648	5.9466486	6.080084	NR_027283	chr17:66196091-66196150	LOC440461
A_23_P317207	6.7731886	6.980636	NM_153340	chr1:110033888-110033947	ATXN7L2
A_23_P160226	4.914469	5.111308	NM_001039464	chr1:55175863-55175922	MROH7
A_24_P225604	8.826118	8.71665	NM_018981	chr2:183623959-183624018	DNAJC10
A_33_P3274930	4.0604835	3.1229777		chr17:015138596-015138537	
A_33_P3225572	9.672739	9.866744		chr7:027278509-027278450	
A_32_P139894	8.607582	8.430393	NM_007314	chr1:179068871-179068812	ABL2
A_23_P337658	3.391696	3.778824	NM_001631	chr2:233323933-233323992	ALPI
A_33_P3285809	6.1581416	6.343542	NM_001166417	chr1:85116133-85116074	SSX2IP
A_33_P3310929	5.1320887	5.073261	NM_003474	chr10:127703502-127703443	ADAM12
A_33_P3369286	10.052795	9.3309555	NM_006390	chr12:30781982-30781923	IPO8
A_33_P3385775	8.353155	8.757874	NM_025069	chr8:37556337-37556396	ZNF703
A_23_P144476	6.628951	7.1907234	NM_199327	chr4:124324494-124324553	SPRY1
A_33_P3317752	4.7831593	4.995934	NM_033061	chr17:39240459-39240518	KRTAP4-7
A_33_P3289777	4.567013	4.622943	NR_103825	chr4:119554019-119554078	LOC729218
A_23_P106391	6.8592434	6.4987936	NM_020147	chr15:71173920-71173861	THAP10
A_32_P60223	8.564769	8.323123	NM_032329	chr2:242668673-242668732	ING5
A_24_P173325	12.567562	12.784931	NM_001033930	chr19:18685718-18685777	UBA52
A_32_P11894	8.9641	8.927507	NM_152269	chr12:123738478-123741393	C12orf65
A_33_P3230876	7.8525505	7.6024885	NM_001004127	chr13:52603719-52603778	ALG11
A_23_P207319	7.9560714	8.058305	NM_003954	chr17:43340768-43340709	MAP3K14

A_24_P347480	9.396799	8.73814	NM_033116	chr14:75548984-75548925	NEK9
A_23_P165333	11.402478	11.752492	NM_139346	chr2:127806001-127805942	BIN1
A_23_P52336	8.824269	8.014683	NM_170744	chr10:73059597-73059656	UNC5B
A_23_P342000	2.69559	2.3900566	NM_144770	chr21:15600543-15600602	RBM11
A_33_P3372666	4.7677927	5.0877047	NM_033023	chr7:557091-557032	PDGFA
A_23_P152136	9.91538	9.534749	NM_022770	chr16:58439799-58439858	GINS3
A_24_P340941	5.7198677	5.4690814	NR_036507	chr15:49448655-49448714	NDUFAF4P1
A_33_P3333985	9.078637	9.412672		chr8:057389399-057389340	
A_23_P110445	5.008777	5.340018	NM_006051	chr5:139938147-139938088	APBB3
A_24_P90878	7.700168	7.729939	NM_017736	chr16:20745705-20745646	THUMPD1
A_33_P3421178	7.672755	7.4859734	DA381791	chr12:76425001-76425060	
A_23_P202392	8.696886	8.834133	NM_024670	chr10:14944641-14944700	SUV39H2
A_23_P79931	6.0907936	5.764454	NM_139322	chr20:3578630-3581634	ATRN
A_24_P605190	6.7086573	6.6507263	NM_005023	chr5:114547105-114547046	PGGT1B
A_23_P502425	12.12283	11.620441	NM_020409	chr3:179306702-179306643	MRPL47
A_23_P376661	7.3256874	7.598882	NM_005600	chr1:161089963-161090022	NIT1
A_33_P3319261	5.2633705	5.475896	NM_001286823	chr9:140260788-140260729	EXD3
A_23_P252962	7.4508257	7.4189415	NM_001001132	chr21:35209668-35209727	ITSN1
A_24_P365180	4.137195	3.856232	NM_032160	chr18:65174204-65174145	DSEL
A_24_P284805	4.189258	4.3141623	NM_001284286	chr22:31529621-31529680	INPP5J
A_33_P3352340	6.648364	6.75027	NM_001190460	chr17:39346832-39346891	KRTAP9-1
A_23_P69958	11.359117	11.642081	NM_001284	chr5:115249541-115249600	AP3S1
A_23_P162807	9.17783	8.838139	NM_005830	chr13:41303704-41303645	MRPS31
A_24_P142305	7.1850433	7.0333943	NM_000517	chr16:222889-222948	HBA2
A_23_P46964	4.186547	4.5438337	NM_017902	chr10:102309134-102309193	HIF1AN
A_33_P3255404	2.617963	2.3900566	NM_005602	chr3:170151824-170151883	CLDN11
A_23_P305210	5.933876	5.8209257	NM_178558	chr7:63980328-63980269	ZNF680
A_23_P80122	8.520983	8.796535	NM_004627	chr21:40765178-40768822	WRB
A_24_P3804	6.1869287	6.0467944	NM_001039651	chr6:31732391-31732450	SAPCD1
A_23_P323143	5.271322	5.215121	NR_027788	chr7:149244836-149244777	ZNF767
A_33_P3441060	5.889145	5.9002466	NM_018452	chr6:157712865-157712806	TMEM242
A_23_P149375	8.257015	7.812969	NM_053055	chr1:151847228-151847169	THEM4
A_24_P288685	6.738529	7.210951	NM_001560	chrX:117892164-117895118	IL13RA1
A_24_P216313	8.08048	8.356015	NM_015966	chr20:34136265-34136324	ERGIC3
A_33_P3393931	5.1325574	5.519458	NR_103541	chr1:54703621-54703680	SSBP3-AS1
A_23_P204801	5.706334	5.9354305	NM_032148	chr12:105198910-105198851	SLC41A2
A_33_P3281066	7.670324	7.624865	NM_001098402	chr21:43408967-43408908	ZBTB21
A_33_P3377786	6.141676	6.44228	BC031259	chr14:106138665-106138606	
A_24_P372134	4.0936646	4.255438	NM_018295	chr7:134849663-134849722	TMEM140
A_23_P311020	9.080048	9.11476	NM_138798	chr2:99786026-99785888	MITD1
A_33_P3216568	4.4082756	4.136576	XM_003403450	chr11:1161827-1161886	MUC5AC
A_23_P121276	6.787264	6.6546235	NM_003157	chr3:52771728-52771669	NEK4
A_23_P90357	7.9882298	8.150258	NM_001060	chr19:3594819-3594760	TBXA2R
A_23_P157170	5.480954	5.8580036	NM_032317	chr7:73096929-73096870	DNAJC30
A_33_P3265956	6.6619644	6.866972	JF432662	chr20:32211677-32211736	CBFA2T2
A_33_P3362321	8.041981	7.6312532	NM_201555	chr2:105990080-105990021	FHL2
A_24_P24790	14.88216	14.840301		chr12:066431181-066431241	
A_23_P165442	7.458365	7.743072	NM_013392	chr2:27664188-27664483	NRBP1
A_33_P3415560	3.901208	4.478866	NM_133466	chr19:36883710-36883651	ZFP82
A_23_P131771	7.099357	6.8997173	NM_199360	chr20:62521922-62521981	TPD52L2
A_24_P104091	7.3490753	6.3087463	NM_153451	chr11:69480423-69480364	ORAOV1
A_23_P251095	11.042234	10.873702	NM_000284	chrX:19377888-19377947	PDHA1
A_33_P3262580	7.011495	7.2226696	AJ133134	chr10:97605337-97605396	ENTPD1
A_33_P3359012	6.9843864	7.264981	NM_004420	chr11:1575341-1575282	DUSP8
A_24_P315638	4.629771	4.85774	NM_001039165	chr11:3249338-3249279	MRGPRE
A_23_P211428	8.28427	7.9543896	NM_134269	chr22:31500343-31500402	SMTN
A_33_P3367447	4.9544587	4.8551674	NM_001161473	chr11:67793516-67793575	ALDH3B1
A_33_P3391167	6.977627	7.099571		chr7:063232261-063232202	
A_23_P68740	4.5372014	4.4010596	NM_000383	chr21:45717745-45717804	AIRE

A_23_P25638	7.4145446	7.2966976	NM_024546	chr13:79188826-79188767	RNF219
A_23_P36647	7.641865	7.7332253	NM_177441	chr17:42268651-42268710	TMUB2
A_33_P3355877	4.368519	4.3876357	NR_103795	chr4:47879145-47879086	NFXL1
A_23_P66715	3.949324	4.1729937	NM_033198	chr17:26882051-26881992	PIGS
A_33_P3287631	11.035692	10.863501	NM_147780	chr8:11700092-11700033	CTSB
A_23_P210920	10.075157	10.086915	NM_000178	chr20:33516441-33516382	GSS
A_24_P4661	9.140172	9.635812	NM_004879	chr11:125453433-125453492	EI24
A_32_P199884	2.3221061	2.3900566	NM_032132	chr1:150676635-150675852	HORMAD1
A_23_P16058	4.4919133	4.5736713	NM_145288	chr19:45575588-45575529	ZNF296
A_33_P3300212	4.1781554	4.3271875	NM_001167820	chr20:36147393-36147334	BLCAP
A_23_P353014	8.902934	8.1207695	NM_172364	chr12:1902076-1902017	CACNA2D4
A_33_P3340254	9.358353	9.022532	NM_003482	chr12:49412839-49412780	KMT2D
A_33_P3358019	6.4902635	6.5820127		chr9:134431521-134431580	XLOC_014512
A_33_P3341540	5.114384	5.3263283		chr6:058245303-058245362	
A_23_P380298	5.069311	5.1499505	NM_001282533	chr20:3143868-3143809	LZTS3
A_33_P3292679	9.474898	9.511553	NM_021249	chr14:35032095-35032036	SNX6
A_33_P3365524	7.0940065	6.9929266	NM_199123	chr14:99876202-99876143	SETD3
A_24_P198820	7.4311857	7.245281	NM_016507	chr17:37690454-37690513	CDK12
A_23_P65386	6.7238193	6.0009828	NM_023112	chr14:94514424-94514483	OTUB2
A_23_P102706	7.267369	7.010354	NM_014723	chr20:1289612-1289671	SNPH
A_33_P3262927	5.5592794	5.3188834	NM_001174100	chr3:51992942-51992883	PCBP4
A_24_P13041	6.417935	5.4975634	NM_145307	chr10:63953284-63953225	RTKN2
A_23_P133227	10.254412	10.151905	NM_002109	chr5:140053796-140053737	HARS
A_24_P364042	4.8536925	4.972337	NM_005972	chr10:47087655-47087714	NPY4R
A_23_P379794	5.724474	5.8251686	NM_178517	chr17:34894368-34894427	PIGW
A_32_P514599	9.562008	9.503187		chr3:036809424-036809365	
A_23_P26629	4.3718824	4.257105	NM_013258	chr16:31213029-31212970	PYCARD
A_24_P66838	7.545857	7.5980215		chr2:206905394-206905453	
A_23_P118105	11.373219	11.358183	NM_004548	chr16:2011549-2011608	NDUFB10
A_24_P393844	7.8236856	7.445252	NM_001384	chr1:44438390-44438449	DPH2
A_33_P3296975	3.5963793	2.3900566	AK093353	chr11:113660724-113660783	
A_23_P379746	6.5257196	6.5454707	NM_144973	chr12:31605000-31604941	DENND5B
A_33_P3299705	12.914449	12.752876		chr16:14989959-14990017	
A_33_P3289561	6.6697254	6.0796795		chr1:016555236-016555177	
A_24_P116242	9.061154	9.904173	NM_014315	chr14:50249300-50249594	KLHDC2
A_24_P366457	13.730812	13.73581		chr6:114160582-114160641	
A_24_P344711	8.841984	8.849172	NM_020132	chr21:45404347-45404406	AGPAT3
A_24_P163405	6.095006	5.942141	NM_006510	chr6:28872413-28872354	TRIM27
A_24_P273143	11.5050535	11.47654	NR_024204	chr2:87820743-87820802	LINC00152
A_33_P3300092	13.074154	12.7895565	NM_001543	chr5:149937714-149937773	NDST1
A_23_P84219	2.3221061	2.3900566	NM_139248	chr3:185225760-185225701	LIPH
A_33_P3239864	4.021579	4.1767635	AK307698	chr15:65223775-65223834	ANKDD1A
A_23_P502575	7.7148037	7.846579	NM_177559	chr20:464048-463989	CSNK2A1
A_33_P3253460	8.363361	8.567611	AK095243	chr17:39807528-39807469	LOC100130654
A_33_P3378031	8.843342	9.016756	NM_152834	chr2:669482-669423	TMEM18
A_23_P93881	9.342943	9.667671	NM_182715	chr7:105731525-105731466	SYPL1
A_33_P3232980	4.6620493	4.5606294	NM_031417	chr19:45808482-45808541	MARK4
A_23_P24433	2.4746342	2.3900566	NM_003793	chr11:66331002-66330943	CTSF
A_24_P357100	10.083246	10.290577	NM_014310	chr22:35949610-35949669	RASD2
A_33_P3213235	4.294701	4.3486643	NM_198492	chr19:7794164-7794105	CLEC4G
A_24_P40978	4.322644	4.237425	NM_138496	chr8:145677764-145676117	CYHR1
A_24_P101812	5.8691053	7.00881		chr12:106565918-106565977	
A_24_P151	4.3709803	3.8719525	NM_003636	chr1:6158564-6158623	KCNAB2
A_33_P3227443	2.952322	2.3900566	NM_175900	chr16:29753866-29753807	C16orf54
A_32_P68746	5.885413	5.702439	NM_018225	chr9:33048163-33048104	SMU1
A_33_P3224324	3.1674504	2.3900566	NM_001143836	chr11:89223692-89223633	NOX4
A_33_P3259801	9.704455	10.099486	NM_199129	chr20:48740766-48740707	TMEM189
A_33_P3256083	4.1905985	4.426459		chr7:056752846-056752787	
A_33_P3349552	8.657967	8.7451515	NM_020764	chr16:2228170-2228111	CASKIN1

A_33_P3380762	3.6524022	2.3900566		chr1:59597676-59597617	HSD52
A_23_P205900	7.3075395	7.1506286	NM_001012338	chr15:88420244-88420185	NTRK3
A_33_P3298024	7.4027796	7.5494266	NM_001144070	chr17:48745220-48745279	ABCC3
A_23_P31116	11.634156	11.67376	NM_018473	chr6:24701794-24701853	ACOT13
A_24_P322741	6.63608	6.856288	NM_000628	chr21:34668516-34668575	IL10RB
A_23_P98963	7.2476115	6.5557346	NM_001277842	chr12:49082595-49082536	CCNT1
A_24_P333567	5.6504116	5.2543535	NR_027447	chr9:139980301-139980242	MAN1B1-AS1
A_23_P7684	6.2265005	6.369954	NM_024565	chr5:159679117-159679058	CCNJL
A_23_P403521	7.391888	7.451811	NM_020192	chr7:39611957-39612016	YAE1D1
A_32_P140706	6.743126	6.7202544	NM_000535	chr7:6026777-6026718	PMS2
A_33_P3371219	4.183828	4.491526	NM_002998	chr8:97623978-97624037	SDC2
A_23_P162165	6.4231787	6.838417	NM_023930	chr11:77727551-77727492	KCTD14
A_23_P47304	9.012252	9.007671	NM_004347	chr11:104868201-104868142	CASP5
A_23_P303260	6.8441806	6.9923267	NM_003569	chr6:132781224-132781165	STX7
A_23_P166663	9.343572	9.329668	NM_012096	chr3:57306405-57306464	APPL1
A_24_P335305	3.8437939	4.303872	NM_006187	chr12:113410131-113410190	OAS3
A_33_P3363061	4.3677444	4.577825	NM_001003790	chr8:37602105-37602164	ERLIN2
A_33_P3367126	6.590539	6.883378	NM_001177693	chr5:73169011-73169070	ARHGEF28
A_33_P3272823	3.9615278	4.5862427	NM_002361	chr19:35802858-35802917	MAG
A_23_P203445	6.922124	6.6069927	NM_001040697	chr11:18553746-18553687	UEVLD
A_23_P312358	6.2872257	6.1250353	NM_152751	chr10:13522938-13494639	BEND7
A_23_P30464	7.8062553	7.565845	NM_030567	chr5:176883144-176883203	PRR7
A_33_P3220095	7.1330433	6.919092	NM_001282933	chr20:32380015-32380074	ZNF341
A_33_P3390950	7.1635284	7.467403	AK125852	chr8:144824106-144824165	FAM83H-AS1
A_23_P250619	7.587784	6.555295	NM_153746	chr6:158094469-158094528	ZDHHC14
A_23_P27627	7.477378	7.4557514	NM_004317	chr19:12858226-12858285	ASNA1
A_23_P1523	7.2334247	6.7015643	NM_014578	chr11:66837936-66837995	RHOD
A_33_P3230548	10.43873	10.546116	NM_014875	chr1:200521383-200521324	KIF14
A_24_P136758	4.900886	4.471589	NM_018028	chr19:39874799-39874858	SAMD4B
A_33_P3408420	4.4287057	4.3457537	NM_001113498	chr14:47311067-47311008	MDGA2
A_33_P3269539	4.388438	4.713256	NM_058174	chr21:47546086-47546145	COL6A2
A_23_P76291	5.7357326	6.0411596	NM_007244	chr12:10999779-10999721	PRR4
A_33_P3424867	4.566374	4.409197		chr15:082993073-082993014	
A_23_P119448	6.75027	6.610898	NM_014931	chr19:55741657-55741598	PPP6R1
A_33_P3276638	7.742671	8.198608	NM_024567	chr8:28909030-28909089	HMBOX1
A_23_P125668	7.957752	8.066126	NM_145305	chrX:118587814-118587873	SLC25A43
A_24_P67552	9.188214	8.618015		chr2:028238959-028238900	
A_33_P3233219	4.898054	4.9626045	NM_005285	chr8:53852785-53852844	NPBWR1
A_23_P17144	10.048634	9.858577	NM_007266	chr2:27873534-27873593	GPN1
A_33_P3411279	4.2490773	4.2256823	NM_001128590	chr6:32006341-32006400	CYP21A2
A_23_P373799	7.5347767	7.4620814	NM_020943	chr2:180810231-180810172	CWC22
A_32_P60065	6.855465	6.413825	NM_004101	chr5:75911558-75911499	F2RL2
A_23_P379327	7.978269	8.130568	NM_001040450	chr15:59149158-59149217	FAM63B
A_23_P76918	10.336729	10.169877	NM_012460	chr14:58875525-58875466	TIMM9
A_23_P423197	11.126478	10.881432	NM_002957	chr9:137331772-137331831	RXRA
A_33_P3242659	4.3488436	4.044282	NM_022113	chr6:17764511-17764452	KIF13A
A_33_P3424612	5.045892	5.0867414	L37726	chr2:89326850-89326791	
A_23_P302681	6.944945	7.192102	NM_001042762	chr7:50512991-50512932	FIGNL1
A_33_P3263287	4.1359234	4.357027	AK126895	chr11:8059834-8059775	LOC100129111
A_23_P30254	13.277287	12.950653	NM_006622	chr5:57750052-57749993	PLK2
A_23_P112187	4.9205503	5.046422	NM_032843	chr9:133778556-133778497	FIBCD1
A_23_P4551	7.2813907	7.241291	NM_015559	chr18:42644693-42644752	SETBP1
A_23_P24044	4.298633	3.9275622	NM_017649	chr10:104837932-104837991	CNNM2
A_33_P3364641	4.196337	3.424055	AK124207	chr19:42749066-42749125	
A_23_P251316	8.272312	8.5632105	NM_001331	chr11:57586315-57586374	CTNND1
A_24_P101786	4.2247343	4.542825	NM_001271644	chr2:43793950-43793891	THADA
A_23_P93082	6.4831843	7.0707483	NM_138459	chr6:118028097-118028156	NUS1
A_23_P144639	6.95589	7.1796584	NM_018502	chr5:140024696-140024754	TMCO6
A_23_P25503	9.69383	9.275441	NM_001079673	chr13:49783462-49783521	FNDC3A

A_23_P49674	9.074507	8.672512	NM_173728	chr17:8224944-8225003	ARHGEF15
A_23_P378450	3.862328	3.387757	NM_144614	chr19:7051175-7051234	MBD3L2
A_33_P3256054	8.618396	8.34443	NM_022372	chr16:2259234-2259293	MLST8
A_23_P31747	9.392318	9.451744	NM_032847	chr8:124238789-124232528	C8orf76
A_33_P3370163	5.0203404	5.291043		chr15:030696848-030696789	
A_24_P398064	8.212493	8.158091	D87447	chr9:35757656-35757715	RGP1
A_33_P3276369	3.8165815	4.612163	NM_016371	chr1:162762509-162762568	HSD17B7
A_33_P3290955	7.0854564	7.4184885	NR_024207	chr8:145172632-145172680	KIAA1875
A_24_P253251	10.050291	9.65705	NM_003045	chr13:30084152-30084093	SLC7A1
A_23_P142345	4.0157146	4.2336187	NM_002777	chr19:847827-847886	PRTN3
A_32_P49867	4.3916926	4.240854	NM_001195520	chr12:133179847-133179788	LRCOL1
A_23_P20463	12.93904	12.822369	NM_006265	chr8:117858409-117858350	RAD21
A_23_P134953	7.9962783	8.408279	NM_001122	chr9:19116429-19116370	PLIN2
A_23_P154522	7.8836904	8.287753	NM_020744	chr2:42935109-42935168	MTA3
A_23_P3602	5.1418796	5.1438036	NM_001243657	chr16:77775921-77775980	NUDT7
A_33_P3424112	5.4042773	5.810209	NM_024612	chr17:57665354-57665413	DHX40
A_23_P155463	4.889369	4.9905977	NM_024512	chr3:46557445-46557386	LRRC2
A_32_P100430	6.0913506	6.0869665	XR_242328	chr7:107383512-107383453	
A_33_P3421571	8.702243	8.8864975	NM_213589	chr2:204304250-204304191	RAPH1
A_33_P3271945	5.2053676	4.7155333		chr9:19056989-19056930	HAUS6
A_23_P129118	7.5526357	7.5737195	NM_005707	chr15:65410501-65410442	PDCD7
A_23_P212213	7.248744	7.149357	NM_015453	chr3:9424904-9424963	THUMPD3
A_23_P137046	7.497777	7.708162	NM_022567	chrX:41334570-41334629	NYX
A_23_P54953	7.1022043	7.185267	NM_013260	chr17:73698564-73698623	SAP30BP
A_33_P3298043	5.1510715	5.2871056	NM_001079528	chr16:16315484-16315425	ABCC6
A_33_P3767773	11.314068	11.218395	NM_020765	chr1:19401072-19401013	UBR4
A_24_P415624	6.2317057	6.3352227	NM_014938	chr12:122618454-122618513	MLXIP
A_33_P3228190	4.688001	4.252627	NM_001199860	chr1:6142274-6142333	KCNAB2
A_33_P3258274	7.557701	7.813557	AB209866	chr2:188349577-188349518	TFPI
A_23_P67042	5.9103317	5.697415	NM_017947	chr18:33840112-33846744	MOCOS
A_23_P217088	9.88731	9.813455	NM_000476	chr9:130630712-130630653	AK1
A_32_P221958	9.950915	9.919792	NM_001077685	chr10:51464420-51464361	AGAP7
A_33_P3287223	8.685622	9.2442	NM_001935	chr2:162849045-162848986	DPP4
A_23_P3823	9.497883	9.816582	NM_001122957	chr16:31123668-31123727	BCKDK
A_33_P3407895	4.431709	4.8652554	NM_001195833	chr19:39359768-39359709	RINL
A_23_P387031	7.2565556	8.822646	NM_173465	chr5:177664864-177664805	COL23A1
A_23_P320242	2.3221061	2.3900566	NM_152748	chr7:86509592-86509533	KIAA1324L
A_33_P3299934	7.453931	7.2686586	NR_036581	chr17:10707105-10707046	LINC00675
A_33_P3436732	7.2484536	7.077627	NR_027695	chr21:39580495-39580554	DSCR10
A_33_P3306624	3.8356023	3.8427186	NM_001524	chr17:40337465-40337406	HCRT
A_23_P147296	11.332406	11.321251	NM_015700	chr2:69627514-69623382	NFU1
A_33_P3374903	4.894684	4.9370356		chr14:035704538-035704597	
A_33_P3278475	9.46782	8.968374	NM_002158	chr2:48606257-48606316	FOXN2
A_32_P183970	9.265098	9.787751	NM_001130448	chr15:41064286-41064345	C15orf62
A_33_P3250438	7.2548265	7.0838795	XM_005260074	chr19:16763364-16763305	SMIM7
A_23_P84922	4.804265	4.971315	NM_018486	chrX:71681900-71571676	HDAC8
A_23_P83579	7.2404776	7.2368402	NM_014862	chr15:80890050-80890109	ARNT2
A_23_P314760	8.802346	8.976288	NM_016203	chr7:151254256-151254197	PRKAG2
A_23_P356677	3.874026	3.750865	NM_001130690	chr6:165745021-165744962	PDE10A
A_23_P347610	8.277242	8.331448	NM_012206	chr5:156456630-156456571	HAVCR1
A_24_P47547	11.264172	11.471643	NM_006325	chr12:131357427-131357568	RAN
A_24_P160104	8.603679	8.608422	NM_018943	chr22:18613820-18613879	TUBA8
A_33_P3423401	2.69682	3.3800702	W87749	chr19:37308486-37308427	ZNF790
A_23_P376188	7.529879	7.6120987	NM_001100912	chr10:13483671-13483612	BEND7
A_23_P93641	7.1754704	5.992008	NM_020299	chr7:134225939-134225998	AKR1B10
A_33_P3294149	4.2208376	4.4450827	XM_005252686	chr10:135139755-135139696	CALY
A_33_P3260100	10.473253	10.493313	NM_138493	chr6:37450774-37450715	CCDC167
A_23_P18465	8.742505	8.716154	NM_002913	chr4:39290134-39290075	RFC1
A_23_P258190	12.920799	12.823801	NM_001628	chr7:134132097-134130061	AKR1B1

A_33_P3217213	5.609538	5.1682777	NM_005451	chr5:176915129-176915070	PDLIM7
A_24_P301846	7.125003	7.409274	NM_175085	chr21:34897171-34897112	GART
A_33_P3399755	8.566274	8.558071		chr15:43423226-43423167	EPB42
A_33_P3338275	4.964322	5.061861	NR_033579	chr15:82769340-82769399	CSPG4P8
A_33_P3211203	7.3092885	7.3475184	XR_245583	chr6:117994035-117993976	LOC101927919
A_32_P703	5.3836856	4.981118	NR_045484	chr1:85743396-85743455	LOC646626
A_33_P3280805	4.6304655	4.6623907	NM_015842	chr13:76423311-76423370	LMO7
A_23_P68087	10.775003	10.387767	NM_004044	chr2:216213877-216213936	ATIC
A_23_P375566	5.238506	5.2554526	NM_178509	chr17:53111534-53111593	STXBP4
A_33_P3636590	9.161217	9.374902	NM_003848	chr3:67425690-67425631	SUCLG2
A_23_P207811	8.668288	8.375235	NM_006451	chr5:43526482-43526423	PAIP1
A_33_P3691168	4.4657393	4.215723	NM_001242638	chr5:55195900-55195959	IL31RA
A_23_P357929	4.146162	4.096596	NM_015139	chr1:67474829-67474770	SLC35D1
A_23_P252711	9.4007225	9.312776	NM_016027	chr8:71550792-71550089	LACTB2
A_33_P3322288	8.341616	8.655212	NM_001134433	chr3:28378290-28378231	AZI2
A_33_P3256344	8.4562025	8.936651	AK128866	chr19:047913319-047913260	
A_23_P217712	5.5049148	5.7739253	NM_001669	chrX:2835852-2833682	ARSD
A_24_P252945	3.821354	3.8735876	NM_032966	chr11:118766856-118766915	CXCR5
A_23_P39561	8.064532	8.216555	NM_080678	chr2:238934042-238939244	UBE2F
A_33_P3415923	7.593876	7.391888	NM_175736	chr12:50038814-50038755	FMNL3
A_33_P3389286	6.0920196	5.4881616	NM_006142	chr1:27190888-27190947	SFN
A_33_P3287879	8.11927	8.287058	NM_003536	chr6:27778041-27778100	HIST1H3H
A_33_P3395028	6.4846783	5.8338084		chr3:101716576-101716635	LOC152225
A_33_P3226546	4.887761	4.907455		chrX:048634229-048634170	
A_23_P116942	3.9173965	4.3765726	NM_002286	chr12:6887081-6887462	LAG3
A_23_P62967	7.51388	7.541534	NM_018662	chr1:232176696-232176755	DISC1
A_33_P3295077	3.6455936	3.71692		chr2:177671821-177671762	
A_24_P341476	12.957378	12.496462		chr16:081509994-081509935	
A_33_P3332885	6.3119054	6.0597925	NM_001197234	chr6:26465550-26465609	BTN2A1
A_23_P204696	4.7477646	5.1990047	NM_004064	chr12:12871119-12871178	CDKN1B
A_23_P161624	8.833031	8.569053	NM_005438	chr11:65660157-65660098	FOSL1
A_32_P220472	9.995915	10.057416	NM_019006	chr15:80430454-80430513	ZFAND6
A_24_P277155	10.874678	10.930885	NM_003071	chr3:148748450-148748391	HLTF
A_33_P3213064	8.900556	9.1260805	NM_005419	chr12:56735443-56735384	STAT2
A_33_P3211734	9.149721	9.056101	NM_001102396	chr1:115316822-115316763	SIKE1
A_23_P63219	9.857082	9.921019	NM_015100	chr1:151377075-151377016	POGZ
A_23_P254212	6.020606	5.8351407	NM_013347	chrX:96140272-96140331	RPA4
A_24_P940666	6.5873065	6.147024	NM_004774	chr17:37561073-37561014	MED1
A_23_P59202	7.5822544	7.995145	NM_005643	chr6:34846146-34846087	TAF11
A_24_P687582	5.6086283	6.0086436	NM_152293	chr4:7058918-7058977	TADA2B
A_23_P258018	4.9587545	4.9747534	NM_002477	chr4:673771-674320	MYL5
A_33_P3231923	9.559931	9.152817	XR_249135	chr2:59506453-59506512	
A_23_P63289	10.539213	10.808954	NM_014188	chr1:1477388-1477329	SSU72
A_23_P2492	2.3221061	2.3900566	NM_001734	chr12:7178093-7178152	C1S
A_24_P212072	8.299614	8.119932	NM_032290	chr5:93966300-93966359	ANKRD32
A_33_P3320152	10.928947	10.761893	NM_003094	chr1:203839049-203839108	SNRPE
A_33_P3382835	5.5453606	6.085961	NM_025081	chr14:24878368-24878427	NYNRIN
A_23_P500886	3.9786258	4.0350027	NM_014343	chr7:100880881-100880822	CLDN15
A_33_P3351615	11.824636	11.876175		chr11:129407893-129407952	
A_23_P22660	5.7527776	5.6328273	NM_006639	chrX:77528764-77528705	CYSLTR1
A_33_P3387781	5.0060673	4.729604	NR_002145	chr1:248154197-248154256	OR2L1P
A_33_P3260175	4.405984	4.582046	BC036435	chr1:16860561-16860502	
A_24_P382119	10.029007	9.784252	NM_004687	chr17:56566975-56566916	MTMR4
A_23_P8848	8.931357	8.873998	NM_018250	chr8:28625314-28625255	INTS9
A_23_P165186	9.059372	9.399107	NM_019104	chr19:36245347-36245406	LIN37
A_33_P3391418	3.7262204	3.74966	NM_001284407	chr3:12790258-12790199	TMEM40
A_23_P77073	8.501423	8.833031	NM_032802	chr15:51014368-51012286	SPPL2A
A_33_P3217649	5.9436197	6.6736746	NM_001048265	chr9:138387457-138387398	C9orf116
A_33_P3400578	5.01703	5.3879523	NM_002126	chr17:53402334-53402393	HLF

A_23_P138461	5.687112	5.5208464	NM_021830	chr10:102753417-102753476	C10orf2
A_24_P276628	6.352267	7.112405	NM_000310	chr1:40555149-40555090	PPT1
A_33_P3283064	5.3498073	5.682889		chr22:021053312-021053371	
A_24_P42136	9.015271	8.577265	NM_000224	chr12:53345577-53345636	KRT18
A_23_P14072	8.6958275	8.519578	NM_002273	chr12:53291085-53291026	KRT8
A_32_P72181	4.4253607	4.5257616	NR_024391	chr18:36787669-36787610	LINC00669
A_23_P18490	8.130568	8.266493	NM_001017405	chr4:1333117-1333176	MAEA
A_33_P3350452	9.456008	9.540005		chr16:016025979-016025920	
A_23_P91702	11.29068	11.257545	NM_003753	chr22:36907649-36907590	EIF3D
A_24_P348989	6.7524714	7.203844	NM_006863	chr19:55111992-55112199	LILRA1
A_33_P3258782	7.673251	7.913951	NM_003916	chrX:15863562-15863503	AP1S2
A_24_P94054	7.4354553	7.4987526	NM_006282	chr20:43615912-43623764	STK4
A_24_P40907	6.242969	6.2403355	NM_203453	chr9:4665048-4665107	PPAPDC2
A_24_P945147	8.7430935	8.557239	NM_004703	chr17:5288797-5288856	RABEP1
A_24_P202558	6.330321	6.144579	NM_015073	chr19:38698769-38698828	SIPA1L3
A_33_P3395146	8.0739155	7.684828		chr7:29685597-29685538	LOC646762
A_23_P64019	10.266235	10.112625	NM_201278	chr11:95567472-95567413	MTMR2
A_23_P15348	9.4609	9.43152	NM_015134	chr17:17088385-17088444	MPRIP
A_33_P3413927	6.618781	7.1163282	NM_017599	chr12:95688085-95688144	VEZT
A_23_P78248	2.3221061	2.3900566	NM_015515	chr17:39079021-39078962	KRT23
A_33_P3220090	3.6818862	3.4309635	NM_001076781	chr6:27369168-27369227	ZNF391
A_23_P356646	6.071889	6.1638346	NR_038327	chr21:9908217-9908158	TEKT4P2
A_23_P117971	13.841615	13.907053	M15530	chr16:58588342-58588401	
A_33_P3408305	5.909088	6.3753843	NM_178842	chr15:100942805-100942746	CERS3
A_32_P20691	9.986378	9.877642	NM_003992	chr15:74922387-74922446	CLK3
A_24_P6903	7.7348027	7.898643	NM_001017992	chr5:56778051-56777992	ACTBL2
A_23_P151634	8.409629	8.263135	NM_007192	chr14:21820393-21820334	SUPT16H
A_33_P3846177	10.315827	10.060962	NM_001478	chr12:58020014-58019955	B4GALNT1
A_24_P323114	7.4024096	7.7796545	NR_001446	chr10:66585482-66585541	ANXA2P3
A_24_P336957	7.8231206	7.8674846	NM_145074	chr2:74758790-74759002	HTRA2
A_24_P306443	3.5349226	3.5926955	NR_037871	chrUn_gl000218:40670-39998	LOC100233156
A_24_P322369	9.315119	9.666323	NM_004869	chr18:61056939-61056880	VPS4B
A_33_P3326682	7.1504865	6.2175555	NM_003001	chr1:161334471-161334530	SDHC
A_32_P919718	4.9401546	4.8351874	NM_178520	chr17:79285139-79285080	TMEM105
A_23_P350551	9.988789	9.8712635	NM_138425	chr12:7055087-7055146	C12orf57
A_24_P24724	8.380923	8.695301		chrX:000970756-000970697	
A_33_P3529860	4.2755194	3.6267188	NM_001005366	chr12:121868234-121868175	KDM2B
A_33_P3647427	6.7888527	6.7568	X52357	chr17:15578835-15578894	ZNF29P
A_23_P214681	3.8395944	3.682104	NM_006238	chr6:35391843-35391902	PPARD
A_33_P3381796	4.4991693	5.002277		chr7:157233772-157233713	
A_24_P927189	4.1871877	3.4024107	NM_138381	chr3:16347385-16347444	OXNAD1
A_33_P3390576	4.1610007	4.710769	NM_001197104	chr11:118362577-118362636	KMT2A
A_33_P3381410	6.6014023	6.441928	NM_006190	chr2:201822244-201822185	ORC2
A_24_P43876	9.283689	9.013919	NM_017998	chr9:77562854-77562795	C9orf40
A_23_P140256	11.493077	11.180854	NM_000270	chr14:20945104-20945163	PNP
A_23_P430201	6.6577177	6.8048954	NM_152446	chr14:80963137-80963078	CEP128
A_33_P3353051	8.897953	8.750048	NM_001040438	chr6:31802884-31802943	C6orf48
A_24_P63030	6.9081197	7.318993	NM_144579	chr2:73169517-73169458	SFXN5
A_33_P3412716	11.796828	11.668272		chr5:055432895-055432954	
A_33_P3271530	4.3894763	3.4241507	NM_024989	chr2:197757965-197757906	PGAP1
A_33_P3371564	5.8602753	5.822172	NR_024361	chr8:8093761-8093820	FAM86B3P
A_33_P3248982	3.9316297	4.235714	NM_012306	chr12:50260739-50260680	FAIM2
A_23_P79927	9.11187	8.802346	NM_006392	chr20:2636023-2636082	NOP56
A_24_P322354	7.6030097	7.160764	NM_001039535	chr18:47919899-47919958	SKA1
A_33_P3259443	5.346734	4.8481255	XR_109175	chr15:76079262-76079321	LOC441728
A_23_P145569	4.463939	4.4775715	NM_153187	chr6:160560885-160564617	SLC22A1
A_23_P14716	8.146511	7.6786447	NM_031284	chr15:73044246-73044187	ADPGK
A_33_P3329974	7.0073843	7.1520195	NM_020770	chr1:151511070-151511129	CGN
A_33_P3257808	8.55826	8.222785	NM_001018115	chr3:10114581-10114640	FANCD2

A_32_P83845	4.8608847	4.0541115	NM_001040708	chr8:80676371-80676312	HEY1
A_23_P94030	11.588425	11.517313	NM_002291	chr7:107564470-107564411	LAMB1
A_23_P141044	8.91022	9.01088	NM_145271	chr16:30581086-30581027	ZNF688
A_24_P31003	6.440217	6.0362897	NM_005262	chr16:2036685-2036744	GFER
A_33_P3285235	4.380305	3.8015695	NM_001136156	chr19:32878432-32878491	ZNF507
A_33_P3314441	4.769288	4.788579	NM_001163315	chr5:107195628-107195569	FBXL17
A_23_P134274	11.272586	10.968346	NM_005837	chr7:100305030-100305089	POP7
A_23_P145096	2.3221061	2.3900566	NM_005084	chr6:46672900-46672385	PLA2G7
A_23_P402610	5.519458	4.831756	NM_012393	chr17:8173037-8173096	PFAS
A_33_P3290082	4.1890616	4.575146	NM_022081	chr22:26860445-26860386	HPS4
A_33_P3215166	4.7307496	4.695652		chr13:062902180-062902239	
A_23_P303317	6.286987	5.965232	NM_025112	chr3:126157049-126156990	ZXDC
A_33_P3236986	5.5043573	5.618151	XR_171867	chr10:104594200-104594259	CYP17A1-AS1
A_23_P155487	5.6270413	5.6445413	NM_006841	chr3:50257592-50257651	SLC38A3
A_33_P3239849	11.549722	11.519245	NM_201397	chr3:49394984-49394925	GPX1
A_32_P41496	4.7290406	4.698636		chrX:40691339-40691280	LOC100132831
A_33_P3242688	5.473322	5.4071894		chr6:031334193-031334134	
A_23_P251785	8.251534	8.275219	NM_024561	chr13:41951067-41951126	NAA16
A_23_P4522	12.216221	12.14043	NM_004786	chr18:54270203-54270144	TXNL1
A_33_P3362668	7.8091354	7.6280293	NR_034006	chr9:68726645-68726704	LOC100132352
A_23_P66525	8.622639	8.404172	NM_006042	chr17:13399309-13399250	HS3ST3A1
A_23_P3186	7.575342	7.615242	NM_001099402	chr14:99977194-99977253	CCNK
A_33_P3322879	3.0449076	3.3781178	BU182564	chr7:29238730-29238671	
A_24_P328872	6.8533173	6.6927137	NM_012073	chr5:10265930-10265989	CCT5
A_23_P334709	11.279803	11.22479	NM_007270	chr7:33046439-33046498	FKBP9
A_33_P3361422	2.3221061	2.3900566	NM_000784	chr2:219679951-219680010	CYP27A1
A_24_P337058	8.313209	8.483959	NM_020307	chr3:156867120-156866364	CCNL1
A_33_P3257155	3.2924128	3.4574878	XM_005248760	chr6:71547513-71547572	SMAP1
A_33_P3235690	7.9723015	8.200577	NM_001081550	chrX:122748019-122747960	THOC2
A_23_P129188	7.957343	8.165924	NM_033429	chr15:68486419-68486360	CALML4
A_33_P3522511	4.402587	4.098418	AB007954	chr1:61596906-61596965	KIAA0485
A_33_P3379841	5.1948266	5.7175436	NR_026928	chr13:33078702-33078643	N4BP2L2-IT2
A_33_P3419419	4.931391	5.336041		chr1:204435959-204435900	PIK3C2B
A_23_P3527	9.04659	8.960728	NM_032940	chr16:57505350-57505409	POLR2C
A_23_P401675	3.6069074	4.011421	AK055094	chr5:68739750-68739809	MARVELD2
A_33_P3370714	4.2590213	4.0929627	NM_001166424	chr6:41712528-41712469	PGC
A_33_P3343715	5.0115423	5.3268948	BC037242	chr2:73144109-73144168	EMX1
A_33_P3665812	5.6836963	5.7412233	AF090921	chr9:6467718-6467659	
A_33_P3398077	4.0368276	4.2054768	NR_038275	chr10:51827341-51827282	FLJ31813
A_23_P169117	11.660463	11.801372	NM_006570	chr9:19050839-19050898	RRAGA
A_33_P3291118	5.622317	5.571309	NM_052888	chr17:30354798-30354857	LRRC37B
A_33_P3409854	9.756641	9.710463	NM_005568	chr17:35300424-35300483	LHX1
A_33_P3833211	7.693483	7.695752	NM_001080449	chr10:70174805-70174746	DNA2
A_23_P70448	3.0051126	2.3900566	NM_005325	chr6:26017410-26017351	HIST1H1A
A_33_P3316379	12.252043	12.295363		chr1:239216043-239215984	
A_24_P169544	6.242008	6.352879	NM_006959	chr19:57932871-57932930	ZNF17
A_33_P3215906	7.208794	7.351565	NM_001037675	chr1:144620034-144620093	NBPF9
A_33_P3278313	9.599537	9.76734	NM_001193460	chr12:65860358-65860417	MSRB3
A_32_P76853	6.9404697	7.418249	NM_001282484	chr15:30694762-30694703	LOC101059918
A_33_P3380693	12.340577	12.311659	NM_207197	chr1:155035192-155035251	ADAM15
A_23_P50137	8.550634	7.9234524	NM_016626	chr18:48701328-48701269	MEX3C
A_33_P3276062	2.346319	2.3900566		chr4:001139755-001139814	
A_33_P3241596	14.156184	14.197328		chrX:003819762-003819703	
A_33_P3319572	5.9839773	6.3746076	NM_001145402	chr19:55873761-55873702	FAM71E2
A_33_P3263664	4.724252	4.813049	NM_181604	chr21:31971126-31971067	KRTAP6-2
A_23_P147605	6.8903165	6.6954584	NM_001003676	chr11:47183448-47183507	C11orf49
A_23_P202587	7.3393126	7.0022535	NM_018330	chr10:118644665-118644606	KIAA1598
A_33_P3306964	8.95796	8.295721	NM_006241	chr3:195250527-195245961	PPP1R2
A_24_P68247	5.877807	5.363632	NM_033017	chr7:99500938-99500879	TRIM4

A_33_P3231572	8.3157	8.563757	AK095012	chr7:1089796-1089855	LOC100130456
A_23_P215051	10.744663	10.733592	NM_018479	chr6:127609954-127609895	ECHDC1
A_33_P3259775	5.7565885	5.6348267	NM_024940	chr8:25270542-25270601	DOCK5
A_24_P366082	7.3526626	6.9813776	NM_015241	chr22:18270952-18270893	MICAL3
A_33_P3361067	4.0913267	3.4299378	NM_004827	chr4:89011562-89011503	ABCG2
A_32_P196854	5.0441837	4.8017397	NM_001143912	chr1:28089083-28089142	FAM76A
A_33_P3518572	7.6811924	7.521411		chr12:30955519-30955578	LINC00941
A_23_P46673	6.455894	6.9864464	DM107643	chr1:176585564-176585505	
A_23_P87827	9.125319	9.396799	NM_001080533	chr12:121161131-121161190	UNC119B
A_23_P259797	5.248654	5.187837	NM_174917	chr16:89199636-89211699	ACSF3
A_24_P360078	8.264049	8.607964	NM_006726	chr4:151186250-151186191	LRBA
A_33_P3354574	3.394719	4.4186783		chr13:44978597-44978538	
A_33_P3303865	5.9034076	6.3307905	NM_198456	chrX:54208993-54208934	FAM120C
A_33_P3419945	8.512772	8.122292	AK130724	chr15:75836950-75836891	
A_24_P126651	8.331448	8.610489	NM_006756	chr8:54900799-54900740	TCEA1
A_23_P49842	5.989883	6.1826515	NM_005148	chr17:26874864-26874805	UNC119
A_24_P219920	6.208538	5.9341984	NM_018708	chr19:4794783-4794842	FEM1A
A_24_P61490	2.3221061	2.3900566	NM_003317	chr14:36985777-36985718	NKX2-1
A_32_P138004	8.334235	8.3961315	NM_207009	chr10:120896964-120897021	FAM45A
A_33_P3221177	6.8426895	7.0879364	NM_153251	chr13:21949276-21949217	ZDHHC20
A_33_P3807062	12.444774	12.025342	NM_018410	chr2:234746088-234746029	HJURP
A_23_P12733	6.134747	5.93692	NM_018649	chr10:71871745-71871804	H2AFY2
A_32_P109683	2.758007	3.164705	NM_001015038	chrX:55102473-55102532	PAGE2B
A_23_P159907	7.8934636	8.292873	NM_030801	chrX:51934568-51934627	MAGED4B
A_33_P3345743	6.08494	5.7465434	NR_003242	chr1:144611135-144611076	PFN1P2
A_23_P146811	7.6810412	7.8403835	NM_030793	chr5:147821610-147821669	FBXO38
A_24_P134319	8.683346	8.653526	NM_001282531	chr20:49506012-49505953	ADNP
A_24_P272061	13.530484	13.59614	NM_012423	chr10:112696699-112696640	RPL13A
A_33_P3385561	14.070338	14.129131	AK130932	chr6:17878744-17878685	
A_33_P3239143	6.632247	6.5866413	NM_198458	chr19:58867813-58867754	ZNF497
A_33_P3390057	13.506524	13.427124	NM_014220	chr3:149086869-149086810	TM4SF1
A_23_P8311	10.631479	10.355734	NM_016614	chr6:24650393-24650334	TDP2
A_33_P3228252	7.6209455	7.7536383	DA666023	chr3:192463008-192463067	
A_33_P3234809	10.397855	10.220207	NM_003466	chr2:113973634-113973575	PAX8
A_23_P129476	6.6242967	6.7648067	NM_004913	chr16:89773869-89773810	VPS9D1
A_33_P3565787	5.0193005	4.659814	NR_027329	chr7:1626121-1626180	PSMG3-AS1
A_23_P424080	8.105259	8.42573	NM_032312	chr2:32530941-32531000	YIPF4
A_33_P3269428	5.031308	5.230797	XM_005261327	chr22:37578842-37578783	C1QTNF6
A_23_P126752	9.283915	9.533902	NM_004930	chr1:19670902-19666103	CAPZB
A_23_P50504	13.17179	13.259013	NM_000146	chr19:49468731-49468790	FTL
A_24_P20777	14.403733	14.618314	NM_000997	chr5:40834588-40834324	RPL37
A_33_P3220242	6.323622	6.0672603	NM_001167575	chr9:26840744-26840685	CAAP1
A_23_P15705	12.735723	12.802589	NM_002798	chr17:4701404-4701589	PSMB6
A_23_P54055	10.794189	10.320704	NM_032876	chr14:23441306-23441247	AJUBA
A_24_P918384	4.1757803	4.039819	NM_022455	chr5:176726576-176726635	NSD1
A_23_P99614	10.843962	11.015629	NM_033271	chr14:105717302-105717361	BTBD6
A_33_P3382100	8.584035	8.015957	NM_001164586	chr1:201197610-201197669	IGFN1
A_24_P123347	8.074123	7.640828	NM_002703	chr4:57260042-57259983	PPAT
A_33_P3429575	10.504001	10.9184675	BC157883	chr1:214656126-214656067	LOC643454
A_23_P127579	10.46669	10.419078	NM_000317	chr11:112104258-112104317	PTS
A_23_P169576	7.763225	7.7074966	NM_019053	chr10:94819106-94819165	EXOC6
A_33_P3465703	4.372172	4.422464	BF304636		SNORA60
A_33_P3330549	4.3979855	4.735733	NM_020428	chr19:10753067-10753126	SLC44A2
A_23_P343594	6.489832	5.9974184	NM_018218	chr2:234385112-234385053	USP40
A_23_P212639	11.802288	11.621851	NM_004593	chr3:185635336-185635277	TRA2B
A_23_P422981	5.095834	5.185197	NM_174899	chr2:230877565-230877624	FBXO36
A_33_P3348469	5.65579	5.362182	NM_030816	chr1:70724745-70724686	ANKRD13C
A_33_P3235706	5.5969405	5.814952	BC048301	chr1:52980792-52980733	ZCCHC11
A_23_P118038	8.375935	8.450116	NM_005796	chr16:67904758-67904817	NUTF2

A_24_P5750	5.921506	6.025563	NM_005551	chr19:51380007-51380179	KLK2
A_24_P150874	8.497133	8.503965	NM_006572	chr17:63005770-63005711	GNA13
A_24_P74896	4.6181345	5.062626	EF107718	chr9:127228630-127228689	GPR144
A_23_P389692	4.313219	4.6989484	NM_020776	chr18:34804890-34804949	KIAA1328
A_33_P3311543	4.0538416	4.2140627	AF130086	chr15:67899741-67899800	
A_23_P154526	8.988998	9.089701	NM_004490	chr2:165349598-165349539	GRB14
A_23_P255876	3.7572494	3.8690088	NM_012144	chr9:34517408-34520657	DNAI1
A_23_P501887	7.158974	6.7320857	NM_013406	chr19:12790482-12790344	DHPS
A_24_P406132	7.597224	7.184255	NM_002754	chr6:36107315-36107374	MAPK13
A_23_P59099	8.031301	7.5541234	NM_013937	chr6:29394698-29394639	OR11A1
A_32_P158746	13.413709	13.3608	NM_000985	chr18:47016870-47015908	RPL17
A_33_P3373560	4.420047	4.574436	NM_001130438	chr9:131388906-131388965	SPTAN1
A_33_P3539345	8.048493	8.12601	NM_004999	chr6:76624789-76624848	MYO6
A_33_P3349651	8.51861	8.437703	NM_015055	chr11:9774221-9774280	SWAP70
A_33_P3381943	6.751296	6.7524714	NR_024549	chr7:86811421-86811480	DMTF1
A_24_P56837	5.52396	5.829169	NM_080863	chr17:42256390-42256449	ASB16
A_23_P58353	13.065256	12.992632	NM_031370	chr4:83275239-83275180	HNRNPD
A_24_P9321	6.8080263	6.6118994	NM_003533	chr6:27839916-27839857	HIST1H3I
A_23_P30315	9.335954	9.1714	NM_033342	chr5:180630267-180630208	TRIM7
A_33_P3418668	6.8872147	6.517535	NR_022011	chr15:25228582-25228641	PWARSN
A_33_P3605352	4.6889343	4.5673327	BC066916	chr3:149419549-149419490	WWTR1
A_24_P294982	6.7989554	6.894033	NM_016485	chr6:142541402-142541461	VTA1
A_33_P3415052	2.3221061	2.3900566	NM_001172292	chr5:156901670-156901729	NIPAL4
A_23_P137814	9.662048	9.349108	NM_004047	chr1:44443706-44443765	ATP6V0B
A_33_P3289356	9.142797	9.215125	NM_001779	chr1:117064585-117061888	CD58
A_23_P423331	4.5951185	3.6726906	NM_032536	chr9:135118106-135118165	NTNG2
A_23_P340263	2.3221061	2.3900566	NM_173662	chr4:154636721-154633710	RNF175
A_23_P73632	2.3221061	2.3900566	NM_000475	chrX:30322698-30322639	NR0B1
A_23_P144490	4.450732	4.724004	NM_017639	chr4:155156009-155155950	DCHS2
A_33_P3433156	6.2039433	5.8276167	NM_001282785	chr22:46748167-46748226	TRMU
A_33_P3390177	4.5358963	4.5165567	NR_033929	chr3:194209094-194209153	LINC00884
A_23_P311640	4.893116	4.4815574	NM_006076	chr7:100163205-100163264	AGFG2
A_23_P70359	7.0806174	6.9311476	NR_024277	chr6:161581396-161581337	AGPAT4-IT1
A_23_P84140	8.53804	7.7648993	NM_001023571	chr3:121488976-121488917	IQCB1
A_23_P367676	8.107004	8.531353	NM_015477	chr15:75663644-75663585	SIN3A
A_33_P3360204	5.8964834	5.5514317		chr19:20349157-20349098	
A_33_P3213997	9.842416	9.786617	NM_001193636	chr14:24475557-24475616	DHRS4L2
A_24_P139094	11.028464	11.14019	NM_003025	chr19:4360629-4360570	SH3GL1
A_24_P66679	6.270206	6.706329	NM_001011713	chr14:57877572-57877631	NAA30
A_33_P3268507	4.8086805	4.779742	NM_001184816	chr19:43025514-43025455	CEACAM1
A_24_P55250	9.726696	9.7093	NM_016063	chr6:125598339-125598280	HDDC2
A_32_P144920	5.516529	6.352037	NM_015509	chr12:8249373-8249433	NECAP1
A_33_P3709525	3.7843618	3.2739313		chr14:65679837-65679778	LOC100128233
A_33_P3253501	9.594027	9.661405	NM_001024599	chr1:149783617-149783558	HIST2H2BF
A_32_P129419	4.262717	4.0659504	NM_024611	chr15:60712848-60712789	NARG2
A_33_P3728698	7.4349627	7.059093		chr8:103822140-103822199	FLJ45248
A_33_P3412767	4.7887297	4.7575493	NM_032184	chr1:85623512-85623453	SYDE2
A_24_P315921	5.5803595	5.388662	AK093659	chr1:39989249-39988134	PPIEL
A_33_P3307495	9.031595	8.709057	NM_001199042	chr15:74471871-74471812	STRA6
A_24_P93754	4.9956822	5.1277637	NM_001146694	chr9:7170372-7170431	KDM4C
A_33_P3267280	2.9623017	3.158652	NM_005730	chr12:58217464-58217405	CTDSP2
A_33_P3405360	6.308099	6.4431863	NM_005388	chr9:125585359-125585300	PDCL
A_23_P207400	8.286522	8.094112	NM_007300	chr17:41196481-41196422	BRCA1
A_23_P21316	5.0786376	5.5185995	NM_021222	chr1:151007111-151007170	PRUNE
A_33_P3422991	8.124513	8.479977	NM_001286139	chr11:92887386-92887327	SLC36A4
A_32_P41526	4.3255787	4.417987	NM_001077685	chr10:51465313-51465254	AGAP7
A_23_P92424	10.619726	9.877373	NM_020368	chr4:71555737-71555796	UTP3
A_23_P13701	8.5632105	9.018806	NM_016056	chr12:66563649-66547184	TMBIM4
A_33_P3277611	7.6133337	7.893499	NM_001080483	chr9:136379871-136379812	TMEM8C

A_33_P3243128	7.9785275	7.827084	NM_032314	chr12:120941414-120941355	COQ5
A_23_P92687	8.392736	8.059801	NM_004394	chr5:10680153-10680094	DAP
A_33_P3393537	7.5497885	7.6983104	NM_001164722	chr1:28520418-28520359	PTAFR
A_33_P3370881	5.942605	6.3319597	XM_005259448	chr19:42413272-42413213	
A_33_P3307163	5.147558	5.275695	NM_001134363	chr10:112540744-112540803	RBM20
A_33_P3360555	10.7189455	10.780368	NM_001253792	chr19:56672202-56672261	ZNF444
A_24_P598406	9.050338	9.370357	NR_023384	chr7:5037654-5037713	RNF216P1
A_24_P148026	3.7052827	2.3900566	XM_003118494	chr1:2113581-2113522	
A_24_P98277	7.3195343	7.3564095	NM_001002296	chr8:41355117-41355176	GOLGA7
A_24_P284324	7.7695527	7.5238204	NM_017728	chr17:72835637-72835696	TMEM104
A_24_P40551	8.861208	9.192389	NM_001080425	chrX:102471282-102471341	BEX4
A_32_P2392	6.033378	6.59822	NM_181077	chr15:34673393-34673334	GOLGA8A
A_33_P3209351	11.168955	11.453451	NM_013417	chr9:94991342-94991283	IARS
A_33_P3423365	9.51797	9.979653	NM_001127663	chr9:124093667-124093726	GSN
A_33_P3244863	4.417831	4.939454	NM_014810	chr1:179961297-179961356	CEP350
A_33_P3374643	8.637051	8.663937	NM_001267803	chr11:73638711-73638770	PAAF1
A_23_P374389	8.80377	9.076081	NM_138499	chr10:134231014-134231073	PWWP2B
A_23_P69100	6.0701985	6.2374167	NM_015595	chr3:153973770-153973829	ARHGEF26
A_23_P142918	8.408651	8.165663	NM_153689	chr2:200791629-200791688	C2orf69
A_24_P212234	4.993625	5.0482645	NM_182632	chr5:1244392-1244451	SLC6A18
A_24_P674924	7.8244104	7.626023	NM_133259	chr2:44113851-44113792	LRPPRC
A_33_P3341424	7.7865067	7.164803	NM_012334	chr5:16662078-16662019	MYO10
A_23_P40315	6.68559	7.0509534	NM_001099407	chr20:18364192-18364133	DZANK1
A_33_P3348802	6.612828	6.444034	BC044750	chr2:206951157-206951216	
A_24_P391568	6.4185367	5.7248383	NM_001668	chr1:150783103-150783044	ARNT
A_23_P218706	5.8920527	6.0518484	NM_024325	chr20:2462907-2462848	ZNF343
A_24_P83922	7.7622375	7.6553907	NM_003093	chr6:34730394-34730453	SNRPC
A_24_P63347	2.3221061	2.3900566	NM_002620	chr4:74720137-74720196	PF4V1
A_33_P3355821	3.5381465	4.41903	NM_021148	chr7:64391729-64391788	ZNF273
A_33_P3308456	6.414462	6.6619644	NM_001282275	chr17:46800593-46800652	PRAC2
A_33_P3266839	3.1664367	4.666588	NM_001145720	chr1:32950872-32950931	ZBTB8B
A_24_P79403	2.6670783	3.4635155	NM_002619	chr4:74846974-74846915	PF4
A_33_P3366221	6.6965504	6.809285	NM_014917	chr1:107867420-107867479	NTNG1
A_23_P53567	9.265607	9.251625	NM_016053	chr12:102406877-102406818	CCDC53
A_33_P3364338	4.9995174	5.1128626		chr3:103782353-103782412	
A_23_P19115	6.43248	6.8449306	NM_018094	chrX:51488586-51488645	GSPT2
A_32_P113584	6.1517434	6.58377	NM_015021	chr6:87973262-87973321	ZNF292
A_33_P3357843	8.263135	8.254156	NM_000232	chr4:52887094-52887035	SGCB
A_33_P3376075	4.630019	4.7038026		chr11:045687675-045687734	
A_33_P3348362	4.3381405	4.581627	NR_027263	chr14:32545243-32545184	ARHGAP5-AS1
A_33_P3415895	6.781881	6.955502	NM_030759	chr10:64913773-64913832	NRBF2
A_23_P88069	9.2574215	7.9376965	NM_005780	chr13:39917176-39917117	LHFP
A_24_P268662	5.070076	5.649888	NM_018394	chr3:111711242-111711301	ABHD10
A_33_P3289456	5.6350527	5.429538		chr2:105761823-105761882	
A_23_P428729	2.3221061	2.3900566	BC007070	chr1:35484448-35484389	ZMYM6
A_33_P3367102	10.454755	10.346785	NR_003242	chr1:144612363-144612304	PFN1P2
A_33_P3399090	7.7313995	7.3029737	NM_001037954	chr11:111893216-111893275	DIXDC1
A_23_P111753	11.761814	11.611178	NM_031903	chr7:42977128-42977187	MRPL32
A_23_P205697	9.155592	8.9118	NM_001933	chr14:75370102-75370161	DLST
A_23_P159956	5.6368937	5.9756556	NM_012216	chrX:107170282-107170341	MID2
A_33_P3378212	11.599654	11.549722	NM_030973	chr19:50340173-50340232	MED25
A_24_P20873	8.186926	8.006344	NM_003495	chr6:27107225-27107284	HIST1H4I
A_33_P3263890	3.1061287	3.3983507	NM_006902	chr1:170708348-170708407	PRRX1
A_32_P173662	3.5858154	2.9982343	NM_003296	chr6:49665601-49663607	CRISP2
A_23_P502797	8.496407	8.903197	NM_020830	chr2:224740990-224740931	WDFY1
A_32_P146659	5.1784678	4.4671946	NR_027040	chr7:149564956-149564897	ATP6V0E2-AS1
A_24_P246692	13.058784	13.036181		chr7:135345181-135345240	XLOC_014512
A_24_P394533	8.483505	8.993664	NM_000434	chr6:31827205-31827146	NEU1
A_24_P293120	7.520683	7.2914166	NM_024327	chr19:44103327-44103386	ZNF576

A_33_P3390873	14.317977	14.374565		chr19:013005577-013005636	
A_33_P3284646	10.067884	10.095954	NM_012162	chr8:145579156-145579097	FBXL6
A_33_P3370060	5.3044133	5.1581755	NM_001199417	chr17:36634055-36634115	ARHGAP23
A_33_P3343605	11.107742	11.101643		chr13:021668494-021668435	
A_23_P86917	11.25917	10.962843	NM_003824	chr11:70053305-70053364	FADD
A_23_P128930	12.103256	11.731224	NM_002806	chr14:53187845-53187904	PSMC6
A_24_P327499	5.557025	5.4414306	NM_014657	chr20:36631035-36627677	TTI1
A_23_P57370	9.051036	8.983018	NM_033070	chr22:17618759-17618700	CECR5
A_23_P341349	8.80174	8.600046	NM_023083	chr2:241538451-241538510	CAPN10
A_23_P171143	7.715541	7.9358225	NM_003270	chrX:99884342-99884283	TSPAN6
A_24_P126628	8.081785	8.299764	NM_015257	chr12:57450095-57450036	TMEM194A
A_23_P133123	10.00566	9.792476	NM_032117	chr4:154335985-154336044	MND1
A_23_P76901	8.477103	8.31382	NM_015549	chr14:65210966-65211025	PLEKHG3
A_33_P3331588	10.2655735	9.977053	NM_031921	chr1:1431507-1431566	ATAD3B
A_33_P3376080	6.9204493	7.0075693	NM_001004451	chr9:125239326-125239267	OR1J1
A_24_P416370	4.3063974	4.24719	NM_024015	chr17:46653191-46653132	HOXB4
A_33_P3334791	6.6274357	6.8422556	NM_001256608	chr1:156264272-156264213	C1orf85
A_23_P115046	6.8027005	6.644926	NM_020365	chr1:45444070-45444011	EIF2B3
A_23_P372255	7.093408	6.8090816	NM_002221	chr1:226819646-226819587	ITPKB
A_23_P433111	7.6788087	7.4447823	NM_153706	chr5:56209808-56210738	SETD9
A_23_P344578	6.559972	6.539898	NM_153707	chr9:18927974-18927915	FAM154A
A_23_P156180	6.654912	6.4364495	NM_003059	chr5:131679470-131679529	SLC22A4
A_23_P59294	7.798022	8.109358	NM_001003699	chr6:7247437-7248785	RREB1
A_33_P3380883	4.946322	4.907116	NR_027302	chr9:100444650-100444591	XPA
A_32_P222961	7.0801945	6.4000835	NM_001012968	chrX:62567175-62567116	SPIN4
A_23_P119102	11.431446	11.146063	NM_003370	chr19:46029738-46029797	VASP
A_23_P22263	9.172905	9.019805	NM_079837	chr16:88110663-88110722	BANP
A_33_P3223488	3.6394033	3.7056785	NM_023037	chr13:32691537-32691596	FRY
A_33_P3405022	4.843958	5.1940513	NM_012318	chr14:045600828-045600769	LETM1
A_23_P3204	10.198149	10.2194	NM_002748	chr15:52358055-52358114	MAPK6
A_23_P205913	5.1355166	4.947069	NM_004727	chr15:65947615-65947674	SLC24A1
A_23_P420293	5.532972	4.768109	NM_145013	chr11:128769709-128769650	C11orf45
A_33_P3388491	16.704073	16.704073	NM_001040125	chr1:19655697-19655756	PQLC2
A_23_P15394	4.260907	4.144621	NM_001251	chr17:7484879-7484938	CD68
A_33_P3285260	7.8033824	7.9121504	XM_005261184	chr21:45561240-45561299	C21orf33
A_23_P142310	11.807949	11.993935	NM_017572	chr19:2037572-2037513	MKNK2
A_33_P3343206	5.890881	6.1155577	XM_005250748	chr7:63210139-63210198	LOC100996414
A_33_P3278144	12.095762	12.183184	XM_005273972	chr11:82924123-82924181	ANKRD42
A_24_P375227	4.256274	4.084467			
A_23_P159406	4.491526	4.455549	NM_003125	chr1:153005304-153005363	SPRR1B
A_24_P127928	10.470321	10.432138	NM_012414	chr1:220323885-220323826	RAB3GAP2
A_33_P3281033	6.3753843	6.374166	BC035876	chr20:15966546-15966605	MACROD2
A_23_P30435	9.852572	10.14686	NM_006058	chr5:150409825-150409766	TNIP1
A_23_P40307	11.015091	10.606829	NM_003092	chr20:16721741-16721800	SNRPB2
A_24_P74070	7.974167	8.018131	NM_032510	chr18:77915404-77915345	PARD6G
A_33_P3416767	5.3268948	5.2349777	NM_032711	chr17:79880543-79880484	MAFG
A_33_P3420235	5.8490157	5.6267347		chr1:183592499-183592440	ARPC5
A_33_P3230073	12.00715	12.103256		chr1:166717778-166717837	
A_23_P130040	9.735139	9.290409	NM_002634	chr17:47481838-47481779	PHB
A_33_P3303951	6.6654634	6.5707808	NM_007342	chr7:23224857-23224916	NUPL2
A_33_P3409675	11.751728	11.802977		chr5:7299797-7299743	LOC442132
A_23_P99853	7.4543805	7.7579827	NM_019600	chr15:52874158-52874099	FAM214A
A_23_P160729	6.917464	6.686458	NM_006594	chr1:114438026-114437967	AP4B1
A_23_P95823	8.731164	8.819264	NM_145080	chr16:27245524-27244425	NSMCE1
A_33_P3336587	4.186993	4.442566	NM_001243538	chr15:31515097-31515038	LOC283710
A_23_P128993	3.807242	3.5989056	NM_033423	chr14:25075768-25075709	GZMH
A_23_P167367	2.3221061	2.3900566	NM_153426	chr4:111538732-111538673	PITX2
A_33_P3395389	6.318448	6.389187	NM_144689	chr19:37581964-37582023	ZNF420
A_33_P3340565	6.3608336	6.6247168	NM_001039503	chr16:31094871-31094812	PRSS53

A_24_P12904	7.282345	7.7893987	NM_032012	chr9:111800330-111798635	TMEM245
A_23_P137209	7.8696556	7.9088755	NM_003334	chrX:47073739-47073798	UBA1
A_33_P3313596	9.31986	9.233305	NM_033487	chr1:1654207-1654148	CDK11B
A_23_P100602	7.009017	7.0308022	NM_005993	chr17:80895163-80895222	TBCD
A_23_P377434	10.199792	10.153238	NM_001080495	chr7:5346514-5346455	TNRC18
A_24_P81947	10.24888	9.34314	NM_014325	chr12:109039269-109039210	CORO1C
A_23_P7791	10.424505	10.464189	NM_024576	chr6:72011813-72011872	OGFRL1
A_32_P144342	8.706629	8.699329	NM_006437	chr13:24995222-24995163	PARP4
A_24_P246636	6.8518085	6.8707294		chr12:053536141-053536200	
A_24_P85557	2.3221061	2.3900566	NM_005302	chr7:124386270-124386211	GPR37
A_24_P14260	6.2427726	6.2717843	NM_014959	chr19:48711576-48711517	CARD8
A_32_P14894	13.3183975	13.3379755	NM_001014	chr6:34392888-34392598	RPS10
A_24_P412512	6.9202237	6.956485	NM_016350	chr14:51204925-51204866	NIN
A_23_P137984	13.705366	13.773476	NM_002966	chr1:151955479-151955420	S100A10
A_32_P203430	6.834096	6.3932695	NM_194325	chr19:35435800-35435859	ZNF30
A_23_P165952	4.364176	4.480255	NM_024855	chr20:37396189-37400210	ACTR5
A_32_P447001	4.620708	4.6521897	NM_001198784	chr15:55710662-55710721	C15orf65
A_33_P3357049	5.567811	5.5379806	NR_026768	chr1:46111983-46111924	RPS15AP10
A_24_P82032	2.3221061	2.3900566	NM_020663	chr14:63757601-63757660	RHOJ
A_33_P3408047	3.9518027	3.994742		chr20:023465238-023465297	
A_23_P48237	7.5625954	7.2455864	NM_006337	chr12:49952502-49952443	MCRS1
A_23_P413193	9.620072	9.495665	NM_145247	chr10:105885925-105885984	SFR1
A_32_P155247	12.132181	12.27826	NM_000146	chr19:49469029-49469088	FTL
A_23_P218770	10.841731	10.788362	NM_002872	chr22:37621511-37621452	RAC2
A_33_P3273258	7.5406065	7.70101	NM_001172655	chr19:53079214-53079273	ZNF701
A_23_P67589	11.251224	11.293382	NM_001100418	chr19:18702951-18703010	C19orf60
A_24_P176493	8.766542	8.733519	NM_015251	chr16:81080715-81080774	ATMIN
A_23_P133236	3.1044378	2.3900566	NM_018934	chr5:140605528-140605587	PCDHB14
A_23_P212329	8.291554	8.286522	NM_015466	chr3:47454665-47454724	PTPN23
A_33_P3406004	6.4029093	6.291986	NM_058181	chr21:47706977-47707036	YBEY
A_23_P91657	7.326207	7.455237	NM_013986	chr22:29688522-29688581	EWSR1
A_33_P3510837	7.197453	8.141565	AK096443	chr17:10622306-10622365	
A_24_P418418	14.851745	14.903694	NM_001021	chr15:82821242-82821183	RPS17
A_33_P3325851	3.3123174	3.7711203		chr3:013968748-013968807	
A_33_P3318377	5.0703864	5.4090295		chr10:127445429-127445488	
A_24_P53976	7.671232	7.969014	NM_002065	chr1:182352796-182352737	GLUL
A_33_P3236416	5.2387943	5.3977327	NM_001004334	chr17:36481658-36481599	GPR179
A_33_P3336038	15.495825	15.677139		chr11:077445416-077445357	
A_33_P3265866	5.7594714	5.477913		chrX:049921065-049921124	
A_24_P401787	3.7458549	3.945817	NM_173353	chr12:72338117-72338176	TPH2
A_23_P142776	9.965059	10.163528	NM_003754	chr11:8013379-8013670	EIF3F
A_24_P272088	4.254793	4.1820617	NM_001024858	chr14:65216731-65216388	SPTB
A_23_P97573	8.862143	8.729915	NM_004698	chr1:150318947-150321671	PRPF3
A_24_P217848	9.747165	9.742509	NM_003510	chr6:27805788-27805729	HIST1H2AK
A_23_P119266	5.124682	5.023176	NM_001375	chr19:12987100-12987041	DNASE2
A_33_P3369436	8.010693	8.50565	XR_109206	chr15:29969579-29969638	LOC100130111
A_23_P218997	11.723917	11.736568	NM_013232	chr5:314812-314871	PDCD6
A_32_P36046	6.127284	6.253069	NM_001013649	chr2:85832769-85832710	C2orf68
A_33_P3285077	9.301542	9.565326	NM_017845	chr4:47455136-47455077	COMMD8
A_23_P335905	6.379514	6.6918445	NM_015690	chr2:219566839-219566898	STK36
A_23_P14493	8.839635	7.416483	NM_018139	chr14:50092351-50092292	DNAAF2
A_33_P3335257	5.560713	5.7032747	NM_152784	chr19:5771011-5771070	CATSPERD
A_32_P50417	7.6962156	7.6563864	AK092531	chr8:12428380-12428439	
A_23_P140301	12.016678	11.818749	NM_002788	chr14:58737175-58737682	PSMA3
A_23_P159952	2.3221061	2.3900566	NM_018476	chrX:102317677-102317618	BEX1
A_32_P40744	6.2374167	5.889145	XM_005276020	chr8:82192013-82191954	LOC101927085
A_33_P3840630	9.02626	9.303729	NM_015030	chr4:48501356-48501297	FRYL
A_33_P3224265	7.333768	7.5195417		chr17:37004208-37004149	
A_33_P3416568	8.329617	8.179239		chr6:143382683-143382742	

A_23_P207742	6.236993	6.441343	NM_003250	chr17:38250008-38250067	THRA
A_33_P3287529	7.4270253	7.2810116	NM_016176	chr1:1153928-1153869	SDF4
A_23_P24515	10.626605	10.929781	NM_000019	chr11:108018009-108018068	ACAT1
A_33_P3300916	2.3221061	2.3900566		chr3:151502623-151502682	LOC201651
A_32_P840463	7.508556	7.3973966	NR_024495	chr10:075471516-075471457	BMS1P6
A_32_P342064	15.845007	16.035227	NM_002032	chr11:61732096-61732038	FTH1
A_23_P164958	7.9590693	8.292097	NM_032040	chr19:46914401-46914342	CCDC8
A_33_P3374563	6.215123	6.3704176	NM_018418	chr14:88904707-88904766	SPATA7
A_23_P1552	7.9789567	8.262749	NM_001814	chr11:88042347-88033771	CTSC
A_33_P3386364	6.2519693	6.2846656	NM_022725	chr11:22646292-22646233	FANCF
A_33_P3264089	4.8017397	4.5386825	NM_001164458	chr7:149992401-149992342	ACTR3C
A_33_P3800664	7.9386106	7.739944	AK090649	chr1:1345812-1345871	
A_33_P3404697	6.9257903	6.504142	NM_153336	chr10:124749773-124749832	PSTK
A_24_P336577	7.0159397	7.189899	NM_019099	chr1:112269251-112269192	FAM212B
A_23_P433016	5.415827	5.647876	NM_001996	chr22:45939377-45942994	FBLN1
A_33_P3216438	6.1027884	6.423235	NM_198546	chr1:16725197-16725138	SPATA21
A_33_P3400653	9.291919	9.090052	XM_005254607	chr15:59951495-59951436	BNIP2
A_33_P3307903	8.326058	8.333729	NM_005192	chr14:54866634-54866693	CDKN3
A_23_P365149	7.631653	7.847318	NM_005881	chr16:31122522-31122661	BCKDK
A_23_P73530	8.336765	8.332292	NM_003828	chrX:149933006-149933065	MTMR1
A_33_P3355230	5.0961995	5.007228	NM_002287	chr19:54868203-54868144	LAIR1
A_23_P117424	6.754805	6.767873	NM_025230	chr14:24592811-24592870	DCAF11
A_23_P630	4.450133	4.27268	NM_018116	chr1:155582085-155582245	MSTO1
A_33_P3297452	7.4889865	7.730931		chr17:014851322-014851263	
A_23_P46982	10.714432	10.677486	NM_022362	chr10:99218296-99218237	MMS19
A_23_P104188	4.9602575	5.8970094	NM_004433	chr1:201984429-201984488	ELF3
A_23_P318860	4.251568	3.9699986	NM_005161	chr11:57003412-57003353	APLNR
A_33_P3283824	5.3682575	4.6182566	NM_001135147	chr4:103172475-103172416	SLC39A8
A_23_P209360	8.169606	7.988655	NM_052920	chr2:23931309-23931368	KLHL29
A_24_P393571	2.3221061	2.3900566	NM_004293	chr9:74866942-74867001	GDA
A_23_P50008	9.484048	9.330807	NM_017775	chr17:15931822-15931881	TTC19
A_23_P50942	7.6657114	7.616477	NM_012233	chr2:135926699-135926758	RAB3GAP1
A_24_P66528	10.456314	10.659481	NM_003133	chr1:225971009-225971068	SRP9
A_23_P10232	4.847164	4.9536033	NM_017935	chr4:102995573-102995632	BANK1
A_24_P218814	5.1361785	4.7643476	NM_002905	chr12:56117833-56118164	RDH5
A_24_P201491	9.299647	9.02626	NM_004927	chr11:64894551-64894610	MRPL49
A_32_P86118	8.328598	7.3421836	NM_001010853	chr6:89874218-89874277	PM20D2
A_23_P94095	6.7646165	5.8987484	NM_198401	chr8:101533431-101533372	ANKRD46
A_23_P77731	4.645342	5.0004463	NM_001888	chr16:21270022-21269963	CRYM
A_24_P160696	3.717558	3.6712523	NM_033364	chr3:119483911-119483970	MAATS1
A_23_P358542	6.922508	6.719896	NM_145754	chr8:145699149-145699208	KIFC2
A_33_P3347417	8.29029	8.255937	NM_015001	chr1:16255108-16255167	SPEN
A_23_P24375	7.751102	7.7050595	NM_017670	chr11:63764649-63764708	OTUB1
A_33_P3231120	4.192582	4.3168373	NM_022900	chr7:94176420-94176479	CASD1
A_33_P3264612	6.8163366	6.862732	NM_139075	chr11:68858013-68858072	TPCN2
A_23_P22926	10.882939	10.811754	NM_002074	chr1:1717507-1717448	GNB1
A_33_P3251617	4.3076954	4.4215746		chr2:47049663-47049722	LINC01118
A_32_P165340	11.704896	11.564201	NM_003133	chr1:225978043-225978102	SRP9
A_23_P205875	4.4720902	4.876343	NR_004859	chr15:85183449-85183508	SCAND2P
A_32_P163125	9.07371	8.309152	NM_147156	chr10:52065619-52065560	SGMS1
A_23_P79251	2.8642492	3.842588	NM_014600	chr2:31490569-31490628	EHD3
A_24_P161973	9.277632	9.246224	NM_015205	chr13:113540918-113540977	ATP11A
A_24_P21044	8.542856	7.930476	NM_032302	chr7:1607480-1607421	PSMG3
A_23_P141730	8.209031	8.364223	NM_001943	chr18:29126460-29126519	DSG2
A_24_P418816	3.0063002	2.3900566	NM_015696	chr1:53074341-53074400	GPX7
A_24_P76854	5.987764	6.1567736	NM_001123387	chr17:39203004-39202945	KRTAP2-1
A_23_P166633	10.006038	10.301746	NM_002213	chr3:124482550-124482491	ITGB5
A_32_P160972	11.598512	11.521332	NM_021243	chr6:139364343-139364402	ABRACL
A_24_P940166	9.16416	9.175742	NM_001015880	chr10:89506954-89507013	PAPSS2

A_23_P83917	5.857787	6.1095476	NM_012308	chr11:67024380-67024439	KDM2A
A_23_P305723	9.45035	9.617048	NM_020948	chr1:67453133-67453192	MIER1
A_33_P3252414	6.6556435	6.541146	NM_199292	chr11:2185218-2185159	TH
A_32_P141238	4.244814	4.298633	NM_001278597	chr12:5671939-5671880	ANO2
A_33_P3301095	7.788666	7.5386486		chr4:003913265-003913206	
A_32_P153773	4.5301065	4.6728063	NM_000718	chr9:141018581-141018640	CACNA1B
A_24_P84808	14.374565	14.376529		chr19:006593823-006593884	
A_33_P3247933	7.374543	7.010625	NM_181453	chr2:109086421-109086480	GCC2
A_33_P3223544	10.30835	10.680951	NM_001282606	chr12:66531836-66531777	TMBIM4
A_33_P3329301	5.977857	6.1098924	AK126557	chr17:62764044-62763985	LOC646014
A_33_P3238410	4.81603	4.7960186	NM_002972	chr22:50899648-50899589	SBF1
A_23_P351275	9.423289	8.862941	NM_181597	chr7:48141567-48142940	UPP1
A_33_P3349265	4.53368	4.4066286	NM_001010904	chr6:49494542-49494601	GLYATL3
A_33_P3290667	9.804258	9.639727	NM_016275	chr3:150344862-150344921	SELT
A_24_P365526	4.1335306	4.2085934	NM_002110	chr20:30676400-30681677	HCK
A_33_P3272580	5.916127	5.819295	NM_032020	chr6:143828434-143828375	FUCA2
A_33_P3413463	10.092132	9.931082	NM_001519	chr14:105675687-105675628	BRF1
A_23_P207666	7.07519	7.241024	NM_004505	chr17:5077642-5077701	USP6
A_33_P3352019	9.002659	8.631449	NM_182826	chr8:27516996-27517055	SCARA3
A_33_P3310104	2.3221061	2.3900566	NM_002639	chr18:61172218-61172277	SERPINB5
A_23_P31389	6.977308	7.39042	NM_013293	chr7:23545801-23545418	TRA2A
A_23_P374782	9.748213	9.759965	NM_001024666	chrX:19554143-19554084	SH3KBP1
A_24_P723735	5.6385036	5.7615438		chr16:031613274-031613215	
A_32_P420563	7.0005636	6.9048233	NM_001017981	chr22:30774868-30774809	RNF215
A_23_P18384	6.8920918	6.977627	NM_213654	chr3:137965455-137965514	ARMC8
A_23_P36305	8.19695	8.406145	NM_033388	chr11:72540556-72540615	ATG16L2
A_23_P131954	12.147322	12.278723	NM_014426	chr20:17923049-17922990	SNX5
A_32_P47107	9.845373	9.961436		chr8:134585970-134586029	
A_24_P149124	7.8930626	7.8145466	NM_004772	chr5:111065150-111065091	NREP
A_24_P79153	6.120202	6.281924	NM_079834	chr19:1925547-1925606	SCAMP4
A_23_P342934	4.3146124	4.326833	NM_005078	chr15:70341255-70341196	TLE3
A_23_P209987	8.743439	8.421174	NM_019014	chr2:113333426-113333485	POLR1B
A_33_P3417950	9.852298	9.715432	NM_005858	chr19:15464414-15464355	AKAP8
A_23_P304897	2.3221061	2.3900566	NM_000623	chr14:96710341-96710400	BDKRB2
A_23_P46907	8.146224	8.283682	NM_006077	chr10:74127511-74127452	MICU1
A_23_P127613	10.665673	10.438267	NM_016146	chr11:118894323-118894382	TRAPPC4
A_33_P3408983	8.756094	8.601424	NM_001127511	chr5:112103018-112103077	APC
A_23_P127385	8.411724	8.512288	NM_000256	chr11:47353223-47353164	MYBPC3
A_33_P3388070	4.8151436	4.475304	DB226000	chr13:112111461-112111402	
A_24_P339560	4.0463743	3.8466387	NM_052884	chr19:50453258-50453199	SIGLEC11
A_33_P3286302	8.213383	8.218246	XR_241046	chr1:2493038-2493097	TNFRSF14
A_23_P213840	10.970034	11.319585	NM_003945	chr5:172421786-172447260	ATP6V0E1
A_23_P3663	8.867994	8.840236	NM_138418	chr16:698363-698422	FAM195A
A_24_P95439	9.795792	9.428607	NM_001014437	chr11:3039103-3038483	CARS
A_24_P382401	5.65385	6.0441427	NM_153358	chr19:12739507-12739566	ZNF791
A_23_P340722	10.051027	10.073771	NM_007235	chr12:64828653-64829429	XPOT
A_33_P3323392	7.335602	7.5365005	BC046635	chr13:64406341-64406282	
A_33_P3244165	16.062252	16.000206	NR_003287	chrUn_gl000220:117142-117201	RNA28S5
A_33_P3266444	7.0118403	6.6644273	NM_022497	chr3:15090184-15090125	MRPS25
A_23_P411335	8.54245	8.703005	NM_152524	chr2:201438513-201438572	SGOL2
A_24_P115971	7.8923926	7.9719305	NM_021932	chr11:213373-214243	RIC8A
A_23_P41476	7.4444027	7.2581863	NM_001080505	chr4:42404181-42404240	SHISA3
A_23_P254404	5.07748	5.177889	NM_153271	chr15:75950515-75950574	SNX33
A_33_P3264179	4.426054	4.782546	NM_178435	chr1:152538372-152538313	LCE3E
A_24_P261005	8.864775	8.98626	NM_138436	chr8:42403872-42407694	SMIM19
A_23_P376557	7.215705	7.3002768	NM_022468	chr16:3110418-3110477	MMP25
A_23_P23748	5.103488	5.6791506	NM_014969	chr1:109513392-109513333	WDR47
A_23_P78664	12.684835	12.640703	NM_005804	chr19:14519947-14519888	DDX39A

A_33_P3357535	6.73715	6.06996	NM_030579	chr16:69500108-69500167	CYB5B
A_33_P3317937	5.0933695	5.0999928	NM_181615	chr21:31988774-31988833	KRTAP20-1
A_33_P3374085	8.837372	8.82123	NM_001137552	chr2:238672724-238672783	LRRFIP1
A_24_P365327	10.166229	10.779298	NM_015578	chr19:34718765-34718824	LSM14A
A_23_P315892	6.2261763	6.350553	NM_013443	chr9:130648124-130648065	ST6GALNAC6
A_33_P3519683	8.881489	8.799836	NM_178547	chr1:33099619-33099560	ZBTB8OS
A_33_P3414422	8.453941	8.319006	NM_020806	chr14:67648373-67648432	GPHN
A_23_P45976	4.4069877	4.553671	NM_002885	chr1:21928234-21926109	RAP1GAP
A_23_P367071	5.648266	5.9172935	NR_024062	chrX:84189670-84189729	UBE2DNL
A_23_P34496	8.312761	8.303669	NM_018056	chr1:32568154-32568213	TMEM39B
A_23_P121011	3.6371386	2.3900566	NM_033027	chr3:39184219-39184160	CSRNP1
A_24_P30567	5.832809	5.852904	NM_139015	chr12:121201145-121201086	SPPL3
A_24_P256552	3.410367	3.4875832	NM_001033505	chr11:33182946-33182887	CSTF3
A_33_P3258146	5.2963486	5.3760595	NM_001258000	chr10:44789941-44790000	LOC100130539
A_23_P130764	5.6489744	5.1627345	NM_013348	chr19:48968459-48968518	KCNJ14
A_33_P3248953	3.1795416	3.1133718		chr5:126387528-126387469	C5orf63
A_32_P104478	7.0336733	6.9758325	NM_018351	chr12:95470741-95470682	FGD6
A_33_P3248644	11.750135	11.91446	NM_005051	chr3:49133429-49133370	QARS
A_33_P3289541	4.986694	4.451868	NM_005934	chr19:6211255-6211196	MLLT1
A_23_P53530	7.3616414	7.1980104	NM_001033050	chr12:107371680-107371621	MTERFD3
A_33_P3258279	2.3221061	2.3900566	NM_001144059	chr11:132184901-132184960	NTM
A_23_P152984	11.69906	10.728455	NM_005782	chr17:79845823-79845764	ALYREF
A_23_P215883	4.0350027	3.377836	NM_001040630	chr8:102699720-102699661	NCALD
A_23_P41327	10.064254	9.611312	NM_017816	chr4:4270336-4270277	LYAR
A_23_P160849	7.2203918	7.6169944	NM_004106	chr1:161188875-161188934	FCER1G
A_33_P3384229	4.057884	4.1905985		chr14:039839905-039839846	
A_33_P3413987	5.3103876	5.4714384	NM_000062	chr11:57367781-57367840	SERPING1
A_33_P3309984	2.968793	2.3900566	NM_000221	chr2:27323555-27323614	KHK
A_23_P64837	6.9198503	6.312803	NM_001031628	chr12:51639735-51639676	SMAGP
A_24_P409519	4.1013584	4.5857553	NM_006906	chr11:18750488-18750429	PTPN5
A_33_P3244753	7.563959	7.974578	NM_001939	chrX:100519426-100519485	DRP2
A_24_P61772	4.6372766	4.826378	XM_003846282	chr17:16827412-16827353	
A_24_P141629	8.5271225	8.500267	NM_022074	chr11:58921635-58921694	FAM111A
A_23_P69188	9.342443	9.789708	NM_206831	chr3:16302170-16302111	DPH3
A_33_P3328445	4.827509	4.710379	XR_243529	chr17:6905420-6905361	LOC100506713
A_23_P413585	4.039371	4.410121	NM_004474	chr1:47905704-47905763	FOXD2
A_33_P3253682	9.062921	8.972734	NM_032472	chr2:201736056-201735997	PPIL3
A_32_P91042	7.203576	7.0405784	NR_027406	chr9:127120692-127120751	LOC100129034
A_33_P3294053	9.781103	9.852572	NM_016466	chr2:97513892-97513833	ANKRD39
A_23_P71855	6.352037	6.6139936	NM_001735	chr9:123715126-123715067	C5
A_23_P168812	6.2012835	5.8293576	NM_145111	chr7:99144679-99144620	FAM200A
A_23_P216655	8.96253	8.731164	NM_014788	chr9:100847293-100847234	TRIM14
A_33_P3363515	14.987498	15.143757		chr14:104170793-104170734	XRCC3
A_33_P3344332	11.907459	12.041058	NM_001000	chrX:118923875-118920641	RPL39
A_23_P115573	10.488684	10.575768	NM_198149	chr1:201861366-201861425	SHISA4
A_23_P336015	5.5859156	5.0680356	NM_015658	chr1:880965-880906	NOC2L
A_33_P3331188	5.7500825	6.0843973	NM_001199417	chr17:36646732-36646791	ARHGAP23
A_24_P18621	8.831302	8.671925	NM_153207	chr12:19672916-19672975	AEBP2
A_23_P133075	5.8650413	6.006634	NM_033115	chr4:107037522-107037463	TBCK
A_33_P3383422	5.8043594	5.7059064	NM_001282991	chr14:24424361-24424420	DHRS4
A_23_P160167	6.8997173	6.6009264	NM_005727	chr1:46651173-46651232	TSPAN1
A_23_P133133	5.728671	5.940391	NM_025144	chr4:113362743-113362802	ALPK1
A_33_P3226810	6.999048	7.7684474	NM_003810	chr3:172224285-172224226	TNFSF10
A_23_P252201	6.338186	5.9736333	NM_018456	chr3:121591565-121591624	EAF2
A_24_P408603	3.629443	3.2657914	NM_171998	chrX:154488063-154488004	RAB39B
A_23_P252211	7.197201	7.5607004	NM_024010	chr5:7900491-7900549	MTRR
A_33_P3399911	4.7330284	4.8533354		chr3:015174985-015174926	
A_33_P3212645	11.954972	11.833675	NM_203458	chr1:145281999-145282058	NOTCH2NL
A_24_P254949	3.822895	2.3900566	NM_021965	chr9:70993208-70993267	PGM5

A_23_P65674	5.6820393	5.9119716	NM_014547	chr15:52192385-52192444	TMOD3
A_24_P270033	5.328771	5.3743773	NM_198275	chr11:118097483-118097424	MPZL3
A_24_P333663	6.326337	6.79023	NM_002748	chr15:52356907-52356966	MAPK6
A_33_P3253634	7.0531864	7.1561666	NR_045529	chr15:82597136-82597195	ADAMTS7P1
A_24_P144620	5.672494	5.690773	NM_018452	chr6:157743786-157739942	TMEM242
A_23_P4628	2.7744288	3.5395339	NM_025027	chr19:58489120-58489061	ZNF606
A_24_P161914	13.345445	13.36665		chr2:050106350-050106409	
A_33_P3346508	6.5139465	6.469849		chr14:104578289-104578348	
A_33_P3413958	6.2409782	6.344099		chr8:011777675-011777616	LOC100509541
A_33_P3260575	10.850822	11.157981	NM_016174	chr9:131199564-131199623	CERCAM
A_24_P143301	4.087855	3.5199642	NM_181337	chr6:24357605-24357664	KAAG1
A_33_P3247659	5.2849793	5.1320887	NM_001110503	chr15:42556349-42556290	TMEM87A
A_33_P3283828	6.679225	6.8397365	NM_001143980	chr16:1484448-1484389	CCDC154
A_33_P3260722	5.8261375	4.8595715	NM_003369	chr11:75855200-75855259	UVRAG
A_33_P3388588	9.394446	9.959835	NR_046200	chr16:89235352-89235411	LOC400558
A_33_P3342111	8.205421	8.202939	NM_194320	chr9:97065232-97065291	ZNF169
A_33_P3263412	4.1137915	4.55462	AK130224	chr10:670469-670410	
A_32_P51894	9.003644	9.022004	NM_031844	chr1:245014677-245014618	HNRNPU
A_24_P49517	6.277448	6.000739	NM_138367	chr8:145947029-145946970	ZNF251
A_33_P3373750	10.526267	10.410517	NM_014299	chr19:15357906-15357847	BRD4
A_24_P409346	6.288436	6.749103	NM_006310	chr17:45660170-45662879	NPEPPS
A_23_P144827	6.9107094	7.14176	NM_012304	chr5:15939565-15939624	FBXL7
A_23_P397899	8.730341	8.683	NM_021218	chr9:115449402-115449343	INIP
A_23_P65609	6.70734	6.46312	NM_015859	chr14:81658868-81651911	GTF2A1
A_23_P39813	5.1972733	5.506155	NM_022492	chr2:74720827-74720886	TTC31
A_33_P3302255	12.010876	12.23697	NM_021999	chr13:48835407-48835466	ITM2B
A_23_P140146	10.907894	10.986447	NM_032036	chr14:94594217-94594158	IFI27L2
A_24_P82880	9.826691	9.806858	NM_003290	chr19:16199863-16199922	TPM4
A_23_P429082	7.4788084	7.1261697	NM_194285	chr11:18628254-18628195	SPTY2D1
A_33_P3224045	10.034136	9.712148	NM_001348	chr19:3958511-3958452	DAPK3
A_33_P3365441	3.832833	3.8666034		chr1:197887743-197887802	
A_33_P3323448	3.2073576	3.5639439	NM_178582	chr20:30126072-30126131	HM13
A_33_P3283167	9.672081	10.215347		chr1:180405277-180405336	
A_23_P145485	3.9188433	3.922708	NM_025217	chr6:150267714-150267773	ULBP2
A_23_P24157	7.8010387	6.9115257	NM_032709	chr10:100146987-100144795	PYROXD2
A_33_P3292829	6.4189086	6.4489226	NM_017988	chr12:100717325-100717384	SCYL2
A_23_P156284	10.623363	10.542131	NM_080881	chr5:176884027-176883968	DBN1
A_23_P255569	9.149407	9.222533	NM_022156	chr17:80016684-80016261	DUS1L
A_24_P382253	8.854102	8.625021	NM_018170	chr18:33569988-33569929	RPRD1A
A_32_P171181	7.11206	7.263638	NM_001039703	chr1:145309945-145310004	NBPF10
A_24_P271773	2.3221061	2.3900566	NR_001280	chr5:140537931-140537990	PCDHB17
A_33_P3335629	7.930994	7.804137	NM_014187	chr16:67262436-67262495	TMEM208
A_33_P3230090	5.6734333	5.7394195	NM_001128619	chr7:135613478-135613419	LUZP6
A_33_P3387272	10.8631	11.570307	NM_001402	chr6:74229639-74229214	EEF1A1
A_23_P24616	5.9536057	6.5195603	NM_170601	chr11:124506217-124506158	SIAE
A_33_P3227896	3.8382351	3.8423502	NR_027145	chr2:110704996-110705055	LIMS3-LOC440895
A_24_P120537	6.4542036	5.9980536	NM_152550	chr5:145439736-145441996	SH3RF2
A_33_P3255194	4.70614	4.7559633	NM_001201482	chr2:179213970-179214029	OSBPL6
A_23_P310532	8.487568	8.371309	NM_138358	chr19:11040645-11040704	C19orf52
A_33_P3210059	9.719589	9.600967	NM_005877	chr22:30728086-30728027	SF3A1
A_33_P3396553	5.2317276	4.9375987		chr1:001423229-001423288	
A_23_P109420	7.259304	7.3346786	NM_014753	chr10:43315722-43315781	BMS1
A_23_P34142	10.705652	10.830881	NM_016303	chrX:102612844-102612903	WBP5
A_33_P3213086	7.7334404	7.404705		chr18:14227639-14227698	
A_33_P3280681	6.4590287	6.8160586	XM_005277062	chr19:54969685-54969744	LENG8
A_23_P159390	8.184968	8.202114	NM_007027	chr3:133329966-133329907	TOPBP1
A_24_P775249	5.2543535	4.8766575	NR_024484	chr18:72260621-72260562	LINC00909
A_23_P209619	9.238633	9.009753	NM_022374	chr2:38522256-38522197	ATL2
A_23_P24004	8.817995	8.491976	NM_001547	chr10:91068397-91068456	IFIT2

A_33_P3321382	5.2145844	5.34919		chr9:43876220-43876279	
A_23_P424269	7.32664	7.5887165	XM_005252327	chr9:98775835-98775894	LOC101928170
A_23_P133375	8.681557	8.527391	NM_138773	chr5:110098257-110098316	SLC25A46
A_33_P3330453	9.463923	9.033585	NM_018313	chr3:52579570-52579511	PBRM1
A_33_P3283599	6.0648212	6.0992336	NR_003366	chr2:95461728-95461669	ANKRD20A8P
A_33_P3334015	3.6490695	2.987292	NM_152914	chr17:21146720-21146661	C17orf103
A_33_P3360382	4.5438337	4.77787	NR_024149	chr14:101372440-101372499	MEG8
A_23_P1641	4.4658537	4.2136874	NM_005133	chr11:66613646-66613705	RCE1
A_33_P3321533	4.109936	4.072492		chr7:140912496-140912555	TMEM178B
A_33_P3411414	3.7923377	3.6335242	NM_001099684	chrX:52936573-52936632	FAM156B
A_33_P3361027	9.270045	9.220818	NM_001008697	chr22:26888011-26887952	TFIP11
A_23_P24365	7.049968	7.209549	NM_017704	chr11:94232501-94232560	ANKRD49
A_23_P23564	4.813389	4.252518	NM_003557	chr1:151220873-151220931	PIP5K1A
A_23_P127079	10.105835	9.9344635	NM_015062	chr10:103909689-103909748	PPRC1
A_33_P3271126	4.720375	4.4949665		chr19:045737511-045737452	
A_23_P25989	6.872841	7.059537	NM_032233	chr14:99865119-99865060	SETD3
A_33_P3325753	6.4953856	6.4371247	NM_001039141	chr22:38172501-38172560	TRIOBP
A_23_P81408	8.928362	9.4971075	NM_182796	chr5:162945313-162945372	MAT2B
A_24_P133488	7.3732224	7.4281096	NM_017955	chr14:105476786-105476727	CDCA4
A_23_P401076	9.165677	8.980867	NM_145006	chr9:95847304-95847363	SUSD3
A_23_P359540	7.518828	7.323043	NM_003540	chr6:26240818-26240877	HIST1H4F
A_33_P3386965	5.095264	3.843077	NM_145167	chr1:159997682-159997623	PIGM
A_23_P333057	4.1387076	4.2642035		chr6:134174190-134174249	MGC34034
A_32_P44512	11.286869	11.425524	NM_003932	chr22:41222489-41222430	ST13
A_33_P3347012	7.525032	7.3831973	DB462629	chr1:230537777-230537718	
A_23_P167129	2.450664	2.3900566	NM_022475	chr4:145658929-145658988	HHIP
A_23_P362228	5.9399705	6.114223	NR_033690	chr1:23698088-23698147	C1orf213
A_33_P3410895	8.0403595	7.9995184		chr7:75544784-75544843	POR
A_24_P361643	3.7203805	3.767065	NM_001039887	chr19:36259696-36259755	C19orf55
A_23_P61406	2.933579	2.3900566	NM_016848	chr9:91628173-91628114	SHC3
A_33_P3292840	3.9384403	3.9406548	NM_001003678	chr11:47185868-47185927	C11orf49
A_23_P27649	5.506957	5.6398215	NM_001080411	chr19:12125839-12125780	ZNF433
A_23_P380815	9.632752	10.027004	NM_015634	chr10:70776472-70776531	KIAA1279
A_33_P3340229	3.4111938	4.27617		chr19:057910345-057910286	
A_33_P3306828	3.7567353	3.386463	XM_005276120	chr2:133076250-133076309	LOC100292952
A_33_P3216938	4.2699604	4.6431317	NR_103765	chr11:10330136-10330195	CAND1.11
A_33_P3393573	9.042736	9.127871	NM_001252053	chr6:150092328-150092387	PCMT1
A_33_P3309734	4.528119	4.340867	NM_152389	chr2:219900172-219900113	CCDC108
A_33_P3592015	9.643215	9.568191	NM_014282	chr9:99252704-99252763	HABP4
A_33_P3269000	3.2191448	3.6642196	NM_001007090	chr8:13425425-13425484	C8orf48
A_23_P205428	2.3221061	2.3900566	NM_005249	chr14:29238589-29238648	FOXG1
A_24_P6428	5.0301604	5.706742	NM_022106	chr20:58521867-58521926	FAM217B
A_23_P36157	7.802499	7.4429994	NM_018093	chr11:62601956-62601802	WDR74
A_24_P41021	8.411174	8.379686	NM_203301	chr14:39867954-39867895	FBXO33
A_23_P17103	3.8717923	4.534923	NM_025244	chr2:99614532-99614473	TSGA10
A_23_P146830	4.867543	3.891614	NM_012140	chr17:79687525-79687584	SLC25A10
A_24_P95038	15.291574	14.809121	NM_021130	chr7:44841005-44841064	PPIA
A_33_P3309832	4.4810905	4.991165	NM_182601	chr10:115537419-115537478	PLEKHS1
A_33_P3407445	5.390135	5.484709	AK090949	chr5:127302431-127302372	FLJ33630
A_23_P393607	10.820656	10.824588	NM_053052	chr1:227968774-227968833	SNAP47
A_24_P341504	6.9115257	6.6509895	NM_017619	chr1:104093659-104094365	RNPC3
A_23_P301340	8.070118	8.2439995	NM_144679	chr17:79202154-79202095	ENTHD2
A_23_P251916	8.832087	8.697972	NM_016033	chr8:87484679-87484620	RMDN1
A_23_P401014	5.6671724	5.8420415	NM_005744	chr15:72847688-72848235	ARIH1
A_33_P3352687	8.339792	8.473993	NR_028326	chr11:128915-128856	LINC01001
A_23_P369994	6.730048	5.69204	NM_004734	chr13:36345567-36345508	DCLK1
A_33_P3369565	3.7545524	3.739795	NM_001195252	chr9:32989828-32989769	APTX
A_23_P62999	4.0154386	4.4001856	NM_004455	chr1:26362746-26362805	EXTL1
A_23_P64770	10.10964	10.104904	NM_004818	chr12:49223805-49223746	DDX23

A_23_P61466	2.3221061	2.3900566	NM_174941	chr12:7521549-7520755	CD163L1
A_33_P3308862	4.4862247	4.254191	NM_001278495	chr10:81470298-81470357	NUTM2B
A_32_P29806	8.00534	7.9440513	NM_003805	chr12:94244211-94244270	CRADD
A_32_P95223	8.494542	8.489635	NR_003262	chr7:76598123-76598064	FDPSP2
A_33_P3341429	9.436007	8.98567	NM_144573	chr1:78383898-78383957	NEXN
A_23_P91001	9.540005	9.481358	NM_019048	chr2:190535246-190535305	ASNSD1
A_33_P3330872	3.8198085	4.039371	NM_078629	chrX:11781992-11782051	MSL3
A_33_P3395738	6.890525	6.8347783	AK127004	chr16:75280329-75280270	BCAR1
A_33_P3379454	5.753783	5.862552	NM_013282	chr19:4960895-4960954	UHRF1
A_32_P475513	4.0294905	3.4251366	NR_003587	chr17:73622248-73622307	MYO15B
A_24_P21447	7.8521566	7.507099	NM_006753	chr9:136197866-136197807	SURF6
A_23_P15511	12.653652	12.853292	NM_004396	chr17:62496344-62496285	DDX5
A_33_P3234864	5.101246	5.229457	NM_007124	chr6:145051508-145051567	UTRN
A_33_P3332066	8.214586	8.5044	NM_212554	chr10:126454076-126454017	METTL10
A_23_P375281	6.2615323	6.126588	NM_080706	chr17:3468894-3468835	TRPV1
A_32_P923011	6.8090816	7.1176033	NR_026818	chr1:35768-35470	FAM138A
A_33_P3215412	7.05478	7.2994494	NM_001136263	chr19:405567-405508	C2CD4C
A_33_P3315764	4.2484884	4.9361567	NM_001126118	chr17:7579372-7579313	TP53
A_33_P3220565	8.8896885	8.595508	NM_152277	chr5:171636775-171636716	UBTD2
A_23_P250283	8.728009	8.821547	NM_004161	chr2:65314287-65314228	RAB1A
A_23_P44569	4.6835628	3.9160337	NM_000392	chr10:101611322-101611381	ABCC2
A_33_P3410409	8.916543	9.160913	NM_001122606	chrX:119575645-119575586	LAMP2
A_32_P326819	6.077261	5.327579	NM_007043	chr12:75892085-75892026	KRR1
A_24_P254177	8.725267	8.4768095	NM_024104	chr19:16757875-16757816	SMIM7
A_33_P3298356	10.049269	10.003825	NM_001206651	chr1:87209181-87209240	SH3GLB1
A_33_P3317988	6.975334	7.5959263	NM_016308	chr1:47838720-47838779	CMPK1
A_33_P3311083	4.444925	4.926345	AK097512	chr19:44000515-44000456	PHLDB3
A_24_P281801	9.278763	9.493958	NM_001137608	chr4:265611-265552	ZNF732
A_24_P253827	8.267304	8.187498	NM_001030006	chr17:34052738-34052797	AP2B1
A_33_P3263989	5.4110737	4.9346213		chr3:106823654-106823595	
A_23_P26759	5.855679	6.020606	NM_138793	chr17:76988753-76988694	CANT1
A_23_P116430	4.5846825	4.619222	NM_005709	chr11:17522614-17519776	USH1C
A_23_P117380	6.1482472	6.3191423	NM_138344	chr14:94395784-94395843	FAM181A
A_23_P360079	2.3221061	2.3900566	NM_207363	chr2:133483287-133483228	NCKAP5
A_23_P45699	10.196302	10.288681	NM_003902	chr1:78414385-78414326	FUBP1
A_33_P3224867	4.4822025	4.804696	NM_001206842	chr12:13256497-13256438	GSG1
A_23_P252536	10.379764	10.347855	NM_030811	chr20:3028653-3028712	MRPS26
A_33_P3233378	3.7527423	3.530705	XR_171112	chrUn_gl000212:65860-65919	
A_33_P3281905	9.566302	9.559931		chr15:93111107-93111048	LINC00930
A_33_P3381044	5.4529543	5.4813147		chr19:056853982-056853923	
A_23_P56288	7.084196	7.3737464	NM_052925	chr19:54972922-54972981	LENG8
A_23_P307392	4.688545	4.5566425	AK024141	chr14:73075947-73075888	DPF3
A_24_P49747	5.6791506	5.8781796		chr9:036303660-036303601	
A_24_P393565	4.6222644	5.693468	XM_005258237	chr18:32948805-32948746	ZNF396
A_33_P3235491	5.618151	5.8163853		chr22:029877703-029877644	
A_33_P3306146	6.291869	5.120509	NM_001145031	chr10:75674614-75674673	PLAU
A_23_P193	6.3844805	6.545926	NM_022774	chr1:40981452-40981511	EXO5
A_23_P129334	4.679688	4.4862247	NM_001287	chr16:1496015-1495956	CLCN7
A_23_P124122	10.225275	10.38916	NM_018663	chr12:133281412-133281470	PXMP2
A_24_P128442	4.825526	4.9331098	NM_152380	chr1:119425783-119425724	TBX15
A_23_P300728	7.0172224	7.3234396	NM_022458	chr7:156476305-156476246	LMBR1
A_33_P3356711	5.9156895	5.3535914	NM_198267	chr7:120596864-120596923	ING3
A_33_P3221353	5.5542865	5.3635626	NM_001009814	chr13:42439932-42439873	VWA8
A_23_P354297	8.647105	8.808923	NM_022092	chr16:847945-848004	CHTF18
A_23_P411806	5.027346	5.7934327	NM_080546	chr9:108136938-108136997	SLC44A1
A_33_P3301219	5.549203	5.8182206	NR_024060	chr9:45727959-45728018	FAM27A
A_24_P100368	6.619705	6.348966	NM_006520	chrX:37701092-37700357	DYNLT3
A_33_P3320212	5.7488456	6.095657		chr16:70190375-70190316	
A_33_P3375710	2.8074114	3.7630057		chr1:007430240-007430299	

A_23_P428842	8.334507	8.573546	NM_138399	chr3:194308712-194308653	TMEM44
A_24_P116871	10.697035	9.95391	NM_006701	chr18:77748251-77737654	TXNL4A
A_24_P371758	8.272485	8.096658	NM_032479	chr5:1798866-1798807	MRPL36
A_23_P391725	8.895091	8.444539	NM_001938	chr5:151821852-151821792	DR1
A_23_P161098	5.9116287	5.5392866	NM_001973	chr1:205589316-205589257	ELK4
A_33_P3376873	6.0164585	5.850315	BC041650	chr16:8956083-8956024	
A_33_P3259393	8.271236	8.167171	NM_178232	chr15:89420640-89420581	HAPLN3
A_23_P425750	8.2439995	8.280262	NM_033415	chr19:19168505-19168564	ARMC6
A_33_P3247838	6.4163766	6.6274357	NM_001136501	chr19:12188542-12188601	ZNF844
A_23_P109768	11.519245	11.7002945	NM_014245	chr3:141464250-141464309	RNF7
A_23_P54692	8.303549	9.13957	XM_005256332	chr16:89982353-89982412	LOC101927910
A_33_P3316313	7.524068	7.5742316	NM_182501	chr2:242035373-242035314	MTERFD2
A_33_P3267410	4.621549	3.818359	NM_001126049	chr10:89619015-89618956	KLLN
A_23_P24796	7.149357	7.571899	NM_032127	chr11:6232816-6232757	FAM160A2
A_23_P436476	10.791874	10.706341	NM_015014	chr1:235295272-235295213	RBM34
A_33_P3211327	4.943234	5.3356514	AK124141	chr11:64856163-64856104	TMEM262
A_33_P3420931	4.1163397	4.7197723		chr12:52796111-52796170	
A_33_P3843358	5.3464503	5.163637	BG944179	chr14:21865360-21865301	SNORD8
A_33_P3233869	6.766816	6.7349663		chr9:38566282-38566257	
A_24_P540057	4.604895	4.1831565	NM_001739	chr16:87960503-87960444	CA5A
A_23_P40025	11.98762	12.115775	NM_014764	chr12:51637402-51637461	DAZAP2
A_23_P41765	4.151139	3.756192	NM_002198	chr5:131819501-131819442	IRF1
A_33_P3325229	5.375417	5.3165846	NR_034031	chrX:3746781-3746722	LOC389906
A_23_P154447	12.305746	11.926203	NM_015934	chr2:203167727-203168114	NOP58
A_23_P326157	3.7530584	4.0144167	NM_022134	chr2:242741440-242742807	GAL3ST2
A_33_P3230436	6.296055	6.403131	NM_182515	chr19:21307669-21307728	ZNF714
A_23_P37676	3.7007225	3.9341667	NM_007223	chr15:40093524-40093465	GPR176
A_33_P3407103	5.900701	6.1467214	NM_017798	chr20:61845276-61845217	YTHDF1
A_33_P3349325	6.278937	6.3393726	NM_001017979	chr4:13476001-13475942	RAB28
A_33_P3270742	4.4686813	4.335229		chr9:000011630-000011571	
A_23_P81492	14.66998	14.639593	NM_001025071	chr5:149826518-149826459	RPS14
A_24_P23034	7.226522	7.557264	NM_021035	chr20:47863115-47863056	ZNFX1
A_33_P3324909	4.0240335	3.46736	NM_001286968	chr19:18391430-18391371	JUND
A_33_P3321793	5.6692467	6.144235	BM973477	chr17:5024626-5024685	
A_23_P253200	10.338907	10.79854	NM_002948	chr3:23961682-23961741	RPL15
A_32_P185628	3.7626448	3.3698013		chr20:004680654-004680595	
A_23_P38446	9.803656	10.064613	NM_021137	chr17:26673908-26673967	TNFAIP1
A_33_P3273364	8.052155	8.400639	NM_024029	chr19:11033784-11033725	YIPF2
A_24_P330385	3.1252067	4.4614043	NM_016609	chr14:23816808-23816749	SLC22A17
A_23_P151614	4.8496637	5.147873	NM_006263	chr14:24606378-24606552	PSME1
A_33_P3271410	5.145991	5.482492	NM_024672	chr4:83840982-83841041	THAP9
A_24_P74371	11.153033	11.791811	NM_000308	chr20:44527241-44527300	CTSA
A_33_P3217609	4.3118668	4.359711	NR_104292	chr22:47309271-47309330	TBC1D22A
A_23_P91221	6.54707	6.8633246	NM_181805	chr20:43246977-43247036	PKIG
A_23_P50146	9.95391	9.885595	NM_213602	chr18:43422461-43422519	SIGLEC15
A_33_P3248602	6.316386	6.389549	NM_001127386	chr10:135485016-135485075	DUX4L2
A_23_P131149	9.353698	9.450476	NM_002552	chr2:148693242-148693183	ORC4
A_24_P416131	14.652729	14.592072	NM_021149	chr16:84599397-84599338	COTL1
A_23_P255827	11.4216175	11.759978	AF300871	chr8:36746755-36746814	TPT1P8
A_32_P185317	4.630957	4.8372684		chr7:156809059-156809118	MNX1-AS1
A_23_P339480	11.596947	11.452857	NM_003642	chr2:172848187-172848246	HAT1
A_33_P3249439	9.7178135	9.551503	NM_001185011	chr22:50961833-50961892	NCAPH2
A_23_P15108	5.7535534	6.455486	NM_031477	chr16:30104106-30104047	YPEL3
A_23_P159839	9.0644455	9.10318	NM_152692	chrX:119760176-119760117	C1GALT1C1
A_23_P165698	7.0239844	7.1294007	NM_024093	chr2:105959526-105959585	C2orf49
A_33_P3312365	7.554539	7.4797087	NM_004793	chr19:5693768-5693709	LONP1
A_24_P368943	5.79386	6.022228	NM_001989	chr7:27285959-27286018	EVX1
A_23_P258689	8.395071	8.528401	NM_017802	chr7:825448-825507	HEATR2
A_23_P73667	5.109939	4.6038575	NM_001031745	chrX:53457971-53458030	RIBC1

A_33_P3277043	4.1716986	3.7680402		chr10:124654056-124654115	
A_23_P128094	2.4230194	2.3900566	NM_019625	chr12:123414131-123414072	ABCB9
A_23_P312344	5.593926	5.439115	NM_016196	chr12:114260452-114260393	RBM19
A_33_P3349269	7.70289	7.8033824	NM_004671	chr18:44400958-44400899	PIAS2
A_23_P319640	6.692226	6.8839045	NR_024606	chr2:74731712-74731771	LBX2-AS1
A_23_P41987	4.923317	5.177419	NM_001496	chr5:137588395-137588336	GFRA3
A_33_P3264965	3.746304	3.6330056		chr1:116966438-116966379	
A_33_P3396612	9.33115	9.139345		chr16:33489569-33489510	
A_33_P3262485	3.9072173	4.109936	NM_145276	chr19:12429457-12429398	ZNF563
A_33_P3501900	4.1747136	3.946026	NR_037160	chr19:56910479-56910538	ZNF582-AS1
A_24_P326491	2.3221061	2.3900566	NM_173576	chr10:27962388-27962329	MKX
A_24_P203689	4.6806602	4.103836	NM_198525	chr15:90172282-90172223	KIF7
A_24_P217758	6.0045958	6.534449	NM_001242336	chr5:153436492-153436551	MFAP3
A_23_P214678	12.074901	11.981831	NM_015921	chr6:33384421-33384362	CUTA
A_23_P345887	7.1201425	6.7606516	NM_001244710	chr2:69547506-69547447	GFPT1
A_24_P298013	4.20743	4.4087796	NM_019096	chr6:43589781-43589510	GTPBP2
A_33_P3577120	7.5073686	7.8814163	BC033227	chr16:2032527-2032586	
A_23_P155556	9.972441	10.407352	NM_001040199	chr3:98234610-98234551	CLDND1
A_33_P3311210	4.1556153	3.8612373	NM_001098169	chr11:122848564-122848505	BSX
A_33_P3362397	3.5658286	3.96212	NR_103825	chr4:119552820-119552879	LOC729218
A_23_P133596	9.567532	9.125319	NM_013235	chr5:31401532-31401473	DROSHA
A_23_P387943	8.480158	8.610956	NM_032982	chr7:143004450-143004509	CASP2
A_33_P3382380	6.112818	5.975012	NM_020131	chr1:156006025-156005966	UBQLN4
A_33_P3372580	9.967492	10.021287	NM_018714	chr17:71204583-71204642	COG1
A_23_P170498	9.612997	9.9698105	NM_173474	chr16:15131933-15131874	NTAN1
A_24_P219378	5.899674	6.0399323	NM_020764	chr16:2227503-2227444	CASKIN1
A_24_P261259	10.413191	10.132268	NM_004566	chr10:6277062-6277121	PFKFB3
A_23_P14515	2.583746	2.3900566	NM_152331	chr14:74062080-74062139	ACOT4
A_33_P3348797	9.141499	9.182727	NM_001135586	chr5:134194726-134194785	C5orf24
A_23_P91468	12.640703	12.414043	NM_002792	chr20:60715987-60715928	PSMA7
A_23_P94204	7.9925804	8.268459	NM_181354	chr8:107763560-107763619	OXR1
A_23_P347059	9.749008	9.301134	NM_173468	chr4:71853584-71853643	MOB1B
A_33_P3329104	5.2362485	5.4499655		chr19:007806644-007806703	
A_33_P3355588	6.101077	5.7937055		chr2:3485111-3485052	
A_24_P393864	7.671803	7.6015034	NM_006608	chr1:114248739-114248680	PHTF1
A_33_P3374049	6.153282	6.021859	NM_138346	chr1:11983138-11983079	KIAA2013
A_23_P502269	7.900362	7.9565725	NM_032311	chr22:42980230-42980171	POLDIP3
A_23_P336198	4.740123	4.0651474	NM_138426	chr7:8128578-8128637	GLCCI1
A_23_P323227	8.990126	9.0531645	NM_001008709	chr11:67166439-67166297	PPP1CA
A_33_P3479449	5.0120397	4.8543344		chr2:102603250-102603309	LINC01127
A_33_P3244669	12.041407	11.528396	NM_018247	chr6:75963452-75963393	TMEM30A
A_23_P54605	10.044097	9.869337	NM_015659	chr16:11933775-11933716	RSL1D1
A_32_P204676	10.391012	9.475957	NM_001444	chr8:82195616-82195675	FABP5
A_24_P217572	5.221714	4.6587615	NM_001957	chr4:148465478-148465537	EDNRA
A_23_P15272	6.6035395	7.033099	NM_001079528	chr16:16315134-16315075	ABCC6
A_23_P357811	11.8051	11.45661	NM_021038	chr3:152182837-152182896	MBNL1
A_33_P3237096	5.8186913	5.9792643	NM_001243195	chr10:121551626-121551685	INPP5F
A_33_P3305840	10.993422	10.957935	NM_001195446	chr2:38976734-38976675	SRSF7
A_23_P157051	4.649636	4.1410036	NM_004722	chr7:99702720-99702920	AP4M1
A_23_P95930	8.884526	10.024526	NM_003483	chr12:66359789-66359848	HMGA2
A_24_P36097	7.2323685	7.2546473	NM_001160266	chr17:5329302-5329361	RPAIN
A_33_P3398719	6.1000805	6.2345233		chr22:22707581-22707640	
A_23_P166248	7.720968	7.949589	NM_004414	chr21:35889068-35889009	RCAN1
A_23_P24275	4.5164533	3.6661646	NR_027709	chr10:1089998-1090057	IDI2-AS1
A_23_P51711	10.128265	9.988388	NM_015849	chr1:15813857-15813916	CELA2B
A_33_P3261902	9.816582	9.949963	NM_001126123	chr18:674224-674165	ENOSF1
A_33_P3417260	7.4734387	7.3767133	NM_017881	chr9:77681733-77681674	NMRK1
A_23_P212310	9.295179	9.134692	NM_001031703	chr3:47537307-47537248	ELP6
A_33_P3308686	6.5287275	5.944304	NM_020785	chr4:15483782-15483841	CC2D2A

A_23_P83028	7.0946627	7.410453	NM_021111	chr9:36124319-36124378	RECK
A_33_P3230541	5.766141	5.6198673		chr17:016961205-016961264	
A_32_P77502	13.490389	13.554734	NM_194247	chr2:178084440-178084499	HNRNPA3
A_33_P3424462	3.3473341	3.325992	NM_001139459	chr1:246811411-246811470	CNST
A_23_P113623	10.922672	10.7253895	NM_032351	chr17:36478912-36478971	MRPL45
A_33_P3343175	2.6983678	3.410715	NM_001565	chr4:76943579-76943520	CXCL10
A_33_P3211628	8.134645	8.380923	NM_004705	chr11:76061045-76060992	PRKRIR
A_33_P3210399	3.7320383	3.2043056	NM_001146037	chr18:43329812-43329871	SLC14A1
A_23_P253896	2.6655288	2.8848116	NM_001033047	chr4:106892414-106892473	NPNT
A_23_P331700	6.027178	5.8409853	NM_001110199	chr7:75916216-75916275	SRRM3
A_23_P215658	8.895346	8.720944	NM_030900	chr7:45140022-45139963	TBRG4
A_33_P3248137	5.243911	5.434974	NM_182487	chr9:127572632-127572691	OLFML2A
A_24_P72750	9.688301	9.926301	NM_006717	chr9:91093209-91093268	SPIN1
A_24_P236251	3.662096	3.417933	NM_003836	chr14:101201048-101201107	DLK1
A_24_P924862	9.24606	8.552269	NM_213589	chr2:204298760-204298701	RAPH1
A_33_P3252834	7.2229443	7.0542707	NM_012396	chr1:201435165-201435106	PHLDA3
A_33_P3324394	3.804892	3.4883623	NM_001286403	chr16:10996598-10996657	CIITA
A_23_P372368	4.3526073	4.339291	NR_033800	chr21:40686498-40686439	BRWD1-IT2
A_24_P142228	15.143757	15.168701	NM_033251	chr16:89629329-89629388	RPL13
A_33_P3349252	6.110897	6.184138	AL512723	chr15:22011903-22011962	
A_24_P245108	5.6510477	4.431709	NM_001286458	chr16:8986423-8986364	USP7
A_33_P3409159	7.6553907	8.31629	NM_015482	chr6:3269758-3269699	SLC22A23
A_33_P3243168	9.024764	9.078285	NM_198055	chr19:59073344-59073285	MZF1
A_32_P70135	9.686546	9.237066	NM_025138	chr13:39584423-39584364	PROSER1
A_32_P101844	5.9141903	5.9711065		chr3:046065435-046065494	XLOC_014512
A_24_P367329	7.0124526	7.05478	XM_496078	chr15:78234123-78234064	LOC440292
A_33_P3369146	5.585233	5.7485943	NM_001001344	chrX:152827623-152827682	ATP2B3
A_33_P3381483	5.0431004	4.924267	NM_001253798	chr19:54083408-54083467	ZNF331
A_33_P3348927	4.207639	4.17778			
A_23_P39799	3.2424812	3.5831459	NM_032603	chr2:74761088-74761029	LOXL3
A_33_P3712341	5.0842814	5.126916	NM_001033886	chr10:44880453-44880394	CXCL12
A_33_P3315060	4.0223274	3.8812041	XM_005245645	chr1:203771403-203771462	
A_33_P3344243	9.783531	8.777382	NM_001008393	chr4:159587916-159587857	C4orf46
A_33_P3343402	3.8417912	3.5610764	NM_001037497	chr6:49986629-49986570	DEFB110
A_23_P159974	5.4361887	5.6734333	NM_033495	chrX:117032474-117032415	KLHL13
A_23_P353744	7.953775	7.832339	NM_032239	chr4:129029966-129030025	LARP1B
A_24_P343559	4.4102507	4.6285396	NM_006180	chr9:87636441-87636500	NTRK2
A_23_P6624	10.566852	10.335277	NM_000373	chr3:124462910-124462969	UMPS
A_33_P3251108	6.900463	6.8463473	NM_001190992	chr3:155546028-155544607	SLC33A1
A_23_P5831	7.7570696	7.348153	NM_134421	chr2:10566980-10567039	HPCAL1
A_23_P97906	7.2033157	7.715835	NM_012229	chr10:104848842-104848783	NT5C2
A_23_P41166	8.136269	7.1587076	NM_001038628	chr3:160802348-160802289	B3GALNT1
A_23_P68121	3.3656616	2.3900566	NM_012455	chr2:113959842-113959901	PSD4
A_33_P3399870	7.7510076	7.7251363	NM_001256126	chr2:169626697-169626756	CERS6
A_33_P3519223	8.709057	8.814234			
A_23_P201764	4.198145	4.2142463	NM_024887	chr1:26796985-26797044	DHDDS
A_33_P3439765	11.489645	11.064225	NM_024824	chr14:89079266-89079325	ZC3H14
A_23_P104607	9.850304	9.795792	NM_002804	chr11:47444493-47444434	PSMC3
A_23_P55342	8.158992	8.005846	NM_004422	chr17:7128974-7128915	DVL2
A_33_P3670415	7.8280287	7.9228106	NM_178557	chr4:2070029-2070088	NAT8L
A_23_P42507	7.6786447	7.727596	NM_139126	chr6:149833364-149833305	PPIL4
A_33_P3377763	5.5146995	5.816819	NR_027084	chr17:21909487-21909546	FLJ36000
A_24_P365954	6.6238213	6.8167267	NM_020457	chr16:67877264-67877323	THAP11
A_33_P3398564	9.158079	9.265461	NM_030649	chr1:1228309-1228250	ACAP3
A_23_P43425	7.5291553	7.4311857	NM_017998	chr9:77562057-77561998	C9orf40
A_23_P74716	8.014938	7.8956575	NM_032998	chr1:161091732-161091673	DEDD
A_23_P323924	9.7054825	9.438278	NM_001002261	chr10:99520559-99520618	ZFYVE27
A_23_P356565	9.224863	9.105363	NM_015324	chr11:6621267-6621208	RRP8
A_24_P181055	6.2056866	5.8692575	NM_006278	chr11:126283477-126283536	ST3GAL4

A_32_P178945	8.987828	8.936964	NM_018566	chr1:207217382-207217323	YOD1
A_23_P44112	10.066513	9.579995	NM_014387	chr16:29001732-29001791	LAT
A_23_P71270	5.185571	5.0694027	NM_001185	chr7:99564778-99564719	AZGP1
A_23_P143692	4.849025	4.4749575	NM_018957	chr22:38046552-38046611	SH3BP1
A_23_P205959	9.541327	9.339448	NM_000693	chr15:101456503-101456562	ALDH1A3
A_33_P3328485	4.7738457	4.9509196	NM_001079526	chr2:214012493-214012434	IKZF2
A_23_P153676	5.568107	5.833468	NM_003260	chr19:2997943-2997884	TLE2
A_33_P3372699	8.446196	8.401861	NM_174977	chr22:30885568-30885509	SEC14L4
A_24_P204244	8.834133	9.269756	NR_001562	chr4:154228642-154228621	ANXA2P1
A_24_P29723	6.881298	6.7646165	NM_000941	chr7:75615490-75615549	POR
A_23_P144465	9.098446	9.043705	NM_005443	chr4:108552893-108552834	PAPSS1
A_33_P3333187	7.5172453	7.5450573		chr1:143906039-143905980	
A_33_P3532659	2.4330802	2.3900566		chr4:187337340-187337281	F11-AS1
A_23_P399146	5.3024926	5.4186745	NM_153263	chr19:58051653-58051712	ZNF549
A_24_P42681	10.027004	10.011255	NM_002808	chr3:184023570-184023629	PSMD2
A_33_P3344765	6.4882607	6.739435		chr16:078389461-078389520	
A_23_P142969	2.4314613	3.414888	NM_021088	chr2:95848323-95848382	ZNF2
A_24_P52004	7.892565	7.899006	NM_001100399	chr4:39825142-39825083	PDS5A
A_23_P166686	10.354605	10.33584	NM_016201	chr3:134074801-134074742	AMOTL2
A_24_P350008	11.859836	11.9127655		chr7:105460053-105460112	
A_24_P104407	7.22361	7.5268216	NM_145728	chr15:99675380-99675439	SYNM
A_33_P3386760	5.4658074	5.43948	NM_145862	chr22:29121334-29121275	CHEK2
A_24_P82466	10.3182335	10.7548	NM_201433	chr17:9814278-9814219	GAS7
A_32_P47554	13.653279	13.542038	NM_005340	chr5:130495150-130495091	HINT1
A_23_P76245	2.931332	3.0685382	NM_014191	chr12:52201872-52201931	SCN8A
A_24_P357726	3.3691695	3.8902595	NM_182707	chr19:43259206-43259147	PSG8
A_33_P3362636	7.704633	7.8205647	NM_001136498	chr17:36891334-36891393	CISD3
A_23_P386356	4.3767314	4.913885	NM_138424	chr9:116856518-116856251	KIF12
A_33_P3404546	5.6201043	5.834904	NM_001271223	chr1:228565353-228565412	OBSCN
A_23_P399156	3.9297974	4.344023	NM_153701	chr19:18183032-18182973	IL12RB1
A_32_P18159	6.2958894	6.4022636	NM_181705	chr5:130535987-130536046	LYRM7
A_23_P135184	8.544758	8.73177	NM_001042368	chr9:135973271-135973212	RALGDS
A_32_P132589	2.3221061	2.3900566	NM_001004339	chr1:53360071-53360130	ZYG11A
A_33_P3265494	6.027674	5.952853	NM_001013732	chr6:47846098-47846039	PTCHD4
A_23_P218456	10.375347	9.241836	NM_012218	chr19:10800232-10800291	ILF3
A_23_P413803	7.714252	7.6282535	NM_152408	chr5:74973642-74970388	POC5
A_23_P4294	6.453229	6.6592803	NM_014519	chr17:5009470-5009411	ZNF232
A_32_P94160	4.1314936	3.0123146	NM_006252	chr1:57180117-57180176	PRKAA2
A_23_P51085	9.577155	9.403229	NM_020675	chr2:169728015-169727956	SPC25
A_33_P3391339	6.274075	6.1230345	XR_253227	chr14:19686959-19687018	
A_32_P103945	6.390808	6.4500837	NM_212543	chr3:118930995-118930936	B4GALT4
A_33_P3619221	8.601868	8.56926	NM_001281773	chr20:45839491-45839432	ZMYND8
A_23_P132856	3.7934995	2.3900566	NM_198565	chr3:196388515-196388574	NRROS
A_33_P3256391	6.421959	6.1402307	NM_139161	chr19:6467158-6467217	CRB3
A_33_P3280811	6.7214737	6.5668926	NM_144965	chr9:130488623-130488682	TTC16
A_33_P3312258	11.072892	11.185614	NM_178014	chr6:30692703-30692815	TUBB
A_23_P10685	10.20616	10.178679	NM_012267	chr19:55773787-55773728	HSPBP1
A_33_P3303464	3.949556	3.299401	NM_175871	chr19:11487330-11487389	SWSAP1
A_23_P129058	8.797929	8.851646	NM_005154	chr15:50791323-50791382	USP8
A_33_P3345796	3.736434	4.0546455	BC016863	chr6:108543500-108543441	SNX3
A_24_P371281	5.2418547	5.2461796	NM_025246	chr3:136573998-136574057	SLC35G2
A_33_P3344831	6.389991	6.513415	NM_018004	chr3:100296198-100296257	TMEM45A
A_23_P153084	8.550305	8.776428	NM_006788	chr18:9525728-9525787	RALBP1
A_24_P272073	7.843071	7.8741093		chr11:031302524-031302465	
A_33_P3384710	5.363632	5.884002	NM_018182	chr17:27085347-27085288	FAM222B
A_33_P3217704	9.143334	9.1564245	NM_025182	chr9:35104254-35104195	FAM214B
A_23_P88904	8.300143	7.874675	NM_002528	chr16:2094667-2093705	NTHL1
A_33_P3304212	8.415231	8.286297	NM_015549	chr14:65210943-65211002	PLEKHG3
A_32_P234738	13.754819	13.851727	NM_000982	chr13:27829472-27830360	RPL21

A_24_P54178	10.080393	10.141388	NM_016040	chr1:93617749-93617690	TMED5
A_24_P158385	6.7445807	6.532503	NM_138462	chr9:140477524-140477465	ZMYND19
A_23_P215070	6.1250353	6.252643	NM_018718	chr7:130036562-130036503	CEP41
A_33_P3462155	6.23512	6.0119514	AK091013	chr5:57047219-57047278	LOC401188
A_23_P417942	8.64294	8.365173	NM_001024948	chr1:94019908-94019967	FNBP1L
A_33_P3377691	8.136764	7.6360703	NM_001008393	chr4:159589571-159589512	C4orf46
A_33_P3339361	7.3894043	7.7212796	NM_199357	chr15:32928084-32928143	ARHGAP11A
A_33_P3354935	7.203074	7.1523905	NM_172212	chr1:110469302-110469361	CSF1
A_33_P3239101	4.993904	5.191717	NM_001267536	chr15:84908664-84908723	GOLGA6L4
A_23_P356484	15.526994	15.472499	NM_001014	chr6:34385296-34385237	RPS10
A_33_P3384988	5.2183614	5.37105	NM_024055	chr5:68399813-68399872	SLC30A5
A_33_P3291831	10.263792	10.648223	NM_018131	chr10:95279478-95279537	CEP55
A_33_P3564394	5.261626	3.2904103	NR_033420	chr17:28951611-28951670	SH3GL1P2
A_33_P3322945	5.150953	5.137442	NM_001008778	chr11:64940405-64940464	SPDYC
A_24_P138022	10.074147	10.147709	NM_014612	chr9:96326549-96326608	FAM120A
A_24_P64393	6.8160586	6.503394	NM_017824	chr10:94112947-94113006	MARCH5
A_23_P115683	10.202089	10.074147	NM_024747	chr10:103827675-103827734	HPS6
A_33_P3808996	9.063402	9.2526655	NM_023071	chr12:49920593-49920652	SPATS2
A_33_P3343452	3.4704924	3.875278	NM_001145029	chr18:14797793-14797852	ANKRD30B
A_33_P3474538	4.6805086	4.536459	NM_007138	chr19:20231608-20231667	ZNF90
A_24_P419028	4.7118855	4.995269	AB014771	chr4:82390257-82390198	MOP-1
A_23_P251795	4.3656516	4.6651964	NM_152742	chr7:99767426-99767367	GPC2
A_33_P3371144	5.275976	5.2445526		chr17:079454868-079454809	
A_33_P3239879	13.371204	12.9688225	NM_032356	chr17:7760064-7760005	LSMD1
A_33_P3640690	9.6098795	9.461298	NM_001128128	chr10:31816261-31816320	ZEB1
A_24_P355876	13.007282	13.0891	NM_003217	chr12:50158418-50158477	TMBIM6
A_33_P3321781	9.838178	9.443718	XR_243729	chr17:56066470-56066411	FLJ44342
A_33_P3302290	2.4730642	2.3900566		chr5:178191952-178191893	AACSP1
A_33_P3268310	2.3221061	2.3900566	NM_001205288	chr2:110656427-110656486	LIMS3L
A_33_P3224675	4.7255893	4.5451117	AK289404	chr1:100606309-100606368	TRMT13
A_23_P8561	6.060107	6.202173	NM_001040457	chr7:75517682-75517741	RHBDD2
A_33_P3221443	6.803239	6.677294	NM_001278677	chr19:24312428-24312487	ZNF254
A_24_P16378	3.9374378	3.9830022	NM_152419	chr8:43014085-43014144	HGSNAT
A_32_P118586	9.472174	9.059744	NM_152678	chr3:57611303-57611244	DENND6A
A_23_P76882	8.34443	8.249246	NM_182852	chr14:20781674-20779900	CCNB1IP1
A_33_P3262758	5.0877047	4.8319254	NM_030930	chr11:67761226-67761167	UNC93B1
A_24_P344976	3.8046186	3.5708957	NM_004312	chrX:69498456-69500068	ARR3
A_33_P3418466	4.8831086	4.8905993		chr3:133210348-133210407	
A_33_P3358745	3.1558383	3.7478096	NM_001093726	chr5:42800881-42800822	SEPP1
A_23_P410859	7.2670283	7.197201	NM_020928	chr5:60841208-60841267	ZSWIM6
A_33_P3354678	5.280503	5.567201	AF230412	chr11:5687534-5687593	TRIM78P
A_33_P3319502	9.056994	8.780723			
A_33_P3340105	5.3034134	5.4773035	NR_033774	chr13:23471724-23471665	BASP1P1
A_23_P18205	7.5201054	7.269024	NM_015106	chr3:51697368-51697427	RAD54L2
A_32_P229132	2.3221061	2.3900566	NM_020066	chr1:240637674-240637733	FMN2
A_23_P1948	10.689648	10.608776	NM_024099	chr11:62430562-62430503	C11orf48
A_33_P3389758	3.8930635	3.8339677		chrY:024085238-024085179	
A_33_P3363804	3.7575111	2.9062765	NM_001242608	chr11:113135858-113135917	NCAM1
A_33_P3289025	4.5616736	4.785174	NM_001135032	chr2:75745242-75745183	EVA1A
A_24_P273865	8.740881	8.828889	NM_004275	chr6:41873366-41873307	MED20
A_23_P134935	6.1505256	6.1586285	NM_001394	chr8:29194441-29194382	DUSP4
A_33_P3351249	5.305481	5.6156273	NM_001100812	chr17:4637866-4637807	CXCL16
A_23_P39542	7.023241	7.0025997	NM_001017927	chr2:120097484-120097425	C2orf76
A_23_P202978	3.3405485	3.385199	NM_033292	chr11:104897602-104897058	CASP1
A_23_P55256	9.266726	8.789278	NM_014897	chr17:47372664-47372605	ZNF652
A_24_P128524	8.921987	8.669269	NM_012405	chr1:6281375-6281316	ICMT
A_33_P3247473	5.312966	5.0208626	NM_181624	chr21:31720906-31720847	KRTAP23-1
A_33_P3420704	5.25791	5.0078254		chr1:143187353-143187412	
A_33_P3270599	12.435078	12.528813	NM_213674	chr9:35684303-35684244	TPM2

A_33_P3278801	4.7187953	5.1856503	AK128758	chr3:197603643-197603702	LRCH3
A_23_P83328	6.950424	6.750862	NM_000118	chr9:130579471-130578317	ENG
A_33_P3356406	4.9052334	5.2127542	NM_005633	chr2:39222352-39222293	SOS1
A_24_P221883	7.874675	8.011356	NM_018622	chr3:183560178-183560119	PARL
A_33_P3345132	3.733395	4.33646	NM_001099694	chr19:53019319-53019378	ZNF578
A_24_P60972	6.91469	7.5042887	NM_139246	chr9:100362808-100362749	TSTD2
A_32_P133518	5.060299	5.0866437	AL565221	chr2:203071219-203071278	
A_33_P3337959	7.703006	7.959534	AK128227	chr1:146090443-146090384	
A_23_P96833	6.764057	6.926665	NM_033438	chr1:159921496-159921437	SLAMF9
A_33_P3438201	7.1399984	7.3168006			
A_23_P13359	6.560277	6.627205	NM_006362	chr11:62562431-62561878	NXF1
A_33_P3272231	7.3771267	6.556939	NM_001136493	chr1:40435568-40435627	MFSD2A
A_23_P107801	7.154015	7.058788	NM_032207	chr19:16632086-16632145	C19orf44
A_23_P141345	6.9614034	6.2010627	NM_001932	chr17:41878935-41878876	MPP3
A_23_P128855	3.6576943	3.5073442	NM_014579	chr14:21469865-21469924	SLC39A2
A_23_P23141	12.832551	12.862791	NM_002107	chr1:226253364-226253423	H3F3A
A_23_P137097	7.3507013	7.5452466	NM_006517	chrX:73753514-73753573	SLC16A2
A_33_P3318646	7.5361857	7.6909347	NM_015722	chr10:135138991-135138932	CALY
A_23_P167298	3.8697941	3.8813987	NM_032149	chr4:100463178-100463237	C4orf17
A_33_P3338360	7.2620134	5.587535	NR_003002	chr14:95999751-95999692	SCARNA13
A_33_P3376527	4.8299465	4.8994846	NM_004854	chr2:101014460-101014401	CHST10
A_23_P372467	8.282781	8.554513	NM_152392	chr2:61413626-61413804	AHSA2
A_33_P3345663	4.88765	5.427053	NM_001193475	chr2:48707094-48707153	PPP1R21
A_23_P125278	2.3221061	2.3900566	NM_005409	chr4:76955674-76955615	CXCL11
A_33_P3281686	4.0324063	3.990836	NM_001077183	chr16:2100427-2100486	TSC2
A_33_P3340007	4.798608	4.8999367	NR_003268	chr13:50466616-50466674	CTAGE10P
A_32_P24372	5.890147	6.1578875	NM_001001417	chr17:34493290-34493232	TBC1D3B
A_33_P3240543	9.773872	9.571548	NM_001042535	chr7:150820665-150820724	AGAP3
A_33_P3542801	4.4772058	4.2697525	XR_242943	chr12:28341327-28341268	LOC729291
A_33_P3383287	7.6434145	7.826402	AF068294	chr9:169680-169739	
A_33_P3419970	11.526488	12.041407	NM_148896	chr17:79860717-79860776	NPB
A_23_P139099	5.5415673	5.474338	NM_001005275	chr11:55136052-55136111	OR4A15
A_23_P14564	2.3221061	2.3900566	NM_003608	chr14:88478025-88478084	GPR65
A_33_P3551349	6.288734	6.357845	AK127450	chr10:43950036-43950095	
A_33_P3237879	3.5712266	2.3900566	AK131480	chr10:9450183-9450242	
A_23_P162782	8.907711	8.793272	NM_018011	chr13:107209409-107196463	ARGLU1
A_33_P3231297	11.39463	11.486273	NM_003851	chr1:167510382-167510323	CREG1
A_23_P19650	2.423629	2.3900566	NM_003381	chr6:153077296-153077355	VIP
A_32_P36694	7.476321	5.5675597	NM_175061	chr7:27870436-27870377	JAZF1
A_23_P391857	4.252627	4.323733	NM_004452	chr14:76966256-76966315	ESRRB
A_23_P155103	9.429392	9.208988	NM_000026	chr22:40755270-40756424	ADSL
A_24_P940517	7.3700233	7.2943497	NM_016297	chr2:70506373-70506432	PCYOX1
A_33_P3211809	5.356612	5.3058987	NM_001122607	chr21:36231846-36231787	RUNX1
A_23_P309515	5.531685	5.9387007	NM_152387	chr2:201354529-201354470	KCTD18
A_33_P3321130	7.836722	8.175696	AK125506	chr8:142186290-142186349	DENND3
A_33_P3226080	3.5495105	4.1157627	NM_207103	chr17:5113969-5113910	SCIMP
A_33_P3365932	6.160348	6.1137123	NM_182905	chr9:17405-17346	WASH1
A_33_P3452003	7.9064164	8.251534	AL049428	chr10:102724881-102724822	LOC143286
A_33_P3414689	3.6949916	3.2458396	AF495723	chr3:46936260-46936319	
A_24_P23245	12.013686	12.091214	NM_002490	chr22:42483077-42482273	NDUFA6
A_33_P3319937	6.738007	6.704705	XR_159504	chr8:12523165-12523106	LOC729732
A_23_P160559	7.2213545	7.356106	NM_004425	chr1:150485797-150485856	ECM1
A_33_P3287502	10.732501	10.8095455	NM_000251	chr2:47709947-47710006	MSH2
A_33_P3214466	5.966026	6.066741	NM_018670	chr15:90293166-90293107	MESP1
A_33_P3220105	3.3118114	3.5103798	NR_034172	chr17:44271044-44271103	KANSL1-AS1
A_33_P3362503	4.035756	4.151589	NM_001101426	chr7:16415777-16415718	ISPD
A_23_P164797	8.851178	8.696886	NM_016202	chr19:56154712-56154771	ZNF580
A_23_P356616	9.091334	9.057381	NM_145804	chr11:34172801-34172742	ABTB2
A_32_P82623	6.322032	6.5179195	NM_178563	chr7:134674061-134678302	AGBL3

A_33_P3218178	4.6018667	4.5773926	NM_001012755	chrX:103343992-103343933	SLC25A53
A_24_P191417	7.9829254	8.033119	NM_005966	chr2:191557095-191557154	NAB1
A_24_P690924	7.7690716	7.8586316	NM_032985	chr20:18531782-18534900	SEC23B
A_33_P3228315	6.6745324	6.7293	NM_052819	chr17:78172496-78172555	CARD14
A_33_P3317473	9.870535	10.047238		chr2:154227466-154227525	
A_33_P3341474	5.506782	4.900118	NM_001080412	chr3:141168501-141168560	ZBTB38
A_23_P408285	8.674129	9.06436	NM_153026	chr12:42853269-42853210	PRICKLE1
A_33_P3237552	6.4006577	6.52512	NM_032843	chr9:133779394-133779335	FIBCD1
A_32_P54242	3.7160702	3.7050292	NM_153686	chr4:17845497-17845438	LCORL
A_24_P73669	7.9282217	8.608967	NM_002094	chr16:11980343-11979088	GSPT1
A_24_P915692	8.499444	8.308677	NM_007350	chr12:76419686-76419627	PHLDA1
A_32_P93149	7.3169575	6.757166	NM_004398	chr11:108594167-108709199	DDX10
A_33_P3279475	5.0450573	5.2670584	AF116680	chr8:48207039-48207098	
A_23_P2223	10.656244	10.750368	NM_002475	chr12:56551281-56551492	MYL6B
A_24_P13475	5.333091	5.451688	NM_001286792	chr13:24880407-24880466	SPATA13
A_33_P3261927	5.8098993	5.3831263	NM_213603	chr7:99085034-99085093	ZNF789
A_23_P47879	9.535366	9.502741	NM_003153	chr12:57489710-57489651	STAT6
A_23_P66306	8.916017	9.107715	NM_019109	chr16:5135196-5135255	ALG1
A_23_P428887	3.990836	3.8434577	NM_153270	chrX:21673785-21673726	KLHL34
A_23_P156310	6.2467203	6.169017	NM_032637	chr5:36170472-36170531	SKP2
A_24_P41570	13.792873	13.657412	NM_002106	chr4:100869267-100869240	H2AFZ
A_33_P3282973	4.8182755	4.8347287		chr8:104212925-104212984	BAALC
A_33_P3364348	6.660277	6.1002436	NM_020647	chr8:75147059-75147000	JPH1
A_33_P3416687	5.8288107	5.781893	XM_005256420	chr17:15580962-15580903	TRIM16
A_24_P144303	7.3143144	7.511671		chrX:98716642-98716600	XRCC6P5
A_33_P3350508	5.284042	5.577944	NM_001080432	chr16:54146840-54146899	FTO
A_32_P27479	2.3221061	2.3900566	NM_145007	chr19:56300207-56297213	NLRP11
A_33_P3417305	4.99088	5.226054		chr9:079655530-079655589	
A_23_P201711	14.973163	14.893461	NM_014624	chr1:153507685-153507255	S100A6
A_23_P92727	8.01675	8.433107	NM_015577	chr5:34831952-34832011	RAI14
A_23_P143190	8.240244	8.536125	NM_002466	chr20:42343796-42343855	MYBL2
A_23_P89727	3.63763	3.3897626	NM_048368	chr18:77475245-77475304	CTDP1
A_23_P101905	6.810757	7.101344	NM_005883	chr19:1473181-1473240	APC2
A_33_P3227842	3.2281957	4.2120757	NM_001166006	chr1:29391669-29391728	EPB41
A_33_P3365870	3.9762836	3.5410755	NM_001143888	chr1:32849509-32849450	BSDC1
A_33_P3421923	2.3221061	2.3900566	NM_021189	chr1:159172872-159172931	CADM3
A_23_P20022	8.5130415	8.49161	NM_013332	chr7:128098381-128098440	HILPDA
A_33_P3260634	7.700428	7.154489	NM_001172646	chr20:9461356-9461415	PLCB4
A_33_P3268167	8.275219	8.454165		chr10:082419955-082420014	
A_33_P3377269	4.475586	4.77281		chr11:132686819-132686760	
A_33_P3394031	3.7167966	3.7570822	NM_183008	chr1:26612015-26611956	UBXN11
A_23_P313734	9.127249	9.213035	NM_033395	chr11:93462932-93463079	KIAA1731
A_33_P3322363	2.3221061	2.3900566	NM_001123366	chr18:61627468-61627527	HMSD
A_24_P134235	3.97397	3.7774332	NM_003685	chr19:6416594-6416535	KHSRP
A_33_P3346533	5.4598413	5.3682575		chr11:090015787-090015728	
A_33_P3229022	9.302583	9.202936		chr11:018616720-018616661	
A_24_P238131	2.3221061	2.3900566	NM_015551	chr3:33192175-33192116	SUSD5
A_23_P325040	9.800841	10.114497	NM_003276	chr12:98928260-98928319	TMPO
A_33_P3299898	7.4547415	7.716153		chr10:48157044-48157103	CTSLP2
A_33_P3240972	4.370503	3.56999		chr15:23104322-23104801	LOC729900
A_23_P108835	8.541524	9.220297	NM_016061	chr2:30383128-30383187	YPEL5
A_33_P3288754	12.349457	11.788418	NM_199249	chr19:51301021-51300962	C19orf48
A_23_P56298	6.0367107	6.2294354	NM_025189	chr19:21240629-21240688	ZNF430
A_33_P3351836	4.9446282	4.9410706		chr2:54610272-54610331	C2orf73
A_24_P332647	6.2256885	6.397167	NM_001161331	chr12:109185865-109185806	SSH1
A_23_P74928	5.1722636	5.749482	NM_001531	chr1:181019346-181019405	MR1
A_33_P3365856	4.180688	4.1072693	CD674797	chr10:31608604-31608545	ZEB1-AS1
A_33_P3787904	15.311133	15.059151	XM_005273494	chr8:42183031-42183090	IKBKB
A_33_P3266873	10.21747	10.378933	NM_001012241	chr17:38292806-38292865	MSL1

A_23_P48121	9.941192	10.204974	NM_024551	chr12:1897392-1897451	ADIPOR2
A_32_P143000	6.623427	6.8943014	NM_015307	chr15:29412755-29412696	FAM189A1
A_33_P3326904	6.6592803	6.2645693	AK125166	chr7:97556709-97556650	LOC441268
A_33_P3355732	3.608161	4.361496	AB096971	chr21:43514074-43514133	UMODL1
A_23_P250294	8.255348	8.282189	NM_016006	chr3:43760031-43760090	ABHD5
A_33_P3285754	4.6027937	4.2849064		chr9:090440804-090440863	
A_23_P31335	9.158515	9.170874	NM_016265	chr7:6730358-6730299	ZNF12
A_23_P4850	4.1699886	4.6304655	NM_145233	chr19:12256413-12256354	ZNF625
A_33_P3226610	11.298914	11.170785	NM_021144	chr9:15474127-15474068	PSIP1
A_33_P3238290	2.3221061	2.3900566	NM_080829	chr20:49202794-49202735	FAM65C
A_33_P3376971	9.489122	7.9962783	NM_024111	chr15:41248631-41248690	CHAC1
A_24_P77364	11.6723385	11.73924	NM_212552	chr2:74362689-74362630	BOLA3
A_24_P134727	7.4290743	7.1994047	NM_003201	chr10:60155172-60155231	TFAM
A_23_P21324	6.968	6.7455087	NM_001271893	chr2:239832044-239832103	TWIST2
A_33_P3416037	10.010293	10.211779	NM_001014812	chr15:64380946-64380887	FAM96A
A_23_P42368	7.9324236	8.19695	NM_016485	chr6:142519744-142525172	VTA1
A_33_P3244122	3.5546281	3.413073	NM_012205	chr2:42994291-42994232	HAAO
A_32_P186731	3.1306412	3.044149	NM_080826	chr20:13280838-13280897	ISM1
A_23_P99741	5.710717	6.2467203	NM_004196	chr14:50796886-50796827	CDKL1
A_33_P3494748	6.2846656	4.345258	NM_194291	chr8:125324320-125324261	TMEM65
A_33_P3360823	7.545901	6.9017806	NM_024942	chr10:124691139-124691080	C10orf88
A_24_P310009	6.4598207	6.903425	NM_153210	chr17:9632242-9632301	USP43
A_33_P3359647	4.057425	4.228411	NM_182647	chr20:62729324-62729383	OPRL1
A_32_P76627	3.9045372	4.0103164		chr17:16722625-16722566	
A_24_P213370	4.4513555	3.8832693	AK129982	chr8:12424308-12424367	
A_24_P99838	6.4102483	6.4218698	NM_013361	chr19:44571257-44571316	ZNF223
A_23_P109547	6.4320083	6.1816583	NM_019008	chr22:39913629-39913688	MIEF1
A_33_P3248424	5.8010283	5.023862	NM_138348	chr5:14699783-14699842	FAM105B
A_23_P141770	7.7115064	7.735581	NM_003826	chr18:10540343-10546328	NAPG
A_32_P486620	3.204598	3.503176	NM_173588	chr11:18726081-18726022	IGSF22
A_33_P3319920	9.418352	9.932364	NM_015015	chr19:5153547-5153606	KDM4B
A_24_P50908	7.460016	6.9781938	AK074623	chr1:228583602-228583543	TRIM11
A_33_P3355014	4.51404	3.9785028	NM_182526	chr14:67937042-67936983	TMEM229B
A_33_P3374758	7.922201	8.302593	NM_006141	chr16:66776401-66776342	DYNC1LI2
A_33_P3371663	8.680833	9.012939	NM_002344	chr15:41795900-41795841	LTK
A_24_P935986	11.300554	10.6179905	NM_005504	chr12:24964511-24964452	BCAT1
A_23_P55064	5.013267	4.795188	NM_018081	chr17:7605086-7605699	WRAP53
A_24_P272873	14.914306	14.971375	NR_004844	chr14:56233429-56233489	RPL13AP3
A_33_P3359250	5.1304355	5.537757		chr1:11735986-11735927	MAD2L2
A_24_P133584	4.4957457	4.9261723	NM_005928	chr15:89442763-89442704	MFGE8
A_33_P3340981	4.268188	4.358812		chr13:40918228-40918169	
A_23_P131096	8.136525	7.8942914	NM_005035	chr19:617843-617784	POLRMT
A_33_P3283601	7.8247776	7.8350854		chr2:130680494-130680435	LOC389033
A_23_P401904	10.0773945	9.895347	NM_001009936	chr9:123632058-123631999	PHF19
A_32_P95914	8.605788	6.944482	NM_198468	chr6:97590265-97590206	MMS22L
A_33_P3663974	4.979901	5.5384865	NM_001252634	chr3:24164537-24164478	THRB
A_24_P160202	3.8363175	4.382538	NM_052839	chr22:50616442-50616501	PANX2
A_33_P3293573	7.6489058	7.6529865	NM_001077621	chr7:73086324-73086383	VPS37D
A_23_P45517	6.331771	6.246881	NM_013239	chrX:299601-299542	PPP2R3B
A_24_P567298	7.904107	8.68201	NM_004909		CSAG2
A_23_P410507	5.032923	5.295468	NM_004158	chr19:6375594-6375535	PSPN
A_33_P3210904	3.9706843	3.7734082	AK095629	chr3:10356718-10356659	SEC13
A_23_P133543	4.170752	4.100115	NM_017415	chr5:136953347-136953288	KLHL3
A_33_P3249135	4.972337	5.220378	NM_001114132	chr2:204082537-204082596	NBEAL1
A_33_P3276997	2.6799695	2.3900566	NM_207577	chr11:75313987-75313928	MAP6
A_24_P54808	8.266493	7.5098395	NM_020803	chr4:88082604-88082545	KLHL8
A_23_P73540	6.2277417	6.2784443	NM_033626	chrX:48927333-48927392	CCDC120
A_33_P3424591	7.123065	7.159929	BC073773	chr14:106207869-106207810	
A_24_P336759	8.121649	8.107697	NM_021960	chr1:150547681-150547622	MCL1

A_23_P336644	6.0318623	6.053719	NM_145034	chr1:179814411-179814352	TOR1AIP2
A_24_P115700	7.101152	7.659268	NM_078470	chr10:101472371-101472312	COX15
A_23_P67847	11.185614	11.342649	NM_024572	chr2:31133429-31133370	GALNT14
A_23_P76488	10.89671	10.179983	NM_001423	chr12:13369562-13369621	EMP1
A_33_P3576797	6.907802	6.758303	AL110203	chrX:73457560-73457501	LOC158863
A_23_P348264	10.1153	9.278763	NM_144652	chr8:38265970-38266029	LETM2
A_24_P200942	4.360976	4.8651037	NM_030935	chr7:100072006-100071947	TSC22D4
A_33_P3225298	4.2008724	4.6009493	NM_001011720	chr8:71646454-71646513	XKR9
A_23_P163027	9.732294	9.413418	NM_005484	chr14:20825902-20825961	PARP2
A_33_P3336992	5.4286184	5.461674	NM_014046	chr6:30587318-30587377	MRPS18B
A_33_P3362806	4.9654875	5.022897	BX647801	chrX:48834722-48834663	GRIPAP1
A_24_P321068	6.4115086	6.766923	NM_001859	chr9:116020983-116021042	SLC31A1
A_24_P276490	4.5037236	4.2730947	NM_007260	chr1:24120413-24120604	LYPLA2
A_24_P411815	9.708796	9.354691	NM_138701	chr7:40172832-40172773	MPLKIP
A_33_P3400389	10.523144	9.5499115	NM_001282735	chr2:201342855-201342914	SPATS2L
A_33_P3279581	6.6553636	6.9204493	AY358648	chr15:64665753-64665694	
A_33_P3356926	4.358812	4.8822794	NM_133174	chr5:139941788-139941729	APBB3
A_33_P3388948	9.073066	8.8300085	NM_001012994	chr9:115637058-115637117	SNX30
A_23_P2203	4.695207	4.191432	NM_016281	chr12:118610340-118610281	TAOK3
A_24_P349547	9.213874	8.981721		chr12:079187482-079187421	
A_23_P141302	7.192122	7.203576	NM_144680	chr17:11880966-11880907	ZNF18
A_23_P353514	4.8976374	4.625881	NM_078628	chrX:11786024-11786083	MSL3
A_32_P89730	5.003954	5.116659		chr12:12508475-12508416	LOH12CR2
A_33_P3283420	5.4277916	5.562982	NM_203390	chr8:94746051-94745992	RBM12B
A_33_P3278033	4.706768	5.072983	NM_001004125	chr9:25677740-25677681	TUSC1
A_24_P386334	4.7346573	4.5512533	NM_020145	chr9:131772116-131772057	SH3GLB2
A_23_P206396	10.6922455	10.553295	NM_001040138	chr16:66597028-66597087	CKLF
A_23_P205529	8.007069	7.999735	NM_022734	chr14:21464427-21464720	METTL17
A_33_P3220723	8.150258	7.8825126	NM_015196	chr4:154557763-154557822	KIAA0922
A_33_P3381781	4.508873	4.7372727		chr1:150414018-150414077	RPRD2
A_33_P3423300	10.972573	10.628368	NM_001136195	chr19:12810076-12810017	TNPO2
A_33_P3230254	6.4497633	6.537677	NM_022346	chr4:17846426-17846485	NCAPG
A_33_P3221408	8.102465	7.7272463	NM_001113226	chr1:108024337-108024396	NTNG1
A_33_P3344277	4.89599	4.947463	BC057389	chr2:20834599-20834540	HS1BP3
A_33_P3503537	8.356657	7.569879	AK091571	chr2:217081345-217081404	LOC285178
A_33_P3375145	2.3221061	2.3900566	NM_203403	chr9:12822833-12822892	LURAP1L
A_23_P109269	5.2580423	5.10911	NM_005560	chr20:60885361-60885302	LAMA5
A_23_P167168	4.4611893	4.501444	NM_144646	chr4:71521933-71521874	IGJ
A_23_P9415	11.462726	11.681082	NM_002197	chr9:32450545-32450604	ACO1
A_23_P129513	10.682583	10.483363	NM_006428	chr16:418350-417746	MRPL28
A_24_P255314	11.090216	11.258942		chr12:006547844-006547903	
A_32_P117422	7.560105	7.6633196	NR_022007	chr7:66764296-66762256	PMS2P4
A_23_P64650	10.468208	10.3624525	NM_005726	chr12:58190361-58190420	TSFM
A_23_P93690	11.210442	11.063074	NM_182776	chr7:99690579-99690520	MCM7
A_32_P98975	5.9509335	6.196733	NM_052849	chr15:40849479-40849420	C15orf57
A_24_P280926	9.724287	9.634634		chr2:26251396-26251337	
A_23_P396917	6.2192936	6.0409646	NM_199124	chr11:122776178-122776237	C11orf63
A_33_P3372941	9.1564245	9.287131	NM_012461	chr14:24709062-24709003	TINF2
A_33_P3360728	13.207613	13.328317	NM_000713	chr19:40953760-40953701	BLVRB
A_33_P3329419	9.427089	9.473838	NM_004408	chr9:131013158-131013217	DNM1
A_33_P3417920	3.8128345	4.3293695	NM_003886	chrX:49957774-49957715	AKAP4
A_24_P183264	10.754381	10.795097	NM_001037637	chr5:72794959-72795018	BTF3
A_23_P320250	10.669281	10.083246	NM_025109	chr17:34851731-34851672	MYO19
A_33_P3268989	5.5173454	5.206815		chr10:073964231-073964172	
A_23_P17880	4.4799194	4.8326592	NM_005740	chr22:39175618-39175559	DNAL4
A_33_P3261197	3.7727985	4.6165667	NM_002486	chr9:100410445-100410504	NCBP1
A_24_P370471	5.5054345	5.7515745	NM_031482	chr5:81354316-81354375	ATG10
A_33_P3338166	8.769144	8.965092	NM_030952	chr1:205271252-205271193	NUAK2
A_23_P395426	3.332469	3.7882981	NM_022105	chr20:61536759-61536700	DIDO1

A_33_P3390731	6.085234	6.530013	NM_001012398	chr16:53526086-53526027	AKTIP
A_33_P3372161	14.160603	13.529404		chr7:156985351-156985410	
A_33_P3239854	5.3807898	5.179677	NM_017758	chr17:18111754-18111813	ALKBH5
A_33_P3310696	9.108971	8.872437	NM_194458	chr1:1190644-1190585	UBE2J2
A_33_P3345474	6.4473295	6.630571	BC036832	chr22:46501501-46501560	
A_23_P213085	4.4401126	4.1330185	NM_001029998	chr4:147175938-147175879	SLC10A7
A_23_P127088	9.923078	9.702502	NM_024040	chr10:104183423-104183280	CUEDC2
A_23_P386268	2.9081366	3.9681444	NM_178497	chr4:76489487-76489546	C4orf26
A_33_P3246193	3.569162	3.5340247	NM_001200049	chr10:134735674-134735615	TTC40
A_23_P211196	3.8494859	3.641938	NR_027129	chr21:46354940-46354881	C21orf67
A_33_P3415430	8.785837	8.392736	NM_005346	chr6:31797870-31797929	HSPA1B
A_23_P33326	4.8533354	4.987467	NM_000679	chr5:159399943-159400002	ADRA1B
A_33_P3386467	9.189386	9.272802	NR_024530	chr2:114379385-114379326	RPL23AP7
A_23_P217755	2.3221061	2.3900566	NM_001649	chrX:9916998-9917057	SHROOM2
A_32_P108156	4.2085934	4.368355	NR_001458	chr21:26946350-26946409	MIR155HG
A_33_P3217218	6.6991687	6.4612007	NM_018361	chr8:6618891-6618950	AGPAT5
A_23_P90533	9.5531435	8.933028	NM_006627	chr19:30106427-30106486	POP4
A_23_P24903	7.010354	6.6049633	NM_176072	chr11:72946916-72946975	P2RY2
A_23_P7074	9.860092	9.689207	NM_032313	chr4:57829743-57829684	NOA1
A_24_P105564	7.639557	7.5741014	NM_005399	chr1:146626925-146626866	PRKAB2
A_33_P3390027	7.8048964	7.7928414	AK022397	chr17:74088767-74088708	EXOC7
A_24_P476086	4.326833	4.6792064		chr6:117056632-117056691	KPNA5
A_33_P3877728	7.566716	7.647891	BX647090	chr17:17729676-17729617	SMCR6
A_32_P140489	2.4466717	2.3900566	NM_001001557	chr8:97154669-97154610	GDF6
A_33_P3325978	7.1907234	7.048508		chr2:012864859-012864918	
A_23_P37415	5.5978923	5.7330456	NM_014701	chr15:49284474-49284415	SECISBP2L
A_33_P3318384	3.6839693	2.3900566		chrX:055535190-055535131	
A_33_P3339231	6.3015623	5.7831755	NM_173569	chr7:138992885-138992944	UBN2
A_33_P3311979	7.1863832	7.2132945	NM_153813	chr16:88601304-88601363	ZFPM1
A_24_P130041	9.739573	9.794039	NM_000786	chr7:91741495-91741463	CYP51A1
A_33_P3244610	5.812622	5.9078116	NM_178460	chr20:1532397-1532338	SIRPD
A_33_P3700794	6.7766104	6.524379	NM_005371	chr12:58163171-58163112	METTL1
A_33_P3285195	2.6642969	2.3900566	XR_109821	chr4:95665017-95664958	LOC100507012
A_33_P3286724	9.892289	10.036879	NM_002663	chr17:4726661-4726720	PLD2
A_33_P3394203	5.303042	5.570007		chr6:43481455-43481396	YIPF3
A_33_P3271171	6.5457497	6.805592	AL133647	chr19:54657070-54657129	CNOT3
A_24_P330971	10.851455	10.916527	NM_013234	chr19:39114821-39116710	EIF3K
A_24_P13715	6.1473427	6.36357	NM_147188	chr15:76225851-76225910	FBXO22
A_33_P3314231	5.9827003	6.230054	NM_057093	chr2:219854976-219854917	CRYBA2
A_33_P3282898	6.1551075	6.0949054	BC020881	chr9:35723457-35723398	TLN1
A_33_P3417980	4.340867	4.533799	XM_005255951	chr16:67701487-67701546	C16orf86
A_24_P824592	7.0902624	7.404065	NM_002139	chrX:135957662-135957516	RBMX
A_24_P302802	8.268309	8.488623	NM_000532	chr3:136035807-136035866	PCCB
A_24_P87931	8.638963	8.841984	NM_145343	chr22:36663090-36663149	APOL1
A_33_P3346108	4.5921555	4.4652786	NM_198483	chr2:218954677-218954736	RUFY4
A_33_P3221568	7.9103703	8.087241	NM_024742	chr16:31476392-31476451	ARMC5
A_23_P78685	8.430808	8.171041	NM_004461	chr19:13034973-13033650	FARSA
A_24_P41975	4.0868816	4.7969613	NM_001083965	chr1:151746339-151746280	TDRKH
A_24_P48587	7.2666388	7.5557213	NM_172341	chr19:36237629-36237688	PSENEN
A_23_P58647	11.015629	11.037768	NM_001903	chr5:138270387-138270446	CTNNA1
A_23_P321473	6.215446	6.1584425	NM_152660	chr1:28087851-28087910	FAM76A
A_24_P143440	8.983403	9.189386	NM_014183	chr20:33114088-33114147	DYNLRB1
A_23_P337729	5.923638	5.9812803	NM_024789	chr10:104236659-104236718	TMEM180
A_33_P3262205	8.669977	8.745718	NM_003915	chr20:34214635-34214576	CPNE1
A_33_P3230399	6.5315247	6.457785	NM_203374	chr19:56132167-56132108	ZNF784
A_23_P398770	7.568716	7.5862675	NM_001130864	chr5:159518862-159518803	PWWP2A
A_33_P3422248	3.5230787	4.219474	XM_005258130	chr18:5895116-5895057	TMEM200C
A_33_P3703637	5.7601995	5.390748		chrUn_gl000222:86032-86091	LOC285300
A_33_P3391005	5.6480737	6.108982	NM_001144967	chr18:56016784-56016843	NEDD4L

A_32_P196193	6.2939854	6.660277	NM_198504	chr3:142680265-142680206	PAQR9
A_33_P3222139	6.6116123	6.653281	NM_001005291	chr17:17715363-17715304	SREBF1
A_24_P39211	5.3364983	5.4792843	NM_198219	chr13:111372118-111372177	ING1
A_24_P372553	7.5530214	7.601702	NM_015608	chr10:127451899-127451958	C10orf137
A_33_P3405114	5.5661325	5.660815	NM_198573	chr9:34521416-34521357	ENHO
A_33_P3221828	8.410017	8.298243	AK056228	chr12:3407385-3407326	
A_23_P256773	9.125559	9.066474	NM_003310	chr2:3192995-3192936	TSSC1
A_33_P3365047	4.6436534	4.632022	NM_006606	chr20:18470505-18470446	RBBP9
A_23_P169278	8.551771	8.353381	NM_015239	chr9:88161772-88161713	AGTPBP1
A_33_P3252093	11.173113	10.937227	NM_006827	chr14:75598261-75598202	TMED10
A_33_P3368049	8.123934	8.002896	NM_001242659	chr1:1533447-1533388	C1orf233
A_24_P191656	6.451957	6.477545	NM_014049	chr3:128629616-128631368	ACAD9
A_24_P289178	8.8604355	8.472649	NM_206967	chr16:85741285-85741226	C16orf74
A_24_P102920	3.1507497	3.7122426	NM_001006622	chr2:128521238-128521179	WDR33
A_33_P3267651	6.3334265	6.32163	NM_014648	chr3:108351859-108351918	DZIP3
A_33_P3397613	4.1695805	4.3277626		chr1:155166684-155166743	
A_33_P3346032	4.042116	4.3661175	AK124942	chr15:30115879-30115938	
A_24_P232696	9.869337	9.890113	NM_139071	chr12:50494275-50494334	SMARCD1
A_23_P4662	11.184292	11.544801	NM_005178	chr19:45263191-45263250	BCL3
A_33_P3304764	4.476927	4.522993		chr1:243231070-243231011	
A_33_P3422479	3.6174834	3.652008	NM_001031717	chr3:9985671-9985730	CRELD1
A_33_P3318444	8.585315	8.471275	NM_001146684	chr17:8296576-8296517	RNF222
A_33_P3288824	3.699401	3.8406975	NM_080720		H2AFB3
A_33_P3251860	3.920118	3.769352	AF130091	chr5:53346966-53347025	
A_23_P119936	3.9848897	3.5402727	NM_138938	chr2:79384367-79384308	REG3A
A_24_P136551	7.046368	6.907656	AB040932	chr17:79528982-79528932	NPLOC4
A_33_P3319943	9.531587	9.8006935	NM_000489	chrX:76872141-76872082	ATRX
A_23_P115036	6.3431454	6.3316355	NM_014652	chr1:44432620-44432679	IPO13
A_24_P273253	5.347445	5.0692396	NM_138420	chr14:105404612-105404553	AHNAK2
A_24_P9883	5.0549912	4.184057	NM_138368	chr11:65544099-65544040	AP5B1
A_23_P501822	8.3233595	8.898809	NM_002230	chr17:39911209-39911150	JUP
A_33_P3220545	3.6026454	3.6872387	NM_001003841	chr5:1223316-1223375	SLC6A19
A_33_P3238969	4.5883703	4.643293	NM_001080488	chr19:1775293-1775352	ONECUT3
A_33_P3266848	5.8163853	6.4803514		chr12:048954372-048954431	
A_33_P3321225	4.179731	4.83057		chr20:35141071-35141012	
A_33_P3299796	3.2460954	4.475586	XM_005276366	chr9:139997703-139997762	MAN1B1
A_33_P3349469	10.589095	10.263947	NM_018188	chr1:1469991-1470050	ATAD3A
A_33_P3329088	3.7292404	4.2744513	NM_002773	chr16:31142813-31142754	PRSS8
A_33_P3219965	9.929283	10.332307	NM_006019	chr11:67818227-67818286	TCIRG1
A_23_P140830	4.33646	4.4246993	NM_024712	chr16:67237298-67237444	ELMO3
A_33_P3653888	8.339475	8.621122	AF274944	chr2:688234-688293	LOC100128185
A_33_P3294297	9.018806	8.927748	NM_194460	chr19:647894-647835	RNF126
A_33_P3325933	5.757711	5.2134404	NM_144495	chrX:48755250-48755309	PQBP1
A_23_P300905	8.595203	8.554984	NM_145012	chr10:35858729-35858788	CCNY
A_33_P3270360	5.0188284	5.49247	NM_001004458	chr11:57982993-57983052	OR1S1
A_33_P3257568	4.7643476	4.9141607	AK097636	chr9:123912863-123912922	CNTRL
A_32_P75094	9.654087	9.093838	NM_032797	chr10:71872124-71872065	AIFM2
A_24_P389491	5.5233126	5.4286184	NM_016035	chr9:131095778-131095837	COQ4
A_33_P3413845	5.7582965	5.0016346	NM_012458	chr19:2425815-2425756	TIMM13
A_24_P380536	8.156484	8.930357	NM_006016	chr6:109691645-109690193	CD164
A_33_P3215720	8.112614	7.583286	NM_006241	chr3:195245974-195245916	PPP1R2
A_23_P66137	4.8249464	5.1304355	NM_014587	chr16:1036893-1036952	SOX8
A_24_P252497	8.395726	8.977334	NM_025195	chr8:126450454-126450513	TRIB1
A_23_P28012	4.429683	3.955484	NM_033196	chr19:20117991-20117932	ZNF682
A_23_P148308	11.472702	11.619747	NM_006743	chrX:48436055-48436114	RBM3
A_33_P3315190	6.4130936	6.4560432	NM_001282666	chr11:12281558-12281617	MICAL2
A_33_P3254756	6.7141123	6.4208064	NR_015439	chr4:68588163-68588222	UBA6-AS1
A_33_P3256773	8.616004	8.564386	NM_021218	chr9:115449528-115449469	INIP
A_23_P47565	13.86687	14.442208	NM_005566	chr11:18428789-18428848	LDHA

A_24_P113824	8.669373	8.37276	NM_014313	chr1:25688274-25688333	TMEM50A
A_33_P3266609	4.374866	4.363854		chr6:108478414-108478355	
A_23_P96291	2.3414683	2.3900566	NM_004988	chrX:152481769-152481710	MAGEA1
A_33_P3344282	4.697659	4.917361	BC038847	chr2:20839872-20839813	HS1BP3
A_33_P3653330	7.241024	7.376853	NM_001281435	chr17:12045140-12045199	MAP2K4
A_33_P3245160	6.6363754	6.7106256		chr17:003057372-003057313	
A_23_P161446	10.870545	10.89671	NM_001494	chr10:5807737-5807678	GDI2
A_23_P54006	10.770266	10.418505	NM_015382	chr14:31569500-31569441	HECTD1
A_33_P3274164	7.949589	7.839519	XM_005259660	chr19:2228287-2228346	DOT1L
A_33_P3357227	3.627508	2.8690095		chr2:096190771-096190712	
A_23_P101699	4.630637	4.666773	NM_032631	chr19:4498020-4498353	HDGFRP2
A_32_P220739	6.0434184	5.7575197	NM_033160	chr9:40771549-40771490	ZNF658
A_33_P3344956	3.980443	4.817397	AK094926	chr18:54306170-54306111	TXNL1
A_23_P151975	4.2286606	4.6055937	NM_016321	chr15:90014814-90014755	RHCG
A_33_P3240637	3.293521	3.535669	AK124325	chr4:170949842-170949901	
A_23_P78734	8.149929	8.5737095	NM_001077186	chr19:50813317-50813376	MYH14
A_24_P309360	10.770705	11.238366	NM_020123	chr10:98281324-98281265	TM9SF3
A_33_P3344477	10.411342	10.630358	NM_001134999	chr14:53324082-53324023	FERMT2
A_33_P3340454	3.823917	3.9434755		chr13:036857632-036857573	
A_33_P3268868	2.3221061	3.0130858		chr1:143738162-143738103	
A_32_P138617	6.7701535	5.9239902	NM_001009899	chr3:113367573-113367514	KIAA2018
A_33_P3333224	7.706561	7.652466	NM_032575	chr16:4389536-4389595	GLIS2
A_33_P3233437	4.1157627	4.0434303		chr18:051668758-051668817	
A_23_P253752	8.633617	8.596901	NM_138419	chr6:136552451-136552392	MTFR2
A_24_P196372	4.293103	4.2234836	NM_001035507	chr2:27278723-27278782	AGBL5
A_23_P59481	6.973555	7.0356708	NM_014671	chr7:157061113-157061172	UBE3C
A_23_P16976	10.373404	10.742546	NM_001153	chr2:70052749-70052808	ANXA4
A_33_P3304603	7.176229	7.5984583	NM_020338	chr10:81073917-81073976	ZMIZ1
A_23_P170839	11.9243355	12.213637	NM_001008491	chr2:242293007-242293066	SEPT2
A_33_P3315331	7.6063466	8.854466	NM_024877	chr19:40728870-40728811	CNTD2
A_32_P16007	2.3221061	2.3900566	NM_207355	chr15:21066542-21066483	POTEB
A_33_P3279241	5.1083612	5.6757274	NM_020747	chr5:123979277-123979218	ZNF608
A_33_P3342957	7.9582644	8.097064	NM_004226	chr2:197002230-197002171	STK17B
A_23_P77135	4.300596	5.1072583	NM_080650	chr15:35664176-35664117	DPH6
A_33_P3323413	2.3221061	2.3900566	NR_026731	chr14:29262313-29262372	C14orf23
A_24_P336417	8.806864	9.072344	NM_004736	chr1:180854901-180854960	XPR1
A_33_P3389188	10.771717	10.556958	NM_001270782	chr10:60147960-60148019	TFAM
A_24_P38081	6.6722155	6.632247	NM_004117	chr6:35541987-35541928	FKBP5
A_33_P3382730	5.179487	5.009262	NM_015155	chr10:910190-910131	LARP4B
A_23_P200396	5.475458	5.712869	NM_019118	chr1:32682076-32682017	TMEM234
A_24_P203502	6.5268483	6.1131067		chr18:055505867-055505926	
A_32_P72940	11.083038	10.936483	NM_007209	chr9:127624207-127623794	RPL35
A_23_P9523	7.5386486	7.9064164	NM_022128	chr2:28004544-28004485	RBKS
A_24_P65910	10.0250435	10.028687	NM_018244	chr20:33890615-33890556	UQCC1
A_24_P152983	13.112333	13.459215		chr5:132279661-132279720	
A_23_P154832	11.049772	11.109577	NM_001003703	chr21:27101958-27097619	ATP5J
A_33_P3319940	6.9020376	7.0152373	NM_000489	chrX:76944381-76944322	ATRX
A_33_P3399474	4.128544	4.3161564		chr2:114647278-114647337	
A_33_P3413335	4.7610846	4.097742	AF390550	chr16:3433611-3433670	
A_23_P99927	11.781786	11.366173	NM_016395	chr15:65864606-65864665	PTPLAD1
A_23_P47226	9.98192	10.289828	NM_020470	chr11:66052184-66052125	YIF1A
A_24_P347704	7.0637445	7.4998083	NM_006565	chr16:67672114-67672173	CTCF
A_33_P3228023	3.6969368	3.8518124	NM_021626	chr17:55065591-55065650	SCPEP1
A_33_P3358938	6.372274	6.0912046	NM_001267843	chr4:56301090-56301031	CLOCK
A_23_P125815	9.49333	9.548752	NM_005676	chrX:47045914-47045973	RBM10
A_24_P942604	6.8875256	6.8202515	NM_006306	chrX:53401212-53401153	SMC1A
A_33_P3223239	5.022994	4.9666862	NM_138729	chr1:113121027-113120968	ST7L
A_33_P3411427	6.2955265	6.7861457	NM_138466	chr19:58879052-58878993	ZNF837
A_33_P3390823	7.065818	6.68779		chr7:000208930-000208989	

A_23_P99292	8.267062	8.125544	NM_006479	chr12:4668182-4668241	RAD51AP1
A_33_P3320062	3.732829	4.233818	NM_002662	chr3:171330156-171330097	PLD1
A_23_P109452	7.274812	7.225155	NM_001005735	chr22:29090020-29085145	CHEK2
A_33_P3285038	8.956146	9.058923	NM_001281731	chr12:50523759-50523700	CERS5
A_33_P3445679	4.402399	4.549224		chr13:40768705-40768646	LINC00548
A_24_P143843	6.7209034	6.6844144		chr4:151642340-151642399	
A_33_P3384260	8.775738	8.683146	XM_005268826	chr12:54680787-54680846	HNRNPA1
A_33_P3255782	12.27826	12.318008		chr5:133758662-133758721	XLOC_014512
A_33_P3253628	4.8201756	4.8914747	AK093737	chr5:177638061-177638002	PHYKPL
A_23_P146209	6.382354	6.8116446	NM_030954	chr8:42711504-42711445	RNF170
A_24_P333802	10.160583	9.542019	NM_032121	chrX:77081964-77081905	MAGT1
A_23_P355447	5.6459565	5.5980997	NM_174976	chr14:77597916-77597857	ZDHHC22
A_23_P107307	5.330603	5.4057217	NM_003984	chr17:26820663-26820722	SLC13A2
A_23_P373119	8.922867	9.025269	NR_002165	chr20:33421521-33421462	HMGB3P1
A_23_P93988	7.131621	7.0869327	NM_005435	chr7:144077494-144077553	ARHGEF5
A_33_P3399718	6.589916	6.731408		chr1:113291032-113291091	LOC128322
A_23_P127195	8.256001	8.150714	NM_003675	chr10:13672451-13672510	PRPF18
A_23_P82975	5.4065456	5.4119425	NM_181661	chr8:100128083-100133444	VPS13B
A_33_P3304576	4.026166	4.398632	NM_001001480	chr11:1651668-1651727	KRTAP5-5
A_23_P328034	5.6128798	5.5669584	NM_153269	chr20:257767-257708	C20orf96
A_24_P58037	7.273	7.197453	XM_373277	chr9:19461168-19461109	LOC392288
A_33_P3390673	3.78409	3.9350274		chr10:38711939-38711998	XLOC_014512
A_23_P131348	7.4886293	7.4604635	NM_025264	chr2:39963924-39963865	THUMPD2
A_24_P190541	6.9590096	6.9787235	NM_018963	chr21:40568557-40568498	BRWD1
A_24_P314571	7.57953	7.248744	AK075287	chr19:11257053-11256994	SPC24
A_33_P3416142	6.7205386	6.8228855		chr1:198868184-198868125	MIR181A1HG
A_33_P3331242	11.92761	11.865379	NM_005754	chr5:151184822-151184881	G3BP1
A_23_P330999	4.4380956	4.7729607	NM_015979	chr6:131895277-131895218	MED23
A_23_P212159	2.3221061	2.3900566	NM_024923	chr3:13358515-13358456	NUP210
A_33_P3267502	8.151702	7.6700344	NM_001009941	chr10:5920117-5920058	ANKRD16
A_32_P74366	6.930388	5.508082	AF088033	chr8:67540976-67540917	VCPIP1
A_23_P321377	5.7479324	5.3141255	NM_152374	chr1:36180163-36180104	C1orf216
A_23_P99811	7.9649215	8.125771	NM_138476	chr14:24683326-24683267	MDP1
A_23_P338603	7.569415	7.5709147	NM_001011667	chr8:57127218-57128998	CHCHD7
A_24_P175427	6.711199	6.8721066	NM_199177	chr9:125042817-125042876	MRRF
A_33_P3373364	8.945919	8.996651	NM_013943	chr1:25167306-25167365	CLIC4
A_33_P3297155	6.634968	6.5154157	NM_003616	chr14:39606093-39606152	GEMIN2
A_24_P382489	7.040091	7.5372605	NM_198580	chr19:17616917-17616976	SLC27A1
A_24_P144601	5.353264	5.3608217	NM_002701	chr6:31133368-31133025	POU5F1
A_23_P319005	10.558288	10.669943	NM_015980	chr5:173535800-173535859	HMP19
A_33_P3251989	8.006344	8.182419	NM_014171	chr2:46851950-46852009	CRIPT
A_33_P3335571	4.591036	4.1496	NR_029192	chr11:71576749-71576690	LOC100133315
A_23_P90223	5.034026	5.067606	NM_152279	chr19:37676478-37676419	ZNF585B
A_33_P3542911	4.7981105	4.697501	BF570948		SNAR-C4
A_32_P52785	4.9679008	5.0017734	NM_015345	chr6:39872302-39872361	DAAM2
A_33_P3281785	3.2517838	2.3900566	NM_006167	chr8:23538095-23538036	NKX3-1
A_24_P374319	6.951689	6.9630666	NM_021183	chrX:131338037-131337978	RAP2C
A_23_P427114	4.818762	4.578207	NM_031899	chr3:39139039-39138980	GORASP1
A_23_P48835	10.482243	10.763625	NM_138555	chr15:69740524-69740583	KIF23
A_33_P3329607	5.566563	5.418165	NM_030981	chr11:66043518-66043577	RAB1B
A_23_P200073	5.8213177	5.657012	NM_020362	chr1:24112839-24112898	PITHD1
A_23_P201605	4.5184884	4.6034927	NM_144977	chr1:197564380-197552367	DENND1B
A_33_P3270009	4.9982147	4.8900676	NM_147223	chr2:24993512-24993571	NCOA1
A_24_P120734	2.3221061	2.3900566	NR_038407	chr7:47805819-47805878	LINC00525
A_23_P203819	10.517489	9.649965	NM_005895	chr12:133345737-133345678	GOLGA3
A_33_P3423610	10.113346	10.393925	NM_004261	chr1:87329101-87329042	SEP15
A_33_P3229181	9.152817	9.087137	NM_020246	chr7:100464565-100464624	SLC12A9
A_23_P151529	5.976597	6.0383997	NM_001252507	chr14:96559848-96559907	C14orf132
A_33_P3225948	5.2195034	5.172125	NM_001042478	chr1:4837750-4837809	AJAP1

A_33_P3404922	3.9565358	3.8586814	NM_001282770	chr10:24813297-24813356	KIAA1217
A_23_P23996	3.769352	3.6341422	NM_000429	chr10:82031792-82031733	MAT1A
A_23_P207194	3.8218002	3.9272358	NM_000515	chr17:61994859-61994800	GH1
A_23_P305245	2.8361146	2.3900566	NM_001168214	chr3:159945373-159945432	C3orf80
A_24_P918843	3.9399173	3.3138983	NM_194301	chr14:36191041-36190991	RALGAPA1
A_33_P3261132	5.7808294	5.522471	NR_001560	chr1:157098403-157098462	CYCSP52
A_33_P3317761	6.8217072	6.4723024		chr12:025070755-025070696	
A_24_P83899	12.815836	13.535304	NM_000842	chr11:88241832-88241773	GRM5
A_23_P362637	7.593309	7.8574986	NM_015255	chr6:42657337-42657396	UBR2
A_33_P3407675	9.47298	9.430641	NM_001031800	chr1:168160678-168160737	TIPRL
A_33_P3368925	3.9995866	4.376402		chr19:36426619-36426678	LRFN3
A_23_P211007	11.103252	11.49802	NM_003489	chr21:16333963-16333904	NRIP1
A_23_P109881	4.4064527	4.4513555	NM_002218	chr3:52847378-52847319	ITIH4
A_33_P3282394	10.492539	9.577562	NM_005934	chr19:6210517-6210458	MLLT1
A_23_P140309	10.756146	10.427385	NM_144578	chr14:55532448-55532507	MAPK1IP1L
A_24_P254965	6.36357	6.3850484	NM_018486	chrX:71788605-71787823	HDAC8
A_23_P217178	2.8728645	3.0135732	NM_005462	chrX:140996897-140996956	MAGEC1
A_23_P319133	9.618252	9.422632	NM_018981	chr2:183643075-183643134	DNAJC10
A_24_P99795	7.2008214	7.0806174	NM_024710	chr19:55966402-55964743	ISOC2
A_23_P23411	8.225903	8.248035	NM_005973	chr1:156764473-156764532	PRCC
A_33_P3364205	8.383715	8.418493		chr7:72304461-72304520	SBDSP1
A_23_P18649	7.6440005	8.429056	NM_024582	chr4:126413229-126413288	FAT4
A_33_P3593546	10.633954	10.617237	NM_001128918	chr14:103969522-103969581	MARK3
A_24_P213783	15.829811	15.845007	NM_000993	chr2:101622498-101622835	RPL31
A_33_P3411296	8.583052	7.792239	NM_005859	chr5:139496212-139496271	PURA
A_24_P194017	7.9460125	7.5497885	NM_015953	chr19:50060250-50060191	NOSIP
A_33_P3267320	4.9297457	4.5484424	NM_052859	chr3:53124962-53124903	RFT1
A_23_P353574	6.351428	6.6341853	NM_133494	chr1:198288674-198288733	NEK7
A_23_P212728	8.1922865	8.545389	NM_001199198	chr3:100043741-100043800	TBC1D23
A_32_P218228	5.6521792	5.8020043	NM_001002034	chr22:42474754-42474813	FAM109B
A_33_P3367316	3.945817	3.268444	NR_004859	chr15:85185635-85185694	SCAND2P
A_33_P3365267	5.225788	5.677155		chr3:046791581-046791522	
A_33_P3398927	6.240066	6.0978947	NM_001204151	chr18:47799107-47799048	MBD1
A_33_P3274397	7.632475	7.5530214	NM_001145414	chrX:85224229-85224170	CHM
A_23_P137173	3.4191144	3.6382713	NM_021992	chrX:101768766-101768707	TMSB15A
A_23_P78383	7.847164	7.938525	NM_013326	chr18:21110361-21110420	C18orf8
A_23_P202720	6.958363	6.6969976	NM_018389	chr11:45834367-45834426	SLC35C1
A_23_P134113	7.9228106	6.746364	NM_052831	chr6:133090609-133090550	SLC18B1
A_24_P318593	6.656313	6.6317334	NM_138355	chr17:45916046-45915987	SCRN2
A_33_P3348046	3.8989344	3.6310792		chr1:220109948-220109889	
A_24_P21752	6.08158	5.9591675	NM_032811	chr11:124500731-124501209	TBRG1
A_33_P3311458	6.353525	6.6260314		chr12:094944157-094944216	
A_33_P3361811	6.9163713	6.8875256	NM_024561	chr13:41936235-41936294	NAA16
A_33_P3526315	6.457785	6.2198424	BC005372	chr3:33590679-33590620	MGC12488
A_33_P3304293	3.9990954	3.8231695	NM_001042751	chrX:123159708-123159767	STAG2
A_24_P292470	5.496832	5.7500052	NM_022803	chr11:73714957-73714898	UCP3
A_33_P3428642	6.944482	6.9644737	NM_004943	chr19:46287419-46287360	DMWD
A_23_P75310	9.315378	8.7361145	NM_021226	chr10:49654256-49654197	ARHGAP22
A_23_P107795	11.509441	11.510384	NM_018035	chr19:41937330-41937271	ATP5SL
A_23_P34568	6.6009264	6.7773056	NM_017825	chr1:36558810-36558869	ADPRHL2
A_33_P3270657	8.821939	8.718547	NM_198947	chr11:58894801-58894860	FAM111B
A_23_P96041	8.780476	8.592503	NM_032227	chrX:109420782-109420841	TMEM164
A_33_P3407985	4.554378	4.1752224	NM_001195386	chr17:38992463-38992522	TMEM99
A_33_P3308949	7.1071124	5.8980055	NM_001918	chr1:100652543-100652484	DBT
A_32_P174365	8.179239	7.494179	NM_015265	chr2:200134506-200134447	SATB2
A_33_P3328123	7.3618717	7.6992087		chr12:093429166-093429225	
A_33_P3404123	4.7344704	4.9791465	NM_001277332	chr17:39432274-39432333	KRTAP9-7
A_33_P3336384	4.7987895	4.9235854	NM_207127	chr10:135193847-135193906	PAOX
A_33_P3337931	5.607813	5.557396		chr1:144898170-144898111	

A_33_P3422233	8.171221	7.9831085	NM_020894	chr4:1381673-1381732	UVSSA
A_24_P942068	7.1926804	7.380995	NM_025185	chr17:61504717-61504776	TANC2
A_23_P204417	6.89246	6.845862	NM_018463	chr12:2933848-2933907	ITFG2
A_23_P79703	10.549927	10.993422	NM_000183	chr2:26512913-26512972	HADHB
A_33_P3360197	2.842342	2.3900566		chr6:90661649-90661708	
A_23_P26124	2.3221061	2.3900566	NM_134260	chr15:60789716-60789657	RORA
A_33_P3297141	7.907981	8.149929	NM_020839	chr3:39137740-39137799	WDR48
A_33_P3682006	7.5676713	7.342049	NR_102735	chr9:136519773-136519714	DBH-AS1
A_23_P80048	7.038493	7.46159	XR_248924	chr20:34146579-34146520	
A_33_P3408212	11.1672	11.461009	NM_003299	chr12:104337650-104340452	HSP90B1
A_23_P70201	9.007671	9.164589	NM_001270	chr5:98191985-98191926	CHD1
A_23_P48669	11.809391	11.8051	NM_005192	chr14:54878384-54882635	CDKN3
A_33_P3218772	5.692346	6.0185823	NM_080599	chr10:11973778-11973719	UPF2
A_33_P3577142	4.9949713	4.601367	AK098143	chr16:89281757-89281698	
A_24_P7750	2.4775794	2.3900566		chr5:146086546-146086485	
A_23_P308483	4.6055937	4.772234	NM_001001671	chrX:19379515-19378951	MAP3K15
A_33_P3352467	3.7737823	3.186857	NM_001050	chr17:71167722-71167781	SSTR2
A_33_P3369667	5.6996655	5.65913	XM_005275760	chr15:84951509-84951450	LOC101930647
A_33_P3237634	7.9027658	8.193811	NM_004089	chrX:106956545-106956486	TSC22D3
A_33_P3263417	5.6822624	5.3689566	NR_015431	chr7:130628979-130628920	LINC-PINT
A_33_P3234814	3.6551075	3.3522503			
A_32_P205637	7.6803985	7.9656906	NM_032521	chr20:49369777-49369836	PARD6B
A_33_P3228959	9.259901	9.332787	NM_016050	chr11:66204673-66204614	MRPL11
A_23_P3502	7.817448	7.3885417	NM_144604	chr16:88694508-88695205	ZC3H18
A_33_P3367396	9.3639555	9.224274	NM_207468	chr1:222923479-222923538	FAM177B
A_33_P3389153	6.852153	6.4598207	NM_005990	chr5:171469310-171469251	STK10
A_33_P3306192	5.874843	6.3434033	NM_001101362	chr15:65370469-65370528	KBTBD13
A_23_P149613	4.848303	5.103488	NM_002021	chr1:171254601-171254660	FMO1
A_23_P104098	5.482492	5.0961995	NM_177402	chr1:202565898-202565839	SYT2
A_23_P23206	8.552848	8.597627	NM_006341	chr1:11735222-11735163	MAD2L2
A_33_P3298612	4.6165667	4.575678		chr3:036342907-036342966	
A_23_P111037	10.915826	11.099552	NM_003529	chr6:26020881-26020940	HIST1H3A
A_33_P3373850	6.251172	6.211047	NM_032259	chr16:734761-734702	WDR24
A_33_P3245575	5.5444603	5.4107275	NM_024656	chr19:17690322-17690381	COLGALT1
A_33_P3287646	13.521543	13.600891	NM_001540	chr7:75933550-75933609	HSPB1
A_32_P50431	5.4922705	5.5020194	NM_003932	chr22:41221617-41221558	ST13
A_23_P93750	11.071348	10.956941	NM_012322	chr7:32526885-32526826	LSM5
A_33_P3392250	6.406855	6.152378	NM_001243770	chr10:102673585-102673643	FAM178A
A_24_P655849	6.4364495	6.497038	NM_001127217	chr13:37419547-37419488	SMAD9
A_33_P3344599	3.3771043	4.475943		chr1:10132588-10132647	UBE4B
A_33_P3309665	4.084678	3.498028	NM_153018	chr17:4999574-4999633	ZFP3
A_23_P145330	8.041555	7.9882298	NM_019052	chr6:31110908-31110849	CCHCR1
A_33_P3245066	7.257102	7.550768	NM_001110781	chr1:1595787-1595728	SLC35E2B
A_23_P33216	11.292916	11.689829	NM_001686	chr12:57036312-57036253	ATP5B
A_33_P3329444	6.1002436	6.408484	NM_182574	chr19:49216315-49216256	MAMSTR
A_23_P166775	3.665751	3.821354	NM_153461	chr3:9974732-9974791	IL17RC
A_33_P3210343	9.421128	9.529115	NM_001987	chr12:12048245-12048304	ETV6
A_23_P99540	11.801372	11.704896	NM_004926	chr14:69254535-69254476	ZFP36L1
A_33_P3221341	4.471589	4.464582	NM_173497	chr10:93221877-93221936	HECTD2
A_23_P201979	7.507805	7.8267827	NM_183013	chr10:35501242-35501301	CREM
A_33_P3394517	5.13411	5.2163076	NM_015092	chr16:18907434-18907375	SMG1
A_33_P3396972	5.7515745	6.023318	AK094477	chr22:18443810-18443751	
A_23_P200143	4.8284826	5.4047327	NM_015726	chr1:160186380-160186321	DCAF8
A_23_P434430	2.519619	2.3900566	NM_152262	chr19:11979911-11979970	ZNF439
A_23_P69431	13.723041	13.86687	NM_000968	chr15:66792637-66792462	RPL4
A_33_P3347932	11.788803	11.7690935	NM_005526	chr8:145538270-145538329	HSF1
A_23_P155288	9.857632	9.752775	NM_024996	chr3:158409282-158409341	GFM1
A_33_P3258056	4.491212	5.3664603	NM_001145028	chr19:14165422-14165363	PALM3
A_33_P3319760	9.253231	9.116058	NM_001277224	chr1:159888703-159888644	TAGLN2

A_23_P7655	7.007165	6.739789	NM_013978	chr5:172590881-172590940	BNIP1
A_23_P97328	9.599248	9.614126	NM_024319	chr1:228288610-228288551	C1orf35
A_23_P29318	10.356567	10.254912	NM_015380	chr22:44385073-44385132	SAMM50
A_33_P3343981	8.003777	8.273283	XM_005257849	chr17:79139542-79139483	AATK
A_23_P71513	9.793108	10.096541	NM_015137	chr8:133025157-133025216	EFR3A
A_33_P3287119	7.2677107	7.482976	NM_001122646	chr2:97541754-97541695	FAM178B
A_33_P3227731	9.408926	9.787374		chr9:018718139-018718080	
A_33_P3243997	7.4577327	6.913238	NM_014614	chr2:54091284-54091225	PSME4
A_32_P407245	5.712641	5.444701	NM_024902	chr12:49743280-49743339	DNAJC22
A_33_P3278455	4.9817295	4.7128105	NM_001242811	chr6:90343223-90343282	ANKRD6
A_23_P315122	7.496139	7.780258	NM_004097	chr2:73161417-73161476	EMX1
A_33_P3292525	8.697972	8.319636	NM_001010866	chr1:9664928-9664987	TMEM201
A_23_P302654	6.412029	6.8664107	NM_018140	chr5:653323-653382	CEP72
A_33_P3234984	4.8271384	5.204869	NM_031473	chr12:110606171-110606230	IFT81
A_32_P192376	4.3634973	3.2050266	NM_006208	chr6:132216052-132216111	ENPP1
A_33_P3236813	3.576063	3.6254165	NM_006143	chr12:12814162-12814103	GPR19
A_33_P3363188	4.237023	3.060061		chrUn_gl000211:91828-91887	FLJ43315
A_33_P3333030	4.8348923	4.4069877	NM_001178133	chr10:104269001-104269060	SUFU
A_23_P23815	8.453723	8.867746	NM_021194	chr1:211748901-211748842	SLC30A1
A_32_P163089	12.679198	12.842008	NM_001145199	chr12:105760440-105761274	C12orf75
A_23_P16915	7.8145466	7.518828	NM_012413	chr2:37599594-37599862	QPCT
A_33_P3405399	4.102263	4.6817727	NM_001184765	chr1:86824530-86824471	ODF2L
A_33_P3337771	10.628776	10.622841	NM_024831	chr8:56737858-56737917	TGS1
A_33_P3382959	7.0976553	6.9915314	NM_012115	chr6:90581049-90581108	CASP8AP2
A_33_P3327539	5.4583526	5.568107	NM_015130	chr4:141543418-141543359	TBC1D9
A_33_P3418576	3.6223674	3.4660888	NR_073096	chr6:158535843-158535784	SERAC1
A_33_P3415491	6.7545004	6.993178	NM_145294	chr16:705829-705888	WDR90
A_23_P211244	7.3278074	7.2371225	NM_206962	chr21:48080822-48081718	PRMT2
A_23_P259141	3.2375503	2.9336782	NM_030776	chr20:56179269-56179210	ZBP1
A_23_P128246	4.531509	4.3554363	NM_007076	chr12:108913068-108913127	FICD
A_32_P52911	9.399486	9.520123	NM_018269	chr2:3502016-3501957	ADI1
A_33_P3315425	7.154971	6.6064177	NM_022840	chr18:2537642-2537583	METTL4
A_23_P89056	8.223541	7.9597774	NM_014669	chr16:56868309-56868659	NUP93
A_32_P226205	6.995493	7.1654177	NM_033400	chr14:23990167-23990108	ZFHX2
A_23_P204277	10.655569	10.898224	NM_177925	chr12:14927815-14927874	H2AFJ
A_33_P3287883	7.086613	6.741004	NR_028327	chr1:663342-663284	LOC100133331
A_24_P324563	8.529832	8.384204	NM_031213	chr19:1877547-1877406	ABHD17A
A_33_P3270776	3.6146867	2.3900566	NM_053044	chr4:8288450-8288509	HTRA3
A_23_P62907	9.160404	9.344291	NM_007348	chr1:161928720-161928779	ATF6
A_23_P131299	5.583713	5.364708	NM_138802	chr2:220072672-220072731	ZFAND2B
A_23_P252825	8.888902	8.818959	NM_012235	chr3:47455533-47455474	SCAP
A_33_P3273399	6.460874	6.3410287	BU739610		
A_33_P3220152	8.076642	7.1754704	NM_014963	chr19:1108327-1108268	SBNO2
A_33_P3376954	5.0344224	4.5912094		chr10:81588027-81588086	XLOC_014512
A_33_P3389261	5.633367	5.905467	NM_001142645	chr2:191371678-191371619	TMEM194B
A_33_P3383656	7.6200957	7.437353	NM_001033575	chr2:241503363-241503422	DUSP28
A_33_P3605969	8.141028	8.5389595	BC071847	chr1:41848710-41848651	
A_33_P3342096	7.587314	7.460921	NM_015035	chr20:39832111-39832052	ZHX3
A_23_P386320	7.529522	7.5402184	NM_033316	chr3:196745930-196745871	MFI2
A_23_P60225	8.056334	8.130052	NM_012203	chr9:37429806-37430539	GRHPR
A_32_P161762	10.446409	10.47119	NM_001024630	chr6:45518216-45518275	RUNX2
A_23_P57941	7.551688	7.5726457	NM_005777	chr3:50106147-50107896	RBM6
A_33_P3219601	6.7148824	6.1482472	NM_007314	chr1:179076912-179076853	ABL2
A_23_P133332	8.946409	8.909851	NM_015084	chr5:71515856-71515797	MRPS27
A_24_P32215	5.935665	6.1551075		chr10:062444634-062444693	
A_33_P3369520	6.5515575	6.3641376	NM_152900	chr1:114228405-114228464	MAGI3
A_23_P253932	9.6318	9.270785	NM_001033030	chr3:138351814-138351873	FAIM
A_33_P3235262	5.489819	5.471758	NM_001135219	chr9:130683885-130683826	PIP5KL1
A_32_P100641	3.806421	3.8204112	XR_111841	chr19:22716218-22716277	LOC100128139

A_33_P3269203	13.234316	13.395286	NM_001207014	chr11:75283743-75283802	SERPINH1
A_23_P111311	4.80793	5.330603	NM_144497	chr6:151674814-151674873	AKAP12
A_33_P3280405	7.3450813	7.2527976	NR_002163	chr13:42017239-42017298	OR7E37P
A_23_P78782	6.9108896	6.6749253	NM_001217	chr19:49142844-49142696	CA11
A_33_P3220347	4.536459	4.47001	NM_001103176	chr7:155294043-155293984	CNPY1
A_33_P3255647	7.812969	7.89793	NM_001199973	chrX:100667226-100667285	RPL36A-HNRNPH2
A_23_P34946	6.514367	6.2473807	NM_014388	chr1:210030587-210030646	DIEXF
A_33_P3412035	5.7905636	5.7516837	NM_018227	chr4:68481613-68481554	UBA6
A_23_P8185	11.977153	12.171502	NM_006519	chr6:159057743-159057684	DYNLT1
A_33_P3367606	3.8700366	3.6483347	NR_026806	chr12:31478431-31478490	FLJ13224
A_24_P182620	5.8338084	5.489242	NM_001408	chr1:109817992-109818051	CELSR2
A_23_P428184	9.487854	9.456871	NM_021065	chr6:26199142-26199083	HIST1H2AD
A_33_P3292851	5.952853	5.650811	XM_005255542	chr16:1371388-1371447	UBE2I
A_23_P128663	10.806917	10.403795	NM_014363	chr13:23903098-23903039	SACS
A_33_P3345414	4.947069	5.162953		chrX:71494164-71494105	RPS4X
A_33_P3368238	3.7909317	3.2197435		chr8:72443413-72443354	
A_33_P3353027	2.3221061	2.3900566	XR_171041	chr7:29188214-29188273	CHN2
A_23_P119617	7.182988	7.1235614	NM_172231	chr19:19388687-19388628	SUGP1
A_33_P3391656	6.8347106	6.5735207	NM_033550	chr20:45313382-45313323	TP53RK
A_23_P127140	7.9300733	7.7740927	NM_014904	chr10:119765427-119765368	RAB11FIP2
A_24_P48057	5.7968717	5.6809883	NM_005853	chr16:54967726-54967785	IRX5
A_23_P211522	7.681879	7.3708105	NM_145738	chr22:39770517-39772074	SYNGR1
A_33_P3217347	7.415511	7.084196	NM_145119	chrX:68380793-68380734	PJA1
A_23_P162228	14.875682	14.652729	NM_001113203	chr12:57106686-57106627	NACA
A_33_P3327822	5.6044765	6.0418897	NM_007341	chr21:40887038-40887097	SH3BGR
A_24_P37519	6.6891823	6.731683	NM_020347	chr3:45866096-45866037	LZTFL1
A_23_P44867	4.075006	3.9470692	NM_016519	chr4:71472470-71472529	AMBN
A_24_P570049	6.9133935	6.6633406	NM_005036	chr22:46639178-46639237	PPARA
A_24_P215765	2.79591	3.0167186	NM_024490	chr15:25923934-25923875	ATP10A
A_33_P3376090	5.920101	6.0701985	NM_001004452	chr9:125282181-125282240	OR1J4
A_23_P41976	8.954823	9.233659	NM_145265	chr5:205179-205120	CCDC127
A_33_P3213557	6.5581617	6.615014	NM_015622	chr7:5940501-5940562	CCZ1
A_24_P135276	9.419148	9.584475	NM_032172	chr7:6194653-6194712	USP42
A_24_P940310	4.8387847	4.590727	NM_014825	chr21:33683708-33683649	URB1
A_23_P333705	6.089078	6.2291303	NM_002498	chr13:52707207-52707148	NEK3
A_24_P215653	9.218625	9.267219	NM_175060	chr14:38724198-38724139	CLEC14A
A_23_P432598	6.9644737	6.3608336	NM_152284	chr8:82670898-82670957	CHMP4C
A_24_P255473	11.15636	11.326168		chr21:037667651-037667591	
A_24_P227927	4.4266434	4.2236996	NM_181078	chr16:27461324-27461383	IL21R
A_24_P941625	4.99617	4.3594093	NM_021916	chr22:24083874-24083815	ZNF70
A_23_P7325	2.3221061	2.3900566	NM_004334	chr4:15720549-15720608	BST1
A_33_P3409477	8.397176	7.984046	NM_032873	chr11:122685098-122685157	UBASH3B
A_23_P52914	4.970324	5.0120397	NM_001001920	chr11:55322421-55322480	OR4C15
A_24_P102981	8.8355465	8.613574	NM_006736	chr2:220151526-220151585	DNAJB2
A_24_P14731	5.2380686	5.264602	NM_013271	chrX:48690521-48690462	PCSK1N
A_32_P189204	10.175518	10.321525	NM_174942	chr12:101018594-101018653	GAS2L3
A_33_P3336387	5.5950646	5.4943213	NM_207128	chr10:135202510-135202569	PAOX
A_32_P103695	10.070845	9.801705	NM_145269	chr8:94738635-94738694	FAM92A1
A_33_P3278159	9.1854515	9.286153		chr6:004024918-004024977	
A_23_P20122	5.723931	5.6935997	NM_024625	chr7:138745502-138745443	ZC3HAV1
A_23_P32414	8.647604	8.136764	NM_016542	chrX:131209867-131209926	MST4
A_32_P44316	11.4669075	11.979293	NM_001402	chr6:74229189-74229130	EEF1A1
A_33_P3301469	8.068656	8.121542	NR_027019	chr2:132905224-132905165	ANKRD30BL
A_23_P215787	6.780546	7.9266677	NM_012257	chr7:106842145-106842204	HBP1
A_33_P3280400	4.9666862	4.9889913	NR_002822	chr7:97596037-97595978	MGC72080
A_23_P120103	3.834327	3.6279416	NM_002252	chr2:18114112-18114171	KCNS3
A_23_P132874	11.136042	10.726288	NM_032359	chr3:99897343-99897402	CMSS1
A_23_P154801	5.8308153	5.6346245	NM_015638	chr20:33591079-33591020	TRPC4AP

A_24_P201064	7.368447	7.502629	NM_001201329	chr8:8993872-8993813	PPP1R3B
A_23_P326142	6.993178	6.924873	NR_027330	chr7:127639207-127639266	SND1-IT1
A_33_P3357935	6.417445	6.66422	NM_153700	chr15:43906145-43906086	STRC
A_23_P145904	12.624529	12.872877	NM_012412	chr7:44873931-44873872	H2AFV
A_33_P3254751	4.84366	4.8303914	AK124217	chr9:130665665-130665606	ST6GALNAC6
A_24_P225961	6.660144	6.463276	NM_004393	chr3:49572194-49572253	DAG1
A_24_P380022	7.4991736	6.1647863	NM_020390	chr3:170606975-170606916	EIF5A2
A_33_P3242075	5.3444	5.42926	XM_001719300	chr16:54971757-54971698	
A_23_P132936	10.055784	9.568821	NM_021928	chr4:177249358-177249417	SPCS3
A_23_P331748	2.511381	2.3900566	NM_001772	chr19:51742998-51743057	CD33
A_33_P3355937	5.384802	5.392952		chr18:74270471-74270530	LINC00908
A_24_P165259	12.100834	11.761814	NM_013328	chr1:226107730-226107671	PYCR2
A_23_P16683	9.270785	8.9197855	NM_017722	chr19:13216113-13215869	TRMT1
A_32_P59302	7.995375	7.7489634	NM_024503	chr1:41972662-41972603	HIVEP3
A_23_P162378	8.398947	8.19973	NM_016122	chr12:94702242-94702183	CCDC41
A_33_P3271387	6.517278	6.276672	NM_138350	chr1:6695557-6695616	THAP3
A_23_P20615	11.04696	11.578044	NM_006401	chr9:100760959-100767303	ANP32B
A_23_P39656	6.1350837	5.9690065	NM_022453	chr2:219529103-219529044	RNF25
A_33_P3411628	10.461803	10.416797	NM_000077	chr9:21970960-21970901	CDKN2A
A_32_P151544	12.5987625	12.163997	NM_000224	chr12:53346116-53346554	KRT18
A_24_P321752	5.780537	5.8098993	NM_015072	chr14:76330039-76330098	TTLL5
A_33_P3388909	4.1972437	4.240522	XR_110101	chr9:140786612-140786553	LOC100133077
A_33_P3325808	8.670724	8.807152		chr7:134419765-134419706	
A_33_P3372466	8.024143	8.144496	NM_138501	chr19:14675604-14675663	TECR
A_23_P132263	8.933505	8.995356	NM_019843	chr22:31835478-31835419	EIF4ENIF1
A_33_P3332690	15.07173	15.130064	BC047718	chr17:29085298-29085357	SUZ12P1
A_24_P74064	7.59341	7.5490217	NM_003409	chr18:5289958-5289899	ZBTB14
A_33_P3287388	11.231499	11.090216		chr7:000304374-000304433	
A_33_P3297217	5.817236	5.0541315	NM_001042402	chr4:76857322-76857263	NAAA
A_33_P3275998	6.607744	6.510577	NM_004703	chr17:5286459-5286518	RABEP1
A_33_P3327697	9.728859	9.540901	NR_027046	chr14:71956365-71956424	LOC145474
A_23_P93823	9.234647	9.3096695	NM_181471	chr7:73649925-73649866	RFC2
A_24_P36847	8.341457	8.69232	NM_001357	chr1:182856256-182856315	DHX9
A_23_P406341	10.081831	9.992921	NM_001001936	chr10:116055189-116055130	AFAP1L2
A_33_P3256560	8.808231	8.783614	NM_006336	chr9:131492128-131492069	ZER1
A_23_P357794	8.9612	8.538822	NM_022045	chr8:121534934-121535571	MTBP
A_24_P288424	9.069089	8.751556	NM_016045	chr20:57608816-57608757	SLMO2
A_23_P35456	8.612374	8.041555	NM_014631	chr10:105353953-105353894	SH3PXD2A
A_32_P101031	5.1054034	3.554862	NM_144586	chr2:133402490-133402431	LYPD1
A_33_P3390342	5.256454	5.493146	NR_034022	chr7:7117985-7117926	LOC100131257
A_24_P166661	7.367898	6.8825684	NM_032936	chr7:77423590-77423531	TMEM60
A_24_P132276	5.9700336	5.677516	NM_018292	chr6:107114919-107114978	QRSL1
A_23_P16538	5.975348	6.0582366	NM_006532	chr19:18554077-18554018	ELL
A_33_P3382157	11.183286	10.947164	NM_007166	chr11:85668853-85668794	PICALM
A_23_P145074	10.396851	11.084706	NM_006813	chr6:89794426-89794485	PNRC1
A_33_P3297302	3.835125	4.103352	NM_001135021	chr2:85584222-85584281	ELMOD3
A_23_P63281	4.136053	4.231446	NM_001029885	chr1:1263506-1263565	GLTPD1
A_33_P3217020	5.371482	5.4549413	NM_203365	chr2:204354754-204354695	RAPH1
A_33_P3764663	4.328448	4.057884	BC067894	chr12:88176017-88175958	MKRN9P
A_33_P3409302	5.60033	5.558278	NM_078470	chr10:101473235-101473176	COX15
A_24_P153853	6.3319597	6.379889	NM_001005207	chr17:57060141-57060082	TRIM37
A_23_P258108	13.887369	13.826841		chr4:169677341-169677400	
A_24_P538403	9.355723	9.038101	NM_005406	chr18:18530291-18530232	ROCK1
A_23_P380379	5.231192	5.0224442	NM_001286548	chr12:75671463-75671404	CAPS2
A_23_P55990	5.4397163	5.2874045	NM_003827	chr19:47996279-47996220	NAPA
A_24_P911607	3.7513537	3.1306412	NM_058238	chr22:46372630-46372571	WNT7B
A_23_P13929	6.4384136	6.4479	NM_031474	chr12:2934527-2934504	NRIP2
A_23_P334664	4.534923	4.4830093	NM_033247	chr15:74315721-74317228	PML
A_33_P3351529	15.059151	15.087955		chr7:152299325-152299266	

A_23_P203773	4.59306	3.6493278	NM_080654	chr11:27371039-27370980	CCDC34
A_23_P310483	8.222618	8.84101	NM_001013842	chr8:22461401-22461460	C8orf58
A_33_P3254946	9.394786	9.478785	NM_024927	chr17:40820030-40819971	PLEKHH3
A_24_P327815	5.5550666	5.0829086	NM_006819	chr11:63967732-63970373	STIP1
A_33_P3383696	7.3047557	7.049968	NM_005876	chr2:220358295-220358354	SPEG
A_24_P271049	7.7050595	7.975696	NM_001035005	chr18:47008752-47008693	C18orf32
A_33_P3278435	9.621325	9.514803	NM_016565	chr11:73583781-73583722	COA4
A_24_P74487	7.7141495	7.453189	NM_058182	chr21:35757828-35757887	SMIM11
A_23_P63655	9.520123	9.475307	NM_005174	chr10:7839099-7840969	ATP5C1
A_23_P336796	7.0209665	6.8665857	NM_173601	chr12:42476664-42476605	GXYLT1
A_33_P3382177	12.615065	12.756387	NM_003255	chr17:76849122-76849063	TIMP2
A_23_P130089	8.361214	8.567085	NM_174887	chr17:26657550-26657491	IFT20
A_23_P259413	10.102343	10.267456	NM_017548	chr3:133305460-133305519	CDV3
A_23_P46315	2.3296914	3.4786782	AK124287	chr1:115125613-115125554	DENND2C
A_33_P3310293	6.3903065	6.1984425	NM_181805	chr20:43218442-43218501	PKIG
A_24_P15621	4.167478	4.8885717	NR_003083	chr16:32890762-32890621	SLC6A10P
A_33_P3575854	8.210513	7.8799024	NR_029407	chr2:128644687-128644745	LOC642361
A_23_P168490	5.361347	5.820335	NM_022373	chr7:35673414-35673355	HERPUD2
A_23_P85598	6.320317	6.390808	NM_020247	chr1:227174695-227174754	ADCK3
A_23_P18447	2.3221061	2.3900566	NM_013261	chr4:23793996-23793937	PPARGC1A
A_33_P3261828	4.3215594	4.6614356		chr15:82623784-82623843	ADAMTS7P1
A_23_P37718	4.198912	3.9848897	NM_001297	chr16:57921905-57921846	CNGB1
A_33_P3313652	5.4352555	5.350876		chr1:228252147-228252088	
A_33_P3373985	6.7878575	6.273546		chr2:074645359-074645300	
A_23_P122545	10.88373	10.977464	NM_002904	chr6:31921587-31921528	NELFE
A_24_P242036	6.3142815	6.7036715	NR_002184	chr22:42970323-42970264	RRP7B
A_33_P3356361	3.7940125	4.112061		chr15:40359360-40359419	SRP14-AS1
A_33_P3284290	5.8431168	6.200718	NM_001201407	chr16:89294911-89294970	ZNF778
A_23_P202408	6.4803514	6.821924	NM_001278185	chr10:69714416-69714357	HERC4
A_33_P3579984	5.665615	5.2400618	AK094521	chr2:8861406-8861347	
A_23_P415015	5.4078474	5.608911	NM_022374	chr2:38523618-38523559	ATL2
A_33_P3380161	9.6535	9.240551	NR_026778	chr1:245004000-245003941	HNRNPU-AS1
A_23_P428992	8.971268	8.805803		chr4:6677408-6677467	LOC93622
A_23_P12572	9.775006	9.643215	NM_033338	chr10:115490494-115490553	CASP7
A_33_P3791123	11.767601	11.819845	NM_001165877	chr22:43035995-43035936	ATP5L2
A_23_P48807	7.759228	7.862738	NM_001001556	chr15:49620301-49620360	GALK2
A_33_P3411097	6.1835327	6.3030963	NM_001145118	chr7:6541472-6541413	GRID2IP
A_23_P170337	7.266201	7.4282966	NM_003748	chr1:19198108-19198049	ALDH4A1
A_33_P3303385	12.330388	12.249262	NM_014865	chr12:6641042-6641101	NCAPD2
A_23_P90980	3.6517437	2.9482398	NM_005383	chr2:233899680-233899739	NEU2
A_33_P3559138	14.257444	14.213579		chr21:25862261-25862320	
A_23_P50697	2.3221061	2.3900566	NM_006905	chr19:43372365-43372306	PSG1
A_33_P3318424	4.2054768	4.306655		chrX:102001038-102000979	
A_23_P126888	6.982904	7.240306	NM_017596	chr1:200939143-200939084	KIF21B
A_23_P154086	9.498183	9.034876	NM_004328	chr2:219528036-219528095	BCS1L
A_23_P217507	7.0953226	6.827089	NM_004729	chrX:2405173-2405114	ZBED1
A_33_P3226377	6.0861936	6.1104774	NM_005042	chr12:11083624-11083683	PRH2
A_33_P3548768	4.5582066	4.62694	CU449054	chr1:152629017-152628958	LINC00302
A_24_P366749	4.386292	4.3019686	NM_001005855	chr1:154305154-154306622	ATP8B2
A_24_P879740	7.0888357	7.090687	NM_005909	chr5:71504756-71504815	MAP1B
A_33_P3235432	5.050267	5.25791	XM_005263489	chr3:138200228-138200287	ESYT3
A_23_P37424	8.321959	8.147621	NM_024063	chr15:45713450-45713509	SPATA5L1
A_23_P41159	5.8400164	6.3336444	NM_174907	chr3:73114662-73114721	PPP4R2
A_33_P3272483	10.23921	10.1218195	NM_032389	chr11:47185932-47185873	ARFGAP2
A_33_P3345294	5.729842	5.8738112		chr6:109636562-109636621	
A_33_P3346663	14.185693	14.233127	NM_004069	chr19:47341486-47341427	AP2S1
A_23_P82929	5.142419	4.3055596	NM_002514	chr8:120436435-120436494	NOV
A_23_P34402	10.848771	11.093014	NM_015331	chr1:160328609-160328668	NCSTN
A_33_P3231367	8.177618	8.023443	NM_013236	chr22:46240711-46240770	ATXN10

A_23_P333063	8.056068	8.35522	NM_003079	chr17:38787921-38787862	SMARCE1
A_32_P180971	8.011172	7.920942	NR_024437	chr2:243037063-243037122	LOC728323
A_23_P35912	10.175759	10.236645	NM_033306	chr11:104815551-104815492	CASP4
A_23_P166196	8.913569	8.956975	NM_015511	chr20:34844644-34844705	AAR2
A_23_P119214	8.09865	7.5169077	NM_006351	chr19:7997542-7996045	TIMM44
A_24_P266728	10.837336	10.682583	NM_004630	chr11:64532338-64532279	SF1
A_33_P3244991	4.205696	4.22773	AK091251	chr15:40077686-40077745	
A_33_P3339436	4.56319	4.4717436		chr15:37156836-37156777	LOC145845
A_24_P791669	3.7570822	4.1980734	NM_001136116	chr5:178461245-178461304	ZNF879
A_23_P212397	8.939609	8.91461	NM_015340	chr3:45590070-45590129	LARS2
A_23_P79816	6.677294	6.9590096	NM_052846	chr20:39989112-39989053	EMILIN3
A_33_P3334292	10.615054	10.676505	AK123006	chr1:143764573-143764514	PPIAL4G
A_33_P3422712	9.04498	9.100168		chrX:137793760-137793819	
A_33_P3218089	4.8908396	5.165998	NM_001185149	chr4:184243080-184243021	CLDN24
A_24_P275873	10.672243	10.776985	NM_005993	chr17:80900511-80900570	TBCD
A_23_P143535	6.091945	5.9804378	NM_033661	chr21:44270319-44270260	WDR4
A_33_P3334102	7.079731	6.742379	NM_174919	chr17:43506778-43506719	ARHGAP27
A_23_P82748	12.662024	12.666818	NM_020189	chr8:110355682-110355741	ENY2
A_33_P3806721	7.4557514	7.5597386	NM_001145348	chr16:27231932-27231991	KDM8
A_23_P48550	9.623254	9.501095	NM_015005	chr14:105362926-105362985	CEP170B
A_33_P3218138	11.667804	11.599654	NM_000937	chr17:7417838-7417897	POLR2A
A_33_P3416682	3.6977086	4.0030646	NM_001242815	chr2:42165651-42165592	C2orf91
A_33_P3211384	4.1390085	4.266387	NM_001145305	chr19:18377090-18377031	KIAA1683
A_24_P246091	3.785995	4.5039487		chr10:91451582-91451523	FLJ37201
A_33_P3375809	5.8990884	5.2862353	NR_033998	chr1:95428751-95428810	LOC729970
A_33_P3424803	13.403207	13.69577	M26429	chr6:031321703-031321644	HLA-C
A_33_P3718152	3.6314244	3.6835792	AK055876	chr14:73946592-73946651	NUMB
A_23_P52147	11.556739	11.390165	NM_001079515	chr1:235612144-235612203	TBCE
A_23_P161218	2.3221061	2.3900566	NM_014391	chr10:92672621-92672562	ANKRD1
A_33_P3559102	3.7591276	3.3110263	BQ213652		LOC440864
A_24_P332651	3.7896152	3.247301	XM_001727011	chr2:11491799-11491858	LOC650157
A_24_P346604	5.0002646	5.751359	NM_001001481	chr8:74737407-74722831	UBE2W
A_33_P3241393	4.8928804	3.9626632	NM_133478	chr2:74443455-74443396	SLC4A5
A_33_P3402091	7.225475	7.2508926	NM_006343	chr2:112786885-112786944	MERTK
A_33_P3369371	2.3221061	2.3900566	NM_002084	chr5:150407032-150407091	GPX3
A_33_P3232393	5.3595815	5.6320124	XR_244448	chr22:47512183-47512124	LOC100128818
A_24_P230282	5.442795	5.581539	NM_016378	chrX:8138605-8138354	VCX2
A_33_P3209279	5.0864778	4.9352293	NM_015278	chr6:148795298-148795357	SASH1
A_23_P24960	7.471079	7.2700787	NM_024678	chr11:78154745-78152143	NARS2
A_24_P43391	9.096307	9.209545	NM_018475	chr4:56284050-56284109	TMEM165
A_33_P3223648	5.6610374	5.5632925	NR_037879	chr2:37430462-37430521	CEBPZ-AS1
A_23_P205789	8.107697	8.235331	NM_002041	chr15:50578281-50578222	GABPB1
A_23_P78571	5.8521075	5.8519506	NM_144613	chr19:55862167-55862108	COX6B2
A_33_P3334436	4.926345	4.961263		chr22:030442750-030442691	
A_33_P3705907	8.1207695	7.944208	NM_058190	chr21:46380023-46380082	FAM207A
A_23_P415827	6.294894	5.88803	NM_015146	chr5:132092310-132092251	SEPT8
A_33_P3257523	4.9360337	4.8617477	NM_001257273	chr6:56966835-56966894	ZNF451
A_24_P551842	12.708443	12.988044	HV444967	chrM:000015588-000015646	CYTB
A_23_P43071	9.80573	9.616726	NM_015942	chr8:97258135-97256297	MTERFD1
A_33_P3252236	6.5732	6.5392265	NR_047510	chr21:30244575-30244516	N6AMT1
A_24_P45005	8.984638	8.913569	NM_024663	chr20:57290406-57290465	NPEPL1
A_32_P22622	4.77787	4.7674174	NM_003703	chr4:2949326-2949267	NOP14
A_23_P487	11.216723	11.020468	NM_012474	chr1:165877122-165877181	UCK2
A_33_P3290909	11.972696	11.845602	NM_001281463	chrX:53426606-53426547	SMC1A
A_33_P3320127	10.403795	10.478286	NM_005370	chr19:16244352-16244411	RAB8A
A_23_P212050	3.9191804	4.0490775	NM_000055	chr3:165490878-165490819	BCHE
A_23_P252145	7.3698006	7.365104	NM_020156	chr7:7283565-7283624	C1GALT1
A_23_P25224	10.45255	10.695754	NM_003651	chr12:10853889-10852231	YBX3
A_33_P3249773	8.689439	8.348151	NM_001146171	chr1:212989740-212989799	TATDN3

A_33_P3285251	4.83928	5.0500007	AK310550	chrX:48929820-48929761	
A_33_P3231613	2.3660016	2.3900566	NM_001129828	chrX:151927955-151928014	CSAG3
A_23_P167789	7.8574986	8.11927	NM_016605	chr5:137684998-137685057	FAM53C
A_24_P942030	5.7784004	5.9248247	NM_003762	chr1:171670045-171669986	VAMP4
A_23_P405216	6.1617103	5.892935	NM_001145083	chr3:40529739-40529798	ZNF619
A_23_P205007	8.765957	8.575386	NM_002271	chr13:98676214-98676273	IPO5
A_33_P3380098	4.216275	4.1265516	NM_001243756	chr12:120651705-120651646	PXN
A_33_P3379091	8.605446	8.683968	NM_145731	chr22:39774334-39774393	SYNGR1
A_24_P850428	12.465715	12.528184	BC070327	chr4:144346386-144346445	
A_23_P307563	8.209644	8.072372	NM_001024660	chr3:124439420-124439479	KALRN
A_32_P19752	6.6974545	6.496127	NM_144664	chr11:95502378-95502319	FAM76B
A_23_P330262	5.53497	5.588645	NM_012398	chr19:3630729-3630670	PIP5K1C
A_33_P3402414	8.712591	9.057875	NM_001788	chr7:35944081-35944140	SEPT7
A_23_P65481	7.176972	7.3601737	NM_007110	chr14:20836584-20836525	TEP1
A_33_P3423220	5.262557	5.3543553	NM_001195528	chr11:74953022-74953081	TPBGL
A_33_P3293180	4.609005	4.9360337	AK093443	chr9:43029058-43028999	FAM95B1
A_33_P3226237	7.0526867	6.5875645	AK125205	chr2:204193090-204193031	
A_32_P102062	4.219327	5.088623	NM_001455	chr6:108985954-108986013	FOXO3
A_23_P426140	6.802417	7.1355968	NM_024646	chr1:53292218-53292277	ZYG11B
A_33_P3420655	8.412128	8.215183	NM_014663	chr1:44171129-44171188	KDM4A
A_23_P82979	3.9064698	4.0495405	NM_006059	chr9:133967284-133967341	LAMC3
A_24_P14367	8.873712	8.600434	NM_002819	chr19:811523-811582	PTBP1
A_24_P636332	9.682794	9.249599	NM_198489	chr11:118886295-118886354	CCDC84
A_32_P129669	6.8242016	6.794613		chrX:106848038-106848097	FRMPD3
A_32_P192545	9.609713	9.688896	NM_001006938	chrX:101395635-101395576	TCEAL6
A_23_P360240	4.9498186	4.997044	NM_138768	chr11:69064410-69064469	MYEOV
A_24_P369656	3.7361557	3.4590755	NM_052978	chr14:51463203-51463144	TRIM9
A_33_P3219121	5.526066	5.6671724	AK131379	chr3:124207525-124207584	KALRN
A_23_P133902	5.849459	5.7933474	NM_014068	chr6:31107731-31107790	PSORS1C1
A_23_P24192	7.09581	6.6242967	NM_015179	chr10:99125880-99123617	RRP12
A_33_P3422258	11.128361	10.915041	NM_001099670	chr8:86129724-86129665	C8orf59
A_33_P3376341	6.2707896	6.391018	NM_001024678	chr8:145747832-145747773	LRRC24
A_23_P68529	9.586984	9.433028	NM_033453	chr20:3204248-3204307	ITPA
A_33_P3210965	8.949158	9.254446	NM_001082538	chr12:111086854-111086913	TCTN1
A_33_P3366301	4.7162085	4.48184	NR_026732	chr14:29247707-29247766	C14orf23
A_23_P407074	7.631954	7.790366	NM_001005360	chr19:10942036-10942095	DNM2
A_23_P215449	9.367606	9.230713	NM_032408	chr7:72855115-72855056	BAZ1B
A_23_P58002	6.166003	6.198016	NM_022171	chr3:49453536-49453595	TCTA
A_33_P3232527	6.969487	6.435804	NM_025191	chr1:184662243-184662184	EDEM3
A_33_P3325395	4.870519	5.435527	AK027069	chr17:75500216-75500275	
A_23_P144531	15.346972	15.403006	AK026323	chr4:113336615-113336674	ALPK1
A_23_P216396	8.980516	8.084062	NM_014285	chr9:133580140-133580199	EXOSC2
A_33_P3589217	9.59147	9.691311	NM_001636	chrX:1508451-1508392	SLC25A6
A_23_P36322	7.3234396	6.9618726	NM_001567	chr11:71949487-71949546	INPPL1
A_23_P11390	6.0646095	6.264395	NM_004679	chrY:16168718-16168777	VCY
A_23_P138480	4.3015985	3.864126	NM_024886	chr10:104210004-104209945	C10orf95
A_33_P3813128	5.03074	4.6889343	NM_004186	chr3:50225956-50226015	SEMA3F
A_33_P3395274	7.453189	7.561698	NM_175907	chr18:72910548-72910489	ZADH2
A_32_P68533	6.6084547	6.8430905	NM_032180	chr2:62052180-62052121	FAM161A
A_33_P3368695	3.4695077	3.4380631	NM_001165967	chr17:8024359-8024300	HES7
A_23_P390190	9.654675	9.472174	NM_014089	chr13:25915846-25915905	NUPL1
A_33_P3413989	3.6877716	4.069604	NM_000062	chr11:57367490-57367549	SERPING1
A_33_P3531828	7.999735	7.7879333	NM_020117	chr5:145557170-145557111	LARS
A_23_P105957	10.595984	10.486848	NM_001102	chr14:69343826-69341703	ACTN1
A_24_P267592	5.0896406	5.2567596	NM_015474	chr20:35533863-35533804	SAMHD1
A_33_P3311755	9.648439	10.016979	NM_138555	chr15:69738370-69738429	KIF23
A_33_P3309491	7.886239	7.8528337	NM_005704	chr1:29653254-29653313	PTPRU
A_24_P366845	3.683772	3.9599283		chr9:115125082-115125141	
A_23_P77661	6.338768	6.5311933	NM_001130913	chr16:31765126-31765185	ZNF720

A_23_P45913	6.474785	6.584602	NM_004814	chr1:31762208-31762149	SNRNP40
A_23_P4536	2.550927	2.3900566	NM_012307	chr18:5393451-5393392	EPB41L3
A_33_P3270337	5.5100803	5.4525347	BF980704	chr10:134137631-134137572	STK32C
A_32_P137035	8.50565	8.168885	NM_006510	chr6:28871913-28871854	TRIM27
A_32_P44453	4.8840113	5.405978	NM_002194	chr2:191231564-191233830	INPP1
A_32_P129214	3.8791947	3.9799573	NR_024090	chr21:22115409-22115350	LINC00320
A_23_P251293	4.1141033	3.2379498	NM_003087	chr10:88719878-88722412	SNCG
A_23_P135977	8.461521	8.465061	NM_001008938	chr11:46765524-46765465	CKAP5
A_23_P154605	8.097656	8.359314	NM_018837	chr20:46286483-46286424	SULF2
A_23_P87591	8.738514	8.865486	NM_006530	chr12:69784126-69784185	YEATS4
A_23_P7397	2.3221061	2.3900566	NM_018930	chr5:140574844-140574903	PCDHB10
A_33_P3226357	6.217925	6.1238475	NM_004473	chr9:100618916-100618975	FOXE1
A_24_P194748	5.339329	5.174217	NM_001080485	chrX:152613798-152613857	ZNF275
A_24_P268786	8.172207	8.167431	NM_018657	chr3:169504229-169504288	MYNN
A_33_P3327200	5.603715	5.5916977	NM_001001396	chr1:203709388-203709447	ATP2B4
A_33_P3338121	12.666818	12.060354	NM_001017402	chr1:209788415-209788356	LAMB3
A_23_P14876	12.847933	13.112333	NM_003134	chr15:40328464-40328405	SRP14
A_24_P223163	6.126812	5.694359	NM_138386	chr4:164069504-164066969	NAF1
A_23_P434473	8.68201	8.874333	NM_178862	chr3:31670855-31674437	STT3B
A_33_P3354414	7.8224087	7.037764	NM_001159	chr2:201534405-201534464	AOX1
A_24_P160466	5.949521	5.736113	NM_052899	chr5:176023009-176022950	GPRIN1
A_23_P51906	12.926729	12.563514	NM_012394	chr1:161070598-161070539	PFDN2
A_33_P3389558	4.2543	3.7146046		chr16:50424886-50424827	
A_33_P3816688	6.996408	6.217925	NM_133263	chr5:149234475-149234534	PPARGC1B
A_33_P3372104	5.2516313	5.2653604		chr6:105750161-105750102	
A_33_P3234138	4.532382	4.8601313	NR_036433	chr3:101242655-101242714	FAM172BP
A_23_P208788	11.193876	10.924485	NM_033520	chr19:38795581-38795640	C19orf33
A_23_P112666	5.490445	5.6691146	NM_012186	chr1:47883514-47883573	FOXE3
A_24_P53519	10.669943	10.458807	NM_005483	chr19:4443140-4443199	CHAF1A
A_32_P21474	6.812612	6.9040623	NM_001010844	chr6:79607930-79607989	IRAK1BP1
A_23_P59718	6.740485	6.9952574	NM_003130	chr7:87835338-87835279	SRI
A_23_P46333	9.6855135	9.706216	NM_007358	chr1:93602318-93602377	MTF2
A_23_P65699	8.338407	8.724403	NM_025137	chr15:44855231-44855172	SPG11
A_33_P3840512	6.928552	7.1028876	NM_014252	chr13:41383905-41383964	SLC25A15
A_33_P3807006	3.6247807	2.3900566	AF279773	chr7:136468900-136468841	LOC349160
A_33_P3420204	7.2870216	6.82589	NM_001098482	chr19:18893076-18893135	CRTC1
A_24_P76740	7.9121504	8.154556	NM_032449	chr1:52818536-52818477	CC2D1B
A_23_P149249	10.858258	11.116295	NM_173852	chr1:155142299-155142010	KRTCAP2
A_24_P22981	2.3221061	3.6204371	NM_021047	chr19:20003475-20003534	ZNF253
A_23_P6935	8.625021	8.949412	NM_198793	chr3:107779622-107778363	CD47
A_33_P3408898	7.397156	7.387179	NM_001008739	chr6:42858090-42858031	C6orf226
A_32_P70927	2.3221061	2.3900566	NM_207339	chrX:55117033-55117813	PAGE2
A_33_P3335535	3.7704906	3.2685897	NR_040046	chr19:35899490-35899549	LOC100128682
A_24_P99090	6.5285797	7.286704	NM_018204	chr13:53035863-53035922	CKAP2
A_23_P89455	8.527391	8.702091	NM_005827	chr17:47781532-47781473	SLC35B1
A_33_P3383283	9.06436	8.825143	NM_032977	chr2:202086244-202086303	CASP10
A_33_P3225600	7.6359453	7.929824	NM_006164	chr2:178095752-178095693	NFE2L2
A_33_P3271241	10.761893	10.87684	NM_021129	chr10:71973305-71973246	PPA1
A_24_P179611	12.063523	11.958658	NM_003292	chr1:186324888-186324829	TPR
A_23_P104689	7.4525914	7.3075395	NM_021975	chr11:65421710-65421651	RELA
A_23_P65733	4.6234207	4.4156876	NM_014106	chr15:35273686-35273627	ZNF770
A_33_P3258008	4.2248807	3.9899778	NM_130466	chr12:109954584-109954643	UBE3B
A_33_P3352712	4.3910346	3.815249	NM_014603	chr17:73000110-73000169	CDR2L
A_33_P3336233	4.465007	3.876667	NM_014235	chrX:153714276-153714217	UBL4A
A_33_P3279708	7.1501226	7.197618	NR_002716	chr11:62609162-62609103	RNU2-1
A_24_P73599	3.5656025	3.1022263	NM_172217	chr15:81604940-81604999	IL16
A_23_P165608	5.2620764	5.403378	NM_004263	chr2:74907746-74907805	SEMA4F
A_33_P3384694	9.97642	10.031974		chr4:135865487-135865546	
A_24_P274640	10.080989	9.805195	NM_001042490	chr5:68882120-68882179	GTF2H2D

A_24_P583225	5.3844595	5.4788713	NR_026999	chr7:39821393-39821452	LINC00265
A_33_P3397314	3.2361717	2.960441	AK093443	chr9:43029095-43029036	FAM95B1
A_23_P301855	4.0996695	3.940104	NM_002338	chr3:115571342-115561379	LSAMP
A_33_P3219578	5.3770685	5.433359	U79264	chr3:147127588-147127529	ZIC1
A_23_P12199	5.459041	4.747389	NM_052943	chr1:27332155-27332096	FAM46B
A_23_P90565	3.7121015	3.9126916	NM_015910	chr2:63540419-63486522	WDPCP
A_33_P3314579	9.337106	9.277632	NM_001142625	chr17:27042719-27042660	RAB34
A_24_P137997	6.11381	6.141676	NM_030580	chr8:145999140-145999081	ZNF34
A_33_P3413325	6.147603	5.52396	NM_001271520	chr10:79396578-79396519	KCNMA1
A_33_P3839760	11.018518	10.948671	NM_017914	chr19:1279163-1279222	C19orf24
A_33_P3216337	10.766441	10.413816	NM_177439	chrX:48344619-48344678	FTSJ1
A_33_P3418025	5.3933115	5.5822864	NM_001334	chr4:156845446-156845387	CTSO
A_33_P3315519	5.1229935	4.4491844	NM_198461	chr2:100900162-100900103	LONRF2
A_23_P404606	6.0362897	6.7866545	NM_153607	chr5:172563805-172563864	CREBRF
A_23_P19543	11.741805	11.330078	NM_003137	chr6:35800957-35800898	SRPK1
A_23_P99405	8.625264	9.037729	NM_003453	chr13:20660278-20660337	ZMYM2
A_32_P180958	8.801311	8.704146	NM_016297	chr2:70507976-70508035	PCYOX1
A_33_P3400324	9.338451	9.24369	NM_024947	chr3:169805493-169805434	PHC3
A_24_P271696	2.3221061	2.3900566	NM_001097592	chrX:52241141-52241200	XAGE1A
A_33_P3245110	3.7718515	4.0288296		chr8:104029510-104029569	
A_33_P3411925	10.774226	10.818659	NM_024100	chr19:994510-994569	WDR18
A_23_P321388	3.4850574	3.677963	NM_153341	chr1:33407886-33404104	RNF19B
A_23_P57658	2.3221061	2.3900566	NM_020386	chr3:192988529-192988588	HRASLS
A_23_P60248	14.67751	14.57595	NM_003329	chr9:113007066-113006449	TXN
A_33_P3375668	14.442208	14.478071		chr22:024665373-024665314	
A_23_P90732	9.906319	9.964607	NM_001077399	chr2:219137494-219137553	PNKD
A_24_P13032	9.237066	9.378145	NM_003338	chr10:60127701-60127760	UBE2D1
A_24_P113815	3.9619133	3.3417273	NM_182838	chr1:1670380-1669848	SLC35E2
A_24_P233878	5.191	5.583713	NM_017686	chr1:118424465-118420736	GDAP2
A_33_P3284404	8.439071	8.691659	NM_145731	chr22:39770496-39770555	SYNGR1
A_23_P10591	9.9257965	9.678596	NM_001004431	chr17:81052490-81052549	METRNL
A_33_P3274478	4.167107	4.059722	NM_001080533	chr12:121159739-121159798	UNC119B
A_23_P8582	7.459077	7.7791004	NM_032581	chr7:22985197-22985138	FAM126A
A_33_P3340404	7.9766006	7.957343	NM_144643	chr4:129805401-129805342	SCLT1
A_33_P3327971	4.126813	4.444333		chrX:131352449-131352390	
A_23_P146217	5.5553613	5.7541347	NM_004874	chr8:38068198-38068257	BAG4
A_23_P80626	6.8347783	7.0032144	NM_207351	chr3:9987299-9987240	PRRT3
A_33_P3398912	9.235094	9.015271	NM_017585	chr9:136336276-136336217	SLC2A6
A_23_P318284	8.022785	7.7035174	NM_015141	chr3:32209871-32209930	GPD1L
A_23_P311192	5.994632	6.1054554	NM_178324	chr9:94841803-94841744	SPTLC1
A_23_P159101	5.1277637	5.489031	NM_022717	chr12:123950545-123950604	SNRNP35
A_23_P58967	11.30575	11.536232	NM_014827	chr1:203823011-203823200	ZC3H11A
A_23_P157879	4.225145	4.0139084	NM_002003	chr9:137801508-137801449	FCN1
A_23_P139958	6.773923	7.1106377	NM_001260	chr13:26978425-26978484	CDK8
A_33_P3413993	4.3789244	4.172011		chr11:57364942-57365001	SERPING1
A_24_P941988	7.398729	6.5320544	NM_014946	chr2:32382122-32382181	SPAST
A_23_P38181	8.093705	8.300883	NM_138619	chr17:73232979-73232920	GGA3
A_23_P385034	8.513751	8.05484	NM_001949	chr6:20493845-20493904	E2F3
A_23_P103968	7.0288687	7.2263336	NM_012067	chr1:19611233-19610614	AKR7A3
A_23_P417415	7.0022535	6.7322598	NM_147161	chr1:55075674-55075733	ACOT11
A_23_P212749	9.014381	9.405342	NM_002111	chr4:3245339-3245398	HTT
A_23_P391689	10.351247	10.643367	NM_001171155	chr19:7695482-7695541	PET100
A_33_P3287028	6.7153206	6.7052073	NM_001788	chr7:35912316-35912375	SEPT7
A_33_P3217332	4.7960186	4.976512	NM_031218	chr19:20045657-20045716	ZNF93
A_23_P8055	6.7097597	6.8324795	NM_007243	chr6:30656220-30656161	NRM
A_33_P3306113	10.234175	9.591877	NM_023007	chr1:227918994-227918935	JMJD4
A_23_P387184	5.204214	4.455132	NM_020464	chr6:138744231-138744172	NHSL1
A_23_P344481	5.735465	5.7968717	NM_152709	chr10:70652436-70652495	STOX1
A_24_P70303	3.8044171	4.2804785	NM_014405	chr17:65029115-65029174	CACNG4

A_23_P258493	9.127871	9.178626	NM_005573	chr5:126172483-126172542	LMNB1
A_23_P107465	4.229718	4.901839	NM_002277	chr17:39550065-39550006	KRT31
A_33_P3396344	3.9321976	4.007866		chr5:1597732-1597673	LOC728613
A_24_P65864	8.226385	8.059056	NM_016143	chr20:1433773-1433714	NSFL1C
A_24_P927474	9.370164	9.447096		chr12:076358613-076358672	
A_23_P69988	11.612892	11.283609	NM_002887	chr5:167946137-167946196	RARS
A_23_P34983	11.429198	11.3610325	NM_006694	chr1:153949221-153948375	JTB
A_24_P125469	2.7707314	2.3900566	NM_006033	chr18:47118360-47118419	LIPG
A_23_P126623	8.546821	8.685317	NM_002631	chr1:10478911-10478970	PGD
A_23_P372923	7.466042	8.066417	NM_001174066	chr8:38315032-38314973	FGFR1
A_32_P141923	14.254614	14.116425	NM_199290	chr17:59668045-59667986	NACA2
A_32_P53486	11.221249	11.094612	NM_001039182	chr16:30204680-30204338	BOLA2B
A_33_P3238525	5.6647162	5.6254625	BC015443	chr16:18938329-18938388	
A_33_P3223780	7.590841	7.803832	NM_002292	chr3:49159203-49159144	LAMB2
A_33_P3290562	9.054983	8.8355465	NM_000168	chr7:42000649-42000590	GLI3
A_33_P3242923	2.3221061	2.3900566	NR_038358	chr14:51832186-51832245	LINC00640
A_24_P381494	6.6608334	6.944681	NM_000617	chr12:51380358-51380299	SLC11A2
A_23_P359870	5.0995274	5.1407185	AJ312026	chr8:10984299-10984240	
A_32_P69368	10.757356	10.516994	NM_002166	chr2:8824282-8824341	ID2
A_23_P213562	6.8228855	7.18543	NM_001992	chr5:76030669-76030728	F2R
A_33_P3474859	8.990242	8.84856	XR_246700	chr9:68414554-68414613	LINC00537
A_23_P33759	9.781686	10.748498	NM_004753	chr1:12628169-12628110	DHRS3
A_23_P55388	4.781681	3.5263214	NM_018143	chr17:40009999-40009940	KLHL11
A_33_P3322859	6.5360694	6.6629696	NM_018645	chr2:239147315-239147256	HES6
A_23_P31532	6.892542	7.044625	NM_080660	chr7:138711492-138711286	ZC3HAV1L
A_33_P3221925	4.18773	4.079998		chr6:121400687-121400628	
A_33_P3482534	6.749457	7.0723777	BC043571	chr20:15873038-15872979	LOC613266
A_33_P3220437	13.467536	13.449977	NM_001167942	chr19:4653833-4653892	TNFAIP8L1
A_23_P339633	6.625289	6.1835327	NM_174931	chr2:37319374-37321259	GPATCH11
A_24_P349466	7.2700787	7.1309085	NM_001005354	chr12:53837468-53837527	PRR13
A_33_P3407601	5.275268	5.3219604		chr17:020841137-020841078	
A_23_P259292	5.0140977	5.6736593	NM_015645	chr11:119209954-119209895	C1QTNF5
A_33_P3343220	10.184152	9.974408	NM_001029885	chr1:1264177-1264236	GLTPD1
A_33_P3283900	6.311244	6.240066	NM_004937	chr17:3566287-3566346	CTNS
A_33_P3290919	8.750403	8.618396	NM_001172415	chr9:33255087-33255028	BAG1
A_33_P3225630	5.6514206	5.8593483		chr19:46580350-46580291	IGFL4
A_33_P3383936	4.4684305	4.6033783	NM_014966	chr3:47884600-47884659	DHX30
A_33_P3676746	8.858656	8.723244	NM_019589	chr14:75302833-75302892	YLPM1
A_33_P3352877	6.630275	6.254555	NM_015087	chr13:36875912-36875853	SPG20
A_33_P3323718	6.568947	5.9821286	NM_001008224	chr15:70947058-70946999	UACA
A_24_P767901	6.194353	6.338768		chr4:074952233-074952174	
A_24_P341677	12.528813	12.713932		chr14:035857541-035857482	
A_23_P396858	6.3526225	6.1182137	NM_031866	chr10:35927437-35927378	FZD8
A_33_P3371237	4.0694017	4.380679	XM_001718671		LOC100131514
A_33_P3309662	11.595769	11.905529	NM_001039848	chr19:1105673-1105732	GPX4
A_33_P3216570	4.196048	4.494543	XM_003119481		MUC5AC
A_24_P112750	7.0761576	7.1097565	NM_005653	chr12:51492572-51489778	TFCP2
A_33_P3315836	4.2974024	4.4732285		chr18:019811943-019812002	
A_24_P228717	6.435534	6.619018	NM_002872	chr22:37627281-37622795	RAC2
A_23_P414899	6.677109	6.6598873	NM_018259	chr11:43465098-43465628	TTC17
A_33_P3227258	3.9134219	3.4781308		chr5:017585670-017585611	
A_32_P34826	3.2736387	2.3900566	NR_026543	chr21:40977927-40977868	C21orf88
A_33_P3248863	13.399237	13.399237	NM_213597	chr17:8272138-8272079	KRBA2
A_23_P32913	8.592503	8.64294	NM_031905	chr7:102739067-102739126	ARMC10
A_24_P37441	7.533314	8.5293255	NM_002610	chr2:173463273-173463332	PDK1
A_24_P213228	11.141994	11.386994		chr7:055799965-055800024	
A_33_P3410459	11.227634	11.254095	NM_001204255	chr4:77079975-77079916	SCARB2
A_24_P70888	9.01923	8.9718895	NM_012401	chr22:50713889-50713830	PLXNB2
A_23_P142560	6.0368824	6.73715	NM_014795	chr2:145146320-145146261	ZEB2

A_23_P376799	10.729543	10.976305	NM_015262	chr10:46287947-46288006	FAM21C
A_23_P167444	10.89695	10.759525	NM_014886	chr5:74066571-74066630	NSA2
A_33_P3256334	6.4323444	6.414828	NM_006249	chr12:11420185-11420126	PRB3
A_33_P3359223	9.565765	9.801134	NM_001256699	chr9:140147875-140147934	C9orf173
A_24_P376391	7.791456	7.896248	NM_015103	chr3:129274457-129274398	PLXND1
A_33_P3447441	6.2034636	5.6866875	AL713660	chr4:10069830-10069771	LOC202025
A_33_P3327192	4.671506	4.841236	DA142060	chr6:24751871-24751930	
A_33_P3414012	4.7084236	4.527561	BG182298	chr4:174243347-174243288	
A_33_P3388564	3.9261038	3.4610558	NM_144607	chr17:7761737-7761796	CYB5D1
A_24_P313096	8.140058	8.3233595	NM_006322	chr13:113140147-113140088	TUBGCP3
A_33_P3267296	11.005549	10.7776785	NM_016594	chr12:49317625-49317566	FKBP11
A_33_P3348744	8.384204	8.453252		chr12:114987607-114987666	
A_33_P3421913	4.5182157	4.629124	NM_014333	chr11:115044486-115044427	CADM1
A_33_P3867584	6.316764	6.2275076		chr6:114189283-114189224	LOC285758
A_33_P3232354	3.7619832	4.227883	NM_183242	chr1:92604920-92604979	BTBD8
A_23_P390172	7.3644357	7.492399	NM_021133	chr1:182543026-182542967	RNASEL
A_23_P81248	11.170785	11.10861	NM_005642	chr5:140698187-140698128	TAF7
A_24_P120115	5.739076	6.1027884	NM_003879	chr2:202025198-202025257	CFLAR
A_33_P3301970	7.730288	8.029421	NM_018571	chr2:202343264-202343323	STRADB
A_33_P3213752	9.402147	9.230438	NM_016408	chr20:31946717-31946658	CDK5RAP1
A_24_P188325	9.784252	9.424498	NM_015984	chr1:192990312-192990253	UCHL5
A_33_P3277096	4.0790615	3.9430315	AJ315539	chr6:31680458-31680399	
A_23_P7361	8.545389	8.732645	NM_024090	chr4:110971230-110971171	ELOVL6
A_23_P110941	6.965787	7.2925625	NM_001512	chr6:52843197-52843138	GSTA4
A_33_P3332982	4.7834463	5.094256	M80915	chr22:22724252-22724311	
A_33_P3321205	3.6319938	3.318596	NM_001159531	chr14:101003549-101003490	BEGAIN
A_23_P431330	6.4483614	5.9866714	NM_175918	chr4:1389548-1389607	CRIPAK
A_33_P3221293	8.014406	7.616477	NM_004403	chr7:24738673-24738614	DFNA5
A_23_P59836	7.5744853	7.147695	NM_018396	chr7:128142516-128142575	METTL2B
A_33_P3326772	11.22479	11.25917	NM_001008395	chr7:99751770-99751829	LAMTOR4
A_32_P82475	8.371309	8.093705	NR_003138	chr14:95999378-95999319	SNHG10
A_23_P88099	5.745365	6.4676266	NM_024979	chr13:113752718-113752777	MCF2L
A_23_P156842	10.602222	10.579005	NM_004280	chr6:8097591-8097532	EEF1E1
A_32_P62090	3.709077	3.778541	NM_001282544	chr17:55822572-55822513	CCDC182
A_33_P3316410	5.562982	5.8425093	NR_026789	chr8:12219964-12220023	FAM66A
A_23_P203841	5.9756556	5.9895625	NM_013449	chr12:56990407-56990348	BAZ2A
A_33_P3380346	4.8501	4.7820888	NM_133330	chr4:1980499-1980558	WHSC1
A_33_P3350232	5.909332	5.6822624	AK127825	chr14:76961394-76961335	
A_33_P3219942	4.0744185	4.521762	NM_001008707	chr14:100404189-100404248	EML1
A_23_P35484	3.6529229	2.3900566	NM_006229	chr10:118365027-118368585	PNLIPRP1
A_23_P393401	4.871009	4.877689	NR_003610	chr16:70010123-70010086	PDXDC2P
A_23_P48826	6.629459	6.756272	NM_182985	chr15:45059866-45059925	TRIM69
A_23_P15798	5.315076	5.405517	NM_031854	chr17:39279576-39279517	KRTAP4-12
A_23_P131263	4.343512	2.3900566	NM_033066	chr2:202509723-202509664	MPP4
A_33_P3302448	7.2532177	7.2077746	NM_001101391	chr19:2289833-2289774	LINGO3
A_33_P3216192	13.312776	13.30039		chr14:064034968-064035027	
A_33_P3422614	4.459026	4.6750994		chr10:16769124-16769065	RSU1
A_23_P163143	9.730458	9.733805	NM_203488	chr14:75520272-75520213	ACYP1
A_23_P212706	10.035442	9.835497	NM_022488	chr3:112255415-112255356	ATG3
A_33_P3287418	6.142747	6.2562675		chr22:023478444-023478385	
A_33_P3399778	4.9746885	4.6533613		chr2:192107768-192107709	
A_23_P60130	7.212036	5.446129	NM_052886	chr8:120257557-120257616	MAL2
A_23_P162579	6.235501	6.006038	NM_014365	chr12:119617464-119624868	HSPB8
A_33_P3261743	3.73493	3.3849654	NM_001145290	chr11:124955854-124955913	SLC37A2
A_33_P3252809	4.238879	3.5142505		chr22:045725231-045725290	
A_33_P3260026	4.91881	4.332615	NM_001185096	chr9:133993162-133993221	AIF1L
A_23_P42514	12.036167	12.043724	NM_030939	chr6:24705229-24705170	C6orf62
A_23_P206661	10.395048	10.247489	NM_000903	chr16:69744333-69744274	NQO1
A_33_P3292126	9.447555	10.1094		chr7:55810519-55810460	XLOC_014512

A_23_P96383	6.9454613	6.3701143	NM_006307	chrX:38008772-38008713	SRPX
A_23_P420831	3.8806016	4.11507	NM_052828	chr6:30119937-30119878	TRIM10
A_23_P50919	8.747749	8.732461	NM_006216	chr2:224842295-224840597	SERPINE2
A_23_P119562	8.148584	8.629563	NM_001928	chr19:863247-863306	CFD
A_24_P23258	8.824706	9.478962	NM_015124	chr22:47075419-47075478	GRAMD4
A_24_P294851	9.092338	9.490767	NM_006355	chr6:25984697-25984756	TRIM38
A_33_P3415445	5.7553544	5.8610554	NM_006609	chr2:128065062-128065003	MAP3K2
A_24_P354496	5.9562774	5.5748367	NR_024008	chr4:184018294-184018235	WWC2-AS2
A_33_P3272347	9.75809	10.094004	AK123720	chr19:46893780-46893721	LOC100128107
A_23_P336854	14.233127	14.350332		chr20:019804289-019804230	
A_33_P3301221	2.661772	3.1621957	AK128431	chr1:2306367-2306308	LOC100129110
A_32_P43050	9.158234	8.997834	NM_004477	chr20:029633913-029633972	FRG1
A_23_P153383	13.038521	13.04334	NM_015414	chr19:5691418-5691555	RPL36
A_23_P30745	10.8740425	11.010656	NM_014623	chr6:42980170-42980111	MEA1
A_23_P1981	4.527407	4.5921555	NM_000207	chr11:2181123-2181064	INS
A_32_P52609	9.301134	8.985969	NM_145693	chr2:11967107-11967166	LPIN1
A_33_P3347320	4.7481575	4.3031263	BC017676	chr10:88725492-88725433	ADIRF-AS1
A_33_P3420841	5.1033015	4.6872306		chr22:36924850-36924791	EIF3D
A_24_P932736	6.5265474	6.5663595	XM_005273634	chr8:28910462-28910521	HMBOX1
A_24_P270890	8.725749	8.778378	NM_001007226	chr17:47677059-47677000	SPOP
A_33_P3279765	4.7089562	4.8225546		chr20:57601087-57601028	ATP5E
A_33_P3369063	5.38536	4.855671	AB529231	chr11:59159089-59159030	
A_33_P3396492	5.0086346	5.1229935	NM_022478	chr14:23517008-23516949	CDH24
A_23_P254831	4.056204	3.8814557	NM_002364	chrX:30237708-30237767	MAGEB2
A_32_P305888	3.7087674	2.3900566	NM_024577	chr5:148381027-148380968	SH3TC2
A_33_P3230948	5.397432	5.4110737	NR_002825	chr19:50478872-50478931	SIGLEC16
A_23_P33433	5.689207	5.71321	NM_001042539	chr16:29821633-29821692	MAZ
A_24_P257579	2.7901242	2.3900566	NM_022140	chr5:111530291-111519800	EPB41L4A
A_23_P72387	6.6139936	6.7488513	NM_001134647	chr4:7760626-7760567	AFAP1
A_24_P256337	7.9762077	8.643824	NM_201269	chr1:91381899-91381840	ZNF644
A_33_P3295786	4.7532444	4.8984876	NM_001042693	chr1:53122668-53122727	FAM159A
A_24_P280706	7.7721267	7.7312455	NM_006704	chr13:53231679-53231737	SUGT1
A_24_P945000	7.478943	7.7452593	NM_182620	chr17:57187821-57187762	SKA2
A_33_P3713035	5.7476063	5.5945225	AL122087	chr7:15874795-15874736	LOC221814
A_23_P127915	6.5048122	5.9912634	NM_030906	chr11:8413725-8413666	STK33
A_33_P3381022	4.192231	3.7910666	BC017037	chr1:6294564-6294505	ICMT
A_23_P164737	9.463057	9.467686	NM_004831	chr19:16685876-16685817	MED26
A_23_P63153	9.551503	9.6855135	NM_007204	chr1:112309563-112309622	DDX20
A_33_P3279545	5.1547446	5.4510813	NM_005734	chr11:33375864-33375923	HIPK3
A_23_P332820	5.390507	5.283816	NM_002190	chr6:52055156-52055215	IL17A
A_23_P87709	2.3221061	2.3900566	NM_024829	chr12:14656690-14656631	PLBD1
A_23_P204246	5.3185954	5.1614933	NM_004426	chr12:9093129-9093188	PHC1
A_24_P566701	8.062497	8.204364	NM_170679	chr5:133509660-133502919	SKP1
A_33_P3256883	4.5650954	4.527732		chr19:050646123-050646064	
A_33_P3405911	5.0557914	5.021658		chrX:73049007-73049066	TSIX
A_23_P90143	15.208674	15.199939	NM_012423	chr19:49994724-49994783	RPL13A
A_33_P3213910	4.842542	5.1829166	NM_181720	chr1:161019478-161019419	ARHGAP30
A_23_P144005	3.8659945	3.4658313	NM_015931	chr3:8661402-8661343	SSUH2
A_33_P3292994	4.7491884	4.329031		chr17:074087919-074087978	
A_33_P3383059	9.590746	9.3168335		chr19:052104986-052105045	
A_23_P81973	5.2673774	5.2607946	NM_014234	chr6:33173493-33173654	HSD17B8
A_33_P3372084	7.9090395	8.1221285		chr5:180409466-180409525	
A_23_P154771	6.524664	6.453749	NM_080611	chr20:30449397-30449338	DUSP15
A_33_P3326530	4.4987373	4.803059	NM_018307	chr17:30510215-30510274	RHOT1
A_23_P22352	8.283025	8.221854	AK001072	chr10:13735772-13735713	FRMD4A
A_23_P213832	7.451811	7.5643873	NM_032566	chr5:147693680-147693739	SPINK7
A_24_P122524	9.058696	8.570539	NM_006784	chr1:118502437-118502496	WDR3
A_23_P79942	7.41978	7.6445456	NM_153638	chr20:3899396-3903902	PANK2
A_24_P919452	6.9813776	6.7078323		chr17:33733963-33733904	

A_33_P3355232	8.049938	7.8062553	NM_145049	chr5:158712890-158712949	UBLCP1
A_23_P76538	8.796535	9.188648	NM_017899	chr12:117476821-117476762	TESC
A_23_P364766	7.2278385	6.960344	NM_001009608	chr20:10603796-10603855	SLX4IP
A_23_P134854	4.7580843	4.4460244	NM_194284	chr8:8561413-8561472	CLDN23
A_33_P3418125	6.5471992	6.239439	NM_006851	chr12:75895656-75895715	GLIPR1
A_33_P3276222	7.903441	8.378722		chr14:035483984-035484043	
A_23_P58706	4.6988163	5.208634	NM_001040129	chr5:147665702-147665761	SPINK13
A_23_P25929	9.517223	9.66034	NM_014239	chr14:75475826-75475885	EIF2B2
A_33_P3367899	5.2452483	4.3741517	NM_005479	chr10:99081604-99081663	FRAT1
A_23_P63067	12.591557	12.5987625	NM_033657	chr1:155708157-155708216	DAP3
A_33_P3356941	4.0716233	3.998157		chr17:028183142-028183201	
A_23_P14062	10.645393	10.530521	NM_020401	chr12:69136376-69136435	NUP107
A_23_P6223	5.625507	5.5592794	NM_020706	chr21:33044126-33044067	SCAF4
A_33_P3286046	8.889839	8.667028	NM_015448	chr10:103361033-103361092	DPCD
A_23_P363255	2.832231	2.3900566	NM_025214	chr18:52568911-52568852	CCDC68
A_24_P416961	6.7226305	6.944234	NM_001670	chr22:19957509-19957450	ARVCF
A_33_P3311371	6.473361	5.774815	NM_198042	chr8:22447120-22447179	PDLIM2
A_33_P3407564	7.255102	7.2912445		chr6:053041275-053041334	
A_32_P124708	5.3212285	5.440895	NM_004852	chr18:55158392-55158451	ONECUT2
A_33_P3251896	5.709736	6.3984184	NM_004307	chr4:40816298-40816239	APBB2
A_33_P3305254	6.2977524	6.578757	NM_005047	chr9:123583243-123583184	PSMD5
A_23_P345564	4.3114743	3.9791381	NM_182647	chr20:62731089-62731148	OPRL1
A_33_P3214432	6.742723	6.8736377	NM_080660	chr7:138720567-138720508	ZC3HAV1L
A_33_P3272442	5.1016927	4.8570404	NR_103455	chr6:28089803-28089744	ZSCAN16-AS1
A_23_P98261	9.686041	9.596842	NM_006396	chr11:65339032-65339091	SSSCA1
A_23_P352435	4.621665	4.4468956	NM_002926	chr4:3433293-3433352	RGS12
A_23_P20777	9.500109	9.598134	NM_033117	chr9:125004149-125004090	RBM18
A_33_P3323929	3.9126916	3.8416853	AK097855	chr10:126915976-126915917	FLJ40536
A_24_P102895	5.9559116	6.38688	NM_015023	chr1:27634323-27634382	WDTC1
A_33_P3319463	6.310614	6.4130936	NM_001145261	chr10:51582877-51582936	NCOA4
A_23_P20363	9.438384	9.6104765	NM_016010	chr8:79629610-79629669	ZC2HC1A
A_23_P47800	9.656734	9.739573	NM_019887	chr12:122692617-122692558	DIABLO
A_23_P371824	9.015497	8.690077	NM_020127	chr1:151555864-151555923	TUFT1
A_23_P20002	6.8818893	7.1748886	NM_020319	chr7:16640171-16640112	ANKMY2
A_33_P3312661	3.7141597	3.378283	AK090819	chr17:78437315-78437256	
A_23_P63010	10.647438	10.724686	NM_181746	chr1:150938158-150938099	CERS2
A_32_P219520	7.1136684	6.692981	NM_014350	chr5:118730231-118730290	TNFAIP8
A_23_P60259	8.367019	8.299614	NM_018112	chr9:108537196-108537255	TMEM38B
A_23_P363896	12.871479	12.847933	NR_027162	chr17:16345210-16345270	C17orf76-AS1
A_24_P406814	7.386234	6.987436	NM_014661	chr10:126308189-126308130	FAM53B
A_24_P36285	4.774487	5.355989	NM_013398	chr19:44612163-44612222	ZNF224
A_33_P3241771	5.2643156	5.211848	XR_109230	chr15:101324317-101324258	LOC440313
A_33_P3259058	10.366955	10.089731	NM_001128592	chr6:3267946-3268005	PSMG4
A_24_P290927	5.5901546	5.524389	NM_014847	chr1:154199802-154201116	UBAP2L
A_33_P3329240	11.12475	11.134429		chr8:37333475-37333416	
A_33_P3330099	7.039104	6.9746675	NM_001669	chrX:2822073-2822014	ARSD
A_23_P141960	6.4284997	6.666659	NM_203344	chr19:40947203-40947144	SERTAD3
A_23_P81690	13.287257	13.253873	NM_001865	chr6:75947606-75947547	COX7A2
A_33_P3416420	5.5514317	5.3044133	NM_145062	chr6:116980048-116979989	ZUFSP
A_33_P3226605	10.478286	10.582666	NM_033222	chr9:15464389-15464330	PSIP1
A_33_P3443165	10.587971	10.373914	NM_001015885	chr20:55953452-55953511	RAE1
A_23_P57697	9.829681	9.873704	NM_020865	chr3:153993757-153993698	DHX36
A_32_P206401	7.2338705	7.143962	NM_181701	chr9:139098749-139098690	QSOX2
A_23_P112429	15.340809	15.226828	NM_007209	chr9:127623708-127622495	RPL35
A_33_P3296858	6.2001753	6.3168545		chr10:43009403-43009344	ZNF37BP
A_23_P76598	10.897618	10.974328	NM_152726	chr13:22067057-22066998	MICU2
A_24_P384397	9.280619	9.32595	NM_133452	chr19:10431850-10431791	RAVER1
A_23_P78871	6.239439	5.8521075	NM_024076	chr19:34304755-34304814	KCTD15
A_23_P151093	8.729408	8.532419	NM_001040436	chr12:32903682-32903012	YARS2

A_33_P3365805	8.277914	8.073342	NM_006493	chr13:77576548-77576607	CLN5
A_24_P804667	6.463276	6.2864375	NM_001043229	chr11:62434315-62434374	METTL12
A_24_P29975	10.957935	10.801881	NM_030978	chr9:127637422-127639207	ARPC5L
A_33_P3862354	5.9623246	6.016718	NM_025204	chr22:50636274-50636333	TRABD
A_33_P3232173	5.2400618	5.373209		chr13:20276734-20276675	PSPC1
A_23_P37598	8.373169	9.041034	NM_012428	chr15:73853376-73853317	NPTN
A_33_P3213468	4.633793	4.6143723		chr5:10650093-10650152	ANKRD33B
A_32_P184727	13.247529	12.902962	NM_002265	chr17:45760373-45760432	KPNB1
A_33_P3228510	4.175599	3.5352712	NM_001258330	chr20:34773197-34773256	EPB41L1
A_33_P3293082	3.296152	3.4106045	NR_040117	chr9:90473045-90472986	LOC392364
A_33_P3380493	3.5554516	3.04725	NM_031286	chr1:26606514-26606573	SH3BGRL3
A_23_P319423	5.30706	5.622317	NM_003740	chr6:39156880-39156821	KCNK5
A_23_P150590	4.0929627	4.59306	NM_080866	chr11:63176314-63177295	SLC22A9
A_23_P130677	4.760214	4.8111076	NM_018687	chr19:11352360-11352419	C19orf80
A_24_P346101	7.0095544	6.9778767	NM_138492	chr5:145139599-145139540	PRELID2
A_24_P398940	10.476372	9.740197	NM_138423	chr15:44707627-44707686	CASC4
A_23_P257609	12.523547	12.549358	NM_000992	chr3:52028113-52028054	RPL29
A_24_P15586	4.9177094	4.8061857	NM_182529	chr7:108203551-108203492	THAP5
A_23_P502678	9.430051	9.707967	NM_031940	chr8:38848823-38848764	TM2D2
A_23_P111141	8.864455	8.840908	NM_004639	chr6:31608171-31608034	BAG6
A_33_P3793307	6.275092	6.5414033	NR_036496	chr2:61368978-61368919	LOC339803
A_33_P3307253	4.701279	3.7359993	NM_012093	chr1:77763572-77763631	AK5
A_24_P355493	5.973171	6.10776	NM_022126	chr10:126177105-126185549	LHPP
A_24_P19677	3.7189708	3.1755428	NM_173065	chr1:24496050-24495991	IFNLR1
A_33_P3412160	5.2467513	5.6426716	NM_001048210	chr1:109486200-109486141	CLCC1
A_33_P3413224	3.9832618	4.388271	BJ995728	chr15:59981503-59981562	
A_23_P31273	3.905865	3.964651	NM_001635	chr7:38423860-38423801	AMPH
A_33_P3414482	12.555401	12.371514	NM_002080	chr16:58741096-58741037	GOT2
A_23_P6196	6.8310814	6.504403	NM_016558	chr20:34542140-34542081	SCAND1
A_23_P250516	3.2111251	2.3900566	XR_243765	chr17:76613698-76613639	
A_23_P204630	7.3867755	7.8722267	NM_021229	chr12:96052034-96051975	NTN4
A_24_P35169	6.644842	6.553556	NM_021167	chr7:92087752-92087811	GATAD1
A_24_P340853	5.1195984	4.6814823	NM_032789	chr8:145059111-145059052	PARP10
A_24_P76879	10.560606	10.461201	NM_003309	chr6:116598155-116598096	TSPYL1
A_23_P93282	11.317156	11.373219	NM_003535	chr6:27858262-27858203	HIST1H3J
A_33_P3228325	11.390165	11.1672	NM_001080391	chr2:231410216-231410275	SP100
A_23_P22444	7.514761	7.191245	NM_002621	chrX:47483742-47483683	CFP
A_23_P345118	6.8844514	6.534383	NM_002648	chr6:37143102-37143161	PIM1
A_33_P3322075	4.427431	4.1768503	AK055942	chr16:88128309-88128368	
A_24_P401768	3.272615	2.3900566	NM_130847	chr11:94554977-94563264	AMOTL1
A_23_P167227	9.335227	9.299998	NM_005327	chr4:108956110-108956169	HADH
A_23_P8380	8.7350025	8.776291	NM_024033	chr7:134851035-134850976	C7orf49
A_33_P3213832	3.9535503	3.703167	DA967691	chr5:122390279-122390338	
A_33_P3812669	9.237932	9.9257965	NM_031412	chr12:10375579-10375637	GABARAPL1
A_23_P328600	7.0359144	6.991062	NM_024959	chr12:113737050-113736991	SLC8B1
A_33_P3409934	7.8131366	7.997722	NM_001013437	chr18:12984132-12986862	SEH1L
A_33_P3494875	8.926376	8.503619		chr2:114464807-114464748	
A_33_P3233560	5.660445	5.5037045		chr10:127495780-127495721	UROS
A_23_P502371	9.525011	9.33168	NM_005392	chr9:96441749-96441808	PHF2
A_23_P50638	4.348324	4.4364705	NM_052972	chr19:4537515-4537456	LRG1
A_23_P431381	7.421149	7.6352296	NM_001134875	chr14:105965354-105965413	C14orf80
A_33_P3209962	3.6546998	3.9641287	NM_153819	chr11:64508971-64508912	RASGRP2
A_33_P3289446	5.379275	5.126113	AK125976	chr2:130564165-130564106	LOC100131048
A_32_P157965	8.886275	8.537611	NM_003908	chr20:32681481-32678350	EIF2S2
A_23_P129157	6.8048954	7.22361	NM_024608	chr15:75647373-75647432	NEIL1
A_32_P435367	13.036181	12.9939575	NM_002810	chr1:151239868-151239927	PSMD4
A_33_P3339311	6.2406435	5.696399	BX387507	chr1:229576105-229576046	
A_24_P143189	13.42417	13.456213	NM_021109	chrX:12994992-12995051	TMSB4X
A_23_P3552	4.916588	4.956098	BC009198	chr16:21822139-21822080	

A_23_P55873	6.718322	6.529649	NM_017721	chr19:14041283-14041342	CC2D1A
A_33_P3332156	5.013651	4.931391	NM_001003665	chr1:226784622-226784681	C1orf95
A_23_P92093	3.9275622	3.4695077	NM_001407	chr3:48674303-48674244	CELSR3
A_33_P3236868	6.8111806	6.760889	NM_005952	chr16:56717962-56718021	MT1X
A_33_P3257518	5.6169224	5.1146674	NR_039985	chr14:62121233-62121292	FLJ22447
A_23_P86822	7.913951	7.8217106	NM_014679	chr11:95564793-95564852	CEP57
A_33_P3354771	6.016176	5.937294	AK057161	chr19:19288990-19288931	MEF2BNB
A_23_P141005	7.9568305	8.254663	NM_001144	chr16:56395757-56395698	AMFR
A_23_P70897	4.8395214	4.4075108	NM_017984	chr7:99998563-99998504	ZCWPW1
A_33_P3332145	12.042385	9.783083	NM_002951	chr20:35869710-35869769	RPN2
A_32_P75284	10.755817	10.943184	NM_032116	chr13:30776968-30776909	KATNAL1
A_23_P399001	10.374713	10.557524	NM_016463	chr5:139062536-139062595	CXXC5
A_33_P3254335	7.6959944	6.9350595	NM_207406	chr4:42113016-42112957	BEND4
A_33_P3397693	8.832397	8.935998	NM_003659	chr2:178403842-178403901	AGPS
A_23_P22682	9.399107	9.399486	NM_016608	chrX:100809416-100809475	ARMCX1
A_23_P29204	8.30494	8.289926	NM_016498	chr22:30824628-30824687	MTFP1
A_33_P3235053	11.137785	11.116045	NM_033452	chr17:73870306-73870247	TRIM47
A_33_P3295523	9.862705	10.082978	NM_005052	chr17:79992018-79992077	RAC3
A_23_P73429	5.0708447	4.920356	NM_005335	chr3:121350254-121350229	HCLS1
A_24_P175909	12.715825	12.071268	NM_004990	chr12:57910373-57910432	MARS
A_32_P110390	5.43677	5.506782	NM_173490	chr5:72424286-72424345	TMEM171
A_33_P3257030	9.978022	10.228441	NM_006455	chr17:39958266-39958207	LEPREL4
A_23_P436138	4.498003	4.5253034	NM_145114	chr14:65550893-65550834	MAX
A_33_P3308387	10.689089	11.1665945	NM_032982	chr7:142989731-142989790	CASP2
A_33_P3217719	5.6676865	5.7597337	NM_005077	chr9:84225208-84225149	TLE1
A_24_P417189	5.744159	5.583158	NM_001395	chrX:152915952-152916011	DUSP9
A_24_P273203	12.200829	12.261019		chr3:75674241-75674182	LOC100287195
A_23_P39034	11.819845	11.861443	NM_003072	chr19:11172673-11172732	SMARCA4
A_24_P345377	9.948254	9.805977	NM_018178	chr1:150618832-150618773	GOLPH3L
A_33_P3222664	7.507099	7.621814		chr6:029800627-029800568	
A_23_P468	4.461025	4.5560465	NM_021179	chr1:169390697-169390638	CCDC181
A_33_P3249046	7.3181543	7.918112	NM_001171092	chrX:106174032-106174091	CLDN2
A_33_P3234580	7.1654177	7.3429127	NM_000050	chr9:133376601-133376660	ASS1
A_24_P106953	6.119257	5.8470173	NM_025072	chr9:130886066-130886007	PTGES2
A_32_P109572	7.156339	7.2255926	NM_001533	chr19:39327359-39327300	HNRNPL
A_33_P3334515	10.738668	11.580002	NM_001282213	chr14:21484991-21484932	NDRG2
A_23_P218784	9.576632	9.851071	NM_006386	chr22:38889757-38888104	DDX17
A_24_P330303	8.627066	8.446472	NM_001042481	chr14:52196628-52196687	FRMD6
A_23_P111054	9.308984	9.201099	NM_021062	chr6:26043565-26043506	HIST1H2BB
A_24_P351283	4.5197463	4.509747	NM_018000	chr2:216810439-216810380	MREG
A_33_P3374878	7.1719007	7.885379	NM_024582	chr4:126412840-126412899	FAT4
A_23_P146066	6.6155086	5.92605	NM_003114	chr8:101252969-101253147	SPAG1
A_33_P3250555	3.9821193	4.0093036	AF295728	chr18:1278629-1278570	
A_24_P269619	9.344716	9.754988	NM_001359	chr8:91031178-91031356	DECR1
A_23_P94660	10.420882	10.224163	NM_018201	chr9:131572622-131572681	TBC1D13
A_33_P3336273	4.5866156	4.804265	NM_001024383	chr12:78604629-78604688	NAV3
A_23_P50389	7.275964	7.359537	NM_020378	chr19:55998511-55998570	NAT14
A_33_P3318237	5.4452553	5.5901546	NM_006244	chr11:64695836-64695895	PPP2R5B
A_23_P364890	4.4974265	4.1619663	NM_014771	chr16:30785578-30785637	RNF40
A_23_P72157	4.786025	4.164139	NM_032219	chr4:675759-675700	MFSD7
A_33_P3343720	4.3546457	4.261179	NM_005747	chr1:22336290-22336349	CELA3A
A_33_P3368750	9.619779	8.70127	NM_001104554	chr15:69699895-69699954	PAQR5
A_23_P318646	12.630185	12.692611	NM_001014	chr6:34392872-34392582	RPS10
A_24_P154006	13.242724	13.207613	NM_005005	chr8:125562088-125562147	NDUFB9
A_33_P3368500	9.297083	9.340292	NM_016004	chr20:42275733-42275792	IFT52
A_23_P34757	8.038364	8.313209	NM_018040	chr1:217604072-217604013	GPATCH2
A_23_P403398	5.6146317	5.565601	NR_002186	chr7:30411448-30411507	DKFZP586I1420
A_23_P68868	12.147997	11.925346	NR_002323	chr22:31371927-31371986	TUG1
A_33_P3350553	7.616419	7.528852		chrX:47516577-47516518	UXT

A_32_P109794	8.530966	7.4823713	NM_145251	chr14:53241406-53241465	STYX
A_33_P3322192	4.4761906	4.5299096		chr12:094534832-094534891	
A_23_P80362	10.528363	10.525234	NM_005008	chr22:42071130-42071071	NHP2L1
A_24_P154573	6.0248156	5.6543145	NM_145291	chr4:4323435-4323494	ZBTB49
A_24_P71700	7.8305397	7.9587917	NM_145166	chr3:42708933-42708992	ZBTB47
A_23_P73809	2.3221061	2.3900566	NM_020871	chrX:114345721-114345662	LRCH2
A_24_P390668	5.0683126	4.2900314	NM_005892	chr17:43323637-43323696	FMNL1
A_33_P3398437	5.2607946	5.647627	AF176922	chr15:42063094-42063153	
A_33_P3324137	6.2070913	6.176173	BC015320	chr20:39667260-39667319	PRO0628
A_24_P706314	10.094971	9.769011	NM_194247	chr2:178087920-178087979	HNRNPA3
A_33_P3399453	7.162508	7.0828576	NM_006135	chr1:113212885-113212945	CAPZA1
A_33_P3280521	7.2477465	7.250166	NM_021647	chr4:170907809-170907750	MFAP3L
A_24_P405981	5.115642	4.5986233	NM_012382	chr5:40712328-40712269	TTC33
A_24_P200854	4.75928	4.9481516	NM_006735	chr7:27140409-27140350	HOXA2
A_23_P127557	3.8137317	4.092436	NM_198439	chr11:105923662-105923603	KBTBD3
A_24_P45367	9.01088	8.426136	NM_020448	chr1:24799313-24799372	NIPAL3
A_33_P3289976	8.491301	8.672915		chr9:067343662-067343603	
A_33_P3273684	3.1036458	3.4089384	NM_001135659	chr2:50149144-50149085	NRXN1
A_33_P3419003	13.925062	13.472548		chr4:124984004-124984063	
A_23_P46412	6.6560206	6.8920918	NM_001130413	chr1:1226313-1226483	SCNN1D
A_33_P3399263	5.162953	5.1866903	NM_001243539	chr10:5995117-5995058	IL15RA
A_33_P3289996	4.895363	4.611724	NM_001080481	chr6:99951701-99951642	USP45
A_24_P118376	10.413816	10.5894575	NM_001102598	chr19:45024733-45024674	CEACAM20
A_33_P3361257	10.91374	10.591174	NM_016391	chr5:175811304-175811245	NOP16
A_33_P3282359	4.745169	4.643806	NR_027995	chr13:19446042-19445983	ANKRD20A9P
A_23_P19369	7.1222467	6.8726306	NM_017640	chr6:25620055-25620114	LRRC16A
A_33_P3354479	14.716869	14.77689		chr10:051532161-051532102	
A_33_P3415648	6.292882	6.415081		chr12:8543467-8543408	LINC00937
A_23_P42649	9.789213	9.8533535	NM_006234	chr7:102116670-102114966	POLR2J
A_23_P250347	5.7913446	5.3630743	NM_001744	chr5:110819929-110819988	CAMK4
A_33_P3353996	5.640476	5.6745505	NM_001145115		PPP1R3G
A_33_P3407700	5.903113	5.9478927	NM_001100389	chr4:165997429-165997370	TMEM192
A_33_P3667484	12.373481	12.362031	NR_024438	chr1:104113812-104113871	ACTG1P4
A_32_P101689	9.223785	9.987476	NM_014888	chr7:120989898-120989839	FAM3C
A_24_P61753	6.866972	6.8698254	NM_015229	chr17:2593491-2593432	CLUH
A_24_P766208	13.666697	13.739934	NM_000967	chr22:39710135-39709699	RPL3
A_33_P3401813	4.0266147	3.269865	AK126599	chr11:65392737-65392678	
A_24_P941912	6.923273	7.478943	NM_138287	chr3:122293039-122293098	DTX3L
A_24_P203765	4.4434347	4.388438	BC000651	chr1:53580455-53574273	SLC1A7
A_33_P3225522	6.1424913	6.5461807	NM_001032731	chr12:113426550-113426609	OAS2
A_24_P184732	4.641884	4.783613	NM_173462	chr14:73711421-73712356	PAPLN
A_33_P3396214	6.85402	7.646867	NM_172229	chr16:3018321-3018380	KREMEN2
A_33_P3410201	4.8737874	4.2883067		chr8:144840382-144840323	
A_33_P3260053	5.9625874	6.1928554	NM_001185095	chr9:133995766-133995825	AIF1L
A_24_P784765	3.8821824	2.8651457	NM_203330	chr11:33724805-33724746	CD59
A_23_P76823	3.618293	3.8878555	NM_199165	chr14:105213294-105213353	ADSSL1
A_23_P30098	4.0975	3.7289357	NM_000670	chr4:100045550-100045491	ADH4
A_33_P3313785	8.618832	8.462435	NM_022757	chr3:123633295-123633236	CCDC14
A_23_P346969	6.785607	6.8486524	NM_006219	chr3:138374340-138374281	PIK3CB
A_33_P3368186	15.835667	15.980293	AY444749	chr7:28662422-28662481	
A_33_P3242833	7.1335535	7.165641	NM_006256	chr1:89270157-89270216	PKN2
A_23_P201551	4.444333	5.3072557	NM_006113	chr1:108113896-108113837	VAV3
A_33_P3261610	8.174527	8.393177	NM_032305	chr1:145456573-145456514	POLR3GL
A_23_P48988	6.5483055	6.41668	NM_003027	chr15:84287094-84287153	SH3GL3
A_23_P80827	9.973661	9.781462	NM_001011537	chr3:197510301-197510360	FYTTD1
A_33_P3214625	8.1732025	7.7580547	NM_003866	chr4:142949267-142949208	INPP4B
A_23_P252106	10.563306	10.520898	NM_003821	chr8:90802345-90802404	RIPK2
A_32_P831181	5.3977327	4.952818	NM_080626	chr12:125497122-125497181	BRI3BP
A_33_P3671506	5.4094343	5.5811143	AK021734	chr5:179340482-179340423	LOC153811

A_32_P78816	11.160891	10.911365	NM_004577	chr7:56078858-56078799	PSPH
A_33_P3310961	3.8020082	3.7262204	NR_038943	chr10:111705396-111705337	ADD3-AS1
A_24_P159837	6.68779	6.638306	XM_005259055	chr19:35174914-35174973	ZNF302
A_24_P210082	7.578952	7.831416	NM_019021	chr11:114270786-114270727	C11orf71
A_33_P3309636	11.608827	11.547043	NM_014267	chr11:16777113-16777172	C11orf58
A_24_P8371	7.6112456	7.7815228	NM_001124758	chr17:4442247-4442306	SPNS2
A_33_P3279109	6.022228	6.079912	BC039601	chr16:3359016-3359075	ZNF75A
A_32_P68942	2.5647292	2.3900566	NR_040113	chr18:14179379-14179438	ANKRD20A5P
A_32_P32254	11.7877	12.212606	NM_001848	chr21:47424836-47424895	COL6A1
A_33_P3346338	4.856745	4.82695	NM_001145088	chr8:124141382-124141441	TBC1D31
A_24_P194962	14.046365	13.987585		chr10:088390851-088390792	
A_24_P306892	4.3558707	4.319015	NM_173593	chr12:661313-661675	B4GALNT3
A_33_P3393341	9.329971	9.349419	NM_022896	chr20:39989162-39989221	LPIN3
A_23_P407115	5.4790106	5.6385036	NM_003559	chr17:36934051-36933992	PIP4K2B
A_33_P3315021	6.838417	7.3520374		chr2:114368431-114368372	RPL23AP7
A_23_P86493	7.0458136	7.1244073	NM_006562	chr10:102986818-102986759	LBX1
A_33_P3290573	6.429083	7.3195343	NM_017633	chr6:82461363-82460186	FAM46A
A_33_P3214343	4.5935965	3.172864		chr3:111445734-111445793	
A_32_P192615	7.5352573	7.5662603	NM_001286074	chrX:70685417-70685476	TAF1
A_33_P3323803	6.009125	5.892348	NM_014644	chr1:144851887-144851828	PDE4DIP
A_23_P139527	3.9548697	4.2168536	NM_002150	chr12:122281665-122277945	HPD
A_23_P20392	8.831393	8.550305	NM_015310	chr8:18385112-18385053	PSD3
A_23_P76969	8.875533	8.611416	NM_015556	chr14:72205922-72205981	SIPA1L1
A_33_P3250323	3.586144	3.990366		chr21:038739171-038739112	
A_23_P302116	6.0729184	6.0470643	NM_177423	chr11:70221056-70221115	PPFIA1
A_24_P351304	7.42642	7.84943	NM_006839	chr2:86373308-86373249	IMMT
A_33_P3380405	8.898809	8.740881	AK123894	chr17:76673586-76673527	CYTH1
A_24_P284353	8.508512	8.638963	NM_032354	chr17:8077404-8077345	TMEM107
A_23_P201279	9.780886	9.896127	NM_006048	chr1:10241075-10241134	UBE4B
A_33_P3371224	10.865489	10.918064	NM_001204831	chr3:43407878-43407819	ANO10
A_33_P3379231	4.1581826	4.0552297		chr17:018633750-018633809	
A_23_P89101	3.6479084	3.828843	NM_144605	chr16:4827777-4827718	SEPT12
A_33_P3366296	2.3221061	2.3900566	NR_026731	chr14:29263854-29263913	C14orf23
A_33_P3308128	3.6768656	3.9843283	AK172772	chr1:115878368-115878309	
A_33_P3372688	3.9546404	4.2875204	NM_001164405	chr17:1174446-1174505	BHLHA9
A_24_P83808	7.3471165	7.8425827		chr8:055435755-055435696	
A_23_P35055	7.5709147	7.840811	NM_014625	chr1:179519810-179519751	NPHS2
A_24_P201153	4.5260878	4.0592923	NM_201629	chr9:71855045-71861646	TJP2
A_33_P3358626	3.3598394	3.1554883	NM_005640	chr18:23971373-23971432	TAF4B
A_23_P379475	9.974408	9.794389	NM_014762	chr1:55315569-55315510	DHCR24
A_33_P3309222	4.5766897	4.5519304		chr6:168081551-168081492	
A_33_P3314500	3.5623446	3.3504	NM_005961	chr11:1016885-1016826	MUC6
A_33_P3212350	2.949856	2.3900566	AK097139	chr7:3082928-3082869	CARD11
A_23_P200493	12.143517	10.969391	NM_002296	chr1:225589341-225589282	LBR
A_33_P3325330	4.9099226	5.0525007		chr6:080770692-080770633	
A_24_P126557	4.44626	4.051344	NM_133452	chr19:10427728-10427669	RAVER1
A_33_P3227077	4.6547594	4.6812544	AK090403	chr15:30814556-30814497	
A_24_P162244	6.016718	5.961754	AB040887	chr15:76672292-76672233	SCAPER
A_33_P3346669	5.5826645	5.665199	NM_016341	chr10:96084735-96084794	PLCE1
A_23_P76622	5.284727	5.3280163	NM_001922	chr13:95095805-95095746	DCT
A_33_P3257232	10.811014	11.122928	NM_006284	chr11:6632132-6632073	TAF10
A_33_P3233953	11.52396	11.20102		chr9:138153022-138153081	
A_33_P3400292	3.8541238	3.5835843		chr12:006862006-006861947	
A_23_P30913	4.047958	3.4704924	NM_033554	chr6:33032907-33032848	HLA-DPA1
A_23_P138194	2.3221061	2.3900566	NM_000433	chr1:183524784-183524725	NCF2
A_23_P431268	8.95778	8.667963	NM_014935	chr1:204188069-204188010	PLEKHA6
A_33_P3347040	7.8302975	7.7610626	NM_001242901	chr19:4685870-4685929	LOC100131094
A_33_P3235701	3.538447	4.405824	XM_005270685	chr1:52929446-52929387	ZCCHC11
A_33_P3369781	6.021859	6.3009615	NM_198920	chr6:83667091-83667032	UBE3D

A_33_P3668839	6.758223	6.6608334	NR_036539	chr11:9481180-9481121	LOC644656
A_33_P3354326	3.7484016	3.6000166		chr2:217186392-217186451	
A_23_P323180	2.3499827	2.3900566	NM_006898	chr2:177037727-177037786	HOXD3
A_32_P221	3.8938715	4.0781546	NM_144602	chr16:49433115-49433174	C16orf78
A_33_P3278501	5.3106036	5.031534		chr13:098128554-098128613	
A_33_P3348569	3.9022124	3.459933	NM_001005284	chr11:56510363-56510304	OR9G4
A_24_P224727	5.495815	5.3143587	NM_004364	chr19:33791412-33791353	CEBPA
A_33_P3329352	5.616771	5.9976482		chr11:013632237-013632178	
A_32_P89352	8.42573	8.443624	NM_080676	chr20:16033184-16033243	MACROD2
A_33_P3282556	6.1515136	5.4861016	NM_024600	chr16:1605184-1605243	TMEM204
A_33_P3217480	9.292695	9.061892	NM_003609	chr16:30004385-30004326	HIRIP3
A_33_P3393360	3.7420728	3.768331	AK124783	chr6:3149458-3149517	LOC100130927
A_23_P42738	8.820303	8.51861	NM_001037163	chr7:6369422-6369363	FAM220A
A_24_P91310	9.137922	9.181051	NM_006503	chr19:40485988-40486047	PSMC4
A_23_P150379	3.9840906	4.055016	NM_144765	chr11:118127915-118127856	MPZL2
A_23_P328323	7.5238204	7.3320637	NM_018211	chr1:65298644-65298703	RAVER2
A_33_P3403708	3.5470848	3.726701		chr10:005036960-005036901	
A_32_P152696	10.558752	10.334401		chr9:022155709-022155768	
A_33_P3881056	6.432891	6.40427	XM_003846604	chr19:7224336-7224280	LOC100996405
A_33_P3360216	4.891752	5.024291	NM_003509	chr6:27776386-27776445	HIST1H2AI
A_33_P3330175	8.515959	8.66456		chr9:66708013-66707954	XLOC_014512
A_23_P434890	10.818659	10.852718	NM_014550	chr22:37886506-37886447	CARD10
A_24_P104538	6.5927296	7.0176992	XM_005257152	chr17:65959543-65959602	BPTF
A_33_P3617190	3.941641	3.9038649	AK124119	chr9:140671107-140671048	LOC651337
A_24_P942321	5.272232	5.3034134	NM_018027	chr10:13686415-13686356	FRMD4A
A_33_P3305173	6.1781034	6.472507	NM_001024455	chrX:71347022-71346963	RGAG4
A_23_P157513	6.180395	6.5792546	NM_005372	chr8:57025995-57025936	MOS
A_33_P3315303	9.231047	9.457976	NM_175068	chr12:53001417-53001358	KRT73
A_24_P329635	9.514394	9.610316	NM_000368	chr9:135766870-135766811	TSC1
A_23_P153583	4.2181735	4.088124	NM_006247	chr19:46890632-46890691	PPP5C
A_23_P254031	6.8603992	6.941675	NM_007344	chr9:135251448-135251389	TTF1
A_24_P48408	6.852448	7.0124526	NM_003799	chr18:13764222-13764281	RNMT
A_24_P364970	7.142825	6.837342	NM_020162	chr17:5346174-5346115	DHX33
A_24_P144346	2.934664	3.332469	Y11328	chr16:33006697-33006756	
A_23_P217009	3.5581872	3.9499357	NM_032596	chr9:34379083-34379024	C9orf24
A_23_P24763	13.877535	14.05889	NM_001017	chr11:17096663-17095974	RPS13
A_33_P3403549	4.494543	5.255596	NM_153261	chr16:50069927-50069986	CNEP1R1
A_33_P3261818	12.304049	12.370544	NM_005917	chr2:63834131-63834190	MDH1
A_33_P3410019	4.0899377	4.2201066		chr18:15197070-15197011	
A_33_P3328637	5.8409853	5.6776047	NM_001173486	chrX:37301211-37301270	PRRG1
A_33_P3402615	8.44478	7.841214	NM_201649	chr1:44462221-44462162	SLC6A9
A_23_P83714	7.845674	7.7348027	NM_173831	chr8:144777433-144777492	ZNF707
A_33_P3231542	8.559543	8.536585	NM_006885	chr16:72816977-72816918	ZFHX3
A_33_P3372074	8.101704	8.897953	NM_001031803	chr17:73571211-73571270	LLGL2
A_33_P3400758	6.845862	7.132299	NM_152791	chr19:2853891-2853950	ZNF555
A_33_P3290602	9.63386	9.905848	NR_102705	chr20:33864829-33864770	MMP24-AS1
A_33_P3327852	4.312263	4.6404276	NM_030808	chr17:8363418-8363477	NDEL1
A_23_P34107	10.444683	10.105376	NM_004699	chrX:153678038-153678244	FAM50A
A_23_P7873	12.218938	11.961438	NM_002388	chr6:52128952-52128893	MCM3
A_23_P118203	4.0231028	3.628381	NM_145252	chr16:2882113-2882172	ZG16B
A_23_P45424	5.3744297	4.8045	NM_012278	chrX:70525129-70525188	ITGB1BP2
A_32_P99100	10.085754	10.044097	NM_002844	chr6:128290037-128289978	PTPRK
A_23_P201035	7.3091335	7.6314287	NM_001005741	chr1:155204686-155204627	GBA
A_24_P152649	8.48667	8.396639	NR_033748	chr19:36913717-36913776	LOC644189
A_32_P81357	7.6157255	7.3490753	NM_016044	chr2:96078237-96078430	FAHD2A
A_23_P32404	7.3646975	7.4393616	NM_002201	chr15:89198784-89198843	ISG20
A_24_P67748	6.8839045	6.7339025		chr6:057841725-057841666	
A_23_P60339	2.3221061	2.3900566	NM_032307	chr9:86554414-86554355	C9orf64
A_23_P166400	5.7636514	6.136686	NM_006477	chr22:29709034-29708975	RASL10A

A_33_P3229017	2.5707793	2.3900566		chr5:137672717-137672776	FAM53C
A_23_P165937	8.974329	9.104214	NM_024918	chr20:35380814-35380755	DSN1
A_23_P52939	4.940711	5.001982	NM_003627	chr11:57252360-57252301	SLC43A1
A_23_P2066	7.917565	8.195724	NM_015957	chr11:34909875-34905028	APIP
A_23_P140050	6.8527513	6.8177214	NM_017569	chr13:37595620-37593511	SUPT20H
A_33_P3215834	3.8304787	3.4812794	NM_020777	chr4:7738826-7738885	SORCS2
A_23_P326893	4.241293	3.4290614	NM_145045	chr19:11531597-11531538	CCDC151
A_23_P211957	11.703935	12.405041	NM_001024847	chr3:30735163-30735222	TGFBR2
A_33_P3406828	4.6311617	4.657349	NR_046439	chr21:009909686-009909627	MAFIP
A_23_P130515	2.5869555	3.175684	NM_001815	chr19:42301831-42312860	CEACAM3
A_23_P88602	6.560549	6.3976564	NM_170677	chr15:37183612-37183553	MEIS2
A_23_P83463	11.093014	11.340458	NM_004261	chr1:87328745-87328686	SEP15
A_33_P3265205	3.2226114	3.7147899		chr17:076968334-076968393	
A_33_P3409154	6.2674503	7.0065827	NM_015482	chr6:3273134-3273075	SLC22A23
A_33_P3287396	3.9437015	3.4108117	AK090626	chr7:228654-228595	LOC100127940
A_24_P203726	13.333627	13.521543		chr1:016155064-016155123	
A_23_P53390	5.8470173	5.468662	NM_002837	chr12:70925829-70918327	PTPRB
A_24_P12539	7.2454896	7.6871543	NM_015483	chr7:32908505-32908446	KBTBD2
A_23_P366394	6.2171454	5.9562774	NM_016653	chr2:174132653-174132712	ZAK
A_33_P3671647	4.3654523	4.450133		chr4:76287610-76287669	LOC441025
A_32_P179676	8.422638	8.653767	NM_016272	chr22:41829804-41829745	TOB2
A_23_P44363	7.7217007	7.698199	NM_020753	chr17:73496548-73496489	CASKIN2
A_23_P78410	9.835497	9.898943	NM_014177	chr18:71825704-71825763	TIMM21
A_23_P142255	5.5471926	5.442795	NM_020209	chr19:4290646-4290705	SHD
A_33_P3311775	4.971315	4.8385677	NM_001013258	chr7:99079885-99079944	ZNF789
A_23_P314115	8.3718815	8.907997	NM_005180	chr10:22619577-22619636	BMI1
A_24_P112447	5.2874045	6.121338	NM_020354	chr10:101465029-101465088	ENTPD7
A_33_P3306616	13.047541	13.11352	AK094078	chr5:43481521-43481462	
A_33_P3503408	6.8102784	6.7061543	BC044606	chr7:5565095-5565154	LOC221946
A_33_P3255274	7.4222326	7.4223742	NM_017623	chr2:97501062-97501121	CNNM3
A_33_P3343828	8.1038885	7.4005136	AK126677	chr16:2611677-2611618	
A_23_P211814	8.09897	8.1038885	NM_002375	chr3:47893446-47893387	MAP4
A_24_P165423	2.377699	2.3900566	NM_052960	chr1:10068313-10075879	RBP7
A_24_P108311	9.624451	9.078637	NM_015277	chr18:56065317-56065376	NEDD4L
A_23_P50418	7.037764	7.3578506	NM_153358	chr19:12740183-12740240	ZNF791
A_33_P3219572	3.5109844	2.3900566		chrX:118156804-118156863	LONRF3
A_33_P3360097	13.160629	13.234316	NM_000485	chr16:88875936-88875877	APRT
A_33_P3354683	10.944987	11.244692	NM_033198	chr17:26880467-26880408	PIGS
A_32_P58937	6.7104645	7.23934	NM_017643	chr17:49255399-49255340	MBTD1
A_23_P315589	5.663086	5.3779573	NM_015264	chr22:45592618-45592559	KIAA0930
A_32_P213965	4.3224087	4.1883764	NR_038309	chr11:42262643-42261363	LOC100507205
A_24_P7965	3.683254	4.2379937	NM_206594	chr1:216850690-216850631	ESRRG
A_23_P74344	7.353409	7.4900613	NM_053053	chr1:166826396-166826337	TADA1
A_24_P276531	5.671688	6.015812	NM_024700	chr1:38002882-38002823	SNIP1
A_23_P145068	8.739958	8.633803	NM_012123	chr6:74210376-74210435	MTO1
A_23_P365844	9.946117	9.529508	NM_153232	chr19:40029628-40029569	EID2
A_33_P3358312	7.947496	7.497777	NM_016023	chr8:92099128-92099187	OTUD6B
A_33_P3359869	8.877682	8.579056		chr20:062512265-062512324	
A_23_P41194	5.6688375	5.1877055	NM_153353	chr3:169511485-169511426	LRRC34
A_33_P3243554	5.7059064	5.5109024	NM_145802	chrX:118783960-118783901	SEPT6
A_33_P3309849	7.9831085	7.688364		chr18:075239380-075239439	
A_33_P3799936	8.699329	8.771379	NM_018125	chr1:18024301-18024360	ARHGEF10L
A_33_P3388466	4.6038575	4.4114265	NM_007048	chr6:26409886-26409945	BTN3A1
A_24_P245815	4.4333725	4.2608123	NM_020437	chr22:26840444-26840503	ASPHD2
A_23_P18641	6.6977286	7.0825806	NM_031953	chr4:186283106-186283165	SNX25
A_23_P90273	4.665757	4.556199	NM_022467	chr19:34264292-34264351	CHST8
A_33_P3385988	5.945197	6.0939283		chr22:50453671-50453612	
A_33_P3363153	8.9718895	8.64259	NM_025219	chr20:62565334-62565393	DNAJC5
A_23_P90601	8.7988825	8.458441	NM_182915	chr2:120022998-120023057	STEAP3

A_33_P3246108	7.273315	6.9291587	NM_001130415	chr12:12940330-12940389	APOLD1
A_33_P3312504	6.298681	6.372566	NM_012455	chr2:113958848-113958907	PSD4
A_23_P216038	7.2946033	7.2476115	XM_005250941	chr8:133837383-133837442	PHF20L1
A_33_P3330074	5.0347986	5.2223177		chr11:3647657-3647716	XLOC_014512
A_24_P379750	5.8981957	6.088118	NM_002357	chr2:70169884-70169943	MXD1
A_33_P3346067	4.222665	3.922895		chr21:015964309-015964250	
A_23_P167017	3.5632875	3.2875266	NM_022135	chr3:119361062-119361003	POPDC2
A_23_P930	5.8096724	5.8213177	NM_001077628	chr1:150240127-150239831	APH1A
A_24_P372048	7.1168222	6.581442	NM_006555	chr7:44253196-44253255	YKT6
A_33_P3298425	8.300675	8.324828	NM_006769	chr1:87805825-87805884	LMO4
A_24_P64407	4.377486	4.841716	NM_005519	chr10:124909135-124909194	HMX2
A_24_P348083	6.3330007	6.0855923	BC093850	chr18:43914943-43915002	
A_33_P3342160	7.530223	7.3949003	NM_018036	chr14:96747798-96747739	ATG2B
A_33_P3367062	4.683971	5.0973554	NM_017673	chr1:185260080-185260139	SWT1
A_23_P8834	6.6957035	6.714589	NM_001979	chr8:27402115-27402174	EPHX2
A_24_P329353	4.0638804	4.462675	NM_032831	chr7:102088213-102088272	ORAI2
A_33_P3546363	7.7269444	7.8477125	BQ950045		LINC01071
A_33_P3229953	11.7054615	11.597534	NM_001958	chr20:62119501-62119442	EEF1A2
A_33_P3245248	7.6073065	7.430369	NR_001566	chr3:169482695-169482636	TERC
A_23_P145463	6.725454	6.854923	NM_015948	chr6:8421052-8420993	SLC35B3
A_33_P3396389	4.599683	4.2760415	NM_000877	chr2:102792046-102792105	IL1R1
A_23_P160354	4.881827	4.993625	NM_181690	chr1:243716200-243716141	AKT3
A_23_P18824	9.750144	9.951485	NM_006999	chr5:6756843-6756902	PAPD7
A_33_P3297285	3.4378512	3.5227022	AK124758	chr22:38386018-38386077	LOC100131667
A_33_P3282978	3.714491	4.129409		chr8:104195618-104195677	
A_33_P3231447	5.440895	5.743804	NM_000210	chr2:173369203-173369262	ITGA6
A_23_P32328	8.196723	8.179366	NM_020408	chr6:5109509-5109450	LYRM4
A_24_P404840	3.8870652	3.9086227	NM_000166	chrX:70444287-70444346	GJB1
A_33_P3246258	6.08317	5.8277535	AK129520	chr21:43314197-43314138	C2CD2
A_33_P3549874	6.0587373	6.238203	XR_243167	chr15:62488804-62488745	
A_24_P161036	10.2289505	10.581555	NM_001037161	chr14:74010384-74010443	ACOT1
A_23_P98345	6.1164565	6.197381	NM_014758	chr11:130746299-130746240	SNX19
A_24_P336551	9.506793	10.198949	NM_199173	chr1:156213055-156213114	BGLAP
A_23_P366216	11.149814	11.158665	NM_003524	chr6:26252222-26252281	HIST1H2BH
A_23_P359636	7.09801	6.8230505	NM_144695	chr1:222904837-222906007	BROX
A_24_P347624	7.713785	7.8971443	NM_022804	chr15:25207266-25207325	SNURF
A_32_P213661	7.6290717	7.516669	NM_144974	chr13:44433966-44433907	CCDC122
A_33_P3347869	4.456169	4.613765	NM_000064	chr19:6686195-6686136	C3
A_23_P47665	2.3221061	2.3900566	NM_005330	chr11:5291180-5291121	HBE1
A_24_P472455	8.366184	8.303098	NM_001663	chr14:50363171-50363230	ARF6
A_24_P97001	9.254446	8.84668	NM_032864	chr1:52883766-52883825	PRPF38A
A_24_P91852	9.116583	9.108001	NM_006520	chrX:37698509-37698450	DYNLT3
A_24_P67142	6.439843	6.5315247	NM_032195	chr21:34931542-34931601	SON
A_33_P3287039	8.794614	8.540859	NM_013342	chr19:54610451-54610392	TFPT
A_24_P135391	9.424498	10.449107		chr8:082720524-082720585	
A_33_P3218797	9.498814	9.89963		chr20:62152934-62152993	PPDPF
A_33_P3570208	5.944029	5.679315	NR_036497	chr3:187869056-187868997	LPP-AS2
A_24_P127312	12.410598	12.324432		chr8:029490041-029490100	
A_33_P3398401	7.9278045	8.111082	NM_016302	chr3:3191790-3191731	CRBN
A_33_P3270192	6.2968583	6.1386127	AW834780	chr2:232996514-232996573	DIS3L2
A_33_P3354061	5.0795765	4.9852476	XM_005265842	chr5:175964554-175964495	RNF44
A_32_P25050	9.4324	9.902269	NM_172037	chr8:74236460-74236519	RDH10
A_33_P3245290	3.0576987	2.3900566		chr9:67270274-67270215	AQP7P1
A_24_P93703	5.0252223	4.805685	NR_036476	chr12:56229824-56229883	TMEM198B
A_33_P3226212	3.060749	2.3900566	NM_001270408	chr21:27078338-27078397	JAM2
A_33_P3354021	7.433795	6.8784175	NR_003572	chr8:158406-158347	RPL23AP53
A_33_P3332865	6.9898434	6.9653244	NM_024663	chr20:57290323-57290382	NPEPL1
A_33_P3371325	4.3715086	3.531308		chrX:037351894-037351953	
A_23_P64990	5.7211494	5.885665	NM_134424	chr12:1022227-1022168	RAD52

A_23_P90895	5.304996	5.569693	NM_152994	chr2:48692699-48692758	PPP1R21
A_33_P3229552	6.2762055	6.094283	NM_020862	chr19:39797577-39797518	LRFN1
A_23_P88873	4.2746973	4.1413417	NM_022041	chr16:81413400-81413459	GAN
A_33_P3234643	5.5925655	5.9466486	NM_017905	chr13:114164583-114164642	TMCO3
A_32_P11230	8.031742	7.9259453	NR_024497	chr10:38741016-38741075	LINC00999
A_23_P34115	9.19851	9.255523	NM_004135	chrX:153052284-153051874	IDH3G
A_23_P70991	9.362156	9.154325	NM_006303	chr7:6063038-6063097	AIMP2
A_33_P3405334	9.163396	8.930827	NM_000405	chr5:150648748-150648807	GM2A
A_33_P3331853	4.0000796	4.0480585	NM_001191057	chr7:31855668-31855609	PDE1C
A_24_P331128	4.001942	4.291148	NM_002068	chr19:3157749-3157808	GNA15
A_33_P3311795	3.5693467	3.2469594	XM_005267001	chr6:135516143-135516202	MYB
A_23_P132405	10.323662	10.252058	NM_014049	chr3:128631840-128631899	ACAD9
A_23_P143643	4.6212325	4.599683	NM_032204	chr22:30188523-30188464	ASCC2
A_33_P3390521	6.082225	6.1564054	NM_023079	chr17:47004420-47004479	UBE2Z
A_32_P204381	6.469849	6.1589174	NM_020313	chr16:57463000-57462941	CIAPIN1
A_24_P79808	5.7000585	6.3931236	NM_020524	chr1:154917198-154917139	PBXIP1
A_24_P88079	6.969096	7.1392946	NM_005961	chr11:1015874-1015815	MUC6
A_23_P68234	4.428004	4.592488	NM_006794	chr2:54080631-54080572	GPR75
A_33_P3394040	7.715835	8.019106	NM_022374	chr2:38523160-38523101	ATL2
A_23_P100326	10.807848	10.502427	NM_001039476	chr16:135898-135839	NPRL3
A_23_P329016	5.5895195	5.4379554	NM_138778	chr9:140459375-140459030	DPH7
A_33_P3212930	3.939113	3.7545524		chr10:091598431-091598372	
A_23_P154539	8.615044	8.657386	NM_012469	chr20:62663273-62663332	PRPF6
A_33_P3370511	6.291459	6.27132	AK128128	chr16:33347330-33347271	
A_23_P80342	5.41534	5.1033015	NM_006116	chr22:39827322-39827381	TAB1
A_23_P358917	4.0511117	4.0716233	NM_000765	chr7:99303096-99303037	CYP3A7
A_23_P205818	4.8636184	4.9065194	NM_001130858	chr15:43826087-43826028	PPIP5K1
A_33_P3270485	12.584641	12.356836	NM_014292	chr22:39260344-39260285	CBX6
A_23_P154688	6.3168545	6.4170046	NM_032034	chr20:3208221-3208162	SLC4A11
A_33_P3257312	9.73489	10.016502	NM_015104	chr11:64662107-64662048	ATG2A
A_24_P120251	10.804405	11.297329	NM_138786	chr3:149040112-149040053	TM4SF18
A_24_P118196	4.7313056	5.062855	NM_001080393	chr3:73016853-73024169	GXYLT2
A_23_P13885	5.8120737	5.520207	NM_001007026	chr12:7051255-7051314	ATN1
A_23_P81926	4.540754	4.6018667	NM_014069	chr6:31105635-31105576	PSORS1C2
A_23_P31376	2.6720374	3.4132137	NM_018334	chr7:110764771-110764830	LRRN3
A_23_P375524	9.88221	10.227076	NM_178352	chr1:152770331-152770390	LCE1D
A_23_P328766	5.447977	5.243499	NM_145287	chr18:14105476-14105417	ZNF519
A_23_P119295	9.071459	8.767604	NM_016581	chr19:11616951-11616892	ECSIT
A_33_P3297468	6.8042493	6.8583007	NM_001177317	chr9:140129070-140129129	SLC34A3
A_33_P3409099	4.63756	4.5616736	NM_000332	chr6:16302089-16302030	ATXN1
A_23_P436526	3.7982254	4.267158	NM_178526	chr19:19223038-19223097	SLC25A42
A_23_P106906	4.6340876	4.731693	NM_002705	chr16:4933127-4933068	PPL
A_33_P3422113	9.222967	9.774781	NR_024063	chr6:28063341-28063400	ZSCAN12P1
A_23_P69058	10.064613	9.866249	NM_000249	chr3:37092163-37092222	MLH1
A_23_P62081	5.0299563	4.121132	NM_003020	chr15:32989106-32989165	SCG5
A_23_P374844	4.0202365	2.3900566	NM_015973	chr11:68458533-68458592	GAL
A_23_P7099	8.479695	8.628057	NM_000027	chr4:178354452-178354393	AGA
A_23_P433785	3.5484123	3.726499	NM_002561	chr17:3577019-3576960	P2RX5
A_23_P361569	8.557839	9.199478	NM_025181	chr2:114475371-114472755	SLC35F5
A_23_P137586	7.760142	7.6911316	NM_006582	chr1:29041171-29041230	GMEB1
A_33_P3304097	6.1751175	6.562399		chr7:148344398-148344339	
A_23_P20225	8.208122	8.536835	NM_015713	chr8:103217234-103217175	RRM2B
A_23_P45864	4.8045	5.132951	NM_003285	chr1:175304874-175299340	TNR
A_24_P333857	6.154572	5.6504116	NM_032291	chr1:67209705-67209764	SGIP1
A_33_P3662553	10.652139	11.255683	NM_006826	chr2:9727565-9725438	YWHAQ
A_23_P59192	12.405041	12.218938	NM_003093	chr6:34741363-34741422	SNRPC
A_24_P65941	4.6016345	4.9524717	NR_026812	chr21:36410835-36410776	RUNX1-IT1
A_24_P241792	6.4989552	6.918786	NM_198893	chr19:53569960-53569901	ZNF160
A_23_P138058	9.879256	9.599248	NM_015658	chr1:879707-879648	NOC2L

A_24_P15658	6.2121286	5.8383026	NM_198273	chr5:89812396-89812337	LYSMD3
A_24_P943997	4.8085327	5.5840483	NM_178815	chr10:18966467-18966526	ARL5B
A_33_P3375665	8.861006	9.050679	NM_001455	chr6:109005888-109005947	FOXO3
A_33_P3256685	8.319006	7.872352	NM_003594	chr1:117645022-117645081	TTF2
A_33_P3771067	3.601262	3.0971663	XR_247724	chr11:112233141-112233200	LOC283140
A_23_P392126	5.018555	5.1689434	NM_001076680	chr17:26205487-26205428	LYRM9
A_33_P3264926	4.980129	4.9114475	NM_015589	chr14:55251276-55251335	SAMD4A
A_23_P398372	9.937018	10.210121	NM_001256526	chr9:139006855-139006796	C9orf69
A_23_P933	8.200364	8.149713	NM_015485	chr1:95712438-95712497	RWDD3
A_24_P84873	10.731542	10.742922		chr5:055571506-055571447	
A_23_P397391	8.683501	8.481113	NM_005306	chr19:35941189-35941248	FFAR2
A_33_P3382147	5.888351	5.890881	AY082592	chr13:33712626-33712567	STARD13
A_33_P3321577	10.184747	9.901852	NM_032408	chr7:72855977-72855918	BAZ1B
A_32_P149536	12.877233	12.898813	NM_006937	chr17:73163974-73163915	SUMO2
A_23_P201790	7.1102037	7.0230346	NM_002481	chr1:202557518-202557577	PPP1R12B
A_33_P3351559	8.215807	7.788958	NM_018638	chr12:22843337-22843396	ETNK1
A_33_P3484775	3.8407798	3.6094966			
A_23_P213102	9.795473	9.818236	NM_016081	chr4:169848826-169848885	PALLD
A_24_P42389	5.2925677	5.7810907	NM_207320	chrX:69283505-69283564	OTUD6A
A_24_P297539	12.486422	12.672419	NM_181801	chr20:44445525-44445584	UBE2C
A_33_P3343485	7.3430705	7.4158463	AY358103	chr7:75362837-75362778	HIP1
A_33_P3315074	7.205462	7.210372		chr22:035834156-035834215	
A_33_P3239620	4.150577	4.2070856	NM_006652	chr20:44141160-44141101	SPINT3
A_24_P238333	9.451744	9.267673	NM_014886	chr15:059693919-059693860	NSA2
A_33_P3313830	5.552494	5.7398787	NR_015360	chr5:127357303-127357244	FLJ33630
A_33_P3709317	4.851143	4.6594286	NR_002964	chr14:103804201-103804260	SNORA28
A_24_P323635	13.024225	12.989869		chr8:034180714-034180655	
A_23_P170491	7.815967	7.4454494	NM_005879	chr3:49866144-49866085	TRAIP
A_23_P62932	12.008162	11.971525	NM_001677	chr1:169101877-169101936	ATP1B1
A_23_P151970	5.652053	5.93297	NM_015322	chr15:68583545-68583604	FEM1B
A_33_P3384835	4.920356	4.522135	XR_246564	chr5:114955830-114955889	
A_23_P65930	8.587215	8.739958	NM_001077268	chr15:41106618-41106677	ZFYVE19
A_32_P232192	6.1816583	5.7694173	NM_001257281	chr2:233208616-233208675	DIS3L2
A_23_P77714	7.5112753	7.7663155	NM_024793	chr16:3586678-3586737	CLUAP1
A_23_P329261	6.1258297	6.5092087	NM_000891	chr17:68176016-68176075	KCNJ2
A_33_P3209741	5.6016874	5.14113	NM_031303	chr18:44603772-44603831	KATNAL2
A_33_P3229873	6.4279485	6.4931545	NM_001143757	chr10:134194951-134195010	LRRC27
A_23_P318904	8.976288	8.336765	NM_019605	chr1:210416034-210416093	SERTAD4
A_23_P170774	8.092535	7.973121	NM_198256	chr2:11585583-11585524	E2F6
A_24_P928901	7.984046	7.905998	NM_001859	chr9:116026107-116026166	SLC31A1
A_23_P92441	11.032312	10.858258	NM_002358	chr4:120981405-120981346	MAD2L1
A_33_P3408752	3.538587	4.046614	XM_002342102	chr1:41848238-41848297	FOXO6
A_23_P147450	10.293218	10.497971	NM_016630	chr15:65255808-65255749	SPG21
A_33_P3210702	11.79233	11.848478		chr5:108120723-108120782	
A_23_P54000	9.11476	9.031595	NM_021249	chr14:35031706-35031647	SNX6
A_23_P153941	3.564893	3.309821	NM_024766	chr2:44993637-44993696	CAMKMT
A_24_P135579	9.921489	9.729539		chr2:038044605-038044664	
A_33_P3227788	7.3280053	7.4313664	NM_148977	chr10:91342846-91342787	PANK1
A_23_P314755	2.3221061	2.3900566	NM_003155	chr8:23700177-23700118	STC1
A_33_P3226167	14.642805	14.417201	NM_022833	chr9:130267699-130267640	FAM129B
A_33_P3356031	5.412839	5.467285		chr1:183485149-183485208	SMG7
A_33_P3323959	6.438869	8.187849	NM_005045	chr7:103113325-103113266	RELN
A_33_P3464900	6.1596065	6.1473427	XR_241567	chr3:133148190-133148131	
A_23_P100392	8.765736	8.577986	NM_007108	chr16:2821589-2821530	TCEB2
A_23_P58953	8.481323	8.743888	NM_000904	chr6:3010361-3010420	NQO2
A_23_P389919	9.276172	9.121038	NM_133330	chr4:1983546-1983605	WHSC1
A_33_P3751230	4.6536636	4.5901003		chr1:1545701-1545760	LOC284628
A_33_P3264780	4.184057	4.888313	NM_001260	chr13:26927961-26928017	CDK8
A_23_P20970	9.992921	9.990233	NM_004707	chr5:115167432-115167373	ATG12

A_24_P358619	6.7842183	7.1699014	NM_001080409	chr19:22940739-22940680	ZNF99
A_23_P214411	7.9531574	8.259128	NM_006708	chr6:38644622-38644563	GLO1
A_23_P31654	14.833921	14.825712	NM_000973	chr8:146015752-146015301	RPL8
A_33_P3380263	6.415782	5.366043	NM_138384	chr10:135234115-135234174	MTG1
A_33_P3401322	9.192389	9.075807	NM_030980	chr1:156692939-156692880	ISG20L2
A_33_P3334548	10.009428	10.322451		chr2:201686907-201686966	BZW1
A_33_P3270317	7.538491	7.8052588	NM_003055	chr10:50820656-50820715	SLC18A3
A_23_P16078	9.23749	9.423289	NM_002573	chr19:42804213-42804154	PAFAH1B3
A_32_P180210	5.8593483	6.39133	NM_019054	chr10:88912251-88912310	FAM35A
A_33_P3321432	2.3221061	2.3900566	NM_016613	chr4:159046215-159046156	FAM198B
A_33_P3266674	4.6188765	5.077089	NM_025224	chr20:62375999-62375940	ZBTB46
A_33_P3415500	8.388139	8.5666275	XR_241714	chr5:14510009-14510068	TRIO
A_24_P337746	8.640721	7.6670737	NM_014504	chr7:66276369-66276428	RABGEF1
A_33_P3259865	5.1528683	4.79404	NR_033186	chr1:178517965-178518024	C1orf220
A_33_P3266419	5.204869	4.9630837	NM_016433	chr12:110290419-110290361	GLTP
A_24_P272515	7.144817	6.1333632		chr2:152042739-152042798	
A_32_P4403	8.412592	8.137252	NM_014117	chr16:9213352-9213411	C16orf72
A_33_P3410589	6.482115	7.0118403	NM_153690	chr3:194409703-194409762	FAM43A
A_23_P109895	6.4612007	6.6951714	NM_001040454	chr3:48663765-48663706	SLC26A6
A_33_P3245228	3.4891388	3.9915545	NM_130852	chr20:31831052-31831111	BPIFA1
A_32_P187663	5.3315516	5.536691	NM_001042416	chr8:195612-195671	ZNF596
A_24_P944640	6.254411	6.5178843	NM_020909	chr2:120936186-120936245	EPB41L5
A_23_P361014	6.1864433	6.122833	NM_020856	chr19:31765938-31765879	TSHZ3
A_23_P44942	4.6609607	5.0203404	NM_025004	chr11:124910525-124910584	CCDC15
A_33_P3413216	7.4178104	7.4499335	NM_001025237	chr11:866798-866857	TSPAN4
A_33_P3313846	5.2424836	5.989883		chr22:20134188-20134247	ZDHHC8
A_23_P122915	10.979827	11.1771145	NM_015379	chr7:97920709-97920768	BRI3
A_33_P3298440	4.7415442	4.0105743	AK055581	chr2:65452756-65452697	LOC729324
A_33_P3274428	14.893461	14.982724		chr19:13900953-13900894	
A_23_P142075	5.88481	5.7333574	NM_001611	chr19:11685584-11685525	ACP5
A_33_P3329023	4.3778048	4.6782546	NM_001252273	chr1:93312831-93312772	FAM69A
A_23_P120933	14.209134	13.420244	NM_001675	chr22:39918577-39918636	ATF4
A_24_P583040	6.5796337	6.5043216	NM_001085430	chr17:54910335-54910276	C17orf67
A_23_P253395	4.316314	4.7706833	NM_006377	chr9:35404491-35404550	UNC13B
A_33_P3365747	6.7250667	6.9264793		chr7:39833597-39833656	LINC00265
A_32_P76156	6.7318225	5.573608	NM_152682	chr4:184561651-184561592	RWDD4
A_23_P2884	7.732072	7.7900705	NM_004569	chr14:68056700-68056641	PIGH
A_23_P124300	5.696103	5.7945023	NM_017429	chr16:81323976-81324035	BCMO1
A_24_P653603	13.088327	12.881577	NM_001086521	chr17:79215020-79215079	C17orf89
A_32_P148345	11.529684	11.584587	NM_001002857	chr15:60639647-60639588	ANXA2
A_23_P23983	8.292273	7.950018	NM_024948	chr10:15820939-15820880	FAM188A
A_33_P3213463	11.932268	11.91863	NM_002496	chr11:67803956-67804015	NDUFS8
A_23_P255057	6.458405	6.8806157	NM_017945	chr3:112301841-112301900	SLC35A5
A_23_P200507	11.968481	12.096716	NM_014184	chr1:224563539-224563598	CNIH4
A_33_P3362869	8.83657	8.548798	NM_001190233	chr3:180705977-180705837	DNAJC19
A_23_P428382	7.5372605	7.8588552	NM_203437	chr2:64812605-64812664	AFTPH
A_24_P345993	5.497326	5.0450573	NM_001746	chr5:179158058-179158117	CANX
A_33_P3293668	8.665142	8.677442	NM_017925	chr9:19374048-19374107	DENND4C
A_23_P166135	8.4710455	8.736635	NM_012255	chr20:21337284-21338413	XRN2
A_33_P3213419	7.05737	7.052526	AK054946	chr12:113528430-113528489	LOC100129447
A_32_P92399	9.93647	10.001595	NM_032382	chr16:69362884-69362825	COG8
A_24_P126139	5.691786	5.712127	NM_016370	chrX:103078011-103077952	RAB9B
A_23_P127406	3.738612	2.3900566	NM_018039	chr11:94732352-94732411	KDM4D
A_33_P3402600	7.918112	7.732954		chr4:102269985-102270044	
A_24_P362540	9.128426	9.436399	NM_003887	chr2:9545306-9545365	ASAP2
A_23_P140907	6.6066446	6.904564	NM_021259	chr16:422210-422151	TMEM8A
A_23_P134167	6.7226973	6.462702	NM_020381	chr6:107474153-107474094	PDSS2
A_33_P3391375	10.044422	9.6702585	NM_198511	chrX:37536552-37536611	LANCL3
A_33_P3404221	4.7333784	4.9184914		chr1:068944643-068944584	

A_23_P77833	5.1647234	5.3464503	NM_024419	chr17:76399905-76399964	PGS1
A_33_P3324810	3.8299763	4.587425	AK127936	chr14:22471815-22471874	
A_23_P128808	3.167152	4.023551	NM_013345	chr14:105515958-105515899	GPR132
A_23_P134477	7.7093277	7.8280287	NM_032350	chr7:1036981-1036922	C7orf50
A_23_P314642	8.064218	8.182639	NM_017666	chrX:129337091-129337032	ZNF280C
A_24_P912439	5.807313	5.8831778	AB075848	chr9:117121825-117121766	AKNA
A_23_P217028	6.900847	6.6991687	NM_001008563	chr9:132643770-132643829	USP20
A_23_P404678	4.489374	4.445162	NM_004283	chr19:11435349-11435290	RAB3D
A_33_P3410093	5.759685	6.4975376	NM_001256644	chr12:96415985-96415926	LTA4H
A_24_P84021	5.76289	5.451829	XM_005265789	chr4:54442003-54442062	LOC101928879
A_23_P259098	4.7736483	5.298943	NM_025231	chr6:28097263-28097322	ZSCAN16
A_24_P170067	8.374796	8.573952		chr10:090378539-090378480	
A_33_P3209895	9.374902	9.326489	NM_001130997	chr13:019956674-019956733	FAM58A
A_33_P3324409	4.880563	5.218017		chr11:49007510-49007451	XLOC_014512
A_24_P941166	5.435765	5.5842094	NM_001001661	chr7:148800153-148800094	ZNF425
A_23_P390032	5.8935404	5.899674	NM_153226	chr10:95661583-95661642	SLC35G1
A_32_P355396	5.4748945	4.9576054	NM_014844	chr14:102968719-102968778	TECPR2
A_23_P255523	6.418319	6.511275	NM_017621	chr7:102097656-102097597	ALKBH4
A_23_P161522	6.5401273	6.7731886	NM_025124	chr11:67232297-67232132	TMEM134
A_33_P3287584	3.962978	3.5212142	NM_133471	chr6:30652961-30652902	PPP1R18
A_24_P750305	5.9930053	6.08494	NR_047519	chr1:789523-789582	LINC01128
A_23_P43800	11.002604	10.526705	NM_015201	chr8:145486576-145486433	BOP1
A_24_P44341	5.017564	4.8559103	NM_020121	chr13:96665632-96665573	UGGT2
A_24_P14531	10.739543	10.942369	NM_014742	chr20:30754894-30754953	TM9SF4
A_33_P3245977	4.8020024	4.7244725	NM_133456	chr5:132159191-132159132	SHROOM1
A_33_P3413188	5.8013544	5.6435766	NR_024493	chrX:134232498-134232439	LINC00087
A_24_P38815	5.7531343	6.1994843	NM_000391	chr11:6634452-6634393	TPP1
A_33_P3322499	4.4223013	3.9857194	NR_028594	chr19:11797275-11797334	ZNF833P
A_24_P114249	5.9296026	5.475253	NM_004482	chr2:166604475-166604416	GALNT3
A_23_P146885	7.1423783	7.1812797	NM_018949	chr17:80333237-80333296	UTS2R
A_33_P3243429	8.216266	8.35242	NM_206997	chr11:67218961-67218902	GPR152
A_33_P3396379	5.7085466	5.700532		chr17:080277362-080277303	
A_33_P3243449	3.5110626	2.99364	NM_001252	chr19:6585926-6585867	CD70
A_23_P204144	4.8815885	5.3780637	NM_002283	chr12:52754483-52754424	KRT85
A_33_P3286973	7.1421585	7.1653967	AK122959	chr1:1270342-1270401	TAS1R3
A_23_P80156	6.7897096	6.9421725	NM_206962	chr21:48078756-48078815	PRMT2
A_33_P3398932	3.4847057	3.6927195		chr18:47797205-47797146	MBD1
A_23_P36658	11.336078	11.460392	NM_145791	chr12:16516907-16516966	MGST1
A_23_P129458	2.3221061	3.1948788	NM_145168	chr16:82031603-82031544	SDR42E1
A_33_P3280666	6.3457665	6.468508	NM_138415	chr22:45279025-45278966	PHF21B
A_32_P219368	7.4067907	7.299123	NM_152858	chr6:160169678-160169737	WTAP
A_33_P3877739	6.137329	6.4298797	AI821758	chr17:17579625-17579684	SMCR2
A_33_P3688869	2.5797272	3.6567376	NR_030728	chr2:132056758-132056817	LOC440910
A_33_P3236558	5.7485943	6.1891155	NM_019118	chr1:32686794-32686735	TMEM234
A_23_P343366	3.119126	3.9354324	NM_152367	chr1:116676755-116676814	MAB21L3
A_32_P40377	9.264494	9.247124	NR_034031	chrX:3735588-3735576	LOC389906
A_32_P198325	6.206378	6.557312	NM_207644	chr22:24981830-24981771	FAM211B
A_33_P3218768	6.7020383	6.786807	NM_080599	chr10:12041965-12041906	UPF2
A_24_P155502	5.876253	6.0806017	NM_005538	chr12:57844125-57844184	INHBC
A_23_P322	8.362057	8.276808	NM_182690	chr1:155041654-155041713	EFNA4
A_33_P3415037	12.627173	12.574505	NM_001184783	chr10:76990753-76990812	VDAC2
A_33_P3413701	6.82589	7.0095544	NM_001040458	chr5:96110275-96110216	ERAP1
A_33_P3403773	6.004886	5.7914987	NM_152484	chr19:37902138-37902079	ZNF569
A_24_P140204	7.218239	7.2892847	NM_017771	chr3:58389602-58394674	PXK
A_23_P170534	3.8301501	3.6618884	NM_004479	chr9:139925373-139925314	FUT7
A_33_P3419806	13.30039	13.319847	NM_001032	chr14:50053004-50052710	RPS29
A_33_P3781228	5.1581755	4.7438927	XR_244191	chr20:48801277-48801218	LOC101927559
A_23_P164691	7.383559	7.443511	NM_002162	chr19:10444713-10444654	ICAM3
A_24_P396375	4.4937706	3.921266	NM_001113347	chr1:21544718-21544659	ECE1

A_23_P211285	10.529908	10.675057	NM_021075	chr21:44328997-44329056	NDUFV3
A_23_P115246	5.3602123	5.3494167	NM_003665	chr1:27695885-27695826	FCN3
A_33_P3239278	4.4445753	4.407875	NM_001004705	chr11:59245535-59245594	OR4D10
A_24_P183292	6.898673	7.28886	XM_005268376	chr5:148875307-148875248	CSNK1A1
A_33_P3327375	3.702842	3.6756327		chr10:124585749-124585808	LOC729800
A_24_P245322	6.441043	6.9359264	NM_212472	chr17:66523996-66525024	PRKAR1A
A_23_P80974	6.199301	6.1004705	NM_005651	chr4:156838588-156839321	TDO2
A_23_P99661	10.250338	10.039202	NM_018071	chr14:21557611-21557670	ARHGEF40
A_24_P347488	12.594133	12.512245	NM_002802	chr14:90738829-90738888	PSMC1
A_33_P3330384	3.7520444	3.6553755	NM_016526	chr11:205529-205470	BET1L
A_33_P3871347	6.024378	6.2001753	NM_001080437	chr2:242032388-242032447	SNED1
A_33_P3368445	8.784786	9.353966		chr1:155121750-155121809	
A_33_P3390591	2.3221061	2.3900566	XR_109813	chr4:188454177-188454118	LOC100506272
A_23_P151497	7.5454154	6.855465	NM_152307	chr14:104002073-104002132	TRMT61A
A_24_P160440	10.84533	10.838594	NM_181462	chr1:228294525-228294466	MRPL55
A_24_P321634	6.7138057	6.9411535	NM_001039650	chr13:20412841-20411904	ZMYM5
A_32_P158302	8.577486	8.477103	NR_024412	chr7:112757587-112757528	LINC00998
A_32_P114896	11.013224	11.088631	NM_006601	chr12:57057118-57057110	PTGES3
A_23_P424712	7.4114285	7.387869	NM_032779	chr2:74701583-74701524	CCDC142
A_23_P168306	9.302041	8.855524	NM_003931	chr6:110421185-110421126	WASF1
A_23_P388331	2.3221061	2.3900566	NM_001187	chr21:11058043-11057984	BAGE
A_23_P349310	8.342077	8.466429	NM_014494	chr16:24835610-24835669	TNRC6A
A_23_P68007	13.38592	12.850865	NM_001679	chr3:141644942-141645001	ATP1B3
A_33_P3308534	6.463605	6.528448	NM_080597	chr18:21948329-21948270	OSBPL1A
A_23_P423389	6.118986	6.0322866	NM_006368	chr9:35733444-35735126	CREB3
A_23_P127793	5.039943	5.643723	NM_153265	chr11:62370702-62370643	EML3
A_24_P410627	8.341716	8.19161	NM_001145125	chr7:140156142-140156083	MKRN1
A_33_P3306504	5.2653604	5.626084	NM_016368	chr19:18546437-18546378	ISYNA1
A_23_P77437	5.632309	5.2290497	NM_019023	chr16:68381576-68382298	PRMT7
A_33_P3420792	4.4036913	3.7849045	NM_014891	chr7:98992648-98992589	PDAP1
A_33_P3262020	4.3072686	4.025467	NM_000606	chr9:139841360-139841419	C8G
A_23_P50591	2.3221061	2.3900566	NM_004823	chr19:38818817-38818876	KCNK6
A_24_P132787	9.59565	9.353349	NM_021252	chr10:27828374-27828433	RAB18
A_23_P500381	5.854482	5.5826645	NM_019859	chr10:92500843-92500784	HTR7
A_23_P132845	4.9983277	4.14197	NM_004366	chr3:184064787-184064728	CLCN2
A_23_P31903	11.707433	11.960109	NM_183057	chr8:145649073-145649014	VPS28
A_33_P3246318	12.168908	12.119818	NM_014248	chr22:41368609-41368668	RBX1
A_24_P7121	2.9290454	3.0279708	NM_024677	chr4:40811777-40811836	NSUN7
A_33_P3267270	4.119974	4.5346184	NM_153035	chr1:54561968-54562027	TCEANC2
A_33_P3214159	8.941779	8.946982	NM_001792	chr18:25530995-25530936	CDH2
A_32_P39003	7.347843	7.409822	NR_027503	chr5:99717080-99717021	LOC100133050
A_33_P3258824	8.685737	8.787651	NM_001200001	chr1:120539866-120539807	NOTCH2
A_33_P3224809	9.689207	9.426797	NM_014339	chr22:17590766-17590825	IL17RA
A_23_P390443	4.243785	4.61751	NM_001145176	chr19:55941121-55941062	SHISA7
A_33_P3299110	5.3743773	5.7601995	NM_152432	chr11:100861597-100861656	ARHGAP42
A_33_P3384498	4.2673845	4.3250723	NR_038944	chr22:49293019-49293078	LOC100128946
A_23_P200829	10.695754	10.719231	NM_015326	chr1:206637556-206637615	SRGAP2
A_24_P384200	8.155939	8.281559		chr4:174555262-174555201	
A_32_P17635	7.49027	7.202446	NM_032102	chr11:94804156-94804215	SRSF8
A_32_P525524	3.250628	3.1311328	NM_178495	chr2:96993944-96994003	ITPRIPL1
A_23_P77493	10.472368	10.470321	NM_006086	chr16:90002437-90002496	TUBB3
A_23_P82162	5.778198	5.498497	NM_003080	chr6:109764264-109764508	SMPD2
A_33_P3258723	6.706329	6.6965504	NM_022762	chr5:177575285-177575344	RMND5B
A_33_P3369706	3.7906368	3.7633429	CK906254	chr5:176170811-176170870	
A_33_P3559060	4.2639728	4.0381484	NM_138814	chr22:44276037-44275978	PNPLA5
A_23_P8482	10.268995	10.5309305	NM_001011553	chr7:35943947-35944006	SEPT7
A_23_P306223	5.1679735	5.2467513	NM_139058	chrX:25022494-25022435	ARX
A_23_P169494	3.9855142	3.8867137	NM_000607	chr9:117087130-117087340	ORM1
A_33_P3342967	3.813318	4.194896		chr5:42951949-42952008	LOC100129186

A_23_P6263	4.631441	4.751215	NM_002463	chr21:42780501-42780560	MX2
A_23_P377376	10.861256	11.005549	NM_001005386	chr2:65496889-65496948	ACTR2
A_33_P3316683	3.700166	3.9984045	NM_152897	chr20:44463047-44463106	SNX21
A_33_P3393370	10.033768	10.170317		chr16:001072739-001072680	
A_23_P356526	5.599586	5.185406	NM_033092	chr11:5687290-5687231	TRIM5
A_33_P3387756	6.569543	6.6886406	NM_001007125	chr20:62714996-62714937	C20orf201
A_23_P52219	6.9317365	7.1825285	NM_006459	chr10:101914641-101912075	ERLIN1
A_33_P3358898	4.023551	3.5162523	NM_001144950	chr19:56030351-56030410	SSC5D
A_33_P3502315	10.047238	10.060403	NM_019095	chr20:6017895-6017954	CRLS1
A_23_P208358	13.958832	14.145188	NM_000991	chr19:55899332-55899391	RPL28
A_24_P358425	5.5780687	5.0441837	NM_174931	chr2:37316907-37316966	GPATCH11
A_23_P417200	5.290685	5.0946903	NM_005652	chr16:69390200-69390141	TERF2
A_23_P128073	12.156997	12.121235	NM_139067	chr12:56557192-56557133	SMARCC2
A_33_P3308055	9.744951	9.136544	NM_001282958	chr2:233448276-233448335	EIF4E2
A_33_P3365676	12.071268	11.482116	NM_016395	chr15:65870605-65870664	PTPLAD1
A_33_P3317460	7.5541234	7.5920215		chr2:132056314-132056255	
A_33_P3214096	9.055459	9.056651	NM_001040619	chr1:212792519-212792578	ATF3
A_33_P3424367	7.3106174	7.5691357	NM_033219	chr9:100849918-100849859	TRIM14
A_24_P376441	5.4578905	5.9255543	NM_001025247	chr1:229735704-229735645	TAF5L
A_24_P89509	7.537717	7.362008	NM_001031712	chr6:126332440-126332499	TRMT11
A_23_P158007	5.1987886	5.4782405	NM_032799	chr9:131484026-131483967	ZDHHC12
A_33_P3307820	3.6310792	3.4026604	NM_001013623	chr6:144258797-144258856	ZC2HC1B
A_23_P150407	6.7628455	7.16656	NM_052854	chr11:46342759-46342818	CREB3L1
A_24_P280901	14.626991	14.693792	NM_000982	chr13:27830412-27830461	RPL21
A_23_P30243	9.68684	9.804258	NM_022350	chr5:96253257-96253316	ERAP2
A_33_P3274710	4.1510496	3.2262068		chr19:017528560-017528501	
A_24_P409330	11.257251	11.109095	NM_181304	chr14:23303471-23303530	MRPL52
A_32_P31618	6.9821906	6.892542	NM_000637	chr8:30536325-30536266	GSR
A_33_P3343090	10.170317	10.251202	NM_018174	chr19:17845263-17845322	MAP1S
A_24_P206328	2.6786113	2.3900566	NM_005020	chr7:31855645-31855586	PDE1C
A_33_P3260134	5.92516	6.1813545	NM_002530	chr15:88476303-88476244	NTRK3
A_23_P127367	10.556721	11.051308	NM_021173	chr11:67119197-67119138	POLD4
A_33_P3409665	3.677963	3.2590847	NM_001007188	chr22:32211912-32211971	DEPDC5
A_24_P268676	7.339755	7.594155	NM_003670	chr3:5026260-5026319	BHLHE40
A_33_P3316835	7.247345	7.010505	AK002193	chr4:113527199-113527140	C4orf21
A_33_P3298413	10.847843	10.875428	NM_006347	chr1:43142350-43142409	PPIH
A_33_P3280784	5.058665	5.104829	NM_025125	chr10:81838482-81838541	TMEM254
A_33_P3356502	11.487306	10.980482	NM_002881	chr2:121052201-121052260	RALB
A_24_P860703	9.568821	9.111509	NR_015366	chr20:37049337-37049278	SNHG17
A_33_P3409447	6.474165	6.2519693	NM_016248	chr13:42877096-42877155	AKAP11
A_24_P67699	15.14681	15.15555	NM_000984	chr17:27050590-27050649	RPL23A
A_33_P3292028	11.632503	11.3043995	NM_139159	chr19:4675303-4675244	DPP9
A_33_P3350393	5.7575197	5.79386	NM_003869	chr16:66978670-66978729	CES2
A_33_P3287338	6.4723024	7.139416	NM_001190981	chr5:55256322-55256263	IL6ST
A_33_P3390349	5.706563	5.765025	AK124271	chr9:136655191-136655250	VAV2
A_33_P3371175	7.01695	6.758223	NM_177538	chr2:204170223-204170282	CYP20A1
A_23_P368558	8.921083	8.940174	NM_015348	chr2:98373333-98373274	TMEM131
A_33_P3300680	6.4107804	6.428755	NM_001256046	chr19:30193835-30193776	C19orf12
A_33_P3407937	5.512102	5.3704867	NM_018390	chrX:208260-208319	PLCXD1
A_32_P208178	15.357016	15.376772	NM_001006	chr4:152025345-152025404	RPS3A
A_24_P376787	5.802602	5.883762	NM_032752	chr1:247473031-247471862	ZNF496
A_24_P333571	4.977684	5.4230795	BC022483	chr1:94667593-94667534	ARHGAP29
A_24_P283928	5.2431164	4.7281837	NM_024068	chr12:56619283-56619454	NABP2
A_23_P219084	5.5342107	5.6016874	NM_032924	chr7:99668343-99668284	ZNF3
A_33_P3244026	7.500573	7.128663	NM_020746	chr20:3856703-3856762	MAVS
A_24_P146670	9.242757	9.050338	NM_014720	chr10:105786654-105786713	SLK
A_23_P16817	9.931082	10.303544	NM_004071	chr2:201719388-201718707	CLK1
A_23_P210708	9.200103	9.374538	NM_001040022	chr20:1920284-1920343	SIRPA
A_23_P218654	9.595449	9.668042	NM_032527	chr20:62367408-62367467	ZGPAT

A_33_P3661631	8.015957	7.978269	NM_032151	chr5:134296360-134296419	PCBD2
A_23_P120953	4.1961727	4.1478133	NM_014509	chr22:42970262-42970321	SERHL2
A_33_P3298267	5.530565	5.5614076		chr2:218934714-218934773	
A_23_P500353	3.2948813	3.4506564	NM_021614	chr5:113831835-113831894	KCNN2
A_23_P92999	3.6452858	3.2687285	NM_016279	chr5:26881049-26880990	CDH9
A_33_P3342448	3.904726	3.0721307	AK172736	chr10:62267881-62267822	ANK3
A_23_P133694	10.3334465	10.24084	NM_001078177	chr6:44201775-44201834	SLC29A1
A_33_P3417695	3.2674284	3.70051	NM_001014440	chr22:50968929-50968870	ODF3B
A_33_P3217465	11.114858	10.722156	NM_021242	chrX:38665568-38665627	MID1IP1
A_33_P3849275	8.4106045	8.515748	NM_001159704	chrX:135293033-135293092	FHL1
A_23_P427148	4.804696	4.6638393	NM_152465	chr17:27030606-27030547	PROCA1
A_23_P258698	5.3962216	5.912923	NM_005908	chr4:103553279-103553220	MANBA
A_33_P3348313	8.735189	8.564769	NM_001031703	chr3:47537651-47537592	ELP6
A_23_P66732	6.1341076	6.2164416	NM_031965	chr17:3629418-3629477	GSG2
A_23_P130376	3.6660063	3.7459493	NM_022068	chr18:10671274-10671215	PIEZO2
A_33_P3557274	6.8474846	7.0897985	XM_003846648	chr2:63745412-63745353	LOC100996924
A_33_P3343845	7.929824	8.138595	NM_175709	chr22:39529778-39529719	CBX7
A_23_P141636	12.343213	12.177249	NM_014740	chr17:78109220-78109161	EIF4A3
A_23_P49145	3.6872387	3.8381178	NM_152338	chr16:29791761-29791821	ZG16
A_23_P160200	6.2685533	5.6365705	NM_023070	chr1:40929073-40929132	ZFP69B
A_32_P24376	5.2129364	4.871302	NM_001165252	chr17:39215637-39215578	KRTAP2-3
A_33_P3418158	4.3856688	4.0910563	NM_001271711	chr9:130166022-130166081	SLC2A8
A_23_P95302	9.872628	9.98218	NM_181578	chr12:118469774-118469833	RFC5
A_24_P179489	6.738345	6.9324303	NM_006642	chr1:243456514-243468066	SDCCAG8
A_33_P3380992	5.8295336	4.987285	NM_001080538	chr7:134262468-134262527	AKR1B15
A_33_P3356255	5.191903	5.2826176		chr10:81916224-81916165	ANXA11
A_32_P174572	6.3097277	5.921506	NR_002774	chr12:13157429-13157488	HTR7P1
A_23_P41629	5.229457	4.7695084	NM_139056	chr5:5319897-5319956	ADAMTS16
A_23_P161918	11.591817	11.144116	NM_024098	chr11:60618471-60618530	CCDC86
A_23_P119714	9.830259	9.827078	NM_001033549	chr19:17389870-17389929	BABAM1
A_24_P69053	5.446971	5.2673774	NM_001080477	chr4:183717844-183717903	TENM3
A_23_P144916	8.45203	7.7263536	NM_005110	chr5:179727826-179727767	GFPT2
A_23_P77401	8.940729	9.146902	NM_018340	chr16:12757607-12757548	CPPED1
A_33_P3278220	8.64259	8.304273	AL832249	chr9:127984607-127984666	RABEPK
A_33_P3228340	5.1407185	4.886935	BC040558	chr2:44019837-44019896	DYNC2LI1
A_33_P3367642	11.486273	11.616554	NM_033200	chr22:50941440-50941381	LMF2
A_24_P134834	6.7273436	6.482497	NM_032276	chr2:227861047-227861106	RHBDD1
A_24_P921477	7.4970016	7.656957	NM_001206987	chr17:74729078-74729137	METTL23
A_33_P3331687	9.306452	9.638132	NM_001145638	chr9:139252936-139252995	GPSM1
A_33_P3395321	10.14983	10.553964	NM_001002032	chr17:73143711-73143652	HN1
A_24_P216421	4.6777945	4.030281	NM_033148	chr2:25656824-25655855	DTNB
A_24_P222911	8.219502	8.046134	NM_001031684	chr2:38971277-38971218	SRSF7
A_33_P3275973	4.374796	5.5328026		chr6:107218153-107218094	LOC100422737
A_33_P3232523	3.8155165	4.1004148	BC006276	chr22:36593748-36593689	APOL4
A_33_P3405500	7.124167	7.255102	NM_014363	chr13:23927984-23927925	SACS
A_33_P3234540	5.5783863	5.9949675		chr16:002390856-002390915	
A_23_P46429	11.026471	10.771115	NM_001554	chr1:86048953-86049012	CYR61
A_23_P20384	10.7832985	10.904322	NM_014462	chr8:38021141-38021082	LSM1
A_23_P215980	4.2096686	4.11421	NM_183009	chr8:95523940-95523881	KIAA1429
A_24_P234415	6.832191	6.5940485	NM_003149	chr3:36588779-36588838	STAC
A_23_P47282	4.0943623	4.458634	NM_021978	chr11:130079815-130079874	ST14
A_33_P3333507	4.8357663	5.434821	NM_020654	chr3:101044770-101044711	SENP7
A_23_P4909	5.8831778	5.9690537	NM_003089	chr19:49601960-49604704	SNRNP70
A_23_P144578	8.352757	8.58181	NM_138335	chr4:44704437-44704378	GNPDA2
A_33_P3224595	6.712003	6.521018	NM_003611	chrX:13769427-13769486	OFD1
A_33_P3280094	10.113871	10.040699	NM_024036	chr11:66627888-66627946	LRFN4
A_33_P3272828	7.02362	6.812739	NM_002853	chr5:34914859-34914800	RAD1
A_33_P3281741	4.7559633	4.578062	NM_001145664	chr2:102022465-102022406	RFX8
A_33_P3243683	3.5043304	2.3900566	NM_005219	chr5:140905927-140905868	DIAPH1

A_23_P308305	7.1452456	7.025795	NM_001243425	chr18:21599978-21600037	TTC39C
A_32_P161913	4.653941	5.03074	NM_001114748	chr1:1470303-1470244	TMEM240
A_33_P3241984	4.209349	3.2652814	NM_015967	chr1:114356520-114356461	PTPN22
A_33_P3335606	7.652466	7.411725		chr1:200183583-200183642	
A_33_P3273020	11.808479	11.884546	NM_004470	chr11:64011457-64011516	FKBP2
A_23_P106127	8.901769	8.81872	NM_014749	chr14:59014698-59014757	KIAA0586
A_33_P3407941	4.463297	4.54605		chr11:000132170-000132229	
A_23_P65518	2.3221061	2.3900566	NM_016651	chr14:59114713-59114772	DACT1
A_23_P155147	7.4454494	7.545857	NM_014838	chr22:50281708-50281767	ZBED4
A_23_P42080	10.572796	10.495914	NM_014051	chr6:52548899-52548958	TMEM14A
A_23_P30655	8.341151	8.584979	NM_004556	chr6:44226617-44226558	NFKBIE
A_33_P3330683	13.612024	13.700625		chr2:137087011-137086952	XLOC_014512
A_33_P3312802	9.610316	9.825396	NR_003680	chr7:77988714-77988773	RPL13AP17
A_33_P3388501	8.059801	8.101704	NM_003465	chr1:203185861-203185802	CHIT1
A_23_P127013	8.48196	8.561216	NM_030756	chr10:114925978-114926037	TCF7L2
A_33_P3314386	9.311007	8.72789	NM_001267578	chr1:179888942-179889001	TOR1AIP1
A_33_P3364854	4.283028	3.721494	NM_018646	chr7:142569442-142569383	TRPV6
A_33_P3274080	5.598241	5.767525		chrY:024450634-024450575	
A_23_P21548	7.420541	7.5182614	NM_024605	chr4:148993428-148993487	ARHGAP10
A_23_P317756	5.0261564	5.8723655	NM_202000	chr16:20793038-20793097	ACSM3
A_33_P3313640	4.417987	4.6016345		chr2:001663722-001663663	
A_23_P160154	6.609808	6.331382	NM_000403	chr1:24123592-24123533	GALE
A_24_P365807	8.340704	8.358159	NM_004429	chrX:68061833-68061892	EFNB1
A_33_P3222659	4.103352	3.6111166		chr15:84872156-84872097	LOC101929847
A_33_P3287959	3.6881678	3.7766414	NM_006989	chr7:102220317-102220258	RASA4
A_33_P3339212	10.970736	10.8740425	NM_004237	chr5:918002-918061	TRIP13
A_23_P46063	8.282475	8.294604	NM_013330	chr1:169200002-169138780	NME7
A_23_P60283	5.7620206	5.7913446	NM_000380	chr9:100437572-100437513	XPA
A_33_P3393927	5.7615438	6.044768	NM_181873	chr1:149901167-149901108	MTMR11
A_23_P218463	8.026605	7.965808	NM_013376	chr19:40928494-40928435	SERTAD1
A_23_P430411	5.505977	6.5732	XM_005261115	chr21:46326865-46321625	ITGB2
A_23_P24987	5.5551105	6.06243	NM_005981	chr12:58141186-58141245	TSPAN31
A_23_P21776	7.884915	8.291226	AK292051	chr15:66786526-66786467	SNAPC5
A_23_P215479	3.9197173	3.8632615	NM_003388	chr7:73819943-73820002	CLIP2
A_33_P3275751	10.998927	11.114081	NM_001204410	chr17:75212949-75213008	SEC14L1
A_33_P3303121	5.591467	4.5691175	NM_002886	chr3:152886164-152886223	RAP2B
A_33_P3235322	4.3951063	3.7095368	NM_032872	chr1:27671923-27671982	SYTL1
A_24_P255218	7.4809484	7.2719283	NM_000259	chr15:52643522-52643463	MYO5A
A_23_P12503	9.581781	9.411673	NM_018230	chr1:229577223-229577164	NUP133
A_33_P3217559	4.8832746	4.866878	NM_003836	chr14:101200586-101200645	DLK1
A_24_P532180	4.744808	4.7255893	NR_026954	chr3:128228842-128228901	LOC90246
A_33_P3245824	10.4429655	10.621693	NM_025078	chr18:77662546-77662487	PQLC1
A_23_P61960	5.6137104	5.425542	NM_145230	chr7:149576953-149577012	ATP6V0E2
A_24_P904903	7.866905	8.014938	NM_004640	chr6:31504360-31504301	DDX39B
A_33_P3270384	11.874897	11.80975	NM_138689	chr11:64012080-64012021	PPP1R14B
A_32_P209624	4.5795975	4.827509	NR_026640	chr20:45092587-45092528	MKRN7P
A_23_P89422	6.0845895	6.120202	NM_080282	chr17:67144146-67144131	ABCA10
A_23_P157038	5.564905	6.0018473	NM_152755	chr7:99722740-99722799	CNPY4
A_33_P3639068	10.119309	10.291005	NM_005264	chr10:117823955-117823896	GFRA1
A_24_P15640	6.0454254	6.139403	BC137009	chr19:4909287-4909228	
A_23_P154488	9.430839	9.213438	NM_033109	chr2:55864694-55863476	PNPT1
A_23_P74663	7.4957895	7.328692	NM_005681	chr1:222734784-222734725	TAF1A
A_33_P3379004	3.988995	4.2108936	AK130723	chr13:99205558-99205499	
A_23_P27822	4.3031263	4.486585	NM_018025	chr19:33603510-33604722	GPATCH1
A_33_P3403044	7.613836	8.144626	NM_005911	chr2:85771178-85771237	MAT2A
A_23_P386942	3.7663755	3.0759823	NM_145173	chr19:2714640-2714581	DIRAS1
A_33_P3235776	3.8827305	4.516033	BX640728	chr6:119327266-119327207	FAM184A
A_33_P3252915	5.2445526	5.585233	NR_037702	chr3:141461507-141461566	RNF7
A_23_P305033	2.561499	2.3900566	NM_152787	chrX:30848882-30848823	TAB3

A_23_P502035	6.135225	6.003866	NM_000155	chr9:34648881-34649043	GALT
A_23_P335848	9.171151	8.993384	NM_057169	chr12:110367679-110367620	GIT2
A_23_P149664	9.052461	9.362856	NM_001079809	chr1:202992196-202992255	TMEM183B
A_33_P3359473	7.792239	8.069704	NM_032701	chr19:55859429-55859488	SUV420H2
A_33_P3409672	3.747529	2.3900566	NM_001242896	chr22:32302415-32302474	DEPDC5
A_23_P74609	13.600891	12.937544	NM_015714	chr1:209849597-209849656	G0S2
A_23_P135611	6.79023	7.0250463	NM_018114	chr3:49053704-49053645	DALRD3
A_32_P226498	6.0102606	6.2677307		chr20:61735320-61735379	HAR1A
A_24_P336848	6.42529	6.4594173	NM_138448	chr2:54365796-54365855	ACYP2
A_33_P3282619	6.6540785	6.4868774	AK130019	chr11:119371186-119371245	USP2-AS1
A_23_P149200	9.624003	9.384023	NM_001255	chr1:43826537-43826596	CDC20
A_23_P140630	5.291143	5.473218	NM_021819	chr15:75114219-75115019	LMAN1L
A_23_P25913	7.836921	8.092535	NM_031427	chr14:74156210-74162655	DNAL1
A_33_P3220827	6.739225	6.1852813	NM_003768	chr1:160183766-160183825	PEA15
A_23_P106069	4.0379205	3.6909308	X58747	chr14:22237165-22237224	
A_33_P3332166	5.2225866	5.248827	NM_183062	chr1:228034024-228034083	PRSS38
A_33_P3384392	9.522331	9.192605	NM_001619	chr11:67053947-67054006	ADRBK1
A_24_P414332	7.4322095	8.222618	NM_005935	chr4:88061248-88061307	AFF1
A_23_P200598	7.027101	6.797023	NM_024544	chr1:20825962-20825925	MUL1
A_23_P139500	3.6778498	2.3900566	NM_030762	chr12:26273691-26273632	BHLHE41
A_23_P86632	7.718913	7.8624277	NM_001033858	chr10:14950558-14950499	DCLRE1C
A_23_P29922	6.3393726	6.6439505	NM_003265	chr4:187005890-187005949	TLR3
A_23_P4400	5.142111	5.2696705	NM_033059	chr17:39273838-39273779	KRTAP4-11
A_23_P70733	5.9866714	5.944029	NM_001033080	chr6:132938784-132938725	TAAR2
A_24_P237661	5.8768315	5.955389	NM_001190438	chr17:16068394-16068335	NCOR1
A_23_P257131	8.137137	8.425447	NM_002618	chr2:61275849-61275908	PEX13
A_33_P3420500	3.7495089	3.0315504	BC034944	chr13:21928107-21928048	ZDHHC20
A_33_P3343073	7.0373945	6.826354	NM_003073	chr22:24167466-24167525	SMARCB1
A_33_P3315355	7.2522726	7.1728954	NM_001160305	chr16:58552942-58553001	SETD6
A_23_P217659	10.403095	10.253834	NM_001018055	chrX:154351125-154351184	BRCC3
A_33_P3270084	6.9211273	6.8036003		chr1:001577426-001577367	
A_24_P215475	4.9868636	5.371482	NM_015394	chr12:133735529-133735588	ZNF10
A_23_P321846	2.4401715	2.3900566	NM_002251	chr20:43721339-43721280	KCNS1
A_33_P3308905	6.8950095	6.87353	NM_001271853	chr6:109484879-109484938	CEP57L1
A_33_P3305758	9.917612	10.0146	NM_001256140	chr2:85628394-85628335	CAPG
A_24_P345451	6.242222	6.8653545	NM_024843	chr2:172411114-172411173	CYBRD1
A_23_P31453	6.9244003	6.963148	NM_012449	chr7:89794004-89794063	STEAP1
A_23_P12526	8.556328	8.748003	NM_005426	chr1:223968290-223968231	TP53BP2
A_23_P205611	6.1741858	6.214266	NM_004124	chr14:54955649-54950433	GMFB
A_33_P3408877	5.4978466	5.474748			
A_33_P3209346	5.4186745	5.3194976	NM_013417	chr9:95043166-95043107	IARS
A_24_P769672	7.5230346	7.6157255	NM_001135570	chr12:104345255-104345196	C12orf73
A_33_P3252605	6.800676	6.8217072	NM_183075	chr4:108874445-108874504	CYP2U1
A_23_P42198	8.41438	8.153679	NM_003534	chr6:26271469-26271410	HIST1H3G
A_23_P86504	8.708657	8.744717	NM_024541	chr10:103605651-103605592	C10orf76
A_33_P3311646	7.323043	7.583662		chr7:101286790-101286849	
A_32_P300427	2.3221061	2.3900566	NM_153360	chr20:57034568-57034509	APCDD1L
A_23_P168368	5.49247	5.65579	NM_025031	chr7:47859325-47859384	C7orf69
A_33_P3273719	7.0328345	7.053636	NM_001242831	chr6:53159170-53159111	ELOVL5
A_23_P217079	8.098239	7.9768934	NM_003863	chr9:130697655-130697596	DPM2
A_23_P327519	6.7737427	6.992693	NM_139164	chr5:110834747-110834688	STARD4
A_33_P3223874	14.58107	14.566067	CR936824	chr10:75182214-75182155	MSS51
A_33_P3228266	9.248102	9.292695	NM_000099	chr20:23614617-23614558	CST3
A_23_P148600	4.394286	3.862328	NR_024616	chrX:47064732-47064791	INE1
A_23_P369328	9.067182	8.761666	NM_145306	chr10:71393191-71393250	C10orf35
A_32_P150735	4.6356325	3.744742		chr7:107271020-107271079	
A_23_P501805	6.9673443	6.968599	NM_145197	chr2:99779128-99779187	LIPT1
A_23_P73660	7.4085526	7.6209455	NM_001011658	chrX:13730648-13730589	TRAPPC2
A_33_P3246163	14.342569	14.254614	NM_000969	chr1:93307337-93307396	RPL5

A_23_P205623	5.0160527	4.687558	NM_030637	chr14:53521323-53521264	DDHD1
A_33_P3316310	4.995552	5.189437	NM_182501	chr2:242039005-242038946	MTERFD2
A_33_P3733417	3.6437268	4.036071	NM_000795	chr11:113280398-113280339	DRD2
A_23_P94533	10.503475	10.757356	NM_001912	chr9:90345326-90345385	CTSL
A_33_P3325285	3.7133684	3.7133684		chr1:226342605-226342664	
A_33_P3243812	4.647216	4.513371	NM_003931	chr6:110429790-110429731	WASF1
A_23_P201996	11.373728	11.383368	NM_016628	chr10:28909391-28909450	WAC
A_23_P250231	5.0734997	5.1024127	NM_019644	chr7:117880040-117882449	ANKRD7
A_24_P393372	6.835864	6.8907247	NM_001100913	chr14:105864372-105864431	PACS2
A_32_P80850	4.8467917	4.7665334	NM_021110	chr8:121381649-121381708	COL14A1
A_33_P3223472	8.416481	8.403543	NM_016627	chr17:66251957-66252016	AMZ2
A_23_P214766	6.0817533	5.5910397	NM_006734	chr6:143072910-143072851	HIVEP2
A_23_P79331	7.9296026	7.854946	NM_022152	chr2:219139611-219139552	TMBIM1
A_33_P3225313	5.1906686	5.2303085	NM_144963	chr8:124787427-124787486	FAM91A1
A_23_P107421	12.146107	12.336824	NM_003258	chr17:76170252-76170193	TK1
A_33_P3415617	3.461348	4.445431		chr10:101941285-101941344	
A_23_P396626	5.9206495	6.124298	NM_007247	chr17:35878116-35878057	SYNRG
A_33_P3384562	8.27455	8.715676	NM_213636	chr5:176917306-176917247	PDLIM7
A_33_P3268338	8.060977	7.8187895	NM_023935	chr20:3171130-3171071	DDRGK1
A_33_P3294524	8.252829	8.361214	NM_001204056	chr18:9255735-9255794	ANKRD12
A_33_P3307795	3.8397765	4.140739		chr2:225264301-225264242	FAM124B
A_33_P3349455	4.7209105	4.620708		chr22:016084263-016084204	
A_33_P3397399	7.2368402	6.9628806	AK301582	chr10:75204417-75204358	PPP3CB
A_32_P221991	4.0257483	3.9449155	NM_014636	chr9:129728187-129739989	RALGPS1
A_23_P48175	11.958658	12.008162	NM_024056	chr12:48362562-48362621	TMEM106C
A_23_P1292	5.9690065	6.4079223	NM_000124	chr10:50666865-50666806	ERCC6
A_23_P30162	4.398632	4.9171495	NM_152778	chr4:128843091-128843032	MFSD8
A_24_P410952	8.218501	7.6489058	NM_003768	chr1:160184044-160184103	PEA15
A_23_P139786	6.492554	5.9774375	NM_003733	chr12:121458379-121458320	OASL
A_33_P3328837	12.397317	12.488769		chr17:041321982-041321923	
A_23_P50872	8.72127	8.652798	NM_004146	chr19:14682759-14677745	NDUFB7
A_24_P221198	5.2496514	5.2815537	NM_203397	chr7:99725936-99725995	MBLAC1
A_33_P3328736	8.248035	8.282781	NM_199342	chr1:43272901-43272842	CCDC23
A_24_P458252	4.2224345	4.610253		chr10:043221301-043221360	
A_24_P98251	9.915903	9.499698	NM_017890	chr8:100889712-100889771	VPS13B
A_24_P365015	2.3221061	2.3900566	NM_006361	chr17:46802203-46802144	HOXB13
A_33_P3228402	3.3894234	3.4285007		chrX:073096726-073096785	
A_23_P63371	3.1469946	3.1761446	NM_003189	chr1:47682477-47682418	TAL1
A_23_P165007	9.606899	9.838178	NM_170604	chr19:38899919-38899860	RASGRP4
A_23_P127128	7.113491	7.341049	NM_022365	chr10:22048227-22048168	DNAJC1
A_23_P125233	2.3221061	2.3900566	NM_001299	chr19:11661005-11661064	CNN1
A_32_P101860	6.417308	6.437312	XM_005257003	chr17:41371986-41372045	TMEM106A
A_23_P141715	12.573992	12.618595	NM_015476	chr18:34376149-34376090	TPGS2
A_33_P3257170	4.43044	4.9464664	XR_241921	chr6:30529146-30529205	PRR3
A_23_P254702	12.110717	11.826785	NM_003472	chr6:18225006-18224948	DEK
A_23_P214354	5.1207557	5.442229	NM_018303	chr6:486031-485972	EXOC2
A_33_P3510335	4.5939517	4.4150877	NM_213655	chr12:978138-978197	WNK1
A_33_P3366224	9.224987	9.513724		chr4:077589366-077589425	
A_24_P251381	5.3303585	6.0330644	NM_024782	chr2:219941048-219940989	NHEJ1
A_33_P3295066	13.851727	13.802238	NR_026673	chr7:20867351-20867410	RPL23P8
A_33_P3337131	3.6681805	4.4028473	NR_027426	chr22:22906241-22906300	LOC648691
A_23_P151405	10.586225	10.697035	NM_018204	chr13:53050394-53050453	CKAP2
A_24_P178444	4.585135	4.674733		chr11:107047965-107048024	
A_23_P256312	2.788288	2.3900566	NM_002447	chr3:49924752-49924693	MST1R
A_24_P68631	8.778378	8.812031	NM_175065	chr1:149859244-149859185	HIST2H2AB
A_23_P41470	7.675034	7.9872103	NM_017631	chr4:169142950-169142891	DDX60
A_23_P41487	8.206033	8.225903	NM_015130	chr4:141542600-141542541	TBC1D9
A_23_P359647	9.136426	8.764149	NM_138714	chr16:69738311-69738370	NFAT5
A_24_P263543	13.250324	13.312776		chr10:091738701-091738760	

A_24_P944588	4.3245964	3.8915024	NM_033196	chr19:20115520-20115461	ZNF682
A_23_P165657	14.282986	13.730812	NM_005415	chr2:113421288-113421347	SLC20A1
A_24_P548866	5.759205	5.347296	NM_001099668	chr3:42824462-42824403	HIGD1A
A_33_P3212490	8.524973	7.8892965	NM_001195573	chr14:95552674-95552615	DICER1
A_23_P19164	8.767188	8.904715	NM_003314	chr5:159476560-159476619	TTC1
A_23_P76109	8.90164	8.839635	NM_145058	chr12:123900062-123900003	RILPL2
A_33_P3285715	9.834597	10.094971	XM_005250869	chr8:144357115-144357174	GLI4
A_23_P253052	9.9246025	10.008818	NM_031462	chrX:149934887-149934828	CD99L2
A_33_P3788355	3.5996022	3.6319938	NR_033989	chr11:19532517-19532458	NAV2-AS4
A_33_P3239317	6.020439	5.8449264	NR_024270		LOC400752
A_24_P261734	11.337995	10.86678	NM_030674	chr12:46577120-46577061	SLC38A1
A_24_P211151	6.754632	6.4200506	NM_020158	chr19:41898840-41898781	EXOSC5
A_23_P27285	8.413981	8.221435	NM_023075	chr18:11884349-11884290	MPPE1
A_33_P3254956	5.3082786	5.2224817	NM_018962	chr21:38391860-38391919	RIPPLY3
A_24_P162979	4.013726	4.067184	NM_024933	chr2:71211943-71212002	ANKRD53
A_23_P88184	6.8652315	7.3359933	NM_018167	chr14:93754458-93754399	BTBD7
A_33_P3332130	11.358183	11.406555	NM_001282621	chrX:118378206-118378265	PGRMC1
A_23_P21363	6.4104643	6.702388	NM_024060	chr11:62201142-62201083	AHNAK
A_33_P3691860	6.243326	6.625035	NM_001166599	chrX:133906117-133906058	FAM122B
A_23_P19673	5.5304294	6.421959	NM_005627	chr6:134490715-134490656	SGK1
A_24_P212152	14.605461	14.67751	NM_025029	chr2:130948209-130948268	MZT2B
A_23_P26674	8.535705	8.587911	NM_014647	chr16:15688367-15688308	KIAA0430
A_33_P3294177	3.7600315	3.553862	AK124222	chr6:36723389-36723330	LOC100131043
A_33_P3222852	4.660556	4.8803	AY203941	chr19:35602929-35602988	
A_24_P555510	7.0155	6.409296	NM_006197	chr8:17887294-17887353	PCM1
A_33_P3465247	7.280778	6.759199	NM_007054	chr5:132032073-132032014	KIF3A
A_33_P3232277	7.5398407	7.816707	NM_004060	chr5:162866292-162866351	CCNG1
A_23_P14284	10.64076	11.016445	NM_006370	chr14:68118087-68118028	VTI1B
A_33_P3357490	3.4954176	2.3900566	NM_001009565	chr2:39405945-39405886	CDKL4
A_33_P3319041	9.105363	8.062946	NM_005342	chrX:150159180-150159239	HMGB3
A_33_P3392087	7.044365	7.3722963	NM_000203	chr4:998257-998316	IDUA
A_24_P104119	7.514962	7.240153	NM_019034	chr12:122216710-122216651	RHOF
A_24_P159635	4.315037	3.4866452	NM_030576	chr17:61773681-61773622	LIMD2
A_33_P3511777	4.5451117	4.2089643	NR_038895	chr18:3896996-3897055	DLGAP1-AS3
A_23_P74330	5.3535914	5.9026475		chr1:168761919-168761978	LINC00626
A_33_P3314468	6.6534534	6.6774325	NM_001100170	chr2:86400863-86400804	IMMT
A_24_P371628	4.876343	4.507066	NM_054027	chr5:14709858-14709799	ANKH
A_24_P82135	5.3325677	5.4016685	NM_014701	chr15:49281776-49281717	SECISBP2L
A_33_P3278868	6.824914	6.6162305	BC062720	chr14:31869535-31869476	HEATR5A
A_33_P3298535	12.079157	12.282138	NM_080748	chr20:34288773-34288832	ROMO1
A_33_P3876985	8.856905	8.941015	NM_016076	chr1:244855262-244855321	DESI2
A_24_P287941	7.5062027	7.2504597	NM_013290	chr17:40724775-40724716	PSMC3IP
A_24_P98975	8.876369	8.861438	NM_033121	chr12:110476921-110476980	ANKRD13A
A_32_P448360	6.221105	6.230562	NR_049729	chr17:42254031-42253972	ASB16-AS1
A_23_P57534	5.0898814	4.898054	NM_006386	chr22:38882199-38882140	DDX17
A_33_P3251322	5.8351407	5.6683784	AK128312	chr3:9742908-9742967	MTMR14
A_23_P36562	6.649584	6.969487	NM_002205	chr12:54789715-54789656	ITGA5
A_23_P155939	6.050591	6.5100265	NM_182524	chr4:86842-86901	ZNF595
A_23_P82000	6.8369226	6.823765	NM_003214	chr6:35441579-35441520	TEAD3
A_33_P3414930	3.9711444	3.9760876	XR_244435	chr22:39487686-39487745	
A_33_P3421867	6.198016	6.4236917	AK126965	chr6:37606891-37606832	MDGA1
A_24_P239811	4.082165	4.4287577	NM_183008	chr1:26609228-26609169	UBXN11
A_24_P361006	9.932953	9.910433	NM_005002	chr12:4777675-4778950	NDUFA9
A_33_P3375736	4.5445795	4.654667	NM_170710	chr4:177102634-177102693	WDR17
A_24_P100551	7.876213	7.4134245	NM_020870	chr4:170015564-170015505	SH3RF1
A_33_P3317725	7.7833104	7.8584876	XR_159139	chr6:30780975-30780916	LINC00243
A_33_P3387931	8.246935	7.9274406	NM_001012267	chr9:95377376-95377435	CENPP
A_33_P3350202	9.102263	9.278418	NM_014484	chr20:49577728-49577787	MOCS3
A_33_P3367994	8.653526	8.540449	NM_138572	chr6:42048457-42048516	TAF8

A_24_P936122	10.238424	10.092132	NM_001001433	chr20:57254353-57254412	STX16
A_33_P3267118	8.842832	8.770113		chr1:21219913-21219854	EIF4G3
A_23_P146654	8.974194	8.969927	NM_004323	chr9:33255124-33255065	BAG1
A_33_P3238007	5.9354305	6.2532864	NM_178043	chr4:129028408-129028467	LARP1B
A_23_P154962	5.255922	4.9817295	NM_015672	chr22:20456128-20456069	RIMBP3
A_24_P365506	2.761815	2.3900566	NM_017671	chr20:6068519-6068460	FERMT1
A_23_P147826	6.052313	6.0328555	NM_021183	chrX:131348187-131339582	RAP2C
A_33_P3883912	8.816158	7.4494224	NM_017665	chr5:132332756-132332697	ZCCHC10
A_24_P138713	6.972079	6.8129225	NM_182922	chr16:50136324-50138886	HEATR3
A_33_P3295640	4.07944	4.134898	NM_004886	chr19:3750838-3750779	APBA3
A_23_P30223	6.5038238	6.434249	NM_001047	chr5:6668693-6668752	SRD5A1
A_33_P3218450	4.6697783	4.119442	NM_001254	chr17:38459354-38459413	CDC6
A_24_P818268	5.2158766	5.3266206		chr15:074357534-074357593	
A_23_P362893	9.465176	9.521969	NM_021961	chr11:12965225-12965284	TEAD1
A_23_P2006	4.1323166	3.7390807	NM_001037329	chr11:6265325-6265384	CNGA4
A_33_P3284854	6.16254	6.358258		chr13:64414947-64414888	
A_23_P420334	5.300311	5.29465	NM_152595	chr15:34396111-34396170	PGBD4
A_33_P3245183	4.145209	4.040558	NM_001098213	chr3:11304879-11304938	HRH1
A_23_P139388	7.2162623	7.5037613	NM_016578	chr11:77377685-77377626	RSF1
A_33_P3242973	4.8546762	5.0766687	NM_006548	chr3:185364863-185363403	IGF2BP2
A_24_P380679	6.261919	5.625786	NM_182597	chr7:112130859-112130918	LSMEM1
A_23_P153640	8.273283	8.200175	NM_032139	chr19:33088371-33088312	ANKRD27
A_33_P3211238	3.6267188	3.8692355	NM_152718	chr11:61026206-61026147	VWCE
A_33_P3379967	5.168523	5.275976	NM_001098478	chr6:29692114-29692173	HLA-F
A_32_P41026	4.55637	3.850552	NM_001024956	chr11:121183280-121183339	SC5D
A_23_P347198	11.031063	11.003538	NM_003111	chr2:174773350-174773291	SP3
A_33_P3330811	5.4143543	5.2056375	NM_001099670	chr8:86126354-86126295	C8orf59
A_24_P141214	7.5643873	7.9450827	NM_198194	chr9:124101935-124101876	STOM
A_33_P3235987	8.488623	8.343842	NM_006223	chrX:71416695-71416754	PIN4
A_24_P243776	9.514012	9.63634	NM_021994	chr7:111982790-111982849	ZNF277
A_33_P3342235	8.412813	8.529621	NM_145057	chr19:54976270-54976211	CDC42EP5
A_23_P217666	14.809121	14.899027	NM_006013	chrX:153628927-153629061	RPL10
A_23_P148568	2.5732121	2.3900566	NM_022053	chrX:101615381-101615322	NXF2
A_23_P54816	12.7275095	12.810267	NM_001694	chr16:2570094-2570153	ATP6V0C
A_23_P345678	4.6267014	4.3530016	NM_033084	chr3:10140927-10140986	FANCD2
A_33_P3284533	4.398746	4.254793	L06884	chr14:22392730-22392789	
A_33_P3325018	9.410728	9.562331	NM_015701	chr2:54045844-54045903	ERLEC1
A_32_P54544	12.477388	12.408525	NM_001762	chr7:56130749-56130808	CCT6A
A_32_P46765	7.9768934	7.658924	NM_001009894	chr12:88443701-88443760	C12orf29
A_33_P3417086	6.401042	6.022605	NM_207330	chr4:48039016-48039075	NIPAL1
A_33_P3422654	6.4801283	6.646076		chr2:70351510-70351451	LOC100133985
A_33_P3273534	14.388734	14.046365	NM_002281	chr12:52679758-52679699	KRT81
A_23_P162846	13.04334	13.382492	NM_005561	chr13:113977667-113977726	LAMP1
A_23_P155301	4.819148	5.390135	NM_145910	chr3:130889661-130889720	NEK11
A_23_P24922	5.1692405	5.2317276	NM_001144869	chr11:74203122-74203063	LIPT2
A_23_P394545	8.208789	8.749625	NM_015275	chr12:105540848-105540907	KIAA1033
A_23_P345942	10.567489	10.404069	NM_174889	chr5:60241158-60368959	NDUFAF2
A_33_P3294449	7.0920453	7.5898666	NM_001017977	chr1:167973948-167974007	DCAF6
A_33_P3404879	3.8204112	3.5576017	NM_012302	chr1:82447600-82447659	LPHN2
A_33_P3304908	6.530618	6.191882	XR_247761	chr19:65936-65877	WASH5P
A_23_P257417	6.6684184	6.9479547	NM_152385	chr2:55404782-55403108	CLHC1
A_23_P166826	9.960455	9.672606	NM_018403	chr3:53321447-53321388	DCP1A
A_23_P74112	7.018408	6.8441806	NM_170743	chr1:24480826-24480767	IFNLR1
A_33_P3242649	8.662472	8.823403	NM_031217	chr11:28042475-28042416	KIF18A
A_33_P3318465	11.214775	11.449564	NM_003352	chr2:203071550-203071491	SUMO1
A_24_P403303	5.606451	4.1323166	NM_198513	chr8:133824957-133825016	PHF20L1
A_23_P7066	14.526909	14.497476	NM_001024921	chr4:39458091-39458032	RPL9
A_33_P3281018	5.067606	5.3691754		chr7:158799418-158799477	LINC00689
A_24_P167984	8.062291	8.31072	NM_015251	chr16:81078373-81078432	ATMIN

A_33_P3350056	11.14019	11.349119	NM_005952	chr16:56717082-56717141	MT1X
A_23_P204579	9.851071	9.604943	NM_003211	chr12:104382442-104382501	TDG
A_24_P207727	7.7796545	7.545901	NM_001003891	chr22:20941373-20941432	MED15
A_33_P3413048	6.772123	6.822432	NM_017514	chrX:153701925-153701984	PLXNA3
A_23_P320113	10.838022	10.629968	NM_080725	chr20:627622-627563	SRXN1
A_33_P3337242	6.606178	6.387122	NM_001271667	chr6:15524832-15524773	DTNBP1
A_33_P3217731	3.0653918	2.3900566		chr9:84214882-84214823	TLE1
A_23_P161022	9.895959	10.049836	NM_012437	chr1:153633864-153633923	SNAPIN
A_24_P16950	7.450178	7.6330523	NM_198893	chr19:53571722-53571663	ZNF160
A_23_P17307	9.679346	9.8649435	NM_018270	chr20:61431334-61431393	MRGBP
A_33_P3741059	6.4395947	5.9509335	AL832882	chr1:117035719-117035660	LOC100506459
A_24_P4054	10.108088	9.945501	NM_003302	chr7:100470307-100470366	TRIP6
A_23_P165090	10.071898	10.183749	NM_024407	chr19:1393247-1393306	NDUFS7
A_33_P3308914	5.6398215	5.934943	NM_006383	chr15:78397052-78396993	CIB2
A_33_P3266550	4.948896	5.0266085	NM_020469	chr9:136130807-136130748	ABO
A_23_P86550	13.109162	13.083107	NM_003750	chr10:120795387-120795328	EIF3A
A_24_P307854	7.3951645	7.021453	NM_024963	chr7:5520556-5520497	FBXL18
A_24_P626931	7.358249	7.060481	NM_198920	chr6:83602508-83602449	UBE3D
A_32_P66020	5.4458346	5.559658	NM_032167	chr16:12142258-12142317	SNX29
A_23_P2725	15.339501	15.2583475	NM_000982	chr13:27829410-27829469	RPL21
A_24_P402690	11.467915	11.741805	NM_030926	chr2:231743882-231743941	ITM2C
A_23_P149946	3.7959723	3.553961	NM_033100	chr10:85976380-85976439	CDHR1
A_23_P109470	5.393713	5.3103876	NM_001002878	chr22:29916082-29916023	THOC5
A_24_P80776	6.551169	6.2332783	NM_001135575	chr6:11138043-11138102	SMIM13
A_33_P3361513	5.7848825	5.308656	NM_001014445	chr17:33462341-33462282	NLE1
A_23_P386168	5.4621897	5.7496853	NM_178511	chr19:47778664-47778723	PRR24
A_33_P3841368	5.810209	5.6996655	AK091672	chr8:427881-427940	
A_33_P3232677	3.6734934	2.3900566		chr22:16376018-16376077	
A_24_P44514	9.426797	9.438384	NM_006384	chr15:90774611-90774409	CIB1
A_33_P3398862	6.6747675	6.7673273	NM_004040	chr2:20648584-20648643	RHOB
A_33_P3373403	4.708026	4.679044	NM_016274	chr1:150129586-150129645	PLEKHO1
A_33_P3255949	9.365772	9.796334	NM_001113756	chr22:50964244-50964185	TYMP
A_24_P298877	9.8533535	9.998257	NM_207356	chr1:3805912-3805853	C1orf174
A_33_P3412847	6.3548594	6.1617103	XR_242709	chr10:3975104-3975163	LOC101927904
A_23_P345460	9.051731	8.588668	NM_015432	chr16:67323234-67323293	PLEKHG4
A_23_P432034	6.5446835	6.774651	NM_173510	chr22:29184752-29184811	CCDC117
A_23_P323685	9.240551	9.084924	NM_003543	chr6:26285556-26285497	HIST1H4H
A_33_P3329013	3.061149	3.0211544	NM_001050	chr17:71166803-71166862	SSTR2
A_33_P3240966	4.1552763	5.2305193		chr3:156432072-156432013	
A_33_P3420402	6.3552938	6.652808	NM_020771	chr6:105177223-105177164	HACE1
A_33_P3243600	3.9551046	3.8122566		chr7:131812594-131812653	
A_33_P3366073	8.184699	7.950395	NR_103802	chr13:50260701-50260642	EBPL
A_23_P98167	5.0764017	5.0371723	NM_030930	chr11:67758982-67758923	UNC93B1
A_24_P38276	9.939985	10.490665	NM_003505	chr7:90897840-90897899	FZD1
A_33_P3318596	4.4120064	4.5194564	NM_003419	chr19:37369023-37369082	ZNF345
A_23_P218068	8.867144	8.915782	NM_019012	chr12:19529106-19529165	PLEKHA5
A_23_P71328	4.3342905	4.817546	NM_030583	chr8:99042755-99042814	MATN2
A_24_P273799	5.458212	5.3516397	NM_152320	chr12:48734400-48734341	ZNF641
A_24_P241815	4.639652	4.730604	NM_002229	chr19:12902998-12903057	JUNB
A_33_P3376365	5.685911	5.754383	BC012091	chr1:6473312-6473253	HES2
A_23_P253158	6.166326	6.3142214	NM_015409	chr12:132564396-132564455	EP400
A_23_P76557	11.202077	11.323172	NM_018838	chr12:95388011-95387952	NDUFA12
A_33_P3402020	6.275365	6.495709	NM_032040	chr19:46914650-46914591	CCDC8
A_23_P89460	10.412121	10.23758	NM_012138	chr17:35378303-35388959	AATF
A_24_P387321	6.6774325	6.8828745	NM_016264	chr19:12383626-12383567	ZNF44
A_23_P103201	9.845592	10.792545	NM_017761	chr1:24289291-24289349	PNRC2
A_33_P3314161	5.7761087	5.671688	NM_001190979	chr12:42550965-42550906	YAF2
A_23_P118427	6.5940485	6.22188	NM_145109	chr17:21217988-21218047	MAP2K3
A_23_P54447	5.336041	5.0823	NR_026813	chr15:77516360-77516301	LINC00597

A_33_P3225507	5.537757	5.852559	NM_001005466	chr14:22102159-22102100	OR10G2
A_33_P3419567	4.3954296	4.2977304	NM_001136566	chr20:1223430-1223489	RAD21L1
A_32_P190488	15.777722	15.709674		chr14:102144580-102144639	XLOC_014512
A_32_P83465	12.855014	12.855895	NM_001039703	chr1:145313371-145313430	NBPF10
A_33_P3605269	4.0011153	4.3923836	NR_027713	chr11:9117250-9117309	KRT8P41
A_23_P75647	5.3850813	5.3075223	NM_006389	chr11:118915856-118915797	HYOU1
A_23_P166438	4.3563113	4.23112	NM_030642	chr22:36125425-36125484	APOL5
A_32_P315395	5.1759424	5.189941	NR_026914	chr17:72206943-72206884	MGC16275
A_33_P3294392	10.486401	10.363201	NM_014329	chr16:67918296-67918355	EDC4
A_24_P56240	7.974578	7.7530303	NM_153634	chr12:39046654-39046595	CPNE8
A_23_P33720	5.861654	5.998763	NM_006567	chr6:5431290-5431349	FARS2
A_23_P201687	6.208288	6.3260336	BC012091	chr1:6473192-6473133	HES2
A_33_P3330991	7.069086	7.0364676	XR_248772	chr1:32012152-32012211	LOC100134237
A_24_P395621	7.003983	6.949849	NM_173079	chr17:41145573-41145632	RUNDC1
A_32_P4018	7.9957533	8.464094	NM_005012	chr1:64646792-64646851	ROR1
A_33_P3321150	10.641827	10.69131	NM_003590	chr2:225338736-225338677	CUL3
A_23_P43898	4.5597486	4.025857	NM_173567	chr1:92518130-92518189	EPHX4
A_33_P3402056	4.2113853	4.6356325	NM_001242480	chr7_gl000195_random:44806-44747	LOC389831
A_23_P96325	7.2960606	7.6810412	NM_017669	chrX:71425169-71425110	ERCC6L
A_23_P38618	6.801427	6.4414644	NM_004278	chr17:16229446-16229505	PIGL
A_33_P3213082	3.9896045	3.9251919	XR_248956	chr20:52191519-52191578	
A_23_P26117	4.237641	4.6894593	NM_006715	chr15:75650581-75649224	MAN2C1
A_32_P206839	9.637447	9.448561	NR_037631	chr2:36581977-36581918	LOC100288911
A_23_P111092	8.967831	9.376506	NM_030883	chr6:29430022-29430081	OR2H1
A_33_P3290577	3.568415	2.8586078	NM_001261828	chr11:60183684-60183743	MS4A14
A_24_P115199	6.1068363	6.0629816	NM_001037165	chr7:4810399-4810458	FOXK1
A_24_P47182	10.892973	11.184292	NM_014000	chr10:75879227-75879286	VCL
A_24_P152527	5.8601484	5.895414	NM_006373	chr17:41170689-41170630	VAT1
A_23_P74581	10.254912	10.069078	NR_024127	chr1:28906090-28905179	SNHG12
A_23_P89601	4.4373007	4.177886	NM_002278	chr17:39616163-39616104	KRT32
A_23_P57868	7.2177043	7.014399	NM_000666	chr3:52022979-52023038	ACY1
A_24_P925635	4.709425	4.376174	NR_024271	chr7:45763450-45763391	SEPT7P2
A_24_P118231	7.489407	7.463497	NM_001003803	chr14:50790809-50792360	ATP5S
A_23_P151133	4.6756554	4.574752	NM_006675	chr12:3394900-3394959	TSPAN9
A_23_P37514	6.387122	5.6898937	NM_015492	chr15:75504433-75504492	C15orf39
A_33_P3312384	2.3221061	3.4570017	NR_027790	chr21:17979483-17979542	LINC00478
A_32_P105549	4.5947	4.238879	NM_001630	chr10:47762959-47763018	ANXA8L2
A_24_P176079	6.7036715	6.8753924	NM_006646	chr13:27262694-27262753	WASF3
A_23_P152727	10.148418	9.814165	NM_014798	chr17:43513358-43513299	PLEKHM1
A_33_P3209055	4.4318576	4.1867557		chr10:094833164-094833223	
A_24_P10137	6.666066	5.8646607	NM_014059	chr13:42042937-42044635	RGCC
A_23_P79911	8.549923	8.643208	NM_006814	chr20:1147718-1147777	PSMF1
A_33_P3304878	4.7505126	4.843958	NM_020945	chr10:50030522-50030581	WDFY4
A_33_P3369190	7.3297386	8.090094	NM_001177306	chr5:102360970-102361029	PAM
A_33_P3347099	4.0493164	4.167954		chr17:21909868-21909927	FLJ36000
A_23_P159671	7.477108	7.267369	NM_000292	chrX:18911319-18911260	PHKA2
A_33_P3318861	6.086866	6.338186	NM_001093730	chr2:207516408-207516349	DYTN
A_23_P412603	3.9128253	3.4671736	NM_144773	chr20:5283328-5283269	PROKR2
A_32_P479743	4.0168524	4.4803886		chr15:70135248-70135306	LINC00593
A_23_P88963	12.262457	12.4784975	NM_000034	chr16:30081324-30081470	ALDOA
A_24_P407323	4.914299	5.288432	NM_177951	chr14:60750198-60752343	PPM1A
A_24_P222684	10.360087	10.189404	NM_001042369	chr1:193053711-193053770	TROVE2
A_33_P3350413	5.34956	5.459754	AK310272	chr10:29769151-29769092	SVIL
A_33_P3256550	5.623542	4.504409	AF190155	chr6:151328182-151328241	MST152
A_33_P3301955	5.8610554	5.6086283	NM_001242318	chr8:66635836-66635777	PDE7A
A_33_P3212188	8.1664915	7.401044	NM_002333	chr19:33699713-33699772	LRP3
A_24_P410516	10.434531	10.391012	NM_198181	chr15:82728575-82728634	GOLGA6L9
A_24_P48791	5.4913054	5.058312	NM_003908	chr20:32676707-32676648	EIF2S2

A_23_P411953	7.627781	7.513136	NM_138300	chr1:154929734-154929675	PYGO2
A_33_P3369979	3.9714642	3.0775893	NM_001004686	chr1:248202548-248202607	OR2L2
A_33_P3338186	9.074278	8.931357	NM_173620	chr17:80400456-80400515	HEXDC
A_33_P3359413	8.272902	8.315836	NM_003021	chr19:2755165-2755106	SGTA
A_33_P3387155	5.7039576	5.3828654	NR_046444	chr12:9810936-9810995	LOC374443
A_24_P65616	6.342806	6.078577	NM_006505	chr19:45166078-45166137	PVR
A_24_P943815	2.3221061	2.3900566	NM_004650	chrX:7867118-7867059	PNPLA4
A_23_P54736	5.343473	5.420381	NM_016541	chr16:848114-848055	GNG13
A_23_P75973	6.3108344	6.1717014	NM_018320	chr11:71708267-71708326	RNF121
A_33_P3473108	4.448333	4.5897827	Z39353	chr1:93306531-93306590	
A_23_P149690	5.5140705	5.159072	NM_018216	chr1:2442189-2442130	PANK4
A_33_P3348529	5.0838223	5.0109844	AK130848	chr2:135039705-135039764	
A_33_P3384845	4.043867	3.6842546	NM_172236	chr20:30805666-30805725	POFUT1
A_33_P3418091	5.226054	4.6189737	NM_001130136	chr15:74428410-74428469	ISLR2
A_23_P155052	3.210587	3.3714979	NM_030641	chr22:36055129-36055188	APOL6
A_33_P3241081	5.344797	5.12359	NM_152763	chr1:109366052-109365993	AKNAD1
A_23_P2573	7.131194	7.0859704	NM_032256	chr12:44783288-44783347	TMEM117
A_23_P377214	7.9326887	7.8581486	NM_144608	chr17:43247340-43247399	HEXIM2
A_33_P3342663	12.318008	12.798718	NM_153202	chr20:3654771-3654712	ADAM33
A_23_P259172	11.890519	11.964029	NM_006280	chrX:153063256-153063571	SSR4
A_23_P106562	4.699743	4.2893443	NM_000512	chr16:88884429-88880889	GALNS
A_24_P291401	3.584369	3.4134204	NM_001031738	chr2:85826387-85826328	TMEM150A
A_33_P3225487	5.917074	5.8407307	NM_016371	chr1:162769658-162769717	HSD17B7
A_23_P209740	12.282138	11.972696	NM_002807	chr2:232037369-232037428	PSMD1
A_33_P3604591	5.2427177	5.448702	AI271839	chr16:2015500-2015441	SNORA78
A_24_P250499	8.42725	8.348719	NM_021215	chr20:36720305-36720364	RPRD1B
A_33_P3269453	8.00945	8.064218	XM_005257152	chr17:65955936-65959450	BPTF
A_23_P37994	3.9443853	4.4985447	NM_016212	chr16:33264020-33264079	TP53TG3
A_23_P158829	6.4261446	6.474434	NM_004313	chr17:4624528-4624587	ARRB2
A_23_P218793	6.1896586	6.1781034	NM_022098	chr22:41322769-41322828	XPNPEP3
A_33_P3315929	6.4471254	6.543825	NM_001244897	chr9:114986473-114986414	PTBP3
A_33_P3585268	12.712016	13.038521	NM_001282619	chr3:50296258-50296317	GNAI2
A_23_P98248	8.159233	8.407054	NM_001033678	chr11:63991675-63991616	TRPT1
A_24_P361158	5.9976482	6.063142	NM_018335	chr14:102808370-102808429	ZNF839
A_33_P3316118	3.8670735	3.640665	NM_001256717	chr6:84262672-84262613	SNAP91
A_23_P71073	4.697144	4.734852	NM_000474	chr7:19155704-19155645	TWIST1
A_23_P96599	8.371536	8.655924	NM_194324	chrX:103220407-103220466	TMSB15B
A_23_P250358	4.00987	3.8849473	NM_017912	chr4:89363667-89363726	HERC6
A_33_P3369239	6.834518	6.719476	NM_006775	chr6:163836292-163836351	QKI
A_33_P3248842	4.8956685	4.8580008	NM_001251874	chr6:74019393-74019334	KHDC1
A_23_P23438	5.9204025	6.0492525	NM_022367	chr1:156146532-156146591	SEMA4A
A_33_P3300158	3.5954995	3.33468	AK094659	chr17:79053060-79053119	LOC100130078
A_23_P96641	7.2275124	7.413887	NM_001039091	chrX:12841243-12841302	PRPS2
A_24_P320545	5.6267347	5.576661	NM_002821	chr6:43128858-43128917	PTK7
A_24_P331655	8.854466	8.900556	NM_001040715	chr16:67209673-67209614	KIAA0895L
A_32_P132438	9.975337	9.788155	NM_003488	chr17:55198512-55198571	AKAP1
A_33_P3317412	3.8455548	3.4411502	NR_003594	chr8:86775231-86775172	REXO1L2P
A_33_P3264331	3.6966443	3.493563	NM_012183	chr1:63790602-63790661	FOXD3
A_33_P3419485	2.3221061	2.3900566	BX106402	chr8:94242585-94242644	
A_33_P3352544	6.8059673	6.8637295	NM_005469	chr20:44472981-44472922	ACOT8
A_23_P141555	2.3221061	2.3900566	NM_013351	chr17:45823389-45823448	TBX21
A_33_P3340769	6.9051113	6.072667	NM_003816	chr8:38962718-38962777	ADAM9
A_33_P3267081	9.454779	9.444198	NM_016643	chr16:30429856-30429915	ZNF771
A_33_P3320888	5.4914503	5.46398	NM_001253775	chr7:137600640-137597823	CREB3L2
A_33_P3362046	5.4814796	5.475148		chr17:018251645-018251586	
A_33_P3334843	4.1820617	3.7420728	AF318333	chr3:171510199-171510140	
A_33_P3226129	5.7937055	5.829858		chr1:001605816-001605875	
A_33_P3235048	5.2871056	3.4040513	NM_001146162	chr11:89450972-89451031	TRIM77
A_23_P91850	11.004181	10.838022	NM_144717	chr3:136729798-136729857	IL20RB

A_23_P401700	4.4749575	4.637734	NM_019043	chr10:26856519-26856578	APBB1IP
A_24_P753476	9.669206	9.510548		chr9:99838292-99838233	LOC340508
A_32_P116206	9.013919	8.326187	NM_001085400	chr4:37612341-37612282	RELL1
A_23_P112260	9.996573	9.753205	NM_001017998	chr9:114429198-114431579	GNG10
A_32_P191084	7.1273365	7.238969	NM_018992	chr16:2758645-2758704	KCTD5
A_24_P200848	7.46299	7.1102037	NM_015132	chr7:17831097-17831038	SNX13
A_33_P3249002	11.242524	11.103252		chr2:109868129-109868188	
A_23_P6464	6.7963	6.921363	NM_003560	chr22:38507934-38507875	PLA2G6
A_23_P123916	4.472479	4.609005	NM_138361	chr9:130263382-130265071	LRSAM1
A_23_P416751	3.1075723	2.3900566	NM_173530	chr19:52870021-52870080	ZNF610
A_23_P215751	9.833323	9.948254	NM_005000	chr7:123181228-123181169	NDUFA5
A_23_P100764	7.79833	7.7185016	NM_016016	chr17:42397438-42397379	SLC25A39
A_33_P3338423	5.838744	6.036212	NM_030964	chr5:141694546-141694487	SPRY4
A_33_P3329652	8.44137	8.684972	NM_133458	chr16:68600971-68601030	ZFP90
A_33_P3335022	4.903976	4.596987	XM_005263996	chr2:98123523-98123464	ANKRD36B
A_24_P103060	7.643187	7.446048	NM_139279	chr2:47130141-47130082	MCFD2
A_33_P3805090	8.74052	9.130964	NM_020840	chr4:159826766-159826825	FNIP2
A_33_P3277943	5.0281353	5.2778354	NM_001286790	chr4:78640418-78640364	CNOT6L
A_33_P3308740	6.0160437	5.678837	NM_006267	chr2:109379969-109380028	RANBP2
A_24_P318967	9.147419	9.169774	NM_003681	chr21:45173586-45175623	PDXK
A_23_P218505	7.9246955	8.404892	NM_000894	chr19:49519464-49519405	LHB
A_33_P3221064	11.534663	11.139745	NM_001042544	chr19:41135661-41135720	LTBP4
A_33_P3229032	11.771183	11.807949	NM_002975	chr19:51228911-51228970	CLEC11A
A_23_P68472	10.956941	10.91374	NM_003859	chr20:49552695-49551725	DPM1
A_33_P3294868	5.1963873	5.049652		chrX:134556222-134556281	LINC00086
A_24_P88696	2.3221061	2.3900566	NM_003469	chr2:224462528-224462469	SCG2
A_33_P3705884	7.493224	7.1186676		chr19:28221266-28221207	LINC00662
A_33_P3361398	4.534425	4.4686813	XM_005259528	chr19:1229297-1229238	C19orf26
A_33_P3335124	14.311911	14.424332	NM_002952	chr16:2014321-2013243	RPS2
A_23_P134433	8.89252	8.990126	NM_001427	chr7:155257437-155257496	EN2
A_33_P3214310	5.367937	5.284042	NM_001012505	chr3:71247097-71247038	FOXP1
A_24_P172990	7.5247245	7.2490845	NM_001605	chr16:70287935-70287876	AARS
A_23_P216622	7.4420834	7.309798	NM_001079802	chr9:108402444-108402503	FKTN
A_33_P3264593	2.4769006	2.3900566	NM_016249	chrX:141290861-141290802	MAGEC2
A_33_P3307875	8.309734	8.5271225	NM_138288	chr14:34902204-34902145	SPTSSA
A_23_P258612	3.387938	3.5959697	NM_016529	chr13:26594329-26594388	ATP8A2
A_23_P203658	6.1098924	6.3530445	NM_007166	chr11:85692268-85692209	PICALM
A_24_P347378	3.0889308	3.0382233	NM_001629	chr13:31338155-31338214	ALOX5AP
A_23_P72697	4.19468	4.695286	NM_178172	chr8:144298966-144299025	GPIHBP1
A_33_P3257683	5.0485096	4.847164		chr5:095551237-095551296	
A_33_P3336262	4.22773	4.1961727	XM_005244811	chr1:1853767-1853708	KIAA1751
A_23_P500282	6.7575736	6.982904	NM_018026	chr11:66012003-66012062	PACS1
A_33_P3349299	4.0887823	4.2440705	NR_046101	chrX:46746920-46746861	CXorf31
A_33_P3218559	5.315427	5.395881	XR_243695	chr17:36607637-36607578	
A_23_P44139	8.479977	7.9694653	NM_000947	chr6:57513196-57513255	PRIM2
A_24_P113221	9.169614	9.28005	NM_006965	chr18:32920195-32919882	ZNF24
A_33_P3412149	7.6986217	8.035044	NM_001048210	chr1:109477444-109477385	CLCC1
A_33_P3364112	7.107771	6.9319887	NM_001278351	chr12:69973500-69973559	FRS2
A_32_P200237	6.644926	6.78486	AJ291676	chr8:11141291-11141232	LOC157740
A_24_P397903	7.456907	7.1729655	NM_016010	chr8:79631797-79631856	ZC2HC1A
A_23_P87964	10.679169	10.731542	NM_001984	chr13:47354114-47351744	ESD
A_33_P3214339	3.6325693	2.923497	NM_153268	chr3:111432833-111432892	PLCXD2
A_33_P3406998	5.207967	5.251264	NM_130438	chr21:38852987-38853046	DYRK1A
A_23_P85777	9.782245	9.965059	NM_014236	chr1:231411050-231411193	GNPAT
A_23_P46627	9.647649	9.775462	NM_015999	chr1:202910325-202910266	ADIPOR1
A_23_P417261	3.955616	4.863826	NM_144715	chr3:19921032-19920973	EFHB
A_33_P3290748	4.6918535	3.8821824	XM_003959952	chr19:12799127-12799186	LOC648044
A_33_P3282364	4.325863	3.5660567	XR_253010	chr12:7282164-7282105	
A_33_P3414789	8.006728	8.111695	NM_024333	chr19:4323373-4323432	FSD1

A_23_P68884	9.736049	9.648439	NM_004147	chr22:31819356-31822619	DRG1
A_24_P389285	4.4695277	4.75111	NR_027330	chr7:127637816-127637875	SND1-IT1
A_33_P3331641	6.996868	6.593056	NR_024421	chr10:77168623-77168682	ZNF503-AS2
A_23_P27724	10.7584715	10.775003	NM_003009	chr19:48287827-48287886	SEPW1
A_32_P144421	6.737535	6.761148	NM_053042	chr4:10441743-10441684	ZNF518B
A_24_P251534	8.355615	8.030168	NM_001008392	chr3:38025822-38025881	CTDSPL
A_23_P214638	6.014383	5.755817	NM_006709	chr6:31848533-31848474	EHMT2
A_33_P3299436	15.723595	15.608545		chr1:234492472-234492413	
A_32_P151823	8.346319	7.886476	BC040619	chr8:107284866-107284925	
A_33_P3242323	4.8987794	4.8179293		chr1:229749969-229749910	TAF5L
A_33_P3225273	3.7767878	3.3411455	NM_001004128	chr1:180155199-180155258	QSOX1
A_33_P3663142	4.432361	4.420047		chr2:20084652-20084711	LINC00954
A_33_P3362521	8.070779	8.086	NM_016474	chr3:14714080-14714139	CCDC174
A_23_P120002	8.319315	8.496407	NM_004510	chr2:231042320-231042261	SP110
A_23_P126416	2.3221061	2.3900566	NM_005424	chr1:43788328-43788387	TIE1
A_23_P315991	5.7717953	6.0182095	NM_178168	chr11:6866954-6867013	OR10A5
A_33_P3216933	4.18544	3.9706843	NM_015191	chr11:111597570-111597629	SIK2
A_33_P3608172	10.846741	11.05041	NM_004489	chr17:7216078-7216019	GPS2
A_33_P3261869	3.9853945	4.219212	NM_001286680	chr8:21894337-21894396	NPM2
A_23_P501538	7.56318	7.4478745	NM_153631	chr7:27146830-27146771	HOXA3
A_33_P3335576	5.3875523	5.4666405		chr7:076751624-076751683	
A_33_P3406110	4.7392197	4.3114743		chr2:242749345-242749286	
A_33_P3272593	8.621122	8.5616	NM_001144002	chrX:16606196-16606137	CTPS2
A_23_P370434	13.395286	13.1391535	NM_001212	chr17:5336307-5336248	C1QBP
A_23_P126803	10.86965	11.231499	NM_005717	chr1:183596590-183596531	ARPC5
A_33_P3230818	4.0420732	3.9490328	NM_001251973	chr6:46424578-46424519	RCAN2
A_23_P29655	9.457976	9.318546	NM_020685	chr3:62319086-62319145	C3orf14
A_33_P3265920	5.8515964	5.8712783	NM_002830	chr2:120734977-120735036	PTPN4
A_23_P91250	2.3221061	2.3900566	NM_001270497	chr20:45129999-45129940	ZNF334
A_33_P3241591	4.1072693	3.847057	AK094219	chr1:6237575-6237516	LOC100131864
A_33_P3324428	5.1697025	5.4567804	NM_003488	chr17:55184479-55184538	AKAP1
A_33_P3321923	12.233913	12.364962		chr15:064821362-064821303	
A_33_P3363186	6.196987	5.984374		chr13:114540940-114540999	
A_23_P65558	10.103716	10.152378	NM_002408	chr14:50089817-50089876	MGAT2
A_33_P3255544	7.965808	8.453723	NM_001280801	chr5:179397470-179397411	RNF130
A_23_P346813	4.955061	5.368075	NM_002578	chrX:110439104-110439163	PAK3
A_23_P14673	2.4913375	2.3900566	NM_020962	chr15:65674038-65673979	IGDCC4
A_24_P89413	7.712366	7.62333	NM_021253	chr6:30311274-30311333	TRIM39
A_33_P3390391	8.25147	8.316685	NM_015711	chr19:48206465-48206524	GLTSCR1
A_23_P131646	9.283024	9.328993	NM_144563	chr2:89050202-89050261	RPIA
A_23_P315212	4.0988445	3.7117038	NM_012344	chr2:11798629-11798570	NTSR2
A_23_P211631	9.778433	10.086377	NM_006486	chr22:45996933-45996992	FBLN1
A_33_P3283480	7.7384562	6.5080175	NM_148170	chr11:88054087-88054028	CTSC
A_33_P3325704	5.3538465	5.2499743	NM_001024209	chr1:153065731-153065672	SPRR2E
A_23_P311740	8.181284	8.051053	NM_015089	chr6:43192245-43192304	CUL9
A_32_P24581	14.937944	14.342569	NM_002954	chr2:55462018-55462077	RPS27A
A_33_P3340205	5.5741463	5.53147	NM_001084393	chr22:24314533-24314592	DDTL
A_23_P218988	9.194231	9.32348	NM_022902	chr5:68425352-68425411	SLC30A5
A_33_P3293266	8.161865	8.183525	NM_032326	chr4:952382-952441	TMEM175
A_33_P3261648	6.884194	6.8479967	NM_025190	chr2:98206048-98205989	ANKRD36B
A_23_P207020	11.215775	11.141994	NM_019613	chr17:80572621-80572562	WDR45B
A_24_P320526	11.463805	11.7054615	NM_024294	chr6:34556043-34555984	C6orf106
A_32_P77098	5.6032095	4.991724	NM_001003682	chr1:29446697-29446638	TMEM200B
A_33_P3369550	12.105285	12.032792	NM_004768	chr1:70716305-70716364	SRSF11
A_24_P245298	9.324963	9.485204	NM_003809	chr17:7461108-7461167	TNFSF12
A_23_P25868	6.826354	6.776064	NM_001011713	chr14:57879042-57879101	NAA30
A_23_P368934	7.586919	7.2219963	NM_001142641	chr12:133161698-133161757	FBRSL1
A_23_P140328	10.102524	9.959128	NM_004713	chr14:50250771-50250712	NEMF
A_23_P86943	7.6898403	8.076642	NM_003139	chr11:126133248-126133189	SRPR

A_32_P144908	11.629008	12.020133	NM_203282	chr19:24309844-24309903	ZNF254
A_33_P3256902	8.792507	8.506923		chr11:67371010-67370951	
A_33_P3365815	3.7553804	3.6350033	AK057458	chr1:157895235-157895176	LOC729866
A_24_P214231	6.936035	7.2246337	NM_001048166	chr1:47716850-47716791	STIL
A_23_P26865	4.0434303	3.1918437	NM_002470	chr17:10532942-10531966	MYH3
A_23_P104692	7.965057	8.251606	NM_145065	chr11:66244673-66244732	PELI3
A_33_P3385387	9.93774	9.857632		chr16:76269320-76269261	XLOC_014512
A_23_P48747	6.446024	6.7479177	NM_138452	chr14:24760771-24760376	DHRS1
A_23_P132378	7.474199	7.6614494	NM_014246	chr22:46757506-46757447	CELSR1
A_33_P3402817	4.9229097	4.8374195	NM_001145148	chr17:43907876-43907935	CRHR1
A_24_P175612	6.425431	6.2195454	NM_178858	chr10:104498374-104498433	SFXN2
A_33_P3244803	9.515749	8.988998	NM_001185039	chr17:73937674-73937615	ACOX1
A_23_P250735	6.379889	6.726369	NM_175709	chr22:39527059-39527000	CBX7
A_23_P23380	7.891637	7.828793	NM_022821	chr1:43829796-43829737	ELOVL1
A_23_P80817	3.598788	4.23094	NM_013259	chr3:111719676-111719735	TAGLN3
A_33_P3227324	9.223946	9.516384	NM_017550	chr19:305635-305576	MIER2
A_23_P52121	8.155733	9.0135565	NM_002614	chr1:145763717-145763776	PDZK1
A_33_P3567967	13.364828	13.287257	AK023417	chr5:86511671-86511612	FLJ11292
A_24_P329924	8.018131	8.174527	NM_025164	chr11:116714247-116714188	SIK3
A_24_P303097	6.59822	6.9309006	NM_031953	chr4:186284717-186284776	SNX25
A_23_P251548	6.719476	6.5014324	NM_144582	chr2:71213585-71213526	TEX261
A_33_P3672482	5.4335537	5.8453474	NM_020886	chr11:113669639-113669580	USP28
A_24_P826348	5.325279	5.250227	NM_198581	chr2:113097441-113097500	ZC3H6
A_24_P27229	4.413885	3.912629	NM_015989	chr12:53553708-53553475	CSAD
A_23_P127652	4.6338997	4.2247343	NM_003455	chr11:123595399-123595340	ZNF202
A_32_P226786	7.1793327	6.810337	NM_173822	chr2:201838745-201838686	FAM126B
A_23_P142239	6.3197737	5.1665487	NM_033557	chr19:38794313-38794254	YIF1B
A_23_P142322	9.964607	10.298027	NM_001280	chr19:1272456-1272515	CIRBP
A_33_P3238651	3.9146872	3.8774645	NM_001039783	chr3:126290860-126290801	TXNRD3NB
A_23_P255376	8.807974	8.439071	NM_017918	chr4:110605745-110606408	CCDC109B
A_23_P99424	8.389629	8.495739	NM_003291	chr13:103330625-103330684	TPP2
A_33_P3288700	7.067952	6.953741	NM_058190	chr21:46363730-46380051	FAM207A
A_33_P3289251	8.233047	8.067129	AK095971	chr8:53628310-53628369	LOC644727
A_24_P282578	9.778924	9.9830475	NM_003145	chr1:155988133-155988074	SSR2
A_33_P3317589	4.5640965	4.6242495	NM_145762	chr20:3640070-3640011	GFRA4
A_33_P3500167	4.0440664	4.58597	NM_001135	chr15:89346980-89347039	ACAN
A_33_P3217804	9.145502	9.035399		chr9:138709692-138709634	CAMSAP1
A_33_P3225873	6.9637947	7.2477465	NR_028324	chr19:197903-197844	LINC01002
A_24_P350228	7.1406965	7.39962	NM_021945	chr6:3410447-3324203	SLC22A23
A_23_P348257	9.220297	9.132524	NM_014840	chr12:106457605-106457546	NUAK1
A_33_P3379377	5.6874986	5.570482	NM_004990	chr12:57884057-57884116	MARS
A_23_P6413	8.947869	9.165245	NM_080430	chr22:31500922-31500863	SELM
A_23_P155477	8.939194	8.734075	NM_016210	chr3:50595542-50595483	C3orf18
A_24_P276791	6.0150747	6.2141004	NM_052940	chr1:54426075-54426134	LRRC42
A_23_P374767	7.41749	7.6856227	NM_015983	chr7:43992881-43992940	UBE2D4
A_33_P3659808	11.455188	11.477748	NM_014389	chr17:4574747-4574688	PELP1
A_33_P3330911	2.586443	2.3900566	NM_003657	chr20:52560576-52560517	BCAS1
A_33_P3357421	4.407875	4.239761	XM_005275767	chr16:1311386-1311327	
A_32_P92505	9.641163	9.589367	NM_182551	chr2:30867018-30867077	LCLAT1
A_33_P3248615	6.752825	6.810757		chr7:008021908-008021967	
A_23_P422071	6.1787486	6.793053	NM_003782	chr6:033246543-033246601	B3GALT4
A_33_P3222203	5.679678	6.0368824	NM_148962	chr2:42989702-42989643	OXER1
A_33_P3377364	4.610823	4.749814	NM_000213	chr17:73753824-73753883	ITGB4
A_33_P3271480	6.7938485	6.3108344	NR_026984	chr2:86247401-86247342	LOC90784
A_33_P3243657	9.018101	9.04498	NM_012192	chr11:6505851-6505910	TIMM10B
A_23_P82334	7.7109704	8.289227	NM_014251	chr7:95749948-95749889	SLC25A13
A_23_P130304	11.73415	10.9747305	NM_006701	chr18:77733605-77733546	TXNL4A
A_23_P157215	10.460293	10.260192	NM_014038	chr7:16746044-16746103	BZW2
A_24_P21887	5.6170397	5.803716	NM_014638	chr1:2436148-2436207	PLCH2

A_33_P3395422	3.8999164	3.7022896		chr10:007455380-007455439	
A_33_P3279920	6.856087	7.2537518	NM_007375	chr1:11082247-11082306	TARDBP
A_23_P79622	8.332292	8.51833	NM_181342	chr2:179330565-179330506	FKBP7
A_23_P156748	8.261554	8.060977	NM_015245	chr6:35058849-35058908	ANKS1A
A_23_P29303	6.1238475	5.717223	NM_015703	chr22:42915753-42914128	RRP7A
A_33_P3860715	4.124735	3.896419	AK128002	chr3:154955625-154955566	FLJ46120
A_24_P98914	10.556138	10.391334	NM_000289	chr12:48539724-48539783	PFKM
A_33_P3412900	6.0518484	6.1231127	NM_001039771	chr14:24895813-24895754	CBLN3
A_33_P3221458	2.449361	2.3900566	NR_002722	chr6:27325667-27325608	ZNF204P
A_23_P127460	6.7792616	7.003581	NM_153253	chr11:65417650-65417917	SIPA1
A_24_P97836	4.74247	4.6380124	NM_002873	chr5:68695972-68706354	RAD17
A_24_P14010	11.227951	10.818123	NM_021079	chr17:43186308-43186367	NMT1
A_23_P150018	8.633803	8.895091	NM_004419	chr10:112270900-112270959	DUSP5
A_23_P214882	6.885841	6.781677	NM_019041	chr6:153310633-153310574	MTRF1L
A_33_P3307980	6.652171	6.5338	AK131345	chr7:92319353-92319294	
A_24_P192994	10.442104	10.242506	NM_013402	chr11:61567185-61567126	FADS1
A_23_P388855	6.163325	6.458615	NM_012330	chr10:76790506-76790565	KAT6B
A_32_P193822	6.6596212	6.8027005	NM_014827	chr1:203797464-203797523	ZC3H11A
A_33_P3535523	3.5291696	3.0536003	BC015720	chr8:49340900-49340841	LOC286068
A_33_P3269650	4.654667	4.83928	AK124574	chr12:8490503-8490562	LOC100128402
A_23_P92994	10.015118	9.994153	NM_020194	chr2:228222249-228222308	MFF
A_33_P3300267	5.6361036	5.5993214	NM_053276	chr2:37041517-37041576	VIT
A_33_P3393412	3.3561711	3.6849465	NM_001001480	chr11:1651498-1651614	KRTAP5-5
A_23_P365719	8.739548	8.803267	NM_003190	chr6:33267777-33267718	TAPBP
A_23_P165360	6.646076	6.855128	NM_001040445	chr2:239353338-239355053	ASB1
A_23_P136504	3.0114553	4.374866	NM_030631	chr14:37149829-37149770	SLC25A21
A_23_P408271	7.9656906	8.443119	NM_016245	chr4:88258426-88258367	HSD17B11
A_33_P3268403	3.6835792	3.707089	AK126139	chr1:209950527-209950586	TRAF3IP3
A_24_P284523	7.537962	7.198926	NM_002446	chr19:40721403-40721462	MAP3K10
A_33_P3287716	4.7516623	5.0344224	AK128048	chr20:43094995-43095054	LOC100129292
A_23_P57667	10.830881	10.440378	NM_032242	chr3:126756142-126756201	PLXNA1
A_33_P3403748	10.980482	10.965921	NM_001085372	chr11:62439789-62439848	C11orf83
A_32_P197489	7.3313723	7.5647116	NM_015995	chr15:31669908-31669967	KLF13
A_23_P117683	12.212606	12.109067	NM_016400	chr15:44093979-44094038	HYPK
A_33_P3284004	4.1265516	2.3900566	NR_027249	chr12:104250976-104250917	GNN
A_23_P94860	10.141388	10.106107	NM_024047	chr4:88379293-88379352	NUDT9
A_33_P3303810	5.6757274	5.5803595	NM_005558	chr1:201350401-201350342	LAD1
A_23_P58983	4.9361567	4.6912065	NM_017772	chr6:37300191-37300250	TBC1D22B
A_23_P328545	4.0333548	3.7591276	NM_014211	chr5:170240453-170240512	GABRP
A_24_P239731	9.190092	9.629495	NM_004776	chr20:48250296-48250237	B4GALT5
A_23_P429535	7.937991	8.032894	NM_012086	chr2:197634742-197634683	GTF3C3
A_23_P13604	12.180212	12.180212	NM_002567	chr12:118583256-118583315	PEBP1
A_33_P3234277	5.3246264	5.7211494	NM_001242524	chr6:33036945-33036886	HLA-DPA1
A_33_P3279681	4.0131817	3.105073	NM_001080401	chr19:46003749-46003808	PPM1N
A_33_P3215803	4.8640523	4.930252	NM_001974	chr19:6937566-6937625	EMR1
A_24_P50753	8.16521	8.174881	NM_199040	chr12:93795781-93795841	NUDT4
A_23_P60657	9.358105	9.744951	NM_015203	chr1:150445919-150445978	RPRD2
A_33_P3389837	9.916611	9.785504	NM_001164372	chr12:110890465-110890406	GPN3
A_24_P271527	9.919011	9.923078	NM_014876	chr22:39081879-39081820	JOSD1
A_33_P3389638	9.984139	9.515749	NM_003703	chr4:2939736-2939677	NOP14
A_23_P59787	9.049707	8.472536	NM_016019	chr7:139107996-139108055	LUC7L2
A_23_P141893	6.871228	6.793709	NM_001080401	chr19:46005458-46005517	PPM1N
A_24_P8088	8.177483	8.160142	NM_153005	chr6:7405537-7410626	RIOK1
A_23_P3681	10.1474085	9.832707	NM_018092	chr16:47115839-47115780	NETO2
A_24_P521994	7.07286	7.5485244	NM_017644	chr3:183402052-183402111	KLHL24
A_33_P3296582	5.184789	4.4853015	NM_024590	chr4:114821697-114821638	ARSJ
A_24_P166645	7.374274	7.2136407	NM_014374	chr7:150070586-150070645	REPIN1
A_24_P89457	5.6585054	6.153989	NM_078467	chr6:36652178-36652237	CDKN1A
A_24_P370096	4.441645	4.309699	NM_006300	chr19:44514938-44514997	ZNF230

A_23_P145376	5.3689566	5.1728754	NM_002754	chr6:36106750-36106809	MAPK13
A_33_P3302125	4.2934566	4.424848	NM_178428	chr1:152671859-152671918	LCE2A
A_33_P3345344	3.2768323	2.3900566		chr10:50572296-50572237	DRGX
A_23_P152406	6.3213396	6.7435813	NM_032330	chr16:55601378-55601437	CAPNS2
A_33_P3330323	6.46929	6.4473295	NM_001080541	chr15:42021388-42021447	MGA
A_33_P3247803	5.8383026	5.7258916	NM_203390	chr8:94744100-94744041	RBM12B
A_33_P3267822	4.5000434	4.192021	NM_001122731	chr22:18348776-18348717	MICAL3
A_32_P143048	4.434273	4.8895473	NM_004799	chr1:52803579-52805830	ZFYVE9
A_33_P3256232	10.011255	10.007291	NM_021933	chr1:12092047-12092106	MIIP
A_33_P3221761	5.7465434	6.112429	NM_014727	chr19:36211782-36211841	KMT2B
A_24_P377499	6.4147234	5.7555637	NM_015550	chr7:24836828-24836769	OSBPL3
A_23_P211064	5.270146	5.1722636	NM_013329	chr21:34131469-34131410	PAXBP1
A_24_P237753	5.3899145	5.1417246	NM_015354	chr9:131767789-131767962	NUP188
A_23_P55917	3.727099	3.7536967	NM_032298	chr19:51125420-51125361	SYT3
A_23_P143474	12.891449	12.758359	NM_001697	chr21:35275858-35275799	ATP5O
A_33_P3312642	3.993741	4.045376	NM_001122716	chrX:125955457-125955516	CXorf64
A_24_P664939	7.4857235	7.220895	NM_001127192	chr3:128886916-128886857	CNBP
A_33_P3377380	5.4576774	5.5620394	AK093659	chr1:39990685-39990626	PPIEL
A_23_P76983	5.6527176	6.101077	NM_025057	chr14:74532317-74532376	CCDC176
A_33_P3335030	10.885145	10.676964	NM_001164315	chr2:97871812-97871870	ANKRD36
A_33_P3237734	7.0905914	6.763817	NM_001040261	chr4:151170764-151170823	DCLK2
A_23_P32903	9.004681	8.679623	NM_000276	chrX:128726414-128726473	OCRL
A_33_P3268174	8.384815	7.79833	NM_152903	chr13:41701940-41701881	KBTBD6
A_33_P3213432	8.40663	8.356344	NM_031905	chr7:102738941-102739000	ARMC10
A_23_P502609	4.6276083	4.699743	NM_004204	chr16:633577-633636	PIGQ
A_24_P870620	2.8986552	2.3900566	NM_002825	chr7:136912112-136912079	PTN
A_23_P70547	3.564704	3.557929	NM_005527	chr6:31777957-31777898	HSPA1L
A_33_P3333677	5.3916025	5.256454		chr15:022706220-022706161	
A_33_P3248992	7.4506154	6.6653943	NM_001609	chr10:124817580-124817639	ACADSB
A_23_P162106	9.99082	9.436943	NM_016055	chr11:73575582-73575641	MRPL48
A_23_P15357	7.4059706	7.5590744	NM_005567	chr17:76968107-76968048	LGALS3BP
A_24_P182281	4.170992	4.294766	NM_001036646	chr1:70820653-70820712	HHLA3
A_24_P944222	5.0324025	5.236816	NM_181783	chr12:88591102-88591161	TMTC3
A_23_P156355	8.197836	8.12757	NM_153354	chr5:87491334-87491275	TMEM161B
A_23_P81158	4.3143535	4.568549	NM_000669	chr4:100260740-100257881	ADH1C
A_33_P3281850	5.217125	4.654356		chr2:27322040-27321981	CGREF1
A_33_P3223749	7.2153597	6.2681413	NM_004229	chrX:40508901-40508842	MED14
A_24_P227069	7.682199	7.3798	NM_020918	chr10:113909886-113909827	GPAM
A_24_P240242	5.366043	5.23658	NM_002375	chr3:47898949-47896820	MAP4
A_33_P3227345	6.6854134	6.984125	NM_054014	chr20:1352855-1352796	FKBP1A
A_23_P2922	7.658924	7.998061	NM_016586	chr14:36768189-36768130	MBIP
A_32_P95739	12.38113	12.734198	NM_000365	chr12:6979746-6979805	TPI1
A_23_P218158	9.900719	9.962095	NM_001284280	chr14:91924609-91924550	SMEK1
A_23_P101642	5.715922	5.790149	NM_002842	chr19:55692827-55692768	PTPRH
A_33_P3417195	4.7233834	4.6752253	NM_203425	chr17:59490582-59490641	C17orf82
A_23_P151710	5.690216	5.9538426	NM_000956	chr14:52794430-52794489	PTGER2
A_33_P3409631	3.750865	3.5721931		chr10:131577632-131577691	
A_33_P3369581	9.157114	8.897478	NM_003755	chr19:10227636-10227577	EIF3G
A_32_P380675	4.7528315	4.7418823	NM_001080423	chr3:14530795-14530736	GRIP2
A_23_P18939	9.3096695	9.771924	NM_002890	chr5:86686765-86686824	RASA1
A_33_P3280597	3.6618884	3.8989344		chr20:056820861-056820802	
A_32_P192823	8.124834	8.021079	NM_175886	chr7:18066555-18066496	PRPS1L1
A_24_P211044	5.01116	5.3740897	NM_001317	chr17:61972330-61972271	CSH1
A_32_P34	10.598296	10.756146	AK092544	chr8:12437900-12437959	LOC100131581
A_33_P3227209	12.028608	11.8323965	NM_006191	chr12:56506846-56506905	PA2G4
A_33_P3356910	9.60557	9.736561	NM_001006938	chrX:101395741-101395682	TCEAL6
A_24_P96780	7.5280356	7.9792733	NM_016343	chr1:214826239-214826298	CENPF
A_33_P3242136	5.203382	5.4598413	NM_001257206	chr5:142077336-142077277	FGF1
A_24_P167877	12.872877	12.688326	NM_001135865	chr16:22547503-22547562	NPIPB5

A_23_P342727	5.9331727	5.697958	NM_178006	chr13:33678315-33678256	STARD13
A_23_P414211	4.030281	3.2765183	NM_006193	chr7:127250825-127250766	PAX4
A_33_P3287972	7.0869327	7.268324	NM_006506	chr3:141328847-141328906	RASA2
A_23_P342668	10.495584	10.310839	NM_005088	chrX:1721323-1721382	AKAP17A
A_24_P385134	8.249246	8.900111	NM_001037582	chr4:83551120-83551061	SCD5
A_33_P3330952	5.0285726	4.7109594	NM_006095	chr4:42410508-42410449	ATP8A1
A_23_P128991	12.210431	11.973688	NM_031210	chr14:78182164-78183839	SLIRP
A_33_P3454679	7.0622106	6.2615323	BU190374		HAB1
A_23_P75204	8.837233	8.594114	NM_017615	chr10:123721025-123720966	NSMCE4A
A_24_P223124	8.723983	8.661682	NM_022763	chr3:172118263-172118322	FNDC3B
A_33_P3272849	5.2715206	4.787405	XM_005266053	chr9:139650100-139650041	LCN8
A_24_P945293	4.632022	5.0166564	NM_016079	chr2:86730587-86730551	CHMP3
A_33_P3237567	6.375927	6.4663606	AK316198	chr6:29912493-29912552	HLA-A
A_23_P50357	9.405915	9.067914	NM_015318	chr19:7537261-7537320	ARHGEF18
A_23_P38864	10.741646	10.934173	NM_006423	chr19:42460901-42460842	RABAC1
A_33_P3288649	6.6320534	6.8643265	NM_018951	chr7:27213023-27211789	HOXA10
A_23_P32253	8.997834	9.6318	NM_005384	chr9:94171459-94171400	NFIL3
A_33_P3296308	9.371264	9.345391	NM_017741	chr4:17802337-17802278	DCAF16
A_33_P3234899	12.840096	12.846558	NM_002795	chr17:36920386-36920445	PSMB3
A_33_P3320718	5.3709574	5.2935114	XM_005255634	chr16:685057-684998	C16orf13
A_23_P94301	9.9344635	10.0773945	NM_003313	chr8:144695453-144695394	TSTA3
A_24_P124992	10.695933	10.712345	NM_002789	chr15:78837978-78838037	PSMA4
A_23_P42087	7.5953555	7.8691874	NM_004332	chr6:3152848-3152907	BPHL
A_33_P3333523	14.035051	14.227888		chr10:120691788-120691729	
A_32_P66364	3.933748	4.1896596	NM_001080545	chr2:182982132-182982191	PPP1R1C
A_24_P320880	6.9626217	6.9598165	NM_022133	chr8:82712472-82712413	SNX16
A_33_P3225843	7.2755003	7.6205926	NM_173463	chr4:24809873-24809814	CCDC149
A_23_P58031	3.274032	2.9158778	NM_004721	chr3:185200168-185200227	MAP3K13
A_33_P3422822	4.48184	4.6212325	NM_020435	chr1:228347464-228347523	GJC2
A_24_P40229	7.1051435	7.3834114	NM_016652	chr20:20018171-20018112	CRNKL1
A_24_P11307	8.851646	9.355723	NM_006621	chr1:110562186-110562245	AHCYL1
A_33_P3256660	7.3704433	7.2658186		chr12:006645742-006645683	
A_32_P112279	6.6798096	6.4710026	NM_001039690	chr16:69152618-69152559	CHTF8
A_33_P3355408	7.4069357	7.388839	NR_038406	chr8:74353450-74353509	STAU2-AS1
A_33_P3415826	3.7844532	3.5468454		chr5:008458432-008458373	
A_33_P3403615	9.147818	9.686041	NM_000801	chr20:1350005-1349946	FKBP1A
A_23_P90911	9.904173	9.375069	NM_020905	chr2:18736163-18736104	RDH14
A_33_P3256738	5.6426716	6.0965266	NM_032818	chr9:35663057-35662998	ARHGEF39
A_33_P3276475	7.010625	7.467115	NM_020412	chr18:11854358-11854417	CHMP1B
A_33_P3314550	6.736287	6.7209034	NM_002866	chr19:18307671-18307612	RAB3A
A_33_P3380772	6.409296	6.373885	NM_080625	chr20:30607145-30607204	CCM2L
A_24_P271323	5.957659	6.301717	NM_030777	chr20:45363893-45363952	SLC2A10
A_23_P25030	4.034044	3.6417801	NM_003725	chr12:57180933-57180992	HSD17B6
A_23_P162970	8.430393	8.318221	NM_024658	chr14:24650726-24649913	IPO4
A_23_P107051	5.659393	5.4936194	NM_003673	chr17:37822740-37822799	TCAP
A_33_P3417222	10.808954	11.219689	AK309268	chr9:35616995-35616936	CD72
A_23_P166280	6.9628806	6.320317		chr21:45642999-45642940	ICOSLG
A_23_P314798	7.470336	6.8265624	NM_144622	chr1:154999101-154999042	DCST2
A_33_P3395976	9.803209	9.638739	NM_145232	chr19:51601835-51601776	CTU1
A_33_P3372566	3.8846362	4.489909		chr6:43402538-43402597	ABCC10
A_33_P3312877	9.199478	9.488879	NM_174923	chr9:35661115-35661174	CCDC107
A_32_P20454	7.8002806	7.8835206	NM_001098398	chr1:160258482-160258423	COPA
A_23_P24623	8.523548	8.632567	NM_019040	chr11:31805185-31805244	ELP4
A_23_P71480	6.2649584	6.340761	NM_005218	chr8:6728240-6728181	DEFB1
A_33_P3415207	5.2037864	5.217824	AF088036	chr2:27534422-27534363	MPV17
A_23_P1775	6.5911217	6.9616833	NM_001382	chr11:118967934-118967875	DPAGT1
A_24_P118938	2.9003072	2.3900566	NM_001256798	chr20:31031812-31031753	C20orf112
A_33_P3404829	5.9049497	6.4947047	BC044661	chr10:134750999-134750940	TTC40
A_33_P3210848	7.519173	7.6372237	NM_001128636	chr7:1787400-1787459	ELFN1

A_32_P163858	9.130964	8.971268		chr10:102123917-102123976	SCD
A_23_P31240	4.319015	3.915629	NM_024637	chr7:99757928-99757869	GAL3ST4
A_32_P118258	15.005991	15.000444	NM_000982	chr13:27830613-27830672	RPL21
A_24_P314351	7.2004614	7.4228215	NM_006777	chrX:119391560-119391619	ZBTB33
A_24_P134356	7.194732	7.632049	NM_014962	chr20:11906389-11906448	BTBD3
A_33_P3273822	4.1784825	3.675928		chr2:48016520-48016461	FBXO11
A_33_P3367726	2.3221061	2.9066715	NR_003539	chrX:95592312-95592253	LOC643486
A_33_P3247252	5.1880226	4.7184463		chr10:027608694-027608635	
A_33_P3395384	4.8563643	4.774487	NM_001143854	chr12:113334902-113334961	RPH3A
A_33_P3348973	5.2506123	5.4632163	NM_001178000	chr2:209168982-209169041	PIKFYVE
A_23_P5163	6.6279907	6.508426	NM_020410	chr19:19756748-19756689	ATP13A1
A_24_P942517	7.535173	7.702751	AB032988	chr20:7958502-7958443	TMX4
A_33_P3330209	4.8910627	4.5520444	NM_001127232	chr7:69755435-69755494	AUTS2
A_23_P60591	9.997562	9.965945	NM_003315	chr17:40133955-40133896	DNAJC7
A_23_P34597	5.2519298	5.4748945	NM_001785	chr1:20945069-20945128	CDA
A_23_P55518	6.6749253	6.7238193	NM_005904	chr18:46446841-46446782	SMAD7
A_23_P75811	9.893087	9.45542	NM_001012662	chr11:62652689-62652748	SLC3A2
A_33_P3333267	4.9460373	4.8611126	NM_001204210	chr17:76236110-76236169	TMEM235
A_33_P3402489	4.181049	4.325863	NM_006187	chr12:113405902-113405961	OAS3
A_23_P253046	9.310556	9.5595045	NM_006759	chr2:64118483-64118542	UGP2
A_23_P57588	9.283353	9.402147	NM_016426	chr22:46725391-46725450	GTSE1
A_33_P3222788	4.838311	4.760214	NR_024467	chr10:93067120-93067061	LOC100188947
A_24_P169148	13.019882	12.93904	NM_002128	chr13:31037696-31037459	HMGB1
A_33_P3315258	5.0639567	5.2637444	NM_004284	chr1:146736128-146736187	CHD1L
A_24_P310894	13.041775	12.771793	NM_006135	chr1:113214149-113214208	CAPZA1
A_33_P3369419	5.4936194	5.6169224	NM_001282861	chr1:155747507-155747448	GON4L
A_23_P410587	6.1381235	6.3332767	NM_024900	chr4:129784662-129784721	JADE1
A_33_P3288135	6.2230353	6.175391		chr5:175264792-175264851	CPLX2
A_23_P24389	5.292966	4.930881	NM_032251	chr11:64123078-64124547	CCDC88B
A_23_P42042	8.1221285	7.925637	NM_020466	chr6:90347584-90347525	LYRM2
A_23_P26021	10.395808	10.269528	NM_004236	chr15:49420020-49419961	COPS2
A_23_P39251	4.144621	3.7674928	NM_001013706	chr19:4523409-4523350	PLIN5
A_33_P3421748	8.934701	9.297856		chr21:034931302-034931361	
A_33_P3569068	7.702538	7.3146825	NM_003550	chr7:1937996-1937937	MAD1L1
A_33_P3398727	5.0766687	5.109939	BC041856	chr22:16148815-16148756	
A_24_P256654	7.0301604	6.7538857		chrX:46407543-46407602	ZNF674-AS1
A_33_P3236117	6.0443254	6.11381		chr3:163719667-163719608	
A_32_P87531	6.2808065	6.172555	NM_001145154	chr1:225156479-225156538	DNAH14
A_24_P681301	14.946226	15.012947	NM_021009	chr12:125396789-125396730	UBC
A_33_P3412295	4.96225	5.2702723	AK126090	chr1:235752062-235752121	LOC645645
A_23_P436145	3.4795754	3.6812403	XR_132807	chr11:130714222-130714163	LOC100507431
A_23_P350045	11.102488	10.771717	NM_005669	chr5:112212209-112212150	REEP5
A_24_P647682	7.746415	7.746415		chr7:055714250-055714309	
A_23_P156049	10.3456335	10.761314	NM_000521	chr5:74014777-74016292	HEXB
A_23_P89941	9.1233015	8.112175	NM_001800	chr19:10677285-10677226	CDKN2D
A_33_P3237220	4.799068	4.463939	NM_033506	chr7:100198679-100198738	FBXO24
A_33_P3328360	7.6801605	7.4587703	NR_027033	chr22:46509748-46509807	MIRLET7BHG
A_23_P253375	5.206669	4.96396	NM_001913	chr7:101926989-101927048	CUX1
A_23_P134809	10.273493	10.044422	NM_003580	chr8:59496443-59496384	NSMAF
A_33_P3251703	6.2127433	6.2977524	NM_001311	chr14:105955069-105955126	CRIP1
A_33_P3866448	2.5620809	2.3900566	BX648501	chr9:16218842-16218901	
A_23_P18806	7.4304924	7.6133337	NM_001024947	chr5:143541882-143541823	YIPF5
A_33_P3296303	10.129342	10.08733	NM_016410	chr9:33280810-33280869	CHMP5
A_33_P3227492	4.368355	4.3308163	NR_103539	chr1:228351895-228351836	IBA57-AS1
A_33_P3294603	10.536037	10.796339		chr16:89262615-89262556	SLC22A31
A_33_P3238196	6.6569138	6.979984	NM_001278171	chr10:38345429-38345488	ZNF33A
A_23_P50217	6.8717256	7.196621	NM_024833	chr19:58231295-58231236	ZNF671
A_33_P3322046	7.045147	7.09801		chr10:072554039-072553980	
A_23_P74252	6.819344	6.8412147	NR_023918	chr1:22357351-22357410	LINC00339

A_24_P168994	4.067184	4.3042755	NM_178571	chr17:15647698-15647757	TBC1D26
A_23_P137057	13.410927	13.453424	NM_001152	chrX:118605131-118605190	SLC25A5
A_23_P91293	8.713093	8.786829	NM_004738	chr20:57020214-57020273	VAPB
A_33_P3395713	5.7223077	5.907382	NM_018963	chr21:40574389-40574330	BRWD1
A_24_P229536	5.0621443	5.1619644	NR_027791	chr21:17909716-17979329	LINC00478
A_33_P3696965	3.9406548	3.826573	AA593742	chr2:98138415-98138474	
A_23_P36689	5.9903355	6.0374002	NM_006992	chr12:7015743-7015802	LRRC23
A_32_P93852	10.220207	10.139103	NM_138369	chr5:173034743-173034684	BOD1
A_23_P161338	11.461009	11.370107	NM_021129	chr10:71962931-71962872	PPA1
A_33_P3290800	4.258389	4.7278957	NM_182540	chrX:134715008-134715067	DDX26B
A_33_P3336617	10.132268	10.154106	NM_000382	chr17:19575195-19575254	ALDH3A2
A_23_P100355	8.84101	8.949158	NM_002720	chr16:30096047-30096106	PPP4C
A_23_P418373	9.088139	9.24477	NM_004050	chr14:23780660-23780719	BCL2L2
A_32_P213624	8.356015	8.1732025	NM_001130841	chr1:100758204-100758263	RTCA
A_33_P3385361	12.127104	11.856677	NM_016167	chr6:13620657-13620716	NOL7
A_23_P200030	7.916069	8.044764	NM_003838	chr1:74672625-74672684	FPGT
A_24_P227971	8.786829	8.717964	NM_016212	chr16:33262376-33262435	TP53TG3
A_24_P311926	12.093865	12.230672	NM_002127	chr6:29798694-29798753	HLA-G
A_33_P3337896	7.0878	6.9458756	AK024906	chr1:144862766-144862707	
A_33_P3341234	10.121149	10.050291	NM_003344	chr7:129473134-129473075	UBE2H
A_32_P98227	4.13461	4.154693	NM_001171610	chr10:88495765-88495824	LDB3
A_32_P162187	6.2729597	6.455894	NM_000063	chr6:31913187-31913246	C2
A_23_P434944	8.210048	9.004435	NM_004516	chr19:10795765-10795824	ILF3
A_33_P3338011	7.3722963	7.444013	NM_144684	chr19:52826052-52826111	ZNF480
A_33_P3271490	7.491425	6.9274693	NM_016836	chr2:161130579-161130520	RBMS1
A_32_P157927	3.5263214	3.0935767	S62210	chr2:89185420-89185479	
A_23_P12858	5.438861	5.6239543	NM_152710	chr10:72534060-72534001	TBATA
A_23_P138967	9.130774	8.96253	NM_003002	chr11:111965707-111965766	SDHD
A_33_P3244640	4.8434606	4.6222644	AF370369	chr10:121217016-121217075	GRK5
A_24_P213354	13.456213	13.38592		chr10:074765855-074765795	
A_32_P45168	9.146902	9.704746	NM_002184	chr5:55230923-55230909	IL6ST
A_23_P97457	8.9814005	9.556538	NM_022831	chr1:222842440-222842381	AIDA
A_23_P9289	4.1282873	4.531614	NM_134428	chr9:3257191-3257132	RFX3
A_33_P3334948	4.5825725	4.728433		chr12:009587882-009587823	
A_33_P3402725	8.090637	7.864944	NM_018489	chr1:155305915-155305856	ASH1L
A_33_P3293524	9.532559	9.7251005	NM_004210	chr10:105352243-105352302	NEURL1
A_23_P308150	5.7510214	5.727585	NM_152424	chrX:63405059-63405000	AMER1
A_33_P3342992	6.9168816	7.101152	NR_004847	chr15:82707625-82707684	UBE2Q2P2
A_23_P320457	6.853625	7.0434155	NM_033088	chr1:110596897-110596956	STRIP1
A_33_P3210556	3.9954464	3.564704	NR_028139	chr15:100884752-100884693	SPATA41
A_24_P150486	8.655212	8.766335	NM_004863	chr14:77974051-77973992	SPTLC2
A_33_P3407042	8.946982	8.740137	NM_080605	chr1:1170309-1170368	B3GALT6
A_23_P415021	5.948743	6.742962	NM_014033	chr12:51326101-51326160	METTL7A
A_33_P3392366	8.045856	8.075453	XR_245823	chr14:105131315-105131256	
A_23_P67151	4.468513	4.4201813	NM_058164	chr19:9964915-9964856	OLFM2
A_24_P56884	7.3817835	7.6397676	NM_152464	chr17:26686386-26687569	TMEM199
A_23_P167812	6.888318	5.739237	NM_153020	chr6:17293731-17293790	RBM24
A_23_P160720	5.9806275	5.697221	NM_018664	chr1:212860087-212860028	BATF3
A_33_P3398074	4.659814	4.641381	AF132201	chr17:67229991-67229932	PRO1804
A_33_P3368830	7.0364676	7.204541	NM_001033667	chr1:160772422-160772481	LY9
A_33_P3396607	10.839266	10.196302	NM_003359	chr4:39500471-39500412	UGDH
A_23_P316582	4.82695	4.894684	NM_152355	chr19:11892951-11893010	ZNF441
A_23_P34968	10.604258	10.513466	NM_001204856	chr1:151141499-151141558	SCNM1
A_23_P47527	10.736013	11.022508	NM_014206	chr11:61557336-61557277	TMEM258
A_23_P329870	9.374538	9.208194	NM_024599	chr17:74467037-74466978	RHBDF2
A_24_P97197	6.2138925	6.336301	NM_182642	chr2:219268088-219269026	CTDSP1
A_23_P374351	7.342567	7.7989483	NM_173562	chr6:36457617-36457676	KCTD20
A_24_P403734	8.193811	8.568335	NM_015481	chr12:54763054-54762995	ZNF385A
A_23_P165494	10.457739	10.594913	NM_015530	chr2:171823014-171823073	GORASP2

A_24_P372625	8.201024	7.7748566	NM_016422	chr11:10533573-10533514	RNF141
A_33_P3353263	6.058585	5.7909365	BC071735	chr1:54651886-54651827	CYB5RL
A_33_P3629678	9.787751	10.097739	NM_000093	chr9:137736327-137736386	COL5A1
A_33_P3256088	4.97957	5.3024926	NR_103825	chr4:119554075-119554134	LOC729218
A_24_P350744	5.104829	5.0959206	NM_014949	chr1:155899601-155899542	KIAA0907
A_33_P3218741	10.898616	11.195654		chr15:089346503-089346562	
A_24_P292020	8.649179	8.520133	NM_006234	chr7:102114833-102114098	POLR2J
A_33_P3349145	5.1868625	5.25691	NM_012263	chr22:43435711-43435652	TTLL1
A_23_P58036	10.311222	10.3192005	NM_020166	chr3:182733114-182733055	MCCC1
A_24_P50368	4.917361	3.813318	NM_001001786	chr11:121986373-121986314	BLID
A_23_P327698	5.0103116	4.9125524	NM_001007527	chr5:36108672-36105277	LMBRD2
A_23_P218486	9.930028	10.11624	NM_016145	chr19:12779350-12779197	WDR83OS
A_33_P3382412	4.4353294	4.463002	NM_001277120	chr19:53342568-53342509	ZNF468
A_33_P3330109	11.829773	12.208192	NM_001243042	chr6:31236676-31236617	HLA-C
A_33_P3399480	3.7038984	2.946763		chr6:69344248-69344189	
A_23_P159539	7.5450573	8.133852	NM_004192	chrX:1522197-1522138	ASMTL
A_33_P3283713	9.8429	9.99082	NM_015407	chr3:52015145-52015204	ABHD14A
A_23_P3775	8.757027	8.575044	NM_018233	chr16:56510212-56510271	OGFOD1
A_33_P3392350	4.4258146	4.6025944		chr11:093800231-093800172	
A_23_P144877	8.722313	8.767188	NM_004045	chr5:151125929-151125870	ATOX1
A_32_P170444	8.959866	8.775856	NM_006713	chr5:32603930-32603989	SUB1
A_23_P26945	7.527775	7.4629064	NM_000263	chr17:40696244-40696303	NAGLU
A_33_P3210866	5.6108627	5.723931	CR749856	chr19:53067543-53067602	ZNF808
A_23_P218079	7.45415	7.760142	NM_018976	chr12:46754684-46754625	SLC38A2
A_23_P391607	6.5357018	6.680815	NM_152285	chr9:140509391-140509450	ARRDC1
A_33_P3375314	2.6732996	3.4907637	NM_006045	chr20:50217461-50217402	ATP9A
A_33_P3234103	10.269918	10.279789	NM_014712	chr16:30995913-30995972	SETD1A
A_33_P3382709	8.360432	8.20571	NM_014319	chr12:65641987-65642046	LEMD3
A_23_P346265	6.0290055	5.960801	NM_024312	chr12:102190514-102190455	GNPTAB
A_24_P159036	14.227888	14.160603	NM_001001	chr14:50085582-50085523	RPL36AL
A_24_P106681	12.945196	12.74947	NM_012479	chr7:75956192-75956133	YWHAG
A_24_P408321	8.055726	7.6598215	NM_144498	chr20:60871128-60871187	OSBPL2
A_33_P3356216	6.294172	6.474165	NM_001655	chr11:118454003-118454062	ARCN1
A_24_P16610	6.9054074	6.967745	NM_032268	chr16:75144300-75144359	ZNRF1
A_23_P140648	10.702837	11.012625	NM_014608	chr15:23003325-23003384	CYFIP1
A_24_P29445	10.662669	10.666563	NM_030969	chr6:10751451-10755417	TMEM14B
A_33_P3209476	10.31369	10.451376	NM_031443	chr7:45116009-45116068	CCM2
A_33_P3424384	3.5020385	4.043867	XM_005264579	chr2:39996103-39996044	THUMPD2
A_23_P412392	8.933028	8.9924555	NM_004892	chr1:145116390-145116449	SEC22B
A_23_P52207	7.3069706	7.1087503	NM_012342	chr10:28971551-28971610	BAMBI
A_32_P57702	10.947164	10.932973	NR_033245	chr7:64043343-64043402	LOC641746
A_24_P82419	10.79854	10.593769	NM_005324	chr17:73774971-73774912	H3F3B
A_33_P3431595	10.292791	9.723866	NM_173687	chr8:144134801-144134860	C8orf31
A_33_P3339276	3.6441417	4.2301226		chr16:20442583-20442643	ACSM5
A_23_P77455	10.377854	10.309881	NM_016062	chr16:66967585-66967526	FAM96B
A_33_P3213645	5.5936413	5.3791523	NM_033266	chr16:23701689-23701630	ERN2
A_33_P3366120	10.493313	10.327527	NM_001110556	chrX:153577323-153577264	FLNA
A_24_P272310	3.2693374	2.973279	NM_205853	chr3:52867392-52867333	MUSTN1
A_24_P126060	10.021875	10.273493	NM_001356	chrX:41208627-41208686	DDX3X
A_32_P142652	12.400431	12.551935		chr13:52035265-52035324	
A_32_P148672	10.727446	10.707723	NM_006938	chr18:19203758-19203816	SNRPD1
A_23_P152651	8.525387	8.742505	NM_007372	chr17:61895992-61896051	DDX42
A_33_P3352449	7.841214	7.537006	NM_019041	chr6:153311110-153311051	MTRF1L
A_24_P377144	4.866433	4.466537	NM_058172	chr4:80899282-80899223	ANTXR2
A_23_P167818	4.205044	4.078477	NM_024581	chr6:119281299-119281240	FAM184A
A_33_P3312150	3.6537406	4.198145	AF172850	chr1:143401032-143400973	LINC00328
A_32_P191895	6.4581013	7.404214	BC045716	chr8:70855144-70855203	
A_33_P3216277	4.8234577	4.5640965	XM_003959913	chr1:205784288-205784347	LOC101059976
A_33_P3311653	8.032574	8.076414	AK124569	chr4:39838843-39838784	PDS5A

A_23_P68665	8.493549	8.23641	NM_007002	chr20:60883102-60883161	ADRM1
A_24_P321525	5.2182875	5.4492664	NM_032918	chr12:15260970-15260911	RERG
A_23_P397856	5.304164	5.314597	NM_001039661	chr11:126163990-126164049	TIRAP
A_33_P3222045	7.042818	6.8903165	NM_201400	chr16:5134584-5134526	FAM86A
A_33_P3214463	3.6651568	3.3111727	NM_001039958	chr15:90321888-90321947	MESP2
A_33_P3420416	4.785796	4.763719	NM_002308	chr17:25967739-25967798	LGALS9
A_33_P3420446	7.901913	8.272312	NM_001161528	chr7:91774317-91774258	LRRD1
A_23_P374149	7.076759	6.7575736	NM_015050	chr6:37448883-37448942	CMTR1
A_24_P371425	5.2056375	4.943586	NM_032166	chr3:48502140-48505144	ATRIP
A_33_P3343007	4.8895473	4.8731413	X68698	chr14:22580888-22580947	
A_33_P3372563	6.1847053	6.294894	NM_033450	chr6:43412934-43412993	ABCC10
A_33_P3396746	6.067007	6.185325	BC042481	chr1:245534958-245535017	KIF26B
A_24_P76666	6.0383997	6.215123	NM_177559	chr20:469405-469346	CSNK2A1
A_24_P7202	6.5330806	6.569543	NM_020738	chr2:8916892-8910918	KIDINS220
A_23_P38723	8.523193	8.655484	NM_015295	chr18:2802664-2802723	SMCHD1
A_23_P412029	4.4701753	4.3477254	NM_144709	chr2:61168287-61168228	PUS10
A_23_P341700	6.046501	5.9609365	NM_001007101	chr9:95608515-95608456	ZNF484
A_23_P13094	3.8427186	3.380513	NM_002425	chr11:102641464-102641405	MMP10
A_23_P320261	2.453327	2.3900566	NM_001035516	chr19:35988194-35988135	DMKN
A_33_P3248900	11.294033	11.55192	NR_028324	chr19:197281-197222	LINC01002
A_23_P153930	4.9467096	5.524776	NM_001616	chr2:148684923-148684982	ACVR2A
A_33_P3261353	5.2696705	5.335348	NR_033732	chrY:21621987-21621928	BCORP1
A_23_P79134	5.8407307	5.442589	NM_024310	chr19:30165789-30165848	PLEKHF1
A_33_P3316293	6.571455	6.3686686	NM_001130849	chr2:231683360-231683419	CAB39
A_23_P141429	3.4210608	3.637313	NM_016428	chr17:47300191-47300250	ABI3
A_23_P11729	6.5621614	6.9755077	NM_001252406	chr1:154989305-154989364	ZBTB7B
A_24_P204043	5.064829	4.9049406	NM_014345	chr6:43316187-43316128	ZNF318
A_23_P91390	5.669467	4.4428396	NM_000361	chr20:23026866-23026807	THBD
A_32_P83784	5.3210583	4.8228607	NM_015230	chr4:36068160-36068101	ARAP2
A_32_P123514	6.072667	5.489956	NM_001114734	chr4:135117717-135117658	PABPC4L
A_33_P3423240	5.672988	5.7970257		chr10:46281346-46281405	FAM21C
A_33_P3298387	5.9924	5.7341924	NM_005030	chr16:23692251-23692310	PLK1
A_33_P3343820	3.628978	3.6078444	NR_028344	chrX:139795889-139795948	LINC00632
A_23_P395534	5.9630685	5.958214	NM_006511	chr1:15988139-15988198	RSC1A1
A_33_P3265224	5.6587152	6.040458	BX648774	chr2:65302195-65302254	CEP68
A_33_P3238993	7.7412186	8.008401	NM_001135187	chr2:228419195-228419254	AGFG1
A_33_P3418120	5.477913	4.7405963	NM_022755	chr9:95375544-95375485	IPPK
A_33_P3292307	4.693516	4.350947	NM_013319	chr1:11334057-11334116	UBIAD1
A_23_P146765	10.22349	10.249646	NM_007218	chr8:125499960-125500019	RNF139
A_32_P78101	4.1798987	3.4537005	NM_032880	chr1:18704906-18704965	IGSF21
A_23_P58390	7.1429796	7.0328345	NM_152400	chr4:113109969-113110028	C4orf32
A_33_P3293925	4.154383	4.280023	NM_176819	chrX:45008855-45008796	CXorf36
A_33_P3322353	9.975906	9.454779	NM_006136	chr7:116557963-116558022	CAPZA2
A_23_P208416	7.818185	8.145222	NM_024881	chr19:16663630-16663571	SLC35E1
A_23_P321349	8.250589	8.311108	NM_138421	chr11:18108553-18108494	SAAL1
A_23_P98930	8.0902405	8.109037	NM_018169	chr12:32145764-32145823	KIAA1551
A_24_P374962	7.443511	7.537717	NR_040584	chr7:74298454-74298395	STAG3L2
A_33_P3345051	4.7181644	5.0130663	NM_001195256	chr19:49931882-49931941	LOC100507003
A_33_P3244021	10.261396	10.254412	NM_020746	chr20:3847884-3847943	MAVS
A_24_P26073	11.038669	11.2830305	NM_133259	chr2:44117011-44116952	LRPPRC
A_33_P3262665	6.702388	6.458405	NM_024597	chrX:135300483-135300424	MAP7D3
A_33_P3285868	7.6627927	7.9556026	NM_134268	chr17:74523520-74523461	CYGB
A_23_P64630	10.426489	10.571863	NM_032015	chr11:119207791-119207850	RNF26
A_23_P43276	5.3571672	5.232462	NM_032777	chr8:37701268-37701327	GPR124
A_24_P911676	7.0570316	7.3556037	NM_003107	chr6:21598001-21598060	SOX4
A_33_P3392921	5.473218	5.346184	NM_001184740	chr11:70281141-70281200	CTTN
A_33_P3211818	3.6010904	3.8060513	NM_001001890	chr21:36171660-36171601	RUNX1
A_23_P87351	10.348134	10.266235	NM_001033	chr11:4159947-4160006	RRM1
A_24_P55465	9.894906	10.010293	NM_145808	chr7:135612318-135612259	MTPN

A_23_P64560	8.181625	8.315317	NM_014489	chr11:3847352-3847411	PGAP2
A_33_P3294404	7.240306	7.4467325	NM_024595	chr1:39469031-39469090	AKIRIN1
A_23_P334218	7.804137	7.783592	NM_145647	chr8:124164161-124164220	TBC1D31
A_33_P3256695	6.7078323	6.612988	NM_017892	chr2:153508166-153508107	PRPF40A
A_23_P373031	6.07217	6.3097277	NM_000719	chr12:2801192-2801251	CACNA1C
A_24_P212565	6.6905255	6.437784		chr5:172190099-172190040	
A_24_P211565	7.7727637	8.640074	NM_031910	chr22:37576349-37576290	C1QTNF6
A_23_P324523	7.380995	7.7346325	NM_153208	chr16:19868558-19868617	IQCK
A_24_P687326	4.2175126	3.7140322	NR_024366	chr9:115881596-115881655	FAM225A
A_23_P33465	9.376117	9.454327	NM_024324	chr22:50320918-50320977	CRELD2
A_33_P3326989	4.577825	4.226607	NM_014226	chr14:102699017-102698958	MOK
A_33_P3237270	4.093318	2.9894497		chr6:159485869-159485810	
A_23_P382045	9.071014	7.924081	NM_020245	chr6:158932662-158932721	TULP4
A_23_P107855	4.928867	4.7650323	NM_032836	chr19:56103119-56103060	FIZ1
A_23_P126057	10.261899	10.636497	NM_002979	chr1:53516467-53516526	SCP2
A_23_P81121	10.368453	10.055784	NM_005033	chr4:122735108-122735167	EXOSC9
A_33_P3412095	4.559161	4.883567	NM_000288	chr6:137147547-137147606	PEX7
A_23_P321034	6.778498	6.6391854	NM_176877	chr1:62628634-62628693	INADL
A_24_P89911	8.783129	8.64419	NM_197956	chr9:130823610-130823551	NAIF1
A_33_P3279798	5.36908	5.120377	NM_015684	chr14:50788252-50788311	ATP5S
A_33_P3248843	2.720292	2.3900566		chr21:26541821-26541762	
A_33_P3376404	3.600648	3.7520444	NM_017883	chrX:48456286-48456345	WDR13
A_23_P53152	8.855524	8.831302	NM_020642	chr11:8940888-8940947	AKIP1
A_23_P69326	6.492014	6.1195416	NM_183393	chr3:62384835-62384776	CADPS
A_33_P3349474	15.012947	14.937944		chr2:053759872-053759813	
A_33_P3264404	4.9320188	5.019925	AK098270	chr7:101818665-101818724	LOC100132593
A_33_P3387616	11.963112	12.561302	NM_052924	chr8:144466330-144466389	RHPN1
A_24_P57047	5.6462955	5.497326	NM_203486	chr19:39998477-39998536	DLL3
A_33_P3233550	12.989869	13.065256	NM_020532	chr2:55200009-55199950	RTN4
A_24_P290263	6.8404818	6.6534534		chrX:047700796-047700736	
A_23_P58877	9.705849	9.231443	NM_020399	chr6:117881675-117881616	GOPC
A_33_P3352432	8.679256	8.679947	NM_001129899	chrX:46333508-46333567	KRBOX4
A_23_P102235	13.34982	13.14254	NM_003096	chr2:70515224-70508782	SNRPG
A_33_P3345614	5.906025	6.0936165		chr19:012811757-012811816	
A_33_P3270147	3.5980043	3.4175453		chr10:94180281-94180340	XLOC_014512
A_33_P3316671	6.1784782	6.1594706		chr7:135614637-135614696	
A_23_P255286	11.233244	11.251224	NM_017951	chr2:130909170-130909111	SMPD4
A_33_P3421907	5.8730683	6.0650187		chr17:025982384-025982325	
A_23_P210948	6.0176544	5.8685837	NM_018244	chr20:33891603-33891544	UQCC1
A_32_P100439	3.7010279	4.356121	NM_152793	chr7:30202311-30202370	MTURN
A_33_P3377744	5.9790945	5.9772577	AK000950	chr6:104242172-104242231	
A_33_P3237344	5.5336294	5.4678526		chr19:055975136-055975077	
A_23_P165574	6.4126034	6.6956124	NM_017969	chr2:128252464-128250944	IWS1
A_23_P139648	4.0777926	4.3074656	NM_000415	chr12:21532144-21532203	IAPP
A_24_P48318	6.534449	6.75226	NM_017748	chr17:36958989-36958437	CWC25
A_33_P3266396	10.7548	10.635602	NM_001029885	chr1:1264205-1264264	GLTPD1
A_23_P208482	5.777221	6.112181	NM_001144904	chr19:7834397-7834456	CLEC4M
A_33_P3337981	4.164031	4.7226048	NM_014845	chr6:110098202-110098261	FIG4
A_23_P310086	5.4928207	5.0082073	NM_152731	chr6:56891269-56891328	BEND6
A_24_P234792	8.559323	7.9014034	NM_001044723	chr5:122952072-122952131	CSNK1G3
A_33_P3420914	6.772848	6.999048	NR_033579	chr15:82753489-82753548	CSPG4P8
A_24_P405190	8.600046	8.700627	NM_201626	chr1:78177523-78177464	USP33
A_23_P141389	14.362864	14.388734	NM_000988	chr17:41154694-41154753	RPL27
A_33_P3296479	6.41238	6.7214737	NM_001204303	chr21:27423406-27423347	APP
A_32_P90210	11.081654	11.0444355	NM_001015892	chr5:68660773-68660715	TAF9
A_33_P3402763	8.481323	8.417896	NM_032390	chr2:122484947-122484888	NIFK
A_32_P20523	7.5441475	7.4889865	NM_145715	chr4:90035736-90035795	TIGD2
A_33_P3257617	4.1907854	4.4848256	NM_001167858	chr1:202400667-202400726	PPP1R12B
A_33_P3252369	5.221336	3.7135909	NM_144632	chr2:103433819-103433878	TMEM182

A_23_P165078	7.887507	7.4962716	NM_031213	chr19:1877600-1877541	ABHD17A
A_23_P12884	6.365079	6.2156506	NM_005308	chr10:121214967-121215023	GRK5
A_24_P205130	8.571553	8.526061	NM_015033	chr9:132649836-132649777	FNBP1
A_24_P152468	7.47064	7.70178	XR_171643	chr7:45118320-45118261	LOC100128364
A_33_P3258320	5.3720565	5.541316	XM_005259941	chr19:9785402-9785343	ZNF562
A_23_P341223	9.121038	8.702243	NM_014851	chr1:6650895-6650836	KLHL21
A_23_P415633	5.2677875	5.3894744	NM_001174103	chr17:26935213-26935154	SGK494
A_33_P3405444	9.804845	9.6982	NM_003941	chr7:123322060-123322001	WASL
A_23_P205584	6.079912	5.528408	NM_001284201	chr14:59971557-59971862	JKAMP
A_33_P3308137	6.397453	6.126498	NM_199136	chr7:23741734-23741793	FAM221A
A_23_P398530	4.2722654	3.8624039	NM_133334	chr4:1949509-1949568	WHSC1
A_33_P3331491	8.030168	8.239718	NM_001162371	chr17:5402831-5402772	LOC728392
A_33_P3217507	5.5459576	5.831295		chr6:163834467-163834526	
A_23_P32785	8.324331	8.0919075	NM_173824	chr3:88206895-88206954	C3orf38
A_23_P216679	5.551651	6.1850634	NM_033331	chr9:99263578-99263519	CDC14B
A_33_P3243887	6.615861	5.3259244	NM_000641	chr19:55875847-55875788	IL11
A_23_P139547	10.7875395	10.905479	NM_006009	chr12:49579472-49579413	TUBA1A
A_23_P131139	9.0647335	9.1318245	NM_052952	chr2:189599336-189599277	DIRC1
A_33_P3210099	3.4020755	3.5514102	NM_020778	chr15:85416651-85416710	ALPK3
A_33_P3507542	10.634556	10.844669	NM_014262	chr12:6948951-6949010	LEPREL2
A_24_P75718	7.7507277	8.01675		chr12:066039961-066039900	
A_24_P67408	7.965456	8.025123		chr1:224044811-224044750	
A_23_P92035	7.4184885	7.4577327	NM_001080517	chr3:9517207-9517266	SETD5
A_23_P75820	4.9629045	5.035814	NM_001136506	chr11:62850856-62850797	SLC22A24
A_33_P3412695	6.538715	6.5469017		chr9:067670983-067670924	
A_23_P205370	4.140739	3.3860488	NM_016150	chr14:94400660-94400601	ASB2
A_23_P27840	8.515499	8.606413	NM_003169	chr19:39966751-39966810	SUPT5H
A_32_P152437	6.718448	6.708885	NM_005100	chr6:151679428-151679487	AKAP12
A_23_P150255	10.850294	10.576109	NM_006328	chr11:66394693-66394752	RBM14
A_23_P8095	7.8292365	8.112337	NM_006913	chr6:32148248-32148307	RNF5
A_32_P210252	12.065742	11.952536	NM_000983	chr1:6246415-6246356	RPL22
A_24_P901986	4.7324286	5.4437594	BC029255	chr16:29829443-29829384	
A_32_P208823	4.1619663	3.6517437	NM_020405	chr17:37220029-37219970	PLXDC1
A_23_P90296	5.3670287	5.543345	NM_004714	chr19:40316404-40316345	DYRK1B
A_33_P3237135	7.780258	7.8831525	NM_004530	chr16:55540518-55540577	MMP2
A_33_P3327300	3.9861171	2.3900566	AK096566	chr19:41191283-41191224	NUMBL
A_23_P421935	5.3591027	5.063305	NM_020218	chr17:42269773-42269714	ATXN7L3
A_23_P27133	8.449537	8.118196	NM_002275	chr17:39670201-39670142	KRT15
A_32_P85042	4.513175	4.268188		chr19:23445144-23445085	
A_33_P3372099	2.3221061	2.3900566	NM_145244	chr4:101107139-101107080	DDIT4L
A_23_P202138	8.734483	8.409629	NM_030971	chr10:102800646-102800705	SFXN3
A_23_P56654	6.4752893	6.6218038	NM_032601	chr2:71337148-71337089	MCEE
A_23_P52373	7.0676546	7.1875763	NM_003635	chr10:75562062-75562003	NDST2
A_24_P353794	7.6763735	7.650261	NM_004481	chr1:230417262-230417321	GALNT2
A_24_P403561	7.801798	7.5218964	NM_002334	chr11:46878608-46878549	LRP4
A_23_P307430	8.315317	8.390783	NM_152344	chr17:42112349-42112290	LSM12
A_23_P115331	9.606231	9.485647	NM_001029882	chr1:27861300-27861242	AHDC1
A_33_P3396831	5.2437596	4.7381115		chr1:156302524-156302465	CCT3
A_33_P3411885	7.2283554	7.49027	AK127423	chr17:19223552-19223611	LOC100128851
A_32_P169735	8.495375	8.583052	NM_144596	chr14:89343741-89343800	TTC8
A_23_P345065	7.8124027	7.501357	NM_016510	chr2:239007826-239007885	SCLY
A_32_P122240	6.3821774	6.618781	NM_001270601	chr1:201083930-201083871	ASCL5
A_33_P3355454	5.163637	5.224886		chr20:18362171-18362230	XLOC_013472
A_23_P36939	4.9729795	4.7900653	NM_012345	chr13:45514020-45513964	NUFIP1
A_23_P158997	5.279377	5.0872536		chr1:247353404-247353345	XLOC_014512
A_33_P3398107	6.2722654	6.7626133	NM_001197129	chr20:43995753-43995812	SYS1
A_23_P418015	7.0741405	7.1051435	NM_014268	chr18:32721368-32721427	MAPRE2
A_33_P3367332	9.447096	9.158079	NM_001195736	chr1:2522799-2522857	FAM213B
A_23_P206382	7.4078307	7.333768	NM_025187	chr16:67181906-67181965	C16orf70

A_23_P324490	7.1305466	7.2517495	NM_014686	chr19:34846363-34846422	KIAA0355
A_23_P217428	5.853474	6.2380342	NM_013427	chrX:11155690-11155663	ARHGAP6
A_23_P210358	10.072767	10.2289505	NM_004987	chr2:109300515-109300574	LIMS1
A_23_P87013	5.4698677	5.6801615	NM_001001522	chr11:117074574-117074963	TAGLN
A_33_P3276153	5.719327	6.0735793	NM_001282472	chr15:30386156-30386215	GOLGA8J
A_23_P53288	11.446595	11.391367	NM_014255	chr12:56704317-56704258	CNPY2
A_23_P500956	6.433487	6.778849	NM_006577	chr2:62451231-62451290	B3GNT2
A_24_P462899	11.028203	10.782586	NM_001012507	chr6:126669623-126669682	CENPW
A_33_P3257993	3.9327245	2.3900566	NM_017831	chr18:29652911-29652970	RNF125
A_33_P3303542	4.9072776	4.8169045	NM_001144950	chr19:56030303-56030362	SSC5D
A_33_P3275707	7.702223	7.360504	NM_001039569	chr2:224620198-224620139	AP1S3
A_23_P436281	8.790585	8.669977	NM_001034077	chr1:149804396-149804455	HIST2H4B
A_32_P202438	4.9179087	4.1972437	AK025716	chr2:128646022-128646081	
A_33_P3236403	11.643463	11.490257	NM_007056	chr19:45574154-45574213	CLASRP
A_23_P318943	3.8777027	3.6585352		chr16:85319477-85319536	LINC00311
A_23_P41854	7.450982	7.1573577	NM_032587	chr5:40855345-40855404	CARD6
A_24_P244442	5.887812	6.048882	NM_032667	chr11:62462126-62462067	BSCL2
A_33_P3287825	6.462418	5.9409943	NM_022742	chr7:128462122-128462181	CCDC136
A_33_P3260021	6.0672603	5.9458184		chr9:044750072-044750013	
A_33_P3409347	4.466537	4.1387076	NM_014047	chr19:13889523-13889582	C19orf53
A_23_P161563	7.8988	8.159233	NM_022337	chr11:87883009-87882950	RAB38
A_24_P941824	7.7613153	8.143421	NM_004798	chr20:30922248-30922307	KIF3B
A_23_P147245	10.782586	10.490299	NM_017784	chr3:31702460-31702401	OSBPL10
A_23_P34877	4.702607	5.2064295	NM_022768	chr1:110884655-110884714	RBM15
A_23_P80278	7.7407207	7.855182	NM_001007467	chr22:32014430-32014489	SFI1
A_33_P3365357	9.23577	9.216414	NM_033158	chr3:50355325-50355266	HYAL2
A_24_P349560	8.696297	8.729713	NM_001968	chr4:99806203-99806144	EIF4E
A_33_P3283231	8.211886	8.188621	NM_206923	chrX:21876702-21876761	YY2
A_23_P16523	11.077067	10.526267	NM_004864	chr19:18499890-18499949	GDF15
A_23_P208293	11.833675	12.269806	NM_001042724	chr19:45392321-45392380	PVRL2
A_33_P3305446	4.2950554	4.5915146	NM_001011878	chr20:29993970-29993911	DEFB121
A_23_P29225	10.105376	10.268995	NM_012179	chr22:32894588-32894647	FBXO7
A_23_P202881	2.3221061	2.3900566	NM_005103	chr11:125322241-125318416	FEZ1
A_23_P2674	5.102203	5.5476017	NM_002272	chr12:53200570-53200511	KRT4
A_33_P3414487	10.18672	10.20569	NM_001202560	chr7:73112240-73112299	WBSCR22
A_23_P168541	7.598107	7.4209137	NM_024067	chr7:6647983-6648042	C7orf26
A_23_P390139	4.0541115	3.4839404	NM_153015	chr8:109796065-109796006	TMEM74
A_23_P139820	5.672081	6.111103	NM_000617	chr12:51384602-51382184	SLC11A2
A_23_P60016	6.466844	6.486087	NR_002734	chr8:67680130-67680071	PTTG3P
A_33_P3310588	4.6460447	4.685571		chr22:024370743-024370802	
A_23_P68072	8.563005	9.07535	NM_032118	chr2:74652257-74652316	WDR54
A_33_P3211404	4.70353	4.349527	XM_005249853	chr7:5529674-5529615	FBXL18
A_24_P198629	3.9672356	4.5507393	NM_001040616	chr15:101109837-101109778	LINS
A_33_P3419481	5.887148	6.0689464		chr3:186491145-186491204	
A_33_P3215838	5.5017366	5.2550597		chr19:51359882-51359941	KLK3
A_33_P3289192	10.544297	10.358837	NM_006620	chr6:135285853-135285794	HBS1L
A_23_P37877	5.910759	5.6521792	NM_022493	chr16:780057-779998	NARFL
A_23_P102571	12.385656	12.578938	NM_020062	chr20:62374774-62374833	SLC2A4RG
A_33_P3246007	11.061378	10.941367	NM_144772	chr1:156564032-156564091	APOA1BP
A_33_P3360684	4.029037	4.094048	NM_001018070	chr11:67208886-67208827	CORO1B
A_23_P24529	5.694901	5.772761	NM_018195	chr11:111955314-111955373	C11orf57
A_33_P3215739	6.7173347	6.4497633	NM_001085451	chr3:100175107-100175166	LNP1
A_23_P129903	9.706216	9.099069	NM_001037330	chr17:18638903-18638962	TRIM16L
A_33_P3384543	10.873119	9.837567	NM_001243766	chr1:46654413-46654354	POMGNT1
A_23_P152218	4.3819904	4.0443444	NM_001950	chr16:67231510-67231789	E2F4
A_33_P3363245	8.688465	8.5167675	NM_007224	chr12:57620167-57620226	NXPH4
A_33_P3392882	2.3221061	2.3900566	NM_207481	chr2:133721356-133721297	NCKAP5
A_23_P51187	4.842098	4.578471	NM_002744	chr1:2116703-2116762	PRKCZ
A_23_P127150	7.5509834	7.18169	NM_006659	chr10:135097447-135097388	TUBGCP2

A_23_P138271	9.468781	9.394446	NM_138795	chr1:202102732-202102673	ARL8A
A_23_P170467	8.912523	9.201738	NM_006537	chr15:63883141-63883200	USP3
A_23_P200386	11.840261	10.981148	NM_006559	chr1:32509136-32509195	KHDRBS1
A_24_P25530	4.2954807	4.4250164	NM_000835	chr17:72843463-72843017	GRIN2C
A_23_P423074	8.046134	7.6706333	NM_015566	chr5:74076074-74076015	FAM169A
A_33_P3394380	4.9121585	5.1693287	NM_004857	chr14:64935925-64935981	AKAP5
A_33_P3383436	8.245813	8.319315	NM_001193636	chr14:24475288-24475347	DHRS4L2
A_33_P3405728	4.8804665	3.8756092	NM_004572	chr12:32943800-32943741	PKP2
A_33_P3372727	8.352683	8.847062	NM_003966	chr5:9035304-9035245	SEMA5A
A_23_P255153	8.094992	8.184968	NM_016024	chrX:129546667-129546726	RBMX2
A_23_P28485	6.7455087	7.155588	NM_012198	chr2:163217169-163217228	GCA
A_24_P245358	12.8290205	13.247529	NM_001001937	chr18:43666447-43666388	ATP5A1
A_33_P3369854	4.8740797	5.0947948	NM_153614	chr11:73681273-73681332	DNAJB13
A_33_P3320762	7.213853	6.94258	NM_004993	chr14:92525010-92524951	ATXN3
A_33_P3364308	4.037304	4.498152	BC040551	chr16:21758641-21758700	OTOA
A_23_P412186	5.518435	5.870351	NR_023392	chr8:146203248-146203189	ZNF252P
A_23_P147465	4.388139	4.279873	NM_004562	chr6:162475188-162475129	PARK2
A_32_P98502	10.731804	10.702837	NM_004255	chr15:75212695-75212636	COX5A
A_33_P3383371	7.3366184	6.9314284		chr9:037435534-037435593	
A_32_P123088	6.5799446	6.9682217	NM_001164468	chr5:114952115-114952056	TMED7-TICAM2
A_23_P501770	3.938717	3.3609467	NM_032166	chr3:48505498-48506255	ATRIP
A_33_P3372207	5.772761	6.5220695		chr1:182151875-182151816	
A_23_P85171	6.513415	6.365798	NM_000117	chrX:153609536-153609595	EMD
A_32_P15706	6.1312428	6.3226027	XM_005271795	chr7:55813103-55813044	LOC101928755
A_23_P210482	9.328993	9.641163	NM_000022	chr20:43248250-43248191	ADA
A_23_P35045	2.3623235	2.3900566	NM_001025598	chr1:161017294-161017235	ARHGAP30
A_23_P163235	2.3221061	2.3900566	NM_001015001	chr15:43988289-43988463	CKMT1A
A_33_P3252048	7.790366	6.767065	NM_001007533	chr17:79791439-79791380	PPP1R27
A_24_P378987	8.614557	9.094298	NM_145177	chrX:2138938-2138879	DHRSX
A_24_P357037	9.780453	9.68858	NM_182688	chr21:46189762-46189703	UBE2G2
A_24_P226008	11.731224	11.879787	NM_007283	chr3:127413832-127411122	MGLL
A_24_P278299	8.307688	8.642183	NM_024701	chr10:5681088-5681029	ASB13
A_33_P3267562	6.3702974	6.0178223	NM_012476	chr2:71160514-71160573	VAX2
A_23_P201596	6.6629696	6.1896586	NM_004037	chr1:110173915-110173974	AMPD2
A_23_P18684	9.397934	8.990242	NM_004362	chr4:141310434-141310375	CLGN
A_23_P310460	2.3221061	2.3900566	NM_153487	chr6:37600354-37600295	MDGA1
A_24_P286114	8.5928135	8.298032	NM_004172	chr5:36688204-36688263	SLC1A3
A_23_P100676	5.5834236	5.0285726	NM_017575	chr17:1963593-1963534	SMG6
A_33_P3326545	6.46863	6.5830164	NM_182492	chr22:25747638-25747579	LRP5L
A_33_P3210885	3.8260193	3.0852613	NM_001282224	chrX:77227198-77227257	ATP7A
A_33_P3238944	4.405824	4.8298655		chr7:142494285-142494226	
A_33_P3364661	6.7889667	6.8848925	DC392227	chr3:49449897-49449838	RHOA
A_23_P333218	9.222533	9.088139	NM_001031711	chr5:172379624-172379683	ERGIC1
A_33_P3297040	2.6624503	3.576063	NM_021635	chr6:138539184-138539125	PBOV1
A_23_P143334	2.7851942	3.2239923	BC126936	chr20:13982999-14032575	MACROD2
A_33_P3815064	4.625038	4.7962537	XR_172429	chr14:77535783-77535842	LOC283575
A_33_P3213288	4.873261	4.9949713	NM_004847	chr6:31584267-31584326	AIF1
A_23_P22614	6.58377	6.660585	NM_145802	chrX:118763459-118763400	SEPT6
A_33_P3369790	4.5524106	4.735596	NM_152657	chr19:38877394-38877335	GGN
A_23_P218892	8.622114	8.569899	NM_182917	chr3:184049566-184049761	EIF4G1
A_23_P316612	7.224336	7.2313833	NM_147193	chr1:53971989-53971930	GLIS1
A_33_P3380071	5.243183	5.340315	NR_038454	chr7:32496788-32496729	LOC100130673
A_23_P722	5.2483006	5.1060743	NM_003176	chr1:115537395-115537548	SYCP1
A_23_P159305	9.542791	9.394786	NM_139215	chr17:34173994-34174053	TAF15
A_33_P3269588	8.344757	8.321356	NM_002040	chr21:27144188-27144247	GABPA
A_33_P3353030	6.883378	6.2865562	NM_003353	chr2:27530324-27530265	UCN
A_24_P131522	7.915614	7.561368	NM_032208	chr2:69476125-69476184	ANTXR1
A_24_P158421	9.491617	8.7585335	NM_020150	chr10:71910538-71910479	SAR1A
A_23_P128728	6.2198424	5.8691053	NM_001172	chr14:68117539-68117598	ARG2

A_24_P383581	9.418226	9.465176	NM_205767	chr19:5679386-5678631	C19orf70
A_33_P3219527	5.207198	5.0567975	XM_005269610	chr10:63944140-63944081	RTKN2
A_23_P158096	11.020546	11.046217	AK021957	chr9:117073738-117073797	COL27A1
A_23_P86570	10.724686	10.625783	NM_004034	chr10:75135371-75135312	ANXA7
A_24_P201171	4.860474	4.2705092	NM_003165	chr9:130454192-130454251	STXBP1
A_33_P3269598	7.258717	7.113491	XM_005252327	chr9:98734804-98734863	ERCC6L2
A_23_P363831	6.039513	6.353525	NM_033406	chr11:33768847-33768788	FBXO3
A_32_P141418	3.8496578	3.4768047	NM_018076	chr10:28101158-28101099	ARMC4
A_33_P3382910	4.641821	4.638547	NM_017676	chr5:102440372-102440313	GIN1
A_33_P3424272	11.15578	11.399037	NM_001083601	chr16:3536894-3536953	NAA60
A_23_P47181	5.297611	5.4386253	NM_130769	chr11:64702516-64702341	GPHA2
A_24_P897062	7.3945017	7.660906	NM_001170905	chr7:63809153-63809212	ZNF736
A_33_P3348244	9.414466	9.589754	NM_015379	chr7:97920744-97920803	BRI3
A_33_P3265355	11.985635	11.85593	NM_003094	chr1:203839075-203839134	SNRPE
A_33_P3412479	7.7610626	7.9150987	NM_182639	chr10:100189352-100189293	HPS1
A_23_P27983	4.319514	4.0580378	NM_005166	chr19:36367423-36367482	APLP1
A_33_P3279276	4.3240037	4.227474	NM_001080423	chr3:14536457-14536398	GRIP2
A_32_P6015	6.035398	6.410648	NM_005515	chr7:156797714-156797655	MNX1
A_33_P3310674	10.713688	10.616875	NM_181838	chr5:139007208-139007267	UBE2D2
A_24_P272352	8.502752	8.5788		chr19:020369737-020369796	
A_33_P3278659	5.9341984	5.3945556	AB128832	chr6:68598273-68598214	
A_33_P3868357	6.5179195	6.408836	DQ323997	chr5:23951852-23951911	
A_33_P3384825	10.545138	10.6714325	NM_015343	chr17:7147064-7147005	CTDNEP1
A_33_P3300817	15.041382	15.005991	NM_001009	chr19:58906104-58906163	RPS5
A_33_P3762918	4.942854	4.6820316	NR_039981	chr7:104622285-104622226	LINC01004
A_23_P159255	9.0948925	9.609042	NM_002845	chr18:8406398-8406457	PTPRM
A_23_P16762	11.174517	11.147322	NM_017892	chr2:153512882-153512823	PRPF40A
A_33_P3277361	3.9504328	3.8333662	NM_001199770	chr2:63280119-63280178	OTX1
A_33_P3297205	7.7781396	7.392498	NM_024627	chr22:19833728-19833669	C22orf29
A_23_P31224	4.1647277	4.3153605	NM_178273	chr7:99971912-99971971	PILRA
A_23_P93360	5.9515257	6.168247	NM_001136	chr6:32148875-32148816	AGER
A_33_P3209541	4.224139	3.8499022	NR_024207	chr8:145172994-145173053	KIAA1875
A_24_P293114	6.045348	6.2364454	NM_020971	chr19:41082026-41082085	SPTBN4
A_33_P3842770	4.2301226	3.8614836	BC022881		LOC644450
A_33_P3268863	6.0462394	6.113184	NM_001271983	chr11:130584203-130584262	C11orf44
A_24_P67929	5.853154	6.155717	NM_017822	chr12:49063025-49062966	KANSL2
A_24_P142269	5.051601	4.6188765	NM_003609	chr16:30005325-30005266	HIRIP3
A_23_P419602	7.4232044	7.698067	NM_004599	chr22:42302199-42302258	SREBF2
A_24_P320171	5.060019	5.435765	NM_152544	chr4:8470009-8470068	TRMT44
A_24_P160874	10.331777	10.160134	NM_001025248	chr15:48633737-48634231	DUT
A_24_P202717	7.341049	7.6190047	NM_018049	chr19:2234154-2234007	PLEKHJ1
A_23_P35467	11.45044	11.021915	NM_018464	chr10:60047439-60047498	CISD1
A_23_P156289	9.326044	10.204594	NM_003999	chr5:38933387-38933446	OSMR
A_23_P307502	3.86843	3.7179508	NR_024274	chr9:21967537-21967596	C9orf53
A_23_P203173	3.167839	2.3900566	NM_001558	chr11:117872070-117872129	IL10RA
A_33_P3259017	10.227076	10.608243	NM_001319	chr19:1980390-1980449	CSNK1G2
A_33_P3323742	7.9868984	9.16902	NM_017761	chr1:24288288-24288347	PNRC2
A_23_P128624	11.5660715	11.216723	NM_024537	chr13:111293837-111293778	CARS2
A_23_P137423	5.3080735	4.6372766	NM_052868	chr1:160061655-160061397	IGSF8
A_23_P67466	11.55609	11.11721	NM_002812	chr19:38871613-38872789	PSMD8
A_33_P3249976	2.7567668	2.3900566	NM_021219	chr21:27087158-27087217	JAM2
A_23_P385938	7.0225463	7.5464706	NM_175854	chr13:28868784-28868843	PAN3
A_33_P3379436	15.878998	15.945585	NR_026802	chr9:65488178-65488119	FAM74A4
A_33_P3398998	6.9493933	7.003071	NM_182500	chr2:11286837-11286896	C2orf50
A_23_P213199	9.148009	9.1674185	NM_001031723	chr4:100822095-100822036	DNAJB14
A_33_P3220728	4.0659504	4.2719517	NM_001003699	chr6:7251136-7251195	RREB1
A_24_P83544	10.690393	10.626795		chr10:010216380-010216439	
A_33_P3663705	7.128085	7.2329884	NM_014675	chr1:17299343-17299402	CROCC
A_33_P3331776	7.364299	7.459077		chr20:042894221-042894280	

A_33_P3397323	6.252349	6.373664	NM_152355	chr19:11894786-11894845	ZNF441
A_24_P156113	8.908539	9.074278	NM_014601	chr19:48246224-48246283	EHD2
A_24_P16913	6.553556	6.9296093	NM_005845	chr13:95815416-95813571	ABCC4
A_24_P136161	7.4842796	7.4506154	NM_001013631	chr1:12908069-12908010	HNRNPCL1
A_23_P373927	5.425268	5.021414	NM_015051	chr9:102741776-102741717	ERP44
A_33_P3302676	5.287931	5.5030675		chr9:82242603-82242662	TLE4
A_33_P3331906	6.5489855	6.934184	NM_006750	chr16:69279658-69279717	SNTB2
A_33_P3325935	10.245322	10.084971	NM_001167989	chrX:48760354-48760413	PQBP1
A_33_P3423830	4.5986233	4.8927026	NM_014719	chr7:143558339-143558280	FAM115A
A_32_P194246	8.003629	7.690025	NM_015226	chr16:11275765-11275824	CLEC16A
A_23_P147199	8.112175	8.257775	NR_024565	chr18:32887664-32887723	ZNF271
A_33_P3325349	9.988388	9.704455	NM_005723	chr4:99393471-99393412	TSPAN5
A_33_P3282688	6.625506	6.512019	XR_250425	chr8:146032346-146032287	LOC100130027
A_33_P3376017	5.1282563	4.590456	AB209470	chr1:36035475-36035416	
A_23_P897	3.7694116	3.325213	NM_023938	chr1:207192288-207192229	C1orf116
A_24_P411121	3.651384	3.9420872	NM_148901	chr1:1140761-1139819	TNFRSF18
A_23_P45560	3.0005288	3.391079	NM_000273	chrX:9693758-9693699	GPR143
A_23_P6490	7.840811	7.957975	NM_015088	chr22:40719222-40719281	TNRC6B
A_23_P308032	7.708162	7.4925723	NM_005387	chr11:3733249-3733190	NUP98
A_23_P4885	4.3444448	4.51039	NM_014203	chr19:50309154-50309390	AP2A1
A_33_P3266783	3.8330789	4.1775837		chr17:65992314-65992255	
A_24_P239606	9.11738	8.478773	NM_015675	chr19:2478178-2478237	GADD45B
A_33_P3391345	3.5620682	3.9855142	XR_254054	chr14:19685716-19685775	
A_33_P3353941	4.606481	4.030805	NM_005026	chr1:9787045-9787104	PIK3CD
A_23_P120566	9.791756	9.848531	NM_001042576	chr20:17597476-17597419	RRBP1
A_24_P129588	8.110642	8.340187	NM_181745	chr10:95347228-95347287	FFAR4
A_23_P256663	8.189287	8.295565	NM_003614	chr22:38221326-38221385	GALR3
A_24_P48162	9.136179	8.968014	NM_002434	chr16:133183-133242	MPG
A_23_P201808	8.630661	8.520672	NM_003713	chr1:56960899-56960840	PPAP2B
A_33_P3250595	4.3449144	3.702842	NM_001001132	chr21:35195841-35195900	ITSN1
A_24_P13230	8.248929	8.514343	NM_002869	chr11:73387372-73387313	RAB6A
A_23_P9392	4.2079325	3.924283	NM_016390	chr9:131586099-131586040	C9orf114
A_23_P42045	10.563667	10.632851	NM_181837	chr6:88376916-88376975	ORC3
A_33_P3348288	5.847641	6.2722654	NM_001242359	chr10:62629276-62629217	RHOBTB1
A_33_P3343690	6.0811105	5.758689	NM_004109	chr11:110335436-110335495	FDX1
A_32_P224666	8.433479	7.8010387	NM_006136	chr7:116558522-116558580	CAPZA2
A_23_P148546	9.755492	9.636728	NM_003491	chrX:153197807-153197548	NAA10
A_33_P3342305	7.107397	6.3543053	NM_007168	chr17:66920902-66920843	ABCA8
A_33_P3325748	5.5710516	5.425033	NM_138632	chr22:38155900-38155959	TRIOBP
A_33_P3412945	9.346716	9.467321	NM_003016	chr17:74732291-74732232	SRSF2
A_24_P169574	7.215926	7.055211	NR_029404	chr7:63894868-63894928	LOC649395
A_23_P335452	8.308677	7.8147993	NM_153367	chr10:81142503-81142444	ZCCHC24
A_23_P22224	11.9127655	11.587203	NM_004095	chr8:37917718-37917777	EIF4EBP1
A_23_P65466	5.286751	5.5610404	NM_032846	chr14:21927987-21927928	RAB2B
A_23_P17065	2.3221061	2.3900566	NM_004591	chr2:228681100-228681836	CCL20
A_23_P116890	6.309342	6.4163766	NM_006249	chr12:11420537-11420478	PRB3
A_33_P3325502	5.10911	4.9641013	NM_004815	chr1:94634524-94634465	ARHGAP29
A_33_P3400248	2.5371668	2.3900566	NM_019851	chr8:16850546-16850487	FGF20
A_33_P3404508	5.698203	5.532972	NM_001144027	chr9:140100702-140100761	NDOR1
A_23_P321913	4.9052334	4.318839	NM_022047	chr6:35288682-35288741	DEF6
A_23_P216966	6.6378703	6.7149825	NM_000962	chr9:125155052-125155111	PTGS1
A_24_P357468	3.8620996	4.005909	D13077	chr14:22315300-22315359	
A_32_P175301	8.619221	8.756094	NM_014957	chr8:142205730-142205789	DENND3
A_23_P89755	9.797848	9.944344	NM_016271	chr18:29710321-29710380	RNF138
A_24_P321511	8.097334	7.603293	NM_016072	chr12:21670468-21670527	GOLT1B
A_23_P44663	3.96212	4.02719		chr14:94832572-94832513	XLOC_014512
A_33_P3360530	4.7337556	4.561072		chr2:228734906-228734847	
A_23_P54079	9.878318	9.818062	NM_017807	chr14:20915409-20915350	OSGEP
A_33_P3359160	10.533703	10.754381	NM_001267608	chr1:155217246-155217187	FAM189B

A_33_P3415663	7.1632843	6.846971	NM_203406	chr5:89754201-89754142	MBLAC2
A_23_P334173	2.3221061	2.3900566	NM_002349	chr2:160660247-160660188	LY75
A_33_P3297003	6.8088727	6.6596212	NR_027451	chr20:34635261-34635202	LINC00657
A_33_P3325011	13.279962	13.403207		chr12:101818354-101818413	
A_23_P62881	4.527561	4.603182	NM_032291	chr1:67210099-67210158	SGIP1
A_33_P3402188	4.6511917	3.8760998	AF116728	chr12:11230356-11230297	
A_33_P3420635	5.315325	5.231988	NM_001004708	chr11:59225263-59225322	OR4D6
A_33_P3247082	7.7843733	7.990855	NR_003083	chr16:32894869-32894810	SLC6A10P
A_23_P43150	8.167171	7.427164	NM_001017926	chr8:124260678-124260657	ZHX1
A_33_P3780901	6.380311	6.078019	NM_001167856	chr12:123776783-123776724	SBNO1
A_23_P19004	4.9605675	5.0002646	NM_023924	chr5:864507-864448	BRD9
A_33_P3383261	8.109037	7.862271	NM_001102396	chr1:115316203-115316144	SIKE1
A_23_P335428	6.8412147	7.119712	NM_152740	chr7:27565813-27565754	HIBADH
A_23_P67913	5.07876	4.88456	NM_013335	chr2:220367157-220368857	GMPPA
A_24_P382319	2.5761263	2.3900566	NM_001712	chr19:43011563-43011504	CEACAM1
A_33_P3394198	5.7456594	5.6684875	NM_004957	chr9:130575644-130575703	FPGS
A_33_P3884005	4.1410036	3.923818	AK000175	chr15:70099234-70099293	LOC93444
A_33_P3417542	4.4606323	4.4471703		chr5:093929268-093929327	
A_23_P336554	4.4450827	4.3979855	NM_134470	chr3:190347694-190347753	IL1RAP
A_33_P3307910	3.6208007	3.82745		chr2:98947963-98947904	
A_33_P3394075	7.885379	8.093032	NM_006912	chr1:155867726-155867667	RIT1
A_24_P68819	7.1096416	7.4957895	NM_152994	chr2:48738541-48738600	PPP1R21
A_23_P396328	7.2576957	7.2679734	NM_003829	chr9:13105942-13105883	MPDZ
A_24_P313822	5.870092	5.77351	NM_005884	chr19:39668337-39668396	PAK4
A_23_P8913	9.509018	10.897618	NM_000067	chr8:86393210-86393269	CA2
A_24_P921933	7.7665563	7.3934693	NM_001078166	chr17:56078449-56078390	SRSF1
A_24_P339126	4.126025	3.9072173	NM_144957	chr16:2871000-2871059	PRSS21
A_23_P99397	5.935665	5.8920527	NM_153251	chr13:21950644-21950585	ZDHHC20
A_33_P3364089	6.2283363	5.8679338	NM_019844	chr12:21036476-21036535	SLCO1B3
A_23_P168788	10.354082	10.763852	NM_001084	chr7:100849487-100849428	PLOD3
A_23_P215875	12.37811	12.2197	NM_015420	chr8:104453758-104453817	DCAF13
A_24_P101629	6.013812	6.430058	NM_001078172	chrX:134186043-134185984	FAM127B
A_33_P3259740	5.3760595	5.2024612	BC119774	chr18:74402401-74402460	FLJ44881
A_24_P481375	7.066556	7.386058	NR_034089	chr1:93803884-93803825	LOC100131564
A_33_P3337154	5.0522685	4.616888		chr20:001871766-001871707	
A_33_P3326225	2.3221061	2.3900566	NM_001101676	chr8:119201758-119201699	SAMD12
A_24_P400376	11.769716	11.3519335	NM_016139	chr1:015931599-015931658	CHCHD2
A_24_P273245	7.9416413	8.209272		chr3:076484244-076484303	
A_23_P251562	8.347727	8.70266	NM_007275	chr3:50362447-50362388	TUSC2
A_24_P42066	6.0856667	6.216202	NR_003267	chr22:18761240-18761202	GGT3P
A_23_P46812	5.536691	6.305276	NM_014912	chr10:93809440-93809381	CPEB3
A_23_P147439	5.373126	5.179369	NM_017492	chr16:28840779-28841203	ATXN2L
A_23_P25073	10.766037	9.809087	NM_021821	chr12:27909106-27909165	MRPS35
A_32_P120638	4.8720303	4.7732296	NM_033547	chr11:77635895-77635836	INTS4
A_33_P3224055	5.179677	5.4027543	AK130567	chr3:126426717-126426776	LOC100132481
A_33_P3449417	12.531344	12.471622	NM_001130089	chr16:75661852-75661793	KARS
A_24_P72518	7.125363	6.584361	NM_015328	chr7:129069898-129069957	AHCYL2
A_23_P13899	14.293259	14.322374	NM_002046	chr12:6646918-6646977	GAPDH
A_24_P116606	6.748188	6.4535213	NM_130791	chr16:78143698-78148897	WWOX
A_33_P3327250	10.621693	10.441656		chr18:029671313-029671254	
A_33_P3341259	4.6139517	4.5266914	AJ409065	chr5:132209315-132209374	LEAP2
A_24_P289188	11.606536	11.606536		chr15:71089345-71089286	
A_24_P331373	4.4886646	4.435634	NM_031444	chr22:24939053-24938994	GUCD1
A_23_P41818	9.957651	9.923998	NM_016107	chr5:32354917-32354858	ZFR
A_33_P3375140	4.851436	2.9597025	AK023946	chr8:6505948-6506007	
A_33_P3232047	13.060701	13.054146	NM_030969	chr6:10757032-10757091	TMEM14B
A_24_P238543	3.8898704	3.120441	NM_002637	chrX:71925089-71915710	PHKA1
A_23_P500601	7.7530303	7.837289	NM_033017	chr7:99488740-99488681	TRIM4
A_23_P6596	4.989251	5.113686	NM_005524	chr3:193854276-193854463	HES1

A_23_P144369	6.4710865	6.9272757	NM_153757	chr4:89617523-89617464	NAP1L5
A_33_P3474175	5.736352	5.6615105	BU601128		SFTA1P
A_33_P3271990	7.936174	7.676975	NM_012079	chr8:145538311-145538252	DGAT1
A_23_P252236	4.65869	3.5656025	NM_000892	chr4:187178445-187178504	KLKB1
A_23_P338401	5.450593	4.8434606	NM_153042	chr6:18222571-18222630	KDM1B
A_24_P114339	5.00903	5.1159587	NM_022780	chr2:87004230-87004289	RMND5A
A_23_P201400	8.109358	8.228674	NM_000309	chr1:161140844-161140903	PPOX
A_24_P84781	4.717512	4.696085	AK021933	chr7_gl000195_random:31018-30959	
A_23_P258944	7.4604635	7.6947494	NM_012328	chr7:108214515-108214574	DNAJB9
A_23_P95764	10.636497	10.523773	NM_002764	chrX:106893857-106893916	PRPS1
A_23_P317324	8.311636	8.374796	NM_005241	chr3:168806898-168806839	MECOM
A_23_P52676	8.00027	7.3686237	NM_053054	chr11:65784573-65784371	CATSPER1
A_33_P3392213	6.671459	6.994342		chr14:053414072-053414013	
A_23_P215048	6.919092	6.569267	NM_014702	chr6:127764999-127764940	KIAA0408
A_33_P3408943	10.203657	9.8630295	NM_024834	chr10:121590857-121590798	MCMBP
A_33_P3214199	5.633006	4.7481575	NM_018181	chr18:56652368-56652427	ZNF532
A_33_P3367565	10.207284	9.955585	XM_005251815	chr9:132658152-132658093	FNBP1
A_24_P142495	10.268453	10.468208	NM_030966	chr17:39190648-39190589	KRTAP1-3
A_33_P3411307	4.595602	4.3622527	AK128269	chr16:11158270-11158329	LOC100130276
A_24_P332971	7.385304	7.3618717	NM_198486	chr6:42854018-42854077	RPL7L1
A_24_P73389	5.4799333	5.626514	NM_001032296	chr13:99114112-99114053	STK24
A_23_P355385	9.948938	9.939497	NM_002721	chr9:127911554-127911495	PPP6C
A_33_P3411388	7.194565	7.024693	NM_001080461	chr7:1276434-1276493	UNCX
A_24_P191847	4.255159	4.0471287	NM_024754	chr5:71648475-71648534	PTCD2
A_33_P3467126	6.477091	6.409606	NM_033107	chr7:90014818-90014877	GTPBP10
A_23_P43613	11.470644	11.501098	NM_182739	chr9:32572889-32571002	NDUFB6
A_23_P151209	7.705762	7.72425	NM_030809	chr12:51455350-51455291	CSRNP2
A_23_P102462	4.531614	4.9162817	NM_153214	chr2:112945026-112945085	FBLN7
A_33_P3213374	5.6365705	5.8481092	NM_006079	chr6:139694671-139694612	CITED2
A_33_P3247237	3.5986586	3.9519958	NM_001131015	chr9:130943088-130943029	CIZ1
A_24_P392230	6.4809527	6.476227		chr7:121874783-121874842	
A_23_P25155	3.9857194	3.4689126	NM_020370	chr12:54756423-54756364	GPR84
A_23_P336992	7.6229615	7.47064	NM_182491	chr7:1197349-1195219	ZFAND2A
A_33_P3334826	5.283169	5.588937	NR_033267	chr12:49783707-49783766	LOC100335030
A_24_P132518	8.094112	8.089643	NM_001556	chr8:42189442-42189501	IKBKB
A_33_P3270628	5.6549926	5.5118265	BE468260	chr1:28265447-28265388	
A_23_P14708	6.968915	7.127136	NM_017661	chr15:56923077-56923018	ZNF280D
A_23_P393880	2.3221061	3.0827966	NM_020340	chr6:138659313-138659372	KIAA1244
A_23_P413285	6.1850634	6.2803955	NM_001080522	chr4:15601242-15601301	CC2D2A
A_24_P405430	8.1086855	8.00534	NM_022173	chr2:70436757-70436698	TIA1
A_33_P3334743	3.8216333	4.0619726	NM_014247	chr4:160263014-160263073	RAPGEF2
A_33_P3211263	9.571092	9.256844	NM_182679	chr1:156564838-156564779	GPATCH4
A_24_P179013	6.873208	6.53583		chr6:003045650-003045709	
A_33_P3736195	5.220851	4.902193	BX647230	chr10:135263173-135263232	DKFZp686M1136
A_33_P3374205	6.608905	6.849412	NM_002417	chr10:129913252-129913193	MKI67
A_32_P198810	5.493146	5.2287993		chr12:12509354-12509295	LOH12CR2
A_23_P209652	10.573208	10.795741	NM_017759	chr2:206869668-206869609	INO80D
A_23_P5131	6.2175555	6.2580824	NM_016368	chr19:18545827-18545768	ISYNA1
A_33_P3211153	8.755485	8.7391205	NM_133450	chr16:4746572-4746513	ANKS3
A_23_P153441	5.782801	6.049731	NM_013312	chr19:12874382-12874227	HOOK2
A_24_P399500	5.1429176	5.0401444	NM_174895	chr19:7696581-7696522	PCP2
A_23_P22672	7.6670737	7.38492	NM_001257231	chrX:111003779-111003838	ALG13
A_33_P3270639	3.6491566	3.7130635		chr15:020193528-020193469	
A_23_P98605	11.037768	10.892973	NM_015853	chr11:62444103-62444044	UBXN1
A_33_P3321369	7.1800604	7.305318	XR_109231	chr15:101414136-101414195	LOC145757
A_23_P77328	4.185155	4.250116	NM_005258	chr15:41059510-41059569	GCHFR
A_23_P89062	5.3723493	4.9992476	NR_024034	chr16:31718232-31718291	CLUHP3
A_33_P3238166	11.287387	11.844175	NM_012293	chr2:1635717-1635659	PXDN

A_23_P132793	11.719934	11.458673	NM_006010	chr3:51426687-51426746	MANF
A_23_P72961	7.3686237	7.3566103	NM_002764	chrX:106893427-106893487	PRPS1
A_33_P3221665	4.045376	3.5184593	NM_001252102	chr1:200943908-200943849	KIF21B
A_23_P50426	11.857566	11.885905	NM_015493	chr19:11275104-11275045	KANK2
A_33_P3368555	4.0110073	3.7832932	AK091998	chr2:61415552-61415611	AHSA2
A_23_P503072	2.714815	2.3900566	NM_148672	chr5:43381984-43381925	CCL28
A_24_P203814	7.621482	7.6560106		chr6:044057362-044057303	
A_33_P3254311	4.219212	4.0644126	U79183	chr7:142510328-142510271	
A_23_P66563	7.1616483	7.297796	NM_018149	chr17:57290836-57290895	SMG8
A_23_P163306	10.241552	10.354605	NM_032866	chr15:57842847-57842906	CGNL1
A_24_P93967	7.74392	7.951111	NM_002024	chrX:147014095-147014243	FMR1
A_23_P322043	9.13384	8.476041	NM_015327	chr1:156219354-156219295	SMG5
A_23_P29855	10.642863	10.786055	NM_003715	chr4:76734902-76734961	USO1
A_23_P44956	14.93526	14.815807	NM_000996	chr3:197680988-197682649	RPL35A
A_23_P428260	6.348966	5.761147	NM_152999	chr7:89866537-89866596	STEAP2
A_23_P120435	5.650811	5.692346	NM_080614	chr20:44404120-44404061	WFDC3
A_24_P379512	7.5969253	7.560105	NM_005482	chr1:77554976-77554917	PIGK
A_24_P109214	8.150106	7.9582644	NM_001645	chr19:45422436-45422495	APOC1
A_23_P372874	13.373993	13.298234	NM_001024210	chr1:153591344-153591285	S100A13
A_23_P202860	7.8556585	7.9811254	NM_024514	chr11:14899691-14899632	CYP2R1
A_33_P3276693	9.488722	9.084606	NM_002632	chr14:75408599-75408540	PGF
A_33_P3249076	6.08483	6.1721883	AK094791	chr10:99360404-99360463	HOGA1
A_23_P216693	7.1261697	7.2484536	NM_004529	chr9:20346539-20346480	MLLT3
A_23_P12620	5.8020043	5.0898814	NM_025235	chr10:93624141-93624200	TNKS2
A_23_P129629	9.581428	9.811309	NM_005954	chr16:56624933-56624991	MT3
A_23_P313476	4.543979	4.356299	NM_032448	chr6:170697485-170697544	FAM120B
A_24_P398810	7.4917502	6.9958844	NM_001969	chr14:103811176-103811235	EIF5
A_33_P3229472	9.120375	8.357157	NM_001012710	chr11:71277283-71277342	KRTAP5-10
A_23_P128323	3.8566804	3.2638743	NM_001038	chr12:6456735-6456676	SCNN1A
A_23_P7101	11.086327	10.463749	NM_006527	chr4:1695032-1694973	SLBP
A_23_P215253	9.819782	9.865618	XM_005250467	chr7:102210334-102207524	
A_23_P319557	4.5100217	3.944608		chr21:46663468-46663527	
A_33_P3282698	3.9837308	3.789216		chr22:23412881-23412940	GNAZ
A_32_P221748	5.86318	5.9428253	BX350880	chr1:226259454-226259395	
A_24_P267686	5.572234	5.4593124		chr22:019005666-019005607	
A_23_P47924	2.3221061	2.3900566	NM_002849	chr12:71032555-71032496	PTPRR
A_24_P818529	4.5853877	4.4486923	Z48511	chrY:2658164-2658223	
A_33_P3319113	3.6082997	2.3900566	NM_017990	chr16:70164384-70164443	PDPR
A_23_P150935	10.256865	10.441019	NM_005480	chr12:49725416-49725475	TROAP
A_32_P88905	6.6277785	6.4730377		chr13:049549271-049549212	
A_23_P154330	10.262342	10.157155	NM_005783	chr2:99935957-99935898	TXNDC9
A_33_P3384997	15.434727	15.415177		chr13:027591747-027591688	
A_23_P86100	4.8994846	5.178631	NM_001007255	chr1:161070036-161070095	KLHDC9
A_33_P3741678	3.498028	3.8038797		chr22:046649075-046649134	
A_32_P6172	6.470277	6.1881924	NR_027387	chr7:152162443-152162502	LINC01003
A_33_P3283669	3.9769983	3.9925227	NM_001256214	chr19:42470805-42470746	ATP1A3
A_23_P436048	4.2708426	4.1644316	NM_012152	chr1:85331185-85331126	LPAR3
A_33_P3702104	8.827332	8.728009	NR_073400	chr1:62120876-62120935	MGC34796
A_33_P3222018	8.83892	8.9854145	NM_007276	chr7:26248079-26248138	CBX3
A_23_P17430	5.214887	4.951394	NM_017495	chr20:55983622-55983681	RBM38
A_33_P3303729	5.5493298	5.848875		chr7:56559837-56559896	XLOC_014512
A_23_P76364	8.407822	8.879908	NM_001769	chr12:6342625-6344426	CD9
A_23_P45396	10.788362	11.061378	NM_004493	chrX:53458440-53458381	HSD17B10
A_24_P226355	5.8867636	5.627821	NM_005493	chr6:13639812-13638146	RANBP9
A_33_P3244818	8.161596	8.3874035	NM_198467	chr7:77408652-77408711	RSBN1L
A_24_P795371	5.0746336	5.2760625	NR_102743	chr15:96809614-96809604	NR2F2-AS1
A_33_P3419720	10.392603	10.1623745	NM_000249	chr3:37092085-37092144	MLH1
A_32_P75661	4.5493793	4.9521637		chr2:010908054-010907995	
A_24_P63799	6.0793085	5.855679	NM_018292	chr6:107115749-107115808	QRSL1

A_33_P3309999	6.6686845	6.7707434	NM_001013628	chrX:125298629-125298570	DCAF12L2
A_33_P3280030	6.5391474	6.4323444	NM_145246	chr10:95441298-95441239	FRA10AC1
A_24_P920048	4.1202407	2.3900566	AK092807	chr19:35658450-35658509	LOC100127972
A_33_P3659678	6.8425665	6.972079	NM_033334	chr9:127280335-127280276	NR6A1
A_33_P3278451	3.8535075	2.3900566	DA717721	chr19:28785183-28785124	
A_24_P53051	7.114929	7.3092885	NM_171846	chr15:63421741-63421800	LACTB
A_33_P3236030	2.3221061	2.3900566	AK130576	chr4:86643083-86643142	ARHGAP24
A_23_P362712	4.2893443	4.319514	NM_001145364	chr7:73152744-73152685	ABHD11
A_33_P3336514	5.595883	5.3159947	XM_005255018	chr16:29499404-29499345	
A_33_P3390017	2.9953513	2.3900566	NM_207445	chr15:39546941-39547000	C15orf54
A_23_P71904	9.366944	9.155772	NM_014064	chr9:132396425-132396484	NTMT1
A_23_P146058	10.171531	10.3456335	NM_001695	chr8:104081415-104081474	ATP6V1C1
A_33_P3236651	10.876233	10.873119	NM_145185	chr19:7979302-7979361	MAP2K7
A_32_P229746	9.969467	10.212109	NM_005494	chr7:157178663-157178722	DNAJB6
A_23_P28733	6.9337826	6.854637	NM_002895	chr20:35627256-35627197	RBL1
A_23_P165148	8.260529	8.341716	NM_006844	chr19:15226024-15225965	ILVBL
A_23_P393620	8.273557	7.9136724	NM_006528	chr7:93516634-93516575	TFPI2
A_33_P3350823	6.1778955	5.908371	NM_016331	chr3:179053245-179053304	ZNF639
A_33_P3241051	3.3344874	2.3900566	NM_139057	chr15:100511813-100511754	ADAMTS17
A_33_P3359724	6.4238467	6.684575	BG717688	chr1:109649379-109649320	C1orf194
A_33_P3253574	4.559529	4.4044957	NM_001005467	chr11:124266436-124266377	OR8B3
A_23_P78053	7.1529408	7.125857	NM_030802	chr17:47787877-47787818	FAM117A
A_23_P154338	5.954315	6.6570716	NM_025202	chr2:233547145-233547204	EFHD1
A_23_P255215	6.3543053	6.0817533	NM_203288	chr7:33134850-33134791	RP9
A_24_P419177	5.517792	5.7180953	NM_014497	chr2:71597130-71607405	ZNF638
A_33_P3386062	5.394251	5.352765		chr14:87388700-87388759	LOC283585
A_23_P116435	6.112181	6.4134684	NR_028044	chr11:2169765-2169824	IGF2-AS
A_23_P129169	3.8134727	4.358273	NM_000781	chr15:74631077-74631018	CYP11A1
A_33_P3229357	5.2235374	5.2666726		chr7:57289113-57289054	
A_24_P339153	5.1380353	5.4194136	NM_032038	chr16:28994184-28994243	SPNS1
A_23_P75921	6.543825	6.634968	NM_145803	chr11:36511240-36511181	TRAF6
A_32_P159535	8.251291	8.534092		chr3:015173477-015173418	
A_33_P3386686	6.967745	7.039104	XM_005273698	chr9:45120416-45120475	LOC100132874
A_24_P940149	5.70728	6.4826727	NM_015500	chr21:43306092-43306033	C2CD2
A_24_P341089	7.3982754	7.193535		chr2:89100699-89100758	ANKRD36BP2
A_23_P251660	9.183765	9.463923	NM_001025091	chr6:30558633-30558692	ABCF1
A_23_P256694	6.0401382	5.8475103	NR_002776	chr21:47671312-47671371	MCM3AP-AS1
A_33_P3398266	6.3063374	5.6640973		chr21:43137642-43137701	LINC00112
A_23_P363399	10.734433	10.356138	NM_030674	chr12:46582066-46582007	SLC38A1
A_33_P3236310	6.846971	7.1201425	NM_001080413	chr7:144096910-144096851	NOBOX
A_33_P3369452	6.7224326	6.380311	NM_001283106	chr22:20052815-20052874	TANGO2
A_33_P3323435	7.707928	7.5431604	NM_033141	chr14:71195186-71195127	MAP3K9
A_24_P149645	12.227722	12.152462	NM_014281	chr8:144898845-144898786	PUF60
A_33_P3324149	5.335348	5.1540875		chr2:237477004-237476945	
A_23_P207650	11.349119	11.837501	NM_000018	chr17:7128439-7128498	ACADVL
A_24_P255005	6.2607627	6.098025		chr17:66122910-66122851	XLOC_014512
A_24_P366644	4.191193	4.5574427		chr1:197658626-197658685	
A_33_P3280930	13.542038	13.44387	NR_002599	chr8:67834344-67834285	SNHG6
A_23_P55802	7.492399	7.2876825	NM_032493	chr19:16345642-16345701	AP1M1
A_23_P317244	3.6260655	3.3195302	NM_173847	chr17:31323970-31324472	SPACA3
A_23_P256641	5.1014047	5.0864778	NM_012282	chrX:108867052-108866993	KCNE1L
A_23_P214544	5.3779573	5.1885986	NM_001509	chr6:28500169-28501768	GPX5
A_23_P251075	5.7434874	5.080429	NM_005491	chrX:149682217-149682276	MAMLD1
A_23_P10156	5.5990453	5.486526	NM_024591	chr17:78973508-78973567	CHMP6
A_23_P164536	8.448746	8.625264	NM_002647	chr18:39661247-39661306	PIK3C3
A_33_P3276519	6.3474927	6.2875304	NM_022372	chr16:2256542-2256601	MLST8
A_24_P280378	7.6920033	7.7504845	NM_014691	chr15:35152282-35149288	AQR
A_23_P48109	5.131476	5.114384	NM_016533	chr12:674505-674446	NINJ2
A_23_P210726	7.4785137	6.9552274	NM_021873	chr20:3786178-3786237	CDC25B

A_23_P168847	6.253949	6.3221216	NM_172366	chr8:28286038-28285979	FBXO16
A_33_P3246203	4.601367	4.413255	NR_026817		LOC148696
A_33_P3287685	10.039858	9.995915	NM_014516	chr19:54659342-54659401	CNOT3
A_23_P80954	4.7594438	4.5151305	NM_022042	chr4:982465-982406	SLC26A1
A_24_P186943	6.1467214	6.3465543	NM_000501	chr7:73474764-73474823	ELN
A_24_P406986	4.9388456	4.792676	NM_199329	chr11:57176654-57175317	SLC43A3
A_23_P500985	4.91091	4.8804665	NM_013945	chr1:19029764-19062158	PAX7
A_23_P3994	9.891387	10.199792	NM_152766	chr17:7306503-7306444	TMEM256
A_33_P3379726	8.230029	8.514037	NM_013301	chr19:56164429-56164488	CCDC106
A_33_P3305851	7.0630517	6.178317	NM_016275	chr3:150346997-150347056	SELT
A_33_P3248072	10.027787	10.0222435	NM_001135844	chr20:1578045-1577986	SIRPB1
A_23_P405531	10.140747	10.274292	NM_021241	chr19:15532996-15532937	WIZ
A_23_P53217	10.000362	9.939985	NM_152991	chr11:85989629-85989688	EED
A_23_P128706	12.448046	12.47343	NM_001376	chr14:102516525-102516820	DYNC1H1
A_33_P3341731	6.16088	6.4442563		chr11:067722814-067722755	
A_33_P3414402	6.4663606	6.261287	NM_001146190	chr18:72493398-72493457	ZNF407
A_33_P3306363	4.380759	4.5425572		chr2:233282707-233282648	
A_33_P3287547	4.3504105	4.210011		chr1:2002740-2002799	PRKCZ
A_24_P11575	7.8898096	7.9590693	NM_016441	chr2:36777827-36777886	CRIM1
A_23_P140967	6.898917	6.965535	NM_000243	chr16:3292835-3292776	MEFV
A_33_P3220020	3.7956796	3.6337686	NR_033899	chr19:22786176-22786235	LOC440518
A_23_P202427	11.188539	10.810476	NM_025130	chr10:71026958-71027017	HKDC1
A_23_P131866	10.641192	10.900652	NM_198433	chr20:54945179-54945120	AURKA
A_23_P369701	7.2905555	7.385669	NM_021214	chr15:81047339-81047398	ABHD17C
A_33_P3347697	2.3221061	2.3900566	NM_002515	chr14:26915179-26915120	NOVA1
A_23_P121326	7.1113124	7.131621	NM_007022	chr3:50391238-50391297	CYB561D2
A_33_P3399943	4.385993	4.184969	NM_021135	chr6:166912124-166912065	RPS6KA2
A_23_P38830	4.438737	4.627167	NM_024762	chr19:58319290-58319231	ZNF552
A_24_P256380	6.5388017	6.104472	NM_024911	chr1:68591687-68591628	WLS
A_33_P3280192	4.5253034	3.7052827	NM_032139	chr19:33135348-33135289	ANKRD27
A_23_P157299	2.4157925	2.3900566	NM_001129	chr7:44153822-44153881	AEBP1
A_33_P3231005	15.1805105	15.205528		chr7:097513693-097513752	
A_33_P3288074	3.7212882	3.561575	BC019017	chr14:96967398-96967339	
A_24_P181944	7.3614907	7.301172	NM_016436	chr20:34458877-34458936	PHF20
A_33_P3259973	2.3221061	2.3900566	NR_036522	chr19:57005675-57005734	ZNF667-AS1
A_32_P170397	4.067804	3.9533222	AK057625	chr15:42188025-42188084	LOC100289090
A_24_P298495	4.0162206	3.7918997	NR_046285	chr5:14714903-14714962	LOC100130744
A_33_P3237704	7.2840858	7.725437		chr9:066489339-066489280	
A_23_P7941	8.022236	7.466707	NM_015950	chr6:43022085-43022026	MRPL2
A_23_P422766	4.6364408	4.7426796	NM_172193	chr14:50219400-50219459	KLHDC1
A_32_P409222	6.0626493	5.866913	NM_033113	chr19:55995696-55995755	ZNF628
A_23_P55319	7.712204	7.801798	NM_004475	chr17:27206782-27206723	FLOT2
A_23_P117778	5.9024568	5.961508	NM_032907	chr15:74743805-74743170	UBL7
A_33_P3270019	4.306655	4.1633596	NM_006540	chr8:71036274-71036215	NCOA2
A_33_P3313055	9.713281	9.9478	NM_000435	chr19:15270548-15270489	NOTCH3
A_33_P3214879	5.637397	5.1974297	NM_002264	chr3:122140844-122140785	KPNA1
A_33_P3226202	7.4859734	7.7843733	NM_015091	chr14:45543533-45543592	FAM179B
A_33_P3364459	7.244122	7.5610423	NM_001283021	chr14:24707813-24707872	GMPR2
A_23_P2097	6.825727	6.514864	NM_018073	chr11:4620339-4620280	TRIM68
A_32_P45974	3.6673467	3.7076478	BC071972	chr2:132524902-132524960	C2orf27A
A_23_P63459	10.96133	11.019741	NM_001012985	chr1:234519591-234519650	COA6
A_33_P3286859	5.031534	4.8608847	NM_016529	chr13:26594064-26594123	ATP8A2
A_33_P3288614	4.949994	4.3262863	XR_245474	chr18:56337300-56337241	LOC101927322
A_23_P156697	4.62448	4.69787	NM_021160	chr6:31655629-31655487	ABHD16A
A_23_P149050	4.6421537	4.469102	NR_024321	chr8:183576-183635	LINC00115
A_23_P331943	9.295967	9.103541	NM_020823	chr6:159055923-159055982	TMEM181
A_33_P3363168	8.008957	8.300143	NM_001282129	chr17:27953041-27952982	SSH2
A_23_P75071	8.090094	8.29041	NM_016195	chr10:91534055-91534114	KIF20B
A_23_P355364	4.204613	4.6139517	NM_032773	chr3:197585744-197592320	LRCH3

A_23_P112512	6.4442563	6.261919	NM_033412	chr9:37887776-37887717	SLC25A51
A_33_P3307775	4.7462993	4.8335714	NM_003677	chr12:123255668-123255727	DENR
A_23_P39223	5.103204	5.4023595	NM_021088	chr2:95849536-95849595	ZNF2
A_23_P17855	6.431649	6.818852	NM_001039141	chr22:38168627-38168686	TRIOBP
A_33_P3220376	5.608911	5.103204		chr15:075614758-075614817	
A_33_P3310286	5.0986476	4.453026		chr5:126154854-126154913	
A_32_P51905	10.466264	10.526943	NM_020444	chr5:175773286-175773228	KIAA1191
A_24_P352116	7.975696	7.671232	NR_003672	chr9:139620028-139619969	SNHG7
A_33_P3337491	9.02135	8.985145	NM_001113324	chr17:73996514-73996573	TEN1
A_23_P60296	7.7494016	7.5566254	NM_012383	chr9:77761675-77761734	OSTF1
A_23_P41437	9.077531	9.127678	NM_018359	chr4:186324741-186324682	UFSP2
A_23_P59153	7.64608	7.362649	NM_021177	chr6:31766276-31765790	LSM2
A_24_P361816	4.83685	5.1653714	BC023973	chr22:22550594-22550653	
A_32_P142028	13.389293	13.071187	NM_031314	chr14:21678927-21678868	HNRNPC
A_24_P377269	11.960109	11.965001	NM_002154	chr5:132440067-132440126	HSPA4
A_23_P39814	9.201738	9.408926	NM_004882	chr2:175213308-175213249	CIR1
A_23_P70748	9.475957	9.368418	NM_031922	chr6:139226166-139226107	REPS1
A_23_P212089	7.632049	8.300486	NM_031419	chr3:101578904-101578963	NFKBIZ
A_23_P83192	11.964029	12.46171	NM_014172	chr9:139745212-139745271	PHPT1
A_33_P3413438	5.1184673	5.4522715	BX648392	chr6:31848218-31848277	
A_23_P138137	8.256505	8.504842	NM_145243	chr1:58946826-58946767	OMA1
A_24_P206758	5.880626	6.0388064	NM_013306	chr11:64807396-64807455	SNX15
A_24_P33982	7.255573	7.0371056	NM_001085423	chr17:62461517-62461576	MILR1
A_23_P422115	7.9990225	8.509039	NM_001048265	chr9:138387180-138387121	C9orf116
A_23_P211110	4.4087796	3.863355	NM_005069	chr21:38122104-38122163	SIM2
A_23_P359616	10.152378	9.686546	NM_025065	chr1:84963143-84963202	RPF1
A_33_P3381318	6.2973003	6.3119054	NM_001109977	chr4:152584588-152584647	FAM160A1
A_33_P3222069	10.160844	9.771303	NM_182965	chr17:74383867-74383926	SPHK1
A_33_P3313528	3.5867677	3.7886894	CU693037	chr17:015878053-015877994	
A_23_P304304	4.6177974	4.900886	NM_004042	chrX:3030282-3030341	ARSF
A_32_P104000	8.044764	8.135785	NM_173475	chr16:20869555-20869496	DCUN1D3
A_33_P3330236	5.0570655	4.9229097		chrX:152995095-152995154	
A_33_P3241184	5.992008	5.6610374		chr3:018581517-018581576	
A_23_P160787	10.703902	10.714432	NM_004565	chr1:10690731-10690790	PEX14
A_33_P3247624	5.573964	5.3466315	NM_001029874	chr12:27850326-27850385	REP15
A_23_P156156	8.869719	8.278589	NM_032280	chr5:80608562-80608621	ZCCHC9
A_24_P151106	2.3221061	2.3900566	NM_020828	chr19:57067376-57067435	ZFP28
A_33_P3542886	5.453352	6.1164565	BU536871		SNAR-G1
A_24_P247106	8.680506	9.244483	NM_016282	chr9:4712513-4712454	AK3
A_24_P125871	9.475307	9.495037	NM_020639	chr21:43159692-43159633	RIPK4
A_24_P46953	6.8437605	6.861288	NM_013257	chr8:67773851-67773910	SGK3
A_23_P331895	7.003581	6.7336273	NM_025250	chr7:2703574-2703633	TTYH3
A_24_P41042	6.22541	6.199301	NM_032848	chr12:113624844-113629164	C12orf52
A_24_P23951	5.5751123	5.6831512		chr22:21472590-21473126	BCRP2
A_23_P73548	6.8400455	6.801427	NM_020137	chrX:48830471-48830412	GRIPAP1
A_23_P339687	10.008818	10.383572	NM_138330	chr19:23836893-23836834	ZNF675
A_24_P296907	5.0195546	5.0324025	NM_152658	chr19:36526357-36526298	THAP8
A_23_P258272	10.092839	10.177082	NM_001039707	chr9:139297207-139297148	SDCCAG3
A_24_P354337	5.320107	5.184789	NM_032146	chr3:97506839-97506898	ARL6
A_23_P4572	12.658161	12.746756	NM_006471	chr18:3255981-3256040	MYL12A
A_33_P3268334	8.106015	8.42205	NM_002629	chr10:99190177-99190236	PGAM1
A_33_P3415678	4.195518	5.0151024		chr22:040825414-040825355	
A_23_P350187	7.6664624	8.009302	NM_001127208	chr4:106200570-106200629	TET2
A_33_P3369663	4.5775013	4.302544	NM_001171945	chr7:881707-881766	SUN1
A_33_P3406811	4.762817	4.346694		chr17:039347046-039347105	
A_33_P3856949	6.1182137	5.836693	AK096098	chr1:19540148-19540207	LOC284513
A_23_P250607	10.710045	11.0597725	NM_005032	chrX:114884316-114884375	PLS3
A_32_P831725	5.211848	4.900208	NM_198562	chr3:49306896-49306837	C3orf62
A_23_P128174	7.8712244	7.8002806	NM_175623	chr12:70209304-70209363	RAB3IP

A_33_P3274560	9.875506	9.826316		chr2:228567379-228567320	SLC19A3
A_24_P85317	4.700128	5.050267	NM_001042572	chr15:93486257-93487662	CHD2
A_23_P129358	7.845008	7.834527	NM_024860	chr16:58553249-58553308	SETD6
A_23_P79587	5.338452	5.275075	NM_001632	chr2:233247198-233247257	ALPP
A_23_P128698	9.180113	9.687826	NM_005842	chr13:80910681-80910622	SPRY2
A_33_P3340718	8.145222	8.550634	NM_021140	chrX:44971035-44971094	KDM6A
A_23_P336678	6.2502546	6.1118627	NM_145171	chr14:63779770-63779711	GPHB5
A_23_P150009	10.319694	10.493952	NM_198046	chr10:99216804-99216863	ZDHHC16
A_23_P88680	11.146063	11.1641865	NM_020154	chr15:34376399-34376340	EMC7
A_23_P134925	9.419783	9.997562	NM_004331	chr8:26270286-26270345	BNIP3L
A_33_P3286481	8.596495	8.549923		chr6:052522393-052522452	
A_24_P128683	3.732614	3.2854636	NM_015680	chr2:220037390-220037331	CNPPD1
A_23_P64232	8.922491	8.6498995	NM_003904	chr11:116649613-116649554	ZNF259
A_23_P134395	9.724951	9.743931	NM_012453	chr7:72984156-72984097	TBL2
A_33_P3229815	4.044582	4.070278		chr20:23106750-23106691	LINC00656
A_33_P3415092	6.9778767	6.7875876	NM_001127899	chrX:49863833-49863892	CLCN5
A_23_P254498	10.51021	10.27731	NM_016492	chr17:8193301-8193360	RANGRF
A_33_P3220277	5.682747	5.516529	XM_005260786	chr20:17640573-17640514	RRBP1
A_24_P154080	4.992422	5.1718807	NM_001397	chr1:21560125-21560066	ECE1
A_33_P3236703	5.884447	5.6549926	NM_001244752	chr9:33568831-33568890	ANKRD18B
A_23_P300867	4.9398	4.554378	NM_138731	chr14:37892123-37969140	MIPOL1
A_23_P136196	6.192753	5.777771	NM_018317	chr4:26756582-26756641	TBC1D19
A_33_P3346193	8.550901	8.998751	NM_001043351	chr1:154143185-154143126	TPM3
A_33_P3375476	9.824819	9.161728	NM_017566	chr16:87741478-87741419	KLHDC4
A_32_P95067	7.4980125	7.8508267	NM_001005353	chr1:65694099-65694158	AK4
A_33_P3331437	3.8513916	4.0000796	NM_001278524	chr10:97923206-97923265	ZNF518A
A_32_P62863	8.693509	8.687806	NM_014575	chr3:159615048-159615107	SCHIP1
A_33_P3271725	4.87599	4.797236	NM_025055	chr15:74627369-74627428	CCDC33
A_33_P3368560	4.382408	4.7324286	BC050395	chr2:61416001-61416060	AHSA2
A_23_P256107	5.0482645	5.300144	NM_006665	chr4:84227383-84223390	HPSE
A_23_P305692	5.4651427	5.815063	NM_153702	chr4:141471662-141471721	ELMOD2
A_24_P659036	5.2356853	5.3916025	NM_001001794	chr22:50753117-50753058	DENND6B
A_33_P3713357	11.426882	11.086327	NM_001627	chr3:105294613-105294672	ALCAM
A_33_P3277178	6.193327	6.3038588	AK093431	chr7:149524038-149524097	
A_33_P3371425	5.3424354	5.806246	NM_020451	chr1:26142117-26142176	SEPN1
A_24_P416411	6.536656	6.290715	NM_000286	chr17:33902177-33902118	PEX12
A_23_P20732	8.447224	8.542856	NM_012204	chr9:135564751-135564810	GTF3C4
A_33_P3252730	2.3221061	2.3900566	NR_027049	chr19:12225433-12225492	ZNF788
A_24_P231494	5.9478927	5.633367	NM_012100	chr2:220250727-220250407	DNPEP
A_23_P382488	4.9232044	4.616297	NM_138360	chr14:24538399-24538589	LRRC16B
A_24_P68222	4.9566693	4.3216734	NR_033381	chrY:2506086-2506145	CD99P1
A_23_P204736	3.8719525	3.529542	NM_005276	chr12:50504625-50504684	GPD1
A_23_P23839	6.274874	6.5750628	NM_001017403	chr1:202288529-202288588	LGR6
A_23_P254594	5.354823	5.339329	NM_000825	chr8:25276966-25276907	GNRH1
A_24_P357406	3.5813982	3.2944489	NM_001006121	chrY:23675236-23675295	RBMY1B
A_33_P3641714	7.875041	8.065376	NM_018381	chr19:10203524-10203583	C19orf66
A_23_P212423	3.8528576	3.2968268	NM_005108	chr3:38455633-38455692	XYLB
A_33_P3247077	7.362649	7.7918043		chr13:044404272-044404331	
A_33_P3294826	5.0881166	5.0838223	NM_001199723	chr1:156675605-156675546	CRABP2
A_23_P365817	12.677298	12.521011	NM_138689	chr11:64012050-64011991	PPP1R14B
A_23_P82047	5.475896	5.1584907	NM_001127715	chr6:147707749-147707808	STXBP5
A_23_P417974	5.641462	5.931076	NM_173039	chr11:77320442-77320501	AQP11
A_23_P45059	8.303961	8.688873	NM_001380	chr10:129250461-129250520	DOCK1
A_33_P3246833	3.917916	3.5329554	NM_173843	chr2:113891531-113891590	IL1RN
A_23_P301476	6.8753924	6.610275	NM_173657	chr3:155481334-155481275	C3orf33
A_33_P3235117	6.1946673	6.1289077	NR_040084	chr21:37447245-37447186	LOC100133286
A_24_P252575	8.465061	9.012252	NM_012197	chr9:125866353-125866412	RABGAP1
A_23_P29953	7.613041	7.4446206	NM_172175	chr4:142654431-142654490	IL15
A_23_P60534	7.924167	8.382776	NM_130459	chr9:130493953-130493894	TOR2A

A_32_P219942	6.9929266	6.789284	NM_033132	chr13:100615740-100615681	ZIC5
A_33_P3265803	4.51039	3.9828248	NM_001002035	chr11:71548549-71548608	DEFB108B
A_23_P27381	6.434149	6.580797	NM_005786	chr18:73000738-73000797	TSHZ1
A_33_P3564399	5.476982	5.71145	NR_033420	chr17:28952259-28952318	SH3GL1P2
A_23_P55011	10.3165455	10.162213	NM_001037984	chr17:79218895-79218836	SLC38A10
A_33_P3364964	4.757071	5.1016927	AK130913	chr8:146045756-146045697	LOC100129596
A_33_P3293187	3.877017	3.5234323	NM_007163	chr18:43212383-43212442	SLC14A2
A_33_P3339253	8.702376	8.89352	AI421806	chr9:33019659-33019600	
A_23_P67399	8.759153	9.002075	NM_001039877	chr19:47222954-47222895	STRN4
A_23_P361604	4.1318874	4.099982	NM_001242671	chr20:18790443-18790384	C20orf78
A_33_P3323486	3.9850662	4.3974323	AB529256	chr11:55235135-55235191	
A_23_P419714	2.6583128	2.3900566	NM_001018072	chr12:108052879-108052938	BTBD11
A_24_P51118	6.527645	6.043935	NM_002451	chr9:21802766-21815476	MTAP
A_23_P18017	4.246602	3.804892	NM_001870	chr3:148614465-148614524	CPA3
A_23_P168761	2.3926988	2.3900566	NM_002851	chr7:121701290-121701349	PTPRZ1
A_23_P31073	5.2855196	5.142419	NM_005375	chr6:135540223-135540282	MYB
A_24_P62615	9.869758	9.995324	NM_006367	chr1:40536121-40536180	CAP1
A_24_P385585	3.8867137	3.5712266	NM_152834	chr2:668758-668699	TMEM18
A_33_P3331752	4.037075	3.388389	XM_005276061	chr7:100610746-100610805	
A_33_P3404739	2.3417442	2.3900566	XM_005248851	chr6:49519820-49519879	C6orf141
A_23_P126075	2.3221061	2.3900566	NM_002245	chr1:233807558-233807617	KCNK1
A_23_P339309	6.7440586	6.8010178	NM_001024674	chr14:74666713-74666772	LIN52
A_23_P111000	7.769651	7.8119936	NM_002800	chr6:32823918-32823977	PSMB9
A_23_P76078	2.4629245	2.3900566	NM_016584	chr12:56734083-56734142	IL23A
A_33_P3338152	7.824994	8.264443	XR_243952	chr19:46815680-46815739	HIF3A
A_23_P334864	6.3706837	6.8708925	NM_173822	chr2:201844541-201844482	FAM126B
A_32_P195401	7.740486	7.682659	NM_173511	chr2:203634375-203634434	FAM117B
A_33_P3234267	6.794613	6.9744635	AK131364	chr19:17202354-17202413	LOC100130442
A_23_P74950	11.571212	10.879935	NM_018715	chr1:17733553-17733494	RCC2
A_33_P3219475	7.899006	7.8525505	NM_001198812	chr9:103339673-103339732	MSANTD3-TMEFF1
A_24_P358305	8.011551	8.068656		chr8:70042275-70042336	XLOC_014512
A_23_P338495	3.678636	4.203611	NM_001282866	chr10:45953813-45953754	MARCH8
A_33_P3310232	15.107864	14.987498	NM_024884	chr14:50710535-50710476	L2HGDH
A_33_P3304242	3.3411455	3.0049174	NM_001281428	chr9:34520920-34520979	DNAI1
A_23_P84651	11.144929	11.252232	NM_003418	chr3:128886681-128886643	CNBP
A_24_P743802	8.5389595	8.557839	NM_133374	chr9:116818750-116818809	ZNF618
A_24_P703830	4.439698	4.1897483	NM_001098622	chr19:13991510-13991569	NANOS3
A_33_P3394175	3.8813987	2.885652	AB306151	chr7:142239651-142239592	
A_33_P3331391	4.261988	4.633143		chr15:090206700-090206759	
A_33_P3337742	4.068737	4.357355			
A_33_P3327491	5.866088	5.973171		chr19:019478929-019478988	
A_33_P3323847	11.677778	11.719934	NM_004260	chr8:145736731-145736672	RECQL4
A_33_P3219146	4.445693	4.152505	NM_001286136	chr11:197663-197722	ODF3
A_23_P1072	9.751658	9.69383	NM_000701	chr1:116944237-116946524	ATP1A1
A_23_P27239	4.4534883	4.674955	NM_002548	chr17:2995686-2995627	OR1D2
A_33_P3295543	6.0865064	6.060107	DA825750	chr4:121086981-121087040	
A_33_P3231670	5.261174	4.606481	NM_031297	chr9:140114758-140114699	RNF208
A_23_P81760	6.6969976	6.716808	NM_006775	chr6:163956097-163956156	QKI
A_24_P159702	4.1429815	4.6007233	NM_005570	chr18:56997928-56997869	LMAN1
A_33_P3228445	7.2107773	7.745549	NM_001680	chr11:117695459-117695400	FXYD2
A_23_P22765	12.284242	12.503775	NM_019056	chrX:47002105-47002046	NDUFB11
A_23_P360744	2.3221061	2.3900566	NM_000448	chr11:36601124-36601183	RAG1
A_33_P3245412	5.528891	5.2895203	NM_144964	chr9:37778904-37778963	TRMT10B
A_24_P195724	12.7516775	12.658161		chrX:032224776-032224837	
A_33_P3314436	5.6752157	5.1650925	NM_001163315	chr5:107356686-107356627	FBXL17
A_24_P713668	11.391367	11.629008	NM_014670	chr2:201688131-201688189	BZW1
A_33_P3350179	3.4974637	4.1975965	N95477	chr17:14207367-14207308	
A_33_P3228558	10.168236	9.98192	NM_001282290	chr17:43471360-43471301	ARHGAP27

A_23_P257583	7.066298	6.897311	NM_015689	chr7:140218518-140218459	DENND2A
A_32_P411592	11.240308	11.013224	NM_052897	chr12:57923572-57923631	MBD6
A_32_P52206	5.482414	5.484569	NM_018264	chr7:66479444-66479503	TYW1
A_24_P406060	6.6954584	6.251172	NM_182757	chr6:18468695-18468754	RNF144B
A_23_P93311	5.9670563	6.014383	NM_013993	chr6:30867378-30867436	DDR1
A_33_P3419545	6.3071094	6.215789	NM_001193478	chr13:49833586-49833645	CDADC1
A_33_P3238777	10.776199	10.917094	NR_103825	chr4:119555002-119555061	LOC729218
A_24_P902052	9.67514	9.80573	NR_024031	chr4:53579709-53579768	DANCR
A_23_P400449	2.7878754	2.3900566	NM_020927	chr16:78013635-78013694	VAT1L
A_33_P3260669	8.653767	8.788225	NM_001256312	chr8:99466971-99466912	STK3
A_23_P87902	7.9188643	7.6329226	NM_003845	chr12:4721849-4722701	DYRK4
A_23_P88710	9.971987	10.048481	NM_015497	chr15:42503478-42503419	TMEM87A
A_32_P10936	2.3221061	2.3900566	NM_004061	chr5:21751368-21751312	CDH12
A_23_P91140	7.9048724	8.1137705	NM_018441	chr2:216904046-216903987	PECR
A_23_P129389	8.688721	8.754484	NM_017740	chr16:85008687-85008628	ZDHHC7
A_23_P213857	4.072492	3.8490343	NM_000587	chr5:40981901-40981960	C7
A_32_P143880	8.873094	8.719109	NM_015955	chr2:32093542-32093483	MEMO1
A_33_P3246863	5.5020194	5.2062054	AK123945	chr16:68386456-68386515	PRMT7
A_33_P3357322	8.596901	7.5822544	NM_001042550	chr9:106903624-106903683	SMC2
A_33_P3316323	10.924485	10.093049	NM_016836	chr2:161131106-161131047	RBMS1
A_24_P242299	10.768726	10.936023	NM_005455	chr1:71529272-71529213	ZRANB2
A_33_P3396469	4.191432	4.206569		chr20:059339522-059339581	
A_33_P3418726	5.50714	4.8516836	NM_014630	chr15:85349427-85349486	ZNF592
A_33_P3387050	10.178679	10.354082	NM_001001795	chr8:145751663-145751604	C8orf82
A_33_P3339173	11.531905	11.643463	NM_001130440	chr1:225977243-225977302	SRP9
A_23_P254648	7.1176033	7.583913	NM_012164	chr9:123533786-123533727	FBXW2
A_33_P3248439	8.8502	8.402662	NM_033446	chr9:129269235-129269294	MVB12B
A_33_P3333485	6.1503167	6.092777		chrUn_gl000220:000095063-000095122	
A_33_P3265564	4.744365	4.534425		chr8:049826771-049826830	
A_33_P3251198	4.292918	3.9918125	AX721082	chr20:8632100-8632159	PLCB1
A_33_P3239287	9.761164	9.891387	NM_001005271	chr17:7816010-7816069	CHD3
A_23_P65022	3.722447	4.0090218	NM_000017	chr12:121176685-121176968	ACADS
A_32_P196047	9.141915	9.210712	NM_181787	chr8:95803693-95803752	DPY19L4
A_33_P3789327	4.359711	4.1340685	AV755695		SNORD12B
A_23_P346982	5.3223495	5.307687	NM_173666	chr5:118175706-118175647	DTWD2
A_32_P153892	9.028399	8.944494	NM_031923	chr10:8007214-8007273	TAF3
A_33_P3243618	5.100659	5.42448	NM_003272	chr1:236343208-236343267	GPR137B
A_33_P3333805	4.301828	4.3837314	NM_015655	chr20:25655729-25655670	ZNF337
A_33_P3414944	4.136576	4.9608455		chr15:91147516-91147457	
A_23_P251647	5.396511	5.2643156	NM_173362	chr5:94990048-94991785	RFESD
A_23_P374294	3.6151032	3.2264159	NM_145023	chr10:32863414-32863473	CCDC7
A_24_P145316	6.693161	6.399115	NM_183040	chr6:15523931-15523872	DTNBP1
A_23_P81770	11.569299	11.660463	NM_003463	chr6:64291197-64291256	PTP4A1
A_33_P3342430	13.529404	12.705259		chrX:153850738-153850679	
A_33_P3247489	3.6116097	3.854578	NM_001037225	chr11:64708109-64708050	C11orf85
A_33_P3301174	11.498434	11.446595	NM_006815	chr12:124082559-124082618	TMED2
A_33_P3386716	10.275137	10.255593	NM_032833	chr1:204372653-204372594	PPP1R15B
A_23_P369733	8.414668	8.4562025	NM_020850	chr16:67757197-67757138	RANBP10
A_24_P409595	5.2782	5.4978466	NM_005456	chr11:45927203-45927262	MAPK8IP1
A_33_P3818959	8.665404	9.047523	NM_152486	chr1:879580-879639	SAMD11
A_23_P47410	4.2060304	4.4401126	NM_138961	chr11:124623564-124623505	ESAM
A_33_P3283054	6.2285786	6.1079926		chr10:094038819-094038760	
A_33_P3392530	2.3221061	2.3900566	NM_001099921	chrX:35821793-35821852	MAGEB16
A_24_P193600	4.202337	3.6846313	NM_003450	chr16:3458426-3458485	ZNF174
A_33_P3352941	5.2862353	5.08128	NR_003136	chr15:76225279-76225220	FBXO22-AS1
A_33_P3477521	4.771539	3.3473341		chr17:48127772-48127713	LOC284080
A_23_P63190	7.9786296	8.210788	NM_002524	chr1:115249992-115249933	NRAS
A_23_P207299	8.151352	8.317097	NM_016125	chr17:58030058-58029999	RNFT1

A_33_P3522525	8.994986	8.873409	NM_025196	chr4:7062315-7062256	GRPEL1
A_24_P232365	3.2103693	2.969816	NM_019043	chr10:26789908-26789967	APBB1IP
A_33_P3238623	7.06269	6.534826		chr2:25258973-25259032	DNAJC27-AS1
A_33_P3289286	4.6801887	4.145209	NM_018202	chr1:25825807-25825866	TMEM57
A_33_P3278187	6.238842	6.6041737	NM_001286990	chr9:99122496-99122437	SLC35D2
A_23_P250380	6.1769648	6.3680882	NM_015274	chr4:6623280-6623339	MAN2B2
A_33_P3377851	5.1518397	5.1906686	NM_020709	chr19:46995505-46995446	PNMAL2
A_23_P140725	5.1468105	5.480509	NM_014714	chr16:1560669-1560610	IFT140
A_32_P41487	14.648371	14.673007	NM_005517	chr1:26802354-26802413	HMGN2
A_32_P73217	7.467403	7.522472	NM_020141	chr1:109639239-109639298	TMEM167B
A_23_P85053	6.3962345	6.142747	NM_005089	chrX:15838394-15840867	ZRSR2
A_33_P3393851	3.5696318	3.2459018	XM_005265182	chr3:10377899-10377840	ATP2B2
A_23_P362824	9.12402	9.293133	NM_001324	chr20:54979011-54979070	CSTF1
A_23_P21436	8.269371	8.146511	NM_015651	chr9:123618304-123618245	PHF19
A_24_P70117	6.6500063	6.3474927	NM_020234	chr15:49917549-49917608	DTWD1
A_23_P43557	5.9038496	6.2265005	NM_024820	chr9:126214622-126214563	DENND1A
A_23_P155332	10.333071	10.500504	NM_020357	chr3:101312884-101312943	PCNP
A_33_P3538279	8.693789	8.775158	XR_158845	chr9:34187856-34187915	PRO2852
A_23_P424002	9.150564	8.932078	NM_002697	chr1:167385244-167385304	POU2F1
A_33_P3415633	5.1663756	5.2531376	NM_001198670	chr11:120201040-120201099	TMEM136
A_33_P3362409	3.3138983	2.3900566	NR_036526	chr9:100057983-100058042	LOC100499484
A_24_P15754	7.308468	6.8072724	NM_006114	chr19:45396180-45397089	TOMM40
A_33_P3372647	4.9451456	4.8647985	DQ098690	chr22:22786597-22786656	
A_23_P144677	8.903197	8.604591	AF178574	chr5:96372835-96372894	LNPEP
A_23_P85783	8.94492	8.493549	NM_006623	chr1:120285529-120285588	PHGDH
A_33_P3276297	4.5386825	4.628356	AY326463	chr6:144167345-144167286	
A_33_P3406552	4.3244085	4.6059556		chr2:150624081-150624022	FLJ32955
A_33_P3283971	6.775287	6.6500063	NM_005007	chr6:31526381-31526440	NFKBIL1
A_33_P3343493	3.9024465	3.8885841	NM_001470	chr6:29574946-29574887	GABBR1
A_24_P398691	5.2702723	5.359779	NM_032859	chr13:108886511-108886570	ABHD13
A_23_P140748	5.1189184	5.3766255	NM_022910	chr16:58547109-58547168	NDRG4
A_33_P3423874	2.3221061	2.3900566	NM_001018100	chr15:57977472-57977531	MYZAP
A_23_P212447	7.191245	7.166653	NM_018262	chr3:129236393-129237964	IFT122
A_33_P3331911	7.148526	6.896516		chr16:022418699-022418758	
A_23_P317184	6.900592	6.8030887	NM_006309	chr3:37100322-37096639	LRRFIP2
A_33_P3336700	7.1472325	7.510259	NM_020859	chr4:77704264-77704323	SHROOM3
A_32_P36143	4.634655	4.8311214	AL523350	chr6:170125657-170125716	
A_23_P419156	4.9223347	4.8990912	NM_001708	chr7:128414662-128414603	OPN1SW
A_23_P413796	10.1725235	9.978022	NM_138443	chr18:43703284-43704800	HAUS1
A_33_P3281003	4.8984876	4.889423	AK127494	chr19:7098790-7098731	
A_33_P3340639	3.7125204	2.3900566	AL832464	chr10:113913749-113913690	GPAM
A_23_P55998	6.401948	5.232784	NM_005628	chr19:47278958-47278899	SLC1A5
A_23_P326170	13.560461	13.583082	NM_001743	chr2:47387407-47387348	CALM2
A_33_P3336198	4.8973045	4.855401	NM_182563	chr16:2259418-2259359	BRICD5
A_32_P214340	5.2666726	4.8740797		chr1:39341759-39341818	
A_23_P425587	6.680435	6.8603992	NM_001001683	chr17:4636387-4636446	MED11
A_23_P255695	7.4751854	8.094536	NM_006632	chr6:25849668-25849609	SLC17A3
A_24_P360269	10.194654	10.692845	NM_003730	chr6:167344538-167343227	RNASET2
A_23_P377664	6.2514095	6.514367	NM_001135745	chr2:202624550-202624491	ALS2
A_33_P3246293	5.048872	4.7621846	NM_001145207	chr6:135359021-135358962	HBS1L
A_23_P168240	7.4343715	7.52836	NM_003913	chr6:4057390-4058989	PRPF4B
A_23_P98532	8.646752	8.7887	NM_015457	chr11:57468340-57468399	ZDHHC5
A_33_P3628409	6.8907247	6.482115	NR_037701	chr2:217084790-217084849	PKI55
A_23_P408239	4.894861	4.825173	NM_001252124	chr2:242053592-242053533	PASK
A_24_P84419	9.444198	9.421664	NM_003371	chr9:136627526-136627467	VAV2
A_24_P270525	10.680951	10.477465	NM_004492	chr15:59934440-59934381	GTF2A2
A_23_P254816	7.168803	6.9330854	NM_004609	chr20:585004-584945	TCF15
A_33_P3423027	4.1997113	3.415958	NM_006177	chr14:24550145-24550086	NRL
A_23_P17012	4.806468	4.6421537	NM_024583	chr2:175293369-175293428	SCRN3

A_23_P152666	10.864839	10.979118	NM_004375	chr17:53039407-53039348	COX11
A_24_P913115	7.568104	8.079461	NM_000314	chr10:89727925-89727984	PTEN
A_23_P141180	7.868469	7.901058	NM_001082968	chr17:17747058-17746999	TOM1L2
A_33_P3250767	6.310251	6.3734694		chr17:000181412-000181353	
A_23_P122876	10.707029	10.783081	NM_005641	chr7:99704771-99704712	TAF6
A_23_P501877	8.29524	8.121316	NM_018197	chr20:50768203-50768144	ZFP64
A_23_P215900	10.900652	10.878106	NM_016240	chr8:27530383-27530442	SCARA3
A_33_P3262854	3.876667	3.2441866	NR_038099	chr2:110682244-110682303	LIMS3L
A_23_P14273	9.651554	10.350857	NM_024071	chr14:104199797-104199856	ZFYVE21
A_33_P3405754	6.114223	6.278309	BC050721	chr1:3759797-3759738	CEP104
A_33_P3308481	6.7707434	6.855777	NR_002768	chr6:144324233-144324174	HYMAI
A_23_P68059	7.9433155	7.8056197	NM_032673	chr2:74732690-74732499	PCGF1
A_23_P42664	12.574505	12.674974	NM_006304	chr7:96318218-96318159	SHFM1
A_33_P3358851	5.62109	5.207655		chr1:068626754-068626695	
A_33_P3354970	4.5138407	4.4052196		chr11:058665727-058665786	
A_32_P133005	4.9806976	5.3291264	NM_024345	chr9:37867002-37867061	DCAF10
A_23_P148513	9.838757	10.164868	NM_005274	chr1:84971699-84967600	GNG5
A_23_P1083	4.9299164	4.911075	NM_002060	chr1:35261095-35261154	GJA4
A_23_P216935	5.1614933	5.052946	NR_026677	chr9:125872202-125872143	MIR600HG
A_24_P31235	8.678358	8.382515	NM_001970	chr17:7213037-7213096	EIF5A
A_24_P138361	12.414043	12.216221	NM_006360	chr11:32623862-32623921	EIF3M
A_33_P3402570	9.979052	9.717627	NM_014167	chr12:82746993-82746934	CCDC59
A_24_P127748	3.6764402	4.124155	NM_015097	chr3:33653556-33653497	CLASP2
A_23_P203790	9.0328665	9.104533	NM_006812	chr12:58115180-58115239	OS9
A_33_P3263497	3.224503	3.7594576	AL833363	chr2:242013337-242013396	SNED1
A_23_P119698	9.471381	9.380321	NM_016579	chr19:8367394-8367335	CD320
A_24_P264207	15.93552	16.084991	NM_002823	chr2:232577209-232577555	PTMA
A_23_P213908	9.384023	9.161412	NM_032177	chr5:125960463-125960522	PHAX
A_23_P107661	8.042814	8.050212	NM_014225	chr19:52729053-52729268	PPP2R1A
A_33_P3379922	3.3725314	2.3900566	NM_000312	chr2:128186439-128186498	PROC
A_23_P206022	4.250492	4.1902328	NM_001004439	chr15:68594130-68594071	ITGA11
A_33_P3393796	8.357157	7.9865203	NM_020810	chr14:61441833-61441774	TRMT5
A_32_P193322	7.7684474	7.6694307	NM_152756	chr5:38938366-38938307	RICTOR
A_33_P3408221	5.928891	6.193327	NR_026952	chr11:43920782-43920841	SEC14L1P1
A_33_P3392447	7.558067	7.442387	NM_003921	chr1:85731997-85731938	BCL10
A_23_P382602	5.4685087	5.5043573	NM_004326	chr1:147097268-147097327	BCL9
A_33_P3403927	7.2679734	7.2223506	NM_080621	chr20:62605559-62605500	SAMD10
A_33_P3264238	5.720145	5.4886947	NM_080764	chr22:22839041-22838982	ZNF280B
A_23_P44546	5.810566	5.85499	NM_001282669	chr1:3801526-3801585	DFFB
A_23_P216108	5.242237	5.3595815	NM_000037	chr8:41513128-41513069	ANK1
A_23_P45592	3.7128406	3.1334674	NR_001533	chrY:9528798-9529334	TTTY8
A_24_P400355	8.2064495	8.0403595	NM_015949	chr7:935912-935971	GET4
A_23_P69242	8.982774	9.4324	NM_182760	chr3:4403152-4403094	SUMF1
A_33_P3405424	6.499136	7.1032615	NM_152899	chr19:50392976-50392917	IL4I1
A_33_P3354267	9.888712	9.900719	NM_024595	chr1:39471205-39471264	AKIRIN1
A_33_P3240200	10.407352	10.20616	NM_015448	chr10:103369350-103369409	DPCD
A_33_P3329462	5.765025	5.9279723		chr13:51095345-51095286	
A_33_P3219459	4.9624214	4.8135147	NM_001114748	chr1:1470625-1470566	TMEM240
A_24_P143686	8.223947	8.797929	NM_001080394	chr8:48642027-48647927	SPIDR
A_23_P38346	4.135301	4.28466	NM_024119	chr17:40253518-40253459	DHX58
A_33_P3378689	8.92687	9.319168	NM_016628	chr10:28905152-28905211	WAC
A_32_P117170	5.075792	5.237528	NM_001122838	chr7:102740667-102740608	NAPEPLD
A_23_P158024	10.666563	10.856531	NM_007234	chr9:34617948-34617889	DCTN3
A_33_P3226425	5.805782	6.099721	NM_001034025	chr12:112451353-112451412	ERP29
A_33_P3271121	6.0949054	6.641053		chr2:235861754-235861813	
A_32_P8402	10.427385	10.498325	NM_006372	chr6:86323723-86323693	SYNCRIP
A_23_P25674	6.585471	6.5515575	NM_001823	chr14:103986525-103986466	CKB
A_33_P3290368	4.2705092	4.5977817	NR_027455	chrX:148615336-148615277	LINC00893
A_23_P123164	5.3550777	5.5710516	NR_002140	chr7:142759720-142759661	OR6W1P

A_33_P3305885	3.7781951	4.222808	NM_006648	chr9:96060265-96060324	WNK2
A_33_P3743508	9.877373	9.845592		chr9:98864127-98864186	LOC158435
A_33_P3249893	4.453151	4.766823	XR_108343	chr2:91911606-91911547	FLJ37786
A_24_P31929	6.8957767	7.337954	NM_000950	chrX:37315899-37315958	PRRG1
A_23_P329768	4.710379	4.470425	NM_014668	chr2:11782680-11782739	GREB1
A_33_P3343155	6.9334254	7.3375807	NM_002072	chr9:80335278-80335219	GNAQ
A_33_P3329378	4.6155605	4.6354437	NM_014461	chr3:1445158-1445217	CNTN6
A_33_P3296181	3.8436704	3.7565856	NM_001001437	chr17:34524139-34524080	CCL3L3
A_33_P3239102	10.983267	10.64076		chr15:082927851-082927792	
A_23_P152066	8.314928	8.271236	NM_174916	chr15:43235340-43235281	UBR1
A_24_P355720	3.6902957	2.3900566	NM_002007	chr11:69588188-69588129	FGF4
A_23_P148609	2.3221061	2.3900566	NM_021796	chrX:133700139-133700080	PLAC1
A_23_P309850	8.681414	8.341616	NM_152260	chr15:40866570-40866629	RPUSD2
A_33_P3274105	8.125347	8.334235		chr2:55451395-55451336	CLHC1
A_23_P47484	4.055016	2.3900566	NM_145016	chr11:58602251-58602192	GLYATL2
A_23_P23074	9.203926	9.115744	NM_006417	chr1:79128479-79128538	IFI44
A_23_P136964	6.378516	6.2226725	NM_000328	chrX:38128962-38128903	RPGR
A_23_P61810	8.939018	9.00023	NM_017450	chr17:79084117-79084176	BAIAP2
A_23_P145437	9.900345	9.916611	NM_017934	chr6:79650509-79650450	PHIP
A_32_P14610	9.906871	10.436537	NM_006457	chr4:95589078-95589137	PDLIM5
A_33_P3361546	5.248827	5.7379885	NM_001032280	chr6:10397085-10397026	TFAP2A
A_23_P389588	6.099934	6.148428	NM_030756	chr10:114911485-114911544	TCF7L2
A_33_P3209162	3.854578	4.0283704	NM_001646	chr19:45448483-45448542	APOC4
A_33_P3238310	5.6683784	5.5942254	NM_001286623	chr21:30464842-30464901	MAP3K7CL
A_24_P123720	5.3818765	4.7479596	NM_007214	chr6:108189385-108189326	SEC63
A_33_P3266489	4.04309	3.7372224	NM_001004486	chrX:130678869-130678928	OR13H1
A_33_P3363500	3.587946	4.0714016		chr22:022645324-022645265	
A_23_P13822	5.6640973	5.7406735	NM_018423	chr12:10771913-10771854	STYK1
A_23_P434900	12.299675	12.491766	NM_144570	chr16:1751289-1751348	HN1L
A_33_P3317815	7.8443165	7.991934	NM_004985	chr12:25378608-25378549	KRAS
A_23_P368711	3.6915417	3.855122	NM_006864	chr19:54724656-54724597	LILRB3
A_33_P3320599	4.5095553	3.9002485		chr2:107557462-107557403	
A_33_P3379157	8.540449	8.486318	NM_001271641	chr6:36936634-36936575	MTCH1
A_23_P64932	5.2986736	5.295876	NM_018157	chr12:107280395-107280454	RIC8B
A_33_P3226560	3.6352131	3.0618823		chr22:018844469-018844410	
A_24_P378788	6.9952574	7.4300046	NM_024881	chr19:16678853-16677420	SLC35E1
A_24_P297888	5.6116652	5.2470474	NM_002451	chr9:21837969-21854648	MTAP
A_33_P3394405	5.6955867	6.025404	DB238770	chr1:9241884-9241825	LOC727721
A_33_P3354499	12.121235	12.132181	XR_242443	chr8:142400328-142400269	
A_23_P52017	12.314587	12.464917	NM_018136	chr1:197053513-197053454	ASPM
A_33_P3264072	7.809428	7.971266	NR_033913	chr9:139643349-139643408	LOC100128593
A_24_P923757	3.4689126	3.3007362	NM_018179	chr12:14577680-14577739	ATF7IP
A_23_P201445	11.091354	11.179016	NM_016076	chr1:244871892-244871951	DESI2
A_24_P40594	4.051344	4.328448	NM_020834	chr14:23743806-23743747	HOMEZ
A_23_P385861	10.134633	10.321963	NM_152562	chr8:25365256-25365315	CDCA2
A_24_P178148	6.4414644	6.6730084	XR_249246	chr2:176993554-176993495	LOC100129455
A_33_P3253653	16.323286	16.355698	NM_001033045	chr2:175296307-175296299	GPR155
A_33_P3394234	7.189899	7.2052517	NM_003434	chr20:18297262-18297321	ZNF133
A_23_P143857	5.562374	5.62877	NM_052953	chr3:26751927-26751986	LRRC3B
A_33_P3303562	4.647652	4.368073		chr21:030699512-030699453	
A_23_P201319	6.9569383	6.773923	NM_032890	chr1:223179111-223179170	DISP1
A_32_P90483	7.135456	7.40123	NM_178509	chr17:53240764-53240823	STXBP4
A_32_P59678	4.303872	3.3950257	NM_001127364	chr7:23731180-23740428	FAM221A
A_24_P275073	2.9506686	2.3900566	NM_139155	chr10:72522132-72522191	ADAMTS14
A_23_P435941	5.046128	4.6794477	NM_138352	chr19:14199590-14199531	SAMD1
A_23_P411157	3.5536644	2.3900566	NM_005430	chr12:49376278-49376337	WNT1
A_33_P3228128	9.4425745	9.427089	NM_202001	chr19:45918183-45918124	ERCC1
A_23_P68700	3.7677674	3.7694116	NM_017833	chr21:34860859-34860800	DNAJC28
A_32_P28685	9.832707	9.765581	NM_003090	chr15:101827887-101827183	SNRPA1

A_23_P132341	8.723244	8.406283	NM_001142964	chr22:42093403-42093462	C22orf46
A_23_P204158	4.2127166	4.4270096	NM_032814	chr12:117290515-117290574	RNFT2
A_24_P90097	8.219979	8.502469	NM_016824	chr10:111894827-111894886	ADD3
A_23_P141549	14.750789	14.731188	NM_001011	chr2:3627779-3627838	RPS7
A_33_P3230788	6.2051687	6.387857		chr9:113468373-113468432	MUSK
A_23_P103532	5.9085836	5.949614	NM_001267609	chr1:168065891-168065832	GPR161
A_23_P143994	5.7149043	5.557025	NM_001018115	chr3:10143006-10143065	FANCD2
A_23_P344531	7.7280116	7.5034723	NM_007286	chr5:150038396-150038455	SYNPO
A_23_P366328	8.440025	8.401019	NM_152415	chr8:17143905-17152523	VPS37A
A_33_P3355649	4.1668434	3.6394033	AK128759	chr4:186920662-186920603	LOC442122
A_23_P170058	11.386994	11.6723385	NM_002794	chr1:36101911-36096888	PSMB2
A_33_P3368159	4.587043	4.541666	NM_032214	chr20:35242768-35242709	SLA2
A_23_P202939	10.465707	10.923334	NM_001642	chr11:130014421-130014480	APLP2
A_33_P3234317	12.500863	12.29028	NM_012250	chr11:14299538-14299479	RRAS2
A_32_P511713	6.704705	6.6167817	NM_173525	chr11:6231561-6231620	C11orf42
A_24_P127021	9.435334	9.594495	NM_003932	chr22:41236646-41231849	ST13
A_33_P3237775	6.3988075	6.473522	NM_005693	chr11:47290221-47290280	NR1H3
A_24_P195476	7.060163	7.006257	NM_000535	chr7:6048632-6045608	PMS2
A_24_P141736	6.303502	6.2584343	NM_006838	chr12:95909063-95909122	METAP2
A_23_P328069	7.5324373	6.909622	NM_000195	chr10:100176196-100176137	HPS1
A_24_P332218	4.315841	4.0919447	NM_001038705	chr3:154055959-154055900	GPR149
A_23_P97365	7.5597386	7.7689185	NM_018103	chr1:90401097-90401156	LRRC8D
A_33_P3329769	6.398038	6.324201	NM_152731	chr6:56857293-56857352	BEND6
A_33_P3239569	4.1496	4.05003	BC027976	chr20:60963037-60963096	RPS21
A_24_P527404	6.7711124	7.258556	NM_004329	chr10:88683628-88683687	BMPR1A
A_23_P80473	6.0357122	6.29346	NM_152889	chr3:126261750-126261809	CHST13
A_23_P337875	4.002593	4.339543	NM_001040202	chr4:79851422-79851363	PAQR3
A_23_P132260	5.660815	5.801613	NM_014303	chr22:30975843-30975784	PES1
A_23_P29248	8.816544	8.933505	NM_003312	chr22:37407072-37407013	TST
A_24_P156288	6.7983904	6.646325	NM_022482	chr20:23352427-23352486	GZF1
A_23_P152678	7.3806868	7.2936006	NM_015681	chr17:19261153-19251135	B9D1
A_23_P145175	7.395819	7.5472713	NM_014345	chr6:43304051-43303992	ZNF318
A_23_P156739	10.321525	10.43873	NM_032340	chr6:33665507-33665448	UQCC2
A_23_P85392	9.483101	9.664207	NM_007033	chr1:2333716-2333775	RER1
A_33_P3314828	3.7856483	4.293872		chr6:052630131-052630072	
A_33_P3283213	4.2813025	4.20261	NR_027295	chr17:43713933-43713992	MGC57346
A_33_P3397905	5.49629	5.7220335	NM_005868	chr7:93621059-93621000	BET1
A_23_P380010	4.8822794	5.2037864	NM_020759	chr15:43012226-43012285	STARD9
A_23_P57059	10.330116	9.711385	NM_017453	chr20:47730324-47730265	STAU1
A_24_P724153	6.2156506	6.216094	CU688199	chr4:152025742-152025683	
A_33_P3242109	6.0298996	5.168523	BC039168	chr12:1084586-1084527	LOC100130219
A_24_P356406	10.356138	9.463057	NM_000430	chr17:2588767-2588826	PAFAH1B1
A_33_P3224105	10.87684	10.984541	NM_001142761	chr15:40686247-40686306	KNSTRN
A_33_P3218584	10.151448	10.091102	NM_015918	chr12:121016908-121016849	POP5
A_24_P223384	6.1195416	6.201584	NM_003513	chr6:26033520-26033461	HIST1H2AB
A_33_P3373469	14.490176	14.239221	NM_002808	chr3:184026780-184026839	PSMD2
A_23_P57293	7.786029	7.5355325	NM_014825	chr21:33686835-33686776	URB1
A_33_P3344201	4.262904	3.2704747	NM_032375	chr19:50372646-50372587	AKT1S1
A_33_P3377045	6.954858	7.52565	NM_017913	chr9:4701968-4702027	CDC37L1
A_23_P160214	5.160466	5.8636384	NM_001080494	chr1:51753563-51753504	TTC39A
A_33_P3268144	7.309798	7.4698744	NM_025040	chr19:52516662-52516603	ZNF614
A_33_P3319491	11.100205	11.006588	NM_015878	chr8:103838887-103838828	AZIN1
A_33_P3314813	3.9653535	3.9569807	NM_001080446	chr11:45928144-45928085	C11orf94
A_23_P164999	7.62333	7.8005495	NM_004359	chr19:541675-541734	CDC34
A_23_P142407	6.7202544	6.7226973	NM_033204	chr19:19790730-19790789	ZNF101
A_23_P48339	7.0266795	7.425973	NM_175605	chr13:21237644-21237703	IFT88
A_23_P211598	8.395311	8.532999	NM_002676	chr22:41973858-41973432	PMM1
A_24_P765552	3.8874273	2.3900566	AY358240	chr3:12949305-12949246	LOC100128644
A_23_P71889	6.5707808	6.739374	NM_153437	chr9:131255046-131256809	ODF2

A_24_P317835	10.0970125	10.1474085	NM_019892	chr9:139323209-139323150	INPP5E
A_33_P3410781	6.502869	6.309009	AK000993	chr7:5941558-5941617	
A_23_P2973	4.022103	3.2752738	NM_001004063	chr14:20404613-20404672	OR4K1
A_24_P41801	5.0910397	4.8975058	NM_005577	chr6:160966519-160963829	LPA
A_24_P301063	4.191862	3.9750147	NM_018173	chr12:6436476-6436535	PLEKHG6
A_33_P3213551	10.708387	10.720389	NM_030974	chr8:145153602-145153543	SHARPIN
A_23_P93217	5.224886	5.183432	NM_153320	chr6:43269944-43270003	SLC22A7
A_33_P3284686	5.514187	5.1225824	NM_145716	chr1:54693957-54692804	SSBP3
A_33_P3212994	11.548651	11.655544	NM_032997	chr10:58117666-58117607	ZWINT
A_23_P55251	10.3192005	10.030988	NM_002204	chr17:48167365-48167424	ITGA3
A_33_P3405571	3.6689906	3.5444462		chr10:044069535-044069594	
A_33_P3433388	4.3397083	4.0874853	AB050003	chr7:27667051-27667110	TSL
A_33_P3211793	5.384243	5.4352264	NR_047662	chr8:12465492-12465433	LOC729732
A_32_P89827	4.432846	4.4695277	NR_002815	chr13:25171688-25171747	TPTE2P6
A_23_P371076	5.3219604	5.039943	AF113122	chr13:74387119-74387060	KLF12
A_33_P3311205	5.1693287	5.340935	NM_001031698	chr12:50037727-50037786	PRPF40B
A_23_P217609	15.102499	15.107864	NM_021029	chrX:100650439-100650768	RPL36A
A_23_P2414	4.1859264	4.0817633	AK311217	chr12:21684967-21685026	C12orf39
A_33_P3278410	5.273039	5.581339	NM_001207008	chr6:168276100-168276159	MLLT4
A_32_P235159	7.767809	7.9664645	NR_024322	chr2:234774498-234774439	MSL3P1
A_23_P26173	9.209545	9.489122	NM_007364	chr15:79614644-79614703	TMED3
A_33_P3278911	6.8878155	7.0015225	XM_005250942	chr8:133837306-133837365	PHF20L1
A_23_P90679	8.137252	8.615044	NM_018571	chr2:202344823-202344882	STRADB
A_33_P3260016	9.833165	9.8565035	NM_152905	chr12:97346926-97346985	NEDD1
A_24_P79755	6.057248	5.8570347	NM_006066	chr1:46032273-46032332	AKR1A1
A_23_P146997	9.935502	9.772537	NM_018360	chrX:16852460-16855753	TXLNG
A_24_P242820	9.812664	9.305747	NM_004622	chr2:122524494-122524553	TSN
A_24_P388433	9.883261	9.518974	NM_002718	chr3:135866561-135866620	PPP2R3A
A_24_P7179	3.9505732	3.7943053	NM_206890	chr21:47961742-47961801	DIP2A
A_33_P3250018	5.0867414	5.0793176	NM_013320	chr12:104480733-104480792	HCFC2
A_23_P64873	3.5931935	3.0528588	NM_001920	chr12:91539893-91539834	DCN
A_24_P106542	4.5739307	4.9806976	NM_032784	chr6:127476517-127476576	RSPO3
A_33_P3217834	10.363949	10.331777	NM_014140	chr2:217347660-217347719	SMARCAL1
A_32_P191004	5.990573	6.0150747	NM_017552	chr2:23971968-23971909	ATAD2B
A_33_P3222698	4.606224	4.9566693	NM_006991	chr3:44686678-44686737	ZNF197
A_23_P337262	2.3221061	2.3900566	NM_153000	chr18:10488470-10488529	APCDD1
A_23_P390596	8.414104	8.616917	NM_006742	chr16:67963478-67963537	PSKH1
A_24_P22939	5.8885803	6.020439	NM_213604	chr19:1506006-1505947	ADAMTSL5
A_33_P3275968	9.288272	8.844103	NM_005065	chr14:81939299-81939240	SEL1L
A_23_P259333	7.573101	7.6528125	NM_016487	chr6:107365527-107372310	C6orf203
A_23_P411814	10.279789	10.403095	NM_021227	chr4:109578716-109578775	OSTC
A_24_P255114	3.4474838	4.298929	AF220493	chr11:7794818-7794759	
A_24_P37939	7.414852	7.369612	XM_005268495	chr5:149823390-149823331	RPS14
A_33_P3315704	5.9482746	6.111329		chr9:035617474-035617533	
A_23_P146134	2.3221061	2.3900566	NM_024025	chr8:33448945-33448886	DUSP26
A_23_P85726	7.7870274	7.808427	NM_033418	chr1:169762265-169762206	METTL18
A_23_P206510	10.6714325	10.846741	NM_012201	chr16:74487070-74485972	GLG1
A_24_P508103	2.3221061	2.3900566	NR_026758	chr2:130783918-130783859	FAR2P1
A_23_P47116	4.4117317	4.582911	NM_003475	chr11:563211-563270	RASSF7
A_33_P3262560	5.0121384	5.1875606	BC093406	chr18:21375992-21376051	LAMA3
A_23_P421175	6.9540567	6.751296	NM_198488	chr8:144806611-144806552	FAM83H
A_23_P97250	9.352462	9.48987	NM_001282860	chr1:155719576-155719517	GON4L
A_23_P258246	10.659481	10.652837	NM_001923	chr11:61067258-61067199	DDB1
A_32_P101699	7.324863	7.207965	XR_243500	chr16:84151080-84151139	
A_24_P88565	6.215789	6.3922043	NM_018845	chr1:155110666-155110725	SLC50A1
A_33_P3307197	9.139345	9.239421	NM_020440	chr1:117532823-117532882	PTGFRN
A_23_P29638	4.378753	4.3550024	NM_184231	chr3:48711855-48711796	NCKIPSD
A_32_P2634	6.5550966	6.4869633		chr10:48952880-48952939	XLOC_014512
A_33_P3244083	5.3828654	5.294143	NM_015254	chr8:29037752-29037693	KIF13B

A_23_P253536	2.7413502	2.3900566	NM_000908	chr5:32784961-32786370	NPR3
A_33_P3357283	4.000514	3.9727883	XM_005276700	chr22:18782008-18781949	
A_23_P16354	2.3221061	2.3900566	NM_032825	chr19:37118399-37118458	ZNF382
A_33_P3414669	6.22188	5.5049148	NM_183353	chrX:73810112-73810053	RLIM
A_23_P106299	12.692611	12.857992	NM_001018108	chr15:44085932-44085991	SERF2
A_33_P3307886	6.0026603	6.300339	NM_001060	chr19:3595228-3595169	TBXA2R
A_24_P162373	9.827381	9.550377	NM_001206998	chr22:29449612-29449671	ZNRF3
A_23_P215517	7.214443	7.5441475	NM_001172428	chr7:23165385-23165444	KLHL7
A_33_P3417626	5.1122	5.33595	NM_198573	chr9:34521161-34521102	ENHO
A_23_P370569	5.00213	4.8142533	NM_152440	chr12:64586780-64586721	C12orf66
A_23_P35082	9.503762	9.97481	NM_031459	chr1:28608194-28608253	SESN2
A_23_P73589	10.294849	10.395808	NM_002444	chrX:64961601-64961660	MSN
A_24_P126425	4.199911	4.185155	NM_032222	chr7:30876340-30878834	FAM188B
A_23_P30050	7.713992	8.073063	NM_006345	chr4:42088738-42088797	SLC30A9
A_32_P64038	8.655924	8.7070675	BC062632	chr17:29361561-29361620	LOC400590
A_33_P3341716	8.264443	7.9124207	NM_003112	chr7:21553991-21554050	SP4
A_24_P201879	6.6951714	6.670829	NM_021645	chr13:52607064-52607123	UTP14C
A_23_P43684	4.9145694	4.559529	NM_017637	chr9:16419008-16418949	BNC2
A_33_P3347477	2.6160283	2.3900566	DA375949	chr11:131747657-131747598	
A_33_P3217786	4.1192713	3.4453812	NR_002817	chr9:67272519-67272460	AQP7P1
A_32_P167239	8.429908	8.177811	NM_152406	chr5:148720325-148720384	AFAP1L1
A_24_P154037	7.6109147	7.554539	NM_003749	chr13:110406704-110406645	IRS2
A_23_P152181	8.816771	8.678138	NM_018119	chr16:22343491-22345008	POLR3E
A_33_P3805085	6.263526	5.9802628	NM_133372	chr5:130979125-130979066	FNIP1
A_33_P3280157	7.1339016	7.069522	NR_001290	chr15:25328768-25328827	SNORD116-19
A_33_P3594214	5.0670686	4.81603	NR_044999	chr11:3412514-3412455	OR7E12P
A_33_P3349827	7.4158463	7.9660287	NM_006743	chrX:48433995-48434054	RBM3
A_33_P3235856	5.2087584	5.090745	NM_006914	chr9:77302043-77302102	RORB
A_33_P3311170	6.745218	6.628951	NR_002593	chr16:29624599-29624540	SLC7A5P1
A_32_P176018	5.077692	4.691503	NM_030812	chr1:18153390-18153449	ACTL8
A_33_P3396807	6.1721883	6.431649	NM_032852	chr1:63284714-63284773	ATG4C
A_23_P153098	9.083764	9.1854515	NM_032124	chr18:44633984-44633925	HDHD2
A_33_P3324004	5.687784	5.7446637	NM_032965	chr17:34324822-34324763	CCL15
A_33_P3242783	4.960019	5.496832	AK131474	chr12:11009730-11009789	LOC440082
A_23_P33154	7.685139	7.2107773	NM_014393	chr8:74461997-74461938	STAU2
A_23_P381714	8.171566	6.6403723	NM_198584	chr8:86195965-86196024	CA13
A_23_P21560	8.43094	8.656458	NM_030797	chr2:16736356-16734228	FAM49A
A_23_P21747	6.6218038	6.917464	NM_019855	chr19:48533434-48533375	CABP5
A_33_P3236259	5.3773136	5.7144113	BX398892	chr2:20254198-20254257	
A_23_P87049	4.582428	4.3739176	NM_003105	chr11:121500247-121500306	SORL1
A_23_P139228	7.8226504	7.641576	NM_012402	chr11:6498089-6498030	ARFIP2
A_33_P3767927	9.711737	9.600181	NM_001018077	chr5:142660788-142660729	NR3C1
A_32_P193288	12.528184	12.534498	NM_000980	chr19:17972198-17972257	RPL18A
A_24_P247978	3.9791381	3.8672743	NM_016089	chr3:48311766-48311825	ZNF589
A_23_P73511	4.2201066	4.137195	NM_001654	chrX:47429320-47429379	ARAF
A_33_P3353170	6.5878897	6.54707	BC051800	chr17:57768128-57768187	CLTC
A_33_P3352822	5.2229905	5.5065866	NM_173631	chr19:57889316-57889375	ZNF547
A_33_P3218135	4.574616	4.3647118	NR_015398	chr12:126947311-126947370	LOC100128554
A_33_P3774867	4.337574	4.262904	BC094703	chr18:3607924-3607983	
A_33_P3372910	6.6118994	6.7318225	NM_014314	chr9:32455457-32455398	DDX58
A_33_P3367247	6.314162	6.8574953	NM_001114395	chr9:17298881-17298940	CNTLN
A_32_P192692	7.24331	6.7983904	NM_001014797	chr10:78629523-78629464	KCNMA1
A_33_P3210443	4.3535147	4.390353	NR_038436	chr14:104322781-104322840	LINC00637
A_33_P3329153	4.6614356	4.4764543	NM_031267	chr7:40102633-40102692	CDK13
A_23_P58819	4.523241	4.272134	NM_022897	chr5:170725916-170725975	RANBP17
A_23_P129556	9.318546	9.323842	NM_000418	chr16:27375999-27376058	IL4R
A_33_P3327479	7.455237	6.9257903	NM_016598	chr3:44956813-44956754	ZDHHC3
A_33_P3412438	5.338795	5.4501214		chrX:001581810-001581869	
A_33_P3404316	7.6282535	6.672037	NR_024430	chr11:121959870-121959811	MIR100HG

A_33_P3234804	7.2985163	7.149177	NM_032783	chr4:169908896-169908837	CBR4
A_32_P88415	3.44086	3.2095723	NM_133371	chr5:150058791-150058850	MYOZ3
A_24_P186346	7.463497	7.51388	NM_022662	chr2:112561345-112561286	ANAPC1
A_23_P167389	9.40266	9.222044	NM_022481	chr5:141033208-141033149	ARAP3
A_33_P3229272	4.3594093	4.000514	NM_014567	chr15:020279587-020279528	BCAR1
A_33_P3286387	6.2226725	5.916127	NM_001145527	chr9:140676751-140676810	EHMT1
A_33_P3265359	8.232075	7.8665066	NM_018645	chr2:239147006-239146947	HES6
A_23_P167081	4.9324436	5.322537	NM_005612	chr4:57798055-57798114	REST
A_33_P3245539	5.192261	6.232185	NM_006029	chr14:74179568-74179509	PNMA1
A_33_P3219240	6.789284	7.0347166	AK131413	chrX:149095487-149095428	LOC642980
A_33_P3385417	4.3511176	3.6877716		chr15:043212362-043212421	
A_23_P250002	6.7479177	6.711199	NM_020771	chr6:105176417-105176358	HACE1
A_33_P3406030	2.746219	2.3900566	NM_032270	chr1:90152110-90152169	LRRC8C
A_33_P3321657	11.433283	11.591817	NM_005529	chr1:22148799-22148740	HSPG2
A_33_P3257817	12.794319	13.191831	AK091697	chr8:37592362-37592303	
A_23_P501961	5.8362103	6.08317	NM_032107	chr20:42169726-42169785	L3MBTL1
A_24_P103886	8.57275	8.582073	NM_004508	chr10:1086678-1086619	IDI1
A_23_P380724	9.542019	9.200103	NM_021826	chr20:3127292-3127233	FASTKD5
A_24_P280897	13.348173	13.416193		chr19:034080305-034080246	
A_24_P145009	4.0935874	4.0126705	NR_003502	chr7:32769148-32769207	ZNRF2P1
A_23_P420610	5.145789	5.022994	NM_138782	chr5:72286310-72286369	FCHO2
A_32_P183918	4.476744	4.528119	NR_033851	chr6:2855885-2855826	MGC39372
A_24_P163920	5.9619026	6.0201945	NM_032228	chr11:13732254-13732313	FAR1
A_23_P219060	6.8784175	7.1022043	NM_022107	chr6:32158711-32158652	GPSM3
A_24_P92183	6.672037	6.6277785	NM_001124756	chr20:43566771-43566830	PABPC1L
A_24_P925314	6.1255116	6.192753	NM_000405	chr5:150649662-150649721	GM2A
A_24_P239177	8.161015	8.344919	NM_018406	chr3:195514839-195514780	MUC4
A_24_P290013	6.131588	6.2507906		chr8:8093452-8094687	FAM86B3P
A_23_P39050	7.623898	8.109566	NM_001080493	chr19:11832463-11832404	ZNF823
A_24_P334640	5.317341	6.0466585	NM_133367	chr6:52271880-52271939	PAQR8
A_24_P128563	8.070492	7.873726	NM_012316	chr1:32638495-32638554	KPNA6
A_24_P314597	5.1442804	5.0067124	AB058740	chr1:35904770-35904711	KIAA0319L
A_33_P3371154	3.6863508	2.3900566	NM_199451	chr10:64430068-64430127	ZNF365
A_23_P37484	9.817288	9.902796	NM_014918	chr15:101716122-101716063	CHSY1
A_33_P3223522	3.5343814	3.0731564		chr5:135160732-135160791	LOC101930049
A_24_P157087	7.794285	7.8682895	NM_033355	chr2:202151838-202151897	CASP8
A_32_P33561	5.0208626	4.308138	NR_024407	chr3:101397150-101397209	ZBTB11-AS1
A_24_P172993	10.43356	10.330116	NM_022066	chr17:74385990-74385931	UBE2O
A_24_P137376	8.71446	9.058696	NM_001001485	chr3:130717172-130717231	ATP2C1
A_23_P6980	3.6338482	2.3900566	NM_001128223	chr3:75790772-75790472	ZNF717
A_24_P139901	5.9770637	6.0648212	NM_002101	chr2:127453654-127453713	GYPC
A_33_P3372212	3.2652814	2.3900566	L14723	chr19:43347505-43347446	PSG10P
A_24_P841677	5.230797	4.8501	NM_198279	chrX:19983543-19983484	CXorf23
A_33_P3412687	7.7164793	7.7527757			
A_23_P90419	2.3221061	2.3900566	NM_025245	chr19:19672597-19672538	PBX4
A_23_P14734	11.508441	11.608827	NM_015920	chr15:63447925-63447866	RPS27L
A_23_P371129	8.772888	8.811309	NM_032444	chr16:3631403-3631344	SLX4
A_23_P130689	6.9838314	6.7651105	NM_032377	chr19:11664827-11664568	ELOF1
A_33_P3346046	7.229013	7.3106174		chr17:066202331-066202272	
A_24_P396720	8.878142	8.694674	NM_002709	chr2:29025683-29025742	PPP1CB
A_33_P3333488	8.901023	8.923914	NM_001040427	chr16:1859176-1859117	HAGH
A_33_P3235841	7.9490924	7.778471	NR_033396	chr11:65009498-65009557	SLC22A20
A_33_P3808371	8.160403	8.172868	AL833480	chr16:34388638-34388697	LOC283911
A_32_P121303	10.043356	9.876237	NM_007172	chr22:45583785-45583843	NUP50
A_33_P3406458	4.463002	4.7994328	NM_152667	chr20:25593924-25593865	NANP
A_33_P3475737	7.358782	7.3434234	NM_001040441	chr1:33066168-33066227	ZBTB8A
A_23_P126212	4.8355975	4.895363	NM_022111	chr1:36204176-36204117	CLSPN
A_24_P168416	5.806246	5.595883	NM_005809	chr19:12912037-12911978	PRDX2
A_33_P3352307	4.6641464	4.8289337	NM_002953	chr1:26898034-26898093	RPS6KA1

A_33_P3283300	5.3577065	5.3707533		chr10:120081877-120081818	FAM204A
A_24_P115511	4.9051003	5.1453376	NM_016322	chr9:123940477-123940418	RAB14
A_24_P135322	8.082838	8.649474	NM_001024629	chr10:33502350-33496622	NRP1
A_23_P24384	9.0832405	8.863527	NM_032251	chr11:64124935-64124994	CCDC88B
A_24_P205364	5.2630157	5.4664574	NM_004169	chr17:18236572-18236513	SHMT1
A_33_P3343066	8.784468	8.812566	NM_003988	chr10:102589603-102589662	PAX2
A_23_P13271	9.864635	9.86725		chr11:056739031-056739090	
A_23_P28730	8.838139	9.008517	NM_020713	chr20:62588356-62588297	ZNF512B
A_32_P209208	4.9403667	4.450732	NM_032606	chr12:75669900-75669841	CAPS2
A_24_P333494	9.425997	9.599537	NM_018994	chr1:16577198-16577139	FBXO42
A_33_P3397520	4.9088283	4.8395214	NM_198699	chr21:46117448-46117507	KRTAP10-12
A_23_P26928	5.731429	5.425268	XM_005257352	chr17:42982663-42982722	CCDC103
A_23_P96965	7.755634	7.643187	NM_030786	chr1:33146361-33146302	SYNC
A_24_P27977	5.374524	4.450347	NM_003307	chr21:45862211-45862270	TRPM2
A_33_P3297930	5.2403016	4.893116	NM_005202	chr1:36563361-36563302	COL8A2
A_33_P3326643	4.078906	4.057425		chr11:17667339-17667398	OTOG
A_33_P3258417	6.149981	6.0226874		chr20:047956915-047956974	
A_33_P3267760	4.725143	4.4631577		chrX:39909128-39909069	BCOR
A_32_P809810	4.335229	3.893187	AK096255	chr9:88801220-88801279	LOC100130433
A_23_P60002	9.707296	9.690945	NM_014673	chr8:109498816-109498875	EMC2
A_33_P3268629	15.018649	15.291574		chr7:39832468-39832527	LINC00265
A_23_P74981	6.830694	6.790106	NM_033213	chr1:247200908-247200849	ZNF670
A_23_P415643	8.123516	8.149119	NM_152652	chr16:30410539-30410598	ZNF48
A_33_P3318668	4.849268	4.424256	NM_199001	chr9:140120703-140120762	C9orf169
A_23_P72627	11.931005	11.5150795	NM_004712	chr17:79669087-79669146	HGS
A_23_P94141	5.765334	5.5907073	NM_001205262	chr8:95444506-95444447	RAD54B
A_33_P3262555	6.4570866	6.1423016	NM_001174118	chr19:1555636-1555577	MEX3D
A_33_P3350638	5.983322	5.9888964		chr16:29810045-29810104	KIF22
A_32_P512061	6.501586	7.038975	NR_002188	chr1:155184923-155184864	GBAP1
A_24_P277747	4.6160293	4.1456714	NM_006586	chr6:42902318-42903352	CNPY3
A_33_P3250015	4.6578655	4.310754	BM473780	chrUn_gl000214:49700-49759	
A_33_P3234521	5.2461796	4.8973045	AK123202	chr15:66618821-66618880	DIS3L
A_24_P405298	8.499219	8.824269	NM_002709	chr2:29024034-29024093	PPP1CB
A_23_P435501	6.5925574	7.185726	NM_198941	chr20:43135555-43135496	SERINC3
A_32_P50924	12.802589	13.024225	NM_001011724	chr13:53217666-53217725	HNRNPA1L2
A_33_P3423825	5.805595	6.046501		chr6:033291182-033291123	
A_24_P277367	8.991687	9.136642	NM_002994	chr4:74861957-74861898	CXCL5
A_33_P3418194	6.1405997	6.2414656	NM_023946	chr8:143845815-143845756	LYNX1
A_33_P3336103	12.069288	12.209432	NM_032862	chr8:144682015-144682074	TIGD5
A_24_P942805	6.854923	6.8347106	NM_001042371	chr16:2262365-2262306	PGP
A_24_P183128	11.112316	11.300191	NM_016619	chr4:84015842-84012077	PLAC8
A_23_P162279	7.448288	7.3369412	NM_018318	chr12:28702878-28702937	CCDC91
A_23_P36888	6.049731	6.215446	NM_138371	chr12:47630220-47630279	PCED1B
A_24_P6083	4.3757524	4.6504917	NM_213720	chr22:24108416-24108357	CHCHD10
A_33_P3261700	7.5106153	7.577094	NM_030816	chr1:70727610-70727551	ANKRD13C
A_33_P3209214	5.388963	5.5173454	NM_001080495	chr7:5353122-5353063	TNRC18
A_33_P3289034	4.7432003	5.0558286	NM_001253837	chr10:102049840-102049781	PKD2L1
A_24_P237804	2.3221061	2.3900566	NM_174981	chr21:14987781-14987839	POTED
A_33_P3342613	10.863501	10.697929	NM_013373	chr22:20133887-20133946	ZDHHC8
A_23_P503182	10.973721	10.906133	NM_021962	chr17:906950-906891	ABR
A_24_P220058	11.5372305	11.455188	NM_012325	chr20:31438009-31438068	MAPRE1
A_23_P107313	6.705792	6.781881	NM_006923	chr17:26982476-26982417	SDF2
A_23_P165783	9.164589	8.661335	NM_024101	chr2:238457898-238461015	MLPH
A_23_P21207	3.12431	3.620914	NM_003335	chr3:49845476-49845325	UBA7
A_23_P99163	8.985969	9.169614	NM_018370	chr12:102317112-102317171	DRAM1
A_33_P3278122	3.308734	3.2109916		chr11:055623885-055623944	
A_23_P158570	5.717761	5.8017426	NM_001609	chr10:124813233-124813292	ACADSB
A_33_P3322283	8.014683	7.8514805	NM_001271650	chr3:28364145-28364086	AZI2
A_32_P494620	3.5410755	3.3226795	NM_182548	chr6:35782332-35782391	LHFPL5

A_33_P3373446	4.0590034	2.873044	XM_005266615	chr13:64321196-64321255	
A_33_P3384721	11.882737	12.019048	NM_005004	chr10:102286309-102286250	NDUFB8
A_23_P166491	4.9141607	4.538168	NM_015705	chr22:40804671-40804826	SGSM3
A_23_P135548	7.5338793	7.7353764	NM_000110	chr1:97543959-97543900	DPYD
A_33_P3263756	8.898661	8.879642	NM_017908	chr19:58992532-58992591	ZNF446
A_23_P163546	9.649965	9.532559	NM_005679	chr16:84211547-84211488	TAF1C
A_23_P162211	2.3221061	2.3900566	NM_018050	chr12:12483006-12482947	MANSC1
A_33_P3413104	3.7258053	3.4704285		chr18:005298653-005298594	
A_33_P3279124	8.844103	9.179171	NM_001169107	chr10:46250458-46250517	FAM21C
A_23_P70688	7.661851	7.3569975	NM_004271	chr6:6655116-6655175	LY86
A_23_P432005	3.868236	3.4604378	NM_152670	chr2:88828571-88828630	TEX37
A_24_P161355	12.467993	12.569995		chr3:062912274-062912333	
A_33_P3419912	3.9002485	3.8397765	NM_001199815	chr7:30537446-30537387	GGCT
A_33_P3374952	13.802238	13.903305	NM_173484	chr1:44600730-44600789	KLF17
A_33_P3554318	3.8089044	3.455466	NM_006458	chr11:6470292-6470233	TRIM3
A_23_P133245	10.481125	10.718498	NM_006083	chr5:140041490-140041549	IK
A_33_P3284518	5.668032	5.7956977	NM_001006947	chr12:100463771-100463712	UHRF1BP1L
A_23_P207564	3.6887453	3.5460422	NM_002984	chr17:34432718-34432777	CCL4
A_24_P380061	4.70164	4.5739307	NM_001025616	chr4:86923279-86923338	ARHGAP24
A_24_P386771	9.690945	10.169423	NM_002710	chr12:111160054-111159995	PPP1CC
A_23_P206830	8.4058275	8.085194	NM_016069	chr16:4390983-4390924	PAM16
A_33_P3210810	5.03683	5.1413255	NR_046213	chr1:203700012-203700071	LINC00260
A_33_P3244194	4.5206065	5.155482	NM_198402	chr3:123213422-123213363	PTPLB
A_33_P3326312	7.048508	7.2213545		chr5:121440706-121440647	
A_33_P3303259	6.0119514	5.990573		chr13:25044112-25044053	PARP4
A_24_P222599	8.661335	8.759468	NM_002613	chr16:2652575-2652634	PDPK1
A_23_P41908	5.937695	6.202997	NM_018691	chr5:153371834-153371775	FAM114A2
A_23_P97021	7.042016	6.682801	NM_024852	chr1:36521552-36521611	AGO3
A_33_P3341676	8.069038	7.4548507	NM_001171894	chr15:100256486-100256545	MEF2A
A_32_P190461	5.4183226	5.769771		chr1:222663141-222663082	
A_32_P84454	3.9626632	3.3972921	NR_033240	chr14:37642565-37642624	SLC25A21-AS1
A_33_P3233010	6.7934976	7.042968	NM_001242867	chr19:55367307-55367366	KIR3DL2
A_33_P3391496	4.728433	4.8287263		chr5:168093778-168093719	SLIT3
A_23_P332374	5.300144	5.038973	NM_007147	chr19:52092551-52092610	ZNF175
A_33_P3408320	7.642337	7.10424	NM_198207	chr19:18988778-18988719	CERS1
A_32_P79434	6.4743757	5.0665193	NM_002847	chr7:157331972-157331913	PTPRN2
A_33_P3310981	5.679315	5.1725016	NM_013250	chr11:6976945-6977004	ZNF215
A_24_P328492	8.317878	7.7141495	NM_144949	chr2:46989289-46989348	SOCS5
A_33_P3333600	3.9356117	3.2756138	EL950728	chr2:42181216-42181157	
A_32_P80597	5.7792945	5.702935	AK026192	chr4:110967485-110967426	ELOVL6
A_33_P3352085	2.8094707	3.2727957		chr13:114523345-114523286	
A_33_P3239587	12.518466	12.646112	NM_001008529	chr17:74675712-74675653	MXRA7
A_23_P331092	7.9879904	8.227285	NM_152571	chr9:139380078-139380137	C9orf163
A_33_P3353343	8.345644	8.216266	NM_016333	chr16:2810440-2810499	SRRM2
A_23_P65918	8.436545	8.362057	NM_002220	chr15:41795640-41795699	ITPKA
A_33_P3371270	8.727272	8.433685		chr22:31375241-31375300	TUG1
A_23_P59397	6.9359264	6.888995	NM_031924	chr6:159398519-159398460	RSPH3
A_23_P205567	5.4379554	5.648266	NM_006255	chr14:62017283-62017342	PRKCH
A_33_P3268838	5.875822	5.8867636	NM_030594	chr15:83212038-83211979	CPEB1
A_33_P3311285	12.333307	12.077678	NM_170707	chr1:156109774-156109833	LMNA
A_24_P91991	7.1052265	6.9211273	NM_178557	chr4:2067569-2067628	NAT8L
A_23_P251486	2.9782877	3.3392787	NM_006438	chr8:120118876-120118935	COLEC10
A_33_P3379017	6.877204	7.181946	NM_015094	chr22:21803629-21803688	HIC2
A_23_P130149	6.44228	6.196987	NM_001976	chr17:4860149-4860296	ENO3
A_24_P417162	7.4946456	8.208369	NM_005647	chrX:9687472-9687531	TBL1X
A_33_P3263651	4.5149117	4.694538	NM_032108	chr19:4550259-4550200	SEMA6B
A_24_P238250	5.385521	5.483293	NM_002307	chr19:39261714-39261655	LGALS7
A_24_P287826	9.493958	9.141915	NM_018124	chr16:74655967-74655908	RFWD3
A_33_P3362601	5.6935997	5.649455		chr1:170636539-170636480	

A_23_P160466	8.744506	8.483089	NM_006996	chr1:169433321-169433262	SLC19A2
A_33_P3381454	7.8165994	7.854397	NM_017860	chr1:151022984-151023043	C1orf56
A_24_P377277	10.742546	10.892431	NM_002154	chr5:132435328-132437493	HSPA4
A_33_P3270034	12.798718	12.469933	NM_001145260	chr10:51590584-51590643	NCOA4
A_23_P369456	6.1118627	6.5072823	NM_033542	chr20:43997295-43997354	SYS1
A_23_P87072	4.907116	4.976909	NM_052959	chr11:124489847-124489906	PANX3
A_23_P131737	9.037729	9.294804	NM_016516	chr2:64120444-64120385	VPS54
A_33_P3383871	8.059056	7.751102	NM_020177	chr5:114856688-114856629	FEM1C
A_24_P365322	4.9259424	5.1233215	NM_152601	chr19:12572600-12572541	ZNF709
A_33_P3314643	4.1996098	5.4313974	NM_015417	chr20:3758211-3758152	SPEF1
A_23_P254120	9.7413645	9.652733	NM_033480	chr6:52957322-52957567	FBXO9
A_24_P63468	2.3221061	2.3900566	NM_031900	chr5:34998912-34998853	AGXT2
A_33_P3284359	3.8110104	3.1518586	AW291512	chrX:30323850-30323791	NR0B1
A_33_P3303215	9.403549	9.535932	NM_001191009	chr1:24298400-24298341	SRSF10
A_23_P136347	7.8939705	7.570449	NM_004447	chr12:15774235-15774176	EPS8
A_24_P399888	8.568335	8.215336	NM_001002876	chr22:42341956-42341289	CENPM
A_24_P662366	12.068261	12.147997		chr11:033258956-033259015	
A_33_P3396129	6.4644713	6.5700097	AB209250	chr16:57790219-57790278	KATNB1
A_23_P308097	10.486848	10.973721	NM_001009552	chr8:30648747-30643799	PPP2CB
A_23_P16834	5.2349777	5.258525	NM_022823	chr2:27715322-27715263	FNDC4
A_23_P316850	8.551384	9.017676	NM_182577	chr19:463426-463367	ODF3L2
A_33_P3292495	3.5740998	2.8856263	AK096395	chr12:6692199-6692258	LOC100127974
A_33_P3310989	10.454265	10.286337	NM_014847	chr1:154243269-154243328	UBAP2L
A_33_P3587376	12.97972	12.868079	NR_024214	chr19:48427077-48427136	SNAR-A3
A_23_P25433	7.40843	7.3109226	NM_020374	chr12:4598660-4598601	C12orf4
A_33_P3302892	3.9223938	3.5967045	BX648241	chr14:52481015-52480956	NID2
A_23_P15714	9.528677	9.169292	NM_006178	chr17:44834580-44834639	NSF
A_23_P135104	10.439302	10.446409	NM_016034	chr9:138396341-138396400	MRPS2
A_23_P41009	4.9749675	5.3488865	NM_015926	chr3:51718629-51733459	TEX264
A_24_P172481	3.043923	3.005788	NM_006074	chr11:5731591-5731650	TRIM22
A_33_P3336720	5.001982	5.0795765	NM_021175	chr19:35775986-35776045	HAMP
A_23_P58521	6.2672176	6.2181005	NM_000082	chr5:60186775-60186716	ERCC8
A_33_P3360997	4.8351874	4.625274	NM_199292	chr11:2190975-2190916	TH
A_33_P3343305	5.1897516	5.1577024		chr19:040391999-040391940	
A_33_P3319155	9.477348	9.607321	NM_001040649	chr2:271879-271938	ACP1
A_23_P22671	10.167114	9.739009	NM_005638	chrX:155172553-155172612	VAMP7
A_33_P3216517	5.502879	5.706563	XM_005251664	chr9:127023798-127023857	NEK6
A_24_P382026	3.940104	3.6469815	NM_020679	chr17:73264206-73263966	MIF4GD
A_33_P3298128	5.9591675	5.287554	NM_002223	chr12:26488383-26488324	ITPR2
A_24_P349821	5.0296793	4.867543	NM_004294	chr13:41834844-41834785	MTRF1
A_23_P3532	10.736747	11.201412	NM_004862	chr16:11642876-11642817	LITAF
A_33_P3766959	9.912645	9.921489	NM_018319	chr14:90509587-90509646	TDP1
A_24_P340066	9.287509	9.285362	NM_001421	chrX:129198964-129198905	ELF4
A_24_P109417	3.75867	4.0223274	NM_198545	chr1:11779765-11779824	DRAXIN
A_33_P3329834	8.729915	8.888165	NM_018061	chr1:109238756-109238815	PRPF38B
A_33_P3375435	5.647627	5.4920416	NM_133447	chr10:081666393-081666452	AGAP11
A_23_P131435	6.139139	6.1688943	NM_014880	chr2:160628550-160628491	CD302
A_23_P167692	6.323497	6.944945	NM_002752	chr5:179669726-179669667	MAPK9
A_24_P229669	6.455486	6.2451625	NR_024074	chr15:23262470-23262529	GOLGA8I
A_24_P396994	11.469954	11.711741	NM_001690	chr3:113530539-113530598	ATP6V1A
A_24_P307384	9.339514	9.231047		chr21:030260173-030260111	
A_23_P67127	6.660585	6.4238467	NM_173633	chr19:42829141-42829200	TMEM145
A_33_P3415859	9.482659	9.819782	AK090476	chr16:3612970-3612911	NLRC3
A_32_P116660	7.1139708	7.2423673	NM_198557	chr2:152105202-152105143	RBM43
A_23_P376591	4.398946	4.600546	NM_206808	chr13:100517164-100518551	CLYBL
A_23_P41380	9.008872	9.393556	NM_002940	chr4:146049642-146049701	ABCE1
A_32_P190416	3.6842546	4.109042	NM_003980	chr6:136663444-136663419	MAP7
A_33_P3257903	6.5433707	7.074496	NM_001512	chr6:52850310-52850251	GSTA4
A_33_P3299254	6.230562	6.292882	NM_013378	chr22:24094996-24094937	VPREB3

A_33_P3224157	3.7273684	4.0857477	NM_001165969	chr17:61784055-61783996	STRADA
A_33_P3246623	4.7561336	4.2361	NM_206886	chr1:93744211-93744270	CCDC18
A_33_P3214298	11.692598	11.757889	NM_000884	chr3:49062188-49062129	IMPDH2
A_23_P46275	11.902544	11.833224	NM_001688	chr1:112003643-112003702	ATP5F1
A_23_P90062	9.10318	9.136179	NM_006145	chr19:14625862-14625803	DNAJB1
A_24_P278172	7.5915465	8.201383	NM_006007	chr9:74975604-74975545	ZFAND5
A_23_P130919	9.626364	9.776659	NM_130807	chr19:2071294-2071235	MOB3A
A_33_P3408953	5.202171	5.6141896	NM_001282879	chr22:22990096-22990155	GGTLC2
A_33_P3383756	6.399115	6.8780727	NM_017778	chr8:38178599-38175553	WHSC1L1
A_24_P127121	11.785743	11.687202		chrX:106375432-106375493	
A_33_P3299834	6.7044044	6.2192936	NM_002695	chr19:1086639-1086580	POLR2E
A_24_P78161	5.881277	5.826772	NM_001031689	chr9:26904726-26904667	PLAA
A_33_P3321293	9.825703	9.780453	NM_178229	chr1:156495257-156495198	IQGAP3
A_33_P3213392	5.6961875	5.4275928		chr13:112278396-112278455	
A_33_P3361309	11.483576	11.593349		chr11:073632669-073632610	
A_23_P84952	8.019106	7.4854198	NM_006521	chrX:48886554-48886495	TFE3
A_23_P250478	5.1575065	5.811692	NM_005391	chrX:24546208-24546267	PDK3
A_33_P3275435	7.6997433	7.9129915	NR_024586	chr7:104651052-104650993	KMT2E-AS1
A_33_P3357337	5.2812834	5.1344557	BC026272	chr6:100005205-100005146	CCNC
A_24_P184555	11.852174	11.589407	NM_002859	chr12:120648324-120648265	PXN
A_23_P395555	7.8793054	7.868469	NM_001032372	chr19:44681704-44681763	ZNF226
A_23_P43079	8.733519	8.921627	NM_017864	chr8:95886809-95888339	INTS8
A_33_P3408244	8.666458	8.960358	NR_033789	chr10:135381393-135381334	SPRNP1
A_33_P3224803	5.43723	6.0089116	NM_000265	chr7:74193443-74193502	NCF1
A_33_P3401621	9.393556	9.328773	NM_031966	chr5:68473872-68473931	CCNB1
A_33_P3209866	5.9696608	6.245355	NM_001256877	chr9:745460-745519	KANK1
A_23_P94754	2.3221061	2.3900566	NM_005118	chr9:117552298-117552239	TNFSF15
A_33_P3312743	4.106397	3.9716053	NM_033187	chr17:39324104-39324045	KRTAP4-3
A_33_P3347168	8.003413	8.338407	NM_005647	chrX:9679756-9679815	TBL1X
A_23_P887	3.8300636	3.1436357	NM_014002	chr1:206664160-206665008	IKBKE
A_33_P3303414	5.971755	5.822859	NM_005907	chr6:119499347-119499288	MAN1A1
A_33_P3210585	9.681005	9.705849	NM_014911	chr2:69685229-69685170	AAK1
A_33_P3369029	3.776004	3.9546404	NR_033579	chr15:82756803-82756862	CSPG4P8
A_33_P3365305	4.063751	3.5096278	NM_001039523	chr2:175619052-175618993	CHRNA1
A_33_P3529859	4.193158	4.2673845	NM_032590	chr12:121868139-121868080	KDM2B
A_33_P3309684	11.966198	12.082267		chr11:107779285-107779344	
A_24_P312692	9.14502	9.004953	NM_006595	chr11:43365367-43365426	API5
A_24_P272967	4.6538334	5.161545	NM_015060	chr7:32598953-32599012	AVL9
A_23_P253791	9.558947	10.311222	NM_004345	chr3:48266856-48266915	CAMP
A_33_P3423185	6.317543	5.9384727	AK024141	chr14:73079235-73079176	DPF3
A_23_P162449	9.97481	10.124041	NM_020762	chr12:64537259-64537318	SRGAP1
A_24_P413941	8.58671	7.9490924	NM_153689	chr2:200792670-200792729	C2orf69
A_33_P3380783	5.1728754	5.2782	AK128032	chr20:60948942-60948883	LOC100128184
A_33_P3408962	6.867626	6.6732945	NM_014191	chr12:52205200-52205259	SCN8A
A_33_P3216292	11.0568905	10.854712	NM_001267571	chr9:100961388-100961329	TBC1D2
A_33_P3294801	5.842947	5.9135303	AK097991	chr17:80534685-80534744	
A_23_P61050	6.6726036	6.2221794	NM_152649	chr16:74706038-74705979	MLKL
A_33_P3327818	5.828716	6.2012835	AL157466	chr9:3452364-3452305	
A_33_P3338793	5.1686964	4.377486	NM_004977	chr19:50819050-50818991	KCNC3
A_33_P3299285	3.4721158	3.240689	NM_001002901	chr1:161697301-161697360	FCRLB
A_33_P3259542	3.685542	3.9054732	NM_019069	chr3:122130796-122130737	WDR5B
A_23_P168951	6.4987936	6.82775	NM_014943	chr8:123986299-123986358	ZHX2
A_23_P133868	6.0909038	6.0994673	NM_019110	chr6:28213097-28213038	ZKSCAN4
A_24_P171873	8.08669	8.197836	NM_012176	chr5:41934337-41934396	FBXO4
A_23_P502553	8.592282	8.314498	NM_171982	chr8:27143339-27143280	TRIM35
A_24_P168726	6.238203	6.0865064	NM_181831	chr22:30077460-30077519	NF2
A_24_P148043	10.361334	10.348134	NM_014864	chr1:179045579-179045638	FAM20B
A_23_P255785	9.167845	9.500109	NM_006153	chr3:136667329-136667388	NCK1
A_23_P46928	10.154841	10.275137	NM_002627	chr10:3178781-3178840	PFKP

A_23_P16908	6.1797347	6.331165	NM_198963	chr2:39025498-39025439	DHX57
A_33_P3279441	8.201096	8.163982	NM_001012511	chr17:45014094-45014153	GOSR2
A_23_P425304	5.6825027	5.775161	NM_016169	chr10:104393028-104393087	SUFU
A_24_P83379	8.121242	8.22765	AK092019	chr4:85730194-85730135	WDFY3
A_33_P3271187	4.260016	3.868236		chr1:241906232-241906173	
A_33_P3341686	11.575249	11.4237995	NR_001564	chrX:73040565-73040506	XIST
A_33_P3319176	2.3221061	2.3900566	NM_001256760	chrX:114143005-114143064	HTR2C
A_23_P425502	9.137022	9.288903	NM_017613	chr21:34950584-34950525	DONSON
A_33_P3379251	3.5360801	3.0536704	NM_001105581	chr18:7231929-7231988	LRRC30
A_24_P333733	9.582312	9.846103	NM_005177	chr17:40674476-40674535	ATP6V0A1
A_23_P43095	2.3221061	2.3900566	NM_024721	chr8:77776697-77776756	ZFHX4
A_33_P3292739	5.4404473	5.2687063	NM_201402	chr8:11994918-11994859	USP17L2
A_33_P3237487	4.6022367	4.6470313	XR_244591	chr2:19167831-19167772	
A_24_P278192	5.835789	5.927511	NM_000476	chr9:130629235-130629176	AK1
A_33_P3396692	10.394686	10.661453	NM_003144	chr6:7289857-7289798	SSR1
A_23_P25835	7.245281	6.9838314	NM_002028	chr14:65529014-65529073	FNTB
A_32_P223140	4.324082	3.8181336	NM_145313	chr10:43691966-43691700	RASGEF1A
A_33_P3325661	4.827888	4.8560667	X51791	chr7:142364651-142364710	
A_33_P3395675	5.0067124	5.1192975	AB776856	chr22:23243336-23243395	
A_23_P36611	6.4257207	6.9556746	NM_181861	chr12:99128417-99128476	APAF1
A_23_P141863	3.6889877	3.564893	NM_014480	chr19:58774199-58774259	ZNF544
A_23_P87082	5.5980997	5.2238917	NM_022370	chr11:124749824-124750352	ROBO3
A_33_P3382513	10.61036	10.31369	NM_058192	chr16:835034-834975	RPUSD1
A_24_P178602	5.1494594	5.3862305	NM_198457	chr19:53269630-53269571	ZNF600
A_33_P3411991	4.8573313	5.0678864	NM_012197	chr9:125760974-125761033	RABGAP1
A_33_P3242748	10.266993	10.207284	NM_001267549	chr20:62330903-62330844	ARFRP1
A_33_P3356004	3.8095436	3.9832618	NR_027287	chr20:62585332-62585391	UCKL1-AS1
A_32_P8015	14.953241	15.049419	NM_001011724	chr13:53217715-53217774	HNRNPA1L2
A_24_P235783	6.0258646	5.9399705	D26121	chr11:64543888-64543829	SF1
A_33_P3248519	13.114494	12.766243	NM_005496	chr3:160132206-160132265	SMC4
A_24_P233786	12.115775	11.939297	NM_052966	chr1:184760286-184760227	FAM129A
A_33_P3316786	5.429538	5.5103707	NM_080759	chr13:72049898-72049839	DACH1
A_33_P3237580	7.523408	6.5861707	NM_001242791	chr17:47924945-47925004	FLJ45513
A_23_P367899	5.122165	5.4735756	NM_000121	chr19:11488666-11488607	EPOR
A_33_P3228709	3.882113	3.7491624	NM_001012503	chr11:71239080-71239139	KRTAP5-7
A_23_P28707	11.679579	11.788803	NM_007346	chr20:61445213-61445272	OGFR
A_23_P217901	2.3221061	2.3900566	NM_001113207	chr1:161007823-161007764	TSTD1
A_24_P410389	4.2977304	4.1683173	NM_015367	chr22:18209937-18209996	BCL2L13
A_24_P209285	9.496357	9.148009	NM_018141	chr6:42174672-42174613	MRPS10
A_23_P18887	6.833027	6.8768167	NM_022132	chr5:70945998-70948542	MCCC2
A_23_P133814	10.930885	10.774226	NM_003531	chr6:26045965-26046024	HIST1H3C
A_23_P143147	8.751556	8.9671335	NM_006602	chr20:61472682-61472623	TCFL5
A_33_P3335621	5.5505266	5.4802513	NM_170750	chrX:107332042-107331983	PSMD10
A_33_P3267482	10.447179	10.22	NM_032435	chr1:233520417-233520476	KIAA1804
A_23_P93269	5.924569	5.616593	NM_003447	chr6:28057187-28057246	ZNF165
A_23_P250948	6.220872	5.9291806	NM_001003694	chr3:9788978-9789037	BRPF1
A_33_P3514859	6.638306	6.5530725	BX538329	chr8:90729687-90729628	LOC100506342
A_33_P3411890	4.17778	3.584369		chr1:161412216-161412157	
A_23_P61508	2.3221061	2.3900566	NM_017581	chr4:40356665-40356724	CHRNA9
A_23_P79661	8.549762	8.675222	NM_019044	chr2:118673442-118673383	CCDC93
A_33_P3222105	12.73136	12.712016	NM_001412	chrX:20146233-20146174	EIF1AX
A_23_P97265	10.204594	9.85144	NM_182679	chr1:156564382-156564323	GPATCH4
A_23_P76749	7.0564175	6.666066	NM_020692	chr14:69821071-69821130	GALNT16
A_33_P3363680	3.8080125	3.512628		chr1:1002000-1002059	XLOC_014512
A_24_P224998	5.822859	5.3628435		chrX:100650477-100650536	
A_24_P213763	7.02382	6.9143105	BC013798	chr22:22279738-22279679	
A_33_P3376239	8.15397	8.326058	NM_182931	chr7:104754468-104754527	KMT2E
A_23_P80773	9.910433	9.875506	NM_021203	chr3:133539152-133539211	SRPRB
A_23_P4962	5.373209	5.6057014	NM_153447	chr19:56569768-56572812	NLRP5

A_33_P3227899	5.314597	4.96362	NR_015361	chr9:69174282-69174223	LOC440896
A_24_P336113	8.457846	8.584265	NM_012295	chr22:24574422-24574481	CABIN1
A_24_P188071	14.780634	14.707729	NM_032704	chr12:49667030-49667089	TUBA1C
A_24_P332926	7.5904126	7.6255507	NM_014884	chr19:19115001-19112449	SUGP2
A_23_P215227	9.656423	9.929586	NM_005494	chr7:157178557-157178616	DNAJB6
A_33_P3393245	6.1589174	6.492014	NM_015050	chr6:37446962-37447021	CMTR1
A_33_P3305117	12.32571	12.523547	AK092835	chr9:32444251-32444192	
A_33_P3339725	2.3221061	2.3900566		chr18:53763445-53763504	
A_23_P21473	8.094422	8.525805	NM_024491	chr3:138219364-138219305	CEP70
A_23_P10135	7.1016593	7.1168222	NM_001001349	chr17:40176199-40176258	NKIRAS2
A_32_P155811	8.485673	7.986657	NM_012120	chr6:47594826-47594885	CD2AP
A_24_P391960	13.106858	13.114494		chr1:182305357-182305416	
A_24_P295590	6.7020383	6.7733545	NM_032023	chr10:45489768-45489827	RASSF4
A_33_P3233005	5.8425093	6.096609	NM_017643	chr17:49270257-49270198	MBTD1
A_33_P3340164	8.64419	8.800205	NM_001199958	chr3:179332782-179332841	NDUFB5
A_23_P390621	5.0371723	5.480764	NM_145048	chr4:20729183-20729242	PACRGL
A_23_P127840	4.0644126	4.147717	NM_013249	chr11:7021342-7021283	ZNF214
A_24_P58647	10.37703	10.494842		chr6:015935330-015935391	
A_33_P3255664	3.9150743	2.3900566	AK093508	chr1:164610621-164610680	PBX1
A_23_P152305	2.3221061	2.3900566	NM_001797	chr16:64980928-64980869	CDH11
A_24_P237936	4.730604	4.8027554	NM_175769	chr2:27375674-27375733	TCF23
A_33_P3252043	14.054972	14.185693	NM_000918	chr17:79801111-79801052	P4HB
A_33_P3378800	14.707729	14.744372	NM_004068	chr3:183901819-183901878	AP2M1
A_24_P306561	10.7724695	10.95074	NM_014972	chr16:89940253-89949803	TCF25
A_24_P357536	4.95572	5.0347986	NM_001190274	chr2:48066061-48066002	FBXO11
A_23_P305140	8.182989	8.13145	NM_144591	chr10:104623852-104623911	C10orf32
A_33_P3404759	3.643443	4.382408		chr1:243327622-243327563	
A_33_P3382125	5.937294	5.877807		chr17:042722899-042722958	
A_23_P25396	5.0860157	5.87568	NM_005123	chr12:100957191-100957250	NR1H4
A_23_P153026	9.771303	9.791756	NM_000152	chr17:78093595-78093654	GAA
A_33_P3269976	4.0852013	3.2811544	BG675116	chr11:68451927-68451986	
A_23_P34930	9.60269	9.397934	NM_005872	chr1:115110626-115110567	BCAS2
A_33_P3297888	6.57842	6.5000267	NM_176816	chr5:68578830-68578771	CCDC125
A_33_P3289422	7.263839	6.9662576	NM_001040185	chr19:53915117-53915176	ZNF765
A_24_P349743	13.092664	13.250324	NM_002295	chr3:39453264-39453441	RPSA
A_23_P204448	11.336784	11.260879	NM_016237	chr12:121746267-121746208	ANAPC5
A_23_P72068	3.807558	3.40978	NM_001500	chr6:1960186-1960127	GMDS
A_23_P157628	8.2853	8.576487	NM_004942	chr8:7754081-7754140	DEFB4A
A_33_P3233731	3.6670442	4.067804		chr1:230141917-230141976	
A_33_P3233891	9.513724	9.376117	NM_006947	chr4:57369756-57369815	SRP72
A_33_P3253975	10.082978	10.492539	NM_000019	chr11:108012358-108012417	ACAT1
A_33_P3285018	5.355989	5.3346024	XM_005276654	chr19:53409388-53409329	ZNF888
A_33_P3833256	4.6358366	4.002361		chr11:9776384-9776325	LOC440028
A_33_P3407618	4.7418823	4.0208216	NR_102747	chr15:82974483-82974542	LOC727751
A_24_P186342	3.9742303	3.558571	NM_020917	chr19:36827646-36827587	ZFP14
A_33_P3325952	3.8635516	4.1232033	AK022213	chr12:123887812-123887753	
A_23_P204998	5.7975492	6.3706837	NM_005766	chr13:99101511-99101570	FARP1
A_23_P309396	3.9820237	4.1512446	NM_178857	chr8:10464670-10464611	RP1L1
A_23_P56249	9.902269	9.970684	NM_001281	chr19:36616418-36616621	TBCB
A_23_P328074	7.988655	7.917565	NM_002968	chr16:51170634-51170575	SALL1
A_33_P3344579	5.8434305	5.238506	NM_000108	chr7:107561430-107561489	DLD
A_33_P3403075	9.462714	9.308984	NM_018304	chr17:57278961-57279020	PRR11
A_24_P787947	5.597333	6.567259	NM_001005404	chr17:57478707-57478766	YPEL2
A_33_P3878772	5.8679338	6.066251	NM_004972	chr9:5127355-5127414	JAK2
A_33_P3242548	3.8215182	3.8877559	NM_018699	chr4:121774633-121774574	PRDM5
A_33_P3274127	4.5554094	4.31917	NM_198572	chr8:145096210-145096269	SPATC1
A_23_P37983	12.129554	12.282789	NM_005947	chr16:56686948-56687007	MT1B
A_23_P72584	5.228609	5.4247904	NM_001039844	chr10:15118665-15118724	ACBD7
A_24_P231546	9.207945	10.431498	NM_016490	chr2:97541686-97541627	FAM178B

A_24_P941787	9.039458	8.82098	NM_003913	chr6:4065127-4065186	PRPF4B
A_23_P433218	4.7839394	4.5306187		chr2:71256464-71256523	OR7E91P
A_32_P212802	8.281007	8.517958	XM_005276001	chr7:66745066-66745125	LOC101930277
A_32_P198303	7.2797995	7.148526	NM_181706	chr11:31454021-31454080	DNAJC24
A_24_P167052	10.090082	10.200877	NM_139312	chr10:27399712-27399653	YME1L1
A_23_P63232	7.286308	7.201231	NM_130898	chr1:153946666-153946725	CREB3L4
A_23_P7250	2.8265624	2.3900566	NM_001263	chr4:85566400-85566459	CDS1
A_23_P553	8.467376	8.109865	NM_025150	chr1:150479514-150479573	TARS2
A_24_P254278	6.7709293	7.018513	NM_203327	chr20:4833375-4833316	SLC23A2
A_33_P3279362	3.5208948	3.8844266	NR_027021	chr21:31121174-31121233	GRIK1-AS1
A_24_P398950	8.613574	8.638212	NM_001039675	chr15:91496887-91496946	UNC45A
A_33_P3302681	6.284218	6.41404		chr9:82267658-82267714	TLE4
A_33_P3244424	4.0752845	2.897799	NM_003094	chr1:203840218-203840277	SNRPE
A_33_P3226060	7.9945493	7.954262		chr7:141170527-141170586	TMEM178B
A_33_P3299977	8.587709	8.473644	NM_024635	chr9:88637030-88637089	NAA35
A_23_P259272	11.441093	11.625049	NM_018639	chr12:118470837-118470778	WSB2
A_23_P342053	7.170895	7.3614907	NM_032626	chr16:24557545-24560286	RBBP6
A_33_P3350758	5.589144	5.3416185	NM_170692	chr1:178442587-178442646	RASAL2
A_33_P3331791	11.185318	11.357716		chr11:003580060-003580118	
A_33_P3269278	5.0807567	5.0121384	NM_153257	chr19:37129746-37129687	ZNF461
A_23_P202206	2.3221061	2.3900566	NM_183239	chr10:106057346-106057405	GSTO2
A_33_P3240497	9.037489	9.728345	AK095730	chr1:45349927-45349868	
A_33_P3629131	6.8491187	6.8831882	BX484257	chr17:15374090-15374149	CDRT3
A_23_P106922	3.0581608	2.9208517	NM_021615	chr16:75507046-75507022	CHST6
A_23_P54963	6.135766	5.7848825	NM_032478	chr17:73897347-73897288	MRPL38
A_32_P105195	5.67811	4.513504	NM_014829	chr5:134166478-134166537	DDX46
A_33_P3306078	4.143141	4.0131817	NR_002147	chr7:98895535-98895594	MYH16
A_24_P303989	6.053719	6.6684184	NM_005180	chr10:22617587-22617995	BMI1
A_33_P3719083	5.815063	5.341107	NM_139320	chr15:30654534-30654475	CHRFAM7A
A_33_P3256888	4.171334	4.0692396		chr9:067343602-067343543	
A_33_P3342481	3.5735462	3.2299857		chr15:020346394-020346335	
A_33_P3232294	4.8215637	4.9498186	NM_002223	chr12:26875390-26875331	ITPR2
A_24_P152635	7.4698744	7.4178104	NM_015959	chr11:57507794-57507853	TMX2
A_23_P86900	7.946617	8.341457	NM_006876	chr11:66113053-66112994	B3GNT1
A_33_P3331282	8.435419	8.7206135	NR_034085	chr5:43016241-43016182	LOC648987
A_32_P174908	9.973049	9.768059	NM_015045	chr10:88195144-88195085	WAPAL
A_23_P31686	6.253069	5.8743153	NM_021174	chr8:22477535-22477594	CCAR2
A_23_P70127	7.1894345	6.8124423	NM_017510	chr5:177022392-177022451	TMED9
A_33_P3369731	4.679923	4.2755194		chr5:176036413-176036472	
A_23_P256391	9.132524	9.149721	NM_002078	chr3:37407741-37407797	GOLGA4
A_33_P3243405	4.8061857	4.291984	NM_007264	chr12:57390319-57390378	GPR182
A_33_P3250730	7.331046	7.603554	NM_182847	chr2:220403427-220403486	ASIC4
A_24_P72394	4.9921308	5.412839	NM_021163	chr7:5106063-5106122	RBAK
A_33_P3241651	3.3426754	3.885419	AL832596	chrX:99940070-99940011	SYTL4
A_23_P6303	13.37882	13.2274275	NM_001025204	chr21:44513222-44513163	U2AF1
A_33_P3390773	5.32281	4.789015	NM_014818	chr11:8637153-8637094	TRIM66
A_33_P3293391	5.0993657	5.41534	BC008503	chr10:48186605-48186664	BMS1P2
A_33_P3301346	3.7612023	3.3550348		chr2:238743452-238743511	RBM44
A_24_P402080	9.766874	9.60557	NM_001025100	chr18:74724904-74724845	MBP
A_33_P3357678	5.9802628	5.5525427	NM_207338	chr15:66840775-66840716	LCTL
A_23_P155900	4.1487174	4.0247326	NM_053036	chr4:73013257-73013316	NPFFR2
A_24_P56281	4.3591633	3.9529974	NM_001002259	chr12:30867985-30867926	CAPRIN2
A_33_P3336968	10.8533325	11.711084	NM_182916	chr3:3170812-3170871	TRNT1
A_33_P3315268	4.676511	3.6048348	NM_173352	chr12:53232819-53232760	KRT78
A_24_P79855	5.3356514	4.7999916	BC104430	chr1:8441038-8440979	
A_33_P3392391	2.3221061	2.3900566	NM_001199752	chr19:50216029-50216088	CPT1C
A_23_P212844	8.776291	8.83892	NM_006342	chr4:1741478-1741717	TACC3
A_24_P271363	9.185095	9.560816	NM_003818	chr20:5171891-5171950	CDS2
A_33_P3272990	7.359719	7.3896704	NM_012181	chr19:18642801-18642742	FKBP8

A_33_P3305521	5.335009	5.4710045	AB029007	chr19:32847630-32847689	ZNF507
A_23_P137532	6.5687633	6.6414237	NM_000302	chr1:12034947-12035006	PLOD1
A_23_P426196	5.3945556	5.4857993	NM_015016	chr19:18261693-18261752	MAST3
A_33_P3349002	4.134432	3.650331		chr1:025735196-025735137	
A_23_P29684	5.8743153	5.60033	NM_015873	chr3:38048466-38048525	VILL
A_23_P169428	11.110586	11.117815	NM_015679	chr9:131071685-131071626	TRUB2
A_23_P71926	6.309174	6.278937	NM_002732	chr9:71627540-71627481	PRKACG
A_23_P138631	9.995324	10.101682	NM_005445	chr10:112362651-112362710	SMC3
A_33_P3212500	3.7771246	3.8130207	AK127934	chr3:50190053-50189994	LOC100129060
A_33_P3300893	4.570512	3.7600315	NM_130475	chr11:47345326-47345385	MADD
A_33_P3359219	8.084883	8.579671	NM_001102560	chr8:38124845-38124786	PPAPDC1B
A_23_P103672	4.1883764	4.0275054	NM_006617	chr1:156638900-156638841	NES
A_33_P3311618	5.777771	5.9324846	NM_004582	chr1:76254921-76254980	RABGGTB
A_23_P133474	2.3221061	2.3900566	NM_002084	chr5:150407859-150407918	GPX3
A_33_P3220422	6.494276	6.6979	NM_182595	chr7:53104196-53104255	POM121L12
A_32_P220696	10.67273	10.229553	NM_017489	chr8:73959611-73959670	TERF1
A_23_P127697	11.497607	11.996162	AB529247	chr11:55822553-55822612	
A_23_P40217	3.549402	3.0973983	NM_018431	chr20:53267203-53267262	DOK5
A_32_P99432	9.205869	9.207945	NM_174894	chr19:7747537-7747596	TRAPPC5
A_33_P3489646	5.497038	5.478556	NM_001080547	chr11:47376523-47376464	SPI1
A_24_P91472	8.999543	9.221506	NM_032306	chr19:6374824-6374883	ALKBH7
A_23_P64669	8.33489	8.090637	NM_017612	chr12:122957778-122957719	ZCCHC8
A_33_P3270311	6.0716825	5.800823	NM_020760	chr2:197064113-197064054	HECW2
A_23_P501193	8.7366	8.721614	NM_170741	chr17:68131523-68131582	KCNJ16
A_23_P404667	3.8466387	3.7868943	NM_001197	chr22:43525495-43525554	BIK
A_33_P3384617	5.803716	4.555053		chr13:19380231-19380172	XLOC_014512
A_33_P3257503	8.069704	7.870595	NR_003930	chr10:29711188-29711247	PTCHD3P1
A_33_P3389689	3.7107906	3.6981108	NM_022356	chr1:43225059-43225000	LEPRE1
A_33_P3216297	7.990084	7.744946	NM_001018077	chr5:142657577-142657518	NR3C1
A_24_P33156	3.8370452	4.4117317	NM_001010982	chr17:76202053-76202112	AFMID
A_33_P3334342	4.8540516	4.989785		chr17:017286802-017286861	
A_24_P352445	9.249599	9.345906	NM_172177	chr12:93895832-93895891	MRPL42
A_33_P3395201	4.7438927	4.738653	DB026495	chr12:48399465-48399406	
A_23_P111843	10.417723	10.645393	NM_006349	chr7:100867339-100867398	ZNHIT1
A_23_P412577	6.7149825	6.6639385	NM_173505	chr18:21180440-21180381	ANKRD29
A_24_P91985	3.4368951	3.1478727	NM_170710	chr4:177103712-177103771	WDR17
A_23_P106694	7.7829137	7.6892924	NM_002768	chr16:89711130-89711071	CHMP1A
A_24_P177585	6.32574	6.7792616	NM_182625	chr2:17963080-17963139	GEN1
A_24_P2648	10.962843	11.039137	NM_005401	chr1:214551312-214549732	PTPN14
A_24_P123245	8.322578	8.210048	NM_031370	chr4:83274916-83274857	HNRNPD
A_33_P3269723	6.9355702	6.4342012	NM_001042697	chr17:15879964-15879905	ZSWIM7
A_33_P3309911	3.8192582	3.0059323	NM_001013407	chr1:13368919-13368978	PRAMEF5
A_23_P12635	8.9197855	9.034696	NM_024326	chr10:104182786-104182845	FBXL15
A_23_P38219	9.091655	9.127249	NM_006445	chr17:1554538-1554479	PRPF8
A_23_P207600	11.321251	11.494883	NM_002815	chr17:30807187-30807537	PSMD11
A_23_P1638	7.7918043	8.29029	NM_031492	chr11:66432668-66432609	RBM4B
A_24_P306469	11.86194	11.880163		chr3:143574757-143574698	
A_33_P3289144	6.9618726	7.070023		chr3:068194525-068194584	
A_32_P135818	13.238498	13.34982	NM_001006	chr4:152024067-152024126	RPS3A
A_24_P70002	8.729713	9.549246	NM_014572	chr13:21547988-21547929	LATS2
A_23_P77430	9.855939	9.780886	NM_019023	chr16:68391062-68391121	PRMT7
A_33_P3406778	4.7057543	4.137748	AK023843	chr14:75413496-75413437	PGF
A_23_P89762	4.29975	4.2776318	NM_194449	chr18:60647180-60647239	PHLPP1
A_23_P338890	11.119062	10.970736	NM_002827	chr20:49200887-49200946	PTPN1
A_23_P315345	3.6105185	4.2635503	NM_006223	chrX:71417550-71417609	PIN4
A_23_P357985	6.3129783	6.342249	NM_001037866	chr2:110583522-110583581	RGPD6
A_23_P317465	8.123161	8.39199	NM_016530	chr15:63559302-63559361	RAB8B
A_23_P159039	11.594166	11.526488	NM_182706	chr8:144873202-144873143	SCRIB
A_33_P3398065	5.484569	4.989251	NM_001003397	chr6:125550352-125550411	TPD52L1

A_33_P3288219	6.7845907	4.8721523	AK127589	chr19:644430-644371	FLJ45684
A_23_P70007	9.852472	9.934178	NM_012484	chr5:162918837-162918896	HMMR
A_23_P363954	3.775573	2.982167	NM_003251	chr11:77775305-77775364	THRSP
A_33_P3341144	5.85527	5.908025	NM_004324	chr19:49464454-49464513	BAX
A_33_P3395883	9.294127	9.080538		chr17:060214338-060214279	
A_33_P3422466	4.0273347	3.8617039	NM_173855	chr12:122090698-122090639	MORN3
A_23_P74088	4.2916856	4.4606323	NM_006983	chr1:1569668-1569846	MMP23B
A_23_P26994	2.3221061	2.3900566	NM_031498	chr17:47284034-47283975	GNGT2
A_23_P58579	6.2636576	6.543509	NM_032765	chr5:180683780-180683721	TRIM52
A_23_P363365	3.8796766	4.0962114	NM_016568	chr5:33937877-33937936	RXFP3
A_23_P251836	3.572587	3.8748732	NM_006174	chr4:164272405-164272464	NPY5R
A_33_P3335865	7.763868	7.1499596	NM_001006657	chr2:20110205-20110146	WDR35
A_23_P125265	12.911448	13.092664	NM_002266	chr17:66042658-66042717	KPNA2
A_24_P322771	9.453505	9.291919	NM_003225	chr21:43783499-43783440	TFF1
A_33_P3393350	10.603556	10.536964	NM_138769	chr16:724111-724170	RHOT2
A_33_P3610768	4.91321	4.7640047	AK124265	chr12:89746783-89746842	LOC100131490
A_24_P330691	12.902962	13.119983	NM_006937	chr17:73164192-73164133	SUMO2
A_23_P349083	8.745718	8.623438	NM_138782	chr5:72385939-72385998	FCHO2
A_23_P120364	12.561302	12.698034	NM_024299	chr20:62153440-62153499	PPDPF
A_33_P3356210	2.3221061	2.3900566	NM_001202439	chr11:17398685-17398744	NCR3LG1
A_23_P140848	7.65073	7.5454154	NM_005792	chr16:82182215-82182156	MPHOSPH6
A_23_P30995	8.71665	8.990627	NM_016230	chr6:84665141-84669561	CYB5R4
A_33_P3807268	11.521332	11.493077	NR_033424	chr1:102359662-102359720	DNAJA1P5
A_23_P255503	6.4371247	6.323139	NM_002572	chr11:117038500-117038559	PAFAH1B2
A_24_P181998	9.427806	9.491156	NR_024448	chr22:24002114-24002055	GUSBP11
A_24_P303091	2.3221061	2.3900566	NM_001565	chr4:76942993-76942934	CXCL10
A_33_P3334448	5.6312113	5.5741463	NR_002324	chr3:39452639-39452698	SNORA62
A_24_P126682	6.3279767	6.296055	NM_022877	chr5:69362947-69363165	SMN2
A_23_P131683	4.097742	3.6497352	NM_012326	chr2:27248794-27248853	MAPRE3
A_33_P3721983	15.168701	15.140327	BC001183	chr11:118304175-118304116	MGC13053
A_33_P3261505	7.4128137	7.2565556	NM_020964	chr18:43427781-43427722	EPG5
A_23_P51646	7.6992087	7.0902624	NM_004073	chr1:45271594-45271654	PLK3
A_33_P3421108	6.0843973	5.9213023		chr4:158558926-158558984	
A_33_P3247372	6.176173	6.3457665	AK124064	chr22:17121568-17121627	TPTEP1
A_33_P3361831	2.3221061	2.3900566	NM_001124759	chr3:75715907-75715966	FRG2C
A_24_P269779	6.6945515	6.361704	NM_004273	chr10:73772610-73772669	CHST3
A_23_P338479	2.3221061	2.3900566	NM_014143	chr9:5468310-5468369	CD274
A_23_P250164	10.910885	10.558752	NM_000187	chr3:120347262-120347203	HGD
A_23_P371865	4.037236	4.064199	NM_152342	chr16:80637874-80637815	CDYL2
A_33_P3233459	3.5866108	3.8165815		chr3:64064096-64064037	LINC00994
A_33_P3301410	9.707726	9.496876	NM_019037	chr8:145135432-145135491	EXOSC4
A_33_P3256793	9.22694	8.890244	NM_020775	chr1:109745792-109745851	KIAA1324
A_33_P3275835	9.386511	9.658663	NM_001134430	chr9:130496638-130496579	TOR2A
A_23_P362183	10.559628	10.296388	NM_173551	chr9:101494363-101494304	ANKS6
A_23_P348281	5.5954523	5.77969	NM_153035	chr1:54564868-54564927	TCEANC2
A_33_P3236082	10.056668	10.026819	NM_001129765	chrX:152037847-152037906	NSDHL
A_24_P311604	6.626774	6.968915	NM_145048	chr4:20715063-20715122	PACRGL
A_33_P3247678	9.656092	9.491617		chr17:000843546-000843487	
A_33_P3349466	3.6548262	3.3730016	NM_001204056	chr18:9284825-9284884	ANKRD12
A_24_P337000	8.208369	8.148584	NM_020458	chr2:47303207-47303266	TTC7A
A_23_P83351	4.5235167	4.868609	NM_015404	chr9:117166194-117165579	DFNB31
A_23_P161686	5.5923405	5.630767	NM_014715	chr11:128838460-128838401	ARHGAP32
A_33_P3394489	9.411673	9.360671		chr22:51022107-51022166	CHKB-AS1
A_23_P403284	8.125544	7.982261	NM_014562	chr2:63284189-63284248	OTX1
A_33_P3356245	4.332615	4.7040234	NM_017879	chr19:58083644-58083585	ZNF416
A_33_P3359984	13.12387	13.219345		chr5:079796758-079796817	XLOC_014512
A_33_P3362296	2.6683445	3.4218853	BC006367	chr20:49625278-49625219	KCNG1
A_33_P3210875	7.4968834	6.7938485	NM_000052	chrX:77302314-77302373	ATP7A
A_24_P228667	8.502469	8.3157	NM_003776	chr22:19422372-19423174	MRPL40

A_33_P3268318	5.7252607	5.6637607	XM_005248144	chr4:17875071-17875012	LCORL
A_33_P3319596	9.209742	8.998333	NM_001283106	chr22:20053388-20053447	TANGO2
A_33_P3292814	2.846241	2.3900566	CR747917	chr10:2540924-2540865	
A_33_P3379644	4.130497	2.3900566	NM_000689	chr9:75540460-75540401	ALDH1A1
A_23_P364437	3.923818	3.2217562	NM_022124	chr10:73575277-73575336	CDH23
A_24_P296254	6.6015377	6.453229	NM_014783	chr15:32931232-32931291	ARHGAP11A
A_33_P3399107	5.2691207	4.5290084	NM_058004	chr22:21067054-21066995	PI4KA
A_24_P173746	7.504654	7.2745175	NM_152663	chr1:178866870-178871278	RALGPS2
A_33_P3393836	7.144097	7.3430705	NM_001166118	chr7:33057111-33057052	NT5C3A
A_33_P3306948	8.67049	8.389629	NM_002336	chr12:12269123-12269064	LRP6
A_23_P43846	2.9909055	3.0976315	NM_001040442	chr5:159659238-159661843	FABP6
A_24_P928969	8.721946	8.909562	NM_001145369	chr9:112138132-112138073	PTPN3
A_33_P3361152	5.155482	4.42808	NR_024452	chr2:47086086-47086145	LINC01119
A_24_P194714	10.091102	10.707029	NM_182565	chr17:74267309-74267368	UBALD2
A_24_P329600	9.822338	9.607912	NM_013438	chr9:86275876-86275817	UBQLN1
A_33_P3289167	4.304839	4.421817	NM_014383	chr19:36206667-36206726	ZBTB32
A_33_P3367087	5.461674	5.3577065	NM_212554	chr10:126447545-126447486	METTL10
A_33_P3415744	8.146942	8.16066	NM_025190	chr2:98121431-98121372	ANKRD36B
A_23_P44684	9.76734	10.175759	NM_018098	chr3:172538330-172538389	ECT2
A_33_P3351566	3.8877559	3.1664367	NM_001039481	chr12:22797290-22797349	ETNK1
A_24_P290502	7.287184	7.125003	NM_181453	chr2:109087469-109087528	GCC2
A_33_P3278906	5.958214	5.33547	NM_198513	chr8:133824058-133824117	PHF20L1
A_32_P163169	7.826234	8.504196	NM_003374	chr5:133311702-133311643	VDAC1
A_33_P3278649	3.324375	4.00987	NM_203468	chr9:139942612-139942553	ENTPD2
A_24_P28619	8.628889	8.531525	NM_144567	chr1:213165809-213165750	ANGEL2
A_23_P27048	8.894363	8.95778	NM_152464	chr17:26688917-26688976	TMEM199
A_33_P3223082	8.288845	6.9398465	NM_014763	chr2:75889260-75889319	MRPL19
A_33_P3239332	12.424421	12.858977	NR_046454	chr18:40113244-40113303	LINC00907
A_23_P135465	6.1830826	5.934448	NM_173811	chr11:46625050-46624991	HARBI1
A_23_P368681	2.3221061	2.3900566	NM_015660	chr7:150389949-150390008	GIMAP2
A_24_P348806	5.999323	5.8899755	NM_175058	chr11:16809290-16809231	PLEKHA7
A_33_P3326553	9.853637	10.454755		chr1:16074418-16074477	TMEM82
A_23_P130169	8.28485	8.652025	NM_014726	chr17:45788549-45788608	TBKBP1
A_33_P3232945	3.9393039	2.9397402	NM_005242	chr5:76130985-76131044	F2RL1
A_23_P147729	8.458701	8.461107	NM_018656	chr12:69158570-69158629	SLC35E3
A_33_P3570223	14.993443	15.018649	BX648289	chr1:27652963-27653022	LOC644961
A_33_P3417437	6.8129225	6.9649267	NM_207397	chr1:27705732-27705673	CD164L2
A_24_P1919	4.3974323	4.2533145	NM_000267	chr17:29541564-29546042	NF1
A_33_P3807593	6.6622534	6.654912	NR_039980	chrX:103367139-103367080	LOC286437
A_33_P3238978	7.5034723	6.336548	NM_139214	chrY:3447857-3447916	TGIF2LY
A_33_P3441576	9.339448	9.45075	NM_001271872	chr1:121130846-121130905	SRGAP2C
A_33_P3363674	7.057898	7.215705	NM_001142590	chr1:41237079-41237138	NFYC
A_23_P50349	7.3546767	7.3366184	NM_004240	chr19:6750570-6750629	TRIP10
A_33_P3381870	8.825143	8.738514	NM_001978	chr8:21939976-21940035	DMTN
A_33_P3283122	5.427053	4.2623096	NM_024949	chr4:184241798-184241857	WWC2
A_23_P5415	9.256844	9.1382885	NM_021824	chr2:201768294-201768353	NIF3L1
A_23_P374082	8.643824	9.241953	NM_033274	chr5:156904534-156904475	ADAM19
A_24_P367602	5.832404	5.885413	NR_002834	chr1:228788013-228788072	DUSP5P1
A_24_P98411	8.536585	7.900362	NM_005347	chr9:127997208-127997149	HSPA5
A_24_P324640	7.839183	7.74392	NM_014480	chr19:58757771-58758113	ZNF544
A_33_P3404889	4.7747927	4.7173386	XM_005270666	chr1:82452653-82452712	LPHN2
A_23_P215265	8.200175	7.7613153	NM_007353	chr7:2769904-2769845	GNA12
A_24_P381604	11.281217	11.275572	NM_021999	chr13:48835913-48835972	ITM2B
A_33_P3414113	6.203273	6.1699114		chr1:46911227-46911286	LOC729041
A_24_P37264	5.5290565	5.557842	NM_014746	chr2:7183304-7183363	RNF144A
A_23_P32861	8.314002	7.3522797	NM_015938	chr3:160968755-160968814	NMD3
A_23_P128375	6.394829	6.401948	NM_032829	chr12:110208051-110208110	FAM222A
A_23_P19590	9.799498	9.580386	NM_003379	chr6:159187729-159187670	EZR
A_23_P332042	4.2028575	4.0709968	NM_004259	chr17:73623320-73623261	RECQL5

A_33_P3355071	5.3194976	5.573964	NM_004641	chr10:22031639-22031698	MLLT10
A_23_P259442	9.013121	9.097973	NM_001873	chr4:166419232-166419291	CPE
A_23_P140994	7.1322618	7.2124596	NM_007242	chr16:70363824-70363883	DDX19B
A_23_P145035	4.3250723	3.39548	NM_153042	chr6:18223320-18223380	KDM1B
A_33_P3419910	5.0500007	5.076062		chr7:030537281-030537222	
A_23_P313961	7.701232	7.974762	NM_005793	chr3:48336122-48336063	NME6
A_24_P268196	6.474434	6.419443	XM_005263508	chr1:9989523-9989464	LZIC
A_24_P277349	11.076153	11.279459	NM_001077207	chr4:83740388-83740329	SEC31A
A_24_P76995	3.4804	3.284977	NM_015336	chr12:77199202-77202864	ZDHHC17
A_23_P58337	9.717627	9.425696	NM_030917	chr4:54325595-54325654	FIP1L1
A_23_P157963	5.391179	5.8506455	NM_017738	chr9:17503067-17503126	CNTLN
A_24_P375819	5.123822	5.5133514	NR_002774	chr12:13155047-13155106	HTR7P1
A_23_P102876	11.736568	11.129029	NM_006585	chr21:30433728-30433669	CCT8
A_33_P3409944	4.7221203	4.4321847	NM_005272	chr1:110145969-110145910	GNAT2
A_23_P60499	9.109683	8.901023	NM_021224	chr9:109773650-109773709	ZNF462
A_23_P17663	4.9747534	4.834055	NM_002462	chr21:42831014-42831073	MX1
A_33_P3217495	4.311078	3.9567325	NM_003041	chr16:31502023-31502082	SLC5A2
A_24_P68079	6.063142	6.4673595	NM_014831	chr3:36868467-36868408	TRANK1
A_33_P3789693	6.366994	5.398681	BC020879	chr9:16526545-16526486	MGC24103
A_24_P253723	6.627205	6.8400455	NR_028502	chr17:1615641-1615582	MIR22HG
A_24_P312189	5.789916	5.6084785	NM_019606	chr7:100030741-100030925	MEPCE
A_33_P3282181	10.018127	10.322798	NM_001164741	chrX:153173028-153172969	ARHGAP4
A_32_P57728	7.744946	7.8236856	NR_003613	chr7:99918653-99918594	PMS2P1
A_24_P300777	7.208109	6.856904	NM_001109	chr10:135076051-135075992	ADAM8
A_23_P426809	6.066741	5.7756615	NM_198236	chr1:156905226-156905167	ARHGEF11
A_32_P150876	3.4681578	4.0480585	NR_015389	chr18:5245815-5245874	LINC00667
A_33_P3259902	9.057875	9.447555		chr6:002341721-002341780	
A_33_P3387300	10.722156	10.871487	NM_001013438	chr3:180688091-180693910	FXR1
A_23_P5654	4.367496	3.5012093	NM_014439	chr2:113676249-113676308	IL37
A_33_P3213822	5.5218563	5.700608	NM_001017424	chr1:215410352-215410411	KCNK2
A_23_P155890	8.945455	8.801311	NM_032693	chr4:80246700-80246641	NAA11
A_23_P149818	6.94258	7.4396563	NM_080599	chr10:11962903-11962844	UPF2
A_23_P166023	9.789708	9.466926	NM_002623	chr20:52831885-52831944	PFDN4
A_23_P4922	8.392894	8.209644	NM_199341	chr19:48700803-48700862	C19orf68
A_23_P64898	5.2355075	5.148025	NM_005810	chr12:9162594-9162653	KLRG1
A_24_P148151	8.765392	8.350047	NM_005999	chr1:231701692-231701751	TSNAX
A_24_P356338	9.297856	9.526562	NM_007285	chr16:75611184-75611243	GABARAPL2
A_33_P3295705	4.600546	4.338269		chr10:001147835-001147776	
A_24_P97931	10.372008	10.5680275	NM_001320	chr6:31637268-31637654	CSNK2B
A_23_P52986	12.002573	12.252043	NM_152718	chr11:61025840-61025781	VWCE
A_24_P365901	7.125857	7.0820775	NM_178562	chr7:128808242-128808301	TSPAN33
A_33_P3310164	8.309152	8.081785	NM_004080	chr7:14188002-14187943	DGKB
A_33_P3408222	3.1431913	2.3900566		chr1:022394850-022394791	
A_23_P214080	5.983026	6.3395624	NM_001964	chr5:137804891-137804950	EGR1
A_33_P3397418	9.373037	9.44701	NM_020119	chr7:138728410-138728351	ZC3HAV1
A_33_P3213259	7.0922	7.4755106		chr19:59094064-59094123	CENPBD1P1
A_33_P3320443	5.5323334	5.6201043	NM_001256299	chr17:43913133-43913192	CRHR1
A_23_P200741	6.385237	6.662472	NM_001937	chr1:168664807-168664748	DPT
A_33_P3339865	3.647695	3.6397746	NM_017422	chr10:5540719-5540660	CALML5
A_33_P3372924	4.575678	5.004606	XM_005273202	chr1:227087089-227087148	ADCK3
A_33_P3239787	4.431224	4.4791803	NM_014699	chr16:31089514-31089573	ZNF646
A_33_P3221034	3.6337686	3.7991781		chr19:57168555-57168614	SMIM17
A_33_P3282390	3.9899778	4.233395	NM_000841	chr6:33989744-33989685	GRM4
A_33_P3279629	7.660906	6.8310814	NM_033199	chr3:48599234-48599175	UCN2
A_23_P371861	3.2011797	3.4800892	NM_173538	chr8:88298829-88298888	CNBD1
A_33_P3377261	9.602171	9.837172	NM_014245	chr3:141462378-141464039	RNF7
A_24_P202139	5.30995	4.3737993	NM_016025	chr16:21668015-21668074	METTL9
A_24_P915371	12.23941	12.214335	NM_001163424	chr2:241070356-241070297	MYEOV2
A_33_P3300297	4.7659097	4.717512		chr7:023017842-023017783	

A_23_P35791	8.024301	7.6959944	NM_003942	chr11:64139354-64139413	RPS6KA4
A_33_P3242863	4.011421	2.3900566	NM_020201	chr17:17250916-17250975	NT5M
A_23_P395566	7.422745	7.473709	NM_024735	chr16:87363104-87363045	FBXO31
A_24_P391104	8.600989	8.606982	NM_002918	chr19:14072614-14072555	RFX1
A_23_P139486	10.828398	11.455929	NM_004642	chr12:123745880-123745821	CDK2AP1
A_33_P3349591	5.600889	5.3212285		chr6:5043050-5043109	LOC100129461
A_23_P373598	5.5253	5.692933	NM_002360	chr7:1582229-1582288	MAFK
A_33_P3359543	7.841826	7.815302	NM_152289	chr19:9718062-9718003	ZNF561
A_33_P3420259	8.661538	9.065522	NR_003287	chrUn_gl000220:118024-118082	RNA28S5
A_33_P3316800	3.655645	3.855227	NM_001621	chr7:17382763-17382822	AHR
A_24_P342807	6.9586844	7.2595544	NM_017964	chr2:32446707-32446766	SLC30A6
A_24_P11965	7.8217106	8.214586	NM_033296	chr4:6642733-6642792	MRFAP1
A_23_P201022	4.0082555	3.6717439	NM_000298	chr1:155260153-155260094	PKLR
A_33_P3278043	6.0774746	5.912217	NM_152292	chr4:100469831-100469772	TRMT10A
A_33_P3340613	3.6078444	4.200978	NR_026759	chr9:42470679-42470738	FAM95B1
A_33_P3396275	4.4877853	5.05112	NM_001130448	chr15:41063123-41063182	C15orf62
A_23_P301995	6.092777	5.9579887	NM_173083	chr1:226453937-226453300	LIN9
A_23_P14464	6.529649	6.9493933	NM_006544	chr14:57675270-57675211	EXOC5
A_23_P4754	10.819522	10.757148	NM_006012	chr19:6368801-6368860	CLPP
A_33_P3238543	5.5024195	5.576347		chr17:79888548-79888607	MAFG-AS1
A_33_P3358521	4.8096094	4.957858	NM_020829	chr9:5774230-5774289	KIAA1432
A_24_P310224	2.4971635	2.3900566	NM_032584	chr19:53643774-53643715	ZNF347
A_24_P182539	6.9976983	6.764057	NM_032852	chr1:63330508-63330567	ATG4C
A_24_P392022	7.7986593	7.775658	NM_201400	chr16:5135614-5135555	FAM86A
A_23_P52738	9.316243	9.158937	NM_014026	chr11:126215496-126215555	DCPS
A_32_P117354	7.38148	7.1477785	NM_014988	chr4:41700725-41700784	LIMCH1
A_33_P3253249	15.430389	15.592702	NM_002032	chr11:61732130-61732071	FTH1
A_23_P310931	4.134898	4.3521595	NM_001841	chr1:24200579-24200520	CNR2
A_24_P200023	5.3168516	5.600889	NM_000877	chr2:102795394-102795453	IL1R1
A_24_P402415	5.878341	5.8828206	NM_153262	chr1:210335095-210335154	SYT14
A_33_P3355503	2.3811224	3.1814582	NM_005250	chr16:86615242-86615301	FOXL1
A_23_P425332	2.3221061	2.9298983	NM_058237	chr14:94745813-94745872	PPP4R4
A_24_P99071	9.617315	9.76096	NM_002271	chr13:98671921-98671980	IPO5
A_23_P68942	14.618314	14.755845	NM_000967	chr22:39710722-39710186	RPL3
A_33_P3286616	6.126588	5.6293697	NM_001569	chrX:153278082-153278023	IRAK1
A_24_P932418	8.426136	7.969709	NM_012305	chr11:1012176-1012235	AP2A2
A_33_P3243652	5.7865705	5.890147	AK129685	chr19:13950921-13950862	LOC100131831
A_33_P3277970	5.0875816	5.139589	XM_003960330	chrUn_gl000222:83481-83455	LOC100996350
A_23_P30020	7.513136	7.312767	NM_030821	chr4:110635616-110635557	PLA2G12A
A_33_P3261182	7.783066	7.6073065	NM_001099271	chr5:74970154-74970095	POC5
A_32_P21255	3.9472055	2.3900566	NM_013309	chr15:45775354-45775295	SLC30A4
A_23_P43566	12.455055	12.268318	NM_014222	chr9:124906467-124906408	NDUFA8
A_23_P160968	7.0647035	6.8151617	NM_018891	chr1:183209301-183209453	LAMC2
A_33_P3381647	5.5822864	5.250826	NM_001626	chr19:40736283-40736224	AKT2
A_23_P312718	7.36116	6.9454613	NM_015723	chr7:108119645-108113078	PNPLA8
A_33_P3351934	12.580866	12.591557	BC070067	chr1:155721303-155721362	MSTO1
A_32_P43826	3.9745135	2.3900566		chr10:42830073-42830014	LOC441666
A_33_P3401252	11.950979	12.222897	AY956769	chr1:92109276-92109335	HSP90B3P
A_23_P314726	8.376837	8.44607	NM_002651	chr1:151264990-151264931	PI4KB
A_33_P3489222	4.4471703	4.632411	NM_001042357	chr10:48738632-48738573	PTPN20B
A_33_P3402495	7.596129	7.693325		chrUn_gl000221:000042001-000041942	
A_33_P3369567	8.118879	7.9721475	NM_001242932	chr11:1908485-1908544	LSP1
A_23_P49082	6.2605047	6.1677475	NM_012225	chr16:1837977-1838036	NUBP2
A_24_P743869	6.888995	7.28298	NM_001195304	chr10:112659070-112659011	BBIP1
A_33_P3279841	10.251202	10.372511	NM_015133	chr16:1820258-1820317	MAPK8IP3
A_23_P28090	7.4988337	7.2782273	NM_003765	chr19:13259883-13256191	STX10
A_23_P408768	6.516247	6.5278416	NM_032482	chr19:2227105-2229821	DOT1L

A_23_P406135	8.004091	8.024143	NM_015662	chr2:27667316-27667257	IFT172
A_33_P3252785	4.6238832	4.7071843		chr10:81904761-81904820	PLAC9
A_33_P3384871	10.224681	10.166229	NM_001142522	chr6:153291957-153291898	FBXO5
A_23_P140423	11.346262	11.487306	NM_004545	chr14:92583912-92583853	NDUFB1
A_23_P34578	11.006588	10.92543	NM_013285	chr1:38034489-38033937	GNL2
A_23_P126266	4.0612245	3.6430433	NM_021958	chr1:221055600-221055659	HLX
A_24_P942002	7.104932	6.9614034	NM_012287	chr3:194996507-194996448	ACAP2
A_23_P334845	4.613765	4.56319	NM_032900	chr10:98985783-98985724	ARHGAP19
A_23_P44244	8.166409	7.566589	NM_003069	chrX:128580725-128580666	SMARCA1
A_23_P30264	5.829858	5.7216115	NM_016338	chr5:61897649-61923013	IPO11
A_23_P328836	7.4052057	7.642805	NM_032440	chr10:98718006-98718065	LCOR
A_33_P3412488	3.443756	3.7612023		chr2:38634009-38633950	
A_23_P151150	9.618504	9.708796	NM_202002	chr12:2967381-2967322	FOXM1
A_33_P3402171	8.445577	8.118	NM_004094	chr14:67853161-67853220	EIF2S1
A_23_P5568	5.6745505	5.657242	NM_032740	chr2:128460675-128460734	SFT2D3
A_23_P88095	6.6225343	6.663813	NM_014832	chr13:75859673-75859614	TBC1D4
A_24_P393958	7.477811	7.681591	NM_007034	chr1:78482033-78482092	DNAJB4
A_24_P127564	5.150092	5.107503	NR_024330	chr19:52206489-52206548	SPACA6P
A_33_P3244931	13.357744	13.498016	NM_001079863	chr2:120129987-120130046	DBI
A_32_P162150	7.645057	7.5619473	NM_152787	chrX:30846272-30846213	TAB3
A_24_P268123	4.31917	4.0943623	NM_174963	chr1:44395824-44395883	ST3GAL3
A_24_P58331	5.341913	5.424179	NM_024819	chr17:43111629-43111570	DCAKD
A_24_P47988	4.5862427	4.5154657	NM_025165	chr15:44065408-44065349	ELL3
A_33_P3302070	8.561877	8.894696		chr22:040965909-040965968	
A_33_P3381899	5.1673365	4.885882	NM_004209	chr16:2043497-2043556	SYNGR3
A_33_P3252141	9.82332	8.939018	NM_019022	chr18:66341020-66340961	TMX3
A_32_P1173	7.6144605	7.308793	NM_138441	chr6:74138526-74138467	MB21D1
A_24_P409857	12.842008	13.013338	NM_004965	chr21:40714707-40714648	HMGN1
A_33_P3351524	5.250227	5.6510477		chr4:009588552-009588493	
A_23_P1343	6.8862453	7.006458	NM_003473	chr10:17757003-17757062	STAM
A_24_P410587	9.062397	9.143881	NR_026903	chr17:62964429-62964370	AMZ2P1
A_24_P500891	9.901192	9.989338	NM_013411	chr1:33475717-33475658	AK2
A_33_P3309206	3.8510103	3.193936	NR_103801	chr15:26870038-26869979	GABRB3
A_23_P7697	10.968346	11.246752	NM_003100	chr5:122163341-122165351	SNX2
A_33_P3392952	8.923914	8.454624	NM_004514	chr17:80562366-80562425	FOXK2
A_23_P61531	8.954116	9.412224	NM_000404	chr3:33038463-33038404	GLB1
A_24_P325146	4.9162817	2.3900566	NM_031960	chr17:39253472-39253413	KRTAP4-8
A_33_P3812038	6.678899	6.3814273	AK074459	chr7:102168451-102168392	LOC340335
A_23_P148984	9.464689	9.97642	NM_018122	chr1:173827125-173827184	DARS2
A_33_P3415962	4.2219644	3.1522424	AK130540	chr6:89676329-89676388	
A_33_P3378925	8.391472	7.7545404	NM_006328	chr11:66394290-66394349	RBM14
A_33_P3301266	5.3586593	5.106386	NM_001159708	chr5:180374681-180374740	BTNL8
A_33_P3413597	11.880163	11.781786	NM_001257102	chr2:201950378-201950437	NDUFB3
A_32_P37360	6.209281	6.033798	NM_145315	chr6:108843534-108843593	LACE1
A_33_P3324675	6.1675234	6.303502		chr19:000298701-000298642	
A_23_P20683	10.276246	9.86145	NM_014878	chr9:2804293-2804234	KIAA0020
A_23_P94703	9.027351	9.242757	NM_014506	chr9:132573112-132573171	TOR1B
A_33_P3258223	5.007228	4.6826572	NM_005916	chr7:99697336-99697277	MCM7
A_33_P3316913	4.3760457	4.5100217		chr14:103746063-103746122	
A_24_P384636	9.390914	9.436007	NM_024038	chr19:12841855-12841796	C19orf43
A_33_P3296707	6.119978	6.039513	NM_001078173	chrX:134154613-134154554	FAM127C
A_33_P3209096	8.974696	9.032493	NM_001779	chr1:117078647-117078588	CD58
A_23_P331908	7.832339	7.7290645	XM_003959932	chr11:45236216-45236275	LOC101060179
A_33_P3326235	4.3262863	4.289626	NM_001003938	chr16:216693-216752	HBM
A_33_P3270636	11.087683	11.316116	NM_001272068	chr3:48511187-48511128	SHISA5
A_32_P44568	11.163998	12.004002	NM_005566	chr9:014921876-014921817	LDHA
A_32_P163247	3.8722234	3.6778498	NM_001768	chr2:87012000-87011941	CD8A
A_33_P3297621	6.9791036	7.2400556	NM_015047	chr1:19545856-19545797	EMC1
A_23_P370544	5.5527735	5.9827003	NM_144708	chr2:190611303-190611362	ANKAR

A_33_P3546070	7.977173	7.9988437	NM_016018	chr8:133859020-133859080	PHF20L1
A_23_P207507	7.6830893	7.170895	NM_003786	chr17:48768701-48768760	ABCC3
A_33_P3234593	4.863826	4.779035		chr16:50383508-50383449	BRD7
A_33_P3394868	6.465493	7.275964	NM_001163724	chr1:3692432-3692491	SMIM1
A_23_P201647	6.8698254	6.7232094	NM_173156	chr1:183522975-183523034	SMG7
A_24_P171182	7.5176635	7.795604	NM_022735	chr1:226333332-226333273	ACBD3
A_24_P122137	8.276808	7.8642025	NM_002309	chr22:30636772-30636713	LIF
A_23_P83453	7.7783127	7.9879904	NM_003074	chr3:47628282-47628223	SMARCC1
A_23_P164148	7.684674	8.049463	NM_170607	chr17:40724283-40724342	MLX
A_33_P3423270	7.2595544	7.223214	NM_001284406	chr3:12775584-12775525	TMEM40
A_23_P99967	8.531525	8.760151	NM_033028	chr15:73030203-73030262	BBS4
A_33_P3370875	4.252518	4.8910627		chr19:040529226-040529167	
A_33_P3299416	6.410998	6.625506	NM_181711	chr12:52409589-52409648	GRASP
A_23_P89902	4.094048	4.476694	NM_005619	chr19:45988845-45988786	RTN2
A_33_P3381245	12.130803	12.0849	NM_004708	chr19:33078221-33078280	PDCD5
A_33_P3348204	4.088974	4.7765884	NM_153027	chr4:165878562-165878621	FAM218A
A_33_P3691615	4.9477825	4.9276757	AK094945	chr4:178598147-178598206	
A_24_P325107	6.0238853	6.4320083	NM_020940	chr10:116615070-116620537	FAM160B1
A_33_P3415698	6.069779	6.1218953	NM_144615	chr19:4292296-4292237	TMIGD2
A_33_P3279456	14.2308	14.362864		chr2:042077385-042077326	
A_23_P47125	3.6111166	3.3555772	NM_153444	chr11:7817922-7817863	OR5P2
A_24_P272225	6.813148	6.699718	NR_027023	chr1:155533617-155533676	ASH1L-AS1
A_33_P3222424	2.6342068	2.3900566	NM_000759	chr17:38173251-38173310	CSF3
A_23_P346884	3.8735876	3.737448	NM_014276	chr20:43942760-43943087	RBPJL
A_33_P3269636	2.3221061	2.3900566	NM_001166034	chr19:36014331-36014272	SBSN
A_33_P3382351	12.22564	12.211215	NM_001004333	chr17:6917791-6917850	RNASEK
A_33_P3401428	7.952551	8.097656	NM_018112	chr9:108510409-108510468	TMEM38B
A_23_P166306	13.284451	13.128433	NM_000071	chr21:44473378-44473319	CBS
A_24_P399630	3.9131722	4.03307	NM_002730	chr19:14203200-14203141	PRKACA
A_33_P3228988	15.218346	15.311133	XM_005260176	chr19:12490563-12490504	
A_23_P168019	5.558278	5.402022	NM_024493	chr6:28333970-28334029	ZKSCAN3
A_23_P57199	5.959612	6.4269557	NM_178311	chr20:23965799-23965740	GGTLC1
A_24_P370156	5.4047327	5.580777	NM_000528	chr19:12760201-12760018	MAN2B1
A_23_P38482	9.357062	9.484858	NM_024297	chr17:7138846-7138787	PHF23
A_23_P134835	3.640665	4.807321	NM_018371	chr8:19262014-19261955	CSGALNACT1
A_23_P159663	9.835789	9.900345	NM_004182	chrX:47516977-47516637	UXT
A_23_P394986	3.6298978	2.3900566	NM_153836	chr2:101965507-101965449	CREG2
A_23_P365614	4.3792	3.7865293	NM_004557	chr6:32162683-32162624	NOTCH4
A_33_P3370404	8.567611	8.5568285	NM_015368	chr11:93915057-93915116	PANX1
A_23_P63209	2.504584	2.3900566	NM_181755	chr1:209907901-209907960	HSD11B1
A_32_P58407	6.9218397	7.2599726	AF070632	chr1:112313618-112313559	KCND3
A_24_P233570	13.174683	13.203878	NM_002793	chr6:170844312-170844253	PSMB1
A_23_P433990	8.299065	8.245282	NM_003119	chr16:89623618-89623677	SPG7
A_33_P3300837	4.2082415	4.216275	NM_001290	chr4:16510299-16510240	LDB2
A_33_P3302657	4.174472	4.2004237		chr9:82189883-82189942	TLE4
A_33_P3212037	5.8915515	6.0171595	AB012143	chr6:89388060-89388001	RNGTT
A_33_P3222753	4.9410706	4.8430343		chr19:53103310-53103369	ZNF137P
A_23_P51039	4.399597	4.446686	NM_002191	chr2:220440173-220440232	INHA
A_23_P354217	4.1449385	4.4895163	NM_153266	chr11:66063843-66063902	TMEM151A
A_33_P3390441	13.3379755	13.048807	NM_002265	chr17:45760899-45760958	KPNB1
A_23_P138541	9.151299	9.278196	NM_003739	chr10:5147789-5147848	AKR1C3
A_33_P3399443	6.329611	6.5799446	XM_005247975	chr4:8234161-8234220	SH3TC1
A_23_P115064	7.938525	7.8425827	NM_001878	chr1:156669638-156669579	CRABP2
A_23_P34093	5.9458184	5.5971766	NM_000402	chrX:153760297-153760238	G6PD
A_33_P3384108	7.048253	6.7826138	NM_194255	chr21:46935144-46935085	SLC19A1
A_24_P139208	7.5757	7.6776342	NM_013396	chr21:17251556-17251615	USP25
A_24_P938614	4.040558	2.3900566	NM_001263	chr4:85572301-85572360	CDS1
A_23_P90790	5.365452	5.0670686	NM_199227	chr2:172945519-172945578	METAP1D
A_23_P83634	3.744742	3.4329987	NM_001139	chr17:7978975-7978916	ALOX12B

A_33_P3226380	5.6799	5.472048	NR_029401	chr1:243220453-243220394	LOC731275
A_23_P97532	7.697406	7.713992	NM_004781	chr1:7837368-7838226	VAMP3
A_23_P87011	5.6736593	5.5198746	NM_001001522	chr11:117075155-117075214	TAGLN
A_23_P136325	6.7131405	6.680435	NM_133264	chr17:38434959-38435018	WIPF2
A_24_P109652	7.6538453	7.7148037	NM_024776	chr15:77400666-77400607	PEAK1
A_23_P2474	6.3148074	6.317543	NM_016319	chr12:6840330-6840389	COPS7A
A_33_P3598466	6.6439505	6.679421	AY956767	chr13:97536256-97536197	HSP90AB6P
A_23_P68851	8.100479	8.43094	NM_001039570	chr22:29542505-29542564	KREMEN1
A_23_P82412	9.205314	8.871297	NM_148956	chr7:72717638-72717509	NSUN5
A_33_P3256858	9.898943	9.99242	NM_001134875	chr14:105965492-105965551	C14orf80
A_23_P2423	10.494842	10.179343	NM_018048	chr12:10758734-10758675	MAGOHB
A_24_P57528	6.9958844	7.0758467	NM_139177	chr17:70642270-70642211	SLC39A11
A_23_P31844	7.5114427	7.6679115	NM_001693	chr8:20078395-20078454	ATP6V1B2
A_24_P317874	8.295721	8.099984	NM_012311	chr10:7801886-7798080	KIN
A_23_P34510	6.1765485	6.154981	NM_198040	chr1:33794554-33794495	PHC2
A_24_P332862	8.960358	9.196188	NM_001076678	chr19:21606725-21606774	ZNF493
A_33_P3407631	4.0649943	3.5971465		chr16:10131057-10131116	
A_33_P3243264	8.193386	7.8443165	NM_001080849	chr9:139257501-139257442	DNLZ
A_33_P3413353	3.7998126	4.222348		chrX:009380351-009380410	
A_33_P3321120	4.393046	4.4954753		chr2:233592754-233592695	
A_23_P83781	9.645909	9.68362	NM_004762	chr17:76670197-76670138	CYTH1
A_23_P342751	5.9225574	6.08483	XR_245427	chr13:64403089-64403030	
A_33_P3263867	2.3221061	2.3900566	NM_002562	chr12:121623474-121623533	P2RX7
A_23_P163258	8.178531	8.243563	NM_020214	chr15:72533855-72533796	PARP6
A_24_P256155	6.76178	6.9684563	NM_001146340	chr10:126136173-126136114	NKX1-2
A_33_P3230189	4.0730977	4.6581135	NM_032229	chr13:86369565-86369506	SLITRK6
A_33_P3229672	5.3141255	5.5327134		chr2:154277068-154277009	
A_33_P3419998	5.0782347	4.9401546	NM_001039960	chr12:51902921-51902980	SLC4A8
A_33_P3329108	9.043705	6.9390464	NM_002451	chr9:21865830-21865889	MTAP
A_33_P3237403	5.383485	5.385521	NM_173531	chr19:21906939-21906880	ZNF100
A_33_P3637909	8.46183	8.4106045	NM_015983	chr7:43995635-43995694	UBE2D4
A_33_P3424267	4.1012607	4.620007	NM_001083601	chr16:3533512-3533571	NAA60
A_33_P3255755	5.116659	5.5205965		chr1:16956730-16956671	CROCCP2
A_23_P86532	5.69204	6.379514	AK026129	chr10:60573780-60573839	BICC1
A_33_P3235204	5.6249933	5.682747	NM_032213	chr2:85617562-85617621	ELMOD3
A_32_P41292	12.705259	12.536509	NM_003375	chr10:76990947-76991006	VDAC2
A_33_P3346473	6.3941	6.660144	NM_001101802	chr11:45951004-45950945	PHF21A
A_33_P3253717	3.9975905	3.2495778		chr1:186274024-186273967	
A_32_P118372	5.46398	5.834753	NM_015693	chr4:128635137-128635196	INTU
A_24_P817863	6.593056	6.808381	NM_032504	chr2:210863469-210863528	UNC80
A_23_P168828	9.27916	9.573547	NM_005655	chr8:103661422-103661366	KLF10
A_33_P3322125	4.073944	3.423327	NM_001136269	chr12:51640424-51640483	DAZAP2
A_23_P99771	11.257545	11.751728	NM_006029	chr14:74178623-74178564	PNMA1
A_24_P916718	4.867213	4.532748	NM_207336	chr7:149463298-149463239	ZNF467
A_23_P202708	6.7326326	6.5841722	NM_003682	chr11:47350979-47351038	MADD
A_24_P117138	6.963524	7.060163	NM_145297	chr19:20829118-20828560	ZNF626
A_33_P3374210	9.814371	10.098356	NM_002417	chr10:129907639-129907580	MKI67
A_33_P3353365	4.153489	4.3504105	BC109207	chr6:25776995-25777054	SLC17A4
A_33_P3318267	5.7741623	5.802402	NR_024580	chr9:139698673-139698614	KIAA1984-AS1
A_23_P157072	10.987789	10.806917	NM_001037283	chr7:2420062-2420121	EIF3B
A_32_P25737	5.8899755	6.629459	NM_001039840	chrX:72905985-72906044	CHIC1
A_23_P171296	10.653233	10.585618	NM_002436	chrX:154007043-154006984	MPP1
A_32_P92783	7.5980215	7.3628874	NM_006819	chr11:63971080-63971570	STIP1
A_23_P98350	9.772176	10.839266	NM_001165	chr11:102208356-102208415	BIRC3
A_33_P3379512	4.8034315	4.6887693	NM_032433	chr19:14815878-14815937	ZNF333
A_33_P3274763	5.2353287	4.884814		chr13:114587281-114587340	
A_24_P192805	2.3221061	2.3900566	NM_001007232	chr11:104971256-104970106	CARD17
A_23_P1615	6.6001396	6.8170037	NM_004214	chr11:65655084-65653848	FIBP
A_23_P316812	5.8449264	6.0496507	NR_002796	chr1:113466600-113466659	AKR7A2P1

A_23_P104509	6.022605	6.135405	NM_014661	chr10:126370389-126370330	FAM53B
A_33_P3265301	4.2040477	4.342222	NM_152219	chr17:38517385-38517326	GJD3
A_23_P35399	11.410988	11.607504	NM_145869	chr10:81917416-81915629	ANXA11
A_23_P419947	6.0606813	6.340506	NM_022443	chr3:158315874-158315933	MLF1
A_33_P3262138	6.3023005	6.3821774	NM_022662	chr2:112540019-112539960	ANAPC1
A_23_P44964	8.235331	8.5130415	NM_001010924	chr10:15254148-15254089	FAM171A1
A_33_P3337540	7.8730693	8.0844345	NM_000874	chr21:34635002-34635061	IFNAR2
A_23_P200792	11.290114	11.335772	NM_024408	chr1:120454917-120454858	NOTCH2
A_23_P217068	15.318853	15.3302355	NM_000976	chr9:130210191-130209982	RPL12
A_23_P305507	9.197699	9.514012	NM_003286	chr20:39752205-39752264	TOP1
A_24_P43810	2.3221061	2.3900566	NM_207006	chr8:124206338-124219405	FAM83A
A_24_P300483	5.800666	6.127775	NM_024054	chr7:42949810-42949751	C7orf25
A_33_P3380587	5.7175436	5.602486	NM_012199	chr1:36386500-36386559	AGO1
A_33_P3232562	8.281347	8.243035	NM_005610	chr1:33145595-33145654	RBBP4
A_33_P3271065	4.3971415	4.31654	NM_001199527	chr21:43547842-43547901	UMODL1
A_23_P41314	3.4573207	3.6179109	NM_000128	chr4:187209847-187209906	F11
A_33_P3297050	7.134044	7.1765575	NM_033087	chr9:101978788-101978729	ALG2
A_23_P339079	5.4813147	5.5859156	NM_152360	chr19:38229314-38229255	ZNF573
A_33_P3221859	4.886935	4.8546762	NM_003030	chr3:157815892-157815833	SHOX2
A_23_P346291	5.0861244	5.1570354	NM_018969	chrX:53106775-53106834	GPR173
A_24_P213715	5.1665487	5.00513	NR_026993	chr22:50172731-50172672	LOC90834
A_24_P186065	6.209438	5.8716455	NM_176815	chr3:93777204-93777145	DHFRL1
A_32_P5251	9.303282	9.396	NM_001024809	chr17:38512928-38512987	RARA
A_23_P62270	8.587911	8.789648	NM_004187	chrX:53221665-53221606	KDM5C
A_33_P3329477	8.54358	8.575974	NR_002803	chr12:6993636-6993695	RPL13P5
A_32_P222684	5.46678	5.3918705	NM_001136239	chr5:122523200-122523259	PRDM6
A_33_P3243248	5.4499655	5.6874986	BU664634	chr20:47861201-47861142	ZNFX1
A_23_P202773	5.7321672	5.382003	NM_001039496	chr11:64072177-64072236	TEX40
A_23_P8664	8.369848	8.603433	NM_021145	chr7:86825190-86825249	DMTF1
A_23_P409623	5.235872	5.983026	NM_003621	chr11:7674673-7674732	PPFIBP2
A_33_P3242873	6.542277	6.6434855	NM_001270879	chr6:166778798-166778739	MPC1
A_23_P9582	10.757148	10.583321	NM_003321	chr16:28855134-28855075	TUFM
A_33_P3378047	6.979429	6.954383	NM_178123	chr2:179973718-179973659	SESTD1
A_23_P26325	4.4796762	4.354182	NM_002987	chr16:57449888-57449947	CCL17
A_23_P163458	8.694257	8.696297	NM_139265	chr15:42191933-42191874	EHD4
A_23_P65712	8.600434	8.783129	NM_031452	chr15:83659019-83659078	FAM103A1
A_33_P3311056	11.684499	11.649645	NM_004168	chr5:256725-256784	SDHA
A_23_P76731	7.6915407	7.9194107	NM_014226	chr14:102695348-102695289	MOK
A_24_P307572	5.1289444	5.195039	NM_033121	chr12:110456217-110456276	ANKRD13A
A_33_P3318963	3.6087756	3.5501974		chr22:38421716-38421775	XLOC_014237
A_24_P277807	11.484997	11.881024	NM_003795	chr6:108533440-108533381	SNX3
A_23_P338168	7.004964	7.2296505	NM_001099784	chr16:30959177-30959236	FBXL19
A_33_P3292919	8.122895	7.70335	NM_001135243	chr5:149779752-149779811	TCOF1
A_33_P3326822	3.6749473	2.9240675		chr7:30739747-30739806	INMT
A_23_P151159	4.98573	4.846062	NM_032300	chr12:110354637-110354696	TCHP
A_23_P366230	5.195039	4.95736	NM_004690	chr6:149982442-149982383	LATS1
A_24_P218006	6.541146	6.226766	NM_032317	chr7:73095367-73095308	DNAJC30
A_24_P142151	5.8570347	6.055647	NM_144598	chr15:99901706-99903359	LRRC28
A_23_P68892	9.68004	9.627621	NM_014306	chr22:32788228-32784029	RTCB
A_33_P3396877	6.4052444	6.1821165	NM_183005	chr10:15145654-15145713	RPP38
A_23_P45999	5.7956977	5.8163147	NM_012168	chr1:11708617-11708558	FBXO2
A_33_P3389023	5.661784	5.5501776	NM_024592	chr4:56237337-56237396	SRD5A3
A_23_P17811	4.5593176	4.4098597	NM_012429	chr22:30818809-30818868	SEC14L2
A_23_P74115	9.104533	9.024764	NM_003579	chr1:46744038-46744097	RAD54L
A_24_P28722	2.3221061	2.3900566	NM_080657	chr2:7037200-7037259	RSAD2
A_23_P202004	2.3221061	2.3900566	NM_020200	chr10:25138148-25138089	PRTFDC1
A_23_P134295	9.45075	9.503762	NM_198949	chr7:2290560-2290619	NUDT1
A_24_P211558	3.4944746	3.5834064	NM_032051	chr22:31740430-31740371	PATZ1
A_23_P372771	3.9334295	4.3718824	NM_020447	chr15:75193321-75193262	FAM219B

A_33_P3392000	6.809185	7.001685	NR_038955	chr9:132264707-132264766	LINC00963
A_33_P3309054	6.7996545	6.5859013	AB209400	chr20:33932049-33931990	
A_33_P3358163	5.12359	4.79203	NM_018958	chr15:24928526-24928585	NPAP1
A_33_P3315027	15.114832	15.149481		chr5:120911602-120911543	
A_33_P3353692	6.0011377	5.8281417	NM_002473	chr22:36722673-36722614	MYH9
A_23_P413815	8.475586	8.94492	NM_173517	chr7:65419484-65419543	VKORC1L1
A_33_P3539223	6.4000835	6.3059993	NM_032818	chr9:35660283-35660224	ARHGEF39
A_33_P3499174	11.199257	10.866096	NM_001265582	chr9:131152831-131152890	URM1
A_24_P188164	5.133809	4.71216	NM_052923	chr6:28539433-28539389	SCAND3
A_33_P3212575	7.0488687	6.741998	NM_005386	chr20:36151971-36152030	NNAT
A_24_P152968	3.815249	3.572587	NM_001353	chr10:5005636-5005695	AKR1C1
A_33_P3358403	9.353966	9.317855	NM_001080849	chr9:139256471-139256412	DNLZ
A_33_P3234849	5.056625	5.3588276	NM_007124	chr6:145157529-145157588	UTRN
A_24_P101561	9.904563	9.445157		chr7:104942264-104942325	
A_24_P194000	11.952536	11.815793	NM_005500	chr19:47713365-47713424	SAE1
A_32_P179837	5.8352833	5.7923627	NM_174903	chr16:2018872-2018931	RNF151
A_33_P3362068	6.824752	6.9108896		chr2:037899733-037899792	
A_33_P3230588	3.357898	3.3748026	DB321672	chr9:68743660-68743719	
A_23_P57364	3.9312317	4.212503	NM_005423	chr21:43767646-43766648	TFF2
A_24_P380132	11.270748	11.29068	NM_203505	chr4:76568305-76568246	G3BP2
A_24_P940803	8.556038	7.9606743	NM_033505	chr2:26618451-26618510	EPT1
A_23_P101111	5.6293697	5.771285	NM_004715	chr18:77514106-77514165	CTDP1
A_24_P378402	3.6926396	2.3900566	NM_152457	chr16:3486899-3486840	ZNF597
A_23_P142013	4.549224	4.955061	NM_020196	chr19:7687308-7687249	XAB2
A_33_P3353791	5.9078116	6.494276	NM_181501	chr5:52249249-52249308	ITGA1
A_33_P3233834	5.351407	6.148676	NM_001190981	chr5:55260096-55260037	IL6ST
A_33_P3217123	11.564201	11.55609	NM_138639	chr19:50177097-50177156	BCL2L12
A_24_P677634	10.878106	10.30715		chr7:66018872-66018813	LOC493754
A_24_P397489	2.455793	2.3900566	NM_001491	chr6:10628471-10628530	GCNT2
A_24_P272290	4.1867557	4.262717	NM_183373	chr6:3723444-3723385	PXDC1
A_33_P3335451	10.073771	10.012118	NM_018622	chr3:183547249-183547190	PARL
A_23_P54576	5.473748	5.529438	NM_005550	chr16:57794651-57794283	KIFC3
A_23_P435407	5.82586	5.886187	NM_001448	chrX:132435488-132435429	GPC4
A_33_P3297853	6.1131067	6.449467	NM_030767	chr9:117098466-117098407	AKNA
A_33_P3813818	4.0292597	3.8604987	AB088847	chr1:65765366-65765425	
A_23_P148821	8.704146	8.557522	NM_001077394	chr1:101455893-101455834	DPH5
A_33_P3260500	3.883936	3.5230787	NR_015445	chr8:144780518-144780577	BREA2
A_23_P207399	9.3381815	9.513282	NM_031858	chr17:41363202-41363261	NBR1
A_23_P432947	2.3221061	2.3900566	NM_013372	chr15:33026075-33026134	GREM1
A_32_P20367	13.661747	13.717503	NM_001011	chr2:3627728-3627787	RPS7
A_24_P105298	14.536229	14.519965	NM_002107	chr1:226259067-226259126	H3F3A
A_23_P18798	2.3221061	2.859664	NM_019119	chr5:140570085-140570144	PCDHB9
A_33_P3370515	10.629968	10.687778		chr17:000041347-000041406	
A_23_P39647	6.731408	6.6742096	NM_005070	chr2:220506385-220506444	SLC4A3
A_24_P649747	6.3765936	6.326145	NM_014753	chr10:43317522-43317581	BMS1
A_33_P3329740	10.540964	11.107742		chrX:040749868-040749809	
A_33_P3362034	3.9395123	3.7154899	NR_038365	chr10:133608124-133608065	FLJ46300
A_32_P25273	14.601013	14.172079	NM_002156	chr2:198351440-198351381	HSPD1
A_23_P405885	2.4517727	3.2062383	NM_138815	chr3:109012699-109012641	DPPA2
A_32_P345659	4.121715	4.4228616	NM_194293	chr3:39225622-39225563	XIRP1
A_33_P3330826	4.874205	4.319883		chr9:97122119-97122178	LOC100132077
A_24_P63522	7.974762	8.481533	NM_002130	chr5:43290176-43290117	HMGCS1
A_24_P202567	7.582761	7.58018	NM_025194	chr19:41246613-41246672	ITPKC
A_24_P97687	4.310104	4.12871	NM_000524	chr5:63256373-63256314	HTR1A
A_23_P500300	4.0864735	4.2324862	NM_033229	chr6:30140403-30140462	TRIM15
A_23_P19517	9.524292	9.143334	NM_002224	chr6:33663772-33663831	ITPR3
A_23_P35970	8.044216	7.863728	NM_001467	chr11:118895479-118895420	SLC37A4
A_33_P3422740	6.6703277	6.740485	NR_027002	chr1:149290979-149291038	LOC388692
A_24_P264063	4.7382298	4.909121	XR_110528	chr11:120041840-120041899	LOC729173

A_24_P889720	14.17963	14.359379	NM_021009	chr12:125396213-125396182	UBC
A_33_P3366124	9.466237	9.284735		chrX:53152865-53152924	
A_24_P136683	4.009615	4.237641	NR_026551	chrX:15721364-15721423	CA5BP1
A_33_P3263824	8.78706	8.862143	NM_176793	chr10:102746907-102746848	MRPL43
A_24_P926195	7.788958	7.56318	NM_006699	chr1:118067852-118067911	MAN1A2
A_32_P88635	8.859724	8.959106	NM_032264	chr1:21810710-21810769	NBPF3
A_24_P75157	3.4327376	2.3900566	NM_004610	chr6:167791460-167790151	TCP10
A_24_P627984	5.1225824	4.568883	NM_014805	chr3:37027665-37027606	EPM2AIP1
A_33_P3414883	4.582046	4.905185		chr3:195668860-195668919	ATMIN
A_24_P37540	4.571393	4.0324063	NM_001198793	chr3:9877427-9877486	ARPC4-TTLL3
A_23_P159893	6.9787235	7.0359144	NM_145234	chrX:109919246-109919187	CHRDL1
A_23_P81392	9.627621	9.528677	NM_015238	chr5:167896127-167896186	WWC1
A_24_P477051	7.011063	7.202219		chr19:034583600-034583541	
A_33_P3392192	8.526847	8.276395	AF176921	chr8:32625320-32625379	
A_23_P309361	5.0973554	4.9739523	NM_144584	chr1:109191030-109190971	HENMT1
A_33_P3386117	9.204564	8.987042	NM_007033	chr1:2336815-2336874	RER1
A_24_P331830	5.6841497	4.6557307	NM_015209	chr1:15361246-15361305	KAZN
A_33_P3243857	8.684528	9.082555	NM_001110	chr15:58889460-58889401	ADAM10
A_23_P365418	5.0304685	4.559161	NM_001195220	chr7:148981780-148981839	ZNF783
A_23_P165414	6.8574953	7.1038284	NM_144711	chr2:170605984-170606043	KLHL23
A_23_P46871	4.669965	4.7491884	NM_018344	chr10:73122622-73122681	SLC29A3
A_23_P203115	7.3056374	7.451602	NM_032780	chr11:118406474-118406533	TMEM25
A_23_P145895	9.118975	9.007405	NR_015381	chr7:86954730-86954671	TP53TG1
A_24_P236522	5.3256803	4.9942474	NM_001243646	chr16:30362760-30362701	CD2BP2
A_33_P3237927	3.946026	4.102263	NM_001164741	chrX:153172894-153172835	ARHGAP4
A_23_P165402	11.340458	11.19707	NM_016047	chr2:24291261-24291202	SF3B14
A_24_P225616	8.082573	7.357461	NM_001034	chr2:10270487-10270546	RRM2
A_33_P3304688	5.009774	5.005409	AF463496	chr13:40015577-40015518	TNAP
A_33_P3589543	6.4916134	7.02362	AK093839	chr11:560045-560104	LOC692247
A_24_P344961	6.490693	6.915638	NM_133265	chrX:112018573-112018514	AMOT
A_33_P3359753	9.241397	9.400412	NM_145257	chr1:229456873-229456814	CCSAP
A_24_P323815	8.757874	8.755757	NM_015057	chr13:77672355-77672296	MYCBP2
A_32_P62008	14.825712	14.818495	NM_007104	chr6:35438062-35438121	RPL10A
A_24_P354615	7.880416	7.9471188	NM_001040446	chr5:32227906-32227847	MTMR12
A_23_P22119	9.608115	9.310556	NM_201380	chr8:144989680-144989621	PLEC
A_23_P156620	6.5154157	6.42418	NM_007149	chr6:27419044-27418985	ZNF184
A_23_P51690	3.902061	4.146751	NM_020407	chr1:156354895-156354954	RHBG
A_23_P20752	6.384126	6.359786	NM_001039803	chr9:90581618-90581559	CDK20
A_24_P47681	8.902313	8.997559	NM_018448	chr12:67707497-67707556	CAND1
A_33_P3250028	7.053636	7.288043	NM_145245	chr19:7929802-7929861	EVI5L
A_33_P3413523	8.663937	8.693509	NM_006716	chr7:87516653-87516712	DBF4
A_24_P44596	9.065522	9.086329	NM_014940	chr16:77233447-77233506	MON1B
A_33_P3231472	5.7984986	5.526066	NM_014506	chr9:132566557-132566616	TOR1B
A_32_P188860	6.613103	6.721819	NM_017563	chr3:57124128-57124069	IL17RD
A_33_P3290672	9.497707	8.177618	NM_016275	chr3:150348174-150348233	SELT
A_33_P3216083	5.3022237	5.59987	NM_173082	chr6:146209221-146209162	SHPRH
A_23_P256542	10.856531	11.110586	NM_014367	chr3:122128730-122128789	FAM162A
A_33_P3261803	4.2875204	4.285987	NM_001441	chr1:46876109-46876168	FAAH
A_24_P15043	7.2550206	7.096572	NM_025010	chr3:47387924-47387983	KLHL18
A_33_P3233608	3.9499357	3.7302296	NM_145664	chrX:140097676-140097735	SPANXB2
A_24_P303193	5.0360875	5.043878	BG036557	chr5:137086624-137086565	
A_23_P57007	5.0410566	5.122322	AF090938	chr20:39665854-39665795	LOC100127886
A_23_P358221	6.7245264	7.0878	NM_015562	chr3:196081140-196081081	UBXN7
A_23_P26184	7.0192537	7.031374	NM_017996	chr15:89055857-89055798	DET1
A_23_P419764	4.7766724	4.6485853	AL834257	chr4:24829109-24829050	CCDC149
A_33_P3325275	7.5156765	7.527312	NM_024958	chr20:334382-334441	NRSN2
A_33_P3365810	10.062071	9.689893	NM_002949	chr17:79673989-79674048	MRPL12
A_23_P141362	7.9642587	8.102255	NM_001466	chr17:42636610-42636669	FZD2
A_33_P3318288	8.190326	8.893131	NM_001014975	chr1:196659246-196659305	CFH

A_23_P500614	4.7318797	4.5939517	NM_001243	chr1:12204161-12204220	TNFRSF8
A_33_P3405897	5.5030127	5.354823	NM_014798	chr17:43552526-43552467	PLEKHM1
A_33_P3361267	4.233818	4.6367464	XR_246457	chr11:100554974-100554915	
A_33_P3365878	13.788274	13.893914	NM_001720	chr1:40223962-40223903	BMP8B
A_23_P28869	7.870595	7.9187074	NM_002836	chr20:3018788-3018847	PTPRA
A_24_P942112	5.626514	4.88261	AK001678	chr2:113334680-113334739	POLR1B
A_24_P358146	4.7479596	4.080409		chr16:000281399-000281458	
A_23_P166910	8.095913	8.428337	NM_015268	chr3:132257167-132257226	DNAJC13
A_24_P126305	6.94847	6.9081197	NM_021646	chr16:4801207-4801148	ZNF500
A_33_P3238032	4.9287295	5.00903	NM_001042784	chr4:77305347-77305288	CCDC158
A_33_P3411372	6.5508084	6.7653294	NR_046369	chr11:118252156-118252097	LOC100131626
A_33_P3888365	7.3834114	7.32664	NM_018364	chr1:114308057-114307998	RSBN1
A_33_P3266958	4.342	3.8967292		chr17:018579643-018579584	
A_33_P3313411	7.875874	7.9409647	NM_001172630	chr19:36278925-36278984	ARHGAP33
A_23_P501547	5.9792643	5.720607	NM_015270	chr12:49160628-49160569	ADCY6
A_23_P15829	6.964199	6.4006577	NM_004618	chr17:18177689-18177630	TOP3A
A_23_P415882	11.380471	11.2388935	NM_001001563	chr19:39980532-39980591	TIMM50
A_23_P67278	6.434249	6.787031	NM_005815	chr19:12541400-12541341	ZNF443
A_24_P49260	6.9040623	7.493564	NM_018327	chr20:13147054-13147113	SPTLC3
A_33_P3248749	7.8764515	8.242495	AK055407	chr6:43640091-43640150	RSPH9
A_23_P49975	11.134429	10.997825	NM_000421	chr17:38974495-38974436	KRT10
A_33_P3312819	5.7792625	5.8620286	NR_028044	chr11:2168656-2168715	IGF2-AS
A_33_P3841755	8.276395	8.272485	AL137758	chr15:97304924-97304865	LOC145945
A_33_P3278664	4.8530793	5.673222	NM_001011515	chr4:95509308-95509367	PDLIM5
A_33_P3407780	11.979293	12.165957	NM_014634	chr22:22273853-22273794	PPM1F
A_24_P55496	3.916531	4.0011153	NM_053001	chr8:99961622-99961681	OSR2
A_33_P3270417	3.9478698	3.3405485		chr20:004732787-004732728	
A_23_P358995	10.232708	10.327096	NM_015525	chr6:82880099-82880040	IBTK
A_33_P3395647	6.5135016	6.8437605	AK125981	chr17:28904982-28905041	
A_33_P3346841	10.711566	10.6830435	NM_001173128	chr1:29070439-29070498	YTHDF2
A_32_P122402	5.77351	6.3790894	NR_026792	chr9:99489445-99489504	LOC441455
A_23_P150852	7.193535	7.324863	NM_015292	chr12:56537790-56537849	ESYT1
A_24_P186986	9.743299	9.47694		chr13:107315845-107315904	
A_23_P90220	5.365794	5.7293005	NM_152655	chr19:37642635-37642576	ZNF585A
A_23_P19210	8.920211	8.38307	NM_032194	chr6:111329244-111329303	RPF2
A_33_P3339376	3.1674995	3.8860931	BC110288	chr2:175435956-175435897	WIPF1
A_23_P213336	6.088118	5.1227202	NM_000800	chr5:141974871-141974812	FGF1
A_33_P3362900	6.6938696	6.6395173	NM_001145710	chr2:24392427-24392486	FAM228B
A_23_P101972	4.0861597	4.235204	NM_144575	chr2:30945827-30945768	CAPN13
A_24_P307175	7.360765	7.2645936	NM_052928	chr17:1683022-1682963	SMYD4
A_33_P3320082	6.2345233	6.3988075	NM_005596	chr9:14307048-14306989	NFIB
A_23_P130974	4.9095044	5.0037847	NM_025249	chr19:18367982-18367923	KIAA1683
A_23_P35414	5.4828987	5.9677114	NM_005398	chr10:93388289-93388230	PPP1R3C
A_23_P204052	9.187328	9.195574	NM_031989	chr12:53849761-53853090	PCBP2
A_33_P3214179	3.8650916	3.7273684		chr22:029280309-029280368	
A_23_P34144	5.1689434	5.4094343	NM_014061	chrX:55479877-55479936	MAGEH1
A_33_P3374559	3.658944	3.098318	NR_024448	chr22:24057258-24057199	GUSBP11
A_23_P31064	8.398382	8.229789	NM_015529	chr6:132617445-132617386	MOXD1
A_33_P3391756	4.9791465	4.95399		chr1:1366001-1365942	
A_23_P331670	9.709972	9.471381	NM_002862	chr20:25278326-25278385	PYGB
A_24_P383330	13.054146	13.0984		chr4:189271055-189270996	
A_24_P183094	11.267124	11.089325	NM_024524	chr3:194123543-194123484	ATP13A3
A_23_P149678	9.231443	9.416334	NM_006099	chr1:145586275-145586334	PIAS3
A_24_P194154	10.7776785	10.498919	NM_002911	chr19:18978975-18979034	UPF1
A_33_P3402918	8.536125	8.891819	NM_007254	chr19:50364766-50364707	PNKP
A_33_P3296313	5.094256	4.7013874		chrX:050213438-050213497	
A_32_P3556	10.532987	10.816252	NM_018206	chr16:46695648-46694523	VPS35
A_33_P3315510	5.3543553	5.9966407	XM_005248963	chr6:35085195-35085254	ANKS1A
A_24_P219053	6.313588	6.350856	AB051491	chr13:45605831-45605890	

A_33_P3322504	5.261828	5.8992558		chr7:155134316-155134375	
A_23_P309381	4.9998446	4.5928073	NM_001040874	chr1:149822628-149822687	HIST2H2AA4
A_24_P271149	5.7081823	6.135766	NM_001101372	chr19:51833227-51833286	IGLON5
A_33_P3340199	6.642375	7.055529		chr4:106041082-106041141	
A_23_P212696	10.029689	10.533703	NM_007085	chr3:120113491-120113432	FSTL1
A_24_P4705	4.846374	4.622403	NM_016147	chr11:73941958-73942017	PPME1
A_23_P63825	9.57195	9.751369	NM_002079	chr10:101157284-101157225	GOT1
A_33_P3252146	7.76454	8.09865	NM_001105574	chr10:124897188-124897247	HMX3
A_23_P43157	9.313232	9.384888	NM_001080416	chr8:67474765-67474706	MYBL1
A_23_P8196	8.029147	7.5707717	NM_002395	chr6:83920855-83920796	ME1
A_33_P3270581	14.545278	13.947929		chr3:138363204-138363145	
A_23_P15832	3.2660892	3.011922	NM_014566	chr17:2966397-2966338	OR1D5
A_33_P3421275	3.893358	3.6606207	NM_152568	chr8:41503888-41503829	NKX6-3
A_23_P132027	5.3868804	5.7510214	NM_003116	chr20:34207653-34208649	SPAG4
A_24_P401601	7.0205083	6.405931		chr19:021145404-021145465	
A_33_P3341499	9.6104765	8.8969	NM_003392	chr3:55499859-55499800	WNT5A
A_24_P298360	7.3794193	7.230195	NM_021070	chr11:65307043-65306896	LTBP3
A_23_P135061	6.610898	6.9383564	NM_003389	chr9:100886921-100886862	CORO2A
A_23_P309246	3.6218941	3.6749473	NM_145115	chr7:99228302-99228361	ZSCAN25
A_23_P25253	9.054295	9.091655	NM_015954	chr12:16190088-16190147	DERA
A_33_P3252441	5.557842	5.462558		chr17:039258066-039258007	
A_33_P3251227	6.8452063	6.750681	AJ132443	chr7:7278507-7278566	C1GALT1
A_32_P217655	8.683146	8.377487	NR_027355	chr1:148951509-148951568	LOC645166
A_33_P3398867	4.1658664	3.4116144	AF438406	chr11:82533532-82533591	GCRG224
A_23_P206724	10.711124	10.914124	NM_175617	chr16:56660853-56660912	MT1E
A_33_P3381361	4.4488425	4.6772003	AF090912	chr16:89590375-89590434	SPG7
A_23_P106174	5.3095818	5.6647162	AJ008005	chr14:73640376-73640435	PSEN1
A_33_P3780572	2.615822	2.3900566		chr4:189459645-189459704	LINC01060
A_23_P32320	3.6013389	3.6934814	NM_024083	chr17:79967402-79968695	ASPSCR1
A_33_P3269149	6.348262	7.048253	NM_017919	chr9:102730888-102730947	STX17
A_33_P3413910	6.539898	6.7461615	NM_024866	chr22:50921310-50921369	ADM2
A_24_P102880	6.2310796	6.386279	NM_020443	chr1:201793533-201793592	NAV1
A_24_P227091	9.322905	9.644272	NM_004523	chr10:94414627-94414686	KIF11
A_23_P46690	3.7286623	3.0060992	NM_203376	chr1:205052964-205052905	TMEM81
A_33_P3387901	3.8952465	3.732614	NM_001126121	chr17:73279612-73279553	SLC25A19
A_24_P16730	3.786128	4.183386	NM_030650	chr2:176791499-176791440	KIAA1715
A_23_P204929	8.807152	8.469908	NM_016248	chr13:42897130-42897189	AKAP11
A_24_P364087	8.804282	8.994986	NM_012139	chr11:17809710-17809651	SERGEF
A_33_P3253857	4.6643257	4.748889		chr19:058557952-058558011	
A_33_P3327270	9.113114	8.82753	NM_001164469	chr5:114915373-114915314	TMED7-TICAM2
A_23_P133095	8.315938	8.237995	NM_014247	chr4:160281049-160281108	RAPGEF2
A_23_P120660	14.417201	14.536229	NM_001024	chr20:60962941-60963394	RPS21
A_32_P19716	6.8139687	6.652691	NM_001080470	chr1:120162453-120162394	ZNF697
A_23_P326319	2.3221061	2.3900566	NM_033201	chr16:15681608-15681667	C16orf45
A_23_P133345	9.695611	9.44618	NM_014666	chr5:157214382-157214323	CLINT1
A_23_P318296	5.8828206	5.9266715	NM_032639	chr7:30102293-30102352	PLEKHA8
A_23_P257043	8.397327	7.605952	NM_005261	chr8:95262007-95261948	GEM
A_24_P706340	8.013159	7.7865067	NM_001080396	chr13:107822431-107822372	FAM155A
A_23_P146644	13.979262	14.224985	NM_001002857	chr15:60641324-60639880	ANXA2
A_24_P220454	8.063571	8.047012	NM_181552	chr7:101893025-101893084	CUX1
A_33_P3281795	11.935347	12.062395	NM_007283	chr3:127407970-127407911	MGLL
A_23_P212475	13.083107	13.17179	NM_016479	chr3:48509336-48509277	SHISA5
A_24_P210675	4.154693	4.003939	NM_017668	chr16:15781297-15781356	NDE1
A_24_P54485	3.9533222	3.832833	NM_032357	chr2:131098542-131098483	CCDC115
A_23_P125717	2.3221061	2.3900566	NM_004538	chrX:92926556-92926497	NAP1L3
A_23_P329890	9.371988	9.344556	NM_174926	chr11:120201251-120201310	TMEM136
A_23_P43490	14.123182	14.192438	NM_058197	chr9:21968098-21968039	CDKN2A
A_33_P3362353	5.5840483	4.896267	NM_001256197	chrX:47424996-47425055	ARAF
A_33_P3268910	7.4925723	8.106397	NM_001258038	chr4:124318918-124318977	SPRY1

A_33_P3293307	5.5328026	5.9242783		chrY:016145404-016145345	
A_23_P304716	5.158676	4.970074	NM_019089	chr1:6475796-6475737	HES2
A_23_P19291	11.420245	11.691533	NM_001069	chr6:3154094-3154035	TUBB2A
A_33_P3213006	10.33998	10.519711	NM_001257305	chr17:39186024-39185965	KRTAP1-4
A_24_P95154	10.101682	10.586225	NM_178234	chr8:15601061-15601120	TUSC3
A_24_P409881	10.699971	10.545507		chr12:079952631-079952690	
A_33_P3370890	5.333804	4.9402943	NM_001033564	chr6:112423914-112423973	FAM229B
A_24_P396327	6.188941	6.363211	NM_138467	chr1:75230968-75231027	TYW3
A_33_P3210303	3.665457	3.3101244	CU688821	chr4:6303474-6303415	
A_24_P882914	6.023318	5.0466356	NM_153810	chr10:120441541-120441482	CACUL1
A_23_P413224	3.8482833	3.9931388	NM_004828	chr6:41304077-41304136	NCR2
A_33_P3310246	2.3221061	2.3900566	NR_038371	chr7:53833897-53833838	FLJ45974
A_23_P92202	5.1877055	5.3882203	NM_021971	chr3:49760101-49760042	GMPPB
A_24_P407235	7.9203825	7.5357842	NM_004075	chr12:107385541-107385482	CRY1
A_33_P3268005	4.390353	4.412528	XM_005251736	chr9:96213430-96213371	FAM120AOS
A_23_P215735	6.10776	6.2121286	NM_018412	chr7:116849952-116859157	ST7
A_33_P3286121	4.053425	3.9211414	NM_001195190	chr1:173606104-173606163	LOC730159
A_32_P206293	8.582634	8.630993	NM_001242797	chr6:26634767-26634708	ZNF322
A_33_P3321711	7.9543896	7.4809484	NM_006809	chr20:43570831-43570772	TOMM34
A_23_P116264	6.55664	6.1460705	NM_006176	chr11:124616792-124616851	NRGN
A_23_P357966	7.636669	7.3450813	NM_052937	chr8:52730327-52730268	PCMTD1
A_24_P8075	6.5178843	6.1258297	XR_243498	chr16:87732084-87732025	KLHDC4
A_24_P940006	5.6328273	5.8027697	NM_001406	chr17:7614123-7614182	EFNB3
A_23_P336513	9.439233	9.1461525	NM_015465	chr5:154270927-154270868	GEMIN5
A_33_P3322328	7.1789603	6.9706755	NM_001981	chr1:51820011-51819952	EPS15
A_24_P285623	10.298027	10.439302	NM_080916	chr2:74185334-74185857	DGUOK
A_33_P3244921	8.716154	8.92687	NM_001207058	chr15:65871517-65871458	VWA9
A_24_P75158	9.742509	9.188214	NM_001099666	chr9:72324577-72324518	PTAR1
A_24_P82106	6.8708925	6.210803	NM_004995	chr14:23315791-23315850	MMP14
A_23_P87532	7.909619	7.866905	NM_001002259	chr12:30862819-30862760	CAPRIN2
A_24_P85942	7.847318	8.1797695	NM_181453	chr2:109116109-109116168	GCC2
A_23_P202334	4.5339804	4.673257	NM_022970	chr10:123237843-123237830	FGFR2
A_23_P337790	6.372566	6.2633395	NM_001042683	chr6:146206540-146206481	SHPRH
A_23_P500410	5.1051993	5.064486	NM_130463	chr6:31512332-31512273	ATP6V1G2
A_24_P34155	4.833164	4.3201756	NM_001122607	chr21:36193642-36193583	RUNX1
A_23_P46829	4.1836185	4.134432	NM_033163	chr10:103531264-103530362	FGF8
A_33_P3263432	4.978095	4.2813025	NM_003637	chr1:145543808-145543867	ITGA10
A_23_P142724	14.77689	14.783885	NM_000998	chr2:217363592-217364043	RPL37A
A_32_P57057	8.168689	8.184296	NM_006313	chr12:62688025-62688084	USP15
A_33_P3359846	6.032581	5.854482		chr5:079439878-079439937	
A_23_P20722	9.546438	9.4007225	NM_003086	chr9:139270092-139270033	SNAPC4
A_32_P234604	11.217627	11.127678	NM_005022	chr17:4849238-4849180	PFN1
A_33_P3395688	2.8142924	3.2522547	NM_001018100	chr15:57967187-57967246	MYZAP
A_24_P132624	5.588937	6.0753603	NM_018376	chr9:107533210-107533269	NIPSNAP3B
A_23_P138665	10.432138	10.693255	NM_005271	chr10:88811085-88811026	GLUD1
A_23_P46396	8.194221	8.296107	NM_021190	chr1:97279985-97280044	PTBP2
A_32_P193080	7.099571	7.523408	NM_052905	chr2:153505480-153505539	FMNL2
A_23_P70355	6.900079	7.116943	NM_004568	chr6:2955771-2954898	SERPINB6
A_33_P3363305	8.610956	8.914458		chr11:071279748-071279807	
A_23_P78888	13.520017	13.3064995	NM_001436	chr19:40325192-40325133	FBL
A_23_P26439	4.038496	4.087855	NM_001042610	chr16:90072057-90071998	DBNDD1
A_33_P3267356	9.13327	9.211214	NM_001256310	chr2:191797529-191797588	GLS
A_24_P242609	9.245571	8.89003	NM_021633	chr1:202860502-202860443	KLHL12
A_23_P92349	6.466616	6.318448	NM_001004356	chr4:1019939-1019998	FGFRL1
A_33_P3304668	3.1517034	3.5923386	NM_000088	chr17:48261568-48261509	COL1A1
A_24_P82142	9.165012	9.2767515	NM_207038	chr15:57579954-57580013	TCF12
A_33_P3296333	3.3839095	2.3900566	NM_001144058	chr11:132206492-132206551	NTM
A_23_P29630	9.363431	8.54358	NM_014041	chr3:52740830-52740889	SPCS1
A_24_P257108	7.5651436	7.6803985	NM_133484	chr2:162060063-162061201	TANK

A_23_P42335	8.165924	8.020627	NM_021922	chr6:35434765-35434824	FANCE
A_23_P46470	10.165661	10.423117	NM_018948	chr1:8072267-8072208	ERRFI1
A_23_P145	8.079461	8.217353	NM_000191	chr1:24128883-24128824	HMGCL
A_33_P3284951	7.2930284	7.045147	NM_006739	chr22:35813789-35813848	MCM5
A_23_P148410	11.165808	11.264172	NM_031894	chrX:31089993-31089934	FTHL17
A_24_P226210	5.954524	6.291459	NM_153223	chr5:122681225-122681166	CEP120
A_23_P33914	2.6948578	2.3900566	NM_017681	chrX:106367003-106366944	NUP62CL
A_23_P217339	5.7733383	6.0045958	NM_005044	chrX:3522850-3522791	PRKX
A_33_P3309646	6.2125974	6.2277417	AK090395	chr7:143427409-143427468	
A_33_P3388067	5.2858086	5.4488754	NM_001159524	chr7:63680485-63680544	ZNF735
A_33_P3288047	3.5831459	2.3900566		chr6:086445567-086445508	
A_33_P3408722	5.360671	5.149114	NM_001184941	chr9:34402449-34402390	FAM219A
A_33_P3217393	4.4348617	4.385993	NM_001024736	chr15:73996699-73996758	CD276
A_33_P3420035	7.5357122	7.598565	NM_181489	chr3:44482828-44482769	ZNF445
A_33_P3811287	9.518099	9.601952	NM_031407	chrX:53561537-53561478	HUWE1
A_24_P67946	2.6468198	3.2209039	NM_199040	chr12:93796090-93796149	NUDT4
A_23_P370574	5.564062	5.4608164	NM_153443	chr19:55241039-55241098	KIR3DL3
A_33_P3292478	8.918445	9.120805	NM_004590	chr17:34308456-34308397	CCL16
A_33_P3397161	6.810337	7.042016	NR_073536	chr11:70709606-70709665	SHANK2-AS3
A_23_P168669	5.159072	5.6626806	NM_021151	chr7:87027998-87028057	CROT
A_32_P68050	6.2494793	6.3056526	NM_012224	chr4:170347344-170345940	NEK1
A_33_P3333337	4.8652554	4.849268	NM_001164213	chr13:47324726-47324785	LRCH1
A_24_P366787	4.9626045	5.243183	NR_024371	chr1:2983914-2980821	LINC00982
A_23_P38468	6.5990314	6.739225	NM_014604	chr17:3567561-3567502	TAX1BP3
A_23_P8119	5.1497073	5.125923	NM_152735	chr6:33424697-33424756	ZBTB9
A_23_P392470	4.7231536	5.0738387	NM_000901	chr4:149000423-149000364	NR3C2
A_23_P39185	3.6493278	3.7845893	NM_138412	chr19:55556622-55556563	RDH13
A_32_P834166	10.937227	11.260027	AF128541	chr12:133413570-133413511	LOC100128843
A_23_P256342	7.6574	7.788666	NM_015132	chr7:17833194-17833135	SNX13
A_23_P48596	6.52901	6.9061484	NM_198232	chr14:21269661-21269602	RNASE1
A_33_P3338335	7.6528125	7.9859276	NM_177401	chr19:1258896-1258955	MIDN
A_33_P3301514	4.0049853	4.3287168	NM_001193582	chr7:107799985-107799926	NRCAM
A_33_P3317558	4.8999367	5.26174	NR_003521	chr15:23196257-23196198	WHAMMP3
A_23_P15692	5.486526	5.486368	NM_017986	chr17:4936164-4936105	SLC52A1
A_24_P358205	13.884267	13.929015		chr6:010214455-010214395	
A_23_P202683	3.986652	3.904726	NM_021924	chr11:617509-617450	CDHR5
A_33_P3332396	5.388662	5.475458	NM_024587	chr1:45125906-45125847	TMEM53
A_33_P3421611	5.3138766	5.4315596		chr2:43460474-43460533	
A_23_P6786	6.1413603	6.1397424	AK124137	chr3:3171928-3171987	TRNT1
A_23_P45524	13.3608	13.418844	NM_014380	chrX:102632908-102632967	NGFRAP1
A_23_P255663	7.593011	7.558831	NM_024641	chr6:96056776-96056835	MANEA
A_23_P502078	5.197784	5.1066284	NM_012324	chr22:51049500-51049559	MAPK8IP2
A_23_P37949	6.51905	6.2607627	NM_024339	chr16:3076557-3076707	THOC6
A_24_P201531	7.9189568	8.240978	NM_001655	chr11:118472982-118473041	ARCN1
A_33_P3219090	8.290501	7.707928	NM_005542	chr7:155101843-155101902	INSIG1
A_33_P3339109	6.4879026	6.597178	CA416988	chr6:43487252-43487311	POLR1C
A_24_P316019	5.2479544	5.183666	NM_001282484	chr15:30699524-30699210	LOC101059918
A_23_P98631	7.6631765	7.701232	NM_181507	chr11:18301077-18301018	HPS5
A_33_P3288904	5.7658234	6.0109215	NM_003899	chr13:111932964-111933023	ARHGEF7
A_33_P3415124	5.5769844	5.557331	AK127211	chr12:124418849-124418790	DNAH10OS
A_33_P3257279	7.2219963	7.2287874	NM_173633	chr19:42829137-42829196	TMEM145
A_33_P3215078	12.818307	12.677298	NM_004077	chr12:56665557-56665498	CS
A_23_P152583	6.473522	6.5829215	NM_001042573	chr17:77084403-77084462	ENGASE
A_23_P370682	3.1842985	2.3900566	NM_138456	chr11:64755631-64755572	BATF2
A_33_P3210338	7.9565725	7.708973	NM_022841	chr15:56382860-56382801	RFX7
A_33_P3383900	3.5008297	3.978881		chr4:184362597-184362538	
A_33_P3385842	4.6710167	4.2028575	NM_145023	chr10:32856759-32856818	CCDC7
A_23_P40693	8.594114	8.180624	NM_001429	chr22:41575033-41575092	EP300
A_23_P163117	2.835895	2.3900566	NM_024644	chr14:73959976-73960035	C14orf169

A_33_P3244568	4.5033407	4.283107	NR_024561	chr19:35563764-35563705	HPN-AS1
A_24_P114183	12.595854	12.531344	NM_002004	chr1:155290269-155290328	FDPS
A_33_P3238815	5.569693	5.4658074	NM_001286491	chr15:44163109-44163050	FRMD5
A_33_P3330404	5.448702	5.6128798	NM_001164379	chr11:47610682-47610741	FAM180B
A_24_P38143	5.97825	5.9366884	NM_017651	chr6:135605470-135605411	AHI1
A_23_P99204	6.6391854	6.306793	NR_015404	chr12:112278006-112277947	MAPKAPK5-AS1
A_24_P565556	7.5896087	7.6377497	NM_019109	chr16:5135348-5135407	ALG1
A_33_P3315423	4.3816524	3.9518027	NM_018207	chr1:33623965-33623906	TRIM62
A_24_P273666	14.154367	14.48668	NM_001077489	chr20:57486004-57486063	GNAS
A_23_P51291	5.960212	6.051505	NM_152268	chr1:55222923-55222864	PARS2
A_23_P346421	6.435328	6.095006	NM_018181	chr18:56651684-56651743	ZNF532
A_23_P210330	8.060247	7.99166	NM_014181	chr2:64688319-64688378	LGALSL
A_23_P500328	3.7211113	3.7597725	NM_000555	chrX:110537669-110537610	DCX
A_23_P68910	5.4525347	5.9682803	NM_001051	chr22:37602917-37602858	SSTR3
A_33_P3296497	4.273923	4.1859264	BC063596	chr6:128812937-128812878	PTPRK
A_23_P57236	4.507066	4.3468695	NM_178026	chr20:33433205-33433146	GGT7
A_33_P3358213	4.118888	4.081486	NM_207421	chr1:17728136-17728195	PADI6
A_23_P165380	11.327057	11.525513	NM_014617	chr2:209025737-209025678	CRYGA
A_23_P202143	9.44976	9.316243	NM_001284388	chr10:103922940-103922999	NOLC1
A_33_P3400843	6.389187	6.5473714	NM_001008223	chr12:49726319-49726260	C1QL4
A_33_P3421515	13.942377	14.091058		chr3:027676788-027676847	
A_24_P391526	9.38109	9.846745	NM_001005333	chrX:51643374-51644689	MAGED1
A_32_P169406	4.633143	4.141499	NR_026656	chr12:54526331-54526390	LOC400043
A_23_P88522	8.969927	8.939194	NM_021077	chr15:85198461-85198402	NMB
A_23_P116694	14.497476	14.242917	NM_001029	chr12:56437923-56437982	RPS26
A_23_P357101	7.256115	7.332473	NM_145298	chr22:39448588-39448647	APOBEC3F
A_24_P101114	5.5484333	5.904831	NM_206999	chr16:58577644-58577585	CNOT1
A_23_P70794	6.55236	6.809185	NM_016277	chr6:57054274-57054215	RAB23
A_23_P86195	5.362182	5.242237	NM_152369	chr1:95358013-95358072	SLC44A3
A_33_P3264855	4.8423223	4.5227675	NR_103448	chr6:28137281-28137340	ZNF192P1
A_33_P3259938	5.03341	5.0986476	AK128153	chr9:140118626-140118567	LOC100130547
A_23_P258978	4.7109594	4.8864484	NM_002077	chr9:127641177-127641118	GOLGA1
A_33_P3369761	10.623973	10.672243	NM_001161779	chr8:94938235-94938294	PDP1
A_32_P182941	12.119818	12.22564	NM_001005	chr11:75111802-75111861	RPS3
A_23_P44295	8.393813	8.003159	NM_015097	chr3:33538683-33538624	CLASP2
A_33_P3410225	7.5557213	6.6490602		chr22:041184900-041184841	
A_23_P133438	5.5726423	5.7684984	NM_019018	chr5:14610510-14610569	FAM105A
A_23_P90659	12.813116	13.078306	NM_014713	chr2:20232535-20232476	LAPTM4A
A_33_P3215848	3.7557251	4.0192595	BC044628	chr16:2016359-2016300	
A_33_P3392802	3.8612373	3.298787		chr2:10008834-10008893	TAF1B
A_33_P3244828	6.9533834	7.1711655		chr14:102231782-102231841	
A_33_P3272352	7.2686586	7.63688		chr2:37440789-37440848	
A_33_P3504659	4.667725	3.9786258	NM_032977	chr2:202084646-202084705	CASP10
A_33_P3257678	4.518847	4.163535	NM_001005464	chr1:149824626-149824685	HIST2H3A
A_23_P257971	5.1149626	5.4281464	NM_001353	chr10:5019910-5019969	AKR1C1
A_33_P3287680	4.231446	4.146162	NM_001077657	chr3:196233970-196233911	C3orf43
A_33_P3293009	8.42036	9.041778	NM_152864	chr20:61872198-61872139	NKAIN4
A_33_P3354322	13.947929	13.942377	NM_201397	chrX:013396702-013396643	GPX1
A_33_P3335042	8.871408	9.3381815	NM_016142	chr11:43775611-43775670	HSD17B12
A_23_P312246	8.953343	8.920335	NM_024725	chr11:96086559-96086500	CCDC82
A_33_P3417318	5.9579887	6.2125974	NM_001277291	chr12:133696960-133696901	ZNF891
A_23_P431305	7.82608	7.8399415	NM_152421	chr9:139618279-139618338	FAM69B
A_24_P312041	4.976512	5.042971	NM_006718	chr6:144262889-144262830	PLAGL1
A_24_P166613	11.296958	10.895468	NM_017549	chr7:37991294-37991353	EPDR1
A_24_P197964	8.202939	7.9891315	NM_014788	chr9:100846713-100846654	TRIM14
A_24_P327050	10.164868	10.426489	NM_016209	chr16:88926301-88926360	TRAPPC2L
A_23_P160481	9.684581	10.26808	NM_018442	chr1:168035668-168037619	DCAF6
A_33_P3735771	4.335672	4.7209105		chrX:115032579-115032520	
A_23_P200252	8.394832	8.629108	NM_032324	chr1:233105679-233105738	NTPCR

A_33_P3371142	4.9333577	5.143901		chrX:009381485-009381544	
A_24_P532864	2.3221061	2.3900566		chr5:82215551-82215492	
A_23_P88470	5.801613	5.8201265	NM_017672	chr15:50853618-50853559	TRPM7
A_33_P3235004	10.003825	10.197508	NM_001256012	chr17:8377599-8377540	MYH10
A_33_P3223056	5.8580036	6.139139	NM_030957	chr19:8645185-8645126	ADAMTS10
A_33_P3364240	4.651446	4.186993	NM_002571	chr9:138457300-138457359	PAEP
A_33_P3290303	4.406177	4.6425405	AK129701	chr13:113392026-113392085	
A_33_P3311668	3.459628	3.5533454	AA489744	chr12:3866906-3866965	
A_33_P3329712	3.7882981	3.6044905		chr11:004731639-004731698	
A_33_P3328154	5.382003	5.3670287	BC014325	chr19:37875829-37875888	ZNF527
A_23_P43810	4.868403	4.689865	NM_206943	chr2:33623836-33623895	LTBP1
A_24_P134074	14.430021	14.490176	NM_001022	chr19:42365231-42373109	RPS19
A_23_P89187	10.183749	10.285808	NM_006148	chr17:37077464-37077523	LASP1
A_24_P294842	4.517812	5.0897336	NM_000332	chr6:16299393-16299343	ATXN1
A_33_P3378895	3.811533	3.5866108		chr8:12623977-12623918	LOC340357
A_33_P3242820	7.311602	7.270641	NR_026927	chr1:2481539-2481480	LOC115110
A_33_P3379463	10.793166	11.15578	NM_007260	chr1:24121848-24121907	LYPLA2
A_24_P159227	2.3446383	2.3900566	NM_020168	chr15:40569328-40569387	PAK6
A_24_P372223	2.7616687	2.3900566	NM_138715	chr8:15965525-15965466	MSR1
A_33_P3315856	8.515748	8.416958	NM_014681	chr19:47885833-47885892	DHX34
A_23_P500206	8.46291	8.620392	NM_153483	chr3:9957731-9957790	IL17RE
A_24_P374586	6.520181	6.629662	NM_000535	chr7:6043422-6043363	PMS2
A_23_P128067	15.140327	15.252923	NM_001035267	chr12:56511329-56511388	RPL41
A_33_P3229196	14.380219	14.46887	NM_004357	chr11:838770-838829	CD151
A_23_P372925	8.311108	8.552848	NM_033426	chr14:77583194-77583253	KIAA1737
A_23_P83200	4.457915	4.393046	NM_152572	chr9:135601142-135601083	AK8
A_24_P20120	9.482103	9.460372	NM_018084	chr2:55515393-55515334	CCDC88A
A_24_P392713	12.1346035	12.304049	AK124741	chr2:61165530-61165471	
A_33_P3311439	2.99031	3.4891388	NM_001024071	chr14:55312549-55312490	GCH1
A_23_P353316	5.4103928	5.629836	NM_020429	chr7:98625237-98625178	SMURF1
A_32_P234145	2.4770436	2.8636558	NM_203349	chr15:49116206-49116147	SHC4
A_23_P208850	13.640844	13.788274	NM_001020	chr19:39924395-39924336	RPS16
A_33_P3242743	6.0994673	5.9405193	NM_001267549	chr20:62330066-62330007	ARFRP1
A_23_P376096	4.501444	4.5822544	NM_182919	chr19:4816695-4816636	TICAM1
A_33_P3365586	6.847789	6.4483614	NM_004738	chr20:57021815-57021874	VAPB
A_33_P3328410	2.4510043	2.8653286		chr11:65233969-65234028	LOC101927789
A_23_P119464	5.892935	6.4547043	NM_033103	chr19:33470925-33470866	RHPN2
A_33_P3284808	5.9317355	6.188941	XM_003403619	chr10:19739359-19739418	C10orf112
A_33_P3256785	11.121452	10.877258	NM_199141	chr19:11033280-11033339	CARM1
A_33_P3397288	4.272134	4.799068	NM_000114	chr20:57899402-57899461	EDN3
A_33_P3327961	6.4968786	6.7248673	NM_001199324	chr19:52494807-52494748	ZNF615
A_33_P3221203	2.3221061	2.3900566	NM_002427	chr11:102813832-102813773	MMP13
A_23_P156632	4.61751	4.7187953	NM_032507	chr6:28269645-28269704	PGBD1
A_23_P168610	5.294986	5.7488456	NM_014399	chr7:16818689-16823048	TSPAN13
A_23_P2397	3.963334	3.9125013	NM_178169	chr12:65082158-65082217	RASSF3
A_33_P3241884	4.841716	4.477987	NM_014654	chr1:31346097-31346038	SDC3
A_33_P3400424	3.6613245	4.609335	NM_001199324	chr19:52496192-52496133	ZNF615
A_33_P3363445	5.0651355	4.7538123	XR_109907	chr2:3579467-3579408	LOC100506014
A_33_P3221960	7.6329226	7.344114	NM_003853	chr2:103068695-103068754	IL18RAP
A_24_P30314	12.149535	12.074901	NM_020680	chr11:65306114-65306173	SCYL1
A_33_P3233125	9.786617	9.973049	NM_002779	chr10:104162436-104162377	PSD
A_33_P3376449	3.7914321	3.717558	NM_173570	chr3:113677290-113677349	ZDHHC23
A_23_P257256	6.6927137	7.0713654	NM_002082	chr5:176868830-176868889	GRK6
A_33_P3341239	12.14043	11.997747	NM_006201	chrX:47089333-47089392	CDK16
A_24_P228637	7.69506	7.833664	NM_001007259	chr21:37416159-37416100	SETD4
A_23_P104641	8.586117	8.485591	NM_013265	chr11:64878902-64878961	VPS51
A_23_P127186	8.636701	8.440714	NM_206862	chr10:124013760-124013819	TACC2
A_23_P23719	7.8535595	7.939571	NM_007259	chr1:150117022-150117081	VPS45
A_33_P3388883	5.1725016	4.9495816		chr1:144873273-144873214	

A_23_P39453	7.2273817	7.313191	NM_203304	chr19:1555264-1555205	MEX3D
A_33_P3297517	7.7325077	7.6517835	NM_001243879	chr3:185655716-185655657	TRA2B
A_23_P203023	9.535932	9.699213	NM_002906	chr11:110100899-110100840	RDX
A_33_P3349646	4.530376	4.9287295	NM_002589	chr4:30726159-30726218	PCDH7
A_23_P62276	6.6631317	6.7224326	NM_024528	chrX:119065970-119065911	NKAP
A_33_P3420862	2.3221061	2.3900566	NM_207339	chrX:55117830-55117889	PAGE2
A_23_P92057	7.9129915	8.286754	NM_006218	chr3:178948163-178951939	PIK3CA
A_23_P368145	7.7346325	7.946102	NM_152281	chr1:170522136-170522195	GORAB
A_33_P3424861	6.28635	6.5300794	NM_001104595	chr22:45736795-45736854	FAM118A
A_33_P3408665	8.461107	8.550901		chrUn_gl000217:000049977-000050036	
A_33_P3401243	6.061604	6.535187	NM_015441	chr1:161953465-161953406	OLFML2B
A_23_P148919	8.186397	8.491301	NM_000098	chr1:53679252-53679311	CPT2
A_24_P298545	9.549081	9.6598015	NM_033468	chr19:22255690-22256282	ZNF257
A_23_P24457	5.6626806	4.7561336	NM_020929	chr11:40136152-40136093	LRRC4C
A_23_P45389	9.042097	9.197699	NM_004251	chrX:13727357-13727416	RAB9A
A_24_P383901	10.825691	11.018518		chr22:041470381-041470322	
A_24_P30923	7.501716	8.505054	NM_003498	chr16:11772637-11772696	SNN
A_33_P3422888	5.852559	6.195802	NM_001243403	chr16:11220142-11220201	CLEC16A
A_23_P52610	9.774015	9.941192	NM_000107	chr11:47260623-47260682	DDB2
A_33_P3257005	4.70234	4.871009		chr2:224569924-224569983	
A_33_P3215178	3.459339	3.6124718	NM_004204	chr16:632976-633035	PIGQ
A_24_P280873	8.328269	8.430808		chr6:066547471-066547412	
A_24_P319684	3.9927993	4.39216	NM_024584	chr2:27849929-27849870	CCDC121
A_23_P414308	6.869364	7.1509075	NM_144606	chr17:17124727-17124668	FLCN
A_23_P162982	7.036346	6.9860287	NM_021004	chr14:24429166-24434983	DHRS4
A_33_P3296789	6.5333643	6.494845	NM_001166422	chrX:71792562-71792503	HDAC8
A_23_P502654	8.168045	8.268956	NM_004169	chr17:18231585-18231526	SHMT1
A_23_P303891	10.22	10.545138	NM_178351	chr1:152777894-152777835	LCE1C
A_23_P167497	4.350947	4.1429815	NM_001962	chr5:106716478-106716419	EFNA5
A_23_P75764	6.5492125	6.445773	NM_001004728	chr11:59210851-59210910	OR5A1
A_23_P107587	9.690325	10.1807995	NM_000271	chr18:21113347-21112218	NPC1
A_23_P419213	5.470087	5.918362	NM_020817	chr3:113683324-113683265	KIAA1407
A_23_P217637	8.468277	8.266092	NM_004085	chrX:100600640-100600627	TIMM8A
A_33_P3302220	6.870051	7.06269	NM_001032287	chr12:95425193-95425134	NR2C1
A_24_P383598	7.273517	7.2162623		chr4:121263539-121263598	
A_33_P3299510	5.055012	4.896345	NM_001008271	chr8:145491109-145491168	SCXA
A_23_P254888	6.7532325	6.432577	NM_003461	chr7:143087000-143087059	ZYX
A_33_P3423984	3.72952	2.3900566	AK096458	chr4:169621207-169621266	PALLD
A_33_P3278318	5.594349	5.5684795	NM_012228	chr10:23410806-23410865	MSRB2
A_23_P433676	6.5577555	6.334667	NM_020951	chr19:37035624-37035565	ZNF529
A_33_P3374684	6.594199	6.790622		chr16:34681497-34681438	
A_23_P322704	7.872838	7.4897265	NM_001079519	chr14:35546376-35548145	FAM177A1
A_23_P113417	6.568267	6.3567286	NM_025161	chr17:79507457-79507398	C17orf70
A_24_P279489	4.4321847	4.498003	NM_001128159	chr17:415342-415283	VPS53
A_33_P3277659	3.1133718	3.0821142	NM_001145029	chr18:14852306-14852365	ANKRD30B
A_33_P3338733	7.3964815	7.5053444	NM_198159	chr3:70014715-70014774	MITF
A_23_P344451	9.765133	9.999372	NM_016073	chr15:83807307-83807248	HDGFRP3
A_24_P861009	4.59671	4.39051	NM_001007246	chr21:40668993-40668934	BRWD1
A_33_P3348494	9.771924	9.364508	NM_001258213	chr13:113831995-113831936	PCID2
A_33_P3254460	7.2240443	7.605247	NM_206539	chr6:43418202-43418143	DLK2
A_32_P116556	6.220334	5.724474	NM_001127464	chr16:88507087-88507146	ZNF469
A_24_P217306	4.606724	4.744365	NM_032153	chr3:147104136-147104077	ZIC4
A_24_P58308	13.82132	13.82132	AF147412	chr6:157298027-157298086	
A_33_P3301286	7.672366	7.675034	NM_003478	chr11:107978373-107978432	CUL5
A_24_P363100	5.509366	5.7000585	NM_001012761	chr5:98115718-98115777	RGMB
A_23_P26895	7.018513	7.3913326	NM_016261	chr17:57937754-57937695	TUBD1
A_23_P21092	8.815406	8.39743	NM_001740	chr16:71419509-71423668	CALB2
A_23_P35066	10.363201	10.461803	NM_015976	chr1:99225718-99225777	SNX7

A_23_P71644	9.790122	9.840464	NM_004629	chr9:35073994-35073935	FANCG
A_33_P3366780	10.622841	10.96133		chr5:179224342-179224283	
A_23_P46182	15.021793	15.07173	NM_001012	chr1:45244296-45244355	RPS8
A_24_P105747	5.7069354	5.571695	NM_001039569	chr2:224622433-224622374	AP1S3
A_24_P46093	11.389118	11.707433	NM_003043	chr3:14530446-14530505	SLC6A6
A_33_P3380311	6.373885	6.2984896	NM_000489	chrX:76778809-76778750	ATRX
A_33_P3268532	6.6227107	6.394829	NM_001018073	chr14:24569694-24569753	PCK2
A_23_P163173	4.933681	5.167614	NM_019839	chr14:24780572-24780631	LTB4R2
A_23_P46017	7.2508926	7.7833104	NM_001008661	chr1:89420862-89418807	CCBL2
A_24_P310256	3.5450401	4.1301117	NM_139284	chr19:35615711-35615652	LGI4
A_23_P148372	8.37027	8.163386	NM_001325	chrX:100092406-100093254	CSTF2
A_24_P88763	4.5519304	2.910947	NM_032603	chr2:74760422-74760363	LOXL3
A_23_P17074	8.228438	8.34699	NM_032357	chr2:131096084-131096025	CCDC115
A_23_P250274	11.513375	11.665095	NM_019594	chr9:131680227-131680286	LRRC8A
A_23_P413788	7.178626	7.6110916	NM_173540	chr10:75535586-75535645	FUT11
A_23_P378288	5.6435766	5.249217	NM_022465	chr12:56432131-56432190	IKZF4
A_24_P418408	9.711143	9.8429	NM_198552	chr1:231155098-231155039	FAM89A
A_23_P39454	2.4009385	2.3900566	NM_024967	chr19:2878321-2878380	ZNF556
A_33_P3245243	5.408061	5.3498073		chr1:118341406-118341465	
A_23_P138139	7.6377497	7.901913	NM_145243	chr1:58971791-58971732	OMA1
A_33_P3349702	5.09132	5.3673244	NR_002821	chr22:38740729-38740670	LOC400927
A_23_P2317	5.0200915	5.3899145	NM_015086	chr12:49389625-49389566	DDN
A_23_P208238	7.0320816	7.2338705		chr19:53100573-53100632	ZNF137P
A_24_P195286	5.824276	5.804196		chrX:111933524-111933583	
A_23_P66682	2.3221061	2.3900566	NM_018952	chr17:46673252-46673193	HOXB6
A_23_P15174	5.7248383	5.874655	NM_005949	chr16:56693077-56693136	MT1F
A_33_P3209615	5.0558286	5.1122	NM_031960	chr17:39254329-39254270	KRTAP4-8
A_24_P346368	9.549246	9.043909	NM_017994	chr7:66423250-66423309	TMEM248
A_23_P429184	8.10305	7.680601	NM_198066	chr14:53242094-53242035	GNPNAT1
A_23_P44166	8.888165	9.0328665	NM_016286	chr17:79994081-79993895	DCXR
A_24_P137434	12.416422	12.472273	NM_080927	chr3:98515261-98515202	DCBLD2
A_23_P314584	8.616917	8.551771	NM_004635	chr3:50686364-50686423	MAPKAPK3
A_33_P3349716	8.31131	8.261865	NM_001010851	chr19:52795893-52795952	ZNF766
A_23_P1782	9.454327	9.588337	NM_002231	chr11:44640896-44640955	CD82
A_23_P136460	7.805396	7.4759235	NM_016603	chr5:137274299-137274240	FAM13B
A_23_P48803	3.4170022	3.9395123	NM_014548	chr15:52101182-52101241	TMOD2
A_23_P89812	10.879935	11.049772	NM_018235	chr18:72187712-72187771	CNDP2
A_33_P3578325	3.9138827	4.456545	NR_000005	chr11:75111452-75111511	SNORD15A
A_24_P11131	11.195654	11.217627	NM_005839	chr1:24999475-24999534	SRRM1
A_33_P3301851	12.222897	12.3095	NM_002419	chr11:65365286-65365227	MAP3K11
A_24_P118011	4.387789	4.1838646	NR_027258	chr2:55511495-55511554	PRORSD1P
A_33_P3219469	9.751369	8.768609	NM_004719	chr12:46313030-46312971	SCAF11
A_33_P3421520	6.1006308	6.6672363	NM_001271840	chr10:124753334-124753275	IKZF5
A_23_P44974	11.3519335	11.10536	NM_014078	chr8:121408269-121408210	MRPL13
A_24_P144773	3.1451497	4.3886995	NM_144726	chr5:158584423-158584402	RNF145
A_33_P3280841	3.899777	3.6250463	NR_027254	chr17:41031657-41031598	LINC00671
A_23_P136246	2.3221061	2.3900566	NM_130902	chr4:46736983-46736924	COX7B2
A_23_P33093	3.1140046	2.3900566	NM_030965	chr1:77529022-77529081	ST6GALNAC5
A_33_P3311907	6.8915024	6.973555	NM_001258457	chr6:13325400-13325341	TBC1D7
A_33_P3252794	9.078285	9.065286	NM_138465	chr8:144359040-144359099	GLI4
A_23_P13364	8.993384	9.238633	NM_005013	chr11:17351767-17351826	NUCB2
A_33_P3334180	4.9208446	5.0146437	AB527144	chr1:2434304-2434363	PLCH2
A_33_P3284463	9.810885	9.860092	NM_177924	chr8:17914026-17913967	ASAH1
A_24_P122050	5.8420415	5.9225574	NM_006814	chr20:1115812-1115871	PSMF1
A_33_P3224371	7.854946	7.6565747	NM_001167989	chrX:48759734-48759793	PQBP1
A_33_P3272520	5.3401675	5.6137104		chr3:097957869-097957928	
A_23_P155441	7.8698473	7.807365	NM_052859	chr3:53126436-53126079	RFT1
A_33_P3806676	9.294467	9.732294	AY751906	chr7:142428912-142428971	
A_33_P3366859	3.6567633	4.031328	NM_001187	chr21:11058221-11058162	BAGE

A_32_P187617	5.4710045	5.119916	NM_030794	chr13:61103254-61103313	TDRD3
A_33_P3286278	11.993554	12.190776	NM_002087	chr17:42430410-42430469	GRN
A_23_P56328	4.41903	4.292639	NM_031310	chr19:17462701-17462642	PLVAP
A_33_P3314121	3.9462295	3.315835		chrY:027567022-027566963	
A_33_P3397935	3.823589	3.647695	AL831877	chr10:5558217-5558158	CALML3-AS1
A_23_P20248	7.3164606	7.7269444	NM_002755	chr15:66783224-66783283	MAP2K1
A_33_P3306397	9.699213	9.313752	NM_004804	chr2:96939819-96939878	CIAO1
A_23_P319617	8.315836	8.395071	NM_019886	chrX:46457408-46457467	CHST7
A_23_P252913	5.434821	5.503987	NM_018190	chr4:122749634-122749575	BBS7
A_23_P19479	5.0226364	5.517792	NM_032454	chr6:31948299-31948463	STK19
A_23_P47704	4.7173386	4.53368	NM_003355	chr11:73686141-73686082	UCP2
A_23_P74895	3.7794213	3.8137317	NM_024674	chr1:26755699-26755758	LIN28A
A_33_P3225983	8.67137	8.590418		chr2:096548086-096548027	
A_23_P77776	11.496664	11.590036	NM_003016	chr17:74730660-74730601	SRSF2
A_24_P366566	6.382735	6.667677	NM_001005338	chr3:97852262-97852321	OR5H1
A_33_P3337318	3.8614836	3.7219412	BX641084	chr7:26241033-26240974	HNRNPA2B1
A_24_P247273	5.5178995	6.0454254		chr11:1910450-1910391	
A_24_P418189	11.3123255	11.598512		chr11:057344201-057344142	
A_23_P142631	5.1140823	5.4965096	NM_054033	chr2:24286286-24286345	FKBP1B
A_33_P3679876	5.113686	4.6939883	NR_038915	chr5:176864950-176864891	PRR7-AS1
A_33_P3227041	10.551152	10.492203	NM_197966	chr22:18218324-18218265	BID
A_32_P177024	7.9127927	7.8982434	NM_016038	chr7:66453156-66453097	SBDS
A_24_P199500	8.158335	7.977173	NM_007212	chr1:185071124-185071183	RNF2
A_33_P3423700	7.6529865	7.358249	AK124002	chr4:102227201-102227142	LOC100131829
A_23_P62920	7.9471188	7.8364506	NM_014970	chr1:169890773-169890714	KIFAP3
A_24_P169073	11.511668	11.923009	NM_182623	chr1:16384334-16384275	FAM131C
A_33_P3232532	4.799622	4.5149117	NM_025191	chr1:184663261-184663202	EDEM3
A_23_P97853	7.2699575	7.392914	NM_025125	chr10:81852040-81852099	TMEM254
A_23_P396777	6.2289433	6.220334	NM_006315	chr4:763321-763380	PCGF3
A_24_P58054	6.6467724	6.515606	NM_015266	chr20:48508374-48508433	SLC9A8
A_24_P10657	8.488365	8.778706	NM_020428	chr19:10754558-10754617	SLC44A2
A_23_P37623	7.4154487	7.8103285	NM_181077	chr15:34672224-34672165	GOLGA8A
A_24_P84048	11.084861	11.208088		chr12:062415821-062415761	
A_23_P45940	10.495914	10.389425	NM_022366	chr1:246704359-246704300	TFB2M
A_23_P164089	10.217921	10.342867	NM_001017368	chr17:33336270-33336211	RFFL
A_33_P3406686	4.8347287	5.5936413	NR_033935	chr2:74593548-74593489	DCTN1
A_33_P3385062	4.7274194	4.177399	NM_024511	chr4:2230469-2230410	HAUS3
A_23_P256526	3.5056396	3.399745	NM_007136	chr3:113954999-113954940	ZNF80
A_23_P309967	6.897311	6.7086573	NM_032486	chr16:23680321-23680380	DCTN5
A_23_P48713	5.552109	5.9062195	NM_152444	chr14:74349133-74350854	PTGR2
A_33_P3556839	3.613526	3.382067	NR_104161	chr11:68914756-68914697	LOC338694
A_33_P3300867	5.567201	5.627474	AK124637	chr4:57289534-57289593	LOC100506514
A_33_P3255914	8.075097	8.496976	NM_013262	chr6:16148409-16148468	MYLIP
A_24_P230057	8.3173485	7.847164		chrX:106672353-106672412	
A_32_P110243	15.523151	15.572144	BC071734	chr11:77524397-77524338	RPS20P27
A_24_P366165	13.962357	13.958832		chr1:165788611-165788670	
A_24_P272548	13.351572	13.279962		chr7:102395663-102395722	FAM185A
A_33_P3394183	7.5883737	7.154015	NM_207395	chr19:58969098-58969157	ZNF324B
A_23_P404134	7.4388566	7.524068	NM_014828	chr14:21966938-21966997	TOX4
A_33_P3347976	7.0707483	6.54191	NM_020774	chr18:19450664-19450723	MIB1
A_24_P48403	9.057381	9.280619	NM_005433	chr18:722336-722277	YES1
A_33_P3422294	11.477276	11.502492		chr5:106530986-106531045	
A_24_P413884	9.529115	9.389446	NM_001809	chr2:27016914-27016973	CENPA
A_33_P3262069	5.895414	6.3413944	NM_001004490	chr11:6789602-6789543	OR2AG2
A_33_P3354975	5.7448006	6.253949	NR_026850	chr1:32697727-32697668	MTMR9LP
A_23_P59950	10.796339	10.561575	NM_015359	chr8:22279836-22279895	SLC39A14
A_33_P3330468	7.4862456	7.2404776	NM_001171080	chrX:106307819-106307760	RBM41
A_23_P259594	6.0040007	6.0462394	NM_016377	chr6:131604361-131604420	AKAP7
A_23_P205031	12.070162	12.254968	NM_001846	chr13:111165261-111165320	COL4A2

A_33_P3296567	7.802153	8.138827	NM_013318	chr9:134343084-134346175	PRRC2B
A_24_P409494	5.4501214	5.2387943	NM_015367	chr22:18138526-18138585	BCL2L13
A_32_P106194	5.8869658	5.5436916	NM_153229	chr17:48358765-48358824	TMEM92
A_33_P3241937	4.9621105	5.214887	NM_019020	chr17:77914006-77913947	TBC1D16
A_23_P67198	5.0016346	5.2087584	NM_015692	chr19:17003871-17003812	CPAMD8
A_24_P171058	8.312256	8.878142	NM_001008495	chr8:91636507-91636448	TMEM64
A_32_P8361	7.693696	7.710408	NM_015329	chr19:19468679-19468738	MAU2
A_24_P222997	6.423975	5.9472656	NM_032143	chr2:135957983-135957924	ZRANB3
A_23_P37545	9.332787	9.082052	NM_024666	chr15:67494168-67494109	AAGAB
A_32_P49844	9.430641	9.494283	NM_012249	chr2:46803379-46803747	RHOQ
A_33_P3780286	5.380128	5.674665		chr13:107030882-107030941	LINC00460
A_33_P3306163	7.5431604	7.743501	NM_001177388	chr14:55605026-55605085	LGALS3
A_33_P3386835	6.2589293	6.326584	XR_171115	chr8:42390012-42389953	SLC20A2
A_23_P112774	5.3308	4.9032574	NM_032611	chr8:142441433-142441492	PTP4A3
A_33_P3293798	5.9673023	6.2039433	NM_198691	chr21:45959580-45959521	KRTAP10-1
A_24_P86993	2.3221061	2.3900566	NM_032801	chr11:134020792-134020851	JAM3
A_23_P96761	9.219402	9.409898	NM_000016	chr1:76227040-76228420	ACADM
A_33_P3299047	5.418895	5.48393		chr1:016569360-016569301	
A_23_P201567	7.5140605	7.3499055	NM_018061	chr1:109243867-109243926	PRPF38B
A_24_P229871	6.639808	6.7250667	BC101214	chr17:71746683-71746624	LINC00469
A_33_P3388771	2.6123	2.3900566	XR_172438		LOC101060810
A_33_P3321507	10.412537	10.698758	NM_001080495	chr7:5352703-5352644	TNRC18
A_33_P3590673	8.540957	8.72921	NR_045196	chr5:9550336-9550395	SNHG18
A_24_P33895	2.4308386	2.3900566	NM_001040619	chr1:212792837-212792896	ATF3
A_33_P3329984	7.674554	7.714539	NM_152657	chr19:38875093-38875034	GGN
A_33_P3385656	5.566322	6.172781	NM_001001343	chr5:156768667-156768608	FNDC9
A_32_P158786	2.8437681	2.3900566	NM_001170633	chr12:64668692-64664447	C12orf56
A_32_P30831	3.8538694	4.3816524		chr15:40986615-40986556	RAD51-AS1
A_23_P200199	7.971266	7.9145374	NM_015913	chr1:52489194-52486657	TXNDC12
A_33_P3357332	4.9786463	4.6801887	XR_171816	chr9:115246889-115246830	
A_23_P427296	3.5905018	3.904319	AF315716	chr3:188868690-188868631	LOC100132319
A_24_P316005	4.380679	5.151685	NM_014857	chr1:174340157-174363138	RABGAP1L
A_33_P3342285	10.836863	10.784255	NM_001010935	chr1:112255449-112255508	RAP1A
A_23_P129246	6.319057	6.341958	NM_025201	chr15:65159956-65160015	PLEKHO2
A_24_P83586	6.329244	6.55664	NM_015506	chr1:45976403-45976462	MMACHC
A_24_P107336	8.633041	8.7988825	NM_052875	chr11:134117288-134117347	VPS26B
A_33_P3223923	7.2456837	7.24439	NM_005313	chr15:44064742-44064801	PDIA3
A_23_P143551	7.6120987	7.5757	NM_001696	chr22:18081053-18080994	ATP6V1E1
A_33_P3388192	2.9865184	2.3900566	NM_144594	chr12:54849804-54849745	GTSF1
A_33_P3348151	5.489956	5.7636514		chr9:043848640-043848699	
A_33_P3354728	9.434757	9.367818		chr14:065735706-065735765	
A_24_P350245	5.649455	5.3748417	NM_024940	chr8:25101212-25101271	DOCK5
A_23_P300484	6.9870863	7.196001	NM_001173408	chr2:220428135-220427396	OBSL1
A_24_P283341	7.453476	7.5595145	NM_022765	chr6:109766194-109766135	MICAL1
A_23_P91076	6.5320544	5.85527	NM_032824	chr2:112875989-112876048	TMEM87B
A_23_P307400	9.385128	9.501452	NM_138363	chr17:62533931-62533990	CEP95
A_23_P303203	7.4467325	7.3094645	NM_152272	chr8:23118881-23118940	CHMP7
A_33_P3278540	9.92691	9.6556225	NM_032111	chr6:44081434-44081375	MRPL14
A_23_P69877	7.6172748	7.508556	NM_152283	chr5:180274691-180274632	ZFP62
A_33_P3257627	3.8262012	4.1141033		chrY:25537642-25537701	XLOC_014512
A_23_P362659	6.2332783	6.313588	NM_002468	chr3:38183932-38183991	MYD88
A_24_P294719	10.414299	10.573208	NM_014613	chr5:175936715-175936774	FAF2
A_33_P3257866	4.200978	4.5672026	NR_003064	chr17:26554452-26554511	PYY2
A_23_P42331	9.033585	8.414936	NM_145901	chr6:34213595-34213654	HMGA1
A_23_P147277	8.086481	8.42725	NM_207346	chr17:73520538-73520597	TSEN54
A_24_P210829	8.195724	8.428577	NM_005009	chr16:449689-450227	NME4
A_24_P256692	6.065276	6.3789616	NM_198551	chr1:222840877-222840936	MIA3
A_33_P3392977	7.6185746	7.7510076	NM_012142	chr15:43486980-43487039	CCNDBP1
A_23_P319792	8.450336	8.275896	NM_019001	chr3:142025671-142025612	XRN1

A_23_P337726	11.70069	11.774466	AK131288	chrM:8836-8895	ATP6
A_24_P1773	7.328373	7.1725574	NM_031490	chr16:48386989-48387048	LONP2
A_33_P3277378	3.5231926	4.0426054		chr22:030661669-030661610	
A_32_P94722	6.915813	6.4315	NM_052893	chr6:38136318-38136259	BTBD9
A_33_P3286576	2.676274	2.3900566		chr9:090583727-090583786	
A_23_P92154	8.82098	9.42421	NM_003925	chr3:129150389-129150330	MBD4
A_24_P358084	5.543907	5.480954	XM_005276515	chr16:12027706-12027484	
A_32_P176911	4.3216734	4.2082415	NR_026774	chr14:102198716-102198775	LINC00239
A_23_P67932	3.6139596	4.176432	NM_000634	chr2:219027904-219027845	CXCR1
A_33_P3250356	4.151778	4.6866617	NM_001162530	chr1:36773712-36773771	SH3D21
A_33_P3421695	4.7194805	4.581826	NM_001098210	chr3:41280687-41280746	CTNNB1
A_23_P166779	3.2275832	3.3960783	NR_024065	chr3:8615322-8615381	LINC00312
A_33_P3352664	5.2783527	5.739076	NM_001173467	chr12:53722172-53722113	SP7
A_33_P3352873	5.05534	5.9870872	NM_000056	chr6:80878596-80878655	BCKDHB
A_23_P41066	7.338168	7.686364	NM_170713	chr3:50367379-50367320	RASSF1
A_33_P3326432	7.0542707	6.869364	NM_003009	chr19:48284568-48284627	SEPW1
A_33_P3277060	4.369185	4.785796		chr17:018324995-018325054	
A_33_P3338928	7.847496	7.6512365	NM_001343	chr5:39371884-39371825	DAB2
A_33_P3222348	4.4807568	4.7358894	AK124642	chr2:31460949-31460890	
A_33_P3292794	3.945577	3.7856483	NM_014311	chr12:54575939-54575880	SMUG1
A_24_P115774	9.794039	9.362156	NM_001166	chr11:102248391-102248450	BIRC2
A_33_P3220406	4.286827	3.05001		chr18:046183929-046183870	
A_33_P3235340	11.040688	10.505079	NM_006773	chr2:118589873-118589932	DDX18
A_24_P307626	5.273772	5.044646	NM_144699	chr1:160143406-160143465	ATP1A4
A_23_P46819	6.380532	6.5022697	NM_033637	chr10:103316590-103316649	BTRC
A_33_P3409245	6.9616833	7.049419	XR_110199	chr6:168067630-168067571	LOC401286
A_33_P3369262	7.0075693	6.9742336	NM_015633	chr12:27117547-27117606	FGFR1OP2
A_33_P3410449	5.5198746	4.811657	NM_153334	chr22:20779338-20779279	SCARF2
A_33_P3299170	9.663436	9.504021	NM_201412	chr16:239053-238994	LUC7L
A_23_P373708	8.907997	8.425108	NR_028334	chr17:26603822-26603763	KRT18P55
A_33_P3387145	7.020099	6.958363	NM_031892	chrX:19702001-19701942	SH3KBP1
A_24_P100234	6.1747637	6.206709	NM_014941	chr22:31328377-31324167	MORC2
A_33_P3390778	7.165641	7.0564175	NM_001256599	chr1:155157386-155157445	TRIM46
A_23_P371765	5.2026043	5.2478275	NM_032261	chr21:47581854-47581585	SPATC1L
A_24_P255845	3.5053272	4.7747927		chr12:016552865-016552804	
A_23_P56922	14.299968	13.992674	NM_002157	chr2:198367803-198367942	HSPE1
A_33_P3212615	10.449107	10.437774	NM_006287	chr2:188329067-188329008	TFPI
A_24_P98524	8.4259205	8.748796	NM_021132	chr10:75196815-75196756	PPP3CB
A_24_P303145	4.305149	3.8207862	NM_054027	chr5:14705088-14705029	ANKH
A_23_P205724	3.771936	3.7982254	NM_017970	chr14:90754761-90754702	NRDE2
A_33_P3367201	5.2281847	4.7659097	NM_052845	chr12:109991620-109991561	MMAB
A_24_P295963	11.458673	11.737551	NM_018976	chr12:46752769-46752710	SLC38A2
A_33_P3268618	8.932896	9.379128	NM_025132	chr4:39287306-39287365	WDR19
A_23_P149206	7.2237763	7.2985163	NM_003780	chr1:44456570-44456629	B4GALT2
A_33_P3253832	10.923334	11.2437935		chr9:131199583-131199642	
A_33_P3209406	3.7103114	3.074744	NM_001270440	chr19:12937218-12937159	RTBDN
A_23_P30813	7.3187623	7.4803042	NM_003541	chr6:27799013-27798954	HIST1H4K
A_33_P3292812	6.2696905	6.4261446	NM_017972	chr14:76638302-76638361	GPATCH2L
A_23_P96350	8.502892	8.681557	NM_007213	chrX:48928960-48928901	PRAF2
A_24_P174503	3.313538	3.6757245	NM_000481	chr3:49455118-49455059	AMT
A_32_P168464	9.585976	9.271452	NM_003688	chrX:41374317-41374258	CASK
A_23_P94683	6.861288	6.932765	NM_002504	chr9:33370705-33370764	NFX1
A_33_P3224483	9.99128	10.120106	BC020495	chr22:51210086-51210027	RABL2B
A_23_P138725	11.35618	11.032312		chr10:99477819-99477878	MARVELD1
A_33_P3325671	3.4275694	4.540754	XM_003403421	chr3:128634045-128633986	LOC100132731
A_24_P303524	9.002075	8.929023	NM_182924	chr7:1474074-1474015	MICALL2
A_24_P118247	5.7446637	5.8981957	NM_001012762	chr16:88780105-88780164	CTU2
A_23_P82128	15.127534	15.26942	NM_001016	chr6:133138639-133138698	RPS12
A_23_P42802	9.725924	9.802197	NM_004911	chr7:148700963-148700904	PDIA4

A_24_P257971	4.813049	4.465007	NM_005094	chr9:131122624-131122683	SLC27A4
A_23_P401472	2.4378006	2.3900566	NM_000740	chr1:240071981-240072040	CHRM3
A_33_P3275801	5.288814	5.2548304	NM_001927	chr2:220291400-220291459	DES
A_33_P3301915	3.958335	3.6237063	NM_201286	chrX:55513049-55512990	USP51
A_33_P3392325	10.8890705	10.767704	NM_001078645	chr13:115038074-115038133	CDC16
A_32_P18440	6.9413056	7.375324	NM_032199	chr10:63856246-63856305	ARID5B
A_24_P162173	2.7377765	2.3900566	NM_001149	chr10:61843293-61842480	ANK3
A_33_P3245238	7.0825806	6.205929	NM_176825	chr2:108926155-108926214	SULT1C2
A_23_P48585	4.0661306	4.344193	NM_005407	chr14:21989302-21989243	SALL2
A_33_P3321796	6.111216	6.2191806	DB153536	chr11:3166134-3166075	
A_33_P3303748	3.6194806	3.5624413	NM_017990	chr16:70170101-70170160	PDPR
A_33_P3298349	4.701006	4.9654875	NM_053017	chr11:3661305-3661246	ART5
A_33_P3281036	11.158665	10.934387	NR_026790	chr6:26527499-26527558	HCG11
A_23_P56673	3.8902595	4.118147	NM_031288	chr2:74682703-74682981	INO80B
A_23_P200535	8.602188	8.761955	NM_014408	chr1:36603463-36603404	TRAPPC3
A_33_P3275412	3.739795	3.9437015	AK127688	chr1:150532941-150532882	ADAMTSL4-AS1
A_24_P943358	4.472822	4.5795975	NM_006716	chr7:87538554-87538613	DBF4
A_33_P3305531	5.2290497	5.218555	NM_001113528	chr11:28135091-28135150	METTL15
A_23_P27424	6.652691	6.8425665	NM_133460	chr19:58433735-58433676	ZNF418
A_33_P3307965	7.5791	7.5445395	NM_001103170	chr1:12788667-12788726	AADACL3
A_33_P3416946	5.4857993	5.6535378	NM_018477	chr14:58702246-58702305	ACTR10
A_33_P3340624	8.318221	8.530966		chr3:038556995-038556936	
A_23_P21734	9.336777	8.939609	NM_001015891	chr5:68651567-68651508	AK6
A_23_P16469	6.492976	6.1022253	NM_001005377	chr19:44169557-44169498	PLAUR
A_33_P3331376	3.9557562	3.8804078	NM_004442	chr1:23240304-23240363	EPHB2
A_33_P3224249	3.6883402	3.8146625	NR_003594	chr8:86775439-86775380	REXO1L2P
A_23_P169178	9.473712	8.967831	NM_006285	chr9:35609924-35609983	TESK1
A_23_P28652	9.928509	10.243943	NM_016085	chr2:27438559-27438618	ATRAID
A_24_P398898	13.122759	13.538105	NM_206839	chr15:79189673-79189732	MORF4L1
A_24_P63262	7.756876	8.145767	NM_002950	chr3:128341192-128341133	RPN1
A_33_P3325006	10.480377	10.327965	NM_005706	chr11:2425044-2425103	TSSC4
A_24_P218979	9.740197	10.037647	NM_031299	chr12:6958498-6958315	CDCA3
A_33_P3383724	5.42677	5.4301987	NM_001012414	chr4:165890823-165890764	TRIM61
A_33_P3301381	12.646112	12.720637	NM_198557	chr2:152106227-152106168	RBM43
A_33_P3302390	5.2297735	5.8918805		chr21:015137006-015137065	
A_33_P3334630	3.446045	3.224503	NM_001128834	chrX:103047357-103047416	PLP1
A_23_P127663	6.5252943	6.6032066	NM_024081	chr11:32875016-32875075	PRRG4
A_23_P43141	13.503417	13.413709	NM_001568	chr8:109228662-109226908	EIF3E
A_33_P3410724	11.925346	12.079157	NM_003001	chr1:161332887-161332946	SDHC
A_33_P3333527	9.6959715	9.403549	NM_018116	chr1:155584673-155584732	MSTO1
A_33_P3280451	3.47884	4.3954296	CU675766	chr6:126080947-126080889	
A_23_P33407	7.2803726	7.366257	NM_004667	chr15:28356983-28356924	HERC2
A_24_P73075	6.5130186	6.654489	NM_017868	chr11:113234558-113234617	TTC12
A_33_P3312885	3.9982526	3.7401774		chr19:54106740-54106681	LOC284379
A_33_P3232458	7.8912153	8.036326	NM_024692	chr2:29404713-29404772	CLIP4
A_33_P3264272	5.2305193	5.384243	NM_015484	chr1:25549021-25548962	SYF2
A_33_P3679901	7.4209137	7.5230346	X64982	chr7:141587554-141587613	
A_33_P3268686	4.479124	4.189258	NM_207102	chr3:48419846-48419905	FBXW12
A_33_P3223208	6.419123	6.400327	NM_016122	chr12:94763789-94763730	CCDC41
A_33_P3289406	4.278018	3.32563	NR_026998	chr15:78285654-78285595	LOC91450
A_23_P111005	9.502087	10.004974	NM_015388	chr6:43479845-43479786	YIPF3
A_33_P3396089	6.602585	6.57842	NM_153811	chr14:61519117-61519176	SLC38A6
A_33_P3416588	4.112061	4.240226	NM_002930	chr18:40503614-40503555	RIT2
A_33_P3246613	8.444273	8.56955	NM_001031737	chr16:772645-772586	CCDC78
A_23_P82693	15.910685	15.8631735	NM_002568	chr8:101716541-101715545	PABPC1
A_33_P3355762	5.155225	4.9392934	XM_005262789	chr4:90033460-90033519	
A_23_P217968	5.774815	5.5342107	NM_016028	chr11:67934074-67934015	SUV420H1
A_33_P3283061	8.813844	9.0832405	AK128128	chr16:33347435-33347376	
A_33_P3350074	8.941634	8.452953	NM_001126121	chr17:73269133-73269074	SLC25A19

A_33_P3267948	5.506155	5.3720565	NM_024103	chr19:6454394-6454335	SLC25A23
A_32_P220715	10.060962	10.070319	NM_022818	chr16:87436758-87436817	MAP1LC3B
A_32_P122754	10.636689	10.710045	NM_080655	chr9:103213052-103213110	MSANTD3
A_23_P5389	9.68858	9.756641	NM_032574	chr2:32254724-32254665	DPY30
A_33_P3214446	6.731683	6.378084	NM_181558	chr13:34409294-34409353	RFC3
A_24_P140391	10.334401	10.097204	NM_001042616	chr4:89443107-89443048	PIGY
A_32_P194962	8.643208	8.65471	BE300169	chr19:49993745-49993510	
A_33_P3620832	4.3625903	4.131294	NM_001007169	chr9:114290027-114290086	ZNF483
A_32_P196142	8.135785	7.7006207	XR_110148	chr18:59273374-59273315	LOC100130938
A_32_P70818	5.023176	4.8257947	NM_006194	chr14:37146621-37146680	PAX9
A_33_P3210969	5.770295	5.528891		chr7:067855975-067855916	
A_23_P165180	9.877642	10.01922	NM_003721	chr19:19310021-19312494	RFXANK
A_23_P77048	8.06064	8.142032	NM_001039355	chr14:100757547-100757488	SLC25A29
A_32_P182394	3.4496841	3.786128	NM_021217	chr19:2934392-2934333	ZNF77
A_23_P435697	8.541426	8.40663	NM_015608	chr10:127452315-127452374	C10orf137
A_24_P92256	6.1886425	6.641115	NM_014853	chr17:2283828-2283887	SGSM2
A_33_P3360227	4.5290084	4.50552	DC397705	chr6:111195730-111195789	
A_33_P3422679	8.305651	8.354477	NM_173348	chr10:75001846-75001905	FAM149B1
A_23_P34546	7.277226	7.034176	NM_006642	chr1:243589784-243589843	SDCCAG8
A_23_P389500	3.819062	4.192582	NM_006507	chr2:79312234-79312175	REG1B
A_32_P204205	6.75226	6.0487638	NM_017420	chr14:61176578-61176519	SIX4
A_23_P68610	9.785504	10.262342	NM_012112	chr20:30388973-30389032	TPX2
A_33_P3223106	4.5108314	4.4660354	NR_034163	chr7:5176246-5176187	ZNF890P
A_23_P251499	3.9782984	3.8420076	NM_002593	chr7:100204067-100204126	PCOLCE
A_24_P144499	14.063696	13.490389	NM_178230	chr1:147954874-147954815	PPIAL4A
A_33_P3328365	6.002944	6.128632	BF355127	chr21:45930829-45930888	TSPEAR-AS1
A_23_P347468	6.5057845	5.8650413	NM_017412	chr8:28421786-28421845	FZD3
A_23_P25706	4.8238697	4.3129134	NM_024734	chr14:95660925-95660250	CLMN
A_23_P368126	12.185336	12.335812	NM_006621	chr1:110564765-110564824	AHCYL1
A_23_P9565	4.8122725	4.9624214	NM_031296	chr4:140396778-140396837	RAB33B
A_33_P3440636	5.6926475	5.256281	AK091759	chr8:052861772-052861831	LOC286071
A_23_P341325	13.826841	13.979262	NM_080746	chr14:47120573-47120514	RPL10L
A_33_P3267612	5.5933204	5.486904	NR_027180	chr5:148803866-148803925	MIR143HG
A_23_P41344	5.9248247	4.803259	NM_001432	chr4:75254009-75254068	EREG
A_33_P3783812	6.3801527	6.38098	AK022255	chr8:142182820-142182879	C8orf60
A_23_P97892	4.969217	5.1692405	NM_003893	chr10:103869399-103869248	LDB1
A_33_P3369914	4.2011547	4.7333784	BC012174	chr1:206702714-206702655	
A_23_P127995	11.85593	11.670954	NM_001293	chr11:77327395-77327336	CLNS1A
A_23_P77590	7.412111	7.5110993	NM_018052	chr16:70721600-70721541	VAC14
A_23_P67391	5.499357	5.386597	NM_007059	chr19:47978420-47978400	KPTN
A_23_P89589	7.5691357	7.66892	NM_002616	chr17:8044326-8044267	PER1
A_24_P260101	2.3221061	3.2270918	NM_007289	chr3:154900750-154900809	MME
A_23_P39718	8.668467	8.639809	NM_001042548	chr2:36779763-36779704	FEZ2
A_23_P16562	8.409205	8.291554	NM_001017392	chr19:19103619-19103560	SUGP2
A_23_P355067	11.023692	11.230084	NM_019026	chr1:165697260-165697201	TMCO1
A_23_P135219	4.092436	3.5751357	NM_005294	chr9:125797459-125797518	GPR21
A_23_P67432	4.787405	5.1353602	NM_152478	chr19:56935345-56935404	ZNF583
A_33_P3258946	4.568549	4.49774	NM_001193286	chr19:39379793-39379734	SIRT2
A_33_P3222892	8.3312845	8.416481	NM_019848	chrX:153715750-153715691	SLC10A3
A_33_P3330125	6.410648	6.28635	NM_001278302	chr12:122710882-122710823	DIABLO
A_33_P3388641	4.415555	4.2494764		chrX:41135972-41136031	
A_23_P391764	4.4185343	4.2916856	NM_198679	chr9:134454649-134454590	RAPGEF1
A_33_P3772996	7.5368094	7.0373945	NM_002731	chr1:84702761-84702820	PRKACB
A_23_P356122	5.850315	6.4517684	NM_001031623	chr6:57033933-57033992	ZNF451
A_23_P379034	10.290577	10.222691	NM_025045	chr22:38480966-38480907	BAIAP2L2
A_23_P111452	10.101191	9.928509	NM_031946	chr7:150841434-150841493	AGAP3
A_23_P18598	7.930737	8.419403	NM_018323	chr4:25279987-25280046	PI4K2B
A_24_P228611	10.535272	10.549927	XR_248924	chr20:34190945-34190628	
A_33_P3433873	9.169143	8.621838	NM_017975	chr15:66841152-66841211	ZWILCH

A_33_P3398946	4.356299	4.326154	NM_015649	chr19:46387337-46387278	IRF2BP1
A_33_P3364038	3.0487838	3.4448693	NM_001145805	chr5:150228172-150228231	IRGM
A_23_P403886	3.9910746	3.2442276	NM_005838	chr11:58477744-58477685	GLYAT
A_33_P3359713	4.2810683	4.2897525	CK825926	chr7:65226732-65226791	
A_23_P28625	10.353097	10.293912	NM_018256	chr2:203745644-203745585	WDR12
A_33_P3385006	3.6050181	3.7727985	NM_001135195	chr12:56631568-56631627	SLC39A5
A_33_P3382493	5.770011	6.18791	NM_001098612	chr19:52146915-52146856	SIGLEC14
A_23_P92842	8.521257	8.794614	NM_001033503	chr5:133942405-133942346	SAR1B
A_33_P3377619	8.223712	7.332962	NM_153810	chr10:120454713-120454654	CACUL1
A_23_P252681	7.22185	7.578952	NM_005017	chr3:195968959-195968900	PCYT1A
A_33_P3221313	6.2302947	6.444951	NM_006733	chrX:100417917-100417976	CENPI
A_32_P135091	7.815302	7.953775	NM_018301	chrX:106310464-106310405	RBM41
A_33_P3417620	4.7749853	4.4835496	BC065719	chrX:15819786-15819845	ZRSR2
A_24_P329597	4.1303205	3.9334295	NM_013438	chr9:86275724-86275665	UBQLN1
A_23_P138805	10.773226	10.591017	NM_012124	chr11:89935104-89935045	CHORDC1
A_33_P3291454	6.9459877	7.011063	NM_198515	chr10:118139356-118139415	CCDC172
A_23_P70571	6.514957	5.744576	NM_006979	chr6:33171800-33171859	SLC39A7
A_33_P3227264	9.388187	9.430051	NM_024011	chr1:1634268-1634209	CDK11A
A_33_P3363310	5.831295	5.6620345		chr11:71279921-71279980	KRTAP5-10
A_33_P3383626	4.5425572	4.042116	XM_003403711	chr19:56758807-56758863	ZSCAN5D
A_33_P3246763	3.6119857	4.2040477	NM_001166579	chr17:74466138-74466197	AANAT
A_23_P330908	8.100261	8.506191	NM_024295	chr8:124026717-124026658	DERL1
A_23_P71419	11.621851	11.420245	NM_006837	chr8:67963495-67958167	COPS5
A_23_P78835	5.6173534	5.5553613	NM_001002836	chr19:56598993-56598934	ZNF787
A_24_P356373	4.5897827	4.7597075	NM_032304	chr16:778356-778415	HAGHL
A_23_P140427	8.275514	8.382174	NM_016337	chr14:100610330-100610389	EVL
A_32_P231493	3.3062353	3.5630393	NR_047482	chr13:99740791-99740850	DOCK9-AS2
A_33_P3316978	6.4981794	6.8748817	NM_207396	chr1:6281243-6281302	RNF207
A_23_P116235	11.308544	11.649141	NM_001012334	chr11:46405231-46405290	MDK
A_33_P3372368	7.0730677	7.1529408	NR_045962	chr12:53343300-53343241	KRT8
A_24_P321626	6.8434644	6.7893896	NM_018082	chr12:106897898-106897957	POLR3B
A_23_P393025	3.7937481	2.3900566	NM_144644	chr4:177105963-177105904	SPATA4
A_23_P200838	6.9582586	7.2870216	NM_003679	chr1:241758694-241758753	KMO
A_33_P3317948	3.726701	2.3900566	NR_033711	chr1:3652607-3652548	TP73-AS1
A_23_P371787	8.356344	8.369179	NM_014734	chr14:70181578-70181637	KIAA0247
A_24_P276102	4.6521897	4.6697783	NM_183404	chr20:35635888-35635829	RBL1
A_33_P3375556	4.451049	4.339959		chr9:068749912-068749971	
A_23_P408094	3.8416853	3.941641	NM_002357	chr2:70142526-70143302	MXD1
A_32_P103291	8.96821	8.871408	NM_022743	chr1:245912856-245912797	SMYD3
A_33_P3251557	5.0947948	5.0995274		chr10:077158263-077158322	
A_33_P3777207	3.7764225	3.4375718	NM_033467	chr1:2524332-2524273	MMEL1
A_33_P3346403	15.738789	15.738789	NM_001099285	chr2:232577214-232577560	PTMA
A_23_P128817	5.4035807	4.913327	NM_004563	chr14:24569401-24571999	PCK2
A_33_P3337026	4.1829467	4.7029185	U17986	chrX:152959440-152959499	SLC6A8
A_33_P3266520	5.1829166	5.5076356	NM_152988	chr19:2339905-2339964	SPPL2B
A_33_P3366146	10.676505	11.004181	NM_001039465	chr14:70238119-70238178	SRSF5
A_24_P345209	8.007641	8.175134	NM_001004023	chr1:206822221-206822280	DYRK3
A_23_P93844	12.214335	12.147322	NM_019059	chr7:22852769-22852710	TOMM7
A_33_P3329344	10.779886	10.886576	NM_004104	chr17:80036275-80036216	FASN
A_33_P3316078	3.9716053	4.158456	NM_001039938	chr13:52023692-52023633	INTS6
A_33_P3304983	6.343542	6.3623505	NM_002736	chr7:106781339-106781398	PRKAR2B
A_33_P3278789	4.817397	5.075404	NM_001135106	chr6:39285680-39285621	KCNK16
A_33_P3295814	5.6620345	5.8601484		chr7:073245261-073245202	
A_33_P3289536	6.6434855	7.136556	NM_001199835	chr7:26412152-26412211	SNX10
A_23_P216080	6.165842	5.601343	NM_032410	chr8:42874160-42874219	HOOK3
A_33_P3273068	6.0735793	5.7553544	AB058691	chr11:44282055-44281996	ALX4
A_24_P365753	4.7558146	3.7453957	NR_027131	chrX:119378826-119378767	NKAPP1
A_32_P49423	14.424332	14.311911	NM_001037738	chr5:170832324-170832382	NPM1
A_23_P65967	7.655101	8.143114	NM_018231	chr16:58700427-58700368	SLC38A7

A_33_P3405921	8.669269	8.714224	NM_001286205	chr16:90111270-90111329	GAS8
A_23_P60517	7.8090315	7.8127027	NM_181425	chr9:71687853-71687912	FXN
A_24_P327011	5.111722	4.625038	NM_021823	chr15:75342483-75342542	PPCDC
A_33_P3247095	3.965974	4.112914		chr11:107643195-107643254	
A_23_P71300	7.4587703	7.486617	NM_018246	chr8:27591203-27591144	CCDC25
A_33_P3287195	4.4096317	4.3523912	AY129018	chr12:118478209-118478268	
A_23_P71241	10.78867	10.919557	NM_014302	chr7:54823522-54820105	SEC61G
A_33_P3289222	8.508735	8.5473795	NM_006369	chr1:46744468-46744409	LRRC41
A_24_P142024	9.609042	9.800266	NM_014169	chr14:24679621-24679562	CHMP4A
A_23_P82674	8.327671	8.524973	NM_001483	chr7:56067247-56067306	GBAS
A_24_P225719	8.475251	8.288845	NM_015387	chr2:198417148-198417207	MOB4
A_33_P3544880	6.6889725	6.41562	BC008131	chr10:6214512-6214571	LOC142937
A_33_P3256095	8.207339	8.55882		chr1:222647359-222647300	XLOC_014512
A_23_P502470	5.6888685	6.0367107	NM_002184	chr5:55236787-55236728	IL6ST
A_23_P72568	9.350999	9.736049	NM_003794	chr3:125165892-125165833	SNX4
A_23_P88559	6.386279	6.460874	NM_000236	chr15:58861011-58861070	LIPC
A_33_P3289113	5.7293005	5.7792945	AK124809	chr17:53029524-53029465	COX11
A_23_P386450	8.617647	8.500457	NM_016218	chr5:74894691-74894750	POLK
A_23_P255884	9.1260805	9.514394	NM_198252	chr9:124094771-124094830	GSN
A_23_P51117	8.575386	8.447488	NM_019002	chr2:67637076-67637135	ETAA1
A_23_P163697	2.3221061	2.3900566	NM_016524	chr16:19236117-19278217	SYT17
A_33_P3344951	6.422513	6.550195	NM_001114632	chr15:42129270-42129329	JMJD7
A_33_P3306068	2.7019267	3.4991338	NM_001033081	chr1:40361179-40361120	MYCL
A_33_P3346498	4.356681	4.4158564	NM_130831	chr3:193333496-193333555	OPA1
A_23_P500799	8.56955	8.664146	NM_001226	chr4:110610704-110610645	CASP6
A_23_P154188	9.084924	9.053422	NM_024545	chr2:128699013-128698954	SAP130
A_24_P208045	8.8117485	8.269793	NM_025191	chr1:184660017-184659958	EDEM3
A_23_P152024	6.023051	5.8273983	NM_004383	chr15:75094365-75094424	CSK
A_23_P170857	6.9274693	6.4294653	NM_002182	chr3:190368968-190369027	IL1RAP
A_32_P18250	8.347288	8.417381	NR_003663	chr2:84518284-84518343	FUNDC2P2
A_24_P297078	6.2451625	6.2808065	NM_020531	chr20:24949557-24944629	APMAP
A_32_P204795	2.3221061	2.3900566	NR_036522	chr19:56989620-57005565	ZNF667-AS1
A_33_P3346302	6.6041737	6.7440586	NM_018449	chr9:33922469-33922410	UBAP2
A_33_P3230926	3.5757124	3.7138996	AK127488	chr2:46879630-46879689	LOC100130429
A_23_P163227	2.3221061	2.3900566	NM_001015001	chr15:43991282-43991341	CKMT1A
A_23_P82108	7.9169664	7.880416	NM_020861	chr6:151686180-151686121	ZBTB2
A_33_P3224135	3.9420872	3.2017844	XM_005273968	chr11:82909624-82909683	ANKRD42
A_33_P3423853	4.1232033	3.3903103	NR_033263	chr20:61145202-61145143	C20orf166-AS1
A_33_P3370751	2.5738075	2.3900566	NM_022739	chr17:62540794-62540735	SMURF2
A_24_P121846	4.1382265	4.0798497	NM_003433	chr19:58944877-58944818	ZNF132
A_33_P3318292	12.881577	12.638172	NM_005066	chr1:35649300-35649241	SFPQ
A_23_P216282	10.401059	10.357729	NM_014629	chr8:1906714-1906773	ARHGEF10
A_23_P207911	5.2693844	5.323369	NM_016113	chr17:16340226-16340285	TRPV2
A_23_P47199	4.396507	4.6276083	NM_018484	chr11:64336192-64337124	SLC22A11
A_24_P336137	3.9080071	3.42669	NM_032561	chr22:38340239-38340180	C22orf23
A_33_P3264444	5.557396	5.5264416		chr6:33265875-33265934	PFDN6
A_23_P153197	8.947336	9.659414	NM_170695	chr18:3458026-3458079	TGIF1
A_33_P3309919	10.225909	10.127692	NM_016104	chr6:116914289-116914348	RWDD1
A_24_P260440	9.008517	8.5928135	NM_002270	chr5:72209488-72209547	TNPO1
A_33_P3316045	10.856181	10.910885	NM_001080453	chr7:1509973-1509914	INTS1
A_33_P3379669	5.1134	5.217125	NM_031297	chr9:140115164-140115105	RNF208
A_23_P157914	6.6167817	6.243326	NM_153267	chr9:72833499-72840711	MAMDC2
A_23_P91697	5.059572	4.6257157	NM_004737	chr22:33669391-33669332	LARGE
A_23_P426636	6.3226027	6.4189086	NM_001620	chr11:62284928-62284869	AHNAK
A_32_P184279	2.3221061	2.3900566	NM_005436	chr10:61548670-61548611	CCDC6
A_23_P87329	8.052992	8.115882	NM_024662	chr11:34167990-34168049	NAT10
A_24_P136094	4.67688	4.3563113	NM_001080414	chr14:91739159-91739100	CCDC88C
A_23_P407142	4.609747	4.679923	NM_033631	chr1:23412495-23412436	LUZP1
A_33_P3264394	3.4875832	3.8703885	AK127311	chr17:79972217-79972276	ASPSCR1

A_33_P3345016	6.068247	6.677109	NM_000871	chr1:20005469-20005528	HTR6
A_33_P3333667	4.462595	4.2774878	NM_001270380	chr11:74617344-74617285	XRRA1
A_23_P3042	9.890113	9.625977	NM_006246	chr14:63841631-63841572	PPP2R5E
A_33_P3321689	5.6182275	5.92516		chr5:090734842-090734901	
A_33_P3281716	5.402022	5.5468464		chr2:133020552-133020610	
A_33_P3398634	10.596728	10.912276	NM_001271943	chr6:41658879-41658820	TFEB
A_23_P91114	7.5587153	7.5844994	NM_013388	chr2:27354097-27354038	PREB
A_23_P73114	7.9100685	8.123934	NM_000313	chr3:93592208-93592149	PROS1
A_33_P3332474	3.7538185	3.3275115	NM_001001413	chr15:22742452-22742511	GOLGA6L1
A_23_P303455	7.6957912	7.60707	NM_153832	chr1:168056852-168055005	GPR161
A_33_P3226955	6.486295	6.8581505		chr10:33538421-33538362	NRP1
A_33_P3378360	11.971525	12.0060215	NM_013237	chr5:176733605-176733664	PRELID1
A_33_P3266898	2.3466394	2.3900566	NM_001941	chr18:28576994-28576935	DSC3
A_23_P354734	9.660485	9.799498	NM_032088	chr5:140892394-140892453	PCDHGA8
A_24_P285768	5.800158	5.6984715	NM_014674	chr3:5260840-5260899	EDEM1
A_23_P85164	9.300844	9.617899	NM_006730	chrX:153630581-153630522	DNASE1L1
A_33_P3383866	8.34699	8.22102	NM_016381	chr3:48508975-48509034	TREX1
A_33_P3210975	6.858878	6.779884		chr6:066545771-066545712	
A_33_P3407835	4.1385226	3.851746	NM_144641	chr3:52283771-52283830	PPM1M
A_33_P3405789	9.351712	9.658953	NM_003324	chr12:3050116-3050175	TULP3
A_24_P25080	9.256479	9.284318	NM_001024916	chr9:70475060-70473457	CBWD5
A_33_P3313065	6.7320857	6.4902635	NM_001001923	chr9:125552062-125552121	OR5C1
A_33_P3340862	9.659721	9.869758	NM_001146685	chr1:1363048-1363107	TMEM88B
A_23_P74778	4.26165	2.3900566	NM_024579	chr1:150253251-150253310	C1orf54
A_23_P45831	10.575768	10.631479	NM_004284	chr1:146766111-146766170	CHD1L
A_32_P12580	7.115824	7.07286	NM_001003652	chr18:45360247-45360188	SMAD2
A_23_P250644	8.163982	7.856241		chr5:133738492-133738433	CDKN2AIPNL
A_24_P364807	6.666659	6.633747	NM_017839	chr16:55620157-55620216	LPCAT2
A_23_P129221	8.24211	8.277242	NM_000137	chr15:80467376-80469880	FAH
A_24_P122682	9.019032	9.081067	NM_004578	chr1:229434766-229438614	RAB4A
A_33_P3523501	5.462558	5.478352	XR_109585	chr19:22798453-22798394	LOC374890
A_32_P215318	4.9338036	5.153731	NM_198839	chr17:35687296-35687237	ACACA
A_33_P3209386	6.8854923	6.8297772	NM_032731	chr17:6547783-6547842	TXNDC17
A_23_P61127	9.783083	9.5531435	NM_024122	chrX:23851614-23851555	APOO
A_24_P150931	3.7453957	3.965826	NM_007225	chr17:47656996-47657055	NXPH3
A_33_P3424153	5.367883	5.096506		chrX:079817332-079817391	
A_23_P210091	9.2661295	9.486972	NM_014860	chr2:27876053-27875994	SUPT7L
A_33_P3348011	3.1666842	2.3900566	NR_002936	chr6:28183182-28183123	TOB2P1
A_23_P316511	3.1137507	3.1837196	NM_002146	chr17:46627361-46627302	HOXB3
A_23_P122896	5.643723	5.7975492	NM_003227	chr7:100225264-100225205	TFR2
A_33_P3356341	5.8141174	6.3877015	NM_001012241	chr17:38290092-38290151	MSL1
A_23_P130113	3.7501924	4.198912	NM_080912	chr17:7004739-7004680	ASGR2
A_24_P256063	9.096531	8.6710615		chr6:112683207-112683148	
A_33_P3301394	9.045373	9.470165		chr3:141584094-141584035	
A_32_P203099	4.8551674	5.1179295	NM_182539	chr6:44247988-44247929	TCTE1
A_33_P3418917	5.5816956	5.613926	NM_001039517	chr1:155290323-155290264	RUSC1-AS1
A_23_P208961	8.719964	8.510844	NM_032853	chr19:1378272-1378331	MUM1
A_24_P156490	10.769851	10.835839	NM_002247	chr10:78644826-78644767	KCNMA1
A_33_P3279660	3.1370826	3.5376527	BC127725	chrUn_gl000218:000058705-000058764	
A_33_P3276520	5.47651	5.4821444	AK123704	chr17:62787383-62787324	
A_23_P124486	4.8140593	4.887761	NM_002833	chr15:75760219-75760160	PTPN9
A_33_P3348639	12.600441	12.448046	NM_001271014	chr14:56146327-56146386	KTN1
A_24_P68908	5.5128508	4.1359234		chr3:185698542-185698601	LOC344887
A_33_P3404470	6.8068495	6.733415	NM_030930	chr11:67758829-67758771	UNC93B1
A_23_P213602	6.8541517	6.296269	NM_001044723	chr5:122950103-122950162	CSNK1G3
A_23_P81087	7.633207	7.909619	NM_020395	chr4:106607850-106604417	INTS12
A_33_P3222689	6.941675	6.6701	NM_001164416	chrX:103296776-103296835	H2BFM
A_24_P125283	8.891819	9.731794	NM_001015053	chr17:42154223-42154164	HDAC5

A_24_P854913	7.1463184	7.0796432	NM_001127395	chr2:208473951-208473892	METTL21A
A_33_P3323945	5.5074406	5.5849223	NM_016564	chr11:787170-787111	CEND1
A_23_P59107	11.7690935	11.385564	NM_014046	chr6:30594091-30594150	MRPS18B
A_33_P3317392	4.871302	5.0690627	NM_133638	chr5:129074234-129074293	ADAMTS19
A_23_P123454	6.092394	5.683467	NM_024815	chr8:21964671-21964612	NUDT18
A_23_P201655	9.893587	10.216342	NM_012333	chr1:39330362-39330303	MYCBP
A_33_P3337259	7.356106	7.2237763		chr19:61457-61398	WASH5P
A_24_P397247	9.551687	9.239731	NM_018115	chr4:76879062-76879003	SDAD1
A_33_P3370284	12.539214	12.611908	NM_001130071	chr19:56207072-56207131	EPN1
A_23_P155376	6.459914	6.492976	NM_015513	chr3:9986511-9986570	CRELD1
A_24_P226278	6.271596	6.143879	NM_015288	chr5:133914865-133914924	JADE2
A_33_P3339336	6.2824883	5.6309433		chr12:8383704-8383645	FAM86FP
A_23_P101407	8.1227	8.778071	NM_000064	chr19:6679434-6679177	C3
A_23_P126313	7.4035707	7.205462	NM_018417	chr1:167779035-167778976	ADCY10
A_24_P133162	7.355002	6.824914	NM_178122	chr1:24683773-24683714	STPG1
A_33_P3226105	4.606927	4.866433		chr22:020475280-020475221	
A_33_P3347152	4.119613	3.870725		chr6:44923034-44922975	SUPT3H
A_33_P3406961	7.198785	7.277226		chr7:66311846-66311905	XLOC_014512
A_33_P3259028	4.2256823	4.474042	AK127592	chr7:138270634-138270693	TRIM24
A_24_P251221	7.6614494	8.283025	NM_006243	chr1:212534415-212534474	PPP2R5A
A_23_P59888	12.856339	12.624529	NR_002182	chr8:102381755-102381814	NACAP1
A_23_P61268	8.603433	8.593726	NM_016458	chr8:145195463-145195522	FAM203A
A_33_P3277514	3.8474054	3.7549007	NM_000061	chrX:100604520-100604461	BTK
A_33_P3244784	2.9187863	3.2451847	NM_173600	chr12:40964424-40964483	MUC19
A_33_P3392892	3.729853	4.0870833	NM_017886	chr3:41288211-41288152	ULK4
A_33_P3215123	8.510643	8.494968	NM_001100426	chr4:99363172-99363231	RAP1GDS1
A_23_P216489	9.536503	9.711143	NM_005476	chr9:36214701-36214642	GNE
A_23_P120776	6.903557	7.198785	NM_005984	chr22:19163090-19163070	SLC25A1
A_33_P3362153	9.609197	9.853773	NM_001190764	chr19:55890672-55890613	TMEM238
A_23_P157679	9.731586	9.676334	NM_032334	chr8:117783781-117783840	UTP23
A_23_P84576	4.9852476	4.955463	NM_053034	chr2:69302724-69302783	ANTXR1
A_24_P3973	11.764043	11.789254	NM_002137	chr7:26232975-26232916	HNRNPA2B1
A_23_P136413	7.5485244	7.802809	NM_016155	chr12:132336244-132336301	MMP17
A_33_P3249259	3.82745	3.7959723	NM_198994	chr20:2411583-2411642	TGM6
A_23_P307310	3.5703301	3.8645387	NM_013227	chr15:89417771-89417830	ACAN
A_32_P387648	10.829411	10.926812	NM_002016	chr1:152274918-152274859	FLG
A_33_P3354181	6.9048233	6.0686703	NM_020122	chr2:85282501-85282560	KCMF1
A_33_P3606465	5.0897336	5.0526075	NR_033754	chr18:2652365-2652306	CBX3P2
A_33_P3289218	7.4612207	7.6985526	NM_014911	chr2:69686742-69686683	AAK1
A_33_P3374398	4.4150877	4.5296726	XM_005275827	chr17:72600167-72600226	
A_33_P3355567	7.1083436	7.4884577	AL833436	chr8:87494784-87494725	
A_23_P81399	10.817555	10.683936	NM_003900	chr5:179263709-179263768	SQSTM1
A_24_P287780	5.5097127	5.5128508	NM_002768	chr16:89715894-89715835	CHMP1A
A_33_P3262043	8.717964	8.514536	NM_004322	chr11:64037542-64037483	BAD
A_23_P42997	7.9673624	8.056068	NM_006693	chr7:99054694-99054753	CPSF4
A_23_P89910	10.157683	10.102343	NM_024707	chr19:45593948-45594007	GEMIN7
A_23_P135084	15.677139	15.537646	NM_000972	chr9:136217529-136217862	RPL7A
A_24_P332482	12.163997	12.192892		chr5:078806278-078806337	
A_33_P3313597	3.673079	3.4745357	XM_003119827	chr20:62475057-62474998	LOC100509861
A_23_P339818	6.7165465	7.710079	NM_183376	chr15:98516895-98516954	ARRDC4
A_33_P3333826	9.489654	9.7413645	NM_173469	chr15:76193256-76193315	UBE2Q2
A_24_P219971	8.833872	8.566274	NM_017896	chr20:61579681-61579740	GID8
A_33_P3587611	5.4325466	4.9988694	BC020828	chr9:35508625-35508684	RPL36AP33
A_23_P406131	6.6044703	6.884735	NM_020422	chr16:21191196-21191255	TMEM159
A_24_P349039	4.4809246	4.20743	NM_020754	chr3:119136009-119136068	ARHGAP31
A_23_P502170	6.462069	6.9186373	NM_015522	chr2:44021679-44021738	DYNC2LI1
A_23_P149852	8.056921	8.270436	NM_032429	chr10:102767095-102767154	LZTS2
A_24_P358321	4.692209	5.0687747	U21012	chr2:89986891-89986950	
A_33_P3408913	2.4272745	2.3900566	NM_001127380	chr11:18260707-18260648	SAA2

A_33_P3381235	5.816819	6.126812	NR_024470	chr20:61294439-61294380	LOC100127888
A_33_P3298492	12.756387	12.761178	NM_005016	chr12:53873211-53873270	PCBP2
A_33_P3334108	7.6472654	7.593876	NM_001004304	chr12:53584595-53584654	ZNF740
A_32_P171313	10.404069	10.18319	NM_021629	chr3:179113988-179113929	GNB4
A_23_P5601	9.222044	8.955202	NM_001381	chr2:74784597-74784656	DOK1
A_24_P88775	2.8835106	3.1930132	NM_032788	chr2:95814209-95814150	ZNF514
A_32_P155460	3.5179923	4.018555		chr21:46419455-46419396	LINC00162
A_23_P92520	5.8946476	5.6723332	NM_012403	chr4:165118708-165118649	ANP32C
A_23_P53363	7.5522075	7.486825	NM_033276	chr12:58345669-58347438	XRCC6BP1
A_23_P83736	7.5975585	7.8461843	NM_001564	chr4:184431876-184431935	ING2
A_24_P285480	10.69131	10.641192	NM_004891	chr2:28002334-28002393	MRPL33
A_23_P139998	6.6756105	6.6919856	NM_177967	chr13:100037723-100037782	UBAC2
A_33_P3333560	4.3181477	3.7743738		chr1:207495536-207495477	
A_23_P394448	6.849412	7.07367	NM_015283	chr7:34971230-34971171	DPY19L1
A_33_P3645888	4.359969	3.8485622	BG943532	chr20:37058255-37058196	SNORA71C
A_24_P355649	6.654194	6.067007	NM_002017	chr11:128681968-128682027	FLI1
A_23_P160742	6.653843	6.8578224	NM_053274	chr1:92737099-92737040	GLMN
A_33_P3305348	4.979638	4.9696865	NM_014939	chr18:29409305-29409246	TRAPPC8
A_33_P3368636	12.700055	12.775692		chr16:028251197-028251138	
A_23_P28015	2.3221061	2.3900566	NM_144693	chr19:8920611-8920552	ZNF558
A_23_P157022	6.646325	6.8878155	NM_152411	chr7:148767436-148767377	ZNF786
A_23_P409168	4.839042	5.0193005	NM_015175	chr3:47050011-47050070	NBEAL2
A_23_P305759	7.6985526	7.8909225	NM_138340	chr18:19236897-19236838	ABHD3
A_23_P99579	6.8911753	7.263839	NM_032490	chr14:93670003-93669944	C14orf142
A_33_P3291459	10.730606	10.789479	NR_037169	chr6:32120638-32120579	LOC100507547
A_32_P197561	5.077089	4.579202	NM_024007	chr5:158124037-158123978	EBF1
A_33_P3211443	4.9899635	4.3244085	NM_015350	chr1:90058543-90058602	LRRC8B
A_24_P374634	6.4315	6.5480156	NM_001164380	chr8:74332923-74332864	STAU2
A_23_P305938	5.295468	5.6886067	NM_007131	chrX:134420419-134420360	ZNF75D
A_33_P3228609	5.9361863	6.308315	NR_046110	chr2:110752003-110752062	LINC01123
A_23_P126649	2.3221061	2.3900566	NM_001258311	chr1:230458233-230458174	PGBD5
A_23_P160582	8.240751	8.402874	NM_031207	chr1:43917493-43917354	HYI
A_33_P3302373	3.5810375	3.83454	NM_015284	chr1:43918245-43918304	SZT2
A_33_P3361701	8.029783	8.115136	NM_001127320	chr1:154234113-154234172	UBAP2L
A_33_P3287710	6.0098352	5.690216	NR_024585		DLG5-AS1
A_23_P309865	6.536378	6.3151207	NM_152695	chrX:134496752-134496811	ZNF449
A_33_P3223116	4.051962	4.39669	AF207702	chr7:139268530-139268471	HIPK2
A_23_P105592	8.24066	7.8807936	NM_000431	chr12:110034941-110035000	MVK
A_24_P65507	11.260879	11.400044	NM_144998	chr17:79976695-79976636	STRA13
A_24_P942773	8.744717	8.691206	NM_007159	chr3:57914804-57914863	SLMAP
A_23_P71591	10.517908	10.2966585	NM_017948	chr9:95060577-95060148	NOL8
A_23_P23017	8.153193	8.262011	NM_017887	chr1:53680117-53680058	C1orf123
A_23_P137403	4.8181047	4.8107843	NM_006369	chr1:46745913-46745277	LRRC41
A_23_P200310	9.478596	9.754465	NM_017779	chr1:68939846-68939812	DEPDC1
A_23_P102925	7.804295	7.632475	NM_005049	chr21:45550593-45550652	PWP2
A_24_P943575	7.7185016	7.5112753	NM_032221	chr20:40031475-40031416	CHD6
A_23_P126528	3.5754492	3.4676225	NM_005549	chr1:111060518-111060459	KCNA10
A_23_P44781	5.3157816	5.2747936	NM_017774	chr6:21232156-21232215	CDKAL1
A_33_P3464555	7.2287874	6.8294153	NR_027322	chr10:12875863-12875922	LOC283070
A_23_P60565	5.5842094	5.7839007	NM_005649	chr5:178139032-178138973	ZNF354A
A_33_P3239112	4.2967534	3.7704906	AB214500	chr9:98236390-98236331	PTCH1
A_33_P3295655	2.3221061	2.3900566	NM_001163	chr9:72042509-72042450	APBA1
A_24_P239076	5.3203697	5.4485435	NM_020070	chr22:23915710-23915651	IGLL1
A_33_P3274049	4.69787	5.0605516	BC022268	chr2:111645497-111645556	ACOXL
A_23_P52127	10.654071	10.685423	NM_032360	chr1:180257526-180257467	ACBD6
A_33_P3265704	12.654364	12.586741	NM_005659	chr22:19438207-19438148	UFD1L
A_23_P93213	3.749434	3.1628013	NM_153320	chr6:43271973-43272458	SLC22A7
A_24_P296568	11.490257	11.596947	NM_006807	chr17:46147546-46147487	CBX1
A_23_P164912	7.409274	7.6167493	NM_022165	chr19:49621654-49621713	LIN7B

A_23_P127095	12.823801	12.818307	NM_032747	chr10:105148909-105148850	USMG5
A_24_P346126	9.716636	9.91538	NM_022968	chr5:70203787-70203846	SERF1A
A_23_P34176	6.8481255	6.585471	NM_015691	chrX:10112233-10112292	WWC3
A_33_P3375133	5.193159	5.075792		chr11:049071607-049071548	
A_24_P12435	7.3564095	7.215926	NM_181782	chr6:126251879-126251938	NCOA7
A_33_P3258191	9.742853	9.601555	NM_001272051	chr16:1470003-1469944	C16orf91
A_33_P3372869	7.1743636	6.898076	NM_007010	chr17:35972502-35972443	DDX52
A_23_P343261	5.1081114	4.8096094	NR_103534	chr1:6299279-6299338	LINC00337
A_33_P3215113	10.574993	10.563306	NM_012317	chrX:140270000-140269941	LDOC1
A_23_P26847	7.4643726	7.529879	NM_000346	chr17:70121841-70121900	SOX9
A_24_P811704	7.4620814	6.9920764	NM_003622	chr12:27802948-27803007	PPFIBP1
A_32_P353798	10.751918	10.785187	NM_030783	chr11:491325-491384	PTDSS2
A_24_P6674	4.284109	4.1781554	NM_001012361	chr9:116078607-116078548	WDR31
A_23_P348208	8.655484	9.028669	NM_005987	chr1:152957897-152957956	SPRR1A
A_33_P3392177	10.822683	10.766037	NM_001256023	chr6:45882033-45881974	CLIC5
A_33_P3366456	6.297576	6.5789366	NM_138574	chr6:22570614-22570673	HDGFL1
A_23_P210538	4.37233	4.3076954	NM_182764	chr20:44997578-44996189	ELMO2
A_33_P3351120	7.425636	7.176006	NM_001261445	chr12:104732949-104733008	TXNRD1
A_23_P75038	8.506512	8.195292	NM_014881	chr10:115594668-115594609	DCLRE1A
A_23_P325119	6.41562	6.0297766	NR_003239	chr20:37079071-37079215	SNHG11
A_32_P90047	10.275385	10.397855	NM_001141936	chr4:2044544-2045639	C4orf48
A_33_P3324781	5.765214	5.7707105		chr4:003559506-003559565	
A_23_P389118	10.173553	10.1431	NM_001025356	chr12:45825919-45825978	ANO6
A_23_P39116	9.415685	9.521087	NM_000234	chr19:48618881-48618822	LIG1
A_33_P3386297	4.803059	4.971819	AF190162	chrX:135240238-135240297	
A_24_P944049	6.1669655	6.0023336	NM_015147	chr2:65313473-65313532	CEP68
A_23_P23616	5.68952	5.894452	NM_032129	chr1:910300-910359	PLEKHN1
A_33_P3305820	3.3392787	3.6100452		chr21:039734290-039734349	
A_24_P291588	6.0178223	6.052743	NM_004423	chr3:183890582-183890641	DVL3
A_23_P169978	5.848717	6.5581617	NM_020747	chr5:123973545-123973486	ZNF608
A_24_P127235	5.42926	5.500589	NM_004327	chr22:23652578-23653900	BCR
A_33_P3209433	5.0144563	4.4266434	NM_021975	chr11:65422045-65421986	RELA
A_33_P3240063	4.915118	4.948896	NR_024182	chr14:94473826-94473885	LINC00521
A_33_P3278200	5.0567975	5.262557	AK126107	chr21:42735919-42735978	MX2
A_33_P3309468	11.339222	11.509441	NM_002850	chr19:5205578-5205519	PTPRS
A_24_P9285	8.908225	9.04659	NM_006816	chr5:176764374-176764203	LMAN2
A_23_P393686	4.8784823	5.0706725	NM_175075	chr8:442672-442613	TDRP
A_24_P91701	6.755336	6.649584	NM_016449	chr22:23974158-23974099	C22orf43
A_33_P3228305	5.505585	5.057622	NM_015071	chr5:142608490-142608549	ARHGAP26
A_33_P3220390	5.2127542	5.040458	BC028180	chr6:160149383-160149442	WTAP
A_23_P35564	7.2581863	7.4069357	NM_015490	chr10:102246472-102246413	SEC31B
A_33_P3417745	10.00099	9.982542	NM_003870	chr15:91045415-91045474	IQGAP1
A_32_P19431	14.478071	14.549507	NM_001011724	chr13:53217750-53217809	HNRNPA1L2
A_23_P146798	8.390245	8.56596	NM_012248	chr16:30455533-30455474	SEPHS2
A_33_P3385870	7.7479515	8.802953	NM_004354	chr4:78087157-78087216	CCNG2
A_23_P66011	2.3221061	2.3900566	NM_019065	chr16:84036014-84036073	NECAB2
A_24_P126262	3.2330863	3.5749323	NM_181704	chr21:11039244-11039185	BAGE4
A_23_P371613	12.107283	12.146107	NM_203298	chr10:75542926-75542985	CHCHD1
A_24_P403244	6.91243	6.8520575	NM_178238	chr7:99957029-99957088	PILRB
A_33_P3265374	10.474838	10.045365	NM_001145722	chr19:19040106-19040047	HOMER3
A_23_P16096	5.4091396	5.5402284	NM_020230	chr19:10218744-10218803	PPAN
A_23_P397417	8.938645	9.377767	NM_153261	chr16:50070599-50070658	CNEP1R1
A_24_P205036	6.2271786	6.5391474	NM_001001411	chr19:22362430-22362371	ZNF676
A_33_P3267208	8.686992	8.646133	NM_022048	chr15:64457775-64457716	CSNK1G1
A_33_P3262495	8.061101	7.657939	NM_032772	chr10:77158027-77157968	ZNF503
A_33_P3302245	5.6866875	5.7594714	NM_012109	chr19:18731602-18731661	TMEM59L
A_23_P132915	6.135405	6.061604	NM_138389	chr4:38942605-38945098	FAM114A1
A_33_P3287611	6.131026	6.2261763	NM_057088	chr12:53183660-53183601	KRT3
A_33_P3406904	4.8726635	4.491212	XR_246464	chr11:108388367-108388426	LOC101928561

A_24_P208345	7.506898	6.5492125	NM_033102	chr1:205627096-205627037	SLC45A3
A_23_P92967	8.629412	8.555081	NM_004531	chr5:52397288-52397229	MOCS2
A_33_P3269740	8.224453	8.360956	NM_001137610	chr8:12283392-12283333	FAM86B2
A_24_P873659	9.560816	9.50061	NR_002819	chr11:65272873-65272932	MALAT1
A_33_P3391517	3.739479	4.037075	XM_005254677	chr15:64446169-64446228	SNX22
A_24_P82630	7.5499377	7.168345	NM_015295	chr18:2804753-2804812	SMCHD1
A_23_P75330	11.710349	11.639368	NM_004966	chr10:43882102-43882043	HNRNPF
A_23_P25735	12.569995	12.33431	NM_002791	chr14:35786496-35786555	PSMA6
A_33_P3403361	8.514936	8.85888	NM_006327	chr10:51613296-51613237	TIMM23
A_33_P3328289	4.816778	4.7342076			
A_33_P3367017	6.486087	5.7081823	NM_001024957	chr11:66108504-66108445	BRMS1
A_33_P3236267	14.197328	14.123182	NR_002728	chr11:2658580-2658523	KCNQ1OT1
A_23_P144896	7.143242	6.768422	NM_005451	chr5:176910887-176910692	PDLIM7
A_33_P3636315	4.780489	4.671253	AK091675	chr8:2149929-2149988	LOC286087
A_24_P268993	6.567259	6.190317	NM_052971	chr5:132210265-132210324	LEAP2
A_33_P3343145	6.1852813	6.3122206	NM_005909	chr5:71500947-71501006	MAP1B
A_33_P3218783	3.3797596	3.5549948	NM_001204404	chr10:61842434-61842375	ANK3
A_23_P213385	10.562885	9.528321	NM_006317	chr5:17276590-17276649	BASP1
A_24_P299685	6.1461954	6.044671	NM_198389	chr1:13940822-13940881	PDPN
A_32_P192594	6.759767	6.459914	BC014066	chr13:020439082-020439141	
A_24_P246351	2.8237262	2.3900566	NM_138411	chr19:50970163-50970104	FAM71E1
A_33_P3316539	8.072372	8.022785	NM_001008539	chr8:17427847-17427906	SLC7A2
A_23_P85693	4.6526093	4.4772058	NM_004120	chr1:89578160-89575896	GBP2
A_33_P3231750	7.147024	7.141903	NR_027130	chr19:21562040-21562099	ZNF738
A_33_P3274332	7.1414194	6.5915704	NM_016021	chr6:90036406-90036347	UBE2J1
A_33_P3262012	6.6247168	6.713484		chr2:220406302-220406243	
A_33_P3261024	5.347135	5.2403016		chr6:137520352-137520411	
A_23_P152791	5.280031	4.838113	NM_004694	chr17:66264963-66264904	SLC16A6
A_33_P3402500	10.610851	10.586732	NM_001165258	chr6:10725210-10725269	TMEM14C
A_33_P3326046	6.1079926	5.7960534	NM_015528	chr17:4846814-4847909	RNF167
A_23_P147984	11.1665945	11.142983	NM_004238	chr2:230631997-230631938	TRIP12
A_33_P3347658	6.2507906	6.2599087		chr1:052719725-052719784	
A_33_P3294159	6.912079	7.0771527	NM_015722	chr10:135141509-135141450	CALY
A_23_P134147	8.295565	7.930994	NM_014797	chr6:109784046-109783987	ZBTB24
A_23_P127068	5.8231864	6.2707896	NM_017893	chr10:102745224-102745283	SEMA4G
A_23_P635	8.364776	8.499219	NM_007221	chr1:156209462-156209521	PMF1
A_33_P3248420	3.828843	4.2275295	NM_001145268	chr7:102427856-102427915	FAM185A
A_33_P3275174	2.4322834	3.2261074	NM_001164457	chr8:7215557-7215498	ZNF705G
A_24_P941217	4.345258	3.9913635	XM_005246297	chr2:223425440-223425499	SGPP2
A_24_P53150	10.192919	9.921932	NM_032271	chr16:2227991-2228050	TRAF7
A_23_P160537	8.242495	8.045444	NM_024037	chr1:26160615-26160556	AUNIP
A_24_P263703	4.5239897	4.049402	AF318327	chr7:139077225-139077284	PP12708
A_24_P84428	10.474212	10.352814	NM_014412	chr1:174973771-174973833	CACYBP
A_24_P287756	10.248001	10.434531	NM_007006	chr16:56463585-56463526	NUDT21
A_23_P412409	9.840464	9.966606	NM_015172	chr1:171562050-171562109	PRRC2C
A_23_P53663	8.962023	8.722313	NM_002583	chr12:79990419-79990360	PAWR
A_33_P3324206	4.053623	4.2543	NM_005144	chr8:21972023-21971964	HR
A_23_P5742	5.5614076	6.087327	NM_024584	chr2:27848758-27848699	CCDC121
A_32_P205553	10.516994	10.275385	NM_016093	chr5:172396613-172396672	RPL26L1
A_24_P278126	10.089412	10.10697	NM_002485	chr8:90946443-90946384	NBN
A_33_P3294917	2.678761	2.3900566	NM_015225	chr9:79244177-79244118	PRUNE2
A_24_P303874	5.3894744	5.685911	NM_173520	chr9:138236188-138236247	C9orf62
A_33_P3850216	9.868034	10.179716	NM_033058	chr8:67087190-67087249	TRIM55
A_33_P3384452	11.077798	11.264576	NM_007111	chr13:114295314-114295373	TFDP1
A_24_P205213	4.040212	4.9297457	NM_198709	chr5:78181467-78181408	ARSB
A_33_P3278118	5.16174	5.4621897	NM_004346	chr4:185553049-185552990	CASP3
A_23_P109072	2.3221061	2.3900566	NM_020436	chr20:50400915-50400856	SALL4
A_23_P137470	8.511744	9.096776	NM_020808	chr1:232534575-232534516	SIPA1L2
A_33_P3268634	6.7973614	6.5061235	NM_032345	chr12:56295845-56295786	WIBG

A_24_P100761	4.4939284	4.4013906	NM_017679	chr17:59469390-59469449	BCAS3
A_33_P3373203	2.3221061	2.3900566	AK126778	chrUn_gl000219:000073534-000073475	
A_33_P3241741	4.344548	4.059485	NR_002962	chr11:9450417-9450476	SNORA23
A_24_P100016	5.647345	5.600584	NM_052852	chr19:20307910-20307969	ZNF486
A_24_P174367	9.434108	8.298608	NM_006241	chr3:195241570-195241511	PPP1R2
A_33_P3273063	5.345203	5.395346	NM_198887	chr6:150057672-150057613	NUP43
A_24_P288993	11.544801	11.477276		chr4:043901303-043901362	
A_33_P3258702	7.37266	7.576757	NM_001201584	chr3:42599130-42599071	SEC22C
A_23_P41246	6.1763186	6.308099	NM_001120	chr4:2933590-2933370	MFSD10
A_23_P82823	7.241291	6.833027	NM_017884	chr8:10623329-10623270	PINX1
A_24_P75456	12.31544	11.756321		chr7:028319095-028319036	
A_24_P101651	4.517907	4.1598215	NR_073432	chrX:151896238-151896179	CSAG4
A_24_P307580	6.1171474	6.1646366	NM_001098523	chr11:20388976-20389034	HTATIP2
A_23_P87500	9.982542	10.324818	NM_014182	chr12:56214137-56214196	ORMDL2
A_33_P3336822	4.092786	4.825526	U09907	chr22:22735478-22735537	
A_24_P98249	7.757497	8.02259	NM_006283	chr8:38709801-38709860	TACC1
A_23_P48964	5.100852	5.1222687	NM_018668	chr15:91545332-91544686	VPS33B
A_32_P198923	8.82123	9.28651	NM_145690	chr8:101960895-101960836	YWHAZ
A_24_P382765	8.236131	8.196723	NM_005008	chr22:42070512-42070453	NHP2L1
A_24_P343271	7.000249	7.1113124	NM_017909	chr6:151742444-151742385	RMND1
A_33_P3344801	4.117345	4.3834295		chr1:43396129-43396070	SLC2A1
A_33_P3412384	4.240226	3.9399173	NM_002457	chr11:1080924-1080983	MUC2
A_23_P304652	3.5103798	3.3602316	NM_178130	chr3:137980577-137980518	NME9
A_33_P3397279	8.152092	8.230398	NM_001242898	chr22:50882760-50882819	PPP6R2
A_32_P115558	6.334667	6.404135		chr17:016285923-016285864	
A_24_P724040	7.899977	7.787652	NM_003092	chr20:16719540-16721021	SNRPB2
A_33_P3344574	7.9187074	8.534889	NM_001098668	chr10:81315682-81315623	SFTPA2
A_24_P170983	4.593394	4.8518696	NM_194312	chr2:239041849-239041908	ESPNL
A_23_P432056	4.415232	4.489956	NM_178568	chr17:1840616-1840557	RTN4RL1
A_23_P35883	3.7110436	2.3900566	NM_017547	chr11:126145322-126145746	FOXRED1
A_23_P161076	4.0325093	2.3900566	NM_001767	chr1:117311778-117311837	CD2
A_33_P3256868	6.0853796	6.2589293	NM_001277945	chr19:53116330-53116271	ZNF83
A_23_P34066	5.9189463	5.98567	NM_176786	chrX:155240267-155240326	IL9R
A_23_P48495	5.2120976	5.278888	NM_004918	chr14:96158867-96158926	TCL1B
A_23_P44617	7.671979	7.693034	NM_016128	chr3:128994046-128994105	COPG1
A_23_P32217	5.5620394	5.4326105	NM_005802	chr9:32541438-32541379	TOPORS
A_23_P202905	4.3201756	5.053908	NM_148910	chr11:126162931-126162990	TIRAP
A_33_P3219770	5.7406735	6.0238853	NM_024529	chr1:193220997-193221056	CDC73
A_23_P23171	4.061023	4.5514517	NM_017629	chr1:36320420-36320479	AGO4
A_33_P3294901	7.596741	7.3546767	NM_001017390	chr16:29474684-29474743	SULT1A4
A_23_P333852	4.210011	4.419346	NM_194252	chr9:124751865-124751806	TTLL11
A_32_P115505	7.1699014	7.3982754	NM_015565	chr21:30301477-30301418	LTN1
A_23_P19243	3.6580908	3.7760732	AF119870	chr6:88410191-88410132	
A_33_P3524912	6.9259367	6.856228		chr10:127398101-127398160	LOC283038
A_33_P3329043	5.736113	5.2620764	BC073826	chr6:1384086-1384027	
A_33_P3421759	6.1638346	6.332418	NM_032195	chr21:34929473-34929532	SON
A_33_P3281807	6.3789616	6.2492123	NM_182612	chr11:770374-770315	PDDC1
A_24_P418619	12.734198	12.834596	NM_001014	chr6:34392604-34392546	RPS10
A_33_P3379571	6.7124376	6.495655	NM_033141	chr14:71196427-71196368	MAP3K9
A_23_P209200	8.924311	9.068874	NM_001238	chr19:30315106-30315165	CCNE1
A_24_P76078	10.358288	10.431042		chr17:027348534-027348475	
A_33_P3230488	10.308756	10.412121	NM_003952	chr11:67202755-67202814	RPS6KB2
A_23_P87363	3.6511471	3.4230654	NM_004314	chr11:3685463-3685523	ART1
A_23_P309207	3.4928098	3.1726813	NM_032679	chr19:52375475-52375416	ZNF577
A_33_P3417502	4.7729607	5.0852904	NM_033131	chr1:228247946-228248005	WNT3A
A_23_P62607	6.3596344	6.286987	NM_021258	chr1:24446278-24446235	IL22RA1
A_33_P3372659	3.8908758	3.7691913		chr15:020318747-020318806	
A_33_P3260689	4.480255	4.4169064	NM_001017990	chrX:154113597-154113656	H2AFB1

A_33_P3423931	4.5520444	4.954335	NM_001001676	chr9:138557125-138557184	LCN9
A_33_P3283780	4.587993	4.244814	NM_015898	chr19:4045405-4045346	ZBTB7A
A_23_P206059	12.032792	11.859836	NM_003981	chr15:91509485-91509426	PRC1
A_33_P3295578	9.272178	9.407259	NM_153333	chrX:102508646-102508587	TCEAL8
A_33_P3221680	15.000444	15.041382	NM_001031	chr2:232121106-232121165	RPS28
A_32_P225604	14.549507	14.438628	NM_000969	chr1:93303074-93303132	RPL5
A_33_P3270102	7.231949	7.278142	NM_194460	chr19:648968-648909	RNF126
A_33_P3343295	7.950018	8.123393	NM_024535	chr16:4404604-4404545	CORO7
A_33_P3261877	8.263453	8.201096	AF131822	chr5:43068998-43069057	LOC100506639
A_32_P103669	8.905421	8.907279	NM_001164404	chr15:75565602-75565661	GOLGA6C
A_33_P3323101	7.8782134	8.06154	NM_001278171	chr10:38343362-38343421	ZNF33A
A_33_P3300864	10.861438	11.100205	NM_001005200	chr11:55873240-55873299	OR8H2
A_23_P57401	5.6227865	5.36908	NM_006767	chr22:21352740-21352799	LZTR1
A_33_P3331326	8.629108	8.769685		chr13:028194669-028194728	
A_23_P144697	7.8310013	7.5922055	NM_002853	chr5:34908952-34908893	RAD1
A_33_P3212109	8.561216	8.199427	NM_016356	chr6:24172239-24172180	DCDC2
A_23_P171359	6.8143554	6.577023	NM_019045	chrX:117583299-117583358	WDR44
A_33_P3282318	6.2501163	6.0130033		chr13:107516299-107516240	
A_33_P3300635	8.775856	8.00085	NM_006212	chr1:207251103-207251162	PFKFB2
A_23_P42288	7.901058	8.197365	NM_025258	chr6:31734279-31734142	VWA7
A_23_P48166	11.254095	10.8631	NM_002822	chr12:44187922-44187863	TWF1
A_24_P576506	4.4830093	4.587714	NM_001012508	chr10:135234431-135234372	SPRN
A_33_P3229863	7.9845934	8.302221	NR_040082	chr15:26298197-26298256	LOC100128714
A_23_P17021	7.4384117	7.2905555	NM_024583	chr2:175292606-175292665	SCRN3
A_24_P371399	6.4382086	6.7881184	NM_173552	chr3:143710438-143710497	C3orf58
A_33_P3309501	7.7428713	7.884915	AK124580	chr14:101325072-101325131	MEG3
A_23_P348121	6.5701447	6.288734	NM_005253	chr2:28637175-28637234	FOSL2
A_33_P3412016	3.8518124	3.63763	NM_020210	chr15:90771408-90771467	SEMA4B
A_33_P3270926	4.5356646	4.221576	XR_110088	chr8:607638-607579	LOC389607
A_33_P3880302	5.588645	6.073313	NM_004442	chr1:23240664-23240723	EPHB2
A_24_P636882	7.5317597	7.5583973	NM_004226	chr2:196998319-196998290	STK17B
A_33_P3227990	4.243161	3.8846362	NM_001025101	chr18:74690971-74690912	MBP
A_33_P3315779	4.3378344	3.882113	NM_001165136	chr4:89317234-89317293	HERC6
A_32_P156892	10.707723	10.835084	NM_003932	chr22:41228585-41226914	ST13
A_23_P77965	5.866913	5.775445	NM_022070	chr17:58121212-58121153	HEATR6
A_33_P3275959	10.127692	10.113871	BC001407	chr10:70285463-70285404	SLC25A16
A_32_P133244	7.7452593	7.919842	NM_002582	chr16:14529951-14529892	PARN
A_23_P127533	10.389425	9.859182	NM_032299	chr11:102932955-102932896	DCUN1D5
A_23_P256158	5.6320124	5.286504	NM_000683	chr4:3770121-3770180	ADRA2C
A_33_P3380372	5.1222687	5.061437		chr2:061816747-061816688	
A_24_P944616	11.652713	11.709219	NM_016287	chr1:21069352-21069293	HP1BP3
A_32_P136351	3.500247	4.621416	NM_052910	chr13:84451607-84451548	SLITRK1
A_24_P116378	7.8005495	7.502299	NM_001018090	chr15:58009403-58009462	GCOM1
A_32_P172848	4.2486897	4.1546097	NM_000167	chrX:30712566-30712625	GK
A_23_P45475	10.4086485	10.272539	NM_000169	chrX:100652937-100652878	GLA
A_23_P143303	8.0844345	8.153017	NM_022575	chr20:2846106-2846881	VPS16
A_23_P131778	7.424576	7.4970818	NM_022077	chr20:35945269-35945328	MANBAL
A_33_P3298539	4.4942994	4.5502033	NM_000039	chr11:116706700-116706641	APOA1
A_23_P63789	12.278723	12.23941	NM_032997	chr10:58117404-58117345	ZWINT
A_24_P235338	3.5639439	2.3900566	NM_007332	chr8:72933685-72933626	TRPA1
A_23_P3302	6.0096025	5.9700336	NM_018365	chr15:56721249-56721190	MNS1
A_24_P117672	6.02203	5.680666	NM_021228	chr19:50161136-50161513	SCAF1
A_33_P3251985	7.9457545	8.327824	NM_022736	chr3:158541965-158542024	MFSD1
A_23_P40072	9.484858	8.983403	NM_001005369	chr2:55470122-55467299	MTIF2
A_33_P3338634	6.0869665	5.8395424	NM_001256006	chr1:210334193-210334252	SYT14
A_33_P3363460	5.9893136	6.2289433	AK128204	chr5:42953976-42954035	LOC100129186
A_23_P28169	10.366301	10.442104	NM_152522	chr2:153616788-153616847	ARL6IP6
A_33_P3398583	2.88785	4.572304		chr6:40312183-40312124	LINC00951
A_32_P59486	9.714747	9.553627	NM_003002	chr11:111965758-111965817	SDHD

A_24_P307827	9.524509	9.523721	NM_001080435	chr15:83503252-83503311	WHAMM
A_33_P3275422	7.679331	7.8903666	NR_024096	chr14:101138994-101139053	LINC00523
A_24_P111134	6.32163	6.1985064	NM_013382	chr14:77741560-77741501	POMT2
A_32_P3914	5.749482	6.1068363	NM_001080529	chr7:29955838-29955897	WIPF3
A_24_P51322	7.384305	7.518671	NM_002016	chr1:152279523-152279465	FLG
A_33_P3290443	8.6690235	8.651706	NR_002569	chr11:93454973-93455032	SCARNA9
A_33_P3368452	7.973121	8.066761	NM_000247	chr6:31382917-31382976	MICA
A_24_P335092	7.100911	8.223712	NM_000331	chr11:18290868-18291310	SAA1
A_23_P301521	8.471275	8.810795	NM_020848	chr10:30301839-30301780	KIAA1462
A_23_P108082	4.0443444	3.5495105	NM_032607	chr19:4172219-4172278	CREB3L3
A_33_P3870056	2.8231683	3.949324	NR_033854	chr12:53437241-53437182	LOC283335
A_33_P3322388	7.5610423	7.6988783	NM_006945	chr1:153012663-153012604	SPRR2D
A_33_P3481987	15.592702	15.723595	NM_213606	chr10:91190965-91190906	SLC16A12
A_33_P3224380	9.063802	8.725267	NM_001098424	chr3:196865193-196865134	DLG1
A_23_P107981	4.110589	4.4754853	NM_004605	chr19:49096042-49100023	SULT2B1
A_24_P49533	7.466359	7.5775023	NM_024646	chr1:53290938-53290997	ZYG11B
A_33_P3361393	11.573592	11.410988	NM_001412	chrX:20146255-20146196	EIF1AX
A_32_P85330	3.4424284	3.2975397	NR_028330	chr15:80216610-80216669	C15orf37
A_23_P104804	5.172639	4.316314	NM_006006	chr11:114117992-114118051	ZBTB16
A_33_P3211569	6.5273714	6.4797554	NM_001005915	chr12:56479217-56479276	ERBB3
A_33_P3257479	5.294448	5.2921166			
A_23_P6615	9.347628	9.381458	NM_001871	chr3:148563321-148563380	CPB1
A_23_P98232	5.4023595	5.1083612	NM_006782	chr11:64854242-64854436	ZFPL1
A_24_P318656	8.921627	9.147419	NM_000212	chr17:45389027-45389086	ITGB3
A_33_P3251771	5.8646607	4.756803	NM_015247	chr16:50835733-50835792	CYLD
A_24_P299911	6.0619726	5.761337	NM_015148	chr2:242046815-242046127	PASK
A_23_P251051	11.656988	11.221249	NM_181832	chr22:30094507-30094566	NF2
A_33_P3386344	6.2490115	6.18722	NM_001018112	chr16:89877175-89877116	FANCA
A_23_P156732	7.1450787	6.928114	NM_024165	chr6:33383929-33383988	PHF1
A_23_P31739	3.863355	2.3900566	NM_030795	chr8:27094248-27094189	STMN4
A_23_P357717	3.9844413	3.711278	NM_021966	chr14:96176337-96176290	TCL1A
A_24_P71938	6.758303	6.637231	NM_005900	chr4:146480219-146480278	SMAD1
A_33_P3243622	7.5844994	7.7507277	NM_015726	chr1:160209948-160209889	DCAF8
A_32_P184796	15.034151	15.183809	NM_053275	chr12:120635201-120635142	RPLP0
A_23_P208866	3.612365	3.5968442	NM_004877	chr19:39819146-39819087	GMFG
A_23_P7679	8.239718	8.485205	NM_153485	chr5:37294554-37294495	NUP155
A_33_P3247403	5.3454976	5.771495	NM_022371	chr1:179051429-179051488	TOR3A
A_33_P3369258	8.452953	8.544116	NM_001171888	chr12:27110616-27110675	FGFR1OP2
A_23_P214011	4.957954	6.066502	NM_004932	chr5:31324207-31324266	CDH6
A_33_P3418798	4.8938246	5.002389	NM_001042690	chr4:3255149-3255208	MSANTD1
A_24_P342312	6.348011	6.3527646	NM_001098816	chr11:78365059-78365000	TENM4
A_24_P294821	3.652648	3.9453075	NM_003898	chr6:158510878-158510937	SYNJ2
A_32_P48134	2.7011223	2.3900566	NR_002793	chr13:53147523-53151206	TPTE2P3
A_33_P3231110	8.466429	8.856905	NM_006537	chr15:63882966-63883025	USP3
A_23_P252471	3.851194	3.1963606	NM_000442	chr17:62400603-62400544	PECAM1
A_33_P3380867	9.018291	8.5399275	NM_012448	chr17:40351255-40351196	STAT5B
A_33_P3211618	4.5928073	4.9478765	AK024389	chr16:81601769-81601828	
A_23_P210939	10.339214	10.145846	NM_181468	chr20:33866998-33866939	EIF6
A_23_P42909	2.8141136	2.3900566	NM_153345	chr7:142984122-142984181	TMEM139
A_33_P3234472	6.6509895	6.647763	NR_034124	chr20:48931397-48931456	LOC284751
A_32_P128656	3.40929	2.3900566	NM_000381	chrX:10414469-10414410	MID1
A_23_P422851	8.4632015	8.292273	NM_138375	chr18:20838542-20838601	CABLES1
A_33_P3228872	5.084015	5.647345	NM_001256619	chr10:100004047-100004106	R3HCC1L
A_23_P168329	4.054351	3.8121443	NM_014464	chr6:54214641-54216155	TINAG
A_24_P322444	11.589407	11.436512	NM_153719	chr19:50410195-50410136	NUP62
A_23_P300150	8.37276	7.996925	NM_172387	chr18:77289219-77289278	NFATC1
A_33_P3359368	5.3092794	5.428461	NM_001277864	chr14:24520448-24520507	DHRS4L1
A_23_P50000	8.0915785	8.533153	NM_024792	chr17:645638-645697	FAM57A
A_23_P128598	7.979789	8.037026	NM_006001	chr13:19751197-19751138	TUBA3C

A_24_P411573	3.9109561	3.655645	NR_002713	chr5:137144553-137144612	NPY6R
A_33_P3252333	8.676715	8.7366	NR_003271	chr17:18965252-18965311	SNORD3B-1
A_23_P398044	8.749027	8.93161	NM_052848	chr19:41830693-41830752	CCDC97
A_24_P382467	4.121132	3.8583322	NM_144564	chr19:2737176-2737117	SLC39A3
A_32_P22338	6.817392	6.981188	NM_013374	chr3:33894107-33894166	PDCD6IP
A_33_P3340060	13.203878	13.176956	NM_012423	chr19:49993522-49993758	RPL13A
A_33_P3306177	5.8987484	6.0248156	NM_153365	chr4:16165046-16164987	TAPT1
A_33_P3321050	9.573547	9.437453	NM_001100165	chr6:144074989-144075048	PHACTR2
A_23_P69109	9.864193	9.606899	NM_021105	chr3:146233121-146233062	PLSCR1
A_33_P3406702	11.361875	11.191802		chr12:104908183-104908242	
A_23_P429020	4.7597075	4.879006	NM_181786	chr19:37854787-37854846	HKR1
A_33_P3343872	5.2303085	5.1831064	NM_001039508	chr20:1617054-1616995	SIRPG
A_24_P334300	5.9264	5.270146	NM_004113	chr3:191860574-191860515	FGF12
A_23_P73012	9.814165	9.861961	NM_032823	chr9:97849233-97849292	C9orf3
A_23_P340149	2.6782026	2.3900566	NM_021998	chrX:84526807-84526866	ZNF711
A_33_P3379056	3.6554358	2.3900566		chr17:079390450-079390391	
A_33_P3354858	5.9804378	6.370951	NM_025147	chr2:198338634-198338693	COQ10B
A_23_P75889	9.966606	10.100403	NM_175932	chr11:250851-251590	PSMD13
A_23_P31372	11.445275	11.483576	NM_005918	chr7:75694196-75694255	MDH2
A_33_P3308764	4.150781	4.582428	NR_033824	chr1:165406225-165406166	RXRG
A_23_P50368	9.317855	9.063802	NM_206818	chr19:54598157-54598098	OSCAR
A_23_P20480	5.6391244	5.808791	NM_018310	chr8:37701951-37701892	BRF2
A_23_P419038	7.986657	7.619623	NM_004898	chr4:56299165-56299106	CLOCK
A_23_P162719	8.56596	8.705704	NM_030932	chr13:60453455-60453396	DIAPH3
A_23_P143952	4.991724	6.378516	NM_024548	chr3:101485649-101485708	CEP97
A_23_P341275	10.4301605	10.130083	NM_015029	chr8:99170489-99170548	POP1
A_33_P3416231	7.63688	7.5713196	NM_152739	chr7:27203204-27203145	HOXA9
A_23_P70095	3.1680632	3.3676536	NM_001025158	chr5:149792281-149792222	CD74
A_33_P3296777	5.737452	5.3770685	NM_006814	chr20:1148367-1148426	PSMF1
A_33_P3407826	11.791811	11.72724		chr1:000666117-000666176	
A_33_P3269019	4.20261	3.859378	AK023337	chr17:21814661-21814720	
A_33_P3230526	4.4824514	4.2415605	NM_015134	chr17:17075120-17075179	MPRIP
A_32_P79190	7.541534	7.6144605	NR_046228	chr1:142699303-142699244	ANKRD20A12P
A_23_P59261	8.573546	8.74205	NM_006670	chr6:83076061-83076120	TPBG
A_24_P247732	3.4719975	3.1403956	NM_021095	chr2:27434388-27430653	SLC5A6
A_24_P126181	7.600404	7.1736164	NM_176880	chr19:19312513-19312454	NR2C2AP
A_33_P3394312	4.5941668	4.738442	NM_001005480	chr7:143807485-143807544	OR2A2
A_23_P79043	11.0597725	11.35618	NM_032635	chr19:36037903-36038051	TMEM147
A_33_P3297978	9.956935	10.175518	NM_004998	chr15:59428708-59428649	MYO1E
A_33_P3379341	2.7204587	2.3900566	NM_001144931	chr22:24373814-24373873	LOC391322
A_32_P147078	5.2639413	4.791488	NM_021097	chr2:40339484-40339425	SLC8A1
A_33_P3317880	7.8642025	7.713785	NR_023392	chr8:146199058-146198999	ZNF252P
A_33_P3251462	9.859182	9.94237	NM_080739	chr20:2796282-2796341	C20orf141
A_23_P3979	9.385975	9.550976	NM_015971	chr17:73258756-73258930	MRPS7
A_33_P3332175	9.174535	9.363431		chrX:051666780-051666839	
A_24_P270460	7.348427	7.8239	NM_005532	chr14:94582967-94583026	IFI27
A_33_P3228072	7.570616	7.651646	NM_153240	chr3:132399705-132399646	NPHP3
A_33_P3359900	7.537006	7.4655676	NM_198449	chr5:49694981-49694922	EMB
A_24_P14932	4.1169705	4.176116	NM_018338	chr3:113085121-113085062	WDR52
A_33_P3318414	5.2687063	4.881827	NM_012292	chr19:1086567-1086626	HMHA1
A_33_P3372886	7.230195	7.205907	NM_175066	chr12:132621201-132621142	DDX51
A_24_P251969	4.587714	3.2582366	NM_000800	chr5:141973506-141973447	FGF1
A_23_P79145	4.02719	4.2186575	NM_144694	chr19:37975762-37975821	ZNF570
A_23_P201097	11.4105015	11.603579	NM_000858	chr1:228336146-228336413	GUK1
A_33_P3425356	9.261974	9.536503	NM_015396	chr3:138015161-138015219	ARMC8
A_33_P3372281	7.755068	6.8474846	NM_001286265	chr6:24418746-24418805	MRS2
A_24_P940620	5.031865	5.245548	XM_005256345	chr17:80115593-80115534	CCDC57
A_33_P3333317	8.195292	7.9405785	NM_001008211	chr10:13180217-13180276	OPTN
A_24_P23546	2.3221061	2.3900566	NM_198956	chr7:20822619-20822560	SP8

A_23_P156807	10.763625	11.0795965	NM_001202519	chr2:202030660-202030717	CFLAR
A_23_P355993	5.9936085	6.1797347	NM_025040	chr19:52521668-52521345	ZNF614
A_24_P323628	9.691311	9.863575	NR_003572	chr8:169299-163661	RPL23AP53
A_33_P3223592	5.5227833	5.971755	NM_000041	chr19:45412590-45412649	APOE
A_33_P3351609	3.7608652	3.9644418		chr16:67572580-67572521	
A_23_P202769	7.64884	7.6172748	NM_005528	chr11:64000251-64000310	DNAJC4
A_33_P3245178	4.5803204	4.6181345	NM_001168399	chrX:102564489-102564430	BEX2
A_23_P80098	10.522473	10.445537	NM_000819	chr21:34876492-34876433	GART
A_24_P193295	10.138359	10.4429655	NM_198686	chr14:65412615-65412556	RAB15
A_23_P318890	3.698854	4.183828	NM_012360	chr16:3255126-3255185	OR1F1
A_33_P3395219	5.4875937	5.6268377	NR_103535	chr1:78353981-78353922	NEXN-AS1
A_24_P380919	12.606205	12.513302	NM_002140	chr9:86583500-86583441	HNRNPK
A_24_P376707	12.488769	12.651925	NM_004494	chr1:156712608-156712549	HDGF
A_33_P3215517	6.200718	5.470087	NM_018715	chr1:17735354-17735295	RCC2
A_24_P284584	6.361704	6.7326326	NM_032497	chr19:9453654-9453713	ZNF559
A_23_P218731	8.262011	8.535971	NM_145858	chr21:34969692-34969633	CRYZL1
A_33_P3708658	4.700623	4.3146124	AK096370	chr11:126220169-126220110	ST3GAL4-AS1
A_24_P59247	10.726288	10.868124		chr12:005141915-005141856	
A_23_P5586	9.354691	9.210001	NM_005791	chr2:71375141-71375200	MPHOSPH10
A_23_P424513	6.7653294	6.5275083	NM_005493	chr6:13622489-13622430	RANBP9
A_33_P3241541	5.6346245	5.7620206	XM_005252254	chr9:108314511-108314570	FSD1L
A_23_P408455	8.924818	9.27188	NM_001104647	chr3:140698479-140698538	SLC25A36
A_33_P3292602	8.811218	9.180113		chr9:014069186-014069245	
A_24_P759477	6.8792677	7.386234	NM_002214	chr7:20455137-20455196	ITGB8
A_23_P341567	3.47435	3.0963132	NM_178833	chr4:103964470-103952924	SLC9B2
A_23_P566	9.266534	9.324963	NM_014947	chr1:42642599-42642540	FOXJ3
A_23_P46222	5.1682777	4.9921308	NM_001282379	chr1:155152361-155152920	TRIM46
A_32_P33434	3.903749	3.5695682	NM_001199814	chr19:9801597-9801538	ZNF812
A_23_P20427	6.7461615	6.478795	NM_015178	chr8:22877436-22877495	RHOBTB2
A_23_P213153	9.971139	9.643016	NM_031372	chr4:83348607-83348420	HNRNPDL
A_32_P226078	4.475943	3.751473	NM_016178	chr1:151742683-151743561	OAZ3
A_23_P39774	8.580446	8.460125	NM_006857	chr2:70131681-70131740	SNRNP27
A_23_P33607	7.40123	7.347843	NM_024683	chr17:29227565-29227506	TEFM
A_23_P255869	8.838591	8.749346	NM_018465	chr9:5361162-5361103	PLGRKT
A_23_P56709	7.621814	7.360765	NM_005667	chr2:86830871-86830812	RNF103
A_33_P3257714	10.120924	10.133879	NM_001025	chr5:81569349-81569290	RPS23
A_23_P88848	4.6820316	4.6554747	NM_017803	chr16:68109364-68110550	DUS2
A_23_P373054	8.469908	8.155733	NM_173826	chr3:44450680-44450739	TCAIM
A_33_P3369969	9.24369	9.527785	NM_001137675	chr16:71891090-71891149	ATXN1L
A_24_P408740	10.584441	10.420882	NM_182523	chr3:28361109-28361168	CMC1
A_24_P941188	6.965535	7.09986	NM_015207	chr1:20239236-20239295	OTUD3
A_24_P304987	8.624057	8.494542	NM_013260	chr17:73703670-73703729	SAP30BP
A_23_P105519	9.668517	9.357597	NM_016312	chr12:14939934-14939875	WBP11
A_24_P80135	8.494388	8.528778	NM_014369	chr2:131132081-131132140	PTPN18
A_23_P24926	10.021111	10.164182	NM_002027	chr8:42940715-42940774	FNTA
A_23_P136058	9.679291	9.797458	NM_001184	chr3:142168423-142168364	ATR
A_24_P329065	6.4362135	6.5925574	NM_007048	chr6:26414743-26414802	BTN3A1
A_24_P399694	8.300883	8.785	NM_033089	chr20:280611-280670	ZCCHC3
A_23_P145957	6.1826515	5.551651	NM_022445	chr7:144149252-144149193	TPK1
A_24_P203072	7.05053	7.2937226	NM_014735	chrX:46919911-46919970	JADE3
A_23_P42282	4.4145055	4.5886135	NM_001002029	chr6:32002329-32002650	C4B
A_23_P89199	11.073485	10.7875395	NM_003653	chr17:17150284-17150225	COPS3
A_23_P210015	5.080429	5.104163	NM_014369	chr2:131132468-131132527	PTPN18
A_33_P3317576	8.017714	7.398729	NM_001195643	chr3:93778084-93778025	DHFRL1
A_33_P3369128	7.07815	7.5152874	AK124556	chr1:143914671-143914730	
A_24_P191312	2.6236923	5.034129	NM_003038	chr2:65250035-65250094	SLC1A4
A_24_P305764	10.976305	10.444419	NM_004595	chrX:22010809-22012467	SMS
A_23_P38167	11.837501	12.053645	NM_022036	chr17:72443413-72443472	GPRC5C
A_24_P50245	4.1004148	4.1939964	NM_006120	chr6:32917477-32917418	HLA-DMA

A_23_P26294	10.721731	11.176093	NM_012467	chr16:1271739-1271680	TPSG1
A_33_P3286422	5.6765804	5.2798133	NM_001018112	chr16:89864823-89864767	FANCA
A_33_P3380101	8.77256	8.662113	NM_018067	chr1:36645937-36645996	MAP7D1
A_33_P3304707	4.459221	3.8209114	Z46314	chr22:23165574-23165633	
A_33_P3289820	9.440264	9.452315	NM_001134382	chr3:12939496-12939437	IQSEC1
A_23_P102351	4.235318	4.0086975	NM_004854	chr2:101009420-101009361	CHST10
A_23_P414281	3.5594864	3.331457	NM_139170	chr16:4799307-4799366	C16orf71
A_33_P3382944	7.6679115	7.730506	NM_198537	chr19:19648334-19648393	YJEFN3
A_33_P3363600	3.8099756	3.874026		chr1:247400734-247400675	
A_24_P401739	8.830631	9.067182	NM_001006634	chr16:24931072-24931013	ARHGAP17
A_23_P319492	6.8977795	6.947619	NM_024556	chr11:126132092-126132151	FAM118B
A_33_P3422374	4.8503513	4.89599	NM_001010868	chr6:88075101-88075160	C6orf163
A_23_P85941	3.978881	3.9962296	NM_006492	chr1:110603170-110603111	ALX3
A_33_P3320272	3.9039798	4.329755	NM_032323	chr1:156261598-156261657	TMEM79
A_33_P3356320	3.9467418	4.937869	NM_001013672	chr17:264081-264140	C17orf97
A_23_P315933	7.606887	7.285628	NM_148912	chr7:73150523-73150464	ABHD11
A_23_P209232	8.773839	8.709604	NM_024692	chr2:29406606-29406665	CLIP4
A_24_P112087	6.9398465	6.9571514	NM_014034	chr6:119229482-119229541	ASF1A
A_32_P517749	8.484755	8.314928	NM_004586	chrX:20168138-20168079	RPS6KA3
A_23_P78289	9.794389	9.751883	NM_032837	chr17:71203606-71203547	FAM104A
A_23_P71537	8.238773	8.613163	NM_024790	chr8:68107632-68107691	CSPP1
A_33_P3330716	7.7392445	8.086481	NM_017759	chr2:206858767-206858708	INO80D
A_23_P35617	7.021453	7.008299	NM_016341	chr10:96087933-96087992	PLCE1
A_24_P302574	7.2818403	6.802417	NM_022353	chr2:190611508-190611449	OSGEPL1
A_33_P3217983	4.4599295	2.3900566	NM_203380	chr10:114188056-114188115	ACSL5
A_23_P157465	7.9044094	7.6440005	NM_005671	chr8:30624343-30624402	UBXN8
A_32_P227845	7.3665733	7.383559	NR_003266	chr3:197346752-197346693	LOC220729
A_23_P29079	4.637734	4.748714	NM_002626	chr21:45744490-45744749	PFKL
A_33_P3265855	4.331378	4.325273	NM_181334	chr22:45258579-45258638	PRR5-ARHGAP8
A_33_P3395442	4.724779	4.517812	NR_015382	chr12:6548232-6548173	CD27-AS1
A_24_P301454	4.0982265	4.4937706	NR_003108	chr17:41283256-41290701	NBR2
A_33_P3342807	8.739321	8.749027		chr7:55815966-55816025	XLOC_014512
A_33_P3365596	7.205907	6.6375914	NM_001283018	chr20:23355225-23355166	NAPB
A_23_P97932	9.488879	9.588752	NM_012228	chr10:23409705-23409764	MSRB2
A_32_P460973	10.059792	10.30224	NM_005516	chr6:30460729-30460788	HLA-E
A_23_P423482	5.962516	6.6015377	NM_152450	chr15:59814801-59814860	FAM81A
A_33_P3327956	7.896248	7.491425	NM_183238	chr12:133498107-133498048	ZNF605
A_23_P157352	9.492376	9.511093	NM_016071	chr7:140710258-140706316	MRPS33
A_23_P253586	5.0075827	5.394251	NM_005128	chr21:37664494-37665639	DOPEY2
A_33_P3284345	6.8637295	7.0741405	NM_004495	chr8:32585521-32585580	NRG1
A_24_P8220	11.022508	11.274025	NM_004807	chr2:129023118-129023059	HS6ST1
A_23_P80008	3.8226655	3.3001502	NM_033118	chr20:30422410-30422469	MYLK2
A_33_P3335228	5.073261	4.3894763	NM_001195296	chr14:37737907-37737966	MIPOL1
A_33_P3344039	4.7130194	4.8508296	NM_007082	chr2:114398474-114398533	RABL2A
A_24_P38347	8.90649	8.574541	NM_001386	chr8:26515178-26515237	DPYSL2
A_23_P44643	6.9483476	6.8796797	NM_016238	chr12:110815226-110813991	ANAPC7
A_24_P75917	4.3769636	4.016538	NM_014695	chr17:16612852-16612911	CCDC144A
A_23_P401547	10.823772	10.999563	NM_015480	chr3:110852740-110852799	PVRL3
A_33_P3375002	3.7390532	3.2175837	NR_038263	chr12:93959469-93959410	SOCS2-AS1
A_23_P212511	6.814513	6.870051	NM_001042601	chr3:180327544-180327603	TTC14
A_33_P3294821	6.5782223	6.390515	NM_177998	chr4:4190593-4190534	OTOP1
A_33_P3227716	7.1442966	7.8988	NM_001037666	chr22:30681194-30681135	GATSL3
A_33_P3420013	6.0959134	6.1517434		chr3:131102087-131102028	
A_23_P8240	2.860574	3.706479	NM_012135	chr6:3851193-3851252	FAM50B
A_23_P252783	8.523925	8.443366	NM_014580	chr9:130169819-130169878	SLC2A8
A_33_P3233645	2.923226	3.3187017	NM_005950	chr16:56700752-56700693	MT1G
A_24_P196851	6.4859343	6.4813247	NM_006289	chr9:35697825-35697766	TLN1
A_33_P3359704	4.916695	4.70353	NM_025069	chr8:37556127-37556186	ZNF703
A_24_P940125	7.025601	7.895301	NM_015455	chr5:180004898-180004957	CNOT6

A_32_P102252	9.407259	9.983906		chrX:080185910-080185969	
A_33_P3405285	13.048807	13.296448	NM_001080554	chr12:13236885-13236826	GSG1
A_23_P211985	5.3366733	5.1575065	NM_017719	chr3:43391802-43391861	SNRK
A_33_P3209869	7.4005136	7.756876	NM_018393	chr11:33094138-33094197	TCP11L1
A_24_P418536	12.336824	12.465715		chr1:119762160-119762099	
A_33_P3279620	5.944304	5.881277	AK094832	chr22:22630310-22630251	
A_33_P3420810	4.9878078	4.8447123		chr2:047274016-047273957	
A_23_P126120	8.051053	8.152431	NM_033319	chr1:173772182-173772123	CENPL
A_33_P3390853	9.748661	9.742853	NR_015367	chrX:56843930-56843989	LOC550643
A_33_P3365178	2.6637154	2.3900566		chr2:090520675-090520616	
A_33_P3414157	7.5742316	7.3223715	NM_024101	chr2:238463765-238463824	MLPH
A_32_P420009	5.5396805	5.672988	NM_147129	chr3:46711650-46711591	ALS2CL
A_24_P44279	5.138221	5.3965774	NM_153348	chr12:117468719-117468778	FBXW8
A_23_P69521	12.419372	12.639039	NM_006835	chr4:77969564-77969505	CCNI
A_33_P3383351	3.9066582	3.6626742	AK057231	chr9:137600825-137600884	COL5A1
A_23_P253723	6.365798	6.345915	NM_006583	chr4:110765590-110765649	RRH
A_23_P382835	5.583158	4.8625727	NM_002563	chr3:152555738-152555797	P2RY1
A_23_P56590	7.1998677	6.7382007	NM_006333	chr2:68273479-68273120	C1D
A_24_P136211	13.526618	13.548189		chr15:022440666-022440607	
A_23_P201432	9.613512	10.379764	NM_001126	chr1:244572746-244572687	ADSS
A_23_P139912	9.406553	9.383031	NM_002178	chr12:53494853-53494912	IGFBP6
A_23_P210496	6.373099	6.3129783	NM_022104	chr20:44576017-44576076	PCIF1
A_23_P157569	4.75111	5.5688586	NM_144650	chr8:67380656-67380715	ADHFE1
A_23_P259012	5.8049917	5.878341	NM_004656	chr3:52435946-52435887	BAP1
A_33_P3245709	7.1885424	7.541861	NM_020336	chr20:37203551-37203610	RALGAPB
A_24_P390928	5.049528	5.455374	NM_024108	chr19:45668207-45668148	TRAPPC6A
A_23_P210554	5.6187806	5.770011	NM_006038	chr20:48520440-48520381	SPATA2
A_32_P111639	6.2625356	6.866215	NM_031422	chr18:24496126-24496067	CHST9
A_23_P39616	6.9421725	7.231739	NM_006190	chr2:201775834-201775775	ORC2
A_23_P28279	6.4428096	6.5577555	NM_005735	chr2:98273139-98273080	ACTR1B
A_24_P465772	12.359329	12.519176	NR_026825	chr12:66152345-66152286	RPSAP52
A_33_P3362952	6.5195603	6.804432	AK125981	chr17:28905101-28905159	
A_23_P203030	6.8167267	6.391721	NM_001931	chr11:111934439-111934498	DLAT
A_33_P3328284	5.8132763	5.5270243		chr12:104680891-104680832	
A_33_P3247175	2.8491411	4.28365	NM_001114357	chr4:186370762-186370821	C4orf47
A_23_P391275	5.715406	5.903113	NM_013441	chr1:24859703-24861600	RCAN3
A_33_P3242493	5.9974184	5.971339	NM_006346	chr13:73357799-73357858	PIBF1
A_33_P3629247	6.315585	6.329952	NM_017844	chr2:241463466-241463407	ANKMY1
A_23_P158938	7.603293	7.6087685	NM_207012	chr10:75883302-75883243	AP3M1
A_33_P3210379	9.44701	9.7798195	NM_052863	chr5:180017165-180017106	SCGB3A1
A_23_P69683	8.543131	8.554378	NM_006323	chr4:110460882-110460941	SEC24B
A_23_P350234	5.172125	5.720145	NM_001012989	chrX:142967301-142967360	UBE2NL
A_23_P75516	5.9068985	5.9809566	NM_003626	chr11:70229626-70229685	PPFIA1
A_23_P15299	6.021639	6.033378	NM_012478	chr17:73842721-73842662	WBP2
A_33_P3285545	4.312472	4.5445795	NM_001305	chr7:73246907-73246966	CLDN4
A_23_P85543	7.681591	7.8766985	NM_007212	chr1:185069031-185069090	RNF2
A_23_P77630	7.657939	6.56385	NM_022818	chr16:87437440-87437499	MAP1LC3B
A_23_P367043	6.901546	7.324353	NR_003595	chr7:48885182-48885123	CDC14C
A_23_P61487	8.591741	8.196144	NM_018205	chr10:72058832-72058773	LRRC20
A_33_P3397568	10.726904	10.486401	NM_015955	chr2:32092958-32092899	MEMO1
A_33_P3237125	4.286493	4.1661677	NM_138797	chr22:38228002-38227943	ANKRD54
A_33_P3378384	3.5783343	3.6839693	NR_003260	chr15:100340036-100339983	DNM1P46
A_33_P3254811	2.7833986	3.8089044	NM_001025266	chr3:184795931-184795872	C3orf70
A_33_P3280739	10.045365	9.943419	NM_001172435	chr2:135927476-135927535	RAB3GAP1
A_33_P3402086	3.6588063	3.8806016		chr2:112766079-112766138	
A_32_P1381	12.695433	12.966296	M12623	chr1:26801948-26802007	HMGN2
A_32_P10100	13.062826	12.896294		chr1:205320559-205320618	
A_23_P28638	8.252111	7.948363	NM_017958	chr2:131904598-131904657	PLEKHB2
A_23_P75441	9.176886	9.301757	NM_032344	chr11:63997022-63997359	NUDT22

A_24_P68311	4.4103794	5.142111	NM_015180	chr14:64416714-64421485	SYNE2
A_23_P75220	7.1765575	7.345435	NM_031212	chr10:101370733-101370674	SLC25A28
A_33_P3309075	7.605247	8.139353	NM_001102426	chr2:101624138-101624079	TBC1D8
A_33_P3256778	3.7766414	3.274618	NM_000614	chr11:58393143-58393202	CNTF
A_23_P96827	3.9942424	4.348914	NM_001639	chr1:159558017-159558076	APCS
A_24_P85158	8.093321	8.485673	NM_012239	chr11:216207-216148	SIRT3
A_23_P31896	8.620392	8.148889	NM_003033	chr8:134471689-134471630	ST3GAL1
A_23_P89587	4.28466	3.4378512	NM_003396	chr17:44952629-44952688	WNT9B
A_33_P3365034	4.265895	3.630889	BX538082	chr2:128412469-128412528	GPR17
A_23_P91910	6.349939	5.8885803	NM_020353	chr3:145910678-145910619	PLSCR4
A_24_P319736	4.8228607	5.0410566	NM_002398	chr2:66799326-66799385	MEIS1
A_24_P287075	5.4477177	5.074171	NM_004579	chr11:64557236-64557072	MAP4K2
A_23_P37988	8.5276375	8.888013	NM_152727	chr16:57180203-57181491	CPNE2
A_33_P3354940	6.0971665	5.8585744	NM_000757	chr1:110472240-110472299	CSF1
A_23_P143127	10.358837	10.039858	NM_019063	chr2:42559572-42559631	EML4
A_33_P3419458	4.851305	4.6186304	NR_003022	chr1:12567392-12567451	SNORA59B
A_33_P3370109	6.206709	6.2958894	NM_001144879	chr8:82592967-82592908	IMPA1
A_23_P93623	10.630793	10.870545	NM_004231	chr7:128505739-128505798	ATP6V1F
A_23_P154740	7.48013	7.5550523	NM_018474	chr20:21209737-21213414	PLK1S1
A_24_P167473	13.627423	13.655237	NM_005719	chr12:110872809-110872750	ARPC3
A_24_P32935	4.7228813	5.2424836	NM_000803	chr11:71929635-71929694	FOLR2
A_23_P350074	5.9202585	5.6032095	NM_001039876	chr19:36494273-36494214	SYNE4
A_24_P67378	6.8761635	7.027101		chr3:172142255-172142213	XLOC_014512
A_23_P30784	6.437784	5.46678	NM_013375	chr6:26600159-26600218	ABT1
A_23_P147845	9.550677	9.814371	NM_015027	chr16:15131214-15131273	PDXDC1
A_23_P126241	8.081436	7.8921127	NM_003760	chr1:21133992-21133933	EIF4G3
A_33_P3226039	7.130479	6.7889667	BC019841	chr2:105882020-105881961	TGFBRAP1
A_33_P3370364	8.785	8.899315	NM_004248	chr10:120353020-120352961	PRLHR
A_33_P3424143	3.2264159	3.1903	BX248747	chr14:75150053-75149994	AREL1
A_23_P370666	3.165598	2.3900566	NM_080661	chr11:58723759-58723818	GLYATL1
A_24_P188878	12.683207	12.695433	NM_033625	chr4:109543285-109543344	RPL34
A_24_P412734	8.952944	9.075166	NM_173502	chr16:31151730-31151671	PRSS36
A_32_P701268	3.3267395	3.6408396	NM_053002	chr3:151150621-151150680	MED12L
A_24_P387869	7.528852	7.55612	NM_006256	chr1:89300815-89300874	PKN2
A_23_P49021	8.302849	8.527874	NM_025234	chr15:78580666-78578452	WDR61
A_23_P76761	9.028669	8.604095	NM_003384	chr14:97347589-97347648	VRK1
A_24_P66780	3.9449155	3.0931568	NM_001010872	chr6:54806024-54806083	FAM83B
A_33_P3216427	8.597941	8.471514	NM_001347	chr4:952735-952676	DGKQ
A_24_P269814	8.9854145	8.507337	NM_001001974	chr10:124191798-124191857	PLEKHA1
A_24_P942370	4.4196014	4.2073326	NM_003774	chr12:89913563-89913504	GALNT4
A_23_P157809	9.11074	9.368131	NM_012212	chr9:114341228-114341169	PTGR1
A_24_P325046	5.2238917	5.194413	AK126219	chr9:37186845-37186904	ZCCHC7
A_33_P3379526	3.9795947	3.9512343	AK127601	chr12:50271558-50271499	
A_33_P3771899	4.980983	4.762817	BQ278507		
A_33_P3278968	4.3457537	4.1276445	NM_002119	chr6:32977336-32977277	HLA-DOA
A_23_P51884	10.2194	10.157683	NM_153339	chr1:1246950-1247009	PUSL1
A_33_P3402350	8.280262	8.436545		chr1:120076628-120076569	
A_33_P3249748	9.4427595	8.90649	NM_016647	chr8:143818291-143818350	THEM6
A_32_P72110	5.788632	5.284636	NM_006505	chr19:45168363-45168422	PVR
A_23_P38365	7.6691027	7.7570696	NM_006852	chr17:60690154-60690213	TLK2
A_33_P3253144	5.1233215	4.59671	NM_024872	chr5:176930812-176930753	DOK3
A_33_P3303449	4.426459	4.970324	NM_001198688	chr1:66088604-66088663	LEPR
A_32_P154342	6.3191423	7.0888357	NM_180991	chr5:101570034-101569975	SLCO4C1
A_32_P212373	8.245282	8.132209	XR_247096	chr1:231664197-231663134	
A_23_P142146	13.835765	13.680013	NM_000979	chr19:49120023-49119441	RPL18
A_23_P339053	6.6331954	6.6191826	XR_243262	chr16:22098520-22098579	
A_33_P3306898	3.9830022	4.1287866	NM_012194	chr11:33689641-33689700	KIAA1549L
A_23_P337424	5.7501287	3.1130505	NM_001018067	chr1:67874360-67874301	SERBP1
A_23_P130343	10.233464	10.456314	NM_198991	chr18:24035570-24035511	KCTD1

A_23_P397238	7.3223715	7.346852	NM_054014	chr20:1352589-1352530	FKBP1A
A_24_P71153	4.2108936	4.6570654	NM_000437	chr1:26286933-26286874	PAFAH2
A_33_P3227556	7.012162	6.678634		chr5:000668788-000668847	
A_24_P156049	7.5726457	7.553856	NM_012319	chr18:33691111-33691052	SLC39A6
A_24_P274795	9.759247	9.2574215	NM_018719	chr7:21940891-21940832	CDCA7L
A_23_P45851	9.547407	9.693462	NM_033055	chr1:100548102-100548161	HIAT1
A_32_P205944	7.599153	7.8048964	NM_005054	chr2:110613489-110613548	RGPD5
A_33_P3215788	6.6191826	5.9984016	NM_001164315	chr2:97911709-97911768	ANKRD36
A_24_P287974	6.871932	6.969983	NM_001271875	chr17:55945576-55945517	CUEDC1
A_23_P138574	6.139403	6.426716	NM_001001976	chr10:123502774-123502715	ATE1
A_23_P334282	6.526367	6.686841	NM_017593	chr4:79800206-79800265	BMP2K
A_23_P64785	4.479533	4.389929	NM_152320	chr12:48736501-48736442	ZNF641
A_23_P135914	10.308033	10.253034	NM_012426	chr16:70606048-70606107	SF3B3
A_33_P3360301	10.038288	10.043356	NM_001199161	chr3:49145632-49145573	USP19
A_24_P379820	5.1483183	5.1494594	NM_030926	chr2:231742895-231742954	ITM2C
A_33_P3243175	9.4846115	9.358105	NM_018129	chr17:46026614-46026673	PNPO
A_23_P344594	4.1049156	4.2261314	NM_153217	chr5:72470166-72470225	TMEM174
A_33_P3219895	7.7837987	7.7967725	NM_001134774	chr11:66035241-66035300	KLC2
A_32_P218989	11.896206	12.342196	NM_004559	chr1:43167755-43167814	YBX1
A_23_P53866	8.852642	8.718428	NM_020751	chr13:40326225-40326284	COG6
A_32_P126311	7.404065	7.7739286	NM_173514	chr5:54922011-54921952	SLC38A9
A_23_P161237	10.136913	10.402258	NM_004193	chr10:104142491-104142550	GBF1
A_23_P154840	13.987585	13.974512	NM_000454	chr21:33040890-33040949	SOD1
A_23_P34888	4.5963774	4.4919133	NM_021797	chr1:111863116-111863175	CHIA
A_23_P408167	7.5475903	7.292723	NM_001004051	chrX:101972466-101972525	GPRASP2
A_33_P3381751	9.093838	8.641719	NM_003253	chr21:32490889-32490830	TIAM1
A_24_P203407	4.331462	4.4714527	NM_001080472	chr20:42935452-42935393	FITM2
A_23_P64129	10.177082	9.302583	NM_006410	chr11:20404824-20404883	HTATIP2
A_23_P58912	8.013339	8.141028	NM_006416	chr6:88221353-88221412	SLC35A1
A_23_P373649	11.2437935	11.464255	NM_006327	chr10:51592587-51592528	TIMM23
A_33_P3269844	8.506191	8.791383	NM_001013653	chr9:140063280-140063221	LRRC26
A_33_P3407235	6.599594	6.6631317		chrX:073351716-073351775	
A_32_P206698	9.665884	9.772176	NM_001826	chr1:154947230-154950471	CKS1B
A_23_P345591	12.258757	12.228779	NM_002787	chr7:42961518-42961459	PSMA2
A_23_P7083	6.913894	6.731195	NR_033339	chr4:1245821-1245880	CTBP1-AS2
A_23_P215154	6.856288	6.644842	NM_016118	chr7:151074937-151074996	NUB1
A_24_P149395	7.3968296	7.1894345	NM_015610	chr7:5273021-5273080	WIPI2
A_33_P3363665	6.5830164	6.715912	NM_001142587	chr1:41232296-41232355	NFYC
A_23_P171223	5.360458	5.2715206	NM_022101	chrX:118678409-118678350	CXorf56
A_33_P3375613	12.162217	12.32571	NM_001204173	chr8:67772367-67772426	C8orf44-SGK3
A_23_P161769	10.553964	11.291861	NM_021603	chr11:117691003-117690944	FXYD2
A_23_P76851	8.805803	8.826118	NM_001039619	chr14:23390227-23390168	PRMT5
A_24_P34944	4.6966443	4.7670393	NM_032094	chr5:140812359-140812418	PCDHGA12
A_33_P3312366	8.278589	8.133371	NM_003935	chr22:22311463-22311404	TOP3B
A_33_P3384628	3.4635155	2.9703932		chr21:15278286-15278227	XLOC_014512
A_33_P3271395	4.784437	4.688001	NR_024489	chr1:2281912-2281853	LOC100129534
A_23_P88731	8.829409	8.756769	NM_002875	chr15:41023607-41023666	RAD51
A_33_P3406861	3.6402662	3.8026693	NM_001270771	chr4:186545375-186545316	SORBS2
A_23_P7212	4.522993	5.5550666	NM_000204	chr4:110662123-110662064	CFI
A_32_P67259	9.99242	10.1958885	NM_004168	chr5:251151-251210	SDHA
A_33_P3734378	3.6065817	2.3900566	NR_027118	chr7:41818918-41818976	INHBA-AS1
A_33_P3412353	9.365152	9.538067	NM_152943	chr12:133781677-133781736	ZNF268
A_33_P3259821	5.885212	5.8730683	NM_001130050	chr13:99512530-99512471	DOCK9
A_33_P3242039	6.4880457	6.382735		chr12:106633792-106633851	
A_32_P42780	5.818388	5.9379463	NR_024565	chr18:32890387-32890446	ZNF271
A_24_P175059	6.3336444	6.063493	NM_004849	chr6:106632811-106632752	ATG5
A_33_P3309804	4.49024	4.5153236	NM_014335	chr15:49172321-49172380	EID1
A_23_P165984	5.5509944	5.315076	NM_080752	chr20:44507335-44507394	ZSWIM3
A_33_P3290487	9.635812	9.371988	NM_001172668	chr16:31072231-31072172	ZNF668

A_23_P347508	8.197365	7.9386106	NM_023077	chr1:53152605-53152546	SELRC1
A_24_P342086	6.7620444	6.95589	NM_001270455	chr16:69943511-69943570	WWP2
A_23_P91891	12.557169	12.467993	NM_004766	chr3:139076652-139076593	COPB2
A_23_P143414	12.724985	12.886798	NM_080748	chr20:34288841-34288900	ROMO1
A_23_P22433	8.266755	8.436712	NM_006915	chrX:46741667-46741726	RP2
A_24_P385611	8.346597	8.531144	NM_003113	chr2:231331037-231331861	SP100
A_33_P3348194	4.6782546	4.5190716		chr6:082897078-082897019	
A_24_P289273	3.652008	3.5109844	BC008094	chr10:86008779-86010179	RGR
A_33_P3342443	4.913327	4.720375	NM_001277335	chr7:102128970-102128911	RASA4B
A_24_P89426	4.2142463	3.6902957	NM_019101	chr6:31625018-31625158	APOM
A_33_P3363091	5.434152	5.09132	U25801	chr16:70808225-70808166	VAC14
A_33_P3351101	5.245548	5.0406623	NM_173555	chr10:71898108-71898049	TYSND1
A_33_P3351606	4.7760625	4.851436	NR_034102	chr8:65290622-65290681	LINC00966
A_23_P154875	11.23222	11.478488	NM_012105	chr21:42647696-42647755	BACE2
A_33_P3282614	10.006217	10.225275	NM_001256699	chr9:140147856-140147915	C9orf173
A_33_P3236902	6.64179	6.7485	XM_005276004	chr7:100550075-100550134	
A_32_P6344	9.215125	9.076658	NM_003010	chr17:12046983-12047042	MAP2K4
A_33_P3242778	5.278888	5.047587	AK127110	chr2:201769662-201769721	LOC100129888
A_33_P3235370	6.147483	6.270461	NM_021061	chr8:146112285-146112226	ZNF250
A_23_P4474	9.775462	9.88731	NM_016097	chr18:44681930-44681874	IER3IP1
A_33_P3258061	8.514343	8.962023	NM_001145028	chr19:14164238-14164179	PALM3
A_33_P3236858	11.171302	11.327766	NM_001042454	chr16:31489205-31489264	TGFB1I1
A_23_P99076	4.3468695	4.142168	NM_005042	chr12:11083298-11083357	PRH2
A_23_P88249	6.8265624	7.1273365	NM_001077351	chr14:23370569-23370510	RBM23
A_23_P13344	13.874355	14.025549	NM_001404	chr11:62327560-62327270	EEF1G
A_33_P3280945	10.785187	10.592379	NR_036473	chr1:28836041-28836100	SNHG3
A_23_P47004	7.6795444	8.264774	NM_018180	chr10:127527612-127526940	DHX32
A_24_P937306	4.5129166	4.7261443	XM_005274614	chrX:15849163-15849104	AP1S2
A_23_P405707	6.4797554	6.1765485	BC128456	chrX:39931331-39931272	BCOR
A_33_P3311637	10.142222	9.841906	NM_016106	chr14:31191705-31191764	SCFD1
A_23_P70818	9.716117	9.774015	NM_005631	chr7:128853237-128853296	SMO
A_24_P206736	8.250126	8.44137	NM_003442	chr11:9549384-9549443	ZNF143
A_33_P3280575	5.7839007	6.171033	CU691877	chr13:024895873-024895814	
A_33_P3344405	6.5396442	6.423975	NM_001145785	chr19:19256457-19256398	MEF2B
A_33_P3370600	4.328203	4.5559044	NM_001162997	chr17:73643577-73643636	SMIM6
A_23_P76102	6.123601	5.9269743	NM_005811	chr12:56143449-56143508	GDF11
A_33_P3320782	4.7116156	4.7328334	NM_000333	chr3:63986252-63986311	ATXN7
A_23_P327370	5.627821	6.0011377	NM_017712	chr19:18480282-18480341	PGPEP1
A_23_P433798	3.5362625	3.2056952	NM_024825	chr19:14043548-14043489	PODNL1
A_33_P3221868	5.687932	5.973379	NM_001173990	chr11:61165387-61165446	TMEM216
A_33_P3233871	7.733671	7.466042	NM_000505	chr5:176829201-176829142	F12
A_33_P3724157	4.122599	4.0612245	NM_198440	chr22:24177425-24177366	DERL3
A_24_P159312	3.751614	4.1695805	NM_001076785	chr16:68334665-68334724	SLC7A6
A_33_P3307957	5.4563956	5.654828	NM_001122674	chr1:94941224-94941283	ABCD3
A_32_P47701	13.660247	14.154367	NM_001402	chr6:74228782-74228723	EEF1A1
A_24_P117954	6.601973	6.429637	NM_194281	chr18:33059356-33059297	INO80C
A_23_P435444	5.171521	5.5024195	NM_032087	chr5:140764880-140764939	PCDHGA7
A_33_P3358233	5.5907073	5.4112873	NM_006617	chr1:156639481-156639422	NES
A_33_P3255964	3.5630393	3.2763505		chr1:111033818-111033877	CYMP
A_32_P42054	7.1812797	6.936035	NM_194249	chr5:140050450-140050391	DND1
A_23_P379614	10.087898	9.645008	NM_007280	chr15:41601624-41601565	OIP5
A_33_P3418710	4.099982	4.0266147	NM_001080830	chr1:12837810-12837869	PRAMEF12
A_33_P3440264	5.424179	5.6187806	NR_034118	chr22:42520486-42520545	NDUFA6-AS1
A_23_P100240	8.218985	8.648005	NM_004062	chr16:66942101-66942042	CDH16
A_33_P3718269	2.8398175	3.4051032	AL389942	chr5:159914327-159914386	MIR146A
A_32_P46981	8.257775	8.1438465	NM_001136180	chr18:77726642-77728111	HSBP1L1
A_24_P316939	8.418493	8.347288	NM_001137550	chr2:238688558-238688617	LRRFIP1
A_33_P3391105	5.524389	5.6635776	NM_002332	chr12:57539213-57539272	LRP1
A_33_P3342469	3.4903047	3.5881126	NM_001005187	chr11:123813633-123813574	OR6T1

A_33_P3809328	4.227016	4.4341288		chr3:152047851-152047792	
A_33_P3288859	9.633379	9.46782	NM_207327	chr22:46639970-46639911	CDPF1
A_33_P3216372	8.734887	8.966146	NM_006633	chr5:76003795-76003854	IQGAP2
A_33_P3292179	14.277659	14.2308	NM_080283	chr17:66971122-66971063	ABCA9
A_33_P3386723	8.141565	7.6472654		chr6:085998178-085998237	
A_23_P405129	5.440761	4.49024	NM_000428	chr14:74965511-74965452	LTBP2
A_33_P3308101	6.502647	7.0940065	XR_242363	chr7:56886608-56886601	
A_23_P385081	4.543372	4.9366612	NM_005697	chr15:75142889-75141018	SCAMP2
A_23_P45917	12.295363	12.320886	NM_001826	chr1:154951527-154951586	CKS1B
A_33_P3355783	3.6532593	3.0026815	XR_247231	chr11:61534787-61534846	LOC100129473
A_24_P944827	6.3567286	5.948743	NM_006395	chr3:11598763-11598822	ATG7
A_23_P433152	7.270641	7.3197293	NM_173487	chr4:130032875-130032934	C4orf33
A_23_P59528	7.8982434	7.9246955	NM_020186	chr7:96810787-96810846	ACN9
A_23_P94159	8.417381	8.422638	NM_183421	chr8:418943-419002	FBXO25
A_33_P3375910	4.7820888	4.479124		chr17:073760698-073760639	
A_33_P3235189	8.640074	8.627401	NM_152345	chr17:27939231-27939290	ANKRD13B
A_33_P3238074	8.535971	9.241397	NM_019008	chr22:39910169-39910228	MIEF1
A_33_P3243364	5.961508	5.438861	NM_005236	chr16:14042239-14042298	ERCC4
A_33_P3390643	5.6412067	6.0897393	NM_015506	chr1:45974686-45974745	MMACHC
A_33_P3310864	4.05003	4.0734673	NM_001131064	chr11:47267283-47267224	ACP2
A_23_P315789	6.779884	6.2692623	NM_000538	chr13:37402541-37402600	RFXAP
A_33_P3388331	5.4761496	6.099934	NM_005766	chr13:99076844-99076903	FARP1
A_33_P3423969	6.78486	6.7092476	NM_001485	chr2:237074384-237074325	GBX2
A_33_P3212172	6.1813545	5.8266964	NM_024798	chr15:64449621-64449680	SNX22
A_33_P3349776	7.6598215	7.7738504	NM_144703	chr20:60705602-60705661	LSM14B
A_23_P159833	12.33431	12.31544	NM_004541	chrX:119007330-119010509	NDUFA1
A_33_P3216232	9.368418	9.157114	NM_004763	chr2:9545927-9545868	ITGB1BP1
A_23_P78372	5.973379	5.9936085	NM_005131	chr18:226901-226842	THOC1
A_33_P3228295	3.7499237	4.0188026		chr9:4827278-4827337	RCL1
A_23_P65532	3.8051293	4.3463597	NM_021255	chr14:56767466-56767525	PELI2
A_33_P3415345	4.7328334	5.0322733	NM_001135745	chr2:202626185-202626126	ALS2
A_24_P305345	4.3367968	4.0147123	NM_021155	chr19:7805407-7805348	CD209
A_33_P3398251	3.5570598	3.8216333	NM_014009	chrX:49107469-49107410	FOXP3
A_23_P39465	2.825122	3.6594696	NM_004335	chr19:17514113-17514054	BST2
A_24_P111242	5.733868	4.37985	NM_148893	chr11:22843736-22843677	SVIP
A_24_P563545	7.1732907	7.2848344	NM_133496	chr1:101444465-101444524	SLC30A7
A_23_P307328	7.8330355	7.673251	NM_007331	chr4:1943382-1943441	WHSC1
A_32_P47643	5.3188834	4.644505	NM_001077710	chr2:38915-38856	FAM110C
A_23_P69970	3.9727883	4.2733684	NM_178424	chr5:157053521-157053462	SOX30
A_24_P241330	7.804492	8.070583	NM_024824	chr14:89076112-89077201	ZC3H14
A_33_P3875565	5.5910397	5.5304294	NR_038394	chr20:22055232-22055291	LOC100270679
A_23_P406448	6.5691133	6.4129434	NM_016370	chrX:103080109-103080050	RAB9B
A_33_P3241489	9.25904	9.099537	NR_037879	chr2:37431823-37431882	CEBPZ-AS1
A_23_P131365	10.786566	10.861256	NM_005006	chr2:206988933-206988874	NDUFS1
A_23_P121282	3.870725	3.3650627	NM_001008269	chr3:48658338-48658279	TMEM89
A_33_P3211014	3.5959697	3.7737823	AY203951	chr16:087492260-087492201	
A_23_P22134	9.702502	9.826691	NM_001717	chr15:83925019-83924960	BNC1
A_33_P3411477	8.907279	8.891129	NM_001001414	chr19:39692461-39692520	NCCRP1
A_33_P3232624	4.2518497	3.1075723	NR_033908	chr1:708428-708370	LOC100288069
A_32_P25514	4.066455	3.2755735	NM_198904	chr5:161582441-161582500	GABRG2
A_33_P3626301	7.109154	7.138208	NR_002833	chr7:35121190-35121131	DPY19L2P1
A_33_P3263379	5.7933474	5.5097127	NR_003045	chr20:17943412-17943353	SNORD17
A_33_P3275290	7.7117677	8.15397	NM_001010983	chr3:52729306-52729247	GLT8D1
A_33_P3274562	4.004188	4.0790615	NM_025243	chr2:228550641-228550582	SLC19A3
A_33_P3361217	3.7146046	4.0899377		chr5:092923207-092923148	
A_23_P388681	7.7900705	7.974167	NM_001419	chr19:8028338-8028279	ELAVL1
A_32_P223017	7.518671	7.5094757	NM_004672	chr1:27682544-27682205	MAP3K6
A_24_P944991	4.44366	4.8348923	NM_173829	chr5:64018946-64018887	SREK1IP1
A_24_P229726	2.9825509	2.3900566		chr14:059294919-059294858	

A_23_P40936	7.321117	7.180576	NM_003298	chr3:15084530-15084589	NR2C2
A_33_P3377519	7.0218616	6.5646777		chr7:27185175-27185116	HOXA6
A_23_P108294	8.615656	8.373526	NM_177543	chr19:282253-282194	PPAP2C
A_23_P252276	3.618431	2.3900566	NM_017700	chr4:106552377-106552436	ARHGEF38
A_23_P127175	10.508797	10.484283	NM_020150	chr10:71912267-71912208	SAR1A
A_32_P141262	9.2067795	9.260273	NM_054026	chr8:17092310-17092251	CNOT7
A_23_P151179	6.144804	6.3179507	NM_022895	chr12:121441566-121441507	C12orf43
A_23_P309950	4.3486643	3.7436988	AL833126	chr10:102685825-102685884	FAM178A
A_33_P3239298	10.886576	11.144929	BC036534	chr22:18909818-18909759	PRODH
A_33_P3353496	12.179079	12.168908	NM_153368	chr10:35897700-35897759	GJD4
A_23_P67864	9.217501	9.09079	NM_004036	chr2:25042332-25042273	ADCY3
A_23_P327069	8.755757	8.792109	NM_014743	chr4:6884726-6884785	KIAA0232
A_24_P143171	4.4228616	4.640093	NM_031442	chrX:34646240-34646181	TMEM47
A_32_P101301	10.8497	10.559628	NM_006590	chr2:85876306-85876365	USP39
A_33_P3289128	5.6595626	5.709736	NM_001137601	chr14:105270800-105270859	ZBTB42
A_24_P305570	8.968014	8.372268	NM_018993	chr20:19982541-19982600	RIN2
A_33_P3303850	4.251352	4.068201	AL834189	chr8:17129512-17129571	VPS37A
A_33_P3344991	6.512328	6.538065	AK122833	chr17:34501150-34501091	
A_24_P48898	11.148535	11.307478	NM_145637	chr22:36622708-36622649	APOL2
A_23_P364837	4.596497	4.669965	NM_015378	chr1:12571638-12571697	VPS13D
A_33_P3355831	4.3622527	4.1650724	NM_004220	chr16:3192087-3192146	ZNF213
A_23_P54556	8.880371	8.817995	NM_014048	chr16:14360546-14360605	MKL2
A_33_P3404360	5.470572	4.4096317		chr13:019308250-019308309	
A_33_P3712008	4.0462236	3.7819698	AL512720	chr4:15485657-15485716	DKFZp547J222
A_23_P55515	5.544874	5.716335	NM_003799	chr18:13742604-13746230	RNMT
A_23_P424582	7.833664	7.835519	NM_030652	chr6:32135924-32135986	EGFL8
A_33_P3333975	9.880226	9.894906	NM_001286835	chr9:70431992-70431987	CBWD5
A_23_P77201	5.1055794	5.265937	NM_032499	chr15:37102058-37102117	C15orf41
A_33_P3369894	9.698454	10.135677	AK125616	chr1:145943914-145943855	
A_33_P3268892	4.363854	3.0069628	NM_001190468	chr5:37814012-37813953	GDNF
A_33_P3311493	2.7412295	2.3900566	NR_026836	chr12:72656394-72656335	TRHDE-AS1
A_33_P3232006	8.322093	8.260529	NM_014235	chrX:153712300-153712241	UBL4A
A_23_P30799	9.080538	9.233384	NM_021018	chr6:26250647-26250588	HIST1H3F
A_33_P3390570	10.490665	10.740824	NM_005933	chr11:118348856-118348915	KMT2A
A_33_P3304133	9.428607	10.170838	NM_138342	chr11:134245578-134245637	GLB1L2
A_33_P3282005	4.78407	5.0429254	NM_020335	chr1:160398186-160398245	VANGL2
A_33_P3296442	7.9872103	8.542141		chr14:088421486-088421545	
A_24_P161018	8.020144	8.290501	NM_017554	chr3:122437444-122437503	PARP14
A_24_P142442	10.742922	10.8533325	NM_015584	chr17:26674388-26674329	POLDIP2
A_23_P500892	8.309002	8.28485	NM_003320	chr11:8127140-8127199	TUB
A_23_P209735	6.562399	6.477784	NM_025139	chr2:232156115-232160928	ARMC9
A_33_P3239954	7.5411205	7.8912153	NM_005151	chr18:213680-213739	USP14
A_33_P3272090	4.2446055	4.571393	NM_014644	chr1:144852259-144852200	PDE4DIP
A_23_P159688	5.2637444	5.6146317	NM_002536	chrX:48420306-48420364	TBC1D25
A_23_P15734	3.6287117	4.4942994	NM_000226	chr17:39722110-39722093	KRT9
A_24_P208737	6.5744877	6.5046453	NR_073566	chr3:10334228-10334287	GHRLOS
A_24_P323598	9.290873	9.328628	NM_001017420	chr8:27662101-27662160	ESCO2
A_24_P257348	8.350047	8.675965	NM_006407	chr3:69154416-69154475	ARL6IP5
A_33_P3257486	4.952818	4.7434745	XM_001719621	chr22:22730666-22730725	
A_24_P279797	9.241836	9.242172	NM_001031727	chr19:13885017-13885076	MRI1
A_33_P3511265	2.6625292	2.3900566	NM_006475	chr13:38137425-38137366	POSTN
A_33_P3280360	3.919983	3.075666	AK128800	chr20:45378939-45378880	LOC100133857
A_24_P144163	11.63739	11.804407		chr17:000560504-000560443	
A_23_P72537	10.520412	10.601184	NM_004208	chrX:129263459-129263400	AIFM1
A_23_P35219	9.341103	9.790903	NM_002497	chr1:211836920-211836861	NEK2
A_23_P92754	4.955463	5.0330067	NM_213647	chr5:176523121-176523180	FGFR4
A_23_P502641	7.5182614	7.741705	NM_032970	chr3:42599114-42597473	SEC22C
A_33_P3425296	7.5856733	7.329007	NR_003525	chr10:27536289-27536230	LRRC37A6P
A_23_P129569	6.2480726	6.449164	NM_024675	chr16:23625395-23625336	PALB2

A_33_P3354564	6.6142893	6.0293546	NM_001083112	chr2:157439359-157439418	GPD2
A_23_P36266	8.819264	9.182217	NM_003477	chr11:35016692-35016751	PDHX
A_23_P160800	3.5402727	3.0072877	NM_021969	chr1:27238242-27238183	NR0B2
A_33_P3221563	7.8766985	7.456062	NM_024742	chr16:31478426-31478485	ARMC5
A_23_P201295	3.6567376	3.0557806	NM_017766	chr1:10707315-10707263	CASZ1
A_33_P3416707	4.931273	5.305557		chr8:141744855-141744796	
A_33_P3231076	5.0678864	5.3385973	NR_024477	chr7:154738429-154738488	PAXIP1-AS2
A_32_P186981	14.411888	14.393929	NM_000985	chr18:47015801-47015742	RPL17
A_33_P3361182	7.807365	7.810819	NM_014976	chr10:105205498-105205557	PDCD11
A_33_P3294836	6.4528074	6.853625	NM_024573	chr6:151789520-151789579	C6orf211
A_33_P3709327	4.787123	5.15201	NR_034138	chr4:66559044-66559103	LOC100144602
A_32_P35220	10.093741	10.187136	NM_001024916	chr9:70486869-70486810	CBWD5
A_33_P3526458	4.5512533	3.2507558	BC043208	chr10:45666706-45666765	
A_33_P3329597	10.10697	9.804688	NM_212550	chr19:45684998-45685057	BLOC1S3
A_33_P3298552	7.7998257	8.146942		chr17:079092859-079092800	
A_32_P155364	15.384792	15.509981		chr8:74203307-74202911	RPL7
A_24_P414786	9.193922	9.1349325	NM_015891	chr6:110553163-110553222	CDC40
A_24_P136641	7.025795	7.090821	NR_033991	chr9:32552870-32552929	TOPORS-AS1
A_23_P149626	6.6317334	6.3983326	NM_198681	chr1:6527281-6527222	PLEKHG5
A_23_P153022	8.845123	9.070186	NM_033184	chr17:39221369-39221320	KRTAP2-4
A_32_P234827	10.320704	9.980834	NM_018120	chr8:66515930-66515871	ARMC1
A_23_P137665	2.3221061	3.1504407	NM_001276	chr1:203148255-203148196	CHI3L1
A_23_P145134	9.034078	9.258051	NM_007045	chr6:167453724-167453783	FGFR1OP
A_23_P411723	3.708399	4.484213	NM_002655	chr8:57074013-57073954	PLAG1
A_33_P3234377	12.057644	12.198631		chr8:130877276-130877335	
A_33_P3413541	8.97832	9.11187	NR_001575	chr5:54824933-54824874	RNF138P1
A_32_P63848	8.649474	8.311843	NM_000436	chr5:41730530-41730471	OXCT1
A_33_P3372844	11.277691	11.015091	NM_019082	chr7:44605466-44605407	DDX56
A_24_P673786	8.549156	8.559323	NM_005028	chr10:22823804-22823766	PIP4K2A
A_33_P3274069	10.227705	10.450223	NM_012265	chr22:29655925-29655866	RHBDD3
A_33_P3316493	4.7258663	4.670289	NM_014149	chr7:134874171-134874112	WDR91
A_32_P202759	5.640936	5.531018	NM_177454	chr2:187611881-187611940	FAM171B
A_24_P941051	7.411255	5.863	NM_015235	chr10:53455459-53455400	CSTF2T
A_33_P3324805	4.984305	5.051601		chr3:063738330-063738271	
A_23_P256059	13.59614	13.781273	NR_029406	chr17:81188142-81188201	FLJ43681
A_24_P926367	10.868124	10.757578	NM_005119	chr1:36770822-36770881	THRAP3
A_24_P305467	8.365173	7.4612207	NM_017660	chr19:19619473-19619532	GATAD2A
A_24_P942211	7.487316	8.0791445	NM_182838	chr1:1659358-1659299	SLC35E2
A_23_P329772	9.035948	8.832397	NM_013245	chr16:69358718-69358777	VPS4A
A_23_P118516	9.108001	9.457227	NM_016078	chr17:18709283-18709342	TVP23B
A_33_P3224362	4.4853015	4.7130194	NM_020528	chr21:47359978-47360037	PCBP3
A_32_P208403	6.132492	6.088787	NM_053064	chr14:52436151-52436210	GNG2
A_23_P120629	3.5622792	3.2063873	NM_001672	chr20:32848311-32850667	ASIP
A_24_P96234	5.728876	5.7422047	NM_031209	chr19:10822939-10823251	QTRT1
A_33_P3571254	5.8402996	5.288814	AL049782	chr13:33066769-33066710	MINOS1P1
A_32_P167705	3.1406722	2.3900566	NM_024783	chr11:47681302-47681243	AGBL2
A_33_P3220045	3.4193766	3.1801	AK126136	chr9:117138769-117138710	AKNA
A_33_P3424467	5.839156	5.771597	NM_152609	chr1:246831802-246831861	CNST
A_24_P50801	4.294564	3.4785054	NM_201264	chr2:206608080-206608139	NRP2
A_23_P215341	7.6612253	7.6109147	NM_017946	chr7:30053880-30053821	FKBP14
A_24_P254551	3.9871426	3.2422993	NM_015185	chrX:62855397-62855338	ARHGEF9
A_23_P94319	7.0586243	6.694638	NM_014867	chr8:1955034-1955093	KBTBD11
A_33_P3241043	16.30209	16.23797		chr6:166478936-166478995	
A_24_P396702	7.210659	7.41749	NM_014880	chr2:160625659-160625600	CD302
A_24_P913227	5.4199696	5.3027515	BC010944	chr5:137542195-137542136	CDC23
A_33_P3260034	3.5852015	3.8763835		chr9:133973541-133973600	
A_23_P416581	4.657349	4.934128	NM_002073	chr22:23467137-23467196	GNAZ
A_24_P43681	5.3766255	5.195775	NM_014063	chr7:44100381-44100440	DBNL
A_24_P623782	2.6340287	3.5048976	NM_001005217	chr4:190946047-190945988	FRG2

A_23_P14928	8.890244	9.010069	NM_020312	chr16:57494748-57494807	COQ9
A_33_P3370521	5.250826	5.823571	AV727532	chr1:120876554-120876495	
A_23_P154315	10.571863	10.319694	NM_182640	chr2:105716308-105716367	MRPS9
A_23_P69617	5.352405	5.816036	NM_003728	chr4:96089773-96089714	UNC5C
A_32_P181297	4.3726587	4.1025653	NR_002330	chr7:116592754-116592695	ST7-AS1
A_33_P3306983	12.688326	12.7016535	NM_170746	chr11:57509630-57509689	C11orf31
A_33_P3368453	9.505235	9.698454	NM_000247	chr6:31382847-31382906	MICA
A_33_P3379406	5.26174	4.8772283	BC071853	chr2:11721329-11721388	GREB1
A_24_P145066	8.349151	8.238773	NM_183416	chr1:10366262-10366321	KIF1B
A_23_P71319	10.039202	10.085754	NM_004462	chr8:11696201-11696260	FDFT1
A_33_P3293202	6.495655	6.912651	NM_177965	chr8:96258592-96258533	C8orf37
A_23_P435610	6.457532	6.7634325	NM_015047	chr1:19545114-19545055	EMC1
A_23_P164000	7.397587	7.2347803	NM_015670	chr17:7474750-7474809	SENP3
A_23_P350059	12.586741	12.702684	NR_003242	chr1:144612091-144612032	PFN1P2
A_24_P188941	15.462484	15.114832	NM_002520	chr5:170837789-170837848	NPM1
A_32_P54553	3.4254012	3.6149092	XM_003960815	chr22:20723843-20723784	USP41
A_33_P3304516	3.805847	3.7735848	NM_021035	chr20:47872404-47872345	ZNFX1
A_33_P3280606	6.419811	6.4995375	BX641018	chr20:56838506-56838447	
A_33_P3307728	3.8381178	3.3439555	NM_001005405	chr11:71293371-71293312	KRTAP5-11
A_33_P3220025	3.827137	3.5418892	NM_001145004	chr15:20737145-20737094	GOLGA6L6
A_33_P3323298	8.687806	7.5124016	NM_002228	chr1:59246570-59246511	JUN
A_23_P2446	8.567085	8.312256	NM_014254	chr12:64202507-64202566	TMEM5
A_23_P64879	4.052773	4.204281	NM_004982	chr12:21918724-21918665	KCNJ8
A_24_P350307	10.594658	10.508126		chr15:078953807-078953747	
A_24_P146211	9.036919	8.965763	NM_021063	chr6:26158474-26158533	HIST1H2BD
A_23_P148194	11.393358	11.623884	NM_018269	chr2:3502720-3502661	ADI1
A_24_P44931	6.248504	6.254915	NM_032868	chr19:4354348-4354407	MPND
A_33_P3352782	6.8745756	6.4713526	NM_024562	chr16:69119024-69119083	TANGO6
A_33_P3332348	14.288418	14.458849	NR_002715	chr14:50053508-50053567	RN7SL1
A_23_P343935	8.749625	9.547588	NM_022051	chr1:231502208-231502149	EGLN1
A_23_P205778	6.6742096	6.9533834	NM_016194	chr15:52414907-52414848	GNB5
A_24_P113572	5.761147	5.5816956	NM_138415	chr22:45277272-45277213	PHF21B
A_33_P3330663	5.8277535	5.916657		chr8:048114175-048114116	
A_23_P5703	4.6587615	5.3315516	NM_175735	chr2:99860483-99858908	LYG2
A_33_P3375755	4.019458	4.022103		chr16:001151381-001151322	
A_33_P3714477	4.3171067	3.9927993		chr7:150144879-150144938	LINC00996
A_23_P399501	12.437426	12.338233	NM_182470	chr15:72491699-72491640	PKM
A_33_P3325262	4.782546	4.56241	NM_005629	chrX:152960189-152960248	SLC6A8
A_33_P3268664	3.6222463	3.176392		chr2:90229353-90229412	
A_33_P3377750	3.3662052	3.2693374	NM_177417	chr19:45854215-45854274	KLC3
A_23_P163955	7.52565	7.415049	NM_007169	chr17:17415847-17412825	PEMT
A_23_P337201	11.45661	11.294666	NM_006947	chr4:57357602-57357661	SRP72
A_24_P367421	8.507699	8.124834		chr15:32783150-32783124	LOC101929887
A_23_P208009	10.200877	10.224681	NM_033280	chr18:56824892-56825917	SEC11C
A_23_P37327	8.554513	8.424528	NM_005050	chr14:74752793-74752734	ABCD4
A_33_P3398196	5.299641	5.848717		chr17:58285098-58285090	USP32
A_33_P3323323	4.192021	3.8729494	NM_198458	chr19:58865784-58865725	ZNF497
A_23_P106204	5.334029	5.5477896	NM_145870	chr14:77797434-77797493	GSTZ1
A_23_P143662	7.1028876	7.273517	NM_014338	chr22:32015337-32015278	PISD
A_33_P3317123	11.0444355	11.130627	NM_004922	chr10:75531826-75531885	SEC24C
A_23_P146855	3.646748	3.3348079	NM_001044370	chr22:43902652-43902711	MPPED1
A_32_P96807	7.977718	8.028448	NM_172071	chr1:173900682-173900623	RC3H1
A_23_P54540	10.093049	9.497707	NM_001013703	chr15:40326645-40327286	EIF2AK4
A_33_P3893191	9.798552	9.800023	NR_036635	chr2:63345141-63345082	DBIL5P2
A_23_P395374	11.270389	11.227951	NM_003539	chr6:26189011-26188952	HIST1H4D
A_33_P3210423	4.104774	3.2946396	NM_004774	chr17:37571339-37571280	MED1
A_33_P3399733	4.9058075	4.7854495	BC101193	chr4:85887742-85887801	WDFY3-AS2
A_33_P3365134	7.385669	7.303387	NM_015316	chr14:104200210-104200151	PPP1R13B
A_23_P140960	11.1641865	10.876233	NM_005003	chr16:23596691-23593672	NDUFAB1

A_24_P397386	5.8436556	6.5505505	NM_002310	chr5:38476001-38475942	LIFR
A_23_P135326	12.020133	12.002573	NM_017443	chr9:116169769-116169710	POLE3
A_24_P627306	8.381455	8.396287	NM_018217	chr20:33703427-33703368	EDEM2
A_23_P43175	11.706069	11.470644	NM_144710	chr2:110300744-110300685	SEPT10
A_24_P205019	7.4187365	7.4059706	NM_021061	chr8:146106827-146106768	ZNF250
A_33_P3375934	7.298196	7.182988	NM_005746	chr7:105888852-105888793	NAMPT
A_23_P311010	4.194896	4.4380956	NM_005840	chrX:155012038-155012097	SPRY3
A_33_P3345643	6.8053393	6.2053976		chr5:711383-711324	ZDHHC11B
A_32_P109604	4.8481255	4.564444	NR_046228	chr1:142713108-142713049	ANKRD20A12P
A_33_P3491294	7.565845	7.7152395	NM_015944	chr16:2579619-2579677	AMDHD2
A_32_P456537	10.18319	10.4172325	NM_030752	chr6:160199547-160199520	TCP1
A_33_P3338417	5.7383823	5.658038	NM_201263	chr1:119573925-119573866	WARS2
A_33_P3299739	7.6011324	7.671979	NM_001123395	chr1:43200754-43200695	CLDN19
A_23_P49924	6.6716356	6.7996545	NM_052935	chr17:39992127-39991482	NT5C3B
A_23_P502808	6.877927	6.760496	NM_178013	chr14:94203650-94203591	PRIMA1
A_33_P3364493	4.967329	4.988945		chr9:068410624-068410565	
A_33_P3327888	3.5415847	2.3900566	AF220264	chr8:140944670-140944729	C8orf17
A_33_P3380236	2.3221061	2.3900566		chr1:59348711-59348652	
A_23_P334955	6.0130033	5.2225866	NM_053279	chr8:11279717-11279658	FAM167A
A_33_P3397655	5.6218414	5.4875937	NM_001166208	chr5:150031836-150031895	SYNPO
A_33_P3385461	8.344919	8.288033	NM_024332	chrX:154348363-154348422	BRCC3
A_23_P208540	11.209715	11.413149	NM_004542	chr19:54609251-54609310	NDUFA3
A_33_P3309365	6.949849	7.2832785		chr1:53904127-53904068	SLC25A3P1
A_24_P94651	10.076309	9.880409	NM_006321	chr3:49022899-49022958	ARIH2
A_32_P148726	4.9171495	4.7502913	AK127789	chr9:66557350-66557409	MGC21881
A_33_P3412184	5.2253885	5.4828987		chr19:56129574-56129633	ZNF865
A_24_P116535	3.8858366	4.4657393	NM_002428	chr16:58080484-58080543	MMP15
A_33_P3760125	12.8388405	12.875509	AK026667	chrY:19664757-19664698	
A_23_P218423	9.681814	9.737185	NM_148887	chr17:45901134-45901075	MRPL10
A_24_P118591	13.903305	13.909961		chr6:159947384-159947324	
A_24_P89971	12.23697	12.182161	NM_033161	chr9:136228402-136228343	SURF4
A_24_P342591	6.714589	7.0686193	NM_012102	chr1:8413045-8412986	RERE
A_33_P3382344	3.4533038	3.1397343	CD694834	chr14:62536295-62536236	
A_23_P15944	8.12964	8.190836	NM_018696	chr18:48513372-48513431	ELAC1
A_33_P3281468	5.105657	5.0288043		chr15:42979340-42979399	STARD9
A_23_P23542	6.547765	6.692226	NM_024813	chr1:92801909-92801968	RPAP2
A_33_P3228931	6.075567	6.164325	NM_001164115	chr20:55025671-55025730	CASS4
A_24_P178654	11.607504	11.662766		chrX:110867823-110867764	
A_33_P3694746	4.2202888	3.435825	AK056786	chr1:43441758-43441817	SLC2A1-AS1
A_23_P353717	5.996834	5.935527	NM_152308	chr16:11445384-11445443	RMI2
A_23_P63026	9.014129	9.272178	NM_006499	chr1:236711313-236711372	LGALS8
A_23_P11032	4.3338885	4.15957	NM_032591	chrX:46466588-46466529	SLC9A7
A_24_P911094	6.1881924	6.4036627	XM_005263579	chr2:114391802-114398981	RABL2A
A_23_P55544	3.4574878	2.3900566	NM_133459	chr18:57102090-57102031	CCBE1
A_33_P3285456	3.4614727	2.3900566	NM_001024679	chr1:152692578-152692637	C1orf68
A_33_P3221788	8.678138	8.562648	NM_020673	chr20:56942283-56942342	RAB22A
A_33_P3362641	9.058923	9.123701	NM_001008388	chr4:103809666-103809725	CISD2
A_24_P23625	7.116943	7.137463	XR_243575	chr17:14252127-14252186	HS3ST3B1
A_23_P153086	10.505745	10.501459	NM_024805	chr18:77806254-77806313	RBFA
A_24_P362904	6.2996783	6.88156	NM_004567	chr3:48555513-48555454	PFKFB4
A_23_P501831	2.6044838	2.9672678	NM_032385	chr5:154198184-154198125	FAXDC2
A_33_P3318746	8.233768	7.873255	NM_001277200	chr1:224567095-224567154	CNIH4
A_33_P3213888	4.395827	4.50456	NM_001162995	chr17:73630124-73630183	SMIM5
A_23_P502832	8.051502	7.8878345	NM_006047	chr20:34237048-34236989	RBM12
A_33_P3302354	5.892473	5.86318	NM_001199281	chr22:24574105-24574164	CABIN1
A_33_P3383094	4.3168373	4.634903		chr7:155188874-155188933	LOC401437
A_32_P38645	3.6254165	3.6924973	NM_182970	chr20:43380733-43380674	RIMS4
A_32_P16489	9.090052	8.983759	NM_145644	chr2:86437658-86437716	MRPL35
A_33_P3225937	5.368075	5.860708	NM_145858	chr21:34994368-34994309	CRYZL1

A_33_P3403082	6.8377895	6.7173347	NR_027686	chr20:62671072-62671131	LINC00176
A_24_P233915	7.237857	7.3817835	NM_001001653	chr1:43852530-43852284	MED8
A_33_P3422429	10.335277	10.4086485	AJ001306	chr1:62580523-62580582	INADL
A_33_P3344861	5.4566154	5.443235	AK124321	chr7:155758948-155759007	LOC389602
A_33_P3411888	4.5635004	3.9310262	XM_005256481	chr17:18607987-18608046	TRIM16L
A_33_P3422897	4.84148	4.707607	NM_005502	chr9:107624071-107624012	ABCA1
A_33_P3409139	4.9261723	4.474537		chr10:114615067-114615126	XLOC_008618
A_33_P3390232	5.9445333	6.0793085	BX648392	chr6:31848313-31848372	
A_24_P722216	9.046381	9.444629	NM_018706	chr10:12165028-12165087	DHTKD1
A_23_P60324	9.892845	9.408501	NM_016172	chr9:138825054-138824995	UBAC1
A_33_P3412055	3.5915792	3.5531652	NM_144690	chr19:56895597-56895538	ZNF582
A_33_P3826455	2.3221061	2.3900566		chr5:12759692-12759751	
A_33_P3245947	6.6125617	6.802895	NM_144999	chr17:79983299-79983358	LRRC45
A_33_P3441021	6.451538	6.1791	NM_001136534	chr12:120078559-120078618	TMEM233
A_24_P408047	7.112405	7.6047416	NM_020904	chr19:49340415-49340356	PLEKHA4
A_33_P3404623	5.186304	4.8271384	NM_017777	chr17:56283137-56283078	MKS1
A_23_P5945	7.186783	7.265576	NM_021931	chr20:37668102-37668161	DHX35
A_23_P210274	9.004435	9.234101	NM_015387	chr2:198415033-198415092	MOB4
A_23_P45726	12.051247	12.100834	NM_005826	chr1:23636602-23636543	HNRNPR
A_23_P134078	8.13145	8.414104	NM_004824	chr6:4954782-4954841	CDYL
A_23_P69293	5.1024127	4.5602593	NM_018397	chr3:53851509-53851450	CHDH
A_33_P3386506	6.506564	6.4581013	NM_001144961	chr6:31515952-31516011	NFKBIL1
A_24_P778836	6.7750454	5.975348	NM_015659	chr16:11928312-11928253	RSL1D1
A_23_P201238	8.536835	8.212493	NM_030918	chr1:151665496-151665939	SNX27
A_33_P3408117	3.8611937	4.195518	NM_001128223	chr3:75786106-75786047	ZNF717
A_23_P22214	4.5251126	4.787123	NM_004157	chr3:48789114-48789055	PRKAR2A
A_24_P933794	8.007996	8.078167	XR_159371	chr22:30831574-30831633	LOC646513
A_23_P157416	6.809285	6.7878575	NM_032164	chr7:99091344-99091285	ZNF394
A_33_P3418448	4.181506	2.3900566		chr22:021713526-021713585	
A_33_P3377760	5.5669584	5.414499	DA946325	chr6:170773783-170773842	
A_23_P39766	8.802953	8.7430935	NM_014905	chr2:191827822-191827881	GLS
A_33_P3325558	9.204849	9.204564	AK022043	chr1:174514552-174514611	RABGAP1L
A_33_P3360773	8.443119	8.534409	NM_005706	chr11:2424872-2424931	TSSC4
A_33_P3285639	4.5151305	5.0217576	NM_025244	chr2:99634745-99634686	TSGA10
A_33_P3291998	6.5061235	6.6324987	AK001106	chr1:178848557-178848616	RALGPS2
A_32_P125549	15.226828	15.208674		chr14:059261717-059261776	
A_24_P29686	7.541861	7.718913	NM_031449	chr7:44809393-44809452	ZMIZ2
A_23_P31602	8.805335	8.65069	NM_003910	chr7:99007777-99008730	BUD31
A_33_P3323999	9.403229	9.407947	NM_002972	chr22:50885295-50885236	SBF1
A_33_P3411392	3.284977	2.3900566		chr20:30075307-30075366	LINC00028
A_24_P18105	10.912276	10.906977	NM_032466	chr8:62537754-62537695	ASPH
A_33_P3408203	8.597183	8.984638	NM_003236	chr2:70683531-70683472	TGFA
A_33_P3231277	9.081562	9.288272	NM_181054	chr14:62211472-62211531	HIF1A
A_23_P97632	11.693324	11.533556	NM_004446	chr1:220142463-220142404	EPRS
A_33_P3246829	4.5857553	4.514499	NM_173843	chr2:113890639-113890698	IL1RN
A_33_P3394809	6.1911983	6.1596065	NM_001282203	chr17:79862351-79862292	PCYT2
A_33_P3337627	9.255523	8.954116	NM_004621	chr11:101323047-101322988	TRPC6
A_33_P3312119	6.740841	5.6883945	NM_001195032	chr6:159331223-159331282	C6orf99
A_23_P43476	6.53278	7.4844866	NM_003383	chr9:2653897-2653956	VLDLR
A_33_P3849600	13.674838	12.926729	M30627	chr11:27911711-27911652	HSP90AA2
A_24_P139191	6.5495	5.728671	NM_031483	chr20:33099135-33099194	ITCH
A_24_P137522	3.646058	3.7772238	NM_019050	chr4:120215256-120215315	USP53
A_33_P3229390	6.1067867	6.3801527		chr15:089061422-089061481	
A_23_P201731	7.7056274	7.563959	NM_004619	chr1:211548188-211548247	TRAF5
A_33_P3375894	6.4079223	6.849818		chr1:209406403-209406462	
A_33_P3313625	4.934392	4.874205		chr13:050194775-050194716	
A_32_P148796	9.262838	9.375848	NM_001077619	chr8:59363782-59363841	UBXN2B
A_24_P84898	6.9887676	7.026047	NM_004111	chr11:61564029-61564087	FEN1
A_23_P255076	6.588115	6.6142893	NM_033411	chr6:83905856-83905915	RWDD2A

A_33_P3267150	4.4858804	4.340288		chr10:003869857-003869798	
A_24_P58529	12.092395	12.349457	NM_032704	chr12:49666285-49666344	TUBA1C
A_24_P562369	5.908371	5.996834			
A_23_P402952	5.2240334	5.3015447	NM_001008747	chr7:143270711-143270770	CTAGE15
A_23_P5611	7.978067	8.042814	NM_018151	chr2:152331786-152331845	RIF1
A_33_P3255046	10.427872	10.504001		chr1:027312321-027312262	
A_33_P3276112	3.714902	3.3833873	XR_246621	chr8:145722802-145722743	
A_33_P3324186	4.7863765	4.769288	NR_046243	chr5:50672961-50672902	LOC642366
A_33_P3247848	6.8036003	6.36254	NM_001144989	chr19:58380814-58380755	ZNF814
A_23_P305092	6.797575	6.551169	NM_019604	chr11:122743164-122743223	CRTAM
A_32_P43465	6.6207337	6.6082516	NM_006962	chrX:47834372-47834313	ZNF182
A_33_P3249524	6.2553296	6.35036	NM_001031618	chr7:102193738-102193797	SPDYE2
A_24_P845072	7.425973	7.69506	NM_144963	chr8:124825812-124825871	FAM91A1
A_33_P3242231	10.208358	10.3673525		chr4:083412578-083412519	
A_23_P41025	11.530851	11.2506695	NM_014366	chr3:52728321-52728380	GNL3
A_33_P3210160	16.23797	16.283295	NM_001195605	chr19:56129784-56129843	ZNF865
A_33_P3284508	6.1717014	6.098605	NM_001174104	chr5:140011377-140011318	CD14
A_23_P401361	4.8697834	4.4932013	NM_020845	chr12:123469034-123468975	PITPNM2
A_23_P14774	3.6614242	3.497074	NM_004390	chr15:79214268-79214209	CTSH
A_23_P99260	11.609968	11.463805	NM_006838	chr12:95908032-95908091	METAP2
A_33_P3313258	6.0153522	5.634031	NM_031921	chr1:1417934-1417993	ATAD3B
A_24_P838947	5.294143	5.192566		chr17:25758247-25758306	TBC1D3P5
A_32_P21384	13.900791	13.77849	NM_000985	chr18:47017232-47017173	RPL17
A_33_P3232937	5.147873	5.446414	NR_040077	chr1:68583320-68583379	GNG12-AS1
A_23_P19663	8.664146	8.819933	NM_001901	chr6:132270189-132270130	CTGF
A_33_P3288359	11.2657795	11.157707	NM_002801	chr16:67968471-67968412	PSMB10
A_33_P3786807	3.1899614	2.3900566	CA436475	chr19:47334133-47334192	SNAR-E
A_33_P3209831	4.4480877	4.8450556	NM_003419	chr19:37370407-37370466	ZNF345
A_23_P370989	12.246999	12.402329	NM_005914	chr8:48888334-48888393	MCM4
A_33_P3608210	6.576109	6.315585	NR_027054	chr9:21454775-21454716	MIR31HG
A_33_P3325843	4.961618	4.7689443	AK097358		FLJ40039
A_23_P69179	12.469933	12.585962	NM_018192	chr3:189674703-189674644	LEPREL1
A_23_P164468	6.9135523	6.923273	NM_014913	chr18:77897118-77897177	ADNP2
A_23_P167828	8.761666	8.562775	NM_016104	chr6:116912107-116914195	RWDD1
A_23_P385322	5.826772	5.4604654	NM_020799	chr10:90676505-90676564	STAMBPL1
A_23_P423695	7.792561	8.187274	NM_006454	chr4:2251339-2251280	MXD4
A_33_P3262452	6.7829804	6.738529	NM_001127649	chr22:18571167-18571226	PEX26
A_32_P14843	9.596351	9.823785	XM_005276535	chr16:74425519-74425578	
A_24_P136182	14.074741	14.156184	NR_026676	chr1:050792068-050792127	RPS2P32
A_23_P416305	6.614682	5.8132763	NM_000990	chr11:8711092-8711151	RPL27A
A_23_P32036	7.7706165	7.85335	NM_017881	chr9:77676379-77676320	NMRK1
A_33_P3333554	3.6000166	3.5980043	BX640643	chr7:149941768-149941709	ACTR3C
A_23_P51918	3.6124718	4.7863765	NM_033127	chr1:177898580-177898521	SEC16B
A_33_P3383084	2.8566086	2.3900566		chr15:079047034-079047093	
A_24_P251599	9.25296	9.074507	NM_001234	chr3:8787416-8787475	CAV3
A_33_P3223980	9.62225	9.975337	NM_001043352	chr1:154129942-154129883	TPM3
A_33_P3357445	6.4837313	6.735289	NM_003211	chr12:104381013-104381072	TDG
A_23_P93302	5.0694027	5.059572	NM_001470	chr6:29570516-29570457	GABBR1
A_33_P3412519	4.964714	4.8154907	NM_001199319	chr22:18566416-18566475	PEX26
A_23_P213441	7.387179	6.5833015	NM_032175	chr5:72877681-72877740	UTP15
A_33_P3357247	7.9469647	7.8939705	NM_025090	chr17:76793024-76792965	USP36
A_32_P83049	9.546795	10.243546	NM_014971	chr2:25381946-25382004	EFR3B
A_24_P30206	10.296388	10.134633	NM_078468	chr10:127522436-127522495	BCCIP
A_33_P3295358	11.331291	11.54629	NM_139314	chr19:8439197-8439256	ANGPTL4
A_33_P3347928	7.961656	7.923118	NM_020307	chr3:156876715-156876656	CCNL1
A_23_P93383	5.4802513	5.893821	NM_004761	chr6:33260865-33260806	RGL2
A_23_P19352	6.211047	5.9663386	NM_006586	chr6:42905830-42905889	CNPY3
A_33_P3345389	8.191202	8.310453	NM_001009984	chr20:3230007-3229948	C20orf194
A_33_P3289296	8.672915	8.929662	NM_183240	chr2:120196037-120196096	TMEM37

A_23_P146325	9.089701	8.77069	NR_002765	chr8:131307736-131307677	ASAP1-IT1
A_23_P131653	7.185267	6.9514723	NM_002643	chr2:46819697-46819638	PIGF
A_33_P3308332	4.4460244	4.995552	NM_021200	chr11:73373788-73373847	PLEKHB1
A_23_P40880	8.045162	8.038364	NM_178868	chr3:32411401-32411460	CMTM8
A_33_P3296871	8.39199	8.302849	NM_006955	chr10:43084664-43084605	ZNF33B
A_23_P361841	4.966156	5.213916	NM_020734	chr12:8929319-8929378	RIMKLB
A_23_P397293	13.998194	13.503417	NM_017527	chr8:143784821-143784880	LY6K
A_24_P192627	5.023452	4.8841643	NM_004529	chr9:20345250-20345191	MLLT3
A_23_P81507	6.2532864	5.8768315	NM_001447	chr5:150884319-150884260	FAT2
A_24_P376556	12.088271	11.902544	NM_018947	chr7:25158296-25158264	CYCS
A_23_P55601	5.838382	5.889778	NM_007345	chr18:74682000-74682059	ZNF236
A_24_P48248	4.453026	4.152668	NM_024032	chr17:42232655-42232714	C17orf53
A_32_P105083	4.9481516	4.4575405		chr2:200710883-200710824	FTCDNL1
A_24_P330263	3.8448188	3.598211	NM_003991	chr13:78470627-78470568	EDNRB
A_23_P69513	3.6561286	3.849791	NM_198229	chr4:3432200-3432259	RGS12
A_32_P528311	7.018832	7.358782	NM_178570	chr11:57235294-57235353	RTN4RL2
A_24_P289139	11.252232	11.017508	NM_001024666	chrX:19610255-19610196	SH3KBP1
A_23_P203391	10.312916	10.213593	NM_001083926	chr11:62159917-62159976	ASRGL1
A_33_P3244274	5.3046308	4.5606623	BC016958		RNF208
A_23_P89073	7.0972757	6.7808757	NM_145911	chr16:71481852-71481793	ZNF23
A_23_P204484	9.129709	8.954513	NM_006861	chr12:120533628-120533569	RAB35
A_23_P103011	7.202219	7.3891296	NM_004914	chr22:23506428-23506487	RAB36
A_33_P3400477	8.574541	8.685737	NM_001282936	chr1:47726185-47726126	STIL
A_23_P203344	8.911225	9.279639	NM_053023	chr11:58387868-58387927	ZFP91
A_23_P40192	9.397516	9.612997	NM_021248	chr20:44802794-44802735	CDH22
A_23_P143016	5.3559065	5.199451	NM_212481	chr2:97218184-97218243	ARID5A
A_23_P314086	11.238366	11.058315	NM_194460	chr19:647645-647586	RNF126
A_23_P127676	10.210121	10.42384	NM_014633	chr11:10800906-10800965	CTR9
A_33_P3372705	7.412342	7.286814	NM_001271870	chr1:143916135-143916194	SRGAP2B
A_24_P131580	7.462261	7.7233543	NM_031313	chr2:233275347-233275406	ALPPL2
A_33_P3335461	5.1814833	5.1686964	AY533203	chr14:106101432-106101492	
A_24_P924591	10.024526	9.414466	NM_007146	chr17:56049012-56048953	VEZF1
A_23_P145424	5.918523	6.0432243	NM_014895	chr6:84834053-84833994	KIAA1009
A_33_P3345314	4.2380733	3.63888	NM_199427	chr20:50713972-50713913	ZFP64
A_33_P3261982	4.889423	4.537688	NM_000947	chr6:57512643-57512702	PRIM2
A_23_P137865	8.797425	8.685622	NM_198883	chr1:155183105-155183164	MTX1
A_23_P320159	5.3205338	5.3520803	XM_005274455	chrX:13682794-13682853	TCEANC
A_33_P3285769	10.833464	10.715368		chr13:019271755-019271814	
A_33_P3240843	5.419146	6.7076406	NM_144649	chr8:133734346-133734287	TMEM71
A_23_P135499	10.14686	9.93774	NM_013943	chr1:25170053-25170112	CLIC4
A_24_P356509	6.0330644	5.4213066	NM_001142571	chr17:33427055-33426996	RAD51D
A_33_P3362088	8.5788	8.984365	NM_001256796	chr12:121671848-121671907	P2RX4
A_24_P3140	8.706441	8.768257	NM_014497	chr2:71654285-71654344	ZNF638
A_24_P161403	12.48328	11.9539795		chr3:166783771-166783830	
A_23_P69020	4.9082055	4.3649826	NM_001105580	chr3:97705765-97705706	GABRR3
A_33_P3376434	6.296438	6.282742	NM_001080517	chr3:9516780-9516839	SETD5
A_23_P76914	8.171041	8.367019	NM_005982	chr14:61112906-61112847	SIX1
A_33_P3220425	2.9422896	2.3900566		chr7:63227776-63227717	XLOC_014512
A_23_P78092	7.680601	7.0073843	NM_001003927	chr17:29645461-29645402	EVI2A
A_23_P4425	10.243546	10.287271	NM_002018	chr17:18148898-18148704	FLII
A_24_P3045	2.998951	2.3900566	NM_032974	chr2:202057711-202057770	CASP10
A_33_P3286334	3.920342	3.739479	NM_018957	chr22:38043288-38043347	SH3BP1
A_33_P3284129	3.7164032	2.3900566	NM_144586	chr2:133402818-133402759	LYPD1
A_23_P129577	3.781042	3.7995932	NM_033208	chr16:3349366-3349307	TIGD7
A_23_P345707	6.8503885	6.890525	NM_152259	chr15:90170742-90170801	TICRR
A_33_P3289207	10.464189	10.833464	BC090950	chr2:69870510-69870451	AAK1
A_33_P3685216	3.8130207	3.9711444	NM_130786	chr19:58858293-58858234	A1BG
A_33_P3226557	3.5872471	3.4150755	NM_001013650	chr3:138738889-138738830	PRR23B
A_33_P3401284	6.35036	6.463605	NR_003051	chr9:35657998-35657939	RMRP

A_33_P3452297	6.300085	6.4882607		chr21:23095718-23095659	LINC00317
A_24_P109351	6.9662576	6.7211423	NM_001009814	chr13:42293806-42293747	VWA8
A_32_P49616	13.319847	13.305304	NM_001959	chr2:207027551-207027610	EEF1B2
A_23_P35256	8.468758	8.375738	NM_006468	chr1:145594972-145594157	POLR3C
A_23_P66117	9.313407	9.544331	NM_032039	chr16:316044-316103	ITFG3
A_23_P30283	7.3670654	7.488234	NM_198507	chr5:99922313-99922372	FAM174A
A_33_P3404189	4.5456724	4.432579	NM_032450	chr8:145303820-145303879	MROH1
A_33_P3354296	10.410517	10.1763115		chrX:18884701-18884760	XLOC_014512
A_33_P3414389	7.058125	7.038493	NM_020979	chr7:101962122-101962178	SH2B2
A_24_P450285	2.5790021	3.0197248	NM_001145018	chr11:119061446-119061130	CCDC153
A_23_P404094	4.543107	4.0891514	NR_027052	chr22:21356852-21356911	THAP7-AS1
A_32_P165116	6.3114586	6.467751	NM_138458	chr2:68364466-68361908	WDR92
A_33_P3409289	5.730037	5.8490157		chr18:053875228-053875169	
A_33_P3272948	5.006938	4.8940053	NM_001145536	chr17:4806148-4806207	C17orf107
A_33_P3421728	6.1900525	5.457038	NM_001346	chr3:185865094-185865035	DGKG
A_23_P217778	8.145767	8.312761	NM_078629	chrX:11793616-11793675	MSL3
A_33_P3262694	11.492129	11.328662		chr16:030831898-030831839	
A_23_P35989	5.9949675	6.059264	NM_139178	chr11:43923132-43923191	ALKBH3
A_23_P154358	4.2379937	4.1741886	NM_144707	chr2:95956518-95956577	PROM2
A_33_P3650353	4.913885	4.610823	AI887274	chr6:133138234-133138175	SNORA33
A_23_P107933	5.1961336	5.853154	NM_016440	chr19:50498484-50498154	VRK3
A_33_P3328921	3.8949456	2.3900566	BX161431	chr14:24391695-24391636	
A_23_P27066	10.291005	10.6323395	NM_003342	chr17:4175034-4174975	UBE2G1
A_33_P3367171	4.9641013	4.781681	AK123243	chr11:62760920-62760861	SLC22A8
A_24_P134653	7.4045157	7.2541447	NM_003611	chrX:13786879-13787218	OFD1
A_33_P3383292	5.270671	4.517907	NR_033940	chr1:99612372-99612431	LOC100129620
A_33_P3284077	4.2818766	4.512703	NM_018230	chr1:229635491-229635432	NUP133
A_23_P23102	6.924873	6.8844514	NM_145238	chr1:33961163-33961222	ZSCAN20
A_33_P3391211	7.601702	7.3047557	NM_001025300	chr18:8639302-8639361	RAB12
A_33_P3339820	14.350332	14.282986	NM_001003680	chr1:66095983-66096042	LEPR
A_23_P135474	10.298828	10.283176	NM_016491	chr1:54683876-54683935	MRPL37
A_23_P206140	2.3221061	2.3900566	NM_018602	chr15:78574350-78574409	DNAJA4
A_24_P381555	5.46975	4.954936	NM_005870	chr13:21722668-21722727	SAP18
A_23_P165879	11.285087	10.421682	NM_016131	chr2:26359998-26360057	RAB10
A_33_P3423121	4.5173216	4.537299	NM_001260492	chr11:110066370-110066311	RDX
A_33_P3301010	3.8936157	3.9258654	NM_001128626	chr18:12453127-12453068	SPIRE1
A_32_P154830	5.8655252	6.165842	NM_014028	chr6:108363318-108363259	OSTM1
A_32_P22257	11.789254	12.039624	NM_004965	chr21:40715046-40714987	HMGN1
A_23_P34396	8.667963	9.0644455	NM_020317	chr1:25571746-25571687	C1orf63
A_32_P214665	4.14197	4.9068217	NM_001102566	chr1:161255021-161255080	PCP4L1
A_23_P204782	5.3763742	5.2280054	NM_020128	chr12:68718983-68718924	MDM1
A_33_P3677020	5.0322733	4.8933277	NR_037158	chr16:27459052-27458993	IL21R-AS1
A_23_P169050	11.593349	11.445275	NM_014018	chr8:80831315-80831256	MRPS28
A_33_P3276585	4.4374905	3.933748	NM_001099224	chr9:27036732-27036791	IFT74
A_33_P3362812	12.198631	12.272379		chr2:176049213-176049272	
A_24_P388528	5.26474	4.96278	NM_173216	chr3:186796052-186796111	ST6GAL1
A_33_P3398917	6.792082	6.7580476	NM_001039614	chr15:74032245-74032186	C15orf59
A_24_P291598	9.643463	9.211948	NM_003363	chr3:49315651-49315592	USP4
A_33_P3867534	7.272501	6.9986625	NM_182751	chr10:13251992-13252051	MCM10
A_23_P383435	10.240225	10.180435	NM_004773	chr17:34851427-34851486	ZNHIT3
A_23_P143987	6.977076	7.1926804	NM_006395	chr3:11468321-11468380	ATG7
A_32_P129894	10.123006	10.95436	NM_001080497	chr9:123363867-123363808	MEGF9
A_33_P3339987	3.7388065	4.0015106		chr6:039007503-039007444	
A_23_P42144	6.2166853	6.0853796	NM_000287	chr6:42931953-42931894	PEX6
A_32_P25437	7.74832	7.36116	NM_001046	chr5:127524728-127524787	SLC12A2
A_23_P29985	7.865557	7.926305	AK022953	chr4:8482974-8483033	TRMT44
A_24_P148796	6.387857	6.6697254	NM_020998	chr3:49721610-49721551	MST1
A_23_P51699	6.5220695	6.120763	NM_004723	chr1:155917401-155917342	ARHGEF2
A_23_P66050	3.8763835	4.1007376	NM_033309	chr16:67182872-67182813	B3GNT9

A_33_P3402035	7.198926	6.862447	NM_004295	chr17:27077917-27077976	TRAF4
A_23_P157865	7.4897265	7.5423965	NM_002160	chr9:117783369-117783310	TNC
A_33_P3395876	5.3628435	5.411585	NM_001111307	chr19:10572615-10572674	PDE4A
A_23_P150350	7.637421	7.631954	NM_022761	chr11:111753899-111754526	C11orf1
A_32_P14457	2.3221061	2.3900566	XR_245759	chr14:45368248-45368189	
A_23_P411188	4.2760415	4.1385226	NM_020402	chr11:3687068-3687009	CHRNA10
A_23_P200222	9.416584	8.730341	NM_033300	chr1:53711278-53711219	LRP8
A_23_P132675	10.898224	10.8497	NM_003365	chr3:48637517-48637106	UQCRC1
A_23_P131899	3.2633066	2.3900566	NM_080489	chr20:1293097-1293038	SDCBP2
A_33_P3367112	5.0680356	4.7911797	BG758909	chr12:120650844-120650903	PXN-AS1
A_33_P3303066	4.7184463	4.3634973	NM_017797	chr19:1986041-1985982	BTBD2
A_33_P3391175	5.4943213	6.052957	AK126356	chr22:49947759-49947818	
A_33_P3409974	3.9893937	4.3171067	NM_001252634	chr3:24158704-24158645	THRB
A_33_P3310189	4.243471	4.1593895	NM_000684	chr10:115806608-115806667	ADRB1
A_23_P210747	9.351271	9.011038	NM_015939	chr20:5918821-5918762	TRMT6
A_33_P3244283	7.478149	7.5247245	NM_033259	chr3:183977062-183977003	CAMK2N2
A_33_P3314341	4.2533145	4.1805053	BC047922	chr9:115380631-115380690	KIAA1958
A_32_P148122	6.245355	6.133232	BC095489	chr2:89953072-89953131	
A_24_P390060	4.8068776	5.731712	NM_138451	chr12:113633351-113633292	IQCD
A_23_P142096	3.5070224	4.1568174	NM_001506	chr19:51274664-51274723	GPR32
A_33_P3382162	8.648005	8.705313	NM_000449	chr1:151314496-151314437	RFX5
A_23_P14340	8.073656	8.44478	NM_014672	chr14:35742683-35742742	KIAA0391
A_23_P386888	3.8885841	3.0531278	NM_005577	chr6:160952549-160952515	LPA
A_23_P154585	8.748003	8.50061	NM_001042633	chr20:44470290-44470349	SNX21
A_33_P3335725	6.1289077	6.339784	NM_000208	chr19:7112410-7112351	INSR
A_33_P3226675	3.8729494	3.3231413		chr17:020462648-020462589	
A_33_P3365963	7.786662	8.06004		chr4:041363168-041363109	
A_33_P3337450	4.641303	5.2155166	XR_248253	chr16:89334513-89334572	
A_33_P3415097	6.715912	6.4750795	NM_001830	chrX:10205535-10205594	CLCN4
A_24_P291826	6.683916	5.918523	NM_001009991	chr6:159181680-159181739	SYTL3
A_33_P3233565	7.995145	8.230689	NM_000375	chr10:127496042-127495983	UROS
A_24_P410797	6.4790163	6.502869	AK125979	chr3:124394079-124394138	KALRN
A_32_P84605	14.982724	14.921533	NM_001025	chr5:81573551-81572318	RPS23
A_33_P3296198	16.435053	16.435053	NM_001164479	chr5:126380533-126380474	C5orf63
A_23_P143885	6.0796795	6.6779284	NM_019555	chr3:56762170-56762111	ARHGEF3
A_33_P3274319	6.6456413	6.5275083	NM_178545	chr1:1849478-1849419	TMEM52
A_23_P54728	8.221435	8.189287	NM_023933	chr16:772388-772447	FAM173A
A_23_P413760	3.800786	3.5231926	NM_175080	chr17:3599255-3599196	P2RX5
A_23_P68970	9.192982	9.051036	NM_014570	chr22:43192987-43192928	ARFGAP3
A_33_P3316903	4.931839	4.9686093		chr8:30240735-30240676	RBPMS-AS1
A_23_P54144	4.92521	4.9743853	NM_001202	chr14:54417377-54417318	BMP4
A_23_P425880	7.8903666	8.0097275	NM_007118	chr5:14508436-14508495	TRIO
A_24_P75680	3.5088837	4.4477634	NM_001258402	chr12:51865202-51865261	SLC4A8
A_23_P115375	10.373048	10.173088	NM_001123375	chr1:120904981-120905040	HIST2H3D
A_24_P807031	4.838113	4.7165117	NM_001017971	chr5:81614067-81614126	ATP6AP1L
A_33_P3360341	2.4601126	2.3900566	NM_001002295	chr10:8117072-8117131	GATA3
A_24_P412976	8.789841	8.668467	NM_018273	chr19:48835676-48835617	TMEM143
A_23_P39550	6.3638177	6.5962133	NM_030923	chr2:135214230-135214171	TMEM163
A_23_P107735	12.060354	12.509143	NM_001783	chr19:42385356-42385415	CD79A
A_23_P32021	7.2383895	7.175806	NM_000136	chr9:97861581-97861522	FANCC
A_33_P3258265	3.6631527	3.7557251	NM_030913	chr1:151108994-151108935	SEMA6C
A_32_P32413	5.364708	5.2661715	NM_015559	chr18:42648247-42648306	SETBP1
A_23_P44849	4.2346096	3.6863508	NM_178863	chr16:29923171-29922471	KCTD13
A_23_P140738	7.8147993	7.8170137	NM_001142864	chr16:88782231-88782172	PIEZO1
A_23_P51317	7.469327	7.1969395	NM_019083	chr1:100615850-100615909	TRMT13
A_33_P3888629	8.157254	7.9065614	NM_001164000	chr3:168801415-168801356	MECOM
A_23_P202117	6.175391	6.417738	NR_046310	chr10:92987458-92987517	PCGF5
A_23_P75630	4.228568	3.9069467	NM_052968	chr11:116660265-116660206	APOA5
A_23_P93973	4.605746	4.8720303	NM_019841	chr7:142609789-142609730	TRPV5

A_23_P99747	4.7038026	5.202057	NM_004196	chr14:50805745-50805686	CDKL1
A_23_P348911	5.094888	4.6520095	NR_027251	chr2:43455024-43455083	LINC01126
A_33_P3354076	3.280357	2.9607322	NR_036433	chr3:101241755-101241814	FAM172BP
A_23_P433369	7.155204	7.0288687	NM_015473	chr14:31761197-31761138	HEATR5A
A_33_P3381064	4.239183	4.3856688	NM_001282301	chr16:60392682-60392623	LOC729159
A_33_P3338491	4.655197	4.774296	NR_003275	chr8:7200733-7200792	LOC392196
A_24_P403459	6.1134305	6.01974	NM_021068	chr9:21187070-21187011	IFNA4
A_23_P67618	4.88261	4.8976374	NM_175872	chr19:35447841-35447782	ZNF792
A_33_P3273854	4.54224	4.672311	NM_207015	chr3:175523323-175523382	NAALADL2
A_33_P3246804	8.369637	8.581188	NM_001165937	chr17:37819508-37819567	STARD3
A_23_P368896	5.1146674	5.0275593	NM_013346	chrX:70280941-70280882	SNX12
A_33_P3227079	12.311659	12.016678	XM_005272231	chr9:134166773-134166832	PPAPDC3
A_33_P3216442	4.639407	4.818762	NM_080680	chr6:33130736-33130677	COL11A2
A_24_P147540	6.8640523	7.198572	NM_001190467	chr19:7934686-7934627	FLJ22184
A_23_P45786	5.7332015	5.4246173	NM_001852	chr1:40766439-40766380	COL9A2
A_24_P354715	8.93161	9.419148	NM_002526	chr6:86204891-86204950	NT5E
A_23_P250629	7.287397	7.3056374	NM_004159	chr6:32810490-32810023	PSMB8
A_33_P3381851	8.02259	8.440025	NM_181688	chr21:46058134-46058193	KRTAP10-10
A_33_P3224819	6.53941	6.7615633	NM_001286646	chr8:142220970-142220911	SLC45A4
A_23_P2143	11.271833	11.454461	NM_014752	chr11:74688489-74688548	SPCS2
A_33_P3239122	3.787242	4.150781	AK310052	chr1:43124921-43124980	PPIH
A_23_P154806	10.1958885	10.2312975	NM_012156	chr20:34818126-34818185	EPB41L1
A_24_P25346	9.348744	9.248102	NM_032830	chr16:69201013-69201072	CIRH1A
A_33_P3350726	6.7748475	7.043859	NM_138711	chr3:12434128-12434187	PPARG
A_33_P3273409	9.006567	9.161217	NM_024881	chr19:16661738-16661679	SLC35E1
A_32_P128209	5.4088244	5.5556493	NR_026936	chr5:95195702-95195761	C5orf27
A_23_P15493	11.201412	11.065624	NM_016077	chr17:57774800-57774741	PTRH2
A_23_P2582	7.6047416	7.538491	NM_015401	chr12:48176815-48176756	HDAC7
A_23_P49459	5.185406	5.159744	NM_030941	chr16:20856018-20856077	LOC81691
A_23_P5089	9.34314	9.351271	NM_001001975	chr19:1242585-1244131	ATP5D
A_23_P335495	5.9138775	5.6677804	NM_001001891	chr2:242164368-242164427	ANO7
A_24_P305933	6.7778907	7.067271	NM_020698	chr12:94962733-94962674	TMCC3
A_23_P139965	2.876757	3.1352508	NM_001010897	chr13:44971760-44971820	SERP2
A_33_P3261408	4.0356164	4.1291876	NM_147196	chr3:46752346-46752405	TMIE
A_24_P173754	7.2966976	7.0172224	NM_030806	chr1:184567549-184588664	C1orf21
A_23_P28263	11.834877	12.107283	NM_021198	chr2:219270601-219270660	CTDSP1
A_33_P3351536	7.5894156	7.914177	NM_173174	chr8:27316844-27316903	PTK2B
A_24_P75230	13.291063	13.315342	NM_033546	chr18:3277808-3277867	MYL12B
A_23_P95130	9.710463	10.052795	NM_207113	chr7:140033747-140033688	SLC37A3
A_23_P25935	6.164159	6.512328	NM_007176	chr14:76117903-76117844	C14orf1
A_23_P218111	10.220846	10.316018	NM_001002236	chr14:94844850-94844791	SERPINA1
A_33_P3313695	4.2794447	4.006128			
A_23_P348323	5.5646954	5.4685087	NM_007144	chr17:36891049-36890990	PCGF2
A_33_P3416448	4.28365	4.4412904	NM_001002860	chr14:93709105-93709046	BTBD7
A_23_P208373	4.1008997	4.7387996	NM_000767	chr19:41523525-41523584	CYP2B6
A_23_P152919	9.305747	9.283024	NM_002532	chr17:5290336-5290120	NUP88
A_23_P99063	5.3011765	6.02203	NM_002345	chr12:91497705-91497646	LUM
A_33_P3609033	2.3221061	2.3900566	NR_033979	chr2:177038003-177037944	HOXD-AS1
A_33_P3230723	5.42905	5.1140823	NM_183416	chr1:10364547-10364606	KIF1B
A_24_P184445	5.4275928	5.292966	NM_002429	chr12:56229481-56229422	MMP19
A_23_P88234	7.7583933	7.8086257	NM_016049	chr14:24608579-24608357	EMC9
A_33_P3329078	5.6177726	5.958472	NM_000559	chr11:5269613-5269554	HBG1
A_33_P3257460	11.16176	11.383992	NM_018973	chr1:155112428-155112369	DPM3
A_23_P414978	3.775355	3.4796302	NM_177533	chr14:105644053-105643330	NUDT14
A_23_P157580	11.4088955	11.94223	NM_005625	chr8:59494885-59494944	SDCBP
A_24_P141332	11.157981	10.94918	NM_172171	chr10:75572426-75572367	CAMK2G
A_23_P82588	9.805977	9.70311	NM_197964	chr7:139030466-139030525	C7orf55
A_33_P3280320	4.9822083	5.0301604	NM_001195127	chr7:330137-330196	LOC100288524
A_33_P3219398	3.7760732	2.9707646	NM_015461	chr18:22804452-22804393	ZNF521

A_33_P3368675	7.5604753	7.6608143	NM_001029863	chr6:170106141-170106200	C6orf120
A_23_P64799	6.809534	6.494957	NM_015665	chr12:53701678-53701488	AAAS
A_33_P3882659	11.788418	11.683837	AY956766	chr3:74152480-74152539	HSP90AB5P
A_23_P214658	4.265482	4.628119	NM_002586	chr6:32152880-32152821	PBX2
A_23_P4007	3.8339677	4.196048	NM_004860	chr17:7496046-7495893	FXR2
A_33_P3278211	4.246003	4.6497374	AK128041	chr10:88728956-88728897	ADIRF-AS1
A_33_P3394727	5.319129	4.8119335	NM_006558	chr8:136569736-136569795	KHDRBS3
A_24_P941922	5.403378	5.603715	BC035590	chr7:135071445-135071386	CNOT4
A_33_P3357979	4.506446	4.0251155	NM_001012614	chr4:1206087-1206028	CTBP1
A_33_P3380682	7.561698	7.8793054	NM_001253676	chr6:134303728-134303787	TBPL1
A_33_P3242369	4.0395274	3.863964	AF370399	chr1:243788824-243788765	
A_33_P3231352	6.2803955	6.1350837	AK022088	chr4:140335114-140335055	
A_33_P3415191	10.057416	10.174321	NM_005603	chr18:55313842-55313783	ATP8B1
A_24_P364296	8.3874035	8.434397	NM_001980	chr12:131285707-131283131	STX2
A_32_P53524	9.017676	8.873094	NM_004822	chr17:9147220-9147279	NTN1
A_23_P358944	11.234691	11.083038	NM_033244	chr15:74328675-74328734	PML
A_33_P3313245	6.854637	7.8255835	NM_001167595	chr5:33998831-33998772	AMACR
A_33_P3321946	8.474438	8.4710455	NM_023948	chr7:100212928-100212987	MOSPD3
A_33_P3230219	11.501098	11.679579	NM_033504	chr1:33360256-33360197	TMEM54
A_33_P3254801	7.446048	7.2407427	NM_007346	chr20:61444942-61445001	OGFR
A_23_P215461	8.22765	7.6664624	NM_002314	chr7:73536663-73536722	LIMK1
A_23_P99942	6.0089116	5.6585054	NM_024652	chr15:101609711-101609770	LRRK1
A_32_P116271	5.8372765	6.1413603	NR_015359	chr4:85890940-85890999	WDFY3-AS2
A_33_P3287862	5.6645303	5.6462955	XM_005276148	chr1:148902576-148902635	LOC101929815
A_24_P246107	4.662624	4.8941097	NM_181600	chr21:31802946-31803005	KRTAP13-4
A_23_P251937	6.3009615	5.084532	NM_030627	chr5:173386991-173387050	CPEB4
A_23_P82286	5.3956013	5.3278275	NM_173059	chr7:100386962-100388615	ZAN
A_23_P206228	8.925426	8.872561	NM_020821	chr15:62144891-62144832	VPS13C
A_33_P3251685	7.122594	7.1862273	NM_020786	chr16:66921452-66921511	PDP2
A_33_P3362587	3.8772392	3.9090233	BC060774	chr10:72532032-72531973	TBATA
A_23_P52031	10.11624	10.626605	NM_002633	chr1:64125582-64125641	PGM1
A_33_P3293529	8.511153	8.174004		chr8:073342072-073342131	
A_32_P38467	11.747846	11.601384	NR_003584	chr4:119200825-119200884	SNHG8
A_23_P35131	4.203796	4.104257	NM_005850	chr1:149898568-149898509	SF3B4
A_33_P3231187	4.9546466	5.235872	NM_001167	chrX:123041089-123041148	XIAP
A_23_P78563	11.681082	11.682007	NM_024292	chr19:9939311-9939531	UBL5
A_33_P3340224	5.1317472	5.5925655	NR_034097	chr7:28280271-28280330	JAZF1-AS1
A_24_P307014	11.590036	11.058243	NM_005104	chr6:32949018-32949077	BRD2
A_23_P114839	4.643293	4.8485985	NM_004468	chr1:38463456-38463397	FHL3
A_33_P3366175	4.575894	4.305149		chr8:61878759-61878700	LOC100130298
A_23_P128956	6.700929	7.136251	NM_021260	chr14:73436448-73436389	ZFYVE1
A_23_P111041	10.479117	10.465707	NM_003525	chr6:26273375-26273434	HIST1H2BI
A_33_P3344308	8.679947	8.594679	NM_001195249	chr9:32973438-32973379	APTX
A_23_P351215	7.1502852	7.19516	NM_005414	chr3:170110094-170110153	SKIL
A_33_P3239989	4.736662	4.282597		chr17:038099752-038099811	
A_23_P53476	14.693792	14.767965	NM_002300	chr12:21788508-21788449	LDHB
A_33_P3372501	6.0629816	6.590539	NM_015316	chr14:104206483-104206424	PPP1R13B
A_23_P429560	5.899844	6.2501163	NM_018984	chr12:109181316-109181257	SSH1
A_23_P92281	9.501095	9.208762	NM_014170	chr3:112718398-112719742	GTPBP8
A_33_P3349259	3.7911944	4.225145	NM_014719	chr7:143557466-143557407	FAM115A
A_33_P3230166	2.9947722	2.3900566	NM_052867	chr13:101706223-101706164	NALCN
A_32_P199252	12.54159	11.882737	NM_001017963	chr14:102550222-102550163	HSP90AA1
A_33_P3281765	8.804487	8.809992		chrY:028137472-028137411	
A_24_P710730	8.274496	7.995375	NR_024054	chr5:69438247-69438188	SMA4
A_23_P160503	10.459755	10.0711355	NM_016066	chr1:193066821-193066762	GLRX2
A_24_P37887	7.4803042	7.4449215	NM_199243	chr5:94957202-94957261	GPR150
A_33_P3261433	7.8610687	8.175956		chr2:074212866-074212807	
A_33_P3335800	4.554063	4.80793	AK095925	chr18:7943646-7943705	LOC100128219
A_23_P390744	10.703346	10.508797	NM_144600	chr16:15959688-15959629	FOPNL

A_23_P150609	5.901946	6.3340693	NM_000612	chr11:2150453-2150394	IGF2
A_24_P590560	5.18885	5.036498	NM_018427	chr16:15165017-15164092	RRN3
A_24_P176714	7.6312532	7.6154704	NM_015681	chr17:19246728-19246669	B9D1
A_23_P259451	10.756537	10.713688	NM_007080	chr4:147110971-147111030	LSM6
A_33_P3313401	11.655544	11.3815155	NM_018947	chr7:25163189-25163135	CYCS
A_32_P207180	9.550207	9.674395	NM_001040025	chr17:79648669-79648610	ARL16
A_33_P3319791	5.3580627	4.417255	NM_002135	chr12:52453226-52453285	NR4A1
A_23_P121250	11.961438	11.978752	NM_001967	chr3:186507026-186507085	EIF4A2
A_33_P3310533	10.444683	9.730458	NM_001012339	chr5:34937517-34937576	DNAJC21
A_33_P3231363	5.726994	5.824589	AK075229	chr8:99735101-99735042	STK3
A_23_P48307	14.656517	14.790661	NM_030979	chr13:25672234-25672293	PABPC3
A_33_P3296862	4.9743853	4.980319	NM_152459	chr16:5094253-5094194	C16orf89
A_33_P3323760	6.500387	6.6568	NR_015447	chr5:43045012-43045071	LOC153684
A_24_P603890	9.940416	10.185372		chr6:121975307-121975366	
A_33_P3393708	3.5517535	3.0038576		chr10:045736561-045736502	
A_24_P256415	3.7918997	2.3900566	NR_024447	chr17:8262709-8262650	LOC100128288
A_33_P3212027	6.4500837	6.6517606	NM_201999	chr4:139978941-139978882	ELF2
A_33_P3333050	8.3064575	8.467376	NM_001011552	chr2:103150238-103150297	SLC9A4
A_23_P317105	5.995077	6.378858	NM_007202	chr17:19809041-19808982	AKAP10
A_24_P17302	4.195213	3.7164032	NM_194458	chr1:1190050-1189991	UBE2J2
A_23_P361405	4.7809567	4.946322	NM_003549	chr3:50330448-50330389	HYAL3
A_33_P3299487	5.717223	6.166326	NM_001039775	chr1:26671913-26671854	AIM1L
A_23_P103942	8.620146	8.535705	NM_018198	chr1:6694234-6694205	DNAJC11
A_23_P3562	7.9706473	7.7285795	NM_032178	chr16:68334647-68334588	SLC7A6OS
A_24_P76546	3.4835072	3.2419398	NM_006990	chr1:27732987-27732928	WASF2
A_33_P3295650	4.795478	4.740899	NM_001163	chr9:72045529-72045470	APBA1
A_33_P3383431	7.207965	7.2428074	NM_001282989	chr14:24429138-24429197	DHRS4
A_24_P453544	4.670289	4.378585	AY239294	chr9:136217923-136217864	
A_23_P67980	3.6956859	4.428004	NM_003709	chr2:207945627-207945568	KLF7
A_33_P3265185	11.939888	11.71756		chr22:38689315-38689256	CSNK1E
A_33_P3214027	4.738653	4.701279	NM_001254732	chr22:24730402-24730461	SPECC1L
A_33_P3223093	7.444013	7.330289	M27336	chr7:38398233-38398174	
A_23_P162142	7.197618	6.928552	NM_015516	chr11:76508863-76508922	TSKU
A_24_P53215	4.3176613	4.156062	NM_199242	chr17:73823642-73823583	UNC13D
A_23_P68866	12.862791	12.958728	NM_001003684	chr22:30165827-30165886	UQCR10
A_33_P3256585	3.916767	3.9720635	XM_005275730	chr12:133049393-133049334	
A_23_P45011	2.3221061	2.3900566	NM_030949	chr6:150570937-150570996	PPP1R14C
A_33_P3292896	5.98567	6.196144	NM_144579	chr2:73215461-73215402	SFXN5
A_24_P81965	7.0667562	7.001111	NM_021033	chr13:98119444-98119503	RAP2A
A_23_P132358	6.856228	6.7160845	NM_173467	chr22:43529193-43529134	MCAT
A_33_P3254306	5.531018	5.419146	AK126951	chr17:17128198-17128139	FLCN
A_23_P151267	8.471797	8.10305	NM_016357	chr12:50569850-50569791	LIMA1
A_23_P127721	4.930881	5.0115423	NM_002559	chr11:57116158-57117269	P2RX3
A_23_P49254	5.907382	6.0828657	NM_005331	chr16:230574-230717	HBQ1
A_33_P3318841	2.3221061	2.3900566	NM_001164479	chr5:126383328-126383269	C5orf63
A_33_P3343545	3.9528687	3.2269053	NM_002503	chr19:39397888-39397947	NFKBIB
A_23_P141484	8.886627	9.469839	NM_018182	chr17:27083841-27083782	FAM222B
A_23_P65217	7.919842	7.9835176	NM_014305	chr13:95226685-95226626	TGDS
A_23_P53018	6.626496	6.76178	NM_054108	chr11:63230920-63230861	HRASLS5
A_24_P135753	9.391273	9.418226	AL832532	chr3:123210797-123210738	PTPLB
A_32_P178800	5.71668	5.1594377	NM_002203	chr5:52389637-52389696	ITGA2
A_23_P40039	8.218655	7.979669	NM_031445	chr2:128619399-128619340	AMMECR1L
A_32_P103837	8.157678	7.3988705	NM_021238	chr12:31433650-31433591	FAM60A
A_23_P47709	5.443235	4.8503513	NM_000803	chr11:71932884-71932943	FOLR2
A_33_P3279501	6.9901743	7.120455	XM_001130287	chr3:39256044-39256103	DSTNP4
A_23_P74668	6.9165077	6.987171	NM_152290	chr1:12821032-12821091	C1orf158
A_32_P117464	6.220217	6.3389993	NM_178496	chr3:192515209-192515150	MB21D2
A_23_P259049	3.5166879	3.5728898	NM_016180	chr5:33964061-33964002	SLC45A2
A_23_P63816	7.0991707	7.244908	NM_030759	chr10:64914040-64914099	NRBF2

A_24_P108779	4.7870264	4.851305	NM_024681	chr22:37458605-37458664	KCTD17
A_23_P155666	5.8442316	5.4361887	NM_001042402	chr4:76842261-76842202	NAAA
A_33_P3318796	6.584361	6.0434184	NM_005860	chr19:683145-683204	FSTL3
A_33_P3385148	9.285682	9.358353		chr1:206288643-206288584	C1orf186
A_24_P193582	9.115744	9.202512	NM_017702	chr16:90025433-90025492	DEF8
A_32_P930685	3.4541726	4.44626		chr4:248597-248656	ZNF876P
A_33_P3386532	4.0655866	4.488246	AK024058	chr17:73900384-73900325	MRPL38
A_23_P342067	8.134951	7.75659	NM_175866	chr1:162493055-162493114	UHMK1
A_33_P3277679	4.2116613	4.3578286		chr15:091576154-091576095	
A_33_P3305438	5.652219	5.7198677		chr7:155684885-155684944	
A_23_P46309	7.7233543	7.397156	NM_001048199	chr1:28865048-28865107	RCC1
A_24_P107897	8.485205	8.378291	NM_015914	chr16:11773285-11773226	TXNDC11
A_32_P98683	6.429637	6.4231787	NM_005937	chr17:36885675-36885734	MLLT6
A_23_P137434	11.458455	11.667804	NM_014372	chr1:51738593-51738652	RNF11
A_23_P50039	4.704943	4.6458216	NM_005913	chr18:13826278-13826337	MC5R
A_33_P3330473	5.208172	4.9045753	XM_005262162	chrX:106356687-106356628	RBM41
A_33_P3263696	3.7194831	3.6506417	NM_001205119	chr11:46690061-46690120	ATG13
A_24_P162293	7.488033	7.509563	NM_178040	chr12:1602323-1602382	ERC1
A_33_P3304312	9.216414	9.196384	NM_001172684	chr15:72577468-72577409	CELF6
A_33_P3242909	3.7135909	3.7235942	NM_145276	chr19:12428539-12428480	ZNF563
A_23_P54900	7.3966427	7.586919	NM_016936	chr16:4932018-4932077	UBN1
A_24_P2948	6.357845	6.5471992	NM_014328	chr1:155296915-155297991	RUSC1
A_33_P3223713	9.114307	9.466237	NM_006357	chr2:181846903-181846962	UBE2E3
A_33_P3289441	4.122872	3.9462295	AK125976	chr2:130561141-130561082	LOC100131048
A_33_P3234015	9.43269	9.151805	NM_014240	chr3:45722696-45722755	LIMD1
A_23_P63847	9.415003	9.344394	NM_003171	chr10:70968688-70968747	SUPV3L1
A_33_P3326672	5.444701	5.299641	NM_001001662	chr9:99579332-99579273	ZNF782
A_33_P3229678	3.7122426	3.4441504		chr1:143668718-143668659	
A_33_P3223607	9.926301	9.473712	NM_001144026	chr9:140113756-140113813	NDOR1
A_23_P40347	9.048733	9.163912	NM_178580	chr20:30156928-30156987	HM13
A_24_P123119	4.7777634	5.6459565	NM_001966	chr3:184909483-184909424	EHHADH
A_33_P3353425	5.129622	5.12279	NM_020935	chr2:219394741-219394682	USP37
A_33_P3349927	3.1256413	2.9729104	CR627036	chr7:158695283-158695342	WDR60
A_23_P371011	6.0201945	6.049929	NM_182490	chr19:44740960-44741019	ZNF227
A_32_P137939	16.106268	16.151003	NM_001101	chr7:5566846-5566787	ACTB
A_32_P213637	7.499661	7.651049	NR_037596	chr4:120326829-120326770	LINC01061
A_23_P156824	6.3260336	6.2271786	NM_000863	chr6:78172972-78172913	HTR1B
A_32_P222383	13.657412	13.859872	NM_005517	chr1:26802192-26802251	HMGN2
A_23_P168576	5.7022214	5.6765804	NM_152992	chr7:76239323-76239291	POMZP3
A_23_P53646	11.590412	10.67273	NM_139207	chr12:76440082-76440023	NAP1L1
A_23_P379945	7.2420197	7.3704433	NM_001080414	chr14:91737797-91737738	CCDC88C
A_23_P158662	8.027285	7.836722	NM_212492	chr17:80014732-80014791	GPS1
A_33_P3281695	5.6423264	5.193734	NM_001243133	chr1:247611747-247611806	NLRP3
A_24_P269432	6.9545684	6.9966755	NM_005868	chr7:93628513-93625606	BET1
A_33_P3411122	4.8625727	4.8329706		chrX:012828415-012828474	
A_33_P3350207	12.261019	12.506763	NM_017885	chr16:3072685-3072626	HCFC1R1
A_33_P3378644	5.322537	5.4397163	NM_004426	chr12:9091964-9092023	PHC1
A_23_P322395	8.75259	8.5856495	NM_182572	chr19:58565691-58565750	ZSCAN1
A_33_P3248265	4.4643526	4.154126	NM_002341	chr6:31548394-31548335	LTB
A_23_P79231	9.195328	8.898661	NM_134442	chr2:208462917-208462976	CREB1
A_24_P865	6.70068	6.679094	NM_206921	chr6:118887116-118887057	CEP85L
A_24_P281975	9.122808	9.11074	NM_024312	chr12:102139417-102139358	GNPTAB
A_24_P157370	5.042971	4.9933352	NM_018725	chr3:53899592-53899651	IL17RB
A_33_P3724155	7.724441	7.7203217	NM_198440	chr22:24176774-24176715	DERL3
A_33_P3383205	4.121409	3.5434268	AB064667	chr13:30167007-30166948	OK/SW-CL.58
A_33_P3244843	9.473838	9.746892	NM_017431	chr2:219688436-219688377	PRKAG3
A_32_P126609	5.8727236	5.861489	NM_015110	chr9:72968897-72968956	SMC5
A_24_P225970	7.168345	7.071212	NM_001012409	chr3:20212674-20212615	SGOL1
A_33_P3352098	6.4294653	6.470692	NM_021201	chr11:60163279-60163338	MS4A7

A_33_P3312975	6.0200443	5.959612	NM_015282	chr2:122098278-122098219	CLASP1
A_23_P86216	11.208988	11.169504	NM_002790	chr1:109954711-109954511	PSMA5
A_23_P258964	13.418844	13.351572	NM_013417	chr9:94972930-94972871	IARS
A_24_P825874	2.8483858	3.6687973	NM_001277406	chr2:131232021-131231962	POTEI
A_24_P377489	9.6982	9.550207	NM_015622	chr7:5963498-5963557	CCZ1
A_33_P3809606	11.570307	11.272586		chr14:51423054-51422995	
A_23_P99996	4.1856174	3.4913092	NM_019066	chr15:23889081-23889022	MAGEL2
A_33_P3759611	5.7341924	5.6851397	NM_000923	chr19:18321843-18321784	PDE4C
A_33_P3264419	10.302844	10.348887	NM_001277813	chr8:64125285-64125344	YTHDF3
A_33_P3216267	5.4170313	5.3844595		chr20:062922409-062922350	
A_33_P3294790	5.0310154	4.771539	AX721280	chr7:33075943-33075884	
A_33_P3379001	4.365801	4.1314936	NM_133369	chr5:176307504-176307563	UNC5A
A_24_P81900	2.5003085	3.1280673	NM_006931	chr12:8072983-8072924	SLC2A3
A_23_P210445	4.7457805	5.2166824	NM_032107	chr20:42170109-42170168	L3MBTL1
A_33_P3745146	2.3221061	2.3900566	NM_001098517	chr11:115102198-115102139	CADM1
A_24_P63563	8.299764	8.346319	NM_014773	chr5:141319526-141319585	KIAA0141
A_33_P3261167	11.4189825	11.556739	NM_002076	chr12:65107283-65107224	GNS
A_24_P381224	9.2263975	9.185095	NM_172230	chr11:64895058-64894999	SYVN1
A_33_P3341329	7.5445395	7.424062		chr12:42706894-42706836	ZCRB1
A_23_P81650	9.743931	8.253811	NM_020199	chr5:133291476-133291417	C5orf15
A_33_P3271634	4.003939	3.980443		chr6:33048209-33048268	HLA-DPB1
A_23_P66608	9.962095	9.633379	NM_021078	chr17:40265294-40265235	KAT2A
A_24_P125561	10.680315	11.204557	NM_014441	chr19:51630519-51631230	SIGLEC9
A_23_P380326	6.2910852	6.07217	NM_018375	chr14:69926101-69926160	SLC39A9
A_23_P165130	8.85888	8.800849	NM_032683	chr19:18307324-18307383	MPV17L2
A_23_P156497	9.32348	9.526957	NM_021814	chr6:53132945-53132886	ELOVL5
A_33_P3365611	4.8154907	4.574846	NM_152265	chr1:52556216-52556275	BTF3L4
A_33_P3359856	5.834904	5.6955867		chr2:132227819-132227760	
A_23_P252764	9.747673	9.60269	NM_139045	chr9:2161718-2161777	SMARCA2
A_33_P3370076	8.718428	9.001504	NM_006521	chrX:48887788-48887729	TFE3
A_33_P3243500	3.8574724	4.0498366	NR_047662	chr8:12517326-12517267	LOC729732
A_33_P3419691	7.052526	6.905698	NR_028038	chr7:99806620-99806561	GATS
A_24_P129232	10.7253895	10.921169	NM_020755	chr6:122764847-122764788	SERINC1
A_33_P3340649	5.0882716	5.197784	NM_001099219	chr21:32410537-32410478	KRTAP19-8
A_32_P150891	8.770113	8.853287	NM_001042517	chr13:60686214-60686155	DIAPH3
A_23_P201483	7.070023	6.9824533	NM_004759	chr1:206906945-206907004	MAPKAPK2
A_23_P429383	3.6626742	3.475429	NM_014213	chr2:176989029-176989088	HOXD9
A_33_P3327702	11.847378	11.872422	NM_004819	chr19:46318759-46318700	SYMPK
A_33_P3359115	8.912196	8.91897	NM_018368	chr6:70385838-70385779	LMBRD1
A_23_P153286	7.999334	8.059565	NM_006630	chr19:44662151-44662210	ZNF234
A_24_P216253	9.801705	9.735139	NM_014902	chr20:35155492-35155551	DLGAP4
A_23_P314250	7.7353764	7.543674	NM_033387	chr9:134133585-134133526	FAM78A
A_23_P32805	2.7326765	3.3217554	NM_017551	chr10:87361903-87361844	GRID1
A_33_P3326927	6.080084	6.17679	NM_006961	chr16:71508784-71508725	ZNF19
A_33_P3304748	6.5359216	6.307554		chr11:000120748-000120689	
A_33_P3422170	7.069522	7.0630517	NM_194247	chr2:178080329-178080388	HNRNPA3
A_33_P3814721	3.2572997	2.3900566	NM_001031853	chr11:15267563-15267622	INSC
A_23_P167509	5.244362	4.862002	NM_001037332	chr5:156822135-156822194	CYFIP2
A_23_P85560	12.430465	12.477388	NM_003757	chr1:32696749-32696808	EIF3I
A_33_P3404706	4.5560465	3.8440468	NM_001030288	chr16:29681212-29681271	SPN
A_23_P105409	4.814845	5.1124616	NM_006301	chr12:53875961-53875902	MAP3K12
A_23_P144816	11.804407	12.113966	NM_003374	chr5:133307961-133307902	VDAC1
A_24_P51855	4.059722	3.9183936	NM_001947	chr3:52089886-52088352	DUSP7
A_23_P379789	4.896345	4.9306517	NM_013305	chr18:44259258-44259199	ST8SIA5
A_23_P14667	4.8385677	4.4991693	NM_004213	chr15:85478730-85486715	SLC28A1
A_33_P3305102	7.606527	7.690593	NM_170776	chr16:57719614-57719673	GPR97
A_33_P3259736	6.066502	6.9821906	NM_014324	chr5:33988020-33987961	AMACR
A_23_P141021	5.063305	5.828716	NM_017839	chr16:55584943-55585002	LPCAT2
A_23_P571	11.8323965	11.896206	NM_006516	chr1:43391933-43391874	SLC2A1

A_33_P3230583	6.8416557	6.9575615	NR_034006	chr9:68728846-68728905	LOC100132352
A_33_P3362048	4.313105	4.0204377		chr17:18256890-18256831	SHMT1
A_23_P200001	8.333178	8.079973	NM_144573	chr1:78408667-78408726	NEXN
A_32_P99347	6.0604877	5.641462	NR_024376	chr9:115867299-115867240	FAM225B
A_33_P3226788	8.382515	8.134951		chr7:006928851-006928792	
A_33_P3265415	10.579517	10.633954		chr7:55816864-55816923	XLOC_014512
A_24_P500422	4.0471287	4.124735	NR_024403	chr6:52533036-52533095	LOC730101
A_33_P3230324	4.707607	4.180688	NM_198189	chr2:238007370-238007429	COPS8
A_33_P3494109	6.9746675	7.093408	XR_243752	chr17:70351165-70351224	
A_23_P149496	9.143881	9.557933	NM_018053	chr1:28294464-28294523	XKR8
A_33_P3357277	6.052957	5.909662		chr9:138246007-138246066	
A_33_P3405068	9.161412	9.266726	NM_020443	chr1:201789378-201789437	NAV1
A_23_P49546	3.8499022	3.2613153	NM_000835	chr17:72838263-72838204	GRIN2C
A_32_P144220	11.270128	11.416096		chr15:042864166-042864225	
A_23_P15182	10.487129	10.505745	NM_012106	chr16:57287470-57287529	ARL2BP
A_23_P427122	3.7469215	3.5417771	NM_053017	chr11:3660925-3660260	ART5
A_33_P3258997	4.080409	4.286493	NM_024917	chrX:100274020-100273961	TRMT2B
A_33_P3367575	5.214275	5.30706	NM_001164502	chr22:31867890-31867831	EIF4ENIF1
A_23_P132248	6.011687	5.7321672	NM_019103	chr22:30138432-30136686	ZMAT5
A_23_P318420	6.3372717	6.6945515	NM_018704	chr1:113002131-113002190	CTTNBP2NL
A_33_P3324454	8.3511	8.379414	NM_000391	chr11:6636189-6636130	TPP1
A_33_P3376644	5.960801	6.1159506	NM_015571	chr6:76426668-76426727	SENP6
A_24_P658584	7.446471	7.4354553	NM_015278	chr6:148872423-148872482	SASH1
A_23_P170290	6.377883	6.736287	NM_018202	chr1:25817960-25818019	TMEM57
A_23_P502336	2.339362	2.3900566	NM_013447	chr19:14843917-14843858	EMR2
A_23_P252775	7.743072	7.79753	NM_175073	chr9:32972644-32972594	APTX
A_23_P309292	4.585535	4.6578655	NM_001109662	chr12:112688911-112688852	HECTD4
A_33_P3316223	7.812608	7.2240443	NM_003082	chr14:62262703-62262762	SNAPC1
A_33_P3392077	9.896716	10.083698	NM_004881	chr2:24300563-24300504	TP53I3
A_23_P258367	4.6762795	4.8874636	NM_001499	chr9:131296126-131296185	GLE1
A_33_P3615334	3.9676802	4.229378	XR_241160	chr1:182098504-182098445	LOC400799
A_32_P35969	6.3246264	5.380128	NM_001190455	chr15:32462222-32462281	CHRNA7
A_23_P78438	10.1623745	10.160583	NM_018255	chr18:33754520-33754579	ELP2
A_33_P3293336	10.139391	9.74446	NM_005264	chr10:117816498-117816439	GFRA1
A_23_P203949	7.3429127	7.3352823	NM_030653	chr12:31257493-31257552	DDX11
A_23_P38408	7.4536934	7.4472713	NM_024835	chr17:34945927-34945986	GGNBP2
A_33_P3339187	3.6450076	2.9453845	NM_001243552	chr17:51064953-51065012	C17orf112
A_23_P380917	12.098886	12.345019	NM_014300	chr15:85213059-85213000	SEC11A
A_33_P3247086	3.49963	3.4350088		chr13:048708553-048708494	
A_23_P138352	5.342674	5.731429	NM_004185	chr1:113063369-113063428	WNT2B
A_24_P348925	7.18543	7.154971	NM_001099402	chr14:99959862-99959921	CCNK
A_23_P163647	12.075822	11.974861	NM_001919	chr16:2289997-2289938	ECI1
A_23_P154986	5.7308474	5.623204	NM_005265	chr22:25023862-25023921	GGT1
A_24_P91830	3.7576106	3.9136825	NM_001009609	chrX:142596891-142596832	SPANXN3
A_32_P109036	5.2810183	5.3391047	NM_001076678	chr19:21609167-21609226	ZNF493
A_33_P3210516	4.9564433	4.4845004		chr2:020988708-020988649	
A_24_P924202	7.0771527	7.0862703	NM_032223	chr11:65404571-65404630	PCNXL3
A_23_P208198	4.389929	4.8201756	NR_024181	chr19:52359174-52359115	ZNF577
A_24_P308506	5.3041306	6.058975	NM_016347	chr2:73927925-73927866	NAT8B
A_23_P37560	5.498497	5.473748	NM_003847	chr15:90226612-90226553	PEX11A
A_23_P426989	7.6005774	7.815967	NM_030645	chr1:249104894-249104835	SH3BP5L
A_23_P167358	6.124298	6.3425756	NM_001039717	chr4:128951675-128951734	C4orf29
A_33_P3304538	8.575044	9.201248	NM_207396	chr1:6280791-6280850	RNF207
A_33_P3405957	9.329668	9.056994	NM_182557	chr11:118768593-118768534	BCL9L
A_33_P3268564	6.5587997	6.1515136	NM_001004722	chr2:106471685-106471744	NCK2
A_33_P3424057	8.870609	8.8174	NM_006210	chr19:57325766-57325707	PEG3
A_33_P3715909	5.7110043	5.4243875	AF264629	chr16:81108497-81108438	
A_24_P902728	6.774651	6.2285786	NM_203364	chr11:34120092-34120151	CAPRIN1
A_32_P126375	6.9682217	6.725741	NM_198270	chrX:17753480-17753539	NHS

A_33_P3393650	6.680068	7.144817	NM_019012	chr12:19436601-19436660	PLEKHA5
A_33_P3329898	3.9280465	4.2783937		chr15:42964889-42964948	STARD9
A_32_P137266	7.39962	7.1350784	NM_032437	chr1:64036777-64038149	EFCAB7
A_23_P204355	6.862447	6.6504245	NM_001135734	chr12:6776387-6776328	ZNF384
A_24_P212127	8.007417	8.157254	NM_006954	chr10:38343382-38343440	ZNF33A
A_24_P19228	8.176613	8.128403	NM_138924	chr19:1399172-1399012	GAMT
A_33_P3252134	8.31382	8.471797		chr16:030309263-030309322	
A_33_P3259507	8.532999	9.140743	NM_012166	chr9:37510982-37510923	FBXO10
A_24_P941831	6.148676	4.500938	NM_001044385	chr2:202485131-202485072	TMEM237
A_33_P3401138	4.4608183	4.7646685		chr17:019043002-019042943	
A_33_P3269208	3.5967045	4.034044	NM_001001850	chr3:93733276-93733217	STX19
A_23_P160862	12.965004	12.928442	NM_031844	chr1:245018727-245018289	HNRNPU
A_23_P146946	4.413255	2.3900566	NM_001323	chr11:65780838-65780897	CST6
A_33_P3282434	8.063941	7.7085047	NM_001166496	chr1:113454562-113454503	SLC16A1
A_33_P3410599	8.340543	9.280882	NM_017633	chr6:82455540-82455481	FAM46A
A_32_P84242	7.658223	7.183969	NM_015566	chr5:74073811-74073752	FAM169A
A_33_P3234118	9.7093	9.114307	NM_001166599	chrX:133903689-133903630	FAM122B
A_23_P130194	12.165957	11.585232	NM_006907	chr17:79890390-79890331	PYCR1
A_24_P917833	9.437731	9.5629635	NM_006827	chr14:75598728-75598669	TMED10
A_24_P167683	4.1568174	4.106397	NM_014216	chr14:93424584-93418338	ITPK1
A_33_P3343182	5.103961	4.8791866	NM_024726	chr2:237233015-237232956	IQCA1
A_33_P3407400	5.595624	5.866286	NM_144722	chr5:35670285-35670344	SPEF2
A_33_P3313302	4.8677993	4.91321	NM_020214	chr15:72550438-72550379	PARP6
A_23_P97990	10.127256	10.408105	NM_002775	chr10:124274338-124274397	HTRA1
A_23_P319808	6.6474338	6.7888527	NM_015054	chr12:100441339-100433540	UHRF1BP1L
A_23_P373742	2.3221061	2.3900566	NM_178556	chr4:189068571-189068630	TRIML1
A_33_P3264895	7.2077746	7.7062483	NM_144593	chr12:49458528-49458469	RHEBL1
A_32_P155506	5.5487933	5.832404	NM_152653	chr3:23631611-23631670	UBE2E2
A_33_P3285565	2.3221061	2.3900566	NM_001306	chr7:73183386-73183327	CLDN3
A_24_P218688	7.332962	7.705762	NM_000694	chr11:67795665-67795724	ALDH3B1
A_33_P3304372	6.5841722	6.472745	NM_018342	chr4:159176254-159176313	TMEM144
A_24_P253215	9.945501	10.051888	NM_006331	chr12:7084400-7084459	EMG1
A_23_P27332	7.8170137	7.8310013	NM_003199	chr18:52895486-52895237	TCF4
A_33_P3309551	7.757888	8.189749	NM_002839	chr9:8314334-8314275	PTPRD
A_23_P258251	6.5340815	6.5495	NM_182314	chrX:129757371-129757353	ENOX2
A_23_P19322	6.383277	6.119257	NM_018322	chr6:39071944-39071885	SAYSD1
A_23_P44768	8.893131	8.996309	NM_013254	chr12:64895443-64895502	TBK1
A_23_P36795	2.345024	2.3900566	NM_005639	chr12:79843822-79843881	SYT1
A_23_P501745	5.820335	5.5913086	NM_153818	chr1:2336914-2336855	PEX10
A_23_P7636	11.335772	11.417451	NM_004219	chr5:159855644-159855703	PTTG1
A_23_P94921	8.7248745	8.830631	NM_006749	chr8:42274152-42274093	SLC20A2
A_33_P3397785	4.697501	5.0103116	NM_001136504	chr1:202574888-202574829	SYT2
A_33_P3589722	6.0428514	6.1456766	NM_000051	chr11:108099942-108100001	ATM
A_33_P3419190	5.120377	4.7839394	NM_001657	chr4:75318293-75318352	AREG
A_23_P200936	10.051888	10.0129385	NM_000254	chr1:237063523-237063582	MTR
A_23_P39356	4.3998265	4.3532295	NM_005303	chr19:35843279-35843338	FFAR1
A_33_P3285092	5.4714384	5.356965	AK126123	chr4:8243806-8243865	LOC100129763
A_23_P20743	2.4142702	2.3900566	NM_032342	chr9:104237694-104237635	TMEM246
A_33_P3287218	11.597534	11.771183	NM_001143679	chr7:142966142-142966201	GSTK1
A_23_P85140	7.7765946	7.9179077	NM_080390	chrX:101382220-101382279	TCEAL2
A_23_P252193	4.5245104	4.396132	NM_002207	chr3:37860557-37860616	ITGA9
A_23_P46604	5.2064295	5.616111	NM_020448	chr1:24779902-24779961	NIPAL3
A_24_P313334	7.031106	6.915813	NM_001199877	chr15:44087952-44088011	SERF2
A_33_P3249185	8.505054	8.459158		chr10:71917549-71917490	SAR1A
A_23_P51410	6.3835096	6.271596	NM_022743	chr1:246490603-246490544	SMYD3
A_33_P3381191	3.243617	3.3022647	NM_024955	chr22:36897397-36897338	FOXRED2
A_23_P94230	6.427474	5.5792437	NM_015364	chr8:74941211-74941270	LY96
A_32_P46214	5.5525427	5.4467254	NM_173653	chr3:142984209-142984150	SLC9A9
A_33_P3594654	8.877874	8.397176	NM_024121	chr2:120975203-120975144	TMEM185B

A_23_P86002	7.8477125	8.042294	NM_016040	chr1:93622008-93621949	TMED5
A_33_P3258546	4.2239614	4.5655484	NM_031501	chr5:140203700-140203759	PCDHA5
A_23_P168726	3.7578514	3.67623	NM_007009	chr7:49977188-49977129	ZPBP
A_24_P107941	10.932973	10.695141	NM_024598	chr16:58055426-58055485	USB1
A_23_P48886	8.520133	8.959866	NM_001110	chr15:58889314-58889255	ADAM10
A_33_P3353116	3.679107	4.104774	NR_027462	chr17:7531402-7531461	SHBG
A_24_P128145	5.1781707	5.526807	NM_001880	chr2:175979528-175979469	ATF2
A_23_P28878	6.4342012	5.9825196	NM_001039140	chr20:3736127-3735119	C20orf27
A_24_P401100	12.346024	12.333307		chr16:016025917-016025856	
A_33_P3370812	8.880909	8.830886	BC028196	chr4:37954280-37954339	TBC1D1
A_32_P203300	8.684972	8.279307	NM_001130679	chr4:99800634-99800575	EIF4E
A_23_P28857	7.196621	7.1850433	NM_018556	chr20:1609978-1609919	SIRPG
A_24_P227211	5.8266964	5.870092	NM_022822	chr11:66034526-66034585	KLC2
A_23_P167920	7.2723584	7.7765946	NM_005618	chr6:170591701-170591642	DLL1
A_24_P416997	3.4068124	3.0894427	NM_145641	chr22:36536995-36536936	APOL3
A_23_P340188	3.2766366	3.1573873	NM_138819	chrX:133988488-133988547	FAM122C
A_33_P3330841	4.382538	4.286827	NM_004507	chr7:48004060-48004001	HUS1
A_33_P3399114	8.087764	8.225527		chr19:59095614-59095673	CENPBD1P1
A_23_P403335	5.3391047	4.6724215	NM_015065	chr11:108379859-108379800	EXPH5
A_33_P3419735	5.678837	5.8231864		chrUn_gl000229:000012551-000012492	
A_23_P152804	13.407759	13.446781	NM_198175	chr17:49238609-49239123	NME1
A_24_P344416	3.0802166	2.3900566	NM_024423	chr18:28571094-28571035	DSC3
A_33_P3310070	4.8027554	4.567013	NM_004514	chr17:80529686-80529745	FOXK2
A_24_P111511	7.1063495	7.3471165	NM_024753	chr2:166731329-166731270	TTC21B
A_23_P92012	8.481804	8.37397	NM_025265	chr3:12574605-12574664	TSEN2
A_33_P3262327	6.3352227	6.617081		chr12:042984638-042984579	
A_33_P3295203	4.384574	4.3934717	NM_001523	chr19:52216452-52216393	HAS1
A_33_P3260062	4.0217314	4.0836267	AK022481	chr19:4038087-4038146	PIAS4
A_33_P3337277	7.156519	7.216741	AK124859	chr8:30428118-30428177	LOC100129846
A_24_P468810	7.128663	6.7845907	NM_005610	chr1:33151434-33151493	RBBP4
A_23_P53557	8.088122	8.138996	NM_002342	chr12:6500070-6500129	LTBR
A_33_P3375383	10.435975	10.33998	NM_003083	chr19:7988075-7988134	SNAPC2
A_33_P3287203	7.430369	7.2267094	NM_004803	chr3:38359783-38359842	SLC22A14
A_23_P146479	9.639727	9.49355	NM_017503	chr9:136227163-136227222	SURF2
A_32_P98298	5.7756615	5.78905	NM_004420	chr11:1579394-1579141	DUSP8
A_33_P3232692	3.0263596	2.3900566	NM_001185156	chr1:207076757-207076816	IL24
A_33_P3216651	3.7147899	4.2974024	NM_001145310	chr11:71819012-71819071	LRTOMT
A_23_P216017	6.692981	6.771787	NM_004901	chr8:23291962-23291903	ENTPD4
A_33_P3334625	4.1276445	4.313219	AK123321	chr2:242971257-242971316	FLJ41327
A_23_P159110	7.944208	8.026939	NM_018656	chr12:69159583-69159642	SLC35E3
A_33_P3503284	3.7880683	3.3338435	NM_001257964	chr18:54815440-54815499	BOD1L2
A_33_P3352048	3.8083	3.5778866	XR_132470	chr1:1111080-1111021	TTLL10-AS1
A_23_P202696	8.433107	8.136525	NM_016506	chr11:47594090-47594031	KBTBD4
A_23_P88303	3.1052556	2.3900566	NM_021979	chr14:65009538-65009597	HSPA2
A_33_P3390637	4.9114475	4.9517794	NM_144712	chr2:220034117-220034058	SLC23A3
A_33_P3234088	4.0867305	3.9347653		chr3:156881213-156881154	
A_23_P14636	9.537712	9.894249	NM_005926	chr15:44097034-44096975	MFAP1
A_23_P85963	7.6169944	7.4991736	NM_001005189	chr1:158517370-158517311	OR6Y1
A_24_P215407	6.790622	6.743126	NM_004397	chr11:118620979-118620920	DDX6
A_33_P3386726	8.868467	8.852642	NM_001198773	chr1:151265014-151264955	PI4KB
A_24_P92267	10.023861	10.123006		chr1:159729165-159729224	
A_33_P3325110	6.8643265	7.0976553	NM_005686	chr1:204086258-204086317	SOX13
A_24_P270829	4.0474653	4.3338885	NM_000173	chr17:4837421-4837480	GP1BA
A_24_P267664	8.28058	8.105752	NR_026543	chr21:40981575-40981516	C21orf88
A_33_P3366141	4.548714	3.738612	AK094244	chr22:32709608-32709667	LOC100130269
A_23_P57819	8.888643	8.898422	NM_014760	chr3:10322695-10322754	TATDN2
A_23_P58676	4.052624	3.5583267	NM_001204375	chr5:32791717-32791776	NPR3
A_23_P53039	7.8508267	7.9724927	NM_002301	chr11:18472532-18472591	LDHC

A_24_P257416	6.3289466	6.375927	NM_002089	chr4:74964401-74964342	CXCL2
A_23_P34537	7.033099	6.5401273	NM_000120	chr1:226032883-226032942	EPHX1
A_23_P13073	8.532848	8.380176	NM_031307	chr11:125763766-125763707	PUS3
A_33_P3295108	7.198572	6.936648	NM_198141	chr15:42645781-42645840	GANC
A_33_P3235975	6.389549	6.5357018	NM_032343	chr3:126633533-126633592	CHCHD6
A_33_P3691916	6.730869	6.678899	NM_014883	chr4:89647940-89647881	FAM13A
A_33_P3230176	4.512016	4.360532	NM_033505	chr2:26611912-26611971	EPT1
A_33_P3227225	5.0959206	5.164337		chr2:132056513-132056454	
A_24_P142743	9.184591	9.580804	NM_004368	chr19:1038261-1038320	CNN2
A_33_P3265514	4.0035124	4.3091745	NM_199261	chr21:10906861-10906802	TPTE
A_32_P191527	7.1523905	6.9711227	NM_020382	chr12:123892738-123892797	SETD8
A_23_P26314	7.575979	7.162099	NM_020807	chr16:58028710-58028651	ZNF319
A_32_P60459	4.4135637	5.0817146	NM_001145373	chr10:23730613-23730672	OTUD1
A_33_P3378514	4.8409386	4.4226356	NM_001083	chr4:120415615-120415556	PDE5A
A_32_P135336	5.8712783	5.5126076	NR_002556	chr16:29476353-29476294	LOC388242
A_23_P13065	7.472636	7.468962	NM_019028	chr11:19194361-19197421	ZDHHC13
A_33_P3285024	6.780275	6.9582586	NM_053045	chr9:140099598-140099539	TMEM203
A_33_P3303380	4.432579	5.023452	XM_005252536	chr10:34635185-34635126	PARD3
A_33_P3375708	3.714231	3.582073		chr5:95169986-95169927	XLOC_014512
A_23_P16214	6.0936165	6.417308	NM_032301	chr19:12800227-12800087	FBXW9
A_33_P3407062	4.6905117	4.7687554	AB306126	chr14:22789024-22789083	
A_33_P3210085	8.565222	8.41609	NM_001047160	chr10:5499460-5499519	NET1
A_33_P3377110	7.948363	7.462261	NM_001134404	chr1:110042969-110043028	CYB561D1
A_32_P125771	4.8218946	4.6670704	NM_015668	chr8:100974663-100973407	RGS22
A_24_P292964	6.630571	6.828534	NM_001278198	chr17:46056238-46057972	CDK5RAP3
A_23_P366983	3.2165656	3.0010397	NM_013381	chr12:73058821-73058879	TRHDE
A_23_P414913	7.607813	7.6546555	NM_022343	chr9:36163763-36163822	GLIPR2
A_33_P3286066	4.03168	4.441645	NM_001134438	chr3:111694456-111694515	PHLDB2
A_33_P3351914	10.981148	11.09206	NM_004544	chr2:240900217-240900158	NDUFA10
A_23_P156970	2.3221061	2.3900566	NM_002402	chr7:130145648-130145707	MEST
A_23_P348138	5.2531376	5.3401675	NM_020831	chr22:40806886-40806827	MKL1
A_24_P19828	5.740258	5.4914503	NM_025077	chr1:45808854-45808913	TOE1
A_33_P3214303	10.169423	10.277651	NM_001244808	chr3:71004993-71004934	FOXP1
A_23_P134517	7.679977	7.5625954	NM_033224	chr7:44922674-44922615	PURB
A_23_P12643	6.8520575	6.824752	NM_020682	chr10:104638748-104650359	AS3MT
A_23_P121586	6.5205765	6.29665	NM_014606	chr4:89629429-89629488	HERC3
A_33_P3376007	11.350119	11.208235	NM_175852	chr1:32663826-32663885	TXLNA
A_24_P363679	8.298608	7.504654	NM_015941	chr8:54684629-54684570	ATP6V1H
A_23_P70701	7.690593	7.7986593	NM_017519	chr6:157529022-157529081	ARID1B
A_33_P3296240	6.492727	6.386614	NM_017738	chr9:17487004-17487063	CNTLN
A_33_P3302320	5.439115	5.38536	XR_242543	chr9:33733032-33732973	
A_24_P153324	13.554734	13.646116		chr13:077555683-077555626	
A_32_P134209	5.4093876	5.513728	NM_001106	chr3:38534307-38534366	ACVR2B
A_24_P102293	5.934943	5.763902	NM_015567	chr13:88331586-88331645	SLITRK5
A_33_P3651911	6.9309006	7.044365	AK092172	chr8:141536829-141536770	
A_23_P34307	7.5741014	7.6050205	NM_005482	chr1:77557523-77557464	PIGK
A_33_P3317731	4.0336676	4.378753	AK130876	chr22:41734185-41734244	
A_23_P400147	9.825396	10.350428	NM_001267723	chr19:46520544-46521376	CCDC61
A_23_P17490	9.288903	9.117691	NM_001009924	chr20:5081129-5081070	TMEM230
A_24_P322395	6.934184	6.8455834	NM_080732	chr19:41313434-41313750	EGLN2
A_33_P3346327	4.0362654	3.1206868	NR_003674	chr9:46687913-46687972	KGFLP1
A_33_P3237260	3.996953	4.1610007	NM_198498	chr11:111156911-111156970	C11orf53
A_23_P416112	4.727216	3.953467	NM_152617	chr3:196198755-196198696	RNF168
A_33_P3269578	7.614846	7.4031577	NM_018313	chr3:52582118-52582059	PBRM1
A_24_P461497	7.9431067	7.9817204		chr19:009629504-009629563	
A_32_P38637	8.249856	8.213596	NM_032534	chr7:149431584-149431643	KRBA1
A_33_P3374170	4.7471685	4.462595		chr3:011276251-011276192	
A_33_P3273623	4.4169064	4.3181477	NM_002989	chr9:34709633-34709574	CCL21
A_24_P400832	4.114725	4.0217314	NR_034082	chr17:5118521-5118580	LOC100130950

A_23_P422794	8.823403	9.018101	NM_173685	chr8:126370013-126379021	NSMCE2
A_33_P3303104	6.283389	6.473361	NM_001185119	chr3:169492289-169492348	MYNN
A_33_P3332302	4.790559	4.038941	NR_027025	chr17:54962202-54962143	MTVR2
A_23_P83098	2.9851525	2.3900566	NM_000689	chr9:75516195-75516136	ALDH1A1
A_23_P161091	7.3869653	7.543785	NM_024772	chr1:35581035-35581094	ZMYM1
A_23_P348159	6.0417585	6.0534463	XM_005249317	chr6:56468910-56468851	DST
A_33_P3336696	16.65262	16.65262	AK311996	chrM:10708-10767	ND4L
A_33_P3303325	7.4407187	6.9992833	XM_003960117	chr1:22649480-22649421	LOC101060363
A_33_P3290888	9.028243	9.25296	NM_003915	chr20:34214719-34214660	CPNE1
A_24_P20032	9.079533	9.207531	NM_000449	chr1:151313395-151313336	RFX5
A_33_P3320538	7.0640116	6.238842	XM_005266601	chr13:25910360-25910419	NUPL1
A_23_P410312	7.155588	6.9973497	NM_207435	chr12:110479148-110479089	C12orf76
A_33_P3405904	5.1173153	4.8746557		chr17:043551785-043551726	
A_23_P349882	8.142032	7.7706165	NM_005388	chr9:125580959-125580900	PDCL
A_33_P3333146	3.7983887	3.3987002	NM_015085	chr17:2934231-2934290	RAP1GAP2
A_23_P408675	7.2520933	7.700428	NM_003972	chr10:93789192-93789251	BTAF1
A_23_P316960	7.068443	7.328573	NM_000837	chr8:145066955-145067014	GRINA
A_33_P3309526	6.1344004	5.9488754	NM_002838	chr1:198725541-198725600	PTPRC
A_24_P369232	2.3221061	2.3900566	NM_031455	chr10:12938688-12938629	CCDC3
A_23_P145388	12.35481	12.117973	NM_014341	chr6:36936110-36936051	MTCH1
A_33_P3376249	14.129131	13.685942	NM_005978	chr1:153533890-153533831	S100A2
A_23_P206792	3.8060513	3.5529222	NM_033410	chr16:30566204-30566145	ZNF764
A_24_P155791	5.3635626	5.355774	NM_024708	chr15:101169942-101170001	ASB7
A_23_P253412	10.561575	10.1319065	NM_019051	chr9:104152509-104152450	MRPL50
A_33_P3881262	4.9926677	5.207198	NM_156039	chr1:36931755-36931696	CSF3R
A_24_P131392	5.221525	5.347834	NM_144713	chr2:38231143-38231293	RMDN2
A_33_P3341970	6.634517	6.6199713	NM_173808	chr1:71868720-71868661	NEGR1
A_23_P154855	4.0642867	4.374522	NM_000219	chr21:35821614-35821555	KCNE1
A_33_P3296024	6.992693	6.9671087	NM_171999	chr18:76757313-76757372	SALL3
A_33_P3218760	10.677486	10.588733	NM_080599	chr10:12077296-12077237	UPF2
A_24_P29001	11.823778	11.890519	NM_014463	chr3:14239595-14239654	LSM3
A_33_P3209915	5.6268377	5.651871	NM_001099666	chr9:72333183-72333124	PTAR1
A_33_P3389603	4.0204377	3.730667	AK131572	chr6:111630997-111631056	
A_23_P216920	9.820163	9.93647	NM_014397	chr9:127114617-127114676	NEK6
A_23_P127964	10.860082	10.985409	NM_199418	chr11:82535525-82535466	PRCP
A_24_P415845	5.5185995	5.5463624	NM_002312	chr13:108860815-108860756	LIG4
A_32_P191860	4.566723	5.092385	NM_001025591	chr19:35084403-35084346	SCGB2B2
A_33_P3305725	5.898294	5.8308153	NR_037167	chr1:178003902-178003843	LOC730102
A_23_P371876	7.2936006	7.1461687	NM_138775	chr11:107375618-107375559	ALKBH8
A_23_P151506	9.810196	9.377115	NM_016445	chr14:67853870-67853811	PLEK2
A_24_P120109	4.082573	4.22915	NM_198963	chr2:39088500-39088441	DHX57
A_32_P139738	5.633725	5.562374	NR_002827	chr16:32163218-32163159	HERC2P4
A_24_P261052	5.7500052	4.7150865	NM_015458	chr8:11184845-11184904	MTMR9
A_33_P3266165	14.761112	15.021793	XM_003118689	chr16:11536126-11536067	
A_33_P3386765	9.936146	10.056668	NM_015407	chr3:52015151-52015210	ABHD14A
A_33_P3321682	9.062575	9.151299		chr2:236673342-236673283	
A_24_P124973	6.1985064	6.373099	NM_138704	chr15:29561261-29561202	NDNL2
A_33_P3275668	11.7002945	11.72962	NM_004639	chr6:31606925-31606866	BAG6
A_24_P341222	8.606982	8.776876	NM_001145432	chr4:25930817-25930876	SMIM20
A_33_P3348239	3.8101814	4.394286	NM_000138	chr15:48700564-48700505	FBN1
A_24_P217904	7.6546555	7.2971725	NM_033502	chr6:42193530-42193471	TRERF1
A_23_P368886	5.930913	4.6194053	NM_175856	chr5:129521308-129521367	CHSY3
A_33_P3323979	3.641938	3.5367186	NR_038976	chr3:131046199-131046140	LOC339874
A_23_P88201	9.673813	9.618252	NM_017917	chr14:35554863-35554804	PPP2R3C
A_23_P160318	10.246744	10.049269	NM_001856	chr1:32118234-32118175	COL16A1
A_24_P217330	5.8859253	5.8052697	NM_032806	chr3:43121424-43121365	POMGNT2
A_23_P26649	7.6280293	7.679977	NM_020677	chr16:4516184-4513823	NMRAL1
A_23_P9135	6.435124	6.752825	NM_033655	chr9:39073363-39073304	CNTNAP3
A_24_P181120	11.616554	11.755066	NM_002624	chr12:53691919-53693096	PFDN5

A_33_P3240757	4.3042755	4.5835066	NM_001007237	chr1:117131569-117131510	IGSF3
A_33_P3411357	7.645339	7.8610687	NR_024157	chr22:19033742-19033683	DGCR11
A_23_P89030	7.7312455	7.7368684	NM_001195124	chr16:87336506-87336447	C16orf95
A_23_P312646	8.992858	8.884228	NM_183241	chr9:139888109-139888269	C9orf142
A_33_P3219803	16.283295	16.323286	NM_001099285	chr2:232577522-232577581	PTMA
A_23_P200096	4.2631316	4.288531	NM_025106	chr1:9416578-9416637	SPSB1
A_23_P323563	8.011028	8.116887	NM_022835	chr19:39915991-39916050	PLEKHG2
A_32_P190951	2.5333455	2.3900566	AY393117	chr14:106967160-106967101	
A_23_P108662	10.18601	10.136913	NM_006302	chr2:74688343-74688284	MOGS
A_33_P3346635	4.959448	5.0563974	AK125281	chr7:197863-197922	LOC100132244
A_33_P3354001	4.457274	4.595816	NM_001167940	chr7:121958550-121958491	CADPS2
A_23_P111981	5.983557	5.6214495	NM_177457	chr8:143852799-143852740	LYNX1
A_23_P95640	8.254663	8.786137	NM_001007544	chr1:206239073-206239014	C1orf186
A_23_P40989	10.058578	10.314959	NM_003940	chr3:179483557-179483616	USP13
A_33_P3375541	5.4261956	5.7476063	NM_000732	chr11:118209862-118209803	CD3D
A_33_P3412560	6.508811	6.813148	AA504818	chr19:11143339-11143280	
A_23_P304237	5.5133514	5.490257	NM_198679	chr9:134453084-134453025	RAPGEF1
A_33_P3289145	7.718177	7.823497	NM_198189	chr2:237998575-237998634	COPS8
A_23_P257198	10.818123	10.721731	NM_002495	chr5:52942229-52954433	NDUFS4
A_33_P3419835	5.9318953	5.2362485	NR_024348	chr16:30931001-30930942	FBXL19-AS1
A_24_P305662	5.6150594	5.32281	NM_022727	chr22:20100983-20100727	TRMT2A
A_33_P3380523	5.308656	5.505585		chr10:25197491-25197432	PRTFDC1
A_33_P3335910	3.9480991	3.4600832	NM_033071	chr6:152757134-152757076	SYNE1
A_23_P319572	4.3287168	3.6058307	NM_001077482	chr1:161199675-161199616	NR1I3
A_24_P884376	8.438221	8.455558		chr7:45854779-45854838	XLOC_014512
A_33_P3247255	5.0330067	5.1493397	NM_017905	chr13:114202669-114202728	TMCO3
A_33_P3340682	5.6214495	5.343785	NM_001113525	chr16:89806251-89806310	ZNF276
A_33_P3232688	4.254191	3.2236214		chrX:1429133-1429192	CSF2RA
A_33_P3282840	7.4998083	7.3821583	NM_001030001	chr14:50044133-50044074	RPS29
A_23_P25605	7.098796	7.312498	NM_032138	chr13:41766213-41766154	KBTBD7
A_23_P135381	2.3221061	2.3900566	NM_001003845	chr2:171574290-171574349	SP5
A_33_P3253812	4.615377	5.373126		chrUn_gl000213:000070595-000070536	
A_23_P60180	8.646215	8.701495	NM_005157	chr9:133762589-133762648	ABL1
A_23_P167201	4.8841643	4.9998446	NM_016955	chr4:25128912-25127374	SEPSECS
A_33_P3405839	10.342867	10.413502	NM_175722	chr2:1440030-1440089	TPO
A_33_P3380612	4.5646634	4.7762656	NM_005529	chr1:22213785-22213726	HSPG2
A_23_P139575	11.993935	11.920317	NM_001005354	chr12:53840341-53840400	PRR13
A_23_P119377	5.1344557	5.143611	NM_004228	chr19:48975593-48975652	CYTH2
A_23_P109427	7.382924	7.5813513	NM_000854	chr22:24325921-24325980	GSTT2
A_32_P47754	4.114524	5.290047	NM_001286233	chr12:7965262-7965203	SLC2A14
A_33_P3271755	3.8543077	3.5858154	NM_031244	chr6:13588636-13588695	SIRT5
A_23_P71972	4.2329817	4.204921	NM_016373	chr16:78458922-78466413	WWOX
A_24_P246173	7.690781	8.065882	NM_004145	chr19:17323382-17323441	MYO9B
A_33_P3389973	4.4575405	4.4441385	NM_001286554	chr6:41764354-41764295	USP49
A_32_P135243	8.118	7.947496	NM_015440	chr6:151355724-151358147	MTHFD1L
A_33_P3611762	7.7200603	7.480794	NR_103446	chr6:52448705-52448764	TRAM2-AS1
A_24_P333106	13.69577	13.8081455		chr14:035163629-035163688	
A_33_P3298587	10.999563	11.119812	NM_014963	chr19:1107721-1107662	SBNO2
A_23_P169409	10.144569	10.154841	NM_148179	chr9:34610612-34610553	RPP25L
A_33_P3258953	6.025563	5.9548836	NR_003290	chr12:132610650-132610709	EP400NL
A_33_P3261246	6.067991	5.9572716	NM_000997	chr5:40831489-40831430	RPL37
A_33_P3390217	5.189437	5.255922	XM_005260310	chr20:30531923-30531982	TTLL9
A_23_P53541	8.7485285	8.5984125	NM_001273	chr12:6680177-6680118	CHD4
A_32_P155416	5.92906	5.45362	NM_024066	chr1:44774039-44750578	ERI3
A_23_P129075	4.589016	4.1668434	NM_024908	chr15:44158615-44158674	WDR76
A_23_P18246	6.344873	6.358537	NM_005283	chr3:46062985-46062926	XCR1
A_23_P118435	12.338233	12.584641	NM_006937	chr17:73164453-73164394	SUMO2
A_24_P199929	5.061861	5.7358723	NM_016516	chr2:64139556-64139497	VPS54

A_33_P3224745	10.391334	10.850294	NM_012320	chr16:68294900-68294959	PLA2G15
A_23_P70480	13.453424	13.291063	NM_003546	chr6:27841008-27840949	HIST1H4L
A_23_P98900	6.88156	7.2840858	NM_025140	chr12:124421269-124421210	CCDC92
A_23_P216052	3.607549	2.3900566	NM_032899	chr8:124220354-124220413	FAM83A
A_23_P258381	8.413672	8.7843275	NM_080862	chr3:140867372-140867431	SPSB4
A_33_P3308744	2.3221061	2.3900566	NM_001105206	chr6:112506499-112506440	LAMA4
A_24_P57367	6.8645773	6.947016	NM_000687	chr20:32878215-32878156	AHCY
A_23_P202170	10.819114	11.002604	NM_012215	chr10:103544394-103544335	MGEA5
A_24_P212129	6.8513355	7.231949	NM_006974	chr10:38306221-38306280	ZNF33A
A_33_P3254660	3.9747875	2.3900566	AK124971	chr22:21307965-21307906	
A_33_P3302393	8.7070675	9.00584	NM_024731	chr16:84695856-84695915	KLHL36
A_33_P3370930	7.2428074	7.302766		chr7:107643457-107643398	LAMB1
A_23_P160438	5.3664603	5.1081114	NM_002479	chr1:203052930-203052871	MYOG
A_33_P3217764	5.120509	4.5727177		chr4:1145860-1145919	FLJ35816
A_23_P163353	10.145846	10.250338	NM_016166	chr15:68468820-68468879	PIAS1
A_32_P18493	12.08983	12.397317		chr4:141287624-141287565	
A_23_P132277	10.288172	10.351247	NM_006739	chr22:35819307-35820178	MCM5
A_32_P183218	8.419894	8.420075	NM_153695	chr9:99148471-99148412	ZNF367
A_24_P643587	5.394764	5.598241	NM_024095	chr12:48542188-48542129	ASB8
A_24_P942441	2.3221061	2.3900566	NM_001135659	chr2:50146765-50146706	NRXN1
A_23_P89884	9.407947	9.159546	NM_005762	chr19:59061599-59061750	TRIM28
A_23_P326691	10.916527	10.805097	NM_005610	chr1:33145808-33145867	RBBP4
A_23_P353125	4.9632845	4.74247	NM_152597	chr15:39909944-39893077	FSIP1
A_23_P109229	3.762886	4.107576	NM_080675	chr20:31590680-31590422	SUN5
A_33_P3331511	5.37105	5.1963873		chr12:116973091-116973150	LINC00173
A_23_P337550	8.524393	8.834667	NM_017602	chrX:48779694-48779635	OTUD5
A_33_P3333033	12.791592	12.960289	AK124883	chr17:2277683-2277742	SGSM2
A_23_P303851	4.099843	4.5108314	NM_176886		TAS2R45
A_23_P169257	4.424256	4.170315	NM_001244	chr9:117666194-117666135	TNFSF8
A_23_P38584	2.3221061	2.3900566	NM_181537	chr17:38933129-38933070	KRT27
A_33_P3387796	5.520207	5.8115587	NM_015157	chr11:118527421-118527480	PHLDB1
A_24_P11791	10.990342	11.027883	NM_002268	chr3:160218102-160218043	KPNA4
A_33_P3291871	3.210745	2.3900566		chr3:046475106-046475047	
A_23_P57110	2.3221061	2.3900566	NM_033409	chr20:741069-741010	SLC52A3
A_23_P97700	9.490767	10.160844	NM_006472	chr1:145442447-145442506	TXNIP
A_24_P317762	7.1587076	7.3139334	NM_002346	chr8:144102816-144103007	LY6E
A_33_P3271246	4.161505	3.6220353		chr9:123561786-123561845	
A_33_P3221793	5.0266085	5.319129		chrX:137793871-137793812	
A_24_P39101	7.4900613	7.674095	NM_031954	chr12:109887019-109886960	KCTD10
A_33_P3209321	6.216094	6.291393	AK096995	chr4:151500399-151500458	
A_23_P130836	7.526454	7.6535287	NM_005317	chr19:549679-549738	GZMM
A_33_P3326922	5.016343	4.464243	AK129975	chr5:156811533-156811592	
A_23_P202345	8.247118	7.988392	NM_032804	chr10:64567804-64567863	ADO
A_23_P15285	7.6212206	7.637421	NM_024109	chr16:8736360-8736419	METTL22
A_23_P200298	8.037224	7.5291553	NM_000028	chr1:100389229-100389288	AGL
A_23_P50331	8.429056	8.490349	NM_014077	chr19:16301870-16301929	FAM32A
A_33_P3297545	5.8685837	5.949162		chr7:142124310-142124251	
A_24_P608302	3.388229	3.6940675	BC012894	chr20:37049947-37049888	
A_33_P3410372	5.8495984	5.745365	NM_153183	chrX:51076242-51076301	NUDT10
A_24_P318990	4.7876883	4.6536636	BC012876	chr22:22682080-22682139	
A_24_P830667	14.05889	14.009318	NM_000982	chr13:27828361-27829379	RPL21
A_23_P31765	8.440352	8.3543005	NM_006823	chr8:79515306-79515365	PKIA
A_23_P311087	9.1358595	9.339848	NM_012482	chr1:200376392-200376333	ZNF281
A_33_P3342300	3.7975965	3.29016	NM_001256272	chr20:25052185-25052126	VSX1
A_33_P3414851	4.9511166	4.6090946	NM_018150	chr1:45111090-45111149	RNF220
A_23_P68106	11.649141	11.841438	NM_021103	chr2:85132798-85133141	TMSB10
A_23_P55688	5.613926	5.866088	NM_017879	chr19:58083072-58083013	ZNF416
A_24_P636318	8.187498	7.924167	NM_001009993	chr2:131806140-131806081	FAM168B
A_33_P3279431	7.9376965	7.1371098	NM_001007024	chr17:28853599-28853658	GOSR1

A_33_P3259548	4.8647985	5.050791	NM_019069	chr3:122133688-122133629	WDR5B
A_23_P131846	9.0484	9.109683	NM_005985	chr20:48605170-48605229	SNAI1
A_23_P108514	6.5817933	6.9387684	NM_001008910	chr2:220113351-220113410	STK16
A_33_P3399864	4.104257	4.315037	NM_175872	chr19:35449481-35449422	ZNF792
A_23_P79259	6.0135727	6.6654634	NM_014521	chr2:235963306-235963365	SH3BP4
A_24_P810697	7.061887	7.221232	NM_001008529	chr17:74676626-74676567	MXRA7
A_23_P141032	11.559139	11.2657795	NM_001861	chr16:85834852-85838569	COX4I1
A_32_P150856	8.232511	7.91077	NR_002144	chr7:128767553-128767612	LOC407835
A_33_P3212982	6.6402187	6.2348223	NM_014798	chr17:43514868-43514809	PLEKHM1
A_23_P145089	12.190776	12.359329	NM_007355	chr6:44219983-44220819	HSP90AB1
A_33_P3317258	4.492619	4.4921365	X15019	chr7:38389015-38388956	
A_33_P3373375	9.694559	9.765133	NM_004356	chr11:2418037-2418096	CD81
A_23_P416036	9.196384	8.795741	NM_017518	chrX:152719858-152713315	HAUS7
A_33_P3292198	4.7732296	5.003235	NM_080675	chr20:31571645-31571586	SUN5
A_23_P23502	4.3199763	4.0655866	NM_001005185	chr1:158736408-158736349	OR6N1
A_33_P3389148	8.111695	8.25495	NM_000455	chr19:1227997-1228056	STK11
A_24_P178631	7.6154704	7.306528	AK027667	chr17_gl000205_random:45223-45282	
A_33_P3417955	3.703727	3.0252945		chr15:33529184-33529125	TMCO5B
A_33_P3215953	8.504842	8.669373	NM_024569	chr1:167742545-167742604	MPZL1
A_33_P3256113	6.1423016	5.776423	NR_027026	chr5:21589381-21589440	GUSBP1
A_23_P359655	8.786137	8.140058	NM_152437	chr12:124499566-124499625	ZNF664
A_23_P8400	8.354477	8.223947	NM_004935	chr7:150751501-150751330	CDK5
A_24_P186539	3.9512343	4.4957457	NM_021155	chr19:7810701-7810642	CD209
A_23_P28590	8.292873	8.032389	NM_024622	chr2:170393797-170393738	FASTKD1
A_23_P116523	3.9230413	4.0840054	AF116668	chr11:33903239-33903298	
A_33_P3220612	8.98626	9.071459	NM_001098784	chr11:65341435-65341494	FAM89B
A_33_P3233649	4.682235	4.68843	NM_001085411	chr5:36227658-36227599	NADK2
A_33_P3366048	2.8515306	2.3900566	NM_001256798	chr20:31034910-31034851	C20orf112
A_33_P3832341	7.882756	7.5066433	BU955376		SCARNA22
A_33_P3369034	12.718337	12.871479	NM_001170714	chr16:75263017-75262958	BCAR1
A_33_P3221448	6.6403723	6.35743	NM_003417	chr19:57734112-57734171	ZNF264
A_23_P256855	7.306185	7.014024	NM_020750	chr6:43490642-43490583	XPO5
A_32_P799227	3.6971428	3.9762836	NM_001278495	chr10:81472181-81472240	NUTM2B
A_24_P359671	4.0891514	4.606724	AB023193	chr1:107952731-107952790	NTNG1
A_24_P286465	8.847062	7.6538453	NM_033224	chr7:44916101-44916042	PURB
A_23_P124570	4.9862895	5.3205338	NM_005246	chr5:108436176-108516462	FER
A_24_P386639	5.8992558	5.864142	NM_016526	chr11:203585-203526	BET1L
A_33_P3380331	6.4895706	6.552709	NM_001144927	chr17:40175899-40175958	NKIRAS2
A_33_P3332724	3.1468031	3.5230083	NM_015120	chr2:73682362-73682421	ALMS1
A_33_P3344156	3.6927195	3.9376726	NM_001105572	chr1:20490544-20490485	PLA2G2C
A_24_P86389	8.948069	8.97832	NM_003514	chr6:27860636-27860577	HIST1H2AM
A_23_P256205	7.0230346	6.424517	NM_014945	chr5:148639682-148639741	ABLIM3
A_33_P3384548	6.5118184	6.536378	AK309505	chr19:46275703-46275762	
A_33_P3334343	4.6260905	4.158574	NM_004667	chr15:28413617-28413558	HERC2
A_23_P78018	6.4619374	6.5430255	NM_018672	chr17:67243589-67243530	ABCA5
A_33_P3351664	5.613639	5.5444603	NM_198082	chr17:80059608-80059549	CCDC57
A_24_P206776	4.5346184	5.190082	NM_001885	chr11:111779635-111779576	CRYAB
A_23_P501435	7.5851126	7.6054716	NM_020536	chr20:18168563-18168622	CSRP2BP
A_24_P7584	4.7698674	4.7834463	NM_025262	chr6:31647012-31646953	LY6G5C
A_23_P333951	7.598565	7.570616	NM_144989	chr1:225155177-225155236	DNAH14
A_23_P75430	9.5521	9.622824	NM_020179	chr11:93211791-93211732	SMCO4
A_33_P3316878	9.717148	9.545637	NM_024536	chr2:220403737-220403678	CHPF
A_23_P306105	8.00085	8.202842	NM_020474	chr18:33291320-33291379	GALNT1
A_33_P3298057	4.376174	4.8873253	NM_001023587	chr3:183705645-183705586	ABCC5
A_23_P144999	8.05273	8.001297	NM_016340	chr5:130762134-130762075	RAPGEF6
A_32_P88349	6.191882	6.1838484	XM_005272745	chr1:149576615-149576594	
A_23_P20823	9.264941	9.506793	NM_052844	chr9:131396209-131396150	WDR34
A_24_P345822	8.609471	9.224987	NM_006070	chr3:100463745-100467021	TFG

A_23_P9603	11.586371	11.842713	NM_006904	chr8:48686360-48686301	PRKDC
A_24_P36944	5.399542	5.7501287	NM_014812	chr1:243289868-243289809	CEP170
A_33_P3414907	4.097224	4.1942787	NM_001200050	chr1:182798740-182798799	NPL
A_33_P3751889	9.207531	9.43269	NM_001135732	chr22:35743895-35743954	TOM1
A_23_P30175	10.382055	10.800127	NM_018695	chr5:65375840-65375899	ERBB2IP
A_33_P3288609	4.2774878	4.152301		chr11:77185567-77185508	PAK1
A_33_P3257457	5.6053553	5.846779	NM_153809	chr9:32630513-32630454	TAF1L
A_33_P3886707	11.441853	11.320887	NM_176869	chr4:106290890-106290831	PPA2
A_33_P3390107	10.693255	11.153033	NR_003286	chrUn_gl000220:154856-154915	RNA18S5
A_23_P330419	12.402329	12.567562	NM_033068	chr19:51297824-51298087	ACPT
A_24_P275828	6.6918445	6.435124	NM_033419	chr17:37827599-37827540	PGAP3
A_23_P204087	4.6135798	4.842542	NM_016817	chr12:113448601-113448660	OAS2
A_24_P86240	6.5833015	6.6094685	NM_198892	chr4:79833009-79833068	BMP2K
A_23_P318039	5.9391475	5.4542656	NM_005444	chr2:219445439-219447728	RQCD1
A_33_P3270941	4.13274	3.9548697	NM_207401	chr1:247273533-247273474	C1orf229
A_24_P739582	6.3056526	6.8434644		chrX:092477455-092477396	
A_33_P3251283	6.793709	6.8867087	NM_198495	chr7:143882559-143882618	CTAGE4
A_23_P337917	5.0917516	5.232066	NM_177444	chr12:27731136-27786317	PPFIBP1
A_33_P3393806	5.5369024	5.2005477	AY987009	chr1:17322413-17322354	ATP13A2
A_32_P232559	7.7858586	7.622586	NR_036502	chr10:6626088-6626147	PRKCQ-AS1
A_23_P77117	8.25495	8.056334	NM_023003	chr15:83793449-83793508	TM6SF1
A_33_P3251932	5.846779	5.780537	NM_207002	chr2:111886352-111886411	BCL2L11
A_24_P926400	6.6826286	6.630275	NM_001001561	chr22:38013746-38013805	GGA1
A_33_P3240507	7.764095	6.833783	NM_138444	chr13:77456107-77456048	KCTD12
A_33_P3273580	4.7055235	5.0938907	AF289570	chr2:233509133-233509192	
A_33_P3258117	7.782379	7.6212206	NM_018063	chr10:96334433-96334492	HELLS
A_32_P42946	4.3091745	3.8487873	NM_182517	chr1:43748556-43748497	C1orf210
A_33_P3308553	5.571309	5.5661325		chr2:194142265-194142206	
A_24_P336276	4.898268	4.940711	NM_013272	chr15:92694193-92705993	SLCO3A1
A_33_P3405916	3.6913633	3.5070224	NR_038368	chr16:33961172-33961113	LINC00273
A_24_P390403	9.565326	9.69522	NM_015138	chr15:41774883-41774942	RTF1
A_33_P3346891	5.927511	5.798969	NM_001144755	chr8:67514728-67514669	MYBL1
A_33_P3336422	9.142192	9.338451	NR_034137	chr11:10900760-10900819	ZBED5-AS1
A_33_P3387621	9.210001	9.208345	NM_033103	chr19:33469558-33469499	RHPN2
A_23_P129956	9.022532	8.921387	NM_004090	chr17:41844207-41844148	DUSP3
A_33_P3282489	6.904564	6.4801283	NM_001097634	chr9:79122271-79122330	GCNT1
A_23_P30884	12.992632	12.794319	NM_001288	chr6:31700105-31700046	CLIC1
A_33_P3827035	4.629124	4.8815885	NR_003291	chr3:197785172-197785113	ANKRD18DP
A_24_P684186	7.8814163	7.802499	NM_198449	chr5:49692394-49692335	EMB
A_23_P141847	9.476523	8.753551	NM_003969	chr19:59067745-59067686	UBE2M
A_33_P3241753	11.938358	11.935347	NM_018838	chr12:95365169-95365110	NDUFA12
A_23_P94501	13.186493	13.166815	NM_000700	chr9:75785002-75785061	ANXA1
A_23_P53856	10.975435	10.642863	NM_014887	chr13:33091850-33091791	N4BP2L2
A_23_P88630	9.213035	8.994392	NM_000057	chr15:91358495-91358554	BLM
A_33_P3246985	7.308793	7.2828026	NM_022359	chr1:144952275-144952216	PDE4DIP
A_33_P3352767	7.3934693	6.960627	NM_002386		MC1R
A_33_P3250840	8.500457	8.560089	NM_144599	chr15:23043421-23043362	NIPA1
A_23_P204324	8.545942	8.789482	NM_012062	chr12:32896876-32896935	DNM1L
A_23_P23356	10.260525	9.812664	NM_016052	chr1:218504518-218504577	RRP15
A_33_P3344733	8.669704	8.54245	NM_025152	chr14:32330340-32330399	NUBPL
A_33_P3374221	4.313822	4.478689	AK127740	chr6:7107997-7107938	LOC643936
A_24_P282343	6.065847	6.418096	NM_003159	chrX:18638000-18638059	CDKL5
A_23_P17382	7.328573	7.2341495	NM_022095	chr20:44577685-44577626	ZNF335
A_23_P387000	3.60956	3.8304787	NM_173683	chr8:10753737-10753678	XKR6
A_23_P213247	9.826316	10.072767	NM_012161	chr4:15607016-15606957	FBXL5
A_33_P3268974	5.0563974	5.147558	NM_001003716	chr17:73646506-73646447	RECQL5
A_23_P101332	9.011038	9.122261	NM_023008	chr19:10664573-10664514	KRI1
A_24_P81298	8.123393	7.7117677	NM_001123355	chr9:127908953-127908894	PPP6C

A_24_P328524	4.6561155	4.7055235	NM_003947	chr3:124238618-124238677	KALRN
A_24_P263653	4.2527356	4.243161	NM_004946	chr5:169188615-169230106	DOCK2
A_23_P257164	4.6189737	4.519987	NM_000481	chr3:49454494-49454435	AMT
A_23_P19754	4.2327843	4.1474714	NM_016352	chr7:129962993-129963052	CPA4
A_33_P3308434	4.794443	4.7581735	NM_024848	chr1:2317328-2317269	MORN1
A_24_P203418	3.847057	3.495605	AF174045	chr14:107179067-107179008	
A_23_P66487	5.700608	5.733868	NM_001098426	chr17:61910173-61910114	SMARCD2
A_23_P59547	8.681746	8.582634	NM_001002009	chr7:33054255-33054196	NT5C3A
A_33_P3258346	3.6350033	3.278096	NM_017523	chr17:6674143-6674202	XAF1
A_23_P29067	4.5194564	4.9388456	NM_005656	chr21:42836963-42836904	TMPRSS2
A_24_P84822	7.337787	7.321117	NR_027028	chr5:21491451-21491510	GUSBP1
A_32_P226567	8.900111	9.102738	NM_023011	chr13:115070517-115070576	UPF3A
A_33_P3253799	6.9298573	7.131194	NM_001040451	chr5:179034600-179034659	RUFY1
A_24_P396197	5.4782405	5.5146995	NM_002743	chr19:11559073-11559380	PRKCSH
A_23_P47991	8.455558	8.482736	NM_015335	chr12:116396747-116396688	MED13L
A_24_P68019	7.1595716	7.1305466	NM_138347	chr19:58200767-58200826	ZNF551
A_33_P3286608	6.937501	7.104003	NM_170725	chr1:249212503-249212562	PGBD2
A_24_P588897	7.377846	7.402326	NM_013272	chr15:92709063-92709122	SLCO3A1
A_23_P48070	6.080374	6.431377	NM_016162	chr12:6760128-6760069	ING4
A_24_P219108	3.7844095	3.850337	NM_031464	chr14:75373785-75373726	RPS6KL1
A_23_P85682	6.3976564	6.53941	NM_005595	chr1:61921914-61921973	NFIA
A_24_P213924	10.169877	10.303805	NM_020947	chr16:84511789-84511730	TLDC1
A_23_P18055	8.778706	9.186398	NR_024615	chr3:55691700-55691641	ERC2-IT1
A_23_P60742	4.5124564	4.7935724	NM_013292	chr16:30387764-30387823	MYLPF
A_23_P78122	4.846614	4.7974305	NM_078471	chr17:27400879-27400820	MYO18A
A_24_P57898	5.1493397	4.8519287	NM_080606	chr20:61637561-61637502	BHLHE23
A_33_P3789894	5.1287575	3.5573633	AW382724	chr2:232320803-232320862	SNORA75
A_23_P257668	5.757236	5.82586	NM_024926	chr7:138874105-138874164	TTC26
A_24_P353300	4.617945	4.566374	NM_001031801	chr22:31669478-31671180	LIMK2
A_24_P229164	3.683025	3.5362625	NM_003959	chr12:123346550-123346609	HIP1R
A_23_P126363	3.6949167	3.0725052	NM_021794	chr1:120436717-120436658	ADAM30
A_33_P3411165	2.807994	2.3900566	NM_153488	chrX:151885589-151885648	MAGEA2B
A_24_P241276	11.042962	10.556721	NM_181503	chr13:37583331-37583390	EXOSC8
A_23_P129014	8.237826	8.320787	NM_145231	chr14:90263611-90263552	EFCAB11
A_33_P3422931	9.932364	9.728859	NM_023936	chr16:1822329-1822270	MRPS34
A_23_P63829	10.350428	10.115826	NM_016299	chr10:14909868-14912614	HSPA14
A_33_P3219651	3.7076478	3.0449076	NM_133468	chr7:34193999-34194058	BMPER
A_33_P3406927	5.0805483	4.2598367	NM_031284	chr15:73045124-73045065	ADPGK
A_33_P3233978	4.1368465	3.6966443	NM_032807	chr10:5958359-5958418	FBXO18
A_24_P801079	7.5713196	7.5156765	AY239294	chr9:136216408-136215841	
A_33_P3339051	11.032902	10.88373	NM_182498	chr19:44111695-44111636	ZNF428
A_33_P3214119	4.352827	4.786576	XM_005257841	chr17:45654466-45654525	NPEPPS
A_24_P8318	6.2164416	6.1505256	NM_014423	chr5:132270181-132270122	AFF4
A_23_P64860	4.721886	5.220024	NM_003006	chr12:109016450-109016391	SELPLG
A_23_P91954	4.1004906	3.9467418	NM_001296	chr3:42906836-42906895	ACKR2
A_24_P176255	6.913615	6.6087503	NM_005254	chr15:50569788-50569729	GABPB1
A_24_P941167	8.298345	7.7224298	NM_030641	chr22:36057118-36057177	APOL6
A_23_P71598	8.385385	8.599048	NM_003829	chr9:13106858-13106799	MPDZ
A_23_P370142	9.303729	9.234647	NM_002572	chr11:117040867-117040926	PAFAH1B2
A_33_P3384462	4.146449	4.037236	NM_024817	chr15:72075663-72075722	THSD4
A_24_P62860	8.55882	8.361498	NM_005843	chr2:152975469-152975410	STAM2
A_33_P3369631	8.774906	8.663836	NM_003758	chr15:44854849-44854908	EIF3J
A_33_P3210860	5.092385	5.3022237	NM_001039886	chr19:53059244-53059303	ZNF808
A_33_P3417141	7.2184057	7.1387525	NM_138442	chr19:18054407-18054466	CCDC124
A_24_P371194	8.194695	8.308253	NM_053050	chr2:74699584-74699525	MRPL53
A_23_P42975	7.074496	7.099357	NM_002736	chr7:106801594-106801653	PRKAR2B
A_33_P3771741	9.880409	10.302844		chr3:95373490-95373431	MTHFD2P1
A_33_P3212754	6.5069947	6.1631536	NM_017561	chr9:97080537-97080478	NUTM2F
A_23_P35230	7.159929	7.539339	NM_172350	chr1:207940437-207940496	CD46

A_33_P3245321	6.856904	7.107397	NM_001012267	chr9:95375371-95375430	CENPP
A_32_P14744	12.898813	13.005178	NM_001019	chr16:18800339-18799442	RPS15A
A_33_P3417176	7.3375807	7.3698006		chr2:242674189-242674130	
A_24_P141707	3.7957575	3.2894647	NM_031479	chr12:57850864-57850923	INHBE
A_33_P3367917	5.6525154	5.8442316	NM_001282130	chr17:28001032-28000973	SSH2
A_33_P3451157	14.145188	14.209134	AK054980	chr8:8024162-8024221	
A_24_P185945	2.3221061	2.3900566	NM_002362	chrX:151092474-151092533	MAGEA4
A_24_P370420	4.9273357	4.7922797	NM_030798	chr7:74470082-74470023	WBSCR16
A_23_P126844	7.9597774	7.575979	NM_148965	chr1:6521304-6521245	TNFRSF25
A_33_P3334773	4.0838785	4.5197463	NM_002478	chr11:17743565-17743624	MYOD1
A_23_P431319	7.8971443	7.82608	NM_173834	chrX:67742695-67742754	YIPF6
A_23_P331928	9.954823	9.695611	NM_133493	chr6:74537828-74537887	CD109
A_23_P15305	9.104091	9.054295	NM_002766	chr17:74307297-74307238	PRPSAP1
A_32_P140139	10.272539	10.3182335	NM_000129	chr6:6144484-6144425	F13A1
A_24_P368575	5.705166	4.986694	NM_003615	chr3:27414644-27414585	SLC4A7
A_33_P3397147	5.4090295	5.8773694	NM_001009993	chr2:131829488-131829429	FAM168B
A_23_P416711	6.5441623	6.662739	NM_152996	chr1:77096543-77096602	ST6GALNAC3
A_33_P3278058	4.728648	5.1381335	NM_138789	chr11:111941220-111941161	PIH1D2
A_23_P32223	8.400198	8.407536	NM_012127	chr9:130929089-130928678	CIZ1
A_33_P3499102	5.9523335	6.213601	AK098115	chrX:47037204-47037263	RBM10
A_33_P3396851	4.074113	4.4348617		chr12:8394190-8394131	XLOC_014512
A_23_P361469	6.8822474	6.5205765	NM_152396	chr3:15457375-15457316	METTL6
A_23_P306933	6.4826727	6.0269537	NM_139283	chr12:110972503-110972444	PPTC7
A_33_P3319640	4.957858	5.0289464	AB208892	chr19:4497037-4497096	HDGFRP2
A_23_P143817	10.040499	10.374713	NM_053025	chr3:123333133-123333074	MYLK
A_33_P3333995	5.605489	4.858256	DB072080	chr16:57807779-57807720	KIFC3
A_33_P3387170	7.7789364	8.023308	NR_034182	chr1:31192359-31194140	MATN1-AS1
A_23_P418431	3.2893255	3.5898833	NR_026784	chr6:88109402-88109459	C6orf164
A_23_P171258	8.397532	8.526847	NM_004299	chrX:74273393-74273334	ABCB7
A_23_P94338	3.7552881	3.9082487	NM_006209	chr8:120581567-120581508	ENPP2
A_33_P3311160	6.760889	7.0972757	AB385295	chr11:47806535-47806476	
A_23_P75839	9.9643135	10.066283	NM_006292	chr11:18502071-18502012	TSG101
A_33_P3294017	9.813002	9.459723	NM_031314	chr14:21677426-21677367	HNRNPC
A_33_P3389133	6.725741	7.193121	NM_001178117	chr10:89268187-89268246	MINPP1
A_23_P202737	6.3718143	6.5975466	NM_003772	chr11:96126198-96126257	JRKL
A_23_P31414	7.831796	7.876213	NM_002553	chr7:103767006-103766947	ORC5
A_23_P2355	10.541378	9.811802	NM_012117	chr12:54639976-54639917	CBX5
A_33_P3280709	4.0485916	4.4706063		chr19:056818615-056818674	
A_23_P321496	4.906703	4.8453155	NR_026916	chr21:14415205-14415264	ANKRD30BP2
A_24_P255654	12.2921915	12.164696		chr12:53547983-53548043	XLOC_014512
A_23_P77415	4.8731413	4.937725	NM_182981	chr16:83999149-83999208	OSGIN1
A_33_P3387831	12.030514	11.92761	NM_024053	chr22:42334819-42334760	CENPM
A_32_P148824	9.136544	9.042736	NM_017847	chr1:186390276-186390335	C1orf27
A_33_P3399765	5.7099257	5.5950646		chr7:156990272-156990213	
A_24_P170095	5.4510813	5.7336807		chr5:179653763-179653704	
A_33_P3218120	7.7605305	8.168689		chr22:019019593-019019534	
A_33_P3333777	6.2599087	5.9704733	NR_024490	chr15:50650157-50650216	GABPB1-AS1
A_33_P3215023	8.415587	8.784712	NM_152734	chr6:36891232-36891291	C6orf89
A_33_P3462960	5.9980536	5.910759	NM_006260	chr13:96443238-96443297	DNAJC3
A_33_P3239512	2.6094058	2.3900566	NM_001102657	chr19:52659186-52659127	ZNF836
A_32_P133884	8.294165	8.287242	NM_001004125	chr9:25677283-25677224	TUSC1
A_23_P80136	4.3055596	3.6613245	NM_003683	chr21:45217868-45217927	RRP1
A_33_P3306024	9.989338	9.727572	NR_002825	chr19:50479016-50479075	SIGLEC16
A_33_P3242304	4.3752465	4.1825676	NM_005425	chr16:11362828-11362769	TNP2
A_23_P389250	5.047587	4.506446	NM_001145010	chr12:27655039-27655098	SMCO2
A_32_P171328	11.848805	11.43738	NM_014501	chr19:55912744-55912685	UBE2S
A_33_P3413394	3.711278	3.3330522	BC110596	chr17:74076920-74076979	ZACN
A_32_P48615	6.6886406	6.4985676		chr15:102277117-102277058	
A_23_P18993	4.970074	5.3501186	NM_152622	chr5:56219152-56219093	MIER3

A_33_P3259705	4.4425077	4.013726	XM_005253044	chr11:406142-406083	SIGIRR
A_23_P5258	3.554862	4.470966	NM_001004465	chr19:16060357-16060416	OR10H4
A_33_P3382769	7.8878345	8.037689	NR_024474	chr15:83084398-83084457	UBE2Q2P3
A_24_P76358	11.826785	12.1429		chr10:097354608-097354669	
A_23_P18518	3.7687075	4.0119896	NM_152402	chr4:118005801-118005742	TRAM1L1
A_33_P3406493	6.534826	6.024997	NM_005458	chr9:101050460-101050401	GABBR2
A_23_P41390	6.003866	5.9010553	NM_018986	chr4:8239319-8239379	SH3TC1
A_33_P3224946	4.015268	4.061023	AK127903	chr5:65867742-65867801	FLJ46010
A_33_P3211213	12.115099	12.130803	NM_001282332	chr19:54697461-54697520	TSEN34
A_32_P161166	6.679094	6.719713	AY927536	chrX:153628916-153628857	
A_24_P231302	3.7021108	3.1000361	NM_020393	chr1:153312903-153309763	PGLYRP4
A_24_P943193	9.758781	8.4423485	NM_006036	chr2:44545965-44545906	PREPL
A_23_P50508	4.502428	4.192818	NM_003706	chr19:48551307-48551248	PLA2G4C
A_23_P7976	7.9862137	7.5338793	NM_005321	chr6:26156928-26156987	HIST1H1E
A_23_P131801	9.09207	9.25904	NM_170693	chr20:42213703-42213762	SGK2
A_33_P3208966	4.7538123	4.813389	AY513606	chr19:41601683-41601741	CYP2A13
A_33_P3340294	3.59469	3.9515328	NM_053043	chr7:155567916-155567975	RBM33
A_23_P171366	15.3302355	15.587135	NM_004651	chrX:47107052-47107204	USP11
A_24_P351420	8.72921	8.21424	NM_016598	chr3:44966911-44966852	ZDHHC3
A_33_P3380582	4.021466	4.07677		chr1:202795380-202795439	
A_24_P340976	13.085549	13.186493		chr3:196714035-196713975	
A_24_P285522	6.6728	7.1058836	NM_003618	chr2:39477451-39477392	MAP4K3
A_24_P252794	8.700627	8.9814005	NM_003591	chr10:35299334-35299275	CUL2
A_23_P40453	7.9450827	7.440307	NM_001236	chr21:37518379-37518438	CBR3
A_33_P3351499	7.4281096	7.1743636		chr17:076842103-076842044	
A_23_P433188	4.902321	4.964714	NM_022482	chr20:23353548-23353607	GZF1
A_33_P3365072	4.8451843	5.0369325	NM_001128600	chr1:152816399-152816458	LCE6A
A_33_P3248664	11.2830305	11.035692	NM_006295	chr6:31745412-31745353	VARS
A_23_P214798	10.581555	10.474838	NM_006372	chr6:86324192-86324133	SYNCRIP
A_23_P160460	9.369582	9.6098795	NM_003115	chr1:162560162-162560221	UAP1
A_33_P3294946	4.266578	3.9896045	AJ630586	chr19:55309679-55309738	1060P11.3
A_24_P401870	4.1272383	4.3792	NM_207511	chr9:139930882-139930941	C9orf139
A_23_P135990	3.186857	3.6332345	NM_005630	chr3:133651840-133651781	SLCO2A1
A_23_P348063	5.6708155	5.8147087	NM_004711	chr22:39780995-39781054	SYNGR1
A_23_P7342	9.065286	9.267944	NM_001075	chr4:69696473-69696532	UGT2B10
A_33_P3275255	6.619018	6.559472	DQ786257	chr1:143918448-143918507	
A_23_P204079	7.468962	7.567213	NM_003717	chr12:53900549-53900490	NPFF
A_23_P315815	7.346287	7.772952	NM_004495	chr8:32474390-32585512	NRG1
A_23_P211252	2.378499	3.9747875	NM_001001438	chr21:47608626-47608567	LSS
A_33_P3229027	8.50061	8.328269	NM_173828	chr5:141020571-141020630	RELL2
A_33_P3304527	4.676006	4.4822025	NM_021035	chr20:47892409-47892350	ZNFX1
A_23_P24751	8.90369	9.1242285	NM_173810	chr11:62505795-62505854	TTC9C
A_33_P3210986	5.955389	6.194353	NM_001145441	chr7:72081717-72081658	TYW1B
A_23_P86133	9.290409	9.404233	NM_002946	chr1:28218364-28218305	RPA2
A_24_P364954	6.8937654	6.785607	NM_024648	chr17:80364314-80363218	OGFOD3
A_23_P79155	5.9056797	5.9056797	NM_001508	chr2:133403578-133403637	GPR39
A_32_P181222	6.511041	6.5330806	NM_002247	chr10:78846275-78844448	KCNMA1
A_23_P371410	5.1755705	5.3203697	NM_207578	chr1:84670691-84670750	PRKACB
A_33_P3226135	5.791157	5.7426653		chr7:075103907-075103848	
A_23_P123193	7.359319	7.061103	NM_020445	chr7:152552373-152552432	ACTR3B
A_23_P20832	8.774071	8.937849	NM_003127	chr9:131395722-131395781	SPTAN1
A_33_P3298406	6.4658656	6.470277	NM_001010895	chr9:98691085-98691144	ERCC6L2
A_33_P3297923	10.698758	10.6778555	NM_001128228	chr9:140086128-140086069	TPRN
A_23_P210176	8.629935	8.614557	NM_000210	chr2:173370556-173370615	ITGA6
A_33_P3300757	6.991843	7.051631	NM_001199942	chr6:83877869-83877928	DOPEY1
A_23_P124417	9.804688	10.067884	NM_004336	chr2:111397340-111395699	BUB1
A_24_P190877	7.527312	7.0691614	NM_030934	chr1:185087449-185087390	TRMT1L
A_23_P148785	7.4239516	7.76454	NM_199344	chr1:168205959-168208378	SFT2D2
A_23_P325676	5.332139	5.1945496	NM_138783	chr19:11597597-11596540	ZNF653

A_33_P3237235	5.035814	4.6526093	NM_001014765	chr1:11721347-11721406	FBXO44
A_33_P3244317	7.416742	6.589916	NM_015154	chr15:81268205-81268146	MESDC2
A_23_P204967	7.920942	7.9762077	NM_017520	chr13:20246111-20246170	MPHOSPH8
A_33_P3376234	8.174004	7.8923926	NM_006608	chr1:114239909-114239850	PHTF1
A_23_P259166	10.519711	10.630793	NM_024863	chrX:102842228-102842287	TCEAL4
A_23_P76951	11.773636	11.830832	NM_030755	chr14:51722559-51722618	TMX1
A_23_P139654	3.6361372	3.5153172	NM_007328	chr12:10603152-10603093	KLRC1
A_33_P3261595	2.3221061	2.3900566	NM_001004441	chr5:79852689-79852630	ANKRD34B
A_33_P3254191	6.868059	7.1008434	NM_020841	chr12:76748399-76748340	OSBPL8
A_33_P3234667	6.981188	7.194565	NM_003439	chr7:99635306-99635365	ZKSCAN1
A_23_P1206	14.192438	14.203674	NM_001026	chr10:79795371-79795430	RPS24
A_33_P3382331	5.0028057	4.5481334	NM_002155	chr1:161495332-161495391	HSPA6
A_23_P70168	12.352022	11.440688	NM_152295	chr5:33467942-33468001	TARS
A_33_P3333863	6.8116446	6.7138057	NM_015622	chr7:5942315-5942374	CCZ1
A_24_P313262	7.498988	7.395819	NM_006628	chr15:52840062-52840003	ARPP19
A_33_P3221119	7.7332253	7.777026	NM_001282861	chr1:155734733-155734674	GON4L
A_33_P3321230	4.293872	4.4223013	NM_052831	chr6:133095440-133095381	SLC18B1
A_24_P214858	6.555295	6.43248	NM_024807	chr6:41158552-41158493	TREML2
A_33_P3277447	9.980834	8.766542	NM_000112	chr5:149366825-149366884	SLC26A2
A_33_P3279820	6.9848776	6.7205386		chr10:022555928-022555987	
A_23_P42116	8.180624	8.346597	NM_005155	chr6:32131273-32131332	PPT2
A_33_P3298173	3.9263957	4.130846	AY746432	chr8:6896171-6896112	DEFA7P
A_33_P3335147	5.802402	5.433122	NM_001018116	chr9:103349793-103349852	MURC
A_24_P209113	6.504142	6.6116123	NM_030571	chr5:141511454-141511829	NDFIP1
A_33_P3299634	7.7006207	7.999334	AB529307	chr9:125512044-125512103	OR1L6
A_23_P23443	11.630226	11.3644285	NM_024329	chr1:15756637-15756696	EFHD2
A_32_P133840	4.346694	4.18544	NM_014858	chr1:205242117-205242176	TMCC2
A_33_P3411204	6.8230505	7.1777368	NM_001491	chr6:10627251-10627310	GCNT2
A_33_P3241299	3.781201	3.495183	NR_036480	chr16:89779129-89779188	VPS9D1-AS1
A_24_P928510	5.3666434	4.8451843	NM_053055	chr1:151846533-151846474	THEM4
A_23_P65963	8.125771	8.233768	NM_016561	chr16:14762285-14762344	BFAR
A_32_P129540	8.635457	8.253407	NR_037629	chr12:108297117-108297058	LOC728739
A_23_P145541	4.595462	4.763501	NM_014845	chr6:110110799-110110858	FIG4
A_33_P3290040	2.681716	3.3965487	NM_001199752	chr19:50216867-50216926	CPT1C
A_33_P3307187	3.6053534	3.3503103	NM_001164749	chr14:33525140-33525199	NPAS3
A_24_P361427	4.5727177	4.5173216	NM_015594	chr17:28890360-28890419	TBC1D29
A_23_P16139	7.324353	7.214982	NM_006387	chr19:16629439-16629380	CHERP
A_33_P3280531	9.966324	10.133285	NM_001257363	chr9:131857134-131857075	CRAT
A_33_P3378216	5.727585	5.741748		chr5:178999683-178999742	
A_23_P204980	7.5602274	6.6824703	NM_020121	chr13:96453984-96453925	UGGT2
A_33_P3273969	9.470915	9.671408	NM_014827	chr1:203821489-203821548	ZC3H11A
A_23_P215675	8.377263	8.279856	NM_018224	chr7:43684877-43680217	COA1
A_33_P3228739	5.347834	5.0261564	NM_001195545	chr17:38100869-38100928	LRRC3C
A_23_P253841	12.456548	12.424421	NM_005526	chr8:145538318-145538377	HSF1
A_24_P92558	4.9392934	4.6179676	NM_001039891	chrX:46359498-46359439	ZNF674
A_33_P3250148	5.9612064	6.0747595	NM_198956	chr7:20823968-20823909	SP8
A_24_P289726	8.296419	8.172207	NM_002809	chr17:38152574-38153624	PSMD3
A_33_P3402304	5.126113	5.472887	NM_001039360	chr18:45554041-45553982	ZBTB7C
A_32_P473302	5.9468946	5.7069354	NR_015375	chr9:2535721-2535662	FLJ35024
A_24_P100830	5.6760745	5.550372	NM_001278412	chr12:31824730-31824671	AMN1
A_33_P3395369	9.665298	9.62225	NM_001634	chr6:111213970-111214029	AMD1
A_24_P226970	5.096506	3.7590268	NM_014951	chr10:64161908-64161967	ZNF365
A_24_P123408	2.620356	2.3900566	NM_014945	chr5:148626089-148627383	ABLIM3
A_33_P3287661	3.1748857	3.8796766	AK126997	chr16:1358800-1358741	LOC100130430
A_33_P3399438	5.570007	5.7110043	NR_034087	chr9:114365175-114365116	LRRC37A5P
A_33_P3343473	5.1839323	5.1697025	NM_207303	chr10:117308976-117309035	ATRNL1
A_23_P373464	4.0085664	4.0408125	NM_002285	chr2:100163726-100163700	AFF3
A_33_P3368039	6.689749	6.9791036	NM_001286520	chr6:44222502-44222443	SLC35B2
A_23_P80353	4.6794477	4.7307496	NM_031488	chr22:41626187-41626246	L3MBTL2

A_23_P99785	5.0406623	5.046128	NM_024643	chr14:75544429-75544488	ZC2HC1C
A_33_P3209176	13.036818	13.088327		chr7:075135559-075135500	
A_33_P3367692	6.5888467	7.318496	NM_001014975	chr1:196646719-196646778	CFH
A_24_P143138	5.3750353	5.261174	NM_004463	chrX:54473805-54473746	FGD1
A_33_P3399172	5.981595	6.1461954		chr22:21024895-21024836	XLOC_014512
A_33_P3415828	4.2136874	3.8769605	CU677870	chr4:110639886-110650787	
A_32_P69166	5.0541315	4.889369	NM_182603	chr11:82959039-82959098	ANKRD42
A_23_P217151	7.971656	7.700168	NM_017776	chrX:46333979-46334036	KRBOX4
A_33_P3353948	6.1684904	6.071078		chr7:048886274-048886333	
A_23_P66777	9.159546	9.027777	NM_001256	chr17:45197878-45197819	CDC27
A_32_P200238	8.709604	7.128085	NR_015379	chr19:15946035-15946092	UCA1
A_23_P398294	9.250853	9.314166	NM_003959	chr12:123347447-123347506	HIP1R
A_23_P137948	10.384741	10.670865	NM_013349	chr1:212619293-212619352	NENF
A_33_P3323822	6.202173	6.0026603	NM_020699	chr1:153777333-153777274	GATAD2B
A_24_P244100	9.631545	9.51847	NM_001002762	chr10:74092885-74092826	DNAJB12
A_23_P259393	7.367791	7.5509834	NM_016329	chr3:52938830-52938771	SFMBT1
A_32_P384562	4.9125524	5.294986	NR_023386	chr1:16794256-16794197	CROCCP3
A_24_P245379	2.3221061	2.3900566	NM_002575	chr18:61570202-61570261	SERPINB2
A_23_P164141	9.571548	9.483101	NM_176863	chr17:40994997-40995056	PSME3
A_24_P179816	5.622055	5.7500825	NM_024330	chr1:153751864-153751923	SLC27A3
A_33_P3851788	5.804196	5.189084	NR_027271	chr19:1267531-1267472	CIRBP-AS1
A_23_P14458	8.188621	7.8657947	NM_144581	chr14:59942918-59942859	L3HYPDH
A_23_P356021	6.936648	6.810077	NM_001018113	chrX:14862096-14862037	FANCB
A_23_P16415	8.359314	8.5276375	NM_002333	chr19:33698336-33698395	LRP3
A_23_P129704	7.745549	8.032574	NM_018975	chr16:75691037-75691096	TERF2IP
A_33_P3421827	5.8865786	5.935804	NM_005332	chr16:204445-204504	HBZ
A_33_P3216433	7.902895	8.22673	NR_003148	chr19:53947715-53947774	TPM3P9
A_23_P348383	7.2305937	6.878755	NM_001080522	chr4:15591276-15597740	CC2D2A
A_24_P305938	6.7114954	6.9051113	NR_000039	chr5:104435265-104435325	RAB9BP1
A_23_P154874	6.8472085	6.60544	NM_006052	chr21:38597763-38597704	DSCR3
A_23_P67339	4.5732055	4.753353	NM_020650	chr19:50045997-50046409	RCN3
A_24_P270144	12.364962	12.635663	NM_001257389	chr12:56119950-56119667	CD63
A_23_P30495	8.65069	8.123516	NM_000859	chr5:74657651-74657710	HMGCR
A_23_P98580	9.151032	9.21717	NM_004265	chr11:61634754-61634813	FADS2
A_23_P60354	8.70432	8.935379	NM_139045	chr9:2192841-2192900	SMARCA2
A_24_P305623	7.202446	7.9829254	NM_006134	chr21:34821954-34821895	TMEM50B
A_23_P74309	4.9196157	4.6981144	NM_014697	chr1:162337975-162338034	NOS1AP
A_33_P3300740	3.756192	4.1856174	BI056255	chr1:32825379-32825320	
A_23_P425104	5.5025764	5.2742515	NM_058243	chr19:15348638-15348579	BRD4
A_23_P65278	6.1230345	6.067991	NM_015678	chr13:36246683-36246742	NBEA
A_23_P161324	5.441803	5.7865705	NM_015901	chr10:74890891-74890950	NUDT13
A_23_P212284	8.753551	8.833872	NM_015426	chr3:52109513-52109454	POC1A
A_32_P116323	7.174615	7.1289716	NM_015092	chr16:18870483-18870424	SMG1
A_33_P3316475	4.906801	4.9983277	NM_207469	chr20:241668-241727	DEFB132
A_23_P15402	10.321202	10.312432	NM_133491	chr17:7529665-7529606	SAT2
A_23_P157726	8.113573	8.540735	NM_017925	chr9:19372372-19372431	DENND4C
A_33_P3274756	9.6847515	10.848771	NM_015018	chr6:83866975-83867034	DOPEY1
A_23_P32615	9.5595045	9.176886	NM_002431	chr14:61435296-61435355	MNAT1
A_23_P214603	8.452592	8.556038	NM_005803	chr6:30697891-30697832	FLOT1
A_23_P145965	8.5352335	8.735189	NM_003596	chr7:65825164-65825223	TPST1
A_23_P147397	6.955502	7.13004	NM_001080463	chr11:103349837-103349896	DYNC2H1
A_24_P187651	5.4792843	5.6843305	NM_001042432	chr16:28498844-28498785	CLN3
A_24_P14464	2.6925063	2.3900566	NM_006103	chr20:44099078-44099137	WFDC2
A_33_P3373185	13.929015	14.247091	NR_024497	chr10:38737840-38737899	LINC00999
A_33_P3521643	11.20318	11.245769	NM_001271618	chr19:55602347-55602288	PPP1R12C
A_23_P354547	5.8913307	6.294408	AK074144	chr14:24636167-24636226	IRF9
A_33_P3236642	5.053573	4.8530793	NM_198551	chr1:222801854-222801913	MIA3
A_32_P150030	7.6700344	7.8079786	NM_003620	chr17:58741635-58741694	PPM1D
A_33_P3363173	4.4949665	4.3654523		chr3:050406435-050406494	

A_23_P208900	6.8748817	6.7291546	NM_032108	chr19:4542955-4542896	SEMA6B
A_33_P3420762	6.432577	6.360353		chr1:008934916-008934975	
A_23_P379550	5.7631006	5.0435247	NM_003680	chr1:33245858-33245799	YARS
A_23_P97423	7.842635	7.266201	NM_017582	chr1:154521395-154521336	UBE2Q1
A_33_P3251522	2.3221061	2.3900566	NM_173800	chr5:115363220-115363279	AQPEP
A_33_P3220994	7.23934	7.204469		chr6:026869979-026870038	
A_33_P3309064	9.258713	8.726617	NM_001184977	chr20:33981977-33981918	UQCC1
A_23_P94911	6.6861315	6.451957	NM_181845	chr19:44352724-44352783	ZNF283
A_33_P3296940	7.481653	7.3690815	NM_022763	chr3:171851276-171851335	FNDC3B
A_23_P94118	8.454165	8.366688	NM_002095	chr8:30464597-30437878	GTF2E2
A_23_P253350	2.3221061	2.3900566	NM_020130	chr8:40011967-40012026	C8orf4
A_23_P400465	12.626408	12.565779	NM_138408	chr6:111288903-111288962	GTF3C6
A_23_P77459	10.811754	10.736747	NM_001018159	chr16:66836909-66836850	NAE1
A_32_P203013	13.692521	13.841615	NR_026667	chr1:201489664-201489723	RPS10P7
A_33_P3318564	5.3707533	4.9605675		chr6:105602308-105606893	BVES-AS1
A_23_P422724	9.938884	10.552189	NM_000943	chr5:122359427-122359368	PPIC
A_23_P124224	8.527874	8.693029	NM_004941	chr17:41601440-41601499	DHX8
A_24_P813550	4.8135147	4.6075706	XM_001716834	chr14:107083360-107083301	
A_33_P3260430	2.3221061	3.22565	NM_005988	chr1:153028658-153028599	SPRR2A
A_24_P100996	2.3221061	2.3900566	NM_213599	chr11:22304318-22304377	ANO5
A_23_P63096	4.240854	4.820693	NM_001004319	chr1:156268672-156268613	VHLL
A_23_P122116	7.178857	7.433795	NM_016222	chr5:176939374-176939200	DDX41
A_24_P920521	4.4158564	5.0144563	NM_203497	chr13:76100318-76100259	COMMD6
A_23_P257905	5.524776	5.8303337	NM_001810	chr20:3765453-3765394	CENPB
A_33_P3389653	3.5112357	2.3900566	NM_001165899	chr5:58264986-58264927	PDE4D
A_24_P66027	7.7949553	8.246935	NM_004900	chr22:39387538-39387597	APOBEC3B
A_23_P47885	7.651646	7.3053923	NM_153377	chr12:59266361-59266302	LRIG3
A_33_P3299386	3.592218	3.2463858	NM_001277092	chr19:58058402-58058343	ZNF550
A_33_P3350566	4.7040234	4.9965653	NM_021096	chr22:40078606-40078665	CACNA1I
A_33_P3355247	5.9478407	6.068247	NM_015621	chr5:150562671-150562612	CCDC69
A_33_P3273624	5.4558	5.4583526	NM_003549	chr3:50330627-50330568	HYAL3
A_24_P103025	12.440556	12.431994	NM_006196	chr2:70315678-70315737	PCBP1
A_33_P3310226	4.482673	4.6710167	AK095213	chr17:43205580-43205521	LOC100129115
A_23_P1819	4.7419868	4.9523377	NM_012378	chr11:124310815-124310756	OR8B8
A_33_P3362861	4.9375987	5.334029	NM_014898	chr19:38123448-38123389	ZFP30
A_23_P385063	7.0347166	7.2755003	NM_058246	chr7:157209570-157209629	DNAJB6
A_23_P57856	7.85335	8.364776	NM_001130845	chr3:187439700-187439641	BCL6
A_33_P3402217	8.476041	8.564591		chr19:038184322-038184263	
A_33_P3378412	4.164139	4.537943		chrX:089294097-089294038	
A_24_P302038	3.9408236	4.082573	NM_014824	chr11:72560800-72554519	FCHSD2
A_33_P3347465	6.478094	6.2649584	NM_144604	chr16:88697913-88697972	ZC3H18
A_23_P61881	10.590803	10.6922455	NM_006321	chr3:49020812-49020871	ARIH2
A_23_P210690	9.790602	9.1012535	NM_021158	chr20:378107-378166	TRIB3
A_23_P159227	5.3775578	5.611101	NM_207191	chr1:155030539-155030598	ADAM15
A_33_P3313110	3.5997684	2.9765527	NM_024690	chr19:9060511-9060452	MUC16
A_33_P3409580	8.763874	8.637417	NR_038389	chr9:140144746-140144687	LOC100129722
A_23_P5976	2.495948	2.3900566	NM_080603	chr20:44512886-44512945	ZSWIM1
A_24_P148717	2.3221061	2.3900566	NM_001295	chr3:46243888-46243829	CCR1
A_23_P83923	8.283859	8.423495	NM_024836	chr1:249143527-249143586	ZNF672
A_33_P3322150	3.6650405	3.348774	BU567630	chr7:17325979-17325920	
A_33_P3263666	10.759525	11.119062	NM_152326	chr14:102973257-102973198	ANKRD9
A_33_P3248580	8.899315	8.922867		chr5:112113457-112113398	
A_23_P342744	6.345915	6.5633907	NM_153713	chr1:145497418-145497477	LIX1L
A_33_P3228573	9.2177305	8.992858	NM_001251888	chr17:79975223-79975282	ASPSCR1
A_23_P105436	4.797236	4.9571886	NM_002834	chr12:112915513-112915698	PTPN11
A_33_P3353125	8.100987	8.199198	BC018676	chr5:43586668-43586609	
A_33_P3299525	7.0509534	7.290197	NM_001029859	chr11:77882362-77882303	KCTD21
A_24_P59607	7.989664	7.084827	NM_001113434	chr17:21436035-21435976	C17orf51
A_33_P3274851	11.178787	11.268042	NM_001862	chr2:98264535-98264594	COX5B

A_24_P67308	11.488429	11.569299	NR_026660	chr7:102781746-102781717	RPL19P12
A_33_P3210900	3.8348007	3.5343814		chr3:10356930-10356871	SEC13
A_33_P3866631	9.167079	8.91022	AL049980	chr11:43881253-43881312	DKFZP564C152
A_23_P25204	13.625151	13.604	NM_213611	chr12:98995317-98995376	SLC25A3
A_23_P167997	9.027777	8.938645	NM_003518	chr6:26216776-26216717	HIST1H2BG
A_33_P3284247	4.3425	4.5241547		chrY:14475225-14475166	GYG2P1
A_24_P156922	5.5264416	5.8915515	NM_001007098	chr1:53446121-53446180	SCP2
A_23_P50455	10.211779	9.948938	NM_002691	chr19:50921206-50921265	POLD1
A_33_P3415340	5.0386653	4.7057543	AK090992	chr2:202644787-202644728	ALS2
A_24_P115967	7.05105	6.621154	NM_006133	chr11:61514384-61514443	DAGLA
A_33_P3265159	4.0397043	3.7292404	BC110990	chr9:128071501-128071560	GAPVD1
A_33_P3410235	4.803994	4.8036456	NM_001276267	chr15:45409747-45409688	DUOXA1
A_33_P3230493	4.030175	3.0714278		chr11:124614601-124614542	
A_33_P3343260	7.58308	7.7735643	XM_005255732	chr16:3103191-3103132	LOC101929668
A_33_P3322594	3.9294965	4.3245964		chrX:16189519-16189578	MAGEB17
A_23_P501451	6.948765	7.029006	NM_001014999	chr16:29468995-29469054	SLX1A
A_33_P3266998	4.5809484	4.5635557	NR_026810	chr17:16693224-16693282	FAM106CP
A_23_P145529	2.3221061	2.3900566	NM_181795	chr6:123046337-123046396	PKIB
A_23_P345081	7.573908	7.4970016	NM_001083956	chr7:99173733-99173792	ZNF655
A_32_P211045	9.740524	8.333178	NM_000791	chr5:79922127-79922068	DHFR
A_33_P3324333	10.613995	10.555244	BC022023	chr18:14185309-14185368	
A_23_P205549	8.79845	8.790585	NM_145113	chr14:65542062-65542003	MAX
A_23_P81048	4.6299295	4.7228813	NM_020860	chr4:27024693-27024752	STIM2
A_33_P3236646	9.326489	9.440915	NM_198551	chr1:222839087-222839146	MIA3
A_24_P131066	8.799836	8.10319	NM_004258	chr1:117568132-117568191	CD101
A_23_P3856	7.2093344	7.0817204	NM_153688	chr16:75205715-75205774	ZFP1
A_23_P96590	5.18042	5.3444	NM_014710	chrX:101913205-101913264	GPRASP1
A_33_P3407230	11.802977	12.049162		chr19:011634611-011634670	
A_24_P29595	5.247188	5.727153	NM_139244	chr6:147704083-147705857	STXBP5
A_33_P3247629	5.5918474	5.641778	NM_015509	chr12:8248816-8248875	NECAP1
A_33_P3225477	4.890281	4.639652	BU659163	chr7:151827889-151827833	LOC731075
A_33_P3678883	15.572144	15.617697	AB051441	chr22:43513904-43513845	KIAA1654
A_23_P134714	10.8495	10.263792	NM_005836	chr8:99114874-99114815	HRSP12
A_33_P3348159	8.652798	8.789841	NM_001145354	chr7:131172662-131172721	MKLN1
A_23_P136916	6.9330854	6.954858	NM_017883	chrX:48460541-48462665	WDR13
A_33_P3374253	4.5655484	4.700128		chr18:077319284-077319225	
A_33_P3378697	5.9861684	6.161351	XR_171111	chr7_gl000195_random:82224-82283	LOC101060081
A_23_P49646	8.071335	8.322093	NM_002767	chr17:18833954-18834013	PRPSAP2
A_33_P3378430	9.053835	8.675007	NM_138400	chr7:156765751-156765810	NOM1
A_23_P313223	6.2244177	6.336094	NM_138471	chr11:63594950-63595009	C11orf84
A_33_P3348614	7.3601737	7.7969275	NM_024665	chr3:176744222-176744163	TBL1XR1
A_23_P162171	9.809087	9.169143	NM_006500	chr11:119180061-119180002	MCAM
A_24_P410582	6.8664107	7.1872997	NM_014667	chr3:11643391-11643332	VGLL4
A_24_P40795	12.356836	12.444774		chr5:043667061-043667002	
A_24_P329939	5.200063	5.1871824	NM_021729	chr11:118949879-118949938	VPS11
A_32_P209989	7.4376683	7.177975	NM_022163	chr15:89010441-89010382	MRPL46
A_23_P422778	4.81012	5.09053	NM_004518	chr20:62038213-62038154	KCNQ2
A_33_P3239579	3.875741	3.312746	NR_034014	chr1:59364904-59364963	LOC100131060
A_33_P3273480	4.7687554	5.055012	NM_001256627	chr11:1482769-1482828	BRSK2
A_23_P406591	2.3221061	2.3900566	NR_003675	chr4:144482143-144482202	GUSBP5
A_33_P3231950	6.405402	6.3596344		chr4:037873666-037873725	
A_33_P3353873	3.9430315	3.5554516		chr19:002524710-002524651	
A_24_P118489	8.170699	8.249856	NM_001080435	chr15:83486765-83486824	WHAMM
A_23_P84359	3.7372224	3.5849314	NM_021015	chrX:48049639-48049580	SSX5
A_23_P306346	4.9025173	4.70614	NM_152420	chr9:77598676-77598617	C9orf41
A_23_P60240	6.8607974	7.1083436	NM_032634	chr9:35089055-35088996	PIGO
A_33_P3370692	3.6942341	3.4191144		chr13:033528344-033528285	
A_23_P301304	9.82879	10.667409	NM_023110	chr8:38270529-38270470	FGFR1

A_23_P75800	5.7633533	5.53497	NM_013401	chr11:61665038-61664979	RAB3IL1
A_33_P3398548	5.1858664	4.2794447	NM_003666	chr1:169365416-169365475	BLZF1
A_23_P139143	7.531129	8.221958	NM_004177	chr11:59569425-59569484	STX3
A_33_P3314466	4.896267	4.668627		chr2:86400803-86400744	IMMT
A_23_P77779	15.963493	15.93552	NM_000981	chr17:37360880-37360939	RPL19
A_23_P210719	7.350527	7.3271914	NM_016143	chr20:1423172-1423113	NSFL1C
A_23_P142447	5.179369	5.2536445	NM_012335	chr19:8586183-8586124	MYO1F
A_24_P38572	5.5948524	5.49629	NM_022917	chr9:33464935-33464159	NOL6
A_23_P94902	2.5806837	2.3900566	NM_198353	chr4:44176402-44176343	KCTD8
A_32_P186138	7.7074966	7.877159	NM_001195131	chr6:99979626-99979685	TSTD3
A_23_P59138	8.073342	8.2064495	NM_002701	chr6:31132237-31132178	POU5F1
A_23_P89123	8.74205	9.03846	NM_006324	chr16:75429079-75429020	CFDP1
A_33_P3231981	12.222317	12.287416	NM_007262	chr1:8045101-8045160	PARK7
A_32_P186226	5.4522715	5.529925	NM_006391	chr11:9459671-9459730	IPO7
A_23_P212655	3.9915545	3.780123	NM_130446	chr3:183205899-183205840	KLHL6
A_24_P255874	5.323536	5.6412067	NR_001317	chr6:29894617-29894558	HCG4B
A_23_P132388	9.852966	9.835789	NM_005138	chr22:50962237-50962178	SCO2
A_23_P139297	8.316685	8.334507	NM_017583	chr11:35829049-35829108	TRIM44
A_33_P3227472	6.093233	6.0767198	NM_138432	chr12:113876021-113876080	SDSL
A_32_P118250	6.5473714	6.5794363	NR_023921	chr14:24408397-24408338	DHRS4-AS1
A_23_P39364	11.137152	10.689089	NM_004838	chr19:19040086-19040027	HOMER3
A_23_P359897	4.1767635	4.300596	NM_030647	chr7:139785105-139785046	KDM7A
A_23_P134744	7.1725574	7.5252542	NM_024787	chr8:33405363-33405304	RNF122
A_32_P54305	11.027037	10.865489	NR_024412	chr7:112757123-112757064	LINC00998
A_33_P3299656	4.605113	4.492619	NM_000328	chrX:38147267-38147208	RPGR
A_24_P221575	5.665078	5.387212	NM_001037442	chr4:71648827-71648887	RUFY3
A_23_P99226	4.172518	3.8188682	NM_012240	chr12:120750879-120750938	SIRT4
A_33_P3313075	5.231343	5.6075444		chr7:045960805-045960864	
A_23_P200126	7.4065447	7.098796	NM_003684	chr1:47023654-47023595	MKNK1
A_24_P152325	8.697246	8.771538		chr7:005600401-005600460	
A_24_P191833	9.208988	9.221853	NM_139168	chr5:65475935-65475994	SREK1
A_24_P108242	7.3018847	7.004964	NM_004287	chr17:45018637-45018696	GOSR2
A_23_P422350	4.0580378	3.8560767	NM_000260	chr11:76893556-76893615	MYO7A
A_24_P286013	11.383992	11.468497	NM_005339	chr4:39779985-39780044	UBE2K
A_32_P206949	5.7496853	5.273039	NM_198276	chr2:62727974-62727915	TMEM17
A_23_P62188	8.675812	8.634847	NM_018684	chrX:64136455-64136396	ZC4H2
A_24_P42014	15.239232	15.1805105		chr6:010119347-010119406	
A_23_P157715	10.866096	11.23222	NM_032902	chr8:145727444-145727503	PPP1R16A
A_33_P3331882	9.269756	9.325253	NM_001098482	chr19:18888231-18888290	CRTC1
A_33_P3415221	12.988044	13.12387		chr3:196263583-196263642	
A_33_P3284662	6.9767504	6.7910037	NM_153232	chr19:40030133-40030074	EID2
A_23_P206960	8.947707	9.232864	NM_003003	chr17:75210225-75210284	SEC14L1
A_24_P637982	10.99204	10.999892	NM_198446	chr1:38275051-38275110	C1orf122
A_24_P289471	8.369179	8.903873	NM_003730	chr6:167365982-167362061	RNASET2
A_33_P3303557	3.7153218	3.2278407		chr21:030968988-030968929	
A_23_P44836	7.7848506	7.8730693	NM_022908	chr3:52561355-52559298	NT5DC2
A_23_P360542	3.50148	2.9651647	NR_023925	chr18:1269322-1269263	LINC00470
A_33_P3216718	6.567596	6.625289		chr1:213602110-213602051	
A_23_P253177	10.934173	11.0568905	NM_001032296	chr13:99104998-99104939	STK24
A_23_P156687	3.00178	2.3900566	NM_001710	chr6:31918992-31919147	CFB
A_33_P3219870	7.392498	7.3507013	NM_020376	chr11:824764-824823	PNPLA2
A_33_P3349597	8.808923	8.851178			
A_24_P602507	4.470425	4.7044983	NM_001013619	chr15:78819820-78819879	HYKK
A_33_P3209646	5.3122234	5.191903	NM_052950	chr13:52336092-52336151	WDFY2
A_33_P3262833	7.147695	7.061887	NR_033350	chr15:23447789-23447848	GOLGA8EP
A_33_P3385782	4.229378	3.8659945	NM_182633	chr7:56007679-56007738	ZNF713
A_24_P401990	7.3404465	7.645339	NM_000400	chr19:45864874-45864815	ERCC2
A_24_P323072	4.981118	4.967329	NM_178332	chr20:3025110-3025169	GNRH2
A_23_P302568	4.8153305	5.0889053	NM_003459	chr2:27477607-27477548	SLC30A3

A_32_P181103	7.58062	7.6801605	NM_000821	chr2:85776450-85776391	GGCX
A_33_P3268695	3.6846313	4.458112	NM_032772	chr10:77158823-77158764	ZNF503
A_24_P57730	8.579671	8.021553	NM_181304	chr14:23304096-23304155	MRPL52
A_24_P414712	6.23246	6.7613773	NM_015695	chr6:36199930-36199989	BRPF3
A_23_P133365	8.699926	8.57275	NM_006706	chr5:145890206-145890265	TCERG1
A_24_P343095	9.293133	9.2661295	NM_000791	chr5:79924927-79924868	DHFR
A_23_P66180	4.8485985	5.5930967	NM_006539	chr16:24373365-24373424	CACNG3
A_33_P3264379	4.5060787	4.4067874	NR_027049	chr19:12224463-12224522	ZNF788
A_24_P2361	3.598548	3.732829	NM_004070	chr1:16358986-16359688	CLCNKA
A_23_P416142	7.265576	7.642337	NM_004087	chr3:196771557-196771498	DLG1
A_23_P146354	8.58634	8.78534	NM_007171	chr9:134399126-134399185	POMT1
A_33_P3346937	4.4968333	5.0791097	NM_001278471	chr12:62902156-62902215	MON2
A_23_P84118	2.3221061	2.3900566	NM_004934	chr5:19473231-19473172	CDH18
A_32_P71943	7.642805	7.3518367	NM_015375	chr1:205112678-205112619	DSTYK
A_33_P3267375	4.3835382	4.119054	NM_000337	chr5:156192946-156193005	SGCD
A_23_P211878	5.919621	5.9197063	NM_001457	chr3:58157442-58157501	FLNB
A_23_P97736	4.0712385	4.107775	NM_014284	chr1:36031889-36031948	NCDN
A_33_P3716128	11.449564	11.325333	NM_005496	chr3:160151581-160151640	SMC4
A_23_P319583	5.7970257	6.035398	NM_014747	chr1:41086628-41086569	RIMS3
A_33_P3319925	4.590727	4.0802355	NM_001258406	chr13:77532009-77532068	IRG1
A_23_P115519	5.2938805	5.2392454	NM_032563	chr1:152552017-152551958	LCE3D
A_33_P3401902	6.497229	6.2572794	NM_001012421	chr9:42396424-42406729	ANKRD20A2
A_24_P132099	9.373334	9.783531	NM_138730	chr6:79913341-79912092	HMGN3
A_24_P220618	11.149379	11.031063	NM_015168	chr19:47567599-47567540	ZC3H4
A_33_P3222539	4.750773	4.7267547	DA751309	chr17:41392608-41392549	
A_32_P466514	5.629836	5.463626	NM_024496	chr14:77491992-77491933	IRF2BPL
A_33_P3218004	4.9569573	4.4474626	NR_040288	chr1:19592551-19592492	AKR7L
A_33_P3402551	6.2879086	5.8434305		chrX:103232601-103232660	H2BFXP
A_24_P917866	11.923009	11.300554	NM_003011	chr9:131457742-131457801	SET
A_23_P436353	9.195574	9.049707	NM_001207008	chr6:168372502-168372561	MLLT4
A_23_P217114	6.1677475	6.4831843	NM_000031	chr9:116149233-116149174	ALAD
A_23_P134204	4.7165117	4.5060787	NM_032599	chr7:128371559-128371618	FAM71F1
A_23_P424597	6.9920764	6.4126034	NM_152482	chr19:1473361-1473302	C19orf25
A_32_P93391	4.737569	4.745923	NM_001105570	chr19:33203790-33203849	NUDT19
A_33_P3373139	5.3691754	5.228609	AK131107	chr1:43894857-43894916	SZT2
A_33_P3271599	7.742431	7.6916647	NM_176816	chr5:68576655-68576596	CCDC125
A_23_P124855	6.939436	6.559972	NM_032226	chr9:37357730-37357789	ZCCHC7
A_33_P3397658	6.969983	6.2553296		chr5:150033735-150033794	SYNPO
A_24_P194337	4.442566	4.266278	NM_022115	chr21:43221875-43221816	PRDM15
A_24_P306964	11.001445	11.182416		chrX:039724756-039724817	
A_23_P124476	7.401044	7.806467	NM_173872	chr4:170641794-170641853	CLCN3
A_23_P90089	6.555956	6.4284997	NM_013976	chr19:13008607-13008666	GCDH
A_33_P3889179	3.9905672	4.042398	NR_027104	chr3:63110678-63110737	LINC00698
A_24_P145047	5.232066	4.6336136	NM_015042	chr15:64977870-64977929	ZNF609
A_23_P321959	9.415828	9.546795	NM_145169	chr6:166743667-166743037	SFT2D1
A_32_P56392	4.9696865	5.4164343	NM_002139	chrX:135961540-135961481	RBMX
A_23_P56680	13.449977	13.194869	NM_001002258	chr2:176043073-176043014	ATP5G3
A_23_P9293	8.387245	8.0198145	NM_004817	chr9:71869444-71869503	TJP2
A_33_P3290085	7.480794	7.573908	NM_032164	chr7:99091264-99091205	ZNF394
A_33_P3293396	7.5202866	7.3181543	NM_001004432	chr1:151772840-151772781	LINGO4
A_24_P453921	4.961007	4.9834547	NR_036680	chr7:32663022-32660675	DPY19L1P1
A_23_P37005	6.512019	6.388875	NM_004294	chr13:41797445-41791359	MTRF1
A_33_P3232557	5.137127	5.283169	NM_001080418	chr1:35331097-35331038	DLGAP3
A_23_P86653	11.208235	11.414224	NM_002727	chr10:70863833-70863892	SRGN
A_33_P3681776	4.3141623	3.9897435	NR_029409	chr11:1331801-1331860	TOLLIP-AS1
A_23_P401084	4.68843	4.587993	NM_174945	chr19:44040017-44040076	ZNF575
A_33_P3239338	10.483363	10.468655	NM_001105540	chr11:46402038-46402097	DGKZ
A_33_P3402773	3.8556492	3.7635486		chr22:024285001-024285060	
A_23_P98282	7.7807646	8.194864	NM_006946	chr11:66453169-66453110	SPTBN2

A_24_P156886	5.6309433	5.616771	NM_001031725	chr1:200618321-200618262	DDX59
A_24_P175460	6.392474	6.3334265	NM_015447	chr9:138700936-138700877	CAMSAP1
A_33_P3233030	12.858977	12.97972		chr6:019144246-019144305	
A_33_P3824237	7.345918	7.09562	NR_038464	chr10:81979351-81979410	LINC00857
A_24_P388703	5.185197	5.295994	XR_244602	chr4:36258205-36258264	
A_33_P3408655	5.1179295	5.0807567	AK310676	chr1:117307950-117308009	CD2
A_33_P3246598	7.5098395	7.5058002	NM_001010906	chr8:27880017-27879958	NUGGC
A_32_P77977	5.7723846	5.3750353	NM_016037	chr1:38489953-38490012	UTP11L
A_33_P3353073	4.4846864	3.9398422	NM_022047	chr6:35280093-35280152	DEF6
A_23_P28815	2.3221061	2.3900566	NM_000782	chr20:52771005-52770946	CYP24A1
A_32_P209230	10.498919	10.471599	NM_133467	chr1:41326794-41326735	CITED4
A_33_P3265419	4.3853736	4.301828		chr16:089525839-089525898	
A_24_P142473	8.123773	8.166818	NM_015343	chr17:7149599-7149424	CTDNEP1
A_33_P3272169	4.6150694	4.150193	NM_003388	chr7:73815837-73815896	CLIP2
A_24_P56484	5.317926	5.712416	NM_032352	chr14:36340379-36340438	BRMS1L
A_33_P3372840	5.7844377	5.633006	NM_001033886	chr10:44871424-44871365	CXCL12
A_33_P3366195	4.1975965	4.459221	NM_014862	chr15:80873589-80873648	ARNT2
A_33_P3388080	7.410246	7.142825		chr14:75763169-75763228	LOC731223
A_33_P3415166	4.1242704	3.930831			
A_33_P3283944	3.7459493	4.170752		chr3:105085962-105085903	
A_23_P133408	4.622114	3.1259353	NM_000758	chr5:131411452-131411511	CSF2
A_24_P181149	6.172555	6.5048122	NM_024685	chr12:76738836-76738777	BBS10
A_32_P755542	8.576487	8.277914	NM_138807	chr3:11832001-11831942	TAMM41
A_23_P13663	9.010069	8.426781	NM_021238	chr12:31435673-31435614	FAM60A
A_24_P64039	7.214982	7.269779	NM_006803	chr8:42028441-42028500	AP3M2
A_23_P126388	10.675057	10.613995	NM_031286	chr1:26607808-26607867	SH3BGRL3
A_24_P595237	6.5700097	6.417445	NM_001105579	chr14:74873032-74872973	SYNDIG1L
A_23_P209183	8.278156	8.297493	NM_024656	chr19:17693237-17693296	COLGALT1
A_23_P130488	7.6095476	7.4886293	NM_000400	chr19:45856574-45856515	ERCC2
A_33_P3325102	4.1897483	4.265482		chr4:056814185-056814126	
A_33_P3349395	8.275896	8.236131	AF411609	chr22:18019144-18019203	CECR2
A_33_P3304883	4.187986	4.0868816	XR_171616	chr7:469712-469653	LOC100131372
A_23_P253561	9.405342	9.19921	NM_024331	chr20:43123082-43123141	TTPAL
A_23_P161719	6.912958	6.8371134	NM_152434	chr11:107197449-107197390	CWF19L2
A_33_P3332135	7.4718556	7.6971006	NM_001143804	chr17:47300802-47300743	PHOSPHO1
A_23_P329740	14.1936245	14.082455	NM_021009	chr12:125396272-125396213	UBC
A_23_P97309	11.713622	12.38113	NM_001229	chr1:15819038-15818979	CASP9
A_23_P60227	4.1427608	4.3363676	NM_005893	chr9:36171208-36171267	CCIN
A_24_P75920	4.691977	4.7610846	NM_001080503	chr19:11462734-11462793	CCDC159
A_23_P311232	7.8795958	7.742431	NM_015282	chr2:122095736-122095677	CLASP1
A_24_P917886	5.210128	5.329921	XM_005275698	chr11:1213031-1213090	LOC101927530
A_23_P111492	3.8978248	3.788034	NM_023948	chr7:100211122-100211181	MOSPD3
A_23_P65230	8.605183	8.324563	NM_032813	chr13:101256471-101256412	TMTC4
A_33_P3467933	4.561072	4.836327	AK096739	chr8:103906319-103906260	LOC286154
A_23_P69493	11.179016	11.848805	NM_001664	chr3:49397271-49397212	RHOA
A_32_P156963	14.44517	14.403733	NM_001614	chr17:79477625-79477566	ACTG1
A_33_P3489737	8.06154	8.200932	NM_020726	chr5:65119097-65119156	NLN
A_24_P7652	5.0109844	4.9179087	NM_152780	chrX:20071076-20071017	MAP7D2
A_23_P216094	7.3708105	7.3316326	NM_004318	chr8:62460683-62438666	ASPH
A_33_P3243399	6.8486524	6.7019353	AY358410	chr3:57876874-57876933	SLMAP
A_32_P514790	8.771538	8.502752	NM_001080419	chr17:73821774-73821833	UNK
A_24_P286054	7.983845	7.8905044	NM_001105251	chr5:79774799-79774858	ZFYVE16
A_33_P3215575	7.280497	7.5064716	NM_018125	chr1:18024085-18024144	ARHGEF10L
A_33_P3213377	5.894452	5.698203	NM_032549	chr7:110303717-110303658	IMMP2L
A_33_P3240951	3.2509978	2.3900566	NM_001280544	chr14:73136788-73136729	DPF3
A_33_P3303015	13.2274275	13.179645	NM_001013	chr19:54710186-54710245	RPS9
A_23_P368393	5.2844033	5.450593	NM_001005526	chr2:198285791-198285246	SF3B1
A_24_P137582	7.14176	7.285084	NM_170681	chr5:74032688-74032629	GFM2
A_23_P140475	8.768609	9.100391	NM_024505	chr15:69348985-69349044	NOX5

A_33_P3209716	5.882281	6.2078514	NM_001206885	chr11:57582867-57582926	CTNND1
A_23_P63343	7.7203217	7.36665	NM_021995	chr1:7907888-7907829	UTS2
A_23_P368909	4.258608	4.462004	NR_027701	chr13:111521859-111521800	LINC00346
A_23_P43412	4.260461	4.851143	NM_018437	chr9:100692550-100692491	HEMGN
A_24_P38951	8.9273205	8.804282	NM_032871	chr11:73107623-73107682	RELT
A_23_P162525	9.084095	8.728976	NM_014503	chr12:101777351-101777410	UTP20
A_23_P394064	13.3064995	13.285886	NM_012232	chr17:40554536-40554477	PTRF
A_24_P322474	7.359537	7.2004614	NM_006202	chr19:10580226-10580285	PDE4A
A_33_P3398667	4.1340685	4.3769636	NM_001166012	chr18:32832922-32832863	ZSCAN30
A_24_P517252	4.6143723	4.6238832	NM_001077620	chr17:74539195-74539935	PRCD
A_23_P54991	8.853287	8.662472	NM_080677	chr17:56166747-56166806	DYNLL2
A_33_P3347522	8.100642	8.556328		chr16:029887696-029887637	
A_33_P3373200	3.71692	3.4155488		chr16:032298819-032298760	
A_33_P3390539	3.669232	3.7795384	NM_152312	chr11:45948027-45948086	GYLTL1B
A_24_P179044	6.5092087	6.726758	NM_016224	chr6:158330798-158330974	SNX9
A_23_P88589	11.279459	11.553421	NM_021005	chr15:96881957-96882016	NR2F2
A_23_P90172	4.7962537	4.652873	NM_014330	chr19:49378127-49378901	PPP1R15A
A_32_P93996	5.6070976	5.0548425	NR_036505	chr9:95382248-95382307	LOC100128361
A_23_P21673	8.772168	8.042591	NM_017794	chr9:20995798-20995857	FOCAD
A_33_P3410251	7.5647116	7.4238477	AK090815	chr2:210661028-210661087	UNC80
A_23_P152107	10.857246	10.745641	NM_194259	chr16:1374948-1375007	UBE2I
A_23_P159650	10.905479	10.944987	NM_001866	chrX:77158204-77158263	COX7B
A_33_P3344169	4.8873253	5.0881166	NM_001033560	chr15:55727163-55727104	DYX1C1
A_33_P3258091	11.554306	11.580425	NM_020216	chr1:201975125-201975184	RNPEP
A_33_P3376214	4.1737785	4.000232	NM_001144829	chr1:146656056-146655997	FMO5
A_33_P3347457	7.056165	8.029783		chr13:029965657-029965598	
A_33_P3229397	12.618595	12.319452	NM_006430	chr2:62096635-62096576	CCT4
A_32_P34116	8.607964	8.867994	NM_138459	chr6:118030280-118030339	NUS1
A_33_P3378935	4.5786757	4.9511166	AB209007	chr11:66391379-66391438	RBM14
A_24_P161959	6.719293	6.910077	NM_199337	chr11:62556871-62557104	TMEM179B
A_23_P77066	8.099984	8.240751	NM_022807	chr15:25220523-25220582	SNRPN
A_32_P57810	3.258608	3.531308	NM_052916	chr17:74141004-74140945	RNF157
A_33_P3379381	4.817546	4.4374905	NM_207014	chr1:67337127-67337068	WDR78
A_24_P74160	13.853955	13.9824295	NM_177542	chr19:46190933-46190874	SNRPD2
A_33_P3365720	5.6230164	5.6423264		chr4:3590841-3590900	LINC00955
A_33_P3324692	6.6504245	6.9107094	NM_001136152	chr3:129813261-129813320	ALG1L2
A_23_P136978	4.581627	3.149965	NM_014467	chrX:99925973-99926032	SRPX2
A_23_P216340	3.5762744	3.4663854	NM_001045556	chr8:134049150-134049091	SLA
A_24_P29594	5.619067	5.508963	NM_006620	chr6:135282505-135282446	HBS1L
A_24_P331904	11.213191	11.45044	NM_017828	chr15:75632493-75632552	COMMD4
A_24_P942250	8.199198	8.238406	NM_025134	chr16:53360496-53360555	CHD9
A_24_P183994	3.8393123	2.3900566	AK056176	chr9:85670564-85670505	RASEF
A_23_P3368	3.7491624	3.8687072	NM_002569	chr15:91425997-91426056	FURIN
A_23_P137689	6.2010627	6.415782	NM_015441	chr1:161953276-161953217	OLFML2B
A_33_P3285911	6.613397	6.4562674	NM_153369	chr6:111590174-111590233	KIAA1919
A_24_P69691	5.1577024	4.0053477	NM_145011	chr10:38239838-38239779	ZNF25
A_33_P3356607	9.461298	9.798756	NM_001033518	chr7:5269265-5269324	WIPI2
A_33_P3315841	4.235204	4.921929		chr4:185926202-185926261	
A_23_P152235	7.346852	7.646952	NM_024336	chr16:54317289-54317230	IRX3
A_24_P107291	7.70335	7.5904126	NM_181699	chr11:111612850-111612791	PPP2R1B
A_23_P330836	3.912629	4.1013584	BC025179	chrX:47343219-47343278	
A_24_P11061	4.4338098	4.966156	NM_153478	chrX:151909253-151909312	CSAG1
A_33_P3281408	9.391754	9.917894	NM_001127401	chr2:30382347-30382406	YPEL5
A_23_P214969	10.460846	10.636689	NM_006079	chr6:139693651-139693592	CITED2
A_33_P3395859	5.531922	5.260845	NM_001193623	chr19:51891601-51891543	LOC147646
A_23_P432610	6.7564597	6.725454	NM_153029	chr16:48572899-48572840	N4BP1
A_23_P164528	8.764688	8.775371	NM_015285	chr18:54696848-54696907	WDR7
A_24_P289043	7.09986	7.935431		chr9:004945132-004945071	
A_24_P168510	6.7094836	5.765214	NM_024069	chr19:18675774-18677943	KXD1

A_23_P144096	5.381627	5.275268	NM_145071	chr3:50644082-50644023	CISH
A_33_P3399581	5.9488754	5.5587397	NM_004412	chr10:17216610-17216551	TRDMT1
A_23_P344988	7.5064716	7.3794193	NM_016513	chr6:52866185-52866126	ICK
A_24_P388810	10.346785	10.206724	NM_003135	chr5:112203114-112203173	SRP19
A_33_P3876192	5.433122	5.384802	BQ708343		IGLV1-44
A_23_P60599	4.458112	4.689187	NM_001072	chr2:234681607-234681665	UGT1A6
A_23_P28697	4.45704	4.4373007	NM_012205	chr2:42994768-42994630	HAAO
A_33_P3418790	4.3578286	4.76834		chr20:3025020-3025079	GNRH2
A_33_P3232798	4.870777	4.6683345	NM_025151	chr8:37756669-37756610	RAB11FIP1
A_33_P3423820	10.663321	10.615054	NM_015117	chr8:144519885-144519826	ZC3H3
A_33_P3879920	11.645525	11.684499	NM_001199119	chr6:30314558-30314617	TRIM39-RPP21
A_33_P3382281	3.4506564	4.045641		chr20:10616953-10617012	SLX4IP
A_23_P69339	9.808617	9.621325	NM_001607	chr3:38164415-38164356	ACAA1
A_24_P413988	11.082241	10.523144	NM_006464	chr2:85545401-85545342	TGOLN2
A_33_P3386487	3.0894427	3.5119681		chr7:031402201-031402142	
A_33_P3342410	8.896365	8.150939	NM_013302	chr16:22299977-22300036	EEF2K
A_33_P3262191	9.300365	9.187789	NM_014427	chr16:89663594-89663653	CPNE7
A_23_P409438	4.800382	5.422309	NM_172138	chr19:39760635-39760694	IFNL2
A_23_P340158	10.8095455	11.072892	NM_014301	chr12:108963027-108963086	ISCU
A_33_P3290949	5.3791523	5.4088244		chr2:86363932-86363991	PTCD3
A_33_P3280213	7.068927	7.353409	NM_001127695	chr20:44523321-44523380	CTSA
A_24_P625382	11.109095	11.433928	NM_003651	chr12:10851700-10851674	YBX3
A_23_P210109	4.6070113	4.292918	NM_019885	chr2:72357489-72357430	CYP26B1
A_23_P126135	10.906133	10.121149	NM_014874	chr1:12073420-12073479	MFN2
A_24_P808522	11.4237995	11.407869	NM_005617	chr5:149829298-149827272	RPS14
A_33_P3253723	5.2110662	5.01541	NM_198098	chr7:30963485-30963544	AQP1
A_33_P3300395	8.434881	8.357563	NM_199294	chr1:10502671-10502730	APITD1
A_32_P405902	3.4764006	2.3900566	NR_026755	chr21:15215466-15215437	C21orf15
A_33_P3351775	8.472649	8.705528		chr1:059465938-059465997	
A_23_P71867	4.176432	4.334748	NM_001142784	chr9:34660510-34660569	IL11RA
A_33_P3323074	4.281467	3.6455936	NM_020133	chr6:161570280-161570221	AGPAT4
A_33_P3508822	11.084706	11.344489	NM_000484	chr21:27253195-27253136	APP
A_32_P40288	2.9550838	2.3900566	NM_052913	chr6:130763993-130764052	TMEM200A
A_33_P3269924	4.599357	4.4425077	XM_005253629	chr12:123319405-123319464	HIP1R
A_33_P3474250	9.298838	8.734483	AK091052	chr11:35839086-35839145	LOC283270
A_32_P74120	7.9750214	7.9416413	NR_024584	chr1:144480846-144480787	LOC728875
A_24_P664850	4.6380124	4.673874	NM_006011	chr15:93011323-93011382	ST8SIA2
A_24_P17719	5.782507	6.594767	NM_015990	chr4:39114673-39114732	KLHL5
A_23_P387630	7.6304746	7.8959913	NM_014725	chrX:67945527-67945586	STARD8
A_33_P3355281	6.335672	5.9790945	NM_017584	chr22:50928406-50928465	MIOX
A_24_P335901	5.8785725	6.019103	NM_006631	chr19:9523810-9523751	ZNF266
A_23_P352950	5.929988	6.2829266	NM_052926	chrX:152157755-152157696	PNMA5
A_23_P330895	5.798969	5.448083	NR_040515	chr19:39398611-39398670	NFKBIB
A_24_P270814	7.269024	7.593309	NM_016823	chr17:1326464-1326405	CRK
A_24_P349616	7.6531897	7.4530363	NM_001076683	chr17:42282767-42282708	UBTF
A_33_P3245218	2.7135367	3.568415	NM_001122679	chr5:167689754-167689813	TENM2
A_33_P3240996	6.281924	6.4044824	XR_158980	chr2:133110835-133110894	FAM201B
A_33_P3245620	6.071078	6.438869		chr4:102076085-102076144	
A_32_P149174	5.1381335	5.17881	NM_001008739	chr6:42858285-42858226	C6orf226
A_24_P161144	4.3617325	4.647216	NM_001136509	chr16:31447051-31446992	ZNF843
A_23_P210869	9.882057	9.973661	NM_032609	chr20:30232674-30232732	COX4I2
A_33_P3358930	5.4766765	5.6961875	XM_005276062		LOC101930441
A_23_P69452	6.358258	6.507464	NM_016216	chr3:137879870-137879830	DBR1
A_33_P3361741	7.7035174	7.0570316	NM_013238	chr13:43683245-43683304	DNAJC15
A_23_P256047	4.836327	4.8054385	NM_022096	chr20:10036886-10036945	ANKEF1
A_23_P75622	13.618645	13.754819	NM_006476	chr11:118279768-118279827	ATP5L
A_33_P3314176	2.8719316	2.3900566	NM_017709	chr1:118170883-118170942	FAM46C
A_23_P41872	5.2717924	4.7505126	NM_007255	chr5:177035912-177035971	B4GALT7
A_33_P3339070	3.729463	3.1615582		chr10:4692436-4692377	LINC00704

A_23_P131526	9.827078	9.699641	NM_024520	chr2:200826633-200828496	C2orf47
A_33_P3307147	3.4805696	4.337574	NM_001166247	chr6:102513722-102513781	GRIK2
A_23_P134176	9.384396	10.02828	NM_001024465	chr6:160103649-160103590	SOD2
A_24_P193498	6.505995	6.8019996	NM_078474	chr15:102190214-102187051	TM2D3
A_33_P3263387	6.3998	6.636947	NM_014640	chr2:219620077-219620136	TTLL4
A_24_P416177	9.468208	8.934218	NM_001114	chr16:50351847-50351906	ADCY7
A_23_P65129	5.767525	5.5734634	NM_032840	chr12:53458705-53458646	SPRYD3
A_33_P3248973	4.308875	4.4997497	AK128820	chr19:7011708-7011767	
A_33_P3258593	7.136556	7.5604753	NM_005039	chr12:11506291-11506232	PRB1
A_32_P202182	4.9630837	4.8218946	NR_003664	chr7:72498539-72498480	SPDYE8P
A_23_P425073	7.702751	7.6795444	NM_002898	chr12:56982765-56982945	RBMS2
A_33_P3279049	5.062626	5.4207287	NM_080599	chr10:11971923-11971864	UPF2
A_33_P3274199	9.617615	9.706449	NM_138349	chr17:27900112-27900171	TP53I13
A_24_P375849	12.496462	12.474697		chr5:116052068-116052127	
A_33_P3262575	8.81872	8.831744	NM_018842	chr7:97922626-97922567	BAIAP2L1
A_23_P400459	6.5789366	6.46863	NM_144964	chr9:37776355-37777609	TRMT10B
A_33_P3380063	5.194413	5.470572	NM_025008	chr1:150531065-150531124	ADAMTSL4
A_33_P3350549	8.361498	7.968419		chr11:067512844-067512785	
A_23_P13479	4.0441394	4.2008724	NM_001004725	chr11:48327950-48328009	OR4S1
A_33_P3302453	2.9049067	2.9320788	NR_026781	chr6:170189004-170188945	LINC00242
A_24_P174755	7.6352296	7.64884	NM_003060	chr5:131731096-131731155	SLC22A5
A_33_P3290235	6.408836	6.4393454	NR_034152	chr20:31196447-31196506	LOC149950
A_33_P3420992	10.712345	10.567489		chrX:061999677-061999736	
A_23_P85952	2.3221061	2.8917742	NM_024901	chr1:111730006-111729947	DENND2D
A_23_P168062	7.9551888	7.9213758	NM_003587	chr6:30622627-30622568	DHX16
A_33_P3253807	8.72299	7.928439	NM_001806	chr19:33873466-33873525	CEBPG
A_23_P259823	7.3977823	7.0225463	NM_017910	chr2:29092446-29087928	TRMT61B
A_33_P3281139	7.3029737	7.1178226	XM_005266303	chr13:46531321-46531262	ZC3H13
A_33_P3378835	8.066126	7.4216957	NM_004252	chr17:72765312-72765371	SLC9A3R1
A_33_P3396159	2.5324557	3.222896	NM_005436	chr10:61552401-61552342	CCDC6
A_24_P204144	14.948836	14.990484		chr6:043331750-043331810	
A_23_P51660	8.362931	8.255348	NM_012222	chr1:45795058-45794999	MUTYH
A_33_P3275381	6.014085	5.208172	NM_001039580	chr4:156289798-156289739	MAP9
A_33_P3334491	4.1234217	3.8677955	NR_026789	chr8:12245468-12245527	FAM66A
A_33_P3393843	4.643806	5.1055794	NM_001282690	chr9:91972355-91972414	SECISBP2
A_33_P3260623	5.0004463	4.6787844	NM_000932	chr11:64033812-64033871	PLCB3
A_24_P944444	6.993589	7.1442966	NM_014994	chr15:42119024-42119083	MAPKBP1
A_33_P3288924	5.45362	5.5429554	AK098478	chr9:130318385-130318326	
A_33_P3292332	10.914124	9.871683	NM_004521	chr10:32298059-32298000	KIF5B
A_23_P146444	6.7296367	6.7620444	NM_003389	chr9:100883421-100883362	CORO2A
A_32_P204218	4.900689	4.8504796	NM_205855	chr7:135418886-135418827	FAM180A
A_23_P253029	6.9706755	6.8641896	NM_032515	chr2:242513018-242513077	BOK
A_23_P92196	5.340935	5.381627	NM_032579	chr3:108475936-108475407	RETNLB
A_23_P26905	8.233505	8.062497	NM_007215	chr17:62476467-62476408	POLG2
A_23_P137073	4.8715506	4.586767	NM_005096	chrX:70459485-70459456	ZMYM3
A_24_P67027	6.098025	6.3114586	NM_003993	chr1:155235718-155235659	CLK2
A_23_P77813	9.068194	9.32161	NM_024619	chr17:80685465-80685524	FN3KRP
A_33_P3216448	4.729604	5.171521	NM_001163771	chr6:33154291-33154232	COL11A2
A_32_P47988	5.7914987	5.9103317	NM_001221	chr4:114680477-114582871	CAMK2D
A_33_P3374087	4.0487113	3.9821193	NM_001080504	chr2:238743008-238743066	RBM44
A_23_P140029	7.276844	7.474199	NM_007106	chr13:30341389-30341330	UBL3
A_33_P3213561	9.0874815	9.508649		chr15:043423828-043423887	
A_24_P163113	10.139103	9.92254	NM_017548	chr3:133293108-133293921	CDV3
A_33_P3215557	5.54274	5.379429	NM_014272	chr15:79058167-79058108	ADAMTS7
A_23_P168567	13.0954075	12.978469	NM_032999	chr7:74174900-74174959	GTF2I
A_33_P3252281	5.709439	5.437442	NM_004100	chr6:133852598-133852657	EYA4
A_33_P3262685	4.05871	3.5693467		chr22:22975037-22974978	POM121L1P
A_33_P3308132	11.973688	12.198191		chr14:19887573-19887514	XLOC_014512
A_24_P346431	7.932238	8.082838	NM_022748	chr7:47315278-47315219	TNS3

A_33_P3396635	8.003159	8.373169	NM_021014	chrX:48206180-48206121	SSX3
A_23_P103690	9.284318	9.478596	NM_006589	chr1:155217344-155217285	FAM189B
A_23_P100056	8.002896	8.131224	NM_194272	chr15:65032375-65032316	RBPMS2
A_33_P3367392	5.553525	5.2026043	NM_032648	chr1:32714400-32714459	FAM167B
A_23_P68505	8.865486	8.995847	NM_001190826	chr20:58522968-58523027	FAM217B
A_24_P49371	8.509924	8.338845	NM_017867	chr4:170653013-170652954	C4orf27
A_33_P3252884	7.8871584	7.896421	NM_001033561	chr17:27232365-27232306	PHF12
A_24_P303647	6.676547	7.193801	NM_152556	chr7:112459482-112459423	C7orf60
A_33_P3410859	4.409197	4.380305	NR_003288	chr6:160517125-160517184	LOC729603
A_24_P356	4.285987	4.5129166	AB028971	chr2:69708481-69708422	AAK1
A_33_P3418611	9.502741	9.770326	NM_001242680	chr19:22499726-22499785	ZNF729
A_33_P3297297	4.4128594	4.0623436		chr2:085596806-085596865	
A_23_P80068	9.687826	9.487854	NM_006806	chr21:18966119-18966060	BTG3
A_33_P3210760	6.514864	6.498874	AI205683	chr15:082635144-082635203	
A_33_P3367106	10.589095	10.584441	NR_003242	chr1:144612298-144612239	PFN1P2
A_33_P3341586	7.384439	7.046675	NM_001037633	chr5:138282487-138282428	SIL1
A_23_P24535	7.4999027	7.568716	NM_017868	chr11:113236997-113237056	TTC12
A_33_P3414362	5.177419	5.1298637	NM_032582	chr17:58329802-58329743	USP32
A_23_P209269	9.128119	9.492376	NM_001033557	chr2:44458389-44458448	PPM1B
A_23_P203994	11.099016	11.05386	NM_002813	chr12:122353833-122354231	PSMD9
A_23_P65370	11.5150795	11.575678	NM_016417	chr14:96010787-96010846	GLRX5
A_24_P127719	6.059264	5.9431615	NM_201589	chr8:144512493-144512434	MAFA
A_24_P919899	3.8038797	3.1442897	AF111705	chr1:47099554-47099495	ATPAF1
A_23_P29924	8.446927	8.429908	NM_032927	chr4:4237590-4237531	TMEM128
A_23_P36187	4.4162335	3.4627028	NM_138567	chr11:1858202-1858261	SYT8
A_23_P133293	9.360671	9.57299	NM_024717	chr5:94042412-94042353	MCTP1
A_33_P3225066	3.8404298	4.0107465	NM_201262	chr10:69583132-69583073	DNAJC12
A_24_P391853	11.027883	10.268453		chr2:180679236-180679295	
A_33_P3402565	2.3221061	2.3900566	NM_004415	chr6:7586052-7586111	DSP
A_33_P3480561	4.3318396	4.4036913	NM_001271025	chr10:4875583-4875642	AKR1E2
A_33_P3218118	4.44946	4.423313		chr22:019019100-019019041	
A_33_P3282693	4.3266883	4.436105	NR_024128	chr17:3214592-3214651	OR3A4P
A_33_P3221432	3.0746794	3.2883577	NM_001037813	chr19:44591105-44591164	ZNF284
A_23_P154643	5.5627766	5.4777975	NM_001719	chr20:55743844-55743791	BMP7
A_24_P272761	5.8303337	5.7170568	NM_020946	chr9:126142528-126142469	DENND1A
A_24_P116909	6.289295	6.2102346	NM_006785	chr18:56416474-56416533	MALT1
A_33_P3362933	7.759449	7.884597	NM_002099	chr4:145035902-145035843	GYPA
A_33_P3322589	8.027766	8.182989	NM_001114636	chr2:58387309-58387250	FANCL
A_33_P3277758	4.674445	4.507998		chr15:028811032-028810973	
A_33_P3276918	6.063493	5.9126263	NM_001195228	chr17:6352667-6352726	FAM64A
A_23_P80062	8.176308	8.305651	NM_003185	chr20:60550411-60550352	TAF4
A_33_P3419026	4.1550503	3.6225758	NM_001142700	chr11:83674065-83674006	DLG2
A_23_P112397	9.86145	9.940416	NM_018998	chr9:139835322-139835263	FBXW5
A_23_P168211	9.090264	9.620072	NM_030796	chr7:55538749-55538690	VOPP1
A_23_P132595	6.9216022	7.309901	NM_014667	chr3:11598018-11597959	VGLL4
A_24_P358328	11.884546	12.185336	NR_002187	chr7:128696929-128696988	TPI1P2
A_33_P3411773	4.9495816	5.2479544	XR_243328	chr16:691098-691039	LOC100130285
A_24_P339869	6.18791	5.6242156	NM_020727	chr21:43407139-43407080	ZBTB21
A_23_P98092	10.877258	11.183286	NM_000274	chr10:126086316-126086257	OAT
A_33_P3262717	4.154126	4.137886	NM_018028	chr19:39870627-39870686	SAMD4B
A_33_P3382595	10.519317	10.5808325	NR_001445	chr6:52860659-52860718	RN7SK
A_33_P3218625	7.8921127	8.018768	NM_001242369	chr9:136335820-136335879	CACFD1
A_23_P213678	10.73748	11.063667	NM_000919	chr5:102365129-102365188	PAM
A_23_P431933	6.01385	5.4819927	NM_032294	chr17:3763939-3763880	CAMKK1
A_33_P3284919	8.317097	8.244591	NM_001178061	chr1:151104255-151104196	SEMA6C
A_23_P207766	8.973663	8.752097	NM_004309	chr17:79826042-79825983	ARHGDIA
A_23_P219013	5.363846	5.5769844	NM_033450	chr6:43416902-43417178	ABCC10
A_24_P466374	8.230689	8.299065	NM_001990	chr1:28297293-28297234	EYA3
A_32_P119569	9.042355	9.063402	NR_033350	chr15:23437935-23438849	GOLGA8EP

A_23_P15639	7.2350373	6.6798096	NM_022344	chr17:30662372-30661830	C17orf75
A_33_P3390102	12.970199	12.615065	NM_005716	chr19:14588633-14588574	GIPC1
A_33_P3327663	6.3686686	6.528289		chr1:223533605-223533546	SUSD4
A_23_P74359	10.269528	9.872628	NM_004078	chr1:201453110-201453051	CSRP1
A_24_P203678	8.735383	9.12239	NM_000019	chr11:108005883-108005942	ACAT1
A_24_P414719	7.046675	7.100911	NM_002505	chr6:41069914-41069973	NFYA
A_24_P303815	4.6981144	5.3800125	NM_152896	chr9:6506080-6506139	UHRF2
A_24_P152188	6.837342	6.9202237	NM_198859	chr3:64079998-64079939	PRICKLE2
A_33_P3367795	5.0823	5.092864		chr9:19379001-19378942	RPS6
A_24_P194081	9.8565035	9.650956	NM_144779	chr19:35651651-35655064	FXYD5
A_24_P252078	8.323123	8.301429	NM_007047	chr6:26378257-26378316	BTN3A2
A_23_P138435	10.912896	10.619726	NM_020338	chr10:81076036-81076095	ZMIZ1
A_23_P140978	4.834055	4.6358366	NM_005741	chr16:3340525-3340584	ZNF263
A_33_P3317664	5.211609	4.9413576	NM_001114618	chr5:180218766-180218707	MGAT1
A_33_P3390335	8.186218	8.630306	NM_012425	chr10:16635447-16635388	RSU1
A_24_P130865	6.2865562	5.7667723	NM_015107	chrX:53989350-53989291	PHF8
A_23_P26358	9.267944	9.145502	NM_015092	chr16:18872071-18872012	SMG1
A_33_P3254844	2.8897042	3.6942341	NM_006890	chr19:42178513-42178454	CEACAM7
A_33_P3328772	5.3278275	5.766141	NM_001190860	chr12:19354851-19354910	PLEKHA5
A_33_P3281363	5.005409	4.9446282	BC037956	chr2:230695852-230695793	TRIP12
A_24_P19752	8.752097	8.46088	NM_002857	chr1:160246867-160246808	PEX19
A_23_P2814	6.6875815	6.8379507	NM_005905	chr13:37422872-37422813	SMAD9
A_33_P3292043	4.88512	4.359969	NM_001005214	chr1:165533125-165533184	LRRC52
A_23_P139558	8.844009	9.035004	NM_022771	chr12:72316935-72316994	TBC1D15
A_23_P500333	6.157459	5.9623246	NM_001009955	chr1:54693945-54692792	SSBP3
A_23_P19482	10.120106	10.453484	NM_013974	chr6:31694977-31694918	DDAH2
A_23_P24077	7.3369412	7.257102	NM_022153	chr10:73510437-73510378	C10orf54
A_33_P3385488	10.880321	10.820656	NM_022551	chr6:33240433-33240492	RPS18
A_33_P3280993	9.088555	9.044441	NR_102326	chr6:30255233-30255174	HCG18
A_33_P3375496	5.1027184	4.9010086		chr1:205687507-205687566	
A_33_P3209537	5.001174	5.100852	NM_001110514	chr20:2740322-2740381	EBF4
A_33_P3402474	7.677927	7.5324373	NM_001031722	chr6:30614515-30614574	ATAT1
A_24_P555170	4.6354437	4.4102507	NM_001243531	chr15:84850807-84850866	LOC100505679
A_23_P255601	5.734159	5.429881	NM_032508	chrX:148853905-148853964	TMEM185A
A_23_P24586	4.4954753	4.270191	NM_032592	chr11:44105056-44105115	ACCS
A_33_P3398236	4.344193	4.87599	AJ890452	chr7:7471198-7471139	COL28A1
A_23_P215525	8.0097275	7.721563	NM_015550	chr7:24843941-24839847	OSBPL3
A_32_P357301	4.329031	4.6196613	NM_016488	chr12:42841908-42841967	PPHLN1
A_33_P3351894	10.6323395	10.734433	NR_038911	chr22:24236690-24236631	LOC284889
A_33_P3339536	11.325022	11.269348	NM_013986	chr22:29696415-29696474	EWSR1
A_24_P166094	9.438278	9.253231	NM_001025595	chr4:153832920-153832979	ARFIP1
A_33_P3231677	4.689865	4.5049996		chr2:000874677-000874618	
A_24_P349151	6.309009	6.4891043	NM_194292	chr1:100549656-100549597	SASS6
A_33_P3369939	7.626023	6.6719956	NM_194283	chr5:34958867-34958926	DNAJC21
A_33_P3363071	4.1573315	4.700623	NM_198510	chrX:54775675-54775616	ITIH6
A_23_P164623	8.473644	8.2343855	NM_032792	chr19:59024971-59024912	ZBTB45
A_32_P52330	6.304735	6.489832	NR_024282	chr19:14185664-14185723	LOC113230
A_23_P348146	3.0035455	3.2536354	NM_001040153	chr13:78337918-78337977	SLAIN1
A_33_P3234490	5.54245	5.372482	NM_001039182	chr16:30205037-30204978	BOLA2B
A_33_P3252359	6.042638	5.818388	NM_203314	chr3:197236713-197236654	BDH1
A_33_P3403733	6.173131	6.1534653	NM_001282321	chr1:16999939-16999998	LOC729574
A_23_P213166	7.6390924	7.508208	NM_018392	chr4:113460845-113460786	C4orf21
A_23_P149419	7.014024	6.996868	NM_022078	chr1:27217429-27217370	GPATCH3
A_23_P20606	9.215517	9.4609	NM_015469	chr9:107521774-107521833	NIPSNAP3A
A_23_P345674	7.388839	6.7817435	NM_021216	chr19:57134699-57134758	ZNF71
A_24_P389959	9.820998	10.120717	NM_016057	chr12:54744570-54744629	COPZ1
A_23_P356101	5.702935	5.9175844	NM_001190274	chr2:48047585-48047526	FBXO11
A_23_P146497	7.950395	7.771772	NM_014811	chr9:138380281-138380340	PPP1R26
A_24_P358868	6.6598873	6.8406477	NM_001267716	chr19:23158862-23158803	ZNF728

A_33_P3267195	7.6565747	7.8712244		chr6:4611267-4611326	KU-MEL-3
A_23_P68949	10.029409	10.155862	NM_003932	chr22:41228665-41228606	ST13
A_23_P163402	4.6894593	4.65869	NM_000499	chr15:75012621-75012562	CYP1A1
A_24_P913716	7.88196	8.30791	NM_145236	chr2:232265452-232265511	B3GNT7
A_33_P3343750	6.54191	6.3330007	NM_152603	chr19:37211307-37211366	ZNF567
A_33_P3351052	7.9317927	8.0194645	NM_002699	chr1:38509710-38509651	POU3F1
A_33_P3281867	4.0411816	4.4594703		chr11:063400095-063400154	
A_23_P148446	10.969391	10.773226	NM_003336	chrX:118718120-118718179	UBE2A
A_23_P371039	3.5789948	2.3900566	NM_002531	chr20:61393800-61393860	NTSR1
A_23_P4798	6.190667	5.8935404	NM_016535	chr19:56156206-56156265	ZNF581
A_23_P88331	10.500504	10.791874	NM_014750	chr14:55615172-55615113	DLGAP5
A_33_P3282291	7.543674	7.5744853	AK097358		FLJ40039
A_23_P20804	8.929023	9.144638	NM_147202	chr9:34398447-34398388	FAM219A
A_33_P3395396	6.591788	6.644638	XM_005258552	chr19:37621153-37621212	ZNF420
A_23_P208208	2.903105	3.1763334	NM_023074	chr19:52392608-52392549	ZNF649
A_33_P3365666	4.8735466	4.9386644		chr7:076610569-076610628	
A_23_P114740	9.07064	9.745846	NM_000186	chr1:196716550-196716609	CFH
A_33_P3329664	4.732232	4.451049	NM_001136273	chrX:152685689-152685748	ZFP92
A_33_P3674851	8.115564	7.836921	NM_001282169	chr13:52986900-52986841	VPS36
A_33_P3318696	6.5750628	6.797575	NM_001008661	chr1:89426909-89426850	CCBL2
A_24_P401830	5.700532	5.3223495	NR_034182	chr1:31199527-31199586	MATN1-AS1
A_24_P295601	4.971138	4.277241	NM_014741	chr11:46695423-46695482	ATG13
A_23_P15564	10.440378	10.405651	NM_016627	chr17:66253042-66253101	AMZ2
A_32_P45738	13.655237	13.925062	NM_002629	chr10:99192856-99192915	PGAM1
A_24_P405960	5.049652	4.7585473	NM_018932	chr5:140590795-140590854	PCDHB12
A_33_P3290739	3.9952137	4.154383	D13071	chr14:22555155-22555214	
A_33_P3301509	3.4016511	2.3900566			
A_23_P257278	5.6239543	5.375851	NM_016082	chr20:31967370-31967311	CDK5RAP1
A_33_P3234347	9.6556225	9.359698	NM_001171689	chrX:109440277-109440218	AMMECR1
A_23_P79545	10.110758	10.190374	NM_003849	chr2:84658770-84658711	SUCLG1
A_32_P58074	14.637443	14.833921	NM_001006	chr4:152024086-152024145	RPS3A
A_33_P3287922	10.125074	10.220846	NM_014657	chr20:36611548-36611489	TTI1
A_33_P3237874	6.994342	7.3894043	NM_001100620	chr12:49717948-49718007	TROAP
A_24_P372012	3.7536967	3.1468031	NM_004968	chr7:8198223-8198164	ICA1
A_23_P202602	9.725694	9.919011	NM_007190	chr10:121700786-121700845	SEC23IP
A_23_P55073	9.664812	9.479867	NM_015462	chr17:65735657-65735716	NOL11
A_23_P139919	6.388875	5.6882505	NM_018413	chr12:105151518-105151577	CHST11
A_23_P46852	8.238406	7.990084	NM_024928	chr10:105657329-105651975	OBFC1
A_23_P210465	4.574846	4.7831593	NM_002638	chr20:43804645-43804704	PI3
A_23_P141394	7.2132945	7.156519	NM_017983	chr17:66417513-66417454	WIPI1
A_23_P41734	10.664523	10.723414	NM_018434	chr5:179382621-179382562	RNF130
A_24_P398500	6.405931	7.056165	NM_022782	chr12:123646722-123646663	MPHOSPH9
A_24_P307869	4.1579337	4.6717644	NM_001015002	chr17:73559153-73559212	LLGL2
A_32_P827528	5.8395424	6.1830826	NM_004230	chr19:10332293-10332234	S1PR2
A_33_P3400708	4.4186783	2.3900566	NM_001159642	chr1:151020016-151020075	BNIPL
A_33_P3627001	5.1953154	5.111722	NM_144962	chr8:22570843-22570784	PEBP4
A_33_P3330039	10.9675045	11.084861	NM_016274	chr1:150131744-150131803	PLEKHO1
A_33_P3210228	4.0093036	4.3132677		chr9:88959844-88959785	ZCCHC6
A_23_P387471	2.3221061	2.3900566	NM_005931	chr6:31478833-31478892	MICB
A_33_P3279720	5.5811143	5.67811		chr20:62332602-62332543	ARFRP1
A_33_P3230688	9.301757	9.44976	NM_052988	chr16:89762692-89762751	CDK10
A_24_P166407	13.431165	13.238498	NM_003544	chr6:26027208-26027149	HIST1H4B
A_24_P234732	5.601343	5.4067554	NM_006454	chr4:2249818-2249759	MXD4
A_23_P90333	6.407298	6.3776855	NM_001033719	chr19:44376876-44376817	ZNF404
A_33_P3292287	6.0328555	6.3714175	NR_022006	chr7:26575881-26575822	KIAA0087
A_23_P309619	6.159211	6.642375	NM_001145206	chr22:25592703-25592762	KIAA1671
A_23_P57379	10.23595	9.829986	NM_003504	chr22:19506385-19508015	CDC45
A_33_P3412722	6.662472	6.517278	NM_013321	chr7:2297121-2297062	SNX8
A_23_P165788	6.357239	6.2490115	NM_001008489	chr2:170558056-170558115	PHOSPHO2

A_23_P3193	8.478321	8.681072	NM_005113	chr14:93305973-93306032	GOLGA5
A_23_P77103	10.602913	9.752222	NM_003104	chr15:45366186-45366245	SORD
A_23_P37283	5.540573	5.40104	NM_006568	chr14:54997660-54997719	CGRRF1
A_24_P263956	8.521143	8.435082	NM_001136262	chr12:74934835-74934894	ATXN7L3B
A_23_P66402	6.9824533	6.4029093	NM_018019	chr17:17396235-17396294	MED9
A_23_P62647	3.6675878	3.4733872	NM_003037	chr1:160580059-160580000	SLAMF1
A_33_P3271711	4.2016134	3.7060003		chr3:004856040-004855981	
A_33_P3245858	4.6570654	4.875279	AK092544	chr8:12436559-12436618	LOC100131581
A_24_P237389	9.39014	8.675812	NM_001412	chrX:20143018-20142959	EIF1AX
A_24_P580248	3.2547474	3.2799208		chr1:120152040-120152099	XLOC_014512
A_23_P204564	6.543509	6.4014664	NM_002480	chr12:80168945-80168886	PPP1R12A
A_33_P3399618	6.6835785	7.007165	NM_001109997	chr14:20897205-20897146	KLHL33
A_23_P357104	9.593039	9.31986	NM_001155	chr5:150483193-150483134	ANXA6
A_24_P85775	4.197734	4.352827	NM_001039477	chr1:28212463-28212522	THEMIS2
A_33_P3345350	4.774296	4.4643526	NM_001276451	chr10:50574222-50574163	DRGX
A_24_P226962	5.8511114	5.7456594	NM_001080398	chr9:114123428-114123369	KIAA0368
A_24_P926960	4.50552	4.9283047	NM_001409	chr1:3405056-3404997	MEGF6
A_33_P3354404	5.8849883	5.7099257	NM_181684	chr21:46086571-46086512	KRTAP12-2
A_24_P221960	4.8897038	5.081925	AF130055	chr8:71486591-71486650	TRAM1
A_33_P3209706	4.6075706	4.389865	NM_001012659	chr3:121309243-121309302	ARGFX
A_32_P918263	8.519578	8.641283		chr7:055706201-055706142	
A_32_P31771	7.863728	8.107004	NM_030650	chr2:176794257-176794198	KIAA1715
A_23_P9362	13.017186	12.932745	NM_002799	chr9:127115950-127115891	PSMB7
A_33_P3399019	3.0421934	3.1778655	NM_001031665	chr19:53452784-53452725	ZNF816
A_23_P22382	10.073164	10.37703	NM_015527	chr16:30368496-30368437	TBC1D10B
A_23_P153945	8.20682	7.759228	NM_001006636	chr2:144703687-144703628	GTDC1
A_33_P3407925	10.07687	10.208823	NM_030938	chr17:57917858-57917917	VMP1
A_24_P48539	3.38095	4.0333548	NM_003830	chr19:52115603-52115544	SIGLEC5
A_33_P3220872	5.6898937	5.499357	NR_033423	chr2:83084187-83084246	LOC1720
A_33_P3256532	5.8343306	6.0314264	NM_032120	chr7:92166213-92166272	RBM48
A_23_P132438	6.728903	7.05105	NM_018133	chr3:135868701-135868642	MSL2
A_24_P157342	9.584815	9.904563	NM_018462	chr3:10157471-10167336	BRK1
A_23_P434352	11.383368	11.39393	NM_001042440	chr5:96108417-96108476	CAST
A_23_P125423	4.879006	5.190321	NM_001733	chr12:7187626-7187567	C1R
A_24_P303594	4.7581735	5.594349	NM_001040135	chr7:152520488-152520547	ACTR3B
A_23_P323898	4.347459	4.6846404	NR_028077	chr6:28347778-28347719	ZSCAN12
A_33_P3366082	9.577562	9.621923	NM_032565	chr13:50237253-50237194	EBPL
A_33_P3289705	7.1513953	7.3252916	NM_001256487	chr3:121412749-121412690	GOLGB1
A_23_P205200	5.6394234	5.6853642	NM_024705	chr13:52342137-52342091	DHRS12
A_24_P419087	3.1148362	2.3900566	NM_006576	chr12:58200287-58200228	AVIL
A_33_P3317442	4.533566	4.4960594	NM_024979	chr13:113743930-113743989	MCF2L
A_33_P3381966	5.6801615	5.360671	AK127597	chr21:44004792-44004733	LOC100129311
A_23_P347432	4.58068	3.9948423	NM_004421	chr1:1271579-1271520	DVL1
A_32_P489130	5.143611	5.312966	NM_153252	chrX:79931980-79931921	BRWD3
A_33_P3349521	10.948671	10.087898	NM_016127	chr8:29920785-29920726	TMEM66
A_33_P3409124	5.3421574	4.940919	NM_005126	chr3:24020698-24020757	NR1D2
A_33_P3289000	4.855401	4.776958		chr2:224634974-224634915	
A_33_P3292864	7.0787315	7.144097	AK090448	chr19:42401459-42401518	
A_32_P213831	3.8597176	4.153489	NM_020704	chr7:129122721-129122780	STRIP2
A_33_P3408757	3.8026693	3.425533	XM_002342102	chr1:41849198-41849257	FOXO6
A_23_P18078	5.191717	5.652219	NM_002888	chr3:158422689-158422630	RARRES1
A_23_P160631	12.837569	12.891449	NM_005998	chr1:156280437-156280378	CCT3
A_33_P3259662	3.4346895	4.548714	NM_003435	chr19:58132500-58132559	ZNF134
A_33_P3264424	6.621353	7.2191863		chr8:064100250-064100309	
A_32_P175349	5.0017734	5.0386653	NR_073178	chr17:76259455-76259396	LOC100996291
A_33_P3222744	5.627474	5.482414	NM_015852	chr7:64435041-64434982	ZNF117
A_24_P408704	2.3221061	2.3900566	NM_004946	chr5:169506070-169506129	DOCK2
A_23_P206760	4.9049406	4.6232796	NM_005143	chr16:72094877-72094936	HP
A_24_P281580	4.869569	5.2979803	NM_178831	chr7:99821578-99821519	GATS

A_33_P3216133	3.965751	3.6222463	NM_024645	chr8:40438744-40438685	ZMAT4
A_24_P3783	10.310839	10.353236	NM_003521	chr6:27783003-27783062	HIST1H2BM
A_33_P3632937	9.387093	9.4249115	XR_171880	chr11:880926-880867	LOC100131262
A_33_P3392927	3.593862	3.1418762	AB529253	chr11:56247036-56246977	
A_23_P318604	8.269793	8.283859	BC004544	chr8:145675046-145674987	CYHR1
A_24_P343255	5.694359	6.057248	NM_057161	chr6:42986844-42986903	KLHDC3
A_23_P43034	9.155949	9.294127	NM_018091	chr8:28047549-28047608	ELP3
A_33_P3261545	4.379977	4.396507	BC028053	chr1:16824838-16824779	
A_24_P382187	7.2828026	7.415511	NM_001552	chr17:38613308-38613367	IGFBP4
A_33_P3210363	5.9175844	6.0506587	NR_027157	chr12:98906810-98906751	TMPO-AS1
A_23_P170901	5.9119716	5.5990453	NM_152410	chr6:163510375-163510434	PACRG
A_33_P3226050	7.313191	8.0915785	NM_001037666	chr22:30681182-30681123	GATSL3
A_33_P3372257	5.0130663	4.2474904	NM_004272	chr5:78669935-78669876	HOMER1
A_33_P3271273	2.589103	3.0629776	NM_002145	chr17:46620956-46620897	HOXB2
A_24_P10233	4.3361483	4.0993257	NM_014326	chr15:64200425-64200366	DAPK2
A_23_P59045	8.0919075	8.169606	NM_021052	chr6:26217554-26217613	HIST1H2AE
A_24_P323545	7.1008434	7.0702577	NM_001077186	chr19:50783507-50783566	MYH14
A_23_P140434	3.0446954	3.1240466	NM_018728	chr15:52484703-52484644	MYO5C
A_23_P36753	10.188309	10.382055	NM_000690	chr12:112247448-112247507	ALDH2
A_24_P72064	5.77969	5.9397116	NM_000163	chr5:42721449-42721508	GHR
A_24_P303480	8.70266	8.388139	NM_006834	chr6:146875650-146875709	RAB32
A_23_P304991	7.6272893	7.012162	NM_000411	chr21:38123655-38123596	HLCS
A_33_P3296697	2.3221061	2.3900566		chr13:19620443-19620384	XLOC_014512
A_23_P333640	4.7029185	5.4843273	NM_173462	chr14:73741117-73741176	PAPLN
A_23_P86470	2.3221061	2.3900566	NM_003956	chr10:90965860-90965801	CH25H
A_23_P327156	2.6746278	2.3900566	NR_024334	chr14:65878097-65878038	FUT8-AS1
A_23_P258002	8.094536	8.093321	NM_017632	chr4:184368910-184368969	CDKN2AIP
A_23_P24723	6.894033	7.421149	NM_016464	chr11:61136153-61136212	TMEM138
A_23_P419202	7.437353	7.4027796	NM_033160	chr9:40772009-40771950	ZNF658
A_24_P149704	5.003235	4.674445	NM_138709	chr9:124543789-124544627	DAB2IP
A_23_P143569	7.8399415	7.896909	NM_033257	chr22:20302879-20302309	DGCR6L
A_24_P340800	3.960866	3.9781132	NM_198484	chr3:40573592-40573651	ZNF621
A_23_P500734	9.221506	9.341103	NM_015100	chr1:151375501-151375442	POGZ
A_24_P15502	9.034876	9.606231		chr8:064321806-064321865	
A_33_P3872301	4.502862	4.4353294	BF570763		SNAR-C3
A_33_P3298105	5.467161	5.361701	NM_001286792	chr13:24877285-24877344	SPATA13
A_23_P250156	10.181066	10.447179	NM_006548	chr3:185362106-185362047	IGF2BP2
A_23_P58280	11.436512	11.12475	NM_018983	chr4:110745600-110745659	GAR1
A_32_P56713	4.29932	4.318446	NM_004327	chr22:23659800-23659859	BCR
A_33_P3373259	5.33547	5.812622	NM_018398	chr3:54913050-54913109	CACNA2D3
A_23_P77145	7.6485343	8.328598	NM_004663	chr15:66181162-66181221	RAB11A
A_24_P230721	5.942141	5.8697453	NM_032856	chr15:85188836-85188777	WDR73
A_33_P3403643	6.629662	6.4981794	XM_003960862	chr11:530808-530867	LOC101059906
A_33_P3570228	6.8124423	7.018408	NR_077215	chr4:141562414-141562355	TNRC18P1
A_33_P3294377	5.8240104	5.8003325	DA880232	chr12:94288901-94288842	
A_24_P99216	5.405319	5.210128	NM_014045	chr14:23345985-23346165	LRP10
A_33_P3414267	4.6832	4.3760457	NM_001052	chr20:23016942-23017001	SSTR4
A_23_P69383	6.5199304	7.3977823	NM_031458	chr3:122247356-122247297	PARP9
A_33_P3330211	5.6380105	5.861654	NM_001127232	chr7:69364365-69364424	AUTS2
A_33_P3406873	8.5293255	8.4058275	NM_001005389	chr1:204944486-204944545	NFASC
A_33_P3334908	5.6353045	5.2844033	NM_145177	chrX:2137617-2137558	DHRSX
A_32_P191786	4.466801	4.827888	NM_133475	chr19:4224159-4224453	ANKRD24
A_33_P3742500	5.8027697	5.832194	BC132887	chr9:79809627-79809686	LOC642947
A_33_P3320548	8.360045	8.413672	NM_007342	chr7:23240317-23240376	NUPL2
A_24_P922357	6.658633	6.1344004	NM_173793	chr22:19431019-19430960	C22orf39
A_24_P27412	6.581442	6.9071007	NM_005701	chr15:75899643-75899584	SNUPN
A_33_P3211473	11.229525	11.2311325	NM_015024	chr8:21864036-21864095	XPO7
A_23_P315364	8.187849	8.293644	NM_002089	chr4:74963044-74962985	CXCL2
A_33_P3286293	6.81907	7.1339016	NM_020248	chr1:9910778-9910719	CTNNBIP1

A_23_P257945	8.628057	8.3173485	NM_005333	chrX:11139073-11139750	HCCS
A_23_P127522	8.3358	8.560414	NM_145014	chr11:125769904-125769963	HYLS1
A_24_P67681	8.282189	8.4927025		chr17:040800668-040800727	
A_23_P151870	6.753975	6.6540785	NM_015554	chr15:69564054-69564113	GLCE
A_24_P223604	10.926812	10.6908455	NM_015496	chr8:95500972-95500913	KIAA1429
A_32_P135517	4.2900314	3.6638668	AK123826	chr16:71497896-71497955	
A_23_P314101	4.9752965	4.4908767	NM_019601	chr22:24584978-24585037	SUSD2
A_33_P3288329	2.4453776	2.3900566	NM_001034954	chr10:97074875-97074816	SORBS1
A_33_P3224660	3.7635486	3.1558383	NM_000033	chrX:153008953-153009012	ABCD1
A_23_P214897	6.136686	6.2234874	NM_144497	chr6:151670006-151670065	AKAP12
A_24_P228302	3.662907	3.7170992	NM_006890	chr19:42178167-42178108	CEACAM7
A_33_P3356075	6.9577208	7.0774117	NM_001261429	chr2:11916259-11916318	LPIN1
A_32_P112881	11.001852	11.045132	NM_001256185	chrX:70279221-70279162	SNX12
A_24_P110062	7.079374	6.7701535	NM_175732	chr11:47594826-47594885	PTPMT1
A_23_P43580	6.0376387	6.4658656	NM_007018	chr9:123935686-123935745	CNTRL
A_23_P65442	7.2848344	7.9724927	NM_006084	chr14:24635500-24635559	IRF9
A_24_P383609	6.717104	5.1004696	NM_199461	chr10:120790867-120790926	NANOS1
A_24_P325520	8.126805	8.804487	NM_002959	chr1:109852492-109852433	SORT1
A_23_P142974	2.875301	2.3900566	NM_001007231	chr2:69053721-69053780	ARHGAP25
A_33_P3257554	6.6814537	6.8143554	NM_007018	chr9:123920261-123920317	CNTRL
A_23_P3514	8.888013	8.693789	NM_013242	chr16:58148719-58147881	C16orf80
A_33_P3323054	4.7469163	4.1823535		chr14:066424251-066424192	
A_24_P315500	4.2118773	4.0051947	NR_024567	chr1:41157533-41157474	LOC100130557
A_24_P59220	9.364508	9.379831	NM_001099771	chr2:130832768-130832709	POTEF
A_32_P98732	7.6360703	7.684674	NM_003643	chr6:52992086-52992027	GCM1
A_23_P23006	11.460392	11.636479	NM_002525	chr1:52255062-52255003	NRD1
A_23_P34744	7.2988186	7.1677513	NM_000396	chr1:150768912-150768853	CTSK
A_23_P141992	4.4001856	4.239183	NM_198706	chr19:5688184-5688243	HSD11B1L
A_33_P3232644	5.723182	5.4352555		chrX:012844296-012844355	
A_33_P3715177	7.3798	7.3673553	NM_001271938	chr19:42881553-42881612	MEGF8
A_33_P3403851	3.8293962	3.7103114		chr11:004497021-004497080	
A_23_P23221	9.056651	8.782967	NM_001924	chr1:68153371-68153430	GADD45A
A_23_P343411	8.727526	8.511744	NM_198576	chr1:991186-991245	AGRN
A_24_P181677	5.266422	5.423615	NM_025078	chr18:77663306-77663247	PQLC1
A_33_P3261031	5.4086924	6.122469		chr11:000125293-000125352	
A_23_P78557	6.402557	6.8333063	NM_017703	chr19:9921477-9921418	FBXL12
A_33_P3217073	11.91863	11.4216175	NM_014394	chr10:85912750-85912809	GHITM
A_33_P3331646	4.0189123	3.5520515	XR_243602	chr17:18855150-18855091	
A_32_P137399	5.503987	5.719327	NR_027123	chr14:56043983-56043924	KTN1-AS1
A_23_P61042	4.325273	4.630637	S55735	chr14:106053930-106053649	
A_24_P98086	10.310577	10.518592	NM_007353	chr7:2768442-2768383	GNA12
A_24_P512054	4.4204073	5.1189184	NM_024045	chr10:70693994-70694054	DDX50
A_33_P3308749	2.3221061	2.3900566	NM_001105207	chr6:112537629-112537570	LAMA4
A_33_P3851346	6.0939283	5.900701	NR_027074	chr15:90048243-90048184	LINC00928
A_23_P170820	10.964012	11.16176	NM_001020658	chr1:31405766-31405707	PUM1
A_23_P91814	7.8956575	7.794285	NM_017875	chr3:39438140-39438199	SLC25A38
A_23_P61778	5.6563096	5.605489	NM_022463	chr17:703653-703594	NXN
A_23_P215037	9.94237	9.849198	NM_152858	chr6:160170023-160170082	WTAP
A_32_P34003	8.638604	8.476421		chr2:164459505-164459446	FIGN
A_23_P144348	6.375584	6.3197737	NM_004787	chr4:20620432-20620491	SLIT2
A_23_P97096	4.6425405	4.821108	NM_014359	chr1:203467900-203467959	OPTC
A_24_P71373	3.8881896	4.5138407	NM_003047	chr1:27426044-27425985	SLC9A1
A_23_P81859	9.28651	9.269537	NM_080596	chr6:27115246-27115305	HIST1H2AH
A_24_P170763	7.2599726	7.7599173	NM_015299	chr14:24910286-24910343	KHNYN
A_23_P137427	11.021915	10.704978	NM_005857	chr1:40759577-40759636	ZMPSTE24
A_23_P92261	8.930357	9.045843	NM_032331	chr3:183976423-183976482	ECE2
A_24_P217234	6.8363543	7.6349745	NM_000341	chr2:44547688-44547747	SLC3A1
A_33_P3759592	15.664499	15.664499	AK023835	chr17:18095126-18095185	FLJ13773
A_23_P438	3.7565856	2.9797847	NM_017773	chr1:203744742-203744801	LAX1

A_23_P69738	3.6286232	2.3900566	NM_023940	chr4:53732842-53732902	RASL11B
A_23_P207125	7.329007	7.311602	NM_020795	chr17:7322736-7322795	NLGN2
A_33_P3393821	5.2904744	5.406931	NM_001733	chr12:7187895-7187836	C1R
A_33_P3423740	4.937725	5.008777	NM_032227	chrX:109352310-109352369	TMEM164
A_24_P217365	8.326876	7.661851	NM_015199	chr3:15708901-15708842	ANKRD28
A_23_P320658	10.218839	10.00099	NM_004725	chr10:124924177-124924236	BUB3
A_33_P3334047	6.25922	6.2494793		chr10:046223306-046223365	
A_24_P316257	5.081925	5.119728	NM_176677	chr16:619159-619218	NHLRC4
A_23_P74349	10.065493	10.1725235	NM_145697	chr1:163325144-163325203	NUF2
A_32_P161033	2.3221061	2.3900566		chr1:143403643-143403702	
A_33_P3249414	7.873255	7.166096	NM_015431	chr1:248043359-248043418	TRIM58
A_23_P132763	2.3221061	2.3900566	NM_016206	chr3:86987218-86987159	VGLL3
A_33_P3304172	6.1392803	5.7633533	AK092533	chr11:1012878-1012821	MUC6
A_23_P127279	9.344556	9.758781	NM_019054	chr10:88950741-88950800	FAM35A
A_23_P413180	6.0825458	6.075567	NM_017440	chr12:68688887-68688828	MDM1
A_23_P409386	10.851663	10.080616	NM_024698	chr11:790555-790496	SLC25A22
A_24_P189533	4.4440975	3.6298978	NM_015036	chr11:94865312-94865371	ENDOD1
A_23_P130531	7.5920215	7.4573317	NM_007065	chr19:10505963-10505904	CDC37
A_23_P207106	6.3250265	6.522181	NM_000747	chr17:7360295-7360354	CHRNB1
A_33_P3216945	3.8163652	4.305903		chr7:153756582-153756523	
A_33_P3215232	4.2440705	4.073944		chr15:102514652-102514593	
A_33_P3286318	13.859872	13.877535	BC011771	chr11:64941728-64941669	
A_23_P42768	6.821227	6.8050785	NM_001110199	chr7:75890675-75890731	SRRM3
A_33_P3260605	12.1429	11.742368	NM_003798	chr9:111754973-111754914	CTNNAL1
A_24_P322611	6.4552407	6.486571	NM_022105	chr20:61541145-61541086	DIDO1
A_23_P389907	9.718708	9.668517	NM_002973	chr12:111890389-111890330	ATXN2
A_33_P3214635	8.548798	7.1490088	NM_001012515	chr18:55215629-55215570	FECH
A_33_P3422659	6.1369133	5.7896137	NM_018972	chr8:75279274-75279333	GDAP1
A_33_P3381771	8.712727	8.658955	NM_024052	chr17:17971635-17971694	GID4
A_23_P83751	5.60605	5.768587	NM_024806	chr11:122830122-122830181	C11orf63
A_33_P3212989	3.803113	3.3528128	NR_015448	chr7:96597886-96597827	DLX6-AS1
A_23_P165239	5.3898025	5.687784	NM_007153	chr19:22154324-22154265	ZNF208
A_23_P13873	7.567213	7.2699575	NM_005439	chr12:6857546-6857487	MLF2
A_23_P166698	9.388833	9.14502	NM_016305	chr3:42636316-42636375	SS18L2
A_23_P112798	7.3786974	6.900267	NM_001312	chr14:105945806-105945957	CRIP2
A_33_P3316248	5.3630743	4.4142246		chrX:000515186-000515127	
A_24_P288954	7.031374	7.1423783	NM_001013694	chr22:26886072-26886131	SRRD
A_33_P3419875	3.8929088	3.4826937		chr9:014824279-014824220	
A_24_P367439	3.7724066	3.0945325	AF068294	chr9:170276-170335	
A_23_P132139	5.7541347	5.9478407	NM_001286462	chr21:47731417-47722479	C21orf58
A_33_P3305790	5.8069324	6.1655846	NM_000603	chr7:150711495-150711554	NOS3
A_24_P335620	12.254968	11.595769	NM_003486	chr16:87863966-87863907	SLC7A5
A_23_P413634	5.494195	5.7622967	NM_024620	chr19:58637975-58637916	ZNF329
A_24_P224116	4.753353	4.8831086	NM_000928	chr12:120760012-120759953	PLA2G1B
A_33_P3337415	8.62549	8.622639	NM_182978	chr18:11882362-11882421	GNAL
A_23_P133770	9.952119	9.950915	NM_138493	chr6:37452608-37451029	CCDC167
A_33_P3219785	7.9988437	7.658223	NM_023016	chr2:110376464-110376523	SOWAHC
A_33_P3357858	2.4734197	2.3900566		chr7:5894007-5894066	ZNF815P
A_33_P3340085	7.0065827	7.1322246	NM_178136	chr22:42998859-42998800	POLDIP3
A_33_P3354434	9.545637	9.369582	NM_033549	chr5:180662748-180662807	TRIM41
A_32_P890614	3.4341893	3.0276055	NR_026682	chr5:172381849-172381790	LOC100268168
A_23_P40078	10.387767	10.481866	NM_003400	chr2:61705895-61705836	XPO1
A_33_P3391290	5.2223177	5.507352	NM_001142483	chr5:111066427-111066368	NREP
A_23_P422511	5.0467806	5.120269	NM_182556	chr11:65143061-65143002	SLC25A45
A_33_P3339741	3.7381186	3.3373988			
A_23_P136671	9.2353325	9.298204	NM_001074	chr4:69978384-69978443	UGT2B7
A_23_P170581	5.372482	4.9096823	XM_005256291	chr16:89301901-89301960	ZNF778
A_33_P3359268	11.099552	11.590412	NM_006339	chr19:3579020-3579079	HMG20B
A_23_P431252	2.4800916	2.3900566	NM_032505	chr3:67061173-67061232	KBTBD8

A_23_P22350	6.5584974	7.2015495	NM_023927	chr5:125829184-125829243	GRAMD3
A_23_P139929	9.391392	9.692306	NM_006817	chr12:112460260-112460319	ERP29
A_33_P3368301	9.198666	9.218336		chr2:74362565-74362528	BOLA3
A_23_P500773	9.426449	9.709972	NM_003188	chr6:91225746-91225687	MAP3K7
A_33_P3407638	7.835964	7.764095	NM_152316	chr11:30359106-30359165	ARL14EP
A_33_P3228102	4.65304	5.0140977	NM_153240	chr3:132440917-132440858	NPHP3
A_32_P115130	12.674974	12.644803	NM_032477	chr9:140446838-140446897	MRPL41
A_23_P359738	7.9930773	8.065063	NM_015630	chr2:149544919-149544978	EPC2
A_23_P163481	9.232655	9.350999	NM_001211	chr15:40512828-40512887	BUB1B
A_24_P3461	9.96834	10.544297	BC172311	chr4:174537399-174537340	MORF4
A_24_P383999	14.519965	14.411888	NM_001006	chr4:152024088-152024146	RPS3A
A_33_P3340666	7.7748566	7.8433633	NM_181611	chr21:31874408-31874349	KRTAP19-5
A_32_P133916	4.7616863	3.3455348	NM_017637	chr9:16409646-16409587	BNC2
A_23_P112241	7.204469	7.5589304	NM_012266	chr9:34998281-34998340	DNAJB5
A_33_P3418716	4.464582	4.68926	NM_025202	chr2:233546958-233547017	EFHD1
A_23_P218476	7.3578506	7.4290743	NM_030578	chr19:41860457-41860398	B9D2
A_33_P3346102	5.8942757	6.1419735	NM_016281	chr12:118587819-118587760	TAOK3
A_23_P12992	12.960289	12.855014	NM_016404	chr11:64084293-64084234	TRMT112
A_33_P3233150	7.623539	8.226385	NM_023072	chr19:13942978-13943037	ZSWIM4
A_23_P27795	3.3886504	2.3900566	NM_021102	chr19:38782859-38782918	SPINT2
A_23_P372334	9.32161	9.245571	NM_144716	chr3:46963448-46963389	CCDC12
A_23_P111978	4.0781546	4.1625237	NM_001282534	chr8:140631179-140631120	KCNK9
A_33_P3410836	8.089643	7.878476	NM_003539	chr6:26189060-26189001	HIST1H4D
A_23_P53588	6.821924	6.7125983	NM_030775	chr12:1755801-1755860	WNT5B
A_23_P390384	5.5675597	5.247188	NM_001037735	chrX:47917665-47917606	ZNF630
A_24_P31275	5.2550597	5.290777	NM_001678	chr17:7560632-7560691	ATP1B2
A_23_P143484	8.486318	8.549156	NM_153681	chr21:38437919-38437860	PIGP
A_23_P3450	6.962084	7.128351	NM_014444	chr15:43696688-43696747	TUBGCP4
A_23_P27744	9.043018	9.177886	NM_001080469	chr19:46214072-46214013	FBXO46
A_24_P113144	6.015543	6.0102606	NM_024857	chr17:29221595-29221654	ATAD5
A_33_P3335940	5.87568	5.8412285	NM_144570	chr16:1741921-1741980	HN1L
A_24_P224776	8.71396	8.720771	NM_012321	chr19:18423452-18420651	LSM4
A_24_P298587	4.897825	5.3536854	NM_001280561	chr8:145577181-145577122	TMEM249
A_33_P3328450	3.6810007	4.1604986	NM_018942	chr4:8869066-8869007	HMX1
A_24_P151582	5.036498	5.4576774	NM_003216	chr22:41794580-41794639	TEF
A_23_P14473	11.341736	11.376524	NM_005776	chr14:54893796-54893737	CNIH1
A_23_P352291	9.332237	9.351712	NM_054016	chr1:24296306-24296247	SRSF10
A_33_P3423202	4.212503	3.6543355		chrY:027634044-027633985	
A_32_P142779	3.579877	3.875741	NM_175882	chr17:43924003-43924062	SPPL2C
A_23_P251173	7.9602785	8.434881	NM_014413	chr7:6063792-6063733	EIF2AK1
A_33_P3389342	4.2319474	4.1573315	NM_212481	chr2:97216361-97216420	ARID5A
A_24_P262355	9.324396	9.305081	NM_002634	chr17:47489150-47489091	PHB
A_23_P306507	6.7773056	7.0923405	NM_033360	chr12:25359211-25359152	KRAS
A_23_P51548	11.065624	10.765096	NM_004528	chr1:165624688-165624747	MGST3
A_23_P26072	4.7180285	4.630957	AF118084	chr15:35271392-35271451	
A_23_P141315	9.068874	8.677659	NM_001014445	chr17:33459260-33459201	NLE1
A_23_P88489	5.6175575	5.1961336	NM_172095	chr15:43924397-43922872	CATSPER2
A_33_P3362737	6.6414237	6.7273436	AK124695	chr15:43011454-43011395	
A_24_P107859	7.674095	7.566843	NM_152594	chr15:38645760-38645819	SPRED1
A_32_P328023	4.626305	4.7157893	NM_018383	chr2:128462093-128462034	WDR33
A_32_P225854	4.9419937	5.0252223	NM_181784	chr2:65538910-65538851	SPRED2
A_24_P349636	15.087955	15.066905		chr17:049580097-049580038	
A_23_P15727	8.6710615	9.028399	NM_021939	chr17:39979229-39979291	FKBP10
A_24_P194503	3.6341422	4.164031	NM_002637	chrX:71799404-71799345	PHKA1
A_33_P3386132	7.27096	7.294896	NM_024093	chr2:105961992-105962051	C2orf49
A_33_P3254013	4.5506067	4.4877853	AK131464	chr4:845421-845362	GAK
A_33_P3901921	5.541316	5.5509944	NM_017915	chr12:102542017-102542076	PARPBP
A_24_P410408	6.1012363	5.4170313	NM_002282	chr12:52710736-52710677	KRT83
A_33_P3345126	4.819787	3.7276106	NM_198539	chr19:37442280-37442339	ZNF568

A_33_P3272437	4.6009493	4.3726587		chrY:24587185-24587244	XLOC_008300
A_23_P89327	3.8482335	3.3553553	NM_020652	chr17:15620700-15620759	ZNF286A
A_32_P155776	13.0325985	13.036818	AY014272	chr2:132384555-132384614	POTEKP
A_24_P769359	8.650884	8.805335	NM_001184985	chr12:1020048-1020107	WNK1
A_33_P3358893	5.3027515	5.150953	NM_001286359	chr16:11369552-11369493	PRM2
A_33_P3252181	7.0195403	6.969096	AK097351	chr19:49831563-49831504	
A_24_P389517	10.078162	10.230425	NM_031262	chr9:86585700-86585236	HNRNPK
A_33_P3288774	6.232185	6.1946673	NM_172071	chr1:173907896-173907837	RC3H1
A_23_P75790	7.4238477	7.514962	NM_013279	chr11:61555786-61555845	MYRF
A_33_P3418766	8.213221	6.8872147	NM_018181	chr18:56653609-56653668	ZNF532
A_23_P148629	11.981831	11.829773	NM_004681	chrY:22749984-22751421	EIF1AY
A_24_P206604	4.9383335	4.3779626	NM_004566	chr10:6266166-6268203	PFKFB3
A_33_P3363100	6.8379507	7.0586243		chr6:029942911-029942852	
A_33_P3308299	3.7590268	3.7877889		chr7:55655866-55655925	
A_24_P914817	7.2658186	7.279097	NM_013255	chr7:131180695-131180754	MKLN1
A_23_P17345	3.4182153	2.3900566	NM_005461	chr20:39314869-39314810	MAFB
A_33_P3297399	5.7349076	5.8444524	NM_017634	chr8:25296831-25293979	KCTD9
A_23_P144071	6.2692623	6.0443254	NM_000094	chr3:48602588-48602401	COL7A1
A_23_P258321	10.070319	9.769889	NM_015969	chr7:56022855-56022914	MRPS17
A_23_P204395	7.9473624	7.989664	NM_023928	chr12:125627381-125627440	AACS
A_23_P85969	9.556107	9.468208	NM_182976	chr1:90493706-90493765	ZNF326
A_32_P793319	4.368073	4.720623	AK098749	chr17:32397764-32397705	
A_24_P675386	6.4517684	5.979962	NM_014267	chr11:16779256-16779315	C11orf58
A_23_P353742	8.301429	8.391472	NM_017749	chr11:46418157-46418098	AMBRA1
A_23_P129829	8.795741	9.026676	NM_139280	chr17:38077558-38077499	ORMDL3
A_33_P3237364	4.7689443	4.575894		chr3:041997231-041997290	
A_33_P3414591	4.6900434	4.4939284	NM_000474	chr7:19156521-19156462	TWIST1
A_24_P239017	8.260393	7.921773	NR_002837	chr16:34404117-34404058	UBE2MP1
A_32_P163533	6.897473	6.8645773	NM_001242797	chr6:26637056-26636997	ZNF322
A_24_P120970	6.7469897	6.3185444	NM_032937	chr9:140510538-140510479	C9orf37
A_33_P3285987	8.332668	8.130319	NM_152636	chr11:28354711-28354770	METTL15
A_33_P3423721	10.0146	10.715145	NM_001271605	chr16:87637922-87637981	JPH3
A_23_P86660	8.509039	8.436866	NM_194247	chr2:178085706-178085765	HNRNPA3
A_23_P10194	7.2458653	7.627781	NM_201575	chr16:29882918-29882859	SEZ6L2
A_23_P315910	4.2890553	2.9406095	NM_145653	chr18:44554926-44554867	TCEB3C
A_24_P96403	6.9479547	6.6974545	NM_001001890	chr21:36160450-36160391	RUNX1
A_23_P148297	9.07535	9.626364	NM_003022	chrX:80553542-80553601	SH3BGRL
A_33_P3395513	5.057622	4.6538334	AK124504	chr5:139230918-139230859	NRG2
A_23_P319895	5.8090053	5.7085466	NM_015048	chr12:122269954-122270013	SETD1B
A_23_P9662	7.0293083	7.218239	NM_005897	chr1:46164774-46164715	IPP
A_33_P3307486	4.8791866	4.8645525	NM_001163547	chr17:35945511-35945452	SYNRG
A_23_P57961	4.6931086	5.5178995	NM_002673	chr3:48450737-48448440	PLXNB1
A_24_P322847	10.003486	9.36001	NM_001018050	chr22:41921926-41921867	POLR3H
A_33_P3382919	5.565601	5.2691207	XR_244127	chr20:61999544-61999603	
A_33_P3531373	3.8980637	3.4609866	XR_252283	chr7:106423149-106423208	
A_33_P3238390	8.787651	8.660514		chr4:110474554-110474495	
A_33_P3239736	5.4112873	5.4458346	NM_019045	chrX:117577590-117577649	WDR44
A_23_P49865	5.511552	5.4830704	NM_015982	chr17:7191649-7191590	YBX2
A_33_P3302025	3.585319	2.3900566	NM_052866	chr9:18622254-18622313	ADAMTSL1
A_23_P70371	9.691617	9.691617	NM_015153	chr6:64423669-64423728	PHF3
A_24_P371053	6.030559	5.8562565	NM_016467	chr2:190635255-190635196	ORMDL1
A_33_P3419321	4.55682	4.234759	NM_015557	chr1:6181282-6181223	CHD5
A_33_P3363254	4.3914757	3.8480606		chr2:131449816-131449757	
A_24_P387609	6.82226	7.127641	NM_030940	chr9:88880440-88880381	ISCA1
A_33_P3230709	11.689829	11.764043	NM_006612	chr17:4927936-4927995	KIF1C
A_33_P3417459	6.058975	5.9331727	NR_023358	chrX:20154363-20154304	SCARNA9L
A_23_P7221	14.646114	14.648371	NM_033625	chr4:109546305-109546364	RPL34
A_23_P13382	6.3926897	5.6760745	NM_001013254	chr11:1913364-1913423	LSP1
A_33_P3379947	12.46171	12.832551	NM_005514	chr6:31321712-31321653	HLA-B

A_23_P62115	9.448561	9.157548	NM_003254	chrX:47444666-47444725	TIMP1
A_33_P3263747	6.6346817	6.807865			
A_24_P192301	2.4906206	2.3900566	NM_006080	chr7:83588248-83588189	SEMA3A
A_23_P388168	7.569879	7.8795958	NM_002867	chr1:52385367-52385308	RAB3B
A_33_P3360665	4.5325503	5.2406187	NM_001105	chr2:158594035-158593976	ACVR1
A_23_P27894	8.473176	8.676395	NM_014649	chr19:5587199-5587140	SAFB2
A_24_P91165	3.5881126	3.618431	NM_199248	chr17:37334331-37334272	CACNB1
A_23_P96976	10.083971	9.883702	NM_016505	chr1:31821677-31821736	ZCCHC17
A_33_P3267740	6.331382	6.5873065	NM_178544	chr19:40521518-40521577	ZNF546
A_33_P3313889	9.944344	9.750144	NM_000113	chr9:132575281-132575222	TOR1A
A_33_P3219303	4.8142533	4.8677993	NM_001807	chr9:135947092-135947151	CEL
A_33_P3289860	9.169292	9.533114	BX648603	chr18:48087024-48087083	
A_33_P3307447	12.061581	12.149535	AX721251	chr3:129115233-129115174	RPL32P3
A_33_P3272075	3.7700546	3.8869314	NM_000911	chr1:29190144-29190203	OPRD1
A_23_P360804	4.9536033	4.1581826	NM_020939	chr6:36708629-36708570	CPNE5
A_33_P3301306	8.19161	8.156484	NM_170606	chr7:151832089-151832030	KMT2C
A_23_P393727	5.0636454	4.2448616	NM_201266	chr2:206659703-206659762	NRP2
A_24_P249253	4.0514874	3.6212702	NM_207163	chr7:123303161-123303220	LMOD2
A_33_P3279371	4.422182	4.629771		chr21:36109412-36109471	XLOC_013927
A_24_P752208	8.04772	7.843071		chr15:035590774-035590715	
A_23_P89543	7.933358	7.914815	NM_014804	chr17:6481972-6481913	KIAA0753
A_33_P3323481	6.449467	6.852153	NM_001199662	chr1:156212556-156212615	PMF1-BGLAP
A_23_P154025	11.578044	11.512401	NM_003142	chr2:170668314-170668373	SSB
A_23_P216894	5.075404	5.150304	NM_001006617	chr9:128200623-128200564	MAPKAP1
A_33_P3333107	5.6157646	5.5527735		chr16:85613722-85613663	
A_23_P37144	7.6050205	7.5106153	NM_080664	chr14:31915237-31915227	DTD2
A_24_P338187	8.083288	8.478321	NM_012207	chr10:70101030-70101364	HNRNPH3
A_24_P252780	5.7739253	6.007289	NM_198514	chr10:115664617-115664676	NHLRC2
A_23_P366682	7.238969	7.0293083	NM_033513	chr19:519055-519114	TPGS1
A_33_P3382423	6.1513586	5.849459	NM_182498	chr19:44111451-44111392	ZNF428
A_33_P3258141	9.683909	9.683909	NM_001278	chr10:101948251-101948192	CHUK
A_33_P3414903	12.408525	12.400431	BC034966	chr1:182778360-182778419	NPL
A_24_P73370	7.2437177	7.6262865	NM_003565	chr12:132407342-132407401	ULK1
A_33_P3323678	7.2223506	6.658633		chr12:064732126-064732067	
A_33_P3227231	3.8092356	2.8850462	AK303492	chr7:148990798-148990857	ZNF783
A_23_P89780	8.521772	8.572544	NM_198129	chr18:21534735-21534794	LAMA3
A_33_P3249589	2.991559	2.3900566	NM_022893	chr2:60687608-60687549	BCL11A
A_23_P209933	8.165663	7.8124027	NM_006464	chr2:85553659-85552091	TGOLN2
A_33_P3479066	4.03307	4.2625504		chr12:006993392-006993333	
A_24_P235429	9.195093	9.465654	NM_005502	chr9:107543763-107543704	ABCA1
A_23_P153360	8.140608	8.16521	NM_024104	chr19:16757313-16757254	SMIM7
A_23_P135164	6.186641	6.4850745	NM_207309	chr9:139978559-139978618	UAP1L1
A_23_P418234	7.6721125	8.311636	NM_015020	chr16:71679219-71679160	PHLPP2
A_33_P3283400	8.748145	8.838591	XM_005275744		LOC100507747
A_23_P23155	6.618429	6.262122	NM_018836	chr1:4842908-4842967	AJAP1
A_33_P3328937	7.090821	7.2398353	NM_001284363	chr17:60599568-60599627	TLK2
A_33_P3346292	4.2801642	3.7209752	AY358682	chr9:33935617-33935558	UBAP2
A_23_P82351	6.700514	6.718018	NM_198428	chr7:33645021-33645080	BBS9
A_33_P3385983	6.0576982	6.3063374	NM_021143	chr19:12243572-12243513	ZNF20
A_24_P283288	5.702439	5.8240104	NM_139013	chr6:36041853-36043663	MAPK14
A_33_P3214012	3.8910928	4.433193	NM_019036	chr6:55360319-55360260	HMGCLL1
A_33_P3339990	4.4838047	4.335672	AK123323	chr19:19571111-19571052	LOC100128429
A_23_P414252	6.8648624	6.8607974	AK315670	chr7:2297009-2296609	SNX8
A_33_P3340040	9.736561	9.314642	NM_032336	chr8:41402042-41402101	GINS4
A_33_P3368139	10.488071	10.453802	NM_005921	chr5:56191750-56191809	MAP3K1
A_33_P3326387	3.9410715	3.7971418	NM_173463	chr4:24854754-24854695	CCDC149
A_32_P162374	8.0630865	7.717366	NM_173529	chr18:51907485-51907544	C18orf54
A_33_P3773261	5.543345	5.279377	XR_249926	chr6:3027560-3027619	LOC101927759
A_24_P254084	5.4843273	5.422681	NM_021915	chr19:12024601-12024660	ZNF69

A_23_P216448	10.497971	10.165661	NM_005596	chr9:14088270-14088211	NFIB
A_24_P754803	15.049419	15.102499	NM_006013	chrX:153628866-153628925	RPL10
A_33_P3327076	3.8960338	3.751614	NM_001001824	chr1:248813291-248813232	OR2T27
A_33_P3313030	4.8184643	4.9324436	NM_024715	chr5:134237214-134237273	TXNDC15
A_33_P3367481	5.5436916	5.291143	NM_004491	chr19:47507274-47507333	ARHGAP35
A_23_P416813	2.3934417	3.2731733	NM_133466	chr19:36883571-36883512	ZFP82
A_33_P3256355	7.1108794	7.295623			
A_24_P104512	8.708113	8.823239	NM_001988	chr17:74003067-74003008	EVPL
A_23_P82478	8.780723	8.315938	NM_019042	chr7:105097161-105097102	PUS7
A_23_P162766	5.979962	6.4567103	NM_015296	chr13:99446650-99446591	DOCK9
A_24_P878388	12.670279	11.816893		chrX:006907761-006907820	
A_23_P314789	4.714142	4.5472045	NM_001039617	chr3:195937533-195936374	ZDHHC19
A_24_P217834	9.959128	9.716636	NM_003530	chr6:26197317-26197258	HIST1H3D
A_23_P143713	5.7555637	5.3923335	NM_021822	chr22:39477481-39477540	APOBEC3G
A_23_P332908	9.844624	9.475201	NM_144982	chr12:72050740-72050681	ZFC3H1
A_24_P261567	4.512703	4.1509485	NM_030792	chr11:75147988-75146649	GDPD5
A_33_P3629151	4.5736713	4.3945856	AI150443	chr17:15008778-15008837	CDRT8
A_23_P26713	14.767965	14.716869	NM_000978	chr17:37006423-37006364	RPL23
A_33_P3384052	4.612163	4.9544587	NM_013435	chr18:56934671-56934612	RAX
A_33_P3396891	7.961073	8.113338	NM_021732	chr10:99437521-99437462	AVPI1
A_23_P165355	8.353823	8.780476	NM_005687	chr2:223489061-223489002	FARSB
A_23_P135966	7.7928414	7.8272705	NM_001282197	chrX:129507180-129507239	SLC25A14
A_32_P24585	6.043813	5.8331738	NM_001017995	chr5:171760845-171760786	SH3PXD2B
A_23_P117933	11.948728	12.00715	NM_004483	chr16:81116042-81115983	GCSH
A_33_P3357470	5.5384865	5.782507	NM_004726	chrX:17151970-17152029	REPS2
A_23_P203586	8.908999	8.951029	NM_016320	chr11:3696806-3696747	NUP98
A_23_P85180	6.7815537	6.5920258	NM_003492	chrX:153248552-153248611	TMEM187
A_33_P3394978	5.6010923	5.5820394	NR_027276	chr11:133902477-133902536	LOC100128239
A_33_P3351999	4.598206	3.4607034		chr14:052043659-052043718	
A_33_P3249509	9.299998	9.196998	NM_205843	chr19:3456573-3456632	NFIC
A_23_P59613	3.9879653	3.628978	NM_003508	chr7:72850343-72850402	FZD9
A_23_P205841	8.683968	8.445577	NM_006901	chr15:72118519-72118460	MYO9A
A_23_P217135	8.396287	8.176613	NM_001080485	chrX:152616825-152616884	ZNF275
A_33_P3322236	5.8816276	5.837058	AK125217	chr3:195415321-195415380	LINC00969
A_23_P406424	9.278418	9.743299	NM_175744	chr1:113245313-113245254	RHOC
A_23_P27180	7.8127027	7.8090315	NM_032141	chr17:28512741-28512800	NSRP1
A_33_P3571824	4.2395954	3.6327348	NM_004829	chr19:55424120-55424179	NCR1
A_23_P319270	8.720771	8.78143	NM_014984	chr17:79163456-79163397	AZI1
A_33_P3238335	8.433338	8.447224	BC060765	chr1:85490292-85490233	MCOLN3
A_23_P7423	10.649348	10.56963	NM_017755	chr5:6599588-6599529	NSUN2
A_33_P3272508	10.137086	10.110758	NM_024026	chr13:21753075-21753134	MRP63
A_24_P419211	9.5077715	9.556107	NM_004685	chr13:25822113-25822054	MTMR6
A_23_P386241	8.734075	8.924311	NM_001042353	chr20:826839-826898	FAM110A
A_33_P3261625	12.282789	12.0726595	NM_021177	chr6:31765235-31765176	LSM2
A_23_P215132	9.42388	9.401131	NM_014149	chr7:134870624-134870565	WDR91
A_24_P110914	5.9895625	5.9213934	NR_024458	chr13:45965526-45965585	TPT1-AS1
A_24_P243749	5.0852904	5.4928207	NM_002612	chr7:95213366-95213307	PDK4
A_23_P324718	6.5414033	6.5550966	NM_203446	chr21:34001886-34001827	SYNJ1
A_33_P3390357	8.174881	7.957752	NM_001128626	chr18:12446604-12446545	SPIRE1
A_33_P3347622	5.2567596	4.7758007	NM_001244767	chr4:71768207-71768266	MOB1B
A_33_P3383762	4.6651964	3.9998786		chr8:067873589-067873530	
A_33_P3335935	6.4307175	5.885212	NM_144570	chr16:1749826-1749885	HN1L
A_23_P17695	5.9505672	5.0350046	NM_018964	chr21:44001065-44001123	SLC37A1
A_24_P247074	2.7391882	2.3900566		chr10:008556476-008556417	
A_33_P3233170	8.938311	8.717649	AK074630	chr8:27205084-27205143	
A_23_P6119	6.9571514	6.9506598	NM_032985	chr20:18526643-18529277	SEC23B
A_23_P65455	7.007976	7.07815	NM_017815	chr14:23421594-23420897	HAUS4
A_33_P3589819	7.6024885	7.8091354	AK026502	chr22:27117555-27117614	LOC100507637
A_33_P3358158	13.002138	12.914449		chr22:25857114-25857173	CRYBB2P1

A_23_P136116	2.3221061	2.3900566	NM_001004320	chr7:15405225-15405166	AGMO
A_33_P3248967	10.030073	10.041927	NM_022474	chr14:67802437-67802496	MPP5
A_24_P573533	8.172868	8.132425	NM_001024916	chr9:70432360-70432301	CBWD5
A_23_P213592	7.3197293	8.045635	NM_014901	chr5:175954250-175954191	RNF44
A_24_P221485	7.890714	7.4295206		chr1:79520790-79520731	XLOC_014512
A_33_P3495962	7.127136	7.2002172	BM918074		SNORA71A
A_33_P3416622	5.979553	6.248001	AK131425	chr21:44748637-44748578	LINC00322
A_33_P3286937	4.4114265	3.8317053	NM_001166163	chr7:94897959-94898018	PPP1R9A
A_24_P46577	9.970684	9.738386	NM_170783	chr6:30030067-30032465	ZNRD1
A_33_P3418511	4.2101736	4.2421136	NR_027756	chr16:73127613-73127672	HCCAT5
A_32_P192970	5.449656	5.817236	NM_170726	chr1:19209897-19209838	ALDH4A1
A_23_P216708	9.843943	8.827332	NM_018339	chr9:79000894-79000835	RFK
A_23_P88351	3.20401	2.3900566	NM_181598	chr14:51095050-51095109	ATL1
A_32_P169131	7.8974786	7.933044	NM_019109	chr16:5135454-5135512	ALG1
A_33_P3226755	5.161072	5.3421574	AK125630	chrX:153601811-153601752	FLNA
A_23_P368484	5.950123	5.69911	NM_207387	chr17:16345428-16345369	FAM211A
A_33_P3245952	4.9306517	5.148839	AK130915	chr9:16786970-16786911	LOC648570
A_24_P151032	5.1391253	5.2633705	NM_002476	chr17:45286758-45286817	MYL4
A_24_P98263	8.428577	8.352757	NM_004215	chr8:110569230-110573084	EBAG9
A_23_P251259	5.5109024	4.7987895	NM_001517	chr6:30879267-30879510	GTF2H4
A_24_P358245	7.1833873	7.390375	NR_003582	chr9:35450183-35450244	ATP8B5P
A_23_P165921	3.9086227	3.4740367		chr20:61665681-61665622	LINC00029
A_23_P58466	11.32902	11.204042	NM_000344	chr5:70248724-70248783	SMN1
A_24_P210399	9.267219	9.887251	NM_004800	chr13:100207838-100207897	TM9SF2
A_33_P3421338	5.153731	5.3303585	NM_001265577	chr17:43006294-43006235	KIF18B
A_24_P360529	4.95399	5.101246	AF332652	chr8:66634719-66634660	PDE7A
A_32_P115375	6.1948824	6.023051	CU677925	chr16:051680116-051680057	
A_23_P372946	8.357869	7.07519	NM_138461	chr3:196051170-196050838	TM4SF19
A_23_P320185	11.016445	11.06859	NM_175614	chr19:5896957-5896569	NDUFA11
A_23_P210048	7.1087503	6.8798294	NM_006037	chr2:239970735-239970676	HDAC4
A_24_P945194	7.84943	7.680694	NM_013374	chr3:33910819-33910878	PDCD6IP
A_33_P3415087	5.9325604	6.042391	NM_001127899	chrX:49856988-49857047	CLCN5
A_23_P159382	8.560639	8.681746	NM_014822	chr4:119644597-119644538	SEC24D
A_33_P3381816	5.120269	4.6287947	NM_183245	chr9:102888743-102888802	INVS
A_23_P77502	5.2134404	5.0354476	NM_001009944	chr16:2139313-2139254	PKD1
A_33_P3350658	3.8692355	2.3900566	NM_177398	chr1:165173170-165173111	LMX1A
A_33_P3342862	5.159935	4.5917215	NM_001204299	chr12:124799228-124799287	ZNF664-FAM101A
A_33_P3260862	4.597598	4.5709414		chr15:75519153-75519094	
A_33_P3822503	4.731065	4.5597486	NM_001330	chr16:30914761-30914820	CTF1
A_23_P134426	2.3221061	2.3900566	NM_001005340	chr7:23314496-23314555	GPNMB
A_23_P138105	4.24719	3.986276	NM_017638	chr1:28661889-28661948	MED18
A_24_P691826	2.3221061	2.3900566		chr17:58162479-58161084	LOC645638
A_33_P3380982	11.30285	10.284864	NM_018247	chr6:75962725-75962666	TMEM30A
A_23_P67453	3.6585352	2.3900566	NM_000363	chr19:55665570-55665511	TNNI3
A_24_P892494	2.3221061	2.3900566	NR_015450	chr4:13547877-13547818	LINC01096
A_23_P8900	12.806373	12.769602	NM_004374	chr8:100899787-100899728	COX6C
A_33_P3267186	6.4431863	6.9907165	NM_001682	chr12:89993004-89992945	ATP2B1
A_33_P3309656	3.3694692	3.9024465		chr3:057741906-057741965	
A_23_P370162	6.2446804	6.2338266	NM_182969	chr11:74552650-74552591	XRRA1
A_23_P382043	7.11322	7.2803726	NM_152729	chr6:116566312-116566371	NT5DC1
A_33_P3297255	10.21678	10.729543	NM_031300	chr5:176734275-176734216	MXD3
A_33_P3407299	10.189404	10.317737	NM_030920	chr1:150192734-150192675	ANP32E
A_33_P3303687	11.204042	11.350119	NM_002802	chr14:90738692-90738750	PSMC1
A_24_P196019	5.9135303	6.1787486		chr7:064197025-064196966	
A_23_P377819	10.670865	11.013473	NM_001039465	chr14:70238133-70238192	SRSF5
A_24_P931443	5.4618015	5.290685	NM_003485	chr14:91698988-91698929	GPR68
A_23_P34628	7.9373684	8.212871	NM_001514	chr1:89319001-89318942	GTF2B
A_32_P36313	6.3768716	6.5755053	NM_145206	chr10:114578395-114578454	VTI1A
A_24_P393611	4.123085	3.5943034	NM_138736	chr16:56374823-56374882	GNAO1

A_33_P3344816	2.3221061	2.3900566	NM_001004301	chr19:53997399-53997458	ZNF813
A_33_P3391536	6.889674	7.170024		chr7:026470201-026470260	
A_33_P3292164	4.3371053	4.0356164	NM_001277332	chr17:39432264-39432323	KRTAP9-7
A_24_P313109	6.7351837	7.040091	NM_016617	chr13:38936027-38936086	UFM1
A_32_P148476	9.268862	9.177562		chrX:025664334-025664275	
A_23_P125639	5.265937	5.355216	NM_003410	chrX:24228852-24228911	ZFX
A_23_P329353	10.124496	9.882057	NM_015463	chr2:68520397-68520338	CNRIP1
A_23_P60472	10.322451	10.377854	NM_018449	chr9:33922053-33921994	UBAP2
A_23_P346006	6.0432243	5.5415673	NM_020739	chr15:55648495-55648436	CCPG1
A_24_P58501	4.2883067	3.6633096	NM_001004195	chr15:102462933-102462874	OR4F4
A_23_P214091	9.837567	10.101191	NM_006330	chr8:54965236-54963711	LYPLA1
A_33_P3337272	6.403056	6.000111	NM_001004354	chr9:140194182-140194123	NRARP
A_24_P163237	4.473373	5.049308	NM_020225	chr4:184938430-184938489	STOX2
A_23_P387523	8.09928	8.134645	NM_001083621	chr1:22856891-22856950	ZBTB40
A_33_P3332382	6.0441427	6.0457153	AK092791	chr1:112939189-112939248	LOC100129406
A_33_P3372237	7.3411303	7.3867755	NM_015420	chr8:104432577-104432636	DCAF13
A_23_P68807	6.0409646	5.987764	BC021857	chr22:22282547-22282488	
A_23_P55616	4.5566425	4.241172	NM_001146037	chr18:43330241-43330300	SLC14A1
A_24_P263672	7.787652	7.694184	NM_014753	chr10:43316001-43316050	BMS1
A_23_P72668	3.8221283	4.1163397	NM_004657	chr2:192700161-192700102	SDPR
A_33_P3286357	4.4421577	4.082165		chr3:161046771-161046712	
A_23_P373584	2.3221061	4.569685	NM_139167	chr8:13947541-13947482	SGCZ
A_33_P3242829	4.0684767	4.032208	AF118089	chr1:89271761-89271820	PKN2
A_24_P147252	5.106386	5.0267715	NM_145911	chr16:71482661-71482602	ZNF23
A_33_P3374293	7.2912445	6.1234136	NM_024854	chr12:21623980-21624039	PYROXD1
A_24_P65373	4.4908767	4.473855	NM_000419	chr17:42453349-42453290	ITGA2B
A_32_P207169	10.1315365	9.827381	NR_024337	chr1:210404928-210404869	SERTAD4-AS1
A_23_P36018	4.2004237	4.3752465	NM_014312	chr11:124619655-124618652	VSIG2
A_33_P3255409	10.859557	10.915826	NM_017916	chr19:49949615-49949556	PIH1D1
A_33_P3299829	12.834596	12.813116	NM_002695	chr19:1088231-1088172	POLR2E
A_33_P3293247	6.1567736	5.9882774	NM_015597	chr9:139236220-139236279	GPSM1
A_33_P3362301	4.198723	3.7862577	AY956761	chr3:183835595-183835654	HSP90AA5P
A_23_P403588	5.7481875	5.1461415	NM_017944	chr11:11980395-11980454	USP47
A_23_P19619	5.9882774	5.931554	NM_002114	chr6:12164823-12164882	HIVEP1
A_24_P943301	4.638547	3.7752476	NM_001080471	chr1:156885677-156885736	PEAR1
A_23_P377888	8.0121565	7.7469187	NM_014967	chr15:31235016-31235075	FAN1
A_24_P160401	6.36254	5.861802	NM_178181	chr3:45153600-45152300	CDCP1
A_24_P9833	5.061437	5.458212	NM_001256696	chr11:45246231-45246290	PRDM11
A_24_P55225	3.8005042	4.3299522	NM_152732	chr6:43623383-43624327	RSPH9
A_23_P36972	5.8619156	6.1369133	NM_007129	chr13:100638017-100638076	ZIC2
A_23_P117980	4.888928	4.957954	NM_005886	chr16:57789933-57790281	KATNB1
A_23_P136012	7.914815	8.463739	NM_012180	chr4:175160186-175158733	FBXO8
A_33_P3379801	5.508963	5.494195	NM_001083908	chr14:50100655-50100596	DNAAF2
A_33_P3364379	7.5737195	7.6830893	NM_020470	chr11:66055350-66055291	YIF1A
A_23_P38015	10.908442	10.931988	NM_001105079	chr16:30682047-30682106	FBRS
A_23_P476	12.886798	12.662024	NM_003953	chr1:167759883-167759942	MPZL1
A_23_P349676	7.8461843	7.600404	NM_001080410	chr2:73481927-73481868	FBXO41
A_33_P3268284	8.180351	8.561877	NM_001494	chr10:5808548-5808489	GDI2
A_33_P3277614	13.11352	12.863685	NM_013341	chr2:174939813-174939754	OLA1
A_33_P3323559	7.292393	7.384305	NM_000394	chr21:44589285-44589344	CRYAA
A_24_P287691	7.7536383	7.6176443	NM_005829	chr15:90377778-90377719	AP3S2
A_32_P160896	6.9296093	7.0416303	NM_177478	chr5:121188259-121188318	FTMT
A_33_P3286929	4.411089	3.324966	XM_005254825	chr15:32781387-32781328	LOC100653133
A_33_P3292241	5.8003325	5.734159		chr8:027847456-027847515	
A_23_P137391	12.729376	12.877233	NM_001428	chr1:8924113-8924054	ENO1
A_33_P3367577	4.1093636	4.537468	DQ656044	chr22:31891999-31891940	EIF4ENIF1
A_32_P18258	12.853292	12.69109	NM_006755	chr11:764914-764973	TALDO1
A_33_P3319026	9.1012535	8.976902	NM_015534	chr1:78030632-78030573	ZZZ3
A_33_P3242965	4.0667005	4.1770935	NM_001039211	chr1:1396237-1396296	ATAD3C

A_32_P52119	6.370951	6.2815695	XR_248668	chr1:173387427-173387368	
A_23_P99917	9.367818	9.303995	NM_032856	chr15:85186449-85186390	WDR73
A_23_P130856	5.5757203	5.805782	NM_032816	chr19:33390784-33378710	CEP89
A_33_P3227467	9.745846	9.726696	NM_004330	chr15:59955154-59955095	BNIP2
A_33_P3278877	4.4428396	3.4265249	NM_002942	chr3:77629168-77629227	ROBO2
A_33_P3356332	5.6358466	5.4933805		chr7:138883232-138883173	
A_24_P362317	11.064225	11.136042	NM_001111	chr1:154554789-154554730	ADAR
A_33_P3377130	6.5805287	6.9848776	NM_005923	chr6:136901526-136901467	MAP3K5
A_23_P28068	5.6684875	5.0304685	NM_133644	chr19:17452718-17452777	GTPBP3
A_23_P40657	5.265798	5.060299	NM_014291	chr22:38212293-38212597	GCAT
A_33_P3382489	4.624734	4.6070113		chr2:12006351-12006410	LOC100506457
A_33_P3224020	10.789479	11.04696	BC033982	chr7:62865027-62864968	ARAF2P
A_23_P91081	4.181201	4.0913267	NM_002354	chr2:47613894-47613953	EPCAM
A_32_P145010	5.940391	5.6994963	NR_046273	chr17:61779010-61779069	LOC729683
A_33_P3315600	10.020062	10.18746	NM_003403	chr14:100744685-100744744	YY1
A_33_P3384287	10.012118	10.424505	NM_002579	chr19:748269-748328	PALM
A_32_P790284	5.1043897	4.75928	NM_031303	chr18:44627536-44627595	KATNAL2
A_33_P3365864	5.734275	6.1371427	NM_001143888	chr1:32834119-32834060	BSDC1
A_23_P58482	7.8386135	8.116404	NM_002406	chr5:180218078-180218019	MGAT1
A_23_P250035	6.739789	6.825727	NM_004168	chr5:235411-236558	SDHA
A_23_P157758	4.2733684	4.3853736	NM_001017969	chr9:5919881-5919822	KIAA2026
A_23_P123853	4.8755803	4.737569	NM_006274	chr9:34689665-34689606	CCL19
A_24_P450596	4.7854495	4.8679266	NM_001193552	chr19:37234678-37234619	ZNF850
A_33_P3281930	9.049988	8.839419	NM_006309	chr3:37095273-37095214	LRRFIP2
A_33_P3377564	4.1025653	4.1298723	NM_006150	chrX:49032147-49032088	PRICKLE3
A_23_P28598	6.605194	6.299017	NM_004405	chr2:172964421-172964362	DLX2
A_24_P45451	6.7914705	6.549695	NM_052931	chr1:160455780-160455721	SLAMF6
A_23_P153692	8.504196	9.027351	NM_006297	chr19:44048318-44047803	XRCC1
A_33_P3308223	3.9519958	3.5757124	NM_001198834	chr1:144882622-144882563	PDE4DIP
A_33_P3233580	4.988945	5.0351105	NM_001282769	chr10:24834162-24834221	KIAA1217
A_24_P32118	7.91077	8.352683	NM_015346	chr14:68213464-68213405	ZFYVE26
A_33_P3237850	3.2060108	4.343512		chr22:16158871-16158812	
A_33_P3297444	7.6926284	8.247517	NM_032548	chr3:127399692-127399751	ABTB1
A_33_P3358457	5.1856503	4.606927	NM_005270	chr2:121746874-121746933	GLI2
A_23_P21706	10.771115	10.293638	NM_001905	chr1:41478082-41478142	CTPS1
A_24_P263937	8.998751	9.0484	NM_199342	chr1:43272997-43272938	CCDC23
A_33_P3224070	4.1825676	3.8448188	NM_033027	chr3:39184605-39184546	CSRNP1
A_33_P3310366	6.1821165	6.4016433	NR_034006	chr9:68727550-68727609	LOC100132352
A_33_P3218188	9.807876	9.703857	NM_032641	chr12:6980172-6980113	SPSB2
A_33_P3229083	9.638739	9.51797	NM_080593	chr6:27114496-27114437	HIST1H2BK
A_24_P165450	3.7031198	4.328869	NM_024686	chr1:84369815-84356159	TTLL7
A_24_P193435	10.943184	10.84107	NM_003257	chr15:29992586-29992527	TJP1
A_24_P203710	9.47271	9.428935	NM_003934	chr9:133513041-133513100	FUBP3
A_24_P379765	8.813404	8.908539	NM_018221	chr2:74392464-74392405	MOB1A
A_33_P3311791	3.7971418	4.330265	NM_001023561	chr19:57956666-57956725	ZNF749
A_33_P3388486	6.4170046	6.275365	NR_037166	chr6:160181349-160181290	LOC100129518
A_23_P214907	7.305318	7.2017236	NM_015440	chr6:151206858-151209024	MTHFD1L
A_23_P19712	11.089325	11.102488	NM_015895	chr6:24786108-24786167	GMNN
A_23_P214026	8.032894	8.219502	NM_001999	chr5:127594232-127594173	FBN2
A_33_P3387696	7.330289	7.4169016	NM_001282606	chr12:66530792-66530733	TMBIM4
A_33_P3322519	4.4156876	4.6278605	NM_138345	chr3:183960057-183960116	VWA5B2
A_24_P97526	8.333238	8.633041	NM_017801	chr3:32523335-32523276	CMTM6
A_24_P109633	4.0030646	4.2954807	NM_001142594	chr14:93403377-93403318	ITPK1
A_32_P216602	6.40654	6.244502	DB229023	chr1:201476464-201476523	
A_23_P48771	7.5784254	7.847496	NM_024952	chr14:91691358-91691417	C14orf159
A_33_P3394494	10.9200325	10.711566	NM_203413	chr17:7163187-7163246	ELP5
A_33_P3362208	4.9691033	4.975405	AK130248	chr5:106758308-106758249	LOC100129233
A_33_P3248325	3.655799	2.3900566	NM_001184938	chr2:120861757-120861816	EPB41L5
A_23_P131723	12.24349	12.684835	NM_006826	chr2:9724897-9724838	YWHAQ

A_33_P3225091	7.183969	6.975334	NM_004756	chr19:41171921-41171862	NUMBL
A_33_P3328973	5.3072557	5.712641	AK129565	chr10:1552545-1552486	
A_33_P3360611	6.580797	6.6363754	NM_001278381	chr17:41952786-41952727	MPP2
A_23_P257201	7.2015495	7.6697435	NM_030963	chr6:127608778-127608837	RNF146
A_33_P3369991	4.1565666	4.25789		chr5:146258644-146258585	
A_33_P3390758	11.696658	11.63315	NM_153201	chr11:122929537-122929478	HSPA8
A_23_P89343	10.783081	10.960623	NM_152244	chr17:46199801-46199860	SNX11
A_33_P3296592	6.4249663	6.393606	NM_020193	chr11:76262527-76262586	C11orf30
A_24_P943472	5.448444	4.865713	NM_005126	chr3:24021460-24021519	NR1D2
A_23_P339119	2.3221061	2.3900566	NM_024560	chr12:81647397-81648642	ACSS3
A_33_P3412349	3.3978205	3.9431856	NM_152943	chr12:133779282-133779341	ZNF268
A_33_P3248794	8.499909	8.384398	NM_005967	chr12:57489162-57489221	NAB2
A_23_P157196	14.334603	14.601013	NR_026676	chr7:23530827-23530888	RPS2P32
A_24_P230916	8.491976	8.580074	NM_001077702	chr1:67424625-67425379	MIER1
A_23_P82859	5.255596	5.0917516	NM_004337	chr8:90939613-90939672	OSGIN2
A_24_P142983	7.3673553	7.3655663	NM_002650	chr22:21064256-21064197	PI4KA
A_32_P130630	6.0916953	5.791157	NM_004731	chr12:60174827-60174886	SLC16A7
A_33_P3335398	3.7543576	4.0150323	NM_001080395	chr17:79094061-79094002	AATK
A_33_P3394933	4.1692634	4.1658664	NM_001017973	chr5:131549669-131549610	P4HA2
A_23_P257956	13.229299	13.308332	NM_004515	chr1:153634727-153634668	ILF2
A_23_P139050	3.853053	4.524761	NM_001002918	chr11:124189582-124189523	OR8D2
A_24_P46725	5.213916	5.7448006	NM_152551	chr6:7611182-7611241	SNRNP48
A_23_P3819	4.5330544	4.627315	NM_023931	chr16:30543568-30543509	ZNF747
A_33_P3409844	4.5002575	3.8960338	NM_015888	chr1:60299149-60299208	HOOK1
A_24_P917026	7.5532804	7.670324	NM_001042492	chr17:29704560-29704619	NF1
A_23_P162010	5.969927	5.8590055	NM_176875	chr11:6293013-6293072	CCKBR
A_33_P3367761	3.8366663	4.595462		chr18:14864526-14864585	XLOC_014512
A_33_P3303463	7.693325	7.596129	AK128128	chr16:33347566-33347507	
A_33_P3262814	5.5652566	6.330321	NM_001001330	chr10:65381688-65381747	REEP3
A_24_P302695	9.9830475	9.840884	NM_145212	chr2:99813795-99813854	MRPL30
A_23_P145501	7.264981	7.605653	NM_004830	chr6:131907904-131907876	MED23
A_33_P3404047	4.8560667	5.0882716		chr1:183604736-183604795	
A_23_P9056	9.9698105	9.751658	NM_014781	chr8:53536078-53536019	RB1CC1
A_33_P3325336	11.142983	11.600517		chrX:097644673-097644732	
A_24_P23411	7.440307	7.6293726	NM_016607	chrX:100881013-100881072	ARMCX3
A_23_P149975	10.094004	10.261899	NM_031453	chr10:14561836-14561777	FAM107B
A_23_P254688	5.6661577	5.6466627	NM_023943	chr3:133114743-133114802	TMEM108
A_23_P63432	2.3221061	2.3900566	NM_017821	chr1:39352339-39352280	RHBDL2
A_23_P13294	4.349527	4.8912983	NM_001004459	chr11:57971569-57971510	OR1S2
A_33_P3371718	9.211214	9.893087	NM_002970	chrX:23803863-23803922	SAT1
A_23_P67829	9.152109	9.673412	NM_025076	chr2:106710329-106710270	UXS1
A_23_P80040	10.579005	10.361334	NM_006404	chr20:33764988-33765047	PROCR
A_23_P13425	10.941367	10.956079	NM_004356	chr11:2418184-2418243	CD81
A_23_P48610	7.1038284	7.272501	NM_144568	chr14:20926537-20926478	TMEM55B
A_33_P3359518	5.202057	5.345203	NM_001085487	chr1:59125723-59125664	MYSM1
A_33_P3237744	8.049077	8.28058		chr19:56052107-56052048	SBK3
A_24_P304449	8.599048	8.594515	NM_014730	chr12:121138976-121139035	MLEC
A_23_P3574	7.942092	7.871766	NM_030819	chr16:67708712-67708653	GFOD2
A_23_P23114	9.30622	9.519344	NM_080391	chr1:32374163-32374105	PTP4A2
A_33_P3238215	8.6498995	9.387709	NM_001278458	chr2:165541428-165541369	COBLL1
A_33_P3321632	5.0525007	5.170755		chr6:080251726-080251785	
A_33_P3257755	8.562648	8.870609		chr9:042797562-042797503	
A_24_P829183	4.945758	5.0910397		chr5:42991176-42991117	FLJ32255
A_32_P223189	9.651842	10.021111	NR_002190	chr1:160287432-160287491	SUMO1P3
A_33_P3248759	7.9556026	7.880964	XM_005255626	chr16:734685-734626	WDR24
A_24_P261929	8.020627	8.092286	NM_206949	chr14:94568310-94568870	IFI27L1
A_33_P3354151	7.605952	7.606527	NM_016074	chr1:149871961-149872020	BOLA1
A_33_P3278068	7.557063	7.1822863	NM_001145353	chr13:41525556-41525497	ELF1
A_33_P3226832	6.6919856	6.009125	NM_001993	chr1:94995139-94995080	F3

A_23_P51679	9.020862	9.258713	NM_005920	chr1:156436644-156436585	MEF2D
A_23_P16944	10.501459	10.28187	NM_001006946	chr2:20401394-20401335	SDC1
A_33_P3354955	8.11729	7.7837987	NM_016649	chr20:13695615-13695556	ESF1
A_32_P107876	6.1525927	5.4261956	NM_025074	chr4:79465254-79465313	FRAS1
A_32_P201773	7.727596	7.481267	NM_015365	chrX:109437714-109437655	AMMECR1
A_24_P11436	4.6683345	4.96225	NM_017904	chr1:55251801-55251742	TTC22
A_33_P3394694	8.026331	8.095573	AK126742	chr16:68626271-68626330	LOC100128498
A_23_P102607	3.3776076	4.122872	BC039860	chr20:40126054-40125995	CHD6
A_23_P201287	7.180576	7.797292	NM_015074	chr1:10441250-10441309	KIF1B
A_33_P3703501	9.314642	9.307959	NM_198321	chr5:153798492-153798551	GALNT10
A_33_P3234124	4.620007	4.8140593	XM_001714385	chr2:239074260-239074319	
A_23_P132738	4.925545	5.5969405	NM_017541	chr3:186256728-186256669	CRYGS
A_32_P66881	7.1215916	6.8163366	NM_138554	chr9:120479236-120479295	TLR4
A_23_P6891	10.598837	10.512553	NM_005875	chr3:40353654-40353713	EIF1B
A_33_P3601163	7.9266677	7.740486		chr19:10762602-10762543	ILF3-AS1
A_23_P258221	8.635883	9.213874	NM_005688	chr3:183637801-183637742	ABCC5
A_24_P238499	4.8504796	5.064829	NM_001012716	chr18:649747-649688	C18orf56
A_33_P3257910	3.8677955	3.3827748	NM_001168214	chr3:159945915-159945974	C3orf80
A_23_P66948	7.488234	7.736155	NM_022751	chr18:29847640-29847581	GAREM
A_23_P372331	7.898643	7.5891547	NM_182916	chr3:3189717-3189776	TRNT1
A_24_P74981	6.0912046	6.4471254	NM_015013	chr1:23405495-23405554	KDM1A
A_23_P34942	5.1430893	4.934392	NM_005665	chr1:93142735-93131511	EVI5
A_33_P3352980	10.861808	10.893549		chr1:015040135-015040194	
A_33_P3240182	3.909256	3.9483678	AF017462	chr14:107034868-107034809	
A_24_P294931	8.661682	8.849381	NM_180976	chr6:42979730-42979789	PPP2R5D
A_23_P320717	5.6343284	5.490097	NM_015386	chr16:70515754-70515695	COG4
A_33_P3227880	6.1737294	6.349939	NM_001197247	chr6:26375985-26376044	BTN3A2
A_33_P3397763	10.267456	10.496923	NM_003811	chr19:6535866-6535925	TNFSF9
A_33_P3253672	10.214254	10.517908	NM_012284	chr12:49952000-49952059	KCNH3
A_33_P3220381	6.8050785	6.8139687	NM_001243737	chr5:64956589-64956648	TRAPPC13
A_24_P85181	8.147621	8.330036	NM_022340	chr3:15111894-15111835	ZFYVE20
A_33_P3320010	6.8157167	7.510902	NM_006089	chrX:18257745-18257686	SCML2
A_23_P56529	7.907851	7.788469	NM_001087	chr2:219130152-219129897	AAMP
A_33_P3329916	14.903694	14.914306	NM_001024662	chr12:112843025-112842976	RPL6
A_23_P67312	6.305276	6.776979	NM_003437	chr19:12299216-12299274	ZNF136
A_24_P255384	9.768059	9.848006		chr9:110802538-110802597	XLOC_014512
A_24_P721898	9.260631	9.357062	NM_001002860	chr14:93704043-93703984	BTBD7
A_23_P309701	7.0895123	7.1360326	NM_002828	chr18:12794039-12793980	PTPN2
A_23_P4041	8.721614	9.120375	NM_021213	chr17:53854622-53854681	PCTP
A_33_P3531204	4.478866	4.598206	NM_001135816	chr13:24465817-24465876	C1QTNF9B-AS1
A_33_P3376379	5.0078254	4.7258663	NM_001139459	chr1:246755126-246755185	CNST
A_24_P397107	8.046732	8.016396	NM_001789	chr3:48199183-48199124	CDC25A
A_23_P161439	4.3293695	4.056716	NM_006829	chr10:88730308-88730367	ADIRF
A_24_P64653	3.9766157	3.651248	NM_152637	chr12:56077637-56077696	METTL7B
A_23_P135454	6.6568	6.7364736	NM_006796	chr18:12340314-12340255	AFG3L2
A_24_P941336	9.444629	9.333678	NM_018128	chr17:2226955-2226896	TSR1
A_33_P3784253	8.868793	8.697246	NM_001128620	chr11:77034194-77034135	PAK1
A_23_P383688	8.834667	8.938311	NM_020745	chr6:44267479-44267420	AARS2
A_24_P914495	5.8519506	5.5227833	NM_004954	chr11:63667435-63667494	MARK2
A_32_P351277	5.298292	5.7603273	BC004968	chr16:728577-728518	
A_24_P865672	10.383572	10.385995		chr15:073192057-073191998	
A_32_P4626	3.0827966	3.0700023	XR_241970	chr6:7540800-7540741	
A_23_P34460	5.4979897	5.9536057	NM_012243	chr1:100483334-100487963	SLC35A3
A_33_P3416479	5.0151024	5.2297735	NM_001258459	chr7:56148841-56148782	PHKG1
A_32_P128701	5.64311	5.4790106	NM_019050	chr4:120216331-120216390	USP53
A_23_P99762	7.4499335	7.679331	NM_021239	chr14:73587194-73587253	RBM25
A_33_P3410206	8.743888	8.816544	NM_004913	chr16:89777241-89777182	VPS9D1
A_33_P3276678	5.3800125	6.0971665	NM_199425	chr20:25059431-25059372	VSX1
A_33_P3342375	2.3221061	2.3900566	NM_175868	chrX:151870745-151870804	MAGEA6

A_33_P3368785	7.9014034	8.08669	NR_024101	chr18:3597314-3597373	DLGAP1-AS1
A_33_P3297415	6.5454707	6.149981	NM_201264	chr2:206562385-206562444	NRP2
A_23_P12896	6.9684563	7.255573	NM_022725	chr11:22644792-22644733	FANCF
A_33_P3220833	7.0914097	6.268288	NM_002577	chr3:196559045-196559104	PAK2
A_24_P305678	9.871683	10.064254	NM_012399	chr22:28247999-28247940	PITPNB
A_33_P3319982	5.0829086	5.2353287	BC015443	chr16:18938207-18938266	
A_23_P320225	4.5691175	4.5951185	NM_147127	chr4:5564743-5564684	EVC2
A_33_P3249529	9.634018	9.339514	NM_014982	chr14:71581937-71581996	PCNX
A_33_P3334092	4.8242035	4.5809484	AK096917	chr22:42227537-42227478	
A_33_P3406661	4.6232796	4.6135798	NM_020431	chr14:77725778-77725837	TMEM63C
A_33_P3226985	6.818852	6.768622	NM_003885	chr17:30818168-30818227	CDK5R1
A_33_P3364808	6.270461	6.134973	NM_002838	chr1:198671524-198671583	PTPRC
A_23_P124327	8.341095	8.1704	NM_022455	chr5:176722574-176722633	NSD1
A_24_P336705	9.719181	9.489654	NM_004582	chr1:76259803-76259862	RABGGTB
A_24_P226108	5.4177313	5.3157816	NM_019027	chr4:40428153-40428094	RBM47
A_33_P3269049	4.0634394	3.800786		chrX:009381389-009381448	
A_33_P3411384	9.389446	9.083764			
A_33_P3261953	4.1104813	3.8630857	NR_103470	chr19:22791181-22791240	LOC100996349
A_23_P75179	9.2526655	9.468781	NM_001200049	chr10:134624466-134623947	TTC40
A_23_P11644	3.1178057	3.5791292	NM_006945	chr1:153012412-153012353	SPRR2D
A_33_P3219115	5.898978	6.0576982		chr4:143238618-143238677	
A_33_P3363260	4.503216	5.045892	NM_173582	chr11:74041421-74041362	PGM2L1
A_24_P306594	3.3383698	2.3900566	XM_003118845	chr1:11849476-11849535	
A_23_P92629	8.599561	8.262327	NM_005869	chr5:64314286-64314345	CWC27
A_33_P3328543	3.4769263	3.386989		chr5:000294315-000294374	
A_33_P3242062	4.2261314	4.1812854		chr9:125451692-125451751	
A_33_P3328659	8.075453	7.9296026	NM_014246	chr22:46760146-46760087	CELSR1
A_33_P3252083	3.9171276	3.581572	NM_021150	chr12:66742859-66742800	GRIP1
A_33_P3225327	7.7637963	7.8140144	NM_024524	chr3:194150863-194150804	ATP13A3
A_23_P63379	4.6989484	4.118888	NM_012113	chr1:150237369-150237428	CA14
A_33_P3287348	2.3221061	2.3900566	NM_004067	chr7:29553778-29553837	CHN2
A_33_P3364293	6.7449064	6.8792677	NR_027420	chr21:9921746-9921687	LOC389834
A_24_P589855	3.752162	3.3396518		chr1:106495502-106495443	
A_24_P378019	7.710749	8.567994	NM_004031	chr11:612702-612643	IRF7
A_33_P3232363	3.736639	3.7603464	AF111851	chr1:31461422-31461481	
A_33_P3355185	4.6090946	4.6022367	NM_000657	chr18:60985460-60985401	BCL2
A_24_P381962	9.499588	9.512687	NM_001030007	chr16:71763227-71763168	AP1G1
A_24_P242361	6.783636	6.5990314	NM_012262	chr1:87575369-87575428	HS2ST1
A_23_P342825	6.867209	6.7469897	NM_022039	chr10:103370907-103370848	FBXW4
A_23_P390734	8.0436325	8.04772	NM_015633	chr12:27118788-27118847	FGFR1OP2
A_32_P195065	5.206815	5.2008133	NM_004263	chr2:74908466-74908525	SEMA4F
A_24_P577694	5.478352	5.7324815	NM_021116	chr7:45762328-45762385	ADCY1
A_23_P218675	4.4431353	4.5963774	NM_006103	chr20:44108669-44108728	WFDC2
A_33_P3267640	6.521847	6.767451	NM_001528	chr4:3451154-3451213	HGFAC
A_33_P3251796	7.10424	7.178626	NM_014317	chr10:27012982-27013041	PDSS1
A_33_P3410232	4.868609	4.9072776	NR_024541	chr6:31681695-31681636	LY6G6E
A_33_P3320393	15.066905	15.127534	NM_016065	chr10:75008755-75008696	MRPS16
A_24_P937855	6.9949	7.208794	NM_001102396	chr1:115313097-115313038	SIKE1
A_24_P85881	4.8289337	4.5891266	NM_153835	chr2:26533618-26533105	GPR113
A_23_P211561	5.399818	5.2969017	NM_152513	chr22:42195336-42195395	MEI1
A_23_P115608	8.013854	8.260393	NM_020824	chr10:24873139-24873080	ARHGAP21
A_33_P3265180	6.966941	6.9183226	NM_152221	chr22:38687535-38687476	CSNK1E
A_24_P106839	4.950611	5.4098186	NM_023034	chr8:38133969-38133910	WHSC1L1
A_33_P3341656	8.964469	8.752386	NM_005920	chr1:156433658-156433599	MEF2D
A_33_P3362249	4.625881	4.888928	XM_005250738	chr7:63226669-63226728	LOC100996380
A_24_P130026	8.253811	8.39743	NM_001015049	chr14:104023781-104023722	BAG5
A_33_P3301709	8.358159	8.892633	NM_001098722	chr1:235714443-235714384	GNG4
A_23_P83403	6.1155577	6.08158	NM_014988	chr4:41694344-41699178	LIMCH1
A_23_P41204	4.3521595	4.342	NM_144635	chr3:184063177-184063236	FAM131A

A_24_P168760	6.820813	7.1139708	NM_004286	chr22:39129283-39129342	GTPBP1
A_33_P3386453	2.834817	3.0756245	NM_001030055	chr14:32623889-32623948	ARHGAP5
A_23_P408830	6.658184	6.7145166	NM_172215	chr12:121687648-121683043	CAMKK2
A_33_P3322082	6.5557346	6.396752	BQ934349	chr17:44580200-44580259	
A_23_P158880	6.776979	6.977076	NM_181900	chr15:81605262-81605203	STARD5
A_23_P63751	10.250599	10.518948	NM_006793	chr10:120931976-120931917	PRDX3
A_23_P166159	8.879642	8.702376	NM_030815	chr20:30533055-30532996	PDRG1
A_23_P324482	4.0705385	3.3905377	NM_020922	chrX:54228452-54225056	WNK3
A_33_P3832680	4.0105743	3.1882143	BC127791	chr10:48249050-48248991	FAM25C
A_23_P19348	4.25789	4.639407	NM_014780	chr6:43006654-43006595	CUL7
A_33_P3332126	6.5866413	6.4844847	AK127520	chr2:238999869-238999928	SCLY
A_33_P3373805	2.3221061	4.3234615		chr5:148450920-148450979	LOC255187
A_33_P3332860	4.5352592	4.6938467	NM_001001433	chr20:57246274-57246333	STX16
A_24_P154142	5.712869	5.909088	NM_181885	chr1:155912337-155912396	RXFP4
A_23_P215819	7.4791174	7.0622106	NM_024061	chr7:99162110-99162169	ZNF655
A_23_P70968	5.00513	4.420999	NM_006896	chr7:27194490-27194431	HOXA7
A_33_P3391603	2.3221061	2.3900566	NM_001105209	chr6:112575046-112574987	LAMA4
A_23_P89155	4.462004	4.056204	NM_001258	chr17:73998494-73998645	CDK3
A_24_P399009	3.7510104	3.2889738	NM_014329	chr16:67916002-67916163	EDC4
A_23_P409553	3.6211858	3.6815782	NM_177951	chr14:60752731-60752790	PPM1A
A_33_P3217998	8.671925	8.858154	NR_040288	chr1:19593853-19593794	AKR7L
A_33_P3262156	4.968377	4.853849	NM_021097	chr2:40342452-40342393	SLC8A1
A_24_P92367	9.268351	8.892191	NM_001017964	chr22:21982583-21982524	YDJC
A_23_P4160	4.1237445	4.505249	NR_003108	chr17:41290923-41291875	NBR2
A_23_P154108	11.096948	11.124192	NM_001378	chr2:172604418-172604477	DYNC1I2
A_23_P418083	5.9679523	5.8676815	NM_181714	chr6:80195326-80195267	LCA5
A_32_P452655	5.754383	5.4177313	NM_001040078	chr17:18398176-18398235	LGALS9C
A_33_P3413514	5.220378	5.067465		chr2:132535257-132535316	
A_24_P98109	8.516182	8.620146	NM_013322	chr7:26413403-26413462	SNX10
A_23_P168592	5.2224817	5.2628107	NM_138771	chr7:23682719-23682778	CCDC126
A_23_P134058	3.9666915	2.3900566	NM_001242629	chr6:13469858-13469799	GFOD1
A_33_P3333810	8.423832	8.114739	NM_003350	chr8:48974169-48974228	UBE2V2
A_33_P3261737	5.074171	5.2783527	NM_001282647	chrX:48763731-48763672	SLC35A2
A_23_P134008	4.8814545	4.568055	BC005991	chr6:99930682-99930623	USP45
A_33_P3337599	11.300191	11.641162	NM_006904	chr8:48686544-48686485	PRKDC
A_23_P101297	5.8247833	5.888195	NM_012155	chr19:46117910-46116918	EML2
A_24_P82957	8.7451515	8.814714	NM_018683	chr20:48569623-48569682	RNF114
A_23_P66891	6.7696767	6.82226	NM_012121	chr17:71280269-71280210	CDC42EP4
A_23_P354705	2.4804037	2.3900566	NM_003034	chr12:22354130-22354071	ST8SIA1
A_33_P3385686	4.7922797	5.0320835		chr6:130454177-130454118	
A_23_P97623	9.404233	9.800841	NM_022157	chr1:39305371-39305312	RRAGC
A_23_P76684	8.401019	8.755485	NM_006054	chr11:63526909-63526968	RTN3
A_33_P3217103	6.917654	7.086613	NM_001031695	chr22:36140089-36140030	RBFOX2
A_24_P290163	6.7176857	6.2882233	AL834164	chr16:22477119-22477178	SMG1P1
A_32_P44394	3.2553728	3.4966273	NM_004833	chr1:159033365-159033306	AIM2
A_33_P3220911	3.2038321	3.928296	NM_004335	chr19:17513807-17513748	BST2
A_24_P810290	8.012765	7.848129	NM_001030059	chr10:122349102-122349161	PPAPDC1A
A_33_P3273285	4.622943	4.7432003	NR_003699	chr19:53884253-53884312	ZNF525
A_23_P109034	7.9234524	7.634612	NM_002999	chr20:43954637-43954578	SDC4
A_33_P3224495	9.202936	8.3511	XR_158796	chr6:73388159-73388218	KCNQ5-IT1
A_33_P3402257	5.8219852	5.670467	BX116876	chr11:60511408-60511467	
A_33_P3344618	8.915782	9.054983	NM_007162	chr6:41651777-41651718	TFEB
A_23_P143748	9.779439	9.385975	NM_015140	chr22:43562901-43562842	TTLL12
A_24_P340776	6.602396	6.792082	XM_005276537	chr16:12023129-12023070	
A_24_P85478	7.6420555	7.5051346	NM_005744	chr15:72877794-72877853	ARIH1
A_23_P17769	10.216342	10.457739	NM_001355	chr22:24313789-24313730	DDT
A_32_P36235	9.33168	8.956146	NM_004907	chr19:13265586-13265645	IER2
A_33_P3273919	4.8076544	4.9223347	AK124578	chr4:1054740-1054681	RNF212
A_23_P94461	5.485355	5.5253	NM_031919	chr9:108234206-108234265	FSD1L

A_23_P14649	13.197346	13.221834	NR_026808	chr15:69096922-69096863	ANP32A-IT1
A_33_P3449097	6.0503607	5.5030127	NM_031945	chr17:79615719-79615778	TSPAN10
A_32_P18470	9.658663	9.740524	NM_001012979	chrX:102528719-102528660	TCEAL5
A_23_P251593	15.2583475	15.16313	NM_007104	chr6:35438429-35438488	RPL10A
A_24_P398130	7.0837283	7.493224	NM_014688	chr10:11502756-11502697	USP6NL
A_33_P3331237	5.0074415	4.686343		chr5:151192282-151192341	G3BP1
A_33_P3243588	7.7793922	7.9103703	NM_003630	chr6:143793334-143793393	PEX3
A_33_P3333787	3.8864613	4.110589	AF132204	chr3:144162967-144163026	
A_33_P3370029	3.5145082	3.8520937	S60780	chr7:38331276-38331217	
A_24_P148811	6.503545	6.401042	NM_003707	chr3:127819515-127819456	RUVBL1
A_24_P925664	7.24439	6.8640523	NM_002392	chr12:69238891-69238950	MDM2
A_33_P3321678	11.3644285	11.308997	AK090756	chr5:63513485-63513544	RNF180
A_33_P3265504	6.9319887	7.275055	NM_001099692	chr10:82003712-82003653	EIF5AL1
A_23_P74278	2.3221061	2.3900566	NM_001037341	chr1:66839789-66839848	PDE4B
A_23_P32135	5.967016	6.2406435	NM_018956	chr9:135762941-135763718	C9orf9
A_33_P3393766	8.156947	8.123773	NM_001130677	chr17:36828068-36828009	C17orf96
A_33_P3350673	4.3976183	4.1338058	NM_001145460	chr4:57516909-57516850	HOPX
A_33_P3216532	6.2672987	6.450806	NM_004444	chr7:100400590-100400531	EPHB4
A_23_P107283	2.3221061	2.3900566	NM_002145	chr17:46620186-46620127	HOXB2
A_24_P287043	10.537747	11.011292	NM_006435	chr11:309109-309168	IFITM2
A_33_P3229221	3.9595985	3.5935073		chr1:36603747-36603688	TRAPPC3
A_32_P46840	4.22915	3.603851	AK090664	chr13:21276863-21276804	LOC729680
A_24_P356130	4.6581135	4.5320187	NM_002757	chr15:67985864-67995691	MAP2K5
A_33_P3320368	9.667671	9.724951	NM_017814	chr19:19230489-19230430	TMEM161A
A_33_P3295228	5.829169	5.8515964	AY358211	chr19:4042430-4042371	
A_33_P3246083	8.857646	8.765736	NM_017897	chr3:25835941-25836000	OXSM
A_24_P49427	3.6543355	3.973514	NM_020864	chr2:226518385-226518444	NYAP2
A_33_P3410484	4.6623907	4.7441754	NM_001193339	chr20:45239145-45239086	SLC13A3
A_33_P3424204	4.575146	4.2211494	NR_002771	chr1:64016248-64016307	DLEU2L
A_23_P144020	3.2052722	3.5125408	NM_175613	chr3:3095585-3095644	CNTN4
A_33_P3276703	6.422281	5.566322	NM_003378	chr7:100805858-100805799	VGF
A_23_P63736	7.1461687	7.186783	NR_026827	chr10:42989735-42989794	LINC00839
A_23_P153586	12.157899	12.044691	NM_006003	chr19:29698415-29698356	UQCRFS1
A_23_P128351	3.477714	4.224139	NM_032680	chr12:3782664-3768814	EFCAB4B
A_23_P20075	5.341107	5.3366733	NM_013389	chr7:44552449-44552390	NPC1L1
A_23_P200489	6.494957	5.875822	NM_014698	chr1:226033746-226033687	TMEM63A
A_23_P379071	4.036071	4.523241	NM_145032	chr7:102462615-102462556	FBXL13
A_23_P380208	7.4618955	6.9404697	NM_024621	chr3:156978988-156978929	VEPH1
A_23_P406521	4.4783087	4.78407	NM_178460	chr20:1515082-1515023	SIRPD
A_23_P28318	8.695301	8.746318	NM_144736	chr2:37475476-37475535	NDUFAF7
A_23_P1676	8.301751	8.37027	NM_001258243	chr11:124967267-124967208	TMEM218
A_24_P362881	4.538808	4.533448	NM_018010	chr3:107880167-107880108	IFT57
A_23_P361820	4.275831	4.5941668	NM_015104	chr11:64662785-64662646	ATG2A
A_33_P3293353	3.996729	4.706068	NM_016121	chr1:215753282-215753341	KCTD3
A_23_P38952	4.6371546	4.621549	NM_133492	chr19:6312480-6312421	ACER1
A_33_P3394243	6.0381446	5.785183	NM_173807	chr1:244773568-244773627	C1orf101
A_23_P397055	5.140354	4.6427674	NM_019015	chr7:150934974-150935033	CHPF2
A_33_P3382271	6.4650373	6.209438	BC033643	chr14:92506805-92506864	
A_33_P3232120	5.125923	5.0980396		chr9:140347023-140346964	NSMF
A_23_P140602	6.404907	6.3132386	NM_001033088	chr15:90814473-90814532	NGRN
A_33_P3416286	7.6243515	7.5856733	NM_006047	chr20:34238408-34238349	RBM12
A_23_P423891	8.138827	8.446927	NM_152707	chr10:70242724-70242665	SLC25A16
A_24_P873764	6.8151617	6.8491187	NM_004327	chr22:23660120-23660179	BCR
A_24_P139773	6.797023	6.76303	NM_138558	chr1:28176844-28176903	PPP1R8
A_33_P3281176	6.997992	6.8452063		chrX:001553923-001553982	
A_33_P3241250	4.9346213	4.7736483	XR_132464	chr7:56876820-56876811	LOC401357
A_23_P18267	6.415081	6.6352577	NM_006545	chr3:50385587-50385306	NPRL2
A_33_P3335590	4.870277	4.97957	NM_001103175	chr16:3077927-3077868	CCDC64B
A_32_P112623	7.522696	7.157105	NR_103714	chr9:67784971-67784912	FAM27E2

A_33_P3286916	4.0886736	4.554728	NM_001195263	chr10:102768572-102768513	PDZD7
A_23_P97860	9.32595	9.276172	NM_000235	chr10:90973457-90973398	LIPA
A_24_P228026	5.8336086	5.935235	NM_144611	chr17:4060275-4060334	CYB5D2
A_23_P394605	8.882125	7.933358	NM_021982	chr5:134063223-134063282	SEC24A
A_23_P257423	7.7879333	7.3763967	NM_144988	chr1:95448646-95448587	ALG14
A_24_P365365	10.47119	10.092465	NM_003200	chr19:1609391-1609332	TCF3
A_23_P211126	8.612051	8.896365	NM_130436	chr21:38887078-38887137	DYRK1A
A_23_P29575	9.847651	9.815199	NM_033281	chr5:68525036-68525095	MRPS36
A_23_P153461	4.578207	4.633498	NM_022737	chr19:11475760-11475819	LPPR2
A_33_P3223964	2.5383234	3.0859137	AF533986	chr14:106733285-106733226	
A_23_P140154	7.8221016	7.75624	NM_001098621	chr14:93653117-93653176	TMEM251
A_33_P3243230	2.820725	2.3900566		chr4:074607982-074608041	
A_23_P35205	6.694638	7.1368923	NM_013441	chr1:24863377-24863436	RCAN3
A_23_P125519	14.899027	14.9299965	NM_001007	chrX:71493113-71492595	RPS4X
A_32_P112910	4.060705	5.0360875	NM_203412	chr1:110655660-110655719	UBL4B
A_23_P49009	4.3411145	4.5157804	NM_153613	chr15:34653630-34652381	LPCAT4
A_33_P3360718	6.198984	6.137902	NM_001114091	chr17:45214580-45214521	CDC27
A_33_P3285815	4.7911797	3.9191804		chr20:44163856-44163797	WFDC6
A_33_P3279871	5.2979803	5.55077	XM_003959954	chr19:21838600-21838659	LOC400682
A_23_P201951	7.4765577	8.0394535	NM_016374	chr1:235331255-235331196	ARID4B
A_33_P3306287	5.4819927	5.34956	NM_024086	chr17:2371115-2371056	METTL16
A_33_P3243343	12.7895565	12.718337		chr14:029062978-029063037	
A_33_P3420068	6.3465543	6.3998	NM_001002296	chr8:41363520-41364625	GOLGA7
A_24_P8494	13.166815	13.109162	NM_006808	chr9:101992669-101992728	SEC61B
A_23_P98744	4.326154	4.260016	NM_001005172	chr11:4471196-4471255	OR52K2
A_23_P130418	11.09206	11.069391	NM_021074	chr18:9122594-9122653	NDUFV2
A_33_P3858476	5.811692	5.7546277	NR_033873	chr2:59290841-59290900	LINC01122
A_23_P62335	7.4970818	7.4420834	NM_018196	chrX:154736669-154736610	TMLHE
A_32_P40547	8.4768095	8.862456	NR_002766	chr14:101295974-101296033	MEG3
A_24_P295543	9.523721	9.6959715	NM_001001342	chr10:102034598-102034539	BLOC1S2
A_23_P338325	6.3856173	6.75872	NM_005230	chr12:96660926-96660985	ELK3
A_33_P3290394	5.1632285	5.4814796	NM_000206	chrX:70327330-70327271	IL2RG
A_33_P3298577	5.190082	5.5054345	NM_001167856	chr12:123780560-123780501	SBNO1
A_33_P3291772	4.7706833	4.0777926		chr18:061164087-061164146	
A_24_P303080	5.8479204	5.8402996	NM_152778	chr4:128839701-128839642	MFSD8
A_23_P210515	5.406931	5.388963	NM_020967	chr20:44690371-44690312	NCOA5
A_23_P355536	9.707967	9.778924	NM_152586	chr10:75257445-75257386	USP54
A_23_P51051	6.7865624	6.4249663	NM_001105537	chr2:219502829-219502770	ZNF142
A_33_P3394140	7.084009	6.407298	NR_024431	chr10:80703155-80703096	ZMIZ1-AS1
A_32_P18547	5.5545835	5.4646254	NM_058181	chr21:47711262-47711321	YBEY
A_33_P3313899	4.9270654	4.313105		chr20:4176271-4176330	LOC728228
A_23_P166333	11.944817	12.010876	NM_005659	chr22:19442346-19442287	UFD1L
A_33_P3335624	11.872422	11.9243355		chrX:107331777-107331718	PSMD10
A_23_P369983	8.001765	7.8231206	NM_174905	chr19:38899560-38899619	FAM98C
A_23_P4944	8.627401	9.034078	NM_005184	chr19:47113758-47113817	CALM3
A_23_P144497	15.56676	15.523151	NM_001006	chr4:152024154-152024213	RPS3A
A_23_P136573	4.5091066	4.839961	NM_003896	chr2:86067218-86067159	ST3GAL5
A_23_P75453	6.275834	6.1784782	NM_130803	chr11:64571621-64571562	MEN1
A_23_P167599	4.61145	3.343542	NM_001034850	chr5:16473404-16473345	FAM134B
A_23_P358009	5.6204033	5.71668	NM_015291	chr1:15897649-15897708	DNAJC16
A_23_P212715	4.579202	5.521083	NM_170662	chr3:105377554-105377495	CBLB
A_23_P32938	8.379414	7.9862137	NM_004398	chr11:108811424-108811483	DDX10
A_33_P3429242	2.5618458	2.3900566	XR_249565	chr4:6689284-6689225	
A_24_P189458	8.443624	8.482451	NM_017615	chr10:123727211-123724883	NSMCE4A
A_24_P177964	8.978119	9.096307	NM_001037806	chr12:50185006-50184947	NCKAP5L
A_23_P54846	11.403077	11.148535	NM_014685	chr16:56977700-56977759	HERPUD1
A_23_P61280	5.171273	5.248654	NM_012133	chr7:130146447-130146388	COPG2
A_24_P299996	5.7996216	5.994632	NM_018130	chr3:72799437-72799378	SHQ1
A_33_P3322050	4.5190716	4.6260905	NM_130438	chr21:38877801-38877860	DYRK1A

A_24_P141786	5.7720876	6.186641	NM_001681	chr12:110783124-110783183	ATP2A2
A_23_P122439	7.307734	7.0301604	NM_181531	chr6:26394026-26394085	BTN2A2
A_33_P3317628	10.007814	9.769509	NM_007183	chr11:404848-404907	PKP3
A_23_P162037	6.9915314	7.337787	NM_001030273	chr11:13408583-13408642	ARNTL
A_33_P3340868	4.4134135	4.7594438		chr19:35067869-35067810	SCGB1B2P
A_23_P92552	7.935431	7.944779	NM_004564	chr4:152593934-152592416	PET112
A_33_P3399840	11.274025	11.008577	NM_006590	chr2:85876234-85876293	USP39
A_23_P331813	4.9509196	4.245359	NM_020832	chr1:151263976-151264035	ZNF687
A_24_P221366	12.868079	12.917785	NM_001019	chr16:18800358-18799461	RPS15A
A_23_P126037	7.3998003	7.996203	NM_012421	chr1:40706263-40706322	RLF
A_24_P71661	3.8970232	2.3900566	NM_006371	chr3:33188806-33188865	CRTAP
A_33_P3351510	3.5732112	2.9129467	NM_014271	chrX:29973954-29974013	IL1RAPL1
A_33_P3314166	4.6667156	3.7336276		chr10:74857881-74857822	XLOC_008852
A_32_P35512	11.67613	11.534663	NM_003142	chr2:170663537-170665006	SSB
A_23_P169934	5.587535	5.8785725	NM_178314	chr12:123956985-123956926	RILPL1
A_23_P63402	7.9802647	8.45166	NM_013296	chr1:109466754-109466813	GPSM2
A_33_P3266265	8.153017	7.8214617	NM_001025780	chr9:74480188-74480129	ABHD17B
A_33_P3736691	7.2914166	7.634221	NM_025189	chr19:21240166-21240225	ZNF430
A_33_P3397795	12.164696	12.135965	AK095489	chr14:60635679-60635738	PCNXL4
A_23_P38649	3.7691913	3.6511471	NM_000529	chr18:13884816-13884757	MC2R
A_33_P3416574	6.5383635	6.9317365	NM_016108	chr6:143458067-143458126	AIG1
A_33_P3246198	3.4791818	4.503216	NM_001010912	chr10:124457287-124457228	C10orf120
A_23_P90311	9.126371	9.227913	NM_182919	chr19:4816054-4815995	TICAM1
A_24_P77968	5.9704733	5.6194897	NM_001458	chr7:128498476-128498535	FLNC
A_33_P3240867	3.7532964	3.5746741	NM_002929	chr13:114324024-114324083	GRK1
A_33_P3630780	3.6824799	2.930361	AL833160	chr8:22536609-22536550	LOC286058
A_33_P3334635	7.390796	7.8565383	NM_001286076	chr1:28218648-28218589	RPA2
A_33_P3315243	4.253704	3.7480354	NM_001164257	chr17:62080413-62080472	C17orf72
A_33_P3369461	5.13186	5.192261	NM_020703	chr1:110049749-110049690	AMIGO1
A_24_P379727	7.3370876	7.016654	NM_032309	chr2:113343632-113343691	CHCHD5
A_33_P3267230	6.750862	6.9626217	NM_001040282	chr17:34797442-34797383	TBC1D3G
A_33_P3863935	3.939814	4.03525		chr10:89605232-89605291	CFL1P1
A_33_P3324949	5.4666405	5.668032	NR_027317	chr3:185435895-185435954	C3orf65
A_23_P156017	9.086329	9.55552	NM_022130	chr5:32125336-32125277	GOLPH3
A_23_P9875	3.6812403	3.6609855	NM_007170	chr1:45809893-45809834	TESK2
A_23_P166336	7.181946	7.2174025	NR_026815	chr22:21058825-21058884	TMEM191A
A_23_P211850	7.626584	7.7109704	NM_020676	chr3:58280291-58280350	ABHD6
A_24_P56467	10.579756	10.618973	NM_001002000	chr14:24708342-24708401	GMPR2
A_32_P154021	4.879803	5.033678	NM_015879	chr18:55036069-55036128	ST8SIA3
A_23_P401606	2.3221061	2.3900566	NM_005711	chr5:83238522-83238463	EDIL3
A_33_P3272663	5.2670584	5.221714	AK130940	chr7:95778898-95778839	
A_24_P406714	7.880964	8.150106	NM_004641	chr10:22031960-22032019	MLLT10
A_24_P270496	2.832371	2.3900566	NM_006491	chr14:26941540-26939652	NOVA1
A_23_P9485	4.27268	3.986652	NM_000608	chr9:117093905-117094115	ORM2
A_23_P128783	8.424352	8.551384	NM_018453	chr14:34985315-34985256	EAPP
A_33_P3250553	2.3221061	2.3900566	NR_023925	chr18:1272503-1272444	LINC00470
A_24_P728006	4.8940053	4.668851		chr5:177482509-177482568	XLOC_014512
A_23_P327140	5.17881	4.4809246	NM_001256071	chr17:78369857-78369916	RNF213
A_24_P356601	5.97851	5.7295656	NM_006460	chr17:43229082-43229141	HEXIM1
A_23_P254081	7.5161796	7.328373	NM_006859	chr4:39478767-39478826	LIAS
A_23_P169039	7.750209	6.9673443	NM_003068	chr8:49830769-49830710	SNAI2
A_33_P3350892	5.6588993	5.405319	AK125987	chr9:140679701-140679642	LOC651337
A_23_P301984	3.636582	3.9990954	NM_001007090	chr8:13425461-13425520	C8orf48
A_23_P129413	8.735666	9.061154	NM_022357	chr16:68009640-68009581	DPEP3
A_23_P150648	4.349293	3.4319863		chr11:2893235-2893294	KCNQ1DN
A_23_P25097	9.713707	9.522331	NM_032338	chr12:66517590-66517531	LLPH
A_33_P3310784	3.2918825	2.3900566	NM_023003	chr15:83805706-83805765	TM6SF1
A_23_P393425	8.954513	9.059977	NM_173797	chr5:78981753-78981812	PAPD4
A_33_P3288189	10.004974	9.519755	NM_014899	chr5:95131797-95131856	RHOBTB3

A_33_P3797403	4.8453155	4.9259424	XR_247842	chr12:58263678-58263619	
A_33_P3288104	4.8772283	4.7777634	BC078153	chr6:39828878-39828937	DAAM2
A_33_P3210667	3.8789263	4.126025	NM_001256717	chr6:84303404-84303345	SNAP91
A_23_P96872	10.554776	10.653233	NM_016126	chr1:54389602-54387407	HSPB11
A_33_P3414202	4.0692396	4.103136	NM_001170704	chrX:131516295-131516236	MBNL3
A_23_P203947	5.447278	5.301297	NM_030653	chr12:31257193-31257252	DDX11
A_24_P348594	3.5486321	2.970314	NM_006953	chr22:45681840-45681899	UPK3A
A_23_P15844	6.540509	6.5772815	NM_032043	chr17:59760967-59760908	BRIP1
A_23_P3212	8.043175	7.9169664	NM_020234	chr15:49935617-49935676	DTWD1
A_23_P203645	6.941936	7.1738963	NM_001039618	chr11:85372650-85372591	CREBZF
A_33_P3240674	8.950091	9.07064	NM_007371	chr9:136905369-136905310	BRD3
A_33_P3325355	9.76096	9.415828	NM_006809	chr20:43571030-43570971	TOMM34
A_33_P3356792	5.027153	4.7194805	NM_178026	chr20:33439170-33439111	GGT7
A_24_P111912	7.2777843	7.2797995	NM_032042	chr5:92953995-92953936	FAM172A
A_33_P3223243	5.1885986	5.479686	AK301869	chr1:113160689-113160630	ST7L
A_32_P72341	9.671408	9.608115	NM_173084	chr3:160153585-160153526	TRIM59
A_33_P3273238	4.407175	3.9365559	NM_207333	chr19:53382828-53382769	ZNF320
A_23_P347048	7.184255	7.591739	NM_030791	chr14:64152108-64152049	SGPP1
A_33_P3359071	9.6467705	9.30622	NM_001082486	chr16:67691487-67691428	ACD
A_33_P3272361	9.501452	9.371264	NM_001281495	chr20:36500365-36500424	CTNNBL1
A_33_P3613688	6.1493683	6.3548594	BE729513		FER1L6-AS2
A_23_P84821	7.040935	6.6979	NM_020236	chr4:78804582-78804640	MRPL1
A_23_P15582	10.616048	10.744322	NM_022167	chr17:48438440-48438499	XYLT2
A_23_P168916	3.9207203	3.4543176	NM_001738	chr8:86240793-86240734	CA1
A_24_P365975	3.758151	4.0634394	NM_005202	chr1:36560922-36560863	COL8A2
A_23_P33072	5.623204	5.518435	NM_032272	chr8:145161055-145161114	MAF1
A_33_P3242952	7.923118	7.9750214	NM_001123168	chr1:206145518-206145577	FAM72A
A_23_P75741	5.4386253	5.4872293	NM_198183	chr11:57319956-57319897	UBE2L6
A_24_P79241	5.999906	5.968609	NM_014948	chr20:3088346-3088287	UBOX5
A_33_P3287263	11.933537	11.83872	NM_016451	chr11:14479168-14479109	COPB1
A_33_P3348752	11.130627	10.926246	NM_006644	chr13:31711568-31711509	HSPH1
A_23_P323943	6.449164	5.977857	NM_178498	chr11:26720020-26719961	SLC5A12
A_23_P88046	8.3991995	8.419093	NM_018210	chr13:111291990-111292049	CARKD
A_24_P158946	3.3903103	3.642939	NM_139241	chr12:32786493-32786552	FGD4
A_24_P39724	6.3532476	6.3399367	NM_144609	chr17:42755739-42755680	CCDC43
A_33_P3301075	3.9403746	3.5620682	S73507	chr14:106815814-106815755	
A_32_P70724	8.0136385	8.765957	NM_006618	chr1:202696804-202696745	KDM5B
A_33_P3336622	9.356319	8.887455	NM_001031806	chr17:19580832-19580891	ALDH3A2
A_24_P384029	7.8403835	8.155133		chr2:174296291-174296230	
A_24_P256037	12.153799	12.305746		chr17:025607775-025607836	
A_32_P41553	6.1688943	5.9742336	NM_144627	chr1:156316711-156316770	TSACC
A_24_P212443	4.634903	4.367496	NM_014671	chr7:156971521-156974250	UBE3C
A_32_P41405	9.460372	9.179697	NM_032361	chr5:175386833-175386774	THOC3
A_23_P15669	6.638522	6.576109	NM_001114118	chr17:3715413-3715354	C17orf85
A_23_P90997	5.4485435	5.390507	NM_148961	chr2:241078673-241078614	OTOS
A_33_P3245987	3.9398422	4.121409		chr20:045113671-045113730	
A_24_P192727	6.721819	6.3835096	XM_005270193	chr10:102827736-102827795	KAZALD1
A_24_P937790	3.8297448	3.7778175	NM_001162914	chr8:144789185-144789126	CCDC166
A_32_P212058	7.4449215	7.4947553	NM_032898	chr3:196434263-196434204	CEP19
A_33_P3417281	6.2815695	6.8157167	NM_018406	chr3:195506369-195506310	MUC4
A_33_P3281613	9.876237	9.917612	AK123276	chr9:138687062-138687121	KCNT1
A_24_P89701	11.72724	11.797928	NM_000883	chr7:128032432-128032373	IMPDH1
A_23_P18692	9.440915	9.253901	NM_000671	chr4:99992455-99992396	ADH5
A_23_P41541	7.146774	7.5864196	NM_138364	chr4:148560221-148560162	PRMT10
A_23_P155868	8.994392	9.426449	NM_006320	chr4:129192464-129192405	PGRMC2
A_33_P3274691	4.5165567	5.0221214	NR_002934	chr10:135278188-135278247	SCART1
A_33_P3259423	9.637137	9.642089	NM_017706	chr5:140050494-140050553	WDR55
A_33_P3403429	4.2275295	4.163724	NM_001129890	chr8:92231371-92231430	LRRC69
A_33_P3402116	9.638132	9.958397	NM_012199	chr1:36389840-36389899	AGO1

A_23_P150693	9.548103	9.191185	NM_014344	chr11:35642109-35642168	FJX1
A_33_P3320943	13.680013	13.174683		chr17:015410670-015410729	
A_33_P3273148	10.185372	10.184747	NM_052850	chr19:13065323-13065264	GADD45GIP1
A_23_P308632	4.5299096	4.253704	NM_152769	chr19:1230319-1230260	C19orf26
A_23_P163059	7.6262865	8.09897	NM_022495	chr14:60600911-60600970	PCNXL4
A_33_P3380567	10.938202	10.908442	NM_030974	chr8:145153595-145153536	SHARPIN
A_23_P212552	8.702091	9.163396	NM_024665	chr3:176739537-176739478	TBL1XR1
A_23_P27367	6.991062	7.4341383	NM_002035	chr18:60998933-60998874	KDSR
A_33_P3362981	5.3266206	5.0842814	NM_018992	chr16:2757433-2757491	KCTD5
A_33_P3406281	6.1456766	6.082225	NM_015474	chr20:35520818-35520759	SAMHD1
A_24_P181506	8.164579	8.11729	NM_014699	chr16:31094712-31094771	ZNF646
A_33_P3385912	4.204281	3.5783343			
A_23_P87279	5.355216	5.4546065	NM_014555	chr11:2427270-2426786	TRPM5
A_23_P415652	5.657012	5.949521	NM_024642	chr9:101608379-101611266	GALNT12
A_23_P342185	8.764149	9.069736	NM_015045	chr10:88197262-88197203	WAPAL
A_33_P3350196	2.7664027	2.8378458		chr14:074521876-074521935	
A_23_P88484	12.058304	11.92667	NM_001025248	chr15:48633763-48634258	DUT
A_33_P3383912	6.5370235	6.5457497	NM_022555		HLA-DRB3
A_33_P3867461	13.308332	13.062826		chr2:145834054-145834113	TEX41
A_33_P3209816	3.7879016	3.703727	NM_207325	chr19:32923635-32923694	DPY19L3
A_32_P157481	6.8428845	6.626496	NM_005236	chr16:14046046-14046105	ERCC4
A_24_P52921	6.471947	6.462069	NM_005504	chr12:24995040-24989496	BCAT1
A_33_P3371305	4.754453	4.6909204	XR_171697	chr7:151507832-151507891	LOC644090
A_33_P3319161	7.859679	7.910946	NM_181425	chr9:71679899-71679958	FXN
A_33_P3441583	10.303805	10.241947	BM453041		
A_33_P3264955	10.511777	10.626126		chr12:042825332-042825273	
A_33_P3401267	3.6021464	4.1382265	NM_001042548	chr2:36787990-36787931	FEZ2
A_24_P268015	7.982395	8.180351	NM_022756	chr1:37967528-37967469	MEAF6
A_33_P3328061	5.745919	5.440761		chr2:225363631-225363572	
A_32_P198731	7.188125	7.792561	NM_001142651	chr5:172118316-172118375	NEURL1B
A_23_P124190	3.7219412	3.1722214	NM_130390	chr11:5655079-5655869	TRIM34
A_23_P160177	6.8463473	6.609808	NM_144699	chr1:160151807-160156110	ATP1A4
A_33_P3265941	7.2255926	7.729416		chr5:012794301-012794242	
A_32_P4792	6.1646366	6.310614	XM_003959995	chr9:42427348-42427407	
A_33_P3291329	7.1178226	6.9216022		chr7:047093109-047093168	
A_23_P78526	8.983262	9.2499075	NM_020219	chr19:45184500-45184559	CEACAM19
A_33_P3275510	5.459754	5.365175	NM_152504	chr20:5844498-5844557	C20orf196
A_32_P130265	8.8969	9.462714	NM_014827	chr1:203822509-203822568	ZC3H11A
A_24_P7085	11.275572	11.336078	NR_002778	chr17:7657861-7657920	RPL29P2
A_33_P3357530	9.951485	9.536938	NM_006598	chr5:1050550-1050491	SLC12A7
A_23_P216766	6.7026134	7.166954	NM_030940	chr9:88879462-88879453	ISCA1
A_23_P409462	7.4903736	7.4610853	NM_173674	chr6:117859979-117860457	DCBLD1
A_33_P3347772	6.473879	6.675275	AK025163	chr2:242275516-242275575	SEPT2
A_33_P3242124	6.612988	6.6155086	NM_033417	chr19:17169471-17169412	HAUS8
A_23_P363647	5.4437594	5.5923405	NM_182540	chrX:134716021-134716080	DDX26B
A_33_P3307894	7.404705	7.5651436		chr1:043601475-043601534	
A_23_P16110	6.4676266	6.297844	NM_001079935	chr19:9362278-9362337	OR7E24
A_23_P417951	2.3221061	2.3900566	NM_033512	chr8:98285844-98285785	TSPYL5
A_24_P944458	5.7327466	5.753783	NM_016133	chr2:118866964-118867023	INSIG2
A_33_P3407990	5.6543145	4.9837446	AK096102	chr1:19727966-19727907	LOC100130193
A_23_P342138	8.811309	8.008226	NM_001040272	chr9:18910193-18910252	ADAMTSL1
A_24_P42693	2.3221061	2.3900566	NM_021187	chr19:16023422-16023363	CYP4F11
A_23_P252700	9.001504	9.13327	NM_014044	chr2:99226502-99227296	UNC50
A_33_P3326892	4.2390995	4.472822	AK130228	chr3:47645495-47645436	LOC100133032
A_33_P3390013	5.570482	4.9679008	NM_006988	chr21:28209959-28209900	ADAMTS1
A_32_P78783	4.6086936	4.544388	NM_001201407	chr16:89295210-89295269	ZNF778
A_33_P3385436	4.671253	4.4810905	NM_001029869	chr5:145464038-145463979	PLAC8L1
A_23_P9319	6.8726306	7.169269	NM_006717	chr9:91083472-91090003	SPIN1
A_23_P159920	6.8867087	6.519291	NM_003639	chrX:153792864-153792923	IKBKG

A_32_P515088	6.822432	6.515929	NM_002035	chr18:60995150-60995091	KDSR
A_33_P3340757	7.494179	7.655101		chr2:131451030-131450971	
A_24_P24002	6.863174	6.772848		chr4:076958140-076958081	
A_23_P386420	6.29346	5.6888685	NM_001516	chr12:124145133-124145192	GTF2H3
A_33_P3320079	7.9217024	7.894802	NM_005596	chr9:14120505-14120446	NFIB
A_33_P3302305	6.73878	7.22185	NM_000709	chr19:41930739-41930798	BCKDHA
A_33_P3240258	11.454461	9.3639555	NM_002951	chr20:35862438-35862497	RPN2
A_33_P3348887	12.019048	12.246999	NM_006195	chr9:128728209-128728268	PBX3
A_24_P298420	5.8506455	6.015543	NM_001039619	chr14:23393907-23393848	PRMT5
A_23_P122387	9.089195	8.769144	NM_018135	chr6:43639551-43639492	MRPS18A
A_23_P210300	10.643367	10.663321	NM_018263	chr2:25962434-25962375	ASXL2
A_24_P307486	12.412671	11.853804		chr6:031487938-031487879	
A_23_P47426	4.0651474	4.63756	NM_014384	chr11:134134935-134134994	ACAD8
A_23_P101742	9.643016	9.624003	NM_172251	chr19:3765301-3767289	MRPL54
A_23_P17512	10.295642	10.198396	NM_080820	chr20:18744415-18744474	DTD1
A_33_P3235925	7.286704	7.2093344	NM_024653	chr7:102065833-102065892	PRKRIP1
A_32_P12610	6.8721066	7.057898	NM_198256	chr2:11586520-11586461	E2F6
A_23_P149470	8.663328	9.203596	NM_004550	chr1:161180137-161180397	NDUFS2
A_23_P67971	5.6084785	5.2630157	NM_138801	chr2:38961144-38961203	GALM
A_23_P256835	7.4758234	7.805396	NM_016030	chr2:3481541-3482650	TRAPPC12
A_33_P3390739	9.093024	8.727272	NM_003932	chr22:41220563-41220523	ST13
A_33_P3373243	4.7585473	4.364402	XR_110135	chr16:34258499-34258558	MGC34800
A_23_P35820	10.125859	10.111377	NM_005507	chr11:65623523-65623464	CFL1
A_23_P200239	7.8272705	7.9507046	NM_018982	chr1:54317610-54317551	YIPF1
A_24_P137897	9.456871	9.12402	NM_001007245	chr7:112115517-112115576	IFRD1
A_24_P13083	5.8147087	5.661784	NM_130783	chr11:44952695-44952754	TSPAN18
A_32_P36942	5.102986	4.8840113	NM_001039693	chr2:200793780-200793721	TYW5
A_23_P131020	8.067129	7.535173	NM_017660	chr19:19617471-19617530	GATAD2A
A_23_P32125	10.089731	10.078162	NM_015160	chr9:139317760-139317819	PMPCA
A_33_P3302312	9.175742	9.334599	NM_203434	chr9:131939832-131939773	IER5L
A_23_P91350	5.918362	5.252795	NM_019593	chr20:5525220-5525161	GPCPD1
A_23_P384650	4.2130003	4.038496	NM_138454	chr19:17571533-17571474	NXNL1
A_33_P3353360	3.8171508	3.6066675	AK290580	chr3:10168101-10168042	BRK1
A_33_P3334828	5.683467	5.7527776	NM_001265587	chr19:17927383-17927324	INSL3
A_33_P3289396	5.3748417	5.423805	NR_028327	chr1:663090-663031	LOC100133331
A_23_P130352	5.5913086	5.7565885	NM_198991	chr18:24056609-24056550	KCTD1
A_23_P128744	2.3221061	2.3900566	NM_000710	chr14:96731011-96731070	BDKRB1
A_33_P3342106	3.758762	4.0009155	NM_015035	chr20:39833327-39833268	ZHX3
A_24_P391368	10.30513	10.406868	NM_013236	chr22:46136359-46136418	ATXN10
A_23_P203488	5.9062195	6.1741858	NM_000543	chr11:6415436-6415495	SMPD1
A_33_P3381259	7.62881	7.7200603		chr9:34524180-34524239	
A_33_P3336527	6.3663826	6.3289466	NM_001285449	chr16:15112762-15112821	PDXDC1
A_33_P3399788	4.234363	3.9543896	NM_001085	chr14:95090330-95090389	SERPINA3
A_23_P259621	10.255213	10.558995	NM_032464	chr7:73644076-73644135	LAT2
A_33_P3411632	8.998333	8.813844	NM_025268	chr14:105996478-105996537	TMEM121
A_24_P297480	5.8301144	5.778853	NM_178861	chr13:98828823-98828764	RNF113B
A_23_P127475	7.4548507	7.7622375	NM_005125	chr11:66373026-66373194	CCS
A_23_P145289	6.599884	6.3859663	NM_005275	chr6:30514921-30514587	GNL1
A_23_P306919	8.030988	8.237826	NM_001013845	chrX:149101863-149100950	CXorf40B
A_33_P3288180	6.7061543	6.5687633	NM_033489		CDK11B
A_33_P3270109	8.158091	8.153193	NM_001286430	chr15:52252156-52252097	LEO1
A_24_P244699	7.2541447	7.63547	NM_018283	chr13:48620499-48620558	NUDT15
A_33_P3372682	7.028169	7.2350373	NR_024009	chr22:46691169-46691110	GTSE1-AS1
A_23_P214222	10.5808325	9.75628	NM_002356	chr6:114182911-114182970	MARCKS
A_23_P7402	6.472745	6.135225	NM_178140	chr5:32110743-32110802	PDZD2
A_23_P78014	6.9742336	7.0262094	NM_016437	chr17:40815474-40817505	TUBG2
A_23_P209449	8.863168	8.855042	NM_003507	chr2:202902930-202902989	FZD7
A_24_P50437	12.189296	12.410598	BC065737	chr3:15183106-15183168	
A_33_P3339687	2.3221061	3.0457435	NM_024804	chr1:247264534-247264475	ZNF669

A_23_P46351	3.302893	4.605113	NM_006862	chr1:151746976-151746917	TDRKH
A_23_P155857	7.6843433	7.6401343	NM_198041	chr4:123814217-123814158	NUDT6
A_33_P3481113	4.8775144	4.697659	NR_038398	chr22:17737811-17737752	CECR3
A_33_P3387956	8.0198145	7.93393	NM_013379	chr9:140006471-140006412	DPP7
A_24_P50157	13.194869	13.357744		chr6:151546913-151546852	
A_33_P3379106	2.3221061	2.3900566		chr16:65318493-65318434	LINC00922
A_23_P82206	9.075807	9.042097	NM_018479	chr6:127611418-127611359	ECHDC1
A_24_P154868	3.9525404	3.4669645	NM_005588	chr6:46803182-46803241	MEP1A
A_23_P34548	6.35064	6.189319	NM_006642	chr1:243581295-243581354	SDCCAG8
A_23_P74887	4.4548426	3.9742303	NM_014654	chr1:31343300-31343241	SDC3
A_33_P3405103	6.3593807	6.8652315	NM_020912	chr16:2990093-2990152	FLYWCH1
A_33_P3253069	4.2569323	4.1747136	AF104255	chr6:131919475-131919416	MED23
A_23_P258570	9.247124	8.74052	NM_002814	chrX:107328209-107328150	PSMD10
A_23_P410998	6.4600964	6.4384136	NM_002868	chr12:56388026-56388085	RAB5B
A_33_P3304369	4.33457	3.7278504		chr1:149832991-149832932	
A_33_P3296499	5.7622967	5.8336086	NM_002844	chr6:128841541-128841482	PTPRK
A_32_P902988	6.040458	6.0503607	NM_002379	chr1:31184277-31184218	MATN1
A_23_P9152	7.9185114	7.678155	NM_005772	chr9:4860885-4860943	RCL1
A_23_P109410	5.4071894	4.786025	NM_030573	chr22:21355565-21355038	THAP7
A_24_P247454	7.7740927	7.348427		chr2:190176666-190176607	
A_23_P168965	6.043935	6.328259	NM_019607	chr8:67592211-67592270	C8orf44
A_32_P109704	10.193796	10.194654	NM_004592	chr12:132250749-132250808	SFSWAP
A_33_P3347387	9.140743	8.875533		chr6:052553565-052553624	
A_33_P3393398	8.204636	8.615402		chr7:043083282-043083341	
A_32_P102935	3.9843283	2.3900566	NM_001008779	chr2:29063246-29063305	SPDYA
A_24_P12690	2.3221061	2.3900566	NM_194294	chr8:39873692-39873751	IDO2
A_23_P57807	7.957505	7.9925804	NM_014160	chr3:12624725-12624784	MKRN2
A_33_P3276068	12.362031	12.348225	AK002042	chr6:116816291-116816232	TRAPPC3L
A_33_P3265950	5.0976057	4.8897038	NM_005093	chr20:32232701-32232760	CBFA2T2
A_23_P206077	7.7499056	7.3280053	NM_022767	chr15:89174900-89174959	AEN
A_23_P164035	8.615402	8.627066	NM_002805	chr17:61907288-61907530	PSMC5
A_33_P3427102	10.911365	10.8946705	NM_153712	chr2:113289845-113289904	TTL
A_32_P68504	5.515504	4.9451456	NM_020923	chr2:207178891-207178950	ZDBF2
A_23_P154065	11.946984	11.868311	NM_006000	chr2:220115129-220115070	TUBA4A
A_24_P394420	3.6594696	4.237023	NM_022754	chr5:174954790-174954849	SFXN1
A_33_P3245665	4.4528284	4.6777945	AY611541	chr7:142334854-142334913	
A_23_P363936	9.059744	9.081562	NM_014278	chr4:128754250-128754309	HSPA4L
A_23_P166421	7.783592	7.9185114	NM_031937	chr22:30688234-30688175	TBC1D10A
A_33_P3228385	10.779298	10.964012	NM_016311	chr1:28562903-28562962	ATPIF1
A_33_P3356731	6.2364454	5.9903355	NM_015332	chr7:44422025-44421966	NUDCD3
A_33_P3235642	5.006343	4.969217	NM_001193272	chr1:205065870-205065811	RBBP5
A_32_P204722	8.44607	8.628889		chr16:074702590-074702649	
A_24_P372217	7.985032	7.458365	NM_007175	chr8:37614638-37614697	ERLIN2
A_24_P21831	6.254127	6.131026	NM_019591	chr12:133588001-133588060	ZNF26
A_33_P3410279	5.7394195	5.5783863	AK090793	chr13:99461622-99461563	DOCK9
A_23_P138835	5.4467254	5.0497556	NM_005186	chr11:64977906-64978326	CAPN1
A_33_P3344127	5.909662	5.609538	NM_003512	chr6:26124461-26124520	HIST1H2AC
A_33_P3282205	3.298787	3.3355749	AK026419	chr9:66565850-66565900	
A_23_P170626	8.514536	8.506681	NM_001011667	chr8:57131066-57131125	CHCHD7
A_33_P3296230	2.3221061	2.869752		chr12:28122587-28122646	
A_33_P3378630	4.135796	4.0934863	AK097743	chr10:44166784-44166725	LOC100131195
A_23_P170352	10.620188	10.25984	NM_002949	chr17:79674001-79674060	MRPL12
A_23_P340890	7.5813513	7.740572	NM_001195753	chr1:6693309-6693368	THAP3
A_23_P53724	3.9998786	3.7464414	NM_014718	chr12:7310251-7310559	CLSTN3
A_23_P146379	5.647729	5.800158	NM_012416	chr9:6012138-6012079	RANBP6
A_23_P328740	4.5241547	5.1649756	NM_001285486	chr2:97163478-97163419	NEURL3
A_33_P3356577	3.8693764	3.996953		chr20:1586579-1586520	
A_33_P3264846	5.1592855	5.4442797	NM_152703	chr7:92760649-92760590	SAMD9L
A_33_P3301980	10.582666	10.823296		chrX:135992058-135992117	

A_23_P133755	7.9166727	8.201474	NM_002726	chr6:105725885-105725826	PREP
A_23_P396115	3.5173705	3.376295	NM_173358	chrX:52677350-52674574	SSX7
A_23_P151046	3.191282	2.3900566	NM_002259	chr12:10598822-10598763	KLRC1
A_33_P3252206	8.62449	8.597941	NM_001128208	chr8:29995141-29995200	LEPROTL1
A_24_P405705	3.8624039	4.4103794	NM_000340	chr3:170714612-170714553	SLC2A2
A_23_P431410	11.117815	10.0028	NM_016836	chr2:161129085-161129026	RBMS1
A_33_P3226492	3.6114812	3.0992198	AF119891	chr3:128532812-128532753	RAB7A
A_33_P3278573	5.764454	5.383485	NM_024859	chrX:49022908-49022967	MAGIX
A_32_P194072	6.7211423	6.6835785	NM_015395	chr7:97846265-97846206	TECPR1
A_23_P250462	10.553295	10.850822	NM_001183	chrX:153664652-153664711	ATP6AP1
A_24_P7946	5.083715	5.3318396	NM_001037126	chr7:133059738-133160122	EXOC4
A_23_P63038	10.557524	10.705652	NM_022356	chr1:43212247-43212188	LEPRE1
A_33_P3326151	5.769009	5.50639		chr16:085219641-085219700	
A_33_P3372413	7.006257	7.143242		chr15:030297892-030297951	
A_33_P3247540	4.2415605	4.242708	NR_027420	chr21:9921687-9921628	LOC389834
A_23_P29994	10.6179905	10.63839	NM_203284	chr4:26432686-26432745	RBPJ
A_33_P3336780	7.6001005	7.0920453	NM_001282291	chr7:150744762-150744821	ABCB8
A_23_P120056	4.2186575	3.9061565	NM_033046	chr2:74653409-74653350	RTKN
A_33_P3294252	8.705528	8.438221	NM_005171	chr12:51214846-51214905	ATF1
A_32_P86150	8.025123	8.170004	NM_001025200	chr16:75238059-75238000	CTRB2
A_33_P3294103	4.9969707	5.00213		chrX:018913892-018913951	
A_23_P30363	10.293912	10.225909	NM_004199	chr5:131528367-131528308	P4HA2
A_33_P3314401	4.1823535	4.265895	NM_006580	chr3:190128216-190128275	CLDN16
A_23_P34606	6.2562675	6.8465133	NM_004958	chr1:11167551-11167492	MTOR
A_24_P58881	13.815597	13.165405		chr2:048110777-048110837	
A_33_P3371904	6.2572794	6.275092	NM_198188	chr9:119188214-119188155	ASTN2
A_23_P41359	6.743329	6.9054074	NM_014487	chr4:142152604-142153702	ZNF330
A_23_P69670	8.2343855	8.554045	NM_018366	chr4:6719061-6719120	BLOC1S4
A_23_P79769	5.6078753	6.2701325	NM_022161	chr20:61869765-61869824	BIRC7
A_33_P3297277	14.376529	14.430021		chr2:064893043-064893102	
A_23_P9458	7.3680067	7.2153597	NM_022490	chr9:37501711-37501770	POLR1E
A_33_P3220415	5.62877	5.776289	AK096026	chr9:34648451-34648510	GALT
A_32_P30649	6.528289	6.7226305	NM_004454	chr3:185764606-185764547	ETV5
A_23_P148057	7.972684	8.158335	NM_016032	chrX:128939478-128939419	ZDHHC9
A_24_P941268	5.062855	5.130788	NM_007220	chrX:15804586-15804645	CA5B
A_33_P3340990	6.9350595	7.4088945	NM_001012398	chr16:53525616-53525557	AKTIP
A_23_P168276	9.052754	9.287509	NM_004865	chr6:134308415-134308474	TBPL1
A_23_P80503	4.3135886	4.695207	NM_133631	chr3:78649450-78649391	ROBO1
A_23_P21838	11.969018	12.08983	NM_033133	chr17:40127069-40127128	CNP
A_32_P27706	8.130752	7.025601	NM_003659	chr2:178408390-178408449	AGPS
A_33_P3330666	8.800849	8.76256	AK094096	chr4:7103125-7103066	FLJ36777
A_23_P95879	14.811477	14.811477	NM_000999	chr17:72205433-72205986	RPL38
A_23_P78944	3.6462855	2.3900566	NM_000479	chr19:2251742-2251801	AMH
A_23_P101516	4.9247394	5.450938	NM_004977	chr19:50826632-50826573	KCNC3
A_33_P3237517	4.847998	5.543907	AK123473	chr6:87933582-87933641	ZNF292
A_33_P3401571	5.928192	6.1064982	NM_002457	chr11:1092337-1092396	MUC2
A_33_P3737504	4.863374	5.3962216	NM_004938	chr9:90322215-90322274	DAPK1
A_24_P322353	5.40104	6.2480726	NM_024430	chr18:43564417-43564358	PSTPIP2
A_33_P3229241	2.3221061	2.3900566	XM_005277398	chr1:149782180-149782121	HIST2H2BF
A_24_P97145	8.667028	8.517411	NM_020738	chr2:8869593-8869534	KIDINS220
A_24_P187023	14.639593	14.605461		chr19:003131203-003131262	
A_33_P3371650	5.499581	5.584559	BC052561	chr2:197034187-197034128	STK17B
A_23_P59005	9.448041	8.71396	NM_000593	chr6:32813269-32813210	TAP1
A_23_P380928	6.6300144	6.8822474	NM_001198793	chr3:9876825-9876884	ARPC4-TTLL3
A_23_P207387	6.4290123	6.355789	NM_032484	chr17:40341285-40341226	GHDC
A_24_P282108	8.863794	8.688721	NM_015113	chr17:3907936-3907877	ZZEF1
A_24_P147407	6.835293	6.871228	NM_153335	chr17:61780480-61780421	STRADA
A_23_P304171	9.28156	9.485801	NM_001145642	chr3:197399033-197398974	KIAA0226
A_32_P219116	8.237995	8.105259	NM_018451	chr13:25457465-25457406	CENPJ

A_23_P251680	8.870055	8.877682	NM_000754	chr22:19951712-19951771	COMT
A_33_P3219055	9.875971	9.665298	NM_001143936	chr15:67818833-67818892	C15orf61
A_33_P3396527	8.463739	8.034847	NM_006467	chr5:89810118-89810177	POLR3G
A_32_P6221	4.3876357	4.283028	NR_024249	chr11:67560120-67560061	FAM86C2P
A_23_P156025	7.018	6.7449064	NM_033267	chr5:2746395-2746336	IRX2
A_23_P8013	11.683837	11.617973	NM_003519	chr6:27775345-27775286	HIST1H2BL
A_33_P3306327	10.046393	9.661062		chr9:139463976-139464035	
A_33_P3346628	3.7795384	3.7177029	NM_001190479	chr15:40573704-40573645	ANKRD63
A_32_P204950	3.561575	4.0162206	BC043001	chr5:16183984-16184043	LOC401176
A_23_P65307	2.3221061	2.3900566	NM_032229	chr13:86368069-86368010	SLITRK6
A_23_P145153	9.400412	9.061487	NM_002598	chr6:170892222-170892163	PDCD2
A_33_P3424297	9.787374	9.67777	NM_016275	chr3:150344826-150344885	SELT
A_24_P36868	9.483844	9.410248	NM_025160	chr1:224573395-224573336	WDR26
A_23_P63929	8.927748	8.922491	NM_018117	chr10:122668613-122668672	WDR11
A_24_P134266	7.797292	7.621482	NM_001728	chr19:583119-583178	BSG
A_33_P3302428	9.958397	9.724287	NM_001142640	chr17:76101674-76101733	TNRC6C
A_33_P3235716	9.201099	9.543051	NM_080861	chr16:1826780-1826721	SPSB3
A_33_P3301524	9.216216	8.702801	NM_001100119	chr14:104164014-104163955	XRCC3
A_33_P3305974	6.985622	7.0396204	NM_003825	chr15:42823617-42823676	SNAP23
A_23_P215318	5.642849	4.8063297	NM_014396	chr7:38763832-38763773	VPS41
A_33_P3212839	7.5864196	7.487316		chr1:045425008-045425067	
A_33_P3311730	4.627167	4.5225496		chr2:132290164-132290105	
A_23_P200260	8.979401	8.601205	NM_014801	chr1:233119980-233119921	PCNXL2
A_33_P3306545	10.903114	11.236134	NM_001172705	chr11:10820638-10820579	EIF4G2
A_24_P157424	10.493952	10.390371	NM_007362	chr3:196662523-196662464	NCBP2
A_23_P1585	10.099486	9.4304	NM_004451	chr11:64084115-64084174	ESRRA
A_23_P23457	5.1067867	4.1836185	NM_001024215	chr1:16095094-16096931	FBLIM1
A_23_P23639	5.4965096	5.612168	NM_153259	chr1:85392254-85392195	MCOLN2
A_23_P91491	11.013473	10.766874	NM_021254	chr21:33974211-33974152	C21orf59
A_23_P141035	4.1732264	4.3852086	NM_005769	chr16:71572278-71572337	CHST4
A_32_P96134	7.7791004	7.799367	NM_015283	chr7:34969084-34969025	DPY19L1
A_23_P50108	10.635602	10.528363	NM_006101	chr18:2610798-2610857	NDC80
A_23_P145146	10.776985	10.509649	NM_002598	chr6:170886691-170886632	PDCD2
A_23_P395911	7.4573317	7.5953555	AK021860	chr19:39436636-39436577	FBXO17
A_23_P163408	7.482976	7.4734387	NM_020843	chr15:76640909-76640850	SCAPER
A_23_P168130	3.7640216	3.8570342	NM_054111	chr6:33689810-33689751	IP6K3
A_23_P160689	6.505162	6.591788	NM_018372	chr1:111490467-111490408	LRIF1
A_23_P313	6.7233744	6.703037	NM_012392	chr1:32096214-32096155	PEF1
A_24_P19268	11.563932	11.829413	NM_182515	chr19:21300714-21300773	ZNF714
A_24_P58122	7.258403	7.5532804	NM_015306	chr1:55532583-55532524	USP24
A_23_P126970	11.856677	11.710349	NM_006335	chr1:201938998-201939057	TIMM17A
A_23_P115190	6.565971	6.511041	NM_002506	chr1:115829353-115829294	NGF
A_24_P38702	6.107572	6.3552938	NM_145285	chr10:101292909-101292968	NKX2-3
A_23_P654	7.8799024	7.899977	NM_003443	chr1:16268719-16268660	ZBTB17
A_33_P3263841	7.624865	7.457054	NM_001278539	chr4:76404624-76404565	RCHY1
A_33_P3213508	3.8632615	3.23215	NM_001161834	chr7:50173722-50198642	C7orf72
A_33_P3416503	7.3632274	7.631653	NM_181843	chr11:67395764-67395705	NUDT8
A_23_P58606	11.376524	11.677778	NM_004060	chr5:162871009-162871068	CCNG1
A_24_P112377	3.81511	2.3900566	NM_138620	chr9:135521287-135517397	DDX31
A_33_P3295690	4.7029557	5.196659	NM_001080524	chr16:3543642-3543583	C16orf90
A_24_P802145	5.973983	5.4566154	NM_005544	chr2:227596677-227596618	IRS1
A_33_P3277228	4.9914947	4.9969707		chr12:009708641-009708700	
A_23_P152344	4.8746557	4.0183277	NM_001520	chr16:27473768-27473709	GTF3C1
A_23_P85893	8.588215	8.925818	NM_144580	chr1:156262743-156262684	C1orf85
A_33_P3236881	11.829413	12.143517	NM_001204088	chr1:19984882-19984941	MINOS1-NBL1
A_23_P7172	9.077146	8.662591	NM_018290	chr4:37863548-37863607	PGM2
A_33_P3260209	6.250575	6.2672176	CD674525	chr16:8941576-8941517	
A_33_P3372426	3.269073	3.3096228	NM_007038	chr21:28290319-28290260	ADAMTS5
A_33_P3391039	4.270148	4.5076966	NM_214711	chr4:71024217-71024276	C4orf40

A_23_P14389	6.710007	6.775287	NM_014977	chr14:23530338-23528670	ACIN1
A_33_P3291636	4.7157893	4.848303	NM_003426	chr22:20761079-20761138	ZNF74
A_32_P213459	2.522724	2.3900566	NM_006557	chr9:1057447-1057506	DMRT2
A_23_P330788	7.8514805	7.900839	NM_015075	chrX:53262123-53262064	IQSEC2
A_33_P3214705	8.771379	8.994227	NM_001100418	chr19:18701688-18702918	C19orf60
A_33_P3246774	4.3550024	4.5352592	XM_005274775	chrX:152584739-152584680	LOC649201
A_24_P57700	9.066474	9.062397	NM_015035	chr20:39807453-39807394	ZHX3
A_23_P121499	7.598882	7.5784254	NM_006005	chr4:6304435-6304494	WFS1
A_33_P3345479	6.363211	6.1169586	NR_027033	chr22:46505802-46505861	MIRLET7BHG
A_23_P42695	10.546509	10.23921	NM_024051	chr7:30536417-30536358	GGCT
A_24_P83615	5.8950424	5.9723973	NM_001033053	chr17:5424946-5424887	NLRP1
A_24_P357266	2.3221061	2.3900566	NM_005314	chrX:16171051-16171110	GRPR
A_33_P3222769	8.477814	8.545942	NM_001013638	chr16:863802-863861	PRR25
A_24_P268160	7.725437	7.953441	NM_178454	chr1:111662564-111662505	DRAM2
A_23_P209689	8.148366	7.4407187	NM_004850	chr2:11322103-11322044	ROCK2
A_23_P217367	6.5278416	6.466844	NM_052936	chrX:107397546-107397605	ATG4A
A_32_P231179	3.703293	3.1236992	NM_144705	chr2:95540731-95541379	TEKT4
A_33_P3405004	8.102255	7.759449	NM_001278624	chr4:47849447-47849388	NFXL1
A_23_P346302	10.371544	9.773872	NM_144723	chr5:140086002-140086061	ZMAT2
A_24_P48204	4.263318	3.3259525	NM_003004	chr17:80279241-80279182	SECTM1
A_33_P3264815	9.730131	9.662343	NM_015059	chr15:63136731-63136790	TLN2
A_33_P3345886	3.6774855	4.0237575	AK131079	chr6:36990760-36990819	FGD2
A_24_P22488	5.098238	4.6838646	NM_003502	chr16:339454-338204	AXIN1
A_23_P148121	7.6255507	7.3321133	NM_001099409	chr11:65357976-65358035	EHBP1L1
A_33_P3221111	4.6427674	4.617945	AL832946	chr11:60978471-60978530	PGA3
A_32_P37867	5.712127	4.881247	NM_001099294	chr22:44640249-44640190	KIAA1644
A_33_P3239184	10.585618	10.650705		chr19:011697228-011697287	
A_23_P376627	7.988392	7.9268246	NM_152783	chr2:242708019-242708078	D2HGDH
A_33_P3351062	5.3560715	5.6010923		chr17:039114704-039114645	
A_24_P167642	4.650141	3.7923377	NM_000161	chr14:55309447-55309388	GCH1
A_24_P242646	2.3761332	2.3900566	NM_004079	chr1:150703572-150703513	CTSS
A_33_P3258752	3.6342437	3.9702024		chr1:170479973-170479914	
A_23_P124760	5.3874063	5.2588563	NM_014815	chr17:38178296-38178237	MED24
A_23_P404565	7.671503	8.181284	NM_030807	chr22:24227187-24227246	SLC2A11
A_23_P60488	8.287753	8.498264	NM_002540	chr9:131263074-131263133	ODF2
A_33_P3268343	14.247091	14.293259	NM_001029891	chrX:77223520-77223461	PGAM4
A_32_P319200	5.4669466	5.4211216	NM_199127	chr22:22989625-22989684	GGTLC2
A_23_P203137	10.301746	10.321202	NM_004788	chr11:118269573-118269632	UBE4A
A_33_P3395743	6.7155766	5.770295	NM_022834	chr1:1376086-1376145	VWA1
A_32_P59811	5.576661	5.4163404	CU692621	chr12:112843816-112844131	
A_33_P3237359	7.650261	8.261554	NM_005342	chrX:150156573-150156632	HMGB3
A_24_P280803	14.592072	14.545278	BC018140	chr20:60963498-60963557	RPS21
A_32_P185481	4.130846	3.4880626	AK127494	chr19:7096467-7096408	
A_24_P355944	9.196998	9.160404	NM_004093	chr13:107142795-107142736	EFNB2
A_23_P385126	9.026676	9.2067795	NM_139160	chr11:33054824-33054883	DEPDC7
A_33_P3278951	4.686343	4.9884644	NM_033512	chr8:98288935-98288876	TSPYL5
A_33_P3360972	5.406355	5.261828	NM_001077594	chr14:103576830-103576889	EXOC3L4
A_33_P3275722	5.019925	5.1391253	NM_021246	chr6:31685522-31685581	LY6G6D
A_32_P116989	3.842588	3.771936	NM_001143978	chrX:103360079-103360138	ZCCHC18
A_23_P218058	3.386463	4.143141	NM_013431	chr12:10560299-10560240	KLRC4
A_24_P40417	8.609337	8.680506	NM_002024	chrX:147031752-147031811	FMR1
A_33_P3358228	5.5799336	5.273772	AK122716	chr10:116049614-116049673	VWA2
A_33_P3317282	7.5968137	8.1280775		chr6:118028262-118028321	NUS1
A_24_P32085	4.1493225	2.3900566	NM_024761	chr9:27326043-27325984	MOB3B
A_23_P101796	4.9476676	5.412567	NM_033025	chr19:15225033-15225092	SYDE1
A_33_P3220247	5.8653693	5.9024568	NM_001003818	chr11:5632513-5632572	TRIM6
A_33_P3328814	4.980319	4.210499	NM_001130110	chr18:42456659-42456718	SETBP1
A_23_P216476	7.510902	7.7474575	NM_014872	chr9:37438478-37438419	ZBTB5
A_23_P317683	8.144626	7.865557	NM_003274	chr21:45525688-45525747	TRAPPC10

A_33_P3277674	5.1982045	5.031308	NM_203373	chr15:63894440-63894499	FBXL22
A_33_P3294002	9.89963	10.260525	NM_017436	chr22:43088187-43088128	A4GALT
A_24_P4334	5.4920416	5.917979	NM_194328	chr9:36344905-36344846	RNF38
A_33_P3323501	2.8113658	3.6188548	NM_001145643	chr15:40648544-40648603	PHGR1
A_23_P376759	7.925637	8.130752	NM_003584	chr2:73993659-73993600	DUSP11
A_23_P250994	8.3428135	8.23133	NM_014885	chr4:145916726-145916667	ANAPC10
A_23_P432626	6.373664	6.2872257	NM_018843	chr7:87473144-87473085	SLC25A40
A_33_P3242264	8.21424	7.84158	NM_199044	chr1:46830607-46830666	NSUN4
A_33_P3242069	3.7943053	4.1479526	NM_001282301	chr16:60392506-60392447	LOC729159
A_24_P402588	3.3403485	2.9885273	NM_018014	chr2:60679374-60679315	BCL11A
A_23_P200976	9.252088	9.368971	NM_001190880	chr1:43917153-43917094	HYI
A_33_P3210909	7.944779	8.073656	NM_001278946	chr3:10353709-10353650	SEC13
A_33_P3263359	4.712425	4.8814545	BC008292	chr12:56322041-56322100	DGKA
A_33_P3347161	7.0144687	7.3446684	NM_005647	chrX:9686805-9686864	TBL1X
A_24_P148907	7.3754406	7.703006	NM_006439	chr4:151504991-151505050	MAB21L2
A_33_P3244181	10.835839	11.0706005	NM_001537	chr16:83842916-83842975	HSBP1
A_23_P388993	7.0884132	6.815504	NM_033390	chr11:110042213-110042272	ZC3H12C
A_33_P3265290	14.478864	14.334603	NM_000986	chr3:101401333-101401274	RPL24
A_23_P19226	8.663836	8.621458	NM_013352	chr6:116758440-116758499	DSE
A_33_P3337719	11.296013	11.32902	NR_004846	chr20:26002105-26002164	LOC100134868
A_23_P128281	2.3221061	2.3900566	NM_007333	chr12:10569289-10568358	KLRC3
A_33_P3401169	5.107503	4.9247394		chr9:33629473-33629532	
A_24_P358591	5.2499743	4.5703783	NM_001105519	chr2:26802176-26802235	C2orf70
A_24_P267997	7.073782	6.7084966	NM_015602	chr1:179887170-179887229	TOR1AIP1
A_24_P363802	9.939497	10.22349	NM_005047	chr9:123579059-123579000	PSMD5
A_24_P328819	6.3931236	6.749457	NM_199053	chr4:184627970-184628029	TRAPPC11
A_24_P160969	4.8090596	5.459041	NM_001258320	chr11:44959766-44959114	TP53I11
A_33_P3413098	7.2423673	6.9254966	NR_024618	chr3:122611203-122611262	LOC100129550
A_33_P3260969	8.490349	8.574082	NM_145202	chr10:135165987-135166046	PRAP1
A_23_P214474	10.179983	10.372008	NM_001134493	chr6:41756987-41757046	TOMM6
A_23_P65427	13.437158	13.517996	NM_002818	chr14:24612636-24612577	PSME2
A_33_P3245168	4.476694	4.902321	NM_001128926	chr7:128323243-128323302	FAM71F2
A_32_P202859	6.0902963	6.357239	NM_181788	chr12:48723983-48724042	H1FNT
A_24_P940115	7.3342414	7.785181	NM_182643	chr8:12941781-12941722	DLC1
A_23_P27515	6.408484	6.4570866	NM_012268	chr19:40883752-40883911	PLD3
A_32_P75357	4.8597	4.3998265	XM_005258322	chr18:30321918-30314177	KLHL14
A_33_P3314192	6.094283	5.633725	NM_001184970	chr22:43267193-43267134	PACSIN2
A_24_P80138	9.884997	9.755492	NM_024065	chr2:101192942-101193001	PDCL3
A_32_P223777	5.649888	5.371787	NM_002184	chr5:55231193-55231134	IL6ST
A_23_P125596	3.5627303	4.099843	NM_014496	chrX:83319318-83319259	RPS6KA6
A_23_P256632	4.751215	5.1468105	NM_005023	chr5:114547953-114547894	PGGT1B
A_23_P65584	6.071398	6.2791376	NM_182476	chr14:74426183-74427892	COQ6
A_33_P3341821	4.049402	3.0834684	NM_176782	chr1:55075562-55075503	FAM151A
A_33_P3378291	5.195775	5.0206747	NM_001001694	chr22:50433061-50433002	IL17REL
A_23_P368278	6.8620243	7.551688	NM_014824	chr11:72548112-72548053	FCHSD2
A_23_P304511	2.7369056	2.3900566	NM_032347	chr18:32838208-32838267	ZNF397
A_23_P81463	8.942505	8.952178	NM_019030	chr5:54557327-54557268	DHX29
A_33_P3386242	3.659069	2.3900566	NM_006314	chr1:26516306-26516365	CNKSR1
A_23_P21134	4.644505	3.962978	NM_004083	chr12:57911526-57911200	DDIT3
A_24_P98385	7.997917	8.356657	NM_019592	chr9:104324212-104324271	RNF20
A_24_P361167	9.794712	9.97661	NM_020920	chr14:21853849-21853790	CHD8
A_33_P3216890	4.785174	4.762603	NM_018440	chr8:81882726-81882667	PAG1
A_33_P3389967	4.4536295	4.3488436	NM_001286554	chr6:41762166-41762107	USP49
A_23_P91015	5.693468	5.5775127	NM_006062	chr2:73453922-73453981	SMYD5
A_24_P32766	10.57857	10.30001		chr5:068630046-068630107	
A_23_P104594	4.0835133	3.3840654	NM_021920	chr11:626770-626711	SCT
A_33_P3407384	5.0889053	5.0976057	BC011404	chr1:33958004-33958063	ZSCAN20
A_23_P50646	5.7280655	5.226857	NM_001193621	chr19:44086139-44086198	PINLYP
A_33_P3367596	4.9356937	4.9055386	NM_001830	chrX:10201678-10201737	CLCN4

A_23_P66311	3.560853	3.3672404	NM_005223	chr16:3707081-3707215	DNASE1
A_33_P3235282	6.3734694	6.5964575	XR_110095	chr9:139511203-139511144	HCCAT4
A_33_P3323019	7.424062	7.6344852		chr1:023997604-023997663	
A_33_P3216955	5.8918805	5.5100803	NM_001242546	chr10:73995557-73995616	ANAPC16
A_33_P3343812	7.016654	7.162698	NM_001004453	chr9:125512830-125512889	OR1L6
A_23_P387552	6.0374002	5.78773	NM_000966	chr12:53604624-53604565	RARG
A_33_P3358908	6.987732	7.168803	NM_001277331	chr17:39422053-39422112	KRTAP9-6
A_24_P194886	8.452227	8.309734	NM_015252	chr2:63086426-63091924	EHBP1
A_23_P168229	12.049162	12.280785	NM_030810	chr6:7882079-7882020	TXNDC5
A_24_P33444	7.8056197	7.4426556	NM_006761	chr17:1257587-1257528	YWHAE
A_32_P54475	5.9663386	5.957659	CU676483	chr8:030210519-030210460	
A_33_P3329949	11.219689	11.126478	NM_004034	chr10:75135734-75135675	ANXA7
A_24_P77681	10.513466	9.988789	NM_006451	chr5:43533850-43529933	PAIP1
A_33_P3382887	8.538477	8.721946		chr18:058462067-058462008	
A_24_P339429	2.3221061	2.3900566	NM_021012	chr17:21323091-21323150	KCNJ12
A_23_P404162	4.270394	4.606224	NM_014707	chr7:18708002-18708061	HDAC9
A_23_P20196	8.858369	8.867144	NM_005720	chr7:98988593-98988652	ARPC1B
A_23_P377882	4.390802	4.489374	NM_172056	chr7:150647043-150646984	KCNH2
A_23_P78802	6.5646777	6.1952257	NM_016457	chr19:47181758-47181699	PRKD2
A_33_P3383856	6.5249357	6.801798	NM_080701	chrX:152710554-152710495	TREX2
A_24_P26554	5.991623	5.7236147	NM_020408	chr6:5108664-5108636	LYRM4
A_23_P11705	8.632089	8.973663	NM_018045	chr1:32830970-32830911	BSDC1
A_23_P73493	9.645744	10.188309	NM_004344	chrX:151996240-151996181	CETN2
A_23_P145238	9.037247	9.547407	NM_080593	chr6:27106250-27106191	HIST1H2BK
A_23_P13998	7.926305	7.9027658	NR_036632	chr12:100550425-100550366	GOLGA2P5
A_33_P3230339	6.9200745	7.047037	NM_007344	chr9:135251457-135251398	TTF1
A_33_P3212394	3.0258932	2.3900566	NM_001124759	chr3:75715831-75715890	FRG2C
A_23_P20443	10.01072	10.203238	NM_021020	chr8:20103761-20103702	LZTS1
A_33_P3802558	5.083472	5.0281353	BC015643	chr7:66020917-66020858	
A_23_P6818	4.1479526	4.0368276	NM_020163	chr3:52468201-52468142	SEMA3G
A_24_P194508	7.7489634	7.795208	NM_001006683	chrX:57146359-57146300	SPIN2B
A_33_P3268555	8.990462	8.826335	NM_001005176	chr2:231103722-231103781	SP140
A_23_P396867	7.761674	7.398059	NM_178582	chr20:30127182-30127241	HM13
A_23_P154345	8.828288	8.9273205	NM_014362	chr2:191069801-191069742	HIBCH
A_33_P3257042	3.3316953	3.1424992	NM_001193552	chr19:37240258-37240199	ZNF850
A_23_P414895	7.1672993	7.1450787	NM_018259	chr11:43469656-43471674	TTC17
A_24_P89891	4.9478765	4.9770308	NM_005658	chr9:123664733-123664674	TRAF1
A_33_P3323188	5.9428253	5.1087103		chr7:150109494-150109553	LOC728743
A_33_P3367541	8.288033	9.522642		chr7:53448296-53448237	XLOC_006444
A_33_P3607600	3.9636765	3.903494	DB304787	chr21:33749867-33749926	SNORA80
A_33_P3305399	5.151685	4.840287	NM_001243491	chr1:150917395-150917454	SETDB1
A_24_P310616	8.378291	8.571179	NM_001042498	chrX:48761226-48761167	SLC35A2
A_33_P3334121	6.746364	6.7344604	XM_005272511	chr1:144621976-144622035	LOC101929455
A_23_P42265	5.328274	4.697144	NM_019101	chr6:31625875-31625934	APOM
A_24_P308851	3.4487383	3.8888106	NM_032087	chr5:140764098-140764157	PCDHGA7
A_23_P135995	9.987476	10.105835	NM_022786	chr1:231136044-231136103	ARV1
A_33_P3374258	4.7212076	4.675468	AK056621	chr1:116381080-116381021	NHLH2
A_24_P341019	7.366257	7.374274	NM_001009924	chr20:5089998-5086918	TMEM230
A_32_P99753	6.1685944	6.016176	NM_207307	chr3:129120522-129120463	EFCAB12
A_33_P3490794	6.7092476	6.5433707	NR_027132	chr15:96051010-96051069	LINC00924
A_33_P3244269	4.486823	4.894861		chrX:102632170-102632111	
A_33_P3258581	7.0654063	7.115824	NR_024425	chr9:130892814-130892873	PTGES2-AS1
A_24_P304723	10.890676	10.896184	NM_000942	chr15:64452396-64452337	PPIB
A_23_P155765	12.9939575	13.373993	NM_002129	chr4:174253072-174253013	HMGB2
A_33_P3216237	10.208823	10.080989	NM_001159767	chr7:16745761-16745820	BZW2
A_33_P3278774	4.784997	4.538808	BC001868	chr19:12402029-12401970	
A_33_P3325131	7.042968	6.6814537	NM_001746	chr5:179137006-179137065	CANX
A_33_P3421418	5.8980055	5.6108627	XM_001717763	chr2:96517499-96517440	LOC100996862
A_23_P207842	6.0789557	6.4710865	NM_001024809	chr17:38513174-38513233	RARA

A_23_P100501	9.811309	9.814969	NM_002134	chr16:4559722-4559781	HMOX2
A_33_P3331856	2.752846	2.3900566	AK126467	chr7:31823215-31823156	PDE1C
A_33_P3417589	5.6401577	5.375417		chr6:162990701-162990760	
A_24_P307653	2.3221061	2.3900566	NM_182767	chr12:85254418-85254359	SLC6A15
A_33_P3351641	2.3221061	2.3900566	NM_198451	chrX:55651695-55651754	FOXR2
A_33_P3373707	6.519291	6.7233744	NM_003569	chr6:132791150-132791091	STX7
A_23_P122443	9.399312	9.6965065	NM_005319	chr6:26056109-26056050	HIST1H1C
A_33_P3399468	9.009753	9.261974	XR_243514	chr16:90235435-90235494	LOC100133182
A_23_P56314	12.495016	12.683207	NM_006830	chr19:1598153-1598094	UQCR11
A_23_P203299	12.208192	12.162217	NM_002901	chr11:32126817-32126876	RCN1
A_23_P335039	7.682659	7.8333907	NM_133474	chr4:433966-433907	ZNF721
A_23_P48217	8.648808	8.966608	NM_030817	chr12:12944179-12944238	APOLD1
A_32_P86739	7.467115	7.1501226	NM_001010911	chr10:21783485-21783426	CASC10
A_33_P3237729	7.7246323	6.7815537	NM_003687	chr5:131609069-131609128	PDLIM4
A_33_P3377229	4.6812544	4.82601	NM_001142946	chr11:2317566-2317507	C11orf21
A_33_P3285580	5.05112	4.463297	NM_145262	chr3:52329208-52329267	GLYCTK
A_23_P343826	4.5514517	4.7104797	NM_033449	chr5:141019875-141019816	FCHSD1
A_23_P78268	9.929586	10.092839	NM_016080	chr17:662753-662694	GLOD4
A_24_P225518	7.939837	7.732791	NM_001029885	chr1:1262622-1262681	GLTPD1
A_23_P432512	7.5145974	7.3391767	NM_148175	chr22:22051823-22051882	PPIL2
A_32_P1701	9.481033	9.297083	NM_016937	chrX:25014903-25014962	POLA1
A_33_P3356106	4.369715	4.272771		chr1:004686903-004686844	
A_24_P301629	5.961754	6.0135727	NM_003445	chr19:44501412-44501471	ZNF155
A_32_P100683	5.483524	5.7308474	NM_001145418	chr22:28374426-28374367	TTC28
A_33_P3301025	10.023302	10.148418	NM_000097	chr3:98298372-98298313	CPOX
A_33_P3322864	4.177399	4.0642867	NM_018645	chr2:239147560-239147501	HES6
A_33_P3296119	4.663101	4.4548426	NM_001198908	chr13:36822803-36822744	CCDC169
A_23_P94053	7.157545	7.526361	NM_003496	chr7:98610354-98610413	TRRAP
A_23_P111194	4.924016	4.711354	NM_012391	chr6:34506166-34506107	SPDEF
A_24_P100673	12.0849	12.069288	NM_016454	chr15:34522139-34522198	EMC4
A_33_P3384502	5.4424706	5.496687	NR_038944	chr22:49290848-49290907	LOC100128946
A_24_P158468	6.5294867	6.3323393	NM_173541	chr10:134261306-134261365	C10orf91
A_33_P3230676	4.1887226	4.229718		chr12:119919023-119918964	
A_23_P62807	11.294666	11.069655	NM_016002	chr1:246930666-246930725	SCCPDH
A_23_P137016	10.799177	11.380471	NM_002970	chrX:23804055-23804114	SAT1
A_23_P114808	6.12611	6.4257207	NM_001024732	chr1:29522753-29522510	MECR
A_23_P363968	5.904831	6.035947	NM_016546	chr12:7247616-7247557	C1RL
A_23_P204252	8.496976	8.11023	NM_002355	chr12:9093716-9093657	M6PR
A_24_P835500	9.5572815	7.942684	NM_001034841	chr16:19132844-19132903	ITPRIPL2
A_23_P259090	7.4396563	7.424576	NM_031438	chr5:102885275-102885216	NUDT12
A_23_P114144	6.226766	5.8950424	NM_014008	chrX:49105691-49105996	CCDC22
A_33_P3214072	5.6317306	5.6172504		chr4:24474070-24474011	PPARGC1A
A_23_P409626	5.088623	4.8920774	NM_001079559	chr11:62483033-62482974	HNRNPUL2
A_23_P3453	9.243197	9.029366	NM_015540	chr15:41809450-41809391	RPAP1
A_23_P159775	7.7735643	7.8300934	NM_004961	chrX:151121679-151121620	GABRE
A_24_P787889	7.0814586	7.2437177	NM_001242313	chr22:20380190-20380249	TMEM191B
A_33_P3268224	6.1169586	5.7044277	NM_145207	chr4:124240537-124240596	SPATA5
A_24_P916614	10.265017	10.048634	NM_005156	chr9:114981200-114981141	PTBP3
A_24_P288722	7.5559635	7.9280467	NM_003688	chrX:41379584-41379525	CASK
A_33_P3396444	6.8742104	6.948765	AK309450	chr9:114354706-114354647	PTGR1
A_24_P22079	4.381217	4.7212076	NM_002015	chr13:41130768-41130709	FOXO1
A_33_P3313660	7.466707	7.2275124	NR_002730	chr6:118824846-118824905	BRD7P3
A_33_P3245674	4.7004395	4.645342	XR_243958	chr19:58666162-58666103	ZNF329
A_23_P43255	11.258942	11.196339	NM_016623	chr8:130853853-130853794	FAM49B
A_23_P304543	6.511275	6.976555	NM_147133	chr9:33347038-33347097	NFX1
A_33_P3562537	7.5589304	7.6243515	NM_020630	chr10:43622213-43622272	RET
A_32_P65589	3.2441866	3.9121625	AJ252276		
A_33_P3334398	5.024291	5.147001	NM_001270500	chr1:9030977-9031036	CA6
A_33_P3360758	4.735733	4.531509		chr20:62044411-62044352	KCNQ2

A_33_P3414695	9.752775	9.718343	NM_016287	chr1:21103222-21103163	HP1BP3
A_33_P3342528	4.6110897	3.7543576	NM_002563	chr3:152554655-152554714	P2RY1
A_23_P147025	3.9962296	3.3935127	NM_004794	chrX:129318758-129318817	RAB33A
A_24_P227450	5.7548275	5.8431168	NM_025247	chr12:112193489-112193548	ACAD10
A_33_P3253070	3.6642196	4.1184773	NM_001270522	chr6:131919535-131919476	MED23
A_23_P64617	6.764489	6.3526225	NM_012193	chr11:86656859-86656800	FZD4
A_33_P3416150	5.6966233	5.54245	NR_027765	chr8:56362173-56362114	SBF1P1
A_23_P319050	2.4142158	2.3900566	NM_145326	chr19:21591532-21591591	ZNF493
A_33_P3213992	5.0037847	5.2413273		chr11:047863643-047863702	
A_23_P87323	8.135235	8.32017	NM_014186	chr11:36295823-36295764	COMMD9
A_24_P33552	14.087634	14.087634		chr6:133318782-133318723	
A_24_P58337	16.035227	15.963493	NM_002032	chr11:61732335-61732276	FTH1
A_24_P785293	10.18218	10.027787	NR_003098	chr11:62619632-62619573	SNHG1
A_23_P151653	8.910944	9.108621	NM_080649	chr14:20924246-20924871	APEX1
A_23_P428849	8.874333	8.459543	NM_144636	chr3:14153820-14153761	CHCHD4
A_32_P86180	5.7810907	5.6364613	NR_046407	chr6:105388387-105388328	LINC00577
A_33_P3413840	5.4681196	5.684789	NM_001205019	chrX:30739101-30746989	GK
A_23_P149649	10.632851	10.624737	NM_003000	chr1:17355135-17354342	SDHB
A_24_P395814	4.6860023	3.7948494	NM_000737	chr19:49526300-49526241	CGB
A_33_P3368328	11.926203	11.5660715	NM_001144831	chr12:7075646-7075587	PHB2
A_33_P3223759	9.282178	9.008872	NM_005121	chr17:60020094-60020035	MED13
A_33_P3374463	6.750681	7.0147424	NM_001242336	chr5:153434531-153434590	MFAP3
A_33_P3361851	10.437774	10.065823	NM_004637	chr3:128533580-128533639	RAB7A
A_33_P3345831	6.2206364	6.089078	NM_001010874	chr4:65144430-65144371	TECRL
A_23_P60286	6.4967732	6.613725	NM_001417	chr12:53427598-53427657	EIF4B
A_33_P3267665	8.8174	8.632089	NM_016353	chr8:17079944-17080003	ZDHHC2
A_23_P56759	7.9179077	8.151352	NM_016618	chr2:88327241-88327182	KRCC1
A_33_P3275055	5.1461415	4.5786757	NM_001160011	chr1:76878040-76878099	ST6GALNAC3
A_23_P300826	7.210372	7.210659	NM_145029	chr6:30619215-30620455	C6orf136
A_33_P3302165	9.558603	9.75067	NM_024527	chr19:17403000-17402941	ABHD8
A_33_P3268954	9.617048	9.656734	NR_024356	chr5:167957395-167957454	FBLL1
A_24_P66105	4.1338058	4.746731	NM_024859	chrX:49024088-49024147	MAGIX
A_32_P170621	4.168629	4.454238	NR_026941	chr10:119809060-119809119	CASC2
A_23_P308974	4.292216	3.7408202	NM_207517	chr15:84708037-84708096	ADAMTSL3
A_33_P3400943	2.3221061	2.3900566	NM_001199054	chr16:4560813-4560754	CDIP1
A_33_P3791118	3.855227	3.878002	BG113928		ATP5L2
A_33_P3336652	10.592379	10.56548	NM_001204054	chr11:77790702-77790643	NDUFC2
A_23_P95050	5.892348	6.147483	NM_001025595	chr4:153831352-153831411	ARFIP1
A_33_P3337044	11.876897	11.956767	NM_017585	chr9:136336528-136336469	SLC2A6
A_23_P137848	11.510384	11.347689	NM_145729	chr1:156707513-156707454	MRPL24
A_23_P410625	9.792476	9.616314	NM_153695	chr9:99157186-99157127	ZNF367
A_23_P396062	4.025467	4.37233	NM_021168	chr16:678322-678381	RAB40C
A_33_P3361707	7.043859	6.783073	NM_002392	chr12:69233570-69233629	MDM2
A_33_P3316233	3.5227022	3.0089178	NM_016067	chr4:84382817-84382876	MRPS18C
A_33_P3336925	6.291393	6.727592	NM_001256429	chr4:95376475-95376534	PDLIM5
A_24_P376339	6.9071007	6.824478	NM_001039577	chr1:1327768-1327709	CCNL2
A_23_P157404	6.6049633	6.6577177	NM_001283	chr7:100804062-100804121	AP1S1
A_33_P3292854	14.878323	14.851745	NM_004343	chr19:13055230-13055289	CALR
A_23_P107483	4.632517	4.9805007	NM_012373	chr17:3324509-3324568	OR3A3
A_33_P3416563	7.737872	8.031301	NM_016108	chr6:143654522-143654581	AIG1
A_32_P159334	6.550195	6.2138925	NM_002907	chr12:21621953-21621894	RECQL
A_23_P46149	7.0558953	7.46299	NM_003272	chr1:236371875-236371934	GPR137B
A_23_P93772	5.369791	5.1764216	NM_019102	chr7:27181606-27181547	HOXA5
A_23_P92602	6.669077	6.321783	NR_002451	chr4:419931-419872	ABCA11P
A_33_P3417123	7.16656	7.453931		chrUn_gl000235:000001907-000001848	
A_24_P398972	5.8412285	5.7357326	NM_016138	chr16:19091103-19091162	COQ7
A_23_P318396	7.5128117	7.4765577	NM_198700	chr11:47490237-47490178	CELF1
A_23_P134384	4.384908	3.6153648	NM_014660	chr7:11142499-11142558	PHF14

A_33_P3290687	8.200577	8.075748	NM_022365	chr10:22218037-22217978	DNAJC1
A_23_P211326	5.8331738	5.6358466	NM_031413	chr22:18033387-18033446	CECR2
A_23_P38154	10.761314	10.930143	NM_004110	chr17:72858709-72858650	FDXR
A_24_P213206	7.594404	7.406079	CR627448	chr6:109107021-109106962	
A_23_P162879	8.899624	9.030642	NM_032374	chr14:104056533-104056592	APOPT1
A_23_P257726	6.613725	7.031106	NM_016481	chr9:100672274-100667248	C9orf156
A_33_P3371769	9.591787	9.387093		chr7:102989031-102989090	
A_33_P3323453	6.7817435	7.0558953	NM_030789	chr20:30137075-30137134	HM13
A_24_P319715	10.016979	10.522473	NM_005742	chr2:10927471-10927412	PDIA6
A_33_P3393170	8.530695	8.595203	NM_004055	chr11:76837137-76837196	CAPN5
A_33_P3382111	5.888195	6.421357		chr1:227163542-227163483	
A_33_P3394395	7.571899	7.135456	NM_001270384	chr1:78343754-78343813	FAM73A
A_23_P131534	4.453865	4.549592	NM_007227	chr2:105859337-105859396	GPR45
A_23_P69810	10.266063	9.558947	NM_032717	chr4:84526584-84526643	AGPAT9
A_33_P3305158	7.4446206	7.1595716	NM_198484	chr3:40580859-40580918	ZNF621
A_23_P25215	9.8649435	9.836361	NM_032941	chr12:21623932-21623251	RECQL
A_33_P3341365	8.126349	8.076875	NM_207111	chr7:5662346-5662287	RNF216
A_33_P3358037	7.2136407	7.177294		chr8:031077477-031077418	
A_33_P3366246	8.047012	7.5879416	NM_020120	chr2:128948902-128948961	UGGT1
A_33_P3304170	4.967735	4.7201	NM_002649	chr7:106547292-106547351	PIK3CG
A_33_P3413732	4.226607	4.4445753		chr16:22528316-22528375	
A_23_P166677	9.325253	9.483844	NM_022736	chr3:158547311-158547370	MFSD1
A_33_P3342076	5.726768	5.6700006	NM_015866	chr1:14109169-14109228	PRDM2
A_24_P382001	8.327824	8.08048	NM_016310	chr16:97487-97428	POLR3K
A_24_P409042	8.821547	8.857646	NM_020240	chr5:130730020-130730079	CDC42SE2
A_33_P3227666	7.9065614	8.1664915	NM_018046	chr5:76360866-76360925	AGGF1
A_23_P201181	2.7581317	2.3900566	NM_012411	chr1:114376990-114376931	PTPN22
A_33_P3396459	4.9455924	4.6835628	NM_001278714	chr3:184081029-184081088	POLR2H
A_23_P19510	4.1116123	4.2810683	NM_001198858	chr6:32726794-32726735	HLA-DQB2
A_33_P3292540	10.287271	10.611015	NM_078626	chr1:51439963-51440022	CDKN2C
A_23_P218126	7.9274406	7.9930773	BC024289	chr14:106208310-106208251	
A_23_P401380	7.1653967	6.5538526	NM_173602	chr12:51141946-51142005	DIP2B
A_23_P37892	8.444057	8.137137	NM_133443	chr16:46964768-46964827	GPT2
A_23_P149099	9.067914	9.385847	NM_005216	chr1:20978961-20978902	DDOST
A_23_P150053	6.768422	6.939436	NM_001613	chr10:90697958-90697899	ACTA2
A_23_P311875	2.3221061	2.3900566	NM_006725	chr11:60787488-60787547	CD6
A_23_P38190	5.1532884	5.46885	NM_139280	chr17:38079397-38078912	ORMDL3
A_24_P359545	9.511093	9.1622305	NM_019610	chr1:89446550-89446491	RBMXL1
A_23_P35354	7.576757	7.3632274	NM_018294	chr10:101993032-101992973	CWF19L1
A_24_P329487	7.434521	7.3488903	NM_174911	chr8:127564939-127564880	FAM84B
A_24_P405621	6.4044824	6.4874387	NM_007184	chr3:52526378-52526437	NISCH
A_33_P3317321	8.207529	7.068528	NM_000367	chr6:18128639-18128580	TPMT
A_23_P214267	5.149114	5.6244025	NM_153840	chr6:46967904-46967845	GPR110
A_24_P202497	9.647271	9.722679	NM_020648	chr18:9402199-9402258	TWSG1
A_23_P304016	5.8085327	6.021639		chr9:45563001-45563060	LOC100132167
A_23_P166797	4.350723	4.604895	NM_022147	chr3:187089255-187089314	RTP4
A_33_P3789056	6.052743	6.22541	NR_027045	chr1:9712762-9712703	C1orf200
A_33_P3215647	5.1940513	5.4578905	NM_138371	chr12:47629913-47629972	PCED1B
A_24_P538478	4.8699656	5.0393543	NM_025205	chr4:17626088-17626147	MED28
A_24_P390583	7.07367	6.742723	NM_020718	chr16:23073163-23073104	USP31
A_33_P3312807	4.36453	4.114725	NR_023915	chr15:25367496-25367555	IPW
A_24_P786713	6.8633246	6.474785		chr12:034402591-034402650	
A_23_P313728	8.897478	9.149407	NM_015475	chr2:33809181-33809122	FAM98A
A_33_P3295917	4.527732	4.1133003	AB102885	chr8:79659322-79659263	IL7
A_33_P3316508	6.291986	6.070945	NM_001015051	chr6:45459689-45459748	RUNX2
A_33_P3271925	10.187136	10.258841	NM_006420	chr20:47652817-47652876	ARFGEF2
A_33_P3215093	5.053908	5.5441704	NM_001039840	chrX:72904088-72904147	CHIC1
A_33_P3317553	8.701495	8.785837	NR_036650	chr15:32822058-32821999	WHAMMP1
A_23_P1602	8.018768	7.193224	NM_006779	chr11:65089746-65089805	CDC42EP2

A_33_P3294222	3.1084669	2.3900566	NM_001013838	chr16:67690724-67690783	RLTPR
A_33_P3289491	3.6934814	4.482673	AK127359	chr11:124630128-124630069	LOC100129069
A_33_P3367251	5.935235	5.9670563	NM_001114395	chr9:17301768-17301827	CNTLN
A_33_P3227506	5.3318396	4.5935965	NM_182641	chr17:65980372-65980431	BPTF
A_23_P125042	4.7917786	4.882966	NM_013360	chr19:44536717-44536773	ZNF222
A_23_P143374	7.15423	7.174615	NM_025176	chr20:25433543-25433484	NINL
A_23_P331479	9.983906	10.137086	NM_133471	chr6:30644336-30644277	PPP1R18
A_33_P3362008	3.2609346	3.655799	NM_002521	chr1:11918391-11918332	NPPB
A_33_P3341524	5.1108904	4.727577	BC004960	chr2:74362402-74362461	
A_23_P38795	7.4488	6.9483476	NM_002029	chr19:52249233-52249174	FPR1
A_23_P54649	8.966608	8.917967	NM_003789	chr16:67188251-67188192	TRADD
A_33_P3683362	3.9217694	3.8262012	BC035844	chr18:29523959-29524018	
A_24_P297098	7.5441074	7.7789364	NM_016436	chr20:34537631-34537690	PHF20
A_33_P3404480	2.5206769	2.3900566	NM_033066	chr2:202510065-202510006	MPP4
A_23_P31407	4.8335714	4.868403	NM_006408	chr7:16839382-16837274	AGR2
A_33_P3373343	4.5497465	4.3535147	NM_001011719	chrX:2951291-2951350	ARSH
A_33_P3220570	11.333959	11.314068	NM_024954	chr10:99330900-99330959	UBTD1
A_23_P218375	9.548752	9.416584	NM_002208	chr17:3623668-3623609	ITGAE
A_33_P3367701	4.227474	4.296473	NM_032227	chrX:109416711-109416770	TMEM164
A_23_P163099	7.3540444	7.287184	NM_002692	chr14:50122470-50122411	POLE2
A_33_P3377294	7.8155136	8.13829		chr10:114905803-114905862	TCF7L2
A_23_P162336	10.228441	10.529908	NM_014515	chr12:70740091-70747654	CNOT2
A_24_P39639	6.0427823	5.949308	NM_058219	chr16:70284830-70284771	EXOSC6
A_33_P3420266	3.7817912	4.182713	NM_001012421	chr9:42368466-42368525	ANKRD20A2
A_33_P3403418	7.8741093	7.9347377	NM_001903	chr5:138266534-138266593	CTNNA1
A_32_P353072	8.223315	7.4035707	NM_018374	chr7:12276527-12276586	TMEM106B
A_33_P3445805	10.030988	9.833323	NM_001164160	chr11:68382661-68382720	PPP6R3
A_23_P164559	7.7062483	7.786029	NM_032436	chr13:115092406-115092465	CHAMP1
A_24_P303199	4.6728063	5.5862513	NM_019087	chr5:53181118-53181059	ARL15
A_33_P3283044	7.0275054	7.4946456	NM_015210	chr18:8831943-8832002	SOGA2
A_23_P26976	3.7426326	3.6852942	NM_001267	chr17:48542134-48542075	CHAD
A_24_P149036	11.275029	10.983267	NM_001387	chr5:146770596-146770537	DPYSL3
A_33_P3236778	5.634031	5.3424354		chr15:042302490-042302549	
A_33_P3237574	9.200206	9.567532	NM_001242758	chr6:29910343-29910402	HLA-A
A_23_P153745	11.1348	11.529684	NM_006332	chr19:18288840-18288899	IFI30
A_33_P3250857	4.364402	4.3331428	XR_247034	chr1:222885997-222885938	AIDA
A_23_P86838	7.4341383	7.4376683	NM_152313	chr11:92880992-92880933	SLC36A4
A_33_P3252925	8.222954	7.8446856	NM_199320	chr4:129796106-129796165	JADE1
A_23_P120942	13.25647	13.189826	NM_001469	chr22:42059942-42060001	XRCC6
A_24_P316102	6.756272	6.7715163	NM_001195630	chr10:21823724-21827811	MLLT10
A_32_P131640	7.3521037	7.1222467	NM_173078	chrX:142716215-142716156	SLITRK4
A_33_P3298861	3.6499975	4.3215594	NM_024628	chr3:124802842-124802783	SLC12A8
A_24_P144377	9.887742	9.864193	NM_014015	chr16:11022840-11022781	DEXI
A_33_P3399598	4.311646	4.106864	AK125109	chr14:74766285-74766344	ABCD4
A_23_P137856	6.5774593	6.654194	NM_002456	chr1:155159726-155158652	MUC1
A_23_P350886	7.93393	7.596741	NM_015349	chr6:42835927-42835986	GLTSCR1L
A_33_P3335999	6.7796063	6.8197575	NM_001199873	chr1:161141787-161141728	B4GALT3
A_33_P3372451	11.583111	11.612892	NM_199184	chr6:43193428-43193369	DNPH1
A_33_P3381292	3.637313	3.8754165		chr11:069924328-069924387	
A_23_P76159	8.589926	7.685139	NM_003566	chr12:93166521-93166462	EEA1
A_24_P388940	4.7013874	4.641884	NM_001042493	chr6:88051269-88051328	SMIM8
A_33_P3221303	5.3058987	5.072648	NM_016602	chr17:40831479-40831420	CCR10
A_23_P75978	6.9387684	7.3091335	NM_030813	chr11:72004607-72004548	CLPB
A_33_P3350488	10.704978	10.856181	NM_016359	chr15:41673178-41673237	NUSAP1
A_32_P449380	4.4418488	4.0744185	NM_001045476	chr9:127619070-127619129	WDR38
A_23_P162702	7.067271	7.119381	NM_001109662	chr12:112598483-112598424	HECTD4
A_32_P385587	5.400199	5.2938805	NM_000032	chrX:55035564-55035505	ALAS2
A_33_P3342126	6.4036627	5.97825	NM_020226	chr4:81125349-81125408	PRDM8
A_23_P74449	4.98754	4.372172	NM_032756	chr1:45794060-45794119	HPDL

A_33_P3210203	7.354584	7.75548	NM_032451	chr16:89937668-89937727	SPIRE2
A_33_P3240702	10.2566	9.984139	NM_002894	chr18:20606172-20606231	RBBP8
A_24_P326708	6.3680882	6.462418	NM_003957	chr11:1483037-1483096	BRSK2
A_23_P364355	9.579017	9.944758	X58329	chr7:142247389-142247330	
A_23_P94636	7.351409	7.4154487	NM_018835	chr9:125616832-125616509	RC3H2
A_24_P677642	6.078577	6.110897	NR_038275	chr10:51826458-51826399	FLJ31813
A_23_P420551	10.626126	10.7584715	NM_007174	chr12:120123690-120123631	CIT
A_33_P3257252	3.6906013	3.4548702	NM_138347	chr19:58199501-58199560	ZNF551
A_33_P3363655	4.9814086	5.1418796	NM_022662	chr2:112636447-112636388	ANAPC1
A_23_P1431	7.5152874	7.8386135	NM_004329	chr10:88683898-88683957	BMPR1A
A_33_P3322553	7.59466	7.636669	NM_030973	chr19:50338303-50338362	MED25
A_23_P43946	9.60426	9.645909	NM_033082	chr12:56151324-56151265	SARNP
A_23_P202750	6.6260314	6.3144464	NM_014039	chr11:93494794-93494853	C11orf54
A_23_P2942	3.4478812	3.2645879	NM_022123	chr14:34270706-34270765	NPAS3
A_32_P49668	4.42808	4.2914405	NR_073404	chr5:68932239-68932298	LOC441081
A_33_P3264835	4.195632	4.423244		chr1:21939590-21939531	RAP1GAP
A_32_P170925	5.5688586	6.9499354	NM_052883	chr3:126329890-126327428	TXNRD3
A_23_P143958	14.359379	14.1936245	NM_001099645	chr3:170584263-170584204	RPL22L1
A_33_P3254634	5.258525	5.225623	NM_006810	chr3:122821281-122821340	PDIA5
A_33_P3228762	9.526957	9.892845	NM_001104647	chr3:140695236-140695295	SLC25A36
A_33_P3321070	3.4132137	2.3900566		chr1:22453872-22453813	WNT4
A_24_P410605	5.7011313	5.5236974	NM_001083592	chr1:64608318-64608377	ROR1
A_33_P3294509	12.635663	12.419372	NM_000610	chr11:35253812-35253871	CD44
A_23_P46170	7.921773	8.077831	NM_201542	chr1:43849857-43849798	MED8
A_33_P3230350	9.196188	9.268351	NM_001098507	chr17:30696623-30696682	ZNF207
A_24_P219552	10.658477	10.741646	NM_003204	chr17:46138281-46138340	NFE2L1
A_24_P38895	13.446781	12.965004	NM_002105	chr11:118964650-118964591	H2AFX
A_24_P218587	6.360148	5.8859253	NM_004268	chr11:93546270-93546329	MED17
A_23_P394395	4.4964685	3.9255233	NM_020433	chr20:42740604-42740545	JPH2
A_24_P400842	7.047037	6.6035395	NM_207517	chr15:84506937-84527546	ADAMTSL3
A_23_P330461	4.461644	4.5004945	NM_144686	chr19:54664278-54664219	TMC4
A_23_P216549	7.3206153	7.1632843	NM_014806	chr9:35561526-35561585	RUSC2
A_33_P3283611	6.82775	6.7026134	NM_001549	chr10:91099762-91099821	IFIT3
A_24_P220897	5.3831263	5.313246	NM_194296	chr5:138732806-138732747	SPATA24
A_23_P6651	6.7981067	6.863174	NM_015224	chr3:56654650-56654591	FAM208A
A_33_P3231695	7.2825003	7.4114285	NM_022103	chr19:56951398-56951339	ZNF667
A_33_P3218832	3.6981108	3.996503	NM_014989	chr6:73112421-73112480	RIMS1
A_33_P3342653	4.9517794	4.8184643	NM_153202	chr20:3657774-3657715	ADAM33
A_23_P433079	7.3710966	7.0544443	NM_032364	chr12:56214981-56214922	DNAJC14
A_24_P941441	9.802197	10.023861	NM_006572	chr17:63007307-63007248	GNA13
A_24_P324314	7.0054936	7.2458653	NM_001008529	chr17:74681187-74679984	MXRA7
A_33_P3375398	7.6560106	7.6691027	NM_006453	chr16:2028684-2028743	TBL3
A_33_P3358799	7.5121803	7.6627927	NM_001281460	chr20:44978437-44978378	SLC35C2
A_33_P3282252	8.214201	8.220393		chr19:050313573-050313632	
A_32_P7316	5.6075444	6.536656	NM_170735	chr11:27677072-27677013	BDNF
A_33_P3364637	5.720607	5.6825027	AK124207	chr19:42747357-42747416	
A_33_P3419334	6.740136	6.900592	NM_006496	chr1:110136872-110136931	GNAI3
A_33_P3418010	9.818062	9.792053	NM_016553	chr19:50411422-50411363	NUP62
A_23_P27571	4.812732	4.8536925	NM_020533	chr19:7593764-7593823	MCOLN1
A_24_P417526	8.175956	7.9706473	NR_003579	chr20:29625889-29625948	FRG1B
A_33_P3409238	4.6938467	4.674185	XR_245638	chr6:168069968-168069909	LOC401286
A_23_P405282	8.460125	8.647604	NR_038359	chr19:51321674-51321733	MGC45922
A_33_P3304081	9.185856	9.2353325		chr5:139988837-139988896	
A_23_P382775	8.993092	9.40266	NM_014417	chr19:47724233-47724174	BBC3
A_23_P25305	4.2123327	4.209349	NM_004316	chr12:103353454-103353513	ASCL1
A_33_P3397051	4.2984324	5.179487	NM_175876	chr1:231471373-231471314	EXOC8
A_33_P3313532	5.0380445	4.7606754	NM_032250	chr9:67927016-67927074	ANKRD20A1
A_24_P5305	4.7044983	4.335478	NM_003119	chr16:89616903-89616962	SPG7
A_33_P3382565	4.8372684	4.916695	NM_018012	chr1:245865842-245865901	KIF26B

A_33_P3238225	5.8236294	6.5257196		chr3:137958316-137958375	
A_23_P500130	8.807254	8.584035	NM_153186	chr9:746006-746065	KANK1
A_33_P3405043	4.0948005	4.521297		chr10:121356646-121356705	
A_23_P500501	6.41612	6.502059	NM_000142	chr4:1810363-1810422	FGFR3
A_23_P73801	8.626282	8.666458	NM_001006640	chrX:102885333-102885392	TCEAL1
A_24_P194730	4.1601877	4.0854425	NM_181846	chr19:58852743-58852802	ZSCAN22
A_23_P254917	4.3601685	3.8658178	NM_006149	chr19:39292983-39292766	LGALS4
A_33_P3279610	6.224964	6.217305	NM_005205	chr16:31439151-31439092	COX6A2
A_33_P3360886	4.695286	4.7677927		chr13:044360275-044360216	
A_23_P60376	12.74947	12.908557	NM_153200	chr9:139757418-139757359	EDF1
A_23_P74467	8.881049	8.90164	NM_014949	chr1:155883487-155883428	KIAA0907
A_24_P125839	6.0021954	5.710717	NM_017447	chr21:19165778-19165719	C21orf91
A_33_P3355090	8.055392	7.809699	NM_080656	chr5:133745654-133745595	CDKN2AIPNL
A_23_P6708	10.461201	10.288172	NR_024388	chr3:196670329-196670388	NCBP2-AS2
A_33_P3358641	7.010505	6.8915024	NM_001137552	chr2:238672559-238672618	LRRFIP1
A_33_P3329149	5.4542656	5.507837	NM_031267	chr7:39990943-39991002	CDK13
A_33_P3270593	5.33595	5.5799336		chr1:028527792-028527851	
A_23_P88134	6.2645693	6.080374	NM_032630	chr14:102822196-102822137	CINP
A_23_P399897	3.4699893	3.2002938	NM_178554	chr3:134322495-134322436	KY
A_23_P200999	6.083721	6.1513586	NM_174963	chr1:44396560-44396619	ST3GAL3
A_23_P47073	7.8905044	7.575342	NM_014023	chr10:1177673-1177732	WDR37
A_33_P3228732	10.130083	10.366955	NR_024480	chr3:194023621-194023562	LINC00887
A_23_P138514	8.270276	8.126349	NM_012071	chr10:22608978-22609037	COMMD3
A_33_P3251672	4.5296726	3.819062	NM_001606	chr9:139918654-139918595	ABCA2
A_23_P386411	9.625171	9.359165	NM_005038	chr4:159630788-159630729	PPID
A_23_P400378	4.7381115	4.862933	NM_001077191	chr2:219128419-219128478	GPBAR1
A_33_P3232682	6.5962133	6.7424994		chr18:12229225-12229166	XLOC_012774
A_33_P3650224	4.7999916	4.8715506	NM_001145139	chrX:70984367-70984426	CXorf49B
A_23_P215534	4.7938843	4.4405904	NM_053043	chr7:155537677-155537736	RBM33
A_23_P139600	6.431377	7.384439	NM_004658	chr12:113537635-113537576	RASAL1
A_33_P3358183	8.260809	8.191027	NM_000959	chr1:78959166-78959225	PTGFR
A_23_P19702	10.676964	10.807848	NM_015093	chr6:149732565-149732624	TAB2
A_33_P3416882	4.3234615	3.6565032	NM_206919	chr4:57389956-57390015	ARL9
A_32_P114215	11.753389	11.858713	NM_203497	chr13:76100703-76100644	COMMD6
A_23_P144627	8.13185	8.025816	NM_018933	chr5:140595978-140596037	PCDHB13
A_33_P3390546	7.332256	7.4026613	AK130802	chr1:201474852-201474793	
A_24_P228149	4.0728226	4.0901523	NM_002274	chr17:39661309-39659920	KRT13
A_33_P3382924	11.547043	12.0555	NM_003118	chr5:151041099-151041040	SPARC
A_33_P3212782	13.44387	13.532852	NM_001743	chr2:47387937-47387878	CALM2
A_23_P58770	3.9082487	4.3340573	NM_004821	chr5:153854852-153854793	HAND1
A_23_P151436	10.16886	10.193796	NM_013338	chr13:37524173-37524114	ALG5
A_23_P124514	4.082787	4.2272363	NM_001364	chr11:83177764-83173091	DLG2
A_23_P148273	6.893416	6.465493	NM_032121	chrX:77084713-77084654	MAGT1
A_24_P164731	7.328692	7.1063495	NM_006858	chr19:10945784-10945725	TMED1
A_32_P524614	6.5755053	6.425431	NM_175734	chr17:7330813-7330872	C17orf74
A_23_P74114	7.209549	7.5161796	NM_182633	chr7:56007983-56008042	ZNF713
A_33_P3323136	3.8378344	3.957381	NM_145010	chr10:25304865-25304806	ENKUR
A_23_P148584	5.785183	5.5459576	NM_144658	chrX:117819952-117820011	DOCK11
A_24_P349965	8.364223	8.333238	NM_007109	chr6:31131799-31131858	TCF19
A_33_P3335391	3.9988756	3.2247522	NM_001039999	chr17:18874792-18874733	FAM83G
A_33_P3420904	6.417738	6.4968786	NM_001129883	chr11:125661436-125661495	PATE3
A_33_P3295283	9.503187	9.825703	NM_005768	chr12:7085411-7085352	LPCAT3
A_24_P64233	6.594767	6.658184	NM_000692	chr9:38397700-38397759	ALDH1B1
A_33_P3349495	5.034129	5.270671	NR_001552	chrY:7569288-7569229	TTTY16
A_23_P398566	2.9718513	2.3900566	NM_173200	chr9:102628985-102629044	NR4A3
A_32_P31992	7.8079786	7.733854		chr1:043369135-043369194	
A_33_P3326914	4.885882	4.9082055	NM_001080496	chr9:35752754-35752813	RGP1
A_23_P215931	9.428035	7.9766006	NM_015344	chr8:29965452-29965511	LEPROTL1
A_23_P390518	5.8273983	5.5542865	NM_003839	chr18:60053258-60053317	TNFRSF11A

A_23_P29046	9.698865	9.707726	NM_001757	chr21:37445281-37445340	CBR1
A_33_P3291891	3.4144351	4.242616	XM_005275728	chr12:131781526-131781585	LOC100996701
A_33_P3268716	8.170004	8.176308	NM_003099	chr15:64436277-64436336	SNX1
A_23_P157527	5.778853	5.5856934	NM_033402	chr8:86057728-86057787	LRRCC1
A_23_P421054	4.596987	5.374524	NM_001031743	chr6:88140808-88140867	C6orf165
A_24_P944299	8.284512	8.281887	NM_003607	chr1:227178114-227178055	CDC42BPA
A_23_P151321	13.57192	13.661747	NM_001024662	chr12:112846082-112844674	RPL6
A_23_P7282	8.400639	8.523925	NM_153702	chr4:141474758-141474817	ELMOD2
A_23_P363778	2.3221061	7.5700827	NM_001463	chr2:183699679-183699620	FRZB
A_23_P76350	5.162595	5.540573	NM_002075	chr12:6954921-6955969	GNB3
A_24_P717262	4.533799	4.8242035		chr19:023779543-023779602	
A_33_P3275495	5.4488754	5.198405	XR_108366	chr1:17521100-17521159	LOC400743
A_23_P9761	7.6397676	7.6986217	NM_001037144	chr17:7852418-7852704	CNTROB
A_23_P355289	8.35242	8.6497135	NR_003226	chr16:90062393-90062452	AFG3L1P
A_23_P104413	8.528778	8.475251	NM_033178	chr10:135481634-135484996	DUX4
A_33_P3268313	6.67606	6.9088264	NM_000290	chr7:44102471-44102412	PGAM2
A_23_P43350	3.8174968	3.586144	NM_005511	chr9:5909735-5909794	MLANA
A_32_P51787	4.481802	4.151778		chr2:113611162-113611221	
A_23_P4161	3.693215	3.6781213	AB023218	chr17:66418229-66418288	ARSG
A_23_P86386	3.3036416	3.364851	NM_024804	chr1:247264316-247264257	ZNF669
A_24_P320759	6.844386	6.63608	NM_024653	chr7:102066878-102066937	PRKRIP1
A_32_P167396	4.328869	3.883936	BC039399	chrX:40597776-40597835	
A_33_P3351097	7.939571	7.763868		chr3:182178446-182178387	
A_33_P3374165	3.779001	3.5355926	AK090994	chr5:170733121-170733062	LOC728145
A_24_P48627	7.646952	7.833254	AK022481	chr19:4039324-4039383	PIAS4
A_23_P82913	6.0650187	6.1705008	NM_017956	chr8:125464767-125464826	TRMT12
A_33_P3266022	3.8121443	3.3385193		chr11:085779706-085779765	
A_23_P501849	12.077678	12.098886	NM_033251	chr16:89629585-89629644	RPL13
A_23_P139585	5.0866437	5.1868625	NM_000924	chr12:54972858-54972917	PDE1B
A_23_P251695	10.458807	10.197037	NM_013248	chr20:23335235-23335294	NXT1
A_23_P167841	4.9027243	4.7073298	NM_019842	chr6:73905075-73905134	KCNQ5
A_33_P3219256	9.718343	9.550677	NM_004329	chr10:88684862-88684921	BMPR1A
A_23_P162120	4.373464	4.292216	NM_006185	chr11:71715302-71715118	NUMA1
A_24_P101226	4.6913385	4.328203		chr14:106610419-106610360	
A_24_P60441	5.0980396	5.5010977	NM_153717	chr4:5815695-5815754	EVC
A_23_P56170	4.9770308	4.649285	NM_005481	chr19:868484-868425	MED16
A_23_P435833	5.2470474	5.1349597	NM_003934	chr9:133507006-133507376	FUBP3
A_33_P3412294	4.111786	4.3757524	NM_181877	chr15:85166329-85166387	ZSCAN2
A_33_P3273136	8.873409	8.625935	NM_001031713	chr6:13807217-13807158	MCUR1
A_32_P110872	5.93692	6.0344667	NM_033110	chr13:101183916-101183857	GGACT
A_33_P3234043	4.2768583	4.222993	AK291620	chr8:22053703-22053762	BMP1
A_24_P398781	7.942684	8.1264925	NM_052873	chr14:76548991-76549584	IFT43
A_33_P3407065	8.404172	8.813404	NM_001243952	chr5:61681395-61681454	KIF2A
A_24_P683011	9.514803	9.579017	NM_001190972	chr8:91971239-91971180	C8orf88
A_24_P208081	3.9717727	4.7181644	NM_022819	chr1:20476009-20476068	PLA2G2F
A_23_P73763	12.659221	13.335219	NM_006014	chrX:153706252-153706193	LAGE3
A_23_P85188	7.628486	7.737872	NM_022838	chrX:101858957-101859016	ARMCX5
A_23_P153372	4.859888	4.599357	NM_032855	chr19:16269004-16269063	HSH2D
A_33_P3266564	4.392532	4.5235167	NM_001012416	chr11:1718926-1718985	KRTAP5-6
A_23_P252808	7.7663155	7.7672606	NM_012477	chr2:74687734-74687793	WBP1
A_23_P393713	3.1604557	3.4848223	NM_002180	chr11:68707319-68707378	IGHMBP2
A_33_P3537875	14.815807	14.750789	BC004943	chr19:18992209-18992150	MGC10814
A_23_P150286	12.204439	12.110717	NM_148976	chr11:14526628-14526569	PSMA1
A_23_P124742	8.271596	8.395311	NM_001277	chr11:67820509-67820450	CHKA
A_33_P3421984	7.387869	7.5998216	NM_012269	chr7:123485310-123485369	HYAL4
A_33_P3366589	4.9756703	5.188344	NM_001005181	chr11:6129817-6129876	OR56B4
A_24_P106112	8.623438	8.748145	NM_000297	chr4:88998599-88998658	PKD2
A_23_P404108	7.0859704	7.2055106	NM_018999	chr10:86277812-86277871	CCSER2
A_33_P3256347	11.260027	11.076153	NM_012459	chr11:111955927-111955869	TIMM8B

A_24_P237486	9.495665	9.5521	NM_004992	chrX:153287382-153287323	MECP2
A_23_P145114	7.7504845	7.257366	NM_001498	chr6:53362256-53362197	GCLC
A_33_P3285156	7.390375	7.6337376	NR_026857	chr17:79003098-79003039	BAIAP2-AS1
A_33_P3530314	8.513567	8.563876	NM_023073	chr5:37107443-37107384	C5orf42
A_33_P3379046	6.9884033	6.568947	XR_112442	chr2:222439103-222439162	LOC729770
A_33_P3234989	7.7267456	8.057941	NM_014055	chr12:110656447-110656506	IFT81
A_23_P211643	4.483186	4.7851133	NM_005036	chr22:46615754-46615813	PPARA
A_23_P352684	6.4868774	6.274075	NM_003861	chr14:69517702-69517643	DCAF5
A_23_P127891	7.2540617	8.046732	NM_170735	chr11:27679959-27679900	BDNF
A_24_P385313	10.887427	10.994911	NM_002840	chr1:44089183-44089242	PTPRF
A_33_P3243337	12.761178	12.774128	NM_019107	chr19:4657636-4657577	C19orf10
A_24_P380348	7.462387	7.3814206	NM_001044305	chr6:71570997-71571056	SMAP1
A_33_P3374180	4.3336215	3.3649092	XM_005260411	chr20:55108074-55108133	FAM209B
A_33_P3224971	13.101461	13.231004		chr3:075789929-075789988	
A_24_P236445	7.660679	8.395726	NM_006141	chr16:66755859-66755800	DYNC1LI2
A_23_P150428	4.8508296	4.6511917	NM_001005325	chr11:123676531-123676472	OR6M1
A_33_P3357149	7.0828576	7.765564	XM_005269918	chr10:88935792-88935851	FAM35A
A_33_P3339961	4.106864	3.2383351	NM_178547	chr1:33087364-33087305	ZBTB8OS
A_33_P3681213	5.807773	5.941796	NM_006036	chr2:44549028-44548969	PREPL
A_33_P3375576	3.8582757	4.216701	NM_003980	chr6:136667095-136667036	MAP7
A_33_P3247180	4.691503	4.700032	NM_032449	chr1:52820340-52820281	CC2D1B
A_23_P42684	4.2935834	3.3634183	XR_108766	chr7:33767896-33767955	FLJ20712
A_24_P80181	8.809408	8.775738	NM_017849	chr2:96916740-96916681	TMEM127
A_23_P25626	8.431492	8.990842	NM_024808	chr13:73329356-73329415	BORA
A_24_P382579	5.995548	5.9523335	NM_000915	chr20:3053096-3053155	OXT
A_33_P3351765	5.0452795	5.4866076	NM_014997	chr7:129773533-129773592	KLHDC10
A_33_P3298102	5.428817	5.490445		chr15:045613408-045613349	
A_23_P40240	8.613993	7.932238	NM_001336	chr20:57570420-57570361	CTSZ
A_24_P168398	5.5962615	5.564177	NM_003451	chr19:9492686-9492745	ZNF177
A_33_P3348884	5.647876	5.3080735	NM_173648	chr2:179698959-179698900	CCDC141
A_24_P5743	6.211321	6.005314	NM_153329	chr19:49972198-49973576	ALDH16A1
A_23_P376488	2.5288482	2.3900566	NM_000594	chr6:31545837-31545896	TNF
A_33_P3357818	2.3221061	2.3900566	NM_000864	chr1:23518447-23518388	HTR1D
A_33_P3269380	4.6194053	4.2234073		chr20:019193034-019192975	
A_33_P3275948	10.3044815	10.2655735	NM_032016	chr7:38269757-38269816	STARD3NL
A_23_P78980	3.9369044	4.0728226	NM_014256	chr19:17923824-17923883	B3GNT3
A_33_P3214042	3.8413146	3.0897925		chr11:064645881-064645940	
A_23_P258814	7.878978	8.149439	NM_080750	chr20:61477141-61477200	DPH3P1
A_33_P3357954	6.0477314	6.351428	NM_004956	chr7:13935691-13935632	ETV1
A_23_P43763	5.069796	5.3364983	NM_015993	chr16:57295873-57295814	PLLP
A_33_P3417810	10.453484	10.228036	NM_024894	chr2:10711841-10711782	NOL10
A_23_P218827	7.6401343	7.6574	NM_199420	chr3:121150761-121150702	POLQ
A_24_P269129	4.492836	4.3378344	NM_153746	chr6:158066818-158068296	ZDHHC14
A_33_P3230951	7.136251	7.06697		chr19:57359775-57359834	MIMT1
A_23_P88740	9.729539	9.852298	NM_018455	chr16:81053871-81056408	CENPN
A_23_P141965	9.8203745	9.892289	NM_033417	chr19:17160709-17160650	HAUS8
A_23_P351320	8.147998	8.0121565	NM_015891	chr6:110550035-110550094	CDC40
A_24_P499215	5.5000196	5.5780687	XR_246528	chr10:46201957-46201898	FAM21FP
A_23_P58763	10.2966585	10.151448	NM_015946	chr5:52097511-52097570	PELO
A_23_P77286	5.0817146	3.024057	NM_024713	chr15:34433509-34433450	KATNBL1
A_33_P3276813	6.1231127	5.9202585	NM_001142800	chr6:65596650-65596591	EYS
A_23_P151471	9.450476	9.478113	NM_001008895	chr13:113918591-113918650	CUL4A
A_33_P3263512	8.557522	8.941779		chr12:122206835-122206776	
A_33_P3380051	5.7831755	5.696103	NM_001145113	chr17:79898785-79898726	MYADML2
A_33_P3347452	6.9556746	6.1392803	NM_021135	chr6:166822961-166822902	RPS6KA2
A_23_P500433	4.5648403	3.571601	NM_052813	chr9:139258502-139258443	CARD9
A_23_P127446	9.452606	9.405915	NM_006268	chr11:65120192-65120251	DPF2
A_32_P100258	6.374166	5.8869658	NR_024279	chr1:16160877-16160818	FLJ37453
A_24_P281908	3.995978	3.4679751	NM_015267	chr12:111652103-111655741	CUX2

A_33_P3398809	9.019805	8.978119	NM_138777	chr9:125085109-125085168	MRRF
A_24_P191067	10.410073	10.61036	NM_001009566	chr1:9789383-9789324	CLSTN1
A_23_P164387	10.512553	10.482243	NM_003079	chr17:38784142-38784083	SMARCE1
A_32_P235796	5.832194	6.2118797	NM_152618	chr4:123665068-123665127	BBS12
A_32_P7015	6.6180677	6.382354	NM_012339	chr10:71267050-71267109	TSPAN15
A_23_P3963	8.114739	7.9735813	NM_014603	chr17:73001557-73001616	CDR2L
A_23_P85716	4.172011	4.237815	NM_021642	chr1:161488022-161488081	FCGR2A
A_23_P250385	6.6701	6.3471212	NM_005322	chr6:27834688-27834629	HIST1H1B
A_24_P67395	4.242708	3.44432	NM_002273	chr12:53294995-53294461	KRT8
A_23_P201672	10.366682	10.128265	NM_015935	chr1:171765987-171766046	METTL13
A_23_P328206	8.606413	8.402374	NM_015221	chr10:101635852-101635793	DNMBP
A_24_P145216	5.504531	5.2345657	BC030635	chrX:3737327-3737268	LOC100291323
A_23_P14975	7.9835176	8.135235	NM_032140	chr16:67697090-67697031	ENKD1
A_33_P3273000	12.578938	12.679198	AK128371	chr8:27309909-27309968	PTK2B
A_33_P3318926	3.8604987	4.021466	NM_006377	chr9:35404113-35404172	UNC13B
A_23_P6963	5.5536327	5.224393	NM_003341	chr3:23930634-23930693	UBE2E1
A_23_P356554	11.3610325	11.308544	NM_004282	chr6:57049439-57049498	BAG2
A_33_P3309859	4.6479607	3.9410715		chr3:194366000-194366059	
A_23_P126103	7.189038	6.157459	NM_001902	chr1:70905041-70905100	CTH
A_23_P215111	4.4067874	4.3336215	NM_020632	chr7:138391206-138391147	ATP6V0A4
A_23_P432360	9.239293	9.089195	NM_000303	chr16:8942790-8942849	PMM2
A_23_P1912	4.296473	4.621665	NM_207341	chr11:60643097-60643156	ZP1
A_23_P163580	4.7150865	4.500659	NM_025082	chr16:67863930-67863871	CENPT
A_33_P3360912	4.360532	3.4243553	AF085243	chr18:74647958-74648017	ZNF236
A_33_P3293207	7.8285284	8.285862	NM_177965	chr8:96257270-96257211	C8orf37
A_33_P3289696	3.8690088	4.2699604	NM_145047	chr1:36887818-36887759	OSCP1
A_23_P400603	2.3221061	2.3900566	NR_015363	chr9:41953446-41953387	MGC21881
A_33_P3326812	4.8905993	5.4325466	AK127768	chr20:62023991-62023932	LOC100130152
A_33_P3376031	4.1604986	3.2033176	NR_047572	chr16:3089072-3089131	LOC100128770
A_23_P33894	8.406283	8.433479	NM_201222	chrX:54838042-54838604	MAGED2
A_33_P3289745	4.521762	4.2746973	XR_247232	chr11:61736625-61736684	
A_23_P166536	8.589294	8.695426	NM_014577	chr22:50167024-50166965	BRD1
A_33_P3292235	4.514499	3.8513916	BC031256	chr4:79808359-79808300	PAQR3
A_23_P59426	8.416958	8.511153	NM_007349	chr7:154735770-154735711	PAXIP1
A_24_P261417	9.806858	9.914526	NM_015881	chr11:11984974-11984915	DKK3
A_23_P54840	12.209432	12.343213	NM_005946	chr16:56673205-56673808	MT1A
A_23_P39263	5.5916977	5.7531343	NM_173480	chr19:2917775-2917834	ZNF57
A_33_P3274009	8.571179	8.563005	AK124556	chr1:143914523-143914582	
A_23_P432591	6.7125115	6.5888467	NM_176816	chr5:68578825-68578766	CCDC125
A_33_P3212112	6.8406477	6.873208	NM_001042496	chr15:34522316-34522257	SLC12A6
A_33_P3221843	6.145152	5.3643913	XM_005267018	chr6:99817362-99817303	COQ3
A_33_P3222932	7.745161	7.613836	NM_001207020	chr22:42305884-42305825	SHISA8
A_23_P209726	7.995593	7.769651	NM_007237	chr2:231177506-231177565	SP140
A_23_P69720	13.201168	13.625151	NM_001154	chr4:122589538-122589479	ANXA5
A_33_P3352103	7.0152373	6.755336	NM_138794	chr1:219386138-219386197	LYPLAL1
A_23_P74914	8.672512	8.260809	NM_014777	chr1:229795276-229795335	URB2
A_33_P3219260	9.220818	9.565765	NM_016484	chrX:69506801-69506742	PDZD11
A_24_P119131	6.0470643	5.8372765	NM_001080825	chr12:122219893-122219952	TMEM120B
A_24_P409824	7.724992	7.5482244		chr9:101370186-101370247	
A_23_P149281	6.4116836	6.2056866	NM_004431	chr1:16455972-16451800	EPHA2
A_23_P85703	5.9397116	5.453352	NM_005686	chr1:204096551-204096610	SOX13
A_33_P3278265	7.5780125	6.8717256	NM_024854	chr12:21622722-21622781	PYROXD1
A_33_P3303469	6.653281	6.5249357		chr16:086007678-086007737	
A_23_P363163	9.233659	9.260934	NM_001008390	chr3:88101825-88101766	CGGBP1
A_24_P371303	8.534092	8.809408	NM_015224	chr3:56657192-56657133	FAM208A
A_23_P16006	7.2810116	7.332256	NM_198457	chr19:53269328-53269269	ZNF600
A_23_P383601	6.061118	6.6148667	NR_029434	chr14:58732910-58732851	FLJ31306
A_23_P255104	8.7843275	8.366184	NM_005779	chr5:77781354-77781295	LHFPL2
A_33_P3306526	8.133371	8.268309		chr11:067276140-067276199	

A_23_P428129	8.978645	8.861208	NM_000076	chr11:2904962-2904903	CDKN1C
A_33_P3408702	4.9988694	5.102203		chr5:93748995-93748936	KIAA0825
A_33_P3318357	4.740899	4.445693	NM_138775	chr11:107422595-107422536	ALKBH8
A_23_P212469	2.3221061	2.3900566	NM_001248	chr3:40469895-40469954	ENTPD3
A_33_P3220470	8.584265	8.792507	NM_005585	chr15:67074263-67074322	SMAD6
A_33_P3369885	3.8927033	3.616033	NM_001099280	chr8:145267904-145267963	MROH1
A_33_P3371984	7.096572	7.051969	NM_205833	chrX:130419158-130419099	IGSF1
A_33_P3290567	8.89003	9.097275	NM_003390	chr11:9611073-9611132	WEE1
A_23_P157283	9.041034	9.561395	NM_024315	chr7:86827335-86827276	TMEM243
A_24_P143492	5.949308	5.9499264	NM_001010974	chr20:49446825-49446884	BCAS4
A_33_P3610406	3.6947963	2.3900566	NR_028501	chr2:233250541-233250482	ECEL1P2
A_23_P61674	6.3179507	6.626774	NM_020666	chr5:178030053-178029994	CLK4
A_33_P3226439	6.5453615	6.1320457		chr9:44239604-44239545	LOC728903
A_33_P3668049	5.4975634	5.5290565	NM_001033568	chr17:30536407-30536464	RHOT1
A_23_P325075	7.5252542	6.3444753	NM_003800	chr6:89320349-89320290	RNGTT
A_24_P942481	8.458441	7.875874	NM_180989	chr13:95280961-95281020	GPR180
A_23_P408473	5.2478275	4.6860023	NM_032450	chr8:145315830-145315889	MROH1
A_23_P213754	11.125825	11.514585	NM_001033112	chr5:138704959-138705018	PAIP2
A_33_P3239634	6.6653943	6.5265474	NM_002505	chr6:41066676-41066735	NFYA
A_23_P121215	5.4951124	5.5336294	NM_003656	chr3:9802404-9802345	CAMK1
A_23_P90463	11.572023	11.54124	NM_016199	chr19:2321602-2321543	LSM7
A_32_P113154	14.46887	14.563592		chr8:012427883-012427824	
A_23_P48358	10.26808	10.730606	NM_000282	chr13:101182384-101182443	PCCA
A_33_P3238052	8.431735	7.8696556	NM_013300	chr12:110927982-110928041	FAM216A
A_23_P143559	4.832362	5.0574703	NM_007098	chr22:19167335-19167276	CLTCL1
A_24_P13311	6.8825684	7.0747805	NM_145648	chr12:129278893-129278834	SLC15A4
A_33_P3636080	5.760868	5.9944687	NM_001040108	chr14:75485674-75485615	MLH3
A_23_P431981	7.561368	7.7407207	NM_001003681	chr22:35691384-35691443	HMGXB4
A_32_P168349	4.4066286	4.630019	NM_138277	chr6:31694319-31694378	C6orf25
A_23_P350172	6.278309	6.283389	NM_001098638	chr11:74552738-74552797	RNF169
A_33_P3362326	8.017231	7.7236543	CR936682	chr2:105974230-105974171	FHL2
A_33_P3257708	10.059064	10.080197	NM_144772	chr1:156563287-156563346	APOA1BP
A_23_P16798	4.2783937	4.1699886	NM_199346	chr2:24344150-24344091	PFN4
A_23_P383118	3.56999	3.7377436	NM_020883	chr1:45482333-45482274	ZSWIM5
A_33_P3240946	6.0828657	5.6312113	NM_001280544	chr14:73128643-73128584	DPF3
A_33_P3399051	4.289626	4.347986	NM_025045	chr22:38493101-38493042	BAIAP2L2
A_32_P46571	2.3221061	2.3900566	NM_017821	chr1:39351574-39351515	RHBDL2
A_32_P199301	8.423021	8.901342	NM_007111	chr13:114290388-114290852	TFDP1
A_33_P3413855	3.63888	3.9565358	NM_152556	chr7:112462336-112462277	C7orf60
A_33_P3368540	5.6882505	5.6350527	XR_249341	chr3:9404584-9404525	
A_33_P3273732	6.073065	6.221105		chr9:137000368-137000309	XLOC_007882
A_24_P323084	3.9357	2.3900566	NR_038080	chr17:79276696-79276637	LINC00482
A_23_P139795	6.2053976	6.478347	NM_002442	chr12:120780269-120780210	MSI1
A_23_P310022	7.3359933	6.653843	NM_019590	chr10:24836459-24836518	KIAA1217
A_23_P422193	5.527629	5.1484375	NM_003173	chrX:48566887-48566946	SUV39H1
A_33_P3218356	7.9811254	8.024301	NM_020141	chr1:109637359-109637418	TMEM167B
A_24_P222835	6.7893896	7.0814586	NM_022753	chr1:33293651-33295592	S100PBP
A_33_P3640101	5.1875606	4.736662		chr19:32881016-32880957	LOC400684
A_33_P3242603	6.350553	6.406855	NR_027023	chr1:155533351-155533413	ASH1L-AS1
A_23_P379630	6.430361	6.4644713	NM_138570	chr17:79225124-79225065	SLC38A10
A_24_P225679	8.353381	7.7558928	NM_005544	chr2:227600207-227600148	IRS1
A_33_P3220213	3.3277721	3.0289886		chr10:023296009-023296068	
A_33_P3248329	6.0394135	6.2567806	NM_021943	chr6:38084440-38084499	ZFAND3
A_24_P380330	6.9473996	7.4525914	NM_024594	chr5:167982931-167982872	PANK3
A_23_P208143	4.807321	5.3185954	NM_001135178	chr18:32825958-32826017	ZNF397
A_33_P3370461	15.15555	14.948836		chr17:29085292-29085351	
A_24_P943393	14.097578	14.035051	NM_001620	chr11:62283459-62283400	AHNAK
A_23_P57760	6.468895	6.9577208	NM_152282	chr3:141013068-141013127	ACPL2
A_23_P155711	8.079973	8.106015	NM_018248	chr4:178283487-178283546	NEIL3

A_33_P3286011	7.410453	7.8221016		chr19:37177388-37177329	
A_24_P49190	7.4908714	7.9473624	NM_181655	chr17:65987591-65987532	C17orf58
A_33_P3414964	5.1570354	4.877057	BC029496	chr19:5273151-5273092	PTPRS
A_33_P3222937	11.133199	11.257883	NM_017727	chr2:27264502-27264561	TMEM214
A_23_P144684	8.294604	8.156947	NM_032290	chr5:94031167-94031226	ANKRD32
A_33_P3274184	4.0247326	3.7677674		chr1:234746102-234746161	
A_23_P48581	7.4733086	7.433317	NM_021944	chr14:23456502-23456443	C14orf93
A_23_P13554	10.069078	10.166935	NM_001007027	chr11:77812147-77812088	ALG8
A_23_P42498	12.509143	12.724985	NM_003795	chr6:108532968-108532909	SNX3
A_32_P223173	5.82648	5.506957		chr9:066514423-066514482	
A_33_P3422133	5.207655	5.343473	NM_006869	chr7:937790-937731	ADAP1
A_33_P3258510	6.0767198	5.9361863	NM_007260	chr1:24121176-24121235	LYPLA2
A_33_P3701139	3.8783827	3.483289			
A_33_P3230976	6.2436275	6.7044044	NM_002726	chr6:105771600-105771541	PREP
A_23_P345928	7.00881	7.190092	NM_032230	chr12:82872791-82872850	METTL25
A_33_P3312735	4.1966934	3.8772392	NM_024989	chr2:197697837-197697778	PGAP1
A_23_P171077	10.477465	10.587971	NM_006579	chrX:48386942-48387001	EBP
A_23_P20558	7.1387525	7.7727637	NM_017913	chr9:4706046-4706105	CDC37L1
A_24_P318939	4.779742	4.70234	NM_015655	chr20:25655624-25655565	ZNF337
A_24_P55148	8.575974	8.48667	NM_021058	chr6:27100377-27100318	HIST1H2BJ
A_23_P65757	11.344489	11.2621565	NM_004701	chr15:59417139-59417198	CCNB2
A_23_P151791	3.7170992	3.4278946	NM_181657	chr14:24785103-24785162	LTB4R
A_33_P3222761	3.8996036	3.5470848		chr14:22279883-22279942	
A_33_P3342633	4.1459556	4.139396	NM_001031719	chr13:52344980-52344921	DHRS12
A_33_P3398533	5.2026505	5.058455	NM_025138	chr13:39587269-39587210	PROSER1
A_33_P3318504	4.554728	4.4782057	AB072904	chr5:75441719-75441778	
A_33_P3359344	7.4031577	7.4857235	NM_182533	chr1:2125138-2125079	C1orf86
A_33_P3234855	5.132951	5.398431	NM_007124	chr6:145148752-145148811	UTRN
A_33_P3368800	6.8324795	6.965787	NM_001278141	chr1:146406524-146406583	NBPF12
A_23_P41292	8.210788	8.230029	NM_001012614	chr4:1205777-1205718	CTBP1
A_33_P3272402	4.9402943	5.5025764	NM_139174	chr16:84230712-84230771	ADAD2
A_33_P3329176	5.0435247	4.731065	X70308	chr14:22337450-22337509	
A_23_P93499	7.6386356	7.886239	NM_016020	chr6:155581505-155581446	TFB1M
A_23_P87973	7.4987526	7.8330355	NM_213590	chr13:50587010-50587069	TRIM13
A_33_P3242703	4.800745	4.9099226	AK292160	chr12:1039034-1038975	RAD52
A_33_P3290338	10.81678	10.590803	NM_001618	chr1:226590004-226589945	PARP1
A_24_P121171	6.051505	6.23246	NM_015517	chr11:119005144-119005203	HINFP
A_23_P52647	11.737551	11.67613	NM_006795	chr11:64620221-64620181	EHD1
A_23_P39076	7.3763967	7.8678026	NM_006270	chr19:50138792-50138733	RRAS
A_23_P209564	8.054498	8.7248745	NM_024843	chr2:172414039-172414098	CYBRD1
A_23_P200056	7.090687	7.1345625	NM_023018	chr1:1683470-1683411	NADK
A_23_P74229	10.778682	11.128361	NM_032017	chr1:36805373-36805314	STK40
A_33_P3389578	11.226423	11.397626		chr12:004218799-004218740	
A_33_P3407643	3.3463435	3.7771246	NM_001145024	chr11:112126186-112126127	PLET1
A_33_P3362562	4.8975058	4.98573	NM_001272043	chr17:58009023-58009082	RPS6KB1
A_23_P386364	4.7830467	4.7231536	NM_144657	chrX:83576878-83576819	HDX
A_23_P157268	7.979163	7.975233	NM_012129	chr7:90044941-90045000	CLDN12
A_23_P160025	9.788155	10.071898	NM_005531	chr1:159024619-159024678	IFI16
A_23_P33791	5.8619156	6.1048865	NM_012446	chr5:80716327-80716268	SSBP2
A_32_P162004	13.585563	13.605386	NM_000992	chr3:52028011-52027952	RPL29
A_33_P3340309	8.522147	8.469263	NM_024671	chr16:30535522-30535463	ZNF768
A_23_P376735	6.9264793	6.8544703	NM_153219	chr19:56114374-56114433	ZNF524
A_33_P3219596	5.8163147	6.2665257		chr15:89941658-89941717	LINC00925
A_32_P180315	4.464243	3.9952137	NM_020893	chr9:100139057-100139116	CCDC180
A_33_P3290174	7.970763	8.158207	NM_206918	chr14:100613034-100612975	DEGS2
A_23_P208310	10.447917	9.102263	NM_012099	chr19:45913903-45913962	CD3EAP
A_33_P3415042	9.030642	9.46335	NM_001096	chr17:40024027-40023968	ACLY
A_33_P3411075	12.368598	12.127104	NM_003088	chr7:5646220-5646279	FSCN1
A_33_P3358914	5.2823396	5.125038	NM_001199417	chr17:36646345-36646404	ARHGAP23

A_33_P3246513	4.1598215	4.03692	NM_001362	chr14:102028602-102028661	DIO3
A_32_P76060	7.828793	7.158597	NM_016396	chr15:44819366-44819425	CTDSPL2
A_33_P3348719	3.6962435	3.3588812	NM_002010	chr13:22278493-22278552	FGF9
A_23_P161297	2.3221061	2.3900566	NM_018245	chr10:50942857-50942798	OGDHL
A_33_P3217619	4.897033	4.931273		chr12:76083366-76083307	
A_23_P69826	10.686781	11.020546	NM_001358	chr4:24531344-24531285	DHX15
A_24_P49349	6.0418897	6.3770676	NM_014857	chr1:174668678-174668737	RABGAP1L
A_33_P3331652	7.063233	7.066298	XR_245688	chr14:24608889-24608830	EMC9
A_23_P98382	11.844175	11.713622	NM_012459	chr11:111955984-111955925	TIMM8B
A_24_P51683	6.113184	6.35064	NM_003936	chr2:219826083-219826142	CDK5R2
A_33_P3363016	6.907656	6.902822	NM_001242790	chr14:105739118-105739059	BRF1
A_33_P3326256	7.656957	7.338572	NM_016065	chr10:75009198-75009139	MRPS16
A_33_P3274832	3.8869314	3.6850758	NR_033958	chr12:2870425-2870366	LOC283440
A_24_P95029	10.560978	9.845925	NM_006024	chr7:27867426-27868292	TAX1BP1
A_23_P52101	9.206402	9.488722	NM_016243	chr1:202931374-202931315	CYB5R1
A_23_P250455	6.5829215	6.5701447	NM_006044	chrX:48683060-48683119	HDAC6
A_23_P411922	6.4975376	6.4916134	NM_001031698	chr12:50038227-50038286	PRPF40B
A_24_P22976	5.9637947	5.880626	NM_001025604	chr19:18124251-18124310	ARRDC2
A_32_P183442	8.201474	7.804295		chr4:132777618-132777559	XLOC_014512
A_24_P132950	6.3704176	6.384126	NM_005969	chr11:2966668-2966609	NAP1L4
A_32_P123629	8.563876	8.3428135	NM_001135993	chr18:21715452-21715511	TTC39C
A_33_P3222703	5.1752524	5.5323334	NM_001024855	chr3:44688869-44688928	ZNF197
A_23_P33187	7.5029564	7.533314	NM_032558	chr9:97191472-97191531	HIATL1
A_24_P277295	9.072344	9.215517	NM_198490	chr3:128806580-128806521	RAB43
A_33_P3368610	9.580804	9.947005	NM_201438	chr12:42792700-42792759	PPHLN1
A_23_P334883	5.964692	5.709439	NM_012309	chr11:70314422-70314363	SHANK2
A_32_P9382	10.56444	10.512367	NM_001071775	chr13:73282693-73282634	MZT1
A_23_P138776	3.3639343	3.5254931	NM_001610	chr11:47264448-47264389	ACP2
A_24_P603224	10.180435	10.124496	NM_006802	chr1:38423731-38423677	SF3A3
A_24_P357518	13.568987	13.585563	NM_000982	chr13:27828370-27829389	RPL21
A_23_P142872	7.5950146	8.003777	NM_031283	chr2:85536678-85536737	TCF7L1
A_23_P308954	5.1445713	5.0075827	NM_030639	chrX:102006979-102007038	BHLHB9
A_23_P128532	8.759468	8.350611	NM_152318	chr12:105385586-105388274	C12orf45
A_33_P3227904	5.058312	5.2253885	NM_207348	chr1:16067824-16067883	SLC25A34
A_23_P253692	2.3221061	2.3900566	NM_001079858	chrX:19007638-19007579	GPR64
A_23_P258164	6.635542	6.638522	NM_001302	chr1:10511688-10511747	CORT
A_33_P3345354	8.565444	8.415587	NM_015997	chr1:156706655-156706714	RRNAD1
A_24_P153511	3.9984045	3.950175	NM_020841	chr12:76746619-76746560	OSBPL8
A_33_P3365616	9.333678	9.457543	NM_152265	chr1:52553899-52553958	BTF3L4
A_33_P3222243	5.387212	5.434152		chr2:130893903-130893962	
A_23_P13524	10.332307	9.987243	NM_032273	chr11:85367414-85367473	TMEM126A
A_24_P87763	9.994153	9.694223	NM_001961	chr19:3977256-3976730	EEF2
A_33_P3247858	4.6866617	4.2245226	NM_001166461	chrX:154019318-154019259	MPP1
A_24_P916141	7.9032598	8.370982	NM_005828	chr17:61671181-61671240	DCAF7
A_23_P34233	10.637595	10.623973	NM_014298	chr16:29708670-29708729	QPRT
A_23_P83835	5.359779	5.2677875	NM_032237	chr8:42977869-42977928	POMK
A_33_P3336686	10.854712	11.125825	NM_004669	chr9:139889119-139889060	CLIC3
A_33_P3323048	5.355774	5.365794	NM_001029998	chr4:147179943-147179884	SLC10A7
A_33_P3231140	6.4298797	6.2855325	NM_001167882	chr4:125585620-125585561	ANKRD50
A_23_P65357	8.748796	8.052992	NM_018373	chr14:70839282-70839223	SYNJ2BP
A_24_P160001	7.221232	7.4842796	NM_054014	chr20:1356196-1356137	FKBP1A
A_23_P78099	4.7969613	4.7760625	NM_000638	chr17:26694806-26694747	VTN
A_24_P65199	4.3532295	3.9666915	NM_052987	chr16:89758894-89759710	CDK10
A_24_P148235	15.480215	15.5145445	NM_001030	chr1:153964053-153964112	RPS27
A_33_P3316394	3.8292212	3.550665	NM_001098491	chr19:58005026-58005085	ZNF419
A_24_P740705	9.652733	9.239293	NM_194326	chr22:39925547-39925488	RPS19BP1
A_33_P3241661	2.3221061	2.3900566	XM_005260905	chr20:2193727-2193786	LOC388780
A_24_P350200	12.775692	13.010773	AK057071	chrM:9410-9469	COX3
A_23_P68547	8.584979	8.797425	NM_182802	chr20:5975064-5975123	MCM8

A_24_P85243	5.0288043	5.418895		chr5:177138619-177138560	FAM153A
A_23_P253012	6.666831	6.619705	NM_017577	chr3:113665696-113665755	GRAMD1C
A_33_P3298942	8.503619	8.777135	AK090832	chr5:160060441-160060382	ATP10B
A_24_P928052	10.555244	10.925762	NM_003873	chr10:33466995-33466936	NRP1
A_23_P302672	2.3221061	2.3900566	BC013592	chr4:101107295-101107236	DDIT4L
A_23_P24555	5.42448	5.6177726	NM_015157	chr11:118527559-118527618	PHLDB1
A_23_P76529	5.9026475	5.953005	NM_000889	chr12:53585216-53585157	ITGB7
A_23_P47682	8.665973	8.622412	NM_020645	chr11:9004858-9004799	NRIP3
A_23_P51508	10.594658	10.350051	NM_007240	chr1:161726752-161726811	DUSP12
A_23_P41888	7.311735	7.446471	NM_153373	chr5:177638915-177635882	PHYKPL
A_23_P2271	4.39051	3.6490695	NM_198965	chr12:28116401-28116342	PTHLH
A_33_P3394786	11.536232	11.417999		chr19:9293628-9293569	XLOC_014512
A_23_P160881	4.4364705	4.2395954	NM_001009568	chr1:28282374-28282517	SMPDL3B
A_24_P56894	6.7424994	7.342567	NM_015986	chr17:29110720-29110661	CRLF3
A_23_P8754	5.758689	6.2729597	NM_005763	chr7:121717911-121716622	AASS
A_23_P27013	2.3221061	2.3900566	NM_024017	chr17:46698674-46698615	HOXB9
A_33_P3294237	7.9743814	8.191725	NM_005955	chr1:38275473-38275414	MTF1
A_23_P503200	10.062711	10.029689	NM_018288	chr6:170104142-170104083	PHF10
A_33_P3316686	7.141903	7.2734313	NM_016230	chr6:84618749-84618808	CYB5R4
A_33_P3266315	9.177562	8.774071	NM_001206651	chr1:87213745-87213804	SH3GLB1
A_23_P370625	7.614069	7.568104	NM_020451	chr1:26144542-26144601	SEPN1
A_33_P3214456	6.3530445	6.3560457	NM_020381	chr6:107595299-107595240	PDSS2
A_33_P3219641	8.442746	8.335191	NM_001098802	chr9:80881548-80881607	CEP78
A_23_P418031	10.338143	9.875971	NM_001136265	chr1:19230868-19230809	IFFO2
A_23_P38254	11.757889	11.630226	NM_018127	chr17:12895771-12895712	ELAC2
A_23_P366366	10.915041	10.504596	NM_014766	chr7:29959882-29959823	SCRN1
A_24_P314179	6.3221216	5.2855196	NM_005239	chr21:40196273-40196332	ETS2
A_24_P419276	5.378422	4.943234	NM_021045	chr10:38120137-38120078	ZNF248
A_23_P147641	6.7084966	6.6905255	NM_003195	chr20:62701165-62701662	TCEA2
A_23_P8797	7.2017236	7.1616483	NR_003614	chr7:72482057-72483660	PMS2L2
A_33_P3272558	10.86678	10.811014	NM_001185011	chr22:50961856-50961915	NCAPH2
A_33_P3422471	4.288531	3.7619832	AK126796	chr10:126181396-126181455	
A_33_P3351276	5.1619644	4.9546466		chr16:032822588-032822529	
A_23_P64661	2.3713326	2.3900566	NM_032496	chr12:57866222-57866163	ARHGAP9
A_23_P312536	3.9689412	3.6126099	NM_198150	chr5:94939895-94939954	ARSK
A_33_P3253239	5.489242	5.9138775		chr1:180922784-180922843	
A_24_P295999	9.84982	10.248001	NM_000616	chr12:6929910-6929969	CD4
A_33_P3416414	8.322927	8.098387		chr8:050652922-050652863	
A_33_P3369245	5.280776	5.304164	XM_005275697	chr11:1997823-1997882	LOC101927645
A_33_P3402329	4.6912065	5.1325574	NR_024607	chrX:133677471-133677412	MIR503HG
A_23_P27005	4.6792064	4.5479126	NM_024308	chr17:34956827-34956886	DHRS11
A_23_P65031	13.427124	13.37882	NM_001037494	chr12:120936092-120936151	DYNLL1
A_23_P259874	7.261459	7.4733086	NM_012430	chr3:122990740-122990799	SEC22A
A_33_P3344482	7.936412	8.388909	NM_001135000	chr14:53326331-53326272	FERMT2
A_33_P3378785	7.617489	7.818185	AK124823	chr11:1687624-1687683	FAM99A
A_23_P258621	10.299533	10.338907	NM_003729	chr1:100757233-100757292	RTCA
A_24_P816384	7.201231	7.229971	NR_003661	chr15:85098273-85098214	UBE2Q2P1
A_33_P3563369	8.13829	8.170699	BG987537	chr16:2012332-2012273	SNORA10
A_32_P29784	11.759978	11.909884	NM_001043352	chr1:154127824-154127780	TPM3
A_33_P3331992	7.084827	7.004422		chr7:155404014-155403955	
A_32_P179746	6.086684	6.180395	XR_246550	chr5:94050473-94050532	LOC101929687
A_24_P157926	5.371787	5.6270413	NM_006290	chr6:138203679-138203738	TNFAIP3
A_23_P203475	10.317737	10.515417	NM_145040	chr11:6340269-6340210	PRKCDBP
A_33_P3268612	4.204215	4.060705	NM_170743	chr1:24483896-24483837	IFNLR1
A_33_P3320533	3.3381543	2.3900566		chr2:86305237-86305178	POLR1A
A_33_P3216200	4.0734673	3.5270605		chr14:064034608-064034667	
A_23_P71094	7.1777368	7.399332	NM_024523	chr7:127221136-127221077	GCC1
A_33_P3384775	4.004646	3.1748857	XM_005261122	chr21:43422652-43422593	ZBTB21
A_23_P41804	4.9834547	5.006343	NM_033120	chr5:1038719-1038778	NKD2

A_23_P213000	9.233384	9.350312	NM_017491	chr4:10076770-10076711	WDR1
A_23_P422540	8.459543	8.541524	NM_015276	chr17:20903621-20903562	USP22
A_23_P138975	8.990842	8.864775	NM_016090	chr11:114278950-114279009	RBM7
A_33_P3240340	8.540735	8.6690235		chr17:000862493-000862552	
A_23_P259357	15.608545	15.495825	NM_001001479	chr22:31043087-31043146	SLC35E4
A_23_P18276	11.039137	11.071348	NM_005778	chr3:50156137-50156196	RBM5
A_23_P218937	3.6458335	3.4472132	NM_032402	chr5:140857807-140857866	PCDHGC3
A_33_P3226643	4.7974305	4.770191	NM_001077198	chr2:220086832-220086773	ATG9A
A_33_P3623118	4.733893	4.9660296	AK054653	chr5:180211946-180211887	LOC153577
A_32_P94660	8.096658	8.385385	NM_052888	chr17:30377022-30377081	LRRC37B
A_23_P35782	7.839519	7.8764515	NM_024771	chr11:63723802-63723861	NAA40
A_33_P3318439	2.3221061	2.3900566	NM_001131034	chr4:1065441-1065382	RNF212
A_33_P3366064	4.250975	3.9867947	NM_199181	chr20:35422908-35422849	SOGA1
A_23_P428827	8.281559	8.063941	NM_018064	chr6:88391436-88391377	AKIRIN2
A_23_P56567	10.337336	9.937018	NM_024775	chr2:39008980-39009039	GEMIN6
A_33_P3253707	10.706341	10.427872	NM_152329	chr14:50081313-50081372	LRR1
A_33_P3340264	4.107576	4.2118773		chr11:017668151-017668210	
A_24_P237757	7.481267	7.742671	AL136621	chr13:20593755-20593814	ZMYM2
A_23_P153628	6.350856	6.530618	NM_033557	chr19:38798303-38798244	YIF1B
A_24_P282043	4.8430343	5.0332203	NM_006969	chr19:53301682-53301623	ZNF28
A_24_P703462	5.908025	6.092394	CD518305	chr3:037184954-037184895	
A_23_P203406	10.327965	10.514431	NM_198335	chr11:62392561-62392502	GANAB
A_23_P145817	11.063667	11.228376	NM_138446	chr7:23349002-23349061	MALSU1
A_24_P928217	5.584559	5.8108625	NM_024293	chr2:220049123-220049182	FAM134A
A_24_P358054	5.2632036	5.2175293	NR_003268	chr13:50466672-50466731	CTAGE10P
A_33_P3261720	6.5886345	6.930388	NR_046110	chr2:110752020-110752079	LINC01123
A_23_P88420	12.512245	12.630185	NM_006156	chr14:24686144-24686085	NEDD8
A_24_P942493	4.1843734	3.9142025	NM_015253	chr17:6027060-6027119	WSCD1
A_23_P207014	7.76504	7.7629046	NM_002876	chr17:56772439-56772498	RAD51C
A_33_P3229789	5.631528	5.6390095		chr19:56662806-56662747	
A_33_P3212167	4.4614043	4.62448	NM_001278344	chr1:236927181-236927240	ACTN2
A_23_P402751	13.0891	13.242724	BC000228	chrM:7987-8046	COX2
A_23_P435477	4.7758007	4.219327	NM_001077263	chr11:117771864-117771805	TMPRSS13
A_23_P41455	5.7336807	5.687347	NM_003305	chr4:122800569-122800510	TRPC3
A_24_P298737	6.262122	6.160348	AK090442	chrX:3789898-3789839	
A_33_P3329878	9.401131	9.419783	NM_207111	chr7:5659660-5659655	RNF216
A_24_P233917	9.509762	9.733401	NM_014774	chr1:47141088-47141029	EFCAB14
A_24_P270728	11.494258	10.554776	NM_001042483	chr16:28548880-28548821	NUPR1
A_23_P60579	8.707744	9.322466	NM_002435	chr15:75190359-75190418	MPI
A_32_P32739	7.522472	7.4999795	NM_153006	chr17:42086302-42086361	NAGS
A_33_P3233843	5.9387007	6.348011	NM_001190981	chr5:55265550-55265491	IL6ST
A_23_P104819	3.6756327	2.3900566	NM_007180	chr11:118531328-118531269	TREH
A_23_P161183	6.535187	6.7176857	NM_022494	chr10:114192034-114190643	ZDHHC6
A_33_P3333820	10.083698	9.2177305	NM_017811	chr9:33920342-33920401	UBE2R2
A_23_P49768	10.060047	10.137608	NM_016504	chr17:48445492-48445433	MRPL27
A_23_P132718	5.571997	5.216123	NM_004636	chr3:50314465-50314524	SEMA3B
A_33_P3287815	10.174321	9.662048	NM_001256910	chr10:70744186-70744245	DDX21
A_23_P126593	12.064273	12.051247	NM_005620	chr1:152005278-152005219	S100A11
A_32_P221799	6.7538857	6.4605894	NM_003514	chr6:27860566-27860507	HIST1H2AM
A_33_P3372488	4.792497	4.399597	BC061582	chr10:97443077-97443018	TCTN3
A_23_P321511	3.706232	3.8572478	NM_178450	chr5:126206442-126206383	MARCH3
A_33_P3249872	8.9118	9.310001	NM_001996	chr22:45959173-45959232	FBLN1
A_23_P30126	2.3221061	2.3900566	NM_005130	chr4:15937391-15937332	FGFBP1
A_24_P159948	4.877057	5.0452795	NM_003224	chr20:62338015-62337745	ARFRP1
A_23_P166459	14.555595	14.398101	NM_002305	chr22:38075652-38075711	LGALS1
A_23_P214046	9.74446	9.698865	NM_012300	chr5:171289064-171289005	FBXW11
A_33_P3268472	8.726617	8.29533	NM_001114173	chr11:88061340-88061281	CTSC
A_32_P71113	6.2677307	6.037298	NM_173690	chr9:127705207-127705148	SCAI
A_24_P169688	6.239206	6.5464697	NM_005931	chr6:31475185-31475244	MICB

A_33_P3356995	4.3649826	4.3397083	XM_005251136	chr8:68087312-68087253	ARFGEF1
A_33_P3310276	4.0381484	4.5493793	XM_005273551	chr8:22179377-22179436	PIWIL2
A_23_P326931	5.533925	5.540865	NM_145170	chr10:75013648-75013589	TTC18
A_33_P3235132	6.719713	6.9298573	AK127783	chr22:22649050-22649109	
A_23_P408376	9.496032	9.511988	NM_025015	chr10:118430849-118430790	HSPA12A
A_23_P71146	6.3461685	6.326337	NM_006230	chr7:44156824-44156765	POLD2
A_33_P3411145	6.111216	5.8849883	NM_001145191	chr4:15691923-15691982	FAM200B
A_23_P202945	5.78905	6.442684	NM_014155	chr11:130108369-130106960	ZBTB44
A_24_P375599	13.604	13.627423		chr19:030447612-030447552	
A_33_P3298960	5.137442	6.0164585	NM_153705	chr11:108350112-108350053	KDELC2
A_23_P200772	8.042294	8.384815	NM_201269	chr1:91380863-91380846	ZNF644
A_23_P45945	14.369245	14.288418	NM_005801	chr17:39847164-39847223	EIF1
A_33_P3274416	4.780237	4.4376726	NR_034179	chr3:128580481-128580422	LOC653712
A_24_P283320	9.122261	9.205869	NM_005389	chr6:150092364-150094294	PCMT1
A_23_P218858	5.23094	4.744542	NM_015429	chr3:100468645-100468586	ABI3BP
A_24_P795594	7.0758467	6.3250265	NM_001077691	chr11:111653292-111653233	ALG9
A_24_P308029	5.5945225	5.3737745	NM_144617	chr19:36246079-36246020	HSPB6
A_33_P3290945	5.4315596	5.7844377	NM_017952	chr2:86348655-86348714	PTCD3
A_23_P17503	7.295623	7.1259456	NM_024704	chr20:16253014-16252955	KIF16B
A_33_P3423791	4.274914	4.053425	NM_198182	chr2:10132177-10132236	GRHL1
A_24_P254705	6.2275076	6.4837313	NM_020394	chr1:247163255-247162725	ZNF695
A_23_P356694	3.957381	3.512135	NM_153324	chr20:30037996-30038055	DEFB123
A_33_P3308263	4.5570655	4.819148	NM_032839	chr3:122578943-122579002	DIRC2
A_23_P166508	5.252795	5.344797	BC038245	chr22:42760626-42760567	
A_23_P113523	10.117916	10.265017	NM_012227	chrX:221516-221457	GTPBP6
A_24_P334130	7.003071	6.5895023	NM_054034	chr2:216288895-216288217	FN1
A_23_P94795	7.3566103	7.5676713	NM_003213	chr12:3149391-3149555	TEAD4
A_32_P538180	5.046422	4.83685	NM_178422	chr1:26188759-26188700	PAQR7
A_33_P3223088	5.3015447	4.5115633		chr2:75897810-75897869	MRPL19
A_24_P937405	8.540859	8.339792	NM_007173	chr11:86522062-86522121	PRSS23
A_23_P327361	5.1990047	5.211609	NM_015263	chr15:51739946-51739921	DMXL2
A_32_P61684	5.7863445	5.4143543	NM_018440	chr8:81880292-81880233	PAG1
A_33_P3308446	7.8624277	8.267062	NM_004040	chr2:20648971-20649030	RHOB
A_33_P3377209	7.5051346	7.3038206	NM_207168	chr1:150599867-150599808	ENSA
A_33_P3220160	7.902265	8.264963	AK096924	chr22:37636907-37636848	RAC2
A_23_P146572	9.107086	9.000854	NM_015392	chr9:139934150-139934091	NPDC1
A_33_P3324651	10.720389	10.56444	NM_001242600	chr20:34328806-34328747	RBM39
A_24_P365025	9.333247	9.423573	NM_003971	chr17:49043155-49043096	SPAG9
A_33_P3325467	8.287402	8.100642	NM_001164824	chr1:35319192-35319133	SMIM12
A_24_P320604	12.171502	12.346024	NM_032131	chr6:109294735-109294794	ARMC2
A_23_P36408	7.881619	8.340704	NM_022916	chr12:122716876-122716817	VPS33A
A_23_P257503	8.44455	8.448131	NM_003922	chr15:63901042-63900983	HERC1
A_23_P164196	7.3081603	7.334392	NM_138281	chr17:48051866-48051925	DLX4
A_23_P70047	11.945889	12.01219	NM_199189	chr5:138665300-138665359	MATR3
A_23_P320862	3.783859	3.278869	NM_152786	chr9:116191529-116191588	C9orf43
A_24_P157156	6.308315	6.360148	NM_001080539	chr2:197590735-197590794	CCDC150
A_24_P91140	9.36001	9.630812	NM_000984	chr17:27051088-27051147	RPL23A
A_23_P97123	10.931988	11.001445	NR_026844	chr1:168215109-168215050	ANKRD36BP1
A_24_P376229	10.270273	10.32852	NM_005499	chr19:34960205-34960264	UBA2
A_33_P3332533	4.084467	4.4134135	NR_040096	chr12:93397593-93397534	LOC643339
A_33_P3243394	5.0608788	4.3971415	AK124200	chr3:57883097-57883156	SLMAP
A_33_P3424882	5.203005	4.784997		chrY:026336173-026336114	
A_23_P17420	2.3221061	2.3900566	NM_003657	chr20:52561533-52561474	BCAS1
A_33_P3358977	8.951388	8.499775	NM_032193	chr11:65485203-65485144	RNASEH2C
A_23_P93389	7.887694	8.096398	NM_006703	chr6:34256123-34256064	NUDT3
A_23_P71821	10.348887	10.894218	NM_006195	chr9:128728847-128728906	PBX3
A_23_P59976	4.779547	4.39859	NM_002066	chr8:143928184-143928243	GML
A_23_P74391	6.479394	7.2099605	NM_014322	chr1:241757732-241757673	OPN3
A_24_P31421	4.666588	4.4720902	NM_015711	chr19:48205351-48205410	GLTSCR1

A_23_P387045	6.751809	7.0322657	NM_174923	chr9:35660807-35660866	CCDC107
A_33_P3224250	4.020775	3.0108423	NM_001048210	chr1:109472215-109472156	CLCC1
A_33_P3412798	2.3221061	2.3900566	NM_001080998	chr10:135438929-135438870	FRG2B
A_23_P133648	4.987467	4.5251126	NM_016255	chr6:17611415-17611474	FAM8A1
A_33_P3366391	6.768622	6.1341076		chr11:124984692-124984751	
A_23_P201047	6.771787	6.900847	NM_007112	chr1:155165848-155165789	THBS3
A_33_P3333603	4.673257	4.370503	NM_022137	chr14:70490104-70490163	SMOC1
A_24_P233078	4.954335	4.4994073		chr17:26554908-26554967	PYY2
A_33_P3282566	8.143421	8.203582	NM_017728	chr17:72834553-72834612	TMEM104
A_33_P3303176	7.091585	7.1455913	NM_001164377	chr11:3239233-3239174	MRGPRG
A_23_P52402	6.5024486	6.5333643	NM_014889	chr10:3182924-3181228	PITRM1
A_33_P3397743	3.6705103	2.9043543		chr11:102622138-102622079	
A_33_P3275163	7.269779	6.8304806		chr3:196197721-196197780	
A_23_P99172	8.348151	8.431492	NM_032358	chr12:551556-551615	CCDC77
A_23_P23575	7.743501	7.65073	NM_014437	chr1:153932251-153932192	SLC39A1
A_23_P118122	4.042767	4.1757803	NM_003834	chr16:318383-318324	RGS11
A_23_P94434	6.828021	6.5774593	NM_001039792	chr9:35906792-35906851	HRCT1
A_23_P85604	9.87139	10.1315365	NM_025160	chr1:224577223-224577164	WDR26
A_24_P943957	7.860559	7.946617	NM_015040	chr2:209222843-209222902	PIKFYVE
A_33_P3240941	9.94519	9.690769	NM_012104	chr11:117159804-117159745	BACE1
A_23_P387057	10.215347	10.325713	NM_178014	chr6:30692522-30692581	TUBB
A_23_P15375	7.6314287	7.2497854	NM_024585	chr17:73126058-73126117	ARMC7
A_24_P303974	10.30224	9.87139	NM_006023	chr10:12288219-12291581	CDC123
A_23_P391637	8.143698	7.2960606	NM_020705	chr16:2553251-2553310	TBC1D24
A_33_P3338559	11.686246	11.290114	NM_005610	chr1:33146199-33146258	RBBP4
A_33_P3320283	4.339959	4.3910346		chr8:026306602-026306661	
A_33_P3252381	7.2971725	7.2203918	NR_015342	chr9:79402286-79402345	PCA3
A_33_P3641427	6.296269	6.9891253	NM_016132	chr15:48434932-48434873	MYEF2
A_23_P29124	6.2627244	6.3663826	NM_000407	chr22:19712183-19712242	GP1BB
A_23_P164228	9.953522	9.713991	NM_005175	chr17:46970798-46971772	ATP5G1
A_33_P3305368	6.7322598	6.649771	NM_138620	chr9:135534118-135534059	DDX31
A_33_P3311770	5.923402	5.954524	NM_213603	chr7:99085134-99085193	ZNF789
A_32_P151782	10.161468	10.198149		chr13:100740961-100740902	XLOC_010709
A_23_P147888	16.13095	16.106268	NM_001004	chr11:000812818-000812875	RPLP2
A_33_P3388938	5.162415	4.9338036	NR_024402	chr16:88741847-88741906	SNAI3-AS1
A_23_P51019	3.4908872	2.3900566	NM_021007	chr2:166246107-166246166	SCN2A
A_23_P147495	8.240978	8.258498	NM_021946	chrX:129191457-129191516	BCORL1
A_33_P3413468	7.555506	7.4618955	NM_001242310	chrX:65815549-65815490	EDA2R
A_33_P3285097	3.8458984	3.4412339		chr9:045414686-045414627	
A_23_P140668	5.5884976	5.4013543	NM_005530	chr15:78462425-78462484	IDH3A
A_33_P3313476	5.446129	5.4091396	NM_001136571	chr13:32877973-32877914	ZAR1L
A_33_P3394789	4.659232	4.393775	XR_247761	chr15:102506671-102506730	LOC100653296
A_23_P381577	4.4835496	2.3900566	NM_145011	chr10:38239068-38239009	ZNF25
A_23_P58506	8.280425	6.9244003	NM_012081	chr5:95220884-95220825	ELL2
A_23_P126486	8.82753	8.875906	NR_026752	chr1:16944885-16944826	CROCCP2
A_23_P342131	6.178317	5.976597	NM_153611	chr11:61117197-61117138	CYB561A3
A_33_P3265945	6.029647	6.0394135	AK129671	chr5:132279131-132279072	
A_23_P154199	10.263947	10.404868	NM_002712	chr2:242099894-242102743	PPP1R7
A_33_P3303394	10.548663	10.664523		chr17:055208895-055208836	
A_23_P501754	2.3221061	2.3900566	NM_000759	chr17:38173710-38173769	CSF3
A_23_P46924	9.92042	9.779439	NM_001007793	chr10:124917262-124917321	BUB3
A_23_P30204	6.8030887	6.6578097	NM_021182	chr5:143200209-143200268	HMHB1
A_24_P84401	4.652873	4.5593176		chr11:093343294-093343235	
A_33_P3288839	4.811657	4.8215637	NM_001001872	chr14:58470872-58470813	C14orf37
A_23_P14193	9.03846	8.377911	NM_002915	chr13:34410688-34410747	RFC3
A_33_P3256500	6.3255253	6.503545	AK095017	chr12:111950637-111950578	ATXN2
A_23_P369641	7.6706333	7.376501	NM_001100878	chr8:144648965-144648906	MROH6
A_24_P659122	7.6512365	7.715541	XM_005272717	chr7:63215032-63215091	LOC101930190
A_33_P3260777	12.135965	12.322415	NM_001662	chr7:127231693-127231752	ARF5

A_23_P25003	8.78534	8.424352	NM_002956	chr12:122756161-122756102	CLIP1
A_33_P3281041	4.6909204	4.354736		chr3:131081332-131081391	
A_23_P209731	8.262749	8.056921	NM_025139	chr2:232209808-232209867	ARMC9
A_23_P171385	7.5124016	7.3018847	NM_032335	chrX:133527626-133527959	PHF6
A_23_P306479	5.1159587	5.1497073	NR_024407	chr3:101396264-101396323	ZBTB11-AS1
A_23_P42096	7.275055	7.4024096	NM_005922	chr6:161536231-161536290	MAP3K4
A_23_P126089	5.5820394	5.8495984	NR_003062	chr1:153112700-153112641	SPRR2C
A_33_P3323384	5.8973475	6.323622	NM_015713	chr8:103219973-103219914	RRM2B
A_33_P3292297	4.58431	4.407175	NM_001282950	chr2:232672286-232672345	COPS7B
A_33_P3362359	3.7774332	3.34811		chr9:33638377-33638436	
A_33_P3390246	4.9032574	4.585535	AK123701	chrY:1463530-1463589	
A_23_P410965	10.393127	10.487129	NM_020888	chr1:33240353-33240412	KIAA1522
A_33_P3285824	10.307714	10.861438	NM_001170330	chr4:120221550-120221491	C4orf3
A_24_P394368	4.8601313	5.593926	NM_025132	chr4:39271715-39274653	WDR19
A_23_P47357	15.472499	15.434727	NM_001028	chr11:118886616-118886557	RPS25
A_33_P3238966	11.900848	11.573592	NM_017921	chr17:79523974-79523915	NPLOC4
A_23_P95736	10.245807	10.556138	NM_001001415	chr19:21720226-21720285	ZNF429
A_23_P208389	10.419801	10.655113	NM_021913	chr19:41767395-41767454	AXL
A_23_P144959	10.831766	11.563932	NM_004385	chr5:82876302-82876361	VCAN
A_33_P3413227	4.1082945	3.6764402	AK096512	chr16:671592-671651	LOC100132363
A_33_P3237644	4.746731	4.259421	NM_001145053	chr17:28401583-28401642	EFCAB5
A_23_P86252	9.353349	9.444025	NM_002642	chr1:172410845-172410786	PIGC
A_23_P94736	7.0333943	6.872841	NM_175039	chr9:130670859-130670800	ST6GALNAC4
A_24_P402825	4.674955	4.811366	NM_018398	chr3:55021774-55038842	CACNA2D3
A_23_P128396	9.46238	9.794712	NM_194271	chr12:121861889-121861948	RNF34
A_33_P3315475	9.516384	9.456326	NM_001080435	chr15:83488180-83488239	WHAMM
A_33_P3314902	7.5887165	7.339755	NM_016252	chr2:32843885-32843944	BIRC6
A_33_P3596355	4.7197723	4.7118855	BG033631		LINC00622
A_24_P58894	9.42421	9.187328		chr9:023682353-023682412	
A_24_P332739	8.192009	8.3512945	NR_027028	chr5:21497136-21497195	GUSBP1
A_23_P67785	7.9150987	7.9188643	NM_001025436	chr2:214174841-214174900	SPAG16
A_32_P142700	5.0692396	5.140354	NM_182520	chr22:24106835-24106894	C22orf15
A_32_P131367	7.1729655	7.414852	NM_007282	chr3:149629843-149638952	RNF13
A_33_P3321136	5.434974	5.669467	NM_014957	chr8:142151363-142151422	DENND3
A_33_P3317797	4.234963	4.0806565	NM_001135585	chr1:9101486-9101427	SLC2A5
A_33_P3315375	2.3221061	2.3900566	NM_001104587	chr17:33677430-33677371	SLFN11
A_23_P309261	7.2719283	7.2383895	NM_005751	chr7:91739783-91739842	AKAP9
A_32_P116058	10.255593	10.665673	NR_015427	chr9:136893129-136893188	LINC00094
A_23_P158533	5.0892935	5.31244	NM_021008	chr11:678736-674767	DEAF1
A_33_P3295870	6.477545	6.42529	NM_001001710	chr9:140138097-140138038	FAM166A
A_33_P3272479	5.3501186	5.2506123	NM_001286681	chr8:21883178-21883237	NPM2
A_33_P3210622	6.476665	6.6402187	NM_024701	chr10:5693260-5693201	ASB13
A_33_P3389363	5.12279	4.9273357	XM_005258778	chr19:41256250-41256191	C19orf54
A_23_P357374	4.219474	4.235318	NM_178449	chr19:49925791-49925732	PTH2
A_32_P145051	3.8769605	3.8713975	XR_241774	chr5:34194462-34194521	LOC646652
A_23_P152768	7.7689185	7.48358	NM_001070	chr17:40765022-40765081	TUBG1
A_33_P3349840	10.766874	11.224058		chr2:024553470-024553529	
A_33_P3258041	4.63536	4.7558146	NM_001037277	chr1:235507616-235507675	GGPS1
A_33_P3339011	6.767873	6.73878	BC034587	chr12:100557089-100557030	GOLGA2P5
A_33_P3302344	6.264395	6.6710477	AK125438	chr10:98827927-98827868	LOC100128320
A_23_P359277	6.684575	4.8697834	NM_024930	chr5:60048063-60048004	ELOVL7
A_23_P133904	3.850552	3.3839095	NM_001011700	chr6:31497903-31497960	MCCD1
A_33_P3363898	12.503775	12.514994	NM_001070	chr17:40767187-40767246	TUBG1
A_23_P593	8.217001	8.626282	NM_021639	chr1:46093492-46093433	GPBP1L1
A_24_P374943	5.450938	6.242969	NM_001338	chr21:18924177-18924236	CXADR
A_23_P252855	10.629435	10.299533	NM_018321	chr5:34925048-34925357	BRIX1
A_33_P3711318	10.243943	10.340965	AK001439	chr8:104255997-104255938	LOC257152
A_33_P3226465	4.747389	4.4277177	NM_199340	chr17:62859079-62859020	LRRC37A3
A_33_P3241021	2.6311734	2.3900566	AK303383	chr12:9907223-9907164	CD69

A_23_P34345	2.3221061	2.3900566	NM_001078	chr1:101203783-101203842	VCAM1
A_33_P3240318	7.068386	7.287397	NM_005029	chr10:103990006-103989947	PITX3
A_23_P210763	8.194864	9.174535	NM_000214	chr20:10619120-10619061	JAG1
A_33_P3379535	7.5328856	7.849162	AK127601	chr12:50271771-50271712	
A_33_P3362886	13.071187	13.354326	AK056419	chr17:46124302-46124243	
A_23_P81529	6.143879	6.6058674	NM_002202	chr5:50689606-50689665	ISL1
A_33_P3404671	4.907455	4.546877	NR_024567	chr1:41154811-41154752	LOC100130557
A_23_P73747	2.3221061	2.3900566	NM_014782	chrX:100910394-100910335	ARMCX2
A_23_P26687	6.915638	6.9013505	NM_015421	chr16:8889594-8889535	TMEM186
A_23_P216118	4.104595	2.3900566	NM_080872	chr8:35651899-35651958	UNC5D
A_33_P3399218	5.3245378	5.2639413		chr1:001668677-001668736	
A_33_P3289835	4.163724	5.3215537	AB208851	chr1:52249650-52249709	OSBPL9
A_24_P569294	9.7378845	9.425997	NM_021107	chr19:39423438-39423496	MRPS12
A_23_P98402	10.881432	11.557556	NM_001040455	chr11:117068077-117068136	SIDT2
A_33_P3234410	10.860369	10.748753		chr16:003546255-003546314	
A_33_P3240229	7.9972854	7.703806	NM_004380	chr16:3775171-3775112	CREBBP
A_33_P3280845	5.7562184	5.105657	NM_006288	chr11:119288946-119288887	THY1
A_33_P3415440	6.7382007	6.578019	NM_006609	chr2:128062198-128062139	MAP3K2
A_32_P191503	8.173709	8.20682	NR_026999	chr7:39834139-39834198	LINC00265
A_33_P3732688	3.9682117	3.8700366	AK125175	chr10:31894736-31894795	LOC646034
A_24_P20200	8.037689	8.041981	NM_001267066	chr2:131888297-131890497	PLEKHB2
A_23_P18493	10.468655	10.898616	NM_080685	chr4:87735935-87735994	PTPN13
A_33_P3252630	5.398431	5.8067565	NM_001278677	chr19:24310723-24310782	ZNF254
A_23_P136547	9.016756	9.223785	NM_017733	chr4:532980-533039	PIGG
A_23_P211445	7.778734	7.422745	NM_016733	chr22:31675654-31675713	LIMK2
A_33_P3238671	4.6470313	4.222665	NM_152504	chr20:5753629-5753688	C20orf196
A_33_P3281940	8.554984	8.600946	NM_001278283	chr2:210884456-210884515	RPE
A_24_P927325	3.986276	3.319814	NM_015531	chr11:73745605-73745546	C2CD3
A_33_P3298159	5.165751	5.0467806	NM_000954	chr9:139876134-139876193	PTGDS
A_24_P392690	10.18659	10.731804		chr15:75613750-75613738	
A_23_P100486	9.705033	10.173553	NM_206824	chr16:31102433-31102374	VKORC1
A_33_P3212919	4.3843813	4.9454775	BX092453	chr13:21294526-21294585	
A_32_P149432	13.483522	13.348173	NM_001416	chr17:7481548-7481704	EIF4A1
A_23_P100001	9.035004	9.003644	NM_207446	chr15:93160848-93160789	FAM174B
A_24_P305784	4.137473	4.3174176	NM_145664	chrX:140097760-140097819	SPANXB2
A_23_P63897	11.865379	11.586371	NM_022802	chr10:126677529-126677470	CTBP2
A_33_P3317623	6.87353	7.3679523		chr19:54102971-54102912	LOC284379
A_23_P70045	12.249262	12.24349	NM_138610	chr5:134670784-134670725	H2AFY
A_23_P142994	7.7934647	7.6997433	NM_152945	chr2:178994231-178994290	RBM45
A_23_P2801	7.815775	7.8836904	NM_172373	chr13:41506984-41506925	ELF1
A_24_P237613	5.1800404	5.6525154	NM_182488	chr13:27643088-27643029	USP12
A_24_P64167	5.290777	5.259678	NM_000962	chr9:125157536-125157595	PTGS1
A_24_P227831	6.615014	6.7942705	NM_004996	chr16:16236024-16236083	ABCC1
A_33_P3338382	4.2168536	3.1891747	NM_080859	chr9:125563153-125563212	OR1K1
A_24_P393838	10.284694	8.680833	NM_014765	chr1:235273345-235273286	TOMM20
A_23_P314191	8.292097	8.394869	NM_015336	chr12:77247154-77247213	ZDHHC17
A_23_P105044	10.626795	10.616048	NM_021134	chr11:1974021-1974080	MRPL23
A_24_P175563	7.9792733	8.097334	NM_145698	chr10:27484212-27484153	ACBD5
A_24_P91405	9.29677	9.6065	NM_152601	chr19:12575490-12575431	ZNF709
A_23_P71379	5.651871	5.145991	NM_005672	chr8:143763976-143764035	PSCA
A_23_P92543	7.6110916	7.5128117	NM_152540	chr4:53740227-53740168	SCFD2
A_24_P381029	12.20641	11.929648	NM_006541	chr10:131977622-131977681	GLRX3
A_33_P3232871	4.4791803	4.2031813	NM_001167670	chr20:2797618-2797677	TMEM239
A_23_P354908	7.257366	6.728903	NM_015039	chr1:183217840-183217781	NMNAT2
A_23_P34700	4.538168	4.784437	NM_000364	chr1:201332504-201332445	TNNT2
A_32_P221256	9.103541	8.947869	NR_003682	chr17_gl000205_random:118716-118775	MGC70870
A_33_P3679768	8.917227	9.254961	AW303581	chr6:30922580-30922639	
A_33_P3390918	4.54605	4.9925957	NM_001007122	chr15:83428119-83428060	FSD2

A_23_P80336	9.029497	8.905421	NM_020243	chr22:39079816-39079875	TOMM22
A_23_P60028	13.271389	13.103125	NM_005648	chr8:74858931-74858872	TCEB1
A_33_P3322724	7.647891	7.74363	NM_007055	chr10:79736263-79736204	POLR3A
A_33_P3340424	9.679792	9.946117		chr10:044100153-044100094	
A_33_P3763846	4.221687	4.0503197	NM_002969	chr22:50695576-50695517	MAPK12
A_33_P3240328	11.974861	11.760796	NM_002653	chr5:134363483-134363424	PITX1
A_23_P259741	7.4947553	8.075097	NM_002971	chr3:18390316-18390257	SATB1
A_24_P299474	3.2258444	2.3900566	NM_001122679	chr5:167690063-167690122	TENM2
A_24_P309095	6.5043216	8.201024	NM_005045	chr7:103113096-103113037	RELN
A_33_P3244322	7.644427	7.5851126	NM_001282521	chr19:50170290-50170349	BCL2L12
A_33_P3815560	6.1705008	6.3540883	NM_018031	chr3:49052673-49052732	WDR6
A_24_P944144	7.318496	7.3719296	NM_080797	chr20:61518663-61518604	DIDO1
A_23_P118095	5.311389	5.5563173	NM_005061	chr16:1995873-1995598	RPL3L
A_33_P3276913	5.3923335	5.88481	BG533310	chr21:038471329-038471388	
A_32_P720220	4.901466	5.2113256	NR_036555	chr12:13524641-13524582	C12orf36
A_24_P203953	7.382641	7.4564056	XR_171055	chr10:11653619-11653678	LOC439951
A_33_P3342598	2.3221061	3.5349226	NM_006259	chr4:82009896-82009837	PRKG2
A_33_P3347241	7.1455913	7.3665733	NR_046228	chr1:142697765-142697706	ANKRD20A12P
A_23_P33045	15.36401	15.318853	NM_000987	chr17:8283239-8283180	RPL26
A_33_P3865368	7.9664645	7.965456	NR_046173	chr8:22960988-22961047	LOC254896
A_33_P3335840	5.4013543	5.6661577	NM_001006623	chr2:128493030-128492971	WDR33
A_33_P3303940	8.611416	8.565222	NM_024332	chrX:154350943-154351002	BRCC3
A_23_P52058	8.382174	8.413404	NM_005646	chr1:234528232-234528173	TARBP1
A_33_P3340792	5.1205773	4.9956822	NM_001243564	chr1:26772863-26772922	DHDDS
A_24_P219266	7.4426556	7.6011324	NM_153260	chr15:42834909-42834850	LRRC57
A_23_P30603	3.585108	2.3900566	NM_003649	chr6:110713880-110713821	DDO
A_33_P3281283	6.4887977	6.0907936	NM_005226	chr9:91619814-91619873	S1PR3
A_33_P3290296	7.7582436	7.9469647	AK125829	chr10:112663496-112663437	BBIP1
A_24_P360722	4.905406	5.138221	NM_014974	chr10:322111-322052	DIP2C
A_24_P143653	4.877689	4.6643257		chr2:130751451-130751512	XLOC_014512
A_23_P165707	6.0261564	5.4132633	NM_174898	chr2:99900843-99900784	LYG1
A_33_P3621701	7.730931	7.9957533	BC048428	chr22:48939193-48939134	
A_32_P76720	5.2760625	5.055211	NM_001031701	chr12:104166903-104166844	NT5DC3
A_33_P3400224	8.189749	8.499444	NM_014934	chr13:96285544-96285488	DZIP1
A_23_P254254	7.2073135	7.68357	NM_000199	chr17:78183396-78183337	SGSH
A_33_P3252594	5.7833557	5.862241		chr1:001824064-001824123	
A_23_P300781	8.440714	8.236721	NM_013316	chr7:135046706-135046647	CNOT4
A_33_P3390086	8.512288	8.253607		chr3:075429579-075429520	
A_33_P3314146	6.336548	5.511552	NM_001042533	chr3:97660805-97660746	MINA
A_23_P35871	7.6608143	7.7280116	NM_024680	chr11:19245734-19245675	E2F8
A_33_P3220445	8.076875	8.100987	NM_001001419	chr5:135513109-135513168	SMAD5
A_23_P92614	6.1320457	5.8082113	NM_133636	chr4:84339272-84337986	HELQ
A_33_P3338693	5.931076	5.7631006	NM_003081	chr20:10287989-10288048	SNAP25
A_33_P3292650	2.3221061	2.3900566	NM_207407	chr4:68918975-68918916	TMPRSS11F
A_23_P141405	14.481888	14.503544	NM_002512	chr17:49248919-49248978	NME2
A_23_P91590	13.209328	13.085549	NM_002882	chr22:20114529-20114588	RANBP1
A_23_P8571	7.4014635	7.297626	NM_080744	chr7:76018708-76018649	SRCRB4D
A_24_P36745	7.980573	7.9324236	NM_144970	chrX:40489315-40489256	CXorf38
A_33_P3399634	4.787907	4.873261	NM_001080521	chr11:13032588-13032647	RASSF10
A_23_P257911	9.802909	9.843943	NM_001032410	chr21:30426744-30426803	USP16
A_32_P181020	6.252643	6.6014023	NM_005510	chr6:31937930-31937787	DXO
A_23_P357760	6.5505505	6.502647	XM_005274516	chrX:2832639-2832580	ARSD
A_32_P112493	4.8679266	5.137127	NM_138370	chr2:42285006-42285065	PKDCC
A_23_P209944	4.701848	5.07748	NM_017750	chr2:85569931-85569872	RETSAT
A_23_P370479	6.1564646	5.9038496	NM_015352	chr20:30826235-30826294	POFUT1
A_23_P131383	8.997145	8.837233	NM_018062	chr2:58386536-58386477	FANCL
A_23_P406702	8.5167675	8.224926	NM_203459	chr1:200829250-200829309	CAMSAP2
A_33_P3363355	3.6746798	3.4942522	NM_022377	chr19:10399139-10399198	ICAM4
A_33_P3237150	2.3221061	2.3900566	NM_001200	chr20:6760810-6760869	BMP2

A_24_P394698	6.9649267	6.907802	NM_181747	chr7:103824440-103808964	ORC5
A_33_P3223397	5.125038	4.698326		chr12:49486752-49486811	
A_33_P3354464	7.4228215	6.877927	NM_005576	chr15:74241855-74241914	LOXL1
A_33_P3256480	8.266092	7.639557			
A_33_P3387771	7.2226696	6.3071094	NM_001039590	chrX:41095755-41095814	USP9X
A_24_P208998	5.475148	4.800745	NM_001656	chr5:64886092-64886033	TRIM23
A_33_P3299882	7.696659	6.5717278	NM_001254752	chr8:97239371-97239312	UQCRB
A_23_P63870	5.7236147	5.8709784	NM_144660	chr10:76936254-76936313	SAMD8
A_33_P3239347	7.0405784	6.9200745	NM_006167	chr8:23536992-23536933	NKX3-1
A_24_P46334	8.344564	8.484477	NM_032547	chr4:141302764-141302823	SCOC
A_33_P3411985	5.6198673	5.7273183		chr9:125707651-125707710	RABGAP1
A_23_P81811	7.4650598	7.4067907	NM_080604	chr6:43473898-43473957	TJAP1
A_24_P873414	11.020468	10.770705	NM_001267062	chr2:131907177-131907236	PLEKHB2
A_23_P27636	7.071212	7.2576957	NM_144566	chr19:12060977-12061036	ZNF700
A_23_P429461	6.549695	6.7628455	NM_145059	chr16:70513961-70514020	FUK
A_24_P167496	4.7135525	4.4536295	NM_031292	chr12:44122939-44122880	PUS7L
A_24_P177568	3.8434577	4.2999516	NM_133473	chr19:21367846-21367905	ZNF431
A_23_P309989	5.998763	6.086684	NM_014924	chr14:55833888-55833829	ATG14
A_23_P35725	2.3221061	2.3900566	NM_031418	chr11:26684671-26684730	ANO3
A_33_P3368339	4.342977	4.8540516	NM_001045477	chr9:99704513-99704572	NUTM2G
A_33_P3314073	3.2581978	2.3900566		chr19:039726978-039726919	
A_24_P207479	6.3677025	6.3760476	NM_133328	chr19:42703180-42703121	DEDD2
A_24_P416660	9.4223	9.283353	NM_001031734	chr19:10421203-10421144	FDX1L
A_33_P3220530	10.236645	9.713707	NM_006275	chr20:42092182-42092241	SRSF6
A_23_P251232	9.575437	9.763165	NR_001543	chrY:21238951-21094689	TTTY14
A_32_P11471	13.893914	13.815597	NM_001997	chr11:64888495-64888262	FAU
A_23_P389391	5.1438036	5.297259	NM_005072	chr16:67978585-67978526	SLC12A4
A_33_P3334443	6.9390464	7.2522726	NM_001252271	chr1:93307814-93307755	FAM69A
A_24_P316430	7.101344	7.332845	NM_002526	chr6:86176992-86181005	NT5E
A_32_P104772	2.3221061	2.3900566		chr9:44125576-44125517	XLOC_014512
A_33_P3335233	6.5215397	6.868059	NM_025130	chr10:71017113-71017172	HKDC1
A_23_P501339	4.5805035	5.042549	NM_006319	chr16:29871908-29870792	CDIPT
A_33_P3405038	5.6141896	5.945197	NR_024564	chr20:19223005-19222946	LOC100130264
A_24_P231057	8.690812	8.521143	NM_148894	chr4:13584292-13583892	BOD1L1
A_24_P211206	9.154325	9.496357	NM_001043352	chr1:154129925-154129866	TPM3
A_23_P255257	7.2215576	7.405538	NM_015397	chr9:34086957-34086898	DCAF12
A_33_P3306510	10.041447	9.985661	NM_001278209	chr6:17615465-17615406	NUP153
A_23_P144453	10.028687	10.371544	NM_033296	chr4:6643751-6643810	MRFAP1
A_33_P3420665	3.9347653	4.434273	AK125780	chr6:42131097-42131156	GUCA1A
A_23_P382705	5.2383523	5.1108904	NM_152588	chr12:83527818-83527877	TMTC2
A_33_P3430391	4.469102	4.9995174	XR_241740	chr5:10505167-10505108	LOC389273
A_33_P3387524	6.3984184	6.5359216	NM_001114120	chr1:68941036-68940977	DEPDC1
A_33_P3250343	9.272802	9.476523	NR_028326	chr11:128579-128520	LINC01001
A_33_P3375950	12.825949	12.830639		chr11:111760066-111760007	
A_24_P288754	7.3499055	7.0953226	NM_002641	chrX:15337804-15337745	PIGA
A_33_P3214849	10.441019	10.481125	NM_153705	chr11:108343124-108343065	KDELC2
A_33_P3255229	4.1007376	4.982411	BC066361	chr4:140453903-140453844	SETD7
A_33_P3234060	4.184969	4.29932	NM_207366	chr7:55861296-55861237	SEPT14
A_33_P3343785	10.692845	10.598837	NM_052865	chr20:17971684-17971743	MGME1
A_33_P3343727	8.644885	8.824068	AK308514	chr1:22332477-22332536	CELA3A
A_23_P316460	7.9053154	7.2215576	NM_138434	chr7:150029689-150029748	ZBED6CL
A_33_P3214650	8.837922	8.33489	NM_000544	chr6:32793281-32793222	TAP2
A_33_P3233666	7.116504	7.109154	NM_001104546	chr10:92665607-92665666	RPP30
A_33_P3328928	5.8276167	6.158279	NM_017593	chr4:79800024-79800083	BMP2K
A_33_P3414228	6.0792513	6.3903065		chr15:022748670-022748611	
A_33_P3348395	4.2605658	4.1601877	XM_005253817	chr12:2958456-2958397	
A_32_P159651	7.5452466	7.7164793	NM_003884	chr3:20195519-20195578	KAT2B
A_33_P3261293	3.9136825	3.9782984	NM_015881	chr11:11986222-11986163	DKK3
A_33_P3370959	4.6504917	4.789667	NM_001732	chr6:26510090-26510149	BTN1A1

A_23_P126613	4.7261443	4.44336	NM_080429	chr1:154297730-154297789	AQP10
A_33_P3307735	4.6533613	4.664393	NM_002547	chrX:67263344-67263285	OPHN1
A_33_P3356220	10.192258	10.341988	NM_001165937	chr17:37819663-37819722	STARD3
A_33_P3220643	8.890718	9.129967	NM_001002913	chr9:130476294-130476235	PTRH1
A_24_P44462	9.769889	9.771654	NM_000366	chr15:63353441-63354441	TPM1
A_24_P232790	5.4301987	5.7015686	NM_001271507	chr14:70037055-70036996	CCDC177
A_23_P143845	7.5380583	7.211272	NM_015508	chr3:156424310-156424369	TIPARP
A_23_P78134	10.457395	10.312594	NM_001014764	chr17:3572740-3572799	EMC6
A_33_P3277808	4.3568783	4.7938843		chr10:124438490-124438549	LOC101928125
A_33_P3319542	4.0283704	4.444925	NM_015063	chr19:47931982-47931923	SLC8A2
A_32_P55241	8.510844	9.329971	NM_001007538	chr13:26619404-26619345	SHISA2
A_23_P218412	7.000963	6.888089	NM_004890	chr17:4863798-4863599	SPAG7
A_33_P3238941	5.067465	5.0299563	NM_147195	chr9:38575669-38575610	ANKRD18A
A_23_P38813	4.7949853	5.1380353	NM_207333	chr19:53384063-53384004	ZNF320
A_23_P147822	7.772952	7.9786296	NM_022772	chr11:726918-726977	EPS8L2
A_32_P108655	11.182416	11.320145	NM_001005353	chr1:65692632-65692691	AK4
A_24_P333716	8.058305	7.725787	NM_022459	chr13:21353576-21353517	XPO4
A_24_P813147	9.55552	9.554891	NM_177987	chr10:93404-93345	TUBB8
A_33_P3259522	8.06004	8.250589	NM_201546	chr1:54605389-54605330	CDCP2
A_33_P3290124	9.303995	9.367606	NM_001142459	chr7:150872848-150872789	ASB10
A_23_P74435	7.9606743	8.033814	NM_024097	chr1:43240919-43240978	C1orf50
A_33_P3272390	6.6502395	5.901946	NM_006267	chr2:109402125-109402184	RANBP2
A_24_P69538	5.4119425	5.46148	NM_138554	chr9:120476856-120476915	TLR4
A_33_P3384312	4.153049	4.7977386		chr8:144640041-144640100	
A_24_P215804	8.70127	8.67137	NM_016951	chr16:66599900-66599959	CKLF
A_24_P45379	5.9812803	5.8137035	NM_014412	chr1:174980527-174980586	CACYBP
A_33_P3414017	6.1218953	6.3004065		chr7:002299242-002299301	
A_23_P44674	2.3221061	2.3900566	NM_001311	chr14:105954700-105954841	CRIP1
A_24_P219785	8.045635	8.605183	NM_005184	chr19:47111489-47111548	CALM3
A_24_P139943	6.537677	6.6220837	NM_022460	chr2:20817935-20817876	HS1BP3
A_33_P3312104	4.7281837	4.9632845	NM_025099	chr17:8130253-8130194	CTC1
A_32_P96719	9.195974	9.551687	NM_024745	chr16:46615134-46615075	SHCBP1
A_32_P71447	9.800266	9.762145	NM_015261	chr11:134022687-134022628	NCAPD3
A_24_P231104	5.763902	5.948556	NM_001003679	chr1:66100649-66100708	LEPR
A_23_P259521	8.789482	8.307688	NM_018268	chr5:76729022-76728963	WDR41
A_23_P37359	5.7960534	5.6527176	NM_014430	chr14:24774705-24774646	CIDEB
A_33_P3269806	4.3019686	4.25975	NM_178523	chr19:52617720-52617661	ZNF616
A_32_P15799	14.239221	14.299968	NM_005517	chr1:26802399-26802458	HMGN2
A_33_P3575529	4.7621846	4.6338997	AF309699		OR5E1P
A_23_P319013	5.4947095	5.923402	NM_152604	chr19:37734004-37734063	ZNF383
A_24_P186274	5.0779324	4.9564433	NM_001007563	chr9:38411463-38411404	IGFBPL1
A_23_P254573	9.208345	8.907711	NM_014377	chr7:102953432-102953070	DNAJC2
A_33_P3383246	4.5115633	4.1449385	NM_001005177	chr11:4824764-4824705	OR52R1
A_23_P91829	9.254961	9.625171	NM_080927	chr3:98517685-98517626	DCBLD2
A_23_P214037	14.805878	14.642805	NM_002520	chr5:170832381-170834736	NPM1
A_23_P343237	6.3746076	6.5956993	NM_144567	chr1:213181760-213181701	ANGEL2
A_33_P3464855	7.735581	7.590841	AL050097	chr3:196858864-196858805	DKFZP586B0319
A_33_P3287428	5.01541	4.3709803	BC107850	chr17:43578758-43578699	LRRC37A4P
A_23_P210608	8.262327	8.629935	NM_006526	chr20:52184137-52184078	ZNF217
A_23_P27147	12.47343	12.580866	NM_001002244	chr17:79858019-79858078	ANAPC11
A_23_P211806	8.489331	8.440352	NM_017724	chr3:37094855-37094796	LRRFIP2
A_33_P3333078	6.5920258	6.4346986	NM_020732	chr6:157531787-157531846	ARID1B
A_23_P138717	11.674963	11.870502	NM_001005339	chr10:121259582-121259523	RGS10
A_23_P153767	7.695752	7.5968137	NM_014371	chr19:15508018-15491422	AKAP8L
A_33_P3257943	15.403006	15.36401	NM_000987	chr17:8280963-8280909	RPL26
A_33_P3410284	3.6740303	3.421444	XM_005254034	chr13:99498278-99498219	DOCK9
A_33_P3248586	6.6490602	6.733033	NM_001127386	chr10:135485016-135485075	DUX4L2
A_33_P3336915	10.960623	11.023692	AB209077	chr3:134070878-134070819	
A_24_P294832	8.608422	8.501745	NM_003463	chr6:64292789-64292848	PTP4A1

A_33_P3341722	9.772537	9.886154	CU692082	chr5:177989089-177989030	COL23A1
A_24_P56317	9.837924	9.781103	NM_144778	chr13:98046226-98046285	MBNL2
A_23_P351734	7.432471	7.5522075	NM_015102	chr1:5922948-5922889	NPHP4
A_33_P3281191	11.065117	11.458455	NM_002508	chr1:236139213-236139154	NID1
A_23_P352535	4.967139	5.102986	NM_015568	chr20:37551462-37551521	PPP1R16B
A_33_P3305208	10.926246	10.361845		chr11:077024996-077025055	
A_33_P3267263	8.470314	8.42036	NR_004400	chr1:17067115-17067174	RNVU1-18
A_33_P3399433	13.267508	12.911448	NM_001258430	chr20:3734216-3734157	C20orf27
A_33_P3839334	6.185747	5.619067	AK055939	chr5:32175300-32175359	
A_23_P22625	8.134212	7.9046955	NM_001042537	chrX:135129073-135129132	SLC9A6
A_33_P3370555	2.3221061	2.3900566	D25284	chr14:88429749-88429690	GALC
A_23_P142389	3.589046	3.9204366	NM_205834	chr19:35758434-35758493	LSR
A_33_P3356230	2.3221061	2.3900566	NM_001004306	chr17:20766856-20766797	CCDC144NL
A_33_P3325023	9.123701	9.525011	NM_015701	chr2:54045197-54045256	ERLEC1
A_23_P314070	11.929648	11.963112	NM_004308	chr11:46698725-46698666	ARHGAP1
A_33_P3381948	9.800023	9.839824	NM_001080436	chr19:34991985-34992044	WTIP
A_32_P214925	4.6242495	5.010709	NM_173678	chr7:143422091-143422150	FAM115C
A_33_P3278407	9.59757	9.596351	NM_001207008	chr6:168265361-168265420	MLLT4
A_23_P316487	6.4479	6.3926897	NM_138635	chr7:44867532-44867473	H2AFV
A_33_P3318763	3.6591873	2.3900566	XR_242313	chr7:92546261-92546320	
A_33_P3228862	5.2798133	5.18885	NM_001129890	chr8:92231287-92231346	LRRC69
A_23_P129128	5.0588627	5.2053676	NM_152334	chr15:102194746-102194687	TARSL2
A_33_P3353242	5.7694173	5.632309	NM_001540	chr7:75931924-75931983	HSPB1
A_33_P3304963	4.555053	4.979901	NM_001143759	chr10:134165146-134165205	LRRC27
A_24_P257478	3.1595542	3.0811496	NM_198721	chr4:109780888-109780829	COL25A1
A_23_P302060	4.82601	4.665757	NM_176891	chr9:21481161-21481102	IFNE
A_23_P128147	15.5145445	15.430389	NM_006082	chr12:49521692-49521633	TUBA1B
A_24_P70993	6.654489	6.8102784	NM_002414	chrX:2638454-2640715	CD99
A_24_P158903	5.9548836	6.1067867	NM_016123	chr12:44181072-44181131	IRAK4
A_23_P50735	5.687347	6.219974	NM_001029997	chr19:35232971-35233030	ZNF181
A_33_P3227284	10.212454	10.139391	NM_181672	chrX:70795533-70795592	OGT
A_23_P58588	6.8072724	7.0715423	NM_003062	chr5:168093426-168093367	SLIT3
A_33_P3406505	5.5402284	5.667593	NM_006532	chr19:18561746-18561687	ELL
A_33_P3306103	4.2474904	3.7160702	NM_005795	chr2:188210713-188210654	CALCRL
A_23_P367816	8.098387	8.484755	NM_032167	chr16:12667879-12667938	SNX29
A_33_P3220853	4.476342	4.5184884		chr2:213698031-213698090	XLOC_014512
A_32_P58280	4.477086	5.305481	NM_019053	chr10:94712120-94714426	EXOC6
A_33_P3354990	5.600584	5.8511114	AK296242	chr6:158438767-158438826	SYNJ2
A_23_P146728	9.359292	9.59757	NM_033087	chr9:101980025-101979966	ALG2
A_32_P149251	6.6091037	6.3453584	NM_152686	chr5:138747474-138747415	DNAJC18
A_23_P385067	2.3221061	3.916767	NM_053277	chr21:36090152-36090211	CLIC6
A_33_P3359306	10.03593	10.142222	NM_004689	chr14:105936735-105936794	MTA1
A_33_P3212650	8.858154	8.807254	BC061520	chr6:125621739-125614053	HDDC2
A_33_P3391596	7.279097	7.478149	NM_001105206	chr6:112429200-112429141	LAMA4
A_33_P3240512	8.311843	7.2548265	NM_138444	chr13:77454410-77454351	KCTD12
A_33_P3225250	4.698326	4.939168	NM_139178	chr11:43911306-43911365	ALKBH3
A_32_P177097	7.52836	7.226522	NM_024341	chr19:7087867-7087926	ZNF557
A_33_P3402838	5.7945023	4.631441	NM_001271507	chr14:70038318-70038259	CCDC177
A_33_P3226665	4.387325	5.2427177	NM_198542	chr19:58016708-58016767	ZNF773
A_33_P3416350	6.7626133	6.94847	NM_147152	chr2:24535177-24535118	ITSN2
A_23_P133995	10.350857	10.3334465	NM_016059	chr6:36822790-36822731	PPIL1
A_23_P84782	10.031974	9.884997	NM_015963	chr2:242524073-242524014	THAP4
A_24_P932016	7.603554	7.700069	NM_031407	chrX:53612078-53612019	HUWE1
A_23_P254768	2.3221061	3.6260655	NR_002139	chr6:29759340-29759281	HCG4
A_23_P126689	6.1210637	6.221509	NM_001014443	chr1:161134724-161134869	USP21
A_23_P42718	9.385847	9.603503	NM_004289	chr7:26225697-26225756	NFE2L3
A_23_P8339	11.262677	11.198201	NM_014161	chr6:160219094-160219153	MRPL18
A_24_P145122	8.454624	8.390245	NM_022731	chr1:205682299-205682240	NUCKS1
A_24_P318897	7.778471	7.682199	NM_001042633	chr20:44471388-44471447	SNX21

A_33_P3294314	4.956098	4.9425673		chr2:009696652-009696593	
A_23_P144384	10.728455	11.009319	NM_017423	chr4:174244878-174244937	GALNT7
A_33_P3612740	6.767065	6.9059525	NM_001032730	chr1:22054468-22054409	USP48
A_23_P155351	4.6007233	5.0160527	NM_000060	chr3:15686430-15686489	BTD
A_32_P175539	9.1622305	9.335227	NM_002902	chr15:77241545-77241604	RCN2
A_33_P3327847	9.664207	9.396374	NM_003901	chr10:72640764-72640823	SGPL1
A_33_P3316026	4.875279	4.754073	NM_003367	chr19:35761626-35761685	USF2
A_23_P169887	9.993919	9.932953	NM_001521	chr2:27548721-27548698	GTF3C2
A_23_P253389	5.3780637	5.2810183	NM_003040	chr7:150771553-150771798	SLC4A2
A_23_P40718	6.214266	5.7582965	NM_001003828	chr22:44553927-44559760	PARVB
A_33_P3286002	4.378585	3.9834235	AK125618	chr16:31105666-31105607	VKORC1
A_23_P350574	6.9085865	6.371586	NM_001002901	chr1:161697713-161697772	FCRLB
A_33_P3260868	4.940919	4.7749853		chrX:070021127-070021068	
A_33_P3307945	7.924081	7.8521566	NM_001277372	chr2:202970568-202970627	LOC100652824
A_23_P85640	5.291043	5.399542	NM_005540	chr1:38327931-38327872	INPP5B
A_33_P3268723	4.4006996	4.513175	NM_019110	chr6:28213229-28213170	ZKSCAN4
A_33_P3295803	4.058416	4.43044	NM_032846	chr14:21928385-21928326	RAB2B
A_33_P3276329	11.230084	11.560005	AK123110	chr7:74803807-74803748	
A_32_P50522	8.960193	9.298838	NM_000801	chr20:1349950-1349891	FKBP1A
A_33_P3303031	11.71756	12.036167	BC133006	chr20:44563229-44563170	FLJ40606
A_23_P252155	11.008577	11.049141	NM_018387	chr9:125887911-125887852	STRBP
A_33_P3388882	4.507998	5.0060673	NR_027425	chr8:11973667-11973726	FAM66D
A_23_P80739	6.504403	6.7923355	NM_006225	chr3:38049181-38049122	PLCD1
A_23_P70670	7.3120055	7.0192537	NM_004233	chr6:14136689-14136748	CD83
A_23_P208698	9.259395	9.410921	NM_002103	chr19:49471657-49471598	GYS1
A_24_P706752	6.445773	6.491426	NM_030821	chr4:110631436-110631377	PLA2G12A
A_33_P3233841	8.746979	9.130774	NM_002184	chr5:55238572-55238513	IL6ST
A_23_P51487	9.478962	8.882342	NM_018284	chr1:89472608-89472549	GBP3
A_24_P82074	4.778125	4.8951983	NM_017437	chr14:92629708-92629767	CPSF2
A_33_P3230658	8.203582	8.004706	NM_005999	chr1:231696888-231696947	TSNAX
A_33_P3832857	8.086163	7.919725	NM_018097	chr15:42859124-42859183	HAUS2
A_23_P152548	9.0256405	9.41423	NM_021626	chr17:55083685-55083744	SCPEP1
A_24_P336596	4.664393	4.9121585	NM_030663	chr1:152856959-152857018	SMCP
A_23_P90523	5.9409943	6.5252943	NM_024578	chr19:17339686-17339745	OCEL1
A_24_P791829	8.237112	7.6926284	NM_020772	chr17:27583112-27583053	NUFIP2
A_33_P3314276	5.781893	6.1751175	NM_181718	chr16:29917316-29917375	ASPHD1
A_33_P3236218	4.5588183	4.66014		chr5:017635043-017635102	
A_32_P9468	5.423805	5.284727	NM_001031617	chr7:1004585-1004526	COX19
A_23_P253317	4.0009155	4.1137915	NM_013308	chr3:150916346-150916287	GPR171
A_24_P373152	8.517958	7.0476274	NM_021914	chr14:35179741-35179682	CFL2
A_24_P935009	7.6625757	7.939837	NM_005207	chr22:21307425-21307484	CRKL
A_32_P216426	11.316116	12.022421	NM_001011724	chr13:53217421-53217480	HNRNPA1L2
A_33_P3273267	5.340018	5.929988		chr21:014469021-014468962	
A_23_P132226	7.1368923	7.244713	NM_001008566	chr22:26922050-26921991	TPST2
A_32_P219660	5.1766086	3.6328933	NM_013453	chrX:140678815-140678874	SPANXA1
A_33_P3399875	6.857108	6.9976983	NM_080825	chr20:32250153-32250212	C20orf144
A_33_P3215028	8.766335	8.665973	NM_152734	chr6:36892266-36892325	C6orf89
A_33_P3265526	3.0665574	3.029858	NM_001110798	chr13:41932794-41932853	NAA16
A_23_P65506	3.9567325	3.275782	NM_000347	chr14:65236444-65236385	SPTB
A_32_P113436	9.56349	10.511777	NM_001011724	chr13:53216921-53216980	HNRNPA1L2
A_33_P3219811	6.300339	6.2230353	XR_245337	chr9:139873794-139873853	PTGDS
A_23_P316974	7.651049	7.130479	NM_003898	chr6:158519231-158519290	SYNJ2
A_24_P41371	4.226743	3.417426	NM_017721	chr19:14034237-14034388	CC2D1A
A_23_P154500	7.455567	7.7582436	NM_175629	chr2:25456474-25456415	DNMT3A
A_33_P3369766	9.267673	9.474898	NM_014166	chr13:48651179-48651120	MED4
A_23_P14302	7.225155	7.3864155	NR_026779	chr14:95873748-95873689	LINC00341
A_23_P126008	6.597178	6.4895706	NM_015112	chr1:46501344-46501403	MAST2
A_23_P376727	4.553671	5.7223077	NM_138326	chr2:135630187-135655945	ACMSD
A_24_P350683	4.0090218	3.783859	BC012121	chr1:27432191-27432132	SLC9A1

A_24_P136866	2.8312836	3.7530584	NM_021097	chr2:40657389-40657330	SLC8A1
A_23_P403081	6.0457153	6.3941	NM_198566	chr5:43492836-43492367	C5orf34
A_32_P41065	4.8092394	3.73493	NM_001017395	chr3:129366905-129366846	TMCC1
A_33_P3395074	5.259678	5.3933115	NM_020360	chr17:7294037-7293978	PLSCR3
A_32_P58425	9.082052	9.488208	NM_153812	chr1:6683812-6683871	PHF13
A_23_P34956	11.881024	11.706069	NM_031420	chr1:151732274-151732215	MRPL9
A_24_P206702	4.5801187	4.7766724	NM_001199206	chr11:94039731-94039790	FOLR4
A_33_P3632866	4.15957	4.4624724	NM_153710	chr9:136270972-136271031	C9orf96
A_23_P845	9.416941	9.204849	NM_032174	chr1:161200179-161200238	TOMM40L
A_33_P3373775	3.9061565	3.903749	NM_001010922	chr1:114419524-114419465	BCL2L15
A_33_P3341269	3.9799573	4.683971		chr19:040176272-040176331	
A_23_P7783	9.087137	9.506932	NM_016604	chr5:137772365-137772424	KDM3B
A_33_P3326349	5.465473	5.1921296	NM_015327	chr1:156238143-156238084	SMG5
A_23_P216610	7.8825126	8.128647	NM_022486	chr9:114803217-114803158	SUSD1
A_23_P333592	4.2322693	4.425459	NM_173631	chr19:57889811-57889870	ZNF547
A_33_P3360917	7.0250463	6.542277	NM_018085	chr1:201853338-201853397	IPO9
A_23_P84872	6.193036	6.7104645	NM_024077	chr9:91973999-91974058	SECISBP2
A_24_P328504	10.589925	10.291437	NM_007237	chr2:231134638-231135329	SP140
A_23_P50815	4.9663167	5.1679735	NM_020659	chr19:54947816-54947875	TTYH1
A_23_P431388	7.1506286	5.342674	NM_144569	chr1:32256093-32256034	SPOCD1
A_33_P3419785	11.774466	12.715825	NM_004052	chr10:133781940-133781881	BNIP3
A_33_P3357658	6.739374	6.199734	NM_003484	chr12:66309240-66309299	HMGA2
A_32_P110472	3.6815782	3.7233605	NM_203395	chr6:150725304-150725363	IYD
A_33_P3294415	4.454549	4.777166		chr17:016893491-016893550	
A_23_P131588	3.8209114	3.608161	NM_014482	chr2:69093132-69093073	BMP10
A_23_P92517	3.904946	4.1012607	NM_031956	chr4:147741319-147724821	TTC29
A_23_P154457	5.5143976	5.1647234	NM_024894	chr2:10711198-10711139	NOL10
A_33_P3393684	4.6490674	4.3735623	NM_001013661	chr1:159824170-159824111	VSIG8
A_33_P3348782	3.5460422	3.4172914	NM_030622	chr19:41712616-41712675	CYP2S1
A_24_P304071	6.230054	6.043813	NM_001547	chr10:91066613-91066672	IFIT2
A_33_P3404989	4.0053477	4.701006	NM_003536	chr6:27778255-27778314	HIST1H3H
A_24_P392480	6.0480757	6.097716	NM_001012643	chr19:46393942-46393883	MYPOP
A_24_P269062	5.9572716	5.8069324	NM_030964	chr5:141690630-141690571	SPRY4
A_23_P44195	6.9755077	7.166331	NM_138962	chr17:55757022-55757081	MSI2
A_23_P7727	2.3221061	5.4174223	NM_001884	chr5:82937382-82937323	HAPLN1
A_33_P3284019	8.934218	8.977723	NM_001012426	chr6:41567638-41567697	FOXP4
A_33_P3239744	4.862933	6.252349	XR_109661	chr21:32932572-32932631	LOC150051
A_24_P13285	4.6814823	5.7741623	NM_006741	chr12:54973524-54973465	PPP1R1A
A_33_P3274528	10.508126	10.516293		chr9:088842307-088842248	
A_32_P73045	8.675007	9.08893	NM_018686	chr12:22214343-22215230	CMAS
A_33_P3213493	4.679044	4.9914947	NM_014232	chr17:8064986-8064927	VAMP2
A_23_P11461	11.330078	11.824636	NM_001032288	chr20:48700691-48699418	UBE2V1
A_33_P3771039	12.652946	12.589778		chr7:121081198-121081139	
A_24_P262127	6.05955	6.6514034	NM_004165	chr16:66956234-66956175	RRAD
A_23_P383986	8.378722	8.164878	NM_015892	chr10:125767360-125767301	CHST15
A_23_P20107	6.9499354	7.15423	NM_015917	chr7:142961668-142961727	GSTK1
A_24_P56252	9.596842	9.039458	NM_002480	chr12:80167752-80167693	PPP1R12A
A_33_P3376925	9.076658	9.237932		chrX:093574757-093574816	
A_33_P3257015	13.638138	13.711731		chr17:061714414-061714473	
A_33_P3352522	8.437703	8.126805	AK092862	chr13:114548583-114548642	GAS6-AS1
A_33_P3213596	4.8580008	5.505977	NM_015028	chr3:170781729-170781670	TNIK
A_33_P3359047	5.150304	5.410092	NM_001195685	chr2:150327475-150327534	LYPD6
A_33_P3235546	4.6959043	5.347135	NM_014747	chr1:41091945-41091886	RIMS3
A_33_P3361147	6.044768	5.9522047	AK130707	chr2:111995399-111995340	
A_23_P66798	4.6474757	4.596497	NM_002276	chr17:39679977-39679918	KRT19
A_23_P63660	9.457227	9.749008	NM_032333	chr10:82191890-82191949	FAM213A
A_33_P3296427	2.3221061	3.03102	NR_015407	chr1:238643763-238643704	LOC339535
A_23_P320878	4.4412904	5.0380445	NM_206914	chr12:58175640-58175699	METTL21B
A_23_P256716	9.19921	9.326044	NM_003601	chr4:144469182-144469241	SMARCA5

A_33_P3422802	7.7007127	7.2064223	NM_025218	chr6:150294682-150294741	ULBP1
A_23_P152356	8.061798	8.147058	NM_003454	chr16:3273438-3273379	ZNF200
A_23_P74097	8.001297	8.494388	NM_003198	chr1:24086017-24086076	TCEB3
A_23_P258093	9.436943	9.617615	NM_006411	chr6:32136254-32136195	AGPAT1
A_23_P168909	3.984783	4.6902776	NM_012082	chr8:106816325-106816384	ZFPM2
A_23_P49816	3.4704285	3.208131	NM_018404	chr17:29285179-29285238	ADAP2
A_23_P384044	6.3955464	3.8858366	NM_152495	chr1:224928164-224928223	CNIH3
A_33_P3211174	5.4326105	5.396511	NM_015897	chr19:4033470-4033529	PIAS4
A_24_P11587	8.036326	8.098239	NM_002618	chr2:61278664-61278723	PEX13
A_33_P3327333	6.0450172	6.0719595	NM_001197123	chr19:50164084-50164025	IRF3
A_23_P431939	6.413825	6.9569383	NM_001531	chr1:181024482-181024541	MR1
A_33_P3288805	3.3022647	4.0730977	NM_001134774	chr11:66033593-66033652	KLC2
A_24_P363477	6.0316124	6.3116655	NM_013393	chr7:2274904-2274845	FTSJ2
A_32_P91902	8.84856	8.717062	NM_030927	chr10:82281858-82281917	TSPAN14
A_24_P84608	8.008401	7.8895426		chr9:022748048-022748109	
A_33_P3259077	8.023443	8.112614	NR_103736	chr12:131850872-131850931	LOC338797
A_33_P3762913	5.4383855	5.5176673	NR_039981	chr7:104628139-104628080	LINC01004
A_23_P74178	9.258051	9.482659	NM_001525	chr1:32092654-32092713	HCRTR1
A_23_P362046	10.357729	10.300921	NM_138779	chr13:103418860-103418801	TEX30
A_23_P74320	7.4223742	7.534216	NM_020423	chr1:169822393-169822334	SCYL3
A_32_P80068	9.998257	10.103716	NM_001004419	chr12:9848143-9848202	CLEC2D
A_23_P142714	8.884228	8.706629	NM_003705	chr2:172641422-172641363	SLC25A12
A_33_P3320804	10.518592	10.575333	NM_001242829	chr3:49761787-49761728	IP6K1
A_33_P3315223	7.1244073	6.718448	NM_006805	chr5:137087151-137087092	HNRNPA0
A_33_P3418516	4.517539	4.5095553	NM_001949	chr6:20490602-20490661	E2F3
A_33_P3324655	5.271161	4.832362	NM_025115	chr8:33356300-33356241	TTI2
A_33_P3391727	6.6993275	6.7954903	NM_016538	chr17:79870353-79870294	SIRT7
A_23_P72737	4.0736966	5.909872	NM_003641	chr11:315209-315268	IFITM1
A_24_P307306	10.906977	10.979827		chr1:120139395-120139336	
A_33_P3350621	6.889866	6.9163713	NM_001111101	chr2:68511385-68511326	CNRIP1
A_33_P3401701	4.1898804	4.173471	NM_001957	chr4:148441029-148441088	EDNRA
A_23_P28057	11.253003	11.237803	NM_003249	chr19:2813491-2813550	THOP1
A_32_P154473	5.9609365	5.3095818	NM_004522	chr2:149883078-149883137	KIF5C
A_33_P3231242	11.257883	11.052067		chr2:170933793-170933734	
A_33_P3405995	7.1872997	6.9721518	NM_058181	chr21:47717606-47717665	YBEY
A_23_P54918	5.480509	5.5635433	NM_153486	chr16:75145886-75145827	LDHD
A_33_P3269338	8.253407	8.137802	NM_015302	chr19:36114452-36114511	HAUS5
A_23_P397455	2.3221061	2.3900566	NM_145259	chr2:158389215-158389156	ACVR1C
A_23_P217297	3.0391808	3.1899614	NM_021998	chrX:84527242-84527301	ZNF711
A_33_P3399593	5.297259	5.22327	NM_024821	chr22:42221784-42221843	CCDC134
A_33_P3273906	5.724071	6.0081553	NM_001004462	chr11:123887013-123887072	OR10G4
A_32_P95823	8.567994	9.09207	NM_014607	chr2:136541520-136541579	UBXN4
A_33_P3325665	3.9599283	3.8710983	BC109228	chr14:24038560-24038619	JPH4
A_33_P3256267	4.144351	4.5245104	XM_005251976	chr9:72333590-72333531	PTAR1
A_33_P3409815	6.9268894	7.2963967		chr4:072053346-072053405	
A_33_P3294162	5.301297	5.186304	NM_004279	chr7:102955073-102955132	PMPCB
A_23_P366726	5.348313	5.560273	NM_201520	chr17:8191215-8191156	SLC25A35
A_33_P3535175	8.501745	8.866261	BX095413	chr20:5177966-5177907	
A_23_P62371	5.1594377	5.0847545	NM_000116	chrX:153649359-153649418	TAZ
A_23_P88753	7.825731	7.7444544	NM_001001410	chr16:1400066-1399902	TSR3
A_33_P3333455	4.958637	4.321408	NM_007046	chr2:27309204-27309263	EMILIN1
A_23_P319565	7.1289716	7.1330433	NM_170753	chr10:50723568-50723509	PGBD3
A_33_P3271284	2.7317452	3.0579708	NM_001256798	chr20:31040747-31040688	C20orf112
A_33_P3341490	4.076367	4.05871	NR_033415	chr17:39782700-39782641	KRT42P
A_33_P3386156	4.0459666	4.8699656	NM_021938	chr19:3284902-3284961	CELF5
A_33_P3404844	4.1417756	4.5554094	BC033233	chr17:36708069-36708010	SRCIN1
A_23_P18317	8.024663	8.049938	NM_017836	chr3:125725329-125725270	SLC41A3
A_33_P3243649	6.213601	6.46929	AK129685	chr19:13950552-13950493	LOC100131831
A_23_P416034	4.7104797	4.91881	NM_017518	chrX:152734696-152734637	HAUS7

A_33_P3279009	10.0129385	10.218839	NM_018942	chr4:8869424-8869365	HMX1
A_23_P308581	10.872307	10.84533	NM_033112	chr6:42997172-42997231	RRP36
A_33_P3739260	6.984602	7.273	NM_006366	chr6:17557226-17557285	CAP2
A_23_P254756	9.754465	10.369637	NM_006016	chr6:109688276-109688217	CD164
A_24_P278460	7.351565	6.310251	NM_032228	chr11:13753079-13753138	FAR1
A_23_P168898	16.204042	16.214172	NM_001023	chr8:56985718-56985659	RPS20
A_23_P150768	5.209199	5.8247833	NM_007256	chr11:74917103-74917162	SLCO2B1
A_33_P3315733	8.844548	8.746979	BC046241	chr21:45480055-45480114	TRAPPC10
A_33_P3415526	12.972569	13.041775		chr1:92694629-92694570	
A_23_P26223	7.404814	7.360915	NM_001024943	chr7:65554654-65557023	ASL
A_32_P430359	10.930143	10.922192	NM_024072	chr12:113596358-113596299	DDX54
A_24_P602871	5.139589	3.8898704	NM_001030060	chr6:147890833-147890892	SAMD5
A_23_P66813	6.741733	6.53278	NM_016538	chr17:79870298-79870239	SIRT7
A_23_P80570	2.5188642	2.3900566	NM_001086	chr3:151545783-151545842	AADAC
A_33_P3330443	6.290221	6.1511784	NM_147189	chr8:59062206-59062265	FAM110B
A_32_P145867	5.474338	5.75221	NM_006188	chr7:97614191-97614132	OCM2
A_33_P3251073	6.121338	6.588115	NM_178545	chr1:1849089-1849030	TMEM52
A_33_P3381464	6.5080175	6.6574426	AK125883	chr1:1196459-1196400	
A_23_P6869	3.6484556	2.3900566	NM_007024	chr3:50395758-50395699	TMEM115
A_24_P207139	7.244908	7.156339	NM_033238	chr15:74338887-74338946	PML
A_32_P194821	13.119983	13.125466	NM_000982	chr10:029188408-029188349	RPL21
A_23_P311616	7.1360326	7.2677107	NM_015167	chr17:74717921-74716563	JMJD6
A_24_P68585	6.0582366	6.365079	NM_183001	chr1:154938644-154938501	SHC1
A_23_P412389	3.40978	4.0082555	NM_003862	chr5:170863258-170876190	FGF18
A_33_P3316061	4.4631577	4.9950895	NR_026551	chrX:15721161-15721220	CA5BP1
A_33_P3257607	6.5461807	6.7287927	NM_001197131	chr1:202509288-202509347	PPP1R12B
A_23_P171255	6.6199713	6.9020376	NM_001551	chrX:69366576-69366635	IGBP1
A_24_P250535	7.0838795	7.3175545	NM_021156	chr20:7962219-7962160	TMX4
A_33_P3289204	6.934741	7.1414194	NM_014911	chr2:69736579-69736520	AAK1
A_33_P3302075	3.116423	2.8957543	NM_019076	chr2:234681212-234681271	UGT1A8
A_33_P3251430	10.2648	10.596728	NM_003489	chr21:16337033-16336974	NRIP1
A_23_P403898	8.840908	9.366944	NM_002829	chr9:112140928-112140869	PTPN3
A_24_P47467	11.528396	11.495451	NM_024092	chr11:60690854-60690913	TMEM109
A_23_P363316	2.3221061	2.3900566	NM_002147	chr17:46668865-46668806	HOXB5
A_23_P28538	8.569899	8.70432	NM_031902	chr2:95770471-95770412	MRPS5
A_33_P3420380	4.11507	3.3895507	NM_001001851	chr10:7618659-7618600	ITIH5
A_33_P3314594	3.6638668	2.849844	NM_175738	chr17:72742734-72742793	RAB37
A_23_P167269	8.594515	8.520983	NM_018352	chr4:164441001-164441060	TMA16
A_23_P211748	8.630306	8.77256	NM_005513	chr3:120501611-120501670	GTF2E1
A_33_P3274090	5.1077957	5.1149626		chr16:067463477-067463418	
A_23_P501134	5.1298637	5.3308	NM_002311	chr17:33328391-33328955	LIG3
A_23_P151280	10.767704	10.30513	NM_032901	chr12:50514125-50514184	COX14
A_23_P325924	7.2400556	7.4369993	NM_001191033	chr2:26412310-26412369	GAREML
A_23_P345830	9.640451	9.7378845	NM_014929	chr2:207656439-207656498	FASTKD2
A_33_P3340180	4.6285396	4.7298727	NM_001190214	chr15:43865670-43865611	PPIP5K1
A_24_P934563	7.224864	7.073782	NM_014342	chr11:47639584-47639526	MTCH2
A_23_P356070	4.6074076	4.660556	NM_002020	chr5:180036955-180036045	FLT4
A_23_P5221	5.1769705	5.1357226	NM_032433	chr19:14831167-14831226	ZNF333
A_24_P276816	5.790149	6.2427726	NM_018254	chr1:211486131-211486190	RCOR3
A_23_P310911	8.439703	8.816158	NM_000386	chr17:28575739-28575680	BLMH
A_23_P351535	4.25293	4.153049	NR_024120	chr17:658041-658100	DBIL5P
A_33_P3219591	6.144235	5.8141174	NM_020954	chr17:78295199-78295258	RNF213
A_33_P3347330	9.544331	9.525609	NM_019059	chr7:22862356-22862297	TOMM7
A_33_P3265222	6.9458756	6.7125115	NM_020775	chr1:109743462-109743521	KIAA1324
A_32_P55860	7.833254	7.9278045	NM_182620	chr17:57208653-57196809	SKA2
A_33_P3269218	8.8300085	8.782466	NM_001703	chr1:32192778-32192719	BAI2
A_33_P3376154	7.4472713	7.6957912	XM_003846260	chr12:132148597-132148538	LOC100996701
A_33_P3398840	6.7910037	6.8592434	NM_001171135	chrX:2406570-2406511	ZBED1
A_33_P3297599	15.02464	15.02464	AF416714	chrX:73806737-73806796	

A_24_P21410	6.3983326	6.007695	NM_022917	chr9:33462012-33461953	NOL6
A_33_P3338116	10.303544	10.662669	NM_002292	chr3:49158643-49158584	LAMB2
A_33_P3323520	4.909121	5.0416117	NM_032424	chr11:105880616-105880557	MSANTD4
A_23_P380181	8.316091	8.371536	NM_006769	chr1:87810504-87810563	LMO4
A_24_P943106	7.2645936	6.834518	NM_001080415	chr3:142778660-142778719	U2SURP
A_23_P47691	4.753558	5.448444	NM_003141	chr11:4406568-4406509	TRIM21
A_33_P3302957	7.688364	7.261459	NM_015432	chr16:67322866-67322925	PLEKHG4
A_33_P3369760	6.726369	6.8518085	NM_001287011	chr9:36162528-36162587	GLIPR2
A_33_P3334250	4.4250164	5.1814833		chr5:38843682-38843623	
A_23_P44996	4.1184773	4.3425	NM_004057	chrX:16672600-16672659	S100G
A_33_P3225567	7.6404552	7.3044124	NM_024917	chrX:100264735-100264676	TRMT2B
A_23_P116587	4.5892935	4.730502	NM_006189	chr11:76814261-76814320	OMP
A_33_P3391135	5.1060743	5.3082786	NM_198827	chr12:131623774-131623833	GPR133
A_24_P23995	8.18557	8.152624	NM_001010858	chr1:228682366-228682425	RNF187
A_33_P3229725	9.307386	9.659721	NM_000487	chr22:51063520-51063461	ARSA
A_23_P392222	8.922142	8.705949	NM_001040011	chr9:131046829-131046888	SWI5
A_23_P164927	5.343785	5.206669	NM_012451	chr19:48879538-48879597	SYNGR4
A_33_P3277012	5.0661306	4.949994	NM_004402	chr1:3775337-3775396	DFFB
A_23_P334990	7.6909347	7.809428	NM_022491	chr12:118855142-118855201	SUDS3
A_33_P3396270	8.139353	8.3991995	NM_001011552	chr2:103150231-103150290	SLC9A4
A_23_P419624	6.1137123	6.516247	NM_006698	chr20:36146328-36146269	BLCAP
A_33_P3344291	10.921169	10.972573	XR_111092	chr10:82012926-82012985	
A_24_P416159	7.120455	7.412342	NM_005611	chr16:53524846-53524905	RBL2
A_23_P138856	12.628096	12.2921915	NM_006442	chr11:65688910-65688969	DRAP1
A_33_P3409062	5.8590055	5.9658065	NM_003332	chr19:36395371-36395312	TYROBP
A_23_P152115	10.479876	10.843325	NM_002513	chr16:1820410-1820351	NME3
A_23_P88580	8.011356	8.580446	NM_006465	chr15:74890377-74890436	ARID3B
A_33_P3406572	3.80554	3.8535075		chr20:57099757-57099816	APCDD1L-AS1
A_33_P3291569	10.8009615	10.727446	NM_016424	chr17:48829915-48829974	LUC7L3
A_23_P59772	5.2595897	5.3811665	NM_000083	chr7:143043683-143043742	CLCN1
A_33_P3340065	4.510277	4.798608		chr9:117416639-117416580	
A_33_P3394615	7.6776342	7.626584	NM_023934	chrX:154261768-154261827	FUNDC2
A_23_P7503	5.571695	5.0296793	NM_138379	chr5:156346452-156346393	TIMD4
A_23_P210829	7.192215	7.6099076	NM_018257	chr20:62907010-62907069	PCMTD2
A_33_P3209396	4.8303914	5.1800404		chr22:22015146-22015205	PPIL2
A_24_P110273	8.075748	8.044216		chr1:198166987-198166928	
A_24_P17722	10.289828	10.579517	NM_198868	chr5:179289828-179289769	TBC1D9B
A_33_P3329549	7.3748274	7.488033	NM_001105079	chr16:30681444-30681503	FBRS
A_33_P3333471	5.6831512	5.6353045	CU687439	chr8:027516462-027516403	
A_24_P404245	5.34919	5.060019	NM_002861	chr17:79862985-79862805	PCYT2
A_33_P3232955	8.00259	8.134212	NM_003950	chr19:17002771-17002830	F2RL3
A_32_P159445	10.209457	10.599971	NM_015075	chrX:53263787-53263728	IQSEC2
A_23_P391844	4.321408	4.9419937	NM_152632	chrX:36008071-36008130	CXorf22
A_23_P65240	9.0135565	10.410073	NM_001845	chr13:110802406-110802347	COL4A1
A_23_P406071	4.0901523	3.479888	AK097098	chr14:69261996-69261937	ZFP36L1
A_23_P38567	7.795604	8.113573	NM_152904	chr17:20140333-20140392	SPECC1
A_33_P3233000	8.651706	8.743439		chr1:142852728-142852669	
A_24_P318134	4.8519287	5.365452	NM_032786	chr12:56515540-56515599	ZC3H10
A_23_P25069	5.5037045	5.5726423	XM_001128260	chr12:31267800-31267369	LOC100509445
A_23_P167194	7.0747805	7.5275235	NM_001812	chr4:68359616-68358671	CENPC
A_23_P25194	2.3221061	3.5789948	NM_003806	chr12:117299112-117299053	HRK
A_33_P3250128	4.379506	4.9320188	NM_052870	chr5:53815343-53815402	SNX18
A_23_P332399	8.691659	8.858369	NM_016315	chr2:189460525-189460584	GULP1
A_33_P3422812	8.062946	8.0902405	NM_001130929	chr7:135361034-135361093	C7orf73
A_24_P114255	7.3988705	7.161092	NM_138799	chr2:8997233-8997174	MBOAT2
A_23_P145681	5.220024	5.0661306	NM_016188	chr7:100245136-100245077	ACTL6B
A_24_P286951	6.648861	7.155204	NM_024889	chr10:115542071-115542130	PLEKHS1
A_23_P108708	9.8712635	10.293218	NM_016494	chr2:85823986-85824045	RNF181
A_23_P370454	4.9660296	5.165289	NM_004732	chr17:7826089-7826030	KCNAB3

A_24_P402438	7.765564	7.255847	NM_003238	chr1:218614784-218614843	TGFB2
A_33_P3262089	4.847023	5.027346	NM_001007249	chr11:124096044-124096103	OR8G2
A_32_P226186	6.2717843	6.2302947	NM_001164665	chr7:138516363-138516304	KIAA1549
A_33_P3406240	5.1072583	5.30995	NM_182828	chr2:20871126-20871185	GDF7
A_23_P84629	4.119054	4.5002575	NM_002917	chr17:80006920-80006638	RFNG
A_33_P3350268	3.9702024	4.204215		chr9:042436430-042436371	
A_23_P2998	7.501357	7.859126	NM_005015	chr14:23240015-23240074	OXA1L
A_23_P410017	6.175616	6.2044716	NM_152715	chr11:120960072-120960131	TBCEL
A_23_P309803	8.621362	8.362812	NM_015694	chr7:149128545-149128486	ZNF777
A_24_P921897	4.884814	4.632517	NM_015888	chr1:60341230-60341289	HOOK1
A_23_P361333	4.1127496	4.0523186	NM_032137	chr3:14770143-14798884	C3orf20
A_24_P179903	11.641162	11.494258	NM_001625	chr1:33478726-33478667	AK2
A_24_P734720	5.78773	5.906025	NM_152523	chr2:208619020-208619079	CCNYL1
A_23_P72651	4.273425	2.8327036	NM_001077693	chr5:138784494-138784435	ECSCR
A_24_P360206	4.421817	4.8525186	NM_018902	chr5:140390896-140390955	PCDHA11
A_33_P3402010	4.7674174	5.294448	AK024445	chr14:71683095-71683036	
A_32_P190049	8.132209	8.084883	NM_001099678	chr3:120044380-120044323	LRRC58
A_23_P363275	8.283682	8.128782	NM_144668	chr12:122441655-122441714	WDR66
A_23_P87150	5.727153	5.170369	NM_004811	chr11:58294890-58294831	LPXN
A_24_P272845	5.821801	6.058585	NM_004947	chr3:51399313-51399372	DOCK3
A_23_P154234	10.341988	10.381052	NM_019896	chr2:75187247-75196568	POLE4
A_23_P95453	2.3221061	2.3900566	NM_004801	chr2:50147661-50147602	NRXN1
A_23_P251893	11.245769	10.907894	NM_152743	chr7:2577572-2577513	BRAT1
A_23_P140675	3.9569807	3.4496841	NM_000119	chr15:43489565-43489506	EPB42
A_24_P408801	3.8917744	3.3516402	XM_005258396	chr19:58353424-58353483	ZNF587B
A_23_P131227	9.510548	9.32565	NM_017735	chr2:33042564-33042623	TTC27
A_33_P3226099	4.2858043	4.092786	NM_178160	chr17:72929512-72929571	OTOP2
A_23_P50399	4.452506	4.0440664	NM_138353	chr19:14071142-14071201	DCAF15
A_33_P3389773	4.357027	4.702607		chr7:064295759-064295700	
A_23_P67271	3.9287295	3.8395944	NM_002741	chr19:14580209-14580268	PKN1
A_23_P300124	6.7388844	6.950424	NM_144775	chr17:18221346-18221405	SMCR8
A_23_P19691	11.163351	11.188539	NM_014320	chr6:138726355-138726414	HEBP2
A_23_P16032	6.6058674	6.987732	NR_002166	chr19:57875057-57876227	TRAPPC2P1
A_33_P3245623	4.542825	5.367937		chrY:009477884-009477825	
A_33_P3320994	5.5065866	5.645251	AL834286	chr1:85647224-85647165	SYDE2
A_24_P935103	8.992191	8.902934	NM_001116	chr16:4012826-4012767	ADCY9
A_33_P3263538	8.366433	9.100807	AF001893	chr11:65206777-65206836	
A_33_P3882264	5.0751424	5.333804	BC028005		GRASPOS
A_24_P114617	6.1159506	5.9156895	NM_014043	chr3:87303698-87303757	CHMP2B
A_23_P92642	7.39042	8.15991	NM_024668	chr5:139844274-139844333	ANKHD1
A_23_P32707	9.345906	9.538143	NM_012291	chr12:53686748-53687109	ESPL1
A_24_P88921	6.246881	6.476665	NM_004628	chr3:14187500-14187441	XPC
A_33_P3404775	12.589778	12.300437	NM_001135653	chr1:40026573-40026514	PABPC4
A_33_P3456233	6.8583007	6.8751817	XR_252672	chr9:139444136-139444195	
A_33_P3379916	8.186604	8.612051	NM_014905	chr2:191829716-191829775	GLS
A_33_P3391316	7.9739103	7.4315934	XR_109656	chrUn_gl000213:70162-70221	
A_23_P143006	5.5390196	5.4708385	NM_015893	chr2:238475717-238475776	PRLH
A_32_P208424	12.782082	12.7275095	NM_002107	chr1:226259602-226259661	H3F3A
A_33_P3400547	8.160142	7.757497	NM_004264	chr12:27182576-27182635	MED21
A_33_P3364721	3.9760876	4.168629		chr21:041102598-041102657	
A_33_P3333038	12.768322	13.060701	NM_002824	chr12:6880059-6880118	PTMS
A_24_P307065	8.792109	8.007417		chr17:074222655-074222596	
A_23_P65797	8.248489	7.892565	NM_022480	chr15:86302632-86302573	KLHL25
A_23_P162127	9.617899	9.515909	NM_021825	chr11:82972738-82972679	CCDC90B
A_24_P202581	3.9785028	3.78409	NM_199002	chr19:42409175-42409372	ARHGEF1
A_33_P3269728	5.3159947	5.884447	NM_001286721	chr13:111558458-111558399	ANKRD10
A_33_P3222380	5.6984715	5.3709574	NM_138420	chr14:105411626-105411567	AHNAK2
A_23_P68998	4.932816	4.7382298	NM_017584	chr22:50927830-50927974	MIOX
A_23_P39024	5.5205965	5.2136645	NM_032332	chr19:12784070-12784129	WDR83

A_24_P287473	8.425447	8.617647	NM_021818	chr14:51100478-51100419	SAV1
A_24_P29401	6.3932695	6.858878	NM_181523	chr5:67596786-67596845	PIK3R1
A_23_P66158	8.318684	8.257322	NM_001012991	chr16:19717835-19717776	KNOP1
A_33_P3417452	7.3518367	6.985622	NM_001103167	chr19:10415538-10415479	ZGLP1
A_33_P3285354	9.001081	9.313407	NM_001144936	chr11:63533339-63533280	C11orf95
A_33_P3220149	8.866261	8.963385	AB209135	chr5:179223213-179223272	MAML1
A_23_P354894	7.302766	7.076759	NM_152603	chr19:37211954-37212013	ZNF567
A_23_P162746	7.928439	8.071335	NM_015974	chr13:20977891-20977832	CRYL1
A_23_P366812	7.2892847	7.48013	NM_001651	chr12:50358821-50358880	AQP5
A_33_P3404950	4.9759316	4.9403667	NM_001204083	chr1:19954508-19954567	MINOS1
A_23_P98686	5.3892198	5.6175575	NM_025092	chr11:295251-295310	ATHL1
A_23_P342091	8.323862	8.589926	NM_145063	chr6:41034833-41034774	OARD1
A_24_P11462	6.6611896	7.1378727	NM_052998	chr1:33585912-33585971	ADC
A_33_P3423570	12.31344	12.440556	NM_024042	chr16:767419-767478	METRN
A_23_P25964	6.6494226	7.0336733	NM_000153	chr14:88399523-88399464	GALC
A_23_P154566	9.636728	9.498962	NM_032883	chr20:42698096-42698155	TOX2
A_24_P363548	6.7657504	6.867209	NM_005338	chr7:75164311-75164252	HIP1
A_23_P28688	9.649215	9.609713	NM_016207	chr2:9599689-9607836	CPSF3
A_24_P321411	8.222785	8.349151	NM_004705	chr11:76061610-76061551	PRKRIR
A_24_P924816	4.478689	3.401237	NM_052885	chr12:40149281-40149222	SLC2A13
A_33_P3628675	4.164521	4.0027494	NM_001083537	chr8:12040434-12040375	FAM86B1
A_24_P941759	6.2791376	6.4428096	NM_017769	chr14:31088739-31088798	G2E3
A_23_P501996	10.661453	11.114858	NM_001032288	chr20:48698274-48698215	UBE2V1
A_23_P357365	7.5911846	7.835964	NM_005862	chr3:136057233-136057082	STAG1
A_33_P3341787	3.855122	4.171334	AF050145	chrX:148584866-148584807	IDS
A_33_P3219750	5.313246	5.6053553	NM_024331	chr20:43118418-43118477	TTPAL
A_23_P154938	9.280882	9.018291	NM_003325	chr22:19318466-19318407	HIRA
A_33_P3383029	6.9291587	7.331046	NM_130439	chr10:112044747-112044806	MXI1
A_23_P31055	10.242506	10.062711	NM_198887	chr6:150045639-150045580	NUP43
A_23_P325631	6.27132	6.3213396	NM_003036	chr1:2239373-2239432	SKI
A_33_P3413905	7.0308022	5.8013544	NM_024866	chr22:50924807-50924866	ADM2
A_24_P262201	7.7251363	7.5176635	NM_001017390	chr16:29475548-29475607	SULT1A4
A_33_P3290179	11.576559	11.511668	NM_003676	chr1:224381081-224381140	DEGS1
A_23_P254852	7.968419	8.060247	NM_194248	chr2:26680157-26680098	OTOF
A_23_P169197	9.589367	10.023302	NM_032303	chr9:115233770-115233829	HSDL2
A_23_P207537	9.894249	9.793108	NM_007026	chr17:35873238-35873297	DUSP14
A_24_P89872	8.570217	8.488365	NM_197977	chr9:104172738-104172797	ZNF189
A_23_P1926	8.892633	9.120174	NM_017841	chr11:61213657-61213716	SDHAF2
A_33_P3302210	5.6281633	5.6896124		chr8:11203536-11203477	LOC100129129
A_33_P3367970	5.731712	5.647729		chr11:112054686-112054745	
A_23_P207367	5.1718807	5.000176	NM_003152	chr17:40463483-40463542	STAT5A
A_23_P48099	9.550976	9.587885	NM_024057	chr12:102471157-102470624	NUP37
A_33_P3322274	4.9363	4.992422	NM_001066	chr1:12252992-12253051	TNFRSF1B
A_33_P3383331	6.8179593	6.759767	NM_012243	chr1:100488947-100489006	SLC35A3
A_24_P235316	9.521969	9.761164	NM_005662	chr8:42260875-42260934	VDAC3
A_23_P146908	7.8239	7.845674	NM_004853	chr17:9408412-9395237	STX8
A_33_P3347971	4.3132677	4.3580875	NM_001025252	chr8:80947166-80947107	TPD52
A_33_P3220615	2.454215	2.3900566	NM_001004317	chr6:105531071-105531130	LIN28B
A_33_P3390284	4.748714	4.6762795	NM_001166600	chrX:133927905-133927846	FAM122B
A_33_P3295009	6.7613773	7.067952	AB209486	chr7:44104960-44105019	
A_32_P66974	7.1846337	7.1811476	NM_004716	chr11:117076257-117076198	PCSK7
A_23_P112251	12.371514	11.945889	NM_001017998	chr9:114432370-114432429	GNG10
A_33_P3357620	6.7212496	5.7562184	NM_024516	chr16:29833749-29833808	PAGR1
A_32_P132396	5.670467	5.578723	NR_024251	chr3:125647696-125647755	FAM86JP
A_24_P90881	4.2669325	3.949556	NM_024922	chr16:67007926-67007985	CES3
A_23_P20045	6.1578875	6.1864433	NM_000466	chr7:92119035-92118686	PEX1
A_33_P3339397	6.6574426	6.8416557	NM_001282687	chr2:218725-218666	SH3YL1
A_24_P396753	6.4937983	6.595303	NM_021643	chr2:12880797-12880856	TRIB2
A_23_P132910	7.591739	8.148366	NM_019027	chr4:40426236-40426177	RBM47

A_24_P346762	5.0057607	4.959448	NM_025182	chr9:35105138-35105079	FAM214B
A_33_P3442605	5.9966407	5.265798		chr8:23088374-23088433	LOC389641
A_33_P3225230	9.776659	9.667	NM_015260	chr19:16991075-16991134	SIN3B
A_32_P149298	4.49774	4.8573313	NM_001129993	chr2:61350543-61350602	KIAA1841
A_23_P145016	9.846103	10.298828	NM_006696	chr5:137492783-137492724	BRD8
A_33_P3402526	5.2778354	5.60605	NM_018590	chr10:43678931-43678990	CSGALNACT2
A_24_P89512	11.357716	11.093978	NM_014739	chr6:136579829-136579770	BCLAF1
A_33_P3423941	2.5020146	2.3900566	NM_003641	chr11:315054-315113	IFITM1
A_33_P3265016	4.007866	3.7718515	NM_000287	chr6:42932588-42932529	PEX6
A_24_P134392	8.849381	8.723983	NM_006948	chr21:15743635-15743576	HSPA13
A_33_P3215487	9.990233	9.906319	NM_014855	chr7:4831340-4831399	AP5Z1
A_33_P3213772	8.806389	8.845123	XM_005277514	chr1:206629446-206629505	
A_33_P3255741	5.201668	4.8086805	NM_007137	chrX:47781551-47781610	ZNF81
A_23_P55477	9.568191	9.262838	NM_000676	chr17:15878851-15878910	ADORA2B
A_23_P128734	12.192892	12.063523	NM_004450	chr14:69846976-69846917	ERH
A_33_P3281710	4.9464664	5.853474	XR_243603	chr17:19059099-19059040	
A_33_P3353372	3.8844266	4.0980496		chr7:156461704-156461646	
A_33_P3407374	7.9350224	8.114321		chr10:028966479-028966420	
A_33_P3365988	5.6786747	5.7250533	NR_046268	chr1:218517630-218517571	LOC728463
A_32_P99902	9.396	9.537712	NM_144597	chr15:83677341-83674427	C15orf40
A_32_P5480	8.989737	8.565444	NM_203463	chr2:169631003-169631062	CERS6
A_33_P3520738	2.3221061	2.3900566	NR_036584	chr19:43330971-43331030	LOC100289650
A_33_P3347392	7.2745175	7.295928	NM_138773	chr5:110097410-110097469	SLC25A46
A_32_P128310	4.1301117	4.332517	NM_017774	chr6:21000518-21000577	CDKAL1
A_33_P3296587	4.96396	5.5484333	NM_000096	chr3:148890708-148890649	CP
A_24_P148750	4.7358894	4.052773	NM_004844	chr3:15298613-15298554	SH3BP5
A_24_P215628	7.4447823	7.3914967	NM_001014283	chr13:114110456-114110397	DCUN1D2
A_33_P3271350	5.165998	4.483186	NM_003640	chr9:111629860-111629801	IKBKAP
A_33_P3405451	4.099282	3.9636765	NR_026963	chr22:28394641-28394700	TTC28-AS1
A_33_P3341509	7.4655676	7.778734		chr4:033012626-033012567	
A_33_P3251841	7.2632966	6.6728	NM_032160	chr18:65179037-65178978	DSEL
A_23_P90804	8.202842	7.980573	NM_145686	chr2:102510718-102510777	MAP4K4
A_33_P3258452	6.735289	5.909332	NM_020375	chr12:4469058-4469117	C12orf5
A_33_P3317979	4.290412	4.468006	NM_001080467	chr18:47352219-47352160	MYO5B
A_33_P3280981	2.3221061	2.3900566		chr9:043062891-043062832	
A_24_P289795	4.1298723	4.1368465		chr1:152629172-152629231	
A_23_P428248	5.2413273	5.4618015	NM_145755	chr3:39180309-39180368	TTC21A
A_23_P386905	5.71864	5.7383823	NM_025241	chr19:4446563-4446504	UBXN6
A_23_P150876	7.3217916	7.162941	NM_024667	chr12:123350139-123350080	VPS37B
A_23_P29185	7.5004487	7.623898	NM_001145418	chr22:28377786-28377727	TTC28
A_24_P226116	6.8170037	6.3690853	NM_057175	chr4:140311796-140311855	NAA15
A_33_P3291708	6.972533	6.917272		chr3:195700927-195700868	
A_24_P50458	12.386805	11.999136	NM_017489	chr8:73944301-73944362	TERF1
A_23_P502882	7.2341495	7.3153296	NM_005093	chr20:32237579-32237638	CBFA2T2
A_23_P91943	3.075159	2.3900566	NM_000882	chr3:159713231-159713290	IL12A
A_23_P58082	8.042591	8.069038	NM_199511	chr3:112324535-112324476	CCDC80
A_23_P203665	5.6762376	6.132492	NM_018367	chr11:76732830-76732889	ACER3
A_23_P5339	9.746892	9.450083	NM_030577	chr2:120439532-120439591	TMEM177
A_33_P3415843	8.258498	8.323862		chr9:131869680-131869621	
A_33_P3266828	6.3453584	6.366994	NM_016446	chr9:35847131-35847190	TMEM8B
A_23_P91430	6.6507263	6.966941	NM_007238	chr20:32295458-32295399	PXMP4
A_33_P3260654	3.4716008	2.3900566	EU030678	chr7:142499821-142499880	
A_33_P3413083	8.783614	8.327671	NM_005614	chr7:151163187-151163128	RHEB
A_24_P382017	8.051934	8.351512	NM_018427	chr16:15154499-15154440	RRN3
A_23_P315252	11.868311	11.993554	AK097322	chrM:11432-11491	ND4
A_23_P87635	8.357563	8.314002	NM_032848	chr12:113629736-113629795	C12orf52
A_33_P3319957	4.783613	4.976081	BC064144	chr1:84992226-84992285	
A_23_P80648	6.696252	7.0195403	NM_033540	chr3:179110836-179110895	MFN1
A_23_P4052	4.261452	4.3538404	AK026312	chr17:18997510-18997569	

A_23_P112162	8.132952	8.355615	NM_012079	chr8:145540294-145540235	DGAT1
A_33_P3344339	2.3221061	2.3900566	NM_172375	chr14:63174084-63174025	KCNH5
A_33_P3278293	9.452315	9.147818	NM_024086	chr17:2319454-2319395	METTL16
A_32_P5276	6.9143105	6.897473	NM_001251962	chr3:153974961-153975020	ARHGEF26
A_23_P205738	4.4376726	4.5920625	NM_138576	chr14:99636080-99636021	BCL11B
A_23_P52176	7.785181	7.598107	NM_003431	chr1:247319518-247319459	ZNF124
A_33_P3243153	9.529508	9.775006	NM_001244710	chr2:69552569-69552510	GFPT1
A_23_P111112	8.407536	8.209031	NM_020442	chr6:30894098-30894157	VARS2
A_23_P93792	6.9254966	7.1479993	NM_000535	chr7:6018228-6017331	PMS2
A_33_P3224027	4.9950895	4.9927425	NM_052897	chr12:57922251-57922310	MBD6
A_33_P3279505	4.4985447	3.9682117		chr3:126880733-126880792	
A_32_P158355	7.8333907	8.067934	NM_001015050	chr3:125650482-125649415	ALG1L
A_33_P3271301	4.953326	4.7353306	NM_181492	chr22:42575675-42575616	TCF20
A_33_P3309271	5.021658	5.1207557	NM_001199323	chr6:8062828-8062769	BLOC1S5
A_24_P316074	14.840301	14.92621		chr6:017531580-017531519	
A_33_P3288023	4.002361	4.1459556	NM_016453	chr3:48716057-48715998	NCKIPSD
A_23_P38011	5.398681	5.461274	NM_014003	chr16:72142431-72142490	DHX38
A_23_P363769	7.924945	7.8131366	NM_002284	chr12:52702840-52702897	KRT86
A_33_P3360296	4.03525	3.886223	NM_001278688	chr10:47701112-47701171	ANTXRL
A_33_P3269631	4.0857477	2.3900566	BQ890675	chr3:174269090-174269149	NAALADL2
A_32_P129968	6.6578097	6.741733	CR936662	chr19:44593675-44593734	ZNF284
A_23_P96285	4.56765	4.256562	NM_022912	chr2:86441557-86441498	REEP1
A_33_P3402103	5.433359	5.214275	NM_001199012	chr1:24718151-24718092	STPG1
A_33_P3327921	4.4594703	4.0975	NM_012249	chr2:46811768-46811827	RHOQ
A_33_P3231432	13.322228	13.480921	NM_001174097	chr12:21791364-21791305	LDHB
A_23_P55091	9.661405	9.571092	NM_017647	chr17:61897259-61897200	FTSJ3
A_23_P251717	7.6221232	7.8113656	NM_007075	chrX:48932825-48932543	WDR45
A_24_P26177	4.322843	4.406177	NM_014703	chr3:51450745-51450471	VPRBP
A_24_P305960	10.3624525	10.246744	NM_181336	chr6:33739247-33739188	LEMD2
A_24_P241318	9.1674185	8.444273	NM_181340	chr14:73426110-73426169	DCAF4
A_24_P409985	5.866286	5.8261375	NM_001011655	chr3:194346714-194346655	TMEM44
A_33_P3387911	9.541637	9.590746		chr14:057509078-057509137	
A_23_P156209	8.377911	8.423021	NM_033414	chr5:16458670-16453251	ZNF622
A_33_P3302791	5.625786	6.014085	XR_242176	chr7:46727536-46727477	LOC730338
A_33_P3413795	9.68362	9.907475	AV727532	chr1:120905128-120905069	
A_33_P3292915	4.5266914	4.6959043	NM_001008657	chr5:149758545-149758604	TCOF1
A_33_P3328619	15.709674	15.835667		chr1:063075696-063075637	
A_24_P417606	14.566067	14.453099		chr1:237144782-237144841	
A_33_P3360540	12.022421	11.915518	NM_006412	chr9:139567678-139567619	AGPAT2
A_33_P3300361	4.018106	4.502862	AK095841	chr15:48769807-48769748	LOC100129397
A_23_P60718	4.8617477	4.6966443	NM_024105	chr22:50297474-50297415	ALG12
A_32_P45493	10.526943	10.308756	NM_006625	chr1_gl000191_random:36433-36374	SRSF10
A_23_P304386	8.84668	8.989737	NM_138820	chr5:175816491-175816550	HIGD2A
A_33_P3326447	4.7021	5.209718	AK125272	chr12:129328117-129328058	LOC100128276
A_24_P222872	2.3221061	3.7764225	NM_001072	chr2:234676521-234676580	UGT1A6
A_23_P114903	4.0139837	4.076367	NM_002155	chr1:161496158-161496217	HSPA6
A_32_P19840	8.939971	8.755114	XM_005276008	chr7:143076846-143076787	LOC100507507
A_23_P57347	8.092286	8.318684	NM_006031	chr21:47865390-47865449	PCNT
A_24_P88850	7.497409	7.712366	NM_012219	chr3:138124161-138124220	MRAS
A_33_P3213747	6.763817	6.635542	NM_014205	chr11:64884131-64884072	ZNHIT2
A_23_P393645	6.169017	5.6953545	NM_139025	chr9:136324399-136324458	ADAMTS13
A_32_P123255	6.122833	5.9034076	NM_001105576	chrX:118894043-118894102	SOWAHD
A_33_P3271885	3.8773804	4.33457		chrY:019671261-019671202	
A_23_P6099	4.067522	3.6105185	NM_015192	chr20:8755212-8755271	PLCB1
A_33_P3401841	6.6458836	6.661681	AK123920	chr18:77515182-77515241	LOC100130453
A_23_P118643	11.636479	11.693324	NM_001968	chr4:99801580-99801521	EIF4E
A_33_P3257140	5.967511	5.7213173	NM_004850	chr2:11323535-11323476	ROCK2
A_33_P3300965	2.6803522	2.3900566	NM_004503	chr12:54424535-54424594	HOXC6

A_23_P106194	4.513371	3.410367	NM_005252	chr14:75748214-75748273	FOS
A_33_P3388135	9.434869	9.743678	NM_018848	chr20:10401254-10401195	MKKS
A_33_P3380417	9.04056	8.439703	NM_001286807	chr13:45967756-45967697	SLC25A30
A_33_P3290792	4.779035	4.6667156	NM_001001953	chr11:123894447-123894506	OR10G9
A_24_P8768	10.520898	10.373048	NM_017971	chr1:1341252-1341193	MRPL20
A_24_P20383	6.3434033	6.288557	NM_005718	chr3:9845571-9845630	ARPC4
A_33_P3259183	5.174006	4.587043	NM_001017961	chr1:166039318-166039259	FAM78B
A_24_P313186	7.5042887	7.6095476	NM_006888	chr14:90873959-90874018	CALM1
A_24_P36299	5.9213934	5.7792625	NM_004491	chr19:47502621-47503601	ARHGAP35
A_33_P3390024	5.228359	5.766877	NM_001282314	chr17:74097243-74097184	EXOC7
A_24_P244470	4.3631735	4.1890616	NM_194358	chr12:56598774-56598715	RNF41
A_23_P87616	12.428605	12.557169	NM_001002031	chr12:54059136-54059077	ATP5G2
A_33_P3335386	10.222691	9.68004	DJ031151	chr17:18872161-18872102	FAM83G
A_33_P3351388	6.476227	6.606178		chr17:055940464-055940523	
A_33_P3316064	5.5556493	5.1532884	XR_110931	chrX:15715524-15715583	CA5BP1
A_33_P3416321	6.968599	7.3616414	NM_012434	chr6:74303341-74303282	SLC17A5
A_23_P148916	9.662343	10.138359	NM_148909	chr1:52253537-52253596	OSBPL9
A_23_P329112	4.5971894	4.3555527	NM_000215	chr19:17937339-17937280	JAK3
A_23_P18422	10.92543	10.547763	NM_007208	chr3:131181203-131181144	MRPL3
A_23_P207280	7.8892965	7.630849	NM_006380	chr17:58520683-58520624	APPBP2
A_23_P329956	6.898076	6.705792	NM_139073	chr2:231865241-231867446	SPATA3
A_33_P3329508	6.326145	6.165349	NM_001143757	chr10:134191347-134191406	LRRC27
A_32_P224850	8.258059	8.480531	XR_249194	chr2:120480892-120480833	LOC101927764
A_33_P3262890	13.259013	13.467536		chr6:028700246-028700305	
A_24_P350576	5.862552	6.689749	NM_015028	chr3:170780412-170780353	TNIK
A_33_P3854030	6.236537	6.4362135	NM_018328	chr2:149267644-149267703	MBD5
A_33_P3410925	5.964403	6.0452843	NM_006563	chr19:12995295-12995236	KLF1
A_24_P103922	7.8255835	7.936174	NM_001329	chr10:126681883-126681824	CTBP2
A_33_P3321697	2.3221061	3.9941556	NM_022140	chr5:111498374-111498315	EPB41L4A
A_23_P55936	11.078617	11.426882	NM_004107	chr19:50029511-50029570	FCGRT
A_23_P211840	7.6892924	7.957505	NM_003968	chr3:69105189-69105036	UBA3
A_33_P3229256	11.688069	11.587711	NM_014595	chr17:73126383-73126324	NT5C
A_23_P167983	7.177294	8.3071785	XM_005249437	chr6:26138822-26138881	HIST1H2AC
A_23_P341532	7.442387	7.212036	NM_031915	chr13:50065911-50065970	SETDB2
A_23_P99906	7.3520374	7.450982	NM_199330	chr15:83518202-83518143	HOMER2
A_33_P3331426	7.5775023	7.0787315		chr2:91910371-91910312	FLJ37786
A_23_P92786	7.9660287	7.66433	NM_018989	chr5:145664280-145665553	RBM27
A_24_P411863	5.7032747	6.021137	NM_182931	chr7:104753597-104753656	KMT2E
A_33_P3258923	2.3221061	2.3900566	NM_005664	chr15:23812919-23812978	MKRN3
A_23_P1029	6.0992336	5.9223757	NM_017459	chr1:17302197-17302138	MFAP2
A_33_P3299421	3.7206414	2.3900566	NM_020880	chr19:58119575-58119634	ZNF530
A_23_P425066	4.88456	4.3767314	NM_000496	chr22:25625428-25625487	CRYBB2
A_23_P430161	7.216741	7.276208	NM_175876	chr1:231469145-231469086	EXOC8
A_23_P368996	5.560273	5.049528	NM_198075	chr11:554830-554889	LRRC56
A_33_P3755633	6.1064982	6.2811785	DA515661	chr17:38183469-38183410	
A_23_P77908	7.816707	6.681672	NM_152908	chr17:19581690-19581631	SLC47A2
A_33_P3263157	10.27731	10.73748		chr7:042153148-042153207	
A_23_P20532	4.484955	3.8830042	AK024257	chr9:43821272-43821331	
A_33_P3558740	4.6367464	3.7141597	AK057616	chr11:65336271-65336212	SSSCA1-AS1
A_33_P3297525	4.223155	4.4318576		chr1:143403454-143403513	
A_24_P338788	8.777135	8.772888	NM_145203	chr13:37678028-37677969	CSNK1A1L
A_23_P370924	10.25834	10.129342	NM_001124767	chr3:52574485-52574544	SMIM4
A_24_P148450	8.42205	8.657967	NM_006357	chr2:181922488-181922547	UBE2E3
A_33_P3268234	6.0806017	6.213171	NM_213656	chr17:39114740-39114681	KRT39
A_33_P3209491	7.7580547	7.5439587	NM_022648	chr2:218664920-218664861	TNS1
A_23_P77228	5.8475103	5.964692	NM_022769	chr15:91187847-91187906	CRTC3
A_23_P134814	7.583662	7.2723584	NM_018105	chr8:42692056-42691997	THAP1
A_33_P3602006	13.013338	12.815836	NM_001286259	chr6:143748303-143748244	ADAT2
A_24_P133017	4.6992245	4.473373	NM_032780	chr11:118404580-118404780	TMEM25

A_24_P922252	4.2744513	4.5372014	AK022348	chr15:45696608-45696667	SPATA5L1
A_33_P3238920	5.437442	5.687932	NM_001243195	chr10:121551397-121551456	INPP5F
A_33_P3312509	4.118147	4.3207	NM_014694	chr9:136440581-136440640	ADAMTSL2
A_23_P66872	8.340187	8.3936615	NM_015721	chr17:648204-648145	GEMIN4
A_33_P3318122	7.292723	7.2374043	NM_020314	chr16:19712383-19712442	C16orf62
A_23_P31536	12.319452	12.070162	NM_003143	chr7:141445323-141445382	SSBP1
A_23_P108303	11.720884	11.783738	NM_005001	chr19:8376334-8376275	NDUFA7
A_33_P3355418	3.772071	4.4783087	NM_152336	chr15:87217546-87217605	AGBL1
A_33_P3268464	4.537299	4.3318396	AK027742	chr17:7491646-7491705	MPDU1
A_24_P18146	7.895301	7.962717	NM_015310	chr8:18388829-18388770	PSD3
A_33_P3377194	8.028448	8.178531	NM_033304	chr8:26721671-26721612	ADRA1A
A_24_P201739	8.955202	9.049988	NM_005475	chr12:111888993-111889052	SH2B3
A_23_P79842	10.608243	10.594404	NM_015937	chr20:44054702-44054761	PIGT
A_33_P3248765	6.2457557	6.478094	NM_015999	chr1:202915627-202915568	ADIPOR1
A_33_P3317825	7.3831973	7.5915465	NM_002524	chr1:115251224-115251165	NRAS
A_24_P212024	3.6606207	2.3900566	AY867113	chr2:114164302-114164361	
A_33_P3402354	3.7706814	3.1587863	L40403	chr14:75287841-75287900	YLPM1
A_24_P324814	7.3271914	7.4052057	NM_138363	chr17:62529021-62529080	CEP95
A_23_P25974	9.979653	8.797011	NM_001010854	chr14:91007027-91006968	TTC7B
A_33_P3304427	3.320714	2.3900566	NM_001190787	chr5:54515713-54515654	MCIDAS
A_33_P3304754	4.7575493	4.60716	BC024905	chr13:103260264-103260323	TPP2
A_23_P214876	7.2313833	7.4785137	NM_004973	chr6:15521805-15521864	JARID2
A_24_P7594	4.5076966	3.5827518	NM_030641	chr22:36063762-36063821	APOL6
A_33_P3397755	6.859538	7.007976	NM_001006618	chr9:128347982-128347923	MAPKAP1
A_33_P3332616	5.383994	5.408061		chrX:47664518-47664577	XLOC_014512
A_33_P3775281	7.3913326	7.259304	NR_104176	chr19:36279837-36279778	LOC644050
A_33_P3412613	6.8273478	6.8426895	NM_001032283	chr12:98942332-98942391	TMPO
A_32_P23795	11.063074	11.404576		chr4:159201331-159201390	XLOC_014512
A_32_P23624	7.9358225	7.8898096	NM_012089	chr1:229652984-229652925	ABCB10
A_33_P3291732	3.07713	3.4020755	XM_005260156	chr19:17517045-17517104	
A_24_P8454	4.541666	4.65304	NM_001130528	chr17:49067859-49067223	SPAG9
A_23_P90612	10.986447	10.829411	NM_005915	chr2:136598276-136598217	MCM6
A_23_P369666	6.3340693	6.501586	AL137703	chr20:45866354-45866295	ZMYND8
A_33_P3416206	4.528322	4.9944134	AF258580	chr16:24931883-24931824	ARHGAP17
A_23_P64567	4.0546455	4.9299164	NM_016147	chr11:73965000-73965059	PPME1
A_33_P3305320	7.3823924	7.6556625		chrY:026423977-026423918	
A_23_P54170	5.5236974	5.7984986	NM_005466	chr14:71060050-71059684	MED6
A_33_P3214785	6.4016433	6.0040007	AK094801	chr1:65888975-65889034	LEPROT
A_24_P101304	4.4307094	4.865967	NM_198539	chr19:37441087-37441146	ZNF568
A_23_P97606	5.6700006	5.8865786	NM_000851	chr1:110260419-110260478	GSTM5
A_32_P12327	5.055211	5.6049213	XR_110056	chr4:53527427-53527486	USP46-AS1
A_24_P605563	4.9096823	4.953326	AY172962	chr22:23243286-23243345	
A_33_P3352958	4.7073298	4.8338327	NM_001164638	chr17:78398840-78398899	ENDOV
A_23_P211785	6.18722	6.32574	NM_003420	chr3:44701889-44701948	ZNF35
A_23_P132285	10.82207	10.879364	NM_001130517	chr22:37425669-37425728	MPST
A_23_P12866	6.607434	6.2674503	NM_153336	chr10:124745844-124746396	PSTK
A_24_P349207	5.690773	5.853998	NR_073404	chr5:68931817-68931876	LOC441081
A_33_P3408054	12.577812	12.42138	AY956763	chr4:13339014-13339073	
A_23_P384532	5.004822	4.8121057	NM_145020	chr18:47777299-47777240	CCDC11
A_24_P276385	4.7502913	4.2477813	NM_205856	chrX:47986665-47990023	SPACA5
A_33_P3346098	6.952334	7.2140336	NM_016151	chr16:29999515-29999574	TAOK2
A_33_P3370132	8.848029	8.881175	NM_012470	chr7:128607418-128607359	TNPO3
A_33_P3408711	6.3719215	6.2685533	NM_001456	chrX:153589903-153589844	FLNA
A_23_P215198	8.923636	9.02583	NM_018264	chr7:66704211-66704270	TYW1
A_33_P3382267	4.2914405	4.510277	NM_014866	chr9:139336239-139336180	SEC16A
A_24_P753161	7.3639693	6.4953856	NM_001204	chr2:203431522-203431581	BMPR2
A_32_P218332	7.294896	7.1429796	NM_174917	chr16:89220744-89220803	ACSF3
A_23_P68155	5.9405193	6.2283363	NM_022168	chr2:163128865-163128806	IFIH1
A_33_P3376651	4.0849824	3.5785632	CR740889	chr17:48475190-48475249	

A_33_P3400258	4.1607265	3.7881026	NM_207321	chr10:96979668-96979727	C10orf129
A_24_P359191	5.9604897	6.2317057	NM_003043	chr3:14487331-14489120	SLC6A6
A_33_P3312638	2.806517	2.3900566	NM_001005851	chr19:40540162-40540103	ZNF780B
A_23_P393758	6.066251	5.720338	NM_138401	chr19:17534589-17534854	MVB12A
A_33_P3271051	9.2442	8.908225	NM_004227	chr7:6201473-6201414	CYTH3
A_33_P3265104	4.8692822	4.175599	NM_001242307	chr10:3190239-3190180	PITRM1
A_33_P3212700	5.425542	5.0779324		chr19:058153052-058152993	
A_23_P137715	7.914177	7.915614	NM_017542	chr1:166823328-166823387	POGK
A_33_P3385765	12.592546	12.73136	NM_001012753	chr19:12091030-12091089	ZNF763
A_33_P3305998	4.7646685	4.310104		chr6:74079726-74079785	
A_23_P161352	7.8488703	8.000462	NM_014241	chr10:17645634-17645575	PTPLA
A_24_P925062	7.3175545	7.4014635	NM_001008528	chr17:74672497-74672438	MXRA7
A_23_P61823	8.229789	8.609471	NM_001031677	chr5:176728758-176728699	RAB24
A_23_P128919	9.506384	9.576338	NM_002306	chr14:55609454-55611879	LGALS3
A_24_P84970	7.7272463	7.289969		chr5:108924995-108925054	
A_33_P3363012	12.42138	12.028608	NM_014502	chr11:60658279-60658220	PRPF19
A_32_P224149	9.349419	9.370164	NM_015258	chr9:115928162-115928103	FKBP15
A_33_P3397486	7.855182	7.765369	NM_004901	chr8:23290353-23290294	ENTPD4
A_33_P3327208	6.1952257	6.521847		chr21:015211610-015211669	
A_33_P3329922	2.5686812	2.3900566	NR_103829	chr10:42905003-42904944	CCNYL2
A_23_P40866	8.840236	8.513567	NM_015642	chr3:114057749-114057690	ZBTB20
A_23_P65651	8.750048	8.240244	NM_004184	chr14:100801100-100801041	WARS
A_24_P64918	6.7435813	6.9982204	NM_014950	chr14:64990003-64990062	ZBTB1
A_33_P3339036	9.410248	9.390914	NM_001110792	chrX:153295787-153295728	MECP2
A_33_P3366484	4.9846764	5.3110394	NM_198047	chr2:191161659-191161600	HIBCH
A_23_P29594	2.3221061	2.3900566	NM_052969	chr3:186845840-186839111	RPL39L
A_33_P3327165	7.7085047	7.724441	NM_206886	chr1:93730275-93730334	CCDC18
A_23_P145657	7.795208	7.7998257	NM_012447	chr7:99811912-99811971	STAG3
A_33_P3246244	5.158902	5.271322		chr16:003500086-003500145	
A_33_P3312301	4.6729326	5.220851	NM_001206999	chr12:120125704-120125645	CIT
A_33_P3265549	4.7851133	5.3807898	NM_001205206	chr21:46945810-46945751	SLC19A1
A_33_P3225268	5.9366884	5.9755073	NM_001004128	chr1:180165656-180165715	QSOX1
A_23_P215435	3.83845	3.2980938	NM_001257190	chr7:72421126-72421185	POM121
A_23_P77993	6.453749	6.5562973	NM_006688	chr17:43037633-43037574	C1QL1
A_33_P3334419	2.5349479	3.825457	NM_031464	chr14:75388835-75388776	RPS6KL1
A_24_P7934	13.966563	13.954257		chr5:017655347-017655288	
A_33_P3420254	6.5633907	6.8957767	NM_017802	chr7:819721-819780	HEATR2
A_24_P290373	5.103758	4.890281	NM_022101	chrX:118672116-118672112	CXorf56
A_33_P3769406	6.278035	6.237657	NM_080476	chr20:33173301-33173242	PIGU
A_33_P3318148	4.3265123	4.3015985	NM_153647	chr14:92922821-92922880	SLC24A4
A_24_P240065	8.495739	8.889372	NM_016079	chr2:86737514-86734691	CHMP3
A_33_P3330264	7.130242	7.003983	NM_001511	chr4:74735646-74735705	CXCL1
A_23_P81212	10.765096	10.503475	NM_016067	chr4:84382182-84382329	MRPS18C
A_33_P3211864	4.504409	2.3900566	NR_028407	chrX:100788387-100788446	ARMCX4
A_23_P134085	9.733805	10.255213	NM_173515	chr6:154726608-154726549	CNKSR3
A_33_P3246580	2.3221061	2.3900566	NM_020340	chr6:138657434-138657493	KIAA1244
A_23_P61180	8.472536	8.618832	NM_018390	chrX:219727-219786	PLCXD1
A_33_P3323928	4.820693	3.2011797		chr10:102133690-102133749	LINC00263
A_23_P104705	4.213416	4.3892045	NM_001532	chr11:66131783-66131724	SLC29A2
A_23_P316150	8.109624	8.316091	NM_020800	chr3:159975086-159975027	IFT80
A_33_P3294217	8.487146	8.366433	NM_003577	chr10:135045003-135045062	UTF1
A_33_P3415957	11.601384	11.367223	NM_004492	chr15:59931124-59931065	GTF2A2
A_33_P3269388	7.451602	6.9901743	NM_001146082	chr19:54682141-54682082	MBOAT7
A_33_P3799692	7.806467	6.140853	BC043009	chr10:33176365-33176306	LOC338620
A_33_P3236177	5.3738594	5.745919	NM_001145	chr14:21162109-21162168	ANG
A_24_P411749	4.666358	5.159935	NM_020455	chr6:142766903-142766962	GPR126
A_33_P3228757	6.1102905	6.1061163	NM_001144989	chr19:58384249-58384190	ZNF814
A_33_P3226070	4.3477254	4.931653		chr4:1548896-1548955	LOC101928558
A_23_P213620	4.5004945	5.1429176	NM_001271948	chr5:145969231-145969172	PPP2R2B

A_23_P203829	5.889778	6.0153522	NM_015394	chr12:133733308-133733367	ZNF10
A_33_P3216487	6.2181005	6.366758		chr11:111329097-111329156	
A_33_P3283848	5.50639	5.5233126	BX646214	chr9:42494615-42494674	
A_23_P137909	13.907053	13.835765	NM_003493	chr1:228612705-228612646	HIST3H3
A_33_P3251470	6.1631536	6.418319		chr16:48672571-48672630	
A_33_P3251821	5.420381	5.351407	NR_003545	chr5:64926285-64926344	TRAPPC13
A_23_P148204	8.638212	8.861006	NM_017944	chr11:11976688-11977582	USP47
A_23_P33022	14.755845	14.88216	NM_021128	chr11:840281-840222	POLR2L
A_33_P3346132	6.940838	7.068443	NM_017987	chr10:70103386-70103327	RUFY2
A_23_P24884	7.2064223	7.5061064	NM_005418	chr11:8715180-8715121	ST5
A_33_P3271323	10.361845	10.256865	NM_012249	chr2:46810064-46810123	RHOQ
A_23_P65870	8.597627	8.79506	NM_012170	chr15:76222250-76222309	FBXO22
A_23_P124988	5.528408	5.4183226	NM_012285	chr17:40311776-40309105	KCNH4
A_33_P3366903	9.7221	10.051027	NM_021615	chr16:75512463-75512404	CHST6
A_23_P205228	5.858223	6.294172	NM_000053	chr13:52507094-52507035	ATP7B
A_33_P3255165	6.067524	5.9907885	XR_110433	chr10:90693582-90693641	ACTA2-AS1
A_24_P124662	9.203596	9.232655	NM_139078	chr12:112330780-112330839	MAPKAPK5
A_33_P3352148	4.3340573	4.2189417	NM_001122772	chr12:58119125-58119066	AGAP2
A_23_P216556	8.138996	7.667664	NM_018424	chr9:112002981-112002922	EPB41L4B
A_23_P367420	3.9331138	3.9316297	NM_003813	chr14:70926263-70926322	ADAM21
A_23_P38505	4.2178783	4.5109634	NM_022059	chr17:4637619-4637560	CXCL16
A_32_P213091	7.3885417	6.1255116	NM_001145205	chr16:13005162-13005221	SHISA9
A_33_P3265024	3.7529037	2.3900566	NM_005418	chr11:8752010-8751951	ST5
A_24_P126691	13.73581	14.016186		chr15:53229076-53229050	XLOC_014512
A_33_P3498613	3.8760998	3.6551075		chrX:102906747-102906688	XLOC_014512
A_23_P90407	3.9925227	2.3900566	NM_012114	chr19:15164420-15164580	CASP14
A_24_P686965	9.553627	8.941634	NM_001103161	chr1:21046321-21046262	SH2D5
A_23_P106844	15.199939	15.1751585	NM_005953	chr16:56643332-56643391	MT2A
A_24_P100277	5.226857	5.011899	NM_007326	chr22:43026905-43024246	CYB5R3
A_33_P3210671	3.8068323	2.3900566	NM_207328	chr2:96690381-96690322	GPAT2
A_33_P3381183	4.215723	3.97577	NR_021485	chr3:168237350-168237409	EGFEM1P
A_23_P142671	4.7071843	4.942854	NM_144631	chr2:27600753-27600694	ZNF513
A_33_P3281465	7.9046955	8.1086855	NM_021095	chr2:27423946-27423887	SLC5A6
A_23_P1387	6.778849	6.4246907	NM_032900	chr10:98982785-98982726	ARHGAP19
A_33_P3351991	3.915629	4.9982147	AK127505	chr16:2567403-2567462	
A_33_P3288569	7.509563	7.902895	NM_001144891	chr14:36780839-36780780	MBIP
A_23_P88691	5.6244025	5.23094	NM_000745	chr15:78885456-78885515	CHRNA5
A_24_P102053	4.5123053	4.8322005	NM_002538	chr5:68847386-68849429	OCLN
A_33_P3210288	16.507711	16.487724	NM_000150	chr19:5830801-5830742	FUT6
A_23_P89163	9.884188	9.9643135	NM_001013839	chr17:74077362-74077303	EXOC7
A_33_P3290403	4.98483	5.5531225	NM_014214	chr18:11999126-11999185	IMPA2
A_23_P106371	5.75221	6.169977	NM_001038640	chr15:74362901-74362842	GOLGA6A
A_23_P375	11.211504	11.30575	NM_018101	chr1:38175149-38175208	CDCA8
A_33_P3300843	7.72425	7.8930626	XR_242365	chr7:56886942-56886883	
A_24_P257359	12.778569	12.7516775	NM_002948	chr3:23960025-23960703	RPL15
A_33_P3424257	12.342196	10.9461355	NM_025146	chr3:113437999-113437940	NAA50
A_33_P3245348	4.3886995	4.6479607	NM_033060	chr17:39341099-39341040	KRTAP4-1
A_23_P147918	11.11167	11.1904335	NM_080388	chr1:153579734-153579675	S100A16
A_33_P3386459	3.3586793	4.2490773	NM_001030055	chr14:32586421-32586480	ARHGAP5
A_33_P3326020	4.9366612	5.1518397	XM_005272466	chr19:8388341-8388282	KANK3
A_23_P163820	6.1095476	5.923638	NM_001080417	chr16:30790245-30790186	ZNF629
A_33_P3209522	13.078306	12.941564	NM_144975	chr17:33594514-33594573	SLFN5
A_23_P142154	9.28005	9.167079	NM_031485	chr19:48956953-48957012	GRWD1
A_23_P50946	3.9801226	4.111436	NM_005855	chr2:238820661-238820720	RAMP1
A_33_P3269359	4.17274	3.704312	NM_139015	chr12:121201342-121201283	SPPL3
A_23_P13797	4.786661	4.2669325	NM_024738	chr12:117153885-117153826	C12orf49
A_23_P55779	10.378933	10.315827	NM_000766	chr19:41601734-41601793	CYP2A13
A_33_P3376478	5.9984016	5.4976926	NM_000102	chr10:104590371-104590312	CYP17A1
A_33_P3728167	5.8955994	6.042195	NM_001795	chr16:66437356-66437415	CDH5

A_33_P3327687	10.100403	10.280151	XR_244229	chr20:1946336-1946395	
A_23_P64792	5.496687	4.993904	NM_014505	chr12:70824623-70824682	KCNMB4
A_33_P3373892	6.3877015	6.011687	AK056059	chr9:88601845-88601904	NAA35
A_33_P3327673	3.8957617	2.3900566	NM_015198	chr7:51152998-51152939	COBL
A_33_P3364520	5.0999928	4.9232044	NM_181602	chr21:31986202-31986143	KRTAP6-1
A_33_P3209404	4.5673327	3.7245502		chr17:66130473-66130414	XLOC_014512
A_23_P326296	5.2633934	4.948641	NM_144987	chr19:36235296-36235237	U2AF1L4
A_23_P97296	10.281297	10.480377	NM_018090	chr1:16786370-16786429	NECAP2
A_33_P3410700	14.091058	14.140438	AK128101	chrM:8449-8508	ATP8
A_24_P7143	6.120763	5.967016	NM_012223	chr2:192160894-192194661	MYO1B
A_23_P168556	9.279421	8.493911	NM_004603	chr7:73113602-73113543	STX1A
A_33_P3276927	7.680694	7.8886166		chr21:018889389-018889330	
A_23_P17316	5.6776047	5.9068985	NM_152864	chr20:61873965-61873906	NKAIN4
A_33_P3287113	5.2749243	4.8238697	XM_005272292	chr4:1374459-1374518	UVSSA
A_33_P3318187	4.9592066	5.1213017	NM_145039	chr16:90036576-90036517	CENPBD1
A_33_P3217689	6.6064177	5.8120737	NM_152405	chr5:78622942-78623001	JMY
A_33_P3413025	5.390748	5.338452	D13075	chr14:22616489-22616548	
A_33_P3240263	7.4912276	7.525961	NM_015932	chr13:29252779-29252838	POMP
A_23_P309198	4.8803	4.879803	NR_027128	chr21:46353414-46353355	C21orf67
A_23_P133582	9.169774	9.030766	NM_004730	chr5:137842459-137842400	ETF1
A_24_P61864	6.9183226	7.6005774	NM_020198	chr17:61833873-61833696	CCDC47
A_24_P125434	6.6639385	6.5587997	NM_032243	chr18:9887992-9888051	TXNDC2
A_23_P19455	8.779782	8.646215	NM_014641	chr6:30667792-30667733	MDC1
A_33_P3296577	7.5595145	7.3806868	NM_013318	chr9:134371651-134371710	PRRC2B
A_23_P86330	10.318715	9.881194	NM_016545	chr1:181059835-181059894	IER5
A_23_P53668	7.819146	7.3945017	NM_006166	chr12:104511944-104511885	NFYB
A_33_P3299781	5.7055063	5.5567527		chr20:3172304-3172245	DDRGK1
A_24_P150466	5.442589	6.02244	NM_001034852	chr14:70498782-70498841	SMOC1
A_33_P3239405	5.928468	5.922137	NM_016456	chr1:201120949-201120890	TMEM9
A_24_P639665	6.0886364	6.539602	XR_242529	chr9:8861154-8861213	
A_33_P3233165	2.9356346	3.999261	XM_005264120	chr2:55766620-55766679	CCDC104
A_33_P3309429	6.4547043	6.764489		chr7:23234444-23234503	NUPL2
A_32_P230196	9.659414	9.455775	NM_001077685	chr10:51464344-51464285	AGAP7
A_33_P3271111	11.799826	11.931005	NM_004148	chr9:95883839-95883780	NINJ1
A_23_P86855	5.9772577	5.705166	NM_014067	chr11:63918797-63918738	MACROD1
A_23_P152028	8.72152	8.589294	NM_016213	chr15:64747270-64747329	TRIP4
A_33_P3403952	3.3085964	4.2842245	BC066931	chr14:99877720-99877661	SETD3
A_23_P47497	11.101643	11.1348	NM_017840	chr11:59573799-59573740	MRPL16
A_23_P70843	7.810819	7.5328856	NM_199186	chr7:134364406-134364465	BPGM
A_33_P3364904	2.3221061	2.3900566	NM_207404	chr3:42958868-42958927	ZNF662
A_33_P3303372	10.084971	10.07687	NM_001184792	chr10:34620141-34620082	PARD3
A_23_P129174	5.849881	5.533292	NM_017691	chr15:71342217-71342276	LRRC49
A_32_P516342	6.50097	6.6494226	NM_182608	chr12:52284985-52285044	ANKRD33
A_24_P370670	8.152624	8.344757	NM_001195156	chr1:35449497-35449438	ZMYM6NB
A_33_P3389018	7.4729104	7.3748274	NM_001001674	chr15:102359143-102359202	OR4F15
A_33_P3209894	6.0055604	5.5352955		chr13:19956734-19956793	XLOC_014512
A_23_P201376	3.109996	2.3900566	NM_014021	chr1:85109841-85109782	SSX2IP
A_23_P81993	9.86725	9.707296	NM_178508	chr6:34214259-34214200	C6orf1
A_33_P3240094	4.5154657	3.9321976	BC073962	chr2:233759387-233759328	NGEF
A_23_P12911	9.908209	9.96834	NM_022338	chr11:68029070-68029011	C11orf24
A_32_P109522	7.546673	7.2620134	NM_145062	chr6:116966908-116957015	ZUFSP
A_33_P3508717	6.322444	5.273427	NR_027183	chr5:180262629-180262688	LINC00847
A_33_P3272189	7.684237	7.675664	NM_032718	chr2:103333845-103333786	MFSD9
A_33_P3278347	8.580074	8.676715	NR_026542	chr21:40969138-40969079	C21orf88
A_33_P3337161	5.69911	5.3875523	NM_001198568	chr14:24793331-24793272	ADCY4
A_33_P3383054	3.7973807	3.698854		chr1:182033758-182033699	
A_33_P3392525	8.386516	8.086163	NM_001661	chr17:41478441-41478500	ARL4D
A_23_P27315	7.8267827	7.235528	NM_032048	chr18:2913979-2914038	EMILIN2
A_33_P3321611	5.5501776	5.5547895	NM_001048183	chr1:28792957-28793016	PHACTR4

A_33_P3353581	7.9124207	7.9203825	NM_001551	chrX:69370070-69370129	IGBP1
A_24_P147169	4.55462	5.1211843	NM_213600	chr15:42434356-42434297	PLA2G4F
A_33_P3327772	6.625829	6.648364	NM_000052	chrX:77302448-77302507	ATP7A
A_33_P3328666	8.675965	8.626977	NM_007097	chr5:175819515-175819456	CLTB
A_24_P403417	2.3221061	2.3900566	NM_004878	chr9:132501569-132501510	PTGES
A_33_P3219454	12.698034	12.442451		chr21:047488450-047488509	
A_32_P538017	5.15664	4.8784823	NM_005995	chr11:67398871-67398812	TBX10
A_33_P3314212	5.002277	4.812732	NM_003631	chr10:51363266-51363207	PARG
A_32_P10272	6.6514034	6.8377895	NR_027775	chr7:74312537-74312596	PMS2P5
A_33_P3390477	10.919557	10.7832985	NM_146388	chr19:10370657-10370716	MRPL4
A_33_P3232478	5.4027543	5.30612	NM_000722	chr7:81579711-81579652	CACNA2D1
A_23_P171270	5.218017	4.9759316	NM_001017991	chrX:154610717-154610776	H2AFB2
A_23_P135157	8.336011	8.591153	NM_001031689	chr9:26905805-26905746	PLAA
A_33_P3305418	6.158279	6.1684904	AK127935	chr10:126226392-126226451	
A_24_P402898	6.9598165	7.6631765	NM_001102653	chr4:146055623-146055564	OTUD4
A_23_P399255	8.49161	7.519173	NM_152737	chr6:13979997-13980056	RNF182
A_23_P30972	6.164325	5.9770637	NM_022091	chr6:101315882-101315823	ASCC3
A_33_P3381117	5.4990044	4.9477825	AK129545	chr2:240269754-240269695	LOC100128563
A_24_P369694	8.22102	8.204636	NM_178587	chr14:102368121-102368180	PPP2R5C
A_33_P3307660	5.288432	3.1979263	NM_001184854	chr16:87744901-87744842	KLHDC4
A_32_P213330	5.9531884	6.088536	NM_001177693	chr5:73200191-73205256	ARHGEF28
A_33_P3270802	11.323172	11.331291	NM_001256456	chr1:1247025-1246966	CPSF3L
A_33_P3350493	5.1627345	4.900689	NM_001286550	chr6:35747269-35747328	CLPSL2
A_24_P90216	9.737185	9.730862	NM_018490	chr11:27387978-27387919	LGR4
A_24_P16036	9.25594	9.326906	NM_001013663	chr2:25013405-25013346	PTRHD1
A_33_P3241428	7.9455476	7.839183	NM_015311	chr2:220415523-220415464	OBSL1
A_24_P18917	6.056863	5.7845826	NM_022773	chr16:904229-904170	LMF1
A_33_P3268793	8.749346	8.522992	NM_138422	chr19:1913384-1913443	ADAT3
A_33_P3226008	5.489031	5.338795	NM_001190259	chr3:111842406-111842347	GCSAM
A_24_P288836	4.096596	3.3776076	NR_001435	chr6:33096422-33096481	HLA-DPB2
A_33_P3237110	5.1684155	4.4258146	NM_001099652	chr14:53104305-53104364	GPR137C
A_33_P3415216	8.675477	8.879179	NM_002437	chr2:27532573-27532514	MPV17
A_32_P189781	2.3221061	2.3900566	NR_026796	chr14:56247937-56247878	LINC00520
A_33_P3281563	5.3090734	4.933681		chr20:010200407-010200466	
A_32_P505730	4.488246	4.51404	BC017967	chr6:33086534-33086592	HLA-DPB2
A_24_P391260	8.7361145	8.543131	NM_004339	chr21:46281093-46276236	PTTG1IP
A_23_P259135	8.825663	9.037489	NM_014071	chr20:33302985-33302926	NCOA6
A_33_P3360392	5.9742336	5.9505672	NR_024129	chr9:98782134-98782075	LINC00092
A_32_P205110	6.703037	6.149144	NM_001453	chr6:1613858-1613917	FOXC1
A_33_P3349444	7.900264	8.200364	NM_000262	chr22:42455935-42455876	NAGA
A_23_P95594	7.60426	7.60426	NM_000662	chr8:18080376-18080435	NAT1
A_23_P356684	10.6908455	10.032911	NM_018685	chr7:36492587-36492646	ANLN
A_24_P396650	6.731195	6.7748475	NM_002953	chr1:26900869-26900928	RPS6KA1
A_23_P91487	5.200999	5.1766086	NM_016940	chr21:30378793-30378734	RWDD2B
A_33_P3288491	4.5472045	4.084678	NM_001081675	chr8:124657974-124657915	KLHL38
A_23_P332960	8.892191	8.978973	NM_001042463	chr11:703993-704052	TMEM80
A_33_P3344451	10.400059	10.650059	NM_032366	chr16:684643-684584	C16orf13
A_23_P203420	8.555744	8.208122	NM_004739	chr11:62360873-62360814	MTA2
A_23_P308673	6.248001	6.0759387	NM_004783	chr16:30003200-30003259	TAOK2
A_24_P219808	9.016097	9.01923	NM_017432	chr19:50363244-50363303	PTOV1
A_33_P3312842	4.509747	4.5638022	NM_147128	chr7:30407227-30407286	ZNRF2
A_23_P24244	9.273457	9.222967	NM_017782	chr10:5805350-5805406	FAM208B
A_33_P3271445	9.163912	9.457054	NM_001130105	chr5:74675230-74675171	COL4A3BP
A_33_P3210827	5.654828	5.734275	NM_002878	chr17:33445583-33445524	RAD51D
A_23_P259901	4.1546097	4.692209	NM_012253	chrX:153558624-153558683	TKTL1
A_33_P3217490	2.3221061	3.3703327	NR_038388	chr22:20325633-20325692	LOC729444
A_23_P167276	8.558071	8.283178	NM_001040202	chr4:79839565-79839506	PAQR3
A_32_P172864	8.698269	9.079533	NM_001010883	chr1:109181622-109181681	FAM102B
A_23_P109235	6.2291303	6.275834	NM_016732	chr20:32663841-32664561	RALY

A_33_P3253747	7.077627	7.3148594	NM_000761	chr15:75048866-75048925	CYP1A2
A_33_P3735158	4.044282	3.7700546	AK093004	chr9:69135853-69135912	LOC286272
A_23_P26254	8.830338	8.763117	NM_016013	chr15:41679773-41679714	NDUFAF1
A_23_P130731	5.835919	5.6401577	NM_024682	chr19:50387617-50387765	TBC1D17
A_23_P354387	11.813187	11.942944	NM_013451	chr10:95066378-95066319	MYOF
A_33_P3331193	3.1352508	2.3900566	DQ926483	chr14:106641741-106641682	
A_23_P317800	9.446329	9.415685	NM_013367	chr4:25419940-25419999	ANAPC4
A_23_P113656	5.526807	5.5627766	NM_006087	chr19:6494704-6494645	TUBB4A
A_23_P67648	8.050745	8.458701	NM_001749	chr19:36640809-36640868	CAPNS1
A_33_P3233774	6.5802917	6.4229097	NM_001032292	chr16:3454983-3455042	ZNF174
A_32_P217510	10.206724	9.897303	NM_032168	chr2:190340013-190340072	WDR75
A_23_P41143	8.065063	7.7267456	NM_001640	chr3:49719361-49719800	APEH
A_33_P3263274	11.648045	11.348846		chr16:002887034-002886975	
A_23_P9443	8.969382	8.975378	NM_016520	chr9:132591728-132591568	C9orf78
A_33_P3378880	8.300486	8.054692	BC130558	chr6:26104130-26104189	
A_33_P3405769	6.4187155	6.030559	NM_001135686	chr19:54573325-54573266	TARM1
A_33_P3399248	8.812566	8.687333	NM_001286706	chr13:38934895-38934954	UFM1
A_23_P15369	4.991165	4.765423	NM_174892	chr17:72518080-72518021	CD300LB
A_32_P33083	2.3221061	2.3900566	NM_016378	chrX:8138161-8138102	VCX2
A_23_P215484	4.7690544	3.5625873	NM_006072	chr7:75401219-75399061	CCL26
A_33_P3380742	11.0706005	11.233244	NM_001167947	chrX:48750791-48750732	TIMM17B
A_24_P194934	12.611908	12.649714		chr4:062971680-062971621	
A_23_P324340	8.221854	8.529832	NM_033510	chr15:40663181-40663240	DISP2
A_33_P3262973	4.9524717	5.549855		chr2:3135828-3135769	
A_23_P66599	9.70004	9.6467705	NM_032353	chr17:40931320-40931379	VPS25
A_33_P3775848	4.8063297	4.79294	NM_001289	chrX:154506625-154506566	CLIC2
A_24_P309317	6.783073	7.2520933	NM_001042465	chr10:73579260-73578848	PSAP
A_33_P3270295	4.006305	4.5947	NM_006653	chr6:41738005-41737946	FRS3
A_33_P3261328	6.643715	6.6993275	NM_002016	chr1:152276505-152276446	FLG
A_33_P3418371	12.615626	12.782082	AY358234	chr19:2294966-2295025	LOC100129831
A_24_P392110	2.3221061	2.3900566	NM_182707	chr19:43258549-43258490	PSG8
A_23_P133585	9.650956	9.336257	NM_001799	chr5:68572420-68572479	CDK7
A_33_P3252479	6.831626	6.625829	NM_001243491	chr1:150917712-150917771	SETDB1
A_33_P3318993	3.697803	4.749543		chr11:055259445-055259504	
A_23_P48535	5.8723655	6.412029	NM_001030055	chr14:32624171-32624230	ARHGAP5
A_23_P71433	9.023564	9.437731	NM_001001481	chr8:74705429-74705370	UBE2W
A_24_P943113	6.1038485	6.607744	NM_173359	chr3:71732228-71732169	EIF4E3
A_23_P312863	12.211215	12.327538	NM_006088	chr9:140137646-140137705	TUBB4B
A_23_P208847	4.7342076	4.693516	NM_016539	chr19:4174575-4174516	SIRT6
A_23_P340333	9.359165	8.934896	NM_033397	chr10:106072118-106072059	ITPRIP
A_33_P3407256	5.9239902	6.499136	NM_001166260	chr5:901470-901529	TRIP13
A_33_P3237413	9.634634	9.575886	AK124190	chr7:99235450-99235509	
A_32_P82189	7.878476	8.020144	NM_032180	chr2:62066799-62066740	FAM161A
A_23_P136232	9.6702585	9.59565	NM_017813	chr8:57875698-57875639	IMPAD1
A_33_P3352268	3.996503	2.3900566		chr10:134243928-134243869	
A_33_P3220202	11.051308	10.813198	NM_013355	chr9:131483128-131483187	PKN3
A_33_P3291776	12.183184	11.876897	NM_018946	chr9:100845266-100845325	NANS
A_33_P3263625	3.192677	2.3900566	NM_004420	chr11:1576369-1576310	DUSP8
A_23_P38497	6.741998	6.441043	NM_003562	chr17:4841477-4841339	SLC25A11
A_24_P350644	9.57299	9.517223	NM_013318	chr9:134375328-134375387	PRRC2B
A_24_P248053	10.94918	10.86965	NM_052963	chr8:144391634-144391575	TOP1MT
A_33_P3240773	4.0495405	4.063045	BC043599	chr3:10057193-10057134	CIDECP
A_23_P385217	9.612076	9.566097	NM_018184	chr3:5222360-5222419	ARL8B
A_24_P393461	8.343842	8.792773	NM_138740	chr1:154185028-154184969	C1orf43
A_23_P162607	4.505249	5.393713	NM_017564	chr12:104160373-104160432	STAB2
A_33_P3383034	11.255164	10.996225	NM_005499	chr19:34960737-34960795	UBA2
A_23_P28772	6.9907165	7.2200007	NM_001048226	chr20:44038912-44038971	DBNDD2
A_23_P205389	5.236816	5.8665214	NM_022151	chr14:93649634-93649575	MOAP1
A_33_P3320748	9.431902	9.647649	NM_001031734	chr19:10421589-10421530	FDX1L

A_33_P3392537	7.161092	6.8533173	NM_004614	chr16:66543411-66543352	TK2
A_33_P3249716	6.9808445	7.5411205		chr2:39892424-39892483	LOC728730
A_23_P21409	7.8909225	7.557701	NM_001042384	chr3:134268989-134269048	CEP63
A_33_P3314446	4.0027494	4.2645354		chr15:22136977-22133286	NF1P2
A_24_P199655	11.325333	11.285087	NM_138959	chr1:116233919-116233978	VANGL1
A_23_P44760	7.1181464	7.3646975	NM_014159	chr3:47058639-47058580	SETD2
A_23_P24129	6.185325	5.933876	NM_012242	chr10:54076687-54076746	DKK1
A_33_P3240348	6.096854	6.5805287	NM_000267	chr17:29557291-29557351	NF1
A_33_P3300117	4.486585	4.431224			
A_23_P257743	6.5100265	6.466616	NM_003028	chr9:37919951-37919892	SHB
A_33_P3242373	6.6328263	6.937501	NM_005935	chr4:88036391-88036450	AFF1
A_33_P3535649	4.846656	3.9504328	NM_032296	chr16:3001141-3001200	FLYWCH1
A_33_P3317376	5.5476017	5.6317306	NM_001665	chr11:3848705-3848646	RHOG
A_33_P3245389	8.152431	8.028761	NM_017799	chr14:57116092-57116151	TMEM260
A_23_P374559	3.856232	2.9363308	NM_181710	chr19:5456551-5456610	ZNRF4
A_33_P3301620	6.959997	6.7737427	NM_001184997	chr7:156437391-156437450	RNF32
A_33_P3295738	6.510577	6.565971	NM_001135639	chr16:57994804-57994745	CNGB1
A_24_P142095	8.775158	8.980516	NM_006738	chr15:86292473-86292532	AKAP13
A_23_P149992	6.950801	7.2813907	NM_020992	chr10:97007103-97007044	PDLIM1
A_23_P45361	9.530449	9.971987	NM_012084	chrX:120183096-120183155	GLUD2
A_24_P601972	5.612168	5.5140705	NM_001143959	chr2:61389991-61390050	C2orf74
A_24_P238510	4.4369593	4.562775		chrX:113625420-113625479	
A_23_P387071	8.517411	8.719964	NM_052857	chr17:33289909-33289968	ZNF830
A_23_P317796	4.533448	3.1409447	NM_178176	chr7:100843532-100842070	MOGAT3
A_24_P89843	5.484709	5.6391244	NM_032687	chr8:145689738-145689679	CYHR1
A_23_P36076	12.69109	12.608693	NM_003146	chr11:57093651-57093592	SSRP1
A_23_P94800	7.835519	7.425277	NM_002961	chr1:153516257-153516198	S100A4
A_33_P3254579	3.802809	3.4420862	NM_031957	chr17:39183286-39183227	KRTAP1-5
A_23_P28582	6.301717	6.6156826		chr2:96197150-96197209	
A_33_P3290924	7.9859276	8.011028	NM_001172415	chr9:33256859-33256800	BAG1
A_23_P24716	9.723866	9.844624	NM_017870	chr11:60704552-60704611	TMEM132A
A_23_P214739	8.128403	8.474821	NM_012160	chr6:99322242-99322183	FBXL4
A_24_P109838	2.330655	2.3900566	NM_182767	chr12:85266530-85266471	SLC6A15
A_33_P3277728	7.0025997	7.0218616	NM_001001132	chr21:35208842-35208901	ITSN1
A_23_P21485	8.641489	8.024663	NM_017933	chr2:229889030-229888971	PID1
A_24_P218265	11.829987	11.991159	NM_003842	chr8:22877835-22877776	TNFRSF10B
A_33_P3289426	8.347969	8.190326	NM_173680	chr7:150095660-150095719	ZNF775
A_33_P3404448	6.610275	6.208538	NM_152551	chr6:7595269-7595328	SNRNP48
A_33_P3373541	5.30612	4.9592066	NM_173717	chr17:12908366-12908307	ELAC2
A_23_P353056	7.7263536	7.530223	NM_014807	chr11:118987673-118987732	C2CD2L
A_23_P207037	5.327579	5.3687115	NM_007261	chr17:72477870-72477929	CD300A
A_24_P80532	5.971339	7.05737	NM_004354	chr4:78082710-78082859	CCNG2
A_23_P7056	6.015812	5.623542	NM_018115	chr4:76871443-76871384	SDAD1
A_23_P170453	7.332473	7.4065447	NM_001900	chr20:23858186-23856894	CST5
A_33_P3356306	5.8781796	5.42677	AF323919	chr2:99860310-99860251	LYG2
A_33_P3320953	9.522642	10.203657	NM_206833	chr19:7989441-7989382	CTXN1
A_33_P3274811	6.475452	6.414462	NM_001014450	chr1:153084715-153084656	SPRR2F
A_33_P3418426	5.968609	6.895335	NM_000341	chr2:44539834-44539892	SLC3A1
A_23_P33558	7.16957	7.176972	XR_109936	chr2:109960662-109960603	LOC729164
A_33_P3235184	11.617973	11.66214		chr17:015347334-015347393	
A_23_P80594	6.205929	6.637438	NM_015184	chr3:17131469-17131528	PLCL2
A_23_P49559	4.730502	5.2520814	NM_181790	chr17:72368298-72368357	GPR142
A_23_P70020	9.813455	9.73489	NM_002622	chr5:139624866-139624807	PFDN1
A_33_P3316273	2.3221061	2.3900566	NM_002983	chr17:34415664-34415605	CCL3
A_33_P3249877	6.003479	6.0856667	NM_001282614	chr12:70037530-70037471	BEST3
A_23_P5392	9.15336	9.352462	NM_004881	chr2:24302356-24300615	TP53I3
A_33_P3250895	4.6278605	3.560503	NM_001099780	chr14:23512135-23512194	PSMB11
A_23_P416468	10.23758	10.266993	NM_025049	chr15:65107975-65107916	PIF1
A_23_P30338	10.56548	10.447917	NM_001239	chr5:86690889-86690271	CCNH

A_33_P3239222	10.112625	9.909222	NM_001037283	chr7:2420018-2420077	EIF3B
A_23_P78543	8.631449	8.293711	NM_005498	chr19:10685629-10685159	AP1M2
A_23_P96623	4.291984	4.287895	NM_000513	chrX:153455551-153455610	OPN1MW
A_23_P314222	8.965763	8.790794	NM_138792	chr15:52242044-52239567	LEO1
A_24_P49214	7.6125917	7.48403	NM_001282671	chr1:2116522-2116463	C1orf86
A_23_P92082	12.769602	12.768322	NM_001064	chr3:53259888-53259829	TKT
A_33_P3226385	4.424848	3.909256		chr2:25431953-25432012	
A_33_P3334308	7.6947494	7.368447	NM_007157	chrX:57623831-57623890	ZXDB
A_33_P3321372	7.2413588	6.9527483	XM_005276334	chr9:43819620-43819679	
A_24_P363005	12.491766	12.700055	NM_181886	chr4:103717850-103717791	UBE2D3
A_33_P3268408	6.3413944	6.1381235		chr6:053246749-053246808	
A_33_P3279715	5.6809883	6.309342	NM_152896	chr9:6421082-6421141	UHRF2
A_23_P71053	6.8389306	6.631089	NM_016447	chr7:24718879-24718938	MPP6
A_24_P169773	9.32565	9.428035	NM_198495	chr7:143882966-143883025	CTAGE4
A_24_P886040	7.6344852	6.8818893	NM_152624	chr5:112356209-112356268	DCP2
A_33_P3376636	6.537446	6.68559	NM_001017989	chr19:46031086-46031027	OPA3
A_23_P31399	10.092465	10.360766	NM_000305	chr7:95034559-95034500	PON2
A_24_P12626	11.909884	11.688069	NM_001753	chr7:116199001-116199060	CAV1
A_33_P3278941	6.8168826	7.258717	NM_001048205	chr14:24649400-24649459	REC8
A_33_P3284453	7.413887	7.0209665	NM_001012279	chr6:127771389-127771330	SOGA3
A_23_P132536	7.9276247	7.985032	NM_001042646	chr3:42266914-42266973	TRAK1
A_33_P3220818	11.167954	10.457395	NM_001201338	chr19:5668391-5668450	SAFB
A_33_P3346680	9.1242285	9.109415		chr16:028711589-028711530	
A_33_P3258330	4.3934717	4.787907	NM_030792	chr11:75236370-75236311	GDPD5
A_33_P3329255	7.5357842	7.625904	BC018681	chr10:75570509-75570450	
A_33_P3357918	11.186832	11.073485	NM_012088	chr19:17632032-17632091	PGLS
A_33_P3251776	5.3381567	5.824276	NM_001142777	chr16:31000282-31000341	HSD3B7
A_33_P3307119	4.0836267	4.2858043	NM_001004759	chr11:4903973-4904032	OR51T1
A_23_P9894	7.3883557	7.3998003	NM_005788	chr11:20529950-20530009	PRMT3
A_23_P53345	7.1822863	7.0801945	NM_020183	chr12:27568866-27571065	ARNTL2
A_24_P310630	8.5399275	8.634343	NM_080632	chrX:118968535-118968476	UPF3B
A_33_P3287735	7.3767133	7.01695	NM_001099412	chr8:41787702-41787643	KAT6A
A_23_P84775	9.774781	9.833165	NM_002669	chr4:155458630-155458571	PLRG1
A_23_P358709	7.8040013	8.013159	NM_020731	chr5:438030-438089	AHRR
A_33_P3312682	10.638592	11.032902	NM_020695	chr19:1815304-1815245	REXO1
A_23_P141194	11.621399	11.287387	NM_001545	chr17:73017278-73017337	ICT1
A_23_P97442	7.6535287	7.2008214	NM_032125	chr1:27662588-27662647	TMEM222
A_33_P3236157	11.2621565	10.479117	NM_003362	chr12:109548738-109548797	UNG
A_23_P214330	9.030188	9.080048	NM_030666	chr6:2833923-2833864	SERPINB1
A_24_P916496	10.135677	9.85832	NM_002737	chr17:64806770-64806829	PRKCA
A_23_P18604	10.289147	10.184152	NM_015907	chr4:17609187-17609246	LAP3
A_33_P3420466	5.7216115	5.9939814	NM_002381	chr2:20194120-20194061	MATN3
A_23_P70583	4.947463	4.7477646	NM_022553	chr6:33219398-33219339	VPS52
A_23_P259189	9.334223	9.26936	NM_013943	chr1:25166518-25167308	CLIC4
A_33_P3853081	4.0990663	2.3900566	M21191	chr10:127354871-127354812	ALDOAP2
A_33_P3253264	5.5754776	5.280776	NM_005341	chr1:6648844-6648903	ZBTB48
A_23_P204536	7.5472713	7.153358	NM_001267595	chr12:48437173-48437114	SENP1
A_33_P3419594	7.808427	7.411255	AK074662	chr21:44190119-44190178	PDE9A
A_23_P46238	9.881194	9.80728	NM_033440	chr1:15793981-15798491	CELA2A
A_23_P100704	7.6856227	7.9053154	NM_139033	chr17:19286634-19286693	MAPK7
A_33_P3404052	5.0832376	4.9752965	NM_001286813	chr5:118729017-118729076	TNFAIP8
A_33_P3272461	10.5680275	9.993337	NM_014168	chr2:170677699-170677640	METTL5
A_23_P51936	3.6465201	4.338008	NM_001561	chr1:7980782-7980723	TNFRSF9
A_23_P71148	10.846319	10.874678	NM_000712	chr7:43846842-43846901	BLVRA
A_23_P163087	10.18746	10.9675045	NM_007361	chr14:52471790-52471731	NID2
A_33_P3367073	7.1677513	7.4937696	XM_005275810	chr17:2627468-2627409	LOC101928991
A_23_P88703	8.293644	8.71446	NM_005657	chr15:43700182-43699751	TP53BP1
A_24_P933492	6.7488513	6.173131	NM_178566	chr9:14693243-14690341	ZDHHC21
A_23_P325438	5.6122456	5.6836963	NM_015171	chr16:28115917-28113258	XPO6

A_33_P3225587	9.17665	8.888902	NM_025146	chr3:113440666-113440607	NAA50
A_33_P3234197	6.6779284	6.4542036	NM_025188	chr1:117653774-117653715	TRIM45
A_24_P414376	8.570539	9.124618	NM_016531	chr4:38702518-38702577	KLF3
A_33_P3261620	4.7046556	4.7116156	NM_004122	chr3:172165543-172165484	GHSR
A_23_P76841	9.616726	9.4223	NM_001042635	chr14:23946514-23946719	NGDN
A_32_P38623	6.197381	6.0428514	NM_017650	chr7:94925547-94925606	PPP1R9A
A_33_P3384432	9.570557	9.591787	NM_001178138	chr3:141671414-141671355	TFDP2
A_23_P140821	2.4848082	2.3900566	NM_016948	chr16:67696162-67696221	PARD6A
A_23_P210348	10.712155	10.5778055	NM_004718	chr2:42577932-42577873	COX7A2L
A_23_P126727	8.394559	7.9739103	NM_024654	chr1:6585834-6585775	NOL9
A_33_P3396473	9.599868	9.789213	NM_001242898	chr22:50882699-50882758	PPP6R2
A_33_P3262670	6.195802	5.531685	NM_001173517	chrX:135295440-135295381	MAP7D3
A_24_P340679	13.14254	12.626408	NM_021130	chr7:44839021-44839301	PPIA
A_23_P37265	8.259859	8.347969	NM_018229	chr14:57756085-57756144	AP5M1
A_23_P143935	7.3437166	7.557063	NM_025163	chr3:196673315-196673256	PIGZ
A_32_P377880	4.519987	2.3900566	NM_001190468	chr5:37812991-37812932	GDNF
A_33_P3287078	3.9729688	3.4734845	NM_022482	chr20:23349507-23349566	GZF1
A_24_P302374	8.12601	8.171566	NM_001286	chr1:11903126-11903185	CLCN6
A_24_P91916	7.7224298	7.224864	NM_018698	chrX:108787550-108787609	NXT2
A_24_P126741	7.2140336	6.8745756		chr5:161178628-161178569	
A_33_P3311663	4.422464	4.0400167	XR_245014	chr2:66666701-66666642	
A_33_P3240518	11.670954	11.594166	NM_001127230	chr1:1309489-1309430	AURKAIP1
A_23_P57709	8.319636	8.064532	NM_013363	chr3:142536972-142536913	PCOLCE2
A_33_P3418597	7.990855	7.8155136	NM_152236	chr22:29708427-29708486	GAS2L1
A_33_P3277988	7.5494266	7.3966427	NM_000629	chr21:34732064-34732123	IFNAR1
A_24_P347880	4.58597	5.013267	NM_021628	chr17:8006732-8006673	ALOXE3
A_32_P13151	3.9948423	3.264452	NR_027995	chr13:19414198-19414139	ANKRD20A9P
A_33_P3286254	11.034128	11.253003	NM_001284	chr5:115249549-115249608	AP3S1
A_33_P3397298	5.4866076	5.306568		chr14:036350821-036350880	
A_33_P3316088	4.5348215	4.1169705		chr1:52509891-52509950	
A_32_P184488	7.5707717	7.6185746	NM_198850	chr19:43979334-43979275	PHLDB3
A_33_P3342917	5.118637	5.4452553	NM_004710	chr17:76168950-76169009	SYNGR2
A_32_P56249	2.9941769	4.0463743	NR_038996	chr12:109490223-109490164	USP30-AS1
A_33_P3272527	7.729939	7.917122	NM_001206491	chr20:3835323-3835382	MAVS
A_33_P3783235	7.6099076	7.123065	AK095104	chr8:125318497-125318438	
A_33_P3424636	4.103785	4.5588183	A25968	chr7:142045681-142045740	
A_33_P3864411	6.005314	6.4790163	NR_045000	chr10:38991167-38991226	ACTR3BP5
A_33_P3268763	11.742368	11.938358	NM_031434	chr7:150778233-150778174	TMUB1
A_33_P3254320	3.56352	4.068737	NM_005490	chr19:6752368-6752309	SH2D3A
A_23_P406986	4.455132	4.5123053	NM_152581	chrX:14939335-14939394	MOSPD2
A_33_P3238355	5.2339945	5.069796		chr2:133043427-133043368	
A_33_P3361457	10.312594	10.790112	NM_000874	chr21:34625061-34625120	IFNAR2
A_33_P3350748	10.086093	10.356567	NM_005556	chr12:52642590-52642649	KRT7
A_32_P34138	4.403175	3.8413146	NM_001146157	chr10:88784387-88784446	FAM25A
A_32_P225355	6.044671	6.3620133	NM_182485	chr4:15070793-15070852	CPEB2
A_33_P3234168	11.766264	11.692598	NM_178812	chr8:98738442-98738501	MTDH
A_24_P373286	7.8883724	7.6359453	NM_014711	chr16:19564500-19564559	CCP110
A_23_P416178	4.0147123	3.5192738	NM_022742	chr7:128454834-128454893	CCDC136
A_24_P494454	4.222808	3.831904	NM_001010862	chrX:57017803-57017744	SPIN3
A_23_P95213	4.4201813	2.3900566	NM_003018	chr8:22021933-22021992	SFTPC
A_33_P3373810	3.6040673	2.3900566		chr5:148489219-148489278	LOC255187
A_33_P3708364	11.328662	11.339222	AK126853	chr5:86614292-86614233	LOC644285
A_23_P54622	8.686109	8.703916	NM_007317	chr16:29815336-29816143	KIF22
A_23_P315286	4.651767	3.807242	NM_138774	chr19:897179-897120	R3HDM4
A_23_P383977	6.342249	6.4220247	NM_006049	chr15:66787689-66786853	SNAPC5
A_23_P202520	10.453802	10.535272	NM_001003408	chr10:116190950-116190891	ABLIM1
A_33_P3236441	8.15991	8.523193	NM_144972	chr11:18500808-18500867	LDHAL6A
A_23_P210643	8.4927025	8.41438	NM_198976	chr20:57568709-57568768	NELFCD
A_23_P314805	5.53147	5.0786376	NM_152487	chr1:95658415-95658474	TMEM56

A_33_P3221134	5.2815537	4.70164		chr2:069826310-069826369	
A_24_P259922	8.438826	8.583701	NM_006055	chr2:211296744-211296685	LANCL1
A_33_P3223678	6.0411596	6.3461685	NM_014564	chr9:139089230-139089171	LHX3
A_23_P35349	7.511671	7.7313995	NM_021738	chr10:29747064-29747005	SVIL
A_33_P3231557	6.7293	7.37266	NM_003671	chr9:99284848-99284789	CDC14B
A_24_P184769	7.7290645	7.5441074	NM_181291	chr14:102689637-102689696	WDR20
A_24_P226755	2.3221061	2.3900566	NM_014729	chr8:59718581-59718522	TOX
A_23_P45108	9.376506	9.574403	NM_017730	chr3:49067605-49067546	QRICH1
A_33_P3254291	4.6196613	4.615377	NM_001190848	chr7:135072846-135072787	CNOT4
A_33_P3245484	3.8361752	3.4452565	AK124361	chr17:76841031-76841090	LOC100131000
A_23_P157784	7.7388678	7.9990225	NM_032593	chr9:35813694-35813541	HINT2
A_23_P408996	4.6787844	4.308523	NM_001080480	chr6:20101206-20101147	MBOAT1
A_33_P3504002	4.245704	3.7405987		chr8:66439649-66439590	LOC286186
A_23_P1962	7.0879364	7.151745	NM_004585	chr11:63313827-63313886	RARRES3
A_33_P3381762	8.33107	8.383715		chr19:4791816-4791757	
A_33_P3327522	4.800843	5.363846	CU680799	chr20:061444208-061444149	
A_24_P24142	13.298884	13.3183975		chr2:174162659-174162718	
A_23_P46095	8.761955	8.772168	NM_152379	chr1:231360035-231359976	C1orf131
A_33_P3274245	4.1872644	4.528322	NM_173627	chr17:78395702-78395761	ENDOV
A_33_P3256105	6.757449	6.9302087	NM_001127386	chr10:135485016-135485075	DUX4L2
A_23_P206612	6.808381	6.9859056	NM_020718	chr16:23079412-23079354	USP31
A_33_P3217958	4.200089	3.9617648	NM_014824	chr11:72549503-72549444	FCHSD2
A_33_P3305093	5.5862513	5.8816276	NM_001103167	chr19:10415811-10415752	ZGLP1
A_24_P726336	9.576338	9.192982	NM_001100164	chr6:144151872-144151931	PHACTR2
A_33_P3340154	5.912923	5.2383523	NM_022719	chr22:19117975-19117916	DGCR14
A_23_P117363	7.8665066	8.155405	NM_001756	chr14:94770689-94770630	SERPINA6
A_33_P3368495	9.187789	8.832087	XM_005261346	chr22:38692271-38692212	CSNK1E
A_23_P254842	8.166818	9.118174	NM_012080	chrX:6967420-6967361	HDHD1
A_33_P3303519	6.3770676	6.3994718	NM_205852	chr12:10168271-10168330	CLEC12B
A_24_P71244	7.3690815	7.3710966	NM_005026	chr1:9788305-9788364	PIK3CD
A_23_P41114	2.3221061	2.3900566	NM_005213	chr3:122056416-122056475	CSTA
A_33_P3400217	6.493614	6.236993	NM_000342	chr17:42330628-42330569	SLC4A1
A_33_P3404701	5.379429	5.2235374	NM_005065	chr14:81946016-81945957	SEL1L
A_33_P3407592	4.6872306	4.86602		chr16:32949302-32949361	XLOC_014512
A_23_P57277	2.5641813	2.3900566	NM_020152	chr21:30547359-30547418	MAP3K7CL
A_33_P3240647	8.004706	7.9812117	NM_001271441	chr21:45749833-45749774	C21orf2
A_33_P3326285	12.958728	12.825949	NR_002578	chr1:173833108-173833049	GAS5
A_23_P254415	14.9299965	15.034151	NM_002295	chr3:39453399-39453458	RPSA
A_33_P3299319	5.9416256	5.0683126	NM_001136137	chr19:55899028-55899087	RPL28
A_23_P142187	10.224163	10.396851	NM_022462	chr19:46846381-46846440	HIF3A
A_33_P3237590	3.8570342	3.0014038	AK123396	chr5:14663329-14663270	
A_24_P88554	9.375069	9.195093	NM_003846	chr1:145523603-145523662	PEX11B
A_33_P3338066	4.2623096	2.3900566	NM_001164586	chr1:201175502-201175561	IGFN1
A_23_P70445	11.755066	11.869513	NM_003532	chr6:26225566-26225625	HIST1H3E
A_23_P155688	2.3221061	2.3900566	NM_021114	chr4:57676212-57676153	SPINK2
A_24_P314451	5.653133	5.378422	NM_000132	chrX:154064755-154064696	F8
A_24_P245838	8.466717	8.407822	NM_002409	chr22:39888092-39888151	MGAT3
A_33_P3334037	9.41423	9.148609	NM_016312	chr2:128007803-128007862	WBP11
A_23_P321261	7.864944	8.09928	NM_002396	chr18:48473496-48473555	ME2
A_23_P362148	4.184182	2.3900566	NM_139072	chr2:230222968-230222909	DNER
A_23_P426944	10.154106	11.226423	NM_006194	chr14:37145552-37145611	PAX9
A_23_P31866	10.408105	10.46669	NM_080651	chr8:118552145-118552204	MED30
A_33_P3373459	8.937849	9.315378	NM_018641	chr7:2473611-2473670	CHST12
A_23_P380754	3.6106772	2.3900566	NM_002769	chr7:142459723-142459782	PRSS1
A_23_P29851	6.5562973	6.3354917	NM_002337	chr4:3526643-3521871	LRPAP1
A_33_P3245449	4.265373	4.6729326	NR_027420	chr21:9919481-9919422	LOC389834
A_23_P1056	7.525961	7.4788084	NM_016334	chr1:147465173-147465232	GPR89B
A_24_P792124	5.0714164	5.743139	NM_002977	chr2:167052099-167052040	SCN9A
A_33_P3622472	9.381458	9.225712	NM_001256119	chrX:153197847-153197788	NAA10

A_23_P54517	9.049128	9.203494	NM_006293	chr15:41871259-41871318	TYRO3
A_33_P3214129	9.099069	8.719505	AK025151	chr9:97826146-97826205	LOC728061
A_33_P3283196	6.617081	6.3973713		chr12:046354525-046354466	
A_23_P21785	6.4605894	6.666831	NM_022072	chr3:93845159-93845218	NSUN3
A_24_P136470	5.418165	5.348313	NR_027795	chr6:26426750-26428156	BTN2A3P
A_23_P313389	9.239731	10.271232	NM_003358	chr9:114691888-114691947	UGCG
A_33_P3325439	8.5737095	8.689439	NM_001001992	chr21:30403034-30403093	USP16
A_33_P3622802	6.276672	6.327351	NM_005470	chr10:27036506-27036447	ABI1
A_24_P358381	4.4319587	4.241293	NM_012227	chrX:231124-231065	GTPBP6
A_33_P3267814	6.3641376	6.2258935	NM_001136004	chr22:18343047-18342988	MICAL3
A_33_P3351316	4.032208	2.9878213	AK091001	chr3:43101640-43101699	FAM198A
A_23_P17456	5.5477896	5.3850813	NM_006065	chr20:1546848-1546134	SIRPB1
A_33_P3338909	12.396936	12.592546	NM_001270439	chr1:183595452-183595393	ARPC5
A_23_P150189	8.255937	8.0355	NM_005590	chr11:94192715-94192656	MRE11A
A_24_P179183	8.560414	8.760824	NM_015208	chr18:9254599-9254658	ANKRD12
A_33_P3371089	7.667664	7.147024	NM_019072	chr5:64961838-64961779	SGTB
A_23_P71830	4.83057	4.4338098	NM_020924	chr9:125681037-125680978	ZBTB26
A_33_P3262024	6.8796797	6.748188		chr3:197372674-197372733	LOC101930159
A_33_P3413671	9.379128	8.888643	NM_007314	chr1:179073242-179073183	ABL2
A_32_P232035	5.46885	4.88512	NR_026776	chr6:26987283-26987224	LOC100270746
A_23_P75609	7.411725	7.382641	NM_014956	chr11:117283690-117283749	CEP164
A_23_P92650	4.292639	3.901208	NM_031947	chr5:140682338-140682279	SLC25A2
A_23_P168771	5.1669846	4.6832	NM_020879	chr7:76924182-76924241	CCDC146
A_23_P125408	4.4290605	3.2004619	NM_031304	chr19:3491420-3491361	DOHH
A_33_P3378354	4.8119335	4.398946		chr13:51589613-51589554	GUCY1B2
A_23_P23266	7.2876825	7.354264	U79751	chr1:169356909-169356968	BLZF1
A_24_P941487	5.4881616	5.465473	NM_001008401	chr19:53960813-53960872	ZNF761
A_32_P165113	8.137802	8.186765	NM_138458	chr2:68358478-68358419	WDR92
A_24_P28295	6.037298	6.2824883	NM_001243765	chr1:174959004-174959063	RABGAP1L
A_33_P3403583	5.170755	5.2026505	AB231740	chr11:56569699-56569640	
A_24_P288448	6.295235	6.3565416	BC110385	chr20:4773654-4773229	RASSF2
A_33_P3308153	7.6087685	7.7334404		chr22:050052149-050052208	
A_23_P85209	5.6442685	5.0832376	NM_000640	chrX:114238650-114238591	IL13RA2
A_33_P3233906	2.3221061	3.3237164	NM_005855	chr2:238820539-238820598	RAMP1
A_33_P3304794	4.450347	4.816511	AK096324	chr19:58912526-58912467	
A_23_P56256	10.467494	10.589925	NM_006233	chr19:36605282-36605136	POLR2I
A_33_P3230037	8.414936	8.711037	AF336885	chr11:64469434-64469375	
A_24_P346855	7.1796584	7.4416747	NM_002417	chr10:129895700-129895641	MKI67
A_24_P124370	9.676334	9.680312	NM_018222	chr11:12551259-12551318	PARVA
A_33_P3257050	4.2272363	4.3695927		chr4:039772003-039771944	
A_33_P3340490	6.628251	6.2027583	NR_033896	chr6:139018366-139018425	FLJ46906
A_23_P205519	6.9744635	6.7812734	NM_022060	chr14:23081028-23081087	ABHD4
A_33_P3357382	7.5996957	7.7819	BC057773	chr1:151399803-151399744	POGZ
A_33_P3353712	9.721608	9.93817	NM_007033	chr1:2333705-2333764	RER1
A_23_P145584	7.827514	8.132952	NM_003344	chr7:129474769-129474710	UBE2H
A_23_P88435	4.7977386	4.9467096	NM_005197	chr14:89623598-89623539	FOXN3
A_33_P3259293	4.8365364	3.894019	AK131347	chr7:140102392-140102333	SLC37A3
A_33_P3308774	6.2191806	6.380532	DB066855	chr1:40429239-40429298	
A_33_P3251552	2.3221061	2.3900566	NM_001029871	chr20:939192-939133	RSPO4
A_33_P3862375	7.451234	7.2390666	NM_001080481	chr6:99880709-99880650	USP45
A_23_P23855	9.224274	9.259901	NM_032305	chr1:145456379-145456320	POLR3GL
A_33_P3620246	6.0415688	6.2925067	XR_109175	chr15:76069736-76069795	
A_33_P3415211	8.149713	8.3064575	NM_002437	chr2:27532634-27532575	MPV17
A_23_P313632	8.037026	8.050745	NM_178155	chr14:66209523-66209582	FUT8
A_23_P130482	5.3239174	5.3859143	NM_198855	chr19:58153543-58153602	ZNF211
A_33_P3242458	5.4646254	5.508731	NM_017836	chr3:125725888-125725829	SLC41A3
A_33_P3235400	8.23641	7.9945493	NM_004494	chr1:156713610-156713551	HDGF
A_33_P3354137	10.608776	10.762769	NM_030885	chr3:48130383-48130324	MAP4
A_23_P94591	10.651138	10.903114	NM_032928	chr9:139687580-139687639	TMEM141

A_33_P3612589	7.686364	7.569415	NM_000489	chrX:76761400-76761341	ATRX
A_24_P239176	7.969709	8.147998	NM_018406	chr3:195514819-195514760	MUC4
A_33_P3351554	9.690769	9.954823	NM_018208	chr1:204100263-204100204	ETNK2
A_23_P73208	6.0808206	6.5541735	NM_144618	chr1:151090847-151090906	GABPB2
A_33_P3351356	6.01974	6.1210637	BC040474	chr8:1851609-1851668	ARHGEF10
A_33_P3247210	6.172781	6.377883		chr2:017935268-017935209	
A_33_P3294446	4.127895	4.5456724	NM_024567	chr8:28866639-28866698	HMBOX1
A_23_P340308	4.995934	5.3246264	NM_176888	chr12:11174380-11174321	TAS2R19
A_23_P251303	7.799457	7.9326887	NM_014866	chr9:139335368-139335309	SEC16A
A_33_P3420426	11.067415	10.688028	NR_027642	chr15:51238166-51238225	DCAF13P3
A_23_P59855	7.1969395	7.4349627	NM_006524	chr7:64293564-64293623	ZNF138
A_23_P70785	5.3859143	4.8284826	NM_001624	chr6:107017763-107017822	AIM1
A_23_P420196	7.3148594	7.4643726	NM_003745	chr16:11348443-11348384	SOCS1
A_23_P434347	4.8522987	5.13186	NM_147152	chr2:24469070-24469011	ITSN2
A_33_P3244951	9.630013	9.782245	NM_001243137	chr15:85682198-85682257	PDE8A
A_23_P157600	6.984125	7.3893185	NM_015214	chr8:38117600-38117659	DDHD2
A_24_P487736	5.7516837	5.499581	NM_198279	chrX:19934553-19934494	CXorf23
A_33_P3372285	4.348914	3.612365	NM_007137	chrX:47755271-47755330	ZNF81
A_33_P3255677	6.0953755	6.0480757		chr5:069403337-069403278	
A_23_P144337	4.3332415	3.7931855	NM_012118	chr4:139966027-139966086	CCRN4L
A_23_P19592	9.481358	9.585976	NM_015599	chr6:83878702-83878643	PGM3
A_23_P142750	9.4971075	9.505653	NM_002759	chr2:37341880-37336386	EIF2AK2
A_23_P121265	8.118196	7.7412186	NM_007284	chr3:52264904-52264046	TWF2
A_24_P216765	10.784255	10.204222	NM_014765	chr1:235277117-235275399	TOMM20
A_23_P376239	9.116058	9.122808	NM_032632	chr14:97010571-97014168	PAPOLA
A_23_P94546	6.5014324	6.3677025	NM_025211	chr9:86356872-86354606	GKAP1
A_33_P3333683	4.540166	4.369185		chr1:243646191-243646132	
A_33_P3340342	9.06761	8.991687	NM_144601	chr16:66647198-66647257	CMTM3
A_33_P3384885	5.819295	5.491036	XM_005252664	chr10:135126205-135126264	ZNF511
A_33_P3309319	9.588337	9.725694	NM_145886	chr11:799239-799180	PIDD
A_24_P336853	8.506923	8.151702	NM_020143	chr2:68402335-68402394	PNO1
A_23_P254442	6.631089	6.518406	NR_036573	chr7:148994188-148994247	LOC155060
A_33_P3412975	9.43152	9.548355	NM_175875	chr19:46268138-46268079	SIX5
A_33_P3414027	2.3221061	2.3900566	NR_015416	chr15:22371028-22371087	LOC727924
A_33_P3291176	5.104163	5.8090053	NM_001017921	chr19:5910197-5910256	VMAC
A_33_P3861706	7.908369	7.6102395	NM_017762	chr15:31231234-31231175	MTMR10
A_24_P21715	6.681672	6.446615	NM_004584	chr11:67165480-67165539	RAD9A
A_33_P3226600	13.36665	13.4944725	NM_003801	chr8:145141059-145141118	GPAA1
A_23_P37391	10.128957	9.224863	NM_001144995	chr14:99978443-99978384	CCDC85C
A_33_P3357062	6.341958	6.1745605	NM_020936	chr12:124101064-124101123	DDX55
A_33_P3338956	6.112429	6.3148074	NM_018184	chr3:5220684-5220743	ARL8B
A_23_P53176	5.662486	5.777221	NM_016725	chr11:71907268-71907327	FOLR1
A_24_P318073	7.192102	7.4594517	NM_032795	chr11:126072937-126072878	RPUSD4
A_33_P3301097	5.5345783	5.0284147		chr4:003912744-003912685	
A_33_P3259817	3.8841343	4.117345	NM_001130048	chr13:99449429-99449370	DOCK9
A_23_P364414	4.6520095	4.3576393	NM_032089	chr5:140784877-140784936	PCDHGA9
A_33_P3245517	2.3981316	2.3900566	NR_024380	chr10:42827945-42827886	LOC441666
A_33_P3411975	4.0840054	3.6647997	NM_015429	chr3:100585783-100585724	ABI3BP
A_23_P18196	11.011292	10.81678	NM_002916	chr3:186508130-186508001	RFC4
A_24_P24972	11.495451	11.484997		chr2:110494802-110494743	
A_24_P101642	5.922137	5.6926475		chr16:33605576-33605635	
A_33_P3317406	11.745298	11.767601	NM_007103	chr11:67379945-67380004	NDUFV1
A_23_P92132	9.179697	8.883937	NM_006764	chr3:50325604-50325545	IFRD2
A_33_P3243707	7.276208	7.16798	NM_014458	chr1:173755657-173755716	KLHL20
A_23_P97810	4.421171	4.7830467	NM_003631	chr10:51027148-51027089	PARG
A_24_P389038	6.528448	6.0285993	NM_005452	chr6:33248744-33248685	WDR46
A_24_P188377	7.439836	6.9438276	NM_000574	chr1:207510071-207510130	CD55
A_23_P358597	9.728345	9.694559	NM_022361	chr6:105606468-105606409	POPDC3
A_32_P228699	8.31629	8.435419	NM_199437	chr11:129769814-129769755	PRDM10

A_23_P166087	8.688873	8.546821	NM_014737	chr20:4760842-4760783	RASSF2
A_33_P3418218	3.9434755	3.2596073	NM_033127	chr1:177902679-177902620	SEC16B
A_23_P371239	8.227285	8.094992	NM_198390	chr16:81744898-81744957	CMIP
A_23_P39088	9.111509	8.856534	NM_001536	chr19:50188248-50189362	PRMT1
A_33_P3399492	8.87285	8.979932	NM_004168	chr5:240531-240590	SDHA
A_33_P3237819	3.766137	3.403376		chr19:037267934-037267993	
A_23_P411881	8.168885	7.9460125	NM_006346	chr13:73573119-73590051	PIBF1
A_33_P3324228	4.3647118	4.5620823		chr16:86369433-86369374	LINC00917
A_24_P38754	5.442229	5.289629	NM_018464	chr10:60048636-60048695	CISD1
A_23_P25684	11.54629	10.083971	NM_016026	chr14:68144288-68144229	RDH11
A_24_P277657	3.8817334	4.5805035	NM_006877	chr6:16290766-16290825	GMPR
A_33_P3252800	2.4494357	3.9015274	NM_002849	chr12:71050544-71050485	PTPRR
A_23_P422849	4.0275054	4.126315	NM_152898	chr7:19184640-19184581	FERD3L
A_24_P174824	9.128883	9.128426	NM_006425	chr5:159828872-159828813	SLU7
A_33_P3255531	4.484213	4.384	NM_001242853	chr11:71589499-71589556	LOC100129216
A_33_P3247392	5.9687257	6.236537	NM_199261	chr21:10951365-10951306	TPTE
A_33_P3356701	10.617237	11.193876	NM_032799	chr9:131483218-131483159	ZDHHC12
A_32_P81768	10.325713	10.395048	NM_174909	chr5:82352693-82352635	TMEM167A
A_23_P254944	2.4182034	2.3900566	NM_000853	chr22:24376392-24376333	GSTT1
A_23_P166122	6.611165	6.5370235	NM_006466	chr20:18464603-18464662	POLR3F
A_23_P361049	10.324818	10.081831	NM_012223	chr2:192289808-192289867	MYO1B
A_33_P3710442	5.5734634	5.4669466	AK021772	chr17:55357314-55357373	FLJ11710
A_23_P302005	6.4218698	6.5584974	NM_006873	chr2:48825258-48825317	STON1
A_23_P25746	5.5610404	5.2409906	NR_027263	chr14:32544949-32544890	ARHGAP5-AS1
A_32_P141724	6.1511784	6.042638	NM_053041	chr20:31315785-31315726	COMMD7
A_23_P59358	8.076414	8.029147	NM_173830	chr6:109471404-109475056	CEP57L1
A_24_P225907	8.855892	8.925426	NM_206831	chr3:16299693-16299634	DPH3
A_23_P15542	4.6817727	3.8693764	NM_000413	chr17:40707039-40707098	HSD17B1
A_33_P3331314	4.124155	2.3900566	AB305744	chr14:22265864-22265923	
A_24_P238744	12.273699	12.385656	NM_001145442	chr14:19988025-19987966	POTEM
A_23_P254271	13.221834	12.993248	NM_032525	chr18:12326476-12326535	TUBB6
A_23_P92230	6.5546293	6.7124376	NM_014779	chr3:150177182-150177241	TSC22D2
A_33_P3299220	5.0166564	5.1701274	NM_025008	chr1:150531141-150531200	ADAMTSL4
A_23_P4036	6.9324303	7.171454	NM_018469	chr17:62225479-62225420	TEX2
A_23_P97064	6.349578	6.329493	NM_018438	chr1:11733440-11733701	FBXO6
A_33_P3315949	4.7655773	5.0580735	NM_001258397	chr17:42980191-42980250	CCDC103
A_23_P384517	11.024889	11.396209	NM_004130	chr3:148745204-148745263	GYG1
A_24_P178423	11.341372	11.262677	NM_031314	chr2:190788815-190788876	HNRNPC
A_23_P70398	7.5984583	7.5975585	NM_001025370	chr6:43746223-43746634	VEGFA
A_33_P3256257	5.771285	6.149564	NM_033061	chr17:39240831-39240890	KRTAP4-7
A_33_P3371115	4.184499	4.7471685		chr9:42893065-42893124	AQP7P3
A_33_P3319281	6.5311933	6.6474338	BC028162	chr2:242162674-242162733	ANO7
A_32_P516818	6.306793	6.0716825	NM_001243729	chr3:157261272-157271051	C3orf55
A_23_P65157	10.082521	9.748661	NM_005694	chr3:119396074-119394045	COX17
A_23_P66038	4.4277177	5.3041306	NM_013304	chr16:67432121-67429417	ZDHHC1
A_33_P3224212	5.237788	4.8122725	NM_001282798	chr2:136481550-136481609	R3HDM1
A_23_P136355	7.05664	7.0353875	NM_018194	chr1:210849341-210849400	HHAT
A_33_P3369153	7.139416	6.4846783	NM_002254	chr2:26149514-26149455	KIF3C
A_33_P3361417	6.963148	6.696031	NM_145267	chr6:71298390-71298449	C6orf57
A_33_P3286041	9.377115	9.120598	NM_018119	chr16:22345280-22345339	POLR3E
A_23_P420373	5.008374	4.46479	NM_175630	chr2:25504453-25504394	DNMT3A
A_24_P376309	11.151855	11.149379	NM_032595	chr17:48211189-48211130	PPP1R9B
A_23_P167005	7.360504	7.469327	NM_014373	chr3:169802254-169802313	GPR160
A_23_P97221	5.514924	5.1929255	NM_015911	chr1:43317799-43317858	ZNF691
A_33_P3360942	7.8678026	8.271596	NM_004866	chr5:77773709-77773768	SCAMP1
A_33_P3324383	5.8017426	5.9436197	NM_001013690	chr12:52211752-52211693	FIGNL2
A_24_P236091	6.9194193	7.5091405	NM_001975	chr12:7032482-7032541	ENO2
A_33_P3318327	5.0580735	4.8640523	AK056275	chr9:116378869-116378810	FLJ31713
A_23_P158725	13.1391535	13.344613	NM_001042422	chr17:80197288-80197347	SLC16A3

A_24_P58204	4.0798497	3.76602	NM_001005567	chr11:5364268-5364209	OR51B5
A_24_P417007	7.634612	7.891637	NM_021974	chr22:38365194-38365253	POLR2F
A_23_P166993	5.296517	4.2380733	NM_004441	chr3:134978114-134978173	EPHB1
A_23_P66017	4.983132	4.790559	NM_145239	chr16:29826939-29826998	PRRT2
A_33_P3294679	5.3309894	4.898268	NR_024007	chr17:17680362-17680303	SMCR5
A_23_P36865	7.751709	7.374543	NM_025114	chr12:88442894-88442835	CEP290
A_33_P3345501	9.848531	9.880226		chr4:153717392-153717333	
A_23_P151662	6.3922043	6.2636576	NM_197957	chr14:65560518-65560459	MAX
A_23_P73420	6.947619	7.2550206	NM_031466	chr8:140742801-140742742	TRAPPC9
A_33_P3419733	5.658328	5.726994	NM_025219	chr20:62560739-62560798	DNAJC5
A_24_P336510	7.2546473	7.040935	NM_002113	chr1:196794705-196794764	CFHR1
A_33_P3216319	4.7201	5.1769705	NM_015144	chr16:87441344-87441285	ZCCHC14
A_23_P149775	9.223632	9.205314	NM_018287	chr10:32095364-32095305	ARHGAP12
A_23_P116642	4.698636	5.6541123	NM_133489	chr12:58019391-58019450	SLC26A10
A_23_P23194	7.6988783	7.6568384	NM_032409	chr1:20977325-20977384	PINK1
A_33_P3299854	8.370047	8.186218	NM_002486	chr9:100435948-100436007	NCBP1
A_33_P3229617	9.776228	9.719181	NM_001099401	chr7:94214729-94214670	SGCE
A_23_P39110	9.166455	9.019032	NM_006666	chr19:49513849-49514295	RUVBL2
A_23_P359904	11.406555	11.091354	NM_182612	chr11:767293-767234	PDDC1
A_33_P3273266	9.586176	9.9246025		chr10:42833150-42833106	LOC441666
A_33_P3213863	4.222348	3.6561286	NM_001012277	chr1:12979760-12979819	PRAMEF7
A_33_P3343196	4.9734597	5.3586593	NM_000096	chr3:148890861-148890802	CP
A_33_P3273444	9.601555	9.385128	NM_006468	chr1:145592690-145592631	POLR3C
A_23_P167559	9.370842	9.88269	NM_144726	chr5:158584724-158584665	RNF145
A_24_P161813	8.608967	8.725749		chr10:42832591-42832532	LOC441666
A_23_P146990	10.73518	10.577028	NM_007013	chr8:87479267-87479326	WWP1
A_23_P59069	10.688028	10.747471	NM_003527	chr6:27861588-27861647	HIST1H2BO
A_33_P3361681	11.370107	11.129656	NM_001017980	chrX:150577709-150577768	VMA21
A_24_P295877	4.9184914	4.0317836	NM_014704	chr1:3742359-3740105	CEP104
A_33_P3348519	5.9538426	5.367883	NR_104178	chr11:71729219-71729278	LOC100128494
A_23_P22548	7.2055106	6.940838	NM_000390	chrX:85117224-85117165	CHM
A_33_P3485976	4.3153605	2.9930372	AK095081	chr11:134357750-134357809	LOC283177
A_33_P3374957	8.968374	9.001081	NM_031918	chr19:1852458-1852399	KLF16
A_23_P131816	10.045073	9.873188	NM_052951	chr20:44439707-44439766	DNTTIP1
A_33_P3325195	9.029497	9.069089	AB028953	chr11:133785472-133785413	
A_33_P3312799	4.216701	3.4711106		chr2:095454776-095454835	
A_24_P363087	8.155405	8.570217	NM_016175	chr5:179264479-179264420	C5orf45
A_33_P3397180	4.1683173	4.2788625	AK128261	chr16:68401287-68401228	LOC100129324
A_24_P68068	5.072983	4.3789244	AK097770	chr12:8052513-8052572	
A_23_P135271	6.9711227	6.647155	NM_001497	chr9:33111524-33111465	B4GALT1
A_33_P3213874	5.193734	4.350077	NM_001004747	chr11:56020479-56020538	OR5T3
A_24_P307974	6.2492123	6.422281	BC033728	chr6:42025277-42025336	TAF8
A_24_P200694	7.771772	7.774537	NM_002931	chr6:33180113-33180172	RING1
A_23_P307761	6.8484516	6.2046285	NM_031215	chr20:60963943-60963884	CABLES2
A_24_P110082	11.619747	11.588425		chr11:017215041-017215100	
A_23_P13033	8.093032	8.337846	NM_002896	chr11:66411486-66411545	RBM4
A_23_P406785	5.1677456	4.970782	NM_199350	chr9:132374599-132374540	C9orf50
A_23_P21495	4.685399	4.8299465	NM_003890	chr19:40354126-40354068	FCGBP
A_33_P3235831	9.208194	9.264494		chr10:065187765-065187824	
A_33_P3240936	5.395881	4.891752	NM_012104	chr11:117161697-117161638	BACE1
A_23_P418493	6.7960086	6.814863	NM_145691	chr17:17921923-17921864	ATPAF2
A_32_P107002	4.0605965	3.492458	NM_032167	chr16:12145978-12146037	SNX29
A_33_P3502037	8.041875	7.704926	NM_012124	chr11:89933613-89933582	CHORDC1
A_23_P128641	6.691071	6.555956	NM_005800	chr13:31233106-31233165	USPL1
A_24_P64126	9.92254	10.21747	NM_003909	chr8:87572942-87573001	CPNE3
A_23_P134755	7.8900347	8.100479	NM_014112	chr8:116421052-116420993	TRPS1
A_33_P3219429	9.357597	9.093024	NM_005871	chr10:112053916-112053857	SMNDC1
A_32_P210572	8.943328	8.846014	NM_001024594	chr1:197876342-197876401	C1orf53
A_33_P3279640	9.491156	9.650277	NM_001194	chr19:617098-617157	HCN2

A_23_P6561	7.7368684	7.6200957	NM_018029	chr3:73112399-73112458	EBLN2
A_33_P3239228	5.665199	5.6559134	XM_005276004	chr7:100549871-100549930	
A_33_P3302115	6.3132386	5.160466	NM_005898	chr11:34124059-34124118	CAPRIN1
A_33_P3249434	7.3949003	6.8541517	NM_001031689	chr9:26903542-26903483	PLAA
A_24_P301557	8.279856	8.054498	NM_014646	chr18:2917332-2917273	LPIN2
A_23_P145874	7.5686164	7.5172453	NM_152703	chr7:92760278-92760219	SAMD9L
A_33_P3218659	2.3221061	2.3900566	NM_001004723	chr14:20296472-20296531	OR4N2
A_33_P3309556	7.0371056	6.394235	NM_006504	chr10:129884088-129884147	PTPRE
A_23_P364465	10.115826	10.233464	NM_022913	chr5:56558753-56558812	GPBP1
A_23_P165989	4.931653	4.5944386	NM_080749	chr20:44517429-44517370	NEURL2
A_33_P3842886	8.920335	8.8502	NM_013284	chr7:44111921-44111862	POLM
A_23_P67952	5.196659	4.022673	NR_026766	chr2:16080141-16080082	MYCNOS
A_23_P149476	8.251606	8.062291	NM_032328	chr1:245246774-245246970	EFCAB2
A_33_P3389060	6.8798294	6.714448	NM_018249	chr9:123165144-123165085	CDK5RAP2
A_23_P334123	8.054692	8.468758	NM_030790	chr16:47192890-47192831	ITFG1
A_23_P369899	10.000765	10.015991	NM_015444	chr3:45266206-45266147	TMEM158
A_33_P3264662	8.583701	7.7007127	NM_001001665	chr2:127941477-127941418	CYP27C1
A_33_P3378509	8.5666275	8.466717	NM_032557	chr4:144142361-144142420	USP38
A_24_P839254	6.682801	6.2034636	NM_001098783	chr3:58305009-58305068	RPP14
A_23_P75500	8.42935	8.12964	NM_001024957	chr11:66105051-66104992	BRMS1
A_33_P3311267	7.4823713	7.1998677	NM_181608	chr21:31859568-31859509	KRTAP19-2
A_33_P3345255	6.2799573	6.067524	NM_016506	chr11:47594541-47594482	KBTBD4
A_23_P208870	6.8707294	6.7963	NM_001626	chr19:40738762-40738703	AKT2
A_23_P65741	9.346394	9.040247	NM_133375	chr15:66625579-66625638	DIS3L
A_23_P147786	7.9289293	7.2305937	NM_014677	chr8:105263899-105263958	RIMS2
A_23_P31085	8.321356	8.616004	NM_015439	chr6:139114248-139114307	CCDC28A
A_32_P167904	2.3221061	2.3900566	NM_138286	chr19:23922803-23922744	ZNF681
A_24_P41939	4.1896596	2.3900566		chr9:081651996-081651936	
A_23_P429950	2.3221061	2.3900566	NM_000216	chrX:8497292-8497233	KAL1
A_24_P236799	7.8388395	7.593011	NM_006868	chr18:9862231-9862290	RAB31
A_23_P307274	5.507837	5.566563	NM_007160	chr6:29555768-29555827	OR2H2
A_33_P3246505	5.2895203	5.1171055	AB209539	chr10:30728298-30728357	MAP3K8
A_24_P291231	5.4399986	5.9189463	NM_016831	chr1:7904641-7904700	PER3
A_24_P177948	5.7597337	5.7784004	NM_173812	chr12:63953486-63953427	DPY19L2
A_33_P3379371	9.615696	9.431902	NM_001005861	chr3:133876118-133876059	RYK
A_33_P3394347	6.0051546	6.5024486	NR_024066	chr3:150797554-150797613	CLRN1-AS1
A_23_P350754	5.5792437	5.597333	NR_045002	chr11:17074167-17074226	OR7E14P
A_24_P185117	6.1386127	6.209281	NM_031430	chr17:1550220-1549875	RILP
A_23_P304450	7.9965186	8.572012	NM_005257	chr18:19781793-19781852	GATA6
A_23_P150931	5.8201265	6.5782223	NM_018113	chr12:49491297-49491238	LMBR1L
A_23_P74799	11.307478	10.901456	NM_213651	chr1:108677551-108677492	SLC25A24
A_23_P111343	9.930538	9.967065	NM_014739	chr6:136582580-136582521	BCLAF1
A_24_P228579	8.434114	8.05273	NM_024120	chr20:13797550-13797769	NDUFAF5
A_23_P84344	6.902822	6.800676	NM_021805	chr11:405937-405878	SIGIRR
A_33_P3263851	5.5930967	5.5551105		chr21:35335427-35335486	
A_24_P830690	5.909872	5.6841497	NM_002613	chr16:2631645-2631704	PDPK1
A_33_P3420011	6.458615	6.2206364		chr16:072038313-072038254	
A_33_P3288694	6.666526	6.6044703	XR_242003	chr6:34665191-34665247	
A_33_P3294578	9.594309	9.458644	NM_002740	chr3:170023598-170023657	PRKCI
A_23_P146456	8.46088	8.45203	NM_001333	chr9:99795211-99795152	CTSV
A_23_P7253	4.3011785	4.3118668	NM_018078	chr4:129131868-129131927	LARP1B
A_23_P79725	4.6025944	4.1784825	NM_022730	chr2:232672788-232672847	COPS7B
A_24_P67988	10.048481	9.958747	NM_031904	chr11:65180932-65180991	FRMD8
A_33_P3362239	7.698067	7.8165994		chr7:073464225-073464284	
A_23_P359214	4.3299522	3.181755	NR_033957	chr10:47151178-47151119	LINC00842
A_24_P943095	7.5922055	7.3732224	NM_001033553	chr17:20217691-20217750	SPECC1
A_24_P402779	3.8966012	3.2990499	NM_005485	chr3:51979914-51980199	PARP3
A_33_P3322373	7.7923717	7.970331	NM_001282632	chr9:136036914-136036855	GBGT1
A_33_P3419139	5.6615105	5.6553197	NM_001127211	chr10:118645949-118645890	KIAA1598

A_33_P3388482	4.2635503	3.7843618	NM_001080825	chr12:122213575-122213634	TMEM120B
A_33_P3236748	4.3554363	4.649636	XR_111551	chr15:93749898-93749957	
A_33_P3261973	4.316964	4.018106	NM_006594	chr1:114442661-114442602	AP4B1
A_23_P214950	11.293382	11.267124	NM_022121	chr6:138412115-138412056	PERP
A_33_P3369696	10.4509535	10.140747	NM_018074	chr19:4269023-4269082	CCDC94
A_33_P3326217	5.0377903	4.863374	NM_207386	chr17:11461425-11461484	SHISA6
A_23_P415006	9.518974	9.47271	NM_015470	chr2:73300950-73300891	RAB11FIP5
A_33_P3480395	5.3346024	5.379275	AK055463	chr22:43593387-43593328	FLJ30901
A_32_P98348	14.140438	14.070338	NR_003699	chr19:53889677-53889736	ZNF525
A_23_P252283	8.602742	8.6958275	NM_032322	chr17:29326723-29326782	RNF135
A_24_P191588	8.182639	7.9431067	NM_000688	chr3:52246277-52246336	ALAS1
A_23_P77568	13.773476	13.653279	NM_001037808	chr16:28746857-28746916	EIF3C
A_33_P3247342	6.595303	6.3962345	NM_001001666	chr2:242135520-242135579	ANO7
A_33_P3329839	11.052067	11.035284	NM_005851	chr11:67274059-67274000	CDK2AP2
A_33_P3323347	7.1097565	6.676547	NM_152475	chr19:58417239-58417180	ZNF417
A_32_P129527	9.014074	8.737355	NM_018341	chr6:170181465-170181524	ERMARD
A_23_P12140	11.780402	11.753389	NM_000969	chr1:93299130-93299189	RPL5
A_23_P170733	8.91897	8.640721	NM_058172	chr4:80826901-80826842	ANTXR2
A_33_P3802116	7.962717	8.392352	XR_247160	chr8:37455070-37455011	
A_33_P3286789	6.0487638	5.928468		chr3:150438469-150438410	
A_23_P24176	8.390783	7.859679	NM_019084	chr10:97820399-97820458	CCNJ
A_33_P3215797	5.7923627	5.769009	NM_001029882	chr1:27877922-27877863	AHDC1
A_33_P3333648	4.7226048	4.2123327	NM_001004754	chr11:5475338-5475397	OR51I2
A_23_P256231	9.328773	9.129709	NM_032145	chr6:146119610-146119551	FBXO30
A_33_P3345708	6.06996	5.527629	NM_130898	chr1:153945484-153945543	CREB3L4
A_33_P3869455	6.6492853	6.8620243	DQ641254	chr13:70712089-70712030	ATXN8
A_33_P3380211	7.231739	7.070635	NM_005751	chr7:91709254-91709313	AKAP9
A_32_P470728	4.4010596	3.703293	NM_145657	chr13:28367769-28367828	GSX1
A_24_P397584	6.675275	6.6238213	NM_003192	chr6:42712911-42712852	TBCC
A_23_P213959	5.2005477	4.684317	NM_133263	chr5:149221927-149225368	PPARGC1B
A_24_P181672	7.024693	6.941936	NM_001009905	chr17:80915095-80914625	B3GNTL1
A_23_P150249	7.4950504	7.410246	NM_006848	chr11:65658775-65658834	CCDC85B
A_33_P3297020	8.308253	7.9673624	NR_026816	chr6:31141572-31141513	PSORS1C3
A_33_P3299279	5.4174223	5.348492	NM_001014279	chr5:43039560-43039501	ANXA2R
[truncated: 30,105 more chars]
